# Supplementary material for: Kinetic resolution of substituted amido[2.2]paracyclophanes via asymmetric electrophilic amination
Source: Nat Commun. 2023 Aug 28;14:5239. doi: 10.1038/s41467-023-40718-8 (PMC10462673; doi:10.1038/s41467-023-40718-8)
Supplement: Supplementary file 1 — Supplementary Information [file 41467_2023_40718_MOESM1_ESM.pdf]

Supplementary Information

Table of Contents

1. **Supplementary Methods**……………………………………………………….…….2

1.1 General Information 2

1.2 Synthesis of racemic [2.2]Paracyclophane derivatives................................... 3

1.3 Kinetic resolution of amido-[2.2]Paracyclophane derivatives 32

1.4 Enantioselective desymmetrization of diamido-PCP 66

1.5 Mechanism studies 67

1.6 Large scale kinetic resolution of **1a** 88

1.7 Derivatizations of the chiral products 89

1.8 Applications of amido-PCP in the development of chiral organocatalyst 98

2. Supplementary Notes

2.1 X-Ray structures 105

2.2 HPLC traces 107

2.3 NMR spectra 317

1. Supplementary Methods

1.1 General Information

Unless otherwise noted, all commercial reagents were used without further purification. Dichloromethane, toluene, ether, THF were purified by passage through an activated alumina column under argon. Thin-layer chromatography (TLC) analysis of reaction mixtures were performed using Huanghai silica gel HSGF254 TLC plates, and visualized under UV or by staining with ceric ammonium molybdate or potassium permanganate. Flash column chromatography was carried out on Huanghai Silica Gel HHGJ-300, 300-400 mesh. Nuclear magnetic resonance (NMR) spectra were recorded using Bruker Avance III HD spectrometer (FT, 500 MHz or 400 MHz for ^{1}H, 126 MHz or 101 MHz for ^{13}C, 471 MHz for ^{19}F, 202 MHz for ^{31}P). Data for ^{1}H NMR were reported as follows: chemical shift (δ ppm downfield from tetramethylsilane and referenced to residual solvent peaks), multiplicity (s = singlet, d = doublet, t = triplet, q = quartet, m = multiplet, br = broad resonance), integration, coupling constant (Hz). Data for ^{13}C NMR were reported in terms of chemical shift. Mass spectral data were obtained from the Agilent Technologies 6230 TOF LC/MS spectrometer in electrospray ionization (ESI^{+}) mode. Optical rotations were measured with an Autopol V Plus/VI digital polarimeter. X-Ray structure analyses were performed using a Bruker D8 Venture X-ray single crystal diffractometer. Enantiomeric excesses were determined on an Agilent 1260 Chiral HPLC using IA, IB, IC, ID and IG columns. The CPA catalysts employed in this works are purchased from bidepharmatech. The racemic products were afforded by using racemic phosphoric acid **A4** as catalyst.

1.2 Synthesis of racemic [2.2]Paracyclophane derivatives

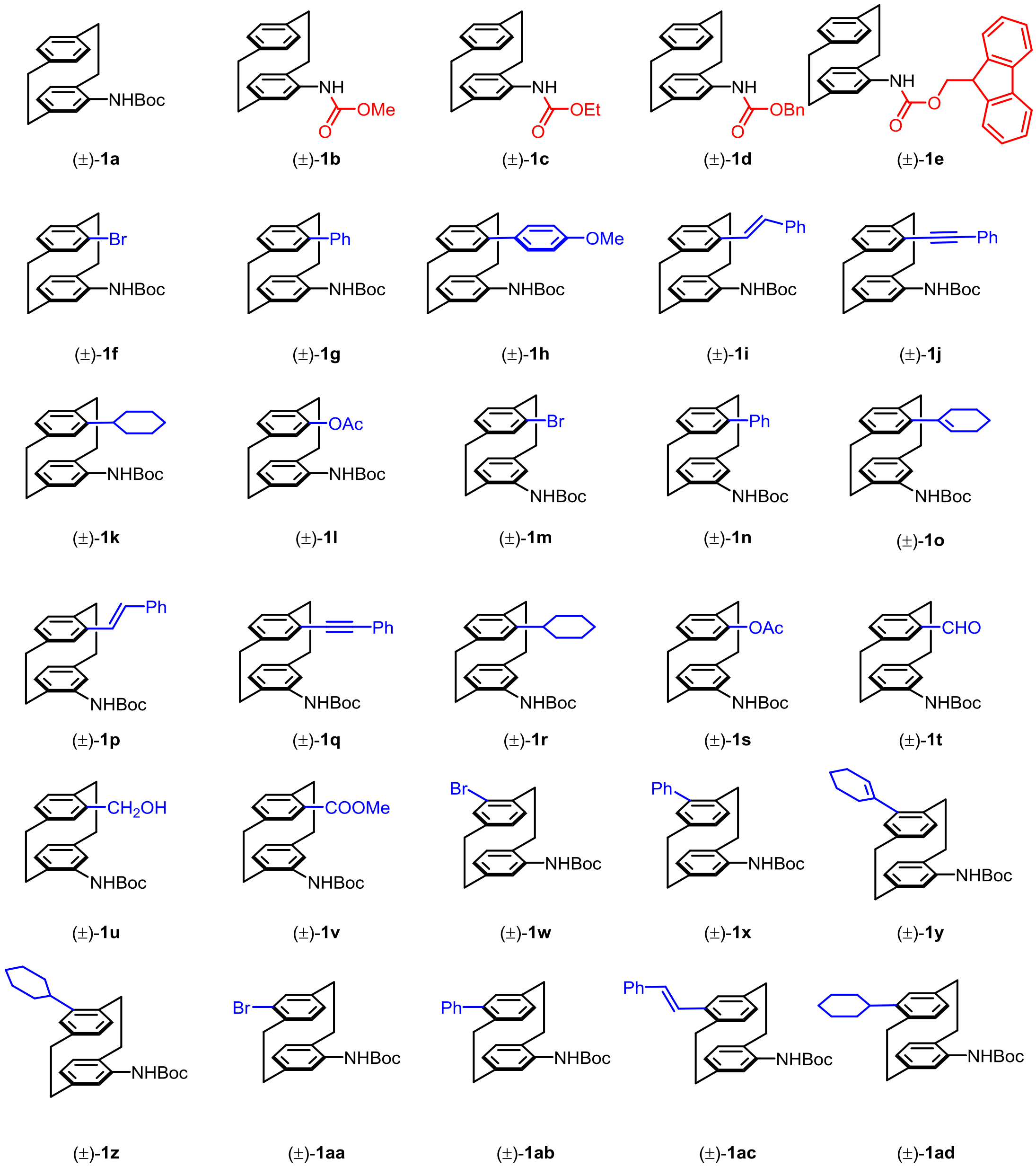

Supplementary Fig. 1. The racemic substrates prepared in this study.

Method A:

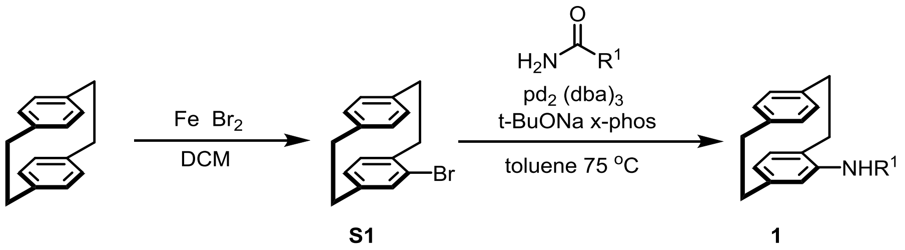

Substrate **1a**, **1b**, **1c**, **1d** was synthesized with Method A

General procedure of **method A**:

Synthesis of S1 (if **S1** is not commercial available): To a solution of [2.2]paracyclophane (10.0 g, 48 mmol, 1.0 equiv.), iron powder (54 mg, 0.96 mmol, 0.02 equiv.) in DCM (80 mL) was added a solution of Br_2 (2.7 mL, 52.8 mmol, 1.1 equiv.) in DCM (20 mL). The mixture was stirred at room temperature for 0.5 h. After completion of the reaction as indicated by TLC analysis, the reaction mixture was quenched with saturated Na_2SO_3 solution (50 mL), and extracted with DCM (3×70 mL). The combined organic layers were washed with brine (30 mL), dried over Na_2SO_4, filtered and concentrated to give a residue, which was triturated with petroleum ether. The precipitate was filtered to afford **S1** (12.3 g, 89%) as a white solid.

Synthesis of 1: To a solution of **S1** (1.0 equiv.), amide (490 mg, 4.18 mmol, 1.2 equiv.), t-BuONa (1.4 equiv.), x-Phos (0.15 equiv.) in toluene was added $Pd_2(dba)_3$ (0.05 equiv.) under N_2 atmosphere. After stirring at 75 ^oC under N_2 atmosphere overnight, the reaction mixture was cooled to rt and concentrated to give a residue, which was purified by column chromatography to afford **1**.

Synthesis of 1e

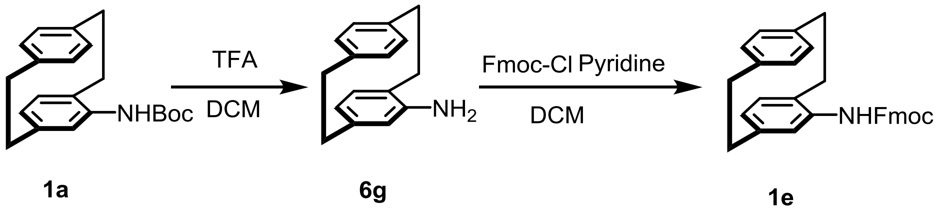

To a solution of **1a** (323 mg, 1 mmol, 1.0 equiv.) in DCM (3 mL) was added TFA (1 mL), and the mixture was allowed to stir at RT for 3h. After completion of the reaction as indicated by TLC analysis, the reaction mixture was neutralized with saturated Na_2CO_3 solution, extracted with DCM (3×10 mL), The combined organic layers were washed with brine (10 mL), dried over Na_2SO_4, filtered and concentrated to afford racemic **6g** (400 mg, 92%) as a white solid.

To a solution of racemic **6g** (220 mg, 0.98 mmol, 1.0 equiv.), pyridine (0.15

mL,1.8 mmol, 1.2 equiv.) in DCM (5 mL) was added Fmoc-Cl (304 mg, 1.18 mmol, 1.2 equiv.) at 0 °C. After stirring at RT for 5h, the reaction mixture was poured into H_2O and then extracted with DCM (3×10 mL), The combined organic layers were washed with brine (10 mL), dried over Na_2SO_4, filtered and concentrated to give a residue, which was purified by column chromatography (petroleum ether:EtOAc = 20:1) to afford **1e** (220 mg, 99%) as white solid.

Method B:

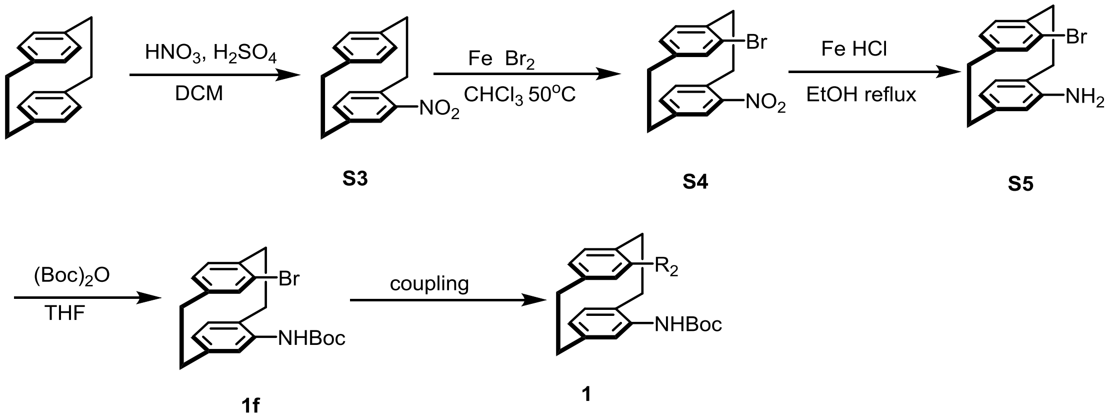

Substrate **1f**, **1g**, **1h** was synthesized with Method B

General procedure of **method B**:

Synthesis of S3: To a solution of [2.2]paracyclophane (2.0 g, 9.6 mmol, 1.0 equiv.) in DCM (190 mL) was added conc.H_2SO_4 (1.25 mL, 23 mmol, 2.4 equiv.), conc.HNO_3 (1.15 mL, 19.2 mmol, 2.0 equiv.) at 0 °C, and the mixture was stirred at room temperature for 0.5 h. After completion of the reaction as indicated by TLC analysis, the reaction mixture was cooled to rt, poured into ice-water (100 mL) and then extracted with DCM (3×50 mL), The combined organic layers were washed with brine (30 mL), dried over Na_2SO_4, filtered and concentrated to give a residue, which was purified by column chromatography (petroleum ether:EtOAc = 10:1) to afford **S3** (1.31g, 54%) as yellow solid.

Synthesis of S4: To a solution of **S3** (506 mg, 2 mmol, 1.0 equiv.), iron powder (11 mg, 0.2 mmol, 0.1 equiv.) in $CHCl_3$ (5 mL) was added a solution of Br_2 (0.13 mL, 2.6 mmol,

1.3 equiv.) in $CHCl_3$ (5 mL), and the mixture was allowed to stir at 50 ^oC for 3 h. After completion of the reaction as indicated by TLC analysis, the reaction mixture was cooled to rt, quenched with saturated Na_2SO_3 solution (30 mL) at 0^oC, and extracted with DCM (3×30 mL). The combined organic layers were washed with brine (10 mL), dried over Na_2SO_4, filtered and concentrated to give a residue, which was purified by column chromatography (petroleum ether:EtOAc = 10:1) to afford **S4** (500 mg, 75%) as a yellow solid.

Synthesis of S5: To a solution of **S4** (370 mg, 1.11 mmol, 1.0 equiv.), iron powder (746 mg, 13.32 mmol, 12 equiv.) in EtOH/H_2O (8 mL/8 mL) was added conc. HCl(2.62 mL, 0.22 mmol, 0.2 equiv.), and the mixture was allowed to stir at reflux for 3 h. After completion of the reaction as indicated by TLC analysis, the reaction mixture was cooled to rt, poured into ice-water (15 mL), neutralized with saturated Na_2CO_3 solution, then extracted with EtOAc (3×10 mL). The combined organic layers were washed with brine (10 mL), dried over Na_2SO_4, filtered and concentrated to give a residue, which was purified by column chromatography (petroleum ether:EtOAc = 10:1) to afford **S5** (310 mg, 92%) as a white solid.

Synthesis of 1f: To a solution of **S5** (310 mg, 1.03 mmol, 1.0 equiv.), TEA (0.17 mL, 1.24 mmol, 1.2 equiv.) in THF (5 mL) was added Boc_2O (0.29 mL, 1.24 mmol, 1.2equiv.), and the mixture was stirred at 60 ^oC for 5 h. After completion of the reaction as indicated by TLC analysis, the mixture was cooled to rt and concentrated to give a residue, which was purified by column chromatography (petroleum ether:EtOAc = 10:1) to afford **1f** (350 mg, 85%) as a white solid.

Synthesis of 1g: To a solution of **1f** (201 mg, 0.5 mmol, 1.0 equiv.), phenylboronic acid (183 mg, 1.5 mmol, 3.0 equiv.), Na_2CO_3 (318 mg, 3.0 mmol, 6.0 equiv.) in dioxane/H_2O (10 mL/1 mL) was added pd(dppf)Cl_2 (203 mg, 0.25 mmol, 0.5 equiv.) under N_2 atmosphere. After stirring at reflux under N_2 atmosphere overnight, the reaction mixture was cooled to rt and concentrated under vacuum to give a residue, which was purified by column chromatography (petroleum ether:EtOAc = 10:1) to

afford **1g** (180 mg, 90%) as a white solid.

Synthesis of 1h: To a solution of **1f** (804 mg, 2 mmol, 1.0 equiv.), 4-methoxyphenylboronic acid (912 mg, 6 mmol, 3.0 equiv.), KF (697 mg, 12 mmol, 6.0 equiv.) in dioxane/H_2O (10 mL/1 mL) was added $Pd(PPh_3)_4$ (462 mg, 0.4 mmol, 0.2 equiv.) under N_2 atmosphere. After stirring at reflux under N_2 atmosphere overnight, the reaction mixture was cooled to rt and concentrated to give a residue, which was purified by column chromatography (petroleum ether:EtOAc = 20:1) to afford **1h** (510 mg, 59 %) as a white solid.

Method C:

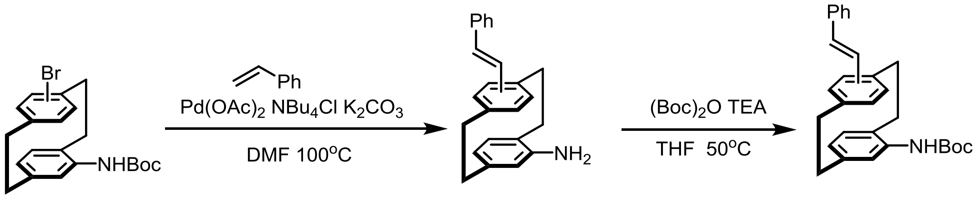

1f, 1m, 1w **S6** **1i, 1p, 1ac**

Substrate **1i**, **1p**, **1ac** was synthesized with Method C

General procedure of **method C**:

Synthesis of S6: To a solution of **1f, 1m** or **1w**, tetrabutyl ammonium chloride (3.0 equiv.), styrene (5.0 equiv.), K_2CO_3 (6.0 equiv.) in DMF was added $Pd(OAc)_2$ (0.2 equiv.) under N_2 atmosphere. After stirring at 100°C under N_2 atmosphere overnight, the reaction mixture was cooled to rt and concentrated under vacuum to give a residue, which was purified by column chromatography to afford **S6**.

Synthesis of 1i, 1p, 1ac: To a solution of **S6** (1.0 equiv.), TEA (1.2 equiv.) in THF was added Boc_2O (1.2 equiv.). The mixture was stirred at 50°C overnight. After completion of the reaction as indicated by TLC analysis, the reaction mixture was cooled to rt and concentrated under vacuum to give a residue, which was purified by column chromatography to afford **1i, 1p** or **1ac**.

Method D:

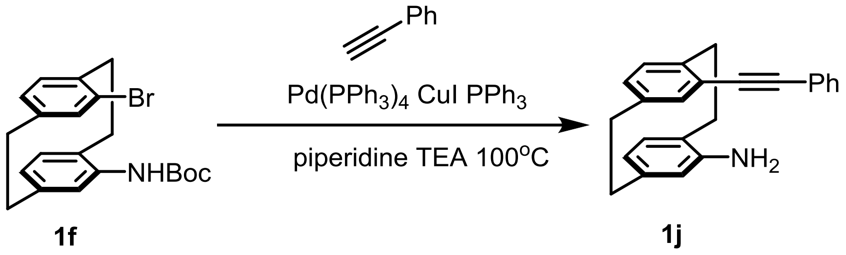

Substrate **1j** was synthesized with method D

General procedure of **method D**:

Synthesis of 1j: To a solution of **1f** (603 mg, 1.5 mmol, 1.0 equiv.), phenylacetylene (0.25 mL, 2.25 mmol, 1.5 equiv.), PPh_3 (79 mg, 0.3 mmol, 0.2 equiv.) and TEA (4 mL) in piperidine (8 mL) was added CuI (57 mg, 0.3 mmol, 0.2 equiv.) and $Pd(PPh_3)_4$ (173 mg, 0.15 mmol, 0.1 equiv.) under N_2 atmosphere. After stirring at 100 ^oC overnight, the reaction mixture was poured into H_2O (10 mL) and then extracted with EtOAc (3 ×15 mL). The combined organic layers were then washed with brine (10 mL), dried over Na_2SO_4, filtered and concentrated under vacuum to give a residue, which was purified by column chromatography (petroleum ether:EtOAc = 10:1) to afford **1j** (150 mg, 24%) as a yellow solid.

Method E:

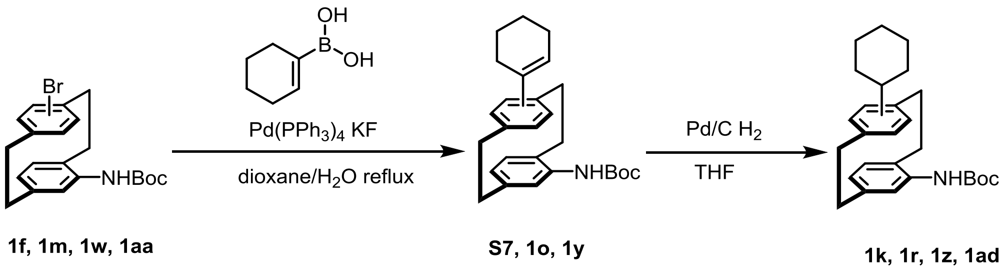

Substrate **1o**, **1y, 1k, 1r, 1z, 1ad** was synthesized with Method E

General procedure for **method E**:

Synthesis of S7, 1o, 1y: To a solution of **1f, 1m, 1w** or **1aa** (1.0 equiv.), cyclohexene-1-boronic acid (3.0 equiv.), KF (6.0 equiv.) in dioxane/H_2O was added $Pd(PPh_3)_4$ (0.2 equiv.) under N_2 atmosphere. After stirring at reflux under N_2 atmosphere overnight, the reaction mixture was cooled to rt and concentrated under

vacuum to give a residue, which was purified by column chromatography to afford **S7, 1o or 1y**.

Synthesis of 1k, 1r, 1z, 1ad: To a solution of **S7, 1o or 1y** (1.0 equiv.) in THF (20 mL) was added Pd/C under H_2 atmosphere. After stirring at rt under H_2 atmosphere overnight, the reaction mixture was filtered and the filtrate was concentrated under vacuum to give a residue, which was purified by column chromatography to afford **1k, 1r, 1z and 1ad**.

Method F:

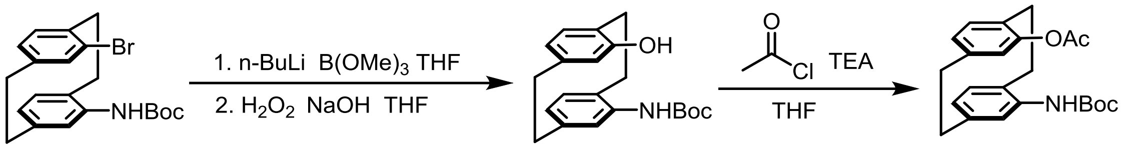

1f **S8** **1l**

Substrate **1l** was synthesized with Method F

General procedure of **method F**:

Synthesis of S8: To a solution of **1f** (1.0 g, 2.5 mmol, 1.0 equiv.) in anhydrous THF (10 mL) was added n-BuLi (2.5 M, 2.2 mL, 5.5 mmol, 2.2 equiv.) under N_2 atmosphere at -78 ^{o}C. After stirring at rt for 0.5 h. $B(OMe)_3$ (1.49 mL, 12.5 mmol, 5.0 equiv.) was added under N_2 atmosphere at -78 ^{o}C. The mixture was warmed to rt, and allowed to stir at rt overnight. Then H_2O_2 (30%wt, 1.02 mL, 10.0 mmol, 4.0 equiv.), NaOH (10 M, 0.3 mL, 3 mmol, 1.2 equiv.) was added, and the mixture was allowed to stir at rt under N_2 atmosphere for 5 h. The reaction mixture was then quenched with saturated NH_4Cl solution (10 mL), extracted with EtOAc (3×15 mL). The combined organic layers were then washed with brine (10 mL), dried over Na_2SO_4, filtered and concentrated under vacuum to give a residue, which was purified by column chromatography (petroleum ether:EtOAc = 15:1-5:1) to afford **S8** (400 mg, 47%) as a white solid.

Synthesis of 1l: To a solution of **S8** (370 mg, 1.09 mmol, 1.0 equiv.), TEA (0.38 mL, 2.73 mmol, 2.5 equiv.) in THF (10 mL) was added acetyl chloride (0.09 mL, 1.31 mmol, 1.2 equiv.) at 0 ^{o}C. After stirring at rt overnight, the mixture was quenched with

saturated NH_4Cl solution (10 mL) and extracted with EtOAc (3×15 mL). The combined organic layers were then washed with brine (10 mL), dried over Na_2SO_4, filtered and concentrated under vacuum to give a residue, which was purified by column chromatography (petroleum ether:EtOAc = 5:1) to afford **1l** (300 mg, 72%) as a yellow solid.

Method G:

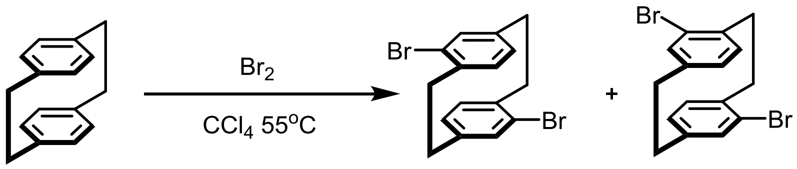

S9 S10

General procedure of **method G**:

Synthesis of S9 and S10: To a solution of [2.2]paracyclophane (4.17 g, 20 mmol, 1.0 equiv.), in CCl_4 (20 mL) was added a solution of Br_2 (6.1 mL, 120 mmol, 6.0 equiv.) in CCl_4 (20 mL). After The stirring at 50°C for 3 h, the reaction mixture was cooled to rt, quenched with saturated Na_2SO_3 solution (60 mL) and extracted with DCM (3×30 mL). The combined organic layers were washed with brine (10 mL) and concentrated under vacuum to give a residue, which was triturated with petroleum ether for 2 times. The precipitate was filtered to afford **S9** (2.6 g, 35.5%) as a white solid. The filtrate was concentrated to give a residue, which was recrystallization by EtOH to afford **S10** (3.3 g, 45%) as a white solid.

Method H:

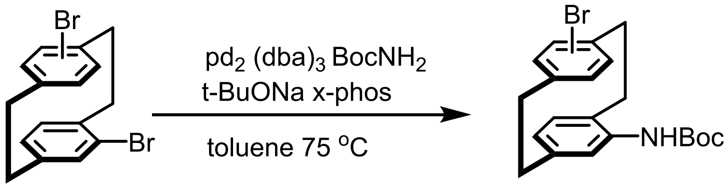

S9, S10, S11 **1m, 1w, 1aa**

Substrate **1m**, **1w, 1aa** was synthesized with Method H

General procedure of **method H**:

Synthesis of 1m, 1w, 1aa: To a solution of **S9, S10** or **S11** (1.0 equiv.), $BocNH_2$(1.2 equiv.), t-BuONa (1.4 equiv.), x-Phos (0.15 equiv.) in toluene was added $Pd_2(dba)_3$ (0.05 equiv.) under N_2 atmosphere. After stirring at 75 ^oC under N_2 atmosphere overnight, the reaction mixture was cooled to rt and concentrated under vacuum to give a residue, which was purified by column chromatography to afford **1m, 1w** or **1aa**.

Method I:

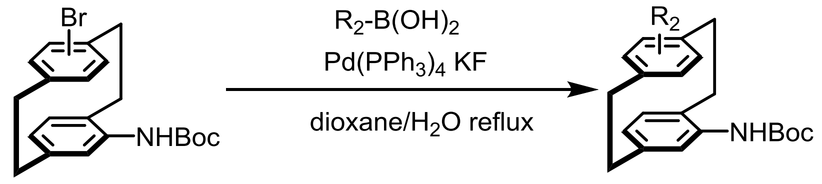

1m, 1w, 1aa **1n, 1x, 1ab**

Substrate **1n, 1x, 1ab** was synthesized with Method I

General procedure of **method I**:

Synthesis of 1n, 1x, 1ab: To a solution of **1m, 1w** or **1aa** (1.0 equiv.), boronic acid (3.0 equiv.), KF (6.0 equiv.) in dioxane/H_2O was added $Pd(PPh_3)_4$ (0.2 equiv.) under N_2 atmosphere. After stirring at reflux under N_2 atmosphere overnight, the reaction mixture was cooled to rt and concentrated under vacuum to give a residue, which was purified by column chromatography to afford **1n, 1x** or **1ab**.

Method J:

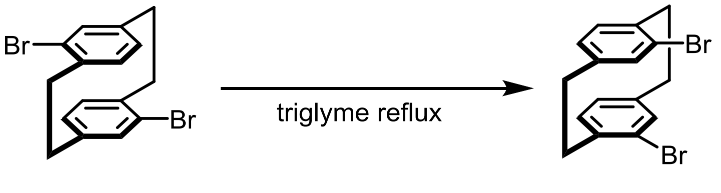

S9 **S11**

General procedure of **method J**:

Synthesis of S11： A solution of **S9** (8.5 g, 23.2 mmol, 1.0 equiv.) in triglyme (30 mL) was heated to reflux under N_2 atmosphere. After refluxing for 4 h, the mixture was

cooled to rt and filtered. The precipitate was added triglyme (30 mL), heated to reflux under N_2 atmosphere. After three more isomerization cycles, the combined filtrate was poured into H_2O (1 L) and then extracted with EtOAc (3×100 mL), The combined organic layers were then washed with brine (50 mL), dried over Na_2SO_4, filtered and concentrated under vacuum to give a residue, which was purified by column chromatography (petroleum ether) to afford **S11** (6.9 g, 81%) as a white solid.

Method K:

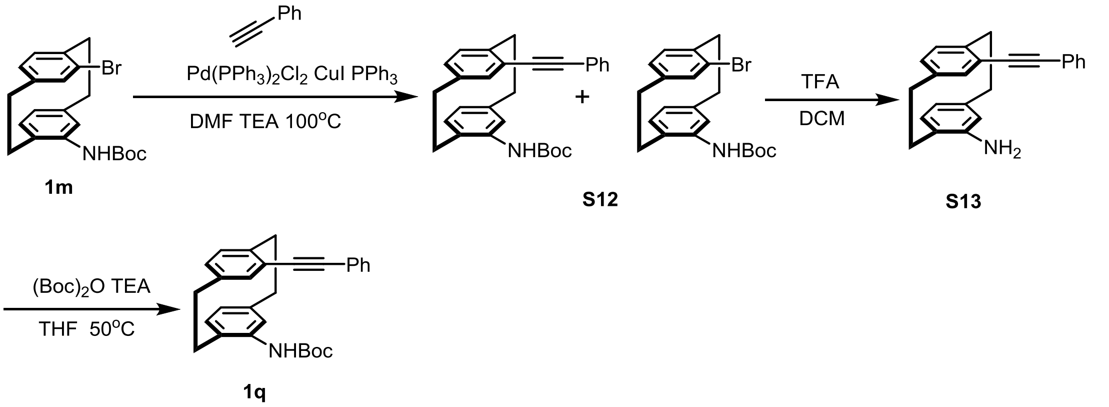

Substrate **1q** was synthesized with Method K

General procedure of **method K**:

Synthesis of S12: To a solution of **1m** (642 mg, 1.6 mmol, 1.0 equiv.), phenylacetylene (1.06 mL, 9.6 mmol, 6.0 equiv.), TEA (4 mL) in DMF (4 mL) was added CuI (61 mg, 0.32 mmol, 0.2 equiv.) and $Pd(PPh_3)_2Cl_2$ (225 mg, 0.32 mmol, 0.2 equiv.) under N_2 atmosphere. After stirring at 100 ^{o}C overnight, the reaction mixture was poured into H_2O (40 mL) and then extracted with EtOAc (3×15 mL). The combined organic layers were then washed with brine (10 mL), dried over Na_2SO_4, filtered and concentrated under vacuum to give a residue, which was purified by column chromatography (petroleum ether:EtOAc = 20:1) to afford **S12** as a mixture of **1m** and **S12** (610 mg, about 50% purity) as brown oil.

Synthesis of S13: To a solution of **S12** (610 mg) in DCM (3 mL) was added TFA (1 mL) at rt. After stirring at rt for 3h, the reaction mixture was neutralized with saturated Na_2CO_3 solution, extracted with DCM (3×10 mL), The combined organic layers were

washed with brine (10 mL), dried over Na_2SO_4, filtered and concentrated to give a residue, which was purified by column chromatography (petroleum ether:EtOAc = 20:1) to afford **S13** (175 mg) as a yellow solid.

Synthesis of 1q: To a solution of **S13** (175 mg, 0.54 mmol, 1.0 equiv.), TEA (0.09 mL, 0.65 mmol, 1.2 equiv.) in THF (5 mL) was added Boc_2O (0.15 mL, 0.65 mmol, 1.2 equiv.) at rt. After stirring at 50 ^oC overnight, the reaction mixture was cooled to rt and concentrated under vacuum to give a residue, which was purified by column chromatography (petroleum ether:EtOAc = 20:1) to afford **1q** (200 mg, 87%) as a yellow solid.

Method L:

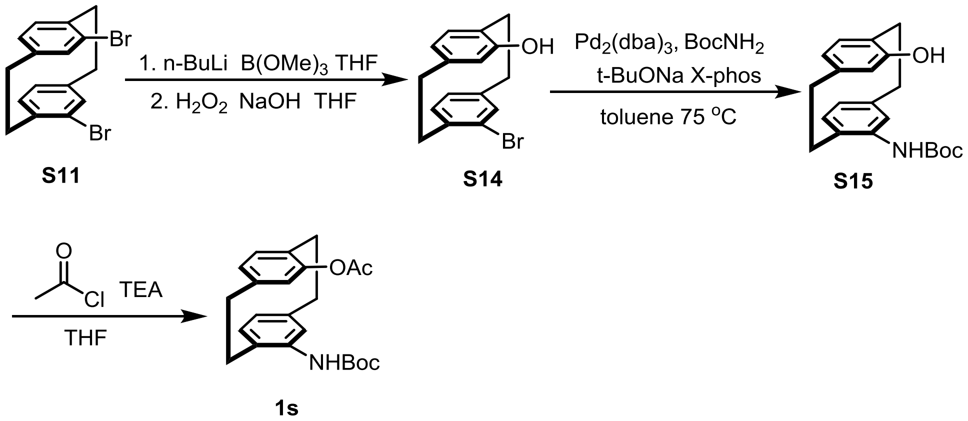

Substrate **1s** was synthesized with Method L

General procedure of **method L**:

Synthesis of S14: To a solution of **S11** (300 mg, 0.82 mmol, 1.0 equiv.) in anhydrous THF (10 mL) was added n-BuLi (2.4 M, 0.38 mL, 0.90 mmol, 1.1 equiv.) under N_2 atmosphere at -78^oC. After stirring at rt for 0.5 h, $B(OMe)_3$ (0.49 mL, 4.1 mmol, 5.0 equiv.) was added under N_2 atmosphere at -78^oC. The mixture was warmed to rt and allowed to stir at rt for 3 h. Then H_2O_2 (30%wt, 0.34 mL, 3.28 mmol, 4.0 equiv.), and NaOH (1 M, 0.98 mL, 3 mmol, 1.2 equiv.) was added, and the mixture was allowed to stir at rt under N_2 atmosphere overnight. The reaction mixture was quenched with saturated NH_4Cl solution (10 mL) and extracted with EtOAc (3 ×15 mL). The combined

organic layers were then washed with brine (10 mL), dried over Na_2SO_4, filtered and concentrated under vacuum to give a residue, which was purified by column chromatography (petroleum ether:EtOAc = 20:1) to afford **S14** (139 mg, 50%) as a yellow solid.

Synthesis of S15: To a solution of **S14** (139 mg, 0.46 mmol, 1.0 equiv.), $BocNH_2$(65 mg, 0.55 mmol, 1.2 equiv.), t-BuONa (62 mg, 0.64 mmol, 1.4 equiv.), x-Phos (33 mg, 0.07 mmol, 0.15 equiv.) in toluene (5 mL) was added $Pd_2(dba)_3$ (21 mg, 0.02 mmol, 0.05 equiv.) under N_2 atmosphere. After stirring at 75^oC under N_2 atmosphere overnight, the reaction mixture was cooled to rt, concentrated under vacuum to give a residue, which was purified by column chromatography (petroleum ether:EtOAc = 10:1) to afford **S15** (96 mg, 62%) as a yellow solid.

Synthesis of 1s: To a solution of **S15** (96 mg, 0.28 mmol, 1.0 equiv.), TEA (0.10 mL, 0.7 mmol, 2.5 equiv.) in THF (2 mL) was added acetyl chloride (0.024 mL, 1.34 mmol, 1.2 equiv.) at 0 ^oC. After stirring at rt overnight, the mixture was quenched with saturated NH_4Cl solution (10 mL) and extracted with EtOAc (3×10 mL). The combined organic layers were then washed with brine (10 mL), dried over Na_2SO_4, filtered and concentrated under vacuum to give a residue, which was purified by column chromatography (petroleum ether:EtOAc = 5:1) to afford **1s** (70 mg, 65%) as a yellow solid.

Method M:

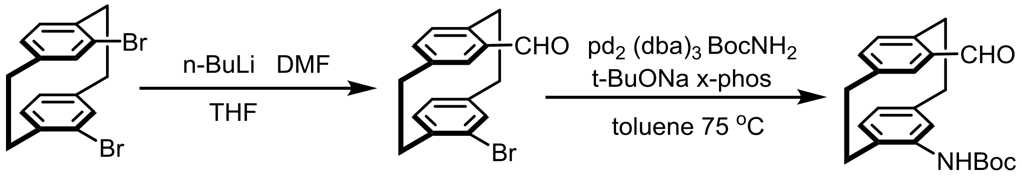

S11 **S16** **1t**

Substrate **1t** was synthesized with Method M

General procedure of **method M**:

Synthesis of S16: To a solution of **S11** (1.0 g, 2.73 mmol, 1.0 equiv.) in anhydrous THF (50 mL) was added n-BuLi (2.4 M, 1.25 mL, 3.00 mmol, 1.1 equiv.) under N_2

atmosphere at -78°C. After stirring at rt for 0.5 h, DMF (1.67 mL, 21.84 mmol, 8.0 equiv.) was added under N_2 atmosphere at -78°C. The mixture was warmed to rt and allowed to stir at rt for 0.5 h. The reaction mixture was quenched with saturated NH_4Cl solution (20 mL) and extracted with EtOAc (3×15 mL). The combined organic layers were then washed with brine (10 mL), dried over Na_2SO_4, filtered and concentrated under vacuum to give a residue, which was purified by column chromatography (petroleum ether:EtOAc = 20:1) to afford **S16** (610 mg, 71%) as a white solid.

Synthesis of 1t： To a solution of **S16** (610 mg, 1.94 mmol, 1.0 equiv.), $BocNH_2$(273 mg, 2.33 mmol, 1.2 equiv.), t-BuONa (261 mg, 2.72 mmol, 1.4 equiv.), x-Phos (139 mg, 0.29 mmol, 0.15 equiv.) in toluene (8 mL) was added $Pd_2(dba)_3$ (91 mg, 0.10 mmol, 0.05 equiv.) under N_2 atmosphere. After stirring at 75°C under N_2 atmosphere overnight, the reaction mixture was cooled to rt and concentrated under vacuum to give a residue, which was purified by column chromatography (petroleum ether:EtOAc = 10:1) to afford **1t** (320 mg, 47%) as a yellow solid.

Method N:

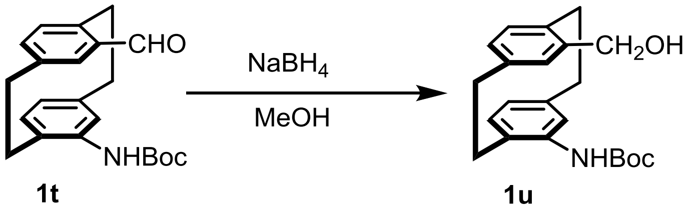

Substrate **1u** was synthesized with Method N

General procedure of **method N**:

Synthesis of 1ad： To a solution of **1t** (140 mg, 0.34 mmol, 1.0 equiv.) in MeOH (3 mL) was added $NaBH_4$ (26 mg, 0.68 mmol, 2.0 equiv.) under N_2 atmosphere. After stirring at rt for 2 h, the reaction mixture was quenched with H_2O and extracted with EtOAc (3×15 mL). The combined organic layers were then washed with brine (10 mL), dried over Na_2SO_4, filtered and concentrated under vacuum to give a residue, which was purified by column chromatography (petroleum ether:EtOAc = 5:1) to afford **1u** (130 mg, 93%) as a white solid.

Method O:

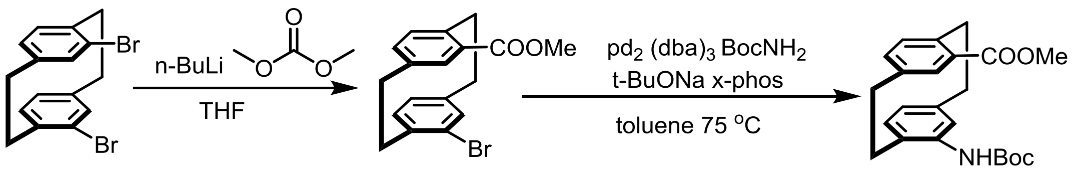

Substrate **1v** was synthesized with Method O

General procedure of **method O**:

Synthesis of S17: To a solution of **S11** (366 mg, 1.0 mmol, 1.0 equiv.) in anhydrous THF (10 mL) was added n-BuLi (2.4 M, 0.46 mL, 1.1 mmol, 1.1 equiv.) under N_2 atmosphere at -78°C. After stirring at rt for 1 h, dimethyl carbonate (0.42 mL, 5.0 mmol, 5.0 equiv.) was added under N_2 atmosphere at -78°C. The mixture was warmed to rt, stirred at rt for 3 h. The reaction mixture was quenched with saturated NH_4Cl solution (10 mL), extracted with EtOAc (3×15 mL). The combined organic layers were then washed with brine (10 mL), dried over Na_2SO_4, filtered and concentrated under vacuum to give a residue, which was purified by column chromatography (petroleum ether:EtOAc = 100:1) to afford **S17** (300 mg, 87%) as a yellow solid.

Synthesis of 1v: To a solution of **S17** (300 mg, 0.87 mmol, 1.0 equiv.), $BocNH_2$(123 mg, 1.05 mmol, 1.2 equiv.), t-BuONa (117 mg, 1.22 mmol, 1.4 equiv.), x-Phos (62 mg, 0.13 mmol, 0.15 equiv.) in toluene (8 mL) was added $Pd_2(dba)_3$ (40 mg, 0.04 mmol, 0.05 equiv.) under N_2 atmosphere. After stirring at 75 ^{o}C under N_2 atmosphere overnight, the reaction mixture was cooled to rt and concentrated under vacuum to give a residue, which was purified by column chromatography (petroleum ether:EtOAc = 10:1) to afford **1v** (150 mg, 45%) as a yellow solid.

Tert-butyl 1,4(1,4)-dibenzenacyclohexaphane-1^2-ylcarbamate **(1a)**

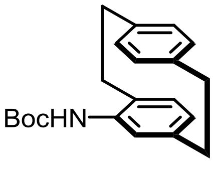

This reaction was performed on 3.48 mmol scale of **S1** according to **method A**, which gave the product **1a** (820 mg, 73% yield) as a yellow solid.

^{1}H NMR (400 MHz, $CDCl_3$) δ 6.80 – 6.77 (m, 1H), 6.72 (s, 1H), 6.55 – 6.49 (m, 2H), 6.44 – 6.39 (m, 2H), 6.38 – 6.35 (m, 1H), 6.24 (s, 1H), 3.27 – 3.19 (m, 1H), 3.18 – 3.10 (m, 1H), 3.08 – 2.96 (m, 5H), 2.82 – 2.72 (m, 1H), 1.56 (s, 9H). ^{13}C NMR (101 MHz, $CDCl_3$) δ 152.9, 141.2, 139.4, 138.9, 137.2, 135.1, 133.3, 133.1, 132.3, 129.0, 128.3, 127.9, 125.2, 80.45, 35.4, 35.1, 33.8, 33.0, 28.5. m/z HRMS (ESI) found $[M-56+H]^+$ 268.1334, $C_{17}H_{18}NO_2^+$ calculated 268.1332.

Methyl 1,4(1,4)-dibenzenacyclohexaphane-1^2-ylcarbamate **(1b)**

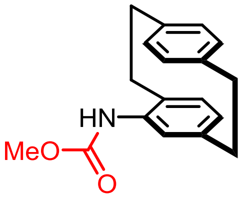

This reaction was performed on 2 mmol scale of **S1** according to **method A**, which gave the product **1b** (150 mg, 27% yield) as a brown solid

^{1}H NMR (400 MHz, $CDCl_3$) δ 6.79 – 6.78 (m, 2H), 6.56 – 6.53 (m, 1H), 6.52 – 6.47 (m, 1H), 6.45 – 6.32 (m, 4H), 3.81 (s, 3H), 3.25 – 3.17 (m, 1H), 3.17 – 3.06 (m, 2H), 3.05 – 3.00 (m, 3H), 3.00 – 2.97 (m, 1H), 2.83 – 2.76 (m, 1H). ^{13}C NMR (101 MHz, $CDCl_3$) δ 154.1, 141.4, 139.5, 138.9, 136.7, 135.2, 133.3, 133.2, 132.3, 128.4, 128.3, 125.4, 52.5, 35.4, 35.1, 33.8, 32.8. m/z HRMS (ESI) found $[M+H]^+$ 282.1490, $C_{18}H_{20}NO_2^+$ calculated 282.1489.

Ethyl 1,4(1,4)-dibenzenacyclohexaphane-1^2-ylcarbamate **(1c)**

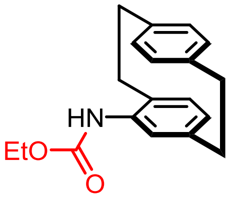

This reaction was performed on 2 mmol scale of **S1** according to **method A**, which gave the product **1c** (336 mg, 57% yield) as a white solid.

^{1}H NMR (400 MHz, $CDCl_3$) δ 6.79 – 6.70 (m, 2H), 6.56 –5.56 (m, 2H), 6.46 – 6.26 (m, 4H), 4.26 (q, J = 7.1 Hz, 2H), 3.25 –3.21 (m, 1H), 3.17 –3.11 (m, 1H), 3.09 – 2.97 (m, 5H), 2.82 – 2.74 (m, 1H), 1.36 (t, J = 7.1 Hz, 3H). ^{13}C NMR (101 MHz, $CDCl_3$) δ 153.67, 141.3, 139.4, 138.9, 136.8, 135.2, 133.3, 133.2, 132.3, 128.3, 125.4, 61.3, 35.4, 35.1, 33.8, 32.8, 14.8. m/z HRMS (ESI) found $[M+H]^+$ 296.1648, $C_{19}H_{22}NO_2^+$ calculated 296.1645.

Benzyl 1,4(1,4)-dibenzenacyclohexaphane-1^2-ylcarbamate **(1d)**

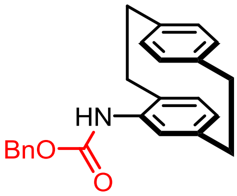

This reaction was performed on 2 mmol scale of **S1** according to **method A**, which gave the product **1d** (560 mg, 78% yield) as a white solid.

^{1}H NMR (400 MHz, $CDCl_3$) δ 7.52 – 7.32 (m, 5H), 6.85 – 6.69 (m, 2H), 6.55 – 6.53 (m, 1H), 6.49 – 6.35 (m, 5H), 5.24 (s, 2H), 3.24 – 3.06 (m, 3H), 3.05 – 2.96 (m, 4H), 2.82 – 2.72 (m, 1H). ^{13}C NMR (101 MHz, $CDCl_3$) δ 153.4, 141.4, 139.5, 138.9, 136.7, 136.3, 135.2, 133.3, 133.2, 132.3, 128.8, 128.6, 128.5, 128.3, 125.4, 67.2, 35.4, 35.2, 33.8, 32.8. m/z HRMS (ESI) found $[M+H]^+$ 358.1802, $C_{24}H_{24}NO_2^+$ calculated 358.1802.

(9*H*-fluoren-9-yl)methyl 1,4(1,4)-dibenzenacyclohexaphane-1^2-ylcarbamate **(1e)**

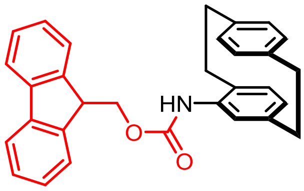

This reaction was performed on 1 mmol scale of **1a** according to **synthesis of 1e**, which gave the product **1e** (220 mg, 90% yield for two steps) as a white solid

^{1}H NMR (400 MHz, $CDCl_3$) δ 7.82 – 7.80 (m, 2H), 7.66 (s, 2H), 7.47 – 7.40 (m, 2H), 7.39 – 7.31 (m, 2H), 6.72 (s, 1H), 6.65 –6.62 (m, 1H), 6.54 –6.52 (m, 1H), 6.47 – 6.38

(m, 4H), 6.35 (s, 1H), 4.69 – 4.57 (m, 2H), 4.32 (t, J = 8 Hz, 1H), 3.23 – 3.08 (m, 2H), 3.07 – 2.88 (m, 5H), 2.80 – 2.72 (m, 1H). ^{13}C NMR (101 MHz, $CDCl_3$) δ 153.4, 144.0, 143.8, 141.6, 141.4, 139.4, 138.9, 136.5, 135.3, 133.3, 133.2, 132.3, 128.6, 128.3, 128.0, 127.9, 127.3, 125.1, 120.2, 66.7, 47.5, 35.3, 35.1, 33.8, 32.9. m/z HRMS (ESI) found $[M+H]^+$ 446.2113, $C_{31}H_{28}NO_2^+$ calculated 446.2115.

Tert-butyl (4^2-bromo-1,4(1,4)-dibenzenacyclohexaphane-1^2-yl)carbamate **(1f)**

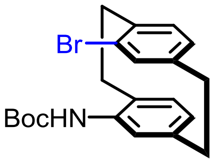

This reaction was performed on 1.03 mmol scale of **S5** according to **Synthesis of 1f**, which gave the product **1f** (350 mg, 85% yield) as a white solid

^{1}H NMR (400 MHz, $CDCl_3$) δ 6.96 (s, 1H), 6.78 (s, 1H), 6.55 (s, 1H), 6.52 – 6.44 (m, 3H), 6.38 –6.35 (m, 1H), 3.63– 3.56 (m, 1H), 3.45 – 3.32 (m, 1H), 3.11 – 2.87 (m, 6H), 1.54 (s, 9H). ^{13}C NMR (101 MHz, $CDCl_3$) δ 152.8, 141.5, 140.5, 138.2, 137.52, 136.0, 135.3, 135.0, 132.6, 128.7, 124.2, 123.5, 80.2, 35.1, 34., 34.33, 31.4, 28.6. m/z HRMS (ESI) found $[M-56+H]^+$ 346.0433, $C_{17}H_{17}BrNO_2^+$ calculated 346.0437.

Tert-butyl (4^2-phenyl-1,4(1,4)-dibenzenacyclohexaphane-1^2-yl)carbamate **(1g)**

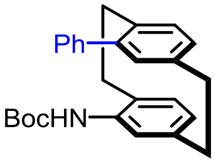

This reaction was performed on 0.5 mmol scale of **1f** according to **Synthesis of 1g**, which gave the product **1g** (180 mg, 90% yield) as a white solid

^{1}H NMR (500 MHz, $CDCl_3$) δ 7.54 – 7.49 (m, 2H), 7.43 –7.40 (m, 2H), 7.33 – 7.30 (m, 1H), 6.83 —6.82 (m, 1H), 6.71 (s, 1H), 6.60 – 5.56 (m, 2H), 6.50 – 6.47 (m, 2H), 5.87 (s, 1H), 3.39 – 3.31 (m, 1H), 3.28 – 3.21 (m, 1H), 3.14 – 3.00 (m, 5H), 2.84 – 2.73 (m, 1H), 1.39 (s, 9H). ^{13}C NMR (126 MHz, $CDCl_3$) δ 152.4, 140.8, 140.7, 140.6, 138.9, 137.3, 136.3, 135.8, 135.1, 132.9, 130.3, 129.3, 128.6, 128.5, 127.6, 126.8, 125.2, 79.9, 35.1, 35.1, 33.8, 31.7, 28.4. m/z HRMS (ESI) found $[M-56+H]^+$ 344.1649, $C_{23}H_{22}NO_2^+$

calculated 344.1645.

Tert-butyl (4^2-(4-methoxyphenyl)-1,4(1,4)-dibenzenacyclohexaphane-1^2-yl)carbamate **(1h)**

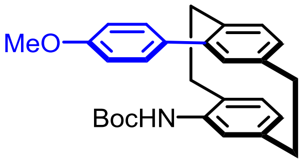

This reaction was performed on 2 mmol scale of **1f** according to **Method I**, which gave the product **1h** (510 mg, 59% yield) as a white solid

^{1}H NMR (400 MHz, $CDCl_3$) δ 7.45 – 7.43 (m, 2H), 6.97 – 6.95 (m, 2H), 6.79 – 6.78 (m, 1H), 6.68 (s, 1H), 6.58 – 6.56 (m, 2H), 6.49 – 6.45 (m, 2H), 5.88 (s, 1H), 3.86 (s, 3H), 3.40 – 3.31 (m, 1H), 3.24 – 3.18 (m, 1H), 3.13 – 2.99 (m, 5H), 2.81 – 2.73 (m, 1H), 1.40 (s, 9H). ^{13}C NMR (101 MHz, $CDCl_3$) δ 158., 152.43, 140.8, 140.3, 138.9, 137.2, 136.1, 135.7, 135.1, 133.3, 132.6, 130.3, 130.0, 129.1, 127.8, 125.4, 114.0, 79.9, 55.4, 35.1, 35.1, 33.8, 31.7, 28.5. m/z HRMS (ESI) found $[M-56+H]^+$ 374.1758 $C_{24}H_{24}NO_3^+$ calculated 374.1751.

Tert-butyl (E)-(4^2-styryl-1,4(1,4)-dibenzenacyclohexaphane-1^2-yl)carbamate **(1i)**

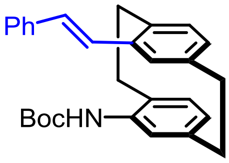

This reaction was performed on 1.5 mmol scale of **1f** according to **Method C**, which gave the product **1i** (360 mg, 74% yield for two steps) as a white solid

^{1}H NMR (400 MHz, $CDCl_3$) δ 7.55 – 7.53 (m, 2H), 7.45 – 7.32 (m, 3H), 7.27 – 7.25 (m, 1H), 6.90 (d, J = 16.1 Hz, 1H), 6.83 – 6.82 (m, 1H), 6.55 – 6.53 (m, 2H), 6.50 – 6.38 (m, 3H), 6.28 (s, 1H), 3.72 – 3.62 (m, 1H), 3.35 – 3.29 (m, 1H), 3.15 – 2.90 (m, 6H), 1.31 (s, 9H). ^{13}C NMR (101 MHz, $CDCl_3$) δ 152.7, 140.6, 139.6, 137.9, 137.7, 137.0, 135.9, 135.2, 134.9, 132.6, 130.6, 129.5, 129.2, 128.7, 128.2, 127.5, 126.7, 126.6, 125.9, 80.1, 35.12, 35.0, 32.2, 31.8, 28.2. $[\alpha]^{25}_D$ = 126.2 (c = 0.5, $CHCl_3$). m/z HRMS (ESI) found $[M-56+H]^+$ 370.1808 $C_{25}H_{24}NO_2^+$calculated 370.1802. HPLC:

Chiralpak IC column, 70:30 hexanes/isopropanol, 1 ml/min; t_R = 6.20 min (minor), 10.56 min (major); 96.4% ee.

Tert-butyl (4^2-(phenylethynyl)-1,4(1,4)-dibenzenacyclohexaphane-1^2-yl)carbamate **(1j)**

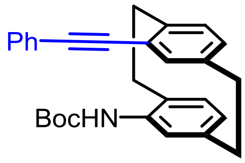

This reaction was performed on 1.5 mmol scale of **1f** according to **Method D**, which gave the product **1j** (150 mg, 24% yield) as a white solid

^{1}H NMR (400 MHz, $CDCl_3$) δ 7.65 – 7.55 (m, 2H), 7.41 – 7.31 (m, 3H), 6.83 (s, 1H), 6.78 (s, 1H), 6.59 – 6.45 (m, 4H), 6.44 – 6.41(m, 1H), 3.84 – 3.77 (m, 1H), 3.53 – 3.47 (m, 1H), 3.16 – 3.01 (m, 5H), 2.99 – 2.91 (m, 1H), 1.30 (s, 9H). ^{13}C NMR (101 MHz, $CDCl_3$) δ 153.3, 142.1, 140.5, 139.5, 137.6, 135.8, 135.2, 134.3, 133.9, 132.5, 131.7, 129.7, 128.5, 128.2, 128.1, 123.7, 121.8, 92.1, 89.2, 79.9, 35.0, 35.0, 32.7, 32.6, 28.3. m/z HRMS (ESI) found $[M-56+H]^+$ 368.1650 $C_{25}H_{22}NO_2^+$ calculated 368.1645.

Tert-butyl (4^2-cyclohexyl-1,4(1,4)-dibenzenacyclohexaphane-1^2-yl)carbamate **(1k)**

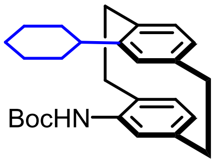

This reaction was performed on 1.5 mmol scale of **1f** according to **Method E**, which gave the product **1k** (200 mg, 43% yield for two steps) as a white solid

^{1}H NMR (400 MHz, $CDCl_3$) δ 6.69 (s, 1H), 6.45 (d, J = 7.7 Hz, 1H), 6.41 (s,, 2H), 6.35 – 6.30 (m, 2H), 6.24 (s, 1H), 3.47 – 3.37 (m, 1H), 3.27 – 3.19 (m, 1H), 3.12 – 3.00 (m, 2H), 2.99 – 2.92 (m, 3H), 2.90 – 2.81 (m, 1H), 2.10 – 1.93 (m, 2H), 1.79 – 1.66 (m, 3H), 1.58 – 1.54 (m, 2H), 1.51 (s, 9H), 1.50 – 1.43 (s, 2H), 1.23 – 1.09 (m, 1H), 0.85 – 0.76 (m, 1H). ^{13}C NMR (101 MHz, $CDCl_3$) δ 152.6, 145.8, 140.9, 139.3, 136.7, 136.5, 135.1, 134.7, 130.9, 128.3, 127.7, 124.2, 124.1, 80.4, 40.5, 38.4, 35.3,

35.2, 31.3, 31.23, 30.8, 28.5, 27.6, 27.5, 26.5. m/z HRMS (ESI) found $[M+H]^+$ 406.2749 $C_{27}H_{36}NO_2^+$ calculated 406.2741.

4^2-((tert-butoxycarbonyl)amino)-1,4(1,4)-dibenzenacyclohexaphane-1^2-yl acetate **(1l)**

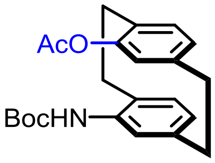

This reaction was performed on 2.5 mmol scale of **1f** according to **Method F**, which gave the product **1l** (360 mg, 34% yield for two steps) as a white solid

^1H NMR (400 MHz, $CDCl_3$) δ 6.68 – 6.51 (m, 2H), 6.55 – 6.43 (m, 3H), 6.40 – 6.37 (m, 1H), 6.25 (s, 1H), 3.35 – 3.27 (m, 1H), 3.25 – 3.14 (m, 1H), 3.03 – 3.01 (m, 4H), 2.92 – 2.83 (m, 2H), 2.33 (s, 3H), 1.53 (s, 9H). ^{13}C NMR (101 MHz, $CDCl_3$) δ 168.8, 153.0, 149.7, 141.3, 140.5, 136.7, 135.3, 135.2, 131.4, 130.6, 129.50, 126.3, 80.1, 35.1, 34.9, 31.9, 29.3, 28.6, 21.1. m/z HRMS (ESI) found $[M-100+H]^+$ 282.1482 $C_{18}H_{20}NO_2^+$ calculated 282.1489.

Tert-butyl (4^3-bromo-1,4(1,4)-dibenzenacyclohexaphane-1^2-yl)carbamate **(1m)**

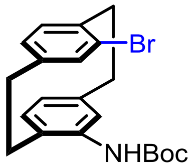

This reaction was performed on 7 mmol scale of **S13** according to **Method H**, which gave the product **1m** (900 mg, 32% yield) as a white solid

^1H NMR (400 MHz, $CDCl_3$) δ 7.00 (s, 1H), 6.90 (s, 1H), 6.56 – 6.45 (m, 3H), 6.39 – 6.37 (m, 1H), 6.17 (s, 1H), 3.43 – 3.37 (m, 1H), 3.31 – 3,25 (m, 1H), 3.14 – 2.97 (m, 3H), 2.96 – 2.72 (m, 3H), 1.54 (s, 9H). ^{13}C NMR (101 MHz, $CDCl_3$) δ 152.8, 141.6, 140.8, 138.8, 136.5, 135.4, 135.1, 132.4 , 131.9, 131.0, 128.8, 126.3, 122.3, 80.7, 35.5, 33.3, 33.1, 32.9, 28.5.m/z HRMS (ESI) found $[M-56+H]^+$ 346.0429 $C_{17}H_{17}BrNO_2^+$calculated 346.0437.

Tert-butyl (4^3-phenyl-1,4(1,4)-dibenzenacyclohexaphane-1^2-yl)carbamate **(1n)**

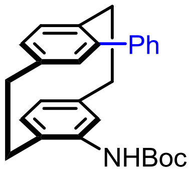

This reaction was performed on 0.6 mmol scale of **1m** according to **Method I**, which gave the product **1n** (230 mg, 96% yield) as a white solid

^{1}H NMR (500 MHz, $CDCl_3$) δ 7.43 – 7.40 (m, 4H), 7.34 – 7.31 (m, 1H), 6.90 (s, 1H), 6.85 (s, 1H), 6.67 (d, *J* = 7.7 Hz, 1H), 6.53 (d, *J* = 7.7 Hz, 1H), 6.47 – 6.45 (m, 1H), 6.41 – 6.39 (m, 1H), 6.35 (s, 1H), 3.47 – 3.45 (m, 1H), 3.29 – 3.21 (m, 2H), 3.03 – 2.95 (m, 1H), 2.90 – 2.80 (m, 3H), 2.53 – 2.44 (m, 1H), 1.61 (s, 9H). ^{13}C NMR (126 MHz, $CDCl_3$) δ 152.8, 141.8, 141.5, 141.0, 138.6, 137.1, 136.7, 135.8, 134.8, 132.2, 129.6, 128.6, 128.3, 127.3, 126.9, 122.5, 80.7, 34.3, 34.2, 33.0, 32.4, 28.5. m/z HRMS (ESI) found $[M-56+H]^+$ 344.1651 $C_{23}H_{22}NO_2^+$ calculated 344.1645.

Tert-butyl (4^3-(cyclohex-1-en-1-yl)-1,4(1,4)-dibenzenacyclohexaphane-1^2 -yl)carbamate **(1o)**

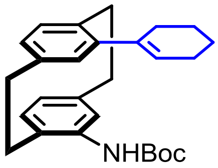

This reaction was performed on 1.4 mmol scale of **1m** according to **Method E**, which gave the product **1o** (390 mg, 70% yield) as a white solid

^{1}H NMR (500 MHz, $CDCl_3$) δ 6.74 (s, 1H), 6.69 (s, 1H), 6.51 – 6.49 (m, 2H), 6.41 – 6.39 (m, 1H), 6.30 – 6.28 (m, 1H), 6.18 (s, 1H), 5.75 – 5.74 (m, 1H), 3.34 – 3.29 (m, 1H), 3.22 – 3.12 (m, 2H), 2.95 – 2.84 (m, 4H), 2.84 – 2.76 (m, 1H), 2.41 – 2.37 (m, 1H), 2.31 – 2.16 (m, 2H), 2.12 – 2.09 (m, 1H), 1.75 – 1.71 (m, 2H), 1.69 – 1.60 (m, 2H), 1.53 (s, 9H). ^{13}C NMR (126 MHz, $CDCl_3$) δ 152.6, 143.9, 141.3, 138.3, 137.9, 136.7, 136.5, 135.1, 134.5, 131.6, 127.4, 127.2, 126.4, 123.1, 80.5, 34.6, 34.4, 33.0,

32.3, 29.9, 28.4, 26.0, 23.5, 22.4. m/z HRMS (ESI) found $[M+H]^+$ 404.2592 $C_{27}H_{34}NO_2^+$ calculated 404.2584.

Tert-butyl (E)-(4^3-styryl-1,4(1,4)-dibenzenacyclohexaphane-1^2-yl)carbamate **(1p)**

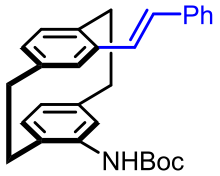

This reaction was performed on 0.9 mmol scale of **1m** according to **Method C**, which gave the product **1p** (200 mg, 52% yield for two steps) as a yellow solid

^{1}H NMR (400 MHz, $CDCl_3$) δ 7.56 – 7.54 (m, 2H), 7.40 – 7.36 (m, 2H), 7.29 – 7.25 (m, 1H), 7.15 (d, J = 16.1 Hz, 1H), 7.04 (s, 1H), 6.90 (d, J = 16.1 Hz, 1H), 6.78 (s, 1H), 6.55 – 6.53 (m, 1H), 6.51 – 6.43 (m, 2H), 6.40 – 6.38 (m, 1H), 6.15 (s, 1H), 3.64 – 3.52 (m, 1H), 3.30 – 3.24 (m, 1H), 3.17 – 3.08 (m, 2H), 3.03 – 2.96 (m, 1H), 2.93 – 2.71 (m, 3H), 1.48 (s, 9H). ^{13}C NMR (101 MHz, $CDCl_3$) δ 152.7, 140.9, 139.6, 138.0, 138.0, 137.4, 136.7, 135.3, 135.1, 132.1, 129.3, 129.1, 128.7, 127.9, 127.6, 127.0, 126.7, 125.7, 123.1, 80.5, 34.4, 33.8, 33.4, 33.0, 28.4. m/z HRMS (ESI) found $[M-56+H]^+$ 370.1790 $C_{25}H_{24}NO_2^+$ calculated 370.1802.

Tert-butyl (4^3-(phenylethynyl)-1,4(1,4)-dibenzenacyclohexaphane-1^2-yl)carbamate **(1q)**

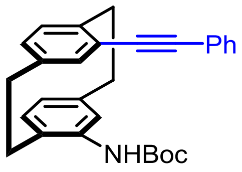

This reaction was performed on 1.6 mmol scale of **1m** according to **Method K**, which gave the product **1q** (200 mg, 30% yield for three steps) as a white solid

^{1}H NMR (400 MHz, $CDCl_3$) δ 7.65 – 7.56 (m, 2H), 7.40 – 7.35 (m, 3H), 6.95 (d, J = 1.9 Hz, 1H), 6.88 (s, 1H), 6.59 – 6.57 (m, 1H), 6.51 – 6.48 (m, 2H), 6.42 – 6.39 (m, 1H), 6.19 (s, 1H), 3.64 – 3.58 (m, 1H), 3.32 – 3.25 (m, 1H), 3.18 – 3.11 (m, 1H), 3.09

– 2.94 (m, 3H), 2.92 – 2.73 (m, 2H), 1.51 (s, 9H). ^{13}C NMR (101 MHz, $CDCl_3$) δ 152.8, 142.2, 141.0, 139.6, 136.5, 135.1, 133.9, 133.2, 132.1, 131.6, 131.0, 128.9, 128.5, 128.3, 124.5, 123.9, 123.8, 92.9, 89.2, 80.5, 34.3, 33.8, 33.4, 33.1, 28.4. m/z HRMS (ESI) found $[M-56+H]^+$ 368.1631 $C_{25}H_{22}NO_2^+$ calculated 368.1645.

Tert-butyl (4^3-cyclohexyl-1,4(1,4)-dibenzenacyclohexaphane-1^2-yl)carbamate **(1r)**

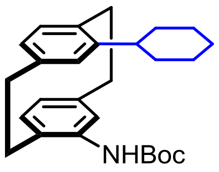

This reaction was performed on 0.55 mmol scale of **1o** according to **Method E**, which gave the product **1r** (210 mg, 96% yield) as a white solid

^{1}H NMR (400 MHz, $CDCl_3$) δ 6.75 (s, 1H), 6.63 (s, 1H), 6.48 (d, J = 7.6 Hz, 1H), 6.43 (d, J = 7.7 Hz, 1H), 6.36 – 6.30 (m, 2H), 6.21 (s, 1H), 3.39 – 3.33 (m, 1H), 3.20 – 3.14 (m, 2H), 3.10 – 2.99 (m, 1H), 2.99 – 2,89 (m, 2H), 2.89 – 2.71 (m, 2H), 2.51 – 2.40 (m, 1H), 2.01 – 1.90 (m, 2H), 1.81 – 1.68 (m, 3H), 1.52 (s, 9H), 1.50 – 1.32 (m, 3H), 1.22 – 1.11 (m, 1H), 0.97 – 0.84 (m, 1H). ^{13}C NMR (101 MHz, $CDCl_3$) δ 152.5, 146.2, 141.2, 138.7, 137.1, 136.9, 135.2, 135.1, 130.5, 126.9, 124.6, 122.6, 80.4, 40.5, 37.3, 34.2, 33.5, 33.3, 32.3, 30.1, 28.4, 27.6, 27.0, 26.5. m/z HRMS (ESI) found $[M+H]^+$ 406.2727 $C_{27}H_{36}NO_2^+$ calculated 406.2741.

4^3-((tert-butoxycarbonyl)amino)-1,4(1,4)-dibenzenacyclohexaphane-1^2-yl acetate **(1s)**

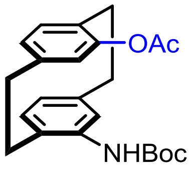

This reaction was performed on 0.82 mmol scale of **S11** according to **Method L**, which gave the product **1s** (70 mg, 20% yield for three steps) as a white solid

^{1}H NMR (400 MHz, $CDCl_3$) δ 6.99 (s, 1H), 6.56 – 6.54 (m, 2H), 6.50 – 6.45 (m, 1H), 6.45 – 6.37 (m, 2H), 6.36 – 6.32 (m, 1H), 3.28 – 3.14 (m, 2H), 3.12 – 3.04 (m, 1H),

3.03 – 2.88 (m, 3H), 2.80 – 2.63 (m, 2H), 2.34 (s, 3H), 1.54 (s, 1H). ^{13}C NMR (101 MHz, $CDCl_3$) δ 169.1, 152.8, 149.2, 141.3, 141.1, 137.0, 135.4, 135.3, 130.2, 130.1, 128.3, 122.5, 80.4, 33.8, 33.2, 33.0, 31.3, 28.5, 21.5. m/z HRMS (ESI) found $[M-56+H]^+$ 326.1380 $C_{19}H_{20}NO_4^+$ calculated 326.1387.

Tert-butyl (4^3-formyl-1,4(1,4)-dibenzenacyclohexaphane-1^2-yl)carbamate **(1t)**

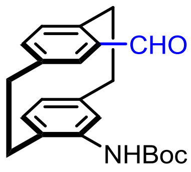

This reaction was performed on 2.73 mmol scale of **S11** according to **Method M**, which gave the product **1t** (320 mg, 33% yield for two steps) as a white solid

^{1}H NMR (400 MHz, $CDCl_3$) δ 9.89 (s, 1H), 7.30 (s, 1H), 6.70 – 6.60 (m, 2H), 6.53 (s, 1H), 6.48 – 6.40 (m, 2H), 6.21 (s, 1H), 4.13 – 4.04 (m, 1H), 3.35 – 3.29 (m, 1H), 3.24 – 3.04 (m, 3H), 3.00 – 2.87 (m, 2H), 2.83 – 2.75 (m, 1H), 1.55 (s, 9H). ^{13}C NMR (101 MHz, $CDCl_3$) δ 192.3, 152.6, 142.6, 141.1, 140.0, 138.1, 137.0, 136.6, 136.0, 135.2, 132.0, 129.5, 127.8, 124.7, 80.6, 34.5, 33.4, 33.1, 32.9, 28.4. m/z HRMS (ESI) found $[M+H]^+$ 352.1914 $C_{22}H_{26}NO_3^+$ calculated 352.1907.

Tert-butyl (4^3-(hydroxymethyl)-1,4(1,4)-dibenzenacyclohexaphane-1^2-yl)carbamate **(1u)**

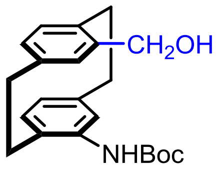

This reaction was performed on 0.34 mmol scale of **1t** according to **Method N**, which gave the product **1u** (130 mg, 93% yield) as a white solid

^{1}H NMR (400 MHz, $CDCl_3$) δ 7.15 (s, 1H), 6.71 (s, 1H), 6.50 – 4.45 (m, 2H), 6.42 – 6.37 (m, 3H), 4.69 – 4.64 (m, 1H), 4.58 – 4.52 (m, 1H), 3.61 (s, 1H), 3.37 – 3.15 (m, 3H), 3.13 – 2.91 (m, 3H), 2.88 – 2.69 (m, 2H), 1.59 (s, 9H). ^{13}C NMR (101 MHz,

$CDCl_3$) δ 154.0, 141.3, 140.5, 139.2, 137.0, 136.1, 135.5, 134.7, 131.4, 128.3, 128.1, 126.3, 120.6, 81.3, 62.9, 33.8, 33.3, 33.0, 32.8, 28.6. m/z HRMS (ESI) found $[M-18+H]^+$ 336.1943 $C_{22}H_{26}NO_2^+$ calculated 336.1958.

Methyl 4^3-((tert-butoxycarbonyl)amino)-1,4(1,4)-dibenzenacyclohexaphane-1^2 -carboxylate **(1v)**

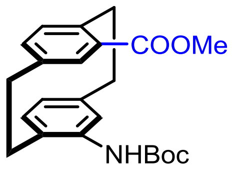

This reaction was performed on 1.0 mmol scale of **1m** according to **Method O**, which gave the product **1v** (150 mg, 39% yield for two steps) as a white solid

^{1}H NMR (400 MHz, $CDCl_3$) δ 7.42 (s, 1H), 6.60 (s, 2H), 6.53 (s, 1H), 6.48 – 6.39 (m, 2H), 6.36 (s, 1H), 4.01 – 3.94 (m, 1H), 3.91 (s, 3H), 3.33 – 3.25 (m, 1H), 3.15 – 2.97 (m, 4H), 2.88 – 2.72 (m, 2H), 1.54 (s, 9H). ^{13}C NMR (101 MHz, $CDCl_3$) δ 168.0, 152.7, 141.8, 141.4, 139.6, 136.9, 136.6, 135.9, 135.2, 131.0, 130.6, 128.2, 124.5, 80.3, 52.1, 35.6, 34.5, 33.2, 33.1, 28.5. m/z HRMS (ESI) found $[M-56+H]^+$ 326.1385 $C_{19}H_{20}NO_4^+$ calculated 326.1387.

Tert-butyl (4^2-bromo-1,4(1,4)-dibenzenacyclohexaphane-1^2-yl)carbamate **(1w)**

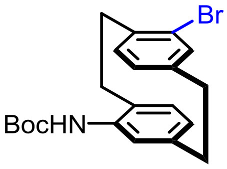

This reaction was performed on 2.5 mmol scale of **S10** according to **Method H**, which gave the product **1w** (320 mg, 32% yield) as a white solid

^{1}H NMR (400 MHz, $CDCl_3$) δ 7.09 (d, J = 7.8 Hz, 1H), 6.93 (s, 1H), 6.77 (d, J = 7.6 Hz, 1H), 6.55 – 6.52 (m, 2H), 6.32 – 6.30 (m, 1H), 6.25 (s, 1H), 3.41 – 3.31 (m, 1H), 3.11 – 2.98 (m, 5H), 2.95 – 2.84 (m, 2H), 1.56 (s, 9H). ^{13}C NMR (101 MHz, $CDCl_3$) δ 152.8, 141.5, 140.8, 138.4, 138.1, 136.8, 131.3, 130.5, 130.0, 127.8, 127.2, 127.1,

124.0, 80.6, 35.0, 34.5, 34.1, 30.3, 28.5. m/z HRMS (ESI) found $[M-56+H]^+$ 346.0437 $C_{17}H_{17}BrNO_2^+$calculated 346.0437.

Tert-butyl (4^2-phenyl-1,4(1,4)-dibenzenacyclohexaphane-1^2-yl)carbamate **(1x)**

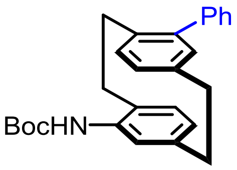

This reaction was performed on 1.0 mmol scale of **1w** according to **Method I**, which gave the product **1x** (383 mg, 96% yield) as a white solid

^1H NMR (400 MHz, $CDCl_3$) δ 7.53 – 7.43 (m, 4H), 7.39 – 7.32 (m, 1H), 7.02 – 6.85 (m, 2H), 6.64 – 6.53 (m, 3H), 6.45 – 6.42 (m, 1H), 6.26 (s, 1H), 3.55 – 3.50 (m, 1H), 3.19 – 2.84 (m, 6H), 2.33 – 2.21 (m, 1H), 1.57 (s, 9H). ^{13}C NMR (101 MHz, $CDCl_3$) δ 152.9, 142.0, 141.2, 141.1, 139.7, 137.8, 136.5, 132.4, 131.6, 131.6, 131.1, 129.7, 128.6, 127.0, 126.9, 124.5, 80.5, 35.1, 35.0, 33.0, 31.6, 28.5. m/z HRMS (ESI) found $[M-56]^+$ 344.1640 $C_{23}H_{22}NO_2^+$ calculated 344.1645.

Tert-butyl (4^2-(cyclohex-1-en-1-yl)-1,4(1,4)-dibenzenacyclohexaphane
-1^2yl)carbamate **(1y)**

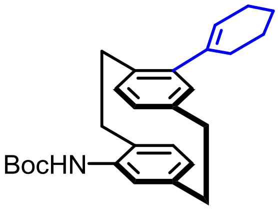

This reaction was performed on 1.5 mmol scale of **1w** according to **Method I**, which gave the product **1y** (600 mg, 99% yield) as a white solid.

^1H NMR (400 MHz, $CDCl_3$) δ 6.86 (s, 1H), 6.74 – 6.72 (m, 1H), 6.57 (d, J = 7.8 Hz, 1H), 6.40 – 6.38 (m, 2H), 6.30 – 6.18 (m, 2H), 5.92 – 5.85 (m, 1H), 3.38 – 3.32 (m, 1H), 3.11 – 2.85 (m, 6H), 2.76 – 2.69 (m, 1H), 2.47 – 2.43 (m, 1H), 2.28 – 2.23 (m, 2H), 2.17 – 2.12 (m, 1H), 1.83 – 1.63 (m, 4H), 1.56 (s, 9H). ^{13}C NMR (101 MHz, $CDCl_3$) δ 153.07, 144.2, 141.0, 139.1, 138.6, 137.4, 136.3, 132.2, 131.0, 130.7, 130.6,

129.0, 127.3, 127.2, 124.7, 80.4, 35.0, 34.9, 33.6, 32.0, 29.7, 28.5, 26.0, 23.5, 22.5. m/z HRMS (ESI) found $[M+H]^+$ 404.2585 $C_{27}H_{34}NO_2^+$ calculated 404.2584.

Tert-butyl (4^2-cyclohexyl-1,4(1,4)-dibenzenacyclohexaphane-1^2-yl)carbamate **(1z)**

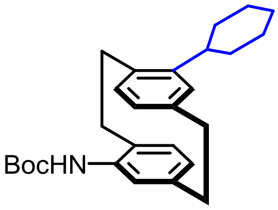

This reaction was performed on 0.77 mmol scale of **1y** according to **Method E**, which gave the product **1z** (230 mg, 74% yield) as a white solid.

^{1}H NMR (400 MHz, $CDCl_3$) δ 6.78 (s, 1H), 6.73 (d, *J* = 7.6 Hz, 1H), 6.44 (d, *J* = 7.7 Hz, 1H), 6.39 – 6.36 (m, 1H), 6.32 – 6.18 (m, 3H), 3.40 – 3.26 (m, 1H), 3.25 – 3.12 (m, 1H), 3.06 – 2.84 (m, 6H), 2.39 – 2.35 (m, 1H), 2.08 – 2.05 (m, 1H), 2.02 – 1.93 (m, 1H), 1.80 – 1.71 (m, 3H), 1.56 (s, 9H), 1.52 – 1.39 (m, 3H), 1.20 (s, 1H), 0.91 – 0.83 (m, 1H). ^{13}C NMR (101 MHz, $CDCl_3$) δ 152.9, 146.8, 141.2, 139.6, 137.4, 136.4, 131.6, 129.9, 129.0, 127.6, 125.2, 80.4, 40.8, 36.9, 35.0, 34.8, 32.1, 31.7, 30.4, 28.5, 27.7, 27.2, 26.5. m/z HRMS (ESI) found $[M+H]^+$ 406.2740 $C_{27}H_{36}NO_2^+$ calculated 406.2741.

Tert-butyl (4^3-bromo-1,4(1,4)-dibenzenacyclohexaphane-1^2-yl)carbamate **(1aa)**

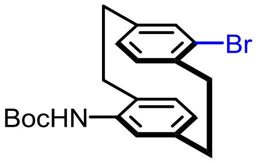

This reaction was performed on 1.0 mmol scale of **S9** according to **Method H**, which gave the product **1aa** (100 mg, 25% yield) as a white solid

^{1}H NMR (400 MHz, $CDCl_3$) δ 6.95 –6.93 (m, 1H), 6.82 – 6.71 (m, 2H), 6.53 – 6.45 (m, 2H), 6.39 (d, *J* = 7.8 Hz, 1H), 6.27 (s, 1H), 3.50 – 3.41 (m, 1H), 3.28 – 3.19 (m, 1H), 3.16 –3.09 (m, 1H), 3.05 – 2.95 (m, 3H), 2.89 – 2.74 (m, 2H), 1.56 (s, 9H). ^{13}C NMR (126 MHz, $CDCl_3$) δ 152.9, 140.8, 140.6, 139.0, 137.3, 134.3, 134.2, 128.3,

128.0, 127.0, 125.4, 125.0, 80.6, 35.4, 33.2, 32.4, 28.6. m/z HRMS (ESI) found $[M-56+H]^+$ 346.0438 $C_{17}H_{17}BrNO_2^+$ calculated 346.0437.

Tert-butyl (4^3-phenyl-1,4(1,4)-dibenzenacyclohexaphane-1^2-yl)carbamate **(1ab)**

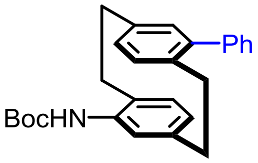

This reaction was performed on 1.0 mmol scale of **1aa** according to **Method I**, which gave the product **1ab** (285 mg, 71% yield) as a white solid

^{1}H NMR (400 MHz, $CDCl_3$) δ 7.53 – 7.41 (m, 4H), 7.37 – 7.35(m, 1H), 6.91 – 6.73 (m, 2H), 6.62 (d, *J* = 7.7 Hz, 1H), 6.57 – 6.47 (m, 2H), 6.46 – 6.39 (m, 1H), 6.35 (s, 1 H), 3.44 – 3.37 (m, 1H), 3.34 – 3.24 (m, 1H), 3.13 – 3.08 (m, 2H), 2.99 – 2.73 (m, 3H), 2.67 – 2.60 (m, 1H), 1.58 (s, 9H). ^{13}C NMR (101 MHz, $CDCl_3$) δ 153.0, 142.5, 141.4, 141.2, 139.2, 137.0, 136.9, 135.3, 134.1, 132.1, 130.0, 129.0, 128.6, 128.2, 126.9, 125.9, 125.5, 80.5, 77.4, 34.6, 33.7, 33.7, 32.7, 28.6. m/z HRMS (ESI) found $[M+H]^+$ 400.2267 $C_{27}H_{30}NO_2^+$ calculated 400.2271.

Tert-butyl (E)-(4^3-styryl-1,4(1,4)-dibenzenacyclohexaphane-1^2-yl)carbamate **(1ac)**

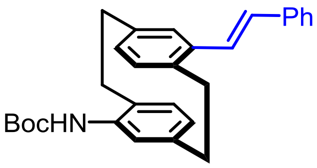

This reaction was performed on 0.89 mmol scale of **1aa** according to **Method C**, which gave the product **1ac** (150 mg, 40% yield for two steps) as a white solid

^{1}H NMR (400 MHz, $CDCl_3$) δ 7.60 – 7.54 (m, 2H), 7.42 – 7.39 (m, 2H), 7.32 – 7.27 (m, 1H), 7.20 (d, *J* = 16.1 Hz, 1H), 6.86 (d, *J* = 16.1 Hz, 1H), 6.80 – 6.70 (m, 2H), 6.62 (s, 1H), 6.54 – 6.45 (m, 2H), 6.37 – 6.35 (m, 1H), 6.28 (s, 1H), 3.62 – 3.51 (m, 1H), 3.29 – 3.19 (m, 1H), 3.15 – 3.01 (m, 3H), 2.98 – 2.79 (m, 3H), 1.57 (s, 9H). ^{13}C NMR (101 MHz, $CDCl_3$) δ 152.9, 140.6, 139.1, 138.2, 138.0, 137.6, 137.0, 134.1, 133.8, 130.4, 129.4, 128.9, 128.1, 127.6, 127.1, 126.6, 126.2, 125.0, 80.5, 34.4, 33.5,

33.4, 32.4, 28.5. m/z HRMS (ESI) found $[M+H]^+$ 426.2424 $C_{29}H_{32}NO_2^+$ calculated 426.2428.

Tert-butyl (4^3-cyclohexyl-1,4(1,4)-dibenzenacyclohexaphane-1^2-yl)carbamate **(1ad)**

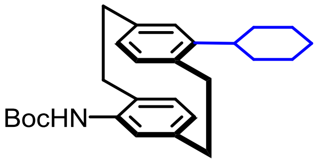

This reaction was performed on 0.57 mmol scale of **S7** according to **Method E**, which gave the product **1ad** (200 mg, 87% yield) as a white solid

^{1}H NMR (400 MHz, $CDCl_3$) δ 6.76 – 6.60 (m, 2H), 6.43 – 6.33 (m, 2H), 6.32 – 6.30 (m, 1H), 6.24 (s, 1H), 6.18 – 6.12 (m, 1H), 3.39 – 3.33 (m, 1H), 3.22 – 3.16 (m, 1H), 3.09 – 2.94 (m, 4H), 2.87 – 2.69 (m, 2H), 2.48 – 2.43 (m, 1H), 2.08 – 1.97 (m, 2H), 1.80 – 1.71 (m, 3H), 1.56 (s, 9H), 1.51 – 1.38 (m, 3H), 1.24 – 1.10 (m, 1H), 0.93 – 0.82 (m, 1H). ^{13}C NMR (101 MHz, $CDCl_3$) δ 152.9, 146.7, 140.9, 138.8, 137.2, 136.9, 134.2, 134.1, 129.3, 128.9, 126.0, 125.4, 125.0, 80.4, 40.9, 36.8, 33.9, 33.4, 33.0, 32.5, 30.5, 28.5, 27.7, 27.2, 26.6. m/z HRMS (ESI) found $[M-56+H]^+$ 350.2112 $C_{23}H_{28}NO_2^+$calculated 350.2115.

1.3 Kinetic resolution of amido-[2.2]Paracyclophane derivatives

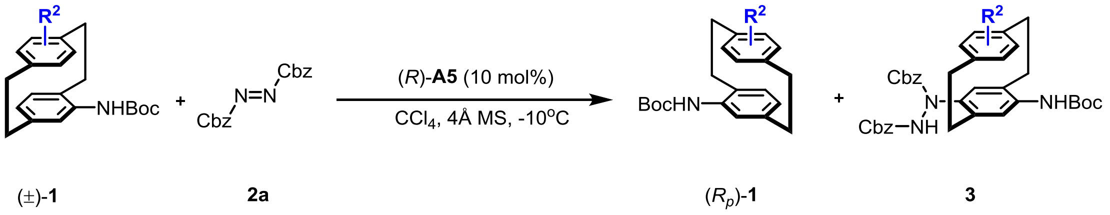

General procedure for kinetic resolution of amido-[2.2]paracyclophane derivatives

To a solution of racemic **1** (0.2 mmol, 1.0 equiv.), (*R*)-**A5** (0.02 mmol, 0.1 equiv.) and activated 4 Å MS (200 mg) in CCl_4 or toluene (1 mL) was added a solution of **2a** (0.14 mmol, 0.7 equiv.) in dry CCl_4 or toluene (1 mL) at designed temperature under N_2

atmosphere. After achieving appropriate conversion as indicated by HPLC analysis of the reaction mixture, the reaction mixture was quenched with Et_3N (20 μL) and concentrated under vacuum to give a residue, which was purified by column chromatography to afford the recovered product **1** and product **3**.

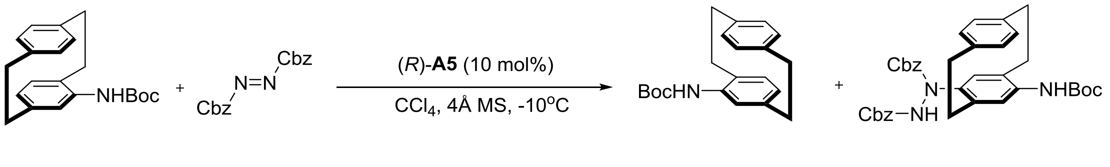

The reaction was performed on 0.2 mmol scale at −10 ℃ in CCl_4 (2 mL) for 12 h and the products were purified by column chromatography (petroleum ether/EtOAc = 8:1 -2:1) to afford the recovered product (*R_p*)-**1a** (31 mg, 48% yield) as a white solid and product **3a** (60 mg, 48% yield) as a yellow solid.

(R_p)-Tert-butyl 1,4(1,4)-dibenzenacyclohexaphane-1^2-ylcarbamate **(1a)**

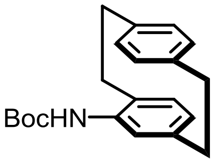

$[\alpha]^{25}_{D}$ = -126.20 (c = 0.5, $CHCl_3$). HPLC: Chiralpak IC column, 70:30 hexanes/isopropanol, 1 ml/min; t_R = 5.09 min (minor), 6.85 min (major); 95% ee.

(S_p)-Dibenzyl1-(1^5-((tert-butoxycarbonyl)amino)-1,4(1,4)-dibenzenacyclohexaphane-1^2-yl)hydrazine-1,2-dicarboxylate **(3a)**

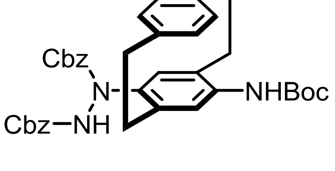

^{1}H NMR (400 MHz, $CDCl_3$) δ 7.49 – 7.26 (m, 10H), 7.17 (s, 1H), 6.87 – 6.70 (m, 2H), 6.66 – 6.40 (m, 3H), 6.24 – 6.17 (m, 2H), 5.32 – 4.95 (m, 4H), 3.25 – 2.81 (m, 6H), 2.75 – 2.67 (m, 2H), 1.56 (s, 9H). ^{13}C NMR (126 MHz, $CDCl_3$) δ 157.0, 155.6, 152.6, 139.3, 138.0, 137.3, 137.0, 135.7, 135.1, 133.0, 131.8, 131.3, 128.7, 128.5, 128.3,

128.3, 128.0, 125.7, 80.7, 68.6, 68.0, 34.8, 33.2, 32.3, 31.3, 28.5. $[\alpha]^{25}_{D}$ = 82.1 (c = 0.5, $CHCl_3$). m/z HRMS (ESI) found $[M+H]^+$ 622.2925 $C_{37}H_{40}N_3O_6^+$ calculated 622.2912. HPLC: Chiralpak IC column, 70:30 hexanes/isopropanol, 1 ml/min; t_R = 11.71 min (major), 13.72 min (minor); 95% ee.

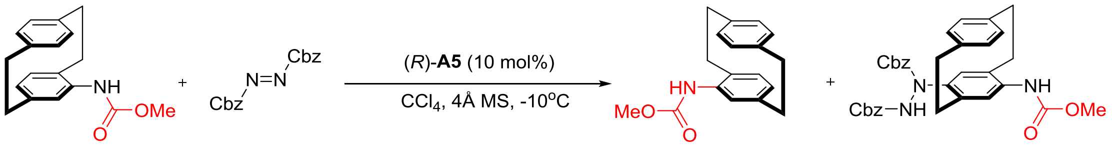

(±)-**1b** **2a** (R_p)-**1b** **3b**

The reaction was performed on 0.2 mmol scale at −10 ℃ in CCl_4 (2 mL) for 11.5 h and the products were purified by column chromatography (petroleum ether/EtOAc = 8:1 -2:1) to afford the recovered product (R_p)-**1b** (28 mg, 50% yield) as a yellow solid and product **3b** (55 mg, 47% yield) as a yellow solid.

(R_p)-Methyl 1,4(1,4)-dibenzenacyclohexaphane-1^2-ylcarbamate **(1b)**

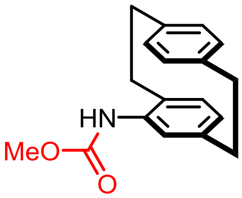

$[\alpha]^{25}_{D}$ = -111.8 (c = 0.5, $CHCl_3$). HPLC: Chiralpak IC column, 70:30 hexanes/isopropanol, 1 ml/min; t_R = 8.57 min (minor), 23.57 min (major); 91% ee.

(S_p)-Dibenzyl-1-(1^5-((methoxycarbonyl)amino)-1,4(1,4)-dibenzenacyclohexaphane-1^2 -yl)hydrazine-1,2-dicarboxylate **(3b)**

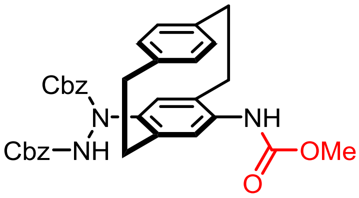

^1H NMR (400 MHz, $CDCl_3$) δ 7.35 – 7.26 (m, 9H), 7.14 (s, 1H), 6.83 – 6.77 (m, 2H), 6.62 – 6.41 (m, 3H), 6.35 (s, 1H), 6.18 (s, 1H), 5.35 – 4.90 (m, 5H), 3.81 (s, 3H), 3.25

– 2.85 (m, 6H), 2.71 (s, 2H). ^{13}C NMR (101 MHz, $CDCl_3$) δ 157.0, 155.7, 153.9, 139.3, 138.0, 137.4, 136.4, 135.7, 135.6, 133.1, 131.8, 131.4, 128.7, 128.6, 128.5, 128.4, 128.3, 128.3, 127.9, 126.0, 68.7, 68.0, 52.5, 34.8, 33.2, 32.2, 31.4. $[\alpha]^{25}_{D}$ = 66.2 (c = 0.5, $CHCl_3$). m/z HRMS (ESI) found $[M+H]^+$ 580.2445 $C_{34}H_{34}N_3O_6^+$ calculated 580.2442. HPLC: Chiralpak IC column, 70:30 hexanes/isopropanol, 1 ml/min; t_R = 22.03 min (major), 35.48 min (minor); 93% ee.

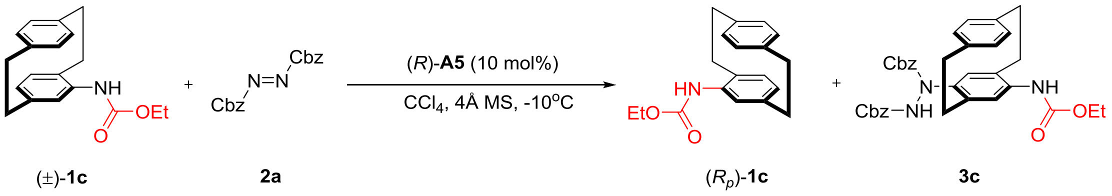

The reaction was performed on 0.2 mmol scale at −10 ℃ in CCl_4 (2 mL) for 11.5 h and the products were purified by column chromatography (petroleum ether/EtOAc = 8:1 -2:1) to afford the recovered product (R_p)-**1c** (29 mg, 49% yield) as a yellow solid and product **3c** (55 mg, 46% yield) as a yellow solid.

(R_p)−Ethyl 1,4(1,4)-dibenzenacyclohexaphane-1^2-ylcarbamate **(1c)**

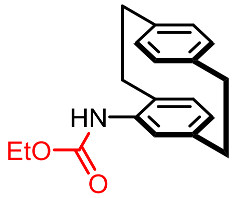

$[\alpha]^{25}_{D}$ = -124.4 (c = 0.5, $CHCl_3$). HPLC: Chiralpak IC column, 70:30 hexanes/isopropanol, 1 ml/min; t_R = 7.45 min (minor), 20.24 min (major); 84% ee.

(S_p)-Dibenzyl 1-(1^5-((ethoxycarbonyl)amino)-1,4(1,4)-dibenzenacyclohexaphane-1^2-yl)hydrazine-1,2-dicarboxylate **(3c)**

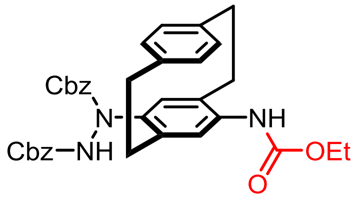

^{1}H NMR (400 MHz, $CDCl_3$) δ 7.35 – 7.26 (m, 9H), 7.16 (s, 1H), 6.83 – 6.70 (m, 2H), 6.60 – 6.42 (m, 3H), 6.34 (s, 1H), 6.18 (s, 1H), 5.27 – 5.00 (m, 5H), 4.26 (q, J = 7.1 Hz, 2H), 3.25 – 2.70 (m, 8H), 1.36 (t, J = 7.1 Hz, 3H). ^{13}C NMR (101 MHz, $CDCl_3$) δ 157.0, 155.6, 153.5, 139.4, 138.0, 137.4, 136.5, 135.7, 135.6, 133.0, 131.8, 131.4, 128.7, 128.6, 128.5, 128.4, 128.3, 128.2, 127.9, 126.0, 68.6, 68.0, 61.4, 34.8, 33.2, 32.2, 31.4, 14.7. $[\alpha]^{25}_{D}$ = 68.7 (c = 0.5, $CHCl_3$). m/z HRMS (ESI) found $[M+H]^+$ 594.2609 $C_{35}H_{36}N_3O_6^+$ calculated 594.2599. HPLC: Chiralpak IC column, 70:30 hexanes/isopropanol, 1 ml/min; t_R = 20.31 min (major), 31.19 min (minor); 90% ee.

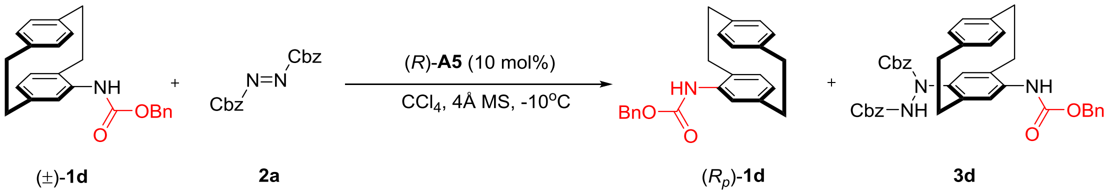

The reaction was performed on 0.2 mmol scale at −10 ℃ in CCl_4 (2 mL) for 12 h and the products were purified by column chromatography (petroleum ether/EtOAc = 8:1 -2:1) to afford the recovered product (*R*$_p$)-**1d** (36 mg, 50% yield) as a white solid and product **3d** (63 mg, 48% yield) as a yellow solid.

(*R*$_p$)−Benzyl 1,4(1,4)-dibenzenacyclohexaphane-1^2-ylcarbamate **(1d)**

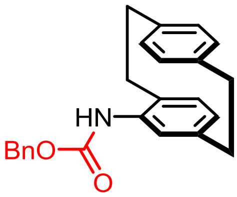

$[\alpha]^{25}_{D}$ = -111.1 (c = 0.5, $CHCl_3$). HPLC: Chiralpak IA column, 70:30 hexanes/isopropanol, 1 ml/min; t_R = 7.48 min (minor), 9.10 min (major); 90% ee.

(*S*$_p$)-Dibenzyl-1-(1^5-(((benzyloxy)carbonyl)amino)-1,4(1,4)-dibenzenacyclohexaphane-1^2-yl)hydrazine-1,2-dicarboxylate **(3d)**

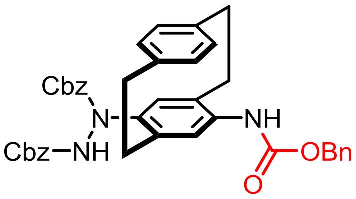

^{1}H NMR (400 MHz, $CDCl_3$) δ 7.49 – 7.26 (m, 15H), 7.15 (s, 1H), 6.85 – 6.75 (m, 2H), 6.60 – 6.37 (m, 4H), 6.17 (s, 1H), 5.30 – 5.18 (m, 5H), 5.05 (s, 1H), 3.26 – 2.84 (m, 6H), 2.80 – 2.55 (s, 2H). ^{13}C NMR (101 MHz, $CDCl_3$) δ 157.0, 155.6, 153.2, 139.3, 138.0, 137.4, 136.3, 136.1, 135.7, 135.4, 133.0, 131.8, 131.4, 128.7, 128.7, 128.5, 128.4, 128.3, 128.3, 127.9, 125.9, 68.7, 68.0, 67.2, 34.8, 33.1, 32.1, 31.4. $[\alpha]^{25}_{D}$ = 78.2 (c = 0.5, $CHCl_3$). m/z HRMS (ESI) found $[M+H]^+$ 656.2759 $C_{40}H_{38}N_3O_6^+$ calculated 656.2755. HPLC: Chiralpak IA column, 70:30 hexanes/isopropanol, 1 ml/min; t_R = 18.18 min (major), 27.56 min (minor); 94% ee.

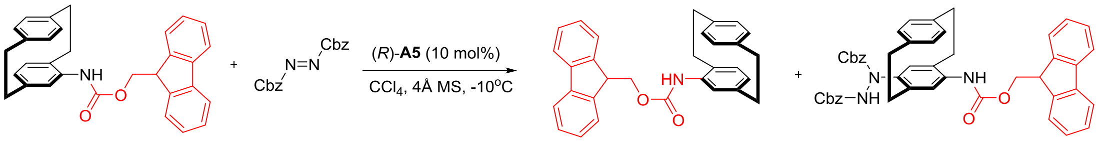

The reaction was performed on 0.2 mmol scale at −10 ℃ in CCl_4 (2 mL) for 12 h and the products were purified by column chromatography (petroleum ether/EtOAc = 8:1 - 2:1) to afford the recovered product (R_p)-**1e** (41 mg, 47% yield) as a white solid and product **3e** (71 mg, 48% yield) as a yellow solid.

(R_p)−(9*H*-fluoren-9-yl)methyl 1,4(1,4)-dibenzenacyclohexaphane-1^2-ylcarbamate **(1e)**

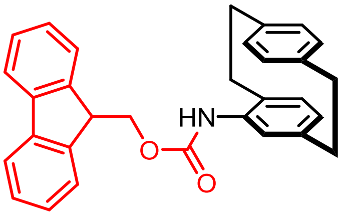

$[\alpha]^{25}_{D}$ = -80.5 (c = 0.5, $CHCl_3$). HPLC: Chiralpak IC column, 60:40 hexanes/isopropanol, 1 ml/min; t_R = 8.56 min (minor), 34.29 min (major); 97% ee.

(S_p)-Dibenzyl 1-(1[5]-((((9H-fluoren-9-yl)methoxy)carbonyl)amino)-1,4(1,4)-dibenzenacyclohexaphane-1[2]-yl)hydrazine-1,2-dicarboxylate **(3e)**

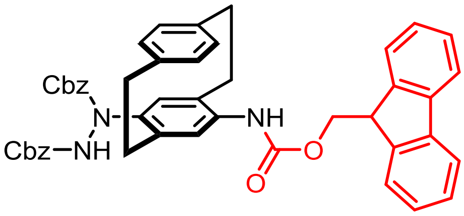

^{1}H NMR (400 MHz, $CDCl_3$) δ 7.81 – 7.60 (m, 3H), 7.46 – 7.26 (m, 13H), 7.13 (s, 2H), 6.67 – 6.40 (m, 4H), 6.32 (s, 3H), 5.26 – 5.00 (m, 5H), 4.72 – 4.56 (m, 2H), 4.31 (s, 1H), 3.21 – 2.98 (m, 3H), 2.98 – 2.50 (d, J = 76.9 Hz, 5H). ^{13}C NMR (101 MHz, $CDCl_3$) δ 157.0, 155.4, 153.2, 143.9, 143.7, 141.6, 139.3, 138.0, 135.7, 135.6, 133.1, 131.9, 131.4, 128.8, 128.7, 128.6, 128.4, 128.0, 128.0, 127.3, 127.3, 125.0, 125.0, 120.2, 120.2, 68.8, 68.2, 68.1, 66.6, 47.5, 34.9, 33.2, 32.3, 31.4. $[\alpha]^{25}_D$ = 52.4 (c = 0.5, $CHCl_3$). m/z HRMS (ESI) found $[M+H]^+$ 744.3086 $C_{47}H_{42}N_3O_6^+$ calculated 744.3068. HPLC: Chiralpak IC column, 60:40 hexanes/isopropanol, 1 ml/min; t_R = 18.30 min (major), 32.84 min (minor); 96% ee.

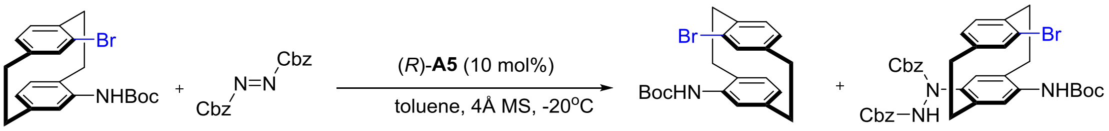

(±)-**1f** **2a** (S_p)-**1f** **3f**

The reaction was performed on 0.2 mmol scale at −20 ℃ in toluene (2 mL) for 16.5 h and the products were purified by column chromatography (petroleum ether/EtOAc = 8:1 – DCM;MeOH = 100:1) to afford the recovered product (S_p)-**1f** (41 mg, 51% yield) as a yellow solid and product **3f** (65 mg, 46% yield) as a yellow solid.

(S_p)-Tert-butyl (4[2]-bromo-1,4(1,4)-dibenzenacyclohexaphane-1[2]-yl)carbamate **(1f)**

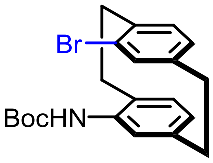

$[\alpha]^{25}_{D}$ = -4.6 (c = 0.5, $CHCl_3$). HPLC: Chiralpak IC column, 70:30 hexanes/isopropanol，1 ml/min; t_R = 13.66 min (major), 22.87 min (minor); 89% ee.

(R_p)-Dibenzyl 1-(4^3-bromo-1^5-((tert-butoxycarbonyl)amino)-1,4(1,4)-dibenzena-cyclohexaphane-1^2-yl)hydrazine-1,2-dicarboxylate **(3f)**

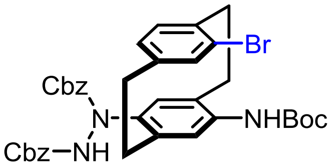

^{1}H NMR (400 MHz, $CDCl_3$) δ 7.37 – 7.27 (m, 10H), 6.99 (s, 1H), 6.84 (s, 1H), 6.62 – 6.37 (m, 3H), 6.18 (s, 1H), 5.32 – 4.93 (m, 5H), 3.60 – 3.45 (m, 1H), 3.30 – 2.85 (m, 4H), 2.85 – 2.57 (m, 3H), 1.54 (s, 9H). ^{13}C NMR (126 MHz, $CDCl_3$new) δ 157.0, 155.4, 152.4, 141.6, 137.5, 137.1, 135.7, 135.5, 135.1, 130.8, 128.7, 128.7, 128.6, 128.4, 128.4, 128.1, 127.2, 124.6, 122.6, 80.5, 68.8, 68.2, 34.4, 33.3, 31.1, 28.6. $[\alpha]^{25}_{D}$ = 46.1 (c = 0.5, $CHCl_3$). m/z HRMS (ESI) found $[M+H]^+$ 700.2003 $C_{37}H_{39}BrN_3O_6^+$ calculated 700.2017. HPLC: Chiralpak IC column, 70:30 hexanes/isopropanol, 1 ml/min; t_R = 10.74 min (major), 12.02 min (minor); 97% ee.

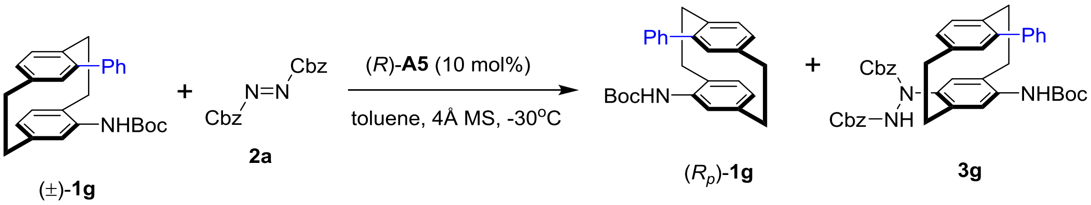

(±)-**1g** (R_p)-**1g** **3g**

The reaction was performed on 0.2 mmol scale at −30 ℃ in toluene (2 mL) for 26.5 h and the products were purified by column chromatography (petroleum ether/EtOAc = 8:1 – DCM;MeOH = 80:1) to afford the recovered product (R_p)-**1g** (39 mg, 49% yield) as white a solid and product **3g** (68 mg, 49% yield) as a white solid.

(R_p)−Tert-butyl (4^2-phenyl-1,4(1,4)-dibenzenacyclohexaphane-1^2-yl)carbamate **(1g)**

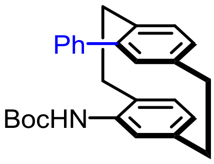

$[\alpha]^{25}_{D}$ = 46.6 (c = 0.5, $CHCl_3$). HPLC: Chiralpak IC column, 70:30 hexanes/isopropanol, 1 ml/min; t_R = 5.97 min (minor), 7.42 min (major); 94% ee.

(S_p)-Dibenzyl 1-(1^5-((tert-butoxycarbonyl)amino)-4^3-phenyl-1,4(1,4)-dibenzenacyclohexaphane-1^2-yl)hydrazine-1,2-dicarboxylate **(3g)**

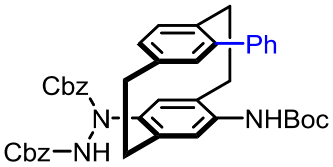

^{1}H NMR (500 MHz, $CDCl_3$) δ 7.51 –7.50 (m, 2H), 7.42 – 7.29 (m, 13H), 7.16 (s, 1H), 6.85 (s, 1H), 6.66 – 6.47 (m, 3H), 6.40– 6.22 (m, 1H), 5.84 (s, 1H), 5.30 – 4.90 (m, 4H), 3.38 – 3.06 (m, 4H), 3.02 – 2.90 (m, 2H), 2.85 – 2.60 (m, 2H), 1.39 (s, 9H). ^{13}C NMR (126 MHz, $CDCl_3$) δ 156.9, 155.6, 152.3, 140.8, 140.5, 138.9, 137.0, 135.8, 135.3, 130.8, 130.0, 129.3, 128.8, 128.7, 128.6, 128.6, 128.4, 128.2, 126.9, 126.0, 80.3, 68.8, 68.2, 34.5, 33.2, 31.1, 29.8, 28.4. $[\alpha]^{25}_{D}$ = -4.6 (c = 0.5, $CHCl_3$). m/z HRMS (ESI) found $[M+H]^+$ 698.3242 $C_{43}H_{44}N_3O_6^+$ calculated 698.3225. HPLC: Chiralpak IC column, 70:30 hexanes/isopropanol, 1 ml/min; t_R = 13.66 min (major), 22.87 min (minor); 96% ee.

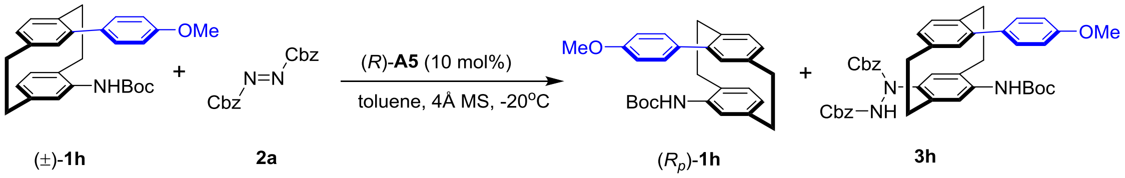

The reaction was performed on 0.2 mmol scale at −20 ℃ in toluene (2 mL) for 11 h and the products were purified by column chromatography (petroleum ether/EtOAc = 8:1 – DCM;MeOH = 100:1) to afford the recovered product (R_p)-**1h** (42 mg, 49% yield) as a white solid and product **3h** (72 mg, 49% yield) as a yellow solid.

(R_p)−Tert-butyl-(4^2-(4-methoxyphenyl)-1,4(1,4)-dibenzenacyclohexaphane-1^2-yl)carbamate **(1h)**

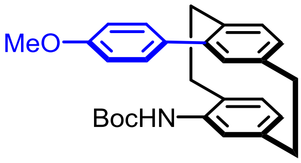

$[\alpha]^{25}_D$ = 65.2 (c = 0.5, $CHCl_3$). HPLC: Chiralpak IC column, 70:30 hexanes/isopropanol, 1 ml/min; t_R = 7.19 min (minor), 9.48 min (major); 96% ee.

(S_p)-Dibenzyl 1-(1^5-((tert-butoxycarbonyl)amino)-4^3-(4-methoxyphenyl)-1,4(1,4)-dibenzenacyclohexaphane-1^2-yl)hydrazine-1,2-dicarboxylate **(3h)**

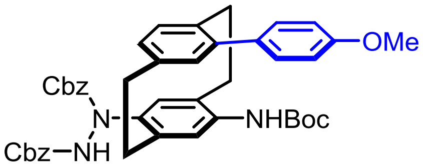

^{1}H NMR (400 MHz, $CDCl_3$) δ 7.44 – 7.32 (m, 11H), 7.14 (s, 1H), 6.97 – 6.91 (m, 2H), 6.81 (s, 1H), 6.67 – 6.41 (m, 4H), 6.40 – 6.20 (m, 1H), 5.85 (s, 1H), 5.30 – 5.26 (m, 2H), 5.15 – 4,95 (m, 2H), 3.85 (s, 3H), 3.31 – 3.25 (m, 1H), 3.21-3.10 (m, 2H), 3.01 – 2.97 (m, 3H), 2.85 – 2.75 (s, 1H), 2.75 – 2.60 (m, 1H), 1.40 (s, 9H). ^{13}C NMR (101 MHz, $CDCl_3$) δ 158.7, 156.9, 155.7, 152.2, 140.1, 138.9, 136.8, 135.7, 135.6, 135.5, 135.0, 133.1, 130.4, 130.2, 129.6, 128.7, 128.6, 128.5, 128.5, 128.4, 128.3, 128.0, 126.3, 114.0, 80.1, 68.6, 68.0, 55.3, 34.3, 33.2, 31.4, 31.0, 28.4. $[\alpha]^{25}_D$ = -18.7 (c = 0.5, $CHCl_3$). m/z HRMS (ESI) found $[M+H]^+$ 728.3340 $C_{44}H_{46}N_3O_7^+$ calculated 728.3330. HPLC: Chiralpak IC column, 70:30 hexanes/isopropanol, 1 ml/min; t_R = 17.78 min (major), 29.62 min (minor); 96% ee.

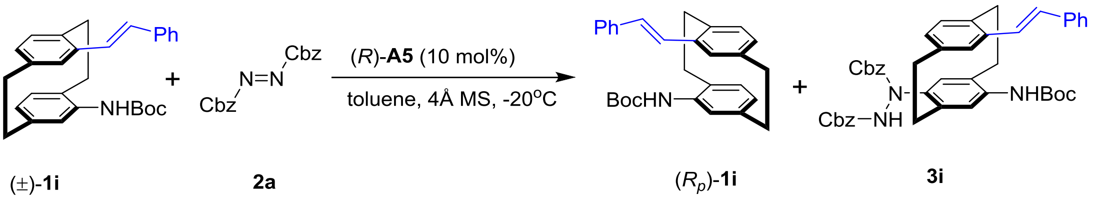

The reaction was performed on 0.2 mmol scale at −20 ℃ in toluene (2 mL) for 12.5 h and the products were purified by column chromatography (petroleum ether/EtOAc = 8:1 – 2:1) to afford the recovered product (R_p)-**1i** (42 mg, 49% yield) as a white solid and product **3i** (72 mg, 50% yield) as a yellow solid.

(R_p)-Tert-butyl (E)-(4^2-styryl-1,4(1,4)-dibenzenacyclohexaphane-1^2-yl)carbamate **(1i)**

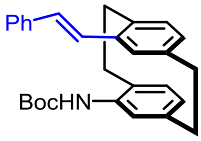

$[\alpha]^{25}_D$ = 126.2 (c = 0.5, $CHCl_3$). HPLC: Chiralpak IC column, 70:30 hexanes/isopropanol, 1 ml/min; t_R = 6.20 min (minor), 10.56 min (major); 96% ee.

(S_p)-Dibenzyl-(E)-1-(1^5-((tert-butoxycarbonyl)amino)-4^3-styryl-1,4(1,4)-dibenzenacyclohexaphane-1^2-yl)hydrazine-1,2-dicarboxylate **(3i)**

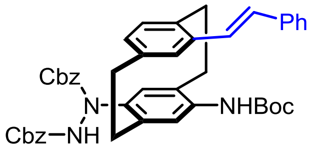

^{1}H NMR (400 MHz, $CDCl_3$) δ 7.58 – 7.50 (m, 2H), 7.48 – 7.26 (m, 13H), 7.25 – 7.06 (m, 2H), 6.95 – 6.79 (m, 2H), 6.65 – 6.35 (m, 3H), 6.30 – 6.05 (m, 2H), 5.35 – 4.90 (m, 4H), 3.70 – 3.55 (m, 1H), 3.31 – 2.64 (m, 7H), 1.28 (s, 9H). ^{13}C NMR (101 MHz, $CDCl_3$) δ 157.0, 155.5, 152.4, 139.8, 137.9, 136.8, 136.6, 135.7, 135.5, 135.0, 131.0, 129.0, 128.7, 128.5, 128.4, 128.3, 128.1, 127.4, 126.6, 80.4, 68.7, 68.0, 34.7, 31.8, 31.0, 30.7, 28.1. $[\alpha]^{25}_D$ = 13.5 (c = 0.5, $CHCl_3$). m/z HRMS (ESI) found $[M+H]^+$ 724.3394 $C_{45}H_{46}N_3O_6^+$ calculated 724.3381. HPLC: Chiralpak IC column, 70:30 hexanes/isopropanol, 1 ml/min; t_R = 14.55 min (major), 21.32 min (minor); 96% ee.

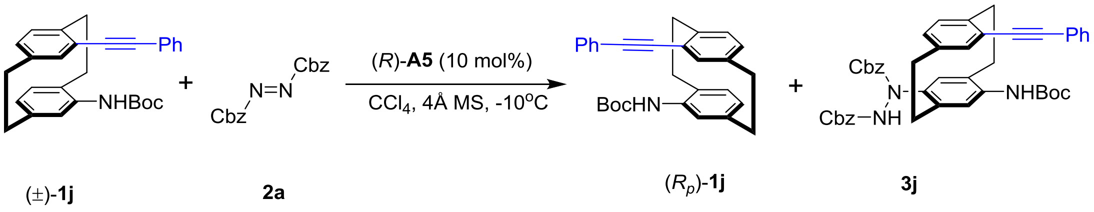

The reaction was performed on 0.2 mmol scale at −10 ℃ in CCl_4 (2 mL) for 20 h and the products were purified by column chromatography (petroleum ether/EtOAc = 8:1 – 2:1) to afford the recovered product **1j** (29 mg, 34% yield) as a yellow solid and product **3j** (49 mg, 34% yield) as a yellow solid.

(R_p)−Tert-butyl-(4^2-(phenylethynyl)-1,4(1,4)-dibenzenacyclohexaphane-1^2-yl)carbamate **(1j)**

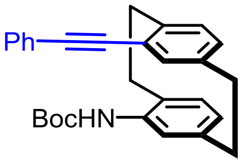

$[\alpha]^{25}_{D}$ = 44.3 (c = 0.5, $CHCl_3$). HPLC: Chiralpak IC column, 70:30 hexanes/isopropanol, 1 ml/min; t_R = 12.10 min (major), 18.38 min (minor); 83% ee.

(S_p)-Dibenzyl-1-(1^5-((tert-butoxycarbonyl)amino)-4^3-(phenylethynyl)-1,4(1,4)-dibenzena cyclohexaphane-1^2-yl)hydrazine-1,2-dicarboxylate **(3j)**

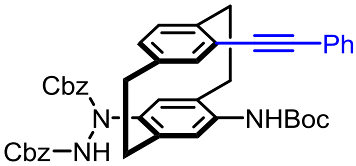

^{1}H NMR (400 MHz, $CDCl_3$) δ 7.65 – 7.50 (m, 3H), 7.47 – 7.27 (m, 13H), 6.83 – 6.70 (m, 2H), 6.63 – 6.40 (m, 3H), 6.23 (s, 1H), 5.26 – 4.90 (m, 4H), 3.80 – 3.68 (m, 1H), 3.41 – 3.35 (m, 1H), 3.30 – 2.90 (m, 6H), 1.28 (s, 9H). ^{13}C NMR (126 MHz, $CDCl_3$) δ 156.9, 155.5, 152.9, 141.1, 139.7, 137.2, 136.8, 135.7, 135.2, 134.1, 132.1, 131.7, 128.7, 128.6, 128.4, 128.3, 128.1, 123.8, 121.2, 91.9, 89.1, 80.2, 68.7, 68.1, 34.5, 32.1, 31.8, 31.0, 28.1. $[\alpha]^{25}_{D}$ = 44.3 (c = 0.5, $CHCl_3$). m/z HRMS (ESI) found $[M+H]^+$ 722.3237 $C_{45}H_{44}N_3O_6^+$ calculated 722.3225. HPLC: Chiralpak IC column, 70:30 hexanes/isopropanol, 1 ml/min; t_R = 12.10 min (major), 18.38 min (minor); 88% ee.

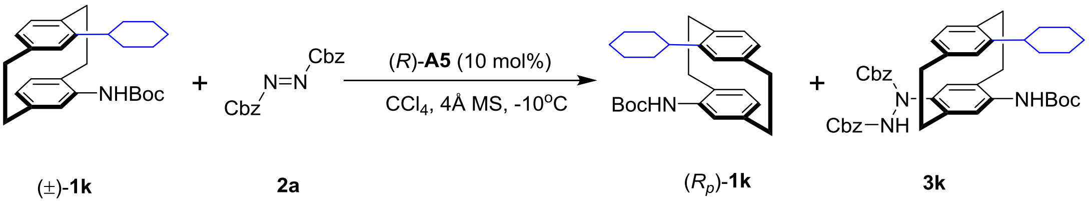

The reaction was performed on 0.2 mmol scale at −10 ℃ in CCl_4 (2 mL) for 4.5h and the products were purified by column chromatography (petroleum ether/EtOAc = 8:1 – 2:1) to afford the recovered product (R_p)-**1k** (39 mg, 48% yield) as a white solid and

product **3k** (70 mg, 50% yield) as a yellow solid.

(R_p)−Tert-butyl (4[2]-cyclohexyl-1,4(1,4)-dibenzenacyclohexaphane-1[2]-yl)carbamate **(1k)**

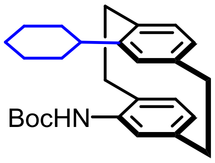

$[\alpha]^{25}_D$ = -75.8 (c = 0.5, $CHCl_3$). HPLC: Chiralpak IC column, 70:30 hexanes/isopropanol, 1 ml/min; t_R = 5.14 min (minor), 5.90 min (major); 98% ee.

(S_p)-Dibenzyl 1-(1[5]-((tert-butoxycarbonyl)amino)-4[3]-cyclohexyl-1,4(1,4)-dibenzena cyclohexaphane-1[2]-yl)hydrazine-1,2-dicarboxylate **(3k)**

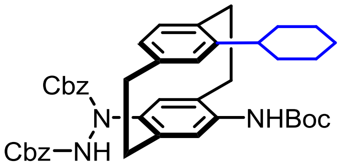

^{1}H NMR (400 MHz, $CDCl_3$) δ 7.48 – 7.25 (m, 10H), 7.17 (s, 1H), 6.74 – 6.29 (m, 4H), 6.18 (s, 2H), 5.30 – 4.80 (m, 4H), 3.40 – 3.25 (m, 1H), 3.20 – 3.15 (m, 2H), 3.00 – 2.60 (m, 5H), 2.12 – 1.90 (m, 2H), 1.75 – 1.65 (m, 3H), 1.57 – 1.53 (m, 2H), 1.51 (s, 9H), 1.48 – 1.35 (m, 1H), 1.20 – 1.10 (m, 1H), 0.87 – 0.75 (m, 1H). ^{13}C NMR (101 MHz, $CDCl_3$) δ 157.0, 155.8, 152.4, 145.3, 139.2, 137.1, 136.7, 135.8, 135.4, 134.8, 128.8, 128.7, 128.6, 128.5, 128.3, 128.2, 128.1, 127.2, 125.4, 80.7, 68.6, 68.1, 40.4, 38.8, 34.9, 31.1, 30.9, 30.7, 30.2, 28.4, 27.5, 27.5, 26.4. $[\alpha]^{25}_D$ = 57.8 (c = 0.5, $CHCl_3$). m/z HRMS (ESI) found $[M+H]^+$ 704.3715 $C_{43}H_{50}N_3O_6^+$ calculated 704.3694. HPLC: Chiralpak IA column, 70:30 hexanes/isopropanol, 1 ml/min; t_R = 8.07 min (major), 9.42 min (minor); 93% ee.

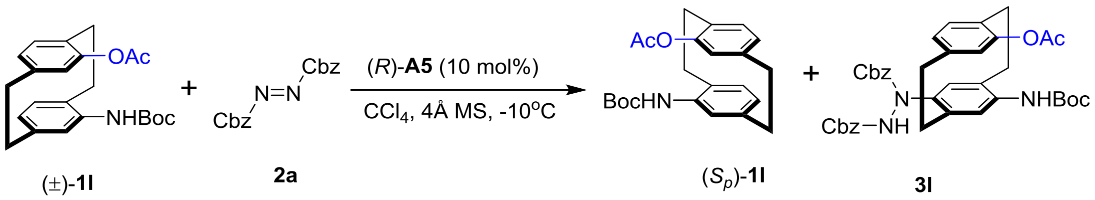

The reaction was performed on 0.2 mmol scale at −10 ℃ in CCl_4 (2 mL) for 4.5h and the products were purified by column chromatography (petroleum ether/EtOAc = 8:1 – 2:1) to afford the recovered product (S_p)-**1l** (38 mg, 50% yield) as a yellow solid and product **3l** (65 mg, 48% yield) as a yellow solid.

(S_p)-4^2-((tert-butoxycarbonyl)amino)-1,4(1,4)-dibenzenacyclohexaphane-1^2-yl acetate **(1l)**

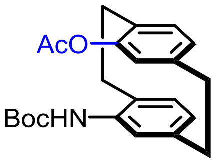

$[\alpha]^{25}_D$ = -66.6 (c = 0.5, $CHCl_3$). HPLC: Chiralpak IC column, 70:30 hexanes/isopropanol, 1 ml/min; t_R = 13.20 min (minor), 15.29 min (major); 88% ee.

(R_p)-Dibenzyl 1-(4^3-acetoxy-1^5-((tert-butoxycarbonyl)amino)-1,4(1,4)-dibenzena cyclohexaphane-1^2-yl)hydrazine-1,2-dicarboxylate **(3l)**

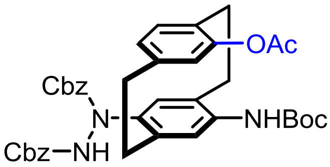

^{1}H NMR (400 MHz, $CDCl_3$) δ 7.48 – 7.27 (m, 10H), 7.18 (s, 1H), 6.70 (s, 1H), 6.57 – 6.16 (m, 5H), 5.40 – 4.90 (m, 4H), 3.35 – 2.98 (m, 4H), 2.90 – 2.60 (m, 4H), 2.32 (s, 3H), 1.53 (s, 9H). ^{13}C NMR (101 MHz, $CDCl_3$) δ 169.0, 157.0, 155.4, 152.6, 149.4, 141.3, 136.6, 135.6, 134.9, 129.9, 128.7, 128.5, 128.3, 128.3, 128.0, 127.1, 125.6, 80.3, 68.7, 68.0, 34.4, 31.8, 31.4, 28.5, 28.4, 28.0, 21.0. $[\alpha]^{25}_D$ = -48.8 (c = 0.5, $CHCl_3$). m/z HRMS (ESI) found $[M+17+H]^+$ 697.3218 $C_{39}H_{45}N_4O_8^+$ calculated 697.3232. HPLC: Chiralpak IA column, 70:30 hexanes/isopropanol, 1 ml/min; t_R = 21.89 min (major), 30.69 min (minor); 93% ee.

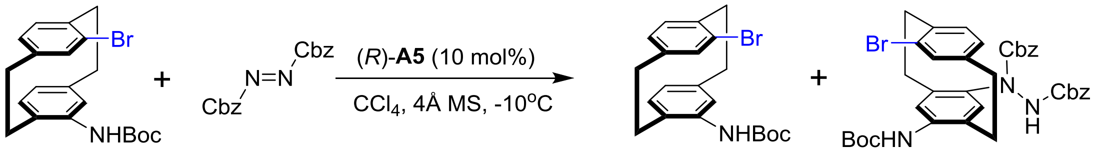

(±)-**1m** **2a** (R_p)-**1m** **3m**

The reaction was performed on 0.2 mmol scale at −10 ℃ in CCl_4 (2 mL) for 12 h and the products were purified by column chromatography (petroleum ether/EtOAc = 8:1 – 2:1) to afford the recovered product (R_p)-**1m** (40 mg, 50% yield) as white solid and product **3m** (68 mg, 49% yield) as white solid.

(R_p)-Tert-butyl (4^3-bromo-1,4(1,4)-dibenzenacyclohexaphane-1^2-yl)carbamate **(1m)**

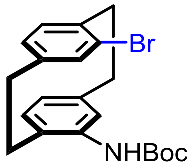

$[\alpha]^{25}_{D}$ = -47.6 (c = 0.5, $CHCl_3$). HPLC: Chiralpak IC column, 95:5 hexanes/ethanol, 1 ml/min; t_R = 10.57 min (minor), 11.62 min (major); 92% ee.

(S_p)-Dibenzyl-1-(4^2-bromo-1^5-((tert-butoxycarbonyl)amino)-1,4(1,4)-dibenzenacyclohexaphane-1^2-yl)hydrazine-1,2-dicarboxylate **(3m)**

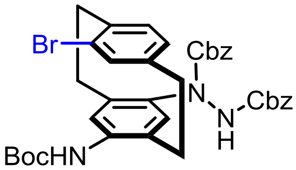

^{1}H NMR (400 MHz, $CDCl_3$) δ 7.51 – 7.26 (m, 9H), 7.26 – 6.87 (m, 4H), 6.64 – 6.37 (m, 2H), 6.25 – 6.15 (m, 2H), 5.38 – 4.91 (m, 4H), 3.37 – 2.46 (m, 8H), 1.54 (s, 9H). ^{13}C NMR (101 MHz, $CDCl_3$) δ 157.0, 155.5, 152.5, 140.7, 138.9, 137.3, 136.4, 135.6, 133.3, 132.0, 131.7, 130.0, 128.7, 128.6, 128.4, 128.4, 128.0, 125.9, 123.1, 80.9, 68.7, 68.1, 34.4, 32.7, 32.5, 30.3, 28.4. $[\alpha]^{25}_{D}$ = 52.1 (c = 0.5, $CHCl_3$). m/z HRMS (ESI) found $[M-56+H]^+$ 346.0429 $C_{17}H_{17}BrNO_2^+$calculated 346.0437. HPLC: Chiralpak IC

column, 70:30 hexanes/isopropanol, 1 ml/min; t_R = 11.70 min (minor), 13.38 min (major); 93% ee.

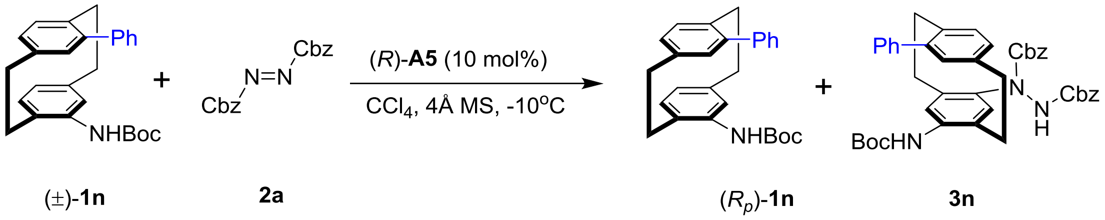

The reaction was performed on 0.2 mmol scale at −10 ℃ in CCl_4 (2 mL) for 13 h and the products were purified by column chromatography (petroleum ether/EtOAc = 8:1 – 2:1) to afford the recovered product (R_p)-**1n** (39 mg, 49% yield) as a yellow solid and product **3n** (68 mg, 49% yield) as a yellow solid.

(R_p)−Tert-butyl (4^3-phenyl-1,4(1,4)-dibenzenacyclohexaphane-1^2-yl)carbamate **(1n)**

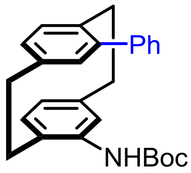

$[\alpha]^{25}_D$ = -13.2 (c = 0.5, $CHCl_3$). HPLC: Chiralpak ID column, 95:5 hexanes/isopropanol, 1 ml/min; t_R = 8.20 min (minor), 9.78 min (major); 93% ee.

(S_p)-Dibenzyl-1-(1^5-((tert-butoxycarbonyl)amino)-4^2-phenyl-1,4(1,4)-dibenzenacyclo hexaphane-1^2-yl)hydrazine-1,2-dicarboxylate **(3n)**

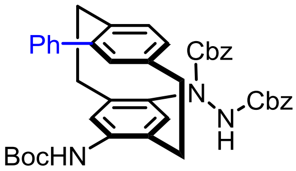

^{1}H NMR (500 MHz, $CDCl_3$) δ 7.51 – 7.26 (m, 15H), 7.15 (s, 1H), 6.99 (s, 1H), 6.75 – 6.50 (m, 3H), 6.40 – 6.10 (m, 2H), 5.35 – 5.15 (m, 3H), 5.03 (s, 1H), 3.31 – 3.10 (m, 3H), 3.05 – 2.70 (m, 4H), 2.45 – 2.25 (m, 1H), 1.60 (s, 9H). ^{13}C NMR (126 MHz, $CDCl_3$) δ 157.2, 155.6, 152.6, 141.7, 140.8, 137.5, 137.1, 136.4, 135.7, 133.6, 132.1,

129.7, 128.7, 128.6, 128.4, 128.3, 128.1, 127.0, 123.7, 81.0, 68.7, 68.1, 33.2, 32.5, 31.6, 31.1, 28.5. $[\alpha]^{25}_{D}$ = 42.1 (c = 0.5, $CHCl_3$). m/z HRMS (ESI) found $[M+H]^+$ 698.3239 $C_{43}H_{44}N_3O_6^+$ calculated 698.3225. HPLC: Chiralpak IA column, 70:30 hexanes/isopropanol, 1 ml/min; t_R = 12.92 min (minor), 24.58 min (major); 92% ee.

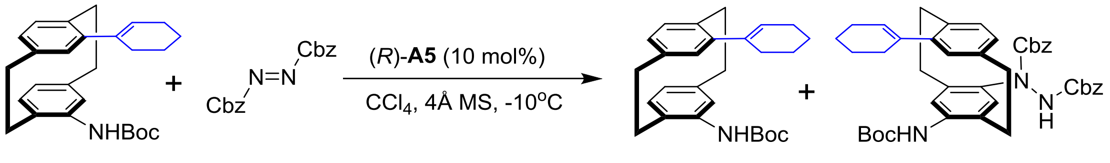

(±)-**1o** **2a** (R_p)-**1o** **3o**

The reaction was performed on 0.2 mmol scale at −10 ℃ in CCl_4 (2 mL) for 23.5 h and the products were purified by column chromatography (petroleum ether/EtOAc = 8:1 – 2:1) to afford the recovered product (R_p)-**1o** (38 mg, 48% yield) as a white solid and product **3o** (70 mg, 50% yield) as a white solid.

(R_p)−Tert-butyl-(4^3-(cyclohex-1-en-1-yl)-1,4(1,4)-dibenzenacyclohexaphane-1^2-yl)carbamate **(1o)**

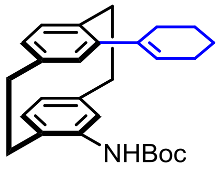

$[\alpha]^{25}_{D}$ = -90.8 (c = 0.5, $CHCl_3$). HPLC: Chiralpak IA column, 98:2 hexanes/isopropanol, 1 ml/min; t_R = 6.03 min (minor), 6.73 min (major); 97% ee.

(S_p)-Dibenzyl 1-(1^5-((tert-butoxycarbonyl)amino)-4^2-(cyclohex-1-en-1-yl)-1,4(1,4)-dibenzenacyclohexaphane-1^2-yl)hydrazine-1,2-dicarboxylate **(3o)**

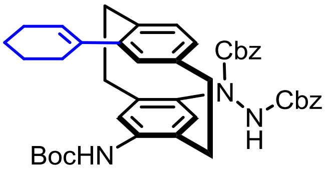

^{1}H NMR (500 MHz, $CDCl_3$) δ 7.45 – 7.31 (m, 10H), 7.13 (s, 1H), 6.76 (s, 1H), 6.70 – 6.40 (m, 3H), 6.25 – 5.90 (m, 2H), 5.71 (s, 1H), 5.30 – 4.90 (m, 4H), 3.20 – 2.80 (m,

6H), 2.75 – 2.55 (m, 2H), 2.40 – 2.30 (m, 1H), 2.25 – 2.05 (m, 3H), 1.76 – 1.58 (m, 4H), 1.52 (s, 9H). ^{13}C NMR (126 MHz, $CDCl_3$) δ 157.0, 155.6, 152.4, 144.0, 138.1, 137.5, 136.7, 136.1, 135.7, 132.8, 131.6, 128.7, 128.5, 128.4, 128.3, 128.1, 127.6, 127.3, 127.0, 126.2, 124.1, 80.7, 68.7, 68.0, 33.3, 32.4, 31.4, 31.0, 30.0, 28.4, 25.9, 23.4, 22.3. $[\alpha]^{25}_D$ = 63.4 (c = 0.5, $CHCl_3$). m/z HRMS (ESI) found $[M+H]^+$ 702.3556 $C_{43}H_{48}N_3O_6^+$ calculated 702.3538. HPLC: Chiralpak IA column, 70:30 hexanes/isopropanol, 1 ml/min; t_R = 8.97 min (minor), 13.32 min (major); 93% ee.

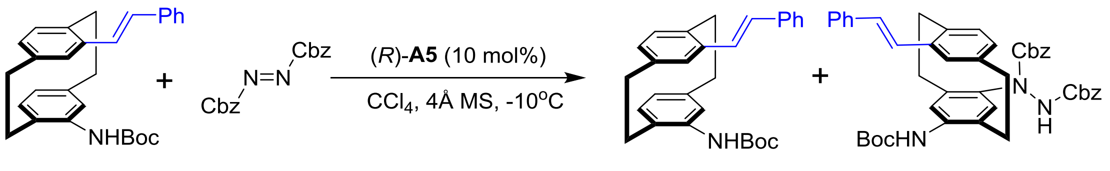

(±)-**1p** **2a** (R_p)-**1p** **3p**

The reaction was performed on 0.2 mmol scale at −10 ℃ in CCl_4 (2 mL) for 13 h and the products were purified by column chromatography (petroleum ether/EtOAc = 8:1 – 2:1) to afford the recovered product (R_p)-**1p** (41 mg, 48% yield) as a yellow solid and product **3p** (70 mg, 48% yield) as a white solid.

(R_p)−Tert-butyl (E)-(4^3-styryl-1,4(1,4)-dibenzenacyclohexaphane-1^2-yl)carbamate **(1p)**

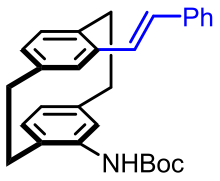

$[\alpha]^{25}_D$ = -43.8 (c = 0.5, $CHCl_3$). HPLC: Chiralpak IB column, 95:5 hexanes/isopropanol, 1 ml/min; t_R = 7.97 min (minor), 9.06 min (major); 98% ee.

(S_p)-Dibenzyl (E)-1-(1^5-((tert-butoxycarbonyl)amino)-4^2-styryl-1,4(1,4)-dibenzena cyclohexaphane-1^2-yl)hydrazine-1,2-dicarboxylate **(3p)**

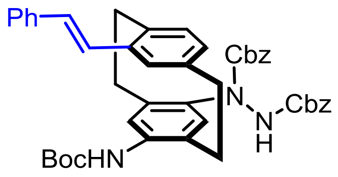

^{1}H NMR (400 MHz, $CDCl_3$) δ 7.57 –7.50 (m, 2H), 7.46 – 7.25 (m, 12H), 7.25 – 7.02 (m, 4H), 6.94 – 6.71 (m, 2H), 6.68 – 6.39 (m, 2H), 6.30 – 6.05 (m, 2H), 5.35 – 4.96 (m, 4H), 3.49 – 2.58 (m, 8H), 1.46 (s, 9H). ^{13}C NMR (101 MHz, $CDCl_3$) δ 157.1, 155.6, 152.4, 138.8, 137.9, 137.2, 136.6, 135.7, 134.9, 133.2, 131.8, 129.4, 128.7, 128.5, 128.5, 128.3, 128.3, 128.0, 127.5, 126.7, 126.7, 125.6, 124.2, 80.7, 68.7, 68.1, 32.8, 32.4, 31.9, 28.4. $[\alpha]^{25}_D$ = 103.4 (c = 0.5, $CHCl_3$). m/z HRMS (ESI) found $[M+H]^+$ 724.3356 $C_{45}H_{46}N_3O_6^+$ calculated 724.3381. HPLC: Chiralpak IB column, 80:20 hexanes/isopropanol, 1 ml/min; t_R = 17.91 min (minor), 23.91 min (major); 95% ee.

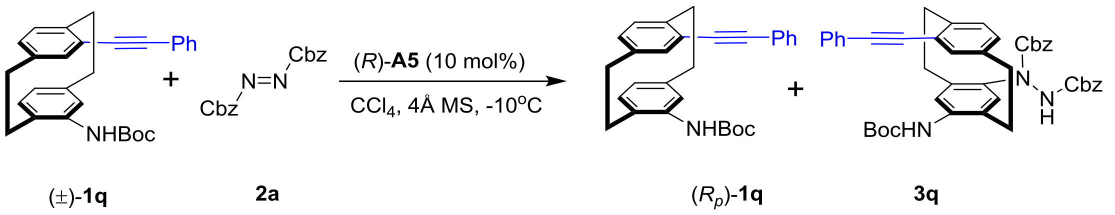

The reaction was performed on 0.2 mmol scale at −10 ℃ in CCl_4 (2 mL) for 13 h and the products were purified by column chromatography (petroleum ether/EtOAc = 8:1 – 2:1) to afford the recovered product (R_p)-**1q** (40 mg, 47% yield) as a yellow solid and product **3q** (71 mg, 49% yield) as a yellow solid.

(R_p)−Tert-butyl-(4^3-(phenylethynyl)-1,4(1,4)-dibenzenacyclohexaphane-1^2-yl)carbamate **(1q)**

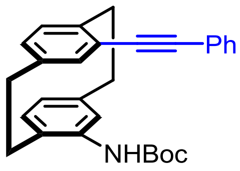

$[\alpha]^{25}_D$ = 108.0 (c = 0.5, $CHCl_3$). HPLC: Chiralpak IA column, 95:5 hexanes/isopropanol, 1 ml/min; t_R = 7.14 min (minor), 8.42 min (major); 98% ee.

(S_p)-Dibenzyl-1-(1^5-((tert-butoxycarbonyl)amino)-4^2-(phenylethynyl)-1,4(1,4)-dibenzena cyclohexaphane-1^2-yl)hydrazine-1,2-dicarboxylate **(3q)**

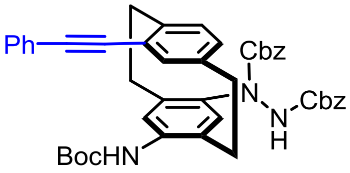

^{1}H NMR (400 MHz, $CDCl_3$) δ 7.59 – 7.57 (m, 2H), 7.50 – 7.19 (m, 13H), 7.18 – 6.80 (m, 3H), 6.70 – 6.40 (m, 2H), 6.35 – 6.10 (s, 2H), 5.42 – 4.92 (m, 4H), 3.68 – 2.45 (m, 8H), 1.53 (s, 9H). ^{13}C NMR (101 MHz, $CDCl_3$) δ 157.0, 155.6, 152.5, 142.1, 138.7, 137.3, 136.4, 135.7, 132.9, 132.1, 131.8, 131.6, 130.3, 128.7, 128.6, 128.5, 128.4, 128.3, 128.2, 128.0, 124.7, 124.1, 123.7, 92.7, 89.0, 80.7, 68.7, 68.1, 33.3, 32.9, 32.5, 30.8, 28.4. $[\alpha]^{25}_D$ = 23.2 (c = 0.5, $CHCl_3$). m/z HRMS (ESI) found $[M+H]^+$ 722.3198 $C_{45}H_{44}N_3O_6^+$ calculated 722.3225. HPLC: Chiralpak IB column, 70:30 hexanes/isopropanol, 1 ml/min; t_R = 9.31 min (minor), 11.99 min (major); 94% ee.

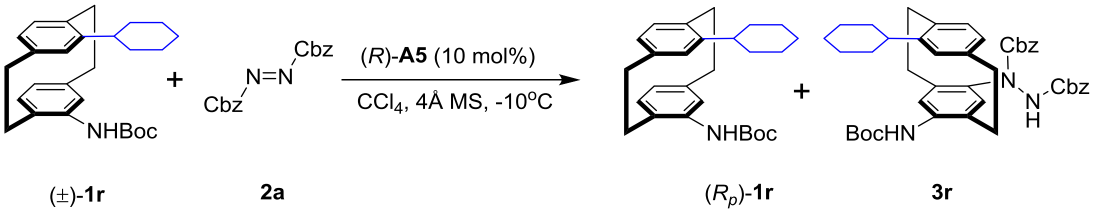

The reaction was performed on 0.2 mmol scale at −10 ℃ in CCl_4 (2 mL) for 12 h and the products were purified by column chromatography (petroleum ether/EtOAc = 8:1 – 2:1) to afford the recovered product (R_p)-**1r** (38 mg, 47% yield) as a white solid and product **3r** (70 mg, 50% yield) as a white solid.

(R_p)−Tert-butyl (4^3-cyclohexyl-1,4(1,4)-dibenzenacyclohexaphane-1^2-yl)carbamate **(1r)**

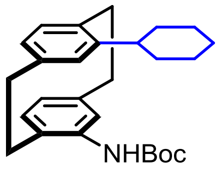

$[\alpha]^{25}_{D}$ = -18.3 (c = 0.5, $CHCl_3$). HPLC: Chiralpak IB column, 95:5 hexanes/isopropanol, 1 ml/min; t_R = 5.58 min (minor), 7.24 min (major); 99% ee.

(S_p)-Dibenzyl 1-(1^5-((tert-butoxycarbonyl)amino)-4^2-cyclohexyl-1,4(1,4)-dibenzenacyclohexaphane-1^2-yl)hydrazine-1,2-dicarboxylate **(3r)**

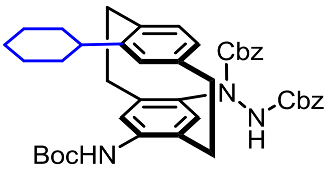

^{1}H NMR (400 MHz, $CDCl_3$) δ 7.50 – 7.50 (m, 10H), 7.15 (s, 1H), 6.93 – 5.84 (m, 6H), 5.15 (d, J = 92.2 Hz, 4H), 3.34 – 2.34 (m, 8H), 1.94 (d, J = 51.6 Hz, 2H), 1.70 (s, 3H), 1.54 (d, J = 18.3 Hz, 9H), 1.48 – 0.78 (m, 6H). ^{13}C NMR (101 MHz, $CDCl_3$) δ 157.0, 155.9, 152.3, 146.0, 137.8, 137.1, 136.8, 135.8, 134.2, 133.0, 130.4, 128.7, 128.6, 128.5, 128.3, 128.3, 128.1, 126.9, 124.6, 123.6, 80.7, 68.6, 68.1, 40.3, 37.5, 32.7, 31.7, 31.3, 30.2, 29.8, 28.4, 27.5, 26.9, 26.5. $[\alpha]^{25}_{D}$ = 20.3 (c = 0.5, $CHCl_3$). m/z HRMS (ESI) found $[M+H]^+$ 704.3672 $C_{43}H_{50}N_3O_6^+$ calculated 704.3694. HPLC: Chiralpak IB column, 90:10 hexanes/isopropanol, 1 ml/min; t_R = 22.69 min (minor), 26.36 min (major); 92% ee.

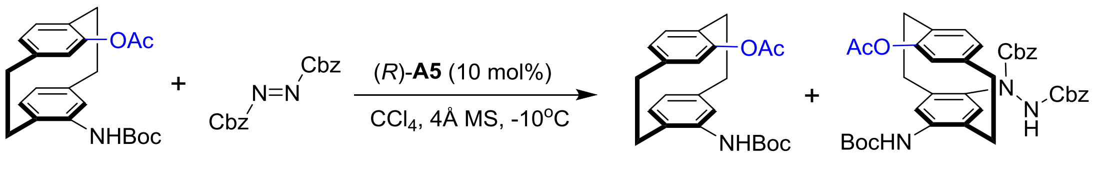

(±)-**1s** **2a** (R_p)-**1s** **3s**

The reaction was performed on 0.2 mmol scale at −10 ℃ in CCl_4 (2 mL) for 25 h and the products were purified by column chromatography (petroleum ether/EtOAc = 8:1 – 1:1) to afford the recovered product (R_p)-**1s** (36 mg, 47% yield) as a white solid and

product **3s** (68 mg, 50% yield) as a white solid.

(R_p)−4[3]-((tert-butoxycarbonyl)amino)-1,4(1,4)-dibenzenacyclohexaphane-1[2]-yl acetate **(1s)**

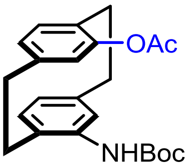

$[\alpha]^{25}_{D}$ = -40.5 (c = 0.5, $CHCl_3$). HPLC: Chiralpak IA column, 80:20 hexanes/isopropanol, 1 ml/min; t_R = 5.42 min (minor), 6.24 min (major); 94% ee.

(S_p)-Dibenzyl 1-(4[2]-acetoxy-1[5]-((tert-butoxycarbonyl)amino)-1,4(1,4)-dibenzena cyclohexaphane-1[2]-yl)hydrazine-1,2-dicarboxylate **(3s)**

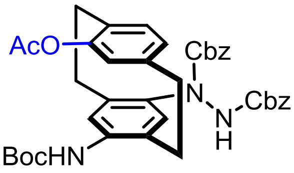

^{1}H NMR (400 MHz, $CDCl_3$) δ 7.49 – 7.31 (m, 10H), 7.10 – 6.85 (m, 1H), 6.70 – 6.39 (m, 4H), 6.12 (s, 1H), 5.40 – 4.93 (m, 5H), 3.27 – 2.55 (m, 8H), 2.32 (s, 3H), 1.54 (s, 9H). ^{13}C NMR (126 MHz, $CDCl_3$) δ 169.3, 157.0, 152.5, 149.2, 140.4, 137.4, 136.9, 135.7, 134.8, 133.6, 130.0, 128.7, 128.6, 128.5, 128.4, 128.3, 128.0, 127.1, 123.3, 122.2, 80.6, 68.7, 68.1, 32. 7, 32.5, 31.0, 29.9, 28.49, 21.5. $[\alpha]^{25}_{D}$ = 41.4 (c = 0.5, $CHCl_3$). m/z HRMS (ESI) found $[M+H]^+$ 680.2958 $C_{39}H_{42}N_3O_8^+$ calculated 680.2966. HPLC: Chiralpak IA column, 80:20 hexanes/isopropanol, 1 ml/min; t_R = 15.76 min (minor), 19.41 min (major); 87% ee.

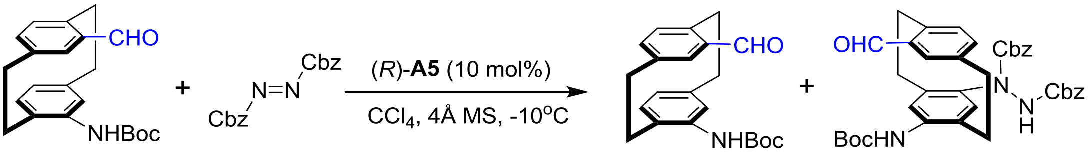

(±)-**1t** **2a** (R_p)-**1t** **3t**

The reaction was performed on 0.2 mmol scale at −10 ℃ in CCl_4 (2 mL) for 13 h and the products were purified by column chromatography (petroleum ether/EtOAc = 8:1 – 2:1) to afford the recovered product (*R*$_p$)-**1t** (36 mg, 51% yield) as a yellow solid and product **3t** (61 mg, 47% yield) as a yellow solid.

(*R*$_p$)−Tert-butyl (4^3-formyl-1,4(1,4)-dibenzenacyclohexaphane-1^2-yl)carbamate **(1t)**

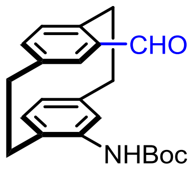

$[\alpha]^{25}_D$ = -58.0 (c = 0.5, $CHCl_3$). HPLC: Chiralpak ID column, 85:15 hexanes/isopropanol, 1 ml/min; t_R = 11.82 min (minor), 13.49 min (major); 87%

(*S*$_p$)-Dibenzyl 1-(1^5-((tert-butoxycarbonyl)amino)-4^2-formyl-1,4(1,4)-dibenzena cyclohexaphane-1^2-yl)hydrazine-1,2-dicarboxylate **(3t)**

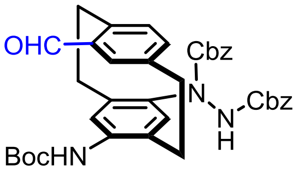

^{1}H NMR (400 MHz, $CDCl_3$) δ 9.88 (s, 1H), 7.47 – 7.28 (m, 10H), 7.12 (s, 1H), 6.72 – 6.57 (m, 2H), 6.55 – 6.32 (m, 2H), 6.20 (s, 1H), 5.27 – 4.95 (m, 5H), 4.02 – 3.85 (m, 1H), 3.31 – 2.92 (m, 5H), 2.82 – 2.60 (m, 2H), 1.55 (s, 9H). ^{13}C NMR (101 MHz, $CDCl_3$) δ 192.6, 157.1, 156.7, 152.4, 142.7, 139.2, 137.9, 137.3, 136.8, 136.4, 135.6, 135.5, 134.3, 131.9, 128.8, 128.7, 128.6, 128.5, 128.4, 128.0, 125.2, 81.0, 68.8, 68.1, 32.6, 32.3, 32.0, 31.9, 28.4. $[\alpha]^{25}_D$ = 68.7 (c = 0.5, $CHCl_3$). m/z HRMS (ESI) found $[M+H]^+$ 650.2875 $C_{38}H_{40}N_3O_7^+$ calculated 650.2861. HPLC: Chiralpak IA column, 70:30 hexanes/isopropanol, 1 ml/min; t_R = 16.48 min (minor), 23.22 min (major); 95% ee.

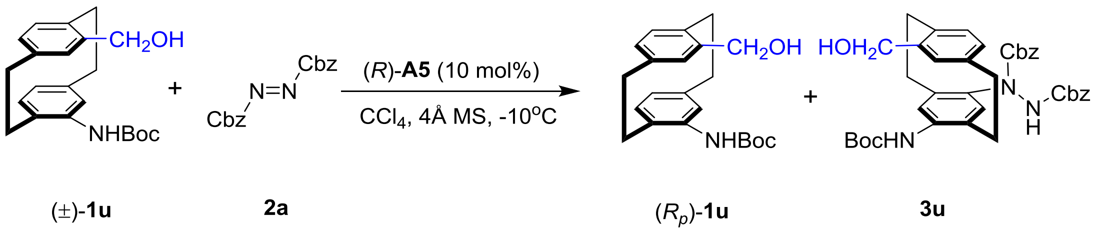

The reaction was performed on 0.2 mmol scale at −10 ℃ in CCl_4 (2 mL) for 15 h and the products were purified by column chromatography (petroleum ether/EtOAc = 8:1 – 1:2) to afford the recovered product (R_p)-**1u** (34 mg, 48% yield) as a white solid and product **3u** (65 mg, 50% yield) as a white solid.

(R_p)−Tert-butyl-(4^3-(hydroxymethyl)-1,4(1,4)-dibenzenacyclohexaphane-1^2-yl)carbamate **(1u)**

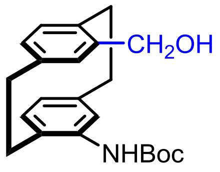

$[\alpha]^{25}_D$ = -112.2 (c = 0.5, $CHCl_3$). HPLC: Chiralpak IA column, 85:15 hexanes/isopropanol, 1 ml/min; t_R = 7.51 min (minor), 8.33 min (major); 97%.

(S_p)-Dibenzyl-1-(1^5-((tert-butoxycarbonyl)amino)-4^2-(hydroxymethyl)-1,4(1,4)-dibenzena cyclohexaphane-1^2-yl)hydrazine-1,2-dicarboxylate **(3u)**

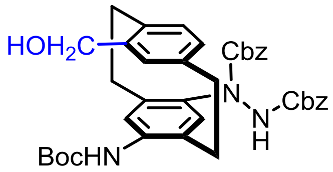

^{1}H NMR (400 MHz, $CDCl_3$) δ 7.51 – 7.26 (m, 9H), 7.09 (s, 3H), 6.79 (s, 1H), 6.63 – 6.35 (m, 3H), 6.18 (s, 1H), 5.40 – 4.95 (m, 4H), 4.75 – 4.45 (m, 2H), 3.60 – 3.30 (m, 1H), 3.25 – 3.04 (m, 3H), 3.01 – 2.55 (m, 4H), 1.58 (d, J = 4.8 Hz, 9H). ^{13}C NMR (101 MHz, $CDCl_3$) δ 157.2, 155.7, 153.8, 139.9, 138.5, 137.5, 136.8, 136.3, 135.7, 135.0, 132.8, 131.4, 128.7, 128.6, 128.3, 128.0, 126.4, 121.9, 81.5, 68.7, 68.1, 62.9, 32.7, 32.5, 31.3, 29.8, 28.5. $[\alpha]^{25}_D$ = 63.6 (c = 0.5, $CHCl_3$). m/z HRMS (ESI) found

[M+17+H]$^+$ 669.3267 $C_{38}H_{45}N_4O_7^+$ calculated 669.3283. HPLC: Chiralpak IA column, 85:15 hexanes/isopropanol, 1 ml/min; t_R = 29.97 min (minor), 33.46 min (major); 94% ee.

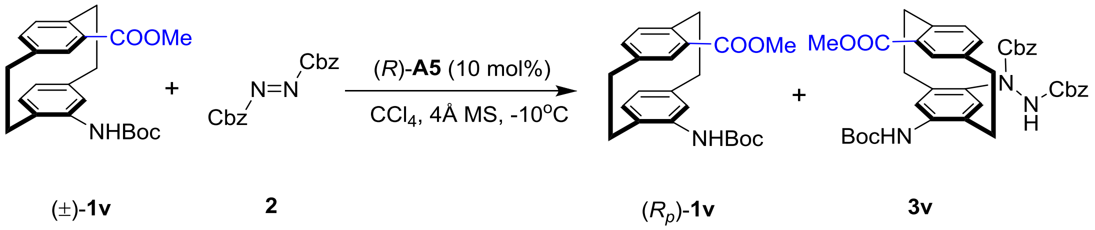

The reaction was performed on 0.2 mmol scale at −10 ℃ in CCl_4 (2 mL) for 13 h and the products were purified by column chromatography (petroleum ether/EtOAc = 8:1 – 1:2) to afford the recovered product (R_p)-**1v** (37 mg, 49% yield) as a yellow solid and product **3v** (66 mg, 49% yield) as a yellow solid.

(R_p)−Methyl 4^3-((tert-butoxycarbonyl)amino)-1,4(1,4)-dibenzenacyclohexaphane-1^2 -carboxylate **(1v)**

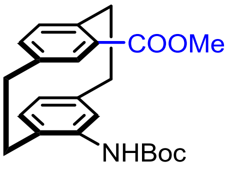

$[\alpha]^{25}_D$ = -48.8 (c = 0.5, $CHCl_3$). HPLC: Chiralpak IC column, 90:10 hexanes/isopropanol, 1 ml/min; t_R = 9.00 min (major), 11.82 min (minor); 92%.

(S_p)-Dibenzyl-1-(1^5-((tert-butoxycarbonyl)amino)-4^2-(methoxycarbonyl)-1,4(1,4)-dibenzenacyclohexaphane-1^2-yl)hydrazine-1,2-dicarboxylate **(3v)**

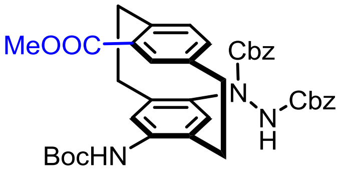

^{1}H NMR (400 MHz, $CDCl_3$) δ 7.49 – 7.27 (m, 9H), 7.17 (s, 2H), 6.73 – 6.15 (m, 5H), 5.63 – 4.77 (m, 5H), 3.90 (s, 3H), 3.80 (s, 1H), 3.29 – 2.58 (m, 7H), 1.54 (s, 9H). ^{13}C

NMR (126 MHz, $CDCl_3$) δ 168.2, 157.0, 155.67, 152.4, 141.8, 138.8, 137.6, 136.7, 136.3, 135.6, 134.2, 130.8, 130.2, 128.7, 128.7, 128.6, 128., 128.34, 128.0, 80.6, 68.7, 68.1, 52.1, 34.4, 32.6, 31.7, 29.8, 28.5. $[\alpha]^{25}_D$ = 62.4 (c = 0.5, $CHCl_3$). m/z HRMS (ESI) found $[M+H]^+$ 680.2957 $C_{39}H_{42}N_3O_8^+$ calculated 680.2966. HPLC: Chiralpak IC column, 80:20 hexanes/isopropanol, 1 ml/min; t_R = 13.16 min (minor), 17.60 min (major); 95% ee.

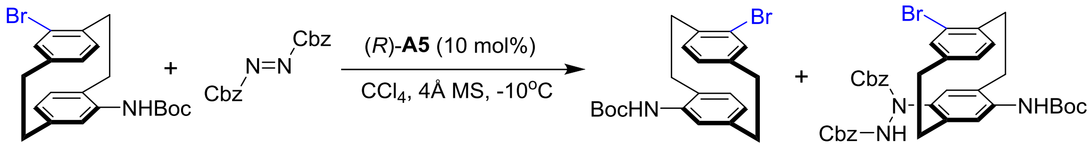

(±)-**1w** **2a** (R_p)-**1w** **3w**

The reaction was performed on 0.2 mmol scale at −10 ℃ in CCl_4 (2 mL) for 12 h and the products were purified by column chromatography (petroleum ether/EtOAc = 8:1 – 2:1) to afford the recovered product (R_p)-**1w** (42 mg, 52% yield) as a yellow solid and product **3w** (68 mg, 49% yield) as a white solid.

(R_p)-Tert-butyl (4^2-bromo-1,4(1,4)-dibenzenacyclohexaphane-1^2-yl)carbamate **(1w)**

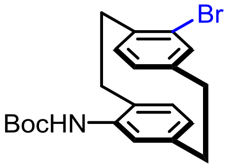

$[\alpha]^{25}_D$ = -199.2 (c = 0.5, $CHCl_3$). HPLC: Chiralpak IC column, 95:5 hexanes/ethanol, 1 ml/min; t_R = 5.76 min (minor), 10.07 min (major); 95% ee.

(S_p)-Dibenzyl-1-(4^3-bromo-1^5-((tert-butoxycarbonyl)amino)-1,4(1,4)-dibenzenacyclohexaphane-1^2-yl)hydrazine-1,2-dicarboxylate **(3w)**

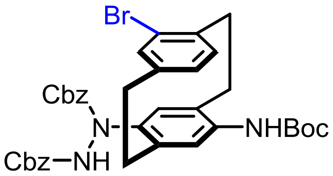

^{1}H NMR (400 MHz, $CDCl_3$) δ 7.50 – 7.27 (m, 7H), 7.25 – 6.75 (m, 4H), 6.71 – 6.52 (m, 2H), 6.27 (s, 1H), 5.49 – 4.85 (m, 5H), 3.48 – 3.12 (m, 2H), 3.04 – 2.65 (m, 6H), 1.56 (s, 9H). ^{13}C NMR (101 MHz, $CDCl_3$) δ 156.8, 155.4, 152.6, 137.5, 135.7, 134.8, 134.5, 131.0, 129.8, 128.6, 128.5, 128.2, 127.8, 126.9, 124.3, 80.9, 68.6, 68.1, 33.7, 33.5, 31.3, 29.6, 28.4. $[\alpha]^{25}_{D}$ = 104.2 (c = 0.5, $CHCl_3$). m/z HRMS (ESI) found $[M+H]^+$ 700.2004 $C_{37}H_{39}BrN_3O_6^+$ calculated 700.2017. HPLC: Chiralpak IC column, 80:20 hexanes/isopropanol, 1 ml/min; t_R = 14.90 min (major), 20.07 min (minor); 98% ee.

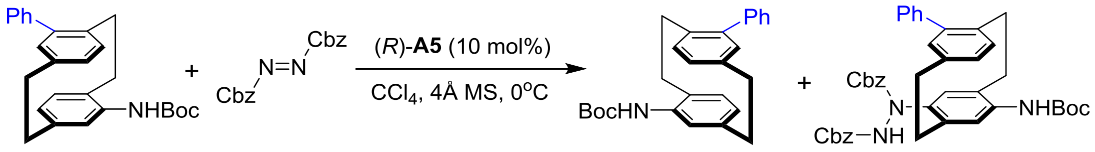

(±)-**1x** **2a** (R_p)-**1x** **3x**

The reaction was performed on 0.2 mmol scale with 0.65 equiv. **2a** at 0 ℃ in CCl_4 (2 mL) for 44 h and the products were purified by column chromatography (petroleum ether/EtOAc = 8:1 – 2:1) to afford the recovered product (R_p)-**1x** (40 mg, 50% yield) as white a solid and product **3x** (67 mg, 48% yield) as a white solid.

(R_p)-Tert-butyl (4^2-phenyl-1,4(1,4)-dibenzenacyclohexaphane-1^2-yl)carbamate **(1x)**

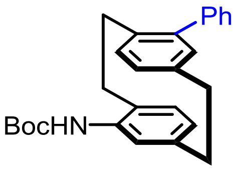

$[\alpha]^{25}_{D}$ = -259.9 (c = 0.5, $CHCl_3$). HPLC: Chiralpak IC column, 95:05 hexanes/ethanol, 1 ml/min; t_R = 6.25 min (minor), 8.81 min (major); 93% ee.

(S_p)-Dibenzyl-1-(1^5-((tert-butoxycarbonyl)amino)-4^3-phenyl-1,4(1,4)--dibenzenacyclo hexaphane-1^2-yl)hydrazine-1,2-dicarboxylate **(3x)**

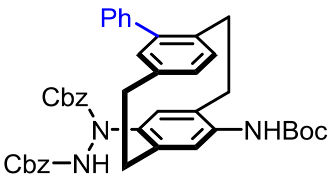

^{1}H NMR (400 MHz, $CDCl_3$) δ 7.62 – 7.29 (m, 10H), 7.25 – 6.88 (m, 6H), 6.81 – 6.14 (m, 5H), 5.53 – 4.83 (m, 5H), 3.52 – 3.40 (m, 1H), 3.25 – 2.55 (m, 6H), 2.25 – 2.07 (m, 1H), 1.58 (s, 9H). ^{13}C NMR (101 MHz, $CDCl_3$) δ 157.5, 152.7, 141.3, 141.0, 137.6, 136.2, 135.6, 135.3, 133.4, 131.3, 129.2, 128.7, 128.6, 128.5, 128.5, 128.2, 127.9, 126.8, 125.6, 80.8, 68.8, 68.5, 34.2, 33.1, 32.7, 31.7, 28.5. $[\alpha]^{25}_{D}$ = 40.5 (c = 0.5, $CHCl_3$). m/z HRMS (ESI) found $[M+H]^+$ 698.3214 $C_{43}H_{44}N_3O_6^+$ calculated 698.3225. HPLC: Chiralpak IA column, 80:20 hexanes/isopropanol, 1 ml/min; t_R = 18.60 min (major), 24.33 min (minor); 94% ee.

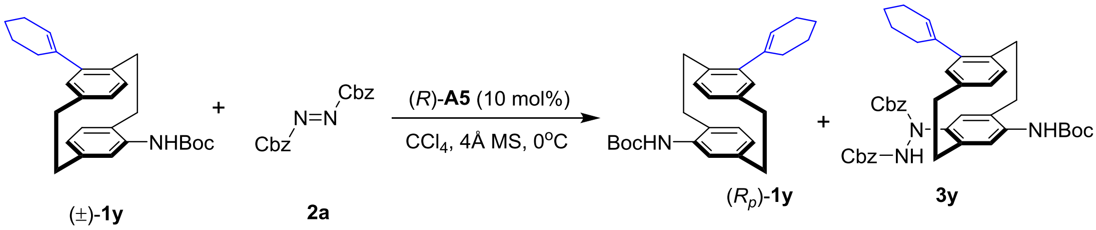

The reaction was performed on 0.2 mmol scale with 0.65 equiv. **2a** at 0 ℃ in CCl_4 (2 mL) for 44 h and the products were purified by column chromatography (petroleum ether/EtOAc = 8:1 – 2:1) to afford the recovered product (*R*$_p$)-**1y** (39 mg, 48% yield) as a white solid and product **3y** (69 mg, 49% yield) as a white solid.

(*R*$_p$)−Tert-butyl-(4^2-(cyclohex-1-en-1-yl)-1,4(1,4)-dibenzenacyclohexaphane-1^{2}yl)carbamate **(1y)**

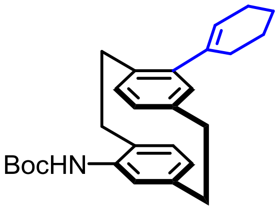

$[\alpha]^{25}_{D}$ = -256.4 (c = 0.5, $CHCl_3$). HPLC: Chiralpak IC column, 90:10 hexanes/ethanol, 1 ml/min; t_R = 5.19 min (minor), 7.18 min (major); 97% ee.

(S_p)-Dibenzyl 1-(1^5-((tert-butoxycarbonyl)amino)-4^3-(cyclohex-1-en-1-yl)-1,4(1,4)-dibenzenacyclohexaphane-1^2-yl)hydrazine-1,2-dicarboxylate **(3y)**

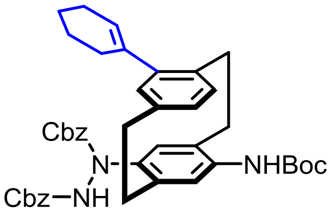

^{1}H NMR (400 MHz, $CDCl_3$) δ 7.57 – 7.27 (m, 8H), 7.23 (s, 3H), 6.90 – 6.75 (m, 1H), 6.70 – 6.08 (m, 4H), 5.95 – 5.75 (m, 1H), 5.54 – 4.65 (m, 5H), 3.77 – 2.42 (m, 9H), 2.36 – 1.76 (m, 5H), 1,70 –1.59 (m, 2H), 1.57 (s, 9H). ^{13}C NMR (101 MHz, $CDCl_3$) δ 157.1, 155.9, 152.7, 143.4, 138.2, 137.4, 135.7, 135.4, 133.3, 130.6, 129.5, 128.7, 128.6, 128.5, 128.2, 127.8, 127.0, 126.4, 125.7, 80.7, 68.6, 68.4, 34.3, 33.1, 32.4, 29.7, 28.5, 28.3, 25.8, 23.5, 22.3. $[\alpha]^{25}_{D}$ = 80.8 (c = 0.5, $CHCl_3$). m/z HRMS (ESI) found $[M+H]^+$ 702.3518 $C_{43}H_{48}N_3O_6^+$ calculated 702.3538. HPLC: Chiralpak IA column, 85:15 hexanes/isopropanol, 1 ml/min; t_R = 26.84 min (major), 34.81 min (minor); 96% ee.

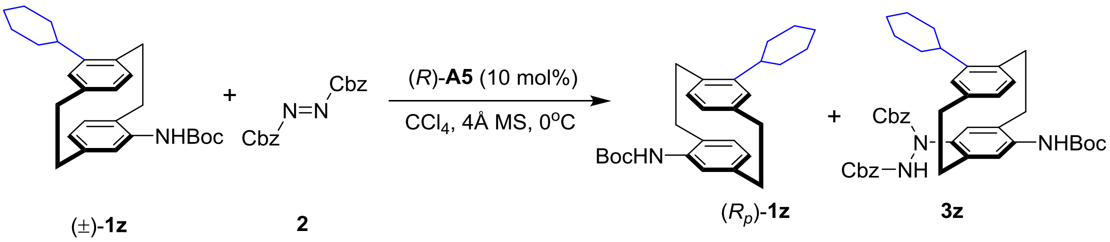

The reaction was performed on 0.2 mmol scale with 0.65 equiv. **2a** at 0 ℃ in CCl_4 (2 mL) for 45 h and the products were purified by column chromatography (petroleum ether/EtOAc = 8:1 – 2:1) to afford the recovered product (R_p)−**1z** (40 mg, 49% yield) as a white solid and product **3z** (66 mg, 47 % yield) as a white solid

(R_p)-Tert-butyl (4[2]-cyclohexyl-1,4(1,4)-dibenzenacyclohexaphane-1[2]-yl)carbamate **(1z)**

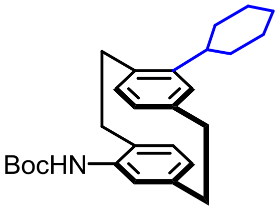

$[\alpha]^{25}_D$ = -106.3 (c = 0.5, $CHCl_3$). HPLC: Chiralpak IC column, 95:5 hexanes/ethanol, 1 ml/min; t_R = 5.76 min (minor), 9.85 min (major); 90% ee.

(S_p)-Dibenzyl-1-(1[5]-((tert-butoxycarbonyl)amino)-4[3]-cyclohexyl-1,4(1,4)-dibenzenacyclohexaphane-1[2]-yl)hydrazine-1,2-dicarboxylate **(3z)**

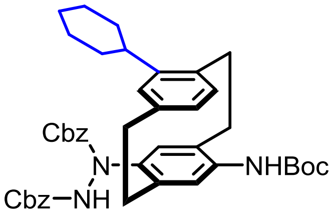

^{1}H NMR (400 MHz, $CDCl_3$) δ 7.52 – 7.29 (m, 7H), 7.25 – 7.12(m, 2H), 7.11 – 6.72 (m, 3H), 6.50 – 6.07 (m, 4H), 5.54 – 4.61 (m, 5H), 3.60 – 3.33 (m, 2H), 3.19 – 2.82 (m, 4H), 2.77 – 2.28 (m, 4H), 2.15 – 1.95 (m, 1H), 1.70 – 1.60 (m, 3H), 1.57 (s, 9H), 1.42 – 1.20 (m, 3H), 1.19 – 0.95 (m, 1H), 0.89 – 0.65 (m, 1H). ^{13}C NMR (126 MHz, $CDCl_3$) δ 157.0, 157.0, 152.7, 146.0, 138.6, 137.2, 136.2, 135.7, 135.4, 133.5, 130.0, 128.7, 128.6, 128.5, 128.1, 127.8, 80.7, 68.6, 68.4, 40.4, 37.5, 34.7, 33.0, 32.3, 32.1, 28.5, 27.6, 26.9, 26.3. $[\alpha]^{25}_D$ = 63.0 (c = 0.5, $CHCl_3$). m/z HRMS (ESI) found $[M+H]^+$ 704.3679 $C_{43}H_{50}N_3O_6^+$ calculated 704.3694. HPLC: Chiralpak IA column, 80:20 hexanes/isopropanol, 1 ml/min; t_R = 14.49 min (major), 21.26 min (minor); 98% ee.

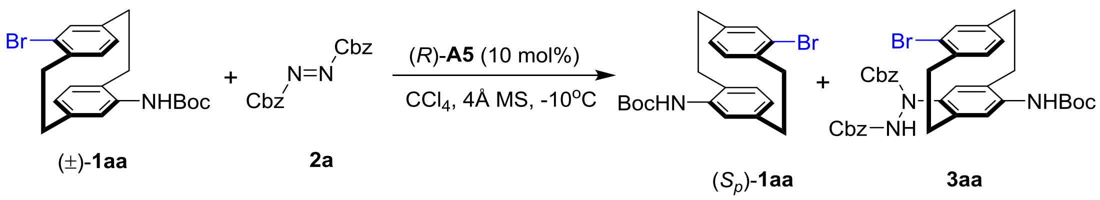

The reaction was performed on 0.2 mmol scale at −10 ℃ in CCl_4 (2 mL) for 36 h and the products were purified by column chromatography (petroleum ether/EtOAc = 8:1 – 2:1) to afford the recovered product (S_p)-**1aa** (38 mg, 48% yield) as a white solid and product **3aa** (69 mg, 49% yield) as a white solid.

(S_p)-Tert-butyl (4^3-bromo-1,4(1,4)-dibenzenacyclohexaphane-1^2-yl)carbamate **(1aa)**

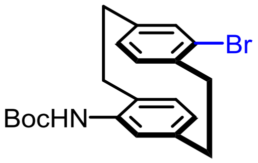

$[\alpha]^{25}_D$ = -22.1 (c = 0.5, $CHCl_3$). HPLC: Chiralpak IC column, 70:30 hexanes/isopropanol, 1 ml/min; t_R = 4.35 min (minor), 6.09 min (major); 86% ee.

(R_p)-Dibenzyl-1-(4^2-bromo-1^5-((tert-butoxycarbonyl)amino)-1,4(1,4)-dibenzenacyclo hexaphane-1^2-yl)hydrazine-1,2-dicarboxylate **(3aa)**

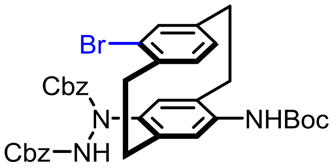

^{1}H NMR (400 MHz, $CDCl_3$) δ 7.38 –7.27 (m, 7H), 7.24 – 6.81 (m, 4H), 6.76 –6.60 (s, 1H), 6.20 –6.00 (s, 1H), 5.94 – 5.35 (m, 3H), 5.34 –4.70 (m, 5H), 3.80 –3.45 (m, 1H), 3.25 – 2.69 (m, 5H), 2.55 – 2.10 (m, 2H), 1.57 – 1.47 (m, 9H). ^{13}C NMR (101 MHz, $CDCl_3$) δ 162.3, 159.2, 155.7, 149.7, 142.1, 141.7, 140.7, 136.6, 135.6, 134.5, 130.9, 128.6, 128.5, 128.4, 127.8, 127.7, 124.8, 81.9, 69.3, 68.6, 67.6, 56.2, 55.5, 33.81, 33.0, 32.6, 31.2, 31.0, 28.5, 28.3. $[\alpha]^{25}_D$ = -30.6 (c = 0.5, $CHCl_3$). m/z HRMS (ESI) found $[M+H]^+$ 700.2018 $C_{37}H_{39}BrN_3O_6^+$ calculated 700.2017. HPLC: Chiralpak IC column, 70:30 hexanes/isopropanol, 1 ml/min; t_R = 8.69 min (major), 25.52 min (minor); 83% ee.

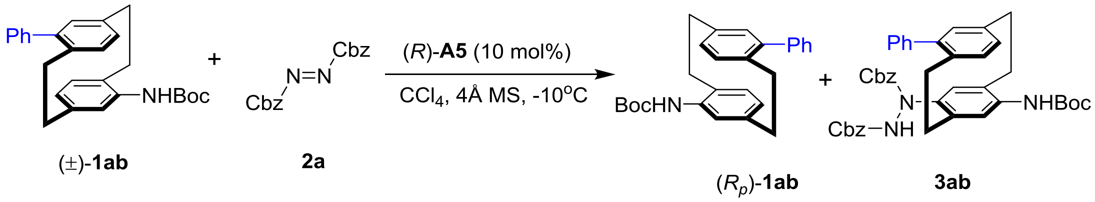

The reaction was performed on 0.2 mmol scale at −10 ℃ in CCl_4 (2 mL) for 36 h and the products were purified by column chromatography (petroleum ether/EtOAc = 8:1 – 2:1) to afford the recovered product (R_p)-**1ab** (38 mg, 48% yield) as a white solid and product **3ab** (70 mg, 50% yield) as a white solid.

(R_p)-Tert-butyl (4^3-phenyl-1,4(1,4)-dibenzenacyclohexaphane-1^2-yl)carbamate **(1ab)**

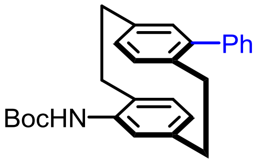

$[\alpha]^{25}_D$ = 2.0 (c = 0.5, $CHCl_3$). HPLC: Chiralpak IA column, 95:5 hexanes/isopropanol, 1 ml/min; t_R = 7.63 min (minor), 8.58 min (major); 93% ee.

(S_p)-Dibenzyl-1-(1^5-((tert-butoxycarbonyl)amino)-4^2-phenyl-1,4(1,4)-dibenzenacyclo hexaphane-1^2-yl)hydrazine-1,2-dicarboxylate **(3ab)**

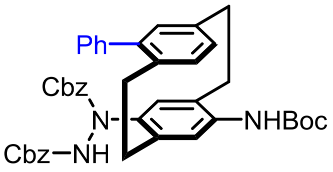

^{1}H NMR (400 MHz, $CDCl_3$) δ 7.54 – 7.26 (m, 10H), 7.24 – 7.10 (m, 3H), 7.11 – 6.79 (m, 5H), 6.07 (s, 1H), 5.90 – 5.70 (m, 1H), 5.65 – 5.50 (m, 1H), 5.30 – 5.10 (m, 1H), 5.10 – 4.90 (m, 2H), 4.83 (s, 1H), 4.63 (s, 1H), 3.45 – 2.85 (m, 5H), 2.50 – 1.85 (m, 3H), 1.56 (s, 9H). ^{13}C NMR (101 MHz, $CDCl_3$) δ 162.5, 159.1, 155.7, 149.8, 142.7, 141.5, 140.4, 140.0, 135.7, 135.0, 134.3, 133.5, 131.3, 130.5, 129.6, 128.9, 128.6, 128.5, 128.5, 128.1, 127.6, 81.9, 77.4, 68.3, 67.5, 55.2, 54.4, 34.6, 32.9, 31.6, 28.5, 28.3. $[\alpha]^{25}_D$ = -62.4 (c = 0.5, $CHCl_3$). m/z HRMS (ESI) found $[M+H]^+$ 698.3215 $C_{43}H_{44}N_3O_6^+$ calculated 698.3225. HPLC: Chiralpak IA column, 70:30 hexanes/isopropanol, 1 ml/min; t_R = 6.10 min (major), 7.25 min (minor); 88% ee.

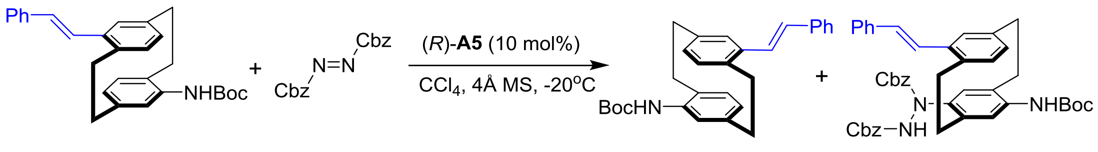

The reaction was performed on 0.2 mmol scale at −20 ℃ in toluene (2 mL) for 256 h and the products were purified by column chromatography (petroleum ether/EtOAc = 8:1 – 2:1) to afford the recovered product (R_p)-**1ac** (43 mg, 51% yield) as a yellow solid and product **3ac** (68 mg, 47% yield) as a yellow solid.

(R_p)−Tert-butyl (E)-(4^3-styryl-1,4(1,4)-dibenzenacyclohexaphane-1^2-yl)carbamate **(1ac)**

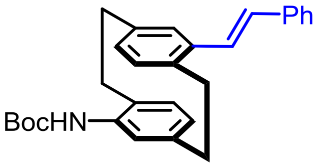

$[\alpha]^{25}_D$ = 28.3 (c = 0.5, $CHCl_3$). HPLC: Chiralpak IC column, 70:30 hexanes/ethanol, 1 ml/min; t_R = 5.33 min (minor), 7.49 min (major); 89% ee.

(S_p)-Dibenzyl-(E)-1-(1^5-((tert-butoxycarbonyl)amino)-4^2-styryl-1,4(1,4)-dibenzenacyclohexaphane-1^2-yl)hydrazine-1,2-dicarboxylate **(3ac)**

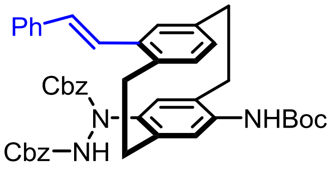

The reaction was performed on 0.2 mmol scale at −10 ℃ in toluene (2 mL) for 256 h and the products were purified by column chromatography (petroleum ether/EtOAc = 8:1 – 2:1) to afford the recovered product **1ac** (43 mg, 51% yield) as yellow solid and product **3ac** (68 mg, 47% yield) as yellow solid.

^{1}H NMR (400 MHz, $CDCl_3$) δ 7.62 – 7.26 (m, 11H), 7.24 – 7.04 (m, 5H), 6.98 – 6.75 (m, 3H), 6.57 (s, 2H), 6.40 – 6.11 (m, 2H), 5.37 – 4.63 (m, 5H), 3.65 – 3.45 (m, 1H), 3.20 – 2.55 (m, 7H), 1.57 (s, 2.6, 9H). ^{13}C NMR (101 MHz, $CDCl_3$) δ 156.0, 154.7, 152.8, 138.6, 136.6, 135.8, 135.6, 135.2, 134.3, 129.6, 129.2, 129.1, 128.8, 128.7, 128.6, 128.5, 128.2, 128.0, 127.7, 126.6, 126.4, 125.8, 125.6, 124.8, 80.7, 68.7, 68.3, 67.7, 33.8, 33.5, 33.4, 31.3, 30.6, 30.4, 28.5. $[\alpha]^{25}_{D}$ = 112.8 (c = 0.5, $CHCl_3$). m/z HRMS (ESI) found $[M+H]^+$ 724.3391 $C_{45}H_{46}N_3O_6^+$ calculated 724.3381. HPLC: Chiralpak IB column, 70:30 hexanes/isopropanol, 1 ml/min; t_R = 16.18 min (major), 26.78 min (minor); 98% ee.

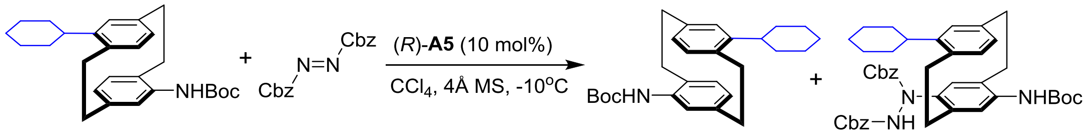

(±)-**1ad** **2a** (R_p)-**1ad** **3ad**

The reaction was performed on 0.2 mmol scale at −10 ℃ in CCl_4 (2 mL) for 24 h and the products were purified by column chromatography (petroleum ether/EtOAc = 8:1 – 2:1) to afford the recovered product (R_p)-**1ad** (38 mg, 47% yield) as a white solid and product **3ad** (70 mg, 50% yield) as a white solid.

(R_p)-Tert-butyl (4^3-cyclohexyl-1,4(1,4)-dibenzenacyclohexaphane-1^2-yl)carbamate **(1ad)**

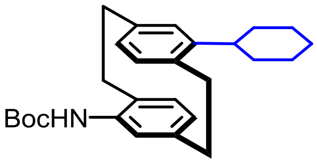

$[\alpha]^{25}_{D}$ = -103.4 (c = 0.5, $CHCl_3$). HPLC: Chiralpak IB column, 80:20 hexanes/ethanol, 1 ml/min; t_R = 5.07 min (minor), 18.89 min (major); 97% ee.

(S_p)-Dibenzyl-1-(1^5-((tert-butoxycarbonyl)amino)-4^2-cyclohexyl-1,4(1,4)-dibenzenacyclohexaphane-1^2-yl)hydrazine-1,2-dicarboxylate **(3ad)**

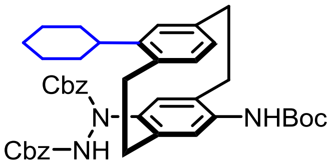

^{1}H NMR (400 MHz, $CDCl_3$) δ 7.60 – 7.27 (m, 7H), 7.25 – 7.00 (m, 2H), 7.00 – 6.22 (m, 4H), 6.10 – 5.70 (m, 1H), 5.60 – 5.30 (m, 2H), 5.31 – 4.50 (m, 5H), 3.63 – 2.42 (m, 8H), 2.38 – 1.63 (m, 8H), 1.60 – 1.45 (m, 9H), 1.43 – 0.99 (m, 3H). ^{13}C NMR (101 MHz, $CDCl_3$) δ 162.6, 159.7, 159.4, 156.3, 155.6, 152.6, 149.6, 146.2, 145.0, 140.5, 139.7, 139.5, 138.0, 136.9, 135.7, 134.4, 129.3, 128.6, 128.5, 128.5, 128.3, 128.2, 128.1, 127.7, 127.5, 127.1, 127.0, 126.1, 81.7, 80.6, 77.4, 69.2, 68.8, 68.4, 68.1, 67.5, 65.2, 56.3, 55.6, 40.3, 39.9, 37.7, 37.3, 32.9, 32.7, 30.9, 30.3, 28.5, 28.3, 27.3, 27.2, 26.8, 26.3. $[\alpha]^{25}_D$ = 3.1 (c = 0.5, $CHCl_3$). m/z HRMS (ESI) found $[M+H]^+$ 704.3691 $C_{43}H_{50}N_3O_6^+$ calculated 704.3694. HPLC: Chiralpak IB column, 95:05 hexanes/isopropanol, 1 ml/min; t_R = 12.46 min (major), 17.46 min (minor); 90% ee.

1.4 Enantioselective desymmetrization of diamido-PCP

Synthesis of achiral diamido-PCP 4a

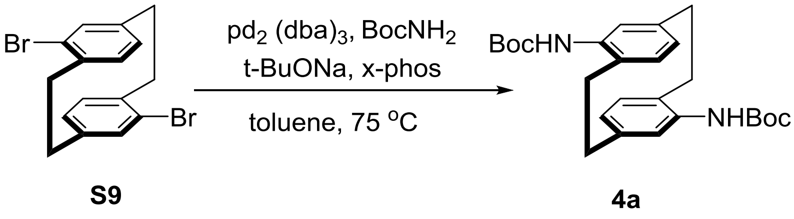

To a solution of **S9** (1.1 g, 3.0 mmol, 1.0 equiv.), $BocNH_2$(1.48 g, 12.6 mmol, 4.2 equiv.), t-BuONa (1.2 g, 12.6 mmol, 4.2 equiv.), x-Phos (215 mg, 0.45 mmol, 0.15 equiv.) in toluene (30 mL) was added $Pd_2(dba)_3$ (137 mg, 0.15 mmol, 0.05 equiv.) under N_2 atmosphere. After stirring at 100°C under N_2 atmosphere overnight, the reaction mixture was cooled to rt and concentrated to give a residue, which was purified by column chromatography (DCM:MeOH = 50:1) to afford crude product which was triturated with EA to afford **4a** (880 mg, 67%) as a white solid.

Di-tert-butyl 1,4(1,4)-dibenzenacyclohexaphane-1^2,4^3-diyldicarbamate **(4a)**

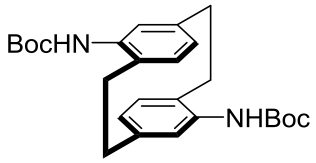

^{1}H NMR (400 MHz, $CDCl_3$) δ 6.74 (s, 2H), 6.56 (dd, J = 7.8, 1.8 Hz, 2H), 6.43 (d, J = 7.8 Hz, 2H), 6.27 (s, 2H), 3.25 – 3.15 (m, 2H), 3.11 – 2.91 (m, 4H), 2.87 – 2.73 (m, 2H), 1.55 (s, 18H). ^{13}C NMR (101 MHz, $CDCl_3$) δ 152.8, 140.1, 137.3, 134.2, 124.7, 80.5, 77.4, 33.2, 32.3, 28.5. m/z HRMS (ESI) found $[M+H]^+$ 739.2596 $C_{26}H_{35}N_2O_4^+$ calculated 439.2591.

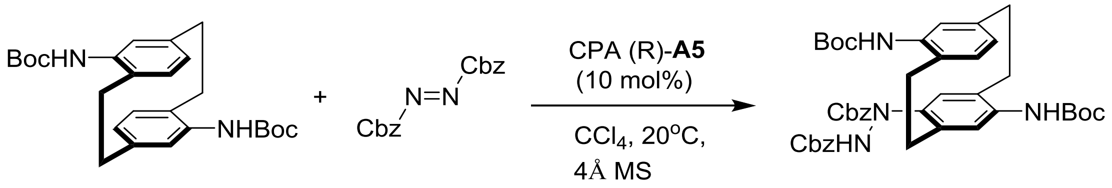

4a (achiral) **2a** (1.5 equiv.) **5a**, 40%, 99% ee

To a solution of racemic **4a** (88 mg, 0.2 mmol, 1.0 equiv.), (*R*)-**A5** (14 mg, 0.02 mmol, 0.1 equiv.) and activated 4 Å MS (500 mg) in dry CCl_4 (10 mL) was added a solution of **2a** (89 mg, 0.3 mmol, 1.5 equiv.) in dry CCl_4 (10 mL) at 20°C under N_2 atmosphere. After stirring at 20 °C under N_2 atmosphere for 40 h, the reaction mixture was quenched with Et_3N (20 μL) and concentrated under vacuum to give a residue, which was purified by column chromatography (petroleum ether/EtOAc = 3:1) to afford **4a** (20 mg, 23%) as a white solid and product **5a** (59 mg, 40%) as a white solid.

(S_p)-Dibenzyl-1-(1^5,4^2-bis((tert-butoxycarbonyl)amino)-1,4(1,4)-dibenzenacyclohexaphane-1^2-yl)hydrazine-1,2-dicarboxylate **(5a)**

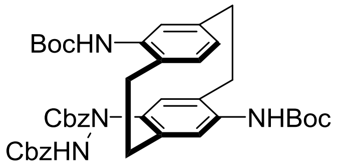

^{1}H NMR (400 MHz, $CDCl_3$) δ 7.51 – 7.27 (m, 10H), 7.05 – 5.72 (m, 7H), 5.50 – 4.70 (m, 5H), 3.85 – 2.27 (m, 8H), 1.65 – 1.39 (m, 18H). ^{13}C NMR (101 MHz, $CDCl_3$) δ 156.6, 152.5, 136.6, 135.8, 135.6, 135.4, 134.8, 134.1, 128.7, 128.6, 128.5, 128.4, 80.7, 69.0, 68.0, 32.5, 31.5, 28.5, 28.4. $[\alpha]^{25}_D$ = 73 (c = 0.5, $CHCl_3$). m/z HRMS (ESI) found $[M+H]^+$ 737.3552 $C_{42}H_{49}N_4O_8^+$ calculated 737.3545. HPLC: Chiralpak ID column, 80:20 hexanes/ethanol, 1 ml/min; t_R = 8.73 min (major); 99% ee.

1.5 Mechanism studies

Tert-butyl 1,4(1,4)-dibenzenacyclohexaphane-1^2-yl(methyl)carbamate **(6a)**

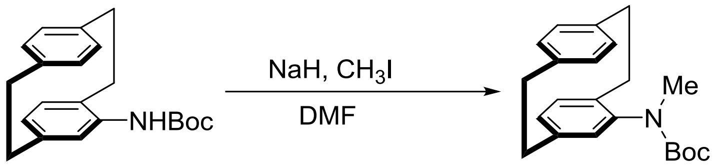

(±)-**1a** (±)-**6a**

To a solution of **1a** (64 mg, 0.2 mmol, 1.0 equiv.) in DMF (1 mL) was added NaH (10 mg, 0.24 mmol, 1.2 equiv. 60% suspension in mineral). After stirring at room temperature for 0.5 h, CH_3I (0.025 mL, 0.4 mmol, 2.0 equiv.) was added at rt, and the mixture was allowed to stir at room temperature for 1 h. After completion of the reaction as indicated by LC analysis, the reaction mixture was quenched with saturated NH_4Cl solution (5 mL), and extracted with EtOAc (3×5 mL). The combined organic layers were washed with brine (5 mL), dried over Na_2SO_4, filtered and concentrated under vacuum to give a residue, which was purified by column chromatography (petroleum ether/EtOAc = 20:1) to afford **6a** (66 mg, 99%) as a yellow solid. ^{1}H NMR (400 MHz, $CDCl_3$) δ 6.68 – 6.59 (m, 2H), 6.57 – 6.50 (m, 1H), 6.43 – 6.36 (m, 2H), 6.34 – 6.26 (m, 2H), 3.44 (s, 3H), 3.18 – 2.86 (m, 8H), 1.41 (s, 9H). ^{13}C NMR (126 MHz, $CDCl_3$) δ 154.8, 140.8, 140.1, 139.5, 139.3, 136.7, 135.0, 133.1, 132.6, 132.6, 131.8, 130.8, 125.1, 80.3, 36.8, 35.5, 35.4, 35.3, 32.8, 28.3. m/z HRMS (ESI) found $[M-56+H]^+$ 282.1480 $C_{18}H_{20}NO_2^+$ calculated 282.1489.

Kinetic resolution of **6a**

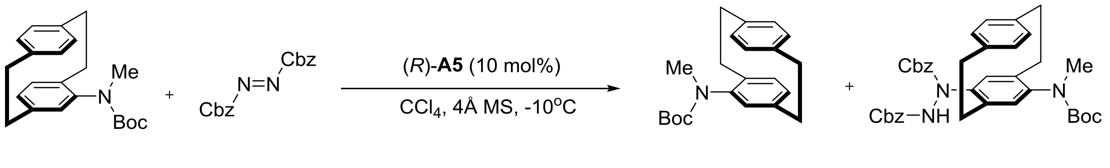

To a solution of racemic **6a** (16.9 mg, 0.05 mmol, 1.0 equiv.), (*R*)-**A5** (3.5 mg, 0.005 mmol, 0.1 equiv.) and activated 4 Å MS (50 mg) in CCl_4 (0.25 mL) was added a solution of **2a** (10.4 mg, 0.035 mmol, 0.7 equiv.) in dry CCl_4 (0.25 mL) at -10°C under N_2 atmosphere. The reaction mixture was stirred at -10 °C for 13 h. No reaction was observed by TLC analysis.

Tert-butyl (2,5-dimethylphenyl)carbamate **(6b)**

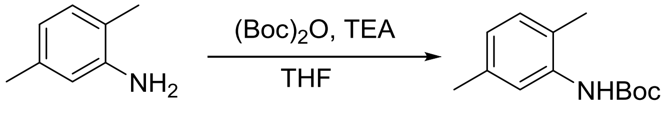

To a solution of 2,5-dimethylaniline (0.37 mL, 3 mmol, 1.0 equiv.), TEA (0.84 mL, 6 mmol, 2.0 equiv.) in THF (6 mL) was added $(Boc)_2O$ (0.83 mL, 3.6 mmol, 1.2 equiv.), and the mixture was stirred at room temperature overnight. After completion of the reaction as indicated by TLC analysis, the reaction mixture was concentrated under vacuum to give a residue, which was purified by column chromatography (petroleum ether/EtOAc = 30:1) to afford **6b** (650 mg, 98%) as a white solid. ^1H NMR (400 MHz, $CDCl_3$) δ 7.66 (s, 1H), 7.02 (d, J = 7.7 Hz, 1H), 6.84 – 6.77 (m, 1H), 6.23 (s, 1H), 2.31 (s, 3H), 2.20 (s, 3H), 1.52 (s, 9H). ^{13}C NMR (101 MHz, $CDCl_3$) δ 153.2, 136.7, 136.2, 130.2, 124.4, 124.0, 121.4, 80.5, 28.5, 21.4, 17.4. m/z HRMS (ESI) found $[M-56+H]^+$ 166.0859 $C_9H_{12}NO_2^+$ calculated 166.0863.

Electrophilic Aromatic Amination of **6b**:

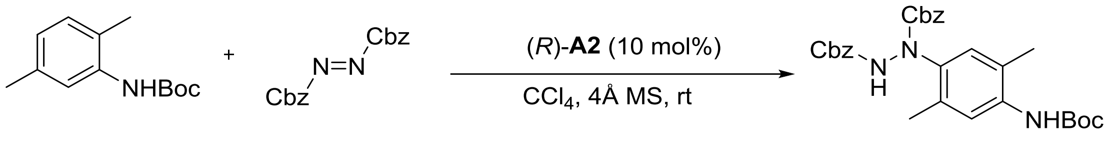

To a solution of racemic **6a** (11.1 mg, 0.05 mmol, 1.0 equiv.), (*R*)-**A5** (3.5 mg, 0.005 mmol, 0.1 equiv.) and activated 4 Å MS (50 mg) in CCl_4 (0.25 mL) was added a solution of **2a** (14.8 mg, 0.05 mmol, 1.0 equiv.) in dry CCl_4 (0.25 mL) at rt under N_2 atmosphere. The reaction mixture was stirred at rt for 13 h. No reaction was observed by TLC analysis.

N-(1,4(1,4)-dibenzenacyclohexaphane-1^2-yl)acetamide **(6c)**

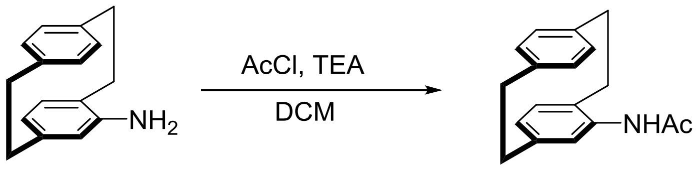

To a solution of **6g** (223 mg, 1 mmol, 1.0 equiv.), TEA (0.21 mL, 1.5 mmol, 1.5 equiv.) in DCM (10 mL) was added AcCl (0.09 mL, 1.2 mmol, 1.2 equiv.) at 0 °C, and the mixture was allowed to stir at room temperature for 0.5 h. After completion of the reaction as suggested by TLC analysis, the reaction mixture was quenched with saturated NH_4Cl solution, and extracted with DCM (3×10 mL). The combined organic layers were washed with brine (10 mL), dried over Na_2SO_4, filtered and concentrated under vacuum to give a residue, which was purified by column chromatography (petroleum ether/EtOAc = 1:2) to afford **6c** (250 mg, 94%) as a white solid. ^{1}H NMR (500 MHz, $CDCl_3$) δ 6.97 (s, 1H), 6.79 (s, 1H), 6.77 – 6.70 (m, 1H), 6.57 – 6.53 (m, 1H), 6.51 – 6.38 (m, 4H), 3.27 – 3.12 (m, 2H), 3.09 – 2.97 (m, 5H), 2.85 – 2.75 (m, 1H), 2.25 (s, 3H). ^{13}C NMR (101 MHz, $CDCl_3$) δ 167.9, 140.9, 139.4, 139.0, 136.4, 135.3, 133.2, 133.1, 132.2, 131.2, 129.4, 128.3, 127.0, 35.2, 35.0, 33.9, 33.2, 24.5. m/z HRMS (ESI) found $[M+H]^+$ 266.1532 $C_{18}H_{20}NO^+$ calculated 266.1539.

Kinetic resolution of **6c**

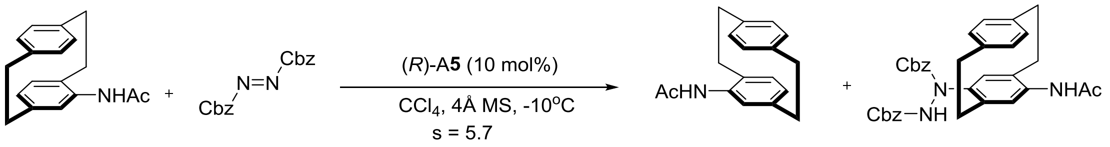

(±)-**6c** **2a** (R_p)-**6c** **7c**

To a solution of racemic **6c** (53 mg, 0.2 mmol, 1.0 equiv.), (*R*)-**A5** (14 mg, 0.02 mmol, 0.1 equiv.) and activated 4 Å MS (200 mg) in CCl_4 (1mL) was added a solution of **2a** (42 mg, 0.14 mmol, 0.7 equiv.) in dry CCl_4 (1mL) at -10 ^{o}C under N_2 atmosphere, After stirring at -10°C under N_2 atmosphere for 22 h, the reaction mixture was quenched with Et_3N (20 μL) and concentrated under vacuum to give a residue, which was purified by column chromatography (petroleum ether/EtOAc = 1:2 - 1:5) to afford the recovered product (R_p)-**6c** (33 mg, 62%) as a white solid, **7c** (42 mg, 37%) as a white solid.

(R_p)-*N*-(1,4(1,4)-dibenzenacyclohexaphane-1^2-yl)acetamide **(6c)**

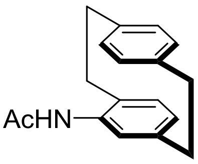

$[\alpha]^{25}_{D}$ = -54.6 (c = 0.5, $CHCl_3$). HPLC: Chiralpak IC column, 70:30 hexanes/isopropanol, 1 ml/min; t_R = 15.61 min (major), 20.47 min (minor); 33% ee.

Dibenzyl-1-(1^5-acetamido-1,4(1,4)-dibenzenacyclohexaphane-1^2-yl)hydrazine-1,2-dicarboxylate **(7c)**

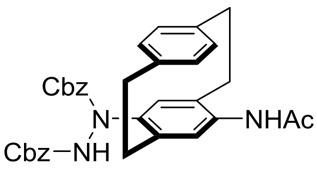

^{1}H NMR (400 MHz, $CDCl_3$) δ 7.39 – 7.26 (m, 9H), 7.00 – 6.39 (m, 7H), 6.19 (s, 1H), 5.40 – 4.90 (m, 5H), 3.25 – 3.00 (m, 4H), 2.93 – 2.64 (m, 4H), 2.24 (s, 3H). ^{13}C NMR (101 MHz, $CDCl_3$) δ 168.0, 157.1, 155.34, 139.4, 138.0, 136.4, 135.7, 133.0, 131.8, 131.5, 130.1, 128.7, 128.6, 128.3, 128.0, 68.7, 68.0, 34.8, 33.3, 32.5, 31.4, 24.7. $[\alpha]^{25}_{D}$

= 72.3 (c = 0.5, $CHCl_3$). m/z HRMS (ESI) found $[M+H]^+$ 564.2478 $C_{34}H_{34}N_3O_5^+$ calculated 564.2493. HPLC: Chiralpak ID column, 65:35 hexanes/isopropanol, 1 ml/min; t_R = 16.69 min (major), 22.25 min (minor); 61% ee.

N-(1,4(1,4)-dibenzenacyclohexaphane-1^2-yl)benzamide **(6d)**

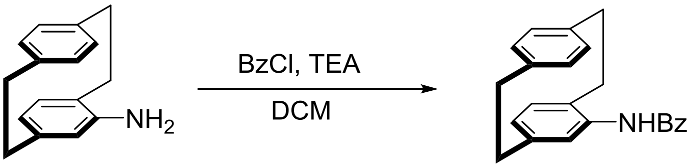

(±)-**6g** (±)-**6d**

To a solution of **6g** (223 mg, 1 mmol, 1.0 equiv.), TEA (0.21 mL, 1.5 mmol, 1.5 equiv.) in DCM (10 mL) was added BzCl (0.14 mL, 1.2 mmol, 1.2 equiv.) at 0 ^oC, and the mixture was allowed to stir at room temperature for 0.5 h. After completion of the reaction as indicated by TLC analysis, the reaction mixture was quenched with saturated NH_4Cl solution, and extracted with DCM (3×10 mL). The combined organic layers were washed with brine (10 mL), dried over Na_2SO_4, filtered and concentrated under vacuum to give a residue, which was purified by column chromatography (petroleum ether/EtOAc = 5:1) to afford **6d** (300 mg, 92%) as white solid. ^1H NMR (400 MHz, $CDCl_3$) δ 7.99 – 7.90 (m, 2H), 7.73 – 7.52 (m, 4H), 7.06 (s, 1H), 6.85 – 6.77 (m, 1H), 6.61 – 6.43 (m, 5H), 3.35 – 3.25 (m, 1H), 3.24 – 3.15 (m, 1H), 3.13 – 2.99 (m, 5H), 2.94 – 2.83 (m, 1H). ^{13}C NMR (101 MHz, $CDCl_3$) δ 164.9, 141.3, 139.6, 138.8, 136.7, 135.3, 135.3, 133.4, 133.3, 132.3, 132.0, 130.2, 129.3, 129.1, 128.3, 127.1, 126.7, 35.3, 35.1, 34.1, 33.3. m/z HRMS (ESI) found $[M+H]^+$ 328.1688 $C_{23}H_{22}NO^+$ calculated 328.1696.

Kinetic resolution of **6d**

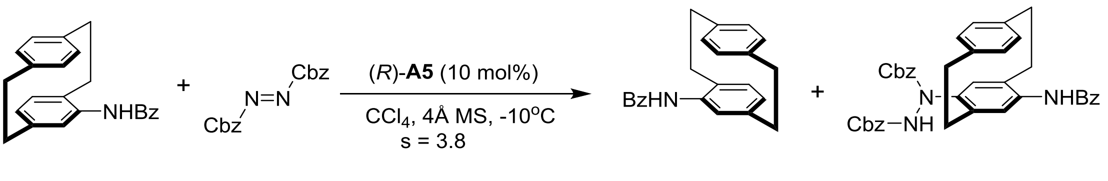

(±)-**6d** **2a** (R_p)-**6d** **7d**

To a solution of racemic **7a** (66 mg, 0.2 mmol, 1.0 equiv.), (*R*)-**A5** (14 mg, 0.02 mmol, 0.1 equiv.) and activated 4 Å MS (200 mg) in CCl_4 (1mL) was added a solution of **2a** (42 mg, 0.14 mmol, 0.7 equiv.) in dry CCl_4 (1mL) at -10°C under N_2 atmosphere. After stirring at -10°C under N_2 atmosphere for 22 h, the reaction mixture was quenched with Et_3N (20 μL) and concentrated under vacuum to give a residue, which was purified by column chromatography (petroleum ether/EtOAc = 5:1 - 1:2) to afford the recovered product (R_p)-**7a** (30 mg, 46%) as a white solid, **7d** (61 mg, 49%) as a white solid.

(R_p)-*N*-(1,4(1,4)-dibenzenacyclohexaphane-1^2-yl)benzamide **(6d)**

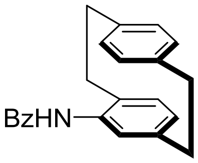

$[\alpha]^{25}_D$ = -43.1 (c = 0.5, $CHCl_3$). HPLC: Chiralpak IA column, 70:30 hexanes/isopropanol, 1 ml/min; t_R = 9.57 min (minor), 11.52 min (major); 46% ee.

Dibenzyl-1-(1^5-benzamido-1,4(1,4)-dibenzenacyclohexaphane-1^2-yl)hydrazine-1,2-di carboxylate **(7d)**

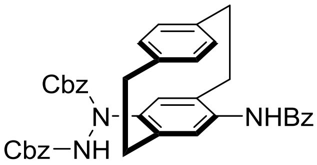

^{1}H NMR (400 MHz, $CDCl_3$) δ 7.98 – 7.87 (m, 2H), 7.72 – 7.51 (m, 4H), 7.45 – 7.26 (m, 9H), 7.22 – 6.79 (m, 3H), 6.67 – 6.47 (m, 3H), 6.21 (s, 1H), 5.44 – 4.91 (m, 5H), 3.36 – 2.65 (m, 8H). ^{13}C NMR (101 MHz, $CDCl_3$) δ 164.9, 157.1, 156.5, 139.6, 137.9, 135.6, 135.2, 133.2, 132.2, 131.9, 131.6, 129.8, 129.1, 128.7, 128.7, 128.6, 128.4, 128.1, 127.1, 68.8, 68.1, 34.9, 33.5, 32.7, 31.5. $[\alpha]^{25}_D$ = 46.1 (c = 0.5, $CHCl_3$). m/z HRMS (ESI) found $[M+H]^+$ 626.2679 $C_{39}H_{36}N_3O_5^+$ calculated 626.2649. HPLC: Chiralpak IA column, 60:40 hexanes/isopropanol, 1 ml/min; t_R = 12.17 min (major), 16.23 min (minor); 42% ee.

N-(1,4(1,4)-dibenzenacyclohexaphane-1²-yl)pivalamide **(6e)**

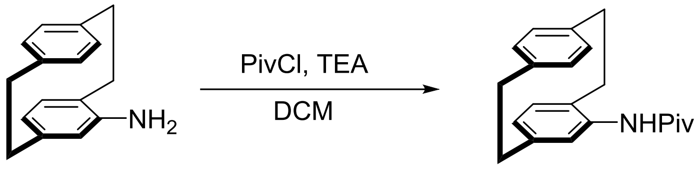

(±)-**6g** (±)-**6e**

To a solution of **6g** (223 mg, 1 mmol, 1.0 equiv.), TEA (0.21 mL, 1.5 mmol, 1.5 equiv.) in DCM (10 mL) was added PivCl (0.15 mL, 1.2 mmol, 1.2 equiv.) at 0 ^{o}C, and the mixture was allowed to stir at room temperature for 0.5 h. After completion of the reaction as indicated by TLC analysis, the reaction mixture was quenched with saturated NH_4Cl solution and extracted with DCM (3×10 mL). The combined organic layers were washed with brine (10 mL), dried over Na_2SO_4, filtered and concentrated under vacuum to give a residue, which was purified by column chromatography (petroleum ether/EtOAc = 8:1) to afford **6e** (300 mg, 98%) as a white solid. ^{1}H NMR (400 MHz, $CDCl_3$) δ 7.22 (s, 1H), 7.05 – 7.01 (m, 1H), 6.72 – 6.66 (m, 1H), 6.57 – 6.50 (m, 2H), 6.47 – 6.39 (m, 3H), 3.25 – 3.15 (m, 2H), 3.10 – 3.01 (m, 3H), 3.00 – 2.89 (m, 2H), 2.85 – 2.78 (m, 1H), 1.41 (s, 9H). ^{13}C NMR (101 MHz, $CDCl_3$) δ 175.7, 141.4, 139.7, 138.5, 137.0, 135.1, 133.3, 133.3, 132.3, 129.2, 128.6, 128.1, 126.3, 39.9, 35.4, 35.1, 33.9, 33.1, 27.9. m/z HRMS (ESI) found $[M+H]^+$ 308.2003 $C_{21}H_{26}NO^+$ calculated 308.2009.

Kinetic resolution of **6e**

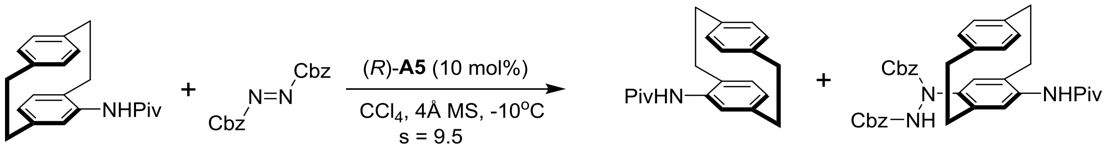

(±)-**6e** **2a** (R_p)-**6e** **7e**

To a solution of racemic **6e** (62 mg, 0.2 mmol, 1.0 equiv.), (*R*)-**A5** (14 mg, 0.02 mmol, 0.1 equiv.) and activated 4 Å MS (200 mg) in CCl_4 (1mL) was added a solution of **2a** (42 mg, 0.14 mmol, 0.7 equiv.) in dry CCl_4 (1mL) at -10°C under N_2 atmosphere. After stirring at -10°C under N_2 atmosphere for 12 h, the reaction mixture was quenched with

Et_3N (20 μL) and concentrated under vacuum to give a residue, which was purified by column chromatography (petroleum ether/EtOAc = 8:1 - 1:1) to afford the recovered product (R_p)-**6e** (25 mg, 41%) as a white solid, **7e** (72 mg, 59%) as a white solid.

(R_p)-*N*-(1,4(1,4)-dibenzenacyclohexaphane-1^2-yl)pivalamide **(6e)**

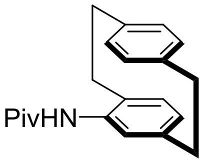

$[\alpha]^{25}_D$ = -165.4 (c = 0.5, $CHCl_3$). HPLC: Chiralpak IC column, 70:30 hexanes/isopropanol, 1 ml/min; t_R = 8.96 min (major), 11.22 min (minor); 80% ee.

Dibenzyl-1-(1^5-pivalamido-1,4(1,4)-dibenzenacyclohexaphane-1^2-yl)hydrazine-1,2-di carboxylate **(7e)**

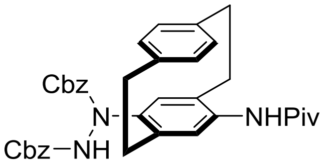

^1H NMR (400 MHz, $CDCl_3$) δ 7.50 – 7.29 (m, 9H), 7.26 – 6.95 (m, 3H), 6.80 – 6.65 (m, 1H), 6.66 – 6.39 (m, 3H), 6.17 (s, 1H), 5.57 – 4.59 (m, 5H), 3.27 – 2.49 (m, 8H), 1.39 (s, 9H). ^{13}C NMR (101 MHz, $CDCl_3$) δ 175.7, 157.1, 155.6, 139.6, 137.7, 137.4, 135.8, 135.6, 135.6, 133.1, 131.6, 128.6, 128.6, 128.5, 128.4, 128.3, 128.2, 128.0, 127.4, 68.6, 67.9, 39.8, 34.7, 33.3, 32.5, 31.3, 27.8. $[\alpha]^{25}_D$ = 85.0 (c = 0.5, $CHCl_3$). m/z HRMS (ESI) found $[M+H]^+$ 606.2946 $C_{37}H_{40}N_3O_5^+$ calculated 606.2962. HPLC: Chiralpak IA column, 60:40 hexanes/isopropanol, 1 ml/min; t_R = 8.22 min (major), 16.67 min (minor); 60% ee.

N-(1,4(1,4)-dibenzenacyclohexaphane-1^2-yl)-4-methylbenzenesulfonamide **(6f)**

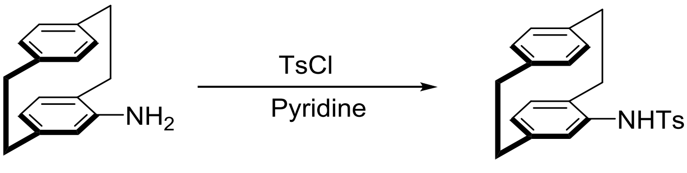

(±)-**6g** (±)-**6f**

To a solution of **6g** (112 mg, 0.5 mmol, 1.0 equiv.) in pyridine (2 mL) was added TsCl (114 mg, 0.6 mmol, 1.2 equiv.), and the mixture was allowed to stir at room temperature for 1 h. After completion of the reaction as indicated by TLC analysis, the reaction mixture was concentrated and extracted with EtOAc (3×10 mL). The combined organic layers were washed with brine (10 mL), dried over Na_2SO_4, filtered and concentrated under vacuum to give a residue, which was purified by column chromatography (petroleum ether/EtOAc = 1:1) to afford **6f** (170 mg, 90%) as a yellow solid. ^{1}H NMR (400 MHz, $CDCl_3$) δ 7.56 (d, J = 8.1 Hz, 2H), 7.17 (d, J = 8.0 Hz, 2H), 6.89 – 6.83 (m, 1H), 6.61 (s, 1H), 6.53 – 6.33 (m, 5H), 5.87 (s, 1H), 3.63 – 3.50 (m, 1H), 3.22 – 3.12 (m, 1H), 3.11 – 2.98 (m, 3H), 2.96 – 2.81 (m, 2H), 2.77 – 2.67 (m, 1H), 2.34 (s, 3H). ^{13}C NMR (101 MHz, $CDCl_3$) δ 143.7, 141.3, 139.7, 139.2, 136.4, 135.7, 134.8, 134.7, 133.5, 133.1, 132.2, 130.9, 130.1, 129.6, 128.8, 127.3, 35.2, 34.9, 34.1, 32.7, 21.7. m/z HRMS (ESI) found $[M+H]^+$ 378.1540 $C_{23}H_{24}NO_2S^+$ calculated 378.1522.

Kinetic resolution of **6f**

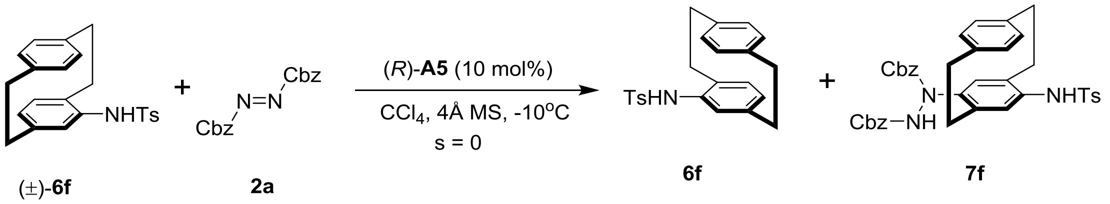

To a solution of racemic **6f** (76 mg, 0.2 mmol, 1.0 equiv.), (*R*)-**A5** (14 mg, 0.02 mmol, 0.1 equiv.) and activated 4 Å MS (200 mg) in CCl_4 (1mL) was added a solution of **2a** (42 mg, 0.14 mmol, 0.7 equiv.) in dry CCl_4 (1mL) at -10°C under N_2 atmosphere. After stirring at -10°C under N_2 atmosphere for 24 h, the reaction mixture was quenched with Et_3N (20 μL) and concentrated under vacuum to give a residue, which was purified by column chromatography (petroleum ether/EtOAc = 2:1) to afford the recovered product **6f** (48 mg, 64%) as yellow solid, **7f** (47 mg, 35%) as yellow solid.

N-(1,4(1,4)-dibenzenacyclohexaphane-1^2-yl)-4-methylbenzenesulfonamide **(6f)**

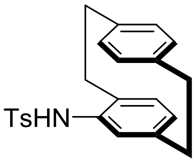

$[\alpha]^{25}_{D}$ = 0 (c = 0.5, $CHCl_3$). HPLC: Chiralpak IC column, 70:30 hexanes/isopropanol, 1 ml/min; t_R = 8.83 min, 20.66 min; 0% ee.

Dibenzyl-1-(1^5-((4-methylphenyl)sulfonamido)-1,4(1,4)-dibenzenacyclohexaphane-1^2-yl)hydrazine-1,2-dicarboxylate **(7f)**

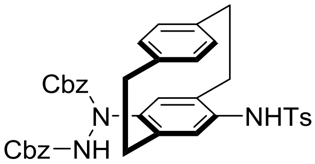

^{1}H NMR (400 MHz, $CDCl_3$) δ 7.88 – 7.23 (m, 10H), 7.16 – 6.19 (m, 9H), 5.83 – 5.42 (m, 1H), 5.24 – 4.74 (m, 6H), 3.39 – 1.70 (m, 11H). ^{13}C NMR (126 MHz, $CDCl_3$) δ 166.3, 156.5, 155.5, 143.6, 141.1, 139.7, 139.5, 139.1, 138.7, 137.7, 135.6, 134.9, 134.7, 133.4, 133.1, 132.2, 132.1, 131.5, 130.9, 130.6, 129.9, 129.5, 129.4, 128.7, 128.6, 128.5, 128.3, 127.7, 127.2, 127.0, 68.7, 67.9, 54.8, 34.4, 34.1, 33.1, 31.7, 21.7. $[\alpha]^{25}_{D}$ = 0 (c = 0.5, $CHCl_3$). m/z HRMS (ESI) found $[M+H]^+$ 676.2485 $C_{39}H_{38}N_3O_6S^+$ calculated 676.2476. HPLC: Chiralpak IC column, 70:30 hexanes/isopropanol, 1 ml/min; t_R = 20.13 min, 25.50 min; 0% ee.

1,4(1,4)-dibenzenacyclohexaphan-1^2-amine **(6g)**

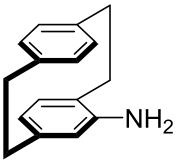

(±)-**6g**

The synthesis of **6g** refers to the general procedure of synthesis of **1e**

^{1}H NMR (500 MHz, $CDCl_3$) δ 7.19 (d, *J* = 7.9, 1H), 6.61 (d, *J* = 7.8 Hz, 1H), 6.41 (d, *J* = 7.7 Hz, 2H), 6.29 (d, *J* = 7.6 Hz, 1H), 6.15 (d, *J* = 7.7 Hz, 1H), 5.39 (s, 1H), 3.44 (s, 2H), 3.18 – 3.09 (m, 3H), 3.06 – 2.94 (m, 3H), 2.90-2.80 (m, 1H), 2.73 – 2.63 (m, 1H). ^{13}C NMR (126 MHz, $CDCl_3$) δ 145.1, 141.1, 139.1, 139.0, 135.4, 133.6, 132.5,

131.6, 126.9, 124.6, 122.9, 122.4, 35.5, 35.1, 33.1, 32.3. m/z HRMS (ESI) found $[M+H]^+$ 224.1428 $C_{16}H_{18}N^+$ calculated 224.1434.

Kinetic resolution of **6g** in standard condition

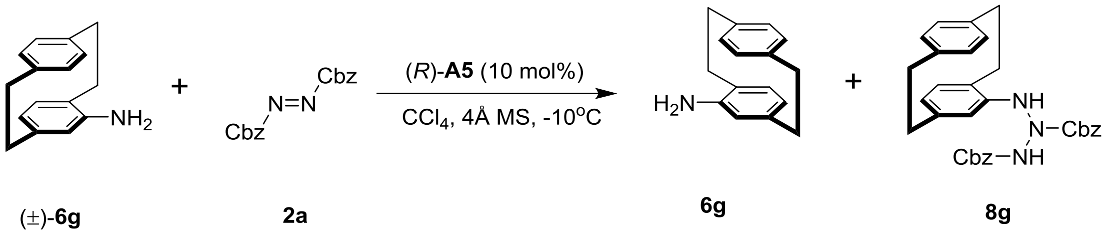

To a solution of racemic **6g** (67 mg, 0.3 mmol, 1.0 equiv.), (*R*)-**A5** (21 mg, 0.03 mmol, 0.1 equiv.) and activated 4 Å MS (300 mg) in CCl_4 (1.5 mL) was added a solution of **2** (63 mg, 0.21 mmol, 0.7 equiv.) in dry CCl_4 (1.5 mL) at -10°C under N_2 atmosphere. After stirring at -10°C under N_2 atmosphere for 20 min, the reaction mixture was quenched with Et_3N (20 μL) and concentrated under vacuum to give a residue, which was purified by column chromatography (petroleum ether/EtOAc = 5:1-1:5) to afford the recovered product **6g** (25 mg, 37%) as a yellow solid and the crude product **8g**. The crude product **8g** was purified by preparative thin-Layer chromatography (DCM/MeOH = 30:1) to afford **8g** (62 mg, 40%) as a yellow solid.

1,4(1,4)-dibenzenacyclohexaphan-1^2-amine **(6g)**

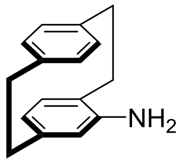

HPLC: Chiralpak IB column, 60:40 hexanes/isopropanol, 1 ml/min; t_R = 9.28 min, 13.84 min; 0% ee.

Dibenzyl 3-(1,4(1,4)-dibenzenacyclohexaphane-1^2-yl)triazane-1,2-dicarboxylate **(8g)**

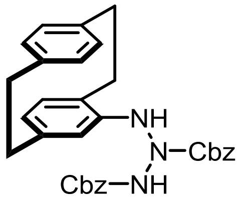

^{1}H NMR (400 MHz, $CDCl_3$) δ 7.60 – 7.26 (m, 7H), 7.25 – 6.36 (m, 7H), 6.29 – 4.61 (m, 9H), 3.29 – 2.57 (m, 6H), 2.40 – 2.11 (m, 2H). ^{13}C NMR (126 MHz, $CDCl_3$) δ 167.9, 156.5, 155.7, 138.9, 137.8, 135.9, 133.0, 132.5, 131.7, 131.6, 130.3, 128.6, 128.5, 128.3, 128.1, 68.4, 67.3, 55.2, 34.1, 33.4, 31.5, 31.2. m/z HRMS (ESI) found $[M+H]^+$ 522.2390 $C_{32}H_{32}N_3O_4^+$ calculated 522.2387. HPLC: Chiralpak IB column, 80:20 hexanes/ethanol, 1 ml/min; t_R = 9.33 min, 12.66 min; 0% ee.

Determination of the structure of **8g**

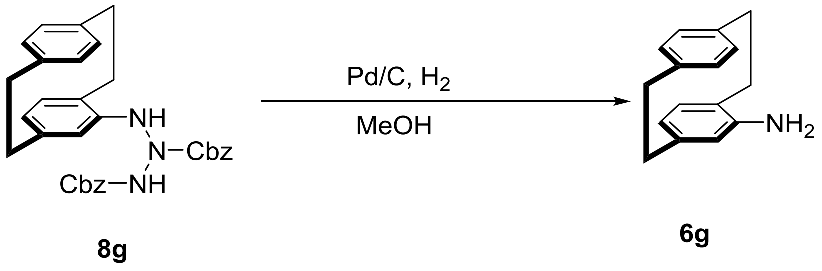

To a solution of **8g** (52 mg, 1 mmol, 1.0 equiv.) in MeOH (3 mL) were added Pd/C (10% wt/wt, 100 mg). The mixture was stirred at rt under H_2 atmosphere overnight. After completion of the reaction as indicated by TLC analysis, the reaction mixture was filtered, and the filtrate was concentrated under vacuum to give a residue, which was purified by column chromatography (petroleum ether/EtOAc = 2:1) to afford **6g** (18 mg, 82%) as a yellow solid. ^{1}H NMR (500 MHz, $CDCl_3$) δ 7.19 (d, J = 7.9, 1H), 6.61 (d, J = 7.8 Hz, 1H), 6.41 (d, J = 7.7 Hz, 2H), 6.29 (d, J = 7.6 Hz, 1H), 6.15 (d, J = 7.7 Hz, 1H), 5.39 (s, 1H), 3.44 (s, 2H), 3.18 – 3.09 (m, 3H), 3.06 – 2.94 (m, 3H), 2.90-2.80 (m, 1H), 2.73 – 2.63 (m, 1H). ^{13}C NMR (126 MHz, $CDCl_3$) δ 145.1, 141.1, 139.1, 139.0, 135.4, 133.6, 132.5, 131.6, 126.9, 124.6, 122.9, 122.4, 35.5, 35.1, 33.1, 32.3. m/z HRMS (ESI) found $[M+H]^+$ 224.1428 $C_{16}H_{18}N^+$ calculated 224.143

Kinetic resolution of **6g** at higher temperature

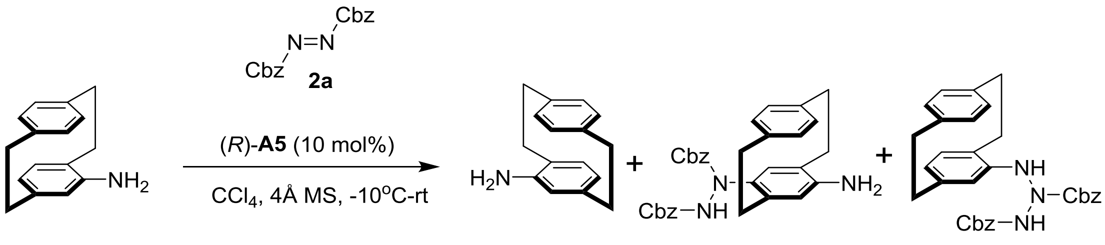

(±)-**6g** **6g** **7g** **8g**

To a solution of racemic **6g** (67 mg, 0.3 mmol, 1.0 equiv.), (*R*)-**A5** (21 mg, 0.03 mmol, 0.1 equiv.) and activated 4 Å MS (300 mg) in CCl_4 (1.5 mL) was added a solution of **2a** (63 mg, 0.21 mmol, 0.7 equiv.) in dry CCl_4 (1.5 mL) at -10°C under N_2 atmosphere. After stirring at -10 °C under N_2 atmosphere for 24 h, the resulting mixture was warmed to rt and allowed to stir at rt for 24 h. The reaction mixture was quenched with Et_3N (20 μL) and concentrated under vacuum to give a residue, which was purified by column chromatography (petroleum ether/EtOAc = 5:1-1:1-1:5) to afford the recovered product **6g** (24 mg, 36%) as a yellow solid, **7g** (16 mg, 10%) as a yellow solid and the crude product **8g**. The crude product **8g** was purified by preparative thin-layer chromatography (DCM/MeOH = 30:1) to afford **8g** (54 mg, 35%) as yellow solid.

1,4(1,4)-dibenzenacyclohexaphan-1^2-amine **(6g)**

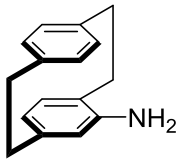

HPLC: Chiralpak IB column, 60:40 hexanes/isopropanol, 1 ml/min; t_R = 9.24 min, 13.86 min; 0% ee.

Dibenzyl-1-(1^5-amino-1,4(1,4)-dibenzenacyclohexaphane-12-yl)hydrazine-1,2-dicarboxylate **(7g)**

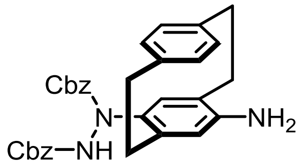

^{1}H NMR (500 MHz, $CDCl_3$) δ 7.54 – 7.26 (m, 9H), 7.26 – 7.10 (m, 3H), 6.68 – 6.05 (m, 4H), 5.42 – 4.94 (m, 5H), 3.70 – 3.30 (m, 2H), 3.24 – 2.28 (m, 8H). ^{13}C NMR (126 MHz, $CDCl_3$) δ 157.0, 144.8, 138.3, 136.0, 135.8, 133.4, 131.5, 131.1, 130.7, 128.8, 128.6, 128.5, 128.4, 128.2, 127.9, 127.2, 124.3, 123.0, 68.5, 68.1, 35.1, 32.6, 31.8, 31.2. m/z HRMS (ESI) found $[M+H]^+$ 522.2390 $C_{32}H_{32}N_3O_4^+$ calculated 522.2387. HPLC: Chiralpak IB column, 60:40 hexanes/ethanol, 1 ml/min; t_R = 8.10 min, 20.71 min; 47% ee

Dibenzyl 3-(1,4(1,4)-dibenzenacyclohexaphane-1^2-yl)triazane-1,2-dicarboxylate **(8g)**

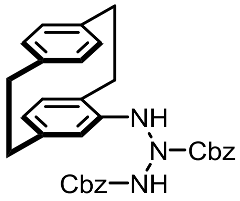

m/z HRMS (ESI) found $[M+H]^+$ 522.2390 $C_{32}H_{32}N_3O_4^+$ calculated 522.2387. HPLC: Chiralpak IB column, 80:20 hexanes/ethanol, 1 ml/min; t_R = 8.93 min, 12.16 min; 14% ee.

Kinetic resolution of **1x** under standard conditions

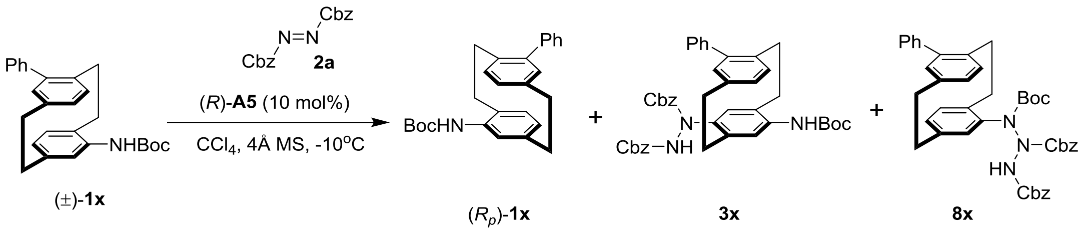

To a solution of racemic **1x** (120 mg, 0.3 mmol, 1.0 equiv.), (*R*)-**A5** (21 mg, 0.03 mmol, 0.1 equiv.) and activated 4 Å MS (300 mg) in dry CCl_4 (1.5 mL) was added a solution of **2a** (63 mg, 0.21 mmol, 0.7 equiv.) in dry CCl_4 (1.5 mL) at -10 °C under N_2 atmosphere. After stirring for 33 h, the reaction mixture was quenched with Et_3N (20 μL) and concentrated under vacuum to give a residue, which was purified by column chromatography (petroleum ether/EtOAc = 8:1-2:1) to afford (R_p)-**1x** (62 mg, 52%) as a white solid and the mixture of **3x+8x** (101 mg, 48%) as a white solid. HPLC: (R_p)-**1x**, Chiralpak IC column, 95:05 hexanes/isopropanol, 1 ml/min; t_R = 6.26 min,

8.89 min; 92% ee. **3x+8x**, Chiralpak IA column, 80:20 hexanes/isopropanol, 1 ml/min; t_R = 7.24 min1(**8x** minor), 8.72 min (**8x** major), 95% ee, Chiralpak IA column, 80:20 hexanes/isopropanol, 1 ml/min; 18.41 min (**3x** major), 23.95 min (**3x** minor); 94% ee. **8x**:**3x** = 19:81.

3x+8x (101 mg) was further purified by column chromatography (DCM/EtOAc = 20:1) to afford **8x** (17 mg, 17%) as a white solid and **3x** (78 mg, 78%) as a white solid.

2,3-dibenzyl 1-(tert-butyl) 1-(4^2-phenyl-1,4(1,4)-dibenzenacyclohexaphane -12-yl)triazane-1,2,3-tricarboxylate **(8x)**

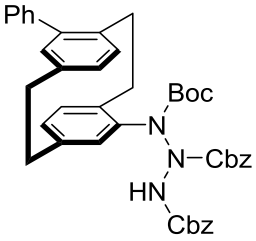

^{1}H NMR (400 MHz, $CDCl_3$) δ 7.63 – 7.24 (m, 13H), 7.19 – 6.44 (m, 5H), 6.32 – 5.95 (m, 1H), 5.90 – 5.60 (m, 2H), 5.40 – 4.74 (m, 5H), 3.68 – 2.12 (m, 8H), 1.57 (s, 9H). ^{13}C NMR (126 MHz, $CDCl_3$) δ 162.6, 159.4, 156.5, 155.6, 142.9, 140.4, 137.5, 135.7, 133.8, 132.6, 131.8, 130.2, 129.0, 128.6, 128.4, 128.2, 127.9, 127.4, 81.9, 68.7, 67.7, 33.9, 32.8, 30.2, 28.4. m/z HRMS (ESI) found $[M+H]^+$ 698.3214 $C_{43}H_{44}N_3O_6^+$ calculated 698.3225.

Determine the structure of **3x** and **8x**

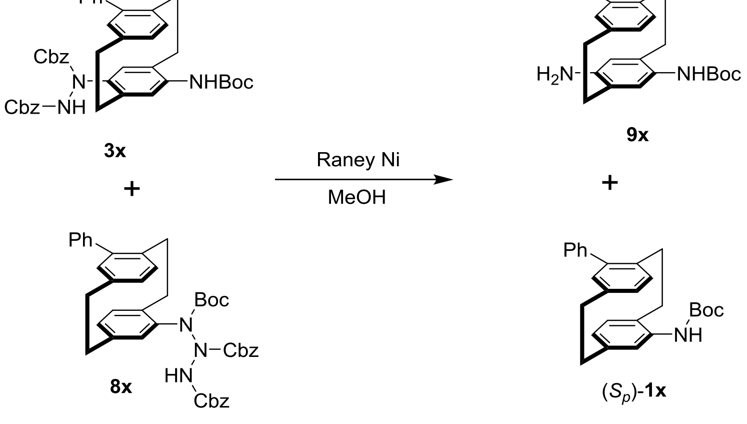

To a solution of the mixture of **3x+8x** (52 mg, 0.75 mmol, 1.0 equiv.) in MeOH (3 mL) were added Raney Ni (100 mg). The mixture was stirred at rt under H_2 atmosphere overnight. After completion of the reaction as indicated by TLC analysis, the reaction mixture was filtered, and the filtrate was concentrated under vacuum to give a residue. The crude ^{1}H NMR of this residue showed **8x**:**3x** = 1:4, which is consist with HPLC analysis result. The residue was purified by column chromatography (petroleum ether/EtOAc = 8:1 - 2:1) to afford **9x** (21 mg, 84%) as a white solid, (S_p)-**1x** (5 mg, 88%) as a white solid. crude ^{1}H NMR (500 MHz, $CDCl_3$) δ 7.59 – 7.41 (m, 5H), 7.38 – 7.32 (m, 1.25H), 7.16 (s, 1H), 6.97 – 6.85 (m, 1.25H), 6.73 – 6.35 (m, 3H), 6.10 (s, 0.25H), 6.10 (s, 1H), 5.67 (s, 1H), 3.55 – 3.40 (m, 3.25H), 3.31 – 2.73 (m, 7.5H), 2.72 – 2.61 (m, 1H), 2.20 – 2.30 (m, 0.25H), 2.09 – 2.01 (m, 1H), 1.57 (s, 2.25H), 1.53 (s, 9H).

(S_p)-Tert-butyl (4^2-phenyl-1,4(1,4)-dibenzenacyclohexaphane-12-yl)carbamate (**1x**)

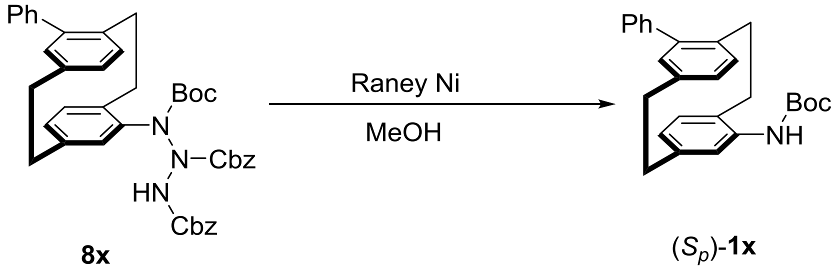

To a solution of **8x** (35 mg, 0.05 mmol, 1.0 equiv.) in MeOH (3 mL) were added Raney Ni (100 mg). The mixture was stirred at rt under H_2 atmosphere overnight. After completion of the reaction as indicated by TLC analysis, the reaction mixture was filtered, and the filtrate was concentrated to give a residue, which was purified by column chromatography (petroleum ether/EtOAc = 8:1) to afford (S_p)-**1x** (15 mg, 75%) as a white solid. ^{1}H NMR (400 MHz, $CDCl_3$) δ 7.62 – 7.29 (m, 10H), 7.25 – 6.88 (m, 6H), 6.81 – 6.14 (m, 5H), 5.53 – 4.83 (m, 5H), 3.52 – 3.40 (m, 1H), 3.25 – 2.55 (m, 6H), 2.25 – 2.07 (m, 1H), 1.58 (s, 9H). ^{13}C NMR (101 MHz, $CDCl_3$) δ 157.5, 152.7, 141.3, 141.0, 137.6, 136.2, 135.6, 135.3, 133.4, 131.3, 129.2, 128.7, 128.6,

128.5, 128.5, 128.2, 127.9, 126.8, 125.6, 80.8, 68.8, 68.5, 34.2, 33.1, 32.7, 31.7, 28.5. m/z HRMS (ESI) found $[M-56]^+$ 344.1640 $C_{23}H_{22}NO_2^+$ calculated 344.1645.

Tert-butyl (1^5-amino-4^2-phenyl-1,4(1,4)-dibenzenacyclohexaphane-1^2-yl)carbamate **(9x)**

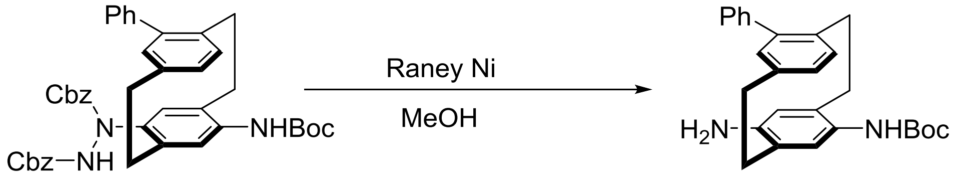

3x **9x**

To a solution of **3x** (35 mg, 0.05 mmol, 1.0 equiv.) in MeOH (3 mL) were added Raney Ni (100 mg), and the mixture was allowed to stir at rt under H_2 atmosphere overnight. After completion of the reaction as indicated by TLC analysis, the reaction mixture was filtered and the filtrate was concentrated under vacuum to give a residue, which was purified by column chromatography (petroleum ether/EtOAc = 8:1) to afford **9x** (15 mg, 72%) as a white solid. ^{1}H NMR (400 MHz, $CDCl_3$) δ 7.48 – 7.42 (m, 2H), 7.40 – 7.34 (m, 2H), 7.30 – 7.23 (m, 1H), 7.09 (d, J = 1.9 Hz, 1H), 6.83 (d, J = 7.7 Hz, 1H), 6.51 (s, 1H), 6.45 – 6.37 (m, 1H), 6.04 (s, 1H), 5.61 (s, 1H), 3.48 – 3.36 (m, 3H), 3.09 – 2.97 (m, 3H), 2.86 – 2.73 (m, 2H), 2.67 – 2.53 (m, 1H), 2.05 – 1.92 (m, 1H), 1.46 (s, 9H). ^{13}C NMR (101 MHz, $CDCl_3$) δ 141.6, 141.4, 141.3, 139.2, 136.4, 132.0, 129.5, 128.9, 128.7, 126.9, 125.1, 119.3, 80.0, 32.9, 32.1, 31.9, 31.3, 28.6. m/z HRMS (ESI) found $[M+H]^+$ 415.2366 $C_{27}H_{31}N_2O_2^+$ calculated 415.2380.

Kinetic resolution of **1x** with the standard conditions

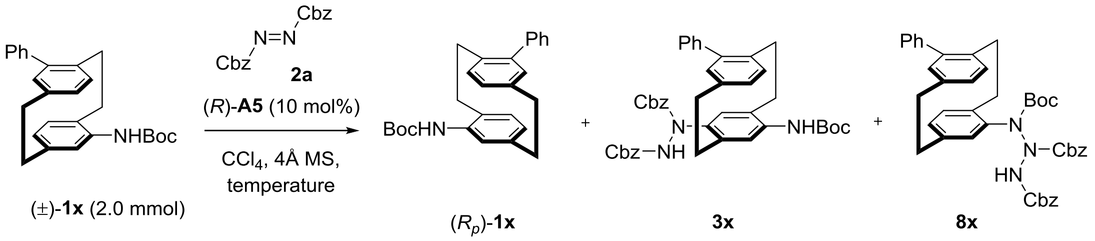

(±)-**1x** (2.0 mmol) (*R*$_p$)-**1x** **3x** **8x**

monitoring the ee value at different reaction time

at -10 °C	(*R*$_p$)-1x	3x	8x	
2 h,	25% ee	~99% ee	~99% ee	**8x**:**3x** = 87:13
5 h,	47% ee	~99% ee	~99% ee	**8x**:**3x** = 75:25
8 h,	62% ee	~99% ee	~99% ee	**8x**:**3x** = 71:29
11 h,	72% ee	~99% ee	~99% ee	**8x**:**3x** = 61:39
25 h,	93% ee	~99% ee	~99% ee	**8x**:**3x** = 35:65
**warmed to 0°C**				
10 h,	96% ee	~99% ee	~99% ee	**8x**:**3x** = 13:87
24 h,	98% ee	~99% ee	~99% ee	**8x**:**3x** = 3:97

Supplementary Table 1. Monitoring the yields of each components.

Time (h)	**1x** （%）	**3x** （%）	**8x** （%）
2	79.8	2.6	17.6
5	67.8	8.1	24.1
8	61.5	11.1	27.3
11	57.9	16.4	25.7
25	51.6	31.4	16.9
35	50.8	42.6	6.6
49	50.3	49.6	0.1

Reaction with no CPA catalyst

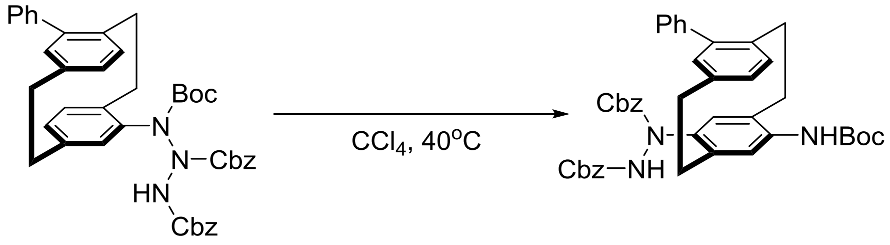

(±)-**8x** (±)-**3x**

24 h, NR

A solution of **8x** (0.1 mmol) in CCl_4 (1.0 mL) was warmed to 40 °C. After stirring at this temperature for 24 h, no reaction was observed by TLC and HPLC analysis.

Kinetic resolution of **8x**

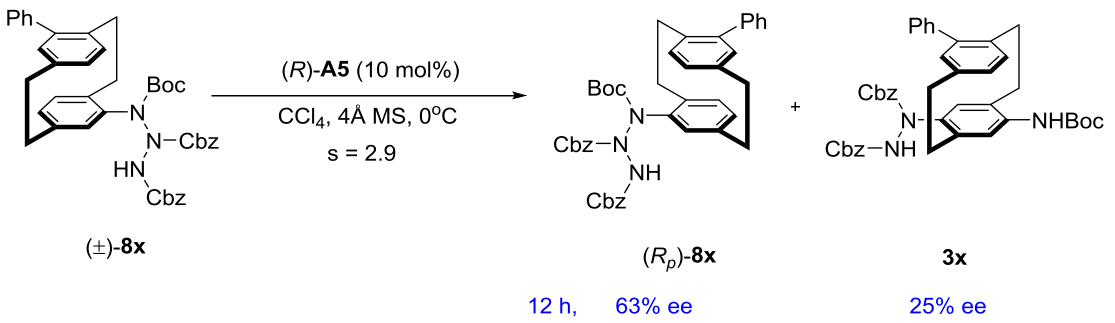

8x (14 mg, 0.02 mmol, 1.0 equiv.), (*R*)-**A5** (1.4 mg, 0.002 mmol, 0.1 equiv.) and activated 4 Å MS (40 mg) were added to a Schlenk tube. Then, dry CCl_4 (0.2 mL) was added to the reaction mixture, which was allowed to stir at 0 ^{o}C under N_2 atmosphere for 12 h.

HPLC: (*R$_p$*)-**8x**, Chiralpak IA column, 80:20 hexanes/isopropanol, 1 ml/min; t_R = 7.37 min (major), 8.97 min (minor); 63% ee. **3x**, Chiralpak IA column, 80:20 hexanes/isopropanol, 1 ml/min; t_R = 19.60 min (major), 20.4 min (minor), 25% ee.

Reaction between **8x** and **1a** catalyzed by (*R)*-**A5** in standard condition

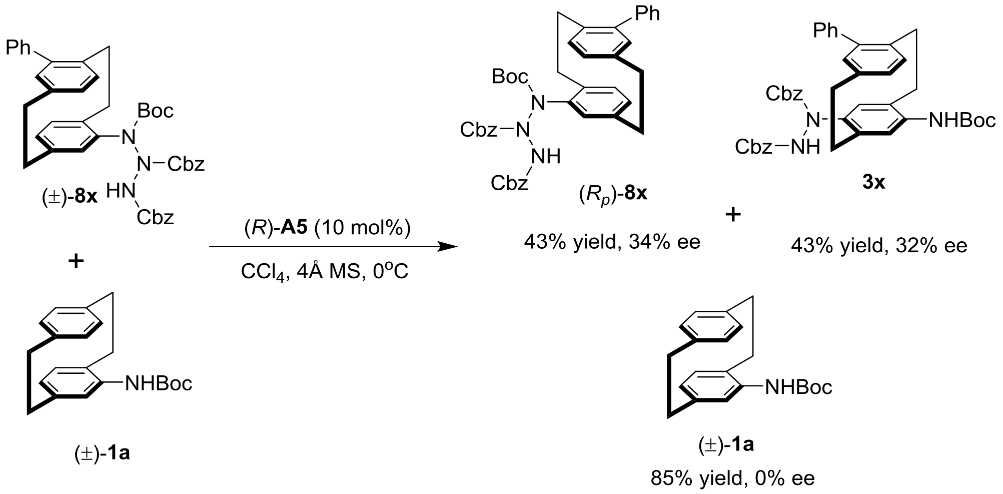

8x (28 mg, 0.04 mmol, 1.0 equiv.), **1a** (13 mg, 0.04 mmol, 1.0 equiv.), (*R*)-**A5** (2.8 mg, 0.004 mmol, 0.1 equiv.) and activated 4 Å MS (40 mg) were added to a Schlenk tube. Then, dry CCl_4 (0.4 mL) was added to the reaction mixture, which was allowed to stir at 0 ^{o}C under N_2 atmosphere for 12 h. Then the reaction mixture was quenched with Et_3N (20 μL) and concentrated under vacuum to give a residue, which was purified by preparative thin-layer chromatography (DCM/EtOAc = 30:1) to afford (*R$_p$*)-**8x** (12 mg, 43%) as a white solid, **3x** (12 mg, 43%) as a white solid and **1a** (11

mg, 85%) as a white solid. HPLC: (R_p)-**8x**, Chiralpak IA column, 80:20 hexanes/isopropanol, 1 ml/min; t_R = 7.18 min (major), 8.71 min (minor); 34% ee. **3x**, Chiralpak IA column, 80:20 hexanes/isopropanol, 1 ml/min; t_R = 18.65 min (major), 25.40 min (minor), 32% ee, **1a,** Chiralpak IC column, 70:30 hexanes/isopropanol, 1 ml/min; t_R = 5.01 min, 6.75 min. 0% ee.

Kinetic resolution of (S_p)-**8x** catalyzed by racemic CPA

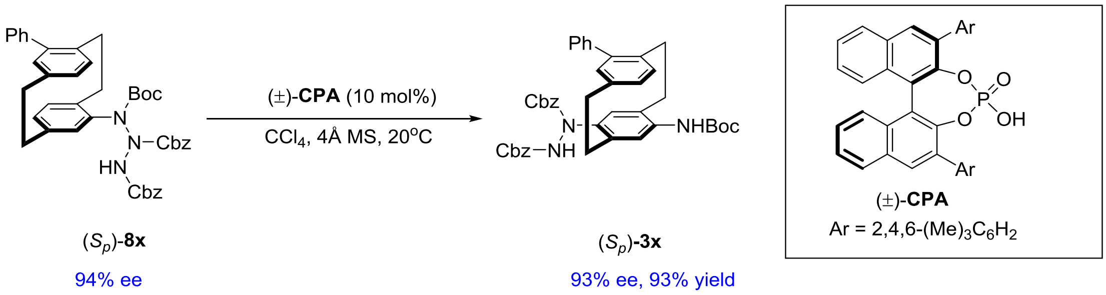

(S_p)-**8x** (28 mg, 0.04 mmol, 1.0 equiv.), racemic **CPA** (2.3 mg, 0.004 mmol, 0.1 equiv.) and activated 4 Å MS (40 mg) were added to a Schlenk tube. Then, dry CCl_4 (0.4 mL) was added to the reaction mixture, which was allowed to stir at 20 ^oC under N_2 atmosphere for 132 h. Then the reaction mixture was quenched with Et_3N (20 μL) and concentrated under vacuum to give a residue, which was purified by column chromatography (PE/EtOAc = 2:1) to afford (S_p)-**3x** (26 mg, 93%) as a white solid, HPLC: Chiralpak IA column, 80:20 hexanes/isopropanol, 1 ml/min; t_R = 18.81 min (major), 25.36 min (minor); 93% ee.

Monitoring the inetic resolution of **1ac** in toluene at -20°C by chiral HPLC analysis

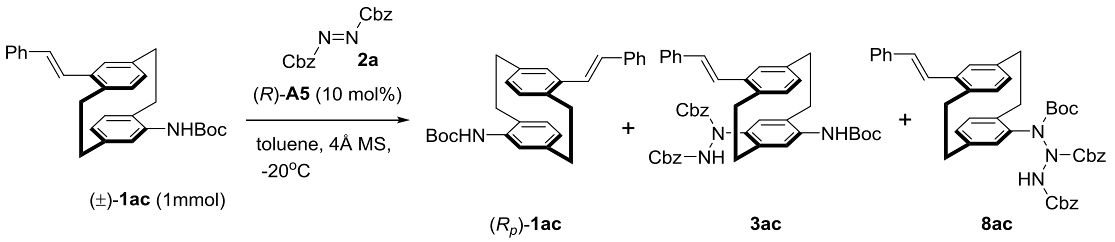

monitoring the ee value at different reaction time

	(R_p)-**1ac**	**3ac**	**8ac**	
5 h,	20% ee	~99% ee	~99% ee	**8ac**:**3ac** = 85:15
11 h,	29% ee	~99% ee	~99% ee	**8ac**:**3ac** = 66:34
23 h,	55% ee	~99% ee	~99% ee	**8ac**:**3ac** = 41:59
35 h,	67% ee	~99% ee	~99% ee	**8ac**:**3ac** = 27:73
47 h,	76% ee	~99% ee	~99% ee	**8ac**:**3ac** = 17:83
59 h,	84% ee	~99% ee	~99% ee	**8ac**:**3ac** = 12:88

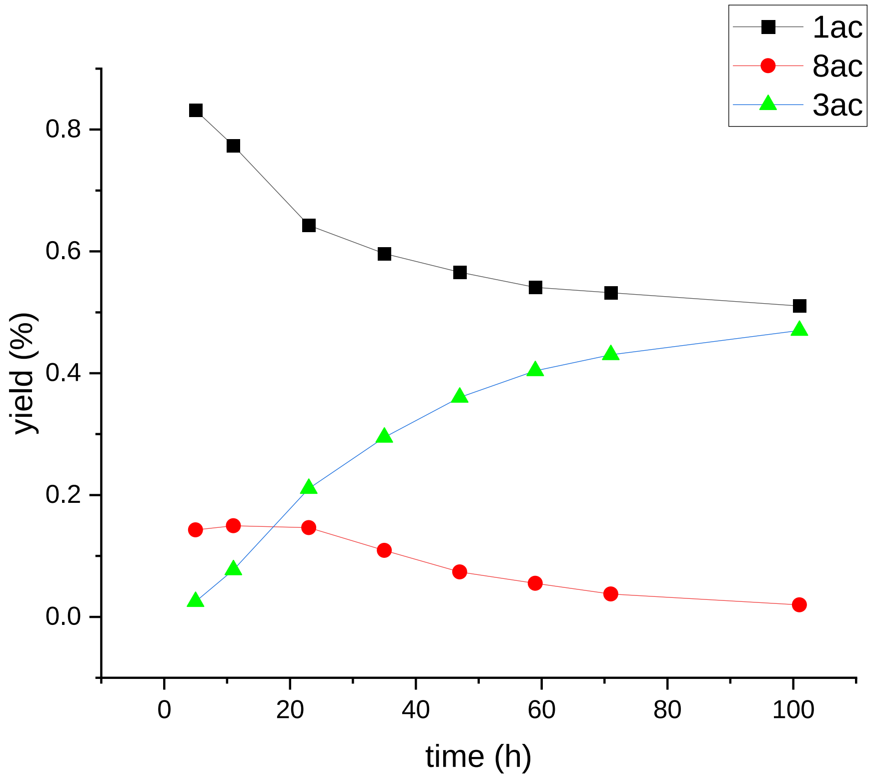

Supplementary Fig. 2. Monitoring the reaction of racemic amido-PCP **1ac** at −20 °C.

1.6 Large scale kinetic resolution of 1a

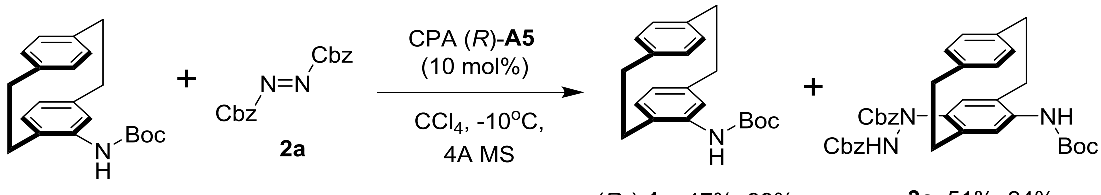

To a solution of racemic **1a** (646 mg, 2.0 mmol, 1.0 equiv.), (*R*)-**A5** (140 mg, 0.2 mmol, 0.1 equiv.) and activated 4 Å MS (2.0 g) in dry CCl_4 (10 mL) was added a solution of **2a** (418 mg, 1.4 mmol, 0.7 equiv.) in dry CCl_4 (10 mL) at -10°C under N_2 atmosphere. After stirring at -10°C under N_2 atmosphere for 13 h, the reaction mixture was quenched with Et_3N (20 μL) and concentrated under vacuum to give a residue, which was purified by column chromatography (petroleum ether/EtOAc = 8:1-2:1) to afford the recovered product (R_p)-**1a** (303 mg, 47%, 99% ee) as a white solid and product **3a** (630 mg, 51%, 94% ee) as a white solid.

1.7 Derivatizations of the chiral products

(S_p)-Tert-butyl 1,4(1,4)-dibenzenacyclohexaphane-12-ylcarbamate **(1a)**

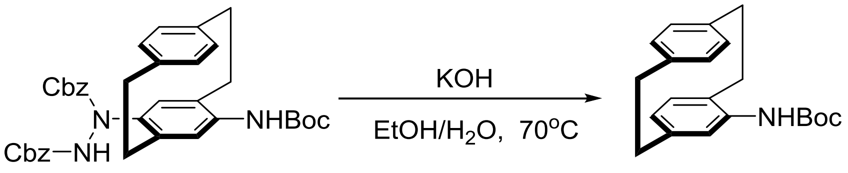

3a, 94% ee (S_p)-**1a**, 94% yield, 94% ee

To a solution of **3a** (62 mg, 0.1 mmol, 1.0 equiv.) in EtOH/H_2O (4 mL/0.1 mL) was added KOH (56 mg, 1 mmol, 10.0 equiv.). After stirring at 70°C overnight, the reaction mixture was concentrated under vacuum to give a residue, which was purified by column chromatography (petroleum ether/EtOAc = 8:1) to afford (S_p)-**1a** (32 mg, 94%) as a white solid. ^{1}H NMR (400 MHz, $CDCl_3$) δ 6.80 – 6.77 (m, 1H), 6.72 (s, 1H), 6.55 – 6.49 (m, 2H), 6.44 – 6.39 (m, 2H), 6.38 – 6.35 (m, 1H), 6.24 (s,

1H), 3.27 – 3.19 (m, 1H), 3.18 – 3.10 (m, 1H), 3.08 – 2.96 (m, 5H), 2.82 – 2.72 (m, 1H), 1.56 (s, 9H).^{13}C NMR (101 MHz, $CDCl_3$) δ 152.9, 141.2, 139.4, 138.9, 137.2, 135.1, 133.3, 133.1, 132.3, 129.0, 128.3, 127.9, 125.2, 80.45, 35.4, 35.1, 33.8, 33.0, 28.5. $[\alpha]^{25}_{D}$ = 152.3 (c = 0.5, $CHCl_3$). m/z HRMS (ESI) found $[M-56+H]^+$ 268.1334, $C_{17}H_{18}NO_2^+$ calculated 268.1332. HPLC: Chiralpak IC column, 70:30 hexanes/isopropanol, 1 ml/min; t_R = 5.05 min (major), 6.88 min (minor); 94% ee.

(*S*$_p$)-Tert-butyl (1^5-amino-1,4(1,4)-dibenzenacyclohexaphane-1^2-yl)carbamate **(9a)**

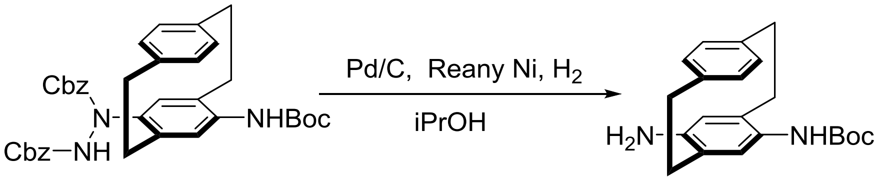

3a, 94% ee   **9a**, 71% yield, 95% ee

To a solution of **3a** (62 mg, 0.1 mmol, 1.0 equiv.) in iPrOH (4 mL) were added Pd/C (150 mg, 10 wt%), and Raney Ni (150 mg). The mixture was stirred at rt under H_2 atmosphere overnight. After completion of the reaction as indicated by TLC analysis, the reaction mixture was filtered and the filtrate was concentrated under vacuum to give a residue, which was purified by column chromatography (petroleum ether/EtOAc = 2:1) to afford **9a** (24 mg, 71%) as a yellow solid. ^{1}H NMR (400 MHz, $CDCl_3$) δ 7.14 (dd, *J* = 7.8, 2.0 Hz, 1H), 6.78 (dd, *J* = 7.8, 2.0 Hz, 1H), 6.48 – 6.43 (m, 1H), 6.40 (dd, *J* = 7.8, 2.0 Hz, 1H), 6.34 (s, 1H), 6.09 (s, 1H), 5.43 (s, 1H), 3.25 – 2.90 (m, 8H), 2.65 – 2.50 (m, 2H), 1.53 (s, 9H). ^{13}C NMR (101 MHz, $CDCl_3$) δ 142.5, 139.1, 138.9, 132.9, 132.0, 129.4, 129.0, 128.4, 125.0, 123.9, 80.0, 33.9, 32.8, 32.6, 31.9, 28.6. $[\alpha]^{25}_{D}$ = 101.9 (c = 0.5, $CHCl_3$). m/z HRMS (ESI) found $[M+H]^+$ 339.2092, $C_{21}H_{27}N_2O_2^+$ calculated 339.2067. HPLC: Chiralpak IA column, 70:30 hexanes/isopropanol, 1 ml/min; t_R = 7.42 min (major), 9.26 min (minor); 95% ee.

(S_p)-Tert-butyl-(1^5-(3-(3,5-bis(trifluoromethyl)phenyl)thioureido)-1,4(1,4)-dibenzenacyclohexaphane-1^2-yl)carbamate **(10a)**

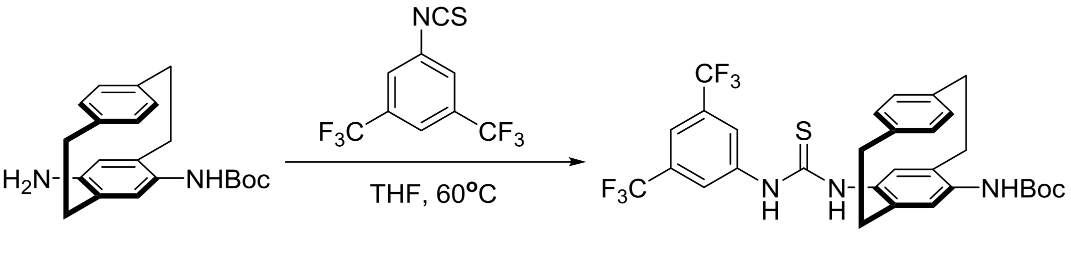

9a, 95% ee   **10a**, 94% yield, 92% ee

To a solution of **9a** (59 mg, 0.17 mmol, 1.0 equiv.) in THF (2 mL) was added 1-isothiocyanato-3,5-bis(trifluoromethyl)benzene (0.0037 mL, 0.20 mmol, 1.2 equiv.), and the mixture was stirred at 60°C for 5 h. After completion of the reaction as indicated by TLC analysis, the reaction mixture was concentrated under vacuum to give a residue, which was purified by column chromatography (petroleum ether/EtOAc = 5:1) to afford **10a** (100 mg, 94%) as a yellow solid. ^{1}H NMR (400 MHz, $CDCl_3$) δ 8.04 (s, 1H), 7.98 (s, 2H), 7.69 (s, 1H), 7.65 (s, 1H), 6.98 (s, 1H), 6.92 (dd, *J* = 8.0, 2.0 Hz, 1H), 6.86 (dd, *J* = 7.9, 1.9 Hz, 1H), 6.56 (dd, *J* = 7.9, 1.9 Hz, 1H), 6.47 (dd, *J* = 8.0, 2.0 Hz, 1H), 6.42 (s, 1H), 6.20 (s, 1H), 3.25 – 3.04 (m, 6H), 2.85 – 2.71 (m, 2H), 1.57 (s, 9H). ^{13}C NMR (126 MHz, $CDCl_3$) δ 179.5, 152.8, 139.0 (q, *J* = 68.0 Hz), 137.7, 132.8, 132.7, 132.1, 131.8, 131.6, 129.9, 129.4, 129.3, 127.8, 124.7, 124.2, 122.0, 119.4, 81.5, 34.5, 33.4, 32.3, 32.2, 28.5. ^{19}F NMR (471 MHz, $CDCl_3$) δ -62.90. $[\alpha]^{25}_D$ = 130.1 (c = 0.5, $CHCl_3$). m/z HRMS (ESI) found $[M+H]^+$ 610.1959, $C_{30}H_{30}F_6N_3O_2S^+$ calculated 610.1957. HPLC: Chiralpak IC column, 95:05 hexanes/isopropanol, 1 ml/min; t_R = 7.91 min (minor), 10.17 min (major); 92% ee.

(S_p)-1,1'-(1,4(1,4)-dibenzenacyclohexaphane-1^2,1^5-diyl)bis(3-(3,5-bis(trifluoromethyl) phenyl)thiourea) **(11a)**

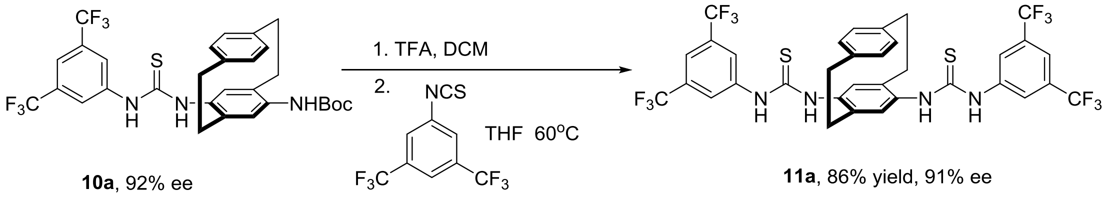

10a, 92% ee   **11a**, 86% yield, 91% ee

To a solution of **10a** (75 mg, 0.12 mmol, 1.0 equiv.) in DCM (3 mL) was added TFA (1 mL). The mixture was stirred at rt for 3 h. As the TLC analysis showed the

completion of the reaction, the reaction mixture was quenched with saturated $NaHCO_3$ solution, and extracted with DCM (3×10 mL). The combined organic layers were washed with brine (10 mL), dried over Na_2SO_4, filtered and concentrated to afford the residue (61 mg) as a yellow solid.

To a solution of the above residue (61 mg, 0.12 mmol, 1.0 equiv.) in THF (2 mL) were added 1-isothiocyanato-3,5-bis(trifluoromethyl)benzene (0.0026 mL, 0.14 mmol, 1.2 equiv.), and the mixture was allowed to stir at 60 ^{o}C overnight. After completion of the reaction as indicated by TLC analysis, the reaction mixture was concentrated under vacuum to give a residue, which was purified by column chromatography (petroleum ether/EtOAc = 2:1) to afford **11a** (80 mg, 86%) as a yellow solid. ^{1}H NMR (400 MHz, $CDCl_3$) δ 8.40 (s, 2H), 7.95 (s, 4H), 7.76 (s, 2H), 7.71 (s, 2H), 7.03 (dd, *J* = 8.0, 2.0 Hz, 2H), 6.57 (dd, *J* = 8.0, 2.0 Hz, 2H), 6.40 (s, 2H), 3.33 – 3.13 (m, 6H), 2.92 – 2.81 (m, 2H). ^{13}C NMR (101 MHz, $CDCl_3$) δ 179.3, 139.4, 139.3, 139.0, 135.2, 133.6, 133.0, 132.3 (q, *J* = 33.3 Hz), 129.5, 125.0, 122.9 (q, *J* = 274.7 Hz), 120.0, 34.5, 32.7. ^{19}F NMR (471 MHz, $CDCl_3$) δ -62.90. $[\alpha]^{25}_{D}$ = 314.8 (c = 0.5, $CHCl_3$). m/z HRMS (ESI) found $[M+H]^+$ 781.1324, $C_{34}H_{25}F_{12}N_4S_2^+$ calculated 781.1324. HPLC: Chiralpak IC column, 95:05 hexanes/isopropanol, 1 ml/min; t_R = 4.25 min (minor), 5.11 min (major); 91% ee.

(S_p)-Tert-butyl (1^5-iodo-1,4(1,4)-dibenzenacyclohexaphane-1^2-yl)carbamate **(12a)**

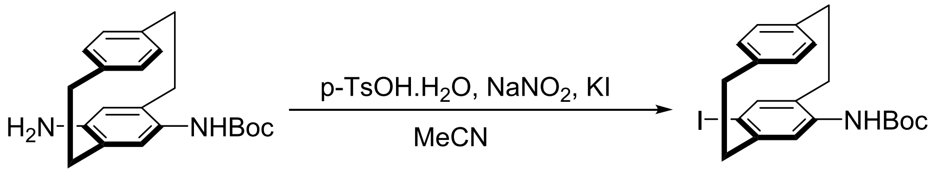

9a, 95% ee **12a**, 58% yield, 93% ee

To a solution of **9a** (34 mg, 0.1 mmol, 1.0 equiv.), p-TsOH.H_2O (57 mg, 0.3 mml, 3.0 equiv.) in MeCN (6 mL) was added a solution of $NaNO_2$ (14 mg, 0.2 mmol, 2.0 equiv.) , KI (42 mg, 0.25 mmol, 2.5 equiv.) in H_2O (0.7 mL) at 0 ^{o}C. After completion of the reaction as indicated by TLC analysis (~1 h), the reaction mixture was quenched with saturated $NaHCO_3$ solution, and extracted with EtOAc (3×5 mL). The

combined organic layers were washed with brine (5 mL), dried over Na_2SO_4, filtered and concentrated under vacuum to give a residue, which was purified by column chromatography (petroleum ether/EtOAc = 10:1) to afford **12a** (26 mg, 58%) as yellow solid. ^{1}H NMR (400 MHz, $CDCl_3$) δ 7.19 (dd, *J* = 7.9, 2.0 Hz, 1H), 6.80 (dd, *J* = 7.8, 2.1 Hz, 2H), 6.74 (s, 1H), 6.53 (dd, *J* = 7.9, 2.0 Hz, 1H), 6.44 (dd, *J* = 8.0, 2.0 Hz, 1H), 6.25 (s, 1H), 3.33 – 3.11 (m, 4H), 3.06 – 2.97 (m, 2H), 2.93 – 2.83 (m, 1H), 2.70 – 2.57 (m, 1H), 1.56 (s, 9H). ^{13}C NMR (101 MHz, $CDCl_3$) δ 152.6, 144.3, 144.2, 139.2, 138.5, 137.6, 132.6, 132.5, 130.1, 129.0, 125.6, 96.52, 80.9, 39.2, 33.6, 33.2, 32.6, 28.5. $[\alpha]^{25}_D$ = 206.8 (c = 0.5, $CHCl_3$). m/z HRMS (ESI) found $[M+H]^+$ 450.0913, $C_{21}H_{25}INO_2^+$ calculated 450.0924. HPLC: Chiralpak ID column, 95:05 hexanes/isopropanol, 1 ml/min; t_R = 6.08 min (major), 6.73 min (minor); 93% ee.

(S_p)-*N*-(4^2-bromo-1,4(1,4)-dibenzenacyclohexaphane-1^2-yl)pivalamide **(13f)**

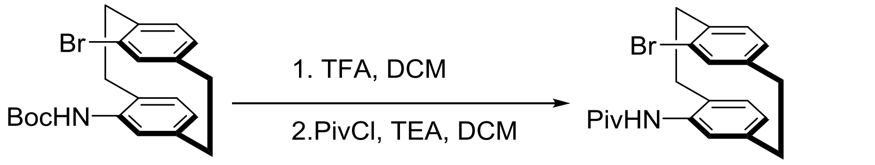

(S_p)-**1f**, 91% ee   **13f**, 93% yield, 90% ee

To a solution of (S_p)-**1f** (121 mg, 0.3 mmol, 1.0 equiv.) in DCM (3 mL) was added TFA (1 mL). After completion of the reaction as indicated by TLC analysis (~3 h), the reaction mixture was quenched with saturated $NaHCO_3$ solution, and extracted with DCM (3×5 mL). The combined organic layers were washed with brine (5 mL), dried over Na_2SO_4, filtered and concentrated under vacuum to afford the residue (90 mg) as a yellow solid.

To a solution of the above residue (90 mg, 0.3 mmol, 1.0 equiv.), TEA (0.050 mL, 0.36 mmol, 1.2 equiv.) in DCM (3 mL) was added PivCl (0.044 mL, 0.36 mmol, 1.2 equiv.) at 0 ^{o}C. After completion of the reaction as indicated by TLC analysis (~0.5 h), the reaction mixture was quenched with saturated NH_4Cl solution, and extracted with DCM (3×5 mL). The combined organic layers were washed with brine (5 mL), dried over Na_2SO_4, filtered and concentrated under vacuum to give a residue, which was

purified by column chromatography (petroleum ether/EtOAc = 5:1) to afford **13f** (108 mg, 93%) as a yellow solid. ^{1}H NMR (400 MHz, $CDCl_3$) δ 7.71 (s, 1H), 6.76 (s, 1H), 6.69 (d, *J* = 1.9 Hz, 1H), 6.57 – 6.52 (m, 3H), 6.44 (dd, *J* = 7.8, 1.9 Hz, 1H), 3.58 – 3.46 (m, 1H), 3.36 – 3.26 (m, 1H), 3.07 – 2.94 (m, 6H), 1.35 (s, 9H). ^{13}C NMR (101 MHz, $CDCl_3$) δ 175.2, 146.8, 141.6, 141.3, 139.3, 137.1, 136.2, 135.5, 134.3, 132.6, 131.7, 124.2, 83.6, 39.5, 36.1, 35.4, 34.9, 32.1, 27.6, 25.6, 24.8.

$[\alpha]^{25}_D$ = 23.4 (c = 0.5, $CHCl_3$). m/z HRMS (ESI) found $[M+H]^+$ 386.1113, $C_{21}H_{25}BrNO^+$ calculated 386.1114. HPLC: Chiralpak IC column, 70:30 hexanes/isopropanol, 1 ml/min; t_R = 12.41 min (minor), 15.91 min (major); 90% ee.

(S_p)-*N*-(4^2-bromo-1^3-(4,4,5,5-tetramethyl-1,3,2-dioxaborolan-2-yl)-1,4(1,4)-dibenzenacyclohexaphane-1^2-yl)pivalamide **(14f)**

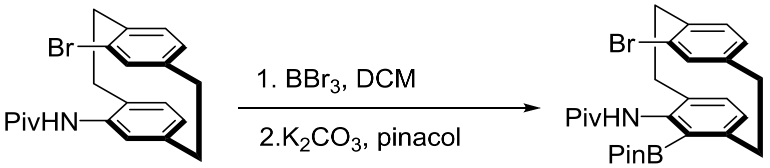

6f, 90% ee **14f**, 80% yield, 91% ee

To a solution of **6f** (88 mg, 0.23 mmol, 1.0 equiv.) in DCM (2.3 mL) was added BBr_3 (0.28 mL, 0.28 mmol, 1.2 equiv.). The mixture was stirred at rt for 3 h, then cooled to 0 °C, quenched with a solution of K_2CO_3 (80 mg, 0.58 mmol, 2.5 equiv.) in H_2O (0.6 mL). Then a solution of pinacol in DCM (0.6 mL) was added, and the mixture was warmed to rt and allowed to stir at rt for 1 h. After completion of the reaction as indicated by TLC analysis, the reaction mixture was added $MgSO_4$, filtered, washed with DCM and concentrated under vacuum to give a residue, which was purified by column chromatography (petroleum ether/EtOAc = 3:1) to afford **14f** (93 mg, 80%) as a yellow solid. ^{1}H NMR (400 MHz, $CDCl_3$) δ 9.36 (s, 1H), 6.70 – 6.50 (m, 4H), 6.43 (d, *J* = 7.8 Hz, 1H), 4.06 – 3.94 (m, 1H), 3.48 – 3.38 (m, 1H), 3.32 – 3.22 (m, 1H), 3.19 – 3.06 (m, 1H), 3.02 – 2.74 (m, 4H), 1.43 (s, 6H), 1.42 (s, 6H), 1.36 (s, 9H). ^{13}C NMR (101 MHz, $CDCl_3$) δ 175.2, 146.8, 141.6, 141.3, 139.3, 137.1, 136.2, 135.5, 134.3, 132.6, 131.7, 124.2, 83.6, 39.5, 36.1, 35.4, 34.9, 32.1, 27.6, 25.6, 24.8. $[\alpha]^{25}_D$ =

70.7 (c = 0.5, $CHCl_3$). m/z HRMS (ESI) found $[M+H]^+$ 512.1985, $C_{27}H_{36}BBrNO_3^+$ calculated 512.1966. HPLC: Chiralpak IA column, 90:10 hexanes/isopropanol, 1 ml/min; t_R = 13.96 min (major), 16.80 min (minor); 91% ee.

(S_p)-4^2-bromo-1^2,1^4-dimethyl-1(5,8)-quinolina-4(1,4)-benzenacyclohexaphane **(15f)**

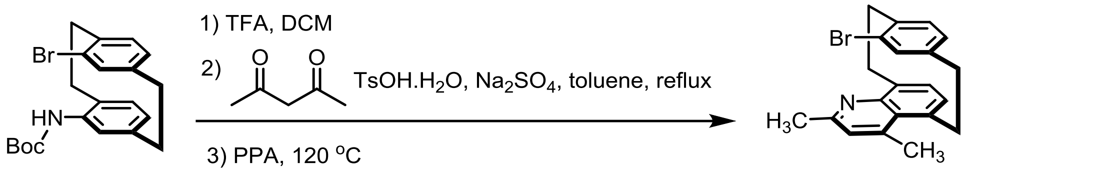

(S_p)-**1f**, 91% ee
15f, 42% for three steps, 96% ee

To a solution of (S_p)-**1f** (400 mg, 1 mmol, 1.0 equiv.) in DCM (5 mL) was added TFA (1 mL), and the mixture was stirred at rt for 3 h. After completion of the reaction as indicated by TLC analysis, the reaction mixture was quenched with saturated $NaHCO_3$ solution, and extracted with DCM (3×10 mL). The combined organic layers were washed with brine (10 mL), dried over Na_2SO_4, filtered and concentrated under vacuum to afford the residue (300 mg) as a yellow solid.

To a solution of the above residue (300 mg, 1 mmol, 1.0 equiv.), pentane-2,4-dione (0.12 mL, 1.2 mmol, 1.2 equiv.), Na_2SO_4 (4 g) in toluene (5 mL) was added $TsOH.H_2O$ (38 mg, 0.2 mmol, 0.2 equiv.) at rt. The mixture was allowed to reflux overnight. Since the TLC analysis indicated the completion of the reaction, the reaction mixture was filtered, the filtrate was concentrated under vacuum to give a residue, which was purified by column chromatography (petroleum ether/EtOAc = 5:1) to afford the corresponding enamine (240 mg, 63%) as a yellow solid. ^{1}H NMR (400 MHz, $CDCl_3$) δ 12.68 (s, 1H), 6.70 (s, 1H), 6.59 – 6.57 (m, 2H), 6.50 (d, J = 7.7 Hz, 1H), 6.42 (dd, J = 7.8, 1.8 Hz, 1H), 6.37 – 6.34 (m, 1H), 5.11 (s, 1H), 3.72 – 3.60 (m, 1H), 3.54 – 3.44 (m, 1H), 3.10 – 2.93 (m, 6H), 2.11 (s, 3H), 1.67 (s, 3H). ^{13}C NMR (126 MHz, $CDCl_3$) δ 195.6, 160.5, 141.3, 140.1, 138.8, 137.8, 136.5, 135.3, 135.0, 134.0, 131.8, 131.7, 128.9, 125.7, 97.1, 35.6, 35.0, 34.7, 30.3, 29.3, 19.9. m/z HRMS (ESI) found $[M+H]^+$ 384.0956, $C_{21}H_{23}BrNO^+$ calculated 384.0958. HPLC:

Chiralpak ID column, 85:15 hexanes/isopropanol, 1 ml/min; t_R = 9.39 min (minor), 10.47 min (major); 96% ee.

A solution of the enamine (240 mg, 0.62 mmol, 1.0 equiv.) in PPA (6 mL) was heated to 120°C, and allowed to stir at 120°C for 2.5 h. After the TLC analysis indicated the completion of the reaction, the reaction mixture was quenched with ice-water, saturated $NaHCO_3$ solution, and extracted with EtOAc (3×10 mL). The combined organic layers were washed with brine (10 mL), dried over Na_2SO_4, filtered and concentrated under vaccum to give a residue, which was purified by column chromatography (DCM/MeOH = 100:1) to afford **15f** (60 mg, 67%) as a yellow solid. ^{1}H NMR (400 MHz, $CDCl_3$) δ 7.00 (s, 1H), 6.91 (d, *J* = 7.2 Hz, 1H), 6.77 (d, *J* = 7.2 Hz, 1H), 6.55 – 6.45 (m, 2H), 5.84 (s, 1H), 4.58 – 4.48 (m, 1H), 3.95 – 3.85 (m, 1H), 3.62 – 3.52 (m, 1H), 3.22 – 3.13 (m, 1H), 3.07 – 2.92 (m, 3H), 2.68 (s, 4H), 2.64 (s, 3H), 2.54 – 2.43 (m, 1H). ^{13}C NMR (101 MHz, $CDCl_3$) δ 156.4, 150.0, 142.4, 139.9, 139.0, 138.6, 136.5, 134.1, 133.4, 133.2, 132.0, 130.8, 127.7, 124.3, 122.5, 38.1, 35.1, 34.8, 30.4, 25.0, 22.9. $[\alpha]^{25}_D$ = 42.9 (c = 0.5, $CHCl_3$). m/z HRMS (ESI) found $[M+H]^+$ 366.0859, $C_{21}H_{21}BrN^+$ calculated 366.0852. HPLC: Chiralpak IB column, 95:05 hexanes/ethanol, 1 ml/min; t_R = 6.51 min (minor), 7.57 min (major); 96% ee.

(R_p)-Tert-butyl (1^5-iodo-1,4(1,4)-dibenzenacyclohexaphane-1^2-yl)carbamate **(16a)**

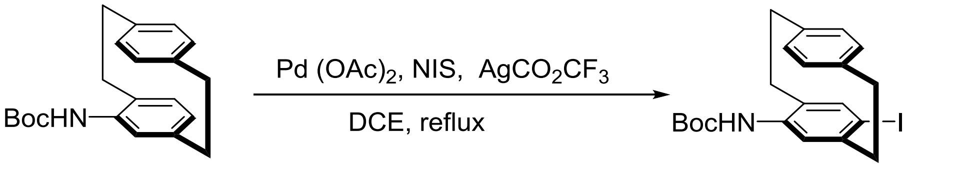

(R_p)-**1a**, 99% ee

16a, 72% yield, 97% ee

To a solution of (R_p)-**1a** (65 mg, 0.2 mmol, 1.0 equiv.), $AgCO_2CF_3$ (9 mg, 0.04 mmol, 0.2 equiv.), NIS (54 mg, 0.24 mmol, 1.2 equiv.) in DCE (5 mL) was added $Pd(OAc)_2$ (9 mg, 0.04 mmol, 0.2 equiv.) at rt. The mixture was allowed to reflux under N_2 atmosphere overnight. After the TLC analysis showed the completion of the reaction, the reaction mixture was concentrated under vacuum to give a residue, which was

purified by column chromatography (petroleum ether/EtOAc = 8:1) to afford **16a** (65 mg, 72%) as yellow solid. ^{1}H NMR (400 MHz, $CDCl_3$) δ 7.19 (dd, J = 7.9, 2.0 Hz, 1H), 6.80 (dd, J = 7.8, 2.1 Hz, 2H), 6.74 (s, 1H), 6.53 (dd, J = 7.9, 2.0 Hz, 1H), 6.44 (dd, J = 8.0, 2.0 Hz, 1H), 6.25 (s, 1H), 3.33 – 3.11 (m, 4H), 3.06 – 2.97 (m, 2H), 2.93 – 2.83 (m, 1H), 2.70 – 2.57 (m, 1H), 1.56 (s, 9H). ^{13}C NMR (101 MHz, $CDCl_3$) δ 152.6, 144.3, 144.2, 139.2, 138.5, 137.6, 132.6, 132.5, 130.1, 129.0, 125.6, 96.52, 80.9, 39.2, 33.6, 33.2, 32.6, 28.5. $[\alpha]^{25}_{D}$ = -156.6 (c = 0.5, $CHCl_3$). m/z HRMS (ESI) found $[M+H]^+$ 450.0913, $C_{21}H_{25}INO_2^+$ calculated 450.0918. HPLC: Chiralpak ID column, 95:05 hexanes/isopropanol, 1 ml/min; t_R = 6.51 min (minor), 7.16 min (major); 97% ee.

(R_p)-*N*-benzyl-1^{1}H-1(4,7)-indola-4(1,4)-benzenacyclohexaphane-1^2-carboxamide **(17a)**

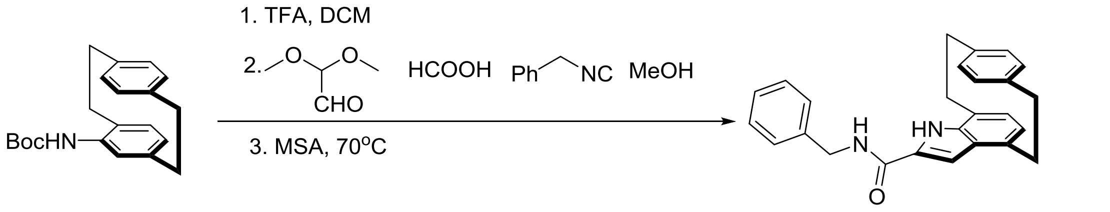

(R_p)-**1a**, 99% ee　　**17a**, 47% yield for 3 steps, 98% ee

To a solution of (R_p)-**1a** (64 mg, 0.2 mmol, 1.0 equiv.) in DCM (3 mL) was added TFA (1 mL), and the mixture was allowed to stir at rt for 3 h. After the TLC analysis indicated the completion of the reaction, the reaction mixture was quenched with saturated $NaHCO_3$ solution, and extracted with DCM (3×10 mL). The combined organic layers were washed with brine (10 mL), dried over Na_2SO_4, filtered and concentrated under vacuum to afford the residue (45 mg) as a yellow solid.

To a solution of the above residue (45 mg, 0.12 mmol, 1.0 equiv.), 2,2-dimethoxyacetaldehyde (0.037 mL, 0.4 mmol, 2.0 equiv.), (isocyanomethyl)benzene (0.049 mL, 0.4 mmol, 2.0 equiv.) in MeOH (0.2 mL) was added HCOOH (0.015 mL, 0.4 mmol, 2.0 equiv.) at rt. After the TLC analysis

showed the completion of the reaction (~4 h), the reaction mixture was concentrated under vacuum to give a residue, which was purified by column chromatography (petroleum ether/EtOAc = 1:1) to afford corresponding amide (77 mg, 80%) as a white solid.

A solution of the above amide in anhydrous MSA was heated to 70 ^{o}C. After stirring at 70°C for 3 h, the reaction mixture was quenched with saturated $NaHCO_3$ solution, and extracted with EtOAc (3×10 mL). The combined organic layers were washed with brine (10 mL), dried over Na_2SO_4, filtered and concentrated under vacuum to give a residue, which was purified by column chromatography (petroleum ether/EtOAc = 2:1) to afford **17a** (30 mg, 49%) as yellow solid. ^{1}H NMR (500 MHz, $CDCl_3$) δ 9.17 (s, 1H), 7.47 – 7.32 (m, 5H), 6.60 (d, *J* = 2.2 Hz, 1H), 6.53 (d, *J* = 7.3 Hz, 1H), 6.50 – 6.41 (m, 3H), 6.39 (dd, *J* = 7.8, 2.0 Hz, 1H), 6.00 (dd, *J* = 7.8, 2.0 Hz, 1H), 5.91 (dd, *J* = 7.8, 2.0 Hz, 1H), 4.78 (dd, *J* = 14.7, 6.0 Hz, 1H), 4.68 (dd, *J* = 14.7, 5.5 Hz, 1H), 3.41 – 3.25 (m, 2H), 2.97 (qt, *J* = 11.1, 4.1 Hz, 5H), 2.89 – 2.80 (m, 1H). ^{13}C NMR (101 MHz, $CDCl_3$) δ 162.1, 138.0, 137.8, 137.6, 134.4, 132.1, 131.7, 130.9, 130.5, 129.0, 128.9, 128.1, 127.2, 126.4, 124.5, 123.8, 103.4, 43.9, 34.7, 33.9, 32.6, 31.2. $[\alpha]^{25}_{D}$ = 82.7 (c = 0.5, $CHCl_3$). m/z HRMS (ESI) found $[M+H]^+$ 381.1953, $C_{26}H_{25}N_2O^+$ calculated 381.1961. HPLC: Chiralpak IC column, 70:30 hexanes/isopropanol, 1 ml/min; t_R = 8.58 min (major), 14.01 min (minor); 98% ee.

1.8 Applications of amido-PCP in the development of chiral organocatalyst

1-(1,4(1,4)-dibenzenacyclohexaphane-1^2-yl)-3-((*R$_p$*,1*R*,2*R*)-2-(dimethylamino)cyclohexyl)thiourea **(19a)**

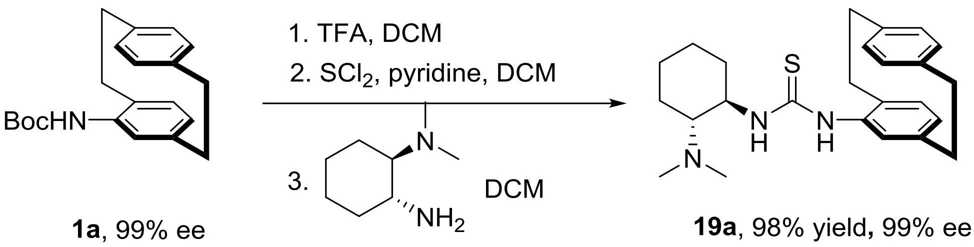

1a, 99% ee **19a**, 98% yield, 99% ee

To a solution of **1a** (32 mg, 0.1 mmol, 1.0 equiv.) in DCM (3 mL) was added TFA (1 mL) at rt. After stirring at rt for 3 h, the reaction mixture was quenched with saturated $NaHCO_3$ solution, and extracted with DCM (3×5 mL). The combined organic layers were washed with brine (5 mL), dried over Na_2SO_4, filtered and concentrated to afford the residue (22 mg) as a yellow solid.

To a solution of the above residue (22 mg, 0.1 mmol, 1.0 equiv.), pyridine (0.016 mL, 0.2 mmol, 2.0 equiv.) in DCM (1 mL) was added SCl_2 (0.011 mL, 0.15 mmol, 1.5 equiv.). The reaction mixture was allowed to stir at rt for 2 h. After the TLC analysis showed the completion of the reaction, the reaction mixture was purified by column chromatography (petroleum ether/EtOAc = 20:1) to afford the crude product.

The above crude product was dissolved in DCM (1 mL), (1*R*,2*R*)-N^1,N^1-dimethylcyclohexane-1,2-diamine (0.031mL, 0.2 mmol, 2.0 equiv.) was added at rt. After stirring at rt for 1 h, the reaction mixture was purified by column chromatography (DCM/MeOH = 15:1) to afford **19a** (40 mg, 98%) as a yellow solid. ^{1}H NMR (400 MHz, $CDCl_3$) δ 7.38 (s, 1H), 6.89 (dd, *J* = 7.9, 2.0 Hz, 1H), 6.64 – 6.50 (m, 4H), 6.46 (dd, *J* = 7.9, 2.0 Hz, 1H), 6.41 (dd, *J* = 7.9, 2.0 Hz, 1H) 6.03 (s, 1H), 3.90 – 3.80 (m, 1H), 3.35 – 3.25 (m, 1H), 3.15 – 2.95 (m, 6H), 2.77 – 2.62 (m, 2H), 2.20 (s, 6H), 1.84 – 1.64 (m, 4H), 1.32 – 1.04 (m, 3H), 0.90 – 0.75 (m, 1H). ^{13}C NMR (101 MHz, $CDCl_3$) δ 178.6, 142.4, 139.9, 139.2, 136.7, 135.8, 134.8, 133.6, 132.9, 132.3, 132.2, 130.2, 128.6, 66.5, 56.0, 40.0, 35.2, 34.8, 34.6, 33.0, 32.5, 25.2, 24.6, 21.5. $[\alpha]^{25}_D$ = -161.4 (c = 0.5, $CHCl_3$). m/z HRMS (ESI) found $[M+H]^+$ 408.2461, $C_{25}H_{34}N_3S^+$ calculated 408.2468.

1-(1,4(1,4)-dibenzenacyclohexaphane-1^2-yl)-3-((R_p,1*S*,2*S*)-2-(dimethylamino)cyclohexyl)thiourea **(20a)**

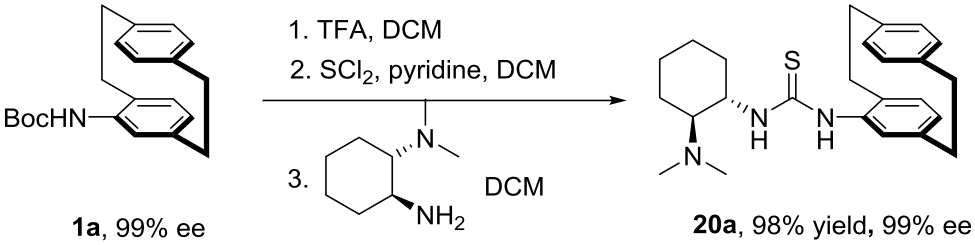

To a solution of **1a** (32 mg, 0.1 mmol, 1.0 equiv.) in DCM (3 mL) was added TFA (1 mL) at rt. After stirring at rt for 3 h, the reaction mixture was quenched with saturated $NaHCO_3$ solution, and extracted with DCM (3×5 mL). The combined organic layers were washed with brine (5 mL), dried over Na_2SO_4, filtered and concentrated under vacuum to afford the residue (22 mg) as a yellow solid.

To a solution of the above residue (22 mg, 0.1 mmol, 1.0 equiv.), pyridine (0.016 mL, 0.2 mmol, 2.0 equiv.) in DCM (1 mL) was added SCl_2 (0.011 mL, 0.15 mmol, 1.5 equiv.). After the TLC analysis showed the completion of the reaction (~2 h), the reaction mixture was purified by column chromatography (petroleum ether/EtOAc = 20:1) to afford crude product.

The above crude product was dissolved in DCM (1 mL), (1*S*,2*S*)-N^1,N^1-dimethylcyclohexane-1,2-diamine (0.031mL, 0.2 mmol, 2.0 equiv.) was added at rt. After the TLC analysis showed the completion of the reaction (~1 h), the reaction mixture was purified by column chromatography (DCM/MeOH = 15:1) to afford **20a** (40 mg, 98%) as a yellow solid. ^{1}H NMR (400 MHz, $CDCl_3$) δ 6.90 (dd, *J* = 7.9, 2.0 Hz, 1H), 6.61 – 6.49 (m, 3H), 6.46 (dd, *J* = 7.9, 2.0 Hz, 1H), 6.40 (dd, *J* = 7.9, 1.9 Hz, 1H), 6.06 (s, 1H), 3.95 – 3.80 (m, 1H), 3.23 – 2.91 (m, 7H), 2.83 –2.70 (m, 2H), 2.18 (s, 1H), 2.04 (s, 6H), 1.85 – 1.67 (m, 3H), 1.33 – 0.97 (m, 4H). ^{13}C NMR (101 MHz, $CDCl_3$) δ 179.0, 142.2, 139.5, 139.3, 136.0, 135.0, 134.8, 133.6, 133.0, 132.2, 131.7, 130.2, 128.3, 66.6, 56.2, 39.9, 35.2, 34.9, 34.5, 32.9, 32.8, 25.2, 24.6, 22.0. $[\alpha]^{25}_D$ = -40.2 (c = 0.5, $CHCl_3$). m/z HRMS (ESI) found $[M+H]^+$ 408.2461, $C_{25}H_{34}N_3S^+$ calculated 408.2468.

Synthesis of other known organocatalysts

1-(3,5-bis(trifluoromethyl)phenyl)-3-((1*R*,2*R*)-2-(dimethylamino)cyclohexyl)thiourea **(24b)**

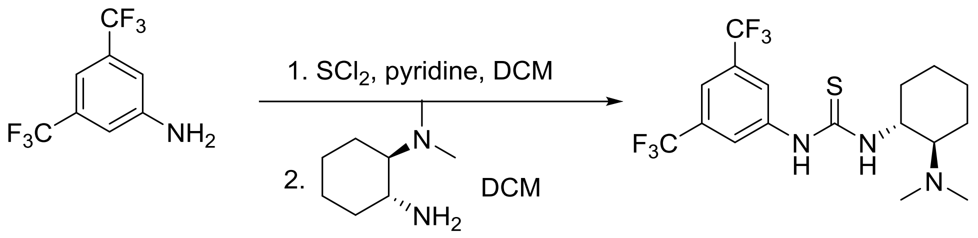

24b, 98% yield

To a solution of 3,5-bis(trifluoromethyl)aniline (23 mg, 0.1 mmol, 1.0 equiv.), pyridine (0.016 mL, 0.2 mmol, 2.0 equiv.) in DCM (1 mL) was added SCl_2 (0.011 mL, 0.15 mmol, 1.5 equiv.), and the reaction mixture was stirred at rt for 2 h. After the TLC analysis showed the completion of the reaction, the reaction mixture was purified by column chromatography (petroleum ether/EtOAc = 20:1) to afford crude product.

The above crude product was dissolved in DCM (1 mL), (1*R*,2*R*)-N^1,N^1-dimethylcyclohexane-1,2-diamine (0.031mL, 0.2 mmol, 2.0 equiv.) was added at rt. After stirring at rt for 1 h, the reaction mixture was purified by column chromatography (DCM/MeOH = 15:1) to afford **24b** (40 mg, 98%) as a yellow solid. ^{1}H NMR (400 MHz, $CDCl_3$) δ 7.83 (s, 2H), 7.61 (s, 1H), 6.24 (s, 1H), 3.80 (s, 1H), 2.65 – 2.40 (m, 2H), 2.32 (s, 6H), 2.00 – 1.63 (m, 4H), 1.40 – 1.04 (m, 4H). ^{13}C NMR (101 MHz, $CDCl_3$) δ 179.4, 139.8, 132.5 (q, J = 35.4 Hz), 123.6, 123.04 (q, J = 273.7 Hz), 118.6, 66.8, 56.2, 40.2, 32.9, 24.9, 24.6, 21.5. ^{19}F NMR (376 MHz, $CDCl_3$) δ -63.08. $[\alpha]^{25}_D$ = -24.4 (c = 0.5, $CHCl_3$).m/z HRMS (ESI) found $[M+H]^+$ 414.1435, $C_{17}H_{22}F_6N_3S^+$ calculated 414.1433.

1-((1*R*,2*R*)-2-(dimethylamino)cyclohexyl)-3-phenylthiourea **(24a)**

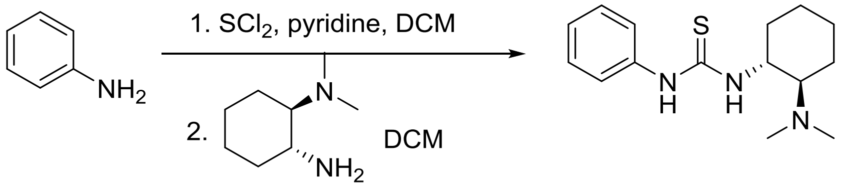

24a, 98% yield

To a solution of aniline (0.09 mL, 1 mmol, 1.0 equiv.), pyridine (0.16 mL, 2 mmol, 2.0 equiv.) in DCM (5 mL) was added SCl_2 (0.11 mL, 1.5 mmol, 1.5 equiv.), and the reaction mixture was stirred at rt for 2 h. After the TLC analysis showed the completion of the reaction, the reaction mixture was purified by column chromatography (petroleum ether/EtOAc = 20:1) to afford the crude product.

The above crude product was dissolved in DCM (1 mL), (1*R*,2*R*)-N^1,N^1-dimethylcyclohexane-1,2-diamine (0.31mL, 2 mmol, 2.0 equiv.) was added at rt. After stirring at rt for 1 h, the reaction mixture was purified by column chromatography (DCM/MeOH = 15:1) to afford **24a** (277 mg, 98%) as a yellow solid. ^{1}H NMR (400 MHz, $CDCl_3$) δ 7.95 (s, 1H), 7.41 – 7.32 (m, 2H), 7.26 – 7.15 (m, 3H), 6.81 (s, 1H), 3.95 – 3.75 (m, 1H), 2.80 – 2.60 (m, 1H), 2.40 – 2.30 (m, 1H), 2.24 (s, 6H), 1.89 – 1.78 (m, 2H), 1.67 – 1.62 (s, 1H), 1.40 – 1.27 (m, 1H), 1.26 – 1.12 (m, 2H), 1.10 – 0.97 (m, 1H). ^{13}C NMR (101 MHz, $CDCl_3$) δ 180.0, 137.3, 129.7, 126.2, 124.5, 67.0, 56.4, 40.0, 32.9, 25.2, 24.7, 21.7. $[\alpha]^{25}_D$ = -96.9 (c = 0.5, $CHCl_3$). m/z HRMS (ESI) found $[M+H]^+$ 278.1683, $C_{15}H_{24}N_3S^+$ calculated 278.1685.

Application of the organocatalysts in the catalytic asymmetric (4+2) annulation

(E)-*N*-(benzo[d]thiazol-2-yl)-1-phenylmethanimine **(21a)**

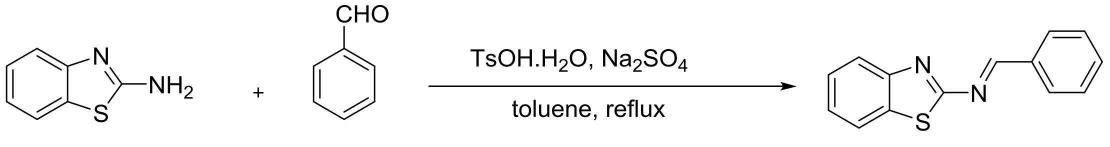

21a

To a solution of benzo[d]thiazol-2-amine (1.05 g, 7 mmol, 1.0 equiv.), benzaldehyde (1.07 mL, 10.5 mmol, 1.5 equiv.), Na_2SO_4 (3 g) in toluene (5 mL) was added $TsOH.H_2O$ (133 mg, 0.7 mmol, 0.1 equiv.). The mixture was allowed to reflux overnight. After the TLC analysis indicated the completion of the reaction, the reaction mixture was filtered and the filtrate was concentrated under vacuum to give a residue, which was purified by column chromatography (petroleum ether/EtOAc = 30:1) to afford **21a** (800 mg, 48%) as a yellow solid. ^{1}H NMR (500 MHz, $CDCl_3$) δ 9.09 (s, 1H), 8.07 – 8.02 (m, 2H), 7.99 (d, *J* = 8.1 Hz, 1H), 7.84 (dd, *J* = 8.0, 1.2 Hz,

1H), 7.59 – 7.56 (m, 1H), 7.54 – 7.46 (m, 3H), 7.40 – 7.36 (m, 1H). ^{13}C NMR (101 MHz, $CDCl_3$) δ 171.9, 166.3, 151.8, 134.8, 134.7, 133.4, 130.4, 129.1, 126.6, 125.2, 123.2, 121.8. m/z HRMS (ESI) found $[M+H]^+$ 239.0638, $C_{14}H_{11}N_2S^+$ calculated 239.0637.

Methyl-2-(benzo[d]thiazol-2-yl)-1-oxo-3-phenyl-1,2,3,4-tetrahydroisoquinoline-4-carboxylate **(23a)**

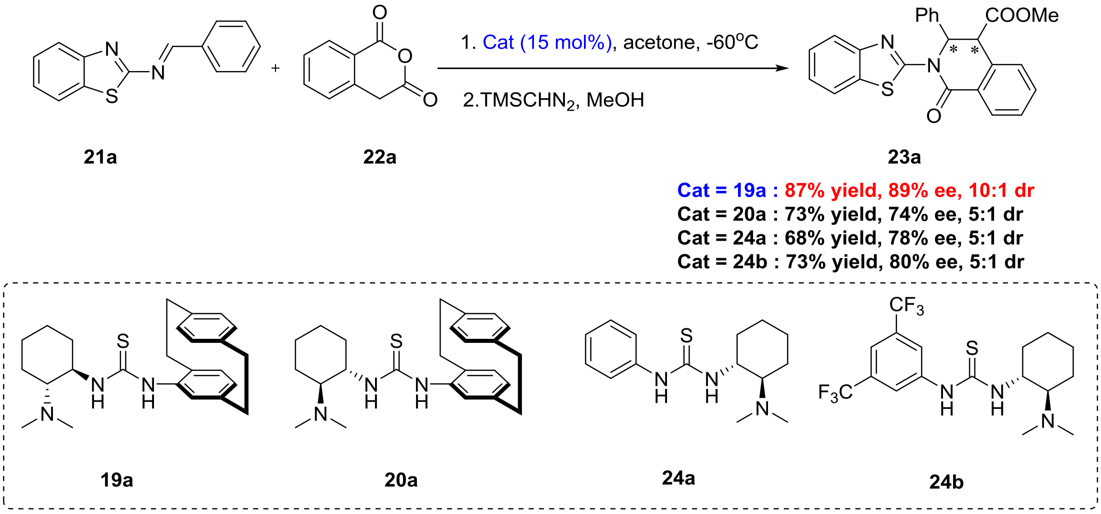

2-Benzothiazolamine **21a** (0.1 mmol), homophthalic anhydride **22a** (0.1 mmol), and catalyst (0.015 mmol) were added to a Schlenk tube. Then, acetone (1 mL) was added to the reaction mixture, which was allowed to stir at -60 °C for 16 h. After completion of the reaction as indicated by TLC analysis, a solution of $TMSCHN_2$ (0.1 mL) in MeOH (0.1 mL) was added, and the reaction mixture was allowed to stir for 30 min at -60 °C. Afterward, the mixture was concentrated under vacuum to give a residue, which was purified by column chromatography to afford the product **23a**. ^{1}H NMR (500 MHz, $CDCl_3$) δ 8.34 – 8.28 (m, 1H), 7.83 (d, *J* = 7.9 Hz, 1H), 7.79 (d, *J* = 8.1 Hz, 1H), 7.55 – 7.47 (m, 2H), 7.42 – 7.35 (m, 1H), 7.30 – 7.28 (m, 2H), 7.24 (s, 1H), 7.21 – 7.12 (m, 5H), 4.29 (s, 1H), 3.67 (s, 3H). ^{13}C NMR (101 MHz, $CDCl_3$) δ 170.7, 163.2, 158.5, 148.4, 138.3, 133.9, 133.1, 129.5, 129.1, 129.0, 128.8, 128.0, 127.9, 126.2, 125.9, 124.0, 121.9, 121.1, 59.9, 53.2, 50.6. m/z HRMS (ESI) found $[M+H]^+$ 415.1102, $C_{24}H_{19}N_2O_3S^+$ calculated 415.1111.

19a as organocatalyst:

$[\alpha]^{25}_{D}$ = 148.7 (c = 0.5, $CHCl_3$). HPLC: Chiralpak IA column, 70:30 hexanes/isopropanol, 1 ml/min; t_R = 11.55 min (minor), 14.24 min (major); 89% ee, 10:1 dr, 87% yield.

20a as organocatalyst:

$[\alpha]^{25}_{D}$ = -94.5 (c = 0.5, $CHCl_3$). HPLC: Chiralpak IA column, 70:30 hexanes/isopropanol, 1 ml/min; t_R = 11.66 min (major), 14.49 min (minor); 74% ee, 10:2 dr, 73% yield.

24a as organocatalyst:

$[\alpha]^{25}_{D}$ = 97.5 (c = 0.5, $CHCl_3$). HPLC: Chiralpak IA column, 70:30 hexanes/isopropanol, 1 ml/min; t_R = 10.21 min (minor), 12.81 min (major); 78% ee, 10:2 dr, 68% yield.

24b as organocatalyst:

$[\alpha]^{25}_{D}$ = 107.2 (c = 0.5, $CHCl_3$). HPLC: Chiralpak IA column, 70:30 hexanes/isopropanol, 1 ml/min; t_R = 10.14 min (minor), 12.78 min (major); 80% ee, 10:2 dr, 73% yield.

2 Supplementary Notes

2.1 X-Ray structures

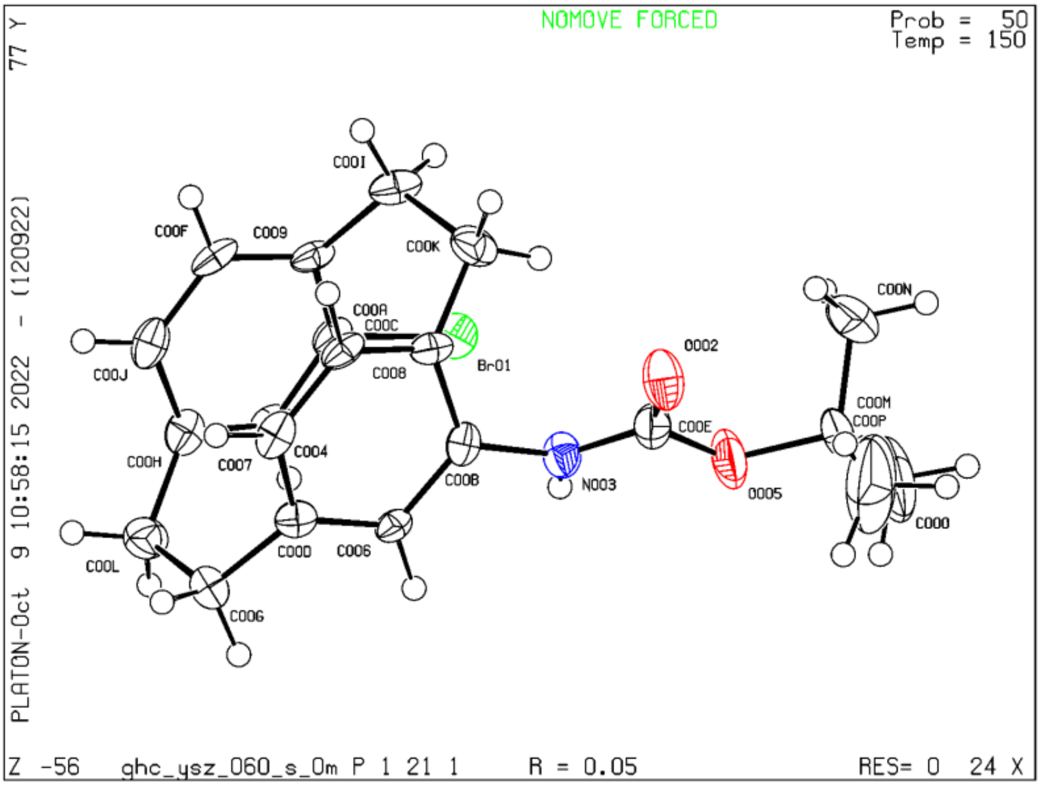

Supplementary Fig. 3 X-ray structure of (S_p)-**1f** (CCDC number 2248134)

Supplementary Table 2. Single crystal data of (S_p)-**1f**

Identification code	
Empirical formula	$C_{21}H_{24}BrNO_2$
Formula weight	402.32
Temperature/K	150.00
Crystal system	monoclinic
Space group	$P2_1$
a/Å	7.4131(3)
b/Å	10.8832(5)
c/Å	12.3016(5)
α/°	90
β/°	100.168(2)
γ/°	90
Volume/Å^3	976.88(7)
Z	2
ρ_{calc}g/cm^3	1.368
μ/mm^{-1}	1.955
F(000)	416.0
Crystal size/mm^3	0.1 × 0.1 × 0.1
Radiation	GaKα (λ = 1.34138)
2Θ range for data collection/°	6.35 to 108.166
Index ranges	-8 ≤ h ≤ 8, -13 ≤ k ≤ 13, -14 ≤ l ≤ 14
Reflections collected	19995
Independent reflections	3562 [R_{int} = 0.0830, R_{sigma} = 0.0780]
Data/restraints/parameters	3562/1/233
Goodness-of-fit on F^2	1.095
Final R indexes [I>=2σ (I)]	R_1 = 0.0471, wR_2 = 0.0892
Final R indexes [all data]	R_1 = 0.0718, wR_2 = 0.0969
Largest diff. peak/hole / e Å^{-3}	0.49/-0.49
Flack parameter	0.075(17)

2.2 HPLC traces

(R_p)-Tert-butyl 1,4(1,4)-dibenzenacyclohexaphane-1^2-ylcarbamate **(1a)**

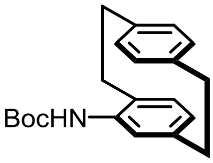

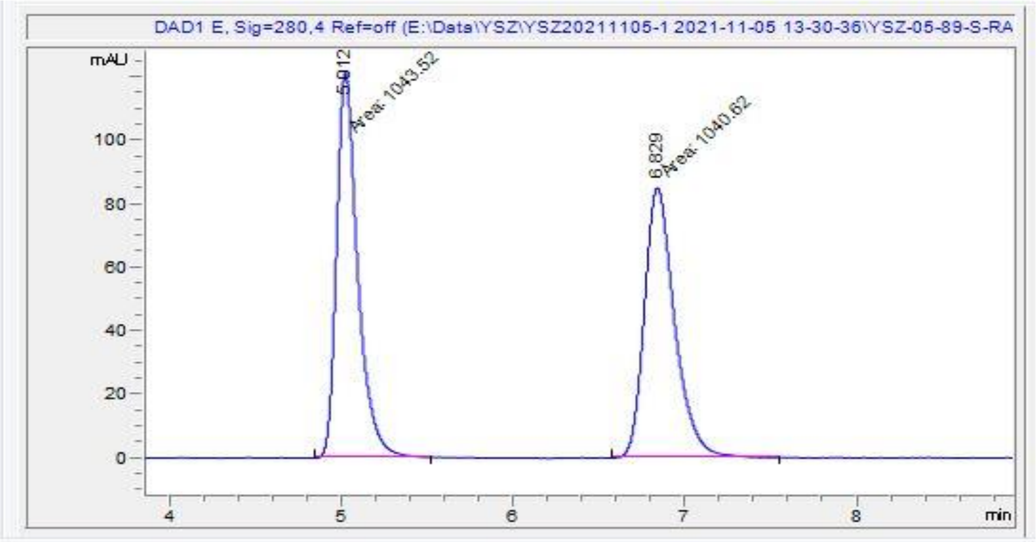

#	Time	Type	Area	Height	Width	Area%	Symmetry
1	5.012	MM	1043.5	121.6	0.143	50.069	0.67
2	6.829	MM	1040.6	85.3	0.2033	49.931	0.728

Supplementary Fig. 4 HPLC spectrum of racemic **1a**

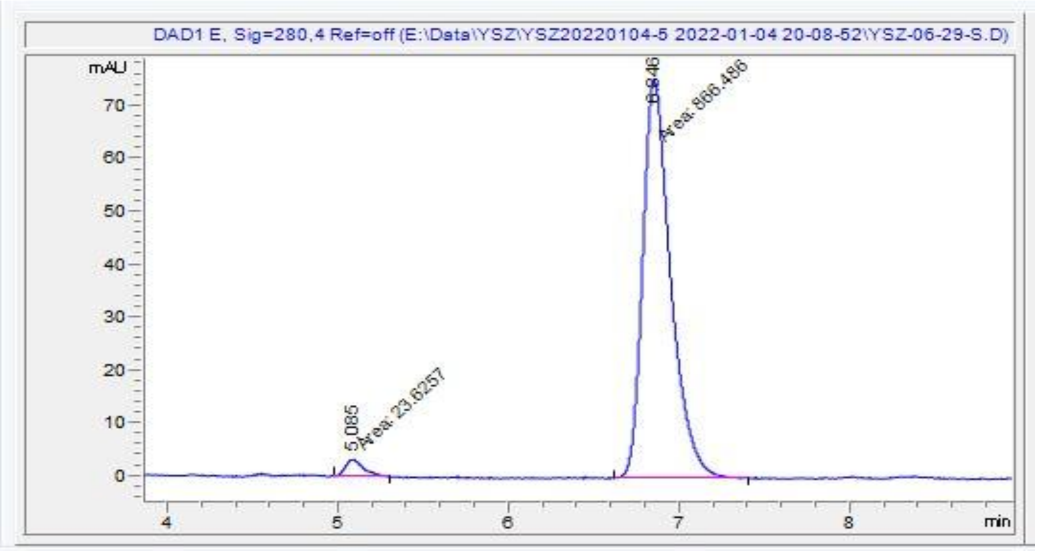

#	Time	Type	Area	Height	Width	Area%	Symmetry
1	5.085	MM	23.6	3.3	0.1209	2.654	0.748
2	6.846	MM	866.5	75.6	0.1911	97.346	0.671

Supplementary Fig. 5 HPLC spectrum of (R_p)**-1a**

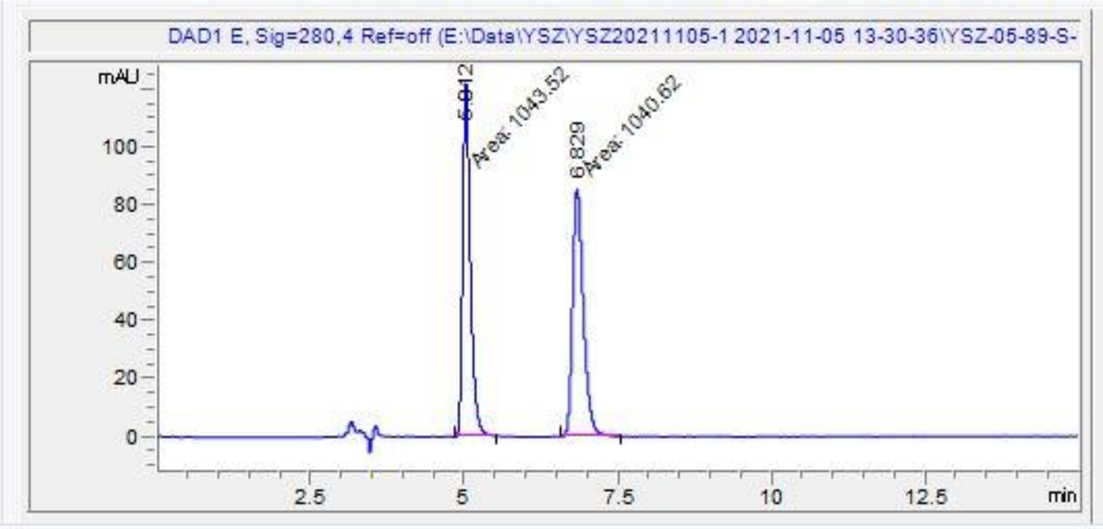

#	Time	Type	Area	Height	Width	Area%	Symmetry
1	5.012	MM	1043.5	121.6	0.143	50.069	0.67
2	6.829	MM	1040.6	85.3	0.2033	49.931	0.728

Supplementary Fig. 6 Full HPLC spectrum of spectrum of racemic **1a**

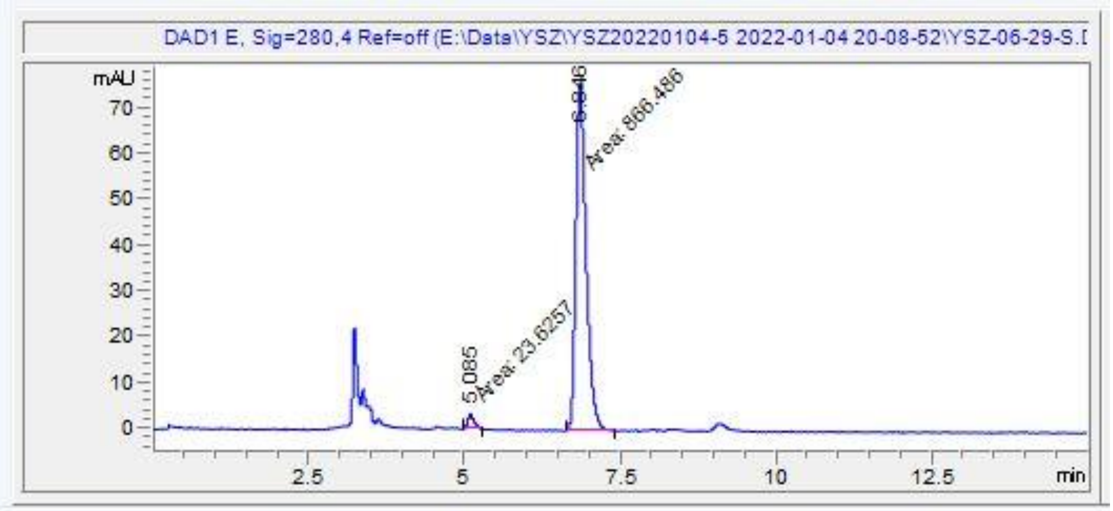

#	Time	Type	Area	Height	Width	Area%	Symmetry
1	5.085	MM	23.6	3.3	0.1209	2.654	0.748
2	6.846	MM	866.5	75.6	0.1911	97.346	0.671

Supplementary Fig. 7 Full HPLC spectrum of (R_p)-**1a**

(S_p)-Dibenzyl-1-(1^5-((tert-butoxycarbonyl)amino)-1,4(1,4)-dibenzenacyclohexaphane-1^2-yl)hydrazine-1,2-dicarboxylate **(3a)**

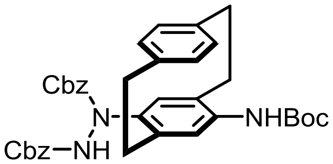

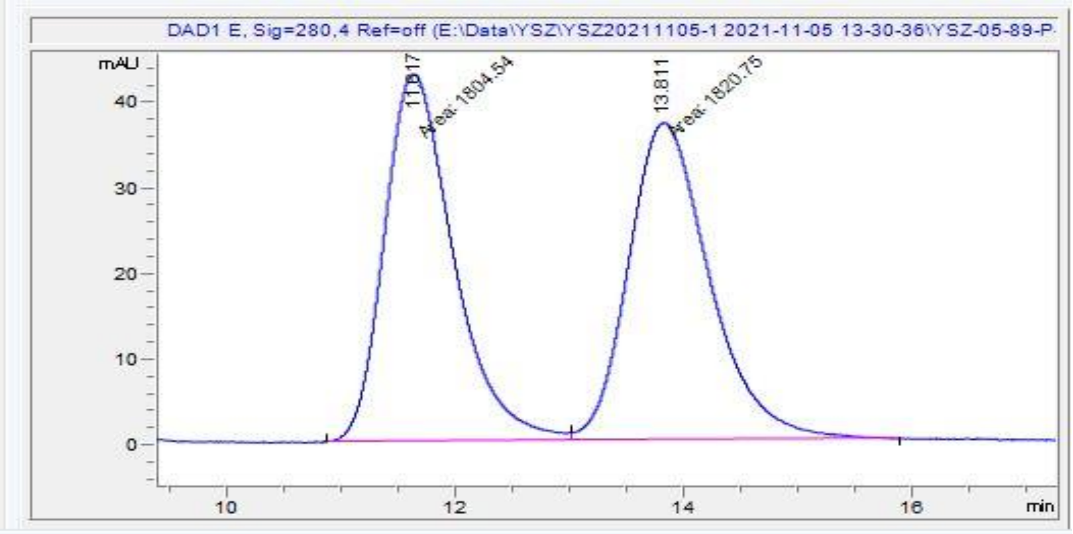

#	Time	Type	Area	Height	Width	Area%	Symmetry
1	11.617	MF	1804.5	42.8	0.7026	49.776	0.675
2	13.811	FM	1820.8	36.9	0.8213	50.224	0.717

Supplementary Fig. 8 HPLC spectrum of racemic **1a**

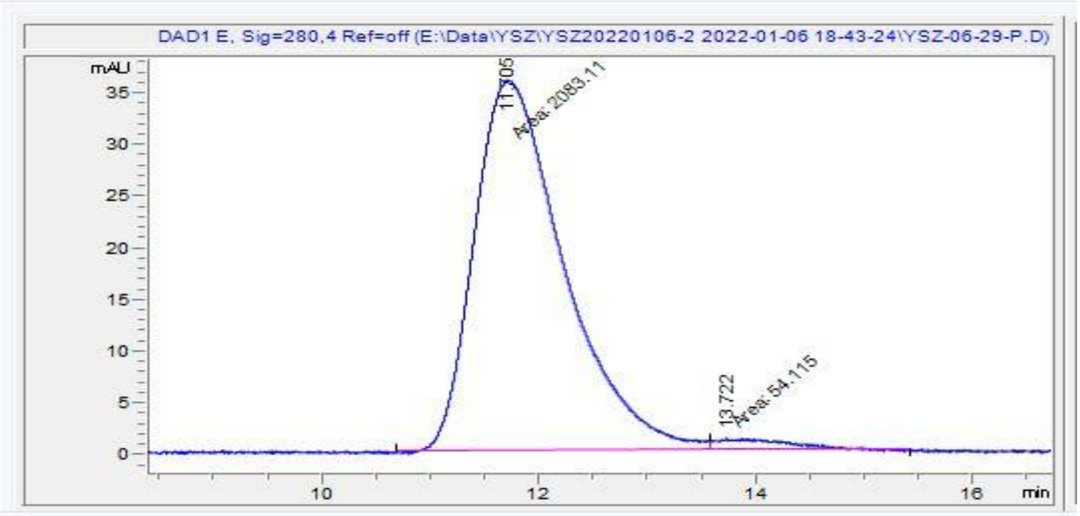

#	Time	Type	Area	Height	Width	Area%	Symmetry
1	11.705	MF	2083.1	36.1	0.9619	97.468	0.571
2	13.722	FM	54.1	1.3	0.7131	2.532	0.168

Supplementary Fig. 9 HPLC spectrum of (S_p)-**3a**

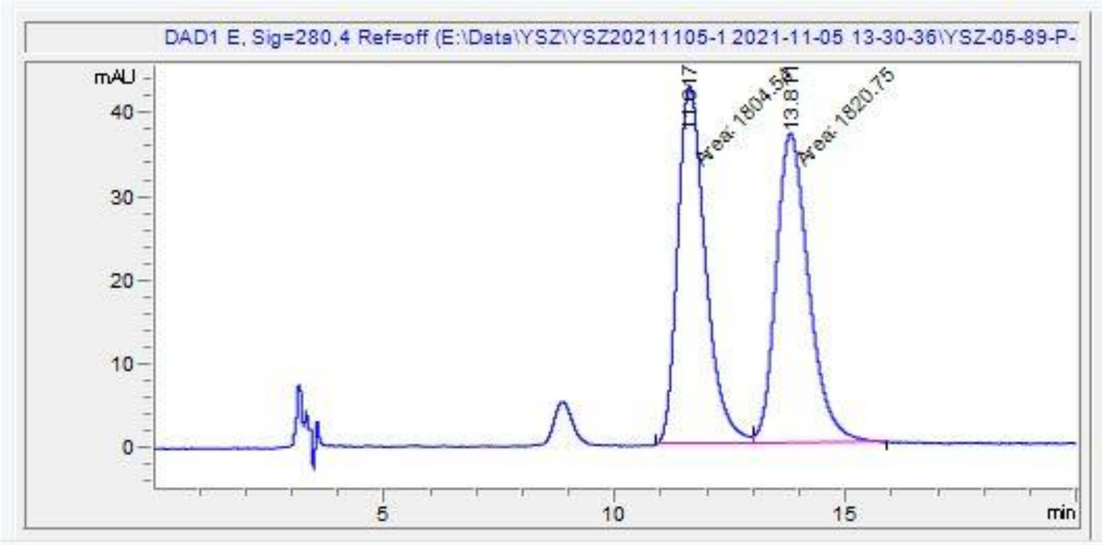

#	Time	Type	Area	Height	Width	Area%	Symmetry
1	11.617	MF	1804.5	42.8	0.7026	49.776	0.675
2	13.811	FM	1820.8	36.9	0.8213	50.224	0.717

Supplementary Fig. 10 Full HPLC spectrum of racemic **3a**

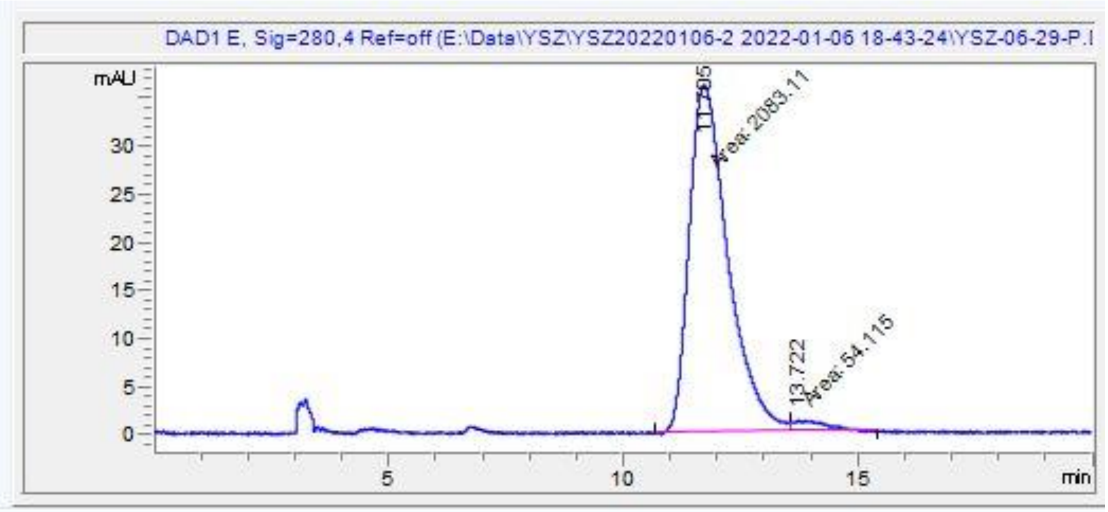

#	Time	Type	Area	Height	Width	Area%	Symmetry
1	11.705	MF	2083.1	36.1	0.9619	97.468	0.571
2	13.722	FM	54.1	1.3	0.7131	2.532	0.168

Supplementary Fig. 11 Full HPLC spectrum of (S_p)-**3a**

(R_p)-Methyl 1,4(1,4)-dibenzenacyclohexaphane-1^2-ylcarbamate **(1b)**

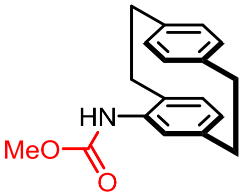

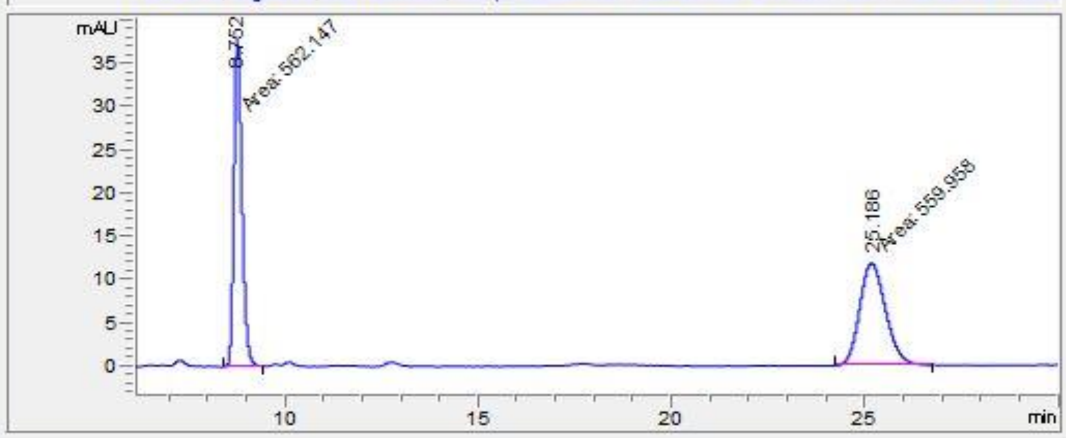

#	Time	Type	Area	Height	Width	Area%	Symmetry
1	8.752	MM	562.1	38.3	0.2444	50.098	0.773
2	25.186	MM	560	12	0.7808	49.902	0.77

Supplementary Fig. 12 HPLC spectrum of racemic **1b**

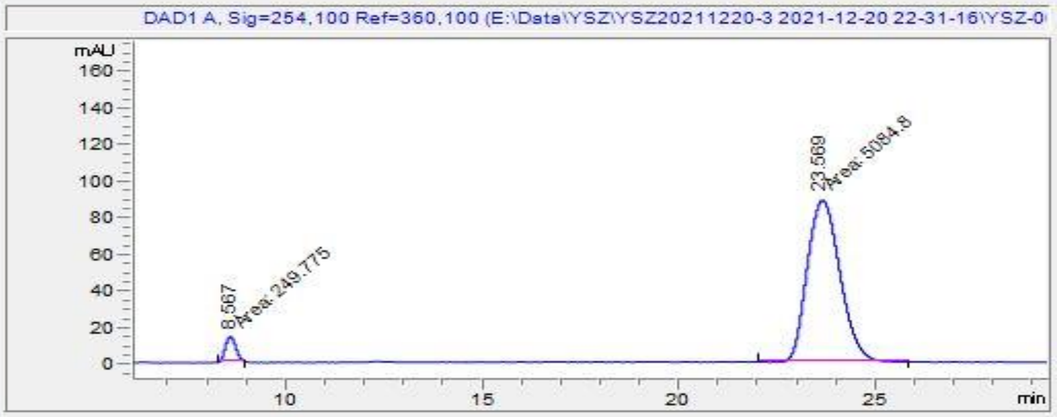

#	Time	Type	Area	Height	Width	Area%	Symmetry
1	8.567	MM	249.8	13.7	0.3035	4.682	0.966
2	23.569	MM	5084.8	88.4	0.9584	95.318	0.791

Supplementary Fig. 13 HPLC spectrum of (R_p)-**1b**

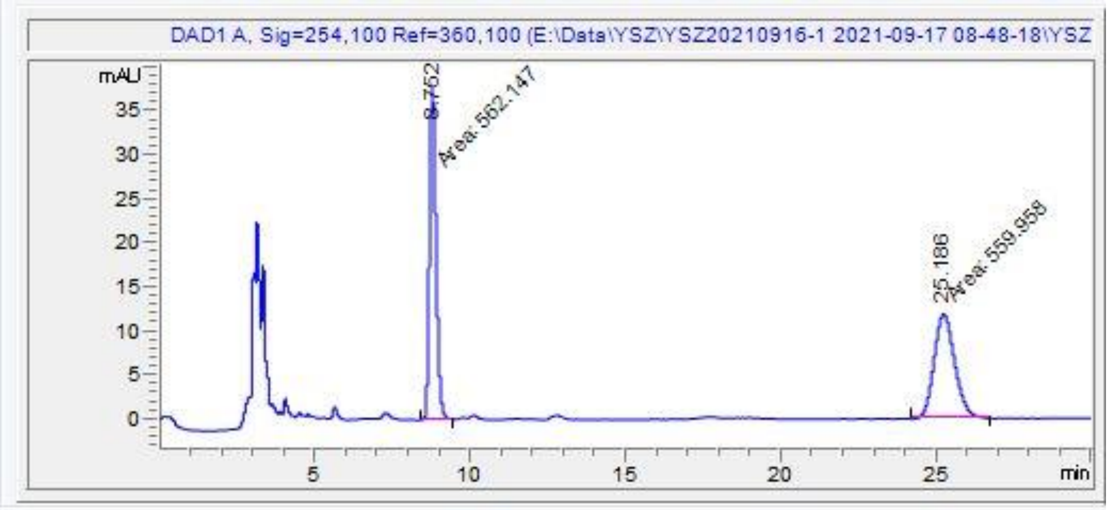

#	Time	Type	Area	Height	Width	Area%	Symmetry
1	8.752	MM	562.1	38.3	0.2444	50.098	0.773
2	25.186	MM	560	12	0.7808	49.902	0.77

Supplementary Fig. 14 Full HPLC spectrum of racemic **1b**

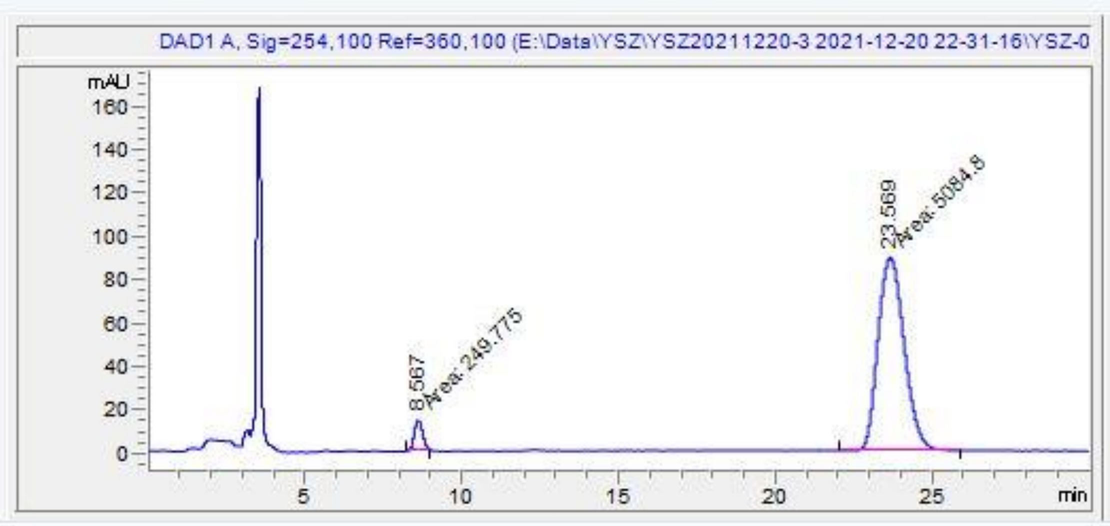

#	Time	Type	Area	Height	Width	Area%	Symmetry
1	8.567	MM	249.8	13.7	0.3035	4.682	0.966
2	23.569	MM	5084.8	88.4	0.9584	95.318	0.791

Supplementary Fig. 15 Full HPLC spectrum of (R_p)-**1b**

(S_p)-Dibenzyl-1-(1^5-((methoxycarbonyl)amino)-1,4(1,4)-dibenzenacyclohexaphane-1^2-yl)hydrazine-1,2-dicarboxylate **(3b)**

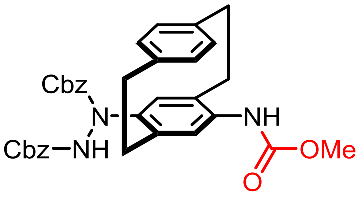

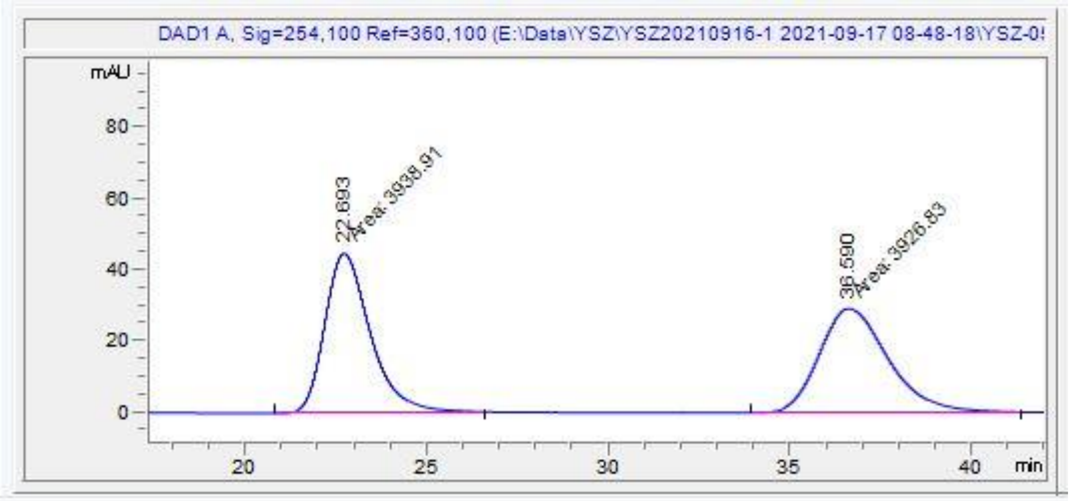

#	Time	Type	Area	Height	Width	Area%	Symmetry
1	22.693	MM	3938.9	45	1.459	50.077	0.689
2	36.59	MM	3926.8	29.5	2.2215	49.923	0.734

Supplementary Fig. 16 HPLC spectrum of racemic **3b**

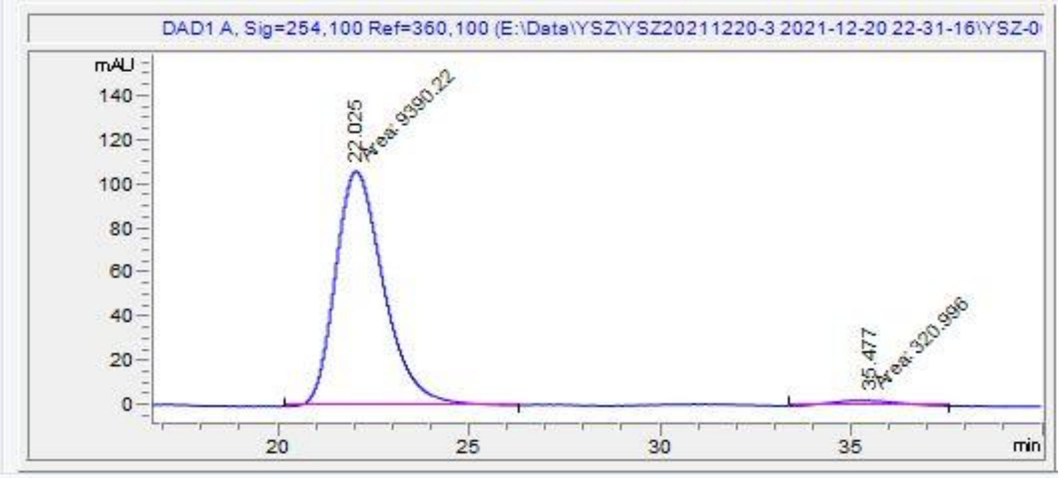

#	Time	Type	Area	Height	Width	Area%	Symmetry
1	22.025	MM	9390.2	105.8	1.479	96.695	0.688
2	35.477	MM	321	2.7	1.9881	3.305	1.239

Supplementary Fig. 17 HPLC spectrum of (S_p)-**3b**

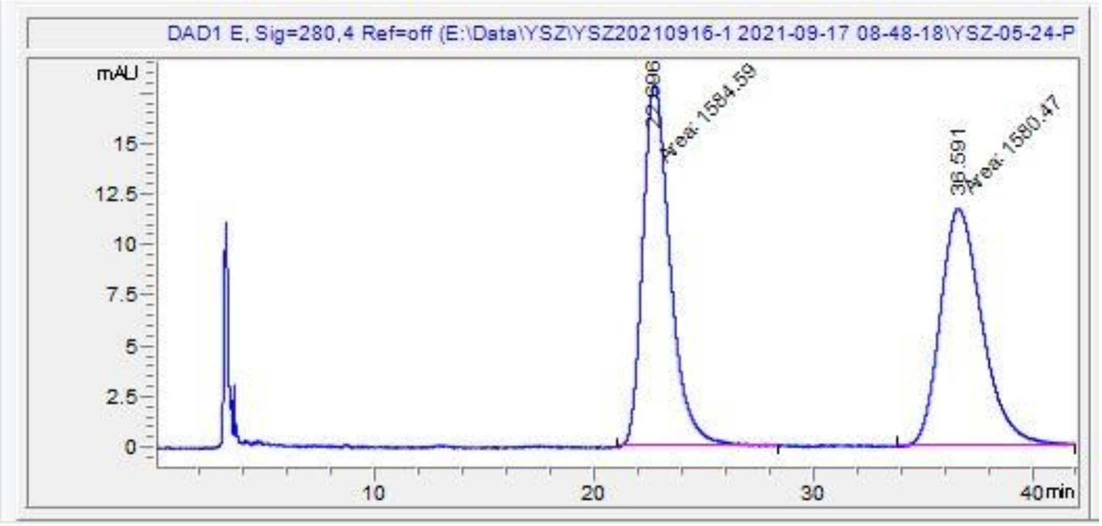

#	Time	Type	Area	Height	Width	Area%	Symmetry
1	22.696	MM	1584.6	18.1	1.4586	50.065	0.692
2	36.591	MM	1580.5	11.9	2.2228	49.935	0.727

Supplementary Fig. 18 Full HPLC spectrum of racemic **3b**

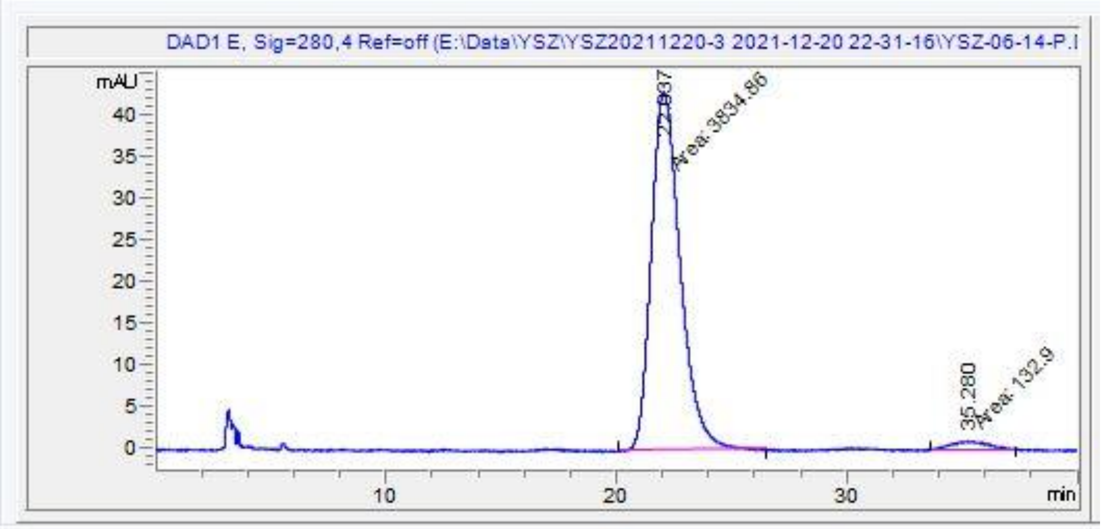

#	Time	Type	Area	Height	Width	Area%	Symmetry
1	22.037	MM	3834.9	43.6	1.4663	96.650	0.717
2	35.28	MM	132.9	1.2	1.8447	3.350	0.893

Supplementary Fig. 19 Full HPLC spectrum of (S_p)-**3b**

(R_p)-Ethyl 1,4(1,4)-dibenzenacyclohexaphane-1^2-ylcarbamate **(1c)**

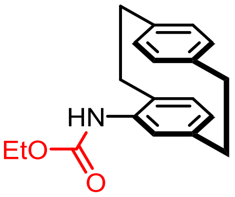

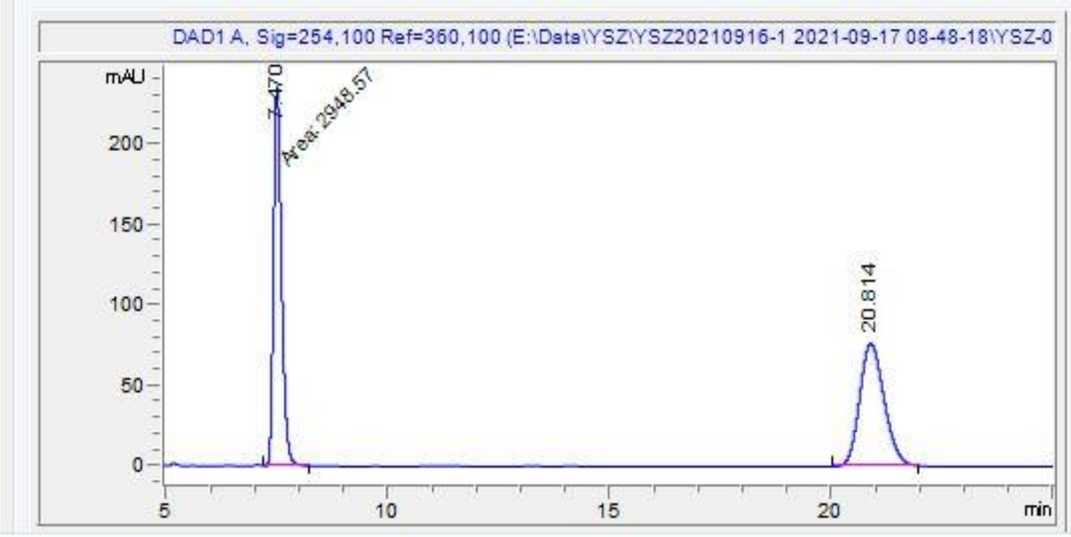

#	Time	Type	Area	Height	Width	Area%	Symmetry
1	7.47	MM	2948.6	236.9	0.2074	50.042	0.719
2	20.814	BB	2943.6	76.7	0.45	49.958	0.79

Supplementary Fig. 20 HPLC spectrum of racemic **1c**

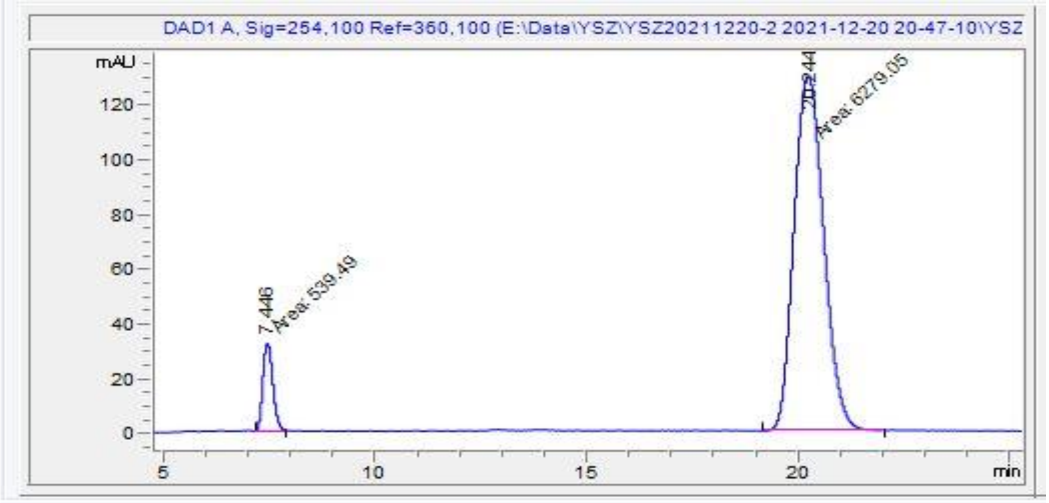

#	Time	Type	Area	Height	Width	Area%	Symmetry
1	7.446	MM	539.5	32.3	0.2787	7.912	0.857
2	20.244	MM	6279	129	0.8111	92.088	0.894

Supplementary Fig. 21 HPLC spectrum of (R_p)-**1c**

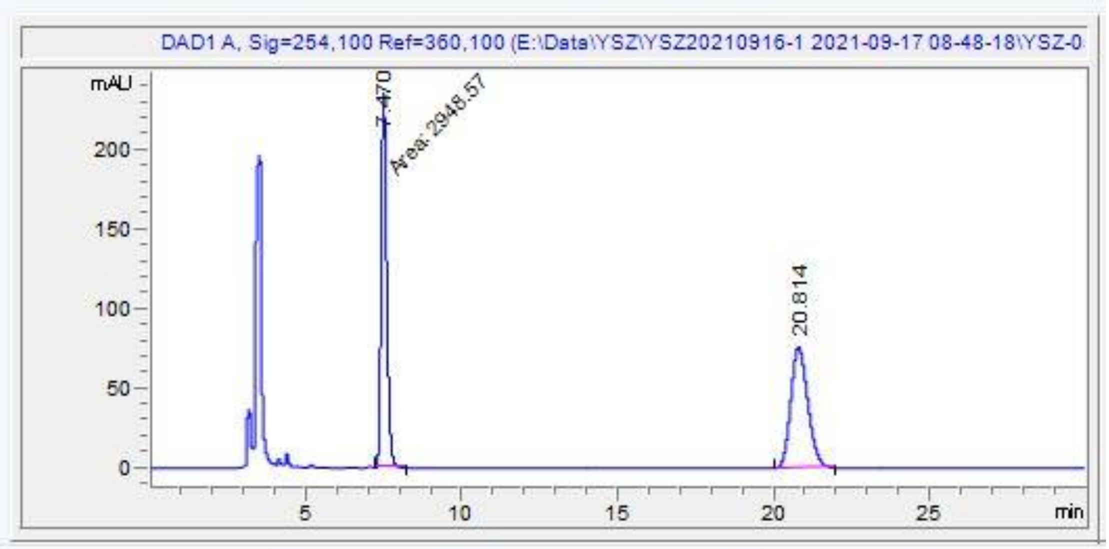

#	Time	Type	Area	Height	Width	Area%	Symmetry
1	7.47	MM	2948.6	236.9	0.2074	50.042	0.719
2	20.814	BB	2943.6	76.7	0.45	49.958	0.79

Supplementary Fig. 22 Full HPLC spectrum of racemic **1c**

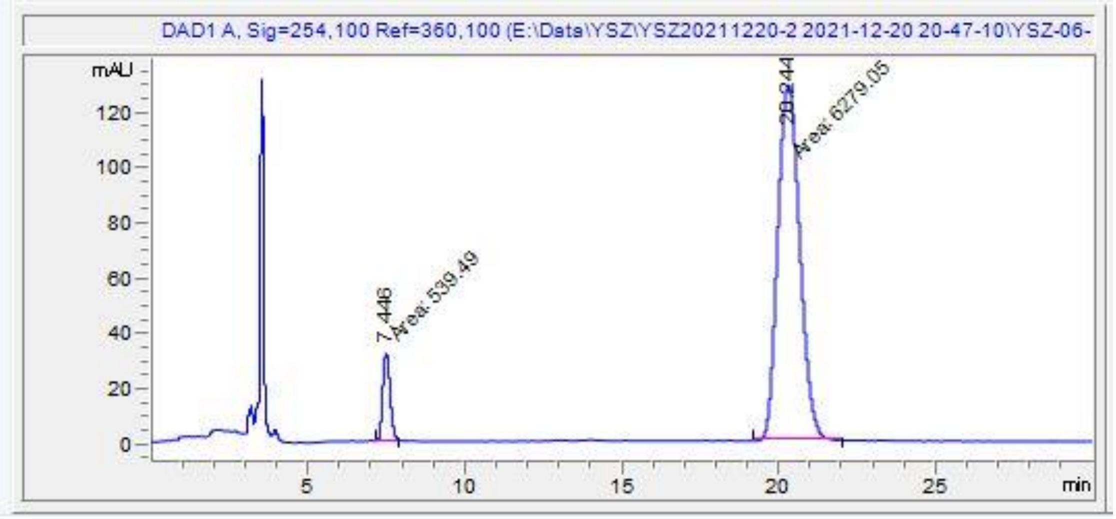

#	Time	Type	Area	Height	Width	Area%	Symmetry
1	7.446	MM	539.5	32.3	0.2787	7.912	0.857
2	20.244	MM	6279	129	0.8111	92.088	0.894

Supplementary Fig. 23 Full HPLC spectrum of (R_p)**-1c**

(S_p)-Dibenzyl 1-(1^5-((ethoxycarbonyl)amino)-1,4(1,4)-dibenzenacyclohexaphane-1^2-yl)hydrazine-1,2-dicarboxylate **(3c)**

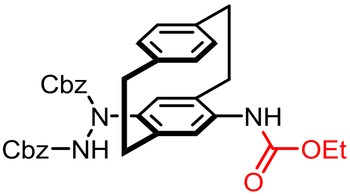

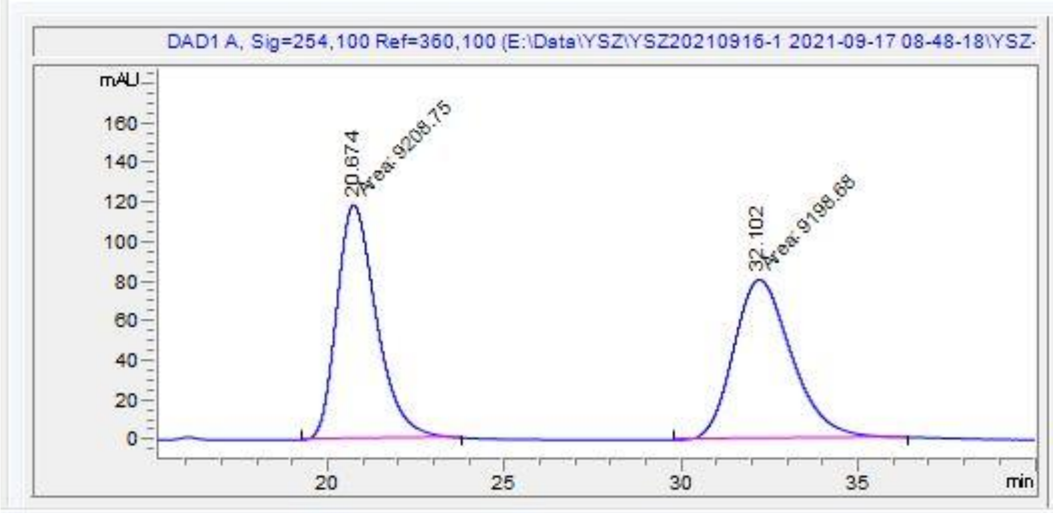

#	Time	Type	Area	Height	Width	Area%	Symmetry
1	20.674	MM	9208.7	117.9	1.3017	50.027	0.688
2	32.102	MM	9198.7	80.3	1.9081	49.973	0.741

Supplementary Fig. 24 HPLC spectrum of racemic **3c**

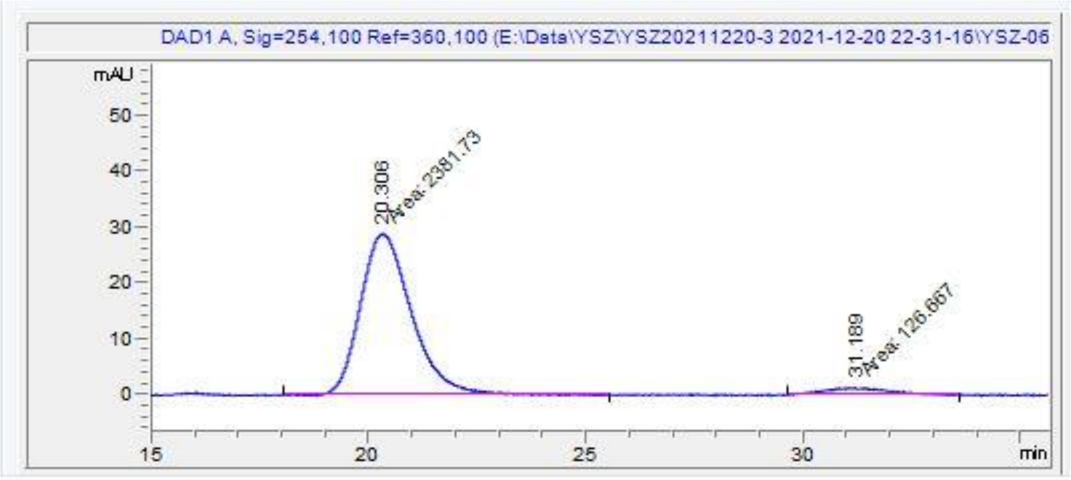

#	Time	Type	Area	Height	Width	Area%	Symmetry
1	20.306	MM	2381.7	29	1.3709	94.950	0.716
2	31.189	MM	126.7	1.3	1.5784	5.050	0.827

Supplementary Fig. 25 HPLC spectrum of (S_p)-**3c**

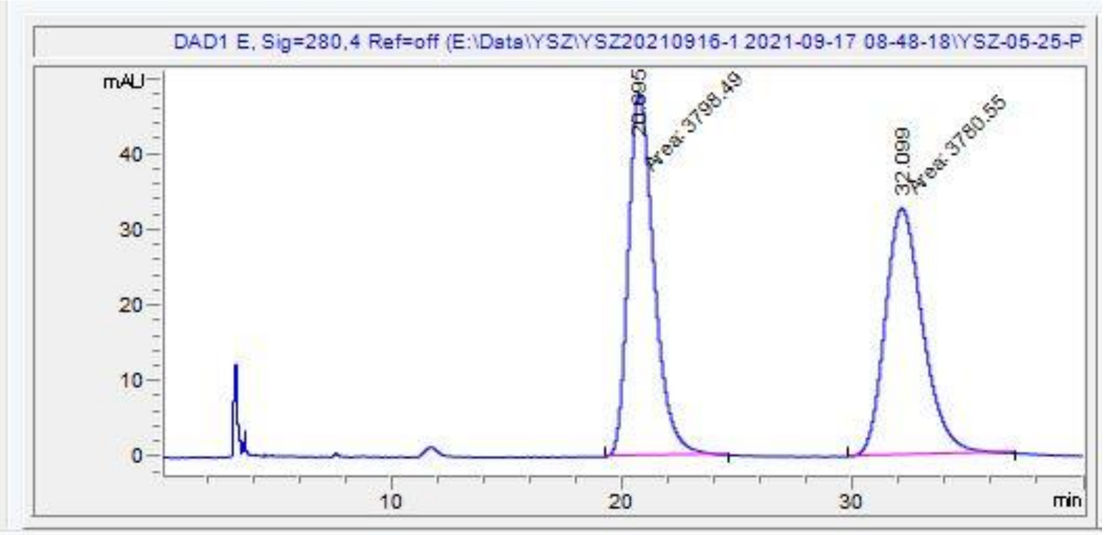

#	Time	Type	Area	Height	Width	Area%	Symmetry
1	20.695	MM	3798.5	48.5	1.3058	50.118	0.726
2	32.099	MM	3780.5	33	1.9077	49.882	0.73

Supplementary Fig. 26 Full HPLC spectrum of racemic **3c**

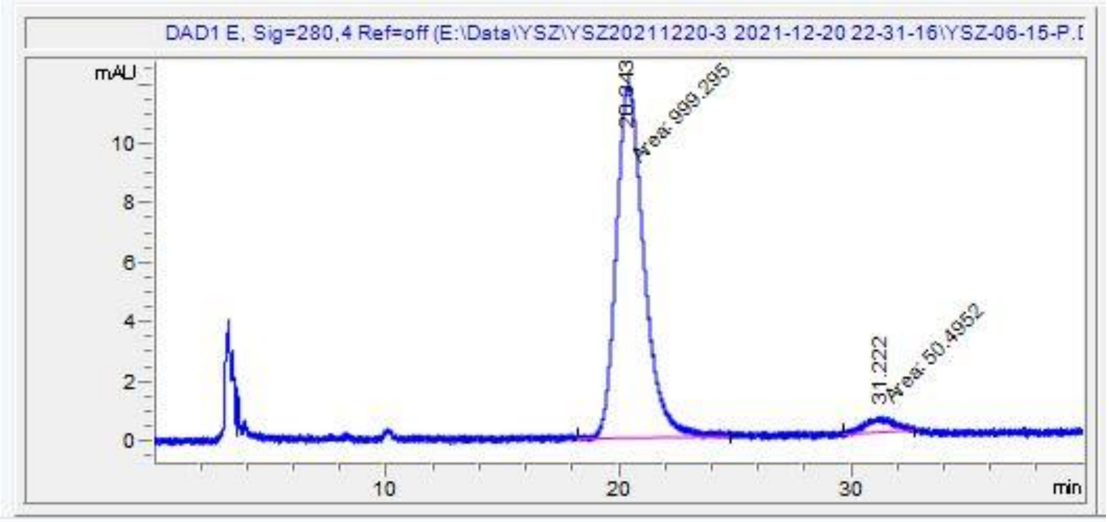

#	Time	Type	Area	Height	Width	Area%	Symmetry
1	20.343	MM	999.3	12	1.3831	95.190	0.799
2	31.222	MM	50.5	6E-1	1.4088	4.810	1.172

Supplementary Fig. 27 Full HPLC spectrum of (S_p)-**3c**

(R_p)-Benzyl 1,4(1,4)-dibenzenacyclohexaphane-1^2-ylcarbamate **(1d)**

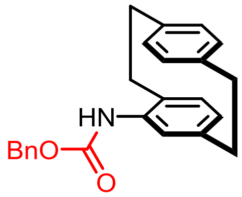

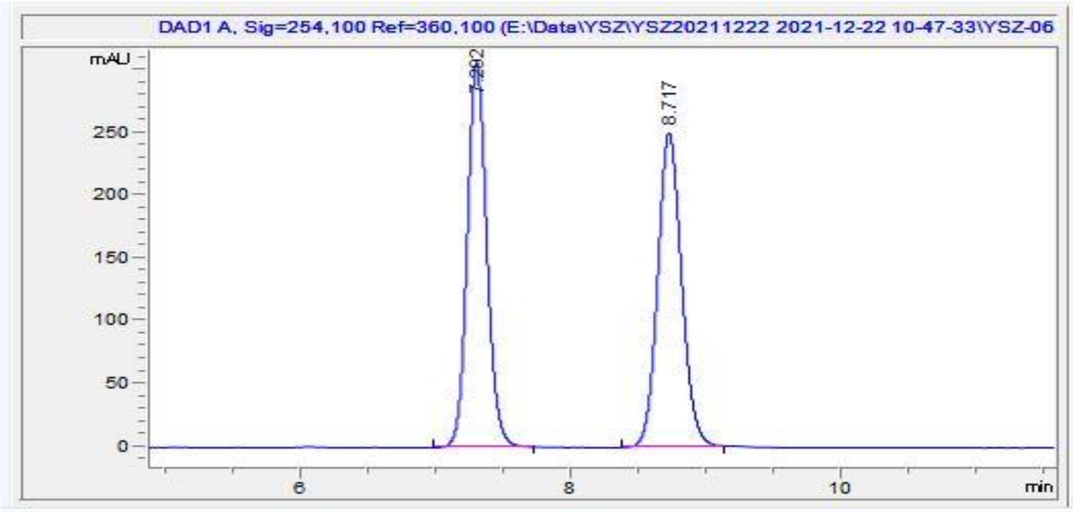

#	Time	Type	Area	Height	Width	Area%	Symmetry
1	7.292	BV R	3073.5	306.5	0.121	50.040	0.829
2	8.717	BB	3068.7	250	0.1482	49.960	0.804

Supplementary Fig. 28 HPLC spectrum of racemic **1d**

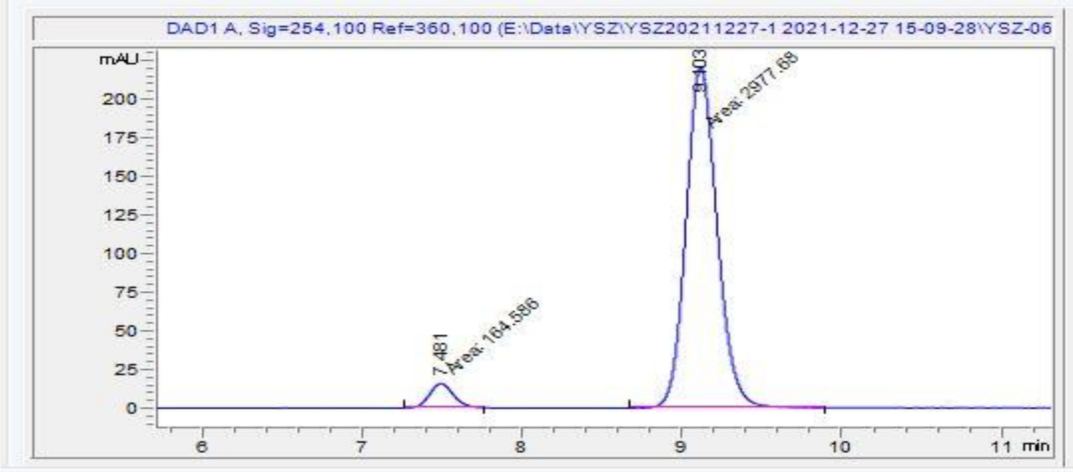

#	Time	Type	Area	Height	Width	Area%	Symmetry
1	7.481	MM	164.6	15.7	0.1751	5.238	0.928
2	9.103	MM	2977.7	220	0.2256	94.762	0.897

Supplementary Fig. 29 HPLC spectrum of (R_p)-**1d**

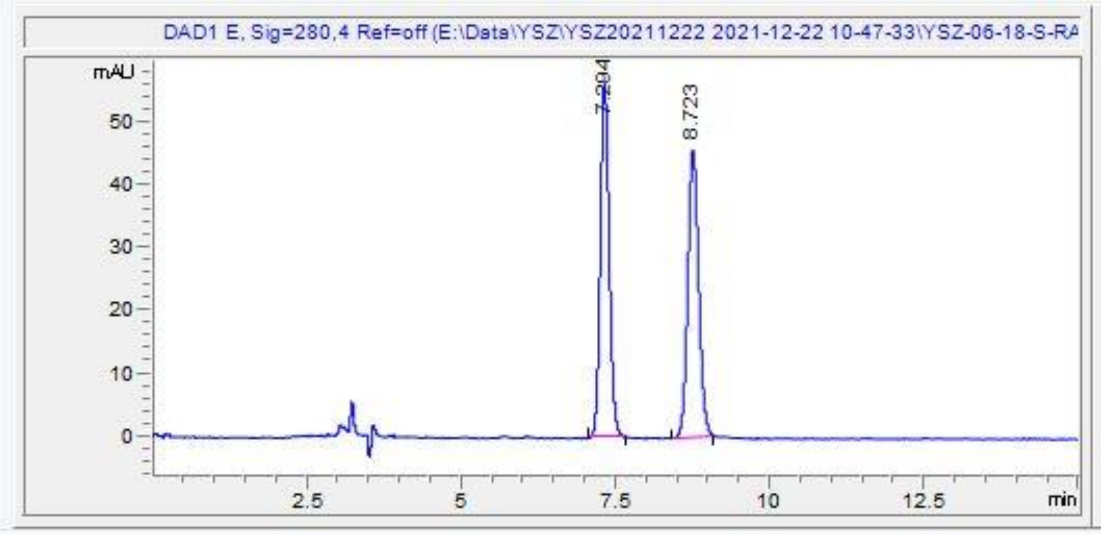

#	Time	Type	Area	Height	Width	Area%	Symmetry
1	7.294	BV R	564.5	56.9	0.1293	50.271	0.878
2	8.723	BV R	558.4	45.8	0.1476	49.729	0.923

Supplementary Fig. 30 Full HPLC spectrum of racemic **1d**

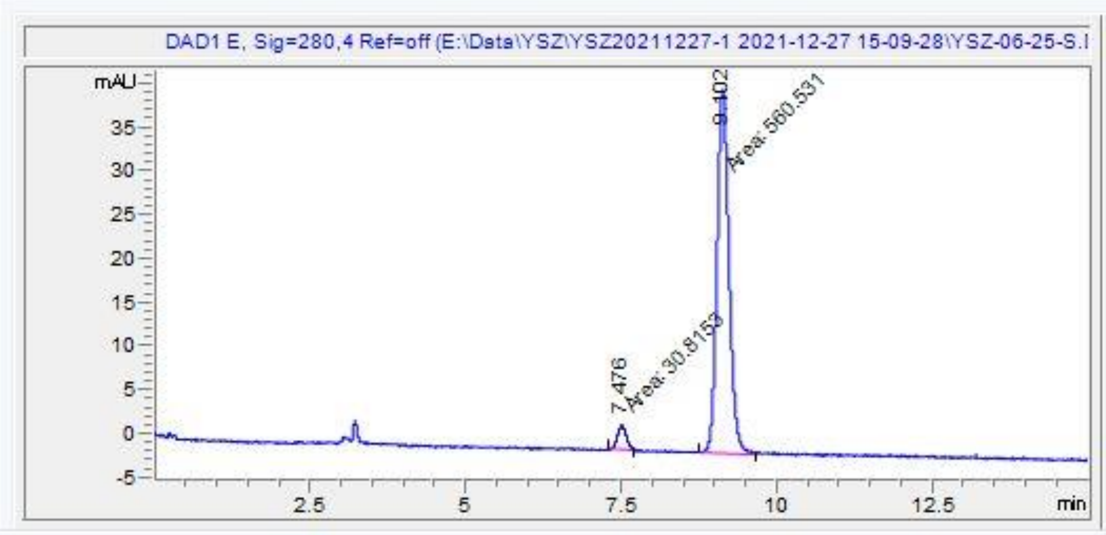

#	Time	Type	Area	Height	Width	Area%	Symmetry
1	7.476	MM	30.8	3	0.1735	5.211	0.692
2	9.102	MM	560.5	41.6	0.2244	94.789	0.856

Supplementary Fig. 31 Full HPLC spectrum of (R_p)-**1d**

(S_p)-Dibenzyl-1-(1[5]-(((benzyloxy)carbonyl)amino)-1,4(1,4)-dibenzenacyclohexaphane-1[2]-yl)hydrazine-1,2-dicarboxylate **(3d)**

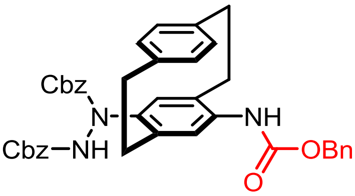

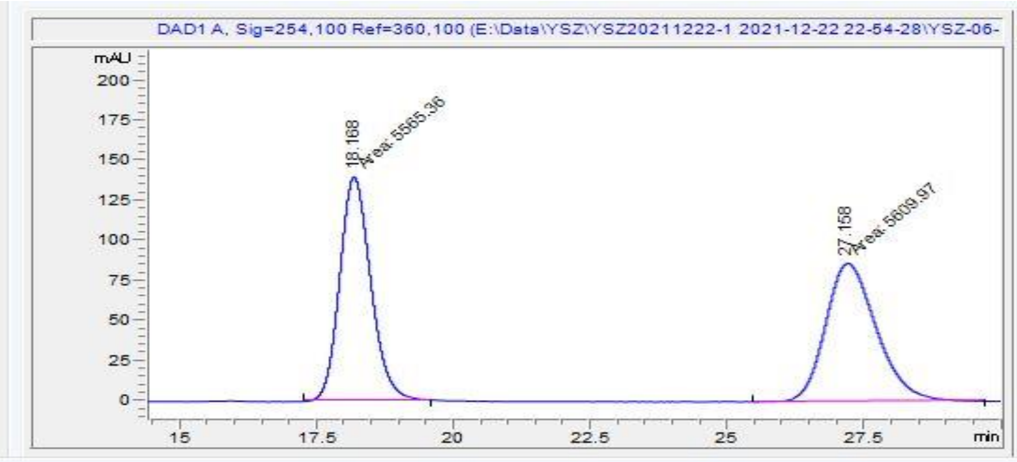

#	Time	Type	Area	Height	Width	Area%	Symmetry
1	18.168	MM	5565.4	140.5	0.66	49.800	0.837
2	27.158	MM	5610	86.7	1.078	50.200	0.719

Supplementary Fig. 32 HPLC spectrum of racemic **3d**

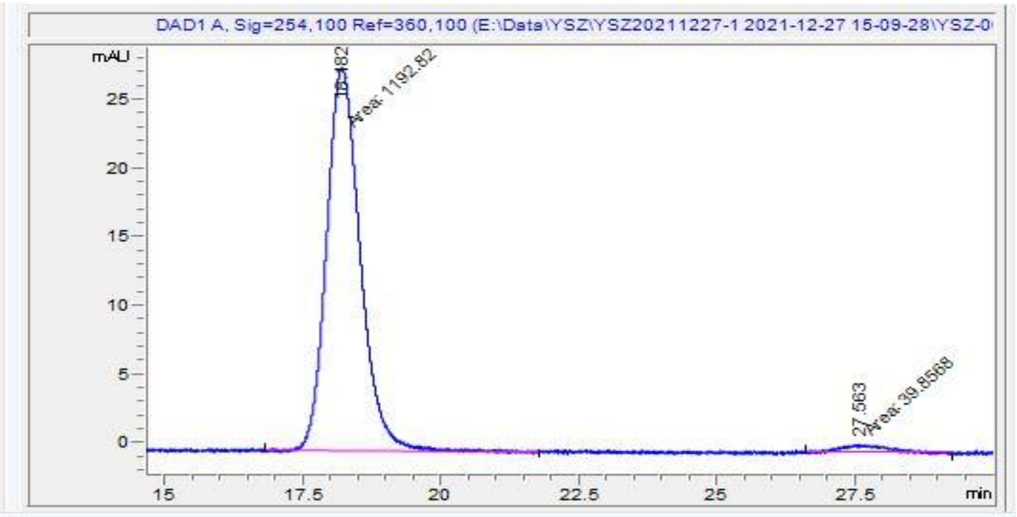

#	Time	Type	Area	Height	Width	Area%	Symmetry
1	18.182	MM	1192.8	28	0.7108	96.767	0.737
2	27.563	MM	39.9	6.5E-1	1.0179	3.233	0.682

Supplementary Fig. 33 HPLC spectrum of (S_p)-**3d**

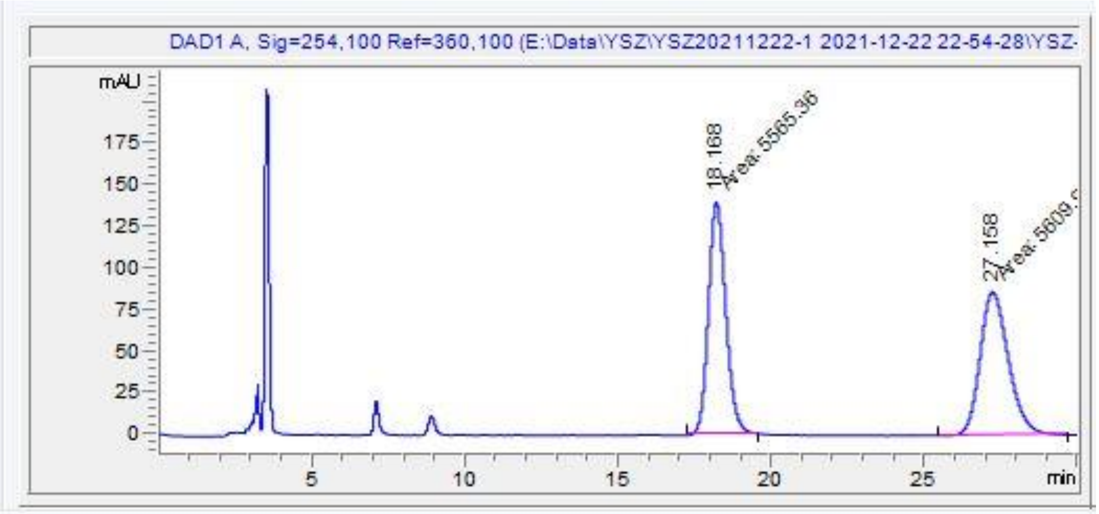

#	Time	Type	Area	Height	Width	Area%	Symmetry
1	18.168	MM	5565.4	140.5	0.66	49.800	0.837
2	27.158	MM	5610	86.7	1.078	50.200	0.719

Supplementary Fig. 34 Full HPLC spectrum of racemic **3d**

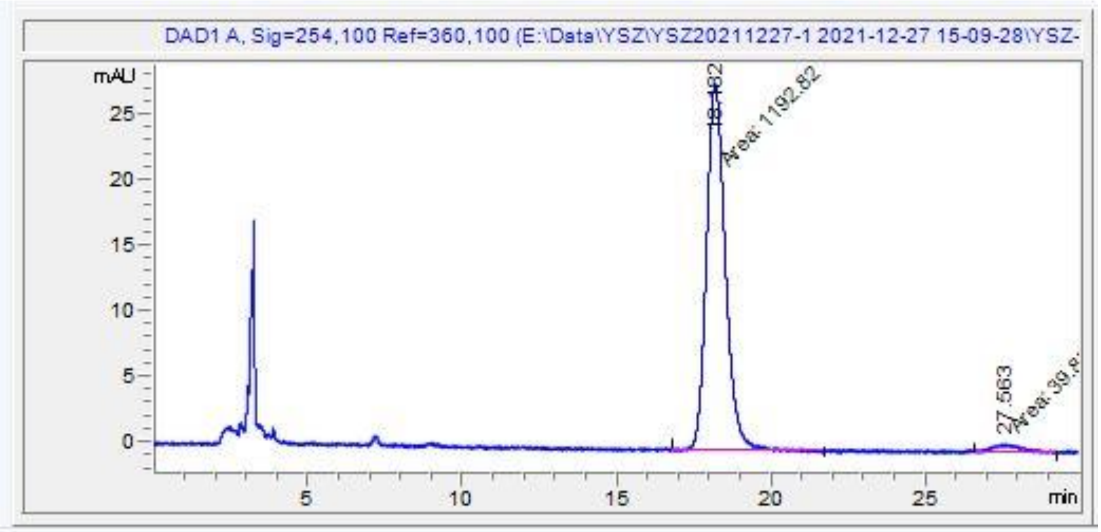

#	Time	Type	Area	Height	Width	Area%	Symmetry
1	18.182	MM	1192.8	28	0.7108	96.767	0.737
2	27.563	MM	39.9	6.5E-1	1.0179	3.233	0.682

Supplementary Fig. 35 Full HPLC spectrum of (S_p)-**3d**

(R_p)- (9H-fluoren-9-yl)methyl 1,4(1,4)-dibenzenacyclohexaphane-1^2-ylcarbamate **(1e)**

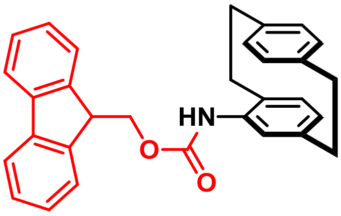

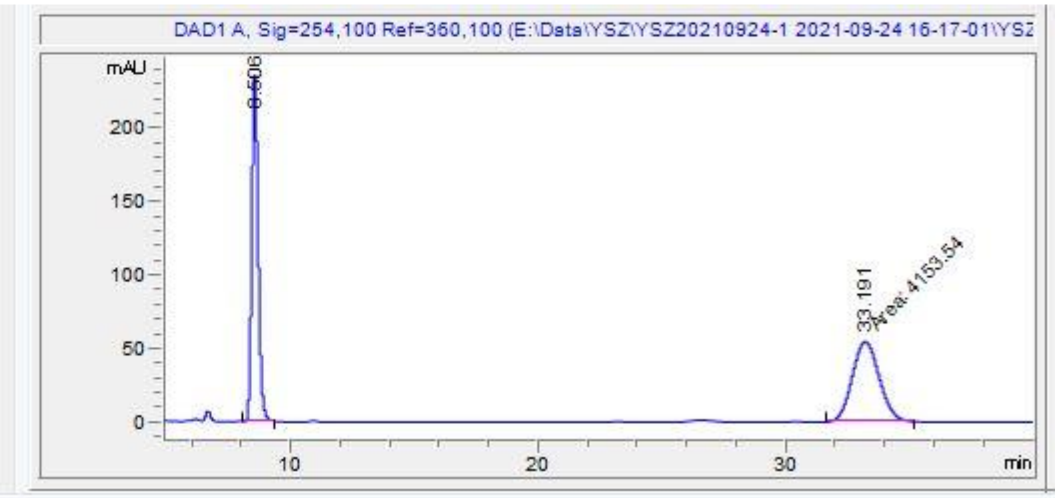

#	Time	Type	Area	Height	Width	Area%	Symmetry
1	8.506	BB	4186	235.8	0.2411	50.194	0.815
2	33.191	MM	4153.5	54.6	1.2679	49.806	0.926

Supplementary Fig. 36 HPLC spectrum of racemic **1e**

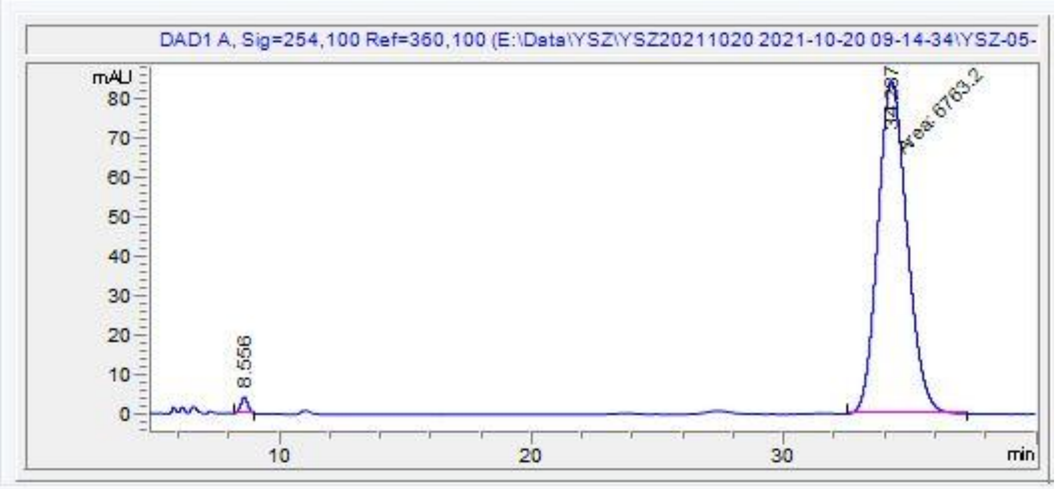

#	Time	Type	Area	Height	Width	Area%	Symmetry
1	8.556	BB	84.6	4.2	0.2348	1.235	0.875
2	34.287	MM	6763.2	83.6	1.3485	98.765	0.834

Supplementary Fig. 37 HPLC spectrum of (R_p)-**1e**

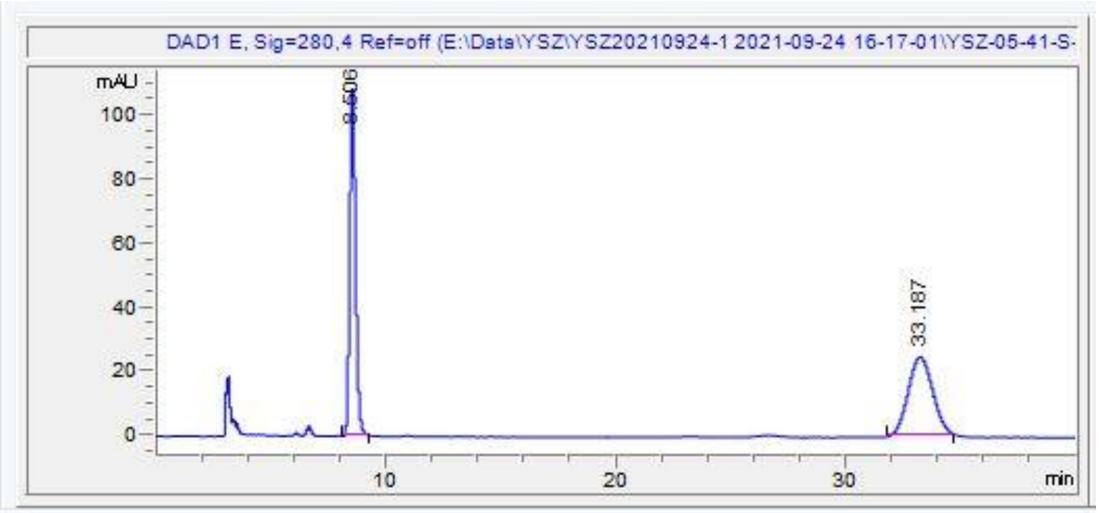

#	Time	Type	Area	Height	Width	Area%	Symmetry
1	8.506	BB	1920.1	108.7	0.2442	51.284	0.813
2	33.187	BV R	1823.9	24.6	0.8655	48.716	0.943

Supplementary Fig. 38 Full HPLC spectrum of racemic **1e**

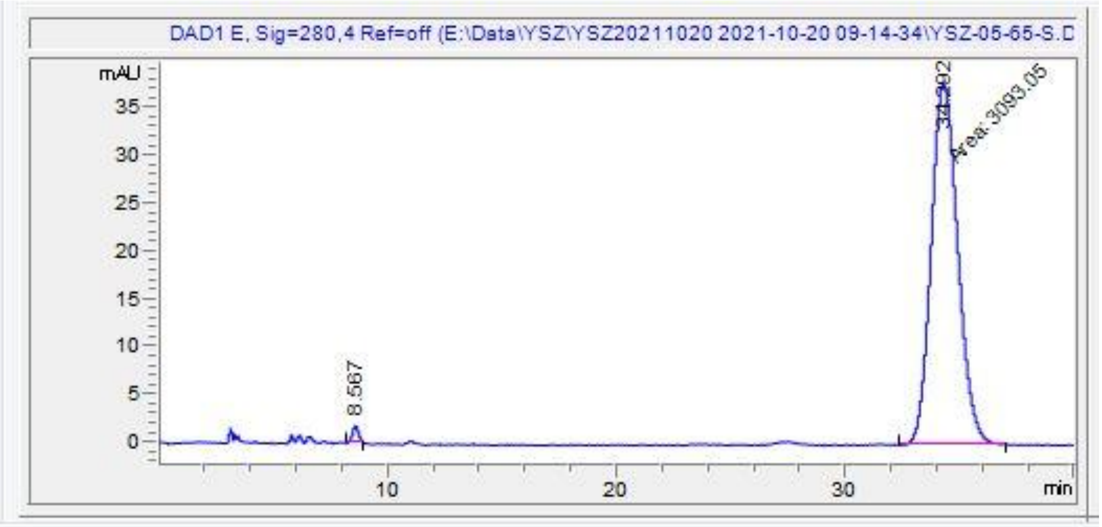

#	Time	Type	Area	Height	Width	Area%	Symmetry
1	8.567	VV R	36.1	1.9	0.2265	1.155	1.017
2	34.292	MM	3093.1	38.1	1.3531	98.845	0.816

Supplementary Fig. 39 Full HPLC spectrum of (R_p)-**1e**

(S_p)-Dibenzyl 1-(1^5-((((9H-fluoren-9-yl)methoxy)carbonyl)amino)-1,4(1,4)-dibenzenacyclohexaphane-1^2-yl)hydrazine-1,2-dicarboxylate **(3e)**

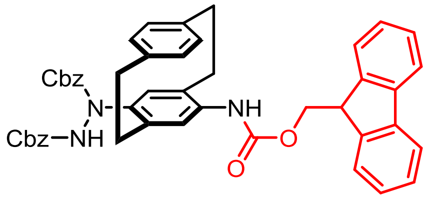

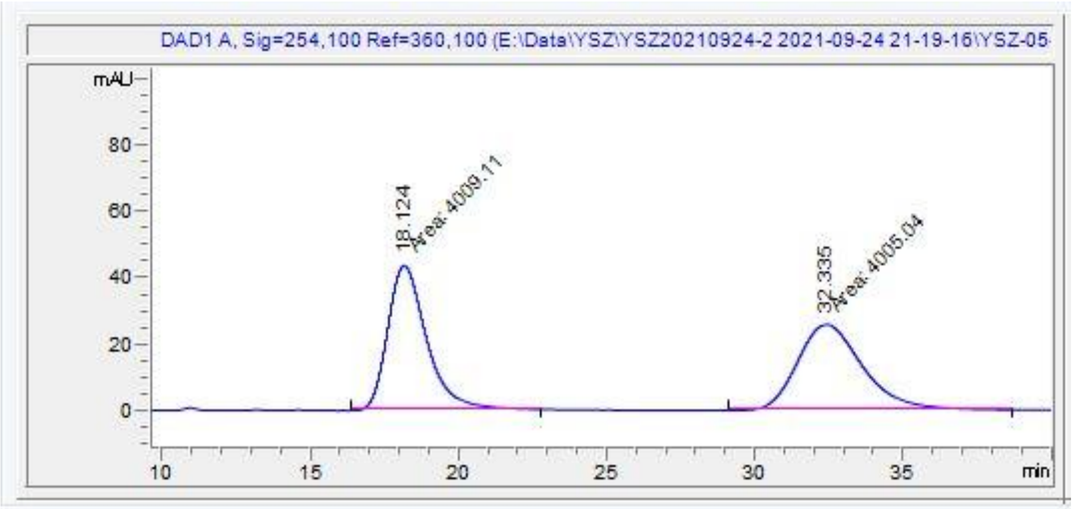

#	Time	Type	Area	Height	Width	Area%	Symmetry
1	18.124	MM	4009.1	43.9	1.5218	50.025	0.707
2	32.335	MM	4005	26.1	2.5569	49.975	0.764

Supplementary Fig. 40 HPLC spectrum of racemic **3e**

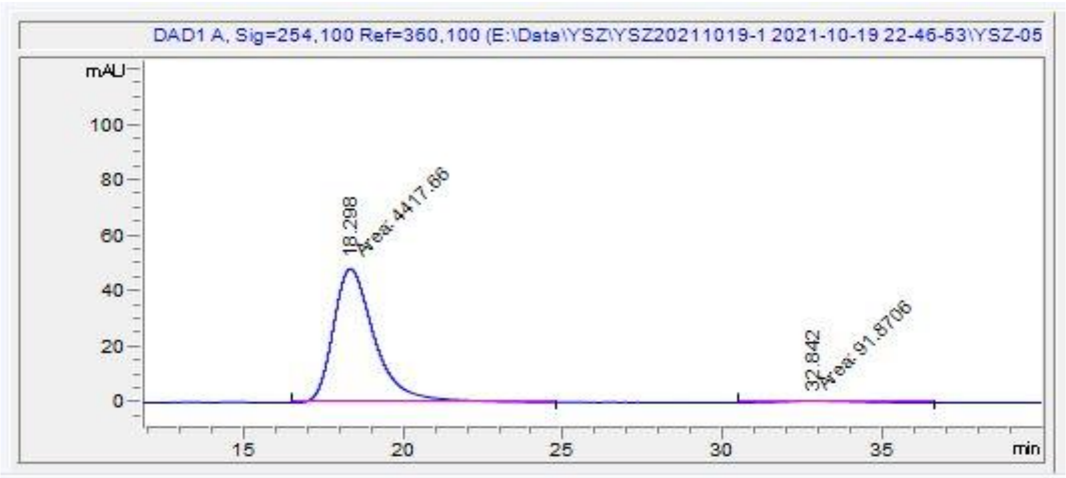

#	Time	Type	Area	Height	Width	Area%	Symmetry
1	18.298	MM	4417.7	48.6	1.5154	97.963	0.677
2	32.842	MM	91.9	6.5E-1	2.3404	2.037	0.833

Supplementary Fig. 41 HPLC spectrum of (S_p)-**3e**

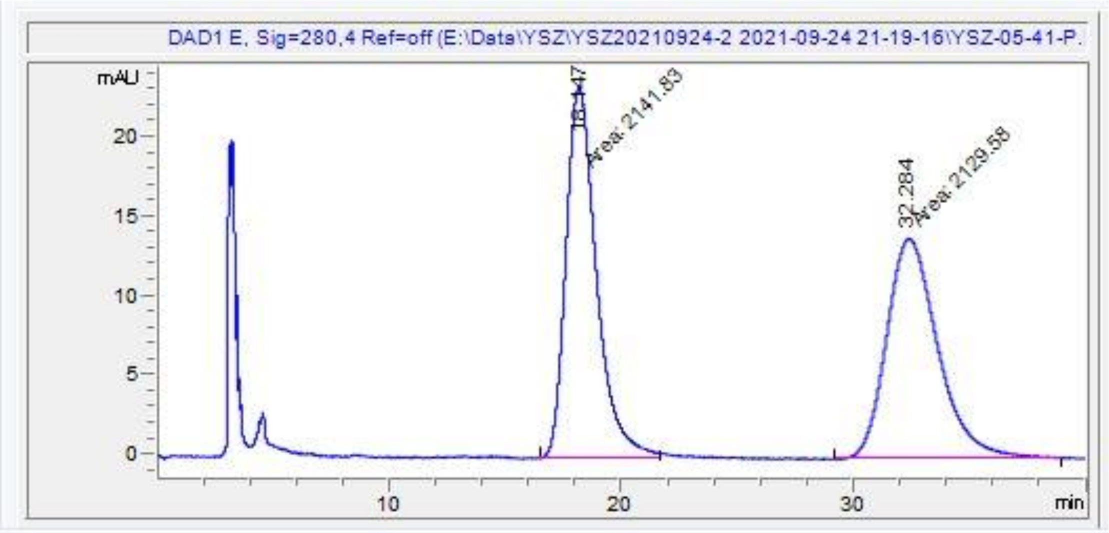

#	Time	Type	Area	Height	Width	Area%	Symmetry
1	18.147	MM	2141.8	23.5	1.5215	50.143	0.752
2	32.284	MM	2129.6	13.9	2.5544	49.857	0.692

Supplementary Fig. 42 Full HPLC spectrum of racemic **3e**

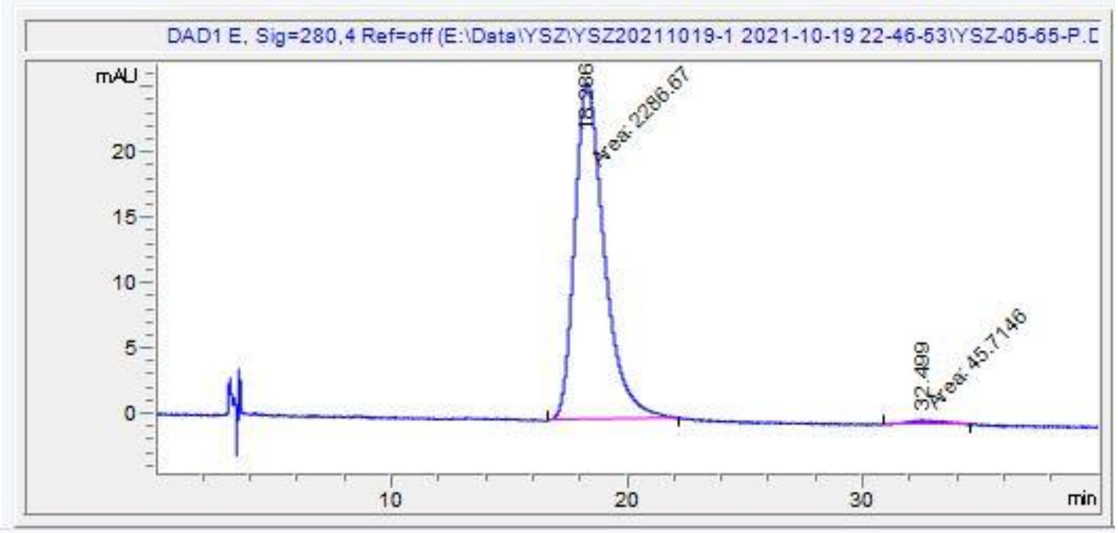

#	Time	Type	Area	Height	Width	Area%	Symmetry
1	18.286	MM	2286.7	25.8	1.4793	98.040	0.682
2	32.499	MM	45.7	4.1E-1	1.8395	1.960	0.735

Supplementary Fig. 43 Full HPLC spectrum of (S_p)-**3e**

(R_p)-Tert-butyl (4[2]-bromo-1,4(1,4)-dibenzenacyclohexaphane-1[2]-yl)carbamate **(1f)**

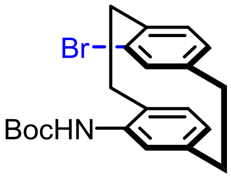

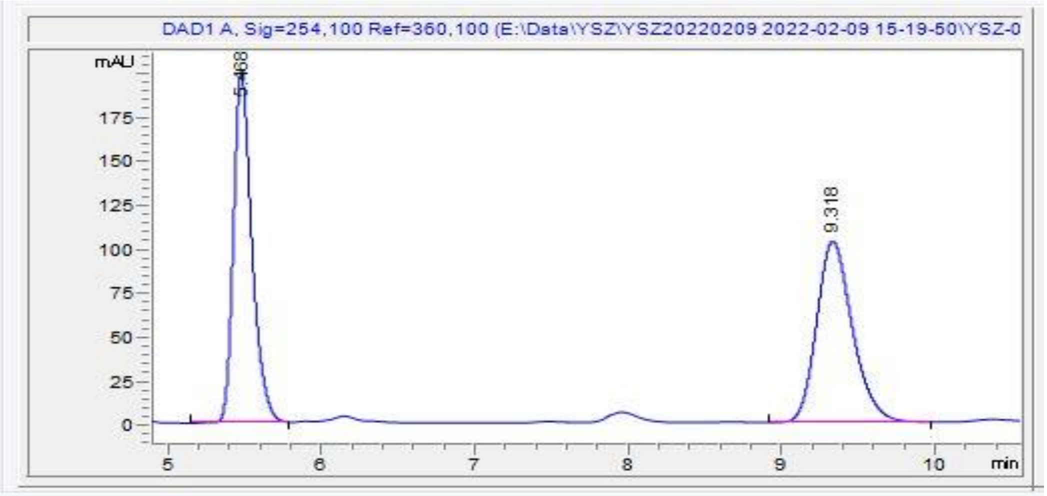

#	Time	Type	Area	Height	Width	Area%	Symmetry
1	5.468	BB	1663.6	199.8	0.1256	49.910	0.71
2	9.318	BB	1669.5	103.1	0.2459	50.090	0.77

Supplementary Fig. 44 HPLC spectrum of racemic **1f**

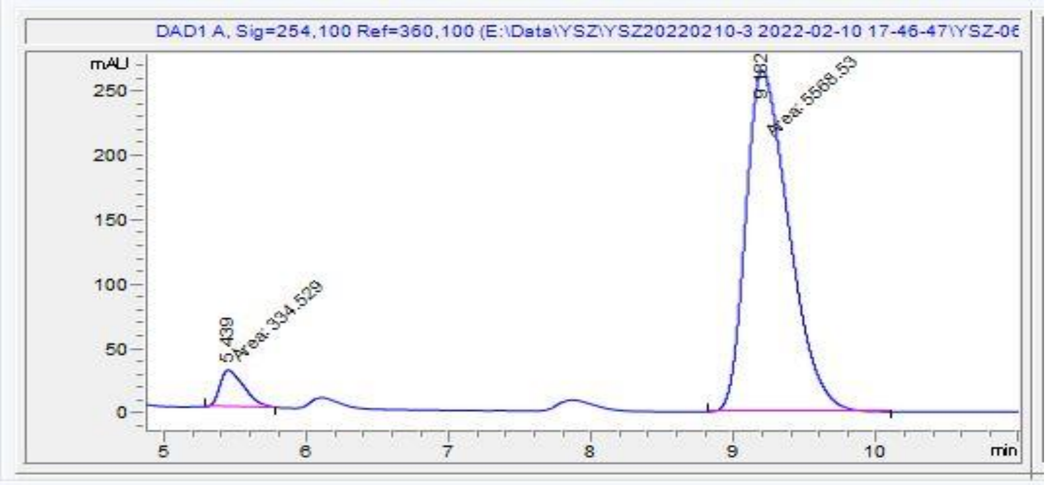

#	Time	Type	Area	Height	Width	Area%	Symmetry
1	5.439	MM	334.5	28.5	0.1953	5.667	0.513
2	9.182	MM	5568.5	263.5	0.3522	94.333	0.612

Supplementary Fig. 45 HPLC spectrum of (R_p)**-1f**

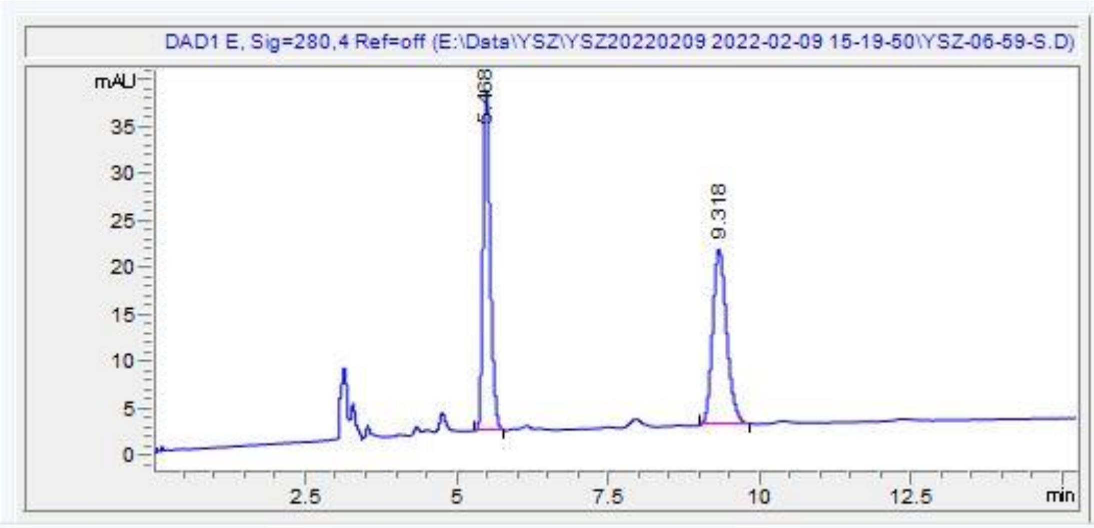

#	Time	Type	Area	Height	Width	Area%	Symmetry
1	5.468	BB	301.7	36.4	0.1262	49.936	0.698
2	9.318	BB	302.5	18.8	0.1896	50.064	0.791

Supplementary Fig. 46 Full HPLC spectrum of racemic **1f**

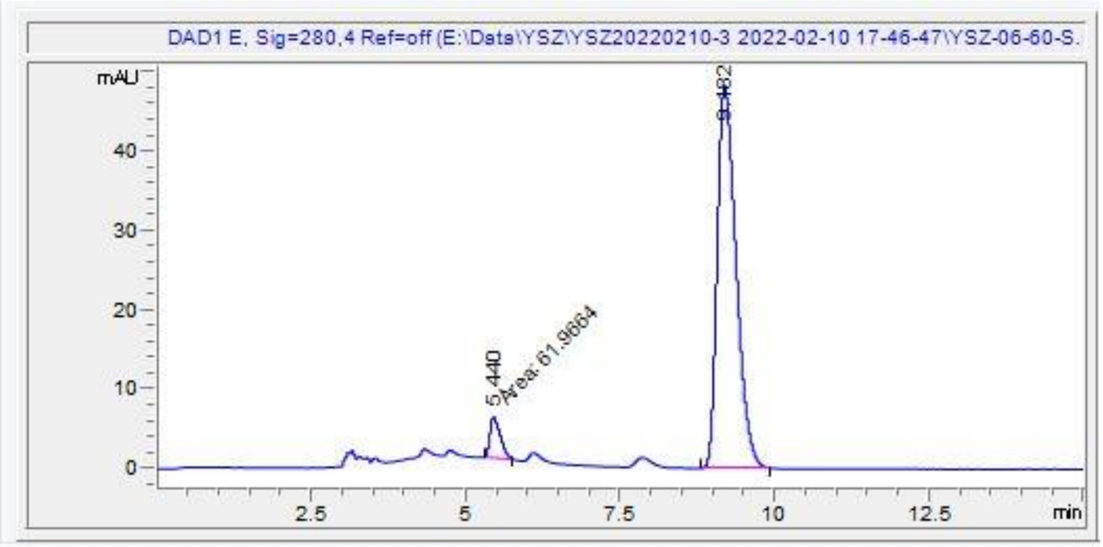

#	Time	Type	Area	Height	Width	Area%	Symmetry
1	5.44	MM	62	5.2	0.197	5.742	0.524
2	9.182	BB	1017.2	48.3	0.2634	94.258	0.609

Supplementary Fig. 47 Full HPLC spectrum of (R_p)-**1f**

(S_p)-Dibenzyl 1-(4^3-bromo-1^5-((tert-butoxycarbonyl)amino)-1,4(1,4)-dibenzenacyclohexaphane-1^2-yl)hydrazine-1,2-dicarboxylate **(3f)**

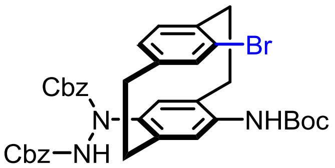

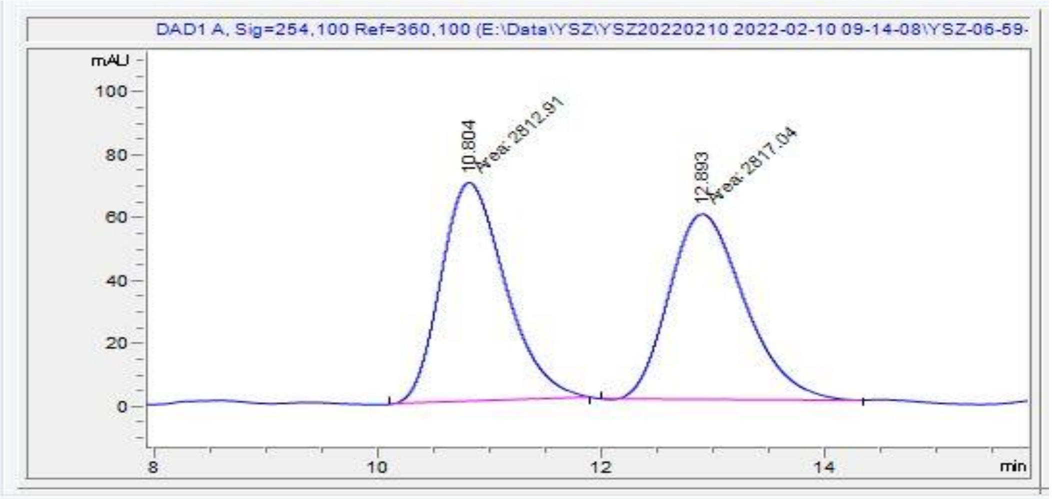

#	Time	Type	Area	Height	Width	Area%	Symmetry
1	10.804	MM	2812.9	69.8	0.6719	49.963	0.748
2	12.893	MM	2817	59.3	0.7915	50.037	0.766

Supplementary Fig. 48 HPLC spectrum of racemic **3f**

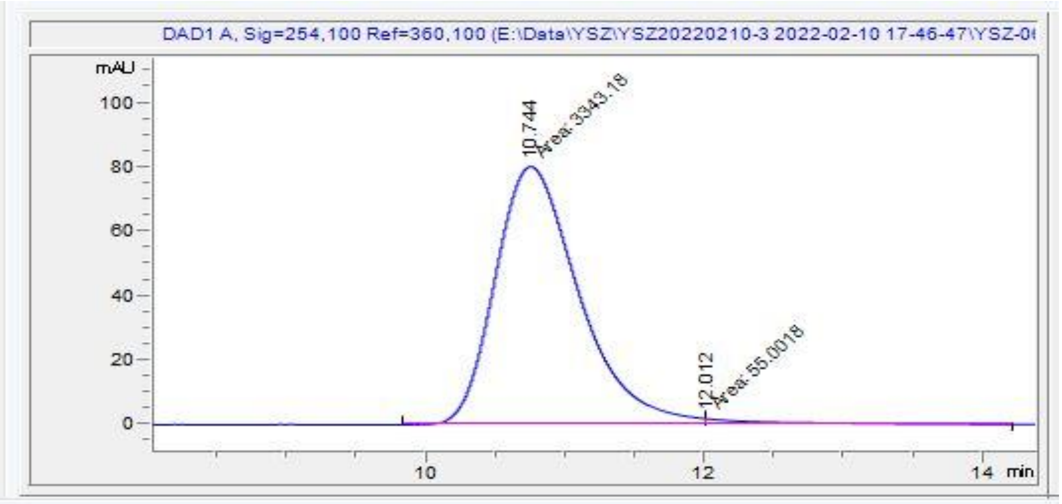

#	Time	Type	Area	Height	Width	Area%	Symmetry
1	10.744	MF	3343.2	79.8	0.6979	98.381	0.698
2	12.012	FM	55	1.7	0.5437	1.619	0

Supplementary Fig. 49 HPLC spectrum of (S_p)-**3f**

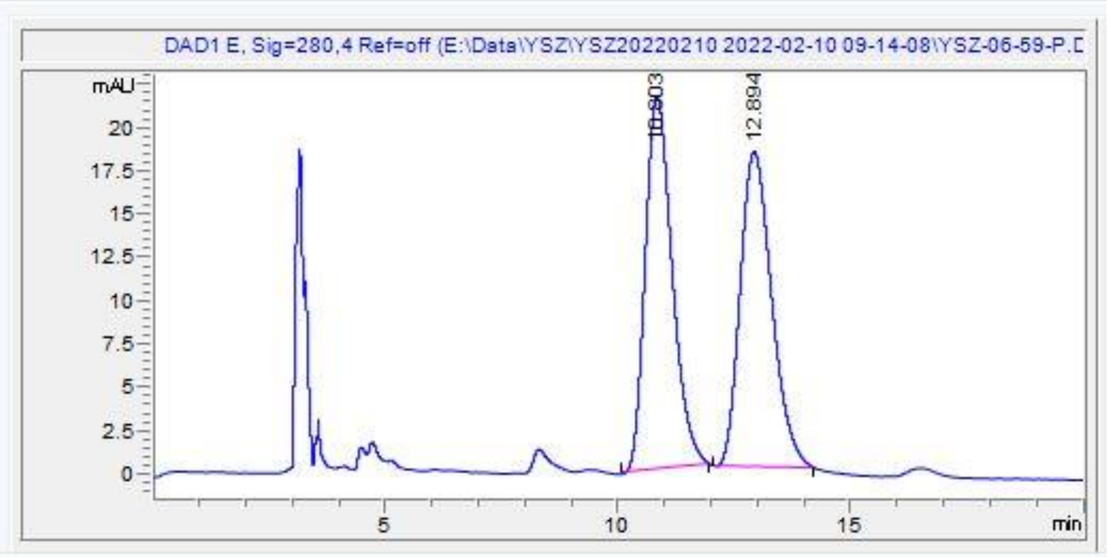

#	Time	Type	Area	Height	Width	Area%	Symmetry
1	10.803	BB	879.1	21.7	0.4767	50.393	0.733
2	12.894	BB	865.4	18.3	0.5527	49.607	0.772

Supplementary Fig. 50 Full HPLC spectrum of racemic **3f**

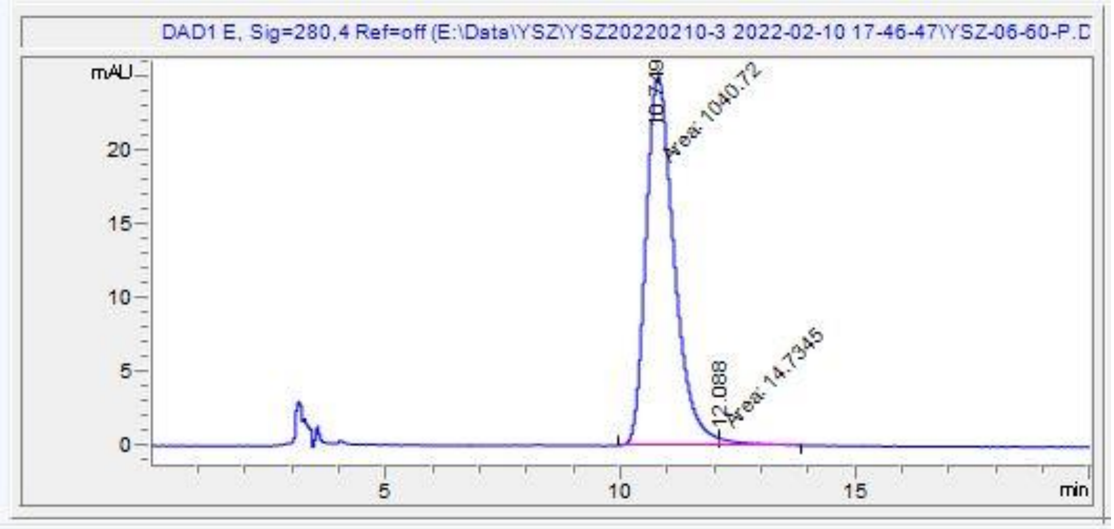

#	Time	Type	Area	Height	Width	Area%	Symmetry
1	10.749	MF	1040.7	24.8	0.6986	98.604	0.718
2	12.088	FM	14.7	4.6E-1	0.5347	1.396	8.38E-3

Supplementary Fig. 51 Full HPLC spectrum of (S_p)-**3f**

(R_p)-Tert-butyl (4^2-phenyl-1,4(1,4)-dibenzenacyclohexaphane-1^2-yl)carbamate **(1g)**

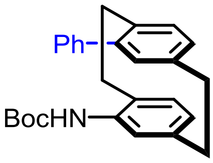

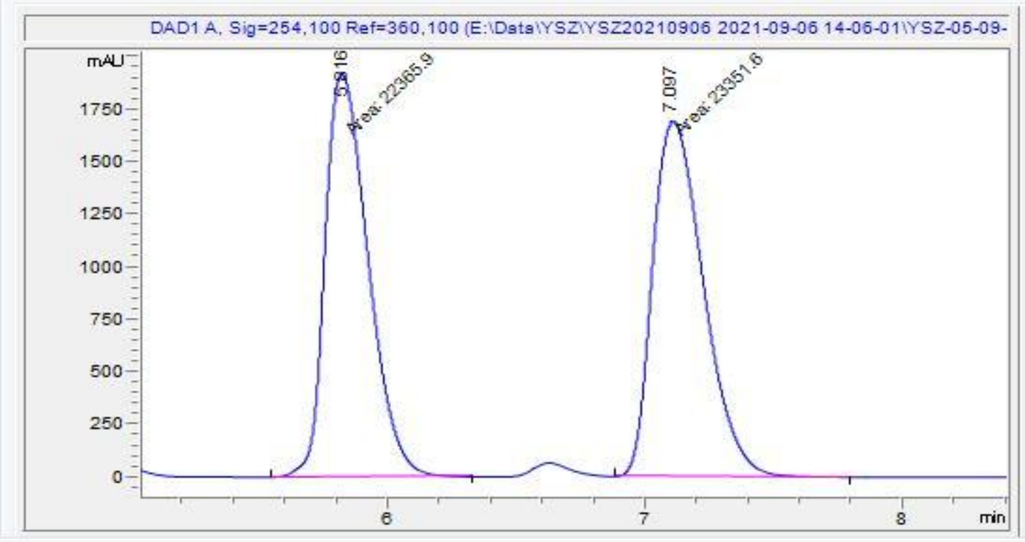

#	Time	Type	Area	Height	Width	Area%	Symmetry
1	5.816	MM	22365.9	1925.5	0.1936	48.922	0.596
2	7.097	MM	23351.6	1694.8	0.2296	51.078	0.574

Supplementary Fig. 52 HPLC spectrum of racemic **1g**

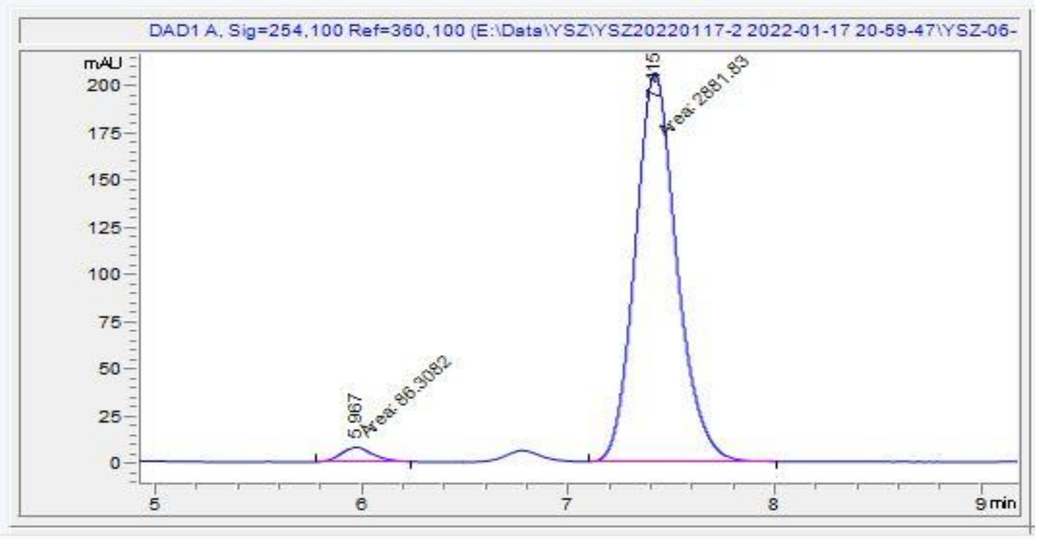

#	Time	Type	Area	Height	Width	Area%	Symmetry
1	5.967	MM	86.3	7.8	0.1851	2.908	0.819
2	7.415	MM	2881.8	205.4	0.2339	97.092	0.845

Supplementary Fig. 53 HPLC spectrum of (R_p)-**1g**

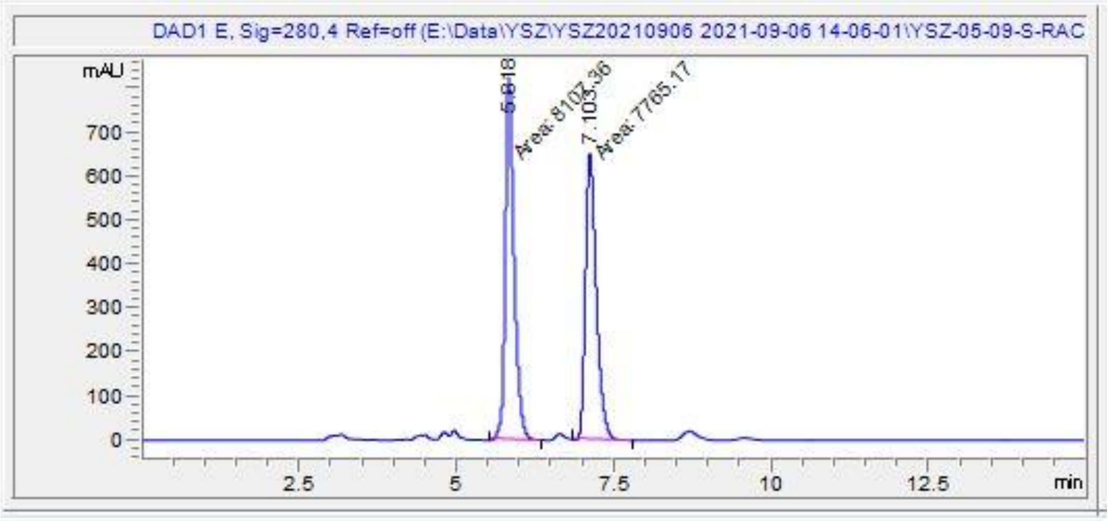

#	Time	Type	Area	Height	Width	Area%	Symmetry
1	5.818	MM	8107.4	824	0.164	51.078	0.677
2	7.103	MM	7765.2	649.6	0.1992	48.922	0.676

Supplementary Fig. 54 Full HPLC spectrum of racemic **1g**

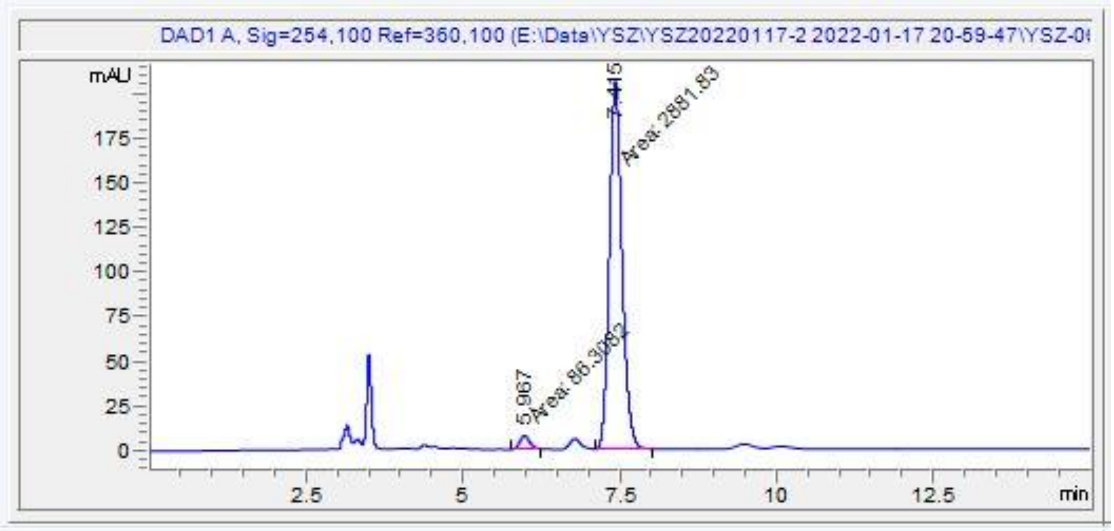

#	Time	Type	Area	Height	Width	Area%	Symmetry
1	5.967	MM	86.3	7.8	0.1851	2.908	0.819
2	7.415	MM	2881.8	205.4	0.2339	97.092	0.845

Supplementary Fig. 55 Full HPLC spectrum of (R_p)-**1g**

(S_p)-Dibenzyl 1-(1^5-((tert-butoxycarbonyl)amino)-4^3-phenyl-1,4(1,4)-dibenzenacyclohexaphane-1^2-yl)hydrazine-1,2-dicarboxylate **(3g)**

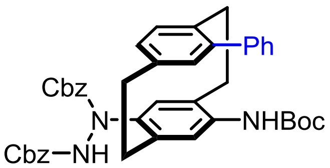

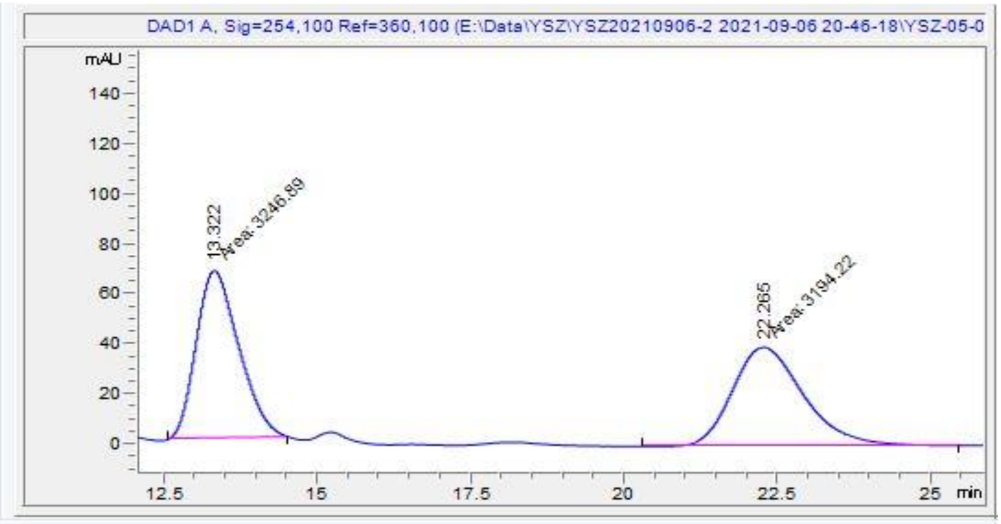

#	Time	Type	Area	Height	Width	Area%	Symmetry
1	13.322	MM	3246.9	67.2	0.8047	50.409	0.76
2	22.265	MM	3194.2	39.6	1.346	49.591	0.778

Supplementary Fig. 56 HPLC spectrum of racemic **3g**

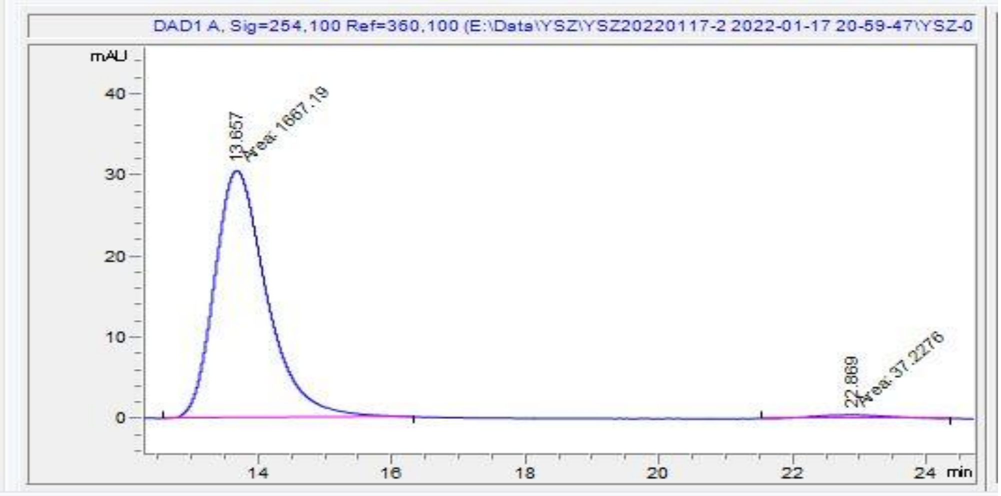

#	Time	Type	Area	Height	Width	Area%	Symmetry
1	13.657	MM	1667.2	30.6	0.9081	97.816	0.692
2	22.869	MM	37.2	4.7E-1	1.317	2.184	0.942

Supplementary Fig. 57 HPLC spectrum of (S_p)-**3g**

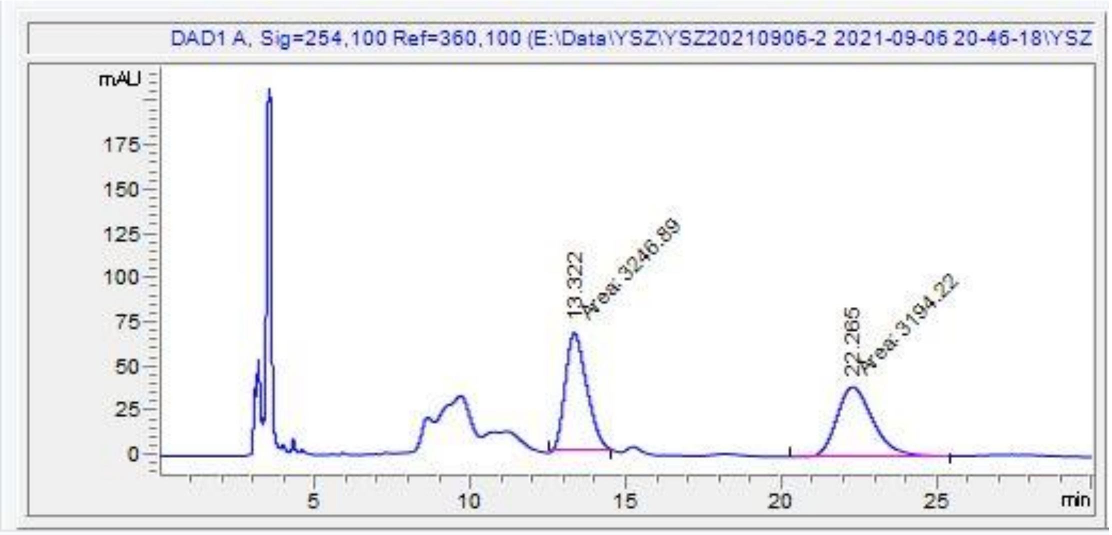

#	Time	Type	Area	Height	Width	Area%	Symmetry
1	13.322	MM	3246.9	67.2	0.8047	50.409	0.76
2	22.265	MM	3194.2	39.6	1.346	49.591	0.778

Supplementary Fig. 58 Full HPLC spectrum of racemic **3g**

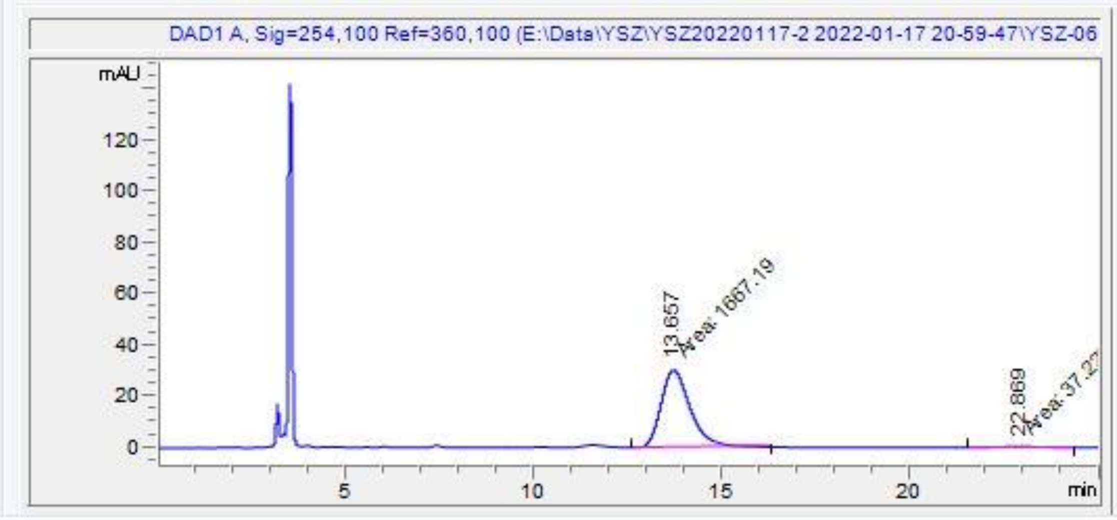

#	Time	Type	Area	Height	Width	Area%	Symmetry
1	13.657	MM	1667.2	30.6	0.9081	97.816	0.692
2	22.869	MM	37.2	4.7E-1	1.317	2.184	0.942

Supplementary Fig. 59 Full HPLC spectrum of (S_p)-**3g**

(R_p)-Tert-butyl-(4^2-(4-methoxyphenyl)-1,4(1,4)-dibenzenacyclohexaphane-1^2-yl)carbamate **(1h)**

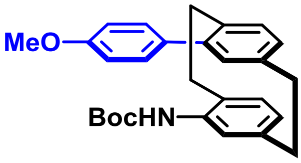

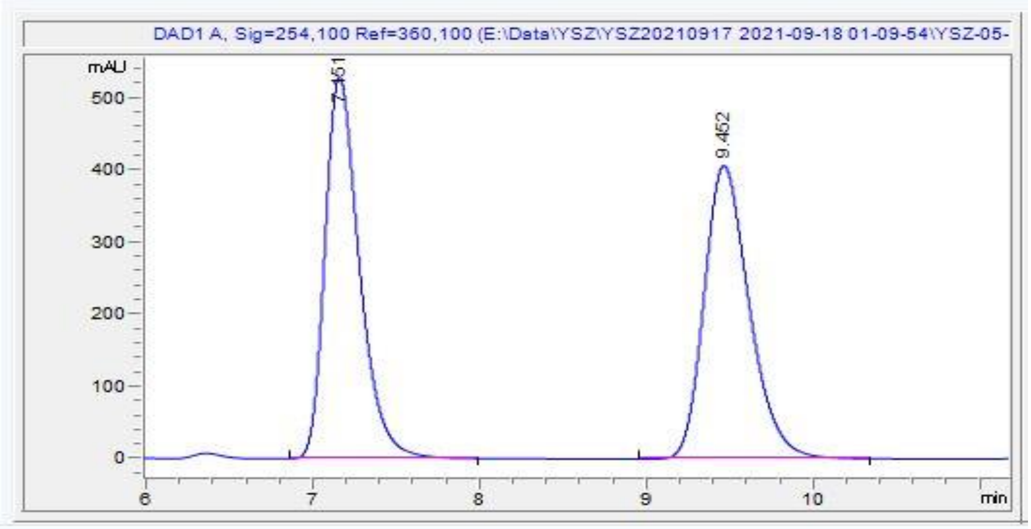

#	Time	Type	Area	Height	Width	Area%	Symmetry
1	7.151	BB	7553.8	528.3	0.1963	49.769	0.68
2	9.452	BB	7623.8	405.9	0.2571	50.231	0.744

Supplementary Fig. 60 HPLC spectrum of racemic **1h**

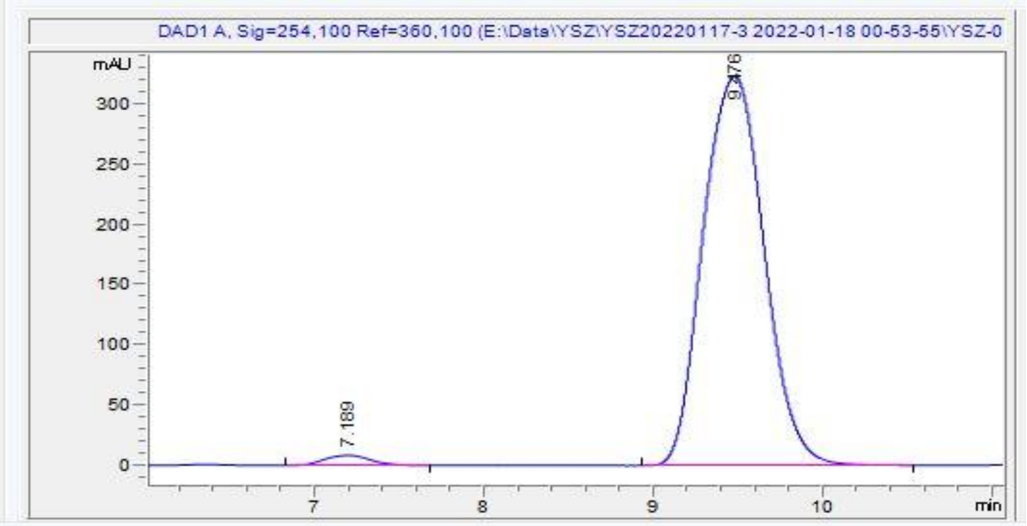

#	Time	Type	Area	Height	Width	Area%	Symmetry
1	7.189	BB	160.4	8.4	0.2265	1.939	1.035
2	9.476	BB	8111.7	323.4	0.3536	98.061	1.007

Supplementary Fig. 61 HPLC spectrum of (R_p)-**1h**

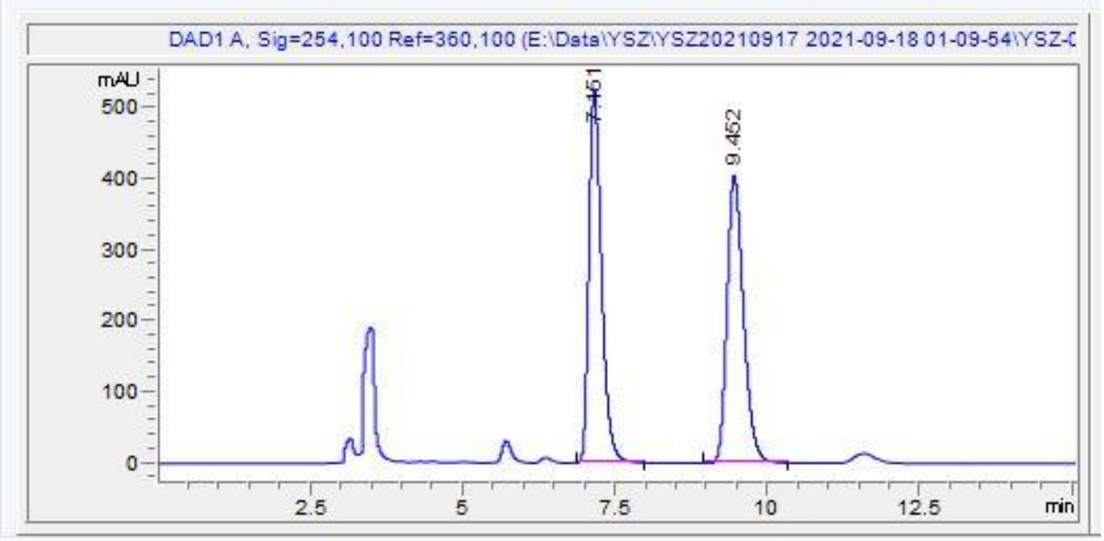

#	Time	Type	Area	Height	Width	Area%	Symmetry
1	7.151	BB	7553.8	528.3	0.1963	49.769	0.68
2	9.452	BB	7623.8	405.9	0.2571	50.231	0.744

Supplementary Fig. 62 Full HPLC spectrum of racemic **1h**

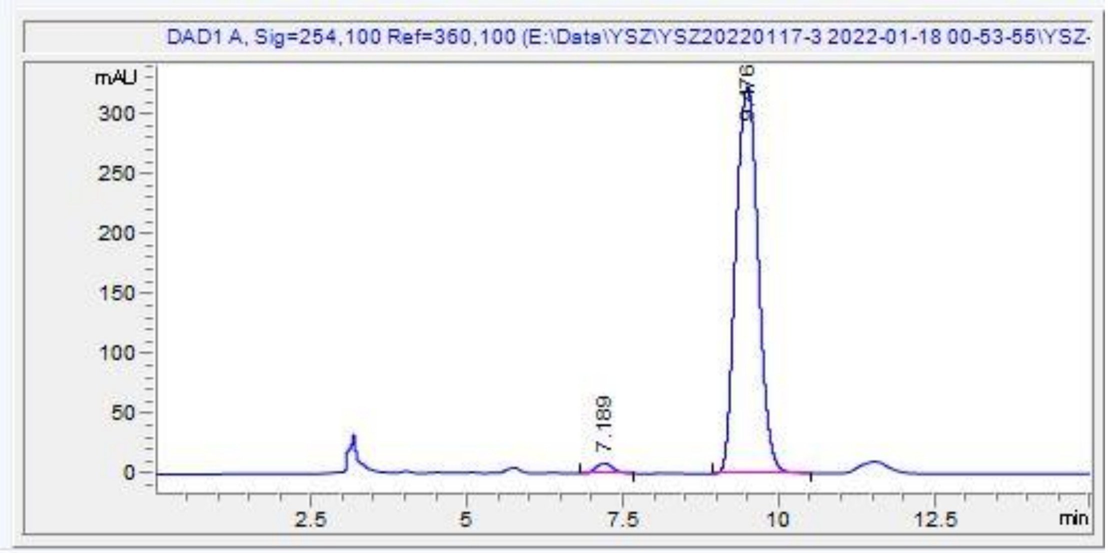

#	Time	Type	Area	Height	Width	Area%	Symmetry
1	7.189	BB	160.4	8.4	0.2265	1.939	1.035
2	9.476	BB	8111.7	323.4	0.3536	98.061	1.007

Supplementary Fig. 63 Full HPLC spectrum of (R_p)-**1h**

(S_p)-Dibenzyl 1-(1[5]-((tert-butoxycarbonyl)amino)-4[3]-(4-methoxyphenyl)-1,4(1,4)-dibenzenacyclohexaphane-1[2]-yl)hydrazine-1,2-dicarboxylate **(3h)**

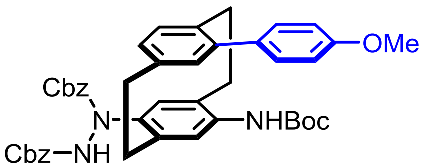

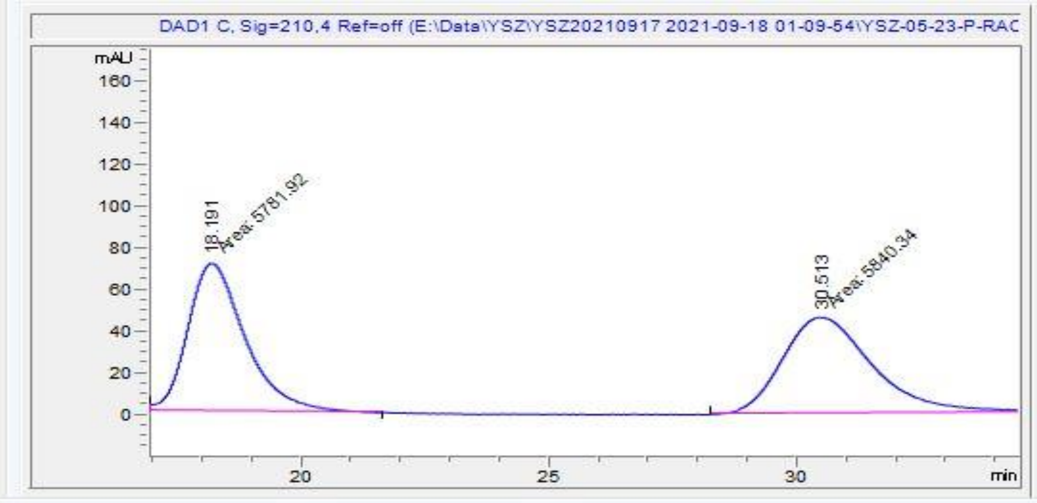

#	Time	Type	Area	Height	Width	Area%	Symmetry
1	18.191	MM	5781.9	71.3	1.3518	49.749	0.708
2	30.513	MM	5840.3	46.1	2.1097	50.251	0.734

Supplementary Fig. 64 HPLC spectrum of racemic **3h**

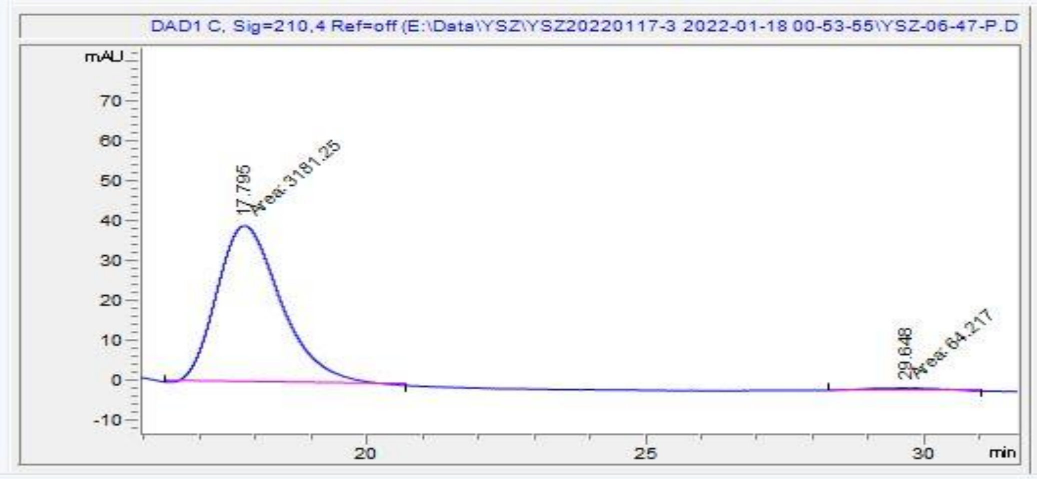

#	Time	Type	Area	Height	Width	Area%	Symmetry
1	17.795	MM	3181.2	39.4	1.3448	98.021	0.749
2	29.648	MM	64.2	7.4E-1	1.4433	1.979	1.066

Supplementary Fig. 65 HPLC spectrum of (S_p)-**3h**

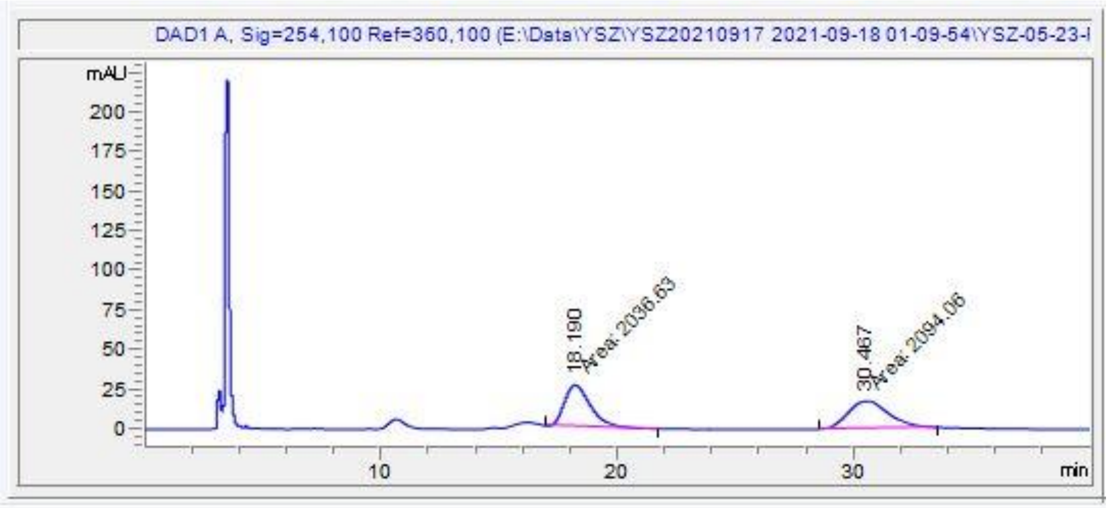

#	Time	Type	Area	Height	Width	Area%	Symmetry
1	18.19	MM	2036.6	26.3	1.2889	49.305	0.687
2	30.467	MM	2094.1	17.4	2.0046	50.695	0.719

Supplementary Fig. 66 Full HPLC spectrum of racemic **3h**

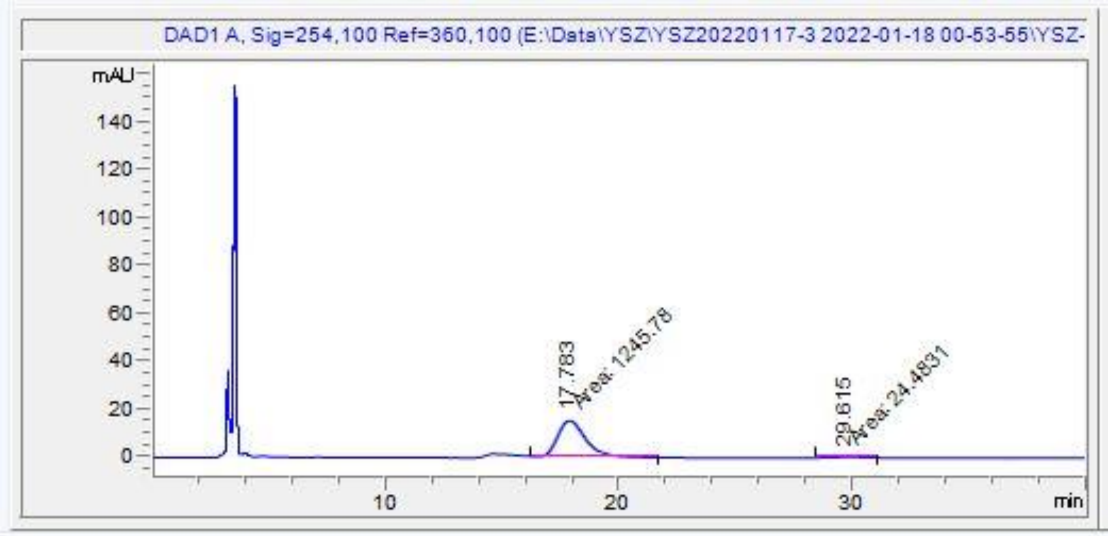

#	Time	Type	Area	Height	Width	Area%	Symmetry
1	17.783	MM	1245.8	15.3	1.361	98.073	0.713
2	29.615	MM	24.5	2.8E-1	1.4406	1.927	0.822

Supplementary Fig. 67 Full HPLC spectrum of (S_p)-**3h**

(R_p)-Tert-butyl (E)-(4^2-styryl-1,4(1,4)-dibenzenacyclohexaphane-1^2-yl)carbamate **(1i)**

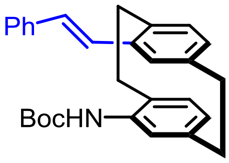

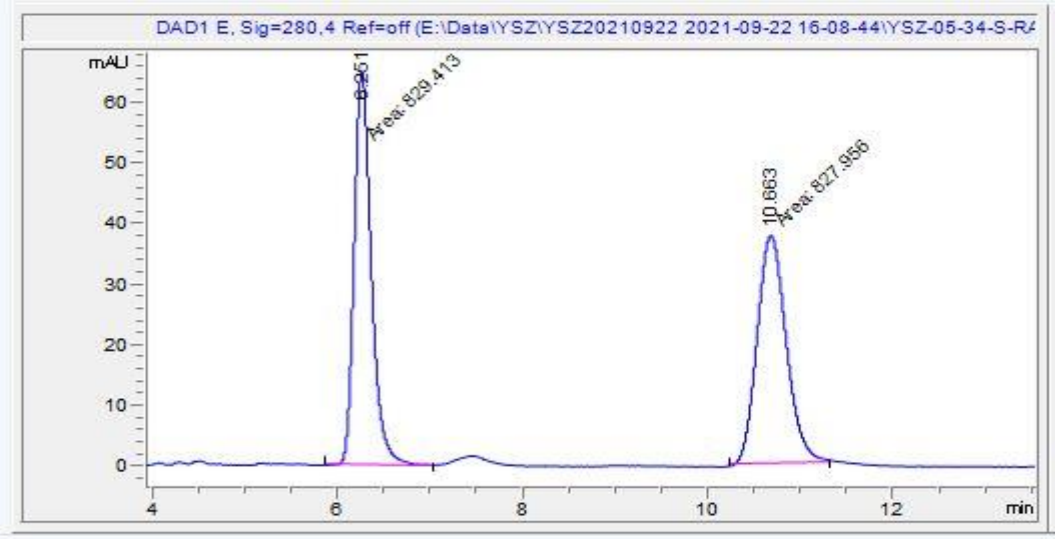

#	Time	Type	Area	Height	Width	Area%	Symmetry
1	6.251	MM	829.4	64.8	0.2133	50.044	0.771
2	10.663	MM	828	37.6	0.3666	49.956	0.858

Supplementary Fig. 68 HPLC spectrum of racemic **1i**

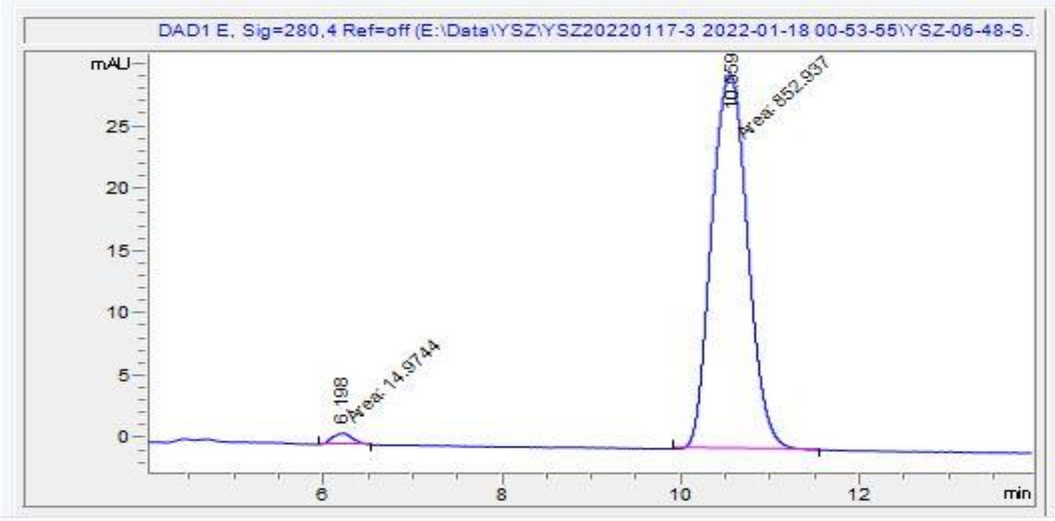

#	Time	Type	Area	Height	Width	Area%	Symmetry
1	6.198	MM	15	9.3E-1	0.269	1.725	0.937
2	10.559	MM	852.9	30.1	0.4718	98.275	1.047

Supplementary Fig. 69 HPLC spectrum of (R_p)-**1i**

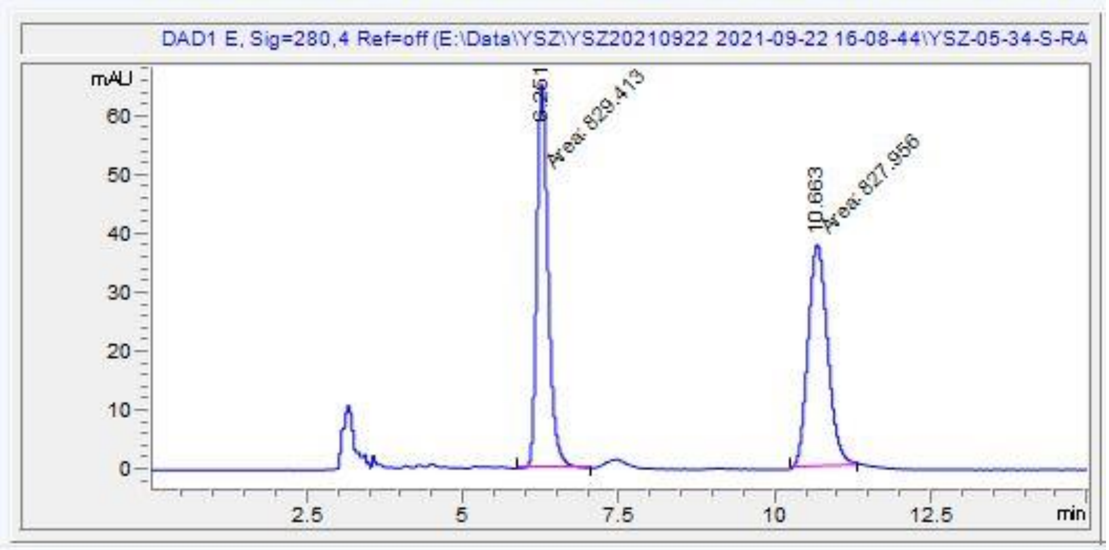

#	Time	Type	Area	Height	Width	Area%	Symmetry
1	6.251	MM	829.4	64.8	0.2133	50.044	0.771
2	10.663	MM	828	37.6	0.3666	49.956	0.858

Supplementary Fig. 70 Full HPLC spectrum of racemic **1i**

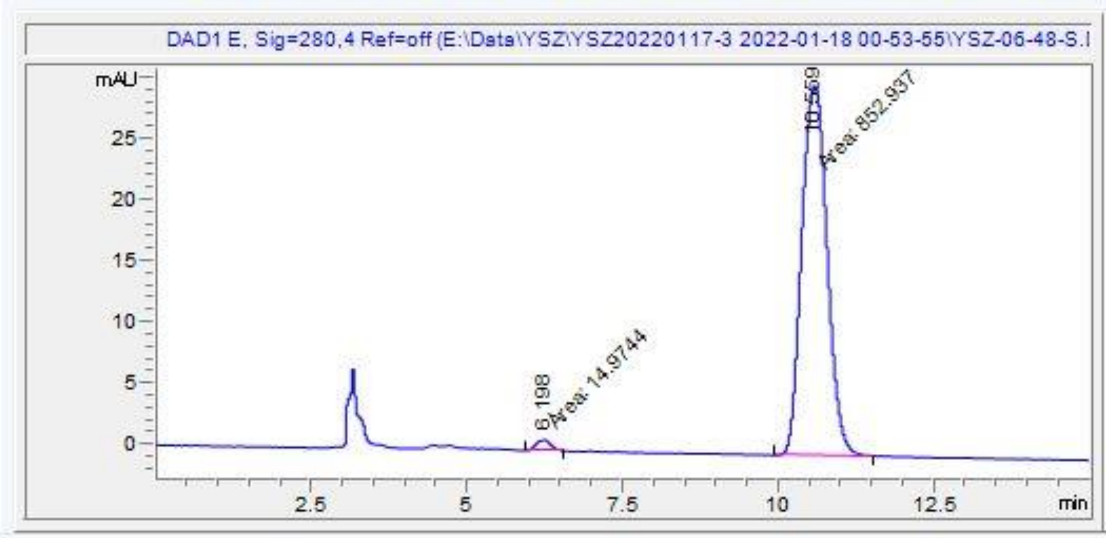

#	Time	Type	Area	Height	Width	Area%	Symmetry
1	6.198	MM	15	9.3E-1	0.269	1.725	0.937
2	10.559	MM	852.9	30.1	0.4718	98.275	1.047

Supplementary Fig. 71 Full HPLC spectrum of (R_p)-**1i**

(S_p)-Dibenzyl (E)-1-(1^5-((tert-butoxycarbonyl)amino)-4^3-styryl-1,4(1,4) -dibenzenacyclohexaphane-1^2-yl)hydrazine-1,2-dicarboxylate **(3i)**

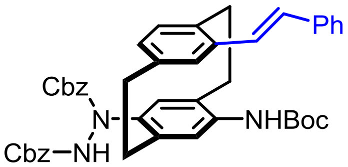

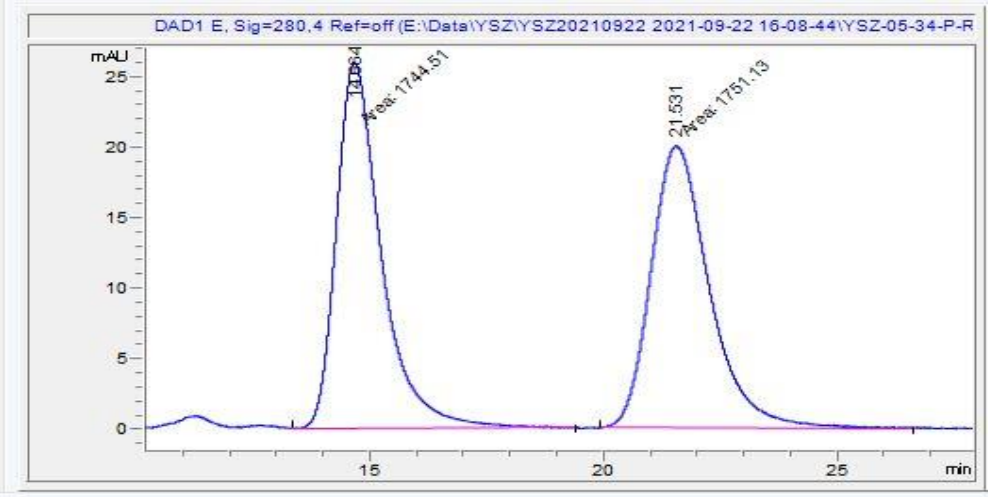

#	Time	Type	Area	Height	Width	Area%	Symmetry
1	14.664	MM	1744.5	26	1.119	49.905	0.662
2	21.531	MM	1751.1	20	1.4569	50.095	0.693

Supplementary Fig. 72 HPLC spectrum of racemic **3i**

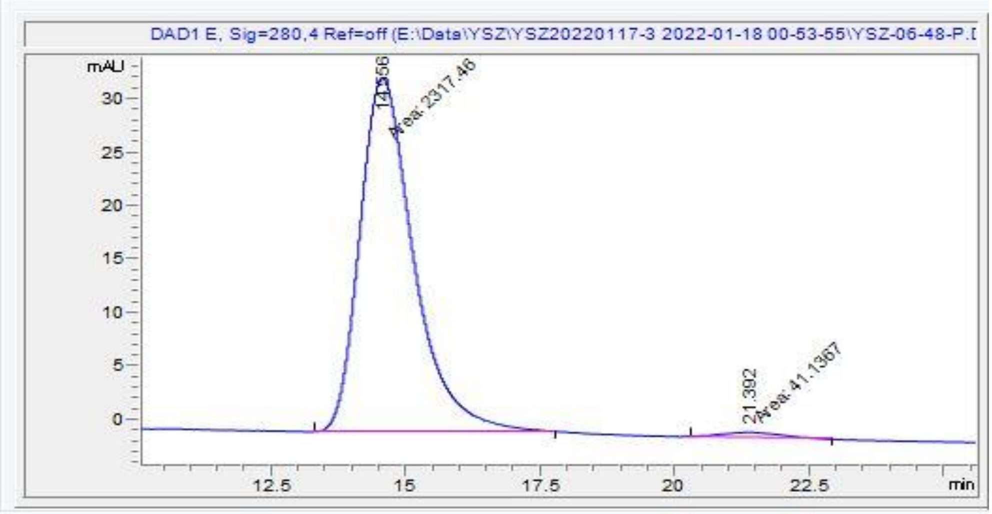

#	Time	Type	Area	Height	Width	Area%	Symmetry
1	14.556	MM	2317.5	33.3	1.1615	98.256	0.685
2	21.392	MM	41.1	5.2E-1	1.3069	1.744	0.764

Supplementary Fig. 73 HPLC spectrum of (S_p)-**3i**

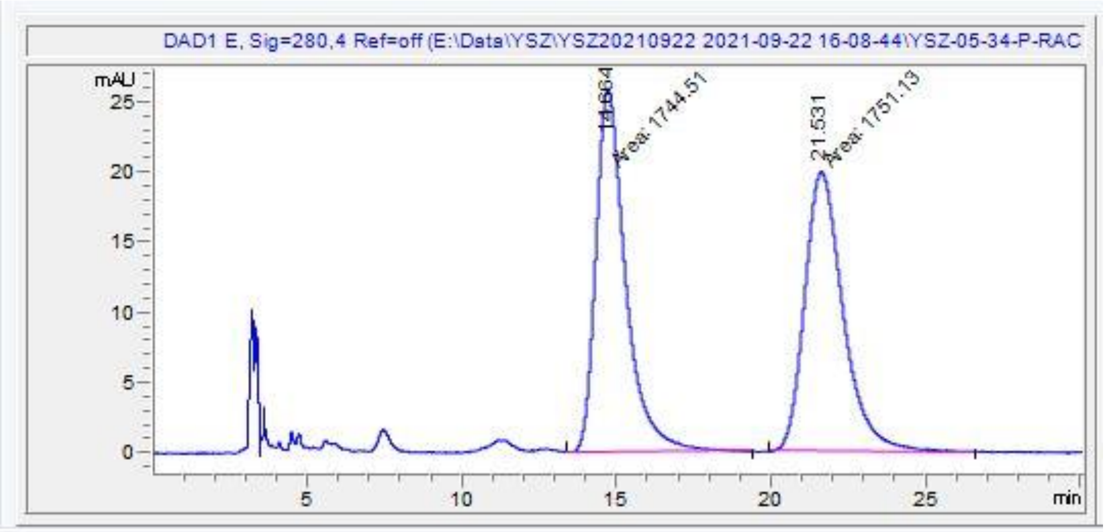

#	Time	Type	Area	Height	Width	Area%	Symmetry
1	14.664	MM	1744.5	26	1.119	49.905	0.662
2	21.531	MM	1751.1	20	1.4569	50.095	0.693

Supplementary Fig. 74 Full HPLC spectrum of racemic **3i**

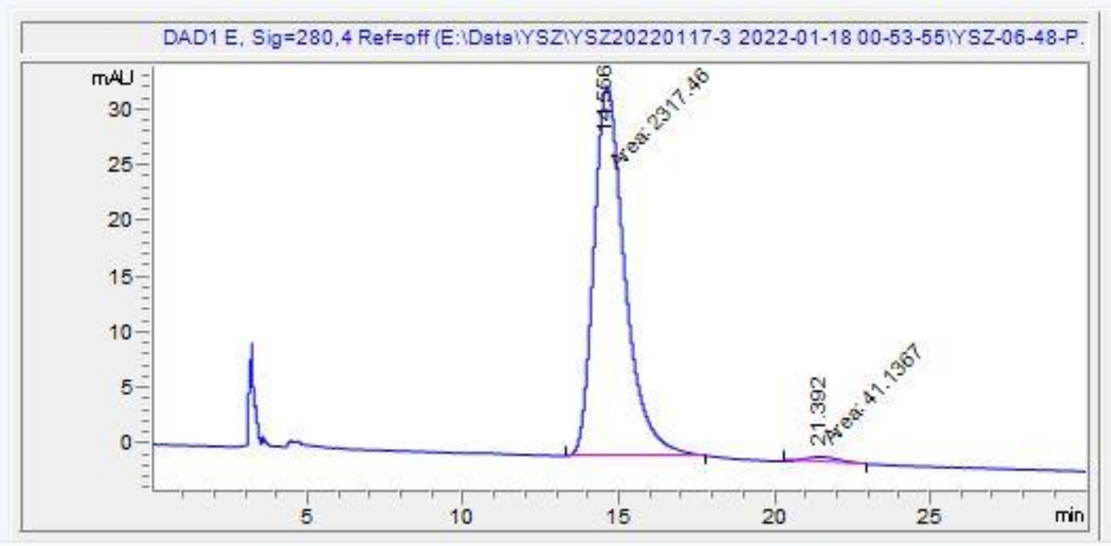

#	Time	Type	Area	Height	Width	Area%	Symmetry
1	14.556	MM	2317.5	33.3	1.1615	98.256	0.685
2	21.392	MM	41.1	5.2E-1	1.3069	1.744	0.764

Supplementary Fig. 75 Full HPLC spectrum of (S_p)-**3i**

(R_p)-Tert-butyl-(4^2-(phenylethynyl)-1,4(1,4)-dibenzenacyclohexaphane-1^2-yl)carbamate **(1j)**

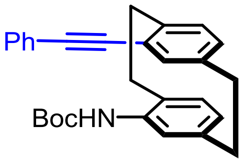

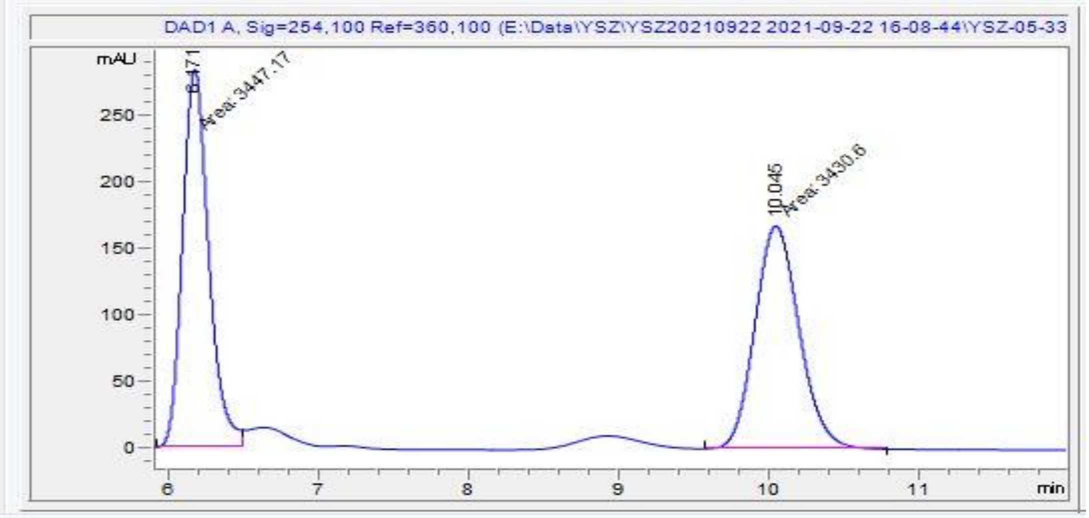

#	Time	Type	Area	Height	Width	Area%	Symmetry
1	6.171	MF	3447.2	283.9	0.2024	50.121	0
2	10.045	MM	3430.6	167.7	0.341	49.879	0.883

Supplementary Fig. 76 HPLC spectrum of racemic **1j**

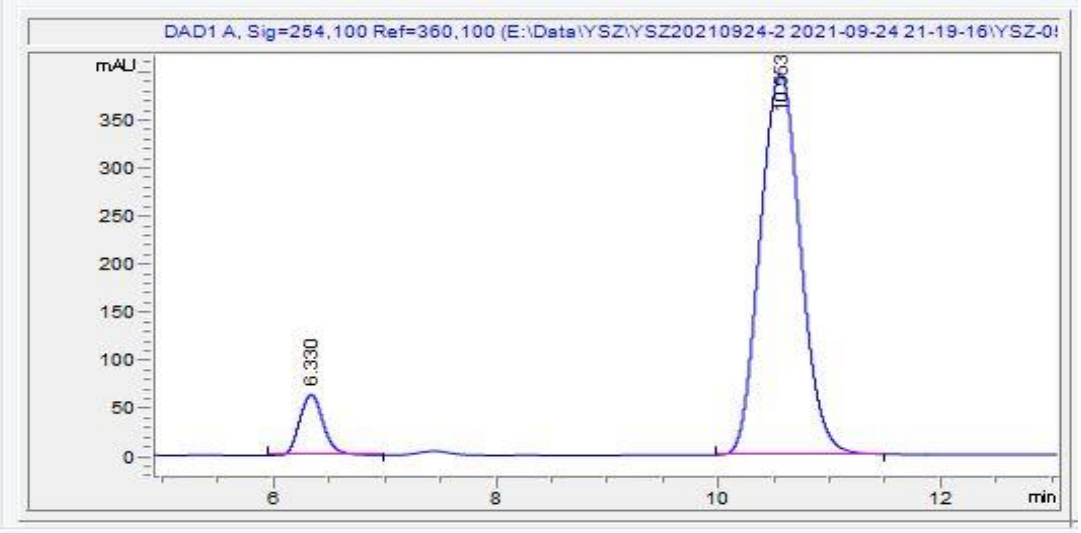

#	Time	Type	Area	Height	Width	Area%	Symmetry
1	6.33	BB	937.1	62.8	0.2142	8.599	0.901
2	10.553	BB	9960.2	394.5	0.3	91.401	0.962

Supplementary Fig. 77 Full HPLC spectrum of (R_p)-**1j**

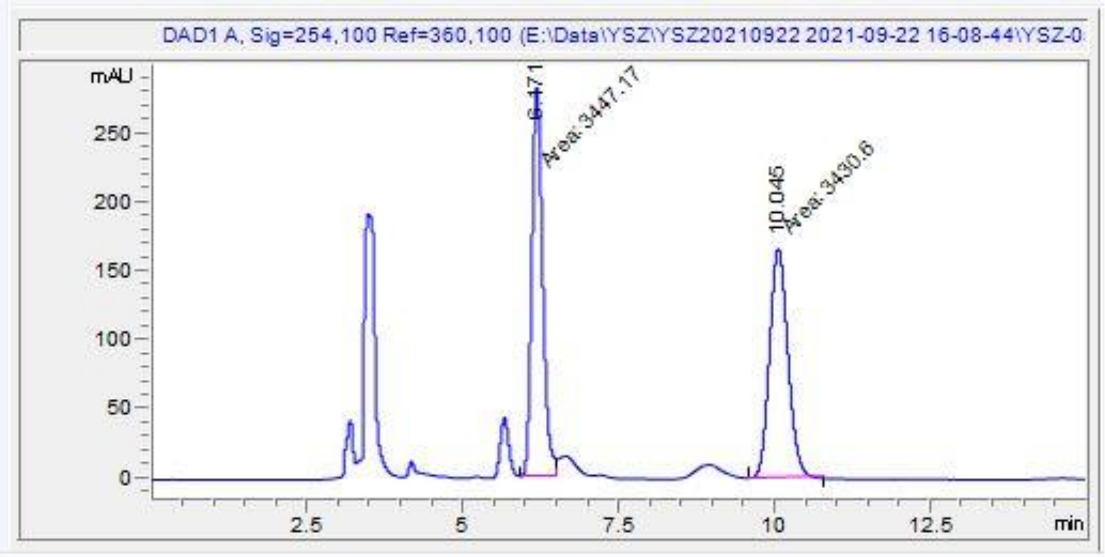

#	Time	Type	Area	Height	Width	Area%	Symmetry
1	6.171	MF	3447.2	283.9	0.2024	50.121	0
2	10.045	MM	3430.6	167.7	0.341	49.879	0.883

Supplementary Fig. 78 Full HPLC spectrum of racemic **1j**

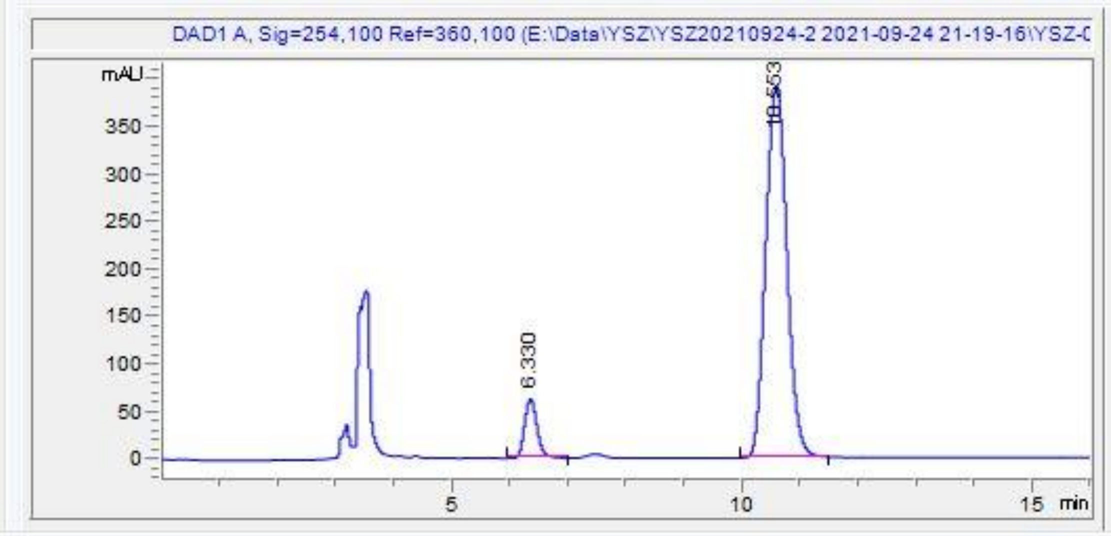

#	Time	Type	Area	Height	Width	Area%	Symmetry
1	6.33	BB	937.1	62.8	0.2142	8.599	0.901
2	10.553	BB	9960.2	394.5	0.3	91.401	0.962

Supplementary Fig. 79 Full HPLC spectrum of (R_p)**-1j**

(S_p)-Dibenzyl 1-(1^5-((tert-butoxycarbonyl)amino)-4^3-(phenylethynyl)-1,4(1,4) -dibenzenacyclohexaphane-1^2-yl)hydrazine-1,2-dicarboxylate **(3j)**

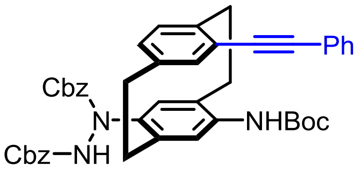

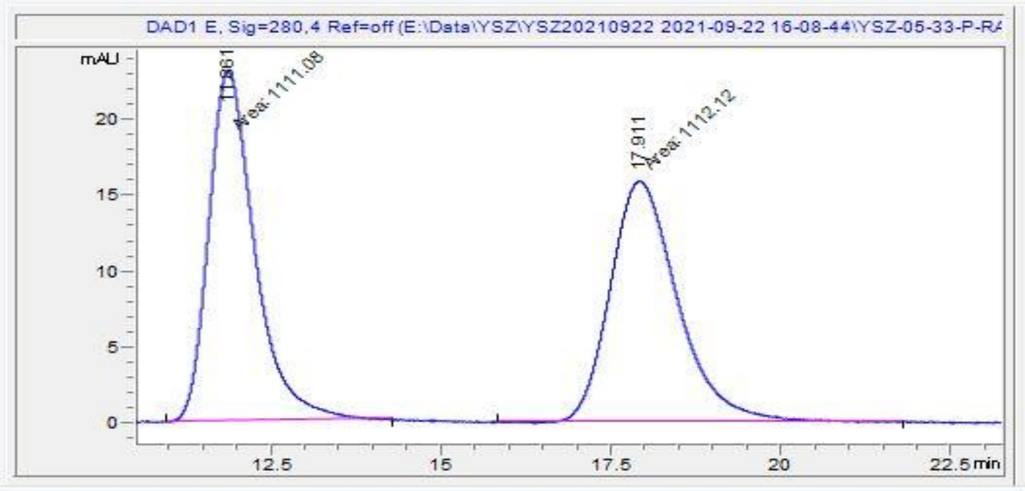

#	Time	Type	Area	Height	Width	Area%	Symmetry
1	11.861	MM	1111.1	23.4	0.7928	49.977	0.693
2	17.911	MM	1112.1	16	1.1561	50.023	0.716

Supplementary Fig. 80 HPLC spectrum of racemic **3j**

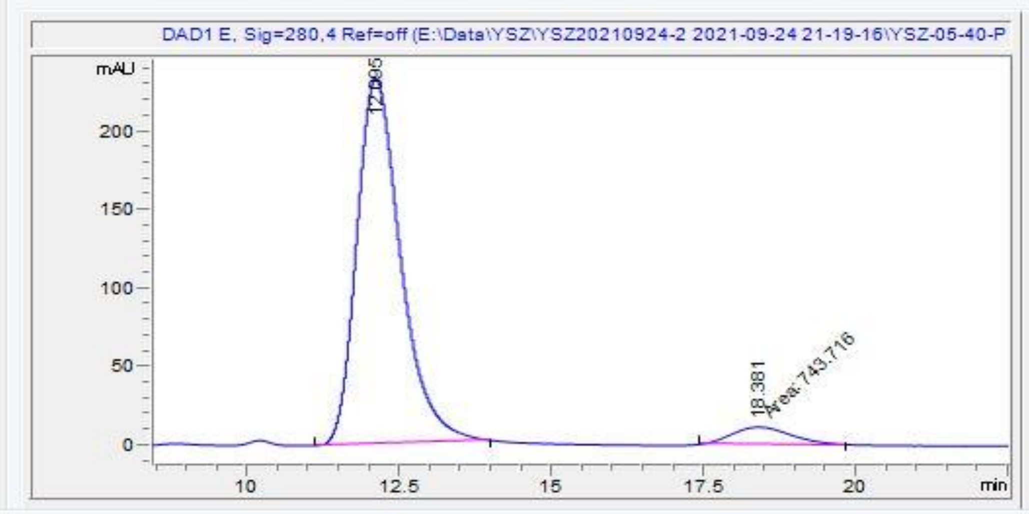

#	Time	Type	Area	Height	Width	Area%	Symmetry
1	12.095	BV R	11713.6	233.1	0.5908	94.030	0.729
2	18.381	MM	743.7	11	1.1293	5.970	0.784

Supplementary Fig. 81 HPLC spectrum of (S_p)-**3j**

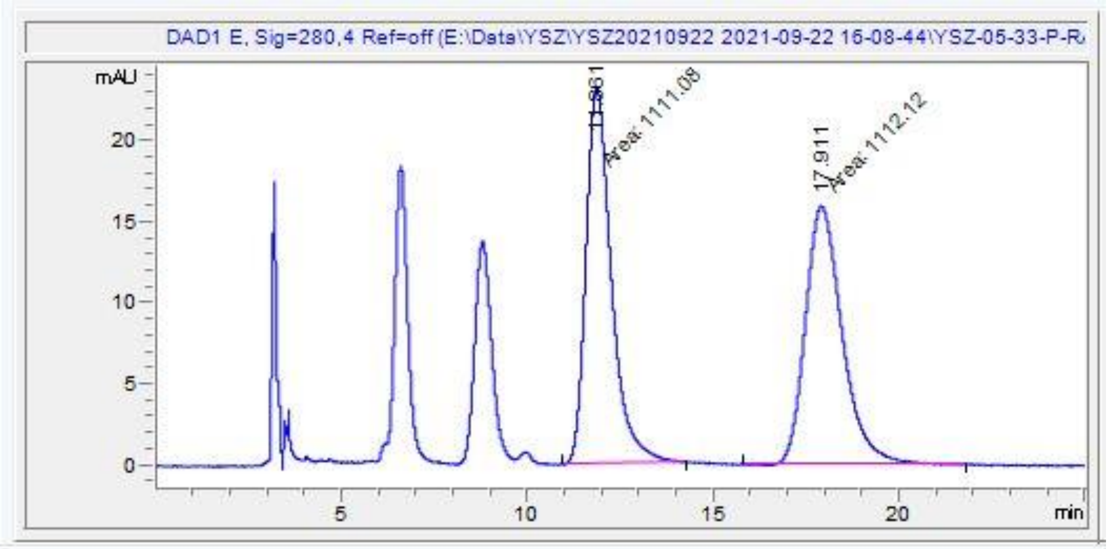

#	Time	Type	Area	Height	Width	Area%	Symmetry
1	11.861	MM	1111.1	23.4	0.7928	49.977	0.693
2	17.911	MM	1112.1	16	1.1561	50.023	0.716

Supplementary Fig. 82 Full HPLC spectrum of racemic **3j**

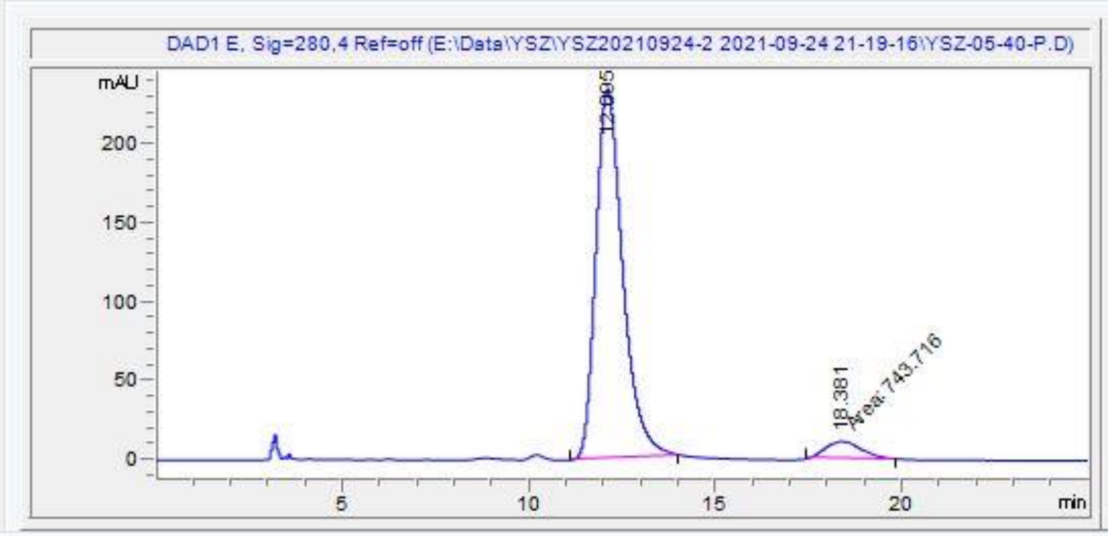

#	Time	Type	Area	Height	Width	Area%	Symmetry
1	12.095	BV R	11713.6	233.1	0.5908	94.030	0.729
2	18.381	MM	743.7	11	1.1293	5.970	0.784

Supplementary Fig. 83 Full HPLC spectrum of (S_p)-**3j**

(R_p)-Tert-butyl (4[2]-cyclohexyl-1,4(1,4)-dibenzenacyclohexaphane-1[2]-yl)carbamate **(1k)**

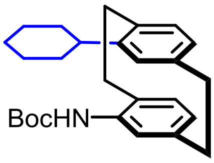

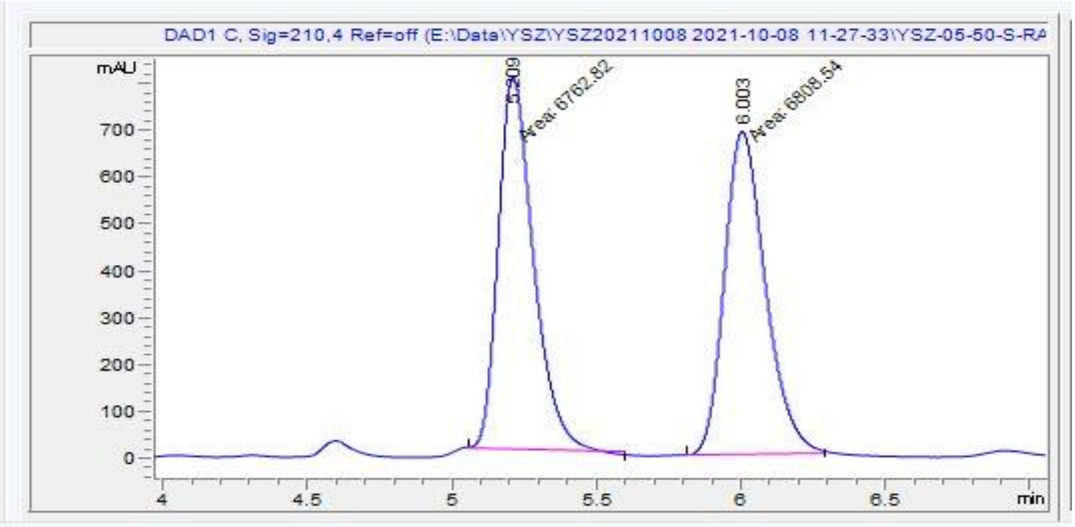

#	Time	Type	Area	Height	Width	Area%	Symmetry
1	5.209	MM	6762.8	793.8	0.142	49.832	0.681
2	6.003	MM	6808.5	688	0.1649	50.168	0.726

Supplementary Fig. 84 HPLC spectrum of racemic **1k**

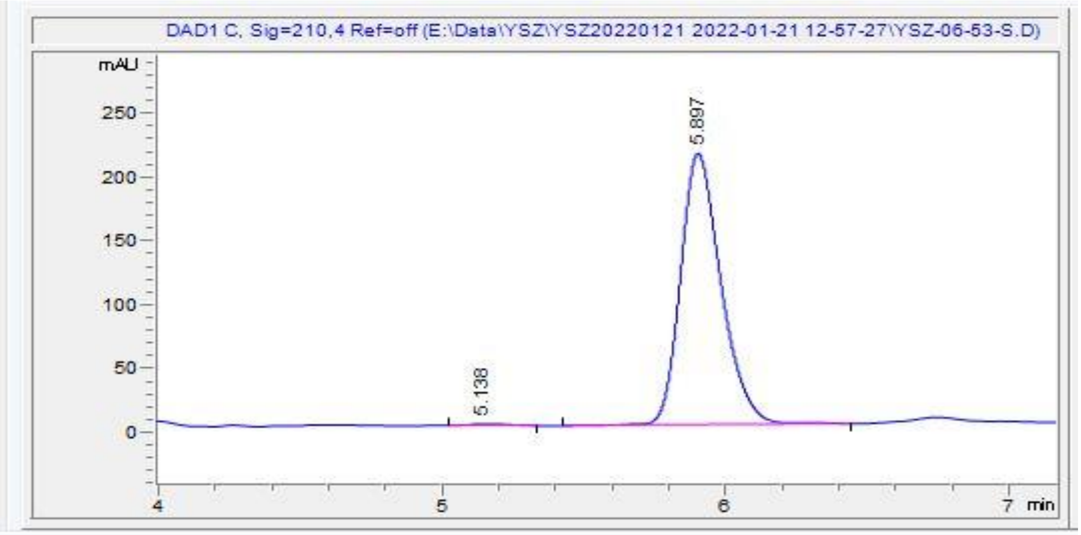

#	Time	Type	Area	Height	Width	Area%	Symmetry
1	5.138	BB	13	1.4	0.1118	0.610	0.513
2	5.897	BV R	2114.9	212.6	0.1513	99.390	0.726

Supplementary Fig. 85 HPLC spectrum of (R_p)-**1k**

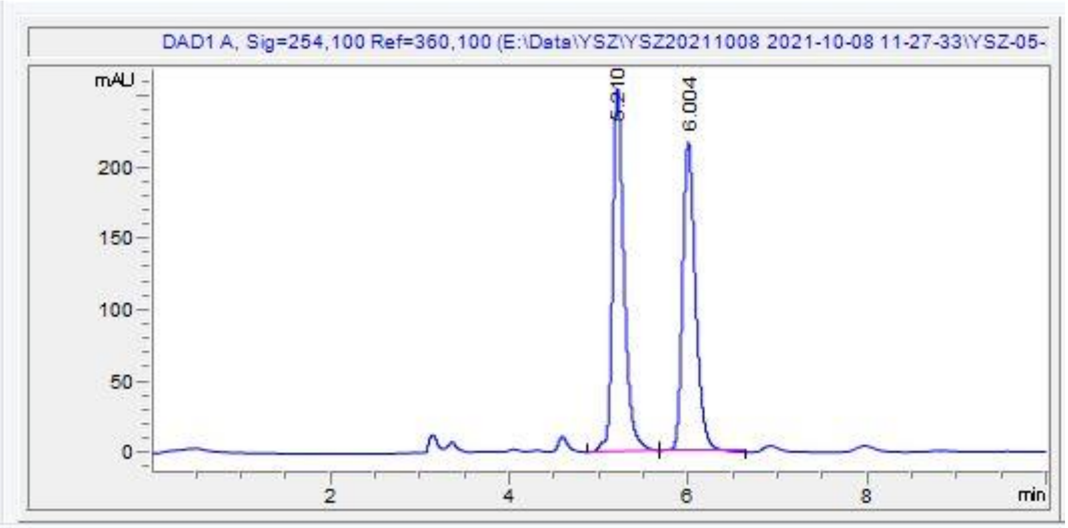

#	Time	Type	Area	Height	Width	Area%	Symmetry
1	5.21	BV	2308.9	254.8	0.1332	50.956	0.674
2	6.004	VB	2222.3	217.7	0.1536	49.044	0.713

Supplementary Fig. 86 Full HPLC spectrum of racemic **1k**

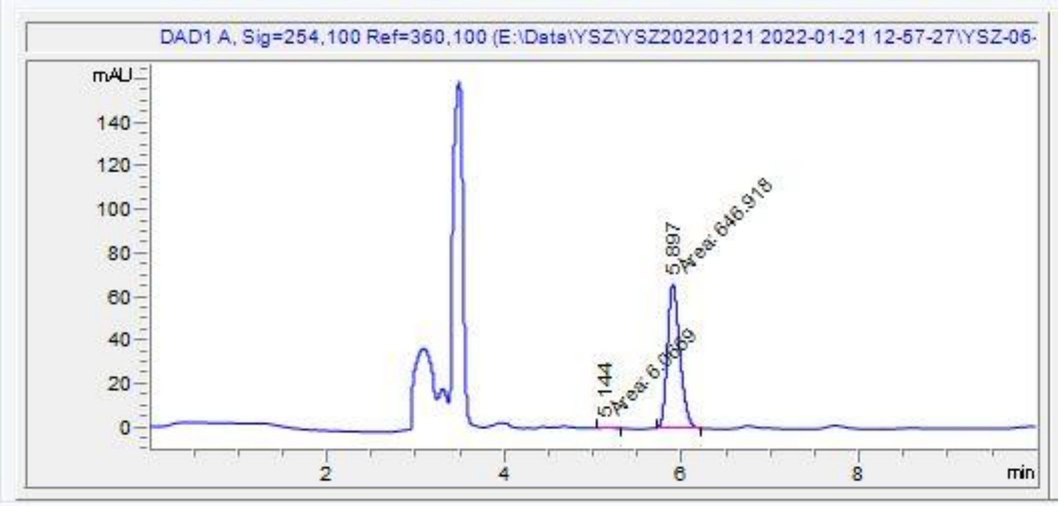

#	Time	Type	Area	Height	Width	Area%	Symmetry
1	5.144	MM	6.1	6.2E-1	0.1629	0.929	0.72
2	5.897	MM	646.9	66.1	0.163	99.071	0.744

Supplementary Fig. 87 Full HPLC spectrum of (R_p)**-1k**

(S_p)-Dibenzyl 1-(1^5-((tert-butoxycarbonyl)amino)-4^3-cyclohexyl-1,4(1,4)-dibenzenacyclohexaphane-1^2-yl)hydrazine-1,2-dicarboxylate **(3k)**

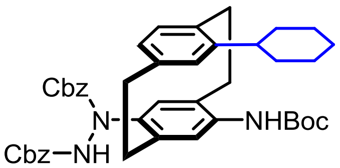

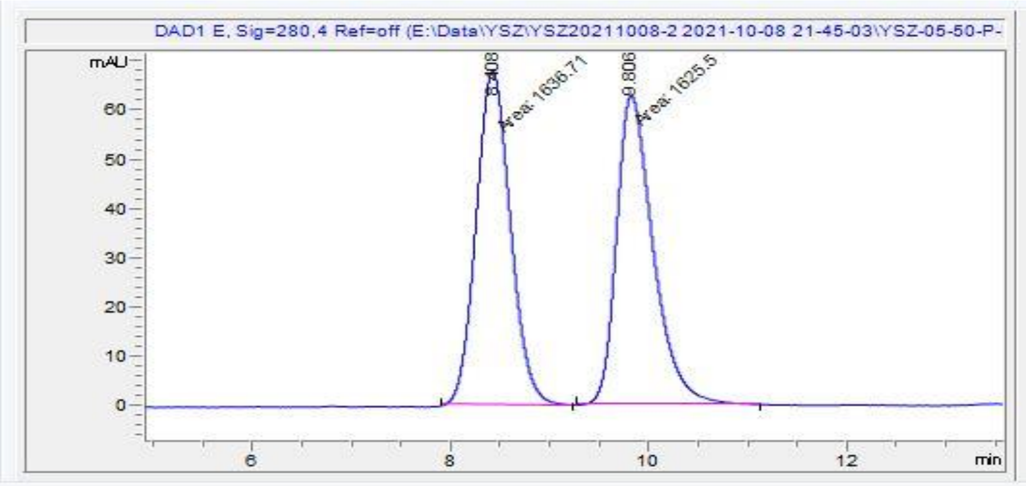

#	Time	Type	Area	Height	Width	Area%	Symmetry
1	8.408	MM	1636.7	67.8	0.4021	50.172	0.851
2	9.806	MM	1625.5	62.9	0.4306	49.828	0.609

Supplementary Fig. 88 HPLC spectrum of racemic **3k**

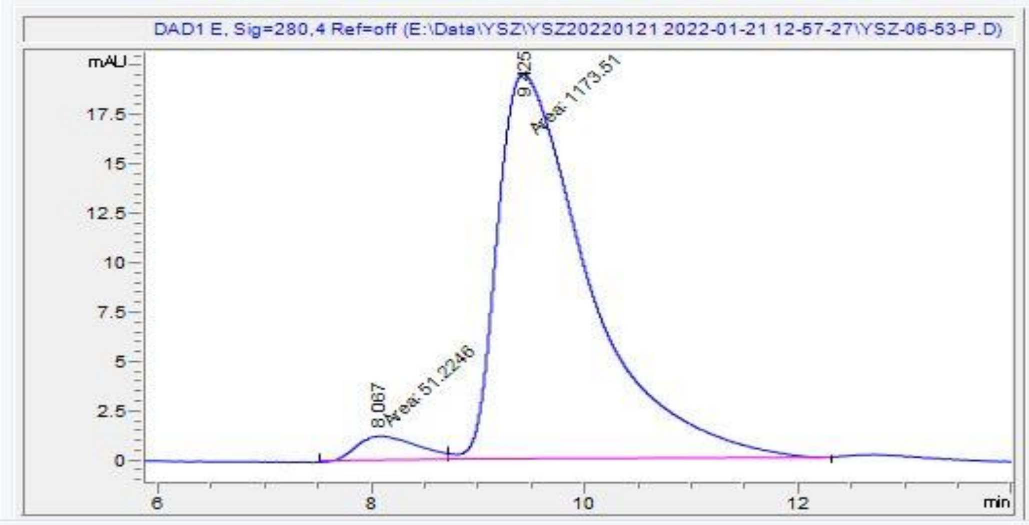

#	Time	Type	Area	Height	Width	Area%	Symmetry
1	8.067	MF	51.2	1.3	0.6637	4.183	0.588
2	9.425	FM	1173.5	19.6	0.996	95.817	0.403

Supplementary Fig. 89 HPLC spectrum of (S_p)-**3k**

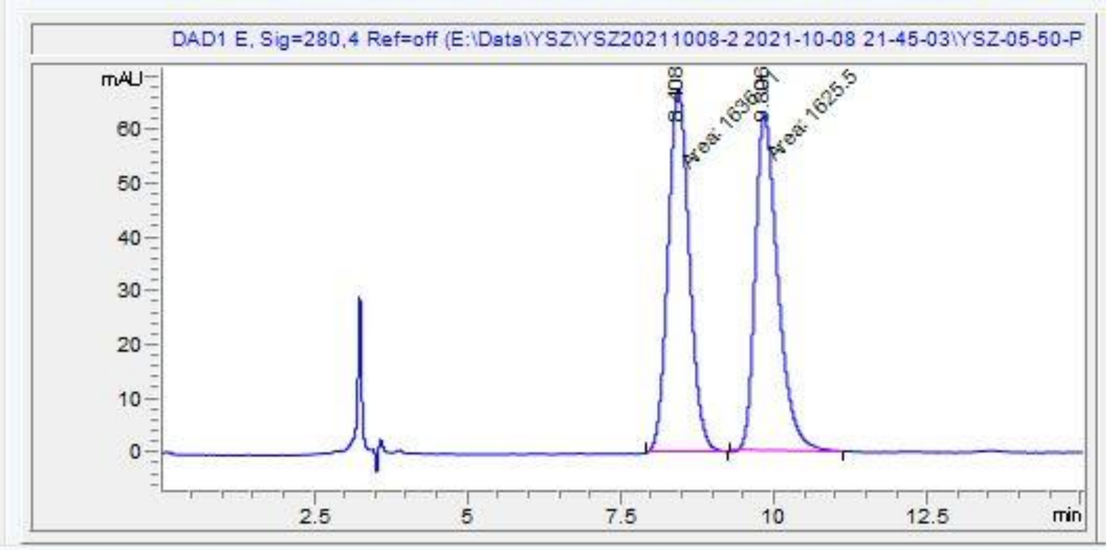

#	Time	Type	Area	Height	Width	Area%	Symmetry
1	8.408	MM	1636.7	67.8	0.4021	50.172	0.851
2	9.806	MM	1625.5	62.9	0.4306	49.828	0.609

Supplementary Fig. 90 Full HPLC spectrum of racemic **3k**

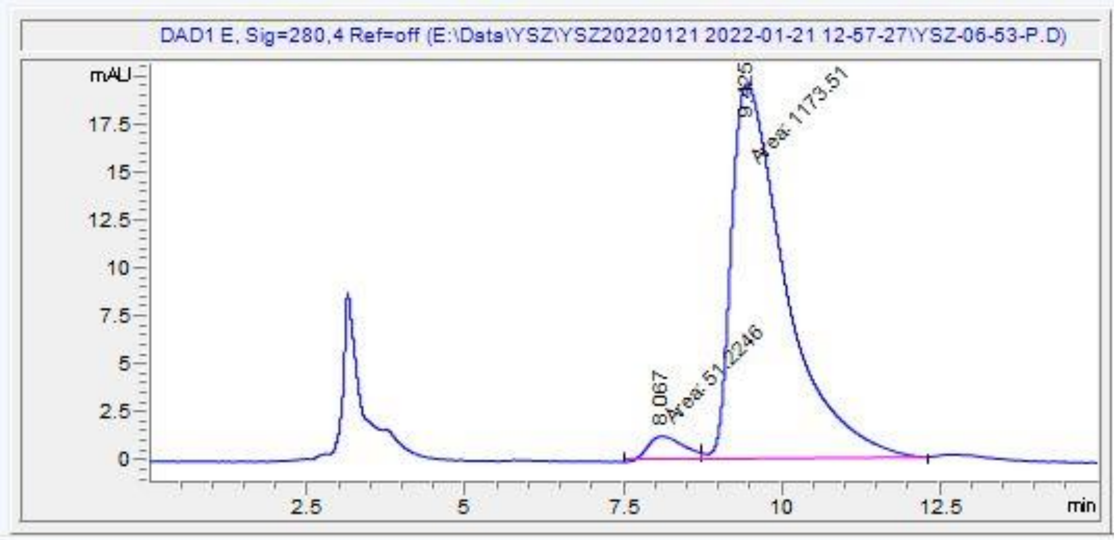

#	Time	Type	Area	Height	Width	Area%	Symmetry
1	8.067	MF	51.2	1.3	0.6637	4.183	0.588
2	9.425	FM	1173.5	19.6	0.996	95.817	0.403

Supplementary Fig. 91 Full HPLC spectrum of (S_p)-**3k**

(S_p)-4^2-((tert-butoxycarbonyl)amino)-1,4(1,4)-dibenzenacyclohexaphane-1^2-yl acetate **(1l)**

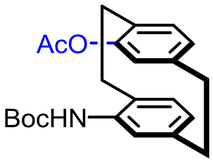

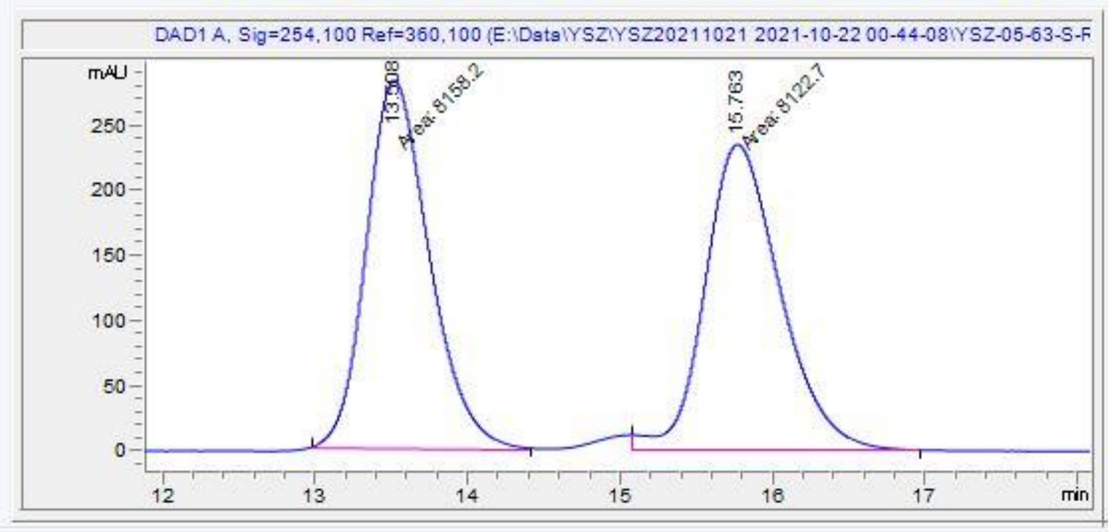

#	Time	Type	Area	Height	Width	Area%	Symmetry
1	13.508	MM	8158.2	282.9	0.4807	50.109	0.76
2	15.763	FM	8122.7	235.2	0.5755	49.891	0.754

Supplementary Fig. 92 HPLC spectrum of racemic **1l**

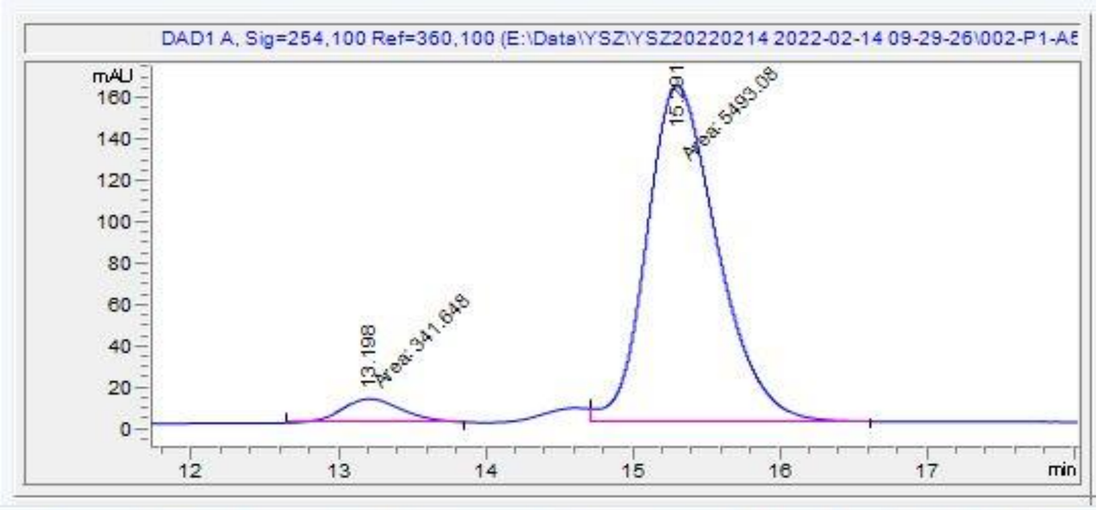

#	Time	Type	Area	Height	Width	Area%	Symmetry
1	13.198	MM	341.6	11.7	0.4851	5.855	0.825
2	15.291	FM	5493.1	163.9	0.5587	94.145	0.722

Supplementary Fig. 93 HPLC spectrum of (S_p)-**1l**

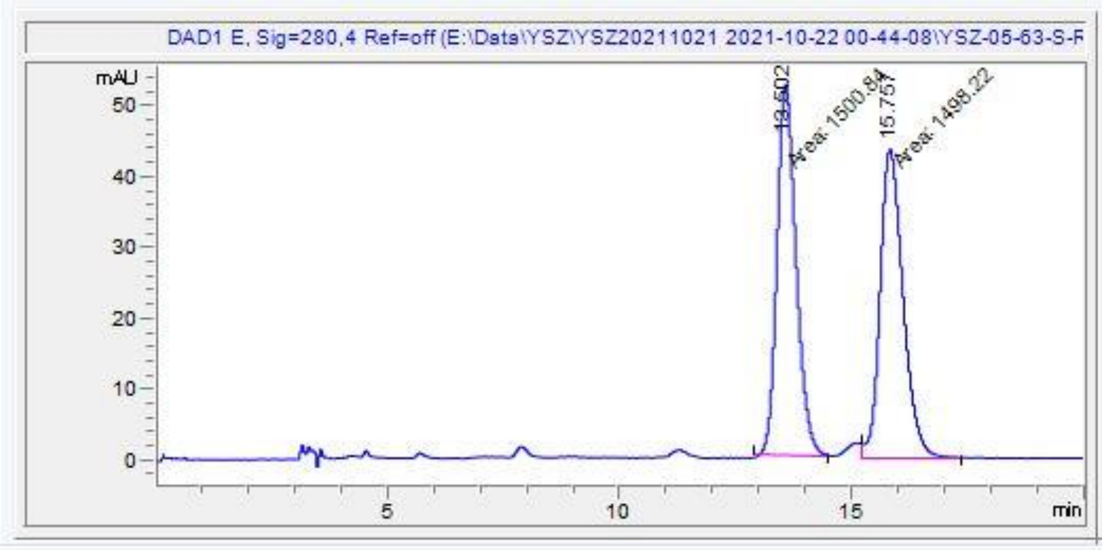

#	Time	Type	Area	Height	Width	Area%	Symmetry
1	13.502	MM	1500.8	52.3	0.4785	50.044	0.718
2	15.757	MM	1498.2	43.6	0.573	49.956	0.692

Supplementary Fig. 94 Full HPLC spectrum of racemic **1l**

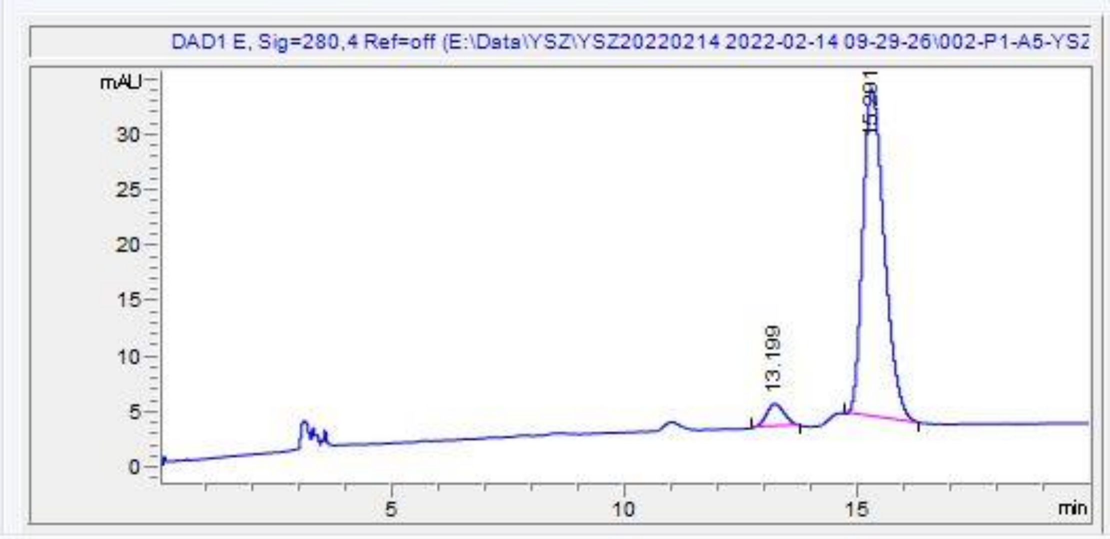

#	Time	Type	Area	Height	Width	Area%	Symmetry
1	13.199	BB	57.1	2.1	0.323	5.679	0.829
2	15.291	BB	947.8	29.5	0.3772	94.321	0.7

Supplementary Fig. 95 Full HPLC spectrum of (S_p)-**1l**

(R_p)-Dibenzyl 1-(4^3-acetoxy-1^5-((tert-butoxycarbonyl)amino)-1,4(1,4)-dibenzenacyclohexaphane-1^2-yl)hydrazine-1,2-dicarboxylate **(3l)**

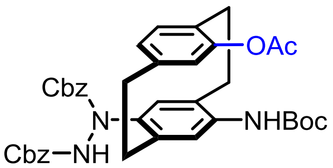

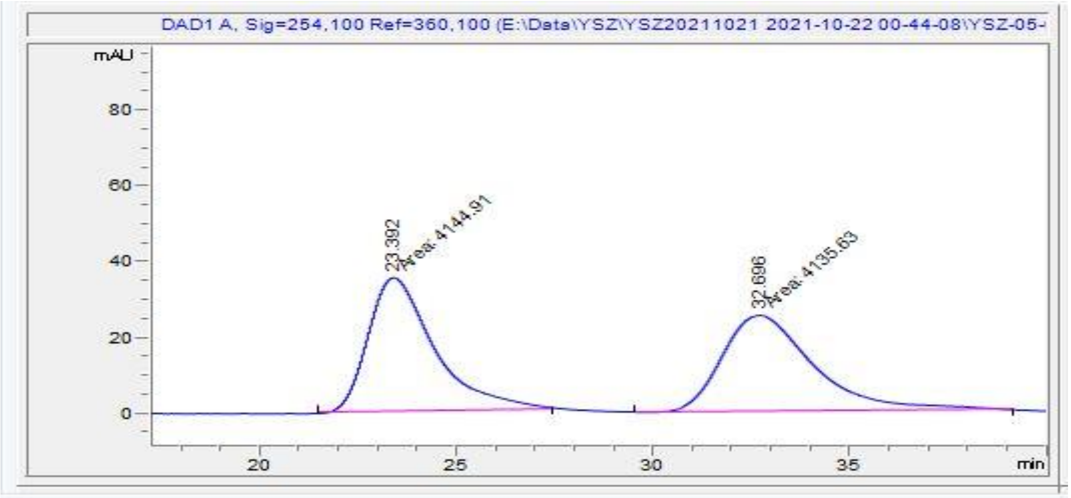

#	Time	Type	Area	Height	Width	Area%	Symmetry
1	23.392	MM	4144.9	35.2	1.9627	50.056	0.619
2	32.696	MM	4135.6	25.3	2.7198	49.944	0.672

Supplementary Fig. 96 HPLC spectrum of racemic **3l**

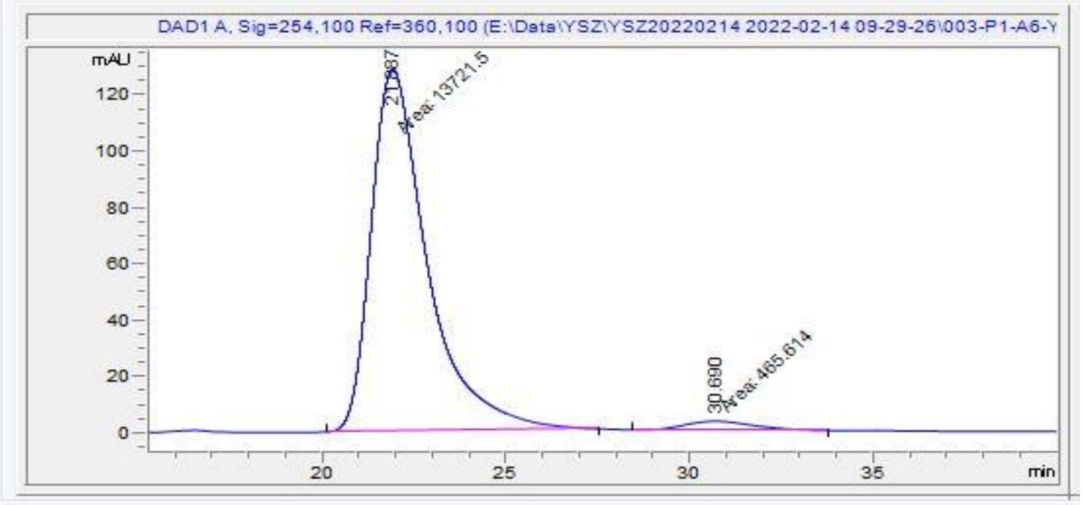

#	Time	Type	Area	Height	Width	Area%	Symmetry
1	21.887	MM	13721.5	128.4	1.7816	96.718	0.587
2	30.69	MM	465.6	3.3	2.3445	3.282	0.716

Supplementary Fig. 97 HPLC spectrum of (R_p)-**3l**

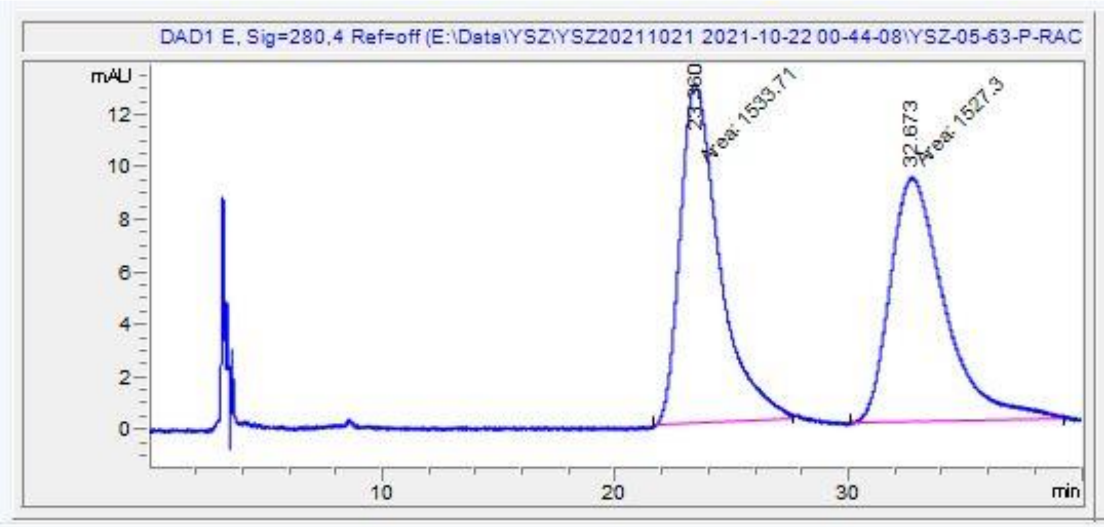

#	Time	Type	Area	Height	Width	Area%	Symmetry
1	23.36	MM	1533.7	13	1.9715	50.105	0.566
2	32.673	MM	1527.3	9.4	2.7182	49.895	0.635

Supplementary Fig. 98 Full HPLC spectrum of racemic **3l**

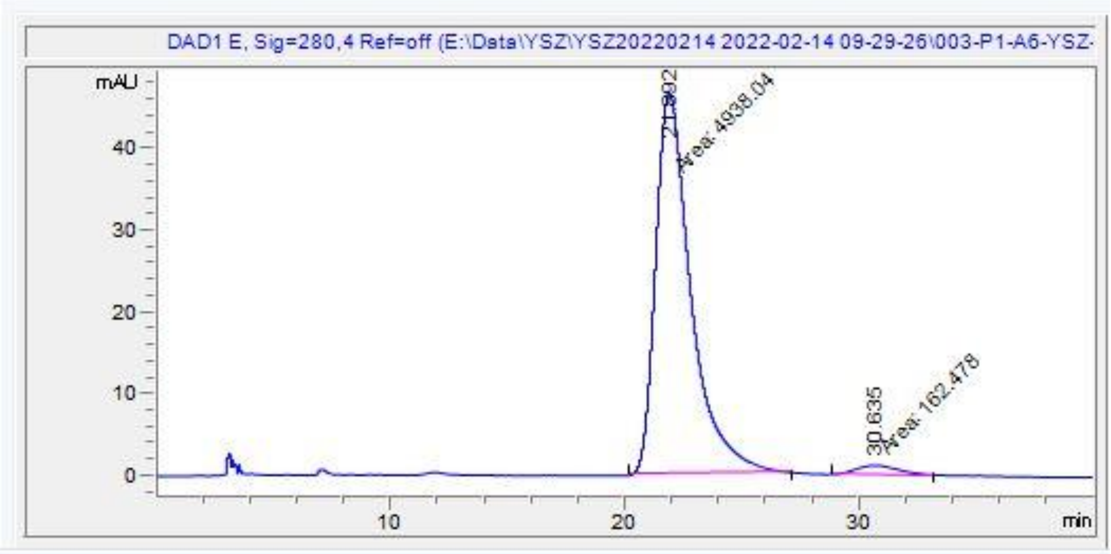

#	Time	Type	Area	Height	Width	Area%	Symmetry
1	21.892	MM	4938	46.9	1.7556	96.814	0.598
2	30.635	MM	162.5	1.2	2.1977	3.186	0.773

Supplementary Fig. 99 Full HPLC spectrum of (R_p)-**3l**

(R_p)-Tert-butyl (4^3-bromo-1,4(1,4)-dibenzenacyclohexaphane-1^2-yl)carbamate **(1m)**

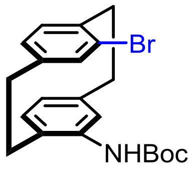

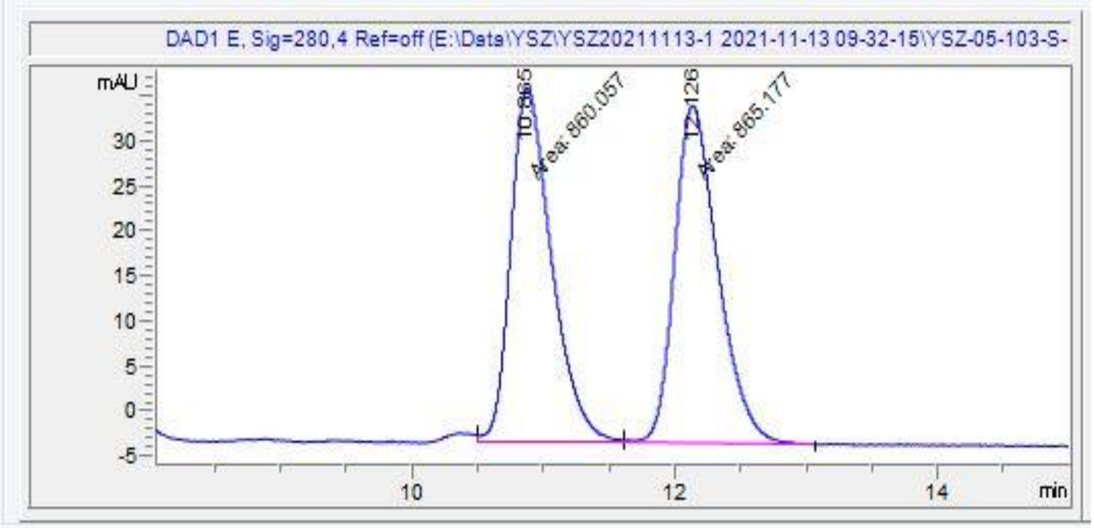

#	Time	Type	Area	Height	Width	Area%	Symmetry
1	10.865	MF	860.1	39.4	0.3636	49.852	0.642
2	12.126	FM	865.2	37.4	0.3851	50.148	0.72

Supplementary Fig. 100 HPLC spectrum of racemic **1m**

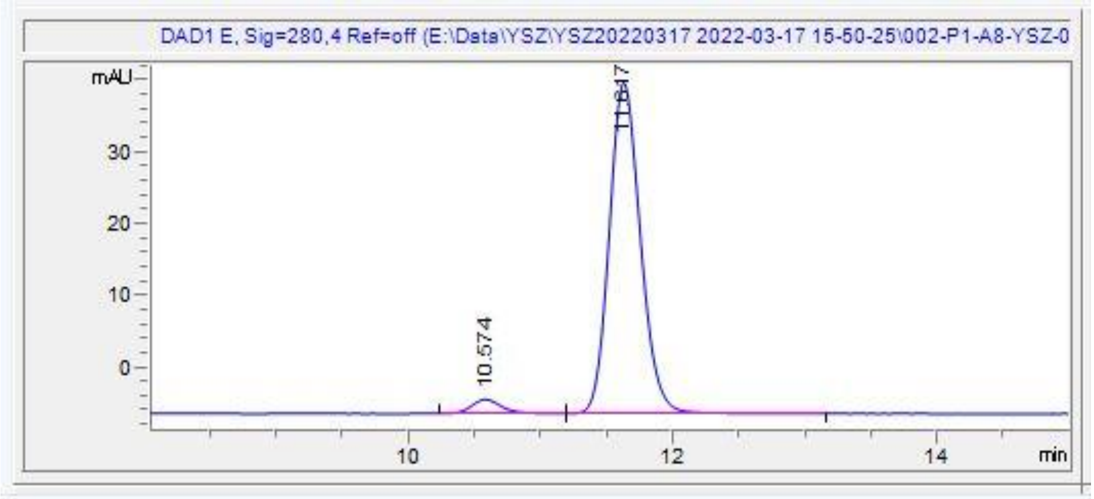

#	Time	Type	Area	Height	Width	Area%	Symmetry
1	10.574	BB	30.6	1.9	0.2435	3.911	0.83
2	11.617	BB	752.1	46.1	0.2524	96.089	0.823

\

Supplementary Fig. 101 HPLC spectrum of (R_p)-**1m**

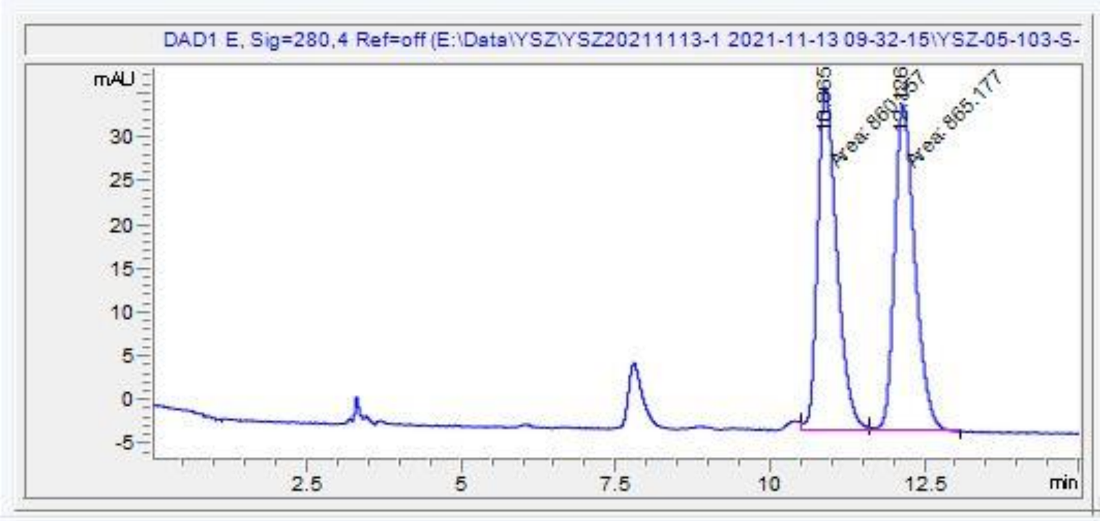

#	Time	Type	Area	Height	Width	Area%	Symmetry
1	10.865	MF	860.1	39.4	0.3636	49.852	0.642
2	12.126	FM	865.2	37.4	0.3851	50.148	0.72

Supplementary Fig. 102 Full HPLC spectrum of racemic **1m**

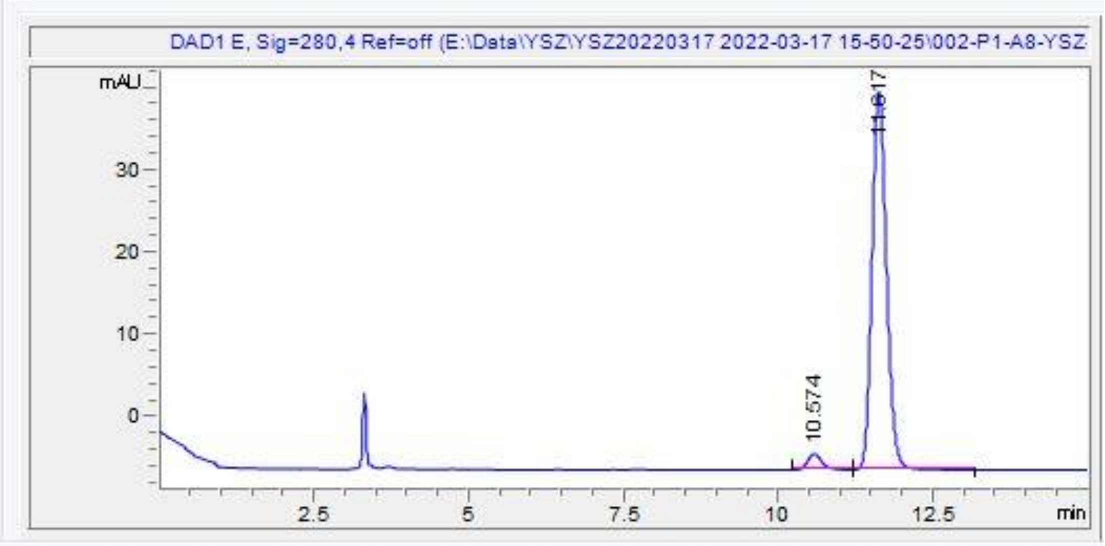

#	Time	Type	Area	Height	Width	Area%	Symmetry
1	10.574	BB	30.6	1.9	0.2435	3.911	0.83
2	11.617	BB	752.1	46.1	0.2524	96.089	0.823

Supplementary Fig. 103 Full HPLC spectrum of (R_p)-**1m**

(S_p)-Dibenzyl 1-(4^2-bromo-1^5-((tert-butoxycarbonyl)amino)-1,4(1,4)-dibenzenacyclohexaphane-1^2-yl)hydrazine-1,2-dicarboxylate **(3m)**

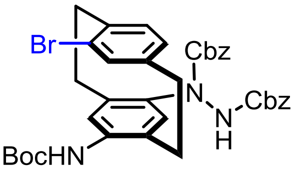

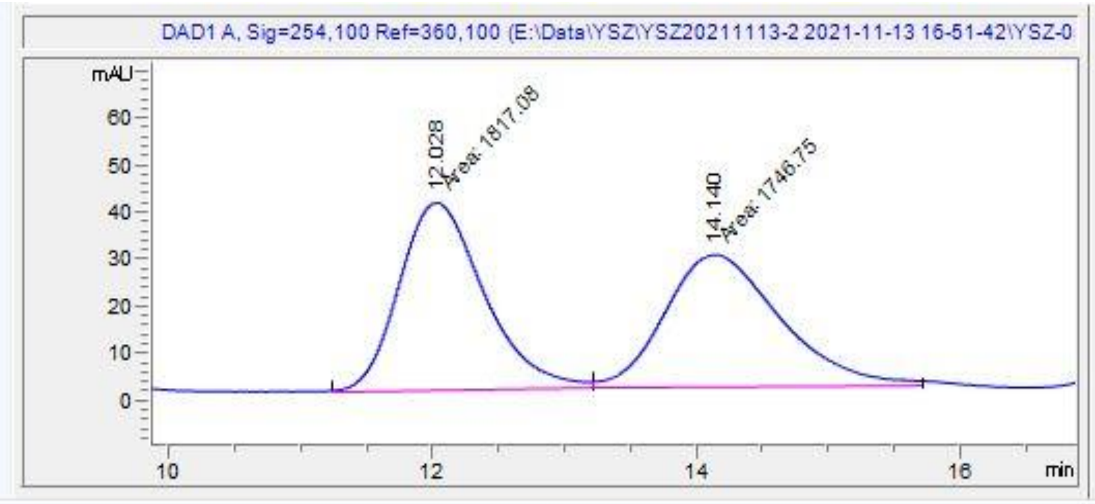

#	Time	Type	Area	Height	Width	Area%	Symmetry
1	12.028	MF	1817.1	40.1	0.7545	50.987	0.775
2	14.14	FM	1746.8	28.4	1.0266	49.013	0.766

Supplementary Fig. 104 HPLC spectrum of racemic **3m**

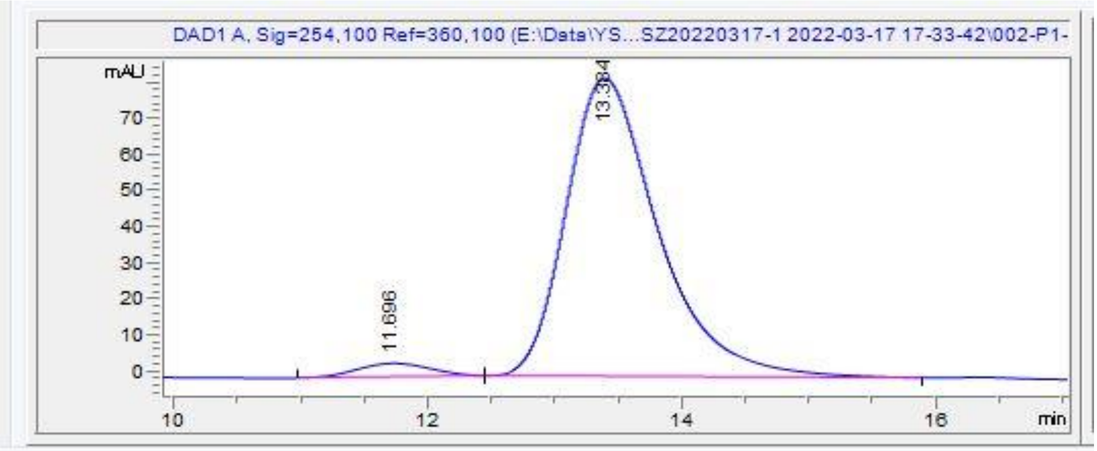

#	Time	Type	Area	Height	Width	Area%	Symmetry
1	11.696	BB	152	3.8	0.4726	3.510	0.888
2	13.384	BB	4178.1	82.9	0.6184	96.490	0.686

Supplementary Fig. 105 HPLC spectrum of (S_p)-**3m**

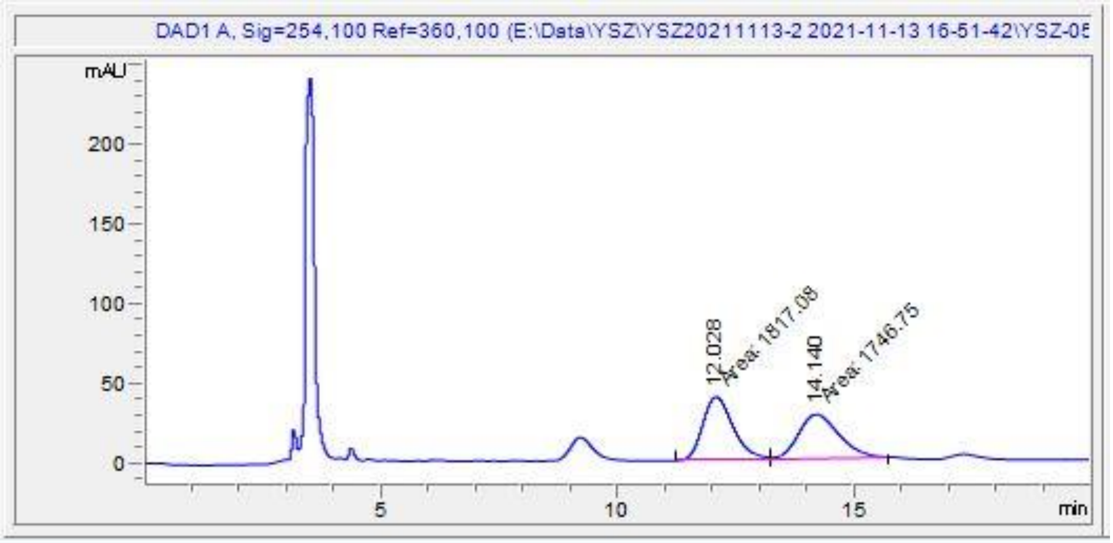

#	Time	Type	Area	Height	Width	Area%	Symmetry
1	12.028	MF	1817.1	40.1	0.7545	50.987	0.775
2	14.14	FM	1746.8	28.4	1.0266	49.013	0.766

Supplementary Fig. 106 Full HPLC spectrum of racemic **3m**

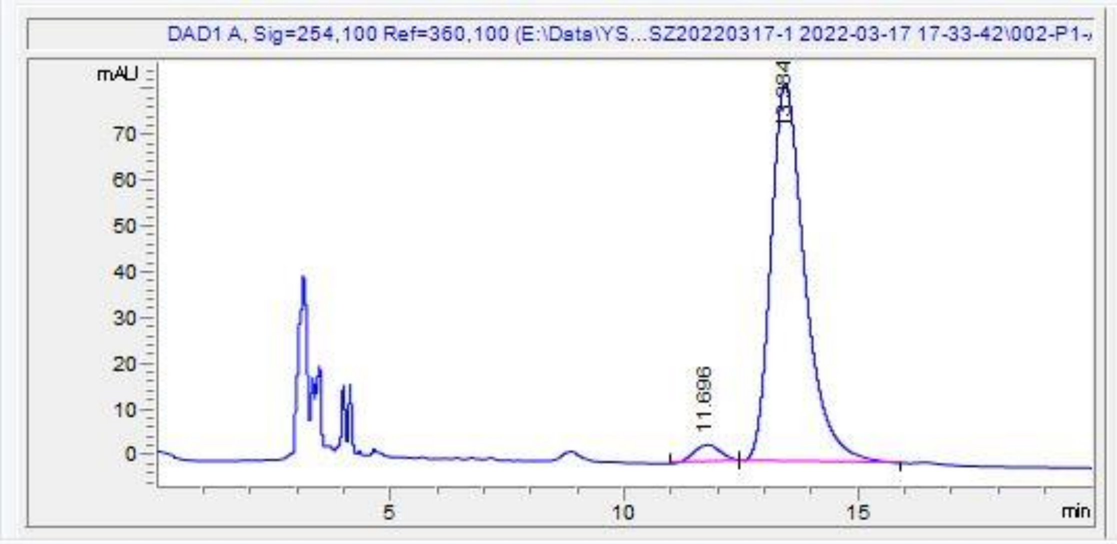

#	Time	Type	Area	Height	Width	Area%	Symmetry
1	11.696	BB	152	3.8	0.4726	3.510	0.888
2	13.384	BB	4178.1	82.9	0.6184	96.490	0.686

Supplementary Fig. 107 Full HPLC spectrum of (S_p)-**3m**

(R_p)-Tert-butyl (4^3-phenyl-1,4(1,4)-dibenzenacyclohexaphane-1^2-yl)carbamate **(1n)**

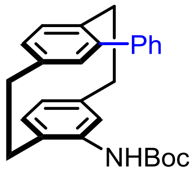

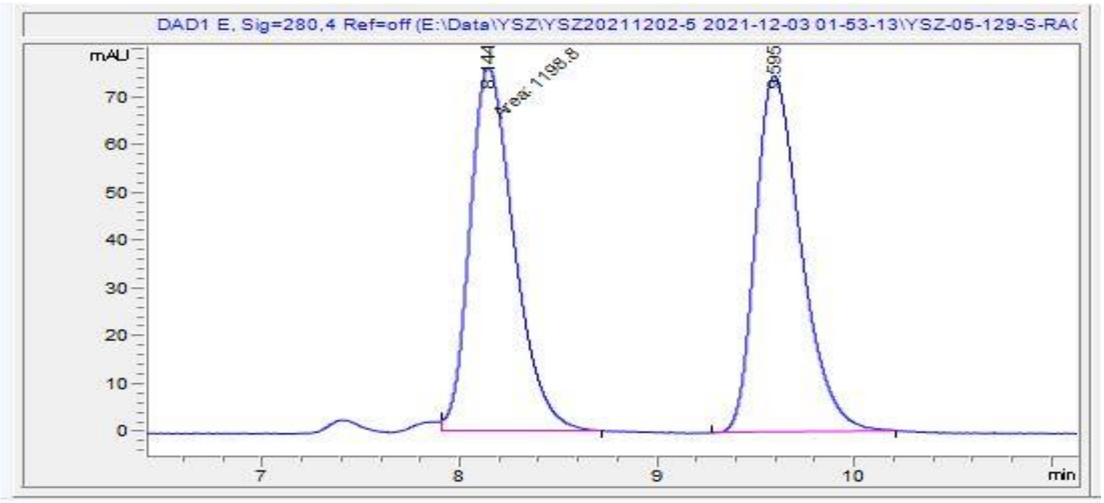

#	Time	Type	Area	Height	Width	Area%	Symmetry
1	8.144	FM	1198.8	76.5	0.2612	49.943	0.724
2	9.595	BV R	1201.6	74.8	0.1893	50.057	0.699

Supplementary Fig. 108 HPLC spectrum of racemic **1n**

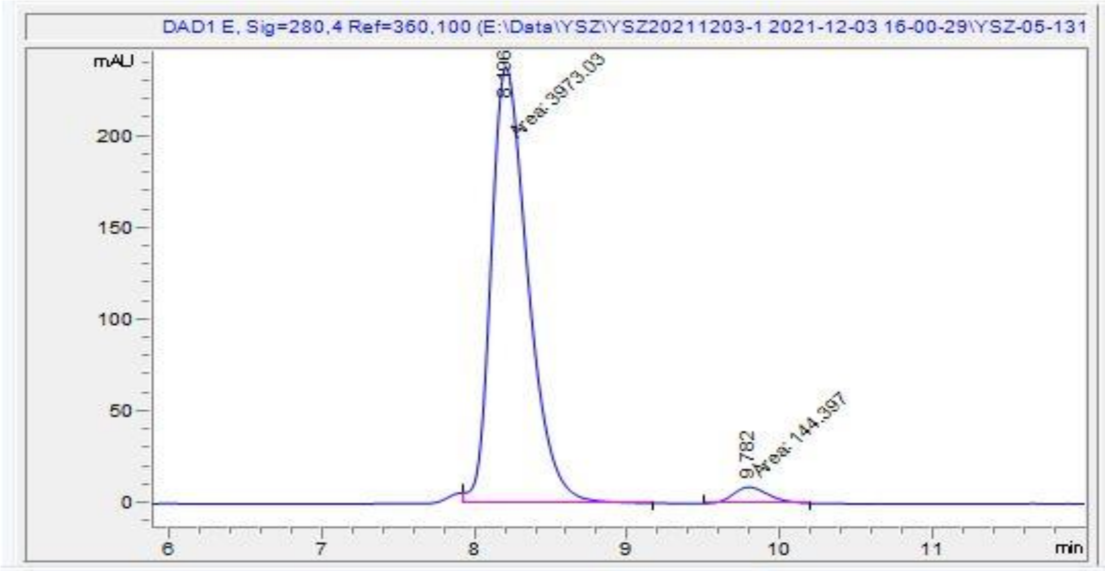

#	Time	Type	Area	Height	Width	Area%	Symmetry
1	8.196	FM	3973	237.3	0.2791	96.493	0.649
2	9.782	MM	144.4	8.9	0.2716	3.507	0.738

Supplementary Fig. 109 HPLC spectrum of (R_p)-**1n**

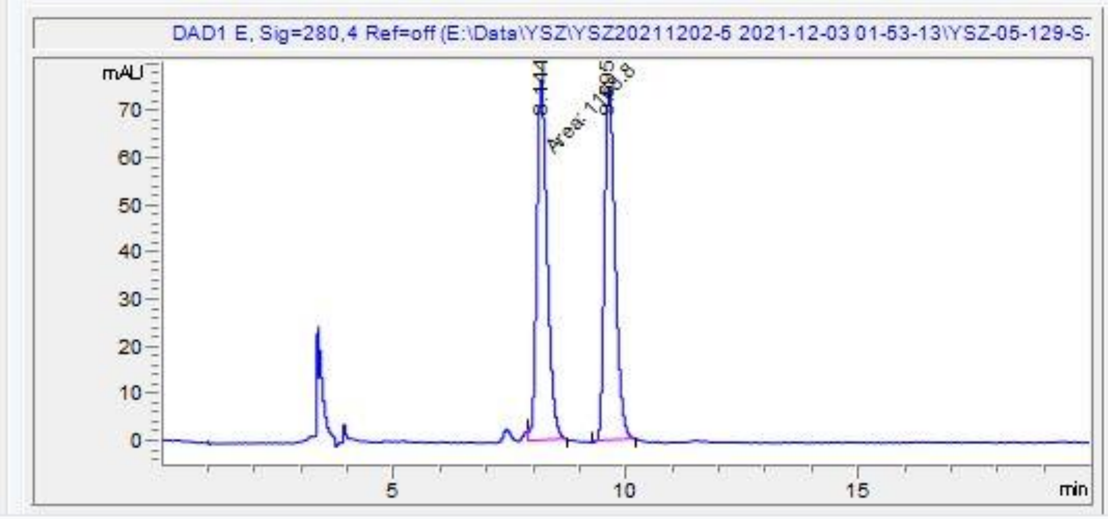

#	Time	Type	Area	Height	Width	Area%	Symmetry
1	8.144	FM	1198.8	76.5	0.2612	49.943	0.724
2	9.595	BV R	1201.6	74.8	0.1893	50.057	0.699

Supplementary Fig. 110 Full HPLC spectrum of racemic **1n**

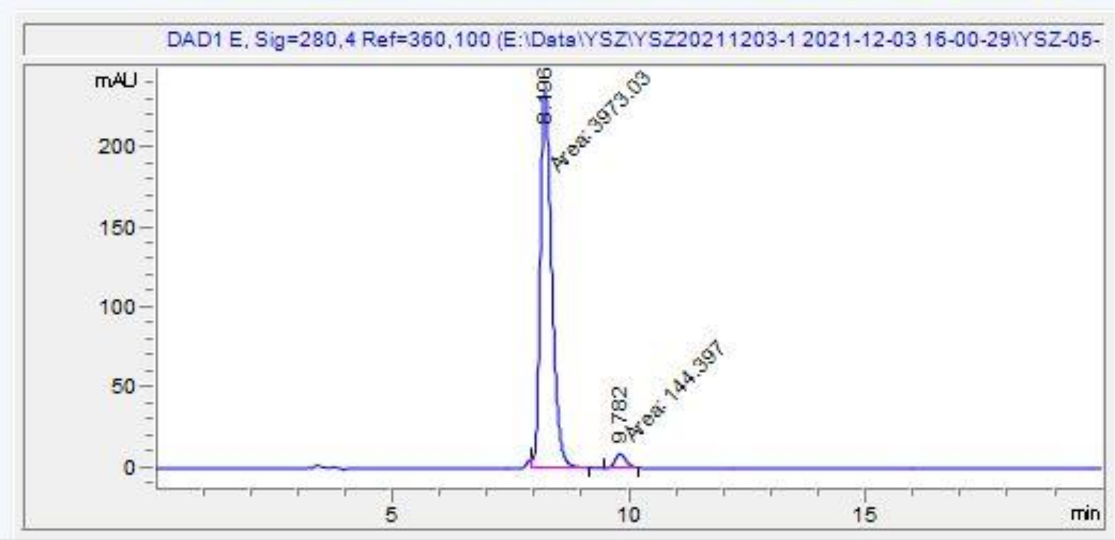

#	Time	Type	Area	Height	Width	Area%	Symmetry
1	8.196	FM	3973	237.3	0.2791	96.493	0.649
2	9.782	MM	144.4	8.9	0.2716	3.507	0.738

Supplementary Fig. 111 Full HPLC spectrum of (R_p)-**1n**

(S_p)-Dibenzyl-1-(1^5-((tert-butoxycarbonyl)amino)-4^2-phenyl-1,4(1,4)-dibenzenacyclo hexaphane-1^2-yl)hydrazine-1,2-dicarboxylate **(3n)**

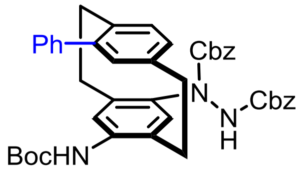

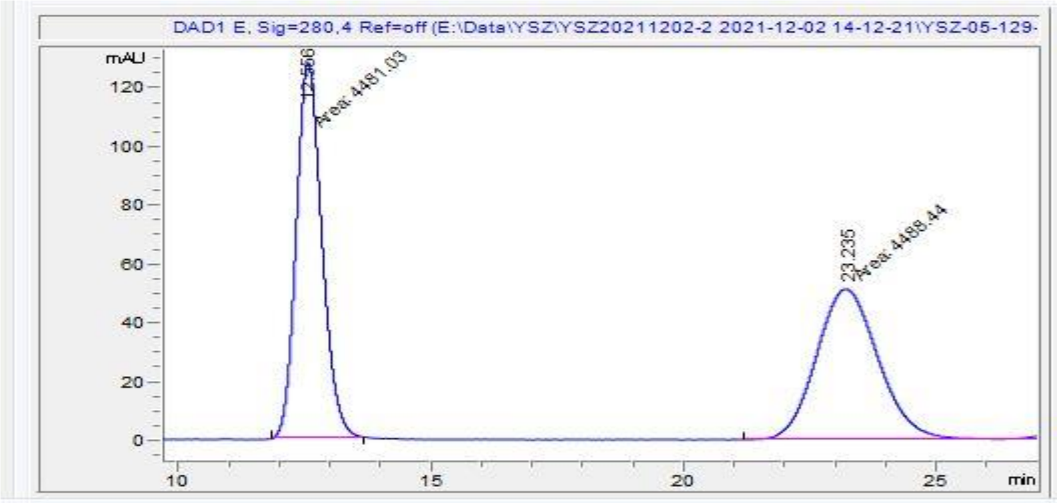

#	Time	Type	Area	Height	Width	Area%	Symmetry
1	12.556	MM	4481	127.4	0.5863	49.959	0.825
2	23.235	MM	4488.4	51.2	1.46	50.041	0.871

Supplementary Fig. 112 HPLC spectrum of racemic **3n**

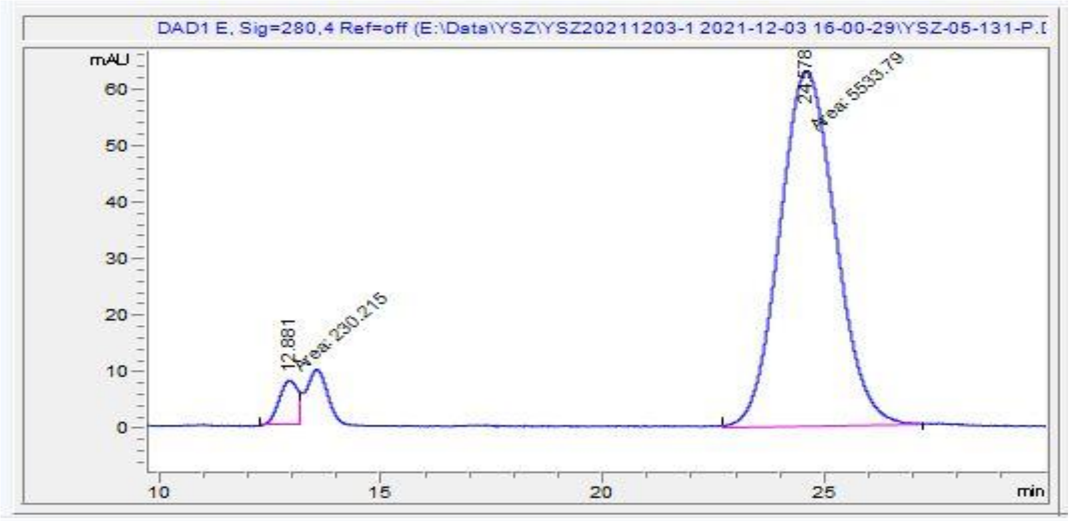

#	Time	Type	Area	Height	Width	Area%	Symmetry
1	12.881	MF	230.2	7.9	0.4849	3.994	0.917
2	24.578	MM	5533.8	62.7	1.4714	96.006	0.887

Supplementary Fig. 113 HPLC spectrum of (S_p)-**3n**

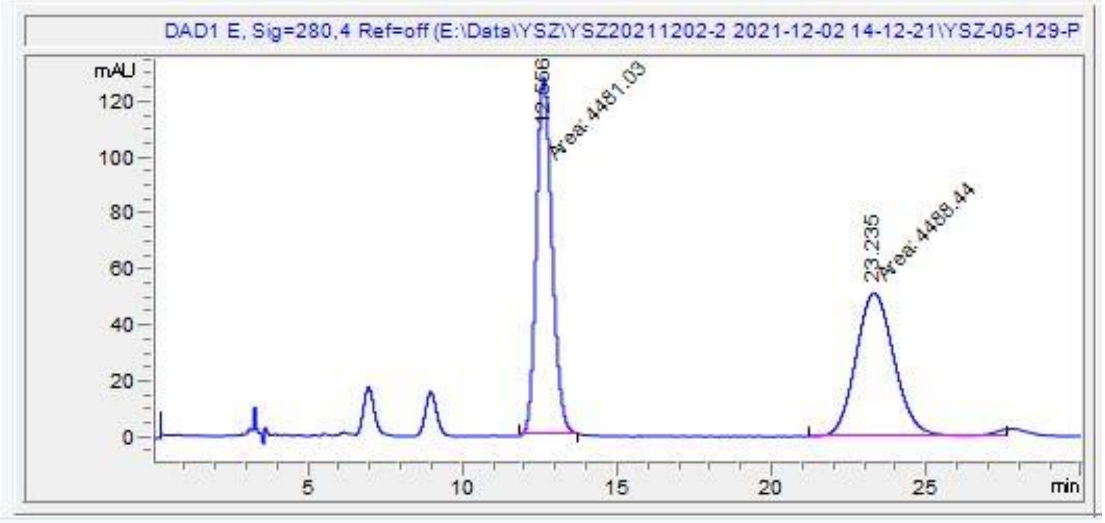

#	Time	Type	Area	Height	Width	Area%	Symmetry
1	12.556	MM	4481	127.4	0.5863	49.959	0.825
2	23.235	MM	4488.4	51.2	1.46	50.041	0.871

Supplementary Fig. 114 Full HPLC spectrum of racemic **3n**

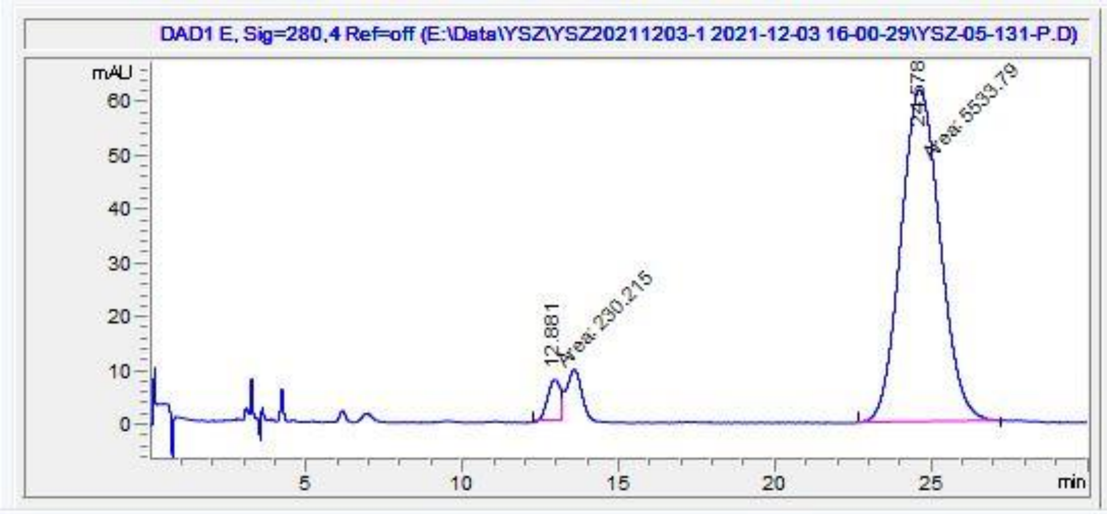

#	Time	Type	Area	Height	Width	Area%	Symmetry
1	12.881	MF	230.2	7.9	0.4849	3.994	0.917
2	24.578	MM	5533.8	62.7	1.4714	96.006	0.887

Supplementary Fig. 115 Full HPLC spectrum of (S_p)-**3n**

(R_p)-Tert-butyl (4^3-(cyclohex-1-en-1-yl)-1,4(1,4)-dibenzenacyclohexaphane-1^2 -yl)carbamate **(1o)**

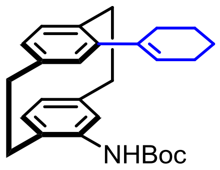

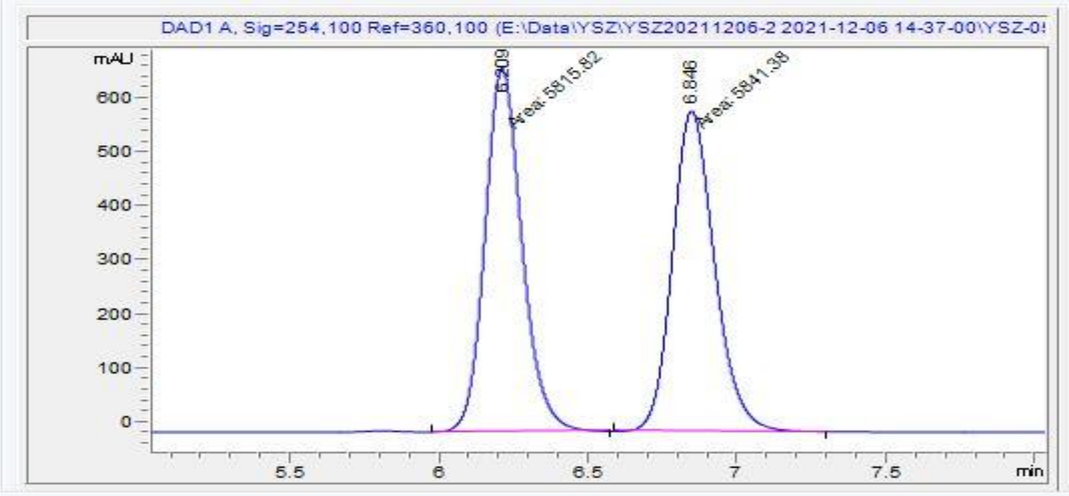

#	Time	Type	Area	Height	Width	Area%	Symmetry
1	6.209	MM	5815.8	672.9	0.144	49.890	0.863
2	6.846	MM	5841.4	593.1	0.1642	50.110	0.836

Supplementary Fig. 116 HPLC spectrum of racemic **1o**

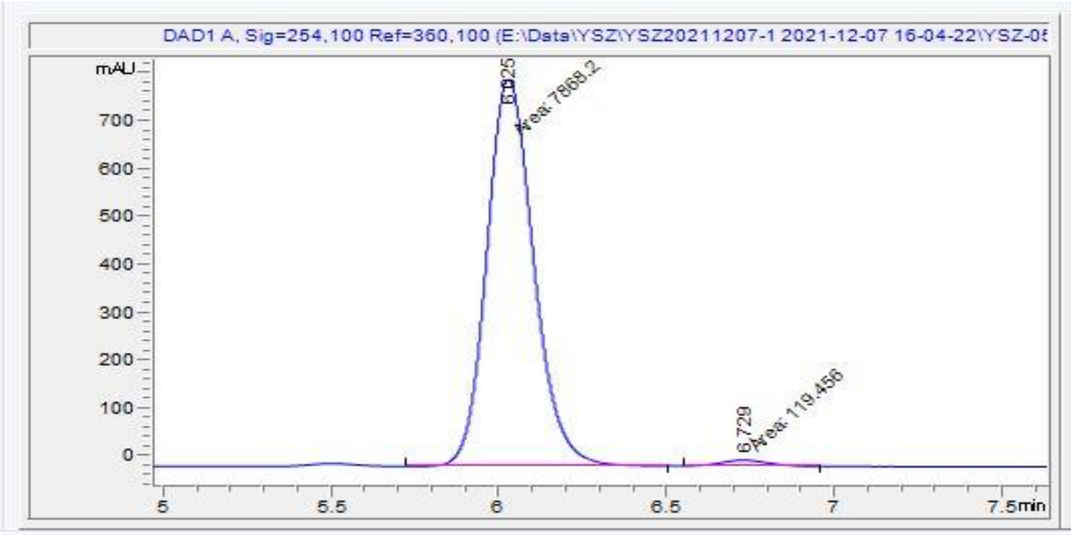

#	Time	Type	Area	Height	Width	Area%	Symmetry
1	6.025	MM	7868.2	808.5	0.1622	98.504	0.806
2	6.729	MM	119.5	12.3	0.1614	1.496	1.006

Supplementary Fig. 117 HPLC spectrum of (R_p)-**1o**

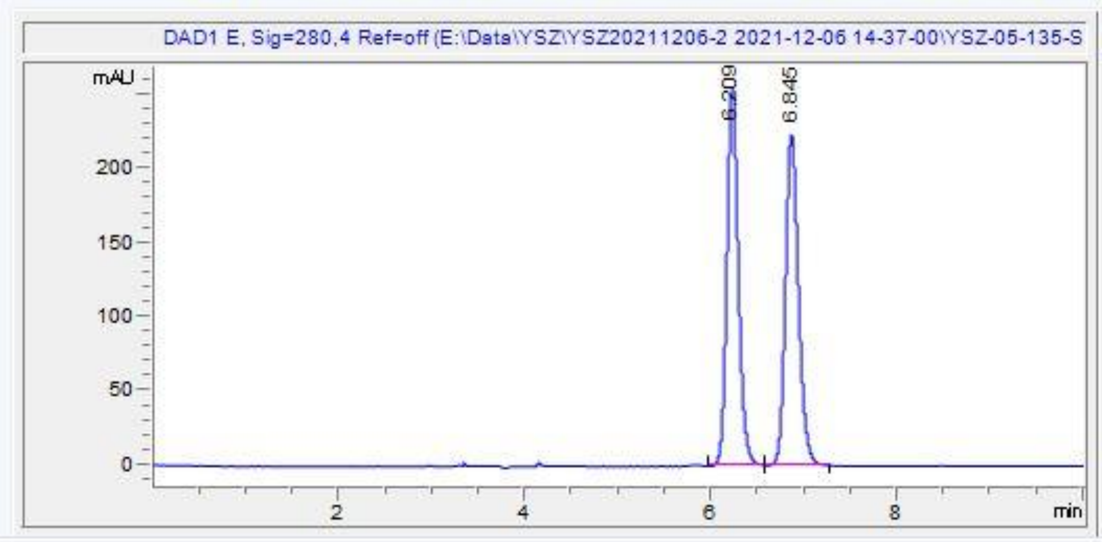

#	Time	Type	Area	Height	Width	Area%	Symmetry
1	6.209	BB	2195.7	256.5	0.1309	50.010	0.822
2	6.845	BV R	2194.9	224.7	0.1495	49.990	0.792

Supplementary Fig. 118 Full HPLC spectrum of racemic **1o**

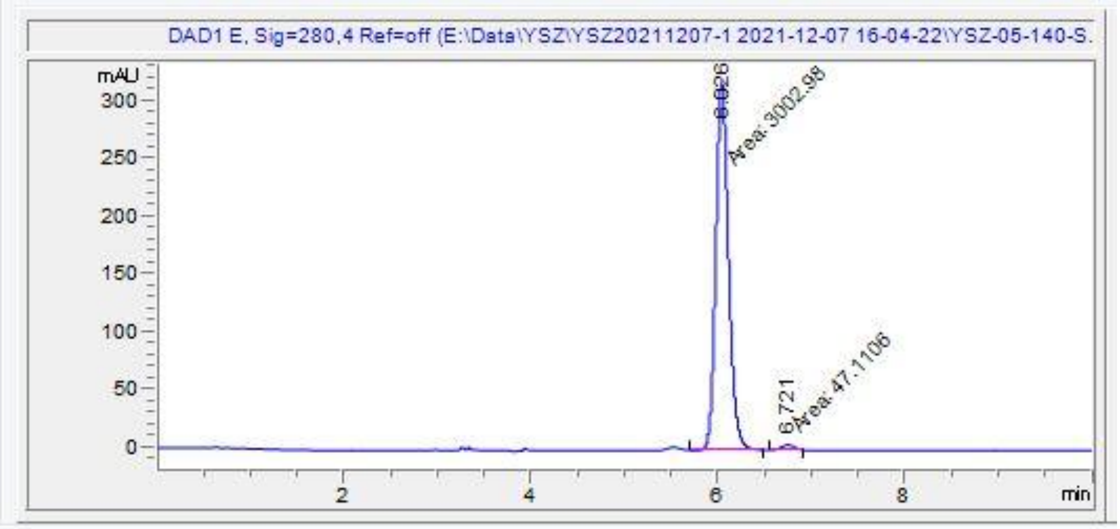

#	Time	Type	Area	Height	Width	Area%	Symmetry
1	6.026	MM	3003	317.9	0.1574	98.455	0.842
2	6.721	MM	47.1	4.8	0.1643	1.545	0.872

Supplementary Fig. 119 Full HPLC spectrum of (R_p)-**1o**

(S_p)-Dibenzyl 1-(1[5]-((tert-butoxycarbonyl)amino)-4[2]-(cyclohex-1-en-1-yl)-1,4(1,4)-dibenzenacyclohexaphane-1[2]-yl)hydrazine-1,2-dicarboxylate **(3o)**

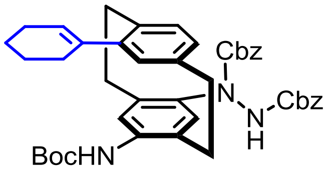

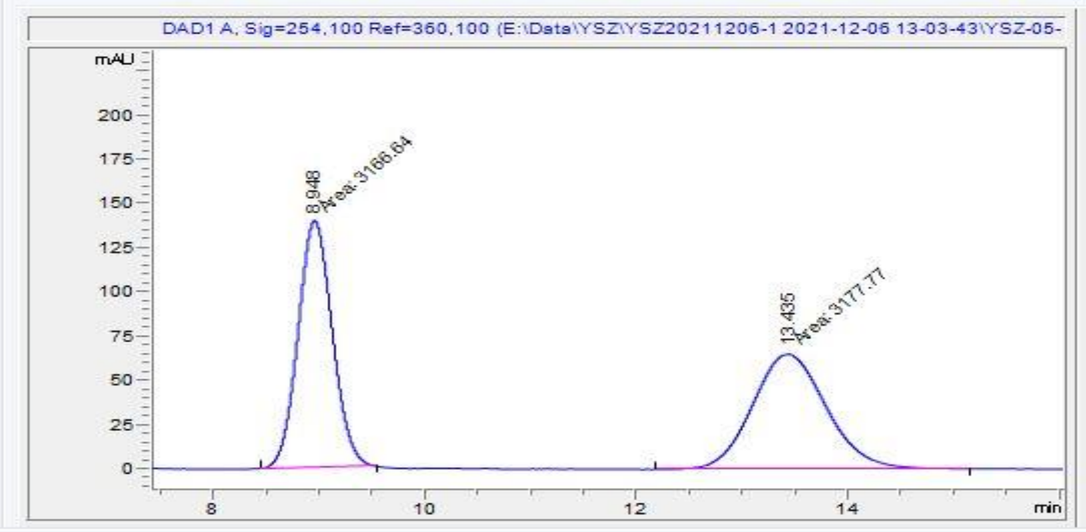

#	Time	Type	Area	Height	Width	Area%	Symmetry
1	8.948	MM	3166.6	139.9	0.3774	49.912	0.909
2	13.435	MM	3177.8	65.1	0.8142	50.088	0.911

Supplementary Fig. 120 HPLC spectrum of racemic **3o**

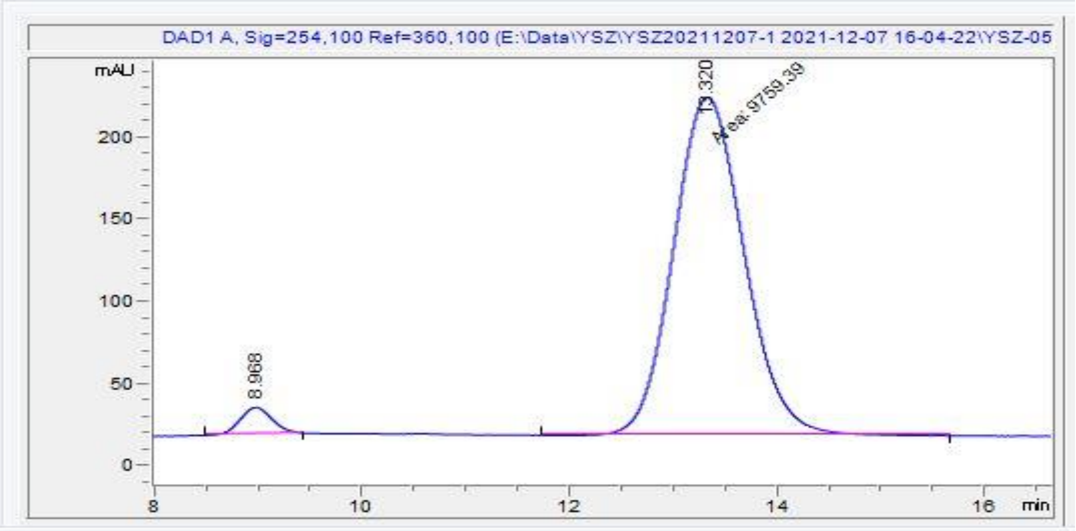

#	Time	Type	Area	Height	Width	Area%	Symmetry
1	8.968	BV R	350.8	16.3	0.2527	3.470	0.949
2	13.32	MM	9759.4	206	0.7894	96.530	0.905

Supplementary Fig. 121 HPLC spectrum of (S_p)-**3o**

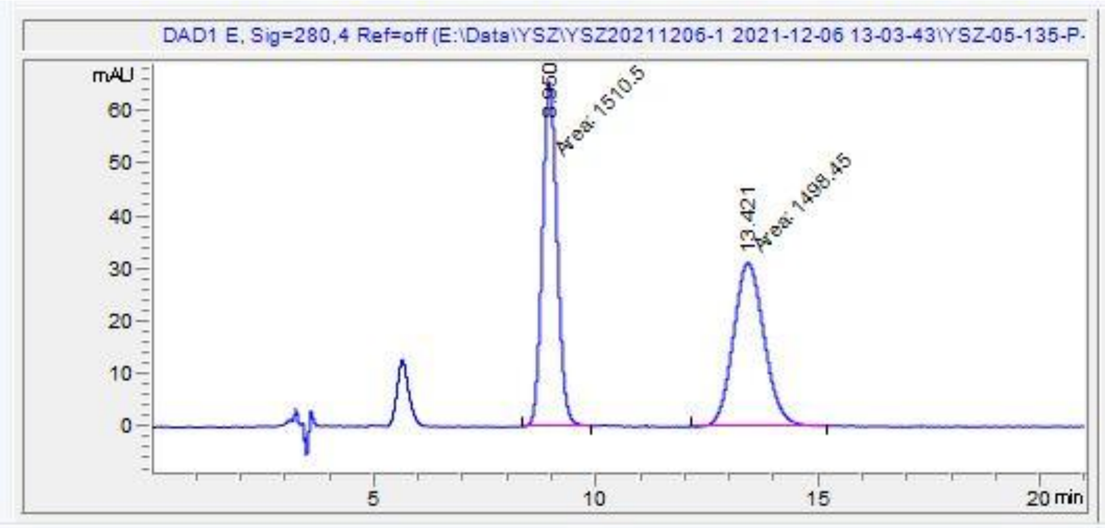

#	Time	Type	Area	Height	Width	Area%	Symmetry
1	8.95	MM	1510.5	65.3	0.3856	50.200	0.907
2	13.421	MM	1498.5	31	0.805	49.800	0.854

Supplementary Fig. 122 Full HPLC spectrum of racemic **3o**

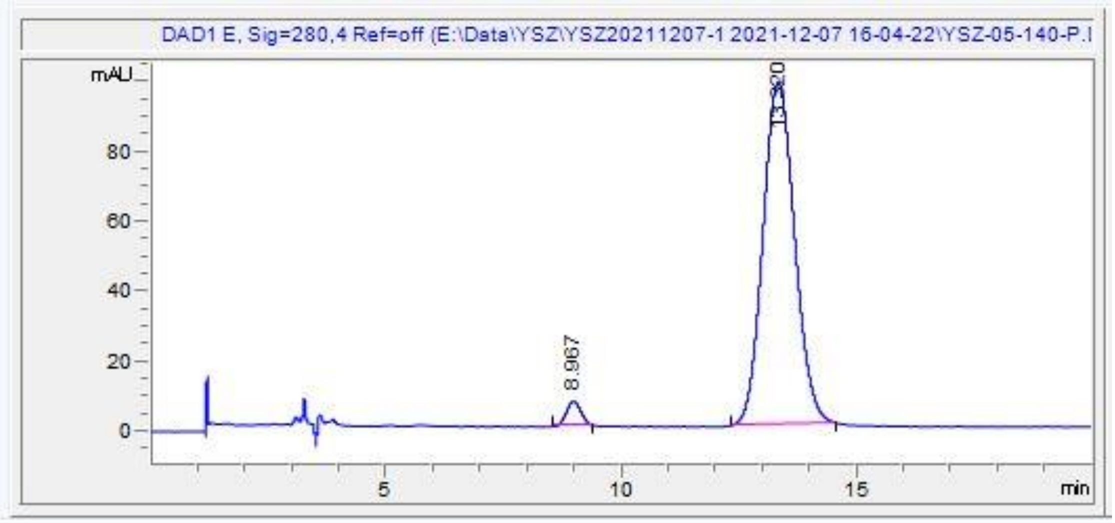

#	Time	Type	Area	Height	Width	Area%	Symmetry
1	8.967	VV R	154.2	7.1	0.2554	3.262	0.951
2	13.32	VV R	4574.6	98.1	0.5493	96.738	0.909

Supplementary Fig. 123 Full HPLC spectrum of (S_p)-**3o**

(R_p)-Tert-butyl (E)-(4[3]-styryl-1,4(1,4)-dibenzenacyclohexaphane-1[2]-yl)carbamate **(1p)**

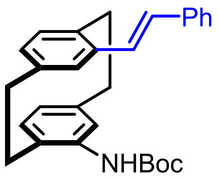

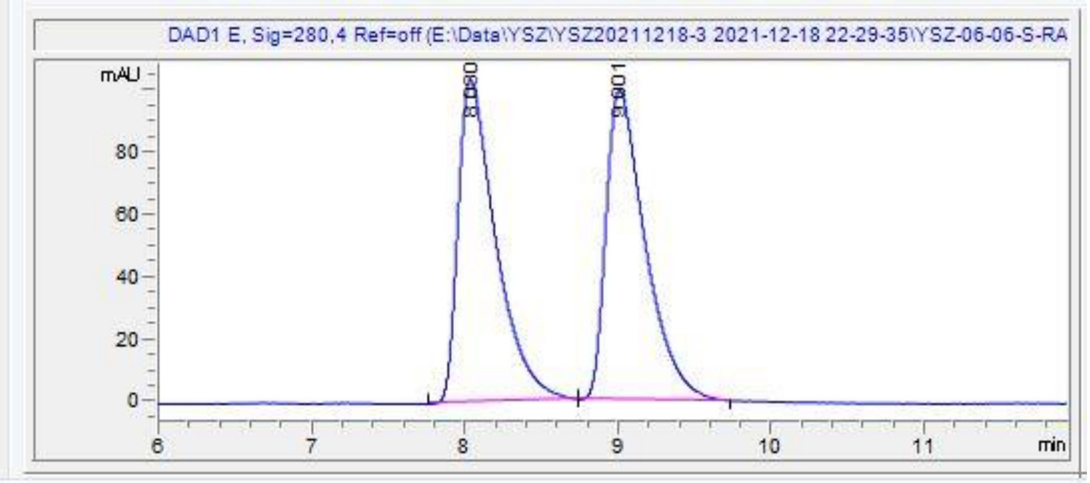

#	Time	Type	Area	Height	Width	Area%	Symmetry
1	8.03	BB	1790.7	103.8	0.2058	50.141	0.506
2	9.001	BV R	1780.6	99.7	0.2099	49.859	0.532

Supplementary Fig. 124 HPLC spectrum of racemic **1p**

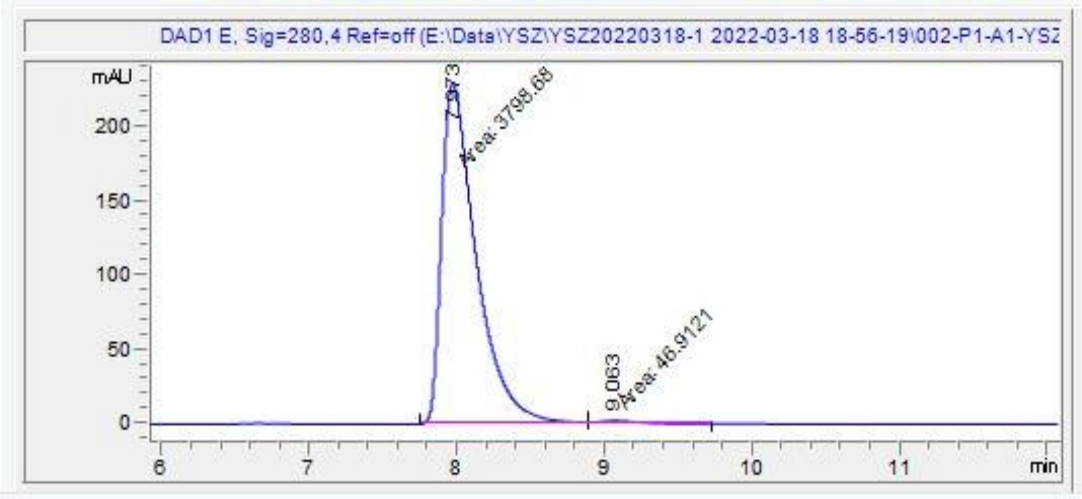

#	Time	Type	Area	Height	Width	Area%	Symmetry
1	7.973	MF	3798.7	230	0.2753	98.780	0
2	9.063	FM	46.9	2.3	0.3406	1.220	0.648

Supplementary Fig. 125 HPLC spectrum of (R_p)-**1p**

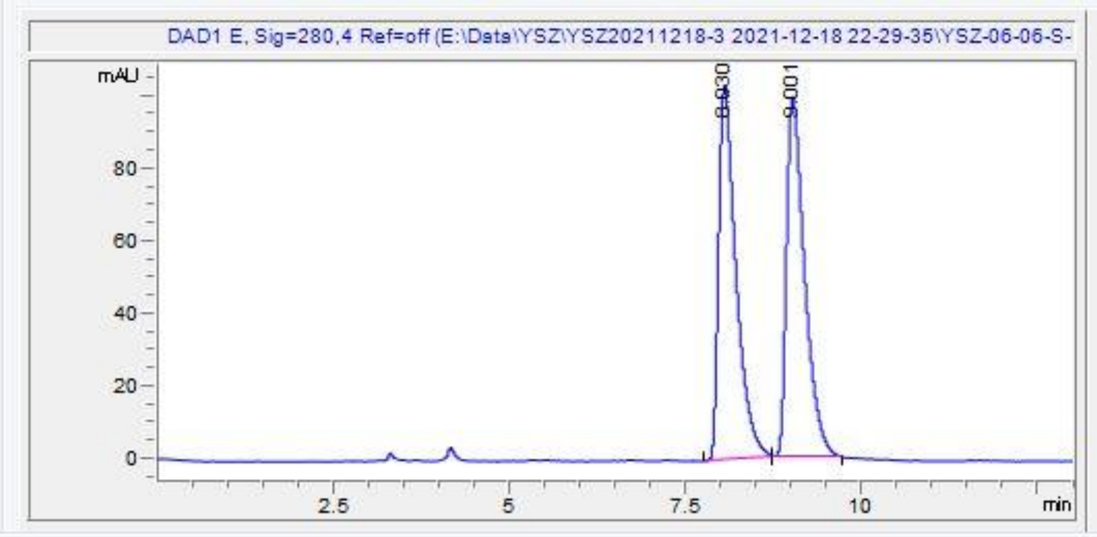

#	Time	Type	Area	Height	Width	Area%	Symmetry
1	8.03	BB	1790.7	103.8	0.2058	50.141	0.506
2	9.001	BV R	1780.6	99.7	0.2099	49.859	0.532

Supplementary Fig. 126 Full HPLC spectrum of racemic **1p**

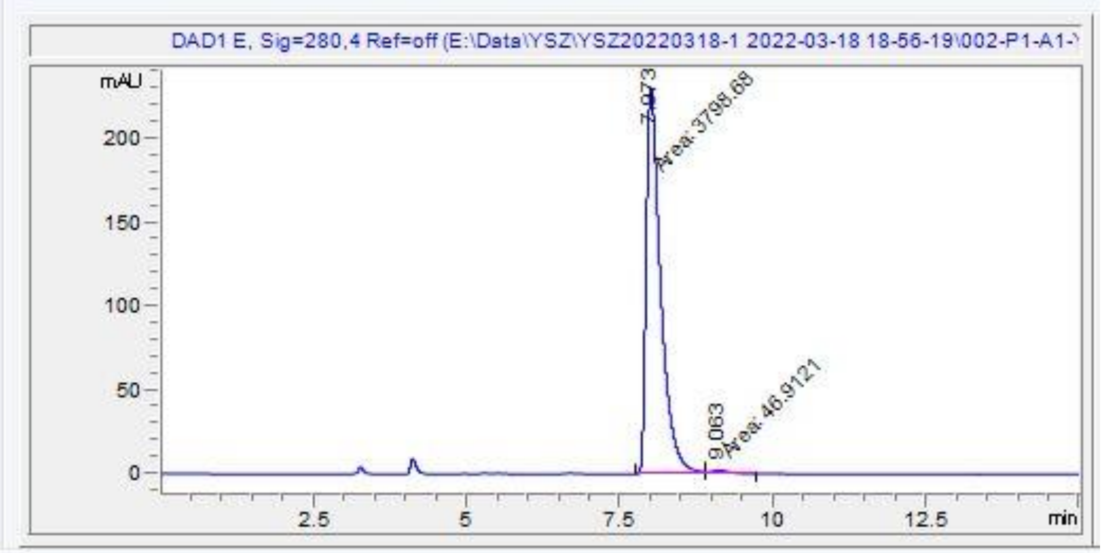

#	Time	Type	Area	Height	Width	Area%	Symmetry
1	7.973	MF	3798.7	230	0.2753	98.780	0
2	9.063	FM	46.9	2.3	0.3406	1.220	0.648

Supplementary Fig. 127 Full HPLC spectrum of (R_p)-**1p**

(S_p)-Dibenzyl (E)-1-(1^5-((tert-butoxycarbonyl)amino)-4^2-styryl-1,4(1,4) -dibenzenacyclohexaphane-1^2-yl)hydrazine-1,2-dicarboxylate **(3p)**

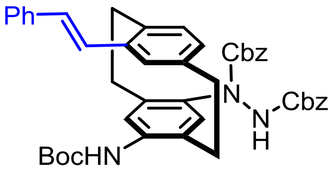

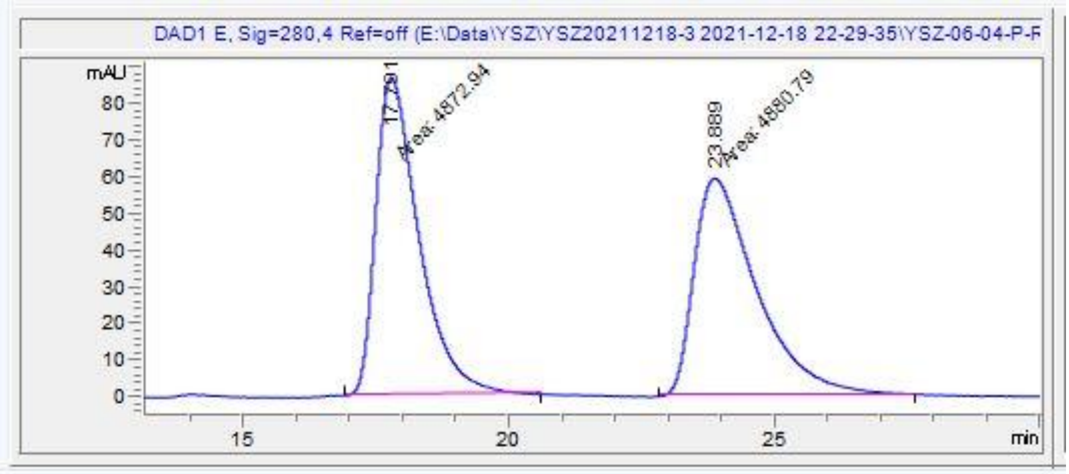

#	Time	Type	Area	Height	Width	Area%	Symmetry
1	17.791	MM	4872.9	86.2	0.9426	49.960	0.582
2	23.889	MM	4880.8	59.3	1.3714	50.040	0.505

Supplementary Fig. 128 HPLC spectrum of racemic **3p**

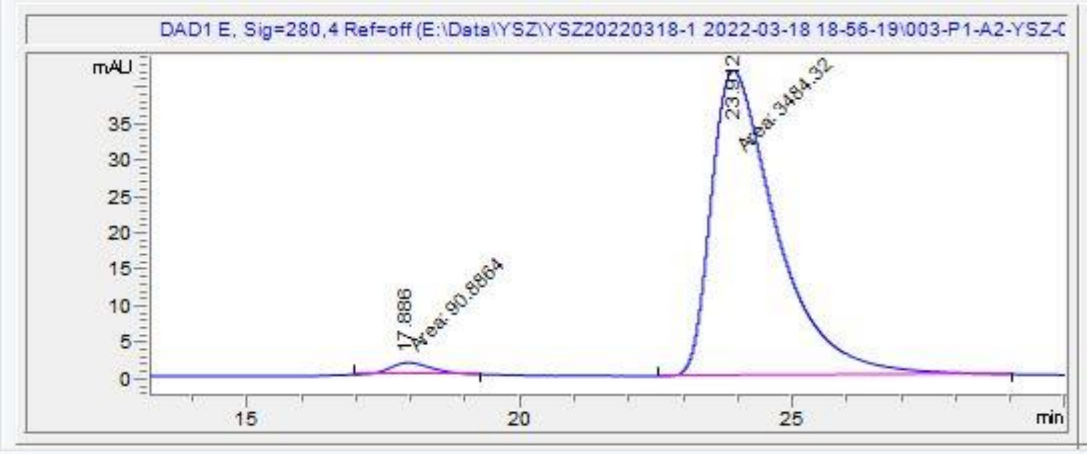

#	Time	Type	Area	Height	Width	Area%	Symmetry
1	17.886	MM	90.9	1.6	0.9209	2.542	0.624
2	23.912	MM	3484.3	41.6	1.3973	97.458	0.488

Supplementary Fig. 129 HPLC spectrum of (S_p)-**3p**

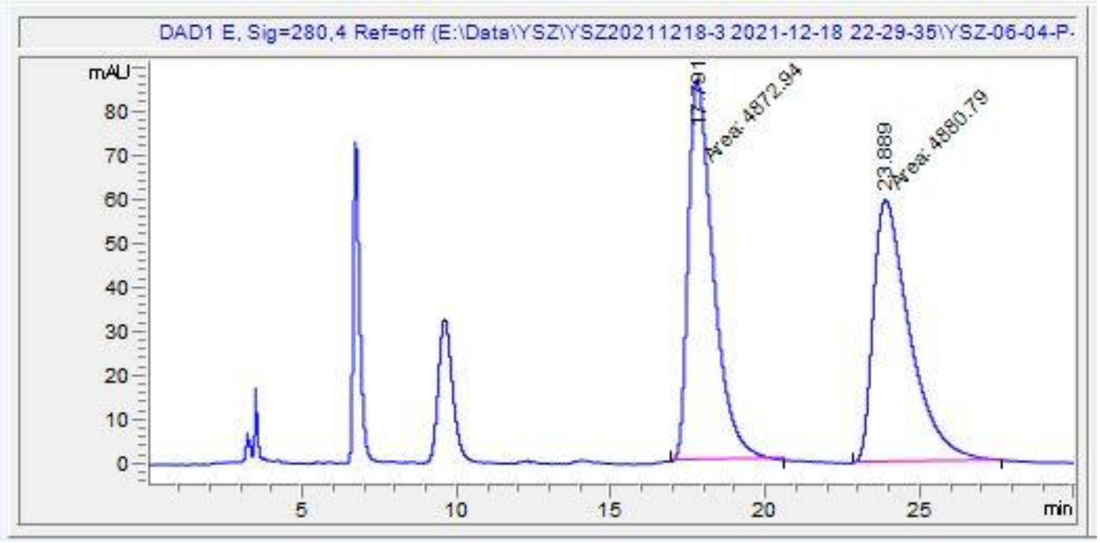

#	Time	Type	Area	Height	Width	Area%	Symmetry
1	17.791	MM	4872.9	86.2	0.9426	49.960	0.582
2	23.889	MM	4880.8	59.3	1.3714	50.040	0.505

Supplementary Fig. 130 Full HPLC spectrum of racemic **3p**

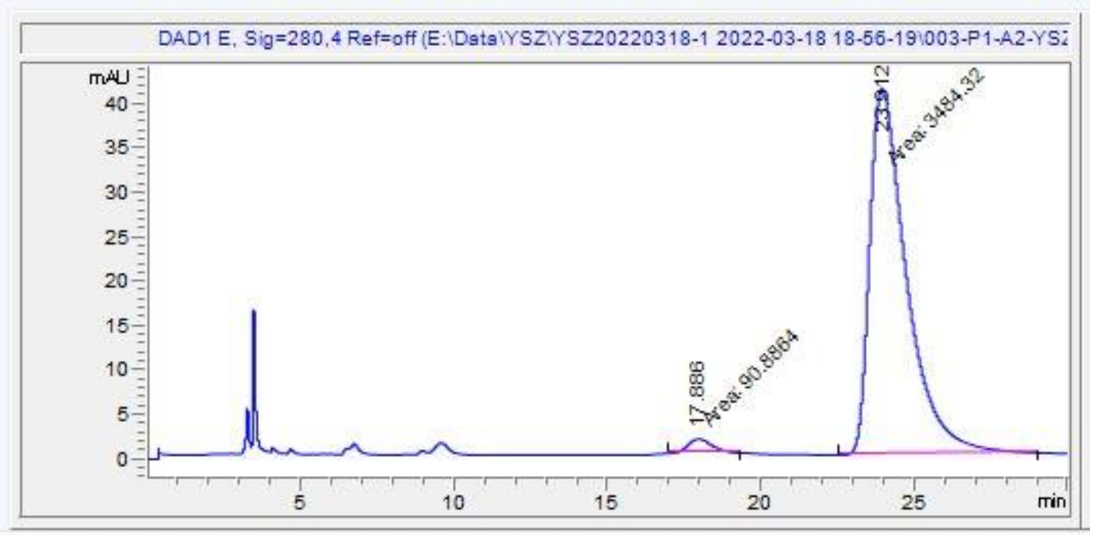

#	Time	Type	Area	Height	Width	Area%	Symmetry
1	17.886	MM	90.9	1.6	0.9209	2.542	0.624
2	23.912	MM	3484.3	41.6	1.3973	97.458	0.488

Supplementary Fig. 131 Full HPLC spectrum of (S_p)-**3p**

(R_p)-Tert-butyl-(4^3-(phenylethynyl)-1,4(1,4)-dibenzenacyclohexaphane-1^2-yl)carbamate **(1q)**

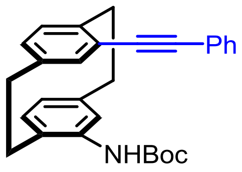

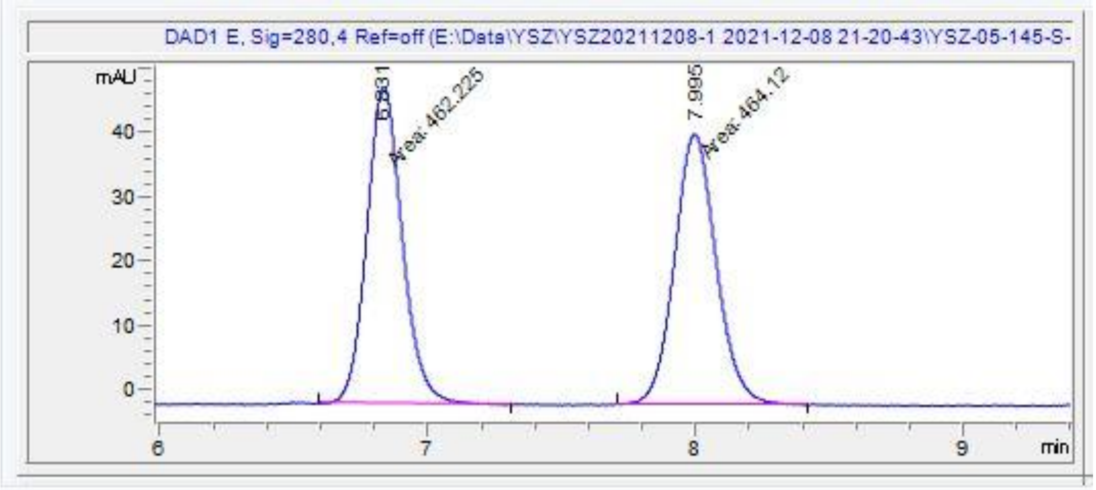

#	Time	Type	Area	Height	Width	Area%	Symmetry
1	6.831	MM	462.2	50	0.1541	49.898	0.871
2	7.995	MM	464.1	42.6	0.1814	50.102	0.904

Supplementary Fig. 132 HPLC spectrum of racemic **1q**

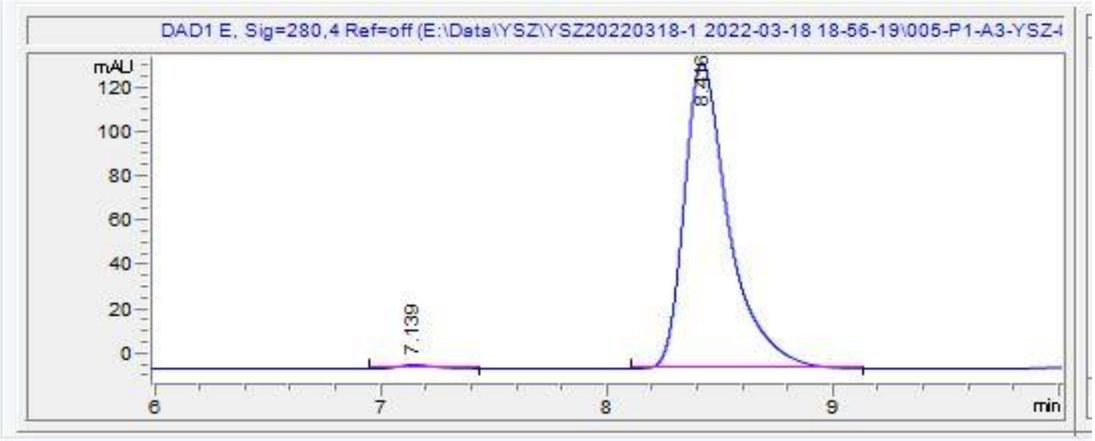

#	Time	Type	Area	Height	Width	Area%	Symmetry
1	7.139	BB	19.2	1.8	0.1275	0.996	0.782
2	8.416	BB	1913.2	138.8	0.2038	99.004	0.616

Supplementary Fig. 133 HPLC spectrum of (R_p)-**1q**

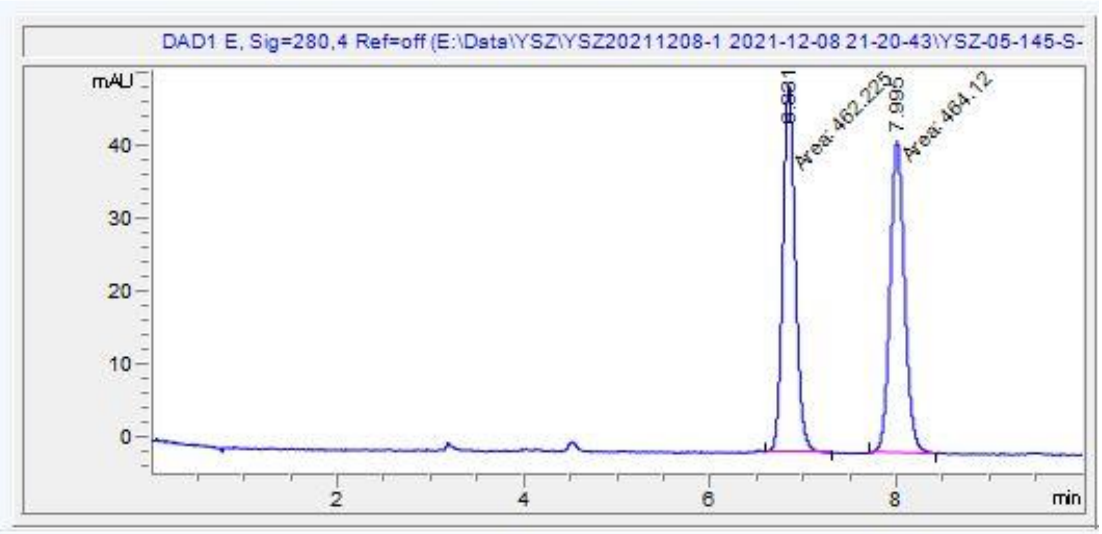

#	Time	Type	Area	Height	Width	Area%	Symmetry
1	6.831	MM	462.2	50	0.1541	49.898	0.871
2	7.995	MM	464.1	42.6	0.1814	50.102	0.904

Supplementary Fig. 134 Full HPLC spectrum of racemic **1q**

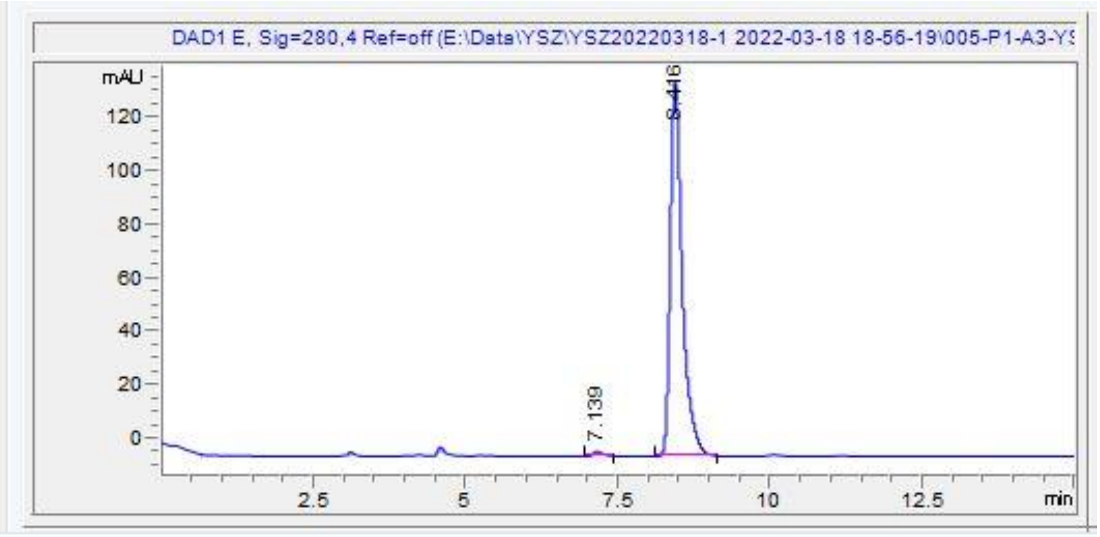

#	Time	Type	Area	Height	Width	Area%	Symmetry
1	7.139	BB	19.2	1.8	0.1275	0.996	0.782
2	8.416	BB	1913.2	138.8	0.2038	99.004	0.616

Supplementary Fig. 135 Full HPLC spectrum of (R_p)**-1q**

(S_p)-Dibenzyl 1-(1^5-((tert-butoxycarbonyl)amino)-4^2-(phenylethynyl)-1,4(1,4)-dibenzenacyclohexaphane-1^2-yl)hydrazine-1,2-dicarboxylate **(3q)**

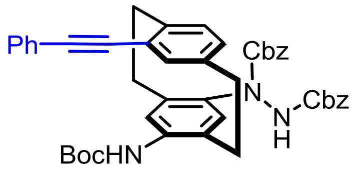

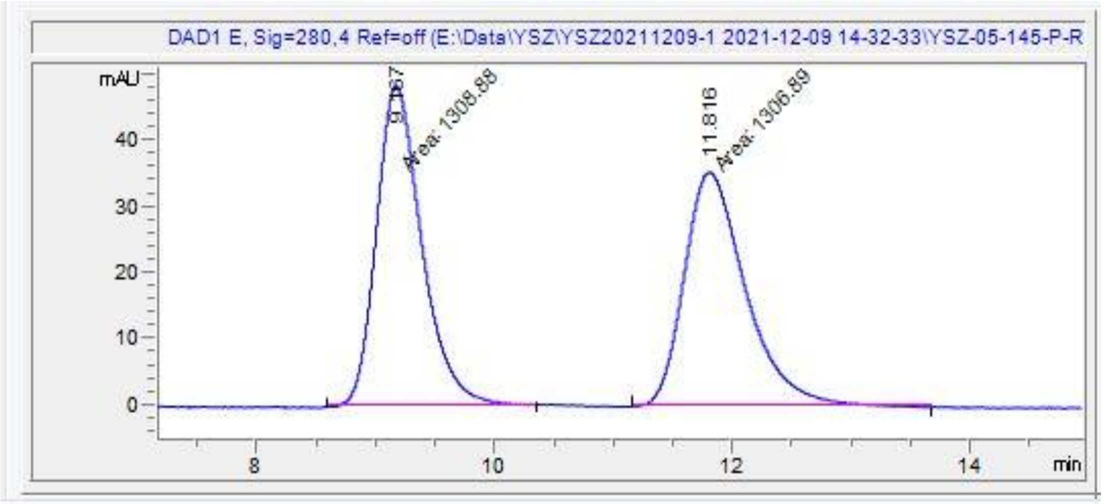

#	Time	Type	Area	Height	Width	Area%	Symmetry
1	9.167	MM	1308.9	49	0.4453	50.038	0.695
2	11.816	MM	1306.9	35.9	0.6059	49.962	0.666

Supplementary Fig. 136 HPLC spectrum of racemic **3q**

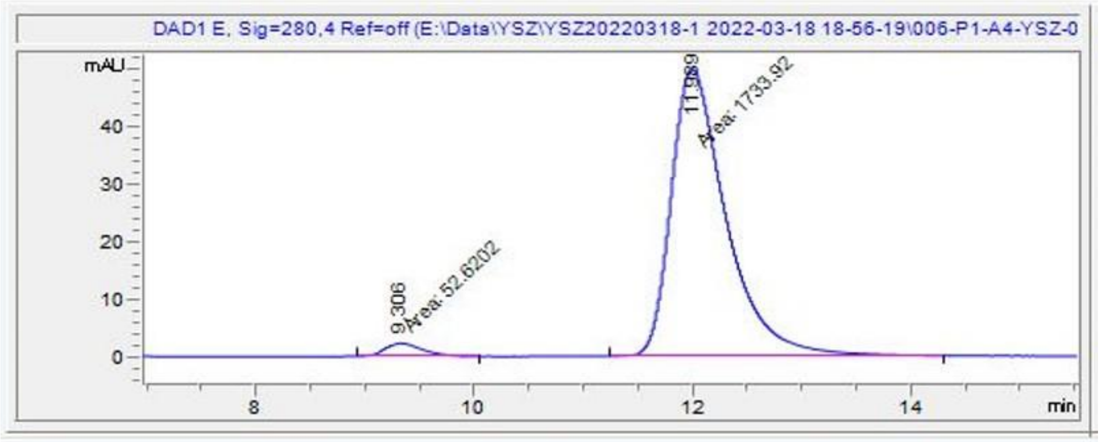

#	Time	Type	Area	Height	Width	Area%	Symmetry
1	9.306	MM	52.6	2.3	0.3887	2.945	0.66
2	11.989	MM	1733.9	49.6	0.5825	97.055	0.603

Supplementary Fig. 137 HPLC spectrum of (S_p)-**3q**

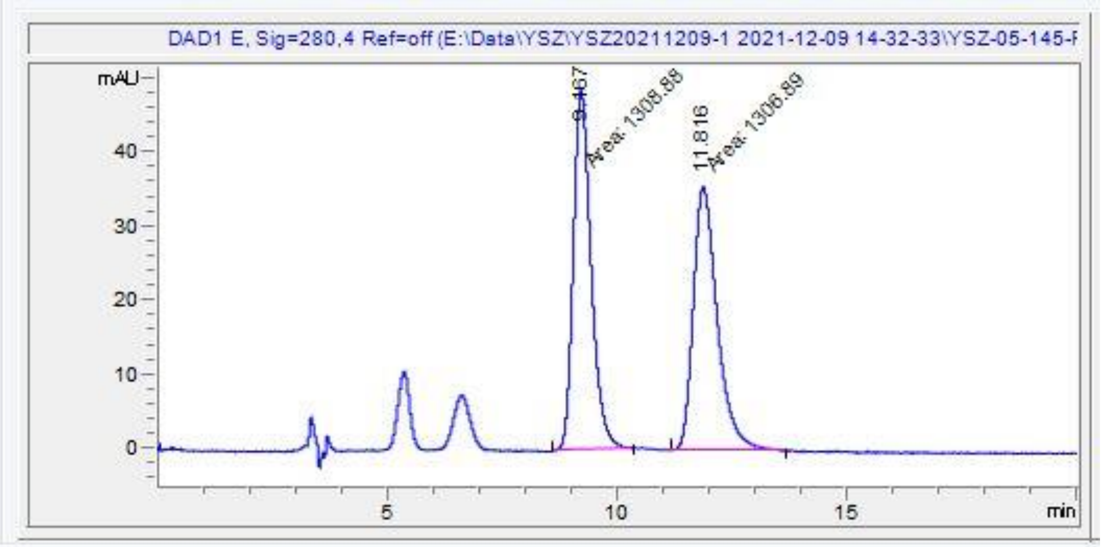

#	Time	Type	Area	Height	Width	Area%	Symmetry
1	9.167	MM	1308.9	49	0.4453	50.038	0.695
2	11.816	MM	1306.9	35.9	0.6059	49.962	0.666

Supplementary Fig. 138 Full HPLC spectrum of racemic **3q**

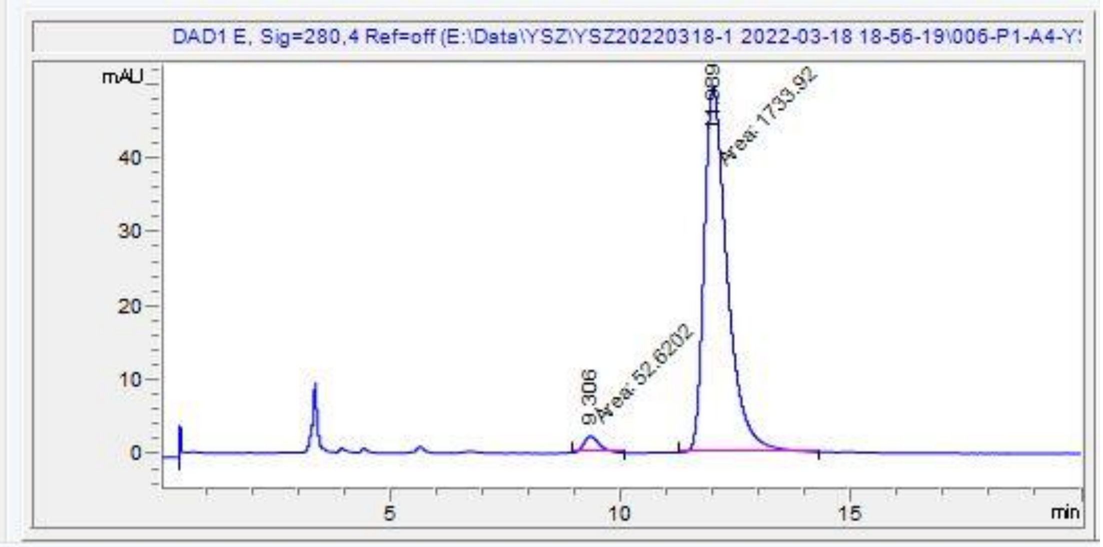

#	Time	Type	Area	Height	Width	Area%	Symmetry
1	9.306	MM	52.6	2.3	0.3887	2.945	0.66
2	11.989	MM	1733.9	49.6	0.5825	97.055	0.603

Supplementary Fig. 139 Full HPLC spectrum of (S_p)-**3q**

(R_p)-Tert-butyl (4[3]-cyclohexyl-1,4(1,4)-dibenzenacyclohexaphane-1[2]-yl)carbamate **(1r)**

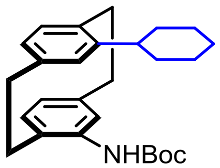

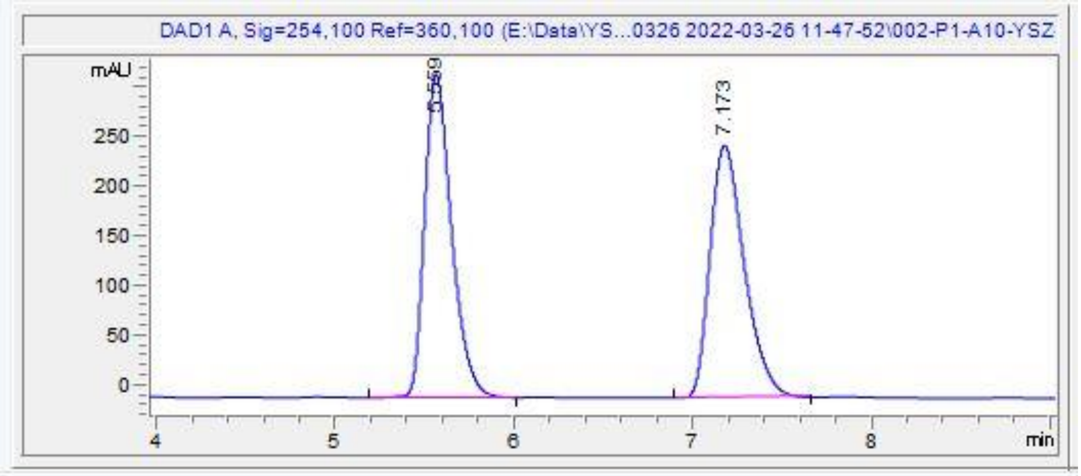

#	Time	Type	Area	Height	Width	Area%	Symmetry
1	5.559	BB	3400.6	324.8	0.1606	50.094	0.696
2	7.173	BB	3387.8	253.5	0.2059	49.906	0.683

Supplementary Fig. 140 HPLC spectrum of racemic **1r**

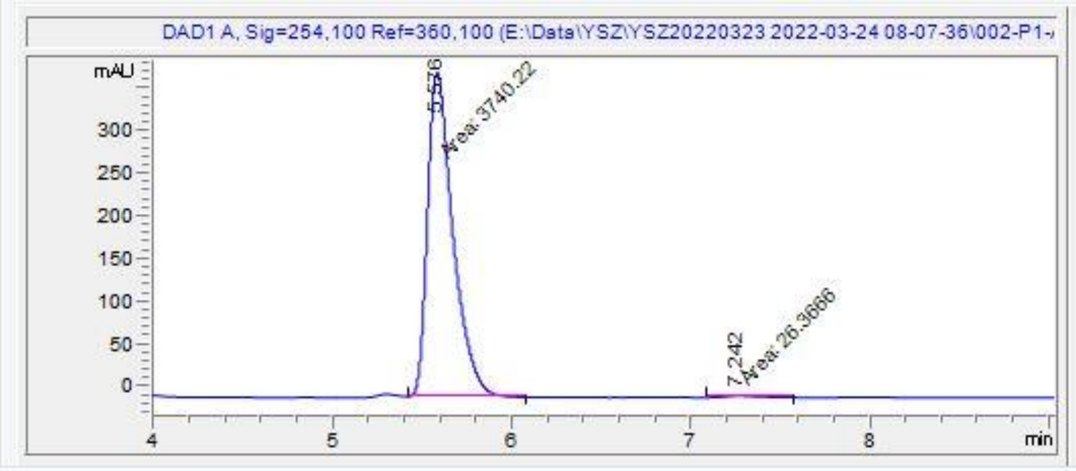

#	Time	Type	Area	Height	Width	Area%	Symmetry
1	5.576	MM	3740.2	381.1	0.1636	99.300	0.581
2	7.242	MM	26.4	2	0.2177	0.700	0.525

Supplementary Fig. 141 HPLC spectrum of (R_p)-**1r**

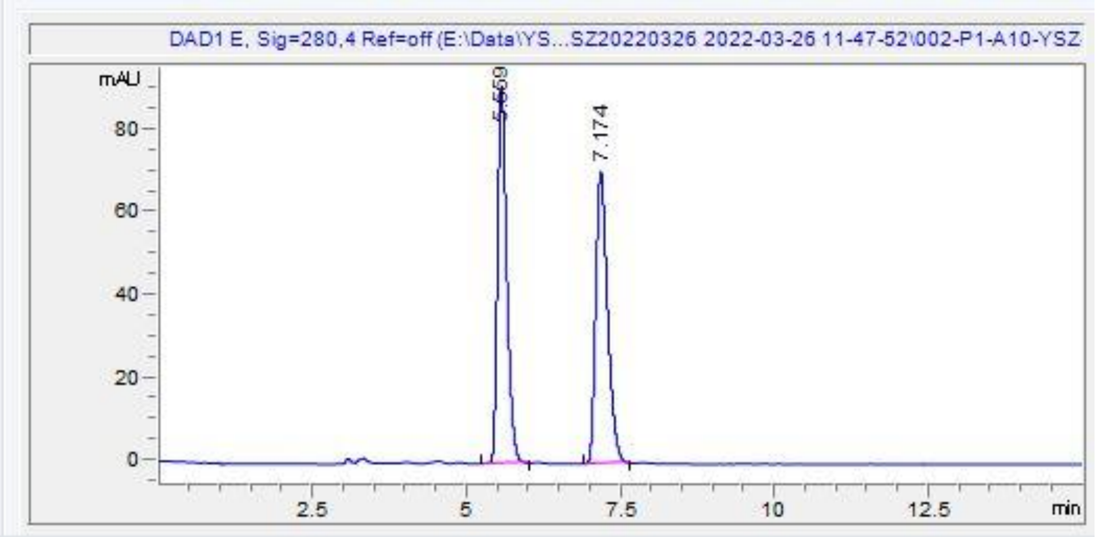

#	Time	Type	Area	Height	Width	Area%	Symmetry
1	5.559	BB	942.4	91	0.1594	50.122	0.696
2	7.174	BB	937.8	70.5	0.1968	49.878	0.689

Supplementary Fig. 142 Full HPLC spectrum of racemic **1r**

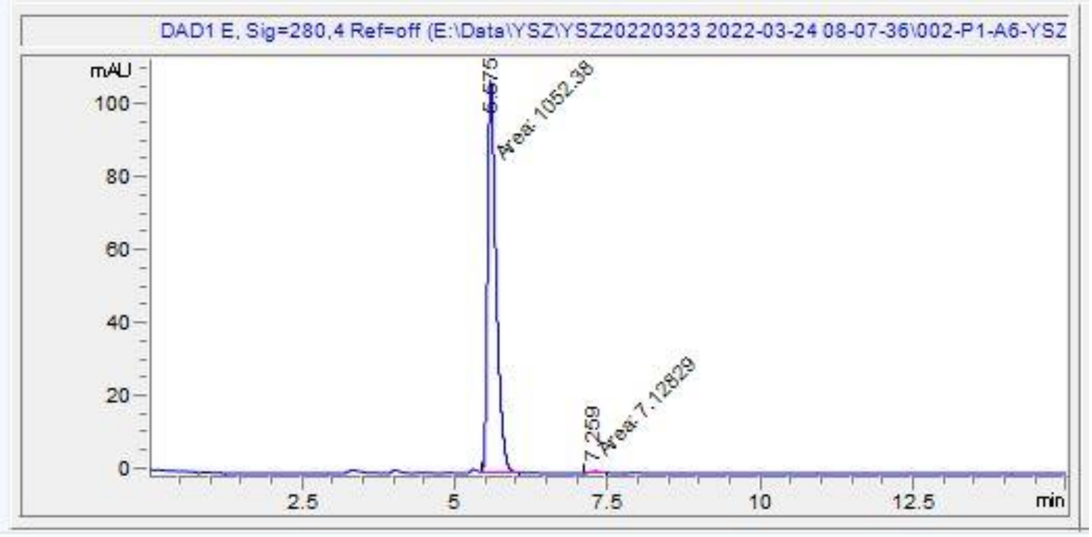

#	Time	Type	Area	Height	Width	Area%	Symmetry
1	5.575	FM	1052.4	108.1	0.1623	99.327	0.579
2	7.259	MM	7.1	5.7E-1	0.2089	0.673	0.769

Supplementary Fig. 143 Full HPLC spectrum of (R_p)-**1r**

(S_p)-Dibenzyl 1-(1^5-((tert-butoxycarbonyl)amino)-4^2-cyclohexyl-1,4(1,4)
-dibenzenacyclohexaphane-1^2-yl)hydrazine-1,2-dicarboxylate **(3r)**

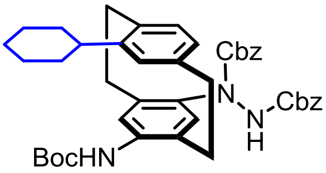

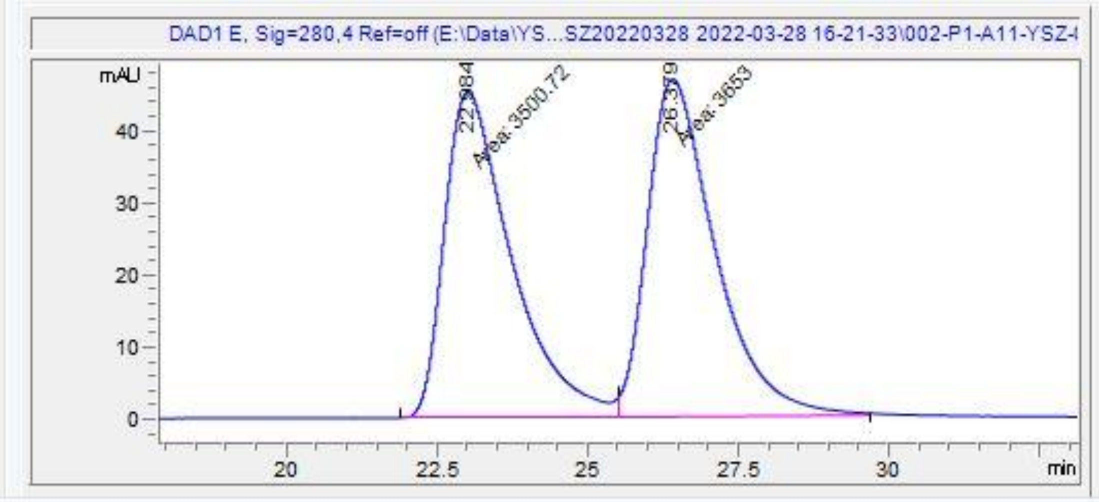

#	Time	Type	Area	Height	Width	Area%	Symmetry
1	22.984	MF	3500.7	45	1.2975	48.936	0
2	26.379	FM	3653	46.6	1.3061	51.064	0.568

Supplementary Fig. 144 HPLC spectrum of racemic **3r**

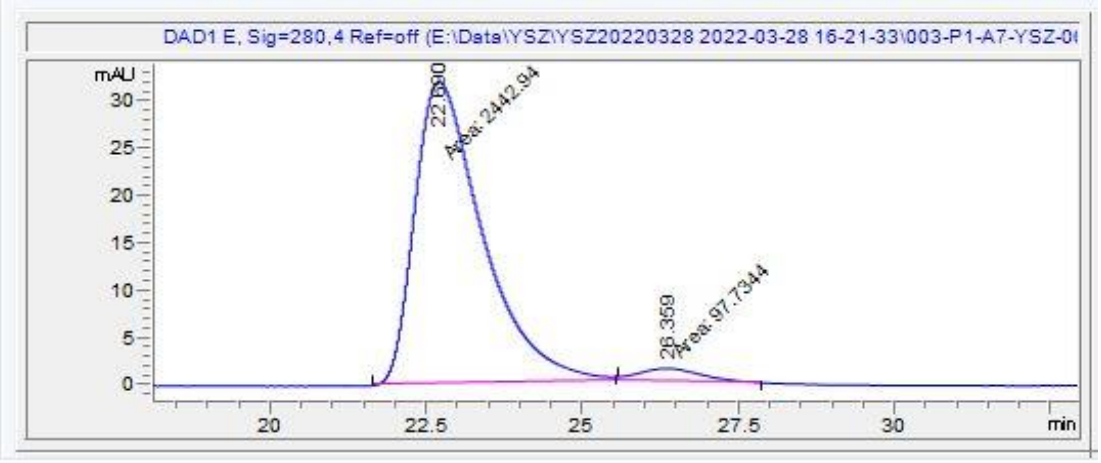

#	Time	Type	Area	Height	Width	Area%	Symmetry
1	22.69	MM	2442.9	32.2	1.2645	96.153	0.522
2	26.359	MM	97.7	1.4	1.1834	3.847	0.762

Supplementary Fig. 145 HPLC spectrum of (S_p)-**3r**

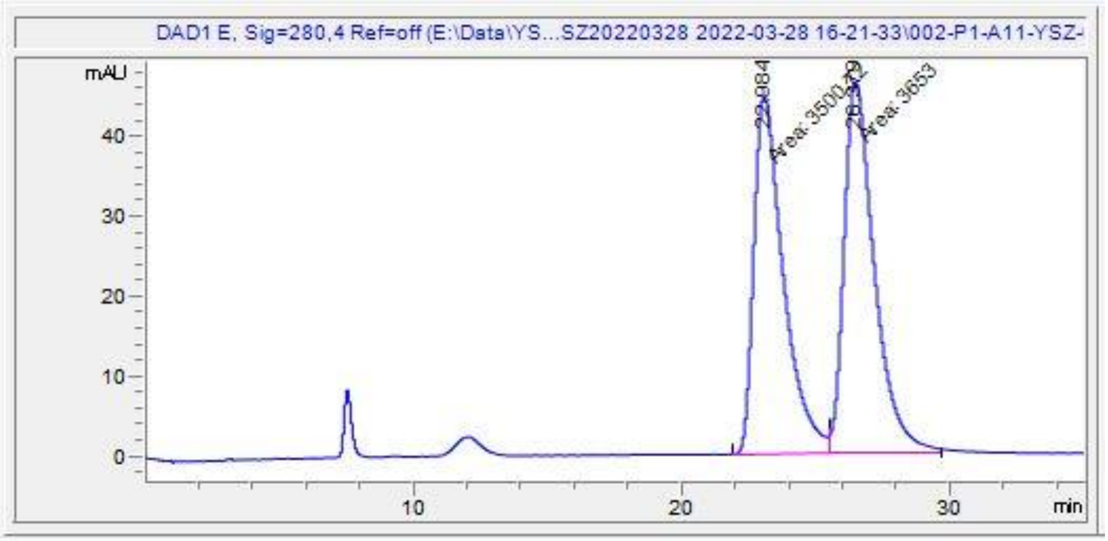

#	Time	Type	Area	Height	Width	Area%	Symmetry
1	22.984	MF	3500.7	45	1.2975	48.936	0
2	26.379	FM	3653	46.6	1.3061	51.064	0.568

Supplementary Fig. 146 Full HPLC spectrum of racemic **3r**

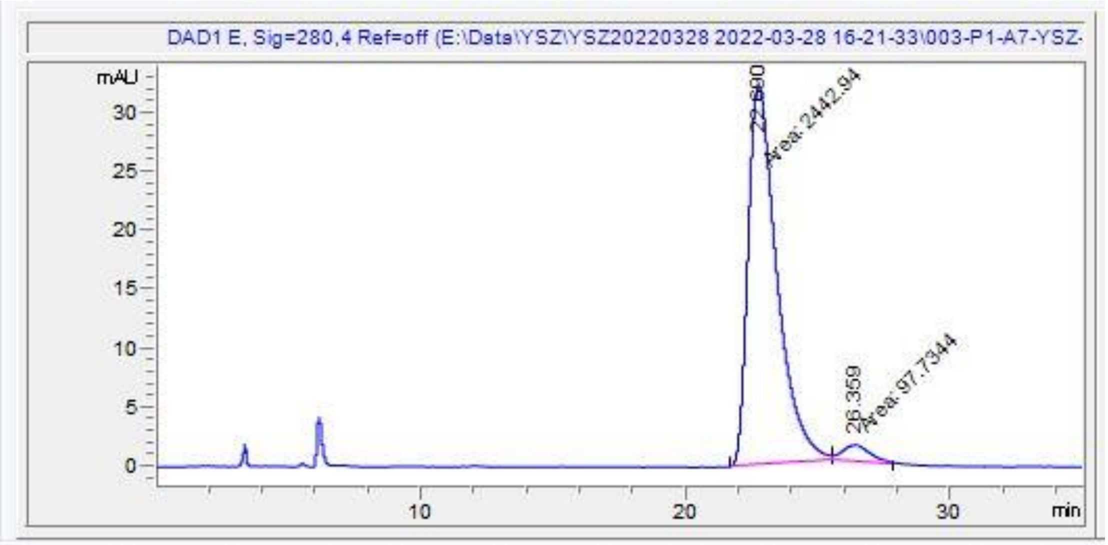

#	Time	Type	Area	Height	Width	Area%	Symmetry
1	22.69	MM	2442.9	32.2	1.2645	96.153	0.522
2	26.359	MM	97.7	1.4	1.1834	3.847	0.762

Supplementary Fig. 147 Full HPLC spectrum of (S_p)-**3r**

(R_p)-4^3-((tert-butoxycarbonyl)amino)-1,4(1,4)-dibenzenacyclohexaphane-1^2-yl acetate **(1s)**

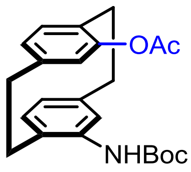

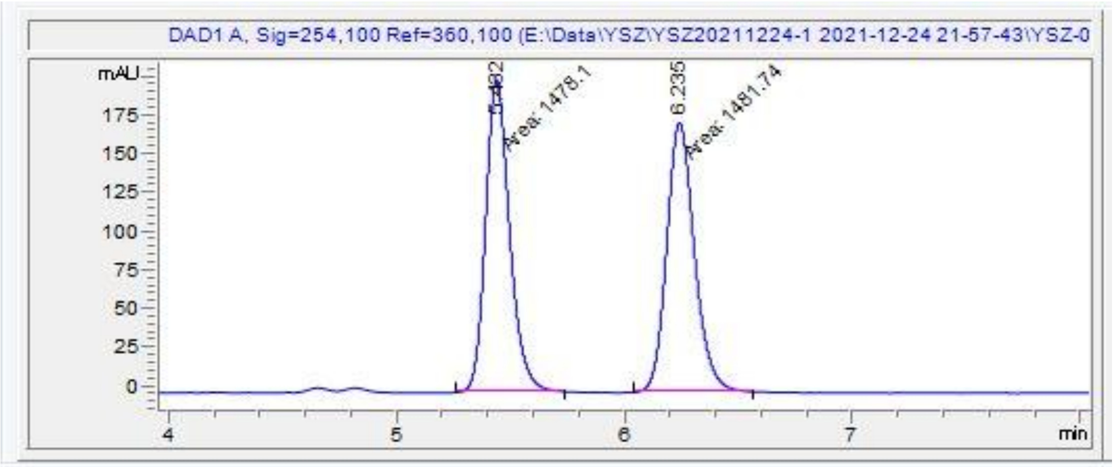

#	Time	Type	Area	Height	Width	Area%	Symmetry
1	5.432	MM	1478.1	202.1	0.1219	49.939	0.792
2	6.235	MM	1481.7	174.5	0.1416	50.061	0.858

Supplementary Fig. 148 HPLC spectrum of racemic **1s**

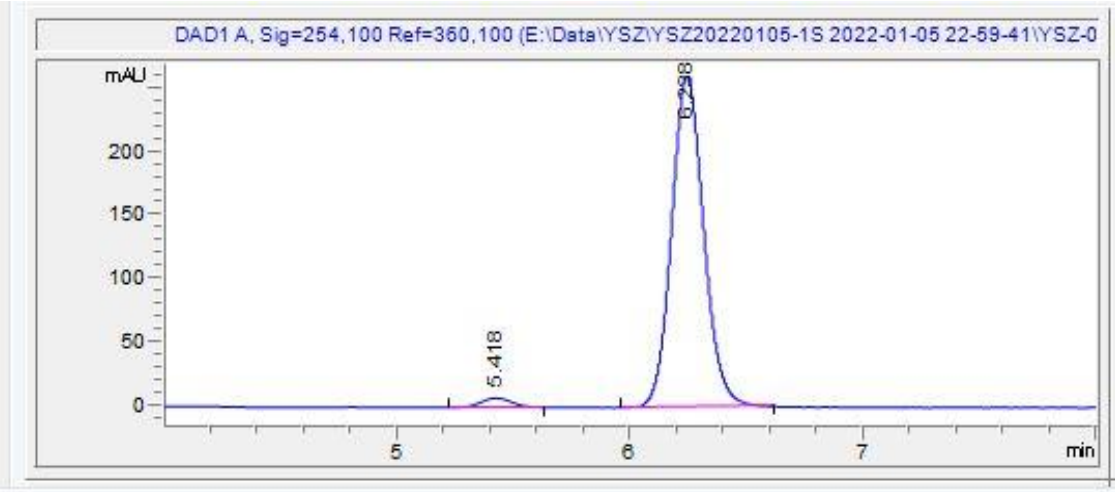

#	Time	Type	Area	Height	Width	Area%	Symmetry
1	5.418	VV R	72	7.7	0.1109	2.776	0.931
2	6.238	VV R	2520.3	261.7	0.1169	97.224	0.796

Supplementary Fig. 149 HPLC spectrum of (R_p)**-1s**

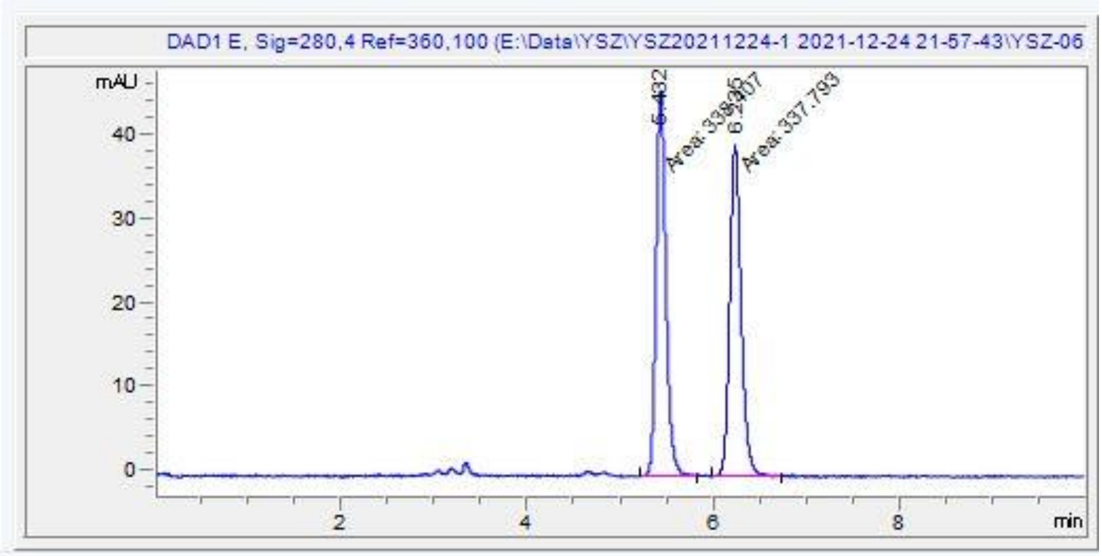

#	Time	Type	Area	Height	Width	Area%	Symmetry
1	5.432	MM	338.4	46	0.1225	50.045	0.775
2	6.235	MM	337.8	39.6	0.1422	49.955	0.833

Supplementary Fig. 150 Full HPLC spectrum of racemic **1s**

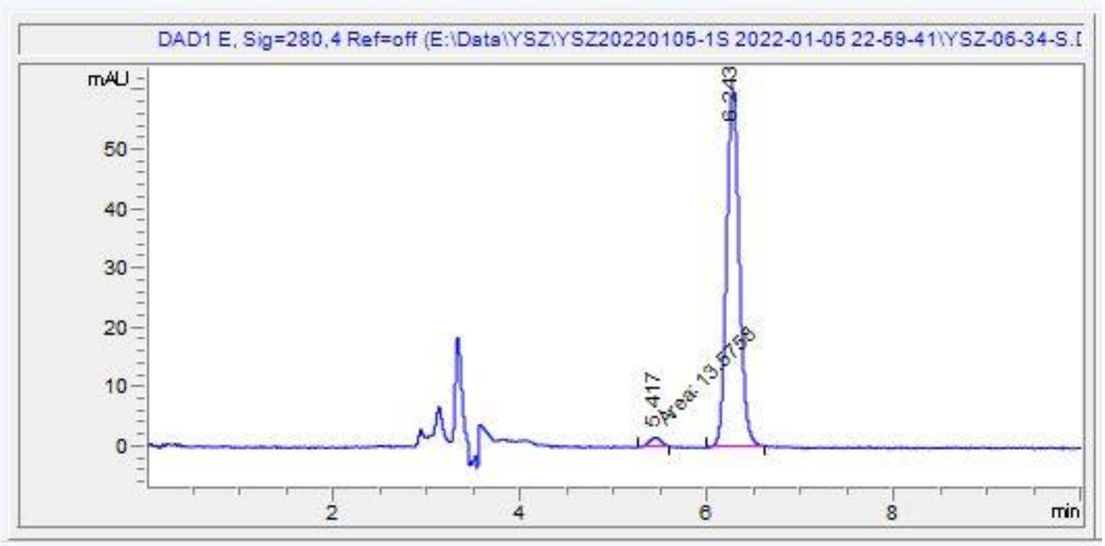

#	Time	Type	Area	Height	Width	Area%	Symmetry
1	5.417	MM	13.6	1.6	0.1396	2.294	0.942
2	6.243	VV R	578.3	60.6	0.1189	97.706	0.911

Supplementary Fig. 151 Full HPLC spectrum of (S_p)-**1s**

(S_p)-Dibenzyl 1-(4^2-acetoxy-1^5-((tert-butoxycarbonyl)amino)-1,4(1,4)-dibenzenacyclohexaphane-1^2-yl)hydrazine-1,2-dicarboxylate **(3s)**

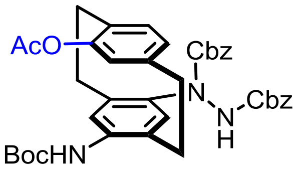

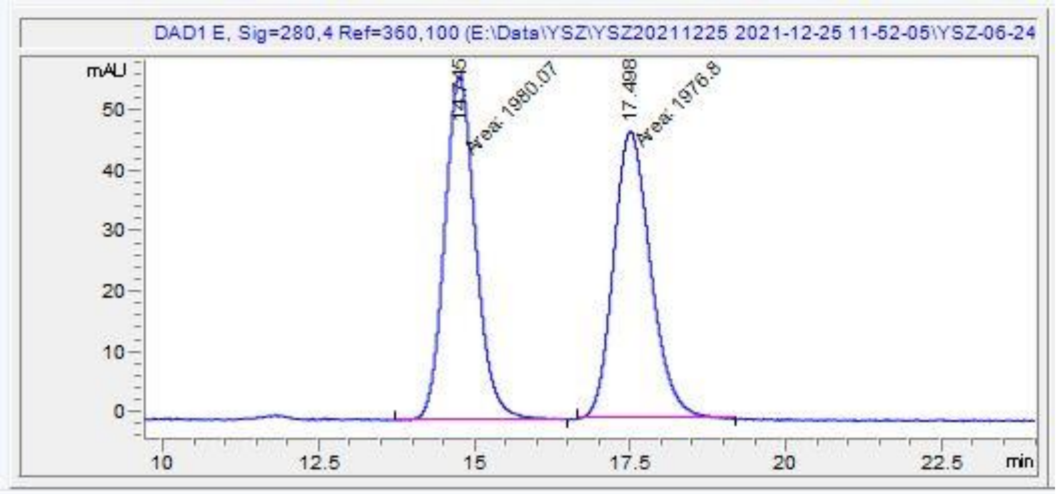

#	Time	Type	Area	Height	Width	Area%	Symmetry
1	14.745	MM	1980.1	56.7	0.5817	50.041	0.842
2	17.498	MM	1976.8	47.5	0.694	49.959	0.8

Supplementary Fig. 152 HPLC spectrum of racemic **3s**

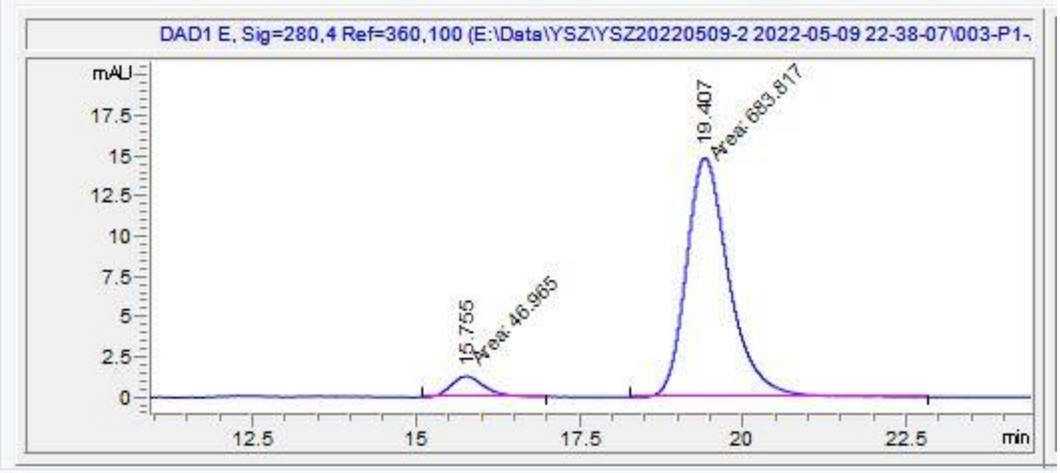

#	Time	Type	Area	Height	Width	Area%	Symmetry
1	15.755	MM	47	1.3	0.5961	6.427	0.807
2	19.407	MM	683.8	15	0.7595	93.573	0.722

Supplementary Fig. 153 HPLC spectrum of (S_p)-**3s**

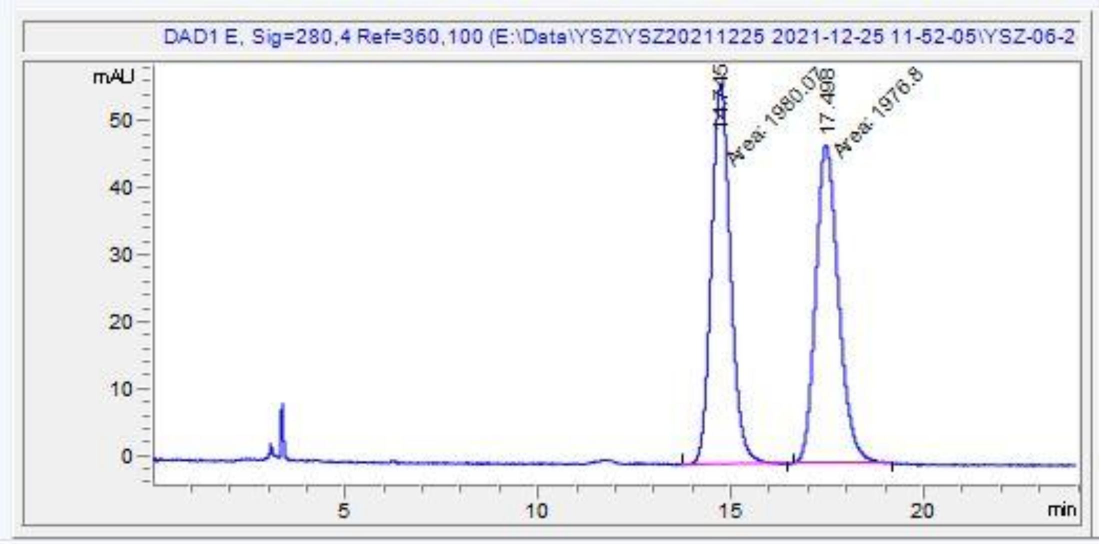

#	Time	Type	Area	Height	Width	Area%	Symmetry
1	14.745	MM	1980.1	56.7	0.5817	50.041	0.842
2	17.498	MM	1976.8	47.5	0.694	49.959	0.8

Supplementary Fig. 154 Full HPLC spectrum of racemic **3s**

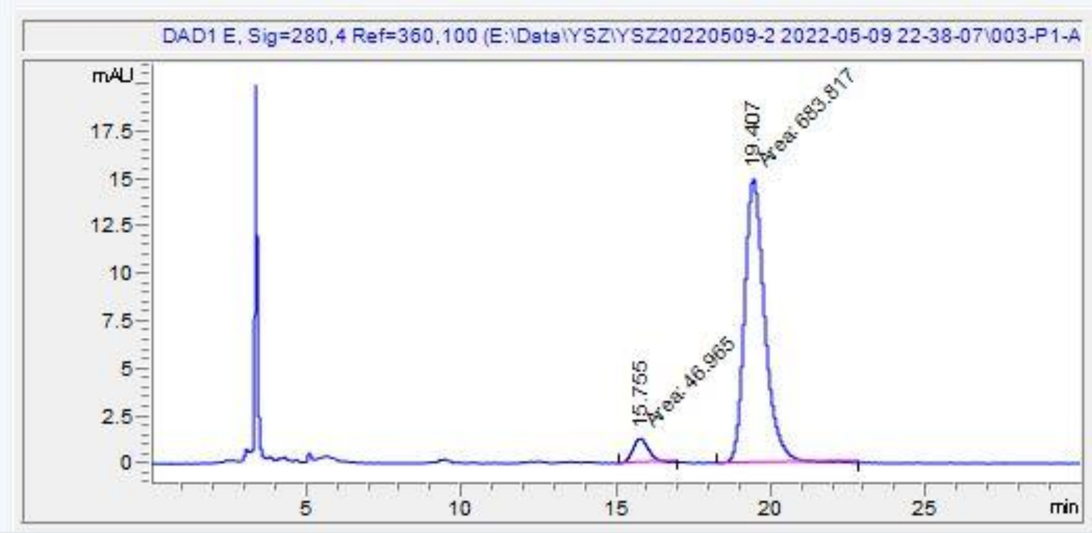

#	Time	Type	Area	Height	Width	Area%	Symmetry
1	15.755	MM	47	1.3	0.5961	6.427	0.807
2	19.407	MM	683.8	15	0.7595	93.573	0.722

Supplementary Fig. 155 Full HPLC spectrum of (S_p)-**3s**

(R_p)-Tert-butyl (4^3-formyl-1,4(1,4)-dibenzenacyclohexaphane-1^2-yl)carbamate **(1t)**

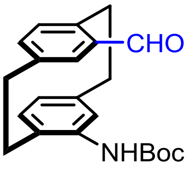

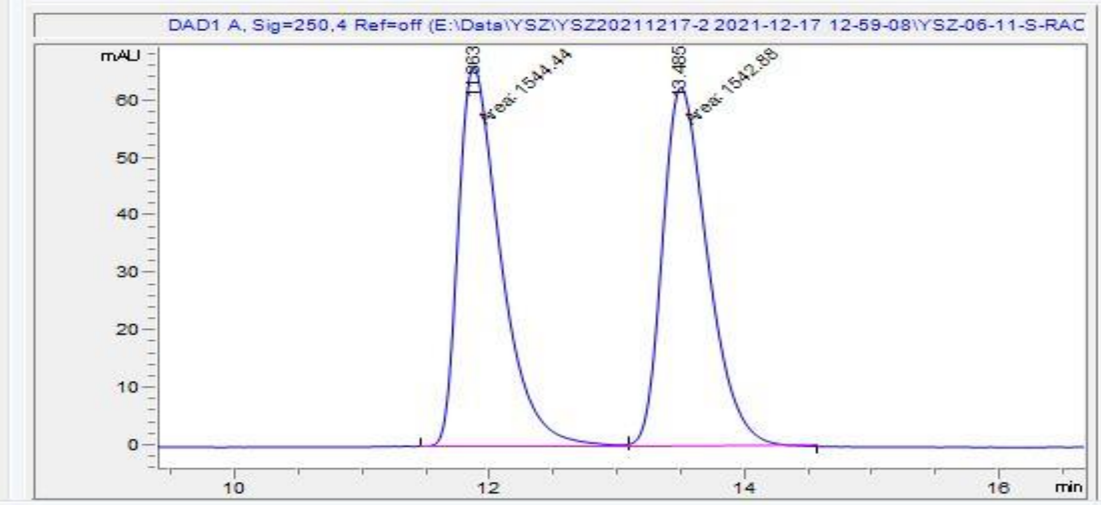

#	Time	Type	Area	Height	Width	Area%	Symmetry
1	11.863	MF	1544.4	66.2	0.3886	50.025	0
2	13.485	FM	1542.9	62.6	0.4109	49.975	0.662

Supplementary Fig. 156 HPLC spectrum of racemic **1t**

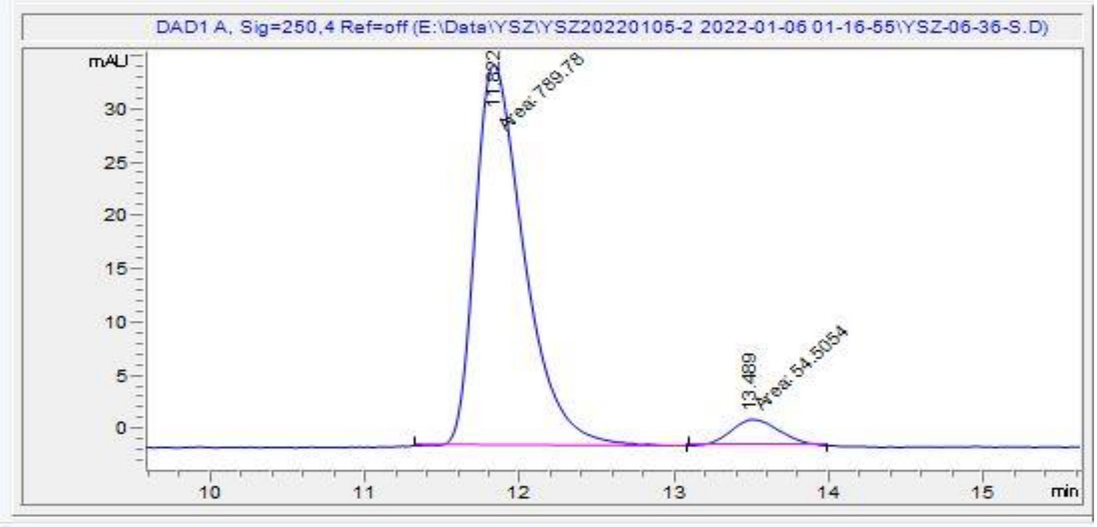

#	Time	Type	Area	Height	Width	Area%	Symmetry
1	11.822	MM	789.8	35.8	0.3681	93.544	0.592
2	13.489	MM	54.5	2.5	0.3702	6.456	0.742

Supplementary Fig. 157 HPLC spectrum of (R_p)**-1t**

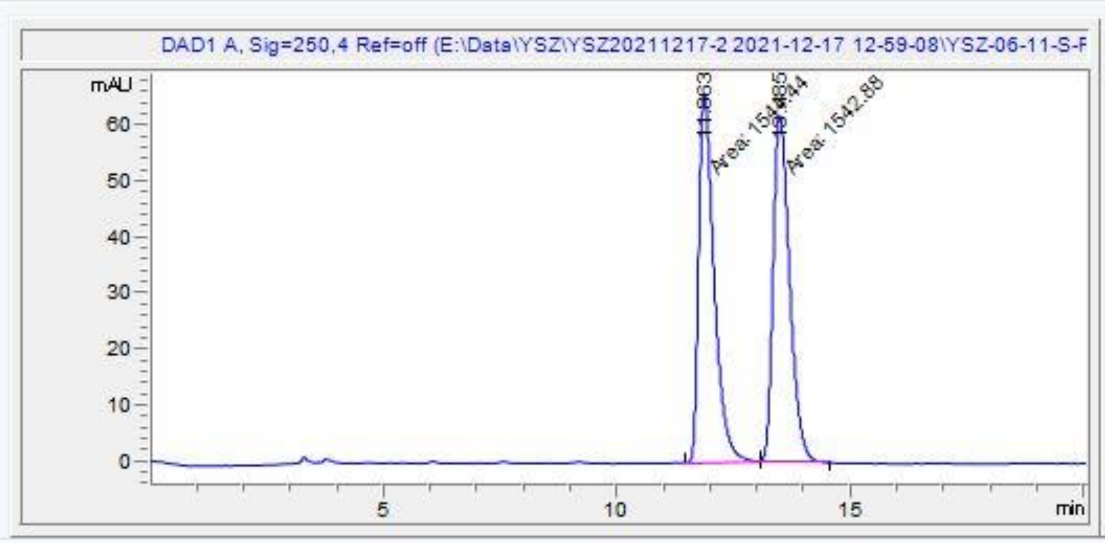

#	Time	Type	Area	Height	Width	Area%	Symmetry
1	11.863	MF	1544.4	66.2	0.3886	50.025	0
2	13.485	FM	1542.9	62.6	0.4109	49.975	0.662

Supplementary Fig. 158 Full HPLC spectrum of racemic **1t**

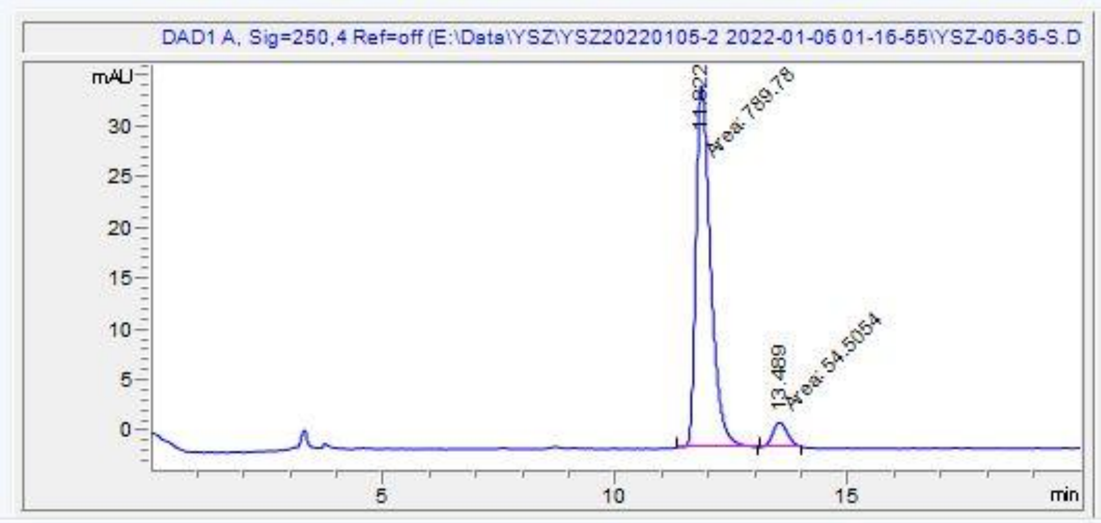

#	Time	Type	Area	Height	Width	Area%	Symmetry
1	11.822	MM	789.8	35.8	0.3681	93.544	0.592
2	13.489	MM	54.5	2.5	0.3702	6.456	0.742

Supplementary Fig. 159 Full HPLC spectrum of (R_p)-**1t**

(S_p)-Dibenzyl 1-(1^5-((tert-butoxycarbonyl)amino)-4^2-formyl-1,4(1,4)-dibenzenacyclohexaphane-1^2-yl)hydrazine-1,2-dicarboxylate **(3t)**

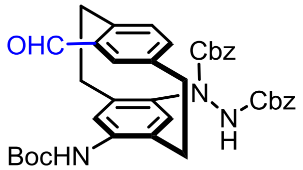

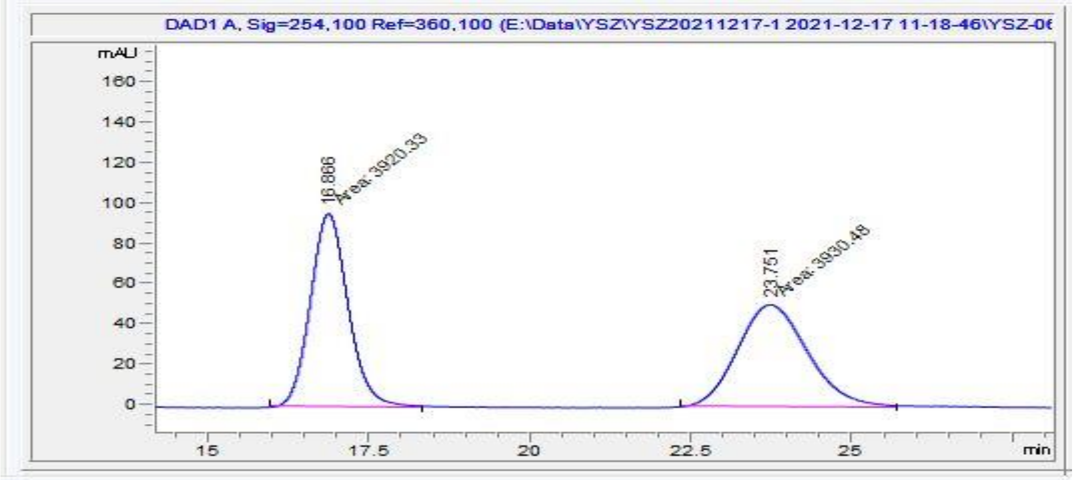

#	Time	Type	Area	Height	Width	Area%	Symmetry
1	16.866	MM	3920.3	95.9	0.6815	49.935	0.893
2	23.751	MM	3930.5	51	1.2851	50.065	0.864

Supplementary Fig. 160 HPLC spectrum of racemic **3t**

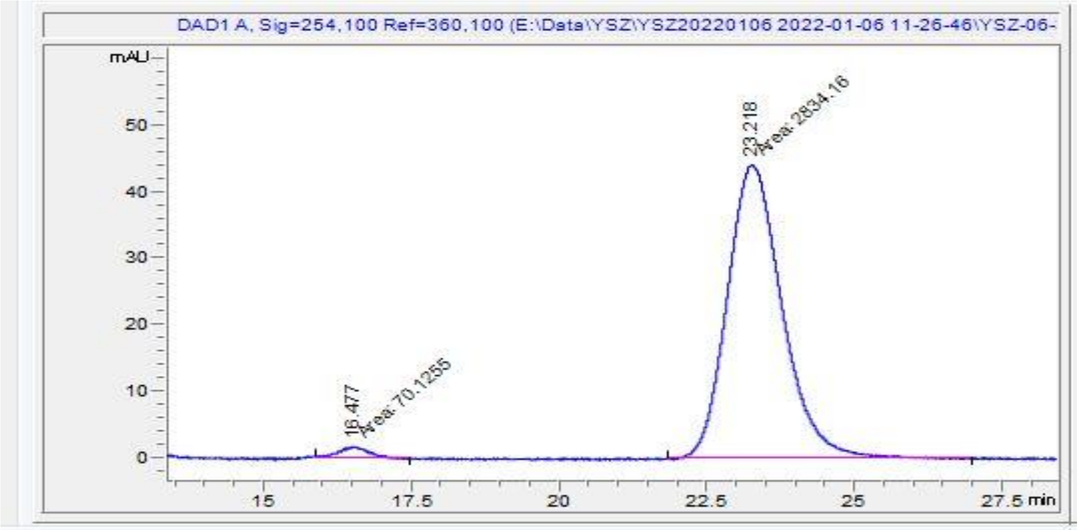

#	Time	Type	Area	Height	Width	Area%	Symmetry
1	16.477	FM	70.1	1.9	0.631	2.415	0.87
2	23.218	MM	2834.2	44	1.0724	97.585	0.751

Supplementary Fig. 161 HPLC spectrum of (S_p)-**3t**

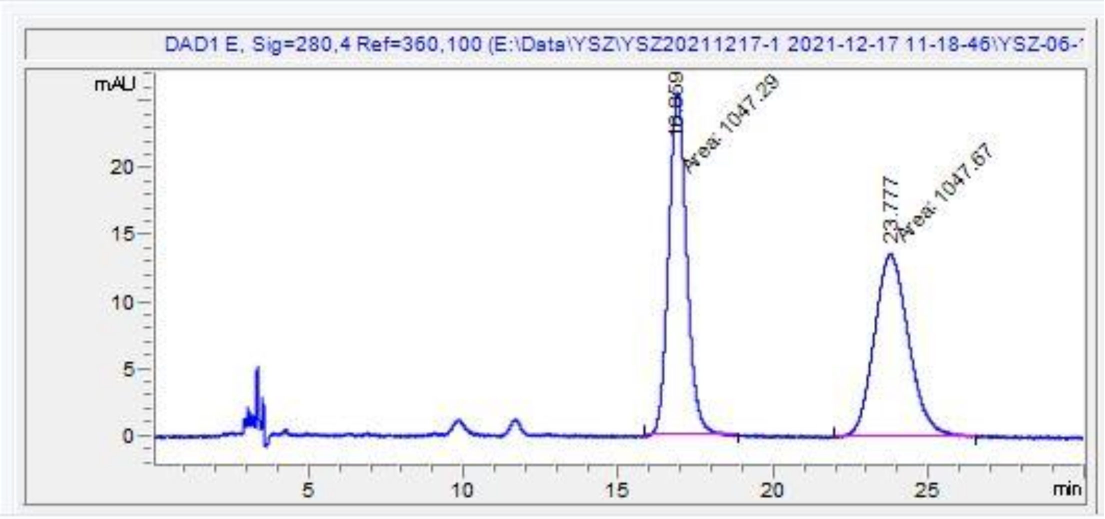

#	Time	Type	Area	Height	Width	Area%	Symmetry
1	16.859	MM	1047.3	25.8	0.6778	49.991	0.871
2	23.777	MM	1047.7	13.7	1.2728	50.009	0.964

Supplementary Fig. 162 Full HPLC spectrum of racemic **3t**

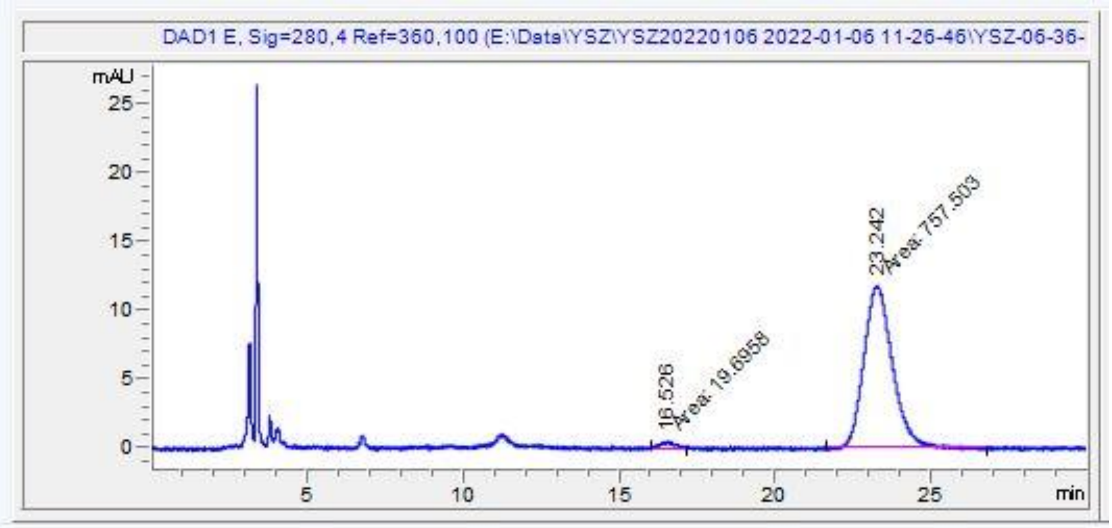

#	Time	Type	Area	Height	Width	Area%	Symmetry
1	16.526	MM	19.7	6.2E-1	0.5308	2.534	0.892
2	23.242	MM	757.5	11.9	1.0631	97.466	0.83

Supplementary Fig. 163 Full HPLC spectrum of (S_p)-**3t**

(R_p)-Tert-butyl-(4^3-(hydroxymethyl)-1,4(1,4)-dibenzenacyclohexaphane-1^2-yl)carbamate **(1u)**

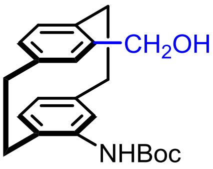

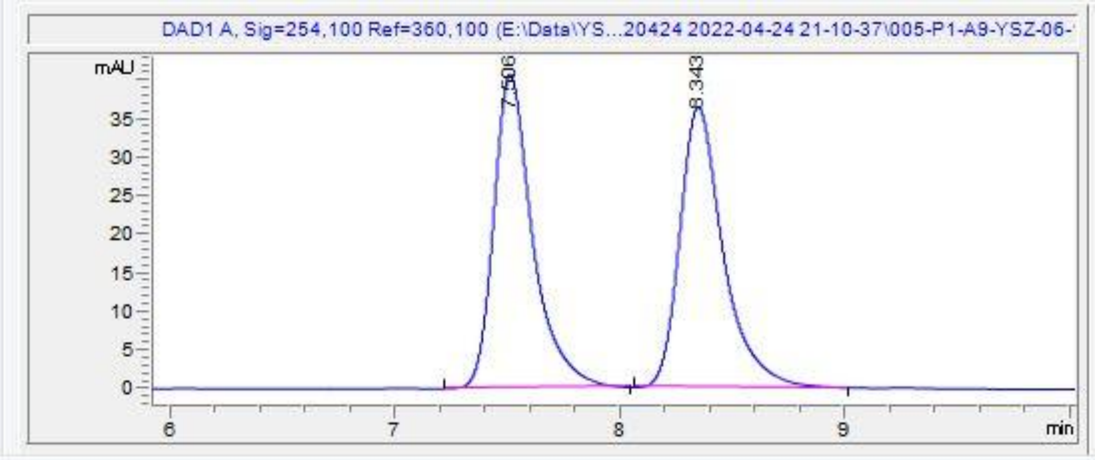

#	Time	Type	Area	Height	Width	Area%	Symmetry
1	7.506	BB	492.9	40.8	0.1788	50.044	0.675
2	8.343	BB	492	36.5	0.1997	49.956	0.684

Supplementary Fig. 164 HPLC spectrum of racemic **1u**

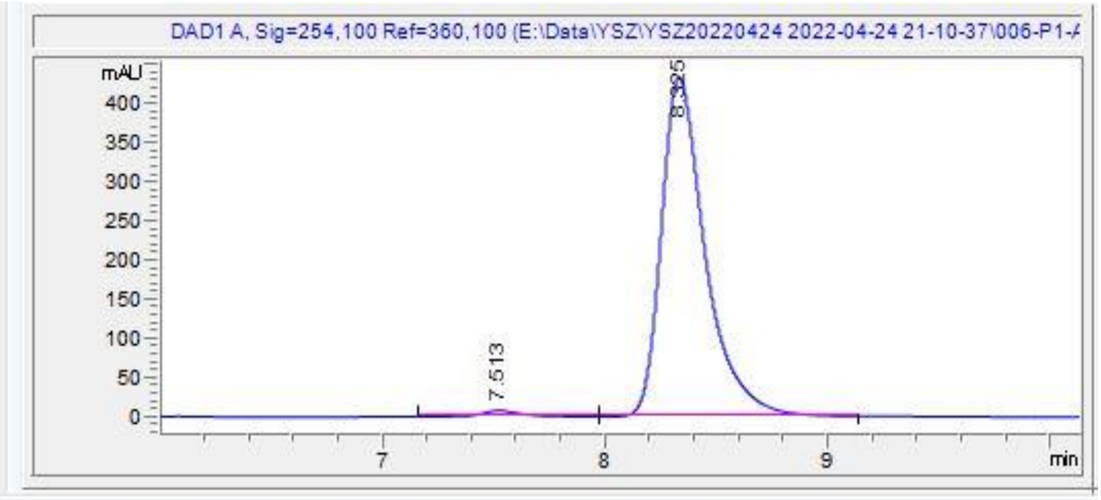

#	Time	Type	Area	Height	Width	Area%	Symmetry
1	7.513	BB	100.3	7.9	0.1658	1.663	0.772
2	8.325	BB	5931.5	432.3	0.2061	98.337	0.632

Supplementary Fig. 165 HPLC spectrum of (R_p)-**1u**

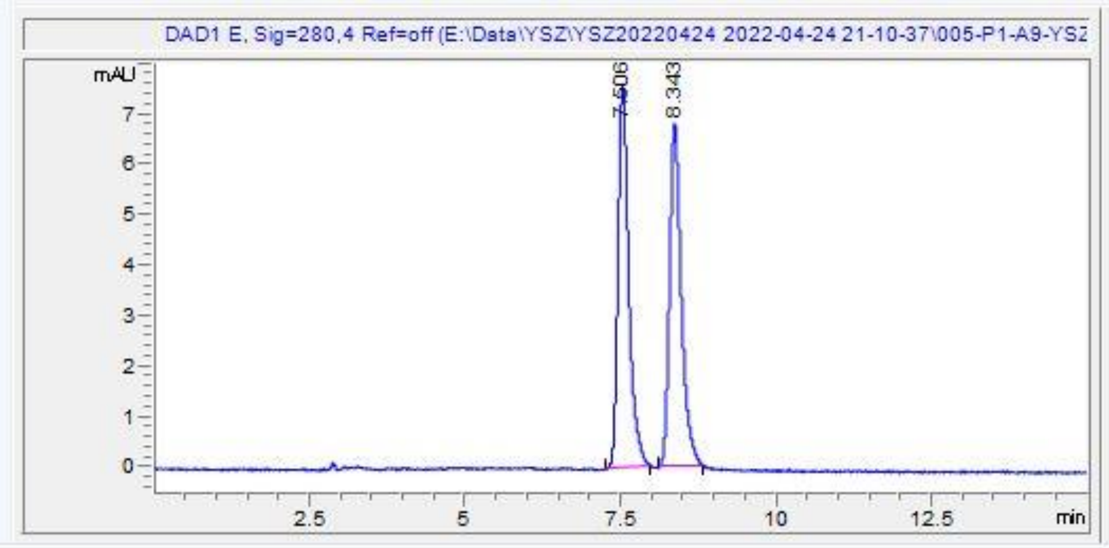

#	Time	Type	Area	Height	Width	Area%	Symmetry
1	7.506	BB	91.7	7.6	0.1421	50.372	0.691
2	8.343	BB	90.4	6.8	0.1564	49.628	0.72

Supplementary Fig. 166 Full HPLC spectrum of racemic **1u**

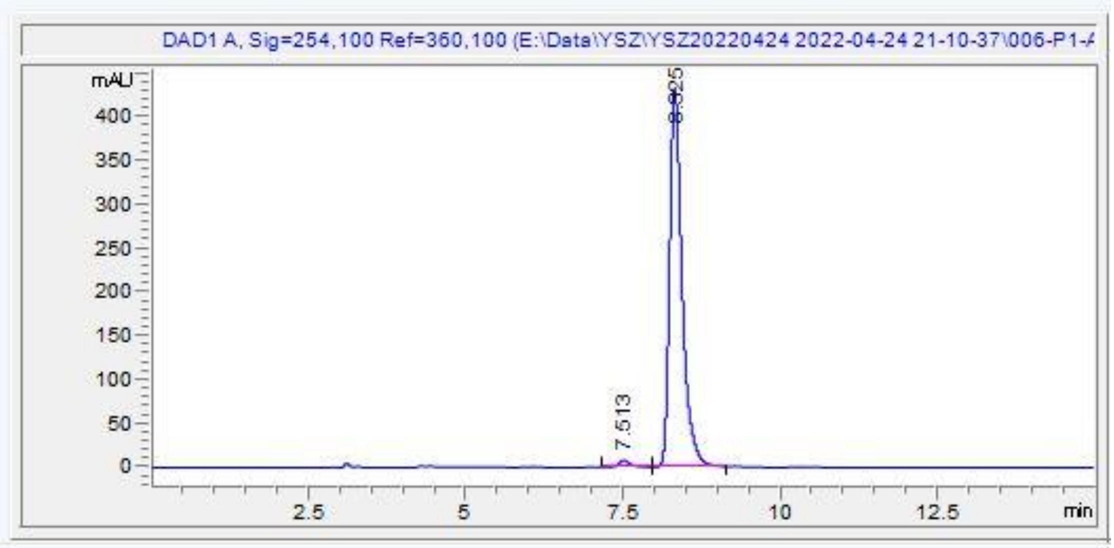

#	Time	Type	Area	Height	Width	Area%	Symmetry
1	7.513	BB	100.3	7.9	0.1658	1.663	0.772
2	8.325	BB	5931.5	432.3	0.2061	98.337	0.632

Supplementary Fig. 167 Full HPLC spectrum of (R_p)-**1u**

(S_p)-Dibenzyl 1-(1^5-((tert-butoxycarbonyl)amino)-4^2-(hydroxymethyl)-1,4(1,4)-dibenzenacyclohexaphane-1^2-yl)hydrazine-1,2-dicarboxylate **(3u)**

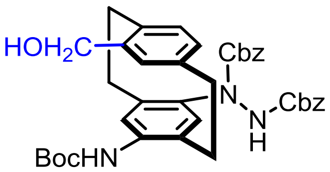

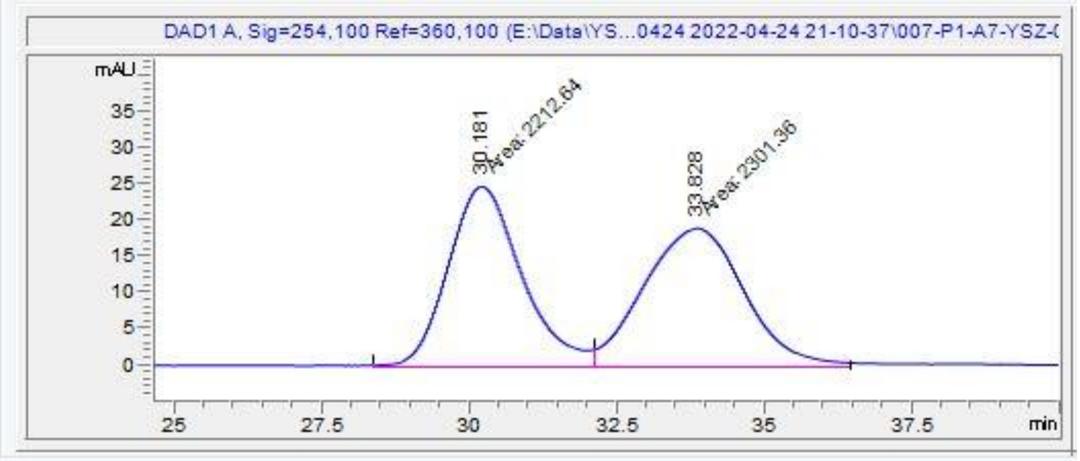

#	Time	Type	Area	Height	Width	Area%	Symmetry
1	30.181	MF	2212.6	25.3	1.4604	49.017	0
2	33.828	FM	2301.4	19.4	1.9743	50.983	1.052

Supplementary Fig. 168 HPLC spectrum of racemic **3u**

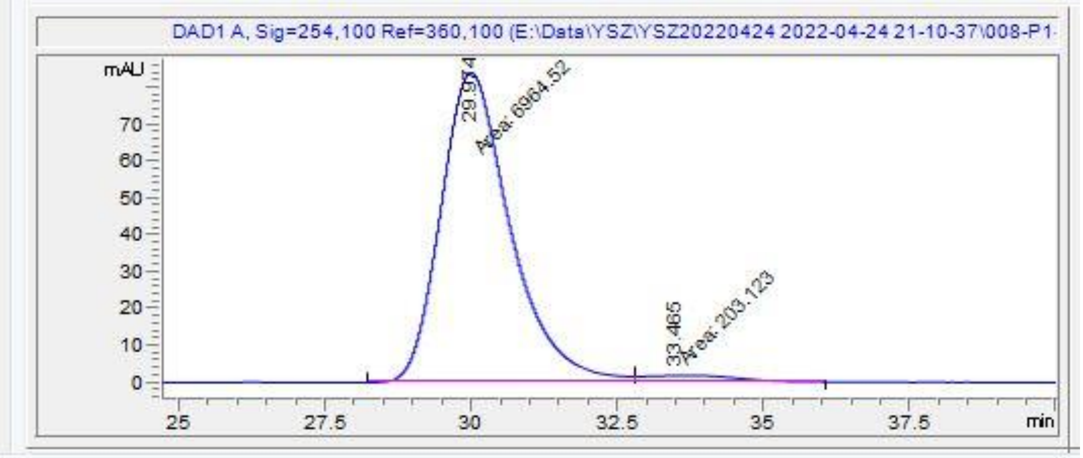

#	Time	Type	Area	Height	Width	Area%	Symmetry
1	29.974	MF	6964.5	84.1	1.3801	97.166	0.731
2	33.465	FM	203.1	1.9	1.8186	2.834	0.488

Supplementary Fig. 169 HPLC spectrum of (S_p)-**3u**

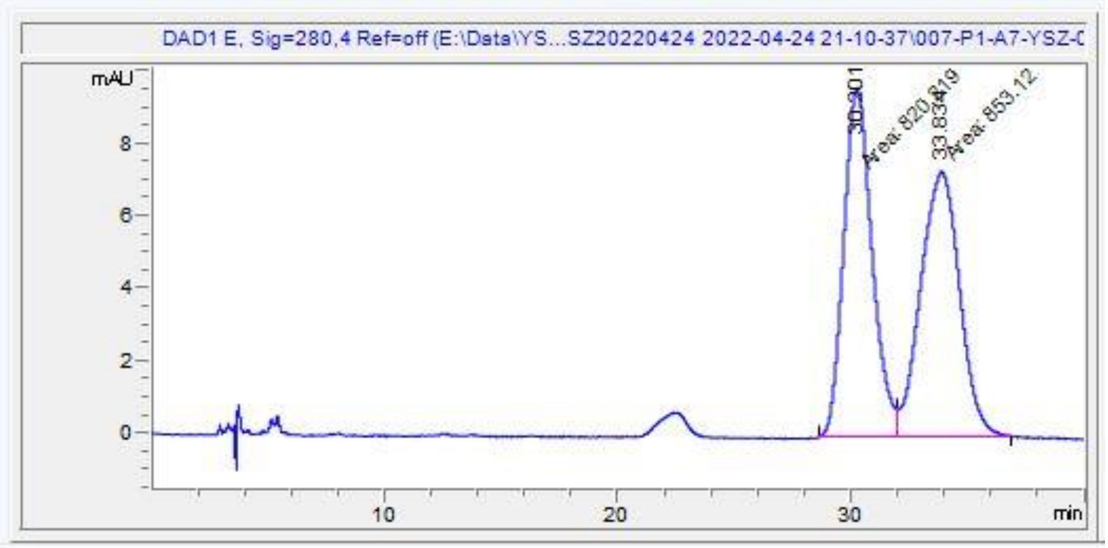

#	Time	Type	Area	Height	Width	Area%	Symmetry
1	30.201	MF	820.2	9.7	1.4134	49.017	0
2	33.834	FM	853.1	7.4	1.9301	50.983	1.109

Supplementary Fig. 170 Full HPLC spectrum of racemic **3u**

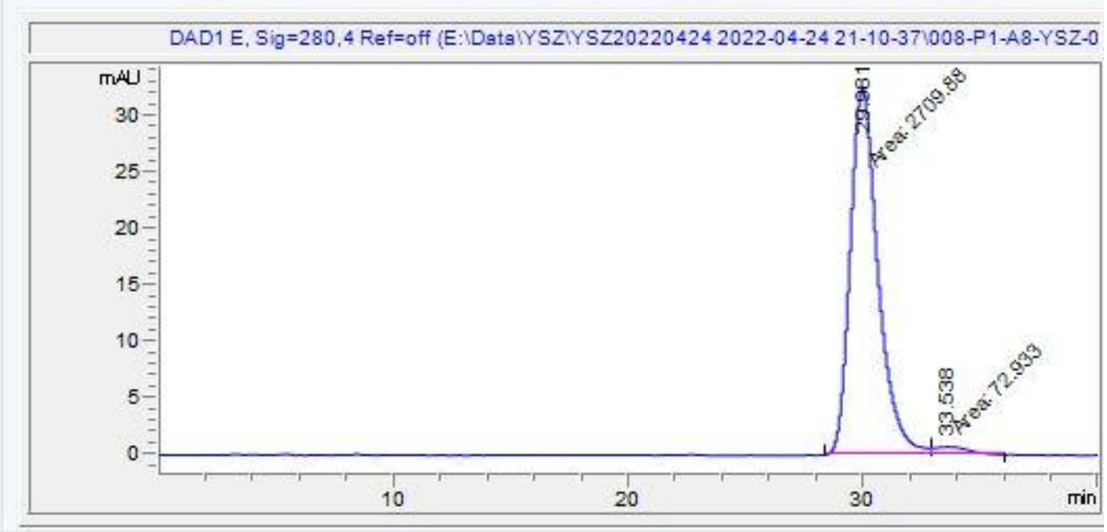

#	Time	Type	Area	Height	Width	Area%	Symmetry
1	29.981	MF	2709.9	32.8	1.379	97.379	0
2	33.538	FM	72.9	7.3E-1	1.6687	2.621	0.482

Supplementary Fig. 171 Full HPLC spectrum of (S_p)-**3u**

(R_p)-Methyl 4[3]-((tert-butoxycarbonyl)amino)-1,4(1,4)-dibenzenacyclohexaphane-1[2] -carboxylate **(1v)**

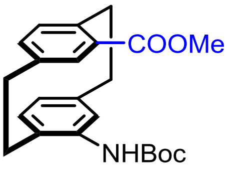

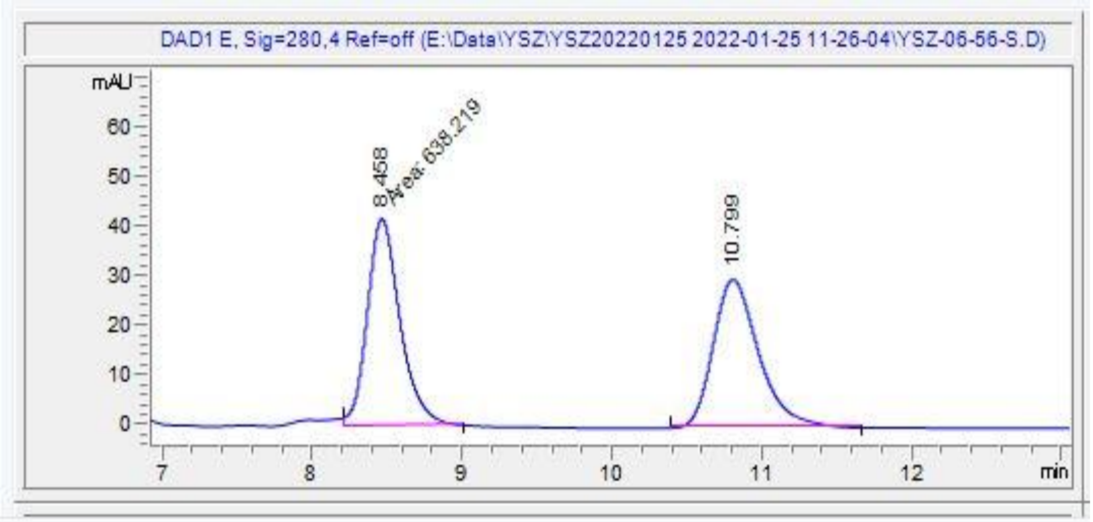

#	Time	Type	Area	Height	Width	Area%	Symmetry
1	8.458	FM	638.2	41.8	0.2547	50.067	0.788
2	10.799	BB	636.5	29.9	0.2585	49.933	0.747

Supplementary Fig. 172 HPLC spectrum of racemic **1v**

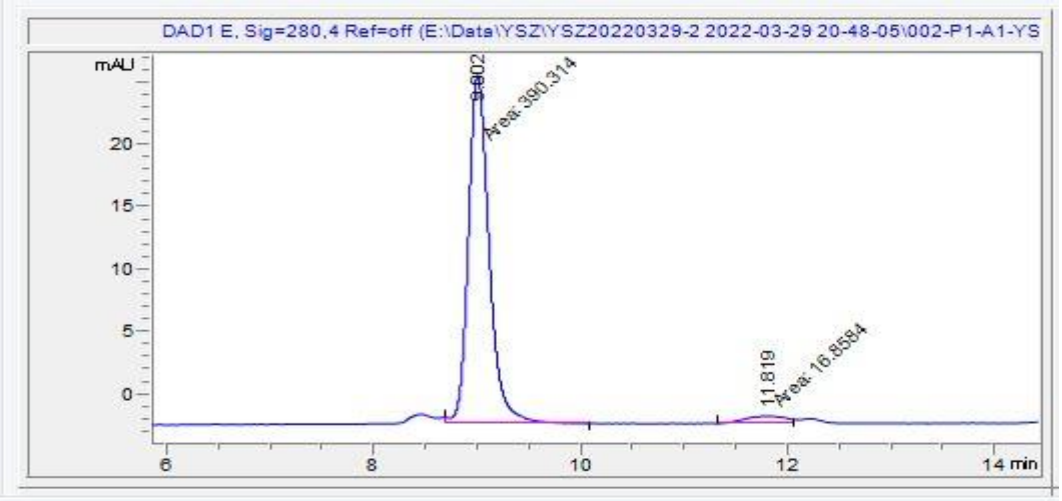

#	Time	Type	Area	Height	Width	Area%	Symmetry
1	9.002	FM	390.3	28	0.232	95.860	0.762
2	11.819	MF	16.9	6E-1	0.4697	4.140	1.388

Supplementary Fig. 173 HPLC spectrum of (R_p)-**1v**

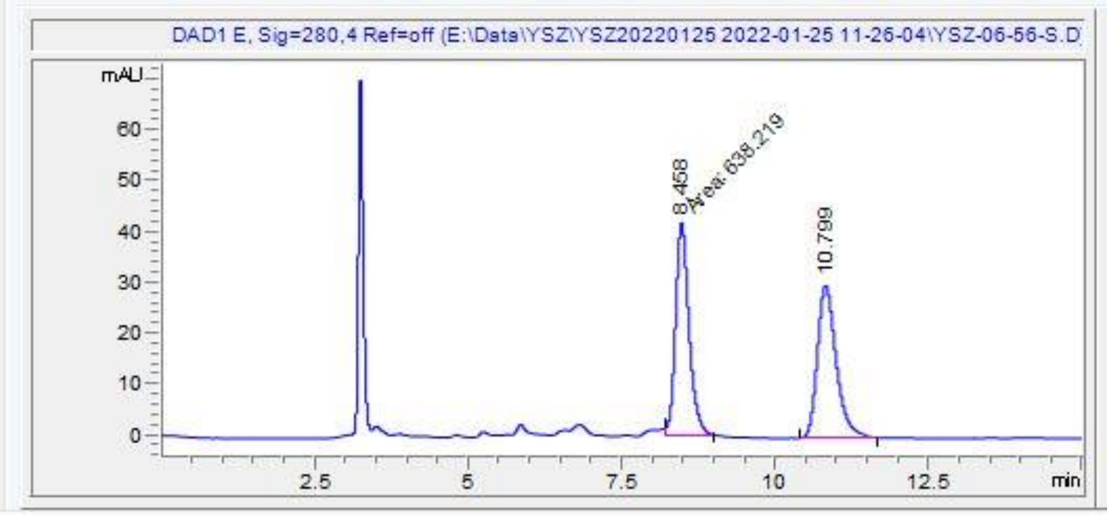

#	Time	Type	Area	Height	Width	Area%	Symmetry
1	8.458	FM	638.2	41.8	0.2547	50.067	0.788
2	10.799	BB	636.5	29.9	0.2585	49.933	0.747

Supplementary Fig. 174 Full HPLC spectrum of racemic **1v**

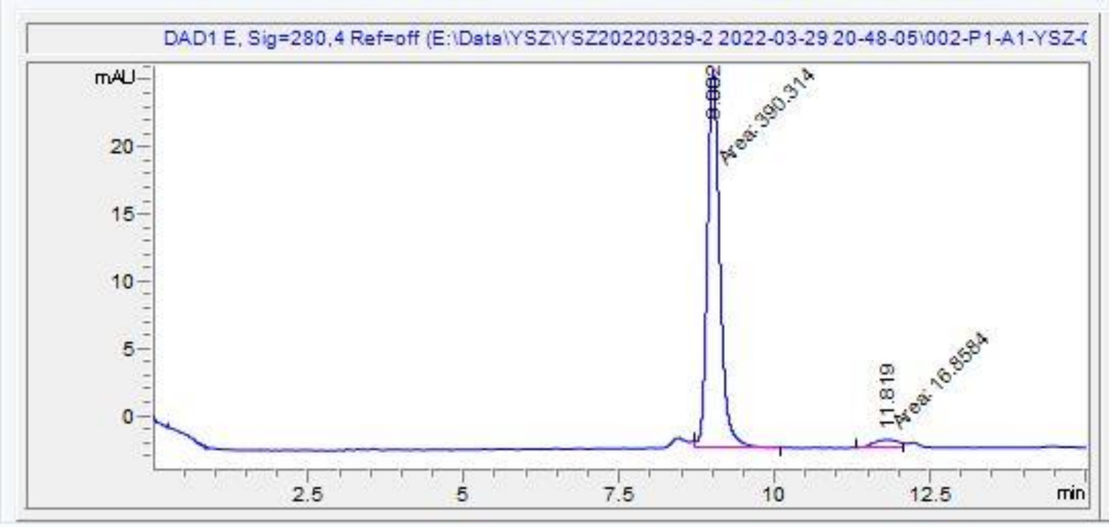

#	Time	Type	Area	Height	Width	Area%	Symmetry
1	9.002	FM	390.3	28	0.232	95.860	0.762
2	11.819	MF	16.9	6E-1	0.4697	4.140	1.388

Supplementary Fig. 175 Full HPLC spectrum of (R_p)-**1v**

(S_p)-Dibenzyl 1-(1^5-((tert-butoxycarbonyl)amino)-4^2-(methoxycarbonyl)-1,4(1,4)-dibenzenacyclohexaphane-1^2-yl)hydrazine-1,2-dicarboxylate **(3v)**

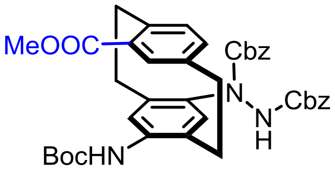

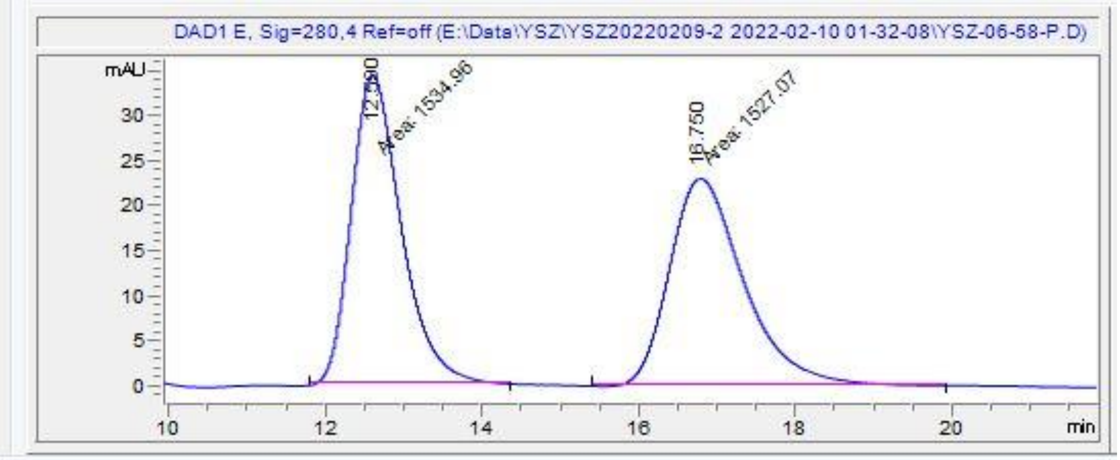

#	Time	Type	Area	Height	Width	Area%	Symmetry
1	12.59	MM	1535	34.6	0.7383	50.129	0.751
2	16.75	MM	1527.1	23.2	1.0954	49.871	0.682

Supplementary Fig. 176 HPLC spectrum of racemic **3v**

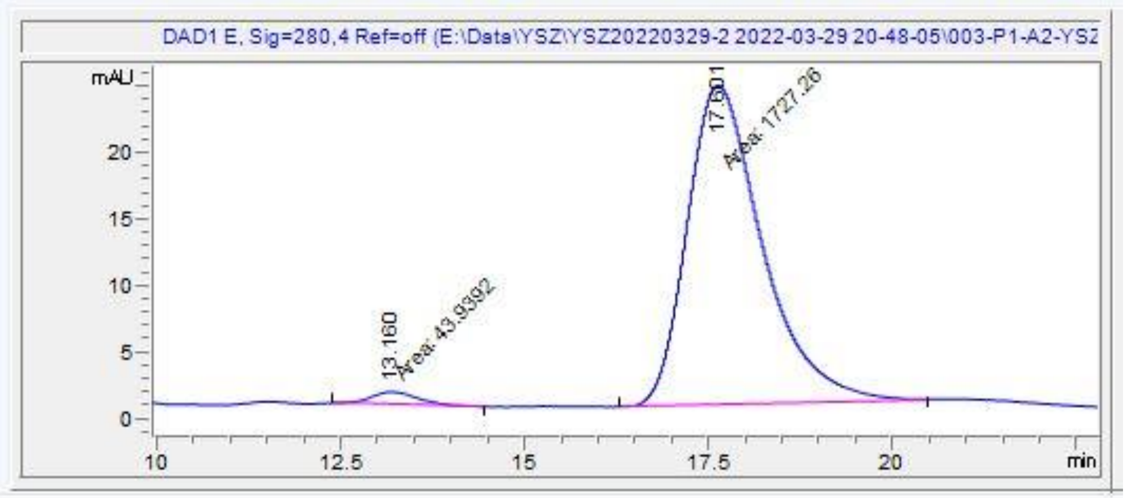

#	Time	Type	Area	Height	Width	Area%	Symmetry
1	13.16	MM	43.9	9.7E-1	0.7514	2.481	0.785
2	17.601	MM	1727.3	24.2	1.1888	97.519	0.65

Supplementary Fig. 177 HPLC spectrum of (S_p)-**3v**

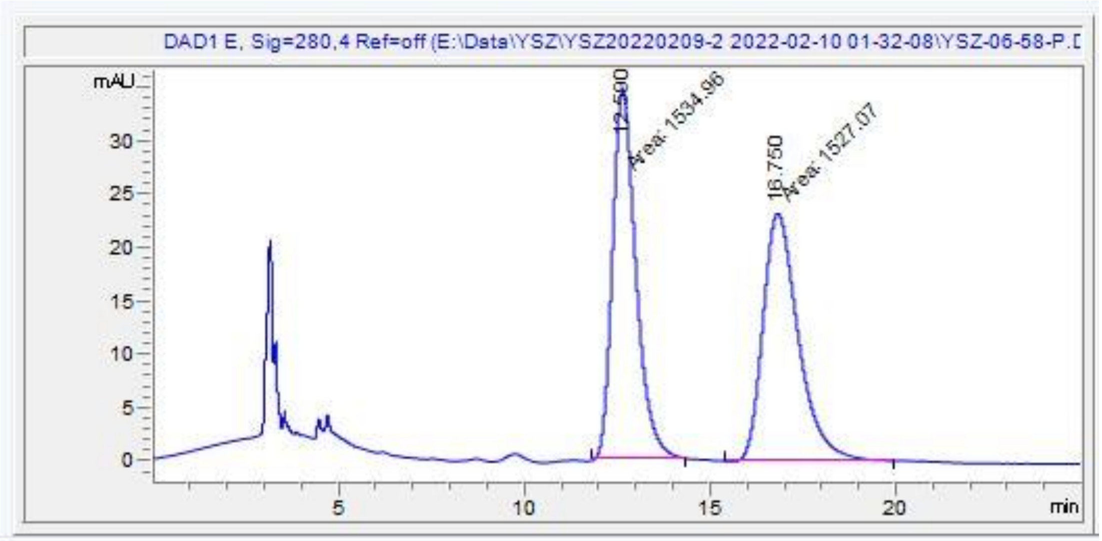

#	Time	Type	Area	Height	Width	Area%	Symmetry
1	12.59	MM	1535	34.6	0.7383	50.129	0.751
2	16.75	MM	1527.1	23.2	1.0954	49.871	0.682

Supplementary Fig. 178 Full HPLC spectrum of racemic **3v**

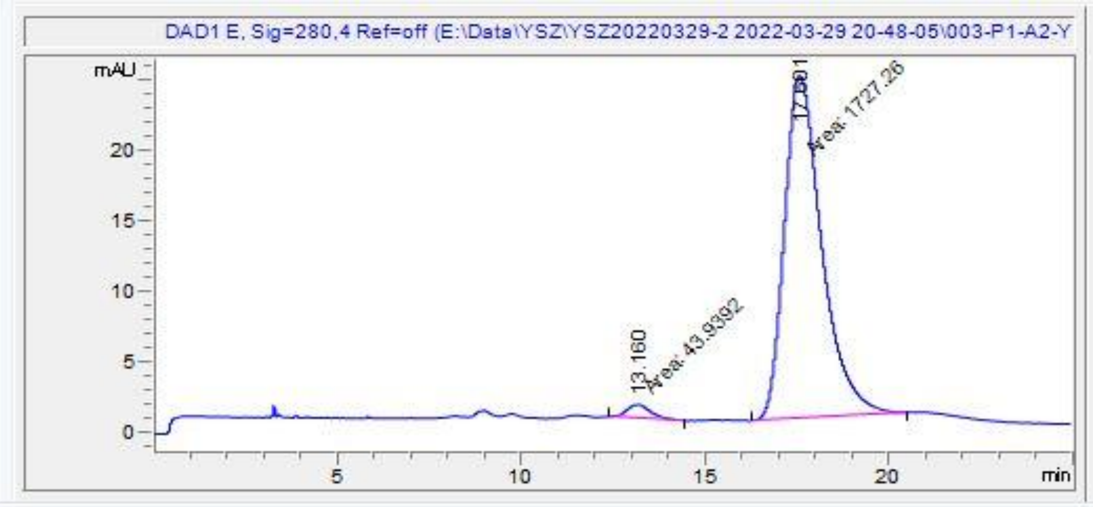

#	Time	Type	Area	Height	Width	Area%	Symmetry
1	13.16	MM	43.9	9.7E-1	0.7514	2.481	0.785
2	17.601	MM	1727.3	24.2	1.1888	97.519	0.65

Supplementary Fig. 179 Full HPLC spectrum of (S_p)-**3v**

(R_p)-Tert-butyl (4^2-bromo-1,4(1,4)-dibenzenacyclohexaphane-1^2-yl)carbamate **(1w)**

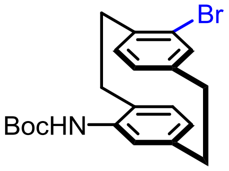

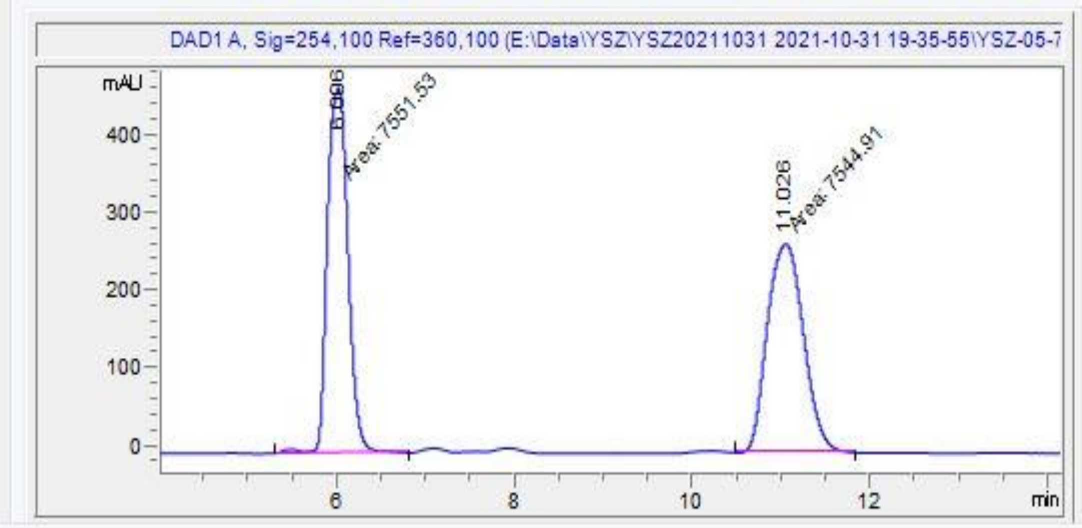

#	Time	Type	Area	Height	Width	Area%	Symmetry
1	6.006	MM	7551.5	467	0.2695	50.022	1.07
2	11.026	MM	7544.9	266.7	0.4715	49.978	1.002

Supplementary Fig. 180 HPLC spectrum of racemic **1w**

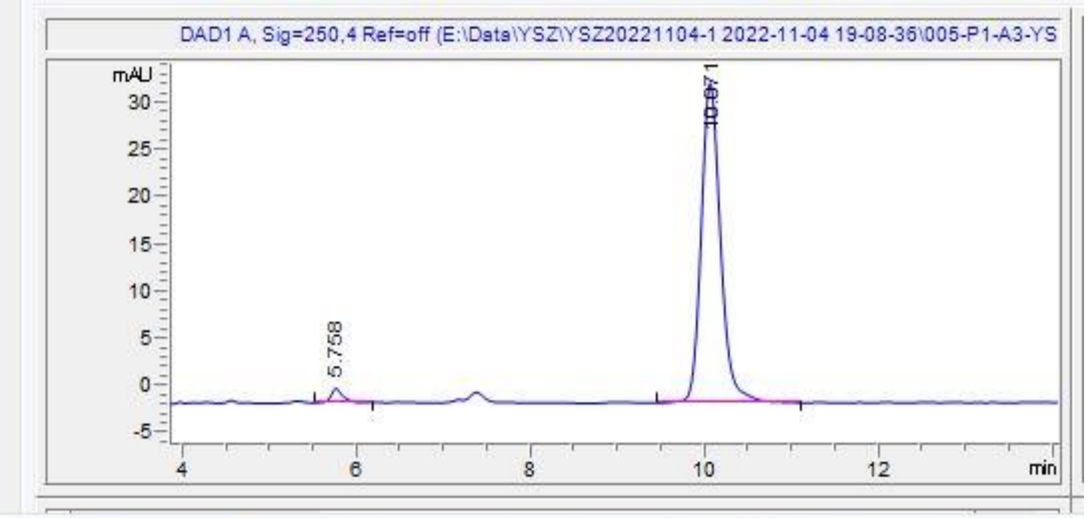

#	Time	Type	Area	Height	Width	Area%	Symmetry
1	5.758	BB	14.2	1.5	0.1367	2.619	0.685
2	10.071	BB	528.3	34.1	0.2369	97.381	0.805

Supplementary Fig. 181 HPLC spectrum of (R_p)-**1w**

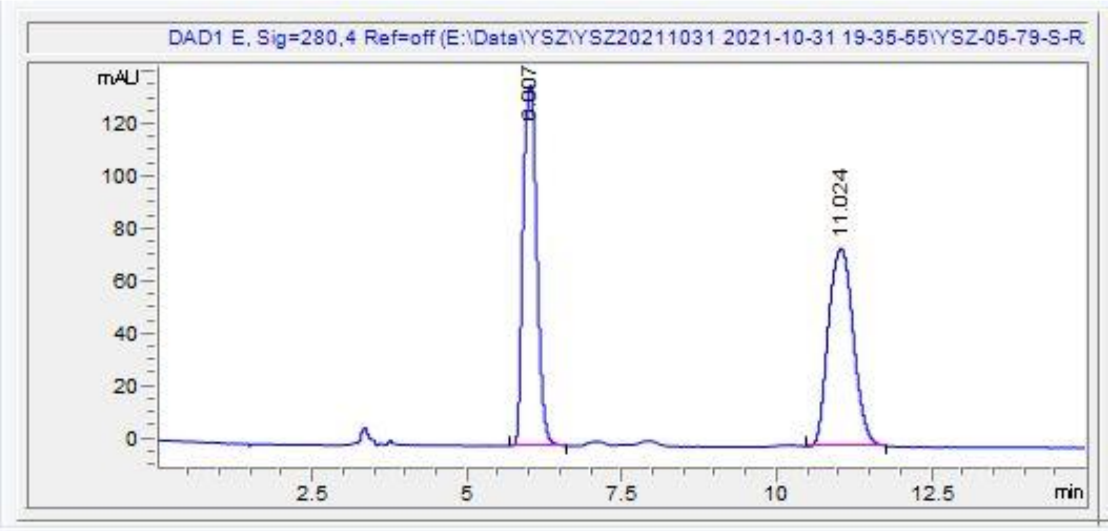

#	Time	Type	Area	Height	Width	Area%	Symmetry
1	6.007	BV R	2135.8	137.7	0.2119	50.223	1.075
2	11.024	BV R	2116.9	75.5	0.3284	49.777	0.992

Supplementary Fig. 182 Full HPLC spectrum of racemic **1w**

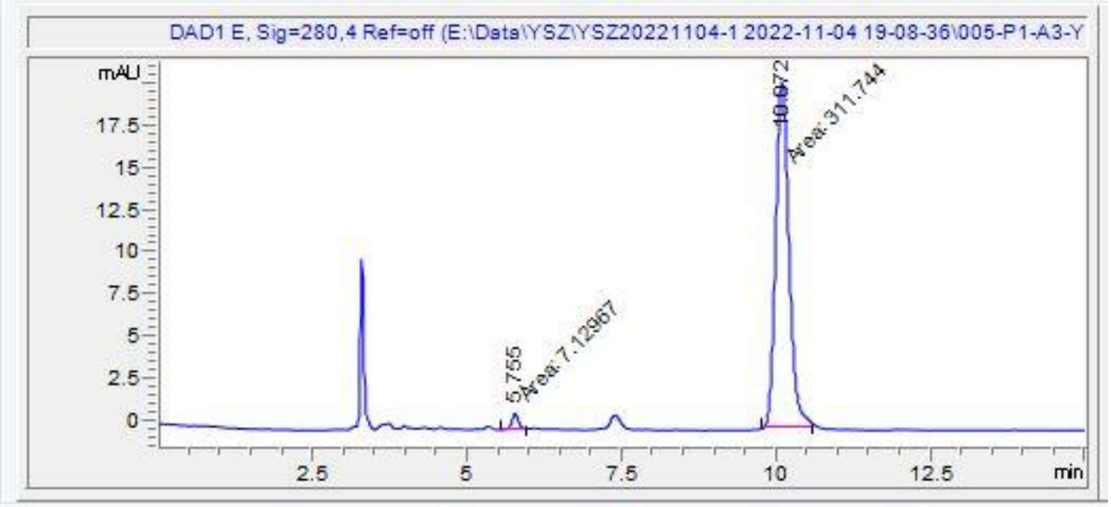

#	Time	Type	Area	Height	Width	Area%	Symmetry
1	5.755	MM	7.1	9.4E-1	0.1267	2.236	0.876
2	10.072	MM	311.7	20.6	0.2524	97.764	0.825

Supplementary Fig. 183 Full HPLC spectrum of (R_p)-**1w**

(S_p)-Dibenzyl 1-(4^3-bromo-1^5-((tert-butoxycarbonyl)amino)-1,4(1,4)-dibenzenacyclohexaphane-1^2-yl)hydrazine-1,2-dicarboxylate **(3w)**

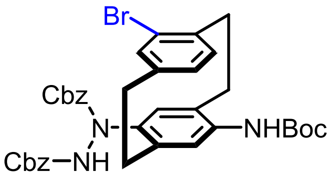

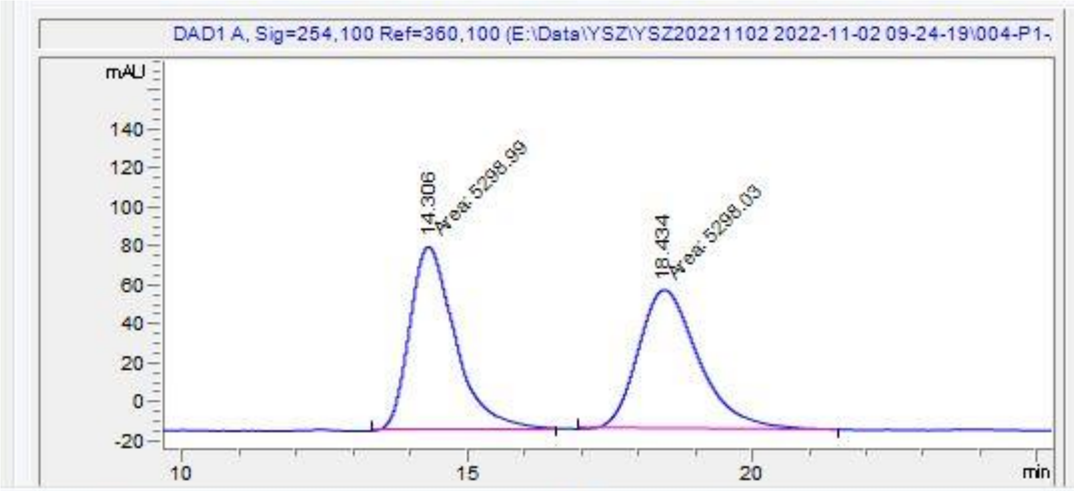

#	Time	Type	Area	Height	Width	Area%	Symmetry
1	14.306	MM	5299	94.2	0.9379	50.005	0.667
2	18.434	MM	5298	71.3	1.2383	49.995	0.69

Supplementary Fig. 184 HPLC spectrum of racemic **3w**

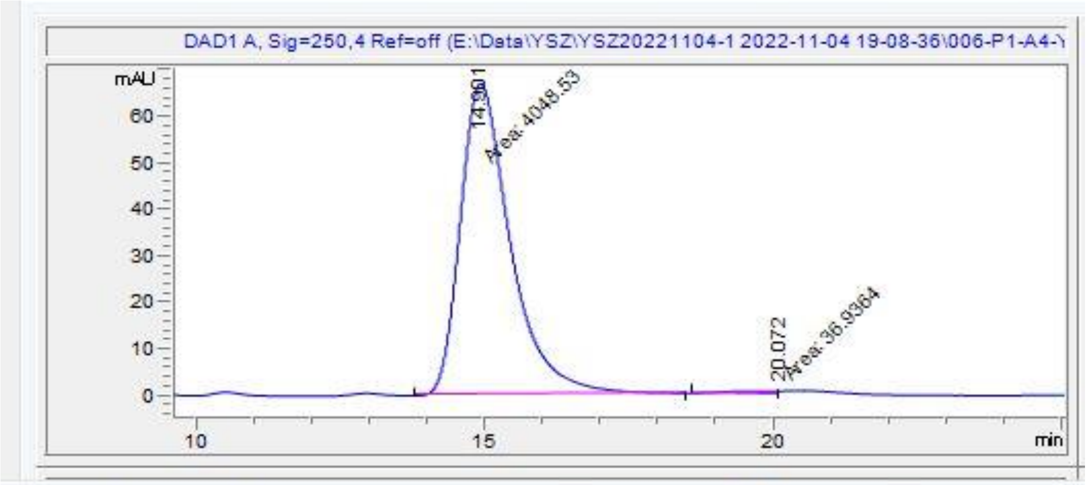

#	Time	Type	Area	Height	Width	Area%	Symmetry
1	14.901	MM	4048.5	66.8	1.0099	99.096	0.652
2	20.072	MM	36.9	6E-1	1.0323	0.904	18.854

Supplementary Fig. 185 HPLC spectrum of (S_p)-**3w**

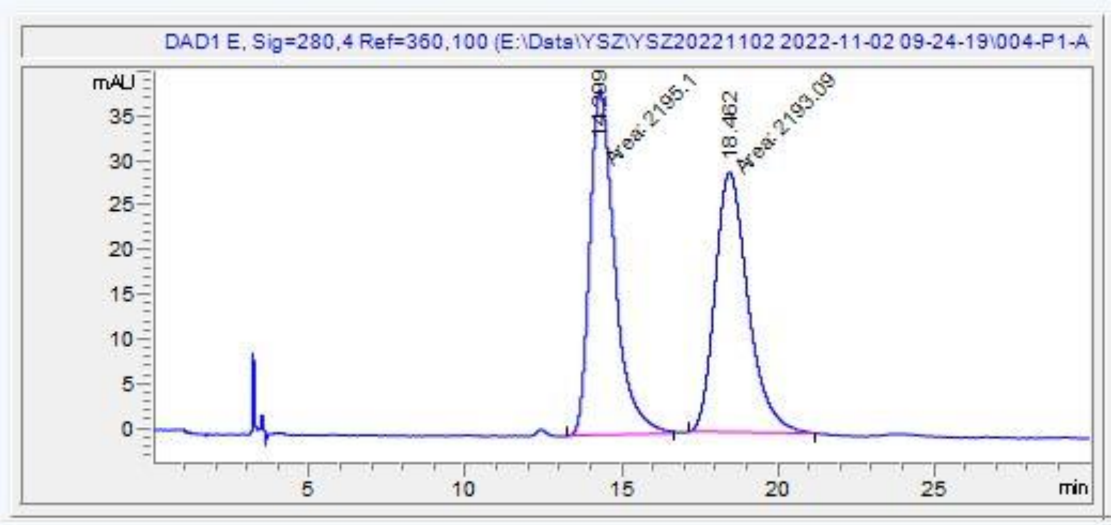

#	Time	Type	Area	Height	Width	Area%	Symmetry
1	14.299	MM	2195.1	38.8	0.942	50.023	0.642
2	18.462	MM	2193.1	29.4	1.2445	49.977	0.755

Supplementary Fig. 186 Full HPLC spectrum of racemic **3w**

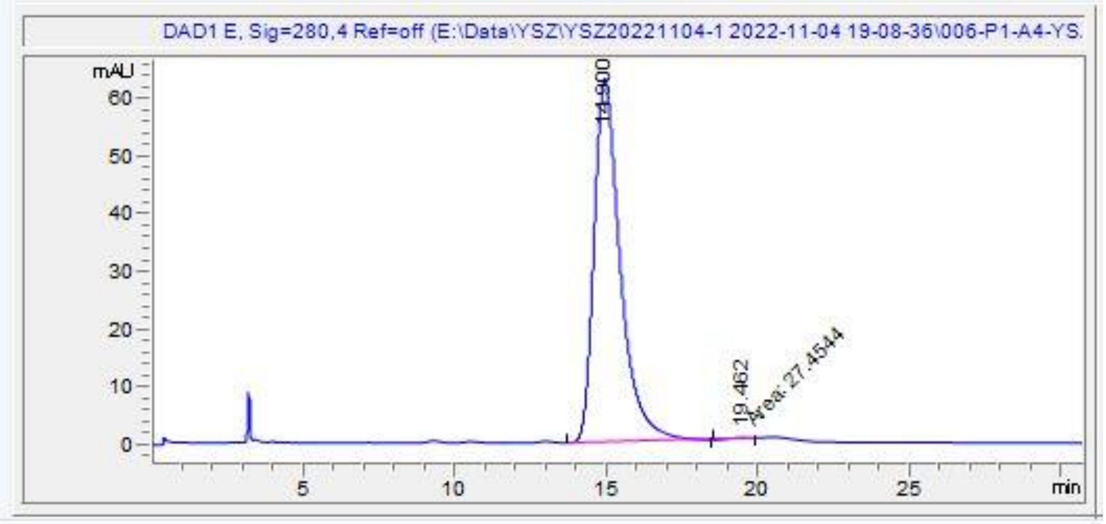

#	Time	Type	Area	Height	Width	Area%	Symmetry
1	14.9	BB	3828.4	63.2	0.8791	99.288	0.632
2	19.462	MM	27.5	4.7E-1	0.9796	0.712	1.312

Supplementary Fig. 187 Full HPLC spectrum of (S_p)-**3w**

(R_p)-Tert-butyl (4^2-phenyl-1,4(1,4)-dibenzenacyclohexaphane-1^2-yl)carbamate **(1x)**

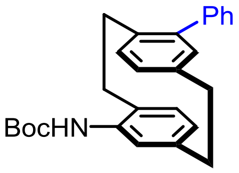

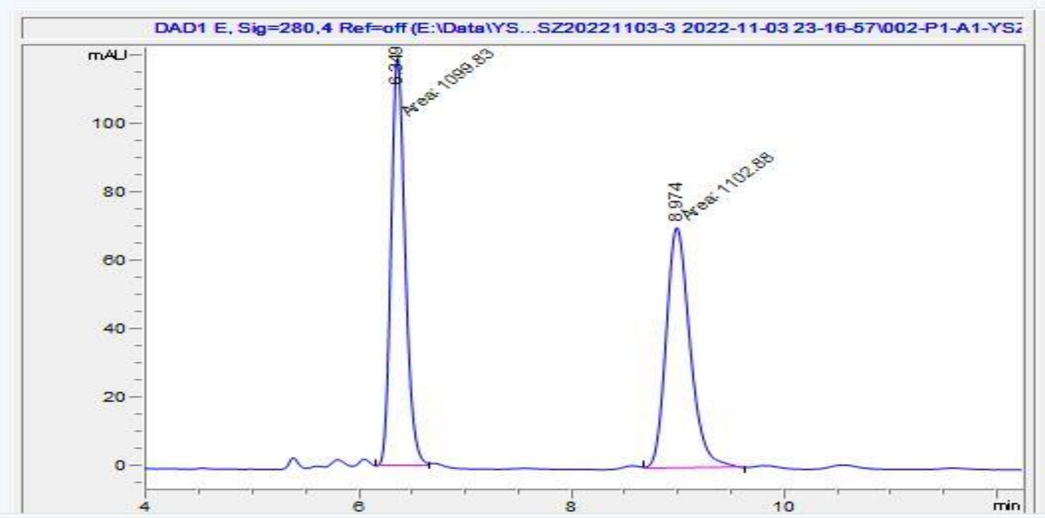

#	Time	Type	Area	Height	Width	Area%	Symmetry
1	6.349	MM	1099.8	119.3	0.1537	49.931	0.806
2	8.974	FM	1102.9	70.5	0.2608	50.069	0.777

Supplementary Fig. 188 HPLC spectrum of racemic **1x**

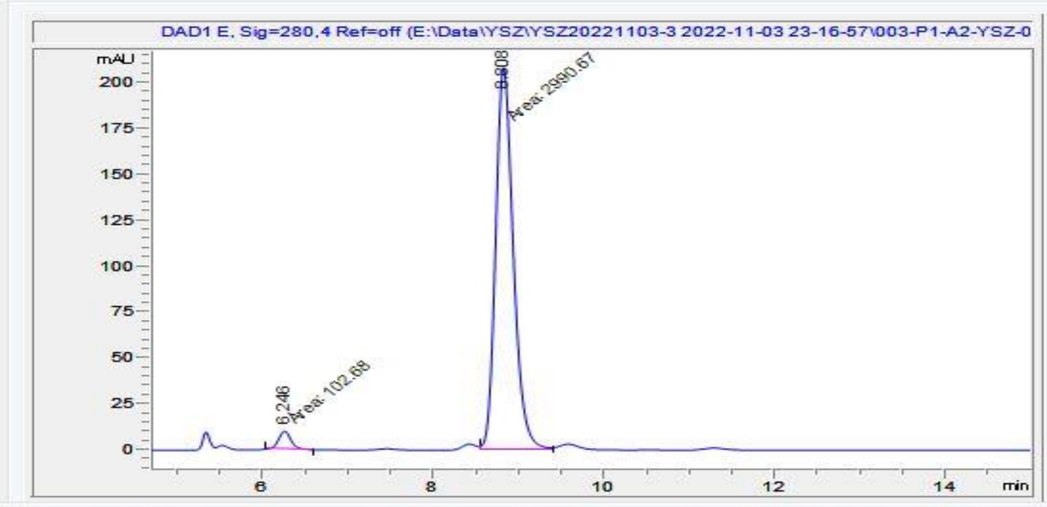

#	Time	Type	Area	Height	Width	Area%	Symmetry
1	6.246	MM	102.7	9.9	0.1734	3.319	0.845
2	8.808	MF	2990.7	207.5	0.2402	96.681	0

Supplementary Fig. 189 HPLC spectrum of (R_p)-**1x**

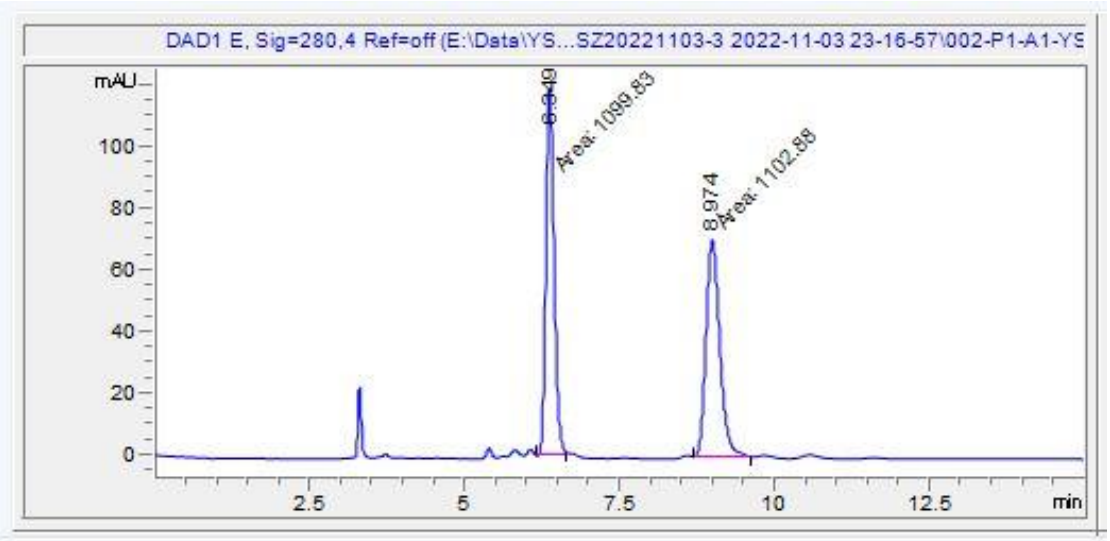

#	Time	Type	Area	Height	Width	Area%	Symmetry
1	6.349	MM	1099.8	119.3	0.1537	49.931	0.806
2	8.974	FM	1102.9	70.5	0.2608	50.069	0.777

Supplementary Fig. 190 Full HPLC spectrum of racemic **1x**

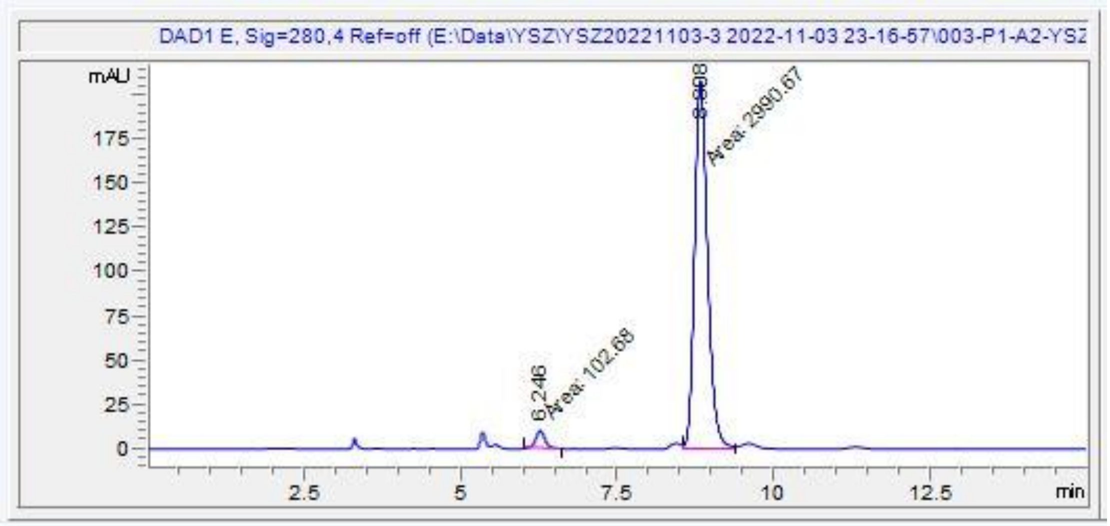

#	Time	Type	Area	Height	Width	Area%	Symmetry
1	6.246	MM	102.7	9.9	0.1734	3.319	0.845
2	8.808	MF	2990.7	207.5	0.2402	96.681	0

Supplementary Fig. 191 Full HPLC spectrum of (R_p)-**1x**

(S_p)-Dibenzyl 1-(1[5]-((tert-butoxycarbonyl)amino)-4[3]-phenyl-1,4(1,4)-dibenzenacyclohexaphane-1[2]-yl)hydrazine-1,2-dicarboxylate **(3x)**

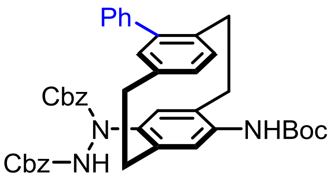

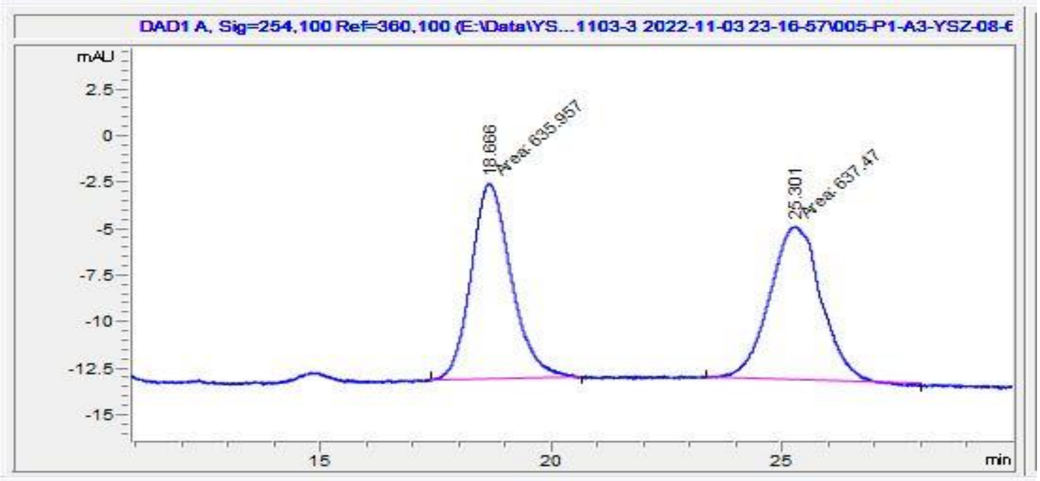

#	Time	Type	Area	Height	Width	Area%	Symmetry
1	18.666	MM	636	10.5	1.0101	49.941	0.827
2	25.301	MM	637.5	8.2	1.2904	50.059	0.97

Supplementary Fig. 192 HPLC spectrum of racemic **3x**

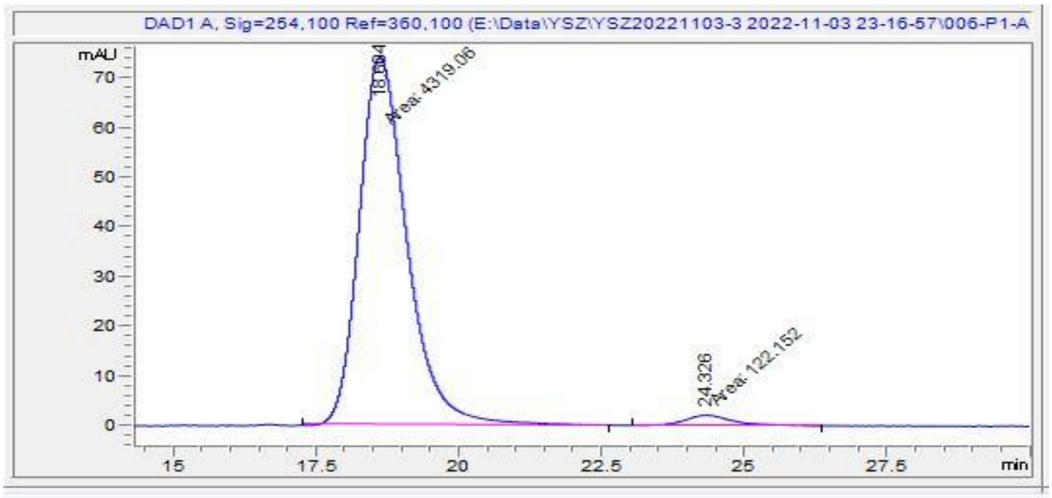

#	Time	Type	Area	Height	Width	Area%	Symmetry
1	18.604	MM	4319.1	74.1	0.9714	97.250	0.74
2	24.326	MM	122.2	2.1	0.9474	2.750	0.849

Supplementary Fig. 193 HPLC spectrum of (S_p)-**3x**

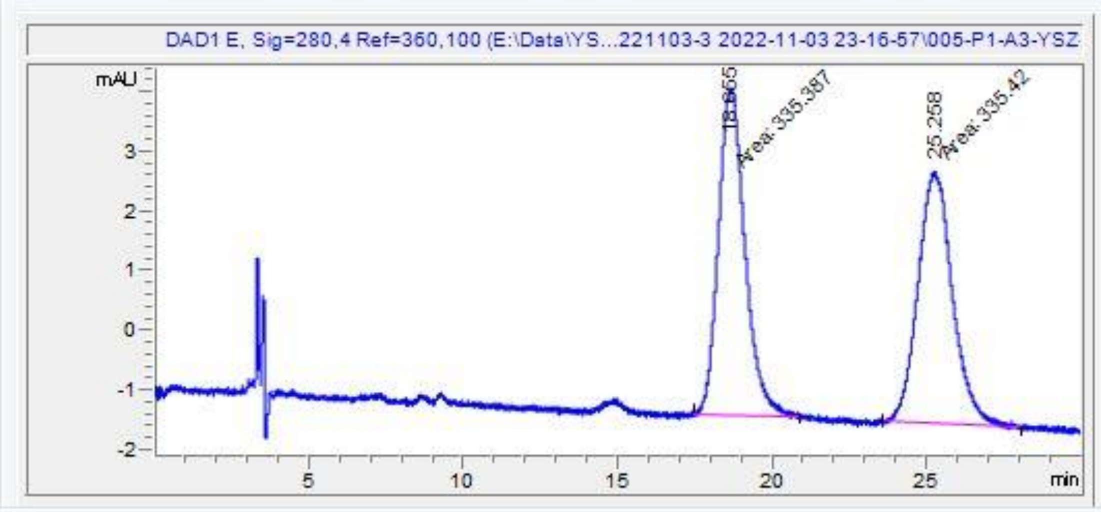

#	Time	Type	Area	Height	Width	Area%	Symmetry
1	18.655	MM	335.4	5.5	1.0094	49.998	0.723
2	25.258	MM	335.4	4.3	1.3093	50.002	0.797

Supplementary Fig. 194 Full HPLC spectrum of racemic **3x**

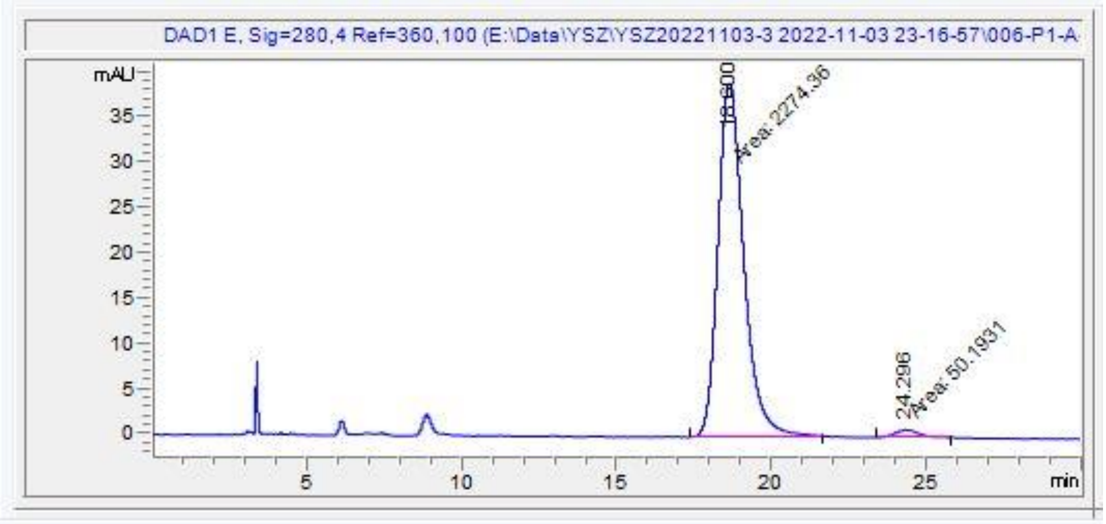

#	Time	Type	Area	Height	Width	Area%	Symmetry
1	18.6	MM	2274.4	39.2	0.9662	97.841	0.735
2	24.296	MM	50.2	9.3E-1	0.8977	2.159	0.782

Supplementary Fig. 195 Full HPLC spectrum of (S_p)-**3x**

(R_p)-Tert-butyl (4^2-(cyclohex-1-en-1-yl)-1,4(1,4)-dibenzenacyclohexaphane -1^2yl)carbamate **(1y)**

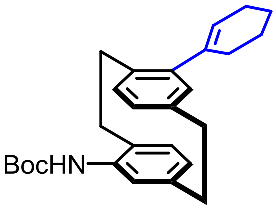

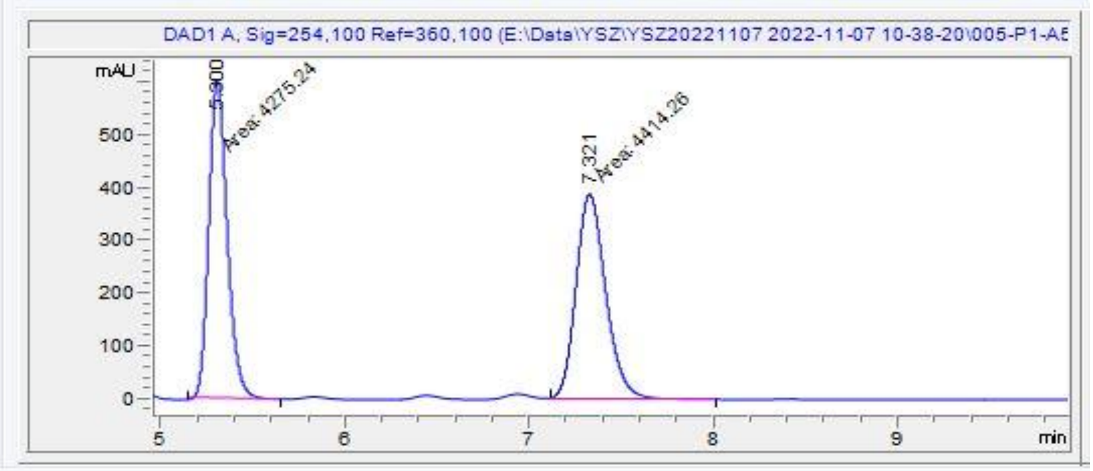

#	Time	Type	Area	Height	Width	Area%	Symmetry
1	5.3	MM	4275.2	610	0.1168	49.200	0.791
2	7.321	FM	4414.3	391.8	0.1878	50.800	0.795

Supplementary Fig. 196 HPLC spectrum of racemic **1y**

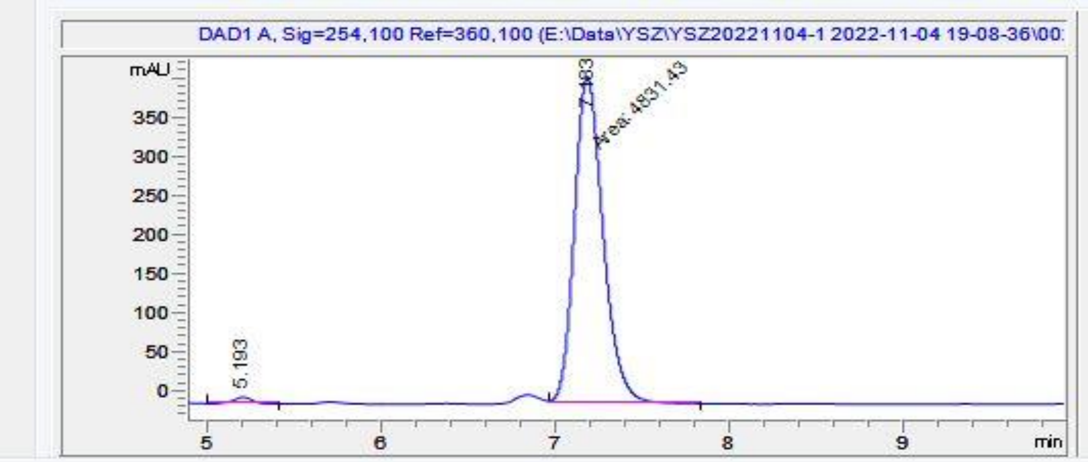

#	Time	Type	Area	Height	Width	Area%	Symmetry
1	5.193	BV R	67	8.7	0.1046	1.369	1.008
2	7.183	FM	4831.4	418.8	0.1923	98.631	0.779

Supplementary Fig. 197 HPLC spectrum of (R_p)-**1y**

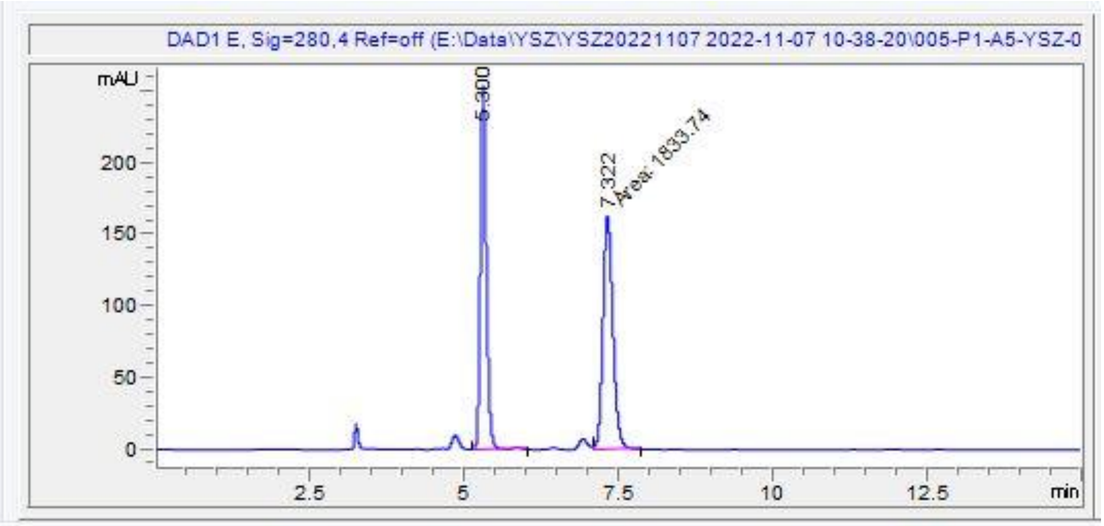

#	Time	Type	Area	Height	Width	Area%	Symmetry
1	5.3	VV R	1773.1	253.9	0.1062	49.159	0.782
2	7.322	FM	1833.7	162.6	0.1879	50.841	0.801

Supplementary Fig. 198 Full HPLC spectrum of racemic **1y**

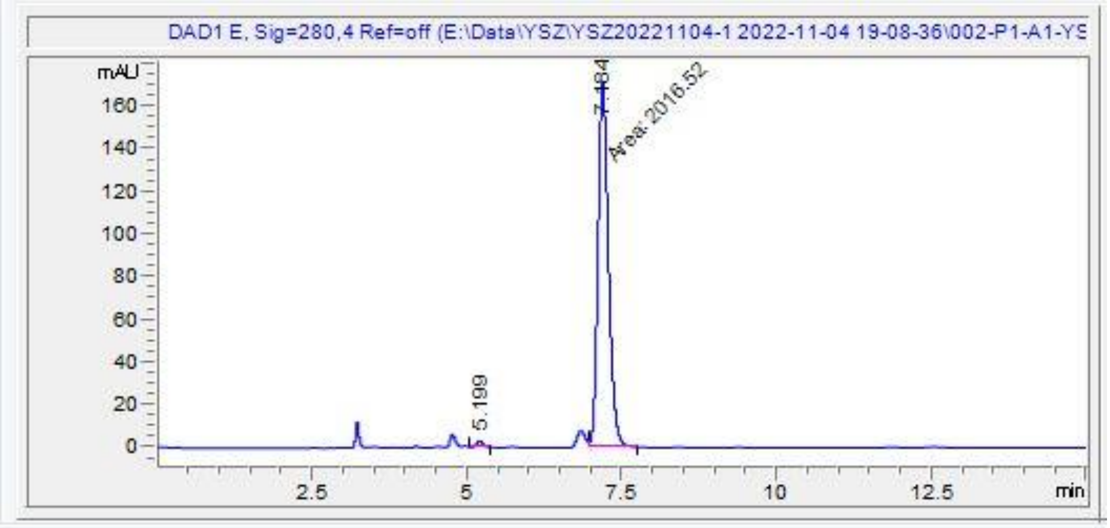

#	Time	Type	Area	Height	Width	Area%	Symmetry
1	5.199	BB	23.1	3.2	0.0857	1.131	1.098
2	7.184	FM	2016.5	172.4	0.195	98.869	0.768

Supplementary Fig. 199 Full HPLC spectrum of (R_p)-**1y**

(S_p)-Dibenzyl 1-(1^5-((tert-butoxycarbonyl)amino)-4^3-(cyclohex-1-en-1-yl)-1,4(1,4)-dibenzenacyclohexaphane-1^2-yl)hydrazine-1,2-dicarboxylate **(3y)**

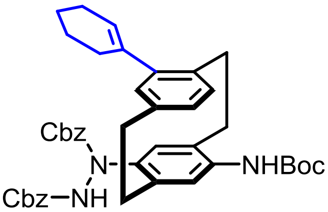

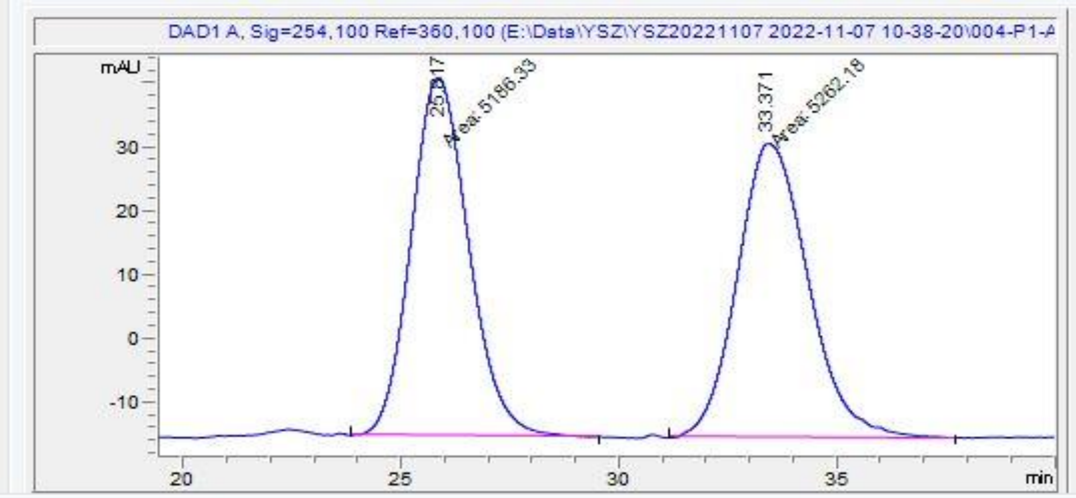

#	Time	Type	Area	Height	Width	Area%	Symmetry
1	25.817	MM	5186.3	56.4	1.5317	49.637	0.855
2	33.371	MM	5262.2	46.4	1.8902	50.363	0.78

Supplementary Fig. 200 HPLC spectrum of racemic **3y**

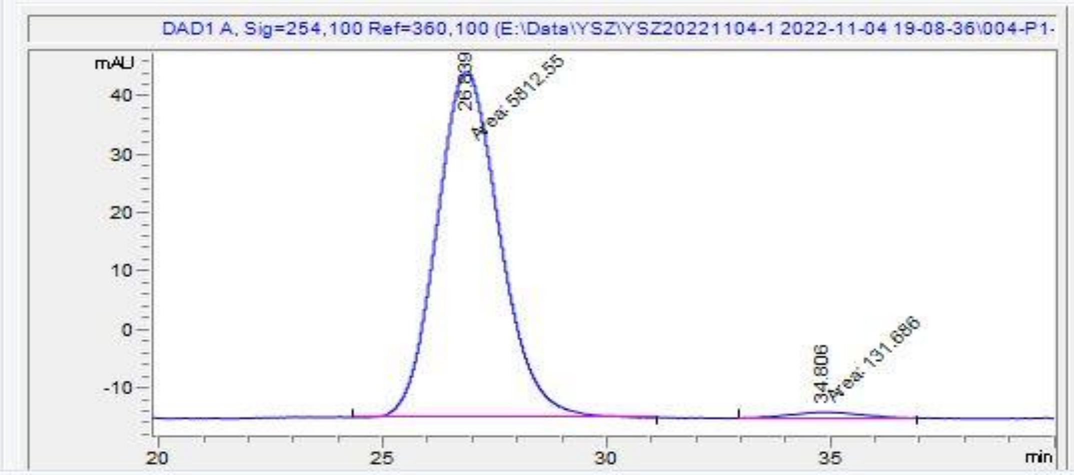

#	Time	Type	Area	Height	Width	Area%	Symmetry
1	26.839	MM	5812.6	59.3	1.6324	97.785	0.882
2	34.806	MM	131.7	1.1	1.9244	2.215	0.715

Supplementary Fig. 201 HPLC spectrum of (S_p)-**3y**

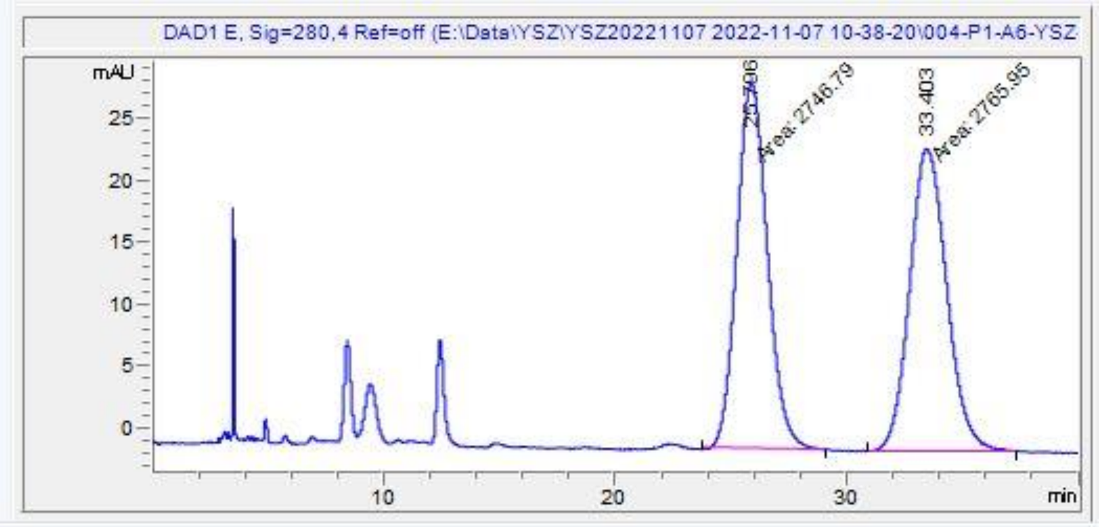

#	Time	Type	Area	Height	Width	Area%	Symmetry
1	25.796	MM	2746.8	29.8	1.5368	49.826	0.824
2	33.403	MM	2765.9	24.6	1.8734	50.174	0.849

Supplementary Fig. 202 Full HPLC spectrum of racemic **3y**

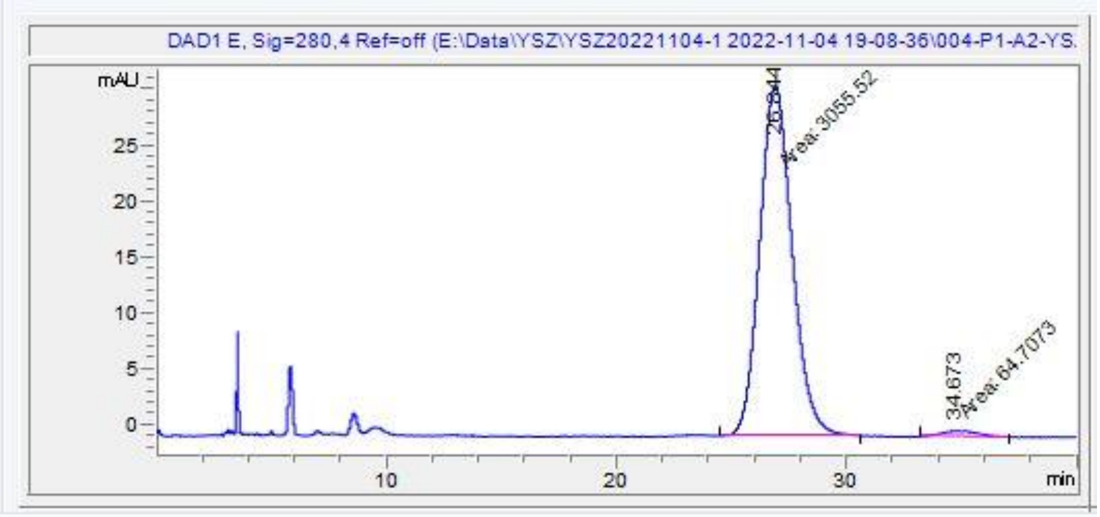

#	Time	Type	Area	Height	Width	Area%	Symmetry
1	26.844	MM	3055.5	31.2	1.632	97.926	0.895
2	34.673	MM	64.7	6.1E-1	1.7818	2.074	0.649

Supplementary Fig. 203 Full HPLC spectrum of (S_p)-**3y**

(R_p)-Tert-butyl (4[2]-cyclohexyl-1,4(1,4)-dibenzenacyclohexaphane-1[2]-yl)carbamate **(1z)**

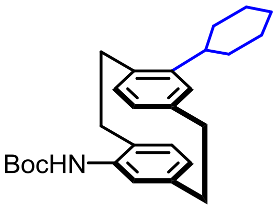

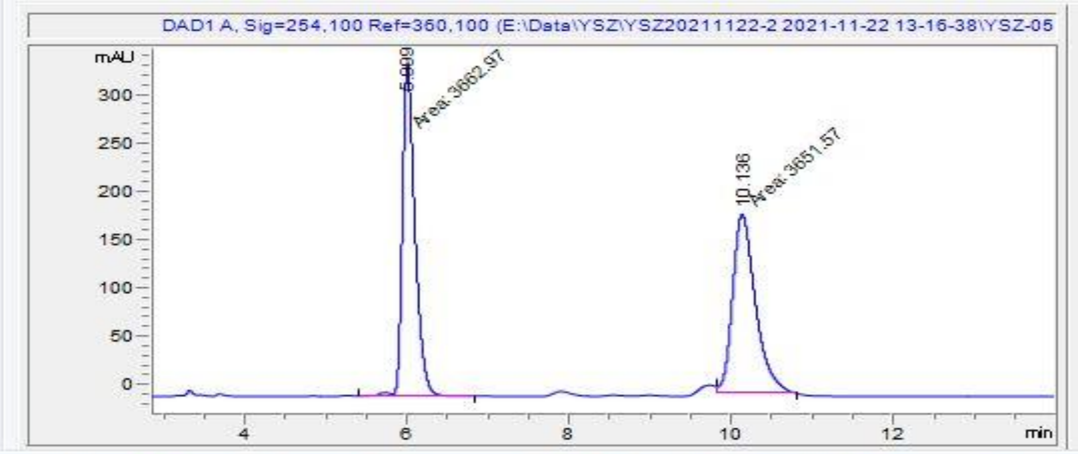

#	Time	Type	Area	Height	Width	Area%	Symmetry
1	5.999	MM	3663	342.5	0.1782	50.078	0.686
2	10.136	MM	3651.6	184.5	0.3298	49.922	0.697

Supplementary Fig. 204 HPLC spectrum of racemic **1z**

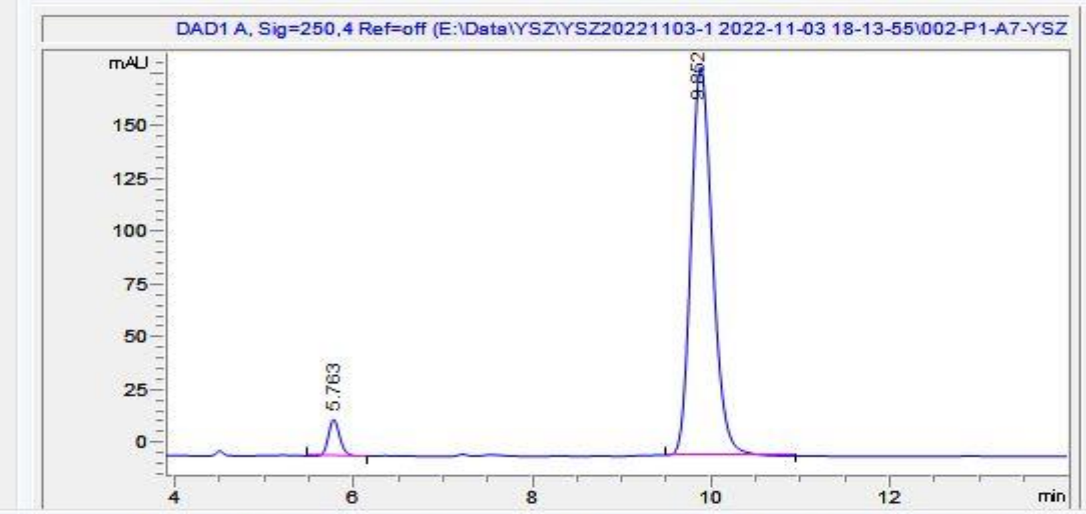

#	Time	Type	Area	Height	Width	Area%	Symmetry
1	5.763	BB	152.9	17.2	0.1359	4.767	0.819
2	9.852	BB	3054.1	183.3	0.2564	95.233	0.777

Supplementary Fig. 205 HPLC spectrum of (R_p)-**1z**

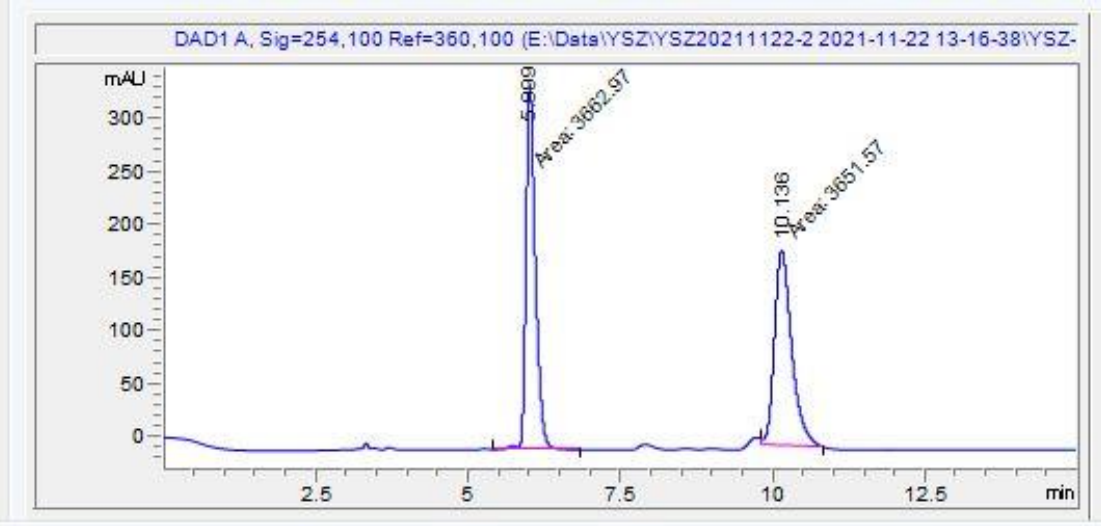

#	Time	Type	Area	Height	Width	Area%	Symmetry
1	5.999	MM	3663	342.5	0.1782	50.078	0.686
2	10.136	MM	3651.6	184.5	0.3298	49.922	0.697

Supplementary Fig. 206 Full HPLC spectrum of racemic **1z**

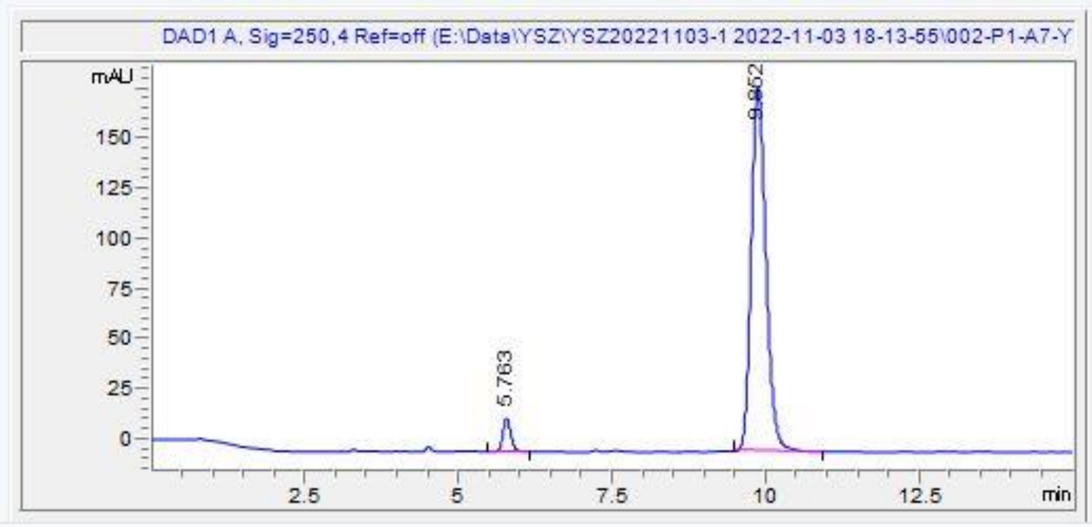

#	Time	Type	Area	Height	Width	Area%	Symmetry
1	5.763	BB	152.9	17.2	0.1359	4.767	0.819
2	9.852	BB	3054.1	183.3	0.2564	95.233	0.777

Supplementary Fig. 207 Full HPLC spectrum of (R_p)-**1z**

(S_p)-Dibenzyl 1-(1[5]-((tert-butoxycarbonyl)amino)-4[3]-cyclohexyl-1,4(1,4) -dibenzenacyclohexaphane-1[2]-yl)hydrazine-1,2-dicarboxylate **(3z)**

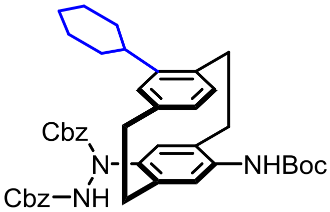

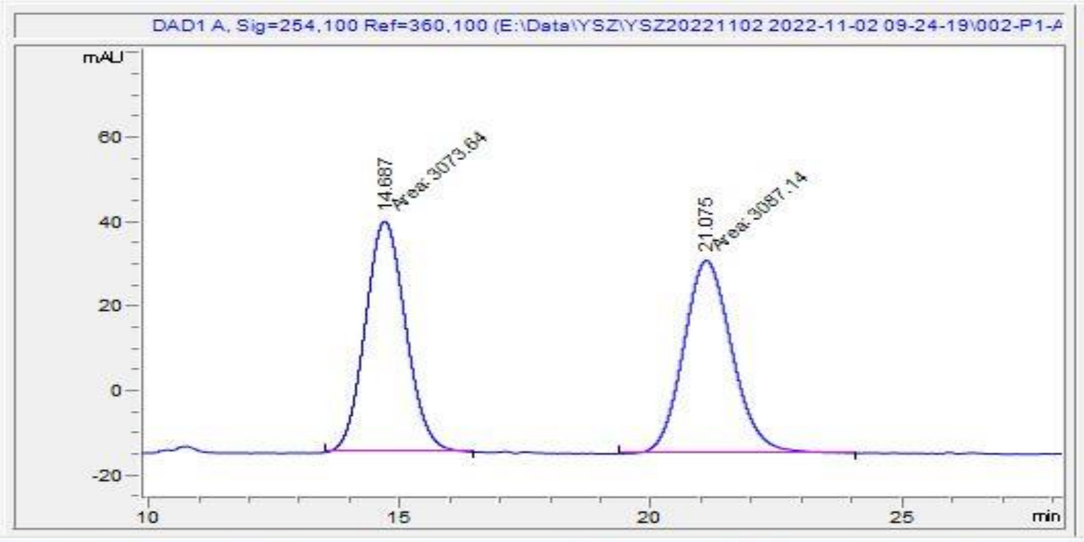

#	Time	Type	Area	Height	Width	Area%	Symmetry
1	14.687	MM	3073.6	54.8	0.9347	49.890	0.954
2	21.075	MM	3087.1	45.8	1.1235	50.110	0.867

Supplementary Fig. 208 HPLC spectrum of racemic **3z**

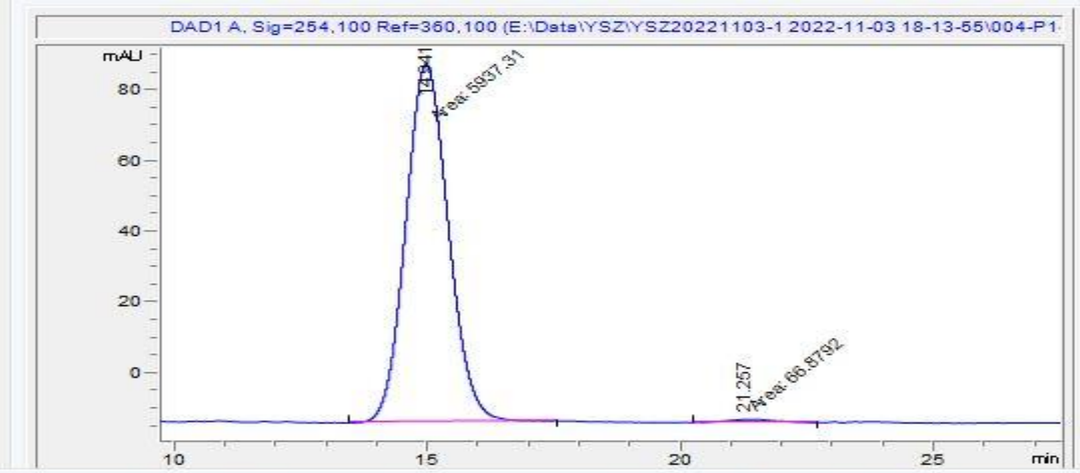

#	Time	Type	Area	Height	Width	Area%	Symmetry
1	14.941	MM	5937.3	102	0.9697	98.886	0.886
2	21.257	MM	66.9	9.8E-1	1.1328	1.114	0.684

Supplementary Fig. 209 HPLC spectrum of (S_p)-**3z**

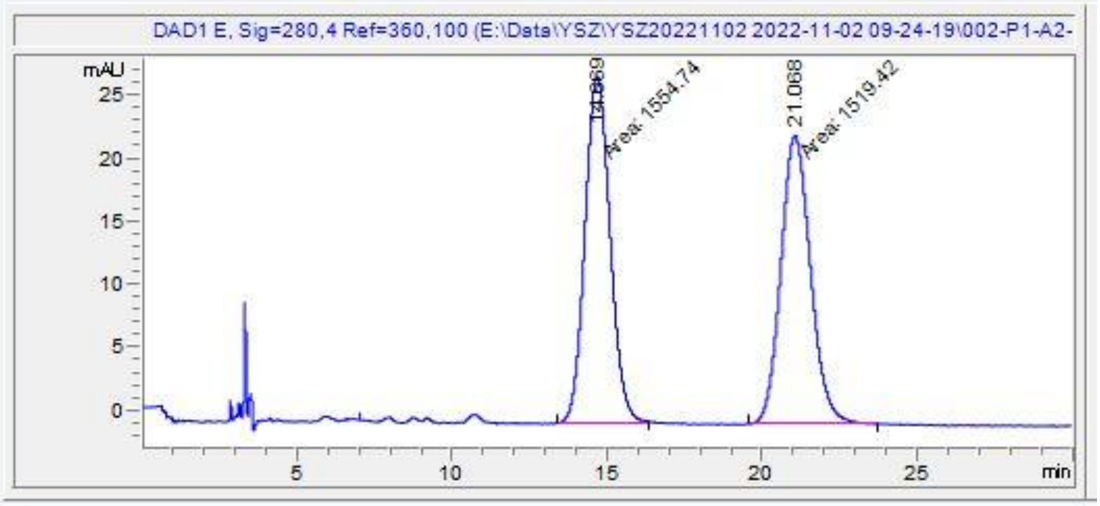

#	Time	Type	Area	Height	Width	Area%	Symmetry
1	14.669	MM	1554.7	27.5	0.9408	50.574	0.882
2	21.068	MM	1519.4	22.8	1.1104	49.426	0.856

Supplementary Fig. 210 Full HPLC spectrum of racemic **3z**

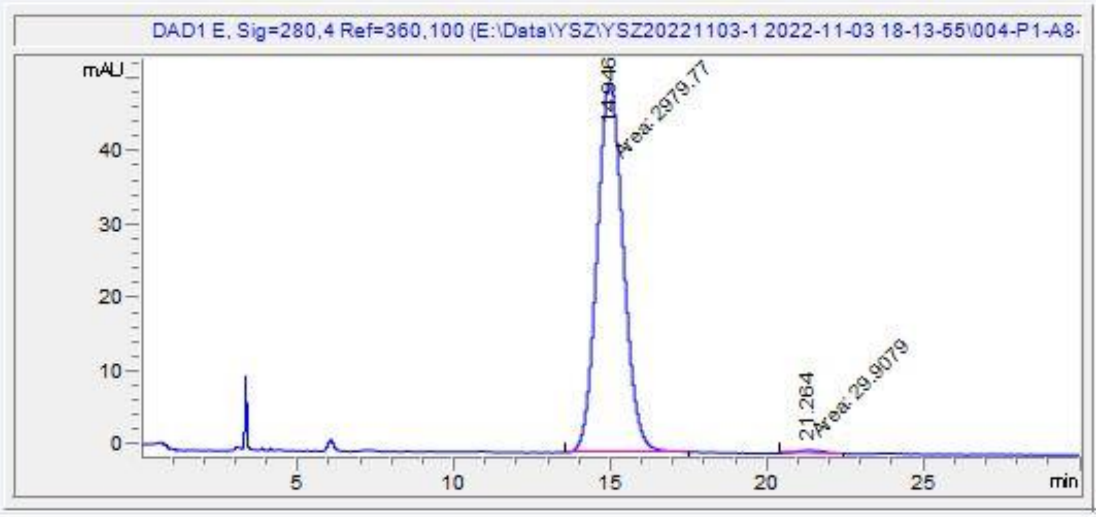

#	Time	Type	Area	Height	Width	Area%	Symmetry
1	14.946	MM	2979.8	51	0.9734	99.006	0.897
2	21.264	MM	29.9	5.1E-1	0.9796	0.994	0.699

Supplementary Fig. 211 Full HPLC spectrum of (S_p)-**1z**

(S_p)-Tert-butyl (4^3-bromo-1,4(1,4)-dibenzenacyclohexaphane-1^2-yl)carbamate **(1aa)**

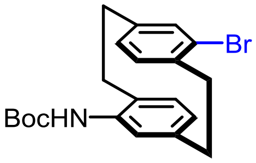

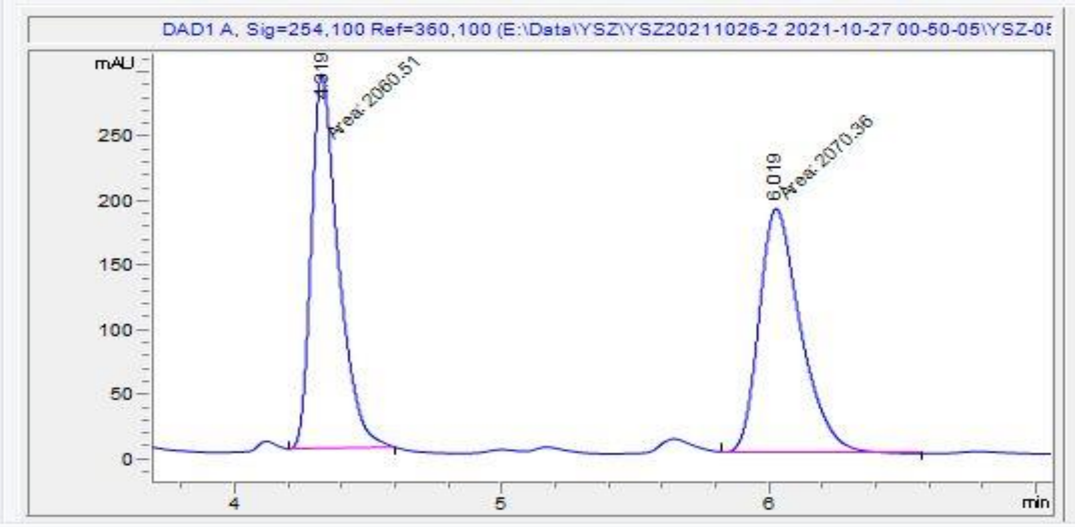

#	Time	Type	Area	Height	Width	Area%	Symmetry
1	4.319	MM	2060.5	290.9	0.1181	49.881	0.585
2	6.019	FM	2070.4	190.3	0.1814	50.119	0.679

Supplementary Fig. 212 HPLC spectrum of racemic **1aa**

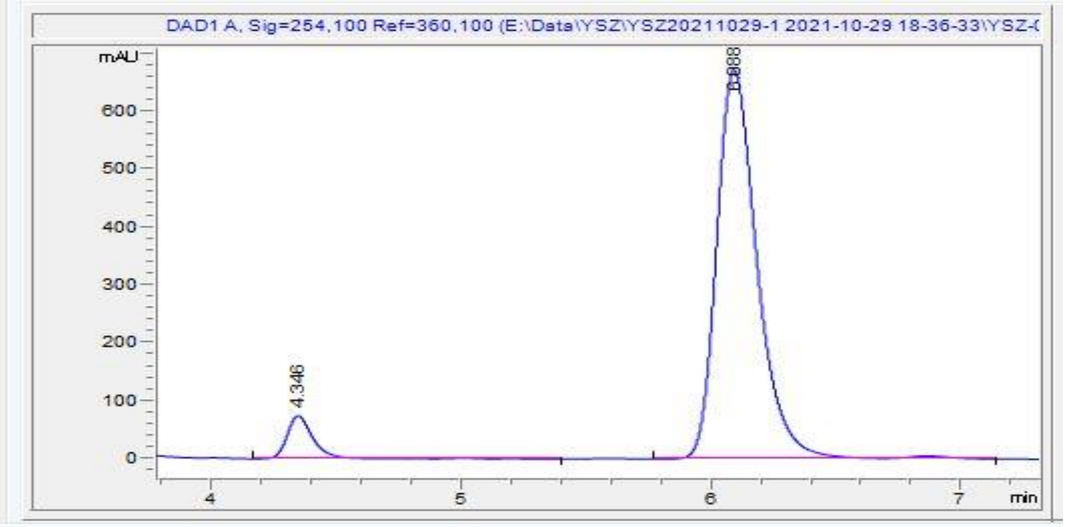

#	Time	Type	Area	Height	Width	Area%	Symmetry
1	4.346	BV R	560.6	74.6	0.1088	6.853	0.652
2	6.088	BV R	7620	674.8	0.1322	93.147	0.687

Supplementary Fig. 213 HPLC spectrum of (S_p)-**1aa**

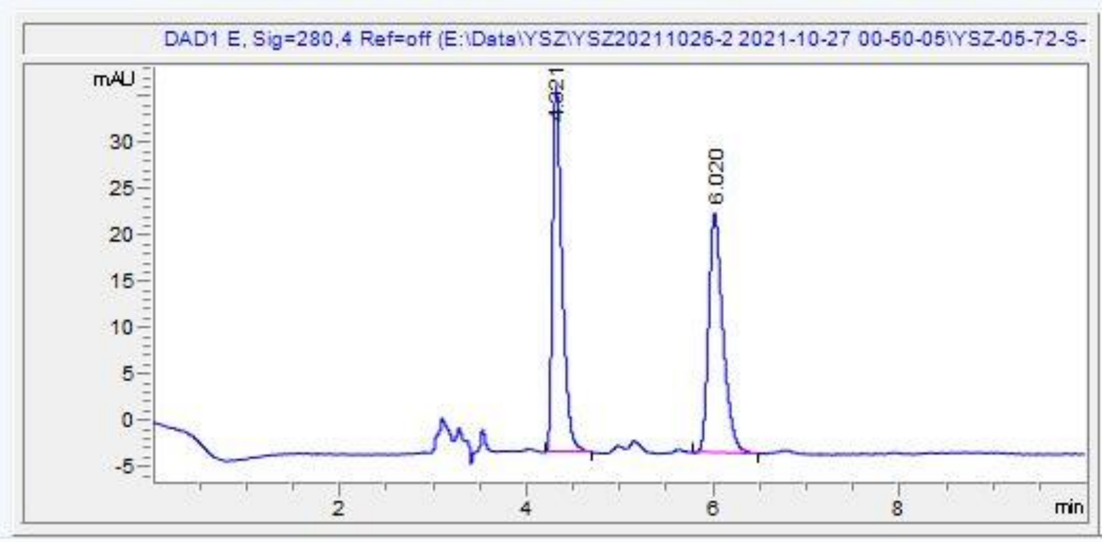

#	Time	Type	Area	Height	Width	Area%	Symmetry
1	4.321	BV R	282.5	39.5	0.1005	50.266	0.565
2	6.02	BV R	279.5	25.8	0.147	49.734	0.678

Supplementary Fig. 214 Full HPLC spectrum of racemic **1aa**

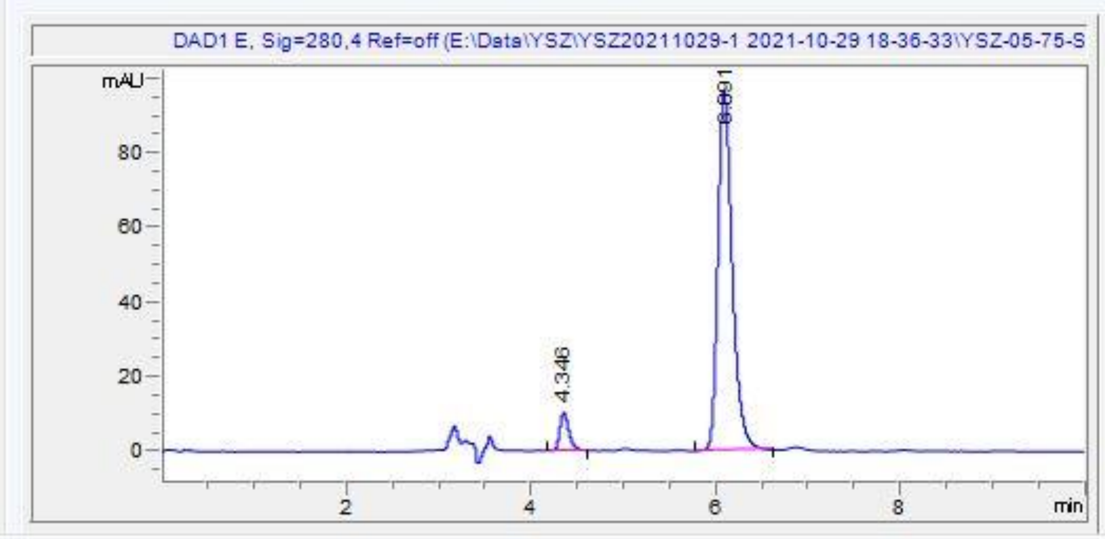

#	Time	Type	Area	Height	Width	Area%	Symmetry
1	4.346	BV R	72.7	10.4	0.0839	6.460	0.675
2	6.091	BB	1052.4	97.1	0.1545	93.540	0.726

Supplementary Fig. 215 Full HPLC spectrum of (S_p)-**1aa**

(R_p)-Dibenzyl 1-(4^2-bromo-1^5-((tert-butoxycarbonyl)amino)-1,4(1,4) -dibenzenacyclohexaphane-1^2-yl)hydrazine-1,2-dicarboxylate **(3aa)**

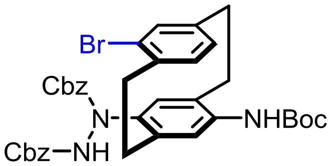

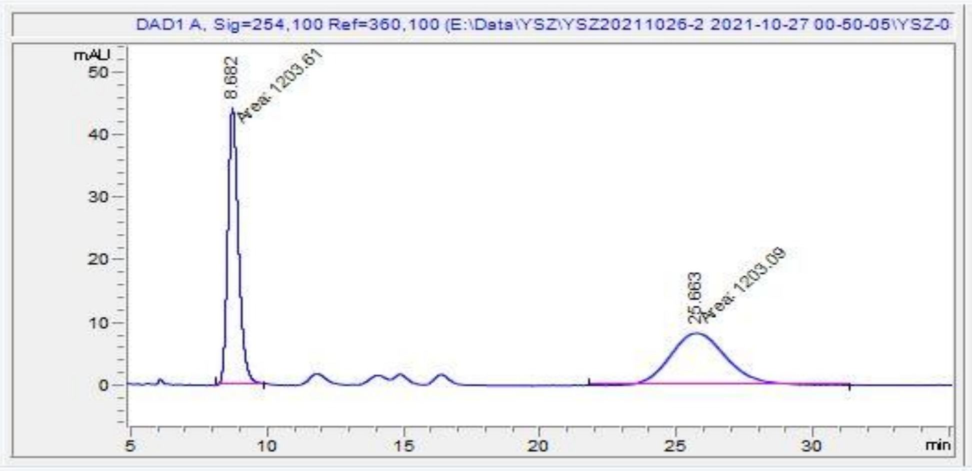

#	Time	Type	Area	Height	Width	Area%	Symmetry
1	8.682	MM	1203.6	44.4	0.4518	50.011	0.758
2	25.663	MM	1203.1	8.4	2.3898	49.989	0.873

Supplementary Fig. 216 HPLC spectrum of racemic **3aa**

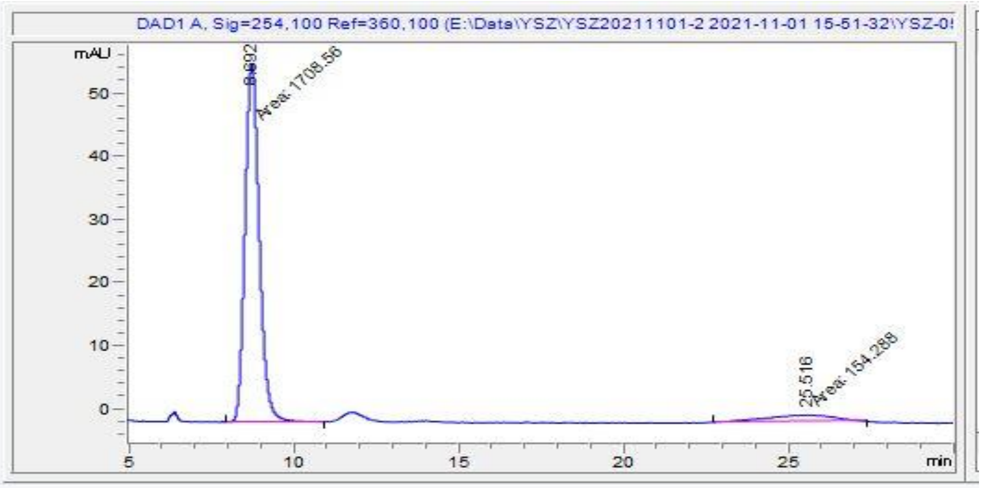

#	Time	Type	Area	Height	Width	Area%	Symmetry
1	8.692	MM	1708.6	56.8	0.501	91.718	0.853
2	25.516	MM	154.3	1.1	2.3744	8.282	1.552

Supplementary Fig. 217 HPLC spectrum of (R_p)-**3aa**

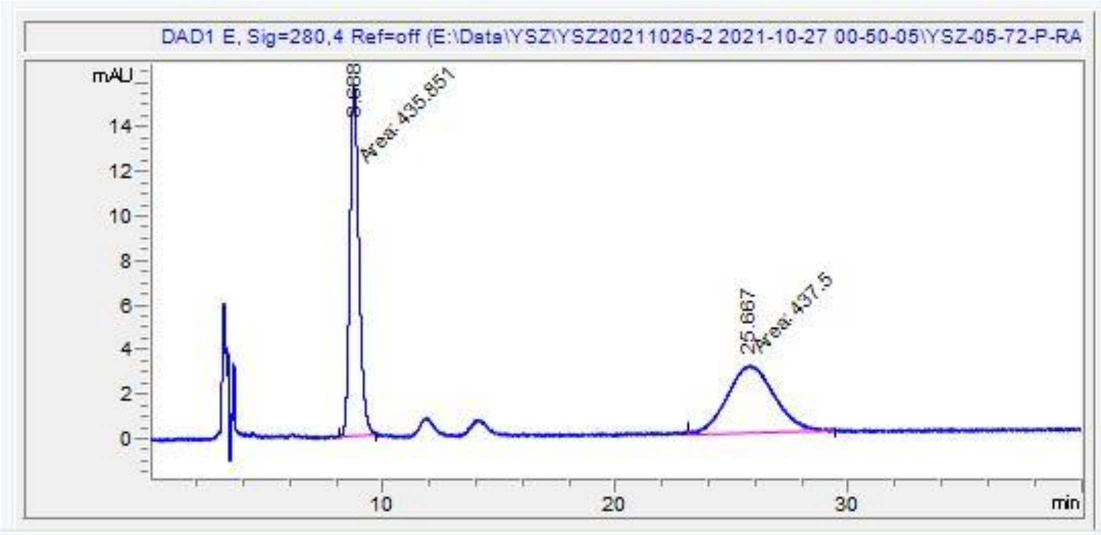

#	Time	Type	Area	Height	Width	Area%	Symmetry
1	8.688	MM	435.9	15.9	0.4572	49.906	0.813
2	25.667	MM	437.5	3.1	2.3589	50.094	0.891

Supplementary Fig. 218 HPLC spectrum of racemic **3aa**

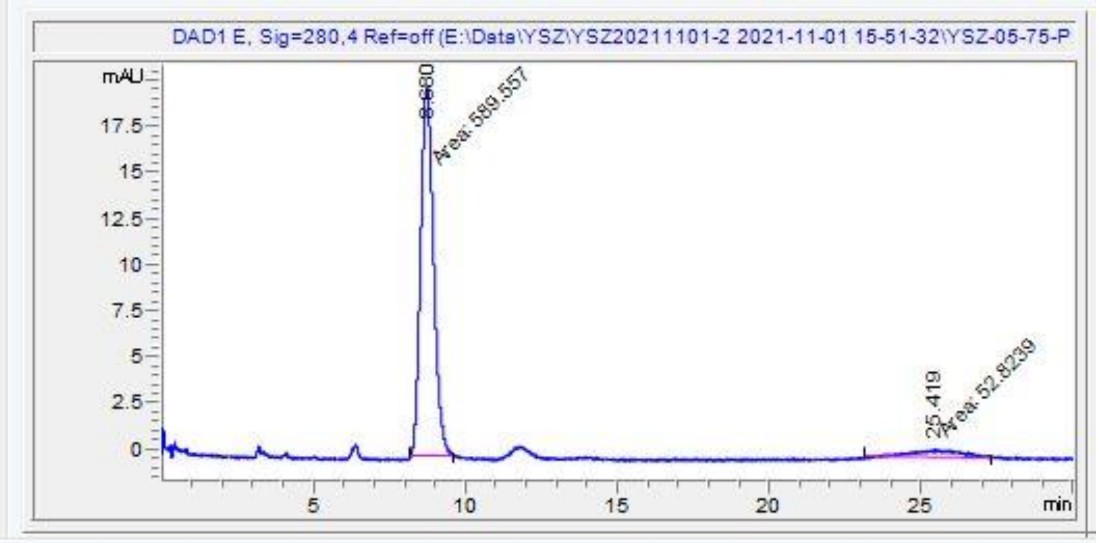

#	Time	Type	Area	Height	Width	Area%	Symmetry
1	8.68	MM	589.6	20.2	0.4876	91.777	0.79
2	25.419	MM	52.8	4.6E-1	1.8943	8.223	0.916

Supplementary Fig. 219 HPLC spectrum of (R_p)-**3aa**

(R_p)-Tert-butyl (4^3-phenyl-1,4(1,4)-dibenzenacyclohexaphane-1^2-yl)carbamate **(1ab)**

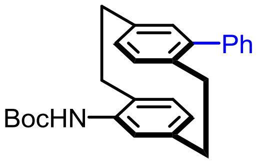

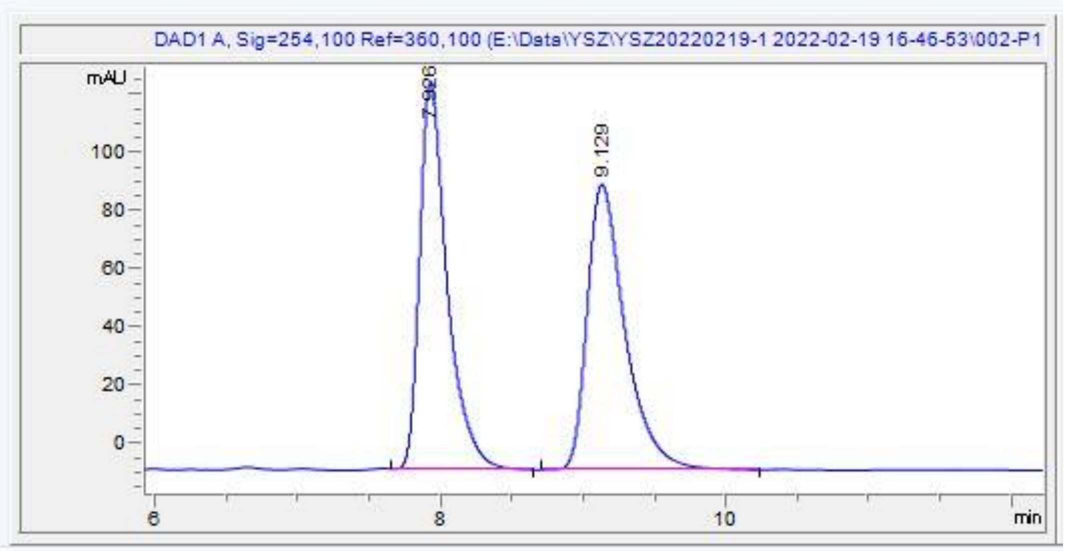

#	Time	Type	Area	Height	Width	Area%	Symmetry
1	7.926	BB	1776.9	132.5	0.2004	49.678	0.601
2	9.129	BB	1800	97.7	0.276	50.322	0.589

Supplementary Fig. 220 HPLC spectrum of racemic **1ab**

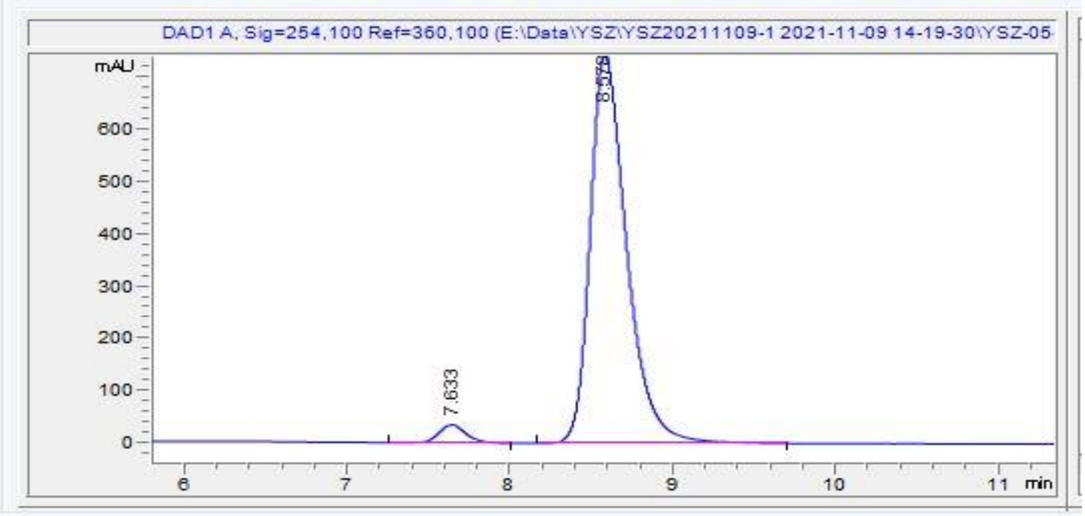

#	Time	Type	Area	Height	Width	Area%	Symmetry
1	7.633	VV R	399.2	34.5	0.1393	3.349	0.842
2	8.578	BV R	11518.4	738.3	0.187	96.651	0.684

Supplementary Fig. 221 HPLC spectrum of (R_p)**-1ab**

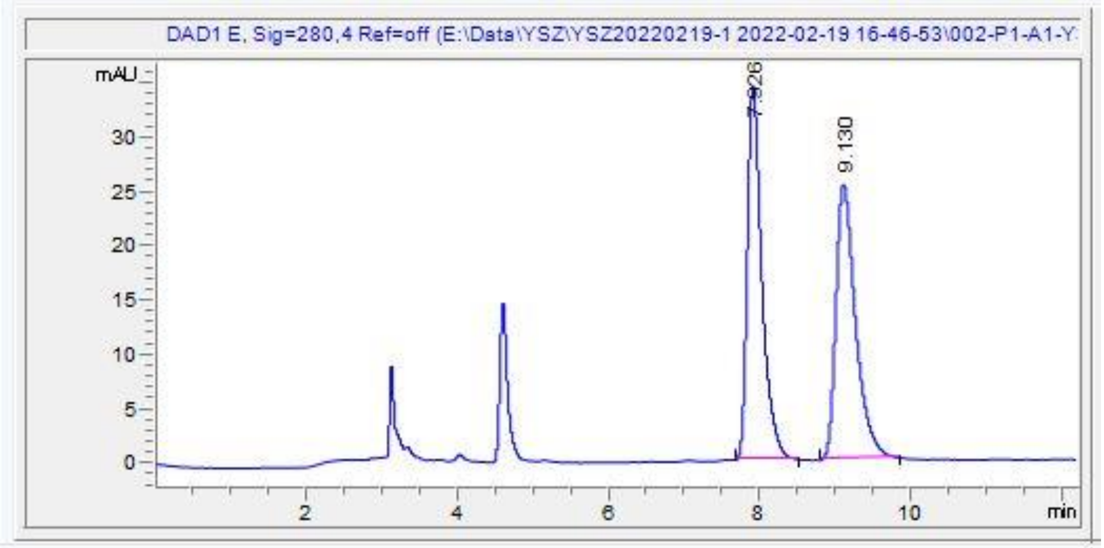

#	Time	Type	Area	Height	Width	Area%	Symmetry
1	7.926	BB	463.1	34.7	0.1934	49.958	0.607
2	9.13	BB	463.9	25.5	0.2225	50.042	0.609

Supplementary Fig. 222 Full HPLC spectrum of racemic **1ab**

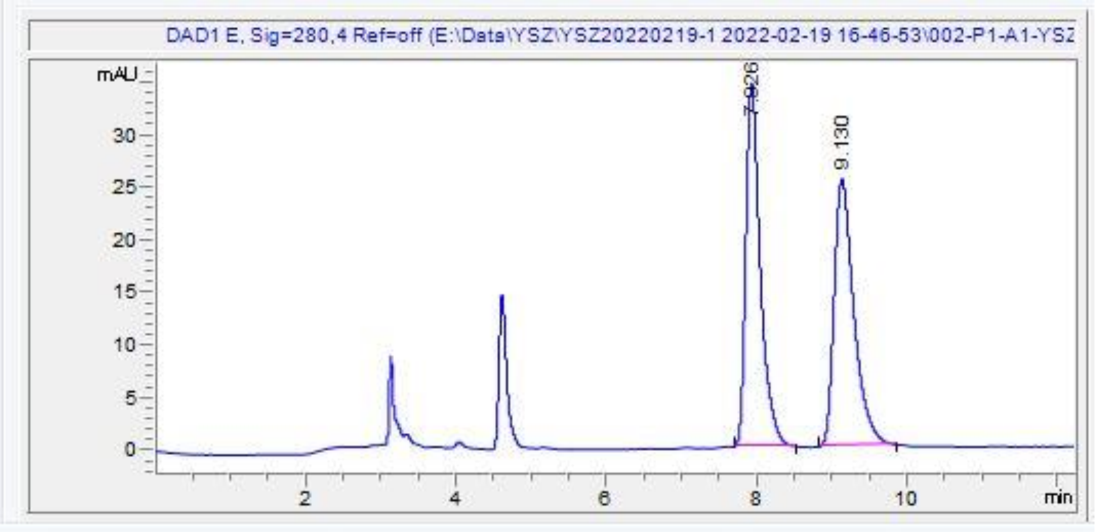

#	Time	Type	Area	Height	Width	Area%	Symmetry
1	7.926	BB	463.1	34.7	0.1934	49.958	0.607
2	9.13	BB	463.9	25.5	0.2225	50.042	0.609

Supplementary Fig. 223 Full HPLC spectrum of (R_p)-**1ab**

(S_p)-Dibenzyl 1-(1^5-((tert-butoxycarbonyl)amino)-4^2-phenyl-1,4(1,4)-dibenzenacyclohexaphane-1^2-yl)hydrazine-1,2-dicarboxylate **(3ab)**

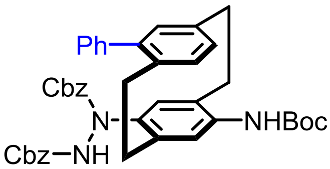

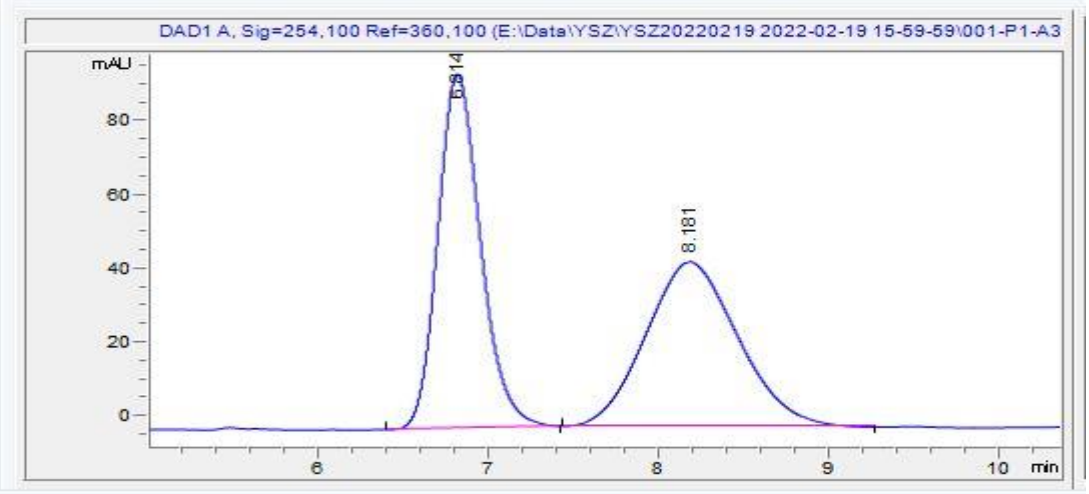

#	Time	Type	Area	Height	Width	Area%	Symmetry
1	6.814	BB	1685.7	96.7	0.2664	49.844	0.8
2	8.181	BB	1696.2	45	0.4537	50.156	0.908

Supplementary Fig. 224 HPLC spectrum of racemic **3ab**

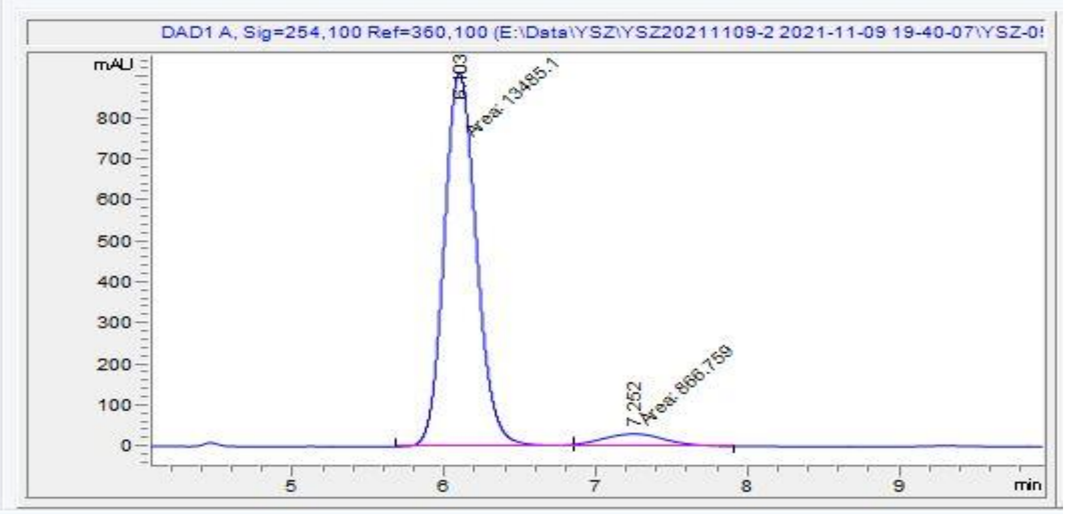

#	Time	Type	Area	Height	Width	Area%	Symmetry
1	6.103	MF	13485.1	906.7	0.2479	93.961	0
2	7.252	FM	866.8	29.7	0.4859	6.039	1.056

Supplementary Fig. 225 HPLC spectrum of (S_p)-**3ab**

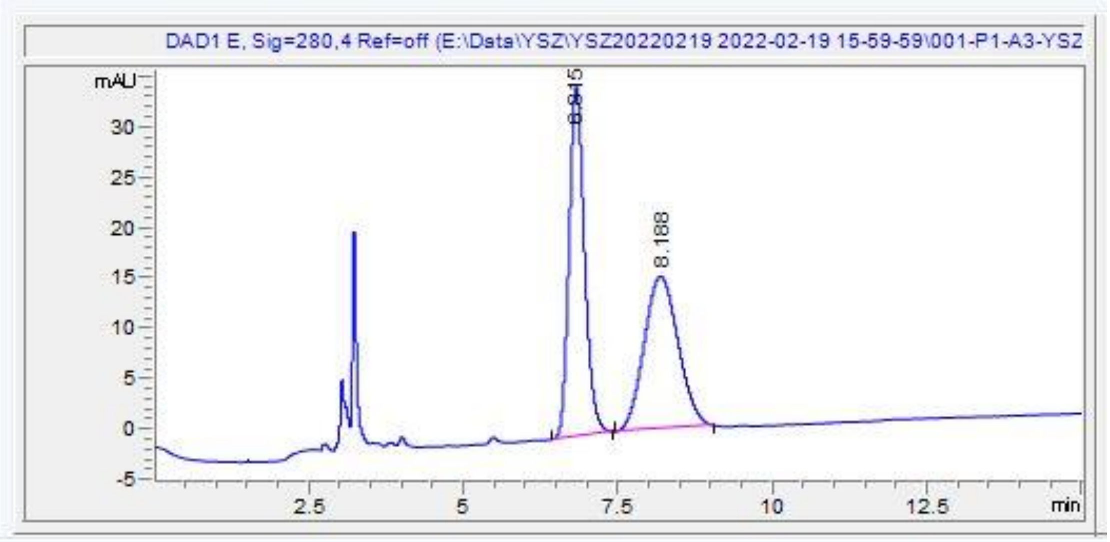

#	Time	Type	Area	Height	Width	Area%	Symmetry
1	6.815	BB	602.6	34.5	0.2446	50.764	0.811
2	8.188	BB	584.4	15.2	0.4517	49.236	0.949

Supplementary Fig. 226 Full HPLC spectrum of racemic **3ab**

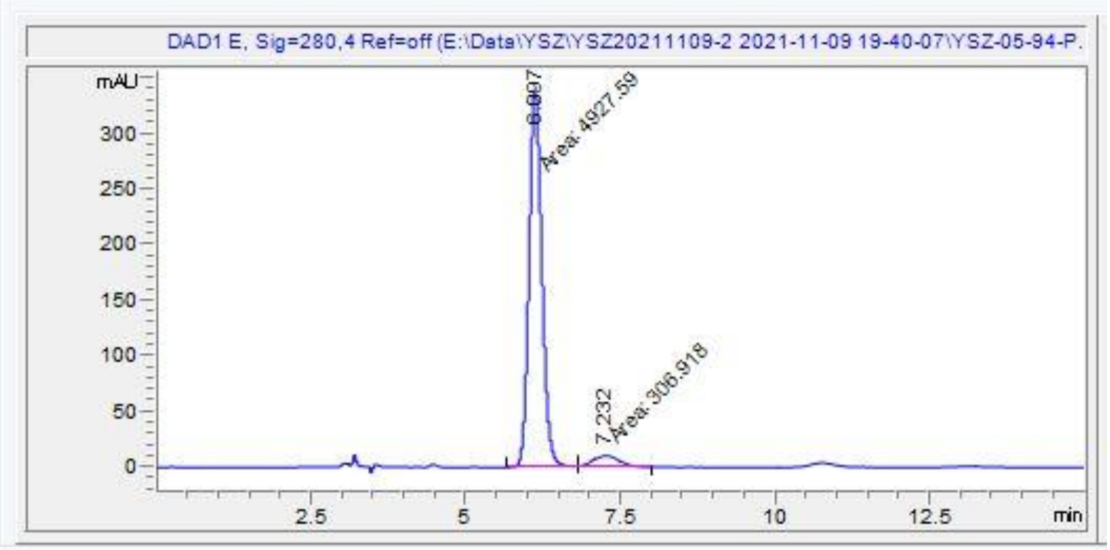

#	Time	Type	Area	Height	Width	Area%	Symmetry
1	6.097	MF	4927.6	339.4	0.2419	94.137	0
2	7.232	FM	306.9	10.4	0.4931	5.863	0.961

Supplementary Fig. 227 Full HPLC spectrum of (S_p)-**3ab**

(R_p)-Tert-butyl (E)-(4[3]-styryl-1,4(1,4)-dibenzenacyclohexaphane-1[2]-yl)carbamate **(1ac)**

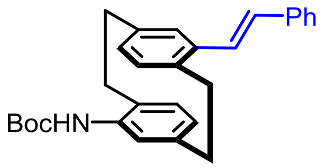

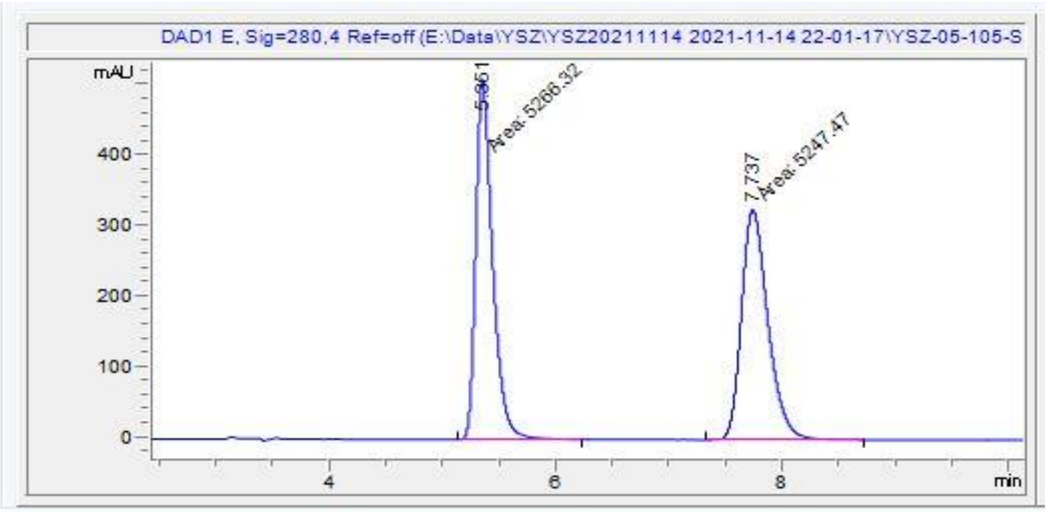

#	Time	Type	Area	Height	Width	Area%	Symmetry
1	5.351	MM	5266.3	508.8	0.1725	50.090	0.636
2	7.737	MM	5247.5	324.5	0.2695	49.910	0.707

Supplementary Fig. 228 HPLC spectrum of racemic **1ac**

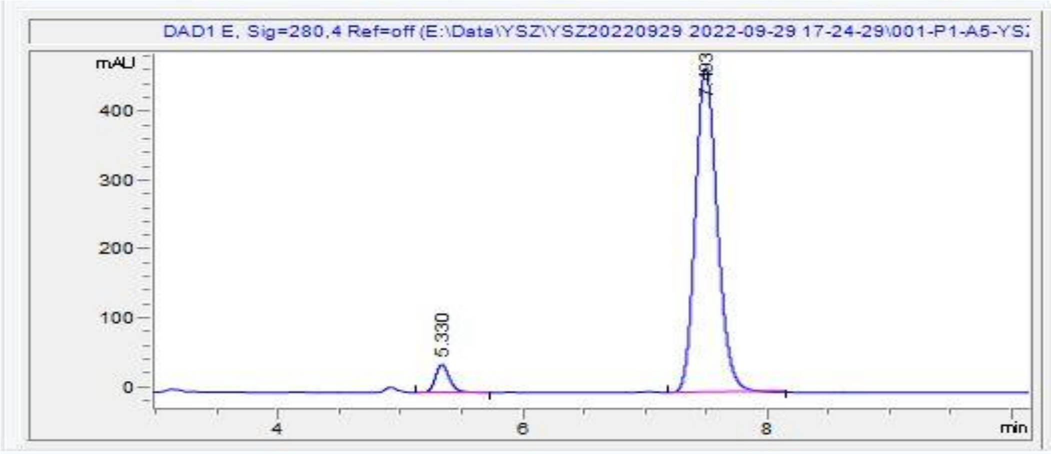

#	Time	Type	Area	Height	Width	Area%	Symmetry
1	5.33	BB	329.6	40.9	0.1169	5.297	0.777
2	7.493	BB	5892.6	470.2	0.1927	94.703	0.807

Supplementary Fig. 229 HPLC spectrum of (R_p)-**1ac**

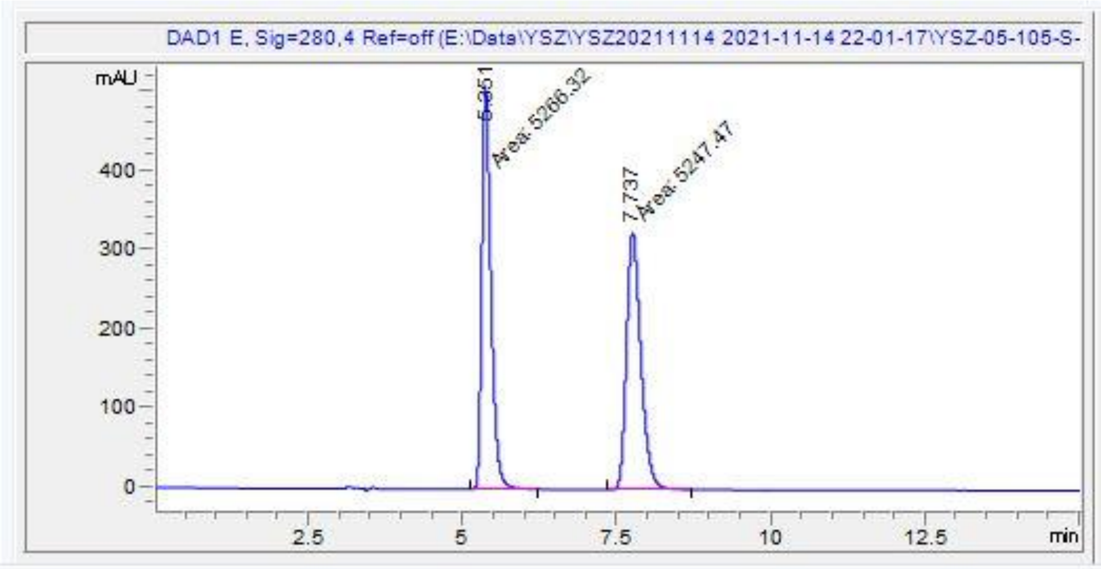

#	Time	Type	Area	Height	Width	Area%	Symmetry
1	5.351	MM	5266.3	508.8	0.1725	50.090	0.636
2	7.737	MM	5247.5	324.5	0.2695	49.910	0.707

Supplementary Fig. 230 Full HPLC spectrum of racemic **1ac**

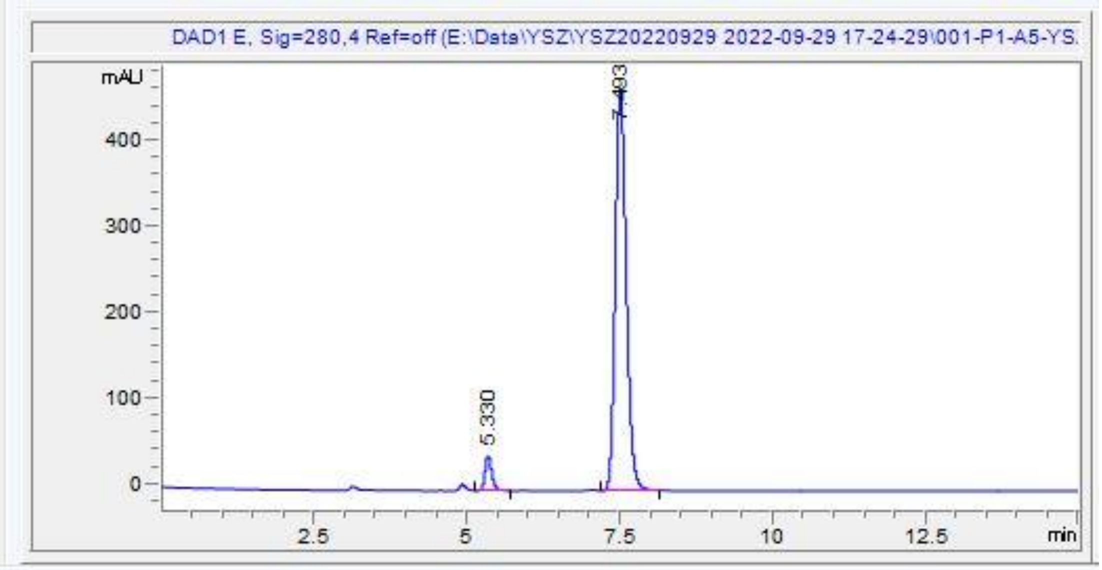

#	Time	Type	Area	Height	Width	Area%	Symmetry
1	5.33	BB	329.6	40.9	0.1169	5.297	0.777
2	7.493	BB	5892.6	470.2	0.1927	94.703	0.807

Supplementary Fig. 231 Full HPLC spectrum of (R_p)-**1ac**

(S_p)-Dibenzyl (E)-1-(1^5-((tert-butoxycarbonyl)amino)-4^2-styryl-1,4(1,4)-dibenzenacyclohexaphane-1^2-yl)hydrazine-1,2-dicarboxylate **(3ac)**

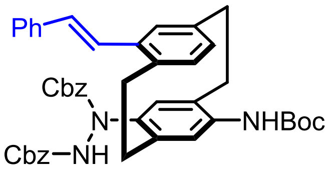

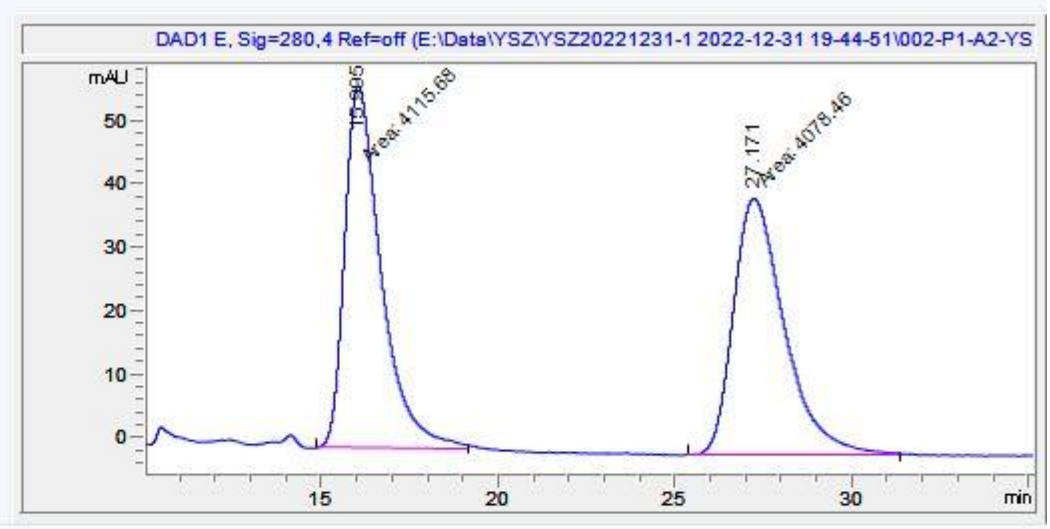

#	Time	Type	Area	Height	Width	Area%	Symmetry
1	15.995	MM	4115.7	57.3	1.1975	50.227	0.55
2	27.171	MM	4078.5	40.7	1.6717	49.773	0.65

Supplementary Fig. 232 HPLC spectrum of racemic **3ac**

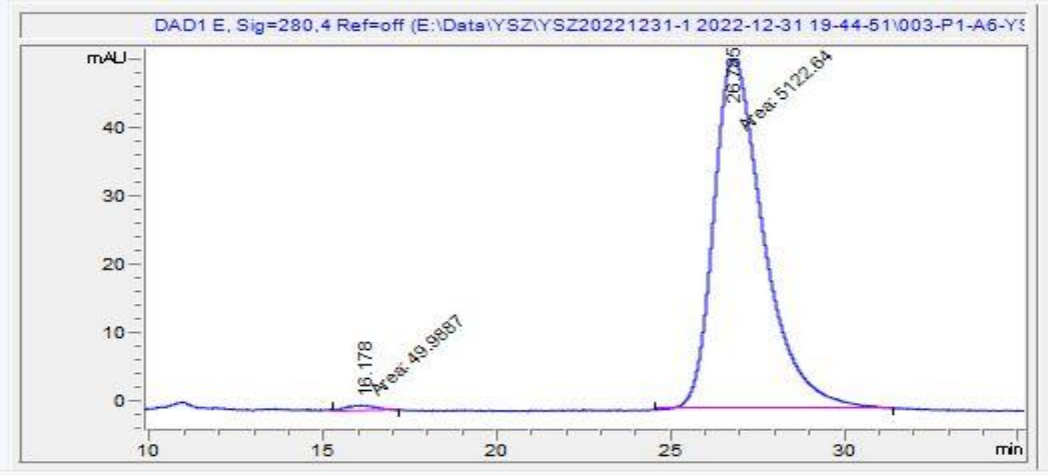

#	Time	Type	Area	Height	Width	Area%	Symmetry
1	16.178	MM	50	8.1E-1	1.0317	0.966	1.539
2	26.785	MM	5122.6	51	1.6755	99.034	0.639

Supplementary Fig. 233 HPLC spectrum of (S_p)-**3ac**

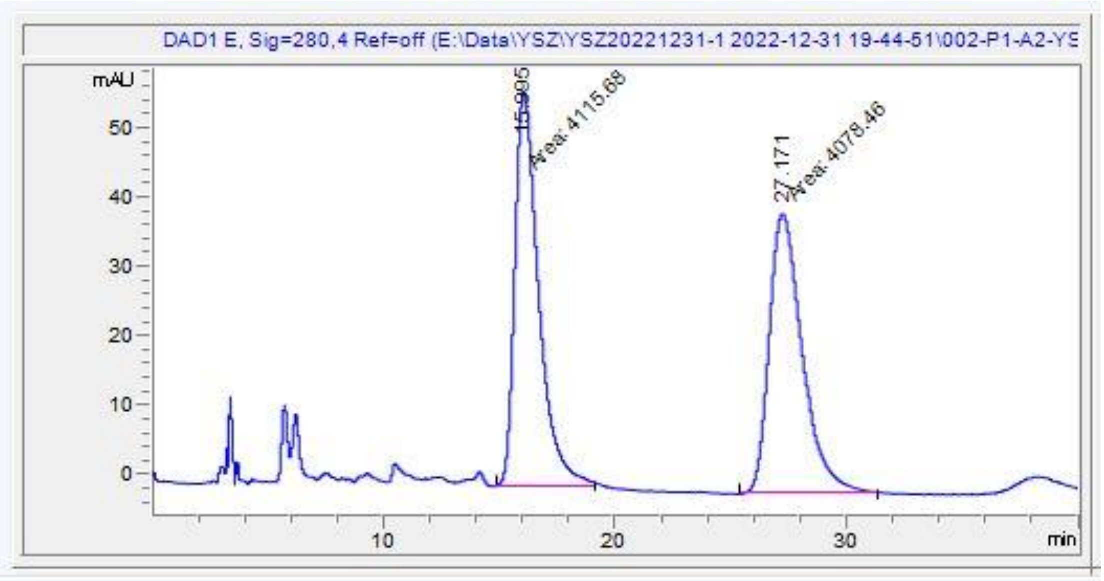

#	Time	Type	Area	Height	Width	Area%	Symmetry
1	15.995	MM	4115.7	57.3	1.1975	50.227	0.55
2	27.171	MM	4078.5	40.7	1.6717	49.773	0.65

Supplementary Fig. 234 HPLC spectrum of racemic **3ac**

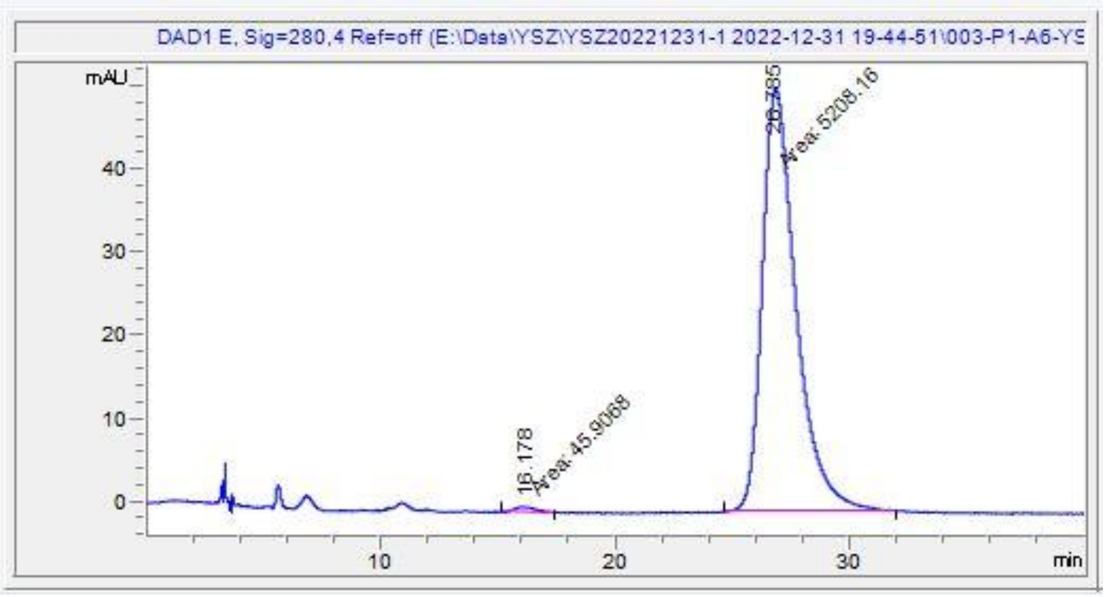

#	Time	Type	Area	Height	Width	Area%	Symmetry
1	16.178	MM	45.9	7.5E-1	1.0184	0.874	1.18
2	26.785	MM	5208.2	51.1	1.6973	99.126	0.635

Supplementary Fig. 235 HPLC spectrum of (S_p)-**3ac**

(R_p)-Tert-butyl (4[3]-cyclohexyl-1,4(1,4)-dibenzenacyclohexaphane-1[2]-yl)carbamate **(1ad)**

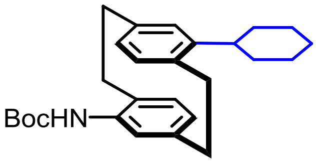

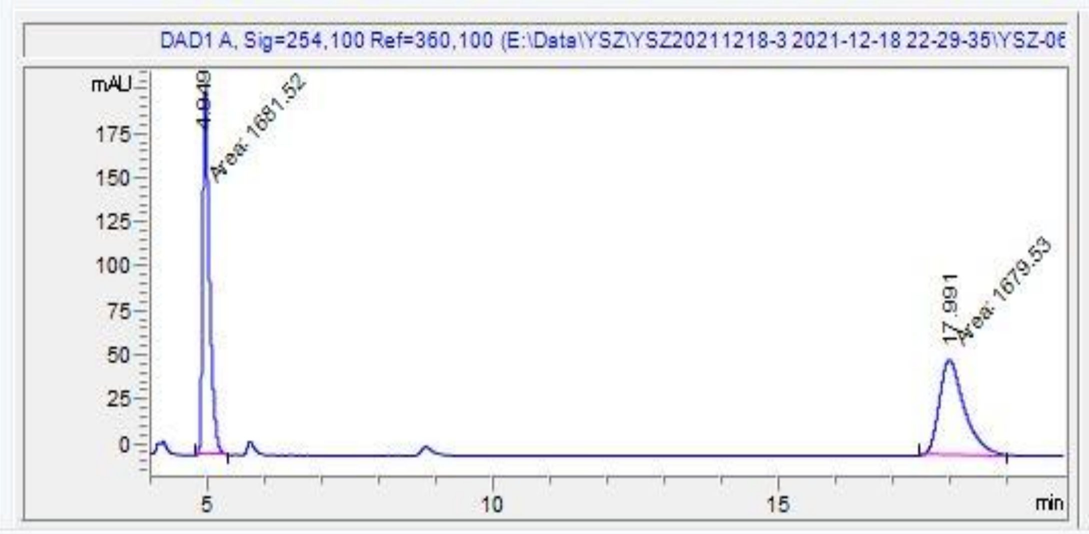

#	Time	Type	Area	Height	Width	Area%	Symmetry
1	4.949	MM	1681.5	205.9	0.1361	50.030	0.568
2	17.991	MM	1679.5	54.2	0.5164	49.970	0.65

Supplementary Fig. 236 HPLC spectrum of racemic **1ad**

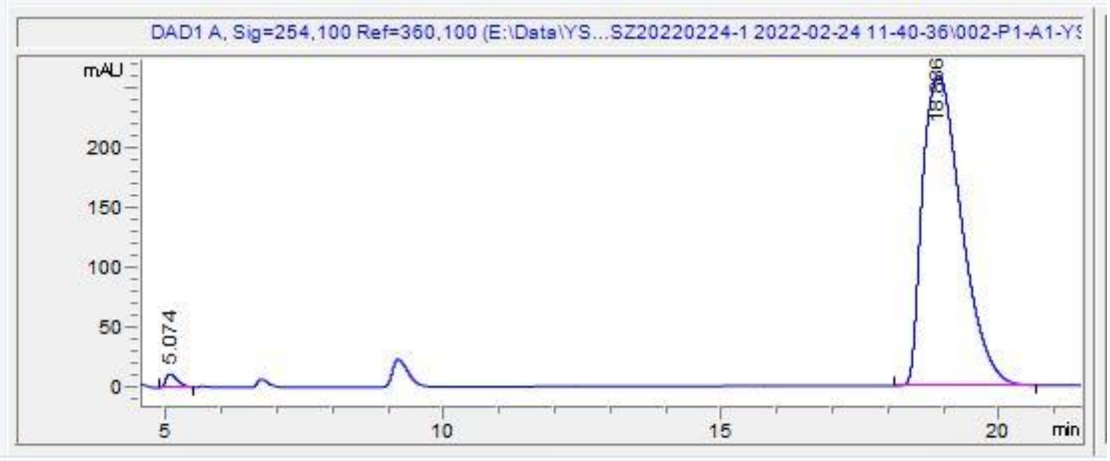

#	Time	Type	Area	Height	Width	Area%	Symmetry
1	5.074	BB	164.5	11	0.1773	1.290	0.723
2	18.886	BB	12590.2	258.9	0.5773	98.710	0.695

Supplementary Fig. 237 HPLC spectrum of (R_p)-**1ad**

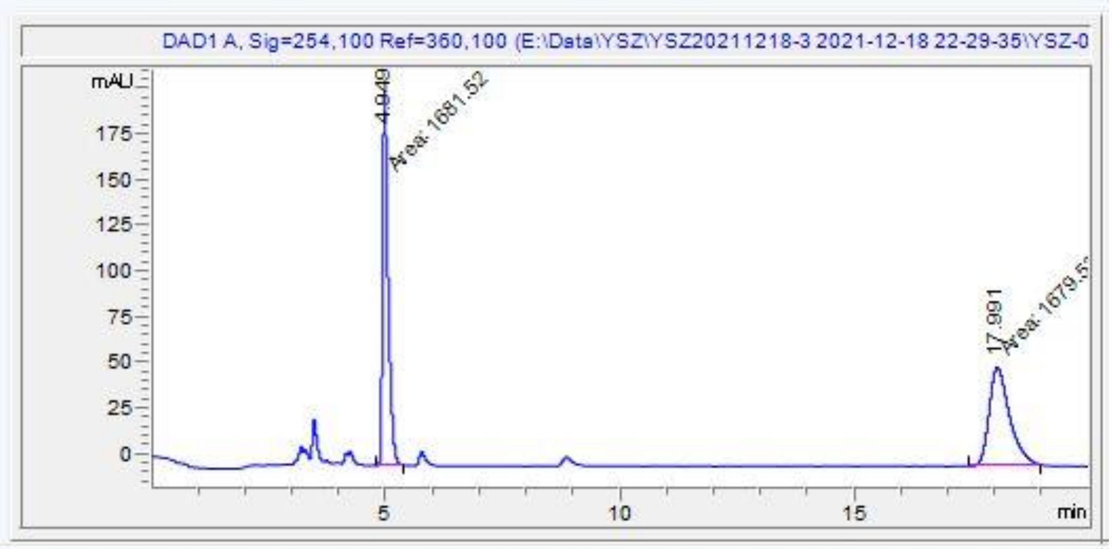

#	Time	Type	Area	Height	Width	Area%	Symmetry
1	4.949	MM	1681.5	205.9	0.1361	50.030	0.568
2	17.991	MM	1679.5	54.2	0.5164	49.970	0.65

Supplementary Fig. 238 Full HPLC spectrum of racemic **1ad**

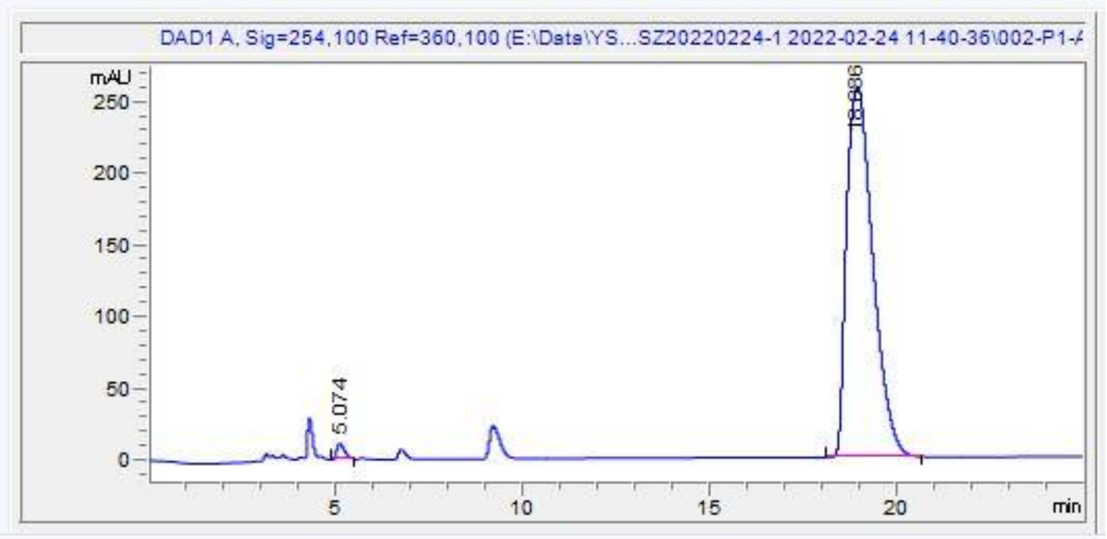

#	Time	Type	Area	Height	Width	Area%	Symmetry
1	5.074	BB	164.5	11	0.1773	1.290	0.723
2	18.886	BB	12590.2	258.9	0.5773	98.710	0.695

Supplementary Fig. 239 Full HPLC spectrum of (R_p)-**1ad**

(S_p)-Dibenzyl 1-(1^5-((tert-butoxycarbonyl)amino)-4^2-cyclohexyl-1,4(1,4)-dibenzenacyclohexaphane-1^2-yl)hydrazine-1,2-dicarboxylate **(3ad)**

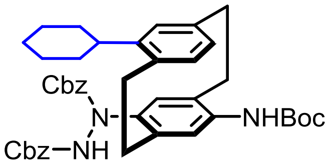

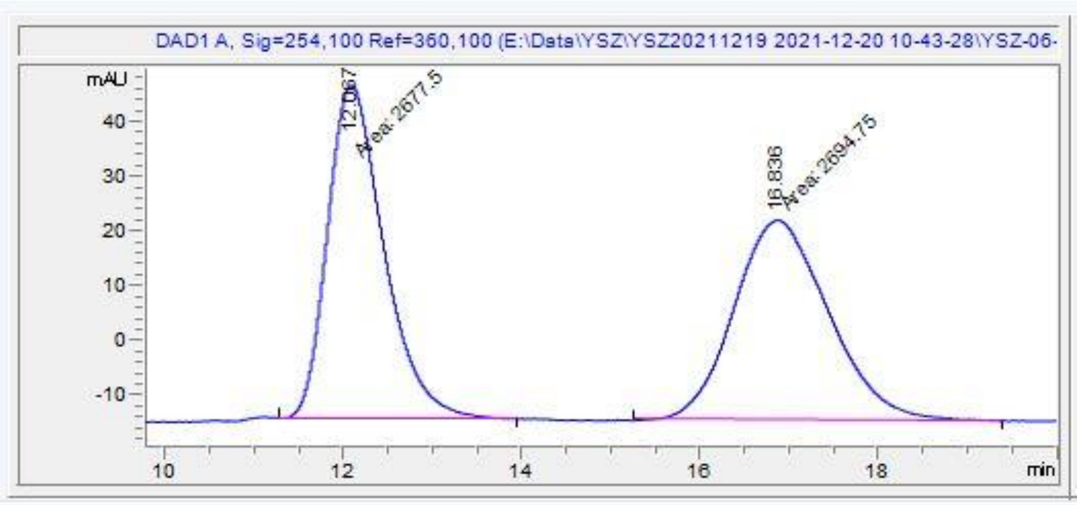

#	Time	Type	Area	Height	Width	Area%	Symmetry
1	12.067	MM	2677.5	60.9	0.7334	49.839	0.682
2	16.836	MM	2694.8	36.5	1.2304	50.161	0.821

Supplementary Fig. 240 HPLC spectrum of racemic **3ad**

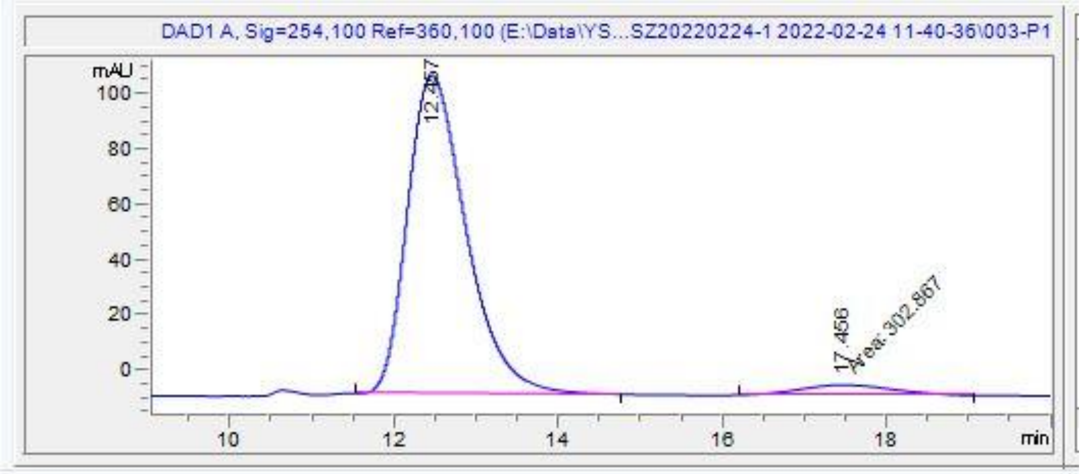

#	Time	Type	Area	Height	Width	Area%	Symmetry
1	12.457	BB	5726	114.9	0.6335	94.976	0.677
2	17.456	MM	302.9	3.8	1.3398	5.024	0.78

Supplementary Fig. 241 HPLC spectrum of (S_p)-**3ad**

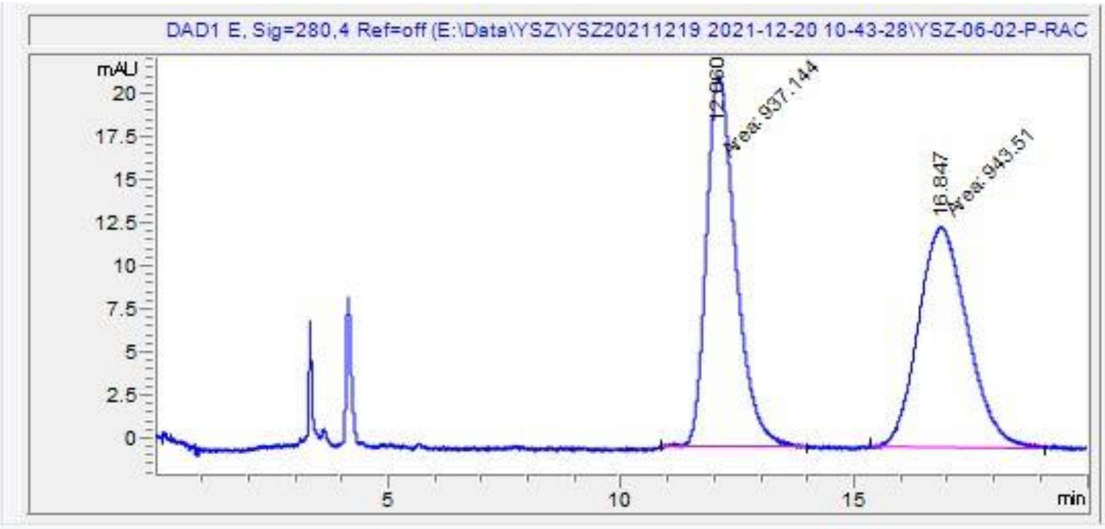

#	Time	Type	Area	Height	Width	Area%	Symmetry
1	12.06	MM	937.1	21.4	0.729	49.831	0.662
2	16.847	MM	943.5	12.8	1.2288	50.169	0.847

Supplementary Fig. 242 Full HPLC spectrum of racemic **3ad**

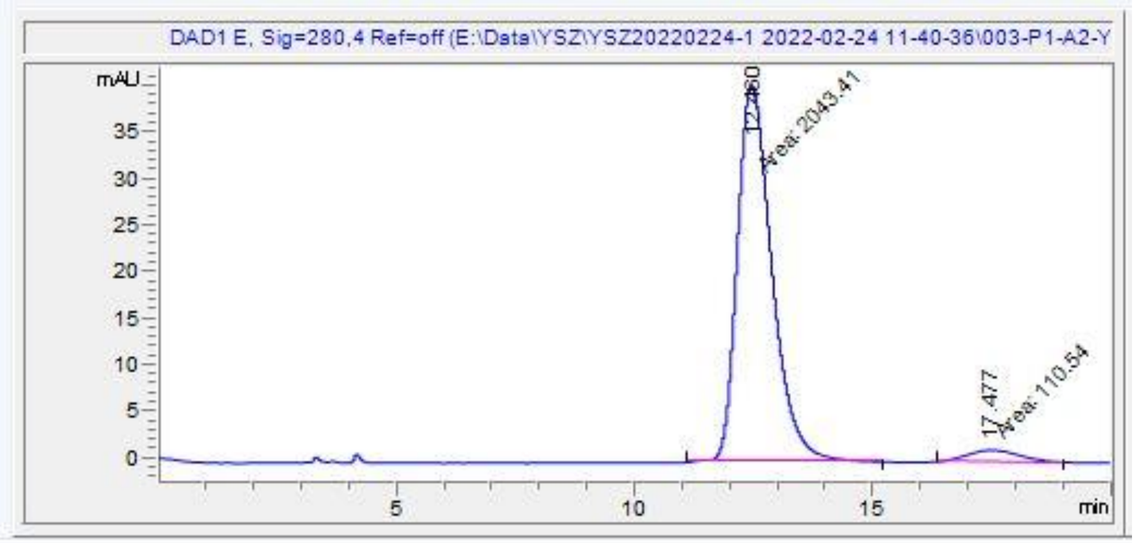

#	Time	Type	Area	Height	Width	Area%	Symmetry
1	12.46	MM	2043.4	40.4	0.8437	94.868	0.692
2	17.477	MM	110.5	1.4	1.3531	5.132	0.835

Supplementary Fig. 243 Full HPLC spectrum of (S_p)-**3ad**

(S_p)-Dibenzyl-1-(1^5,4^2-bis((tert-butoxycarbonyl)amino)-1,4(1,4)-dibenzenacyclohexaphane-1^2-yl)hydrazine-1,2-dicarboxylate **(5a)**

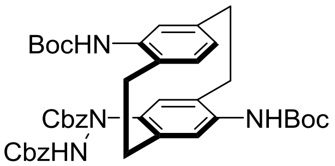

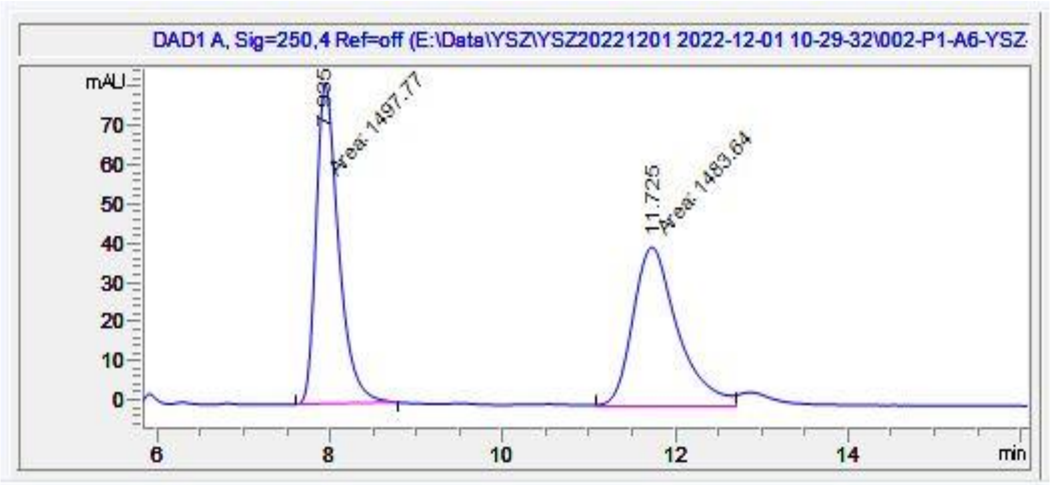

#	Time	Type	Area	Height	Width	Area%	Symmetry
1	7.935	MM	1497.8	81.4	0.3067	50.237	0.648
2	11.725	MF	1483.6	40.6	0.6092	49.763	0

Supplementary Fig. 244 HPLC spectrum of racemic **5a**

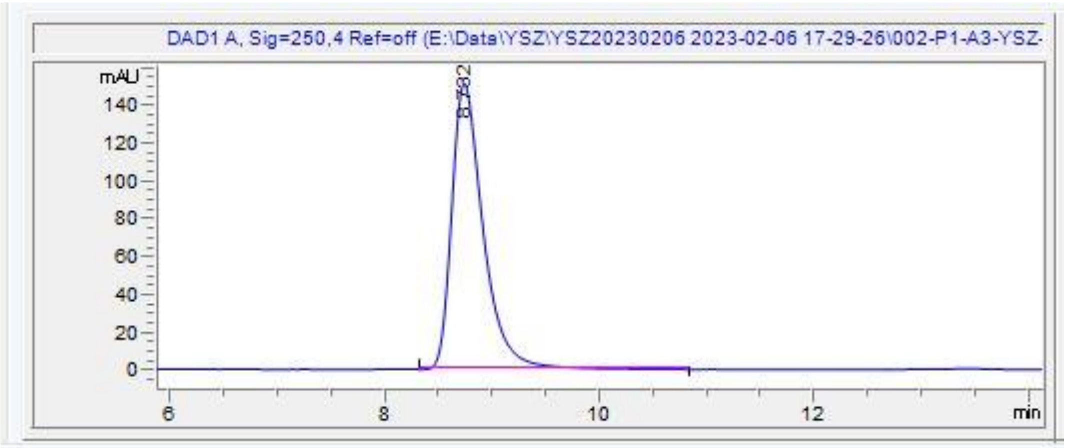

#	Time	Type	Area	Height	Width	Area%	Symmetry
1	8.732	BB	3124.5	152.5	0.309	100.000	0.595

Supplementary Fig. 245 HPLC spectrum of (S_p)-**5a**

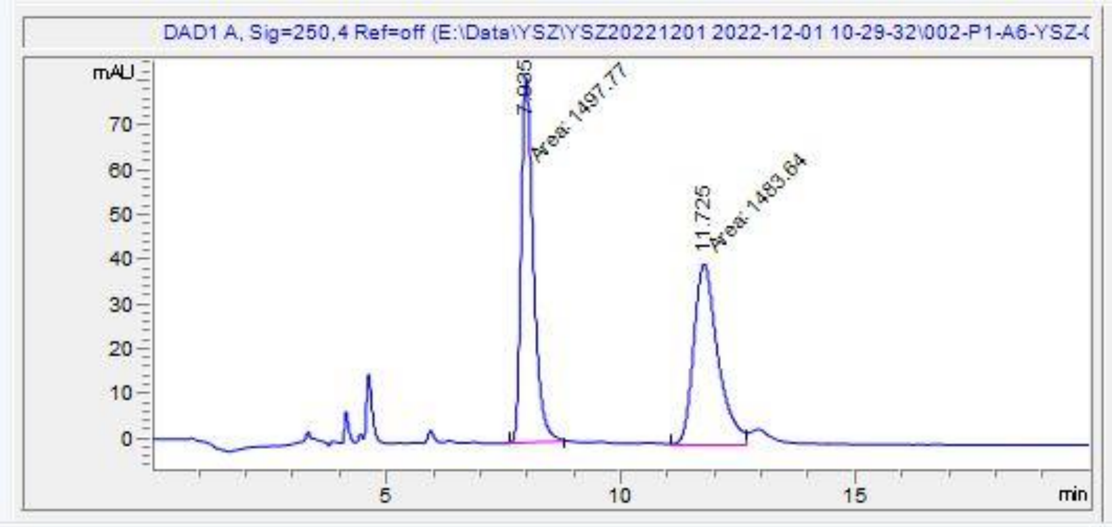

#	Time	Type	Area	Height	Width	Area%	Symmetry
1	7.935	MM	1497.8	81.4	0.3067	50.237	0.648
2	11.725	MF	1483.6	40.6	0.6092	49.763	0

Supplementary Fig. 246 Full HPLC spectrum of racemic **5a**

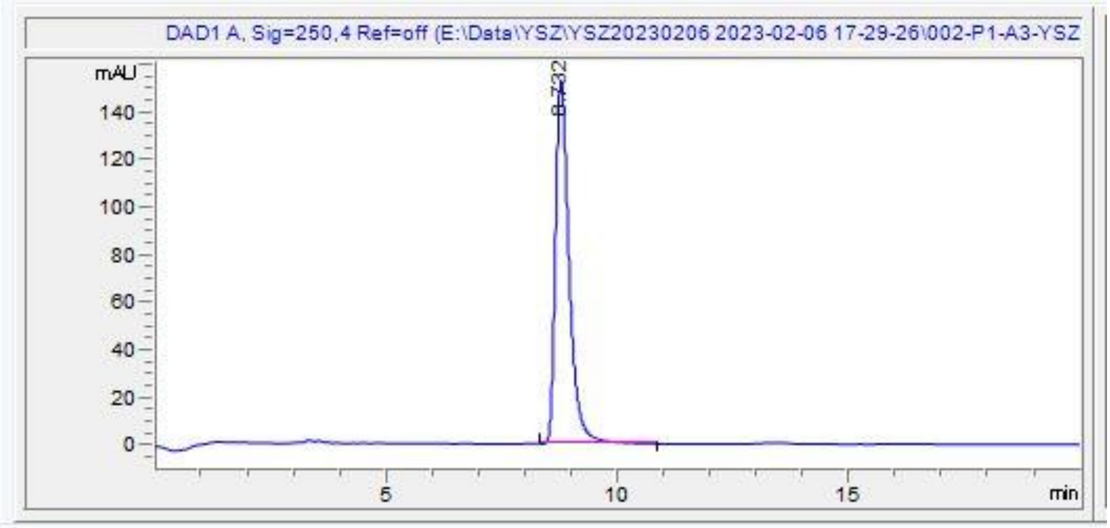

#	Time	Type	Area	Height	Width	Area%	Symmetry
1	8.732	BB	3124.5	152.5	0.309	100.000	0.595

Supplementary Fig. 247 Full HPLC spectrum of (S_p)-**5a**

(R_p)-*N*-(1,4(1,4)-dibenzenacyclohexaphane-1^2-yl)acetamide **(6c)**

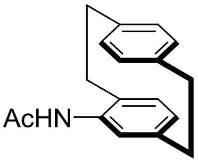

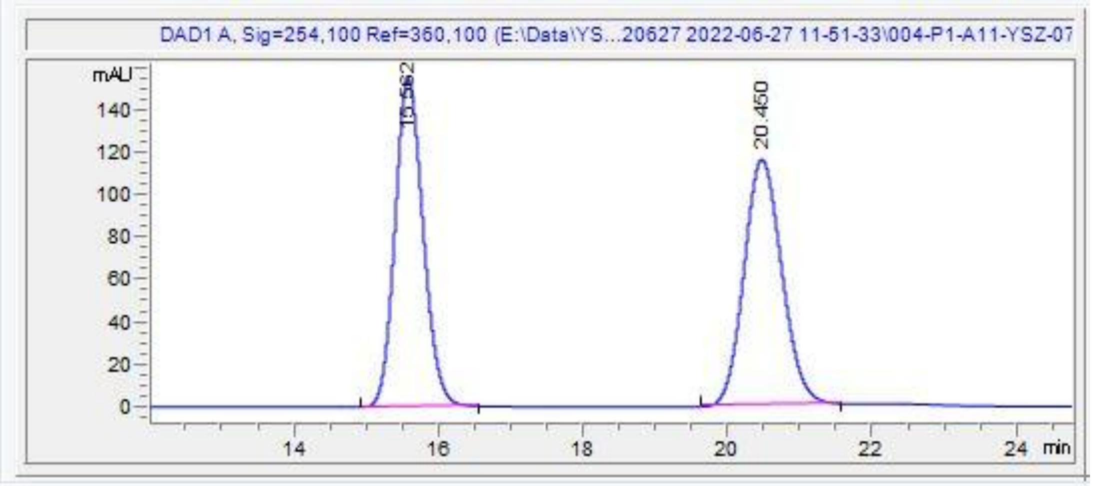

#	Time	Type	Area	Height	Width	Area%	Symmetry
1	15.562	BB	4195.2	154.6	0.3526	49.818	0.864
2	20.45	BB	4225.8	116.1	0.4346	50.182	0.891

Supplementary Fig. 248 HPLC spectrum of racemic **6c**

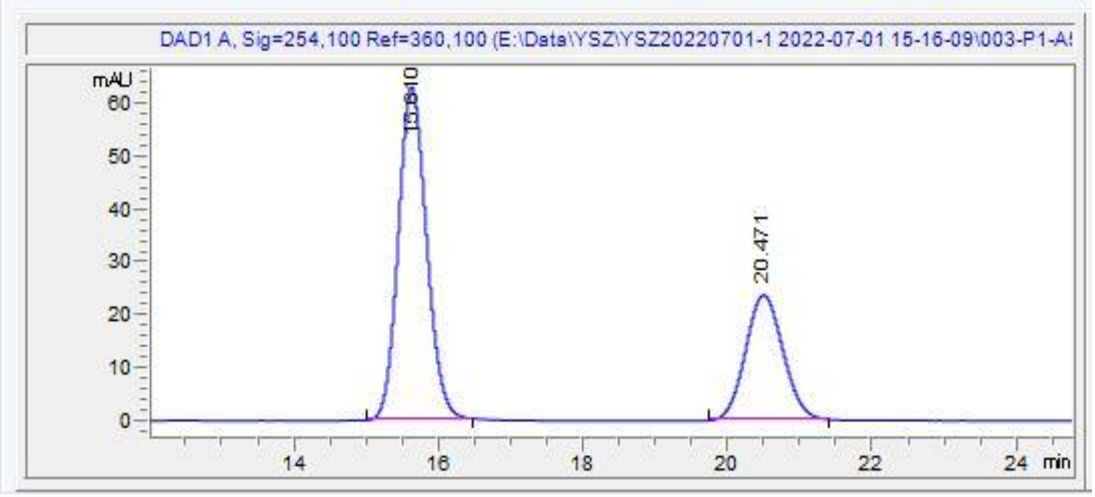

#	Time	Type	Area	Height	Width	Area%	Symmetry
1	15.61	BB	1719	63.3	0.319	66.696	0.877
2	20.471	BB	858.4	23.8	0.4222	33.304	0.912

Supplementary Fig. 249 HPLC spectrum of (R_p)-**6c**

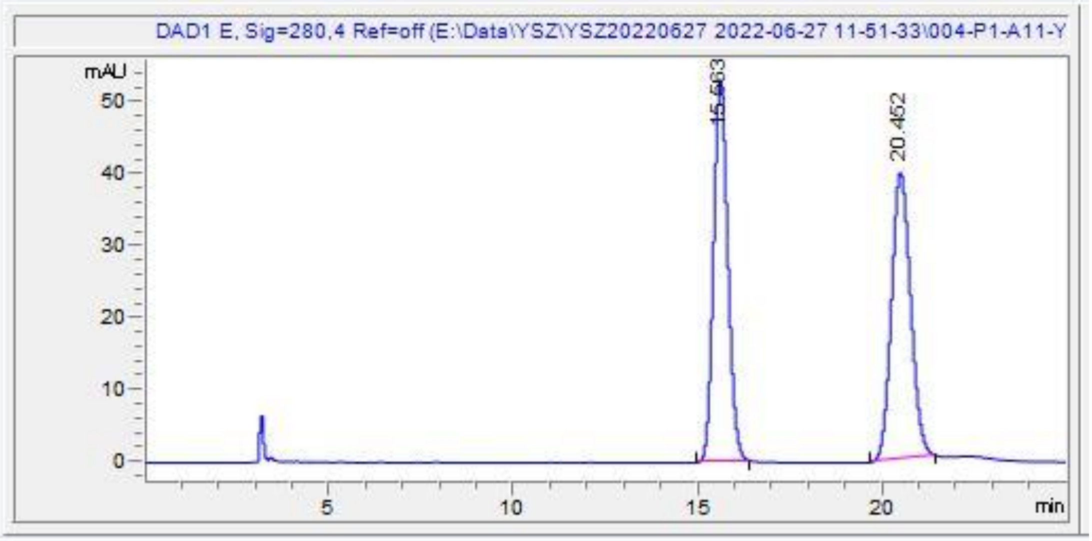

#	Time	Type	Area	Height	Width	Area%	Symmetry
1	15.563	BB	1434.5	53.1	0.3271	49.715	0.856
2	20.452	BB	1450.9	39.9	0.4253	50.285	0.898

Supplementary Fig. 250 Full HPLC spectrum of racemic **6c**

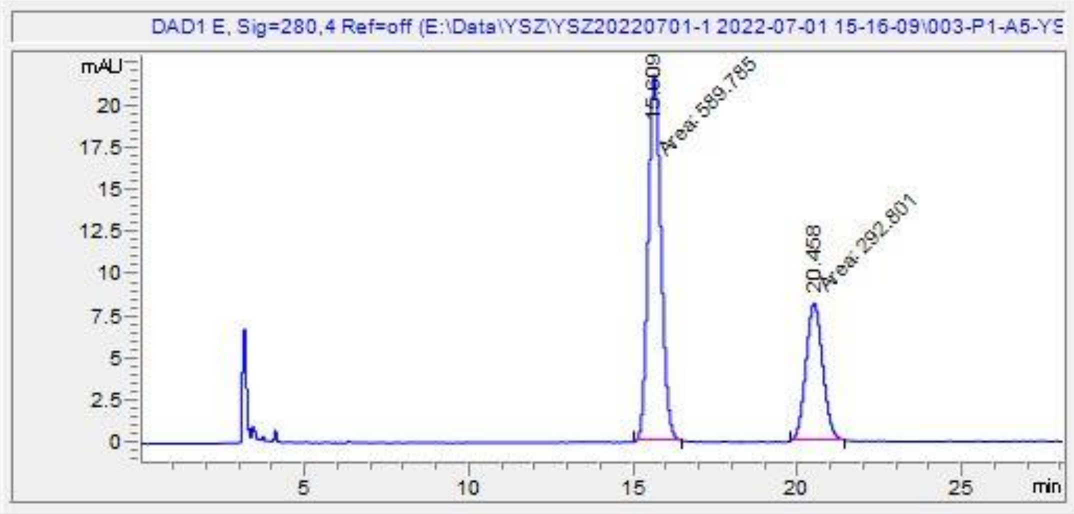

#	Time	Type	Area	Height	Width	Area%	Symmetry
1	15.609	MM	589.8	21.7	0.4522	66.825	0.859
2	20.458	MM	292.8	8.1	0.599	33.175	0.829

Supplementary Fig. 251 Full HPLC spectrum of (R_p)-**6c**

(S_p)-Dibenzyl-1-(1^5-acetamido-1,4(1,4)-dibenzenacyclohexaphane-1^2-yl)hydrazine-1, 2-dicarboxylate **(7c)**

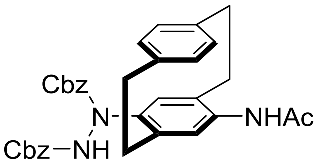

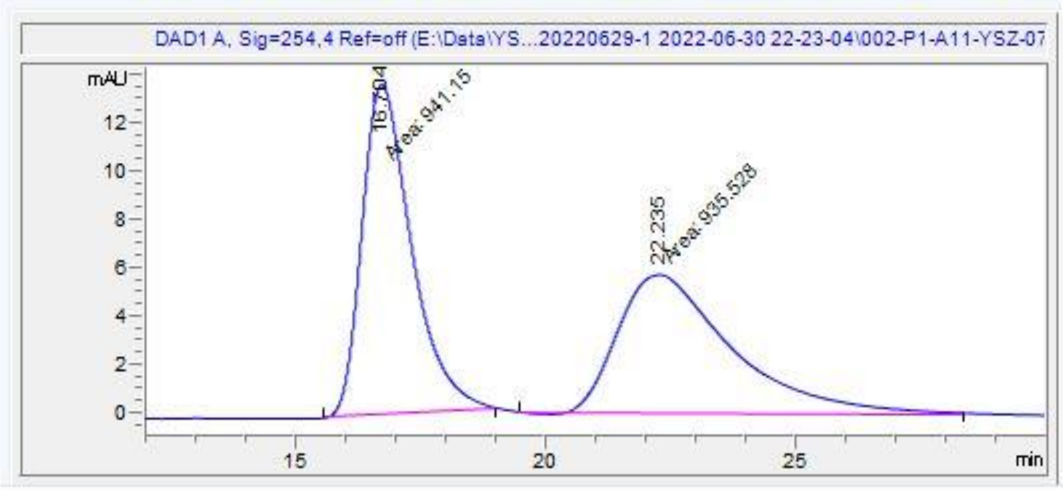

#	Time	Type	Area	Height	Width	Area%	Symmetry
1	16.704	MM	941.2	13.7	1.1489	50.150	0.644
2	22.235	MM	935.5	5.8	2.695	49.850	0.546

Supplementary Fig. 252 HPLC spectrum of racemic **7c**

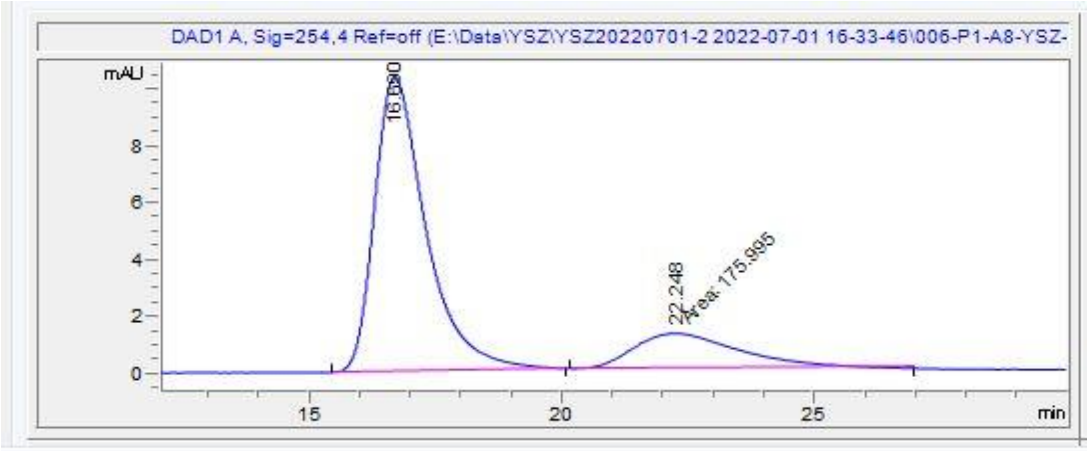

#	Time	Type	Area	Height	Width	Area%	Symmetry
1	16.69	BB	729	10.3	0.9951	80.554	0.617
2	22.248	MM	176	1.2	2.4151	19.446	0.63

Supplementary Fig. 253 HPLC spectrum of (S_p)-**7c**

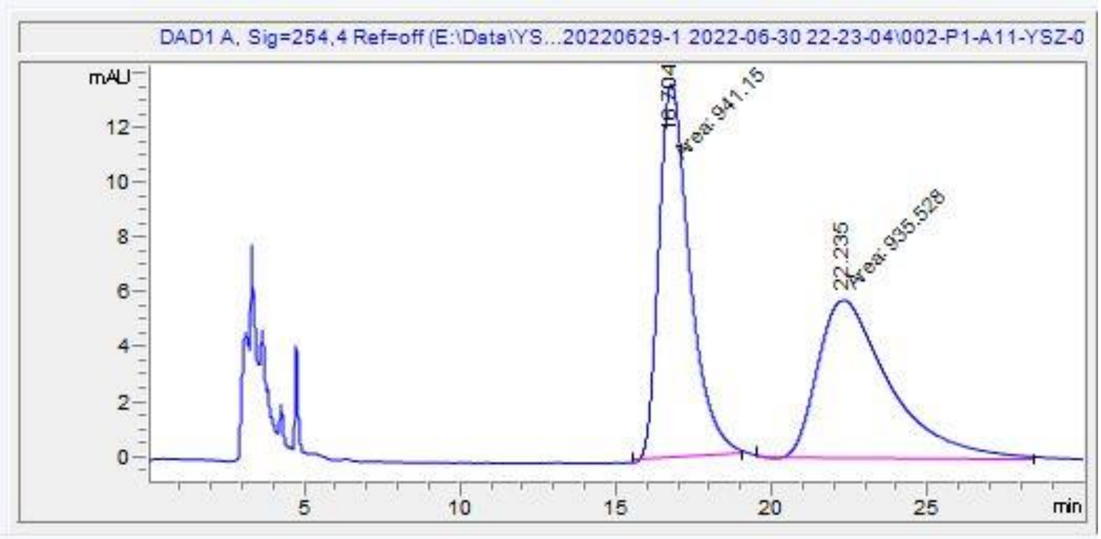

#	Time	Type	Area	Height	Width	Area%	Symmetry
1	16.704	MM	941.2	13.7	1.1489	50.150	0.644
2	22.235	MM	935.5	5.8	2.695	49.850	0.546

Supplementary Fig. 254 Full HPLC spectrum of racemic **7c**

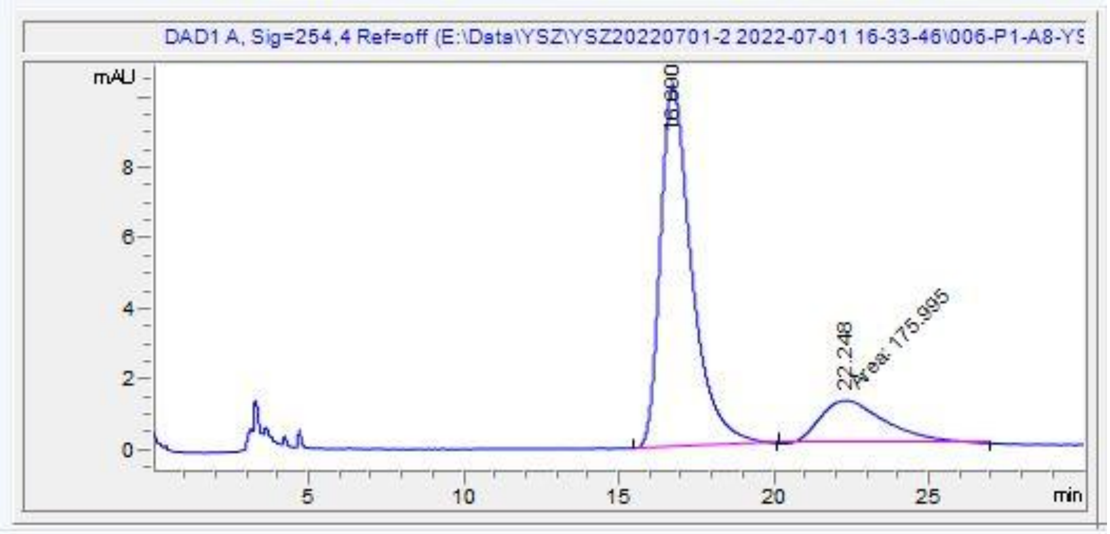

#	Time	Type	Area	Height	Width	Area%	Symmetry
1	16.69	BB	729	10.3	0.9951	80.554	0.617
2	22.248	MM	176	1.2	2.4151	19.446	0.63

Supplementary Fig. 255 Full HPLC spectrum of (S_p)-**7c**

(R_p)-*N*-(1,4(1,4)-dibenzenacyclohexaphane-1^2-yl)benzamide **(6d)**

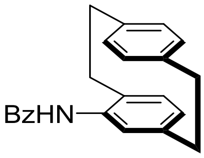

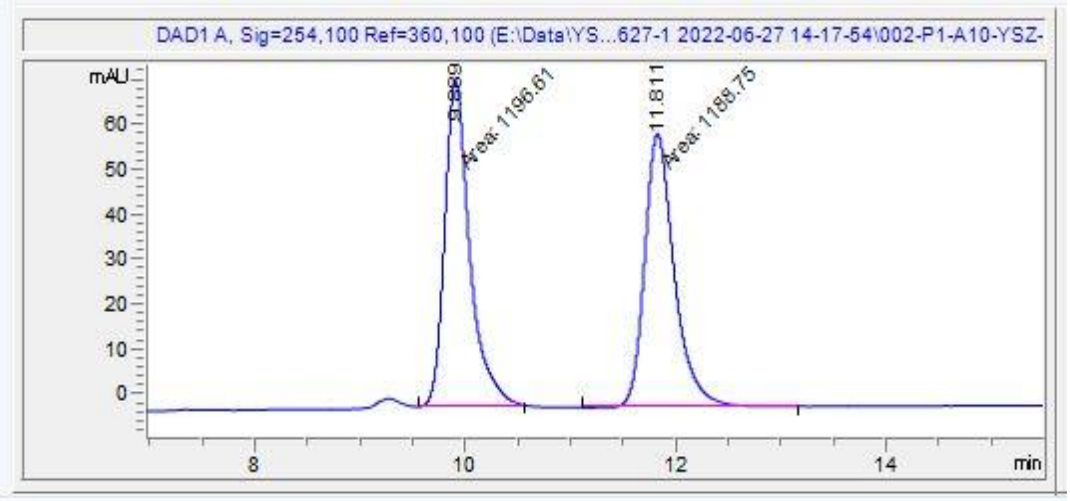

#	Time	Type	Area	Height	Width	Area%	Symmetry
1	9.889	MM	1196.6	72.3	0.276	50.165	0.692
2	11.811	MM	1188.8	60.6	0.3272	49.835	0.738

Supplementary Fig. 256 HPLC spectrum of racemic **6d**

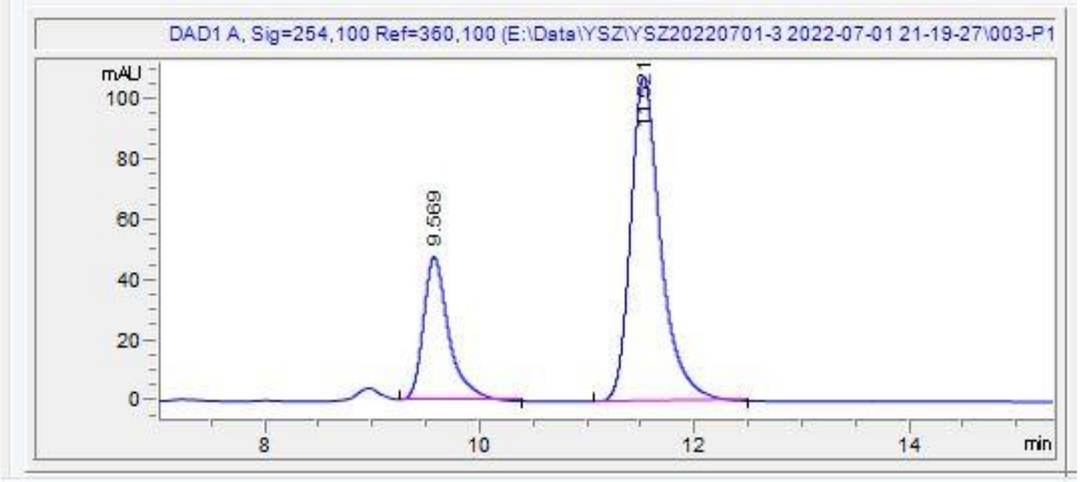

#	Time	Type	Area	Height	Width	Area%	Symmetry
1	9.569	BB	786	47.6	0.238	27.209	0.676
2	11.521	BB	2102.7	107.8	0.2841	72.791	0.705

Supplementary Fig. 257 HPLC spectrum of (R_p)-**6d**

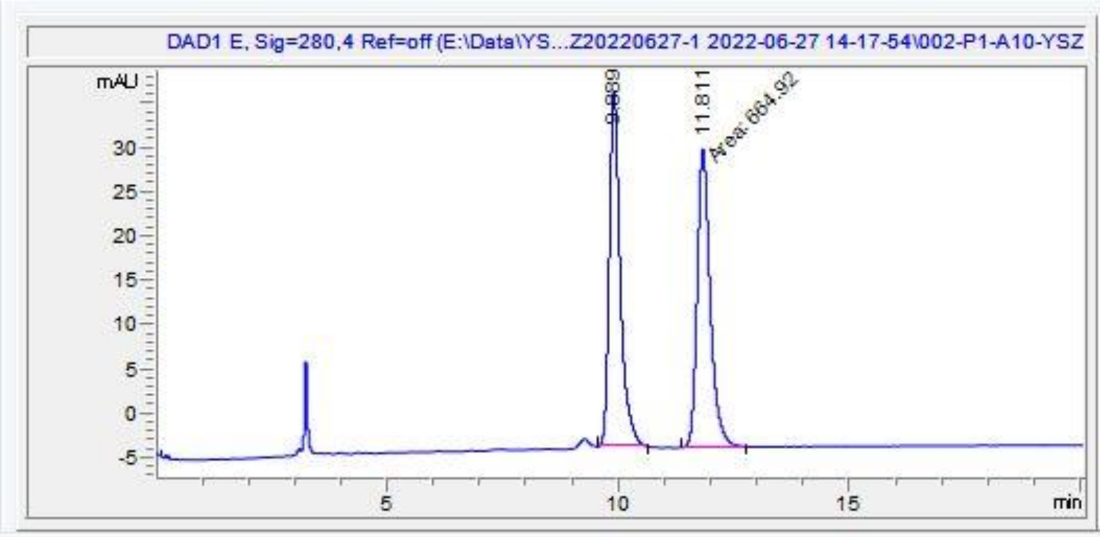

#	Time	Type	Area	Height	Width	Area%	Symmetry
1	9.889	BB	665.7	40.3	0.2203	50.030	0.682
2	11.811	MM	664.9	33.8	0.3279	49.970	0.734

Supplementary Fig. 258 Full HPLC spectrum of racemic **6d**

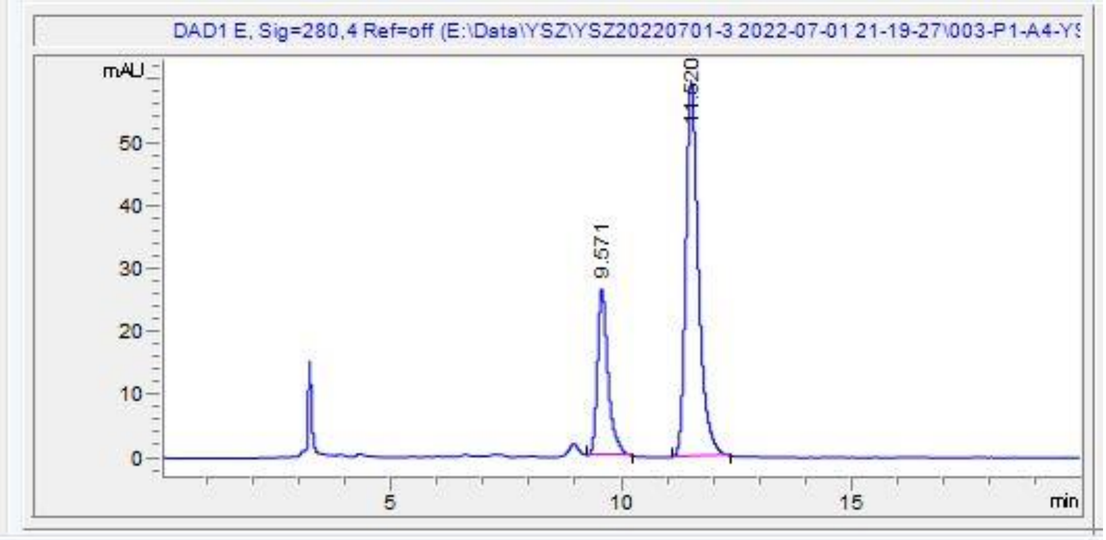

#	Time	Type	Area	Height	Width	Area%	Symmetry
1	9.571	BB	434.3	26.5	0.1938	27.171	0.723
2	11.52	BB	1164.2	59.9	0.2727	72.829	0.704

Supplementary Fig. 259 Full HPLC spectrum of (R_p)-**6d**

(S_p)-Dibenzyl-1-(1^5-benzamido-1,4(1,4)-dibenzenacyclohexaphane-1^2-yl)hydrazine-1,2-dicarboxylate **(7d)**

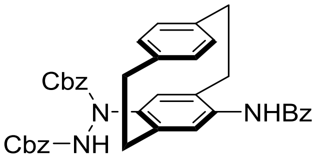

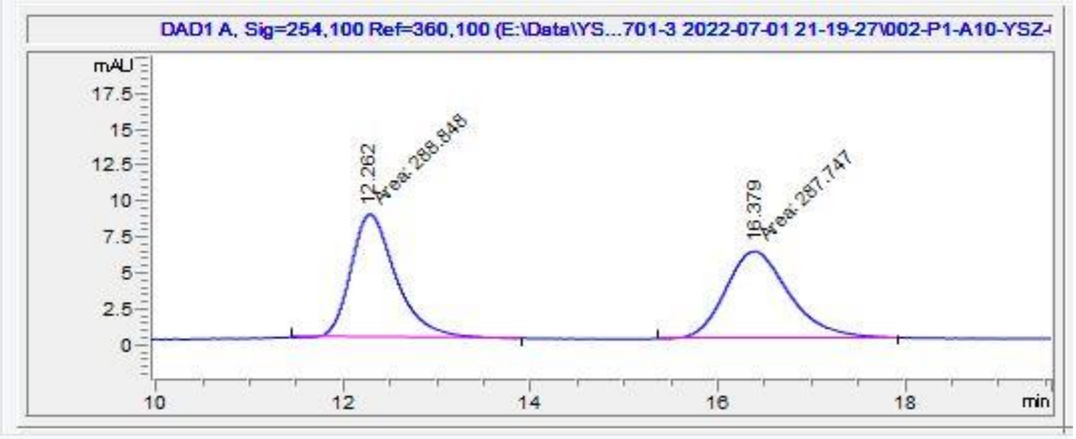

#	Time	Type	Area	Height	Width	Area%	Symmetry
1	12.262	MM	288.8	8.6	0.5603	50.095	0.666
2	16.379	MM	287.7	6.1	0.7924	49.905	0.794

Supplementary Fig. 260 HPLC spectrum of racemic **7d**

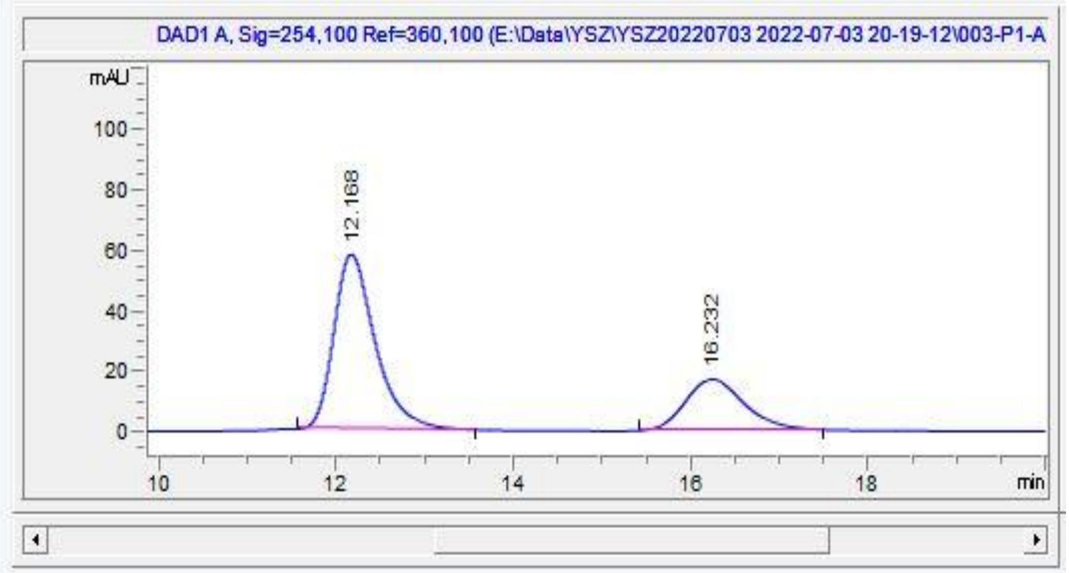

1	12.168	BB	1879.2	57.7	0.386	70.805	0.716
2	16.232	BB	774.9	16.8	0.5398	29.195	0.761

Supplementary Fig. 261 HPLC spectrum of (S_p)-**7d**

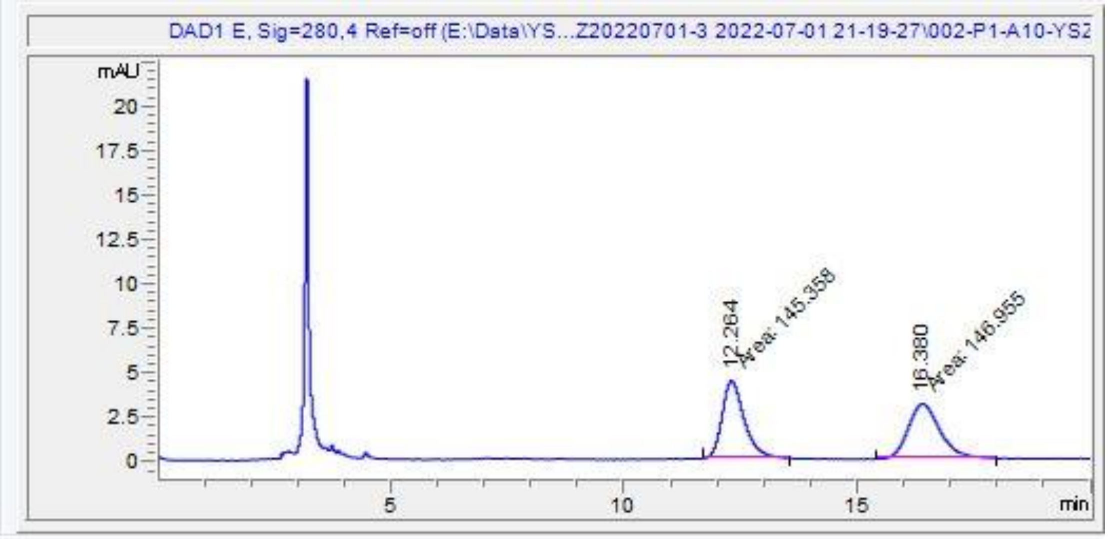

#	Time	Type	Area	Height	Width	Area%	Symmetry
1	12.264	MM	145.4	4.4	0.5547	49.727	0.679
2	16.38	MM	147	3.1	0.7904	50.273	0.83

Supplementary Fig. 262 Full HPLC spectrum of (R_p)-**7d**

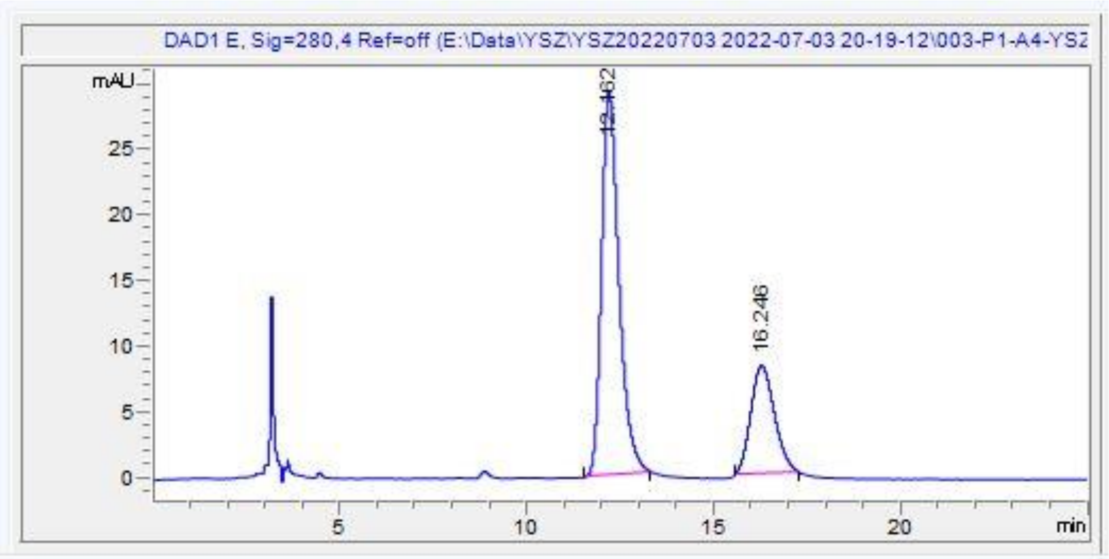

#	Time	Type	Area	Height	Width	Area%	Symmetry
1	12.162	BB	952.5	29.4	0.3795	72.161	0.688
2	16.246	BB	367.5	8.3	0.5178	27.839	0.827

Supplementary Fig. 263 Full HPLC spectrum of (R_p)-**7d**

(R_p)-*N*-(1,4(1,4)-dibenzenacyclohexaphane-1^2-yl)pivalamide **(6e)**

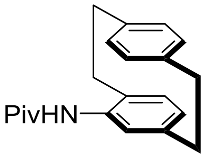

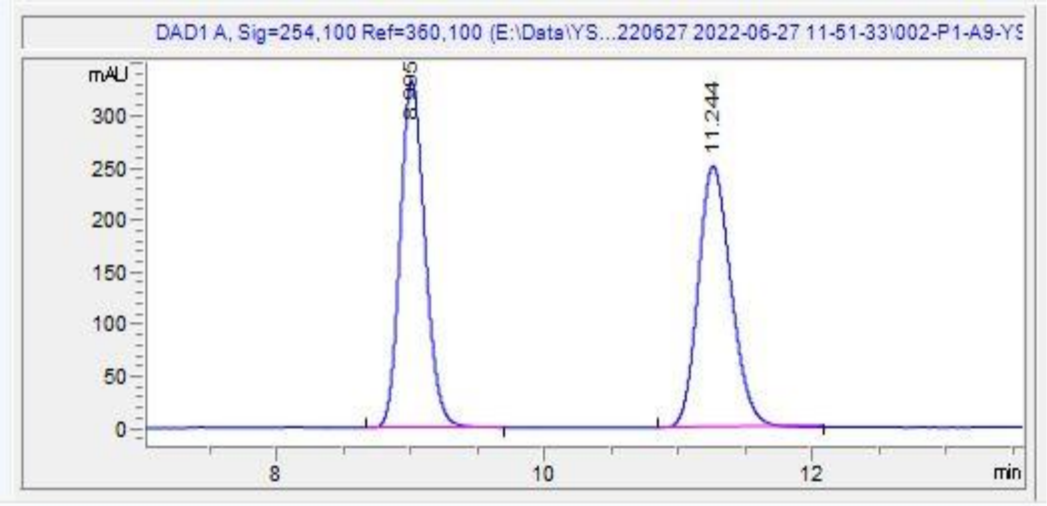

#	Time	Type	Area	Height	Width	Area%	Symmetry
1	8.995	BB	4276.5	333	0.1958	49.934	0.814
2	11.244	BB	4287.8	251.1	0.2592	50.066	0.787

Supplementary Fig. 264 HPLC spectrum of racemic **6e**

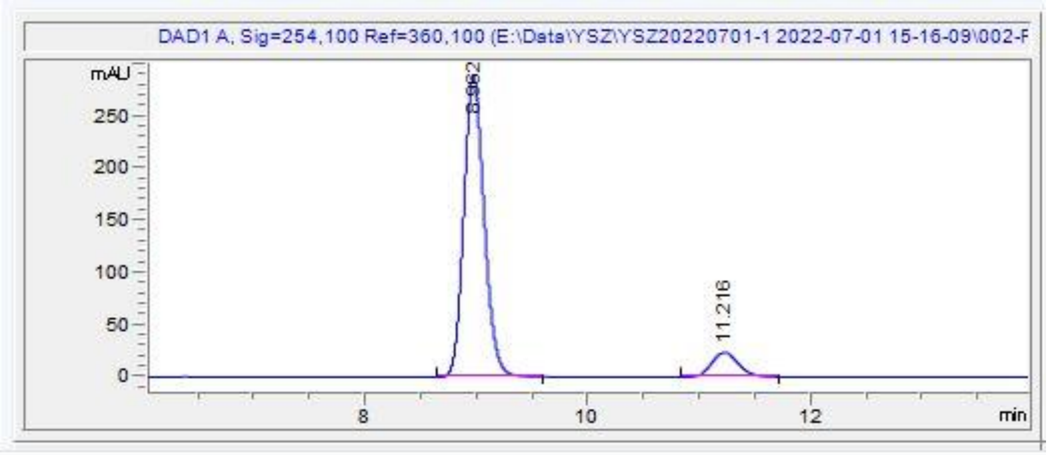

#	Time	Type	Area	Height	Width	Area%	Symmetry
1	8.962	BB	3667.9	288.4	0.1934	90.200	0.829
2	11.216	BB	398.5	23.6	0.2185	9.800	0.853

Supplementary Fig. 265 HPLC spectrum of (R_p)-**6e**

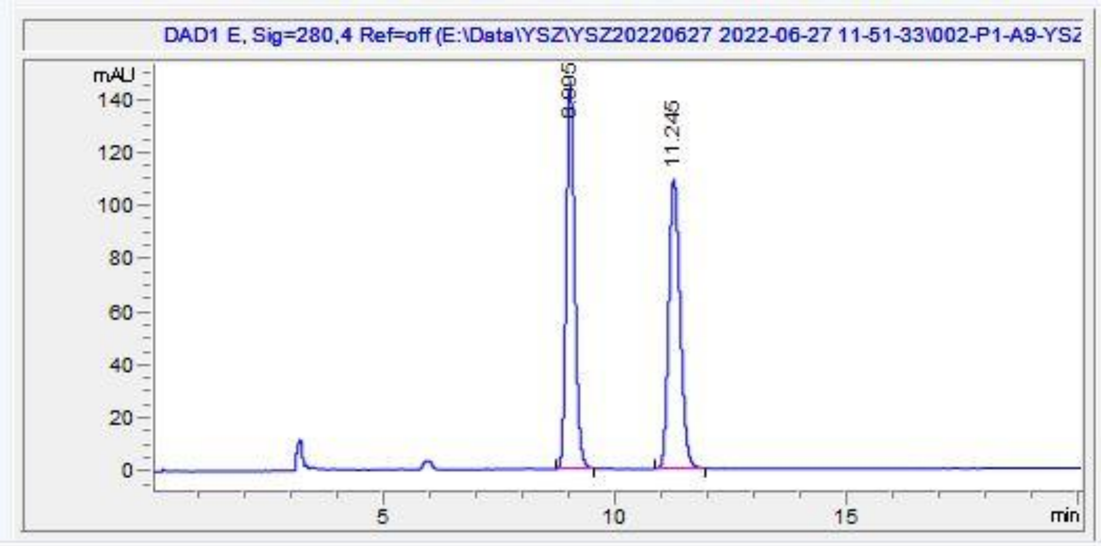

#	Time	Type	Area	Height	Width	Area%	Symmetry
1	8.995	BB	1861.1	145	0.1978	50.114	0.809
2	11.245	BB	1852.7	108.9	0.2494	49.886	0.798

Supplementary Fig. 266 Full HPLC spectrum of racemic **6e**

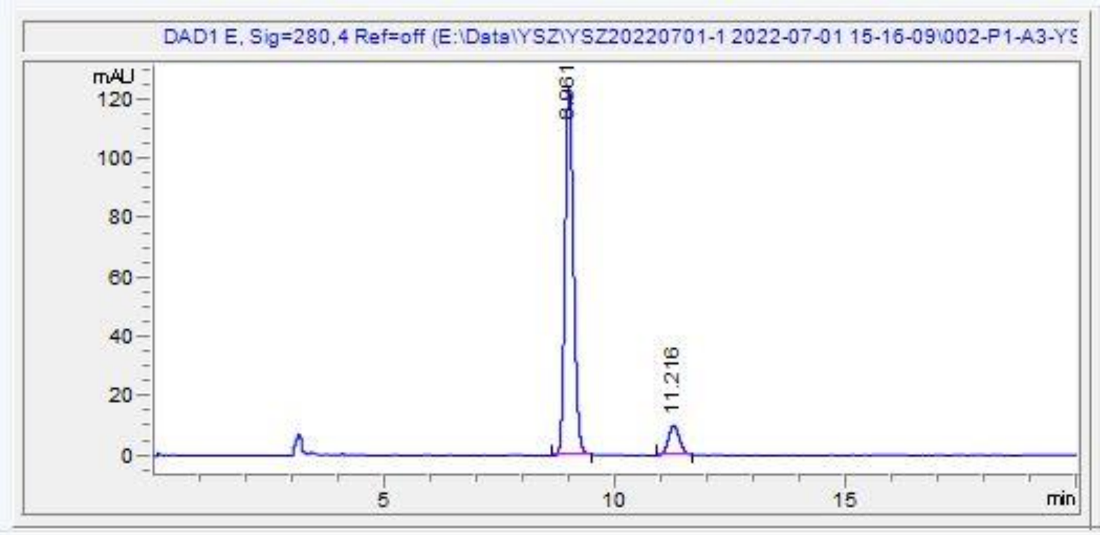

#	Time	Type	Area	Height	Width	Area%	Symmetry
1	8.961	BB	1582.6	125	0.1947	90.344	0.823
2	11.216	BB	169.2	10.1	0.1984	9.656	0.879

Supplementary Fig. 267 Full HPLC spectrum of (R_p)-**6e**

(S_p)-Dibenzyl-1-(1^5-pivalamido-1,4(1,4)-dibenzenacyclohexaphane-1^2-yl)hydrazine-1,2-dicarboxylate **(7e)**

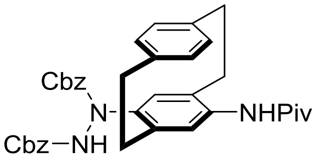

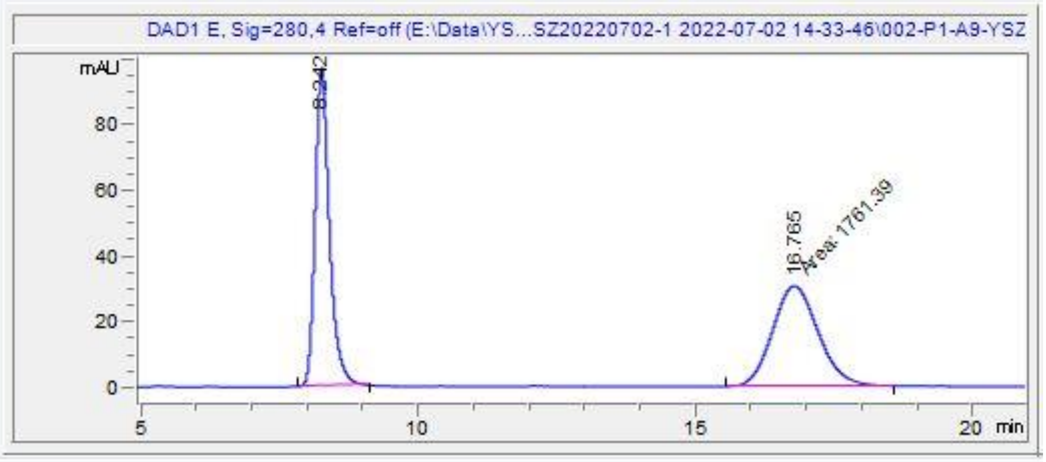

#	Time	Type	Area	Height	Width	Area%	Symmetry
1	8.242	BB	1754.8	96	0.2576	49.906	0.734
2	16.765	MM	1761.4	30.6	0.9589	50.094	0.846

Supplementary Fig. 268 HPLC spectrum of racemic **7e**

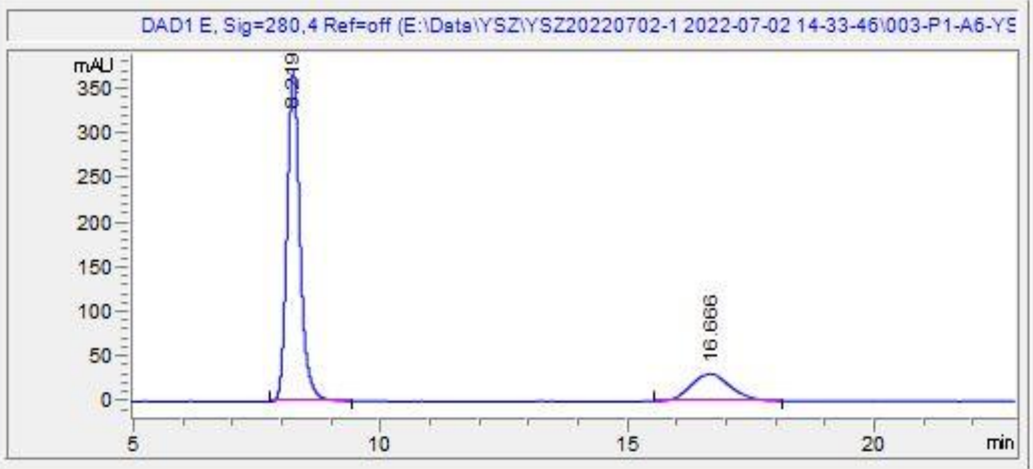

#	Time	Type	Area	Height	Width	Area%	Symmetry
1	8.219	BB	6807.9	370.2	0.2791	79.790	0.86
2	16.666	BB	1724.4	30.6	0.6604	20.210	0.875

Supplementary Fig. 269 HPLC spectrum of (S_p)-**7e**

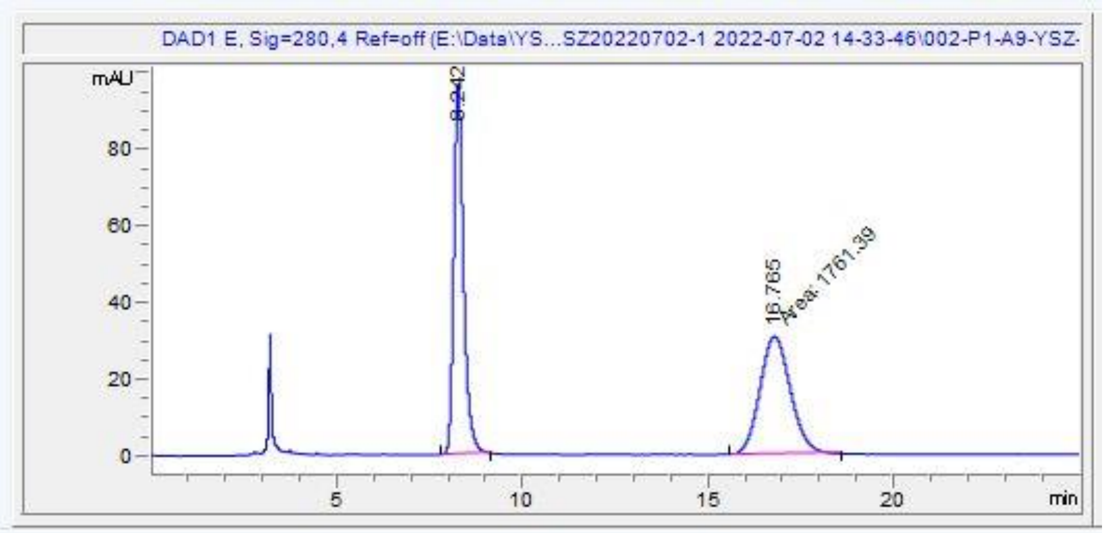

#	Time	Type	Area	Height	Width	Area%	Symmetry
1	8.242	BB	1754.8	96	0.2576	49.906	0.734
2	16.765	MM	1761.4	30.6	0.9589	50.094	0.846

Supplementary Fig. 270 Full HPLC spectrum of racemic **7e**

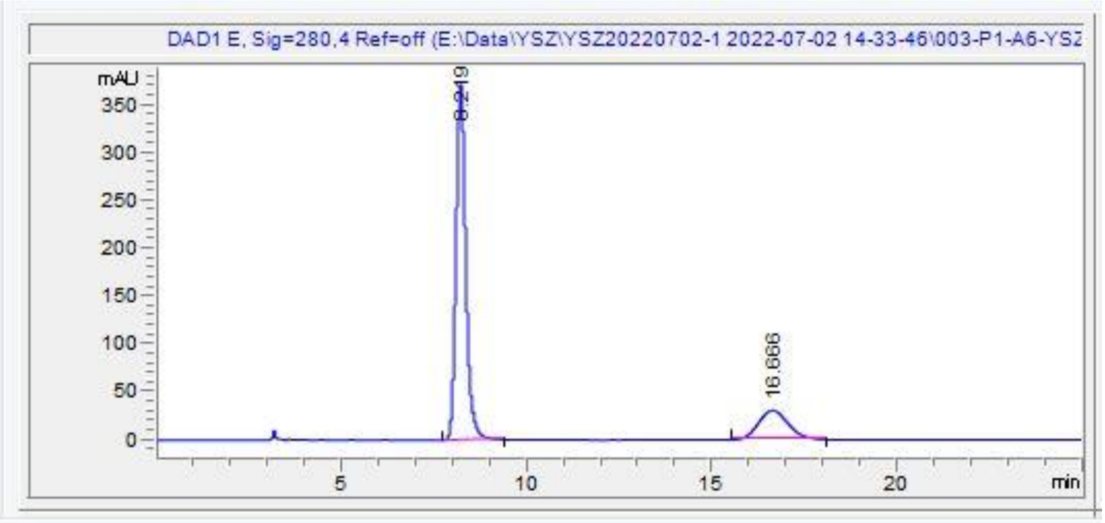

#	Time	Type	Area	Height	Width	Area%	Symmetry
1	8.219	BB	6807.9	370.2	0.2791	79.790	0.86
2	16.666	BB	1724.4	30.6	0.6604	20.210	0.875

Supplementary Fig. 271 Full HPLC spectrum of (S_p)-**7e**

N-(1,4(1,4)-dibenzenacyclohexaphane-1^{2}-yl)-4-methylbenzenesulfonamide **(6f)**

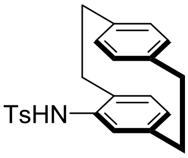

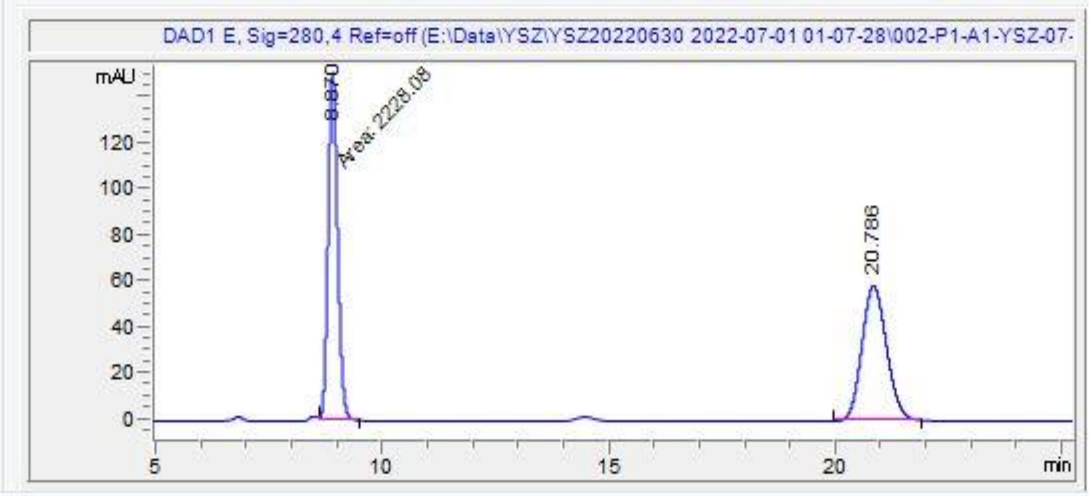

#	Time	Type	Area	Height	Width	Area%	Symmetry
1	8.87	FM	2228.1	151.8	0.2446	50.094	0.831
2	20.786	BB	2219.7	58.9	0.4415	49.906	0.863

Supplementary Fig. 272 HPLC spectrum of racemic **6f**

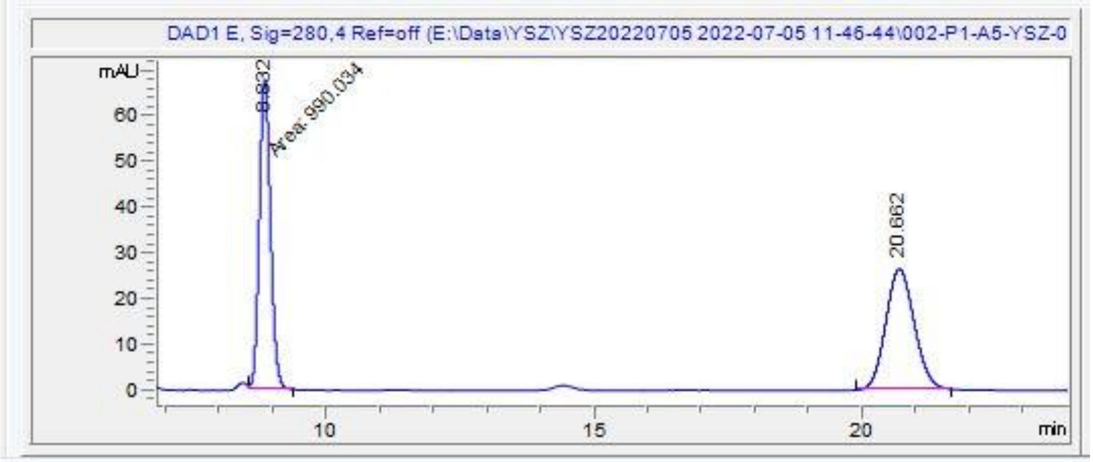

#	Time	Type	Area	Height	Width	Area%	Symmetry
1	8.832	FM	990	68.6	0.2405	50.069	0.849
2	20.662	BB	987.3	26.7	0.4346	49.931	0.923

Supplementary Fig. 273 HPLC spectrum of (R_p)**-6f**

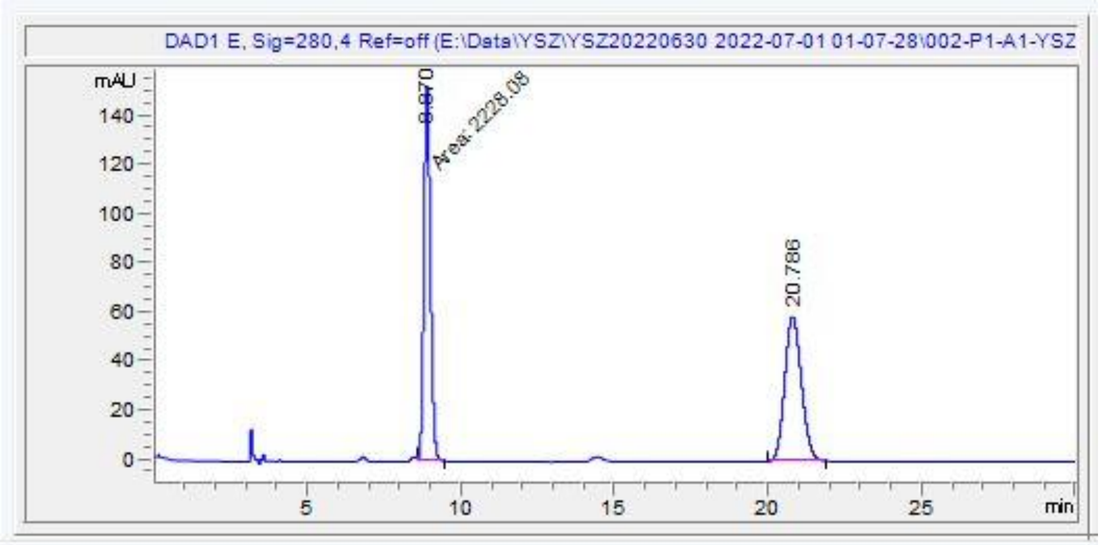

#	Time	Type	Area	Height	Width	Area%	Symmetry
1	8.87	FM	2228.1	151.8	0.2446	50.094	0.831
2	20.786	BB	2219.7	58.9	0.4415	49.906	0.863

Supplementary Fig. 274 Full HPLC spectrum of racemic **6f**

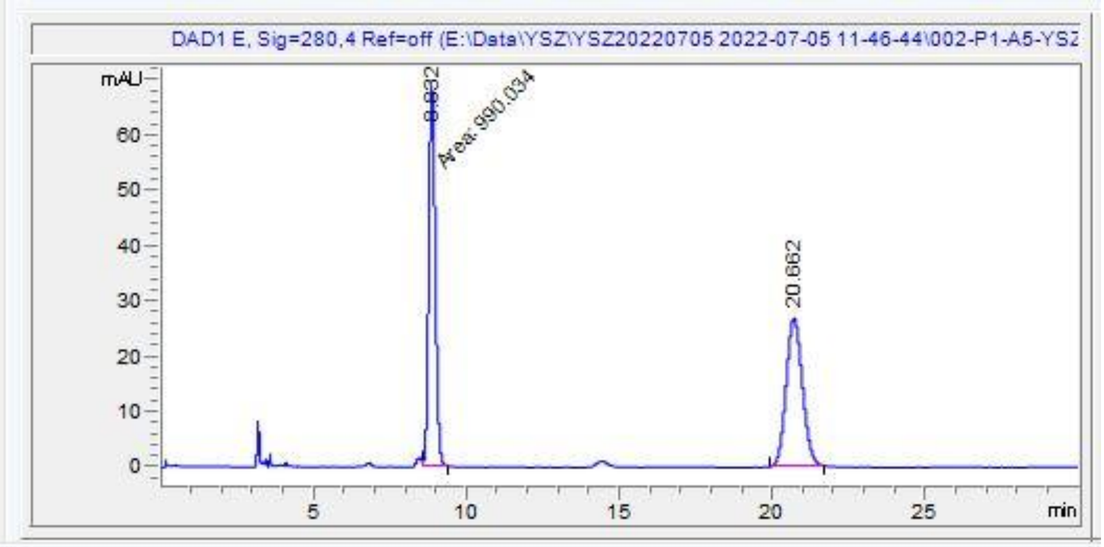

#	Time	Type	Area	Height	Width	Area%	Symmetry
1	8.832	FM	990	68.6	0.2405	50.069	0.849
2	20.662	BB	987.3	26.7	0.4346	49.931	0.923

Supplementary Fig. 275 Full HPLC spectrum of (R_p)-**6f**

Dibenzyl-1-(1^5-((4-methylphenyl)sulfonamido)-1,4(1,4)-dibenzenacyclohexaphane-1^2-yl)hydrazine-1,2-dicarboxylate **(7f)**

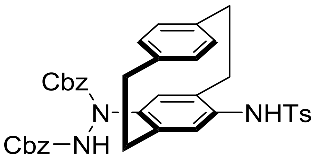

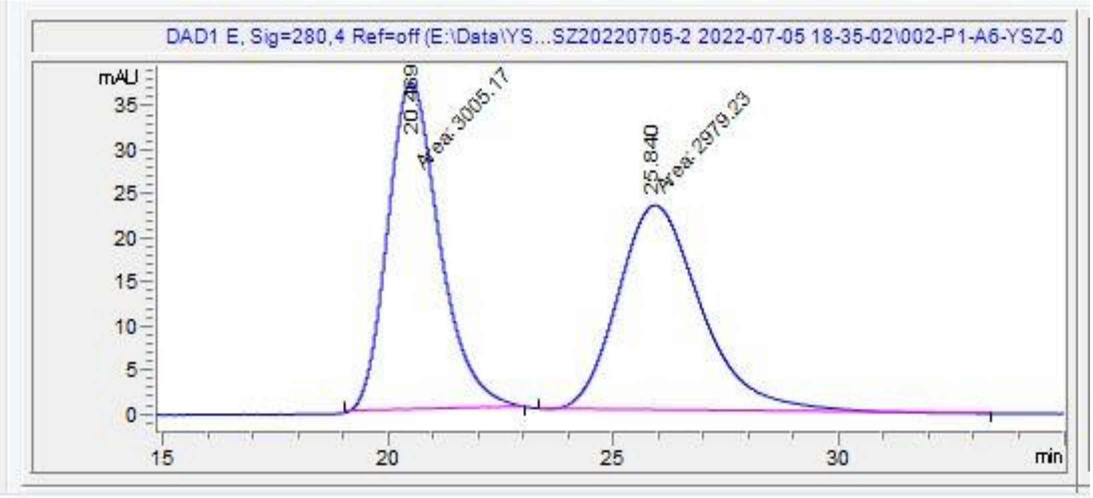

#	Time	Type	Area	Height	Width	Area%	Symmetry
1	20.469	MM	3005.2	37.3	1.3444	50.217	0.809
2	25.84	MM	2979.2	23.2	2.1391	49.783	0.727

Supplementary Fig. 276 HPLC spectrum of racemic **7f**

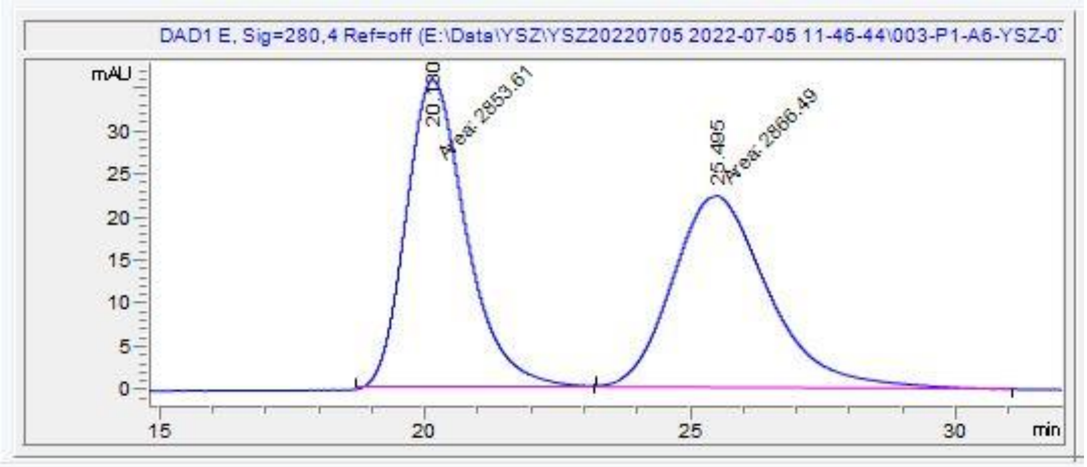

#	Time	Type	Area	Height	Width	Area%	Symmetry
1	20.13	MM	2853.6	35.9	1.3263	49.887	0.747
2	25.495	MM	2866.5	22.4	2.129	50.113	0.811

Supplementary Fig. 277 HPLC spectrum of (S_p)-**7f**

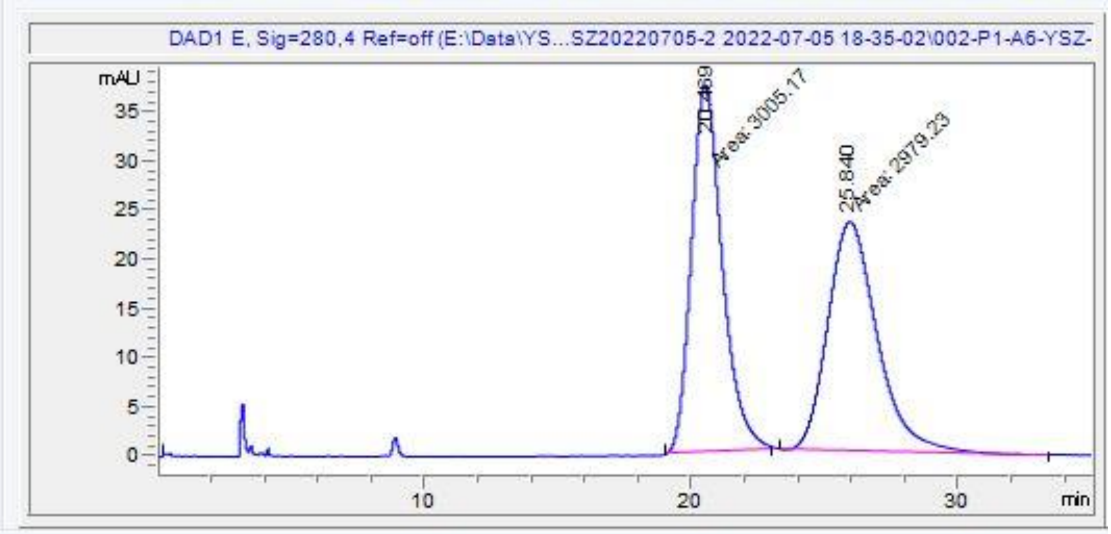

#	Time	Type	Area	Height	Width	Area%	Symmetry
1	20.469	MM	3005.2	37.3	1.3444	50.217	0.809
2	25.84	MM	2979.2	23.2	2.1391	49.783	0.727

Supplementary Fig. 278 Full HPLC spectrum of racemic **7f**

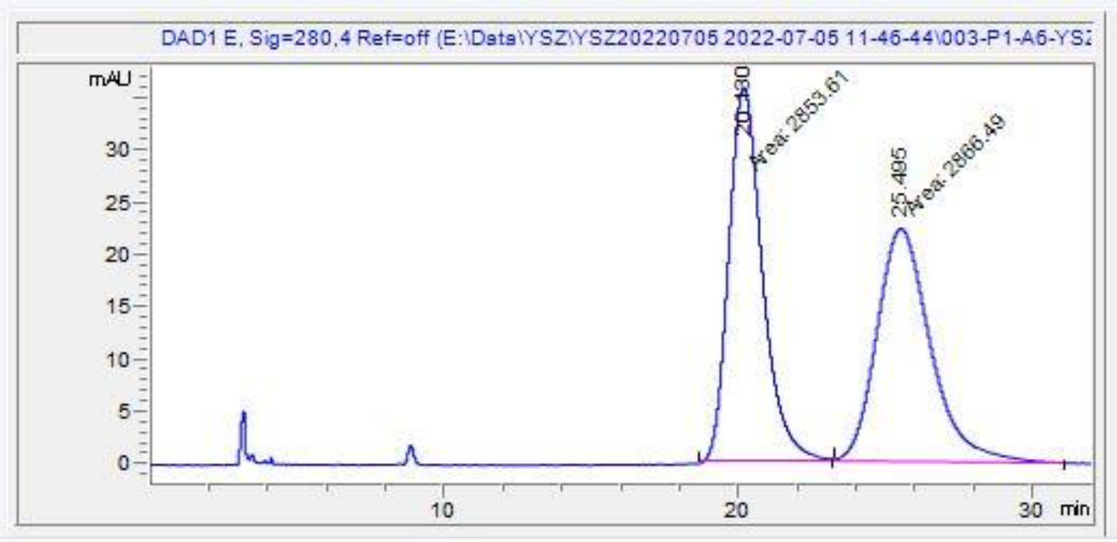

#	Time	Type	Area	Height	Width	Area%	Symmetry
1	20.13	MM	2853.6	35.9	1.3263	49.887	0.747
2	25.495	MM	2866.5	22.4	2.129	50.113	0.811

Supplementary Fig. 279 Full HPLC spectrum of (S_p)-**7f**

1,4(1,4)-dibenzenacyclohexaphan-1^2-amine **(6g)**

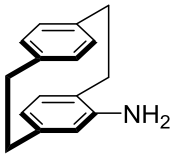

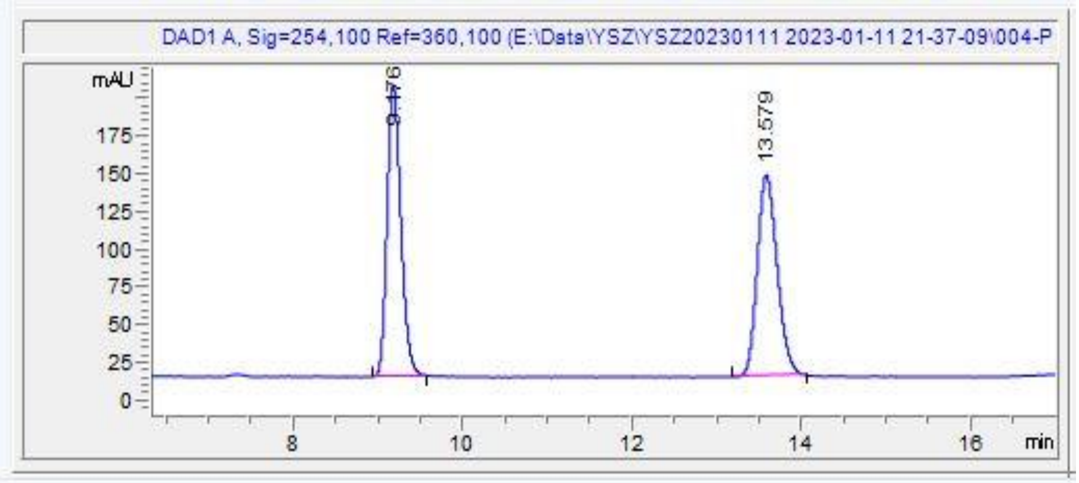

#	Time	Type	Area	Height	Width	Area%	Symmetry
1	9.176	BV R	2188.7	193.6	0.1384	49.903	0.834
2	13.579	VV R	2197.2	133.5	0.195	50.097	0.782

Supplementary Fig. 280 HPLC spectrum of racemic **6g**

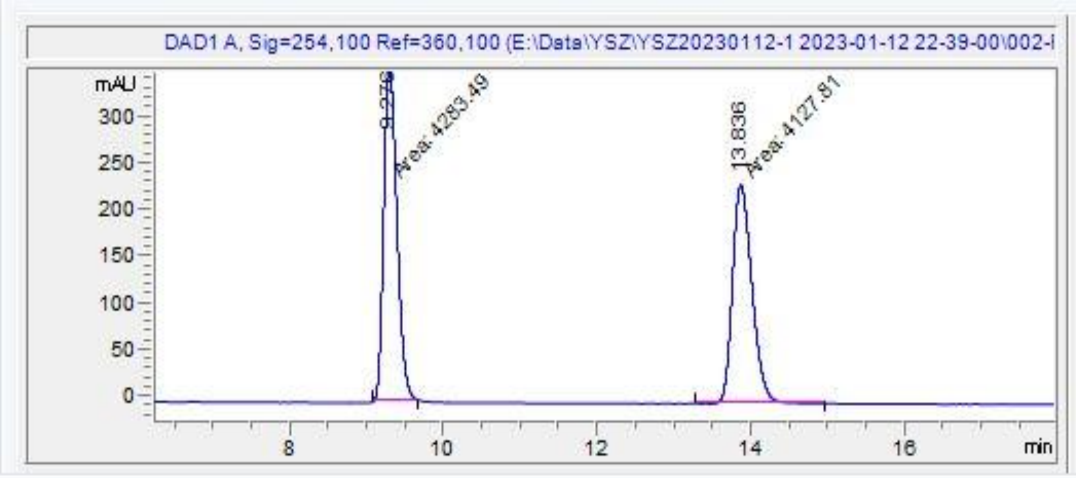

#	Time	Type	Area	Height	Width	Area%	Symmetry
1	9.276	MM	4283.5	359	0.1989	50.925	0.693
2	13.836	MM	4127.8	235.4	0.2923	49.075	0.702

Supplementary Fig. 281 HPLC spectrum of (R_p)**-6g**

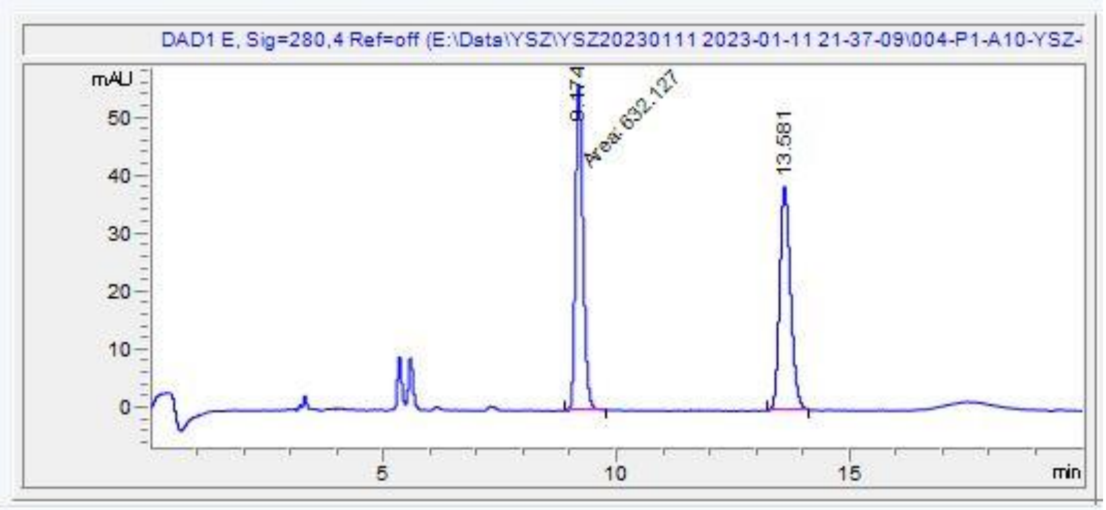

#	Time	Type	Area	Height	Width	Area%	Symmetry
1	9.174	MM	632.1	56	0.188	50.043	0.796
2	13.581	VV R	631	38.4	0.1981	49.957	0.782

Supplementary Fig. 282 Full HPLC spectrum of racemic **6g**

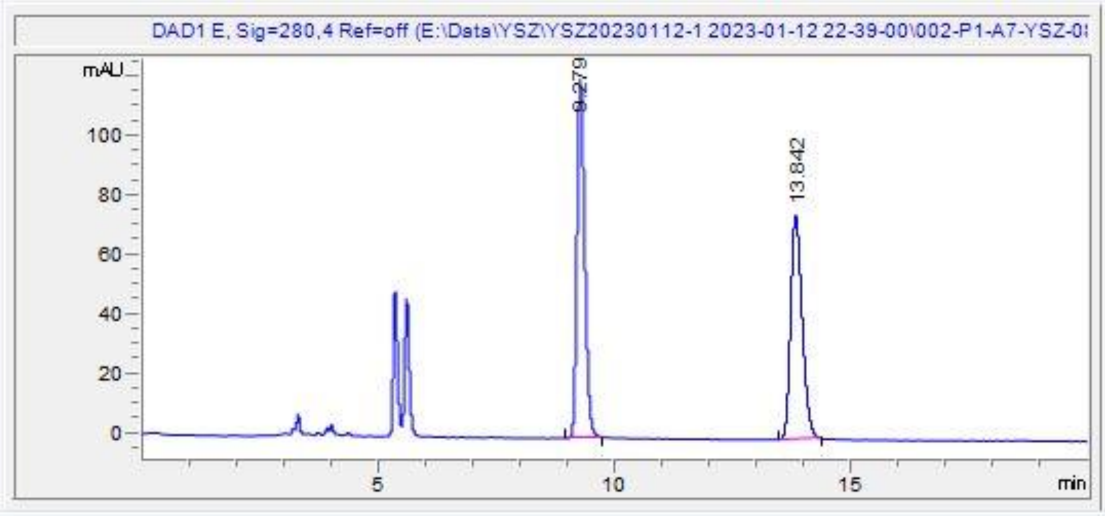

#	Time	Type	Area	Height	Width	Area%	Symmetry
1	9.279	VV R	1389.2	121.3	0.1667	51.993	0.734
2	13.842	VV R	1282.7	75.6	0.2005	48.007	0.781

Supplementary Fig. 283 Full HPLC spectrum of (R_p)-**6g**

Dibenzyl 3-(1,4(1,4)-dibenzenacyclohexaphane-1^{2}-yl)triazane-1,2-dicarboxylate **(8g)**

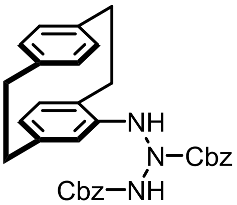

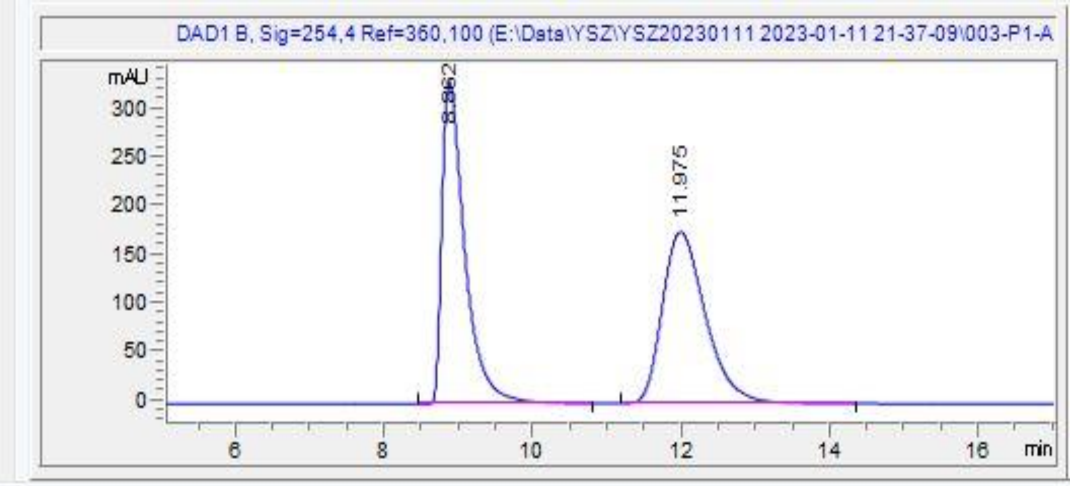

#	Time	Type	Area	Height	Width	Area%	Symmetry
1	8.862	BB	7130.2	333.7	0.3213	49.995	0.435
2	11.975	BB	7131.7	177.4	0.617	50.005	0.685

Supplementary Fig. 284 HPLC spectrum of racemic **8g**

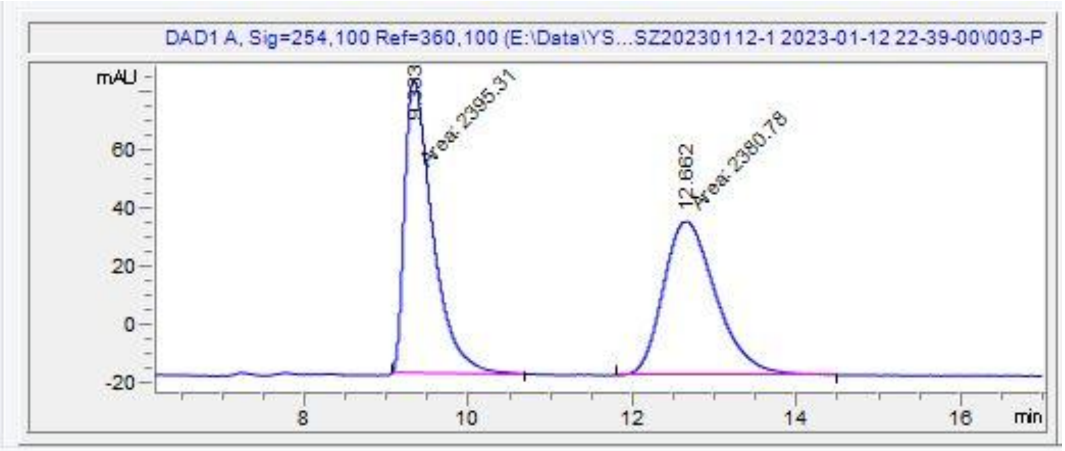

#	Time	Type	Area	Height	Width	Area%	Symmetry
1	9.333	MM	2395.3	100.5	0.3971	50.152	0.48
2	12.662	MM	2380.8	52.7	0.7531	49.848	0.746

Supplementary Fig. 285 HPLC spectrum of (S_p)-**8g**

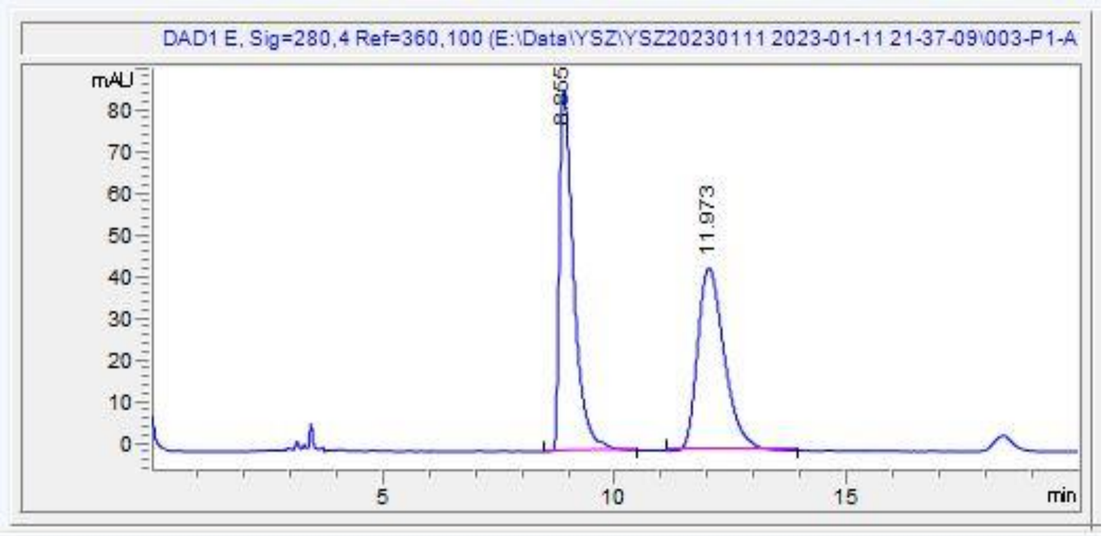

#	Time	Type	Area	Height	Width	Area%	Symmetry
1	8.855	BB	1858	87.3	0.3202	51.050	0.423
2	11.973	BB	1781.5	44.1	0.617	48.950	0.682

Supplementary Fig. 286 Full HPLC spectrum of racemic **8g**

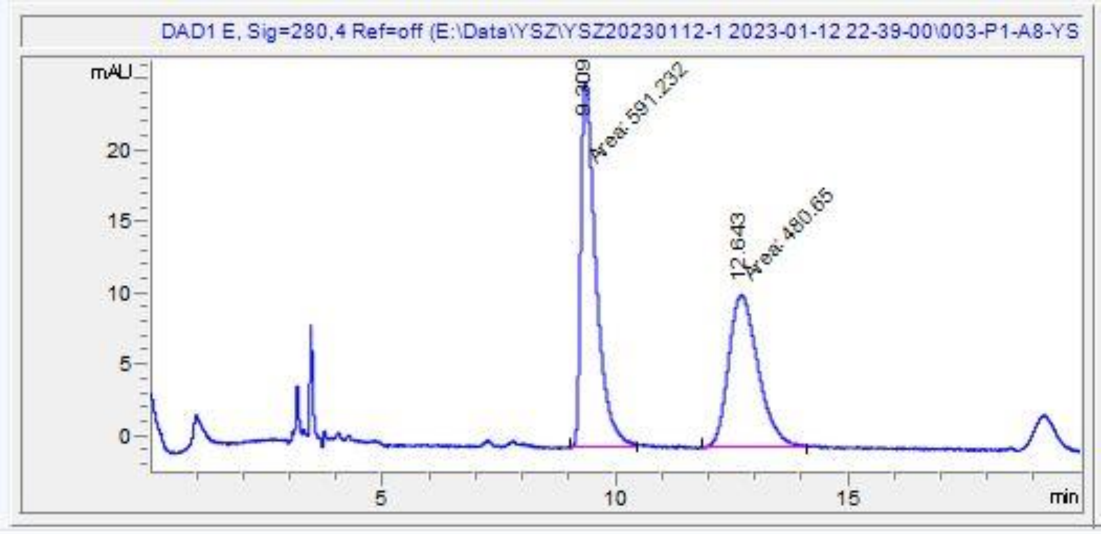

#	Time	Type	Area	Height	Width	Area%	Symmetry
1	9.309	MM	591.2	25.8	0.3814	55.158	0.422
2	12.643	MM	480.7	10.8	0.7418	44.842	0.683

Supplementary Fig. 287 Full HPLC spectrum of (S_p)-**8g**

1,4(1,4)-dibenzenacyclohexaphan-1^2-amine **(6g)**

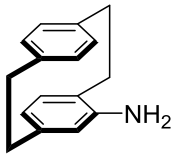

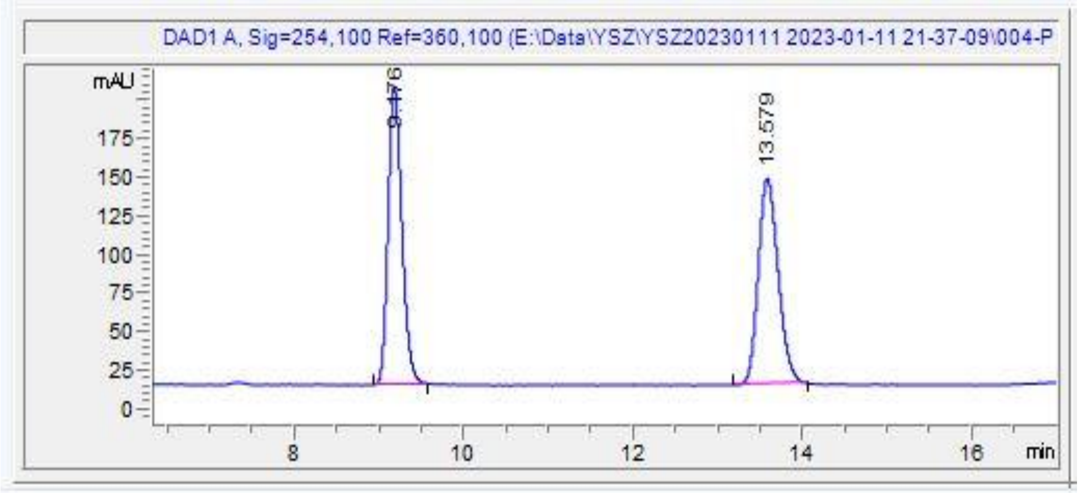

#	Time	Type	Area	Height	Width	Area%	Symmetry
1	9.176	BV R	2188.7	193.6	0.1384	49.903	0.834
2	13.579	VV R	2197.2	133.5	0.195	50.097	0.782

Supplementary Fig. 288 HPLC spectrum of racemic **6g**

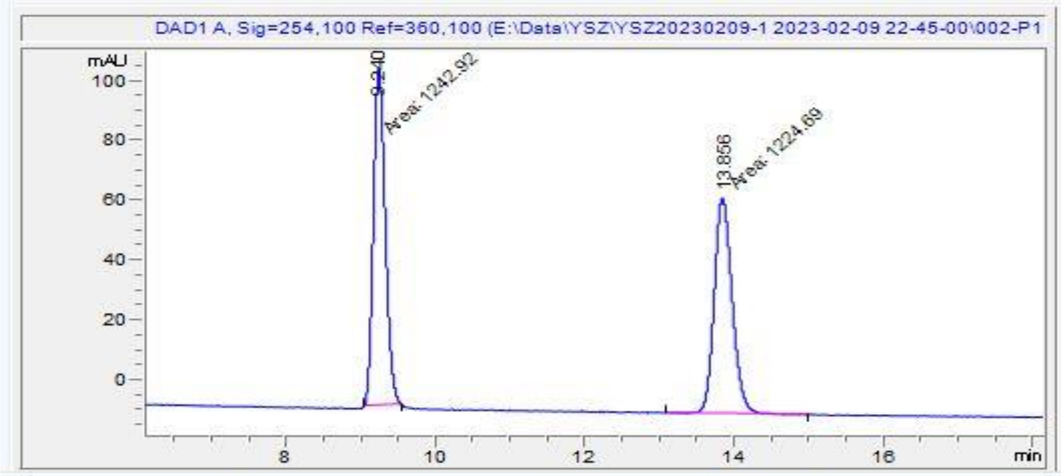

#	Time	Type	Area	Height	Width	Area%	Symmetry
1	9.24	MM	1242.9	112.4	0.1843	50.369	0.783
2	13.856	MM	1224.7	72.1	0.2831	49.631	0.897

Supplementary Fig. 289 HPLC spectrum of (R_p)**-6g**

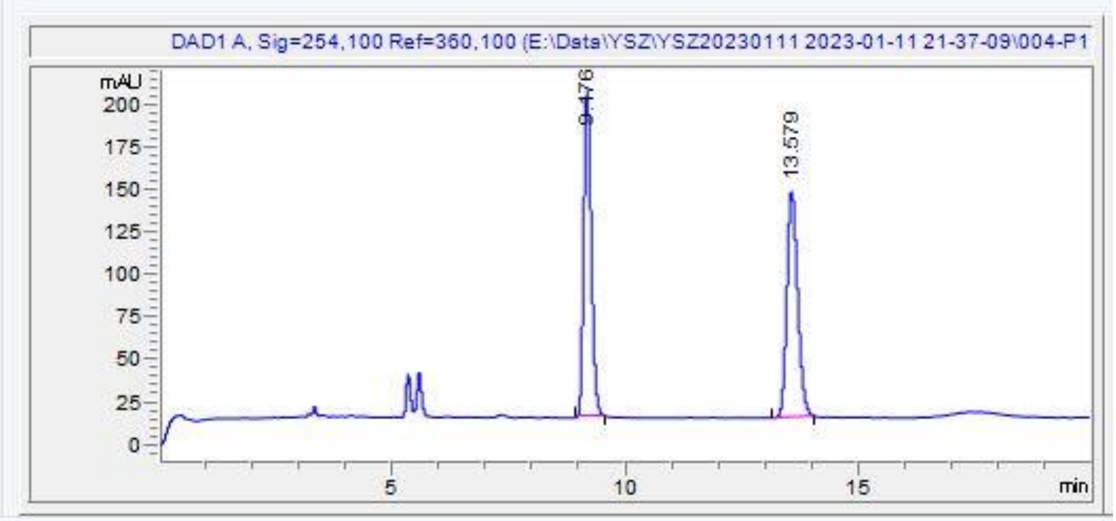

#	Time	Type	Area	Height	Width	Area%	Symmetry
1	9.176	BV R	2188.7	193.6	0.1384	49.903	0.834
2	13.579	VV R	2197.2	133.5	0.195	50.097	0.782

Supplementary Fig. 290 Full HPLC spectrum of racemic **6g**

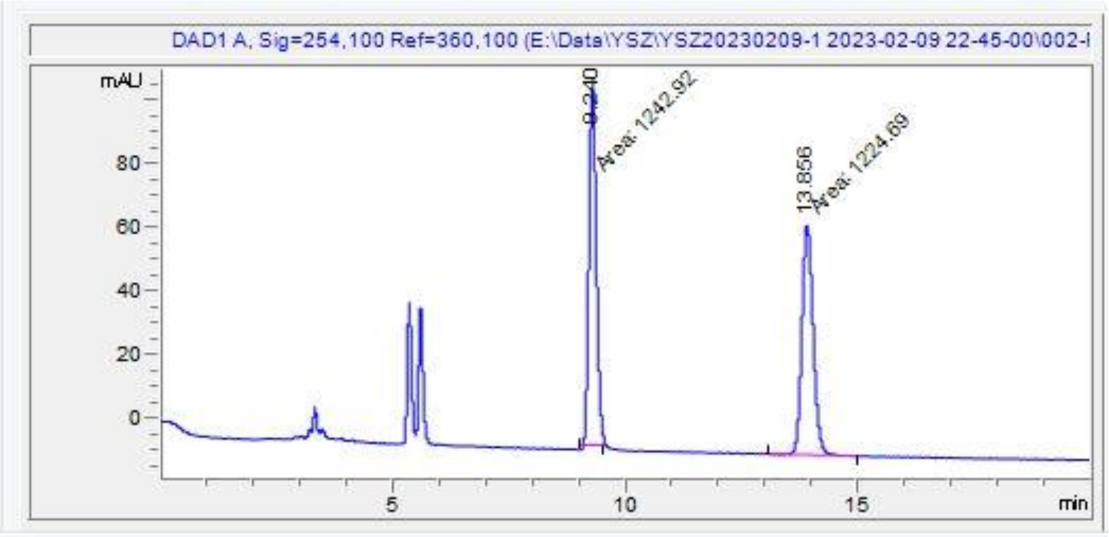

#	Time	Type	Area	Height	Width	Area%	Symmetry
1	9.24	MM	1242.9	112.4	0.1843	50.369	0.783
2	13.856	MM	1224.7	72.1	0.2831	49.631	0.897

Supplementary Fig. 291 Full HPLC spectrum of (R_p)-**6g**

Dibenzyl-1-(1^5-amino-1,4(1,4)-dibenzenacyclohexaphane-1^2-yl)hydrazine-1,2-dicarb oxylate **(7g)**

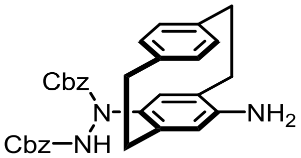

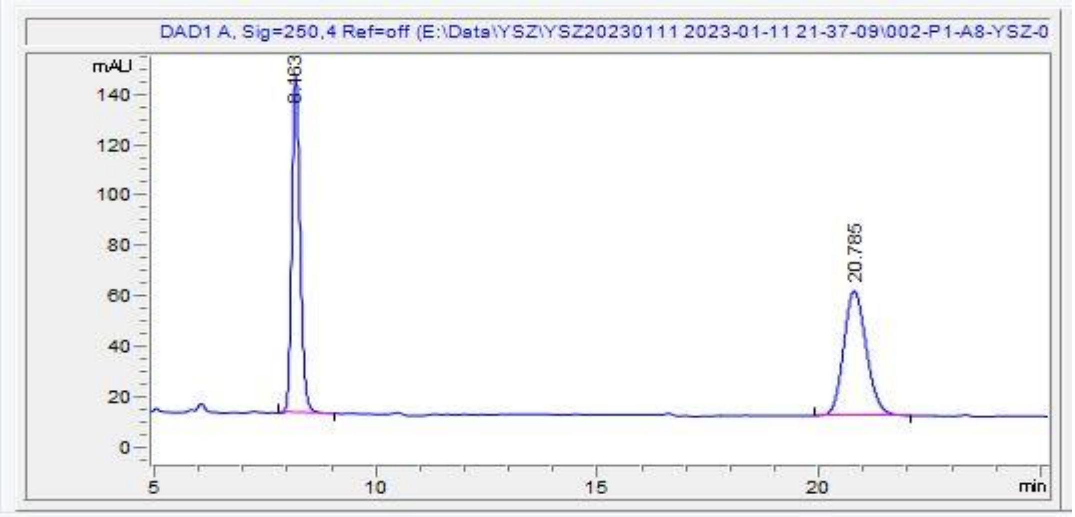

#	Time	Type	Area	Height	Width	Area%	Symmetry
1	8.163	BB	1776.7	134.2	0.2025	50.164	0.792
2	20.785	BB	1765.1	49.5	0.5524	49.836	0.835

Supplementary Fig. 292 HPLC spectrum of racemic **7g**

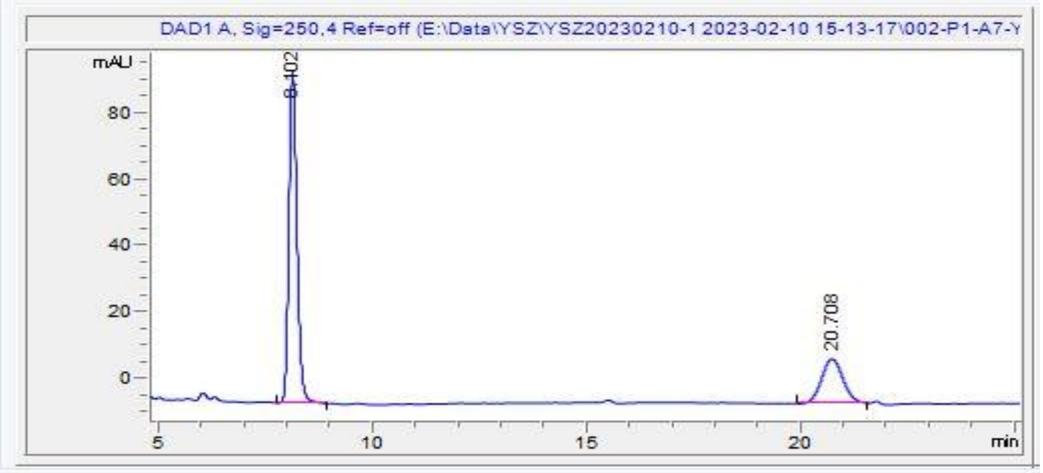

#	Time	Type	Area	Height	Width	Area%	Symmetry
1	8.102	BB	1312.1	100.4	0.2005	73.684	0.76
2	20.708	BB	468.6	13.5	0.5226	26.316	0.914

Supplementary Fig. 293 HPLC spectrum of (S_p)-**7g**

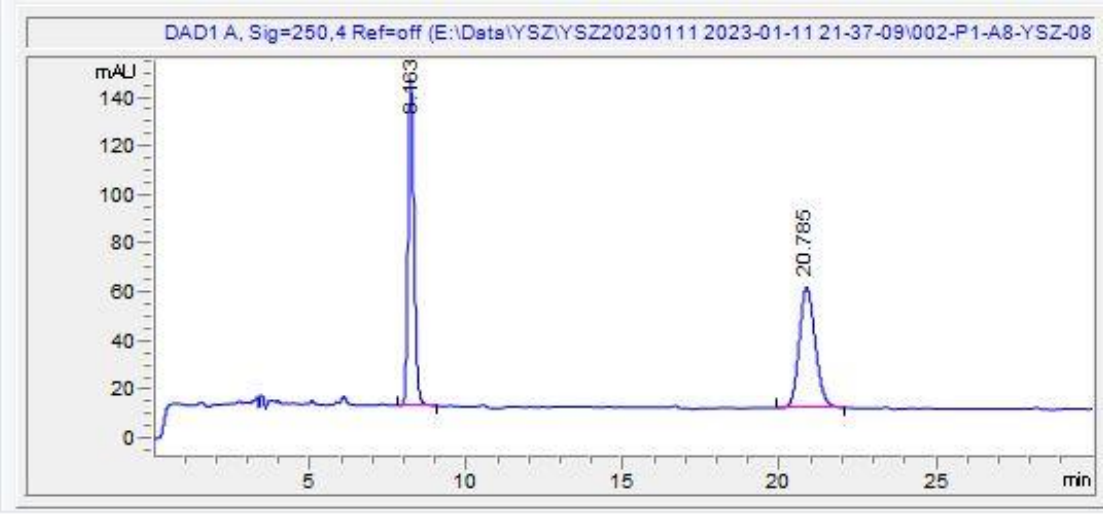

#	Time	Type	Area	Height	Width	Area%	Symmetry
1	8.163	BB	1776.7	134.2	0.2025	50.164	0.792
2	20.785	BB	1765.1	49.5	0.5524	49.836	0.835

Supplementary Fig. 294 Full HPLC spectrum of racemic **7g**

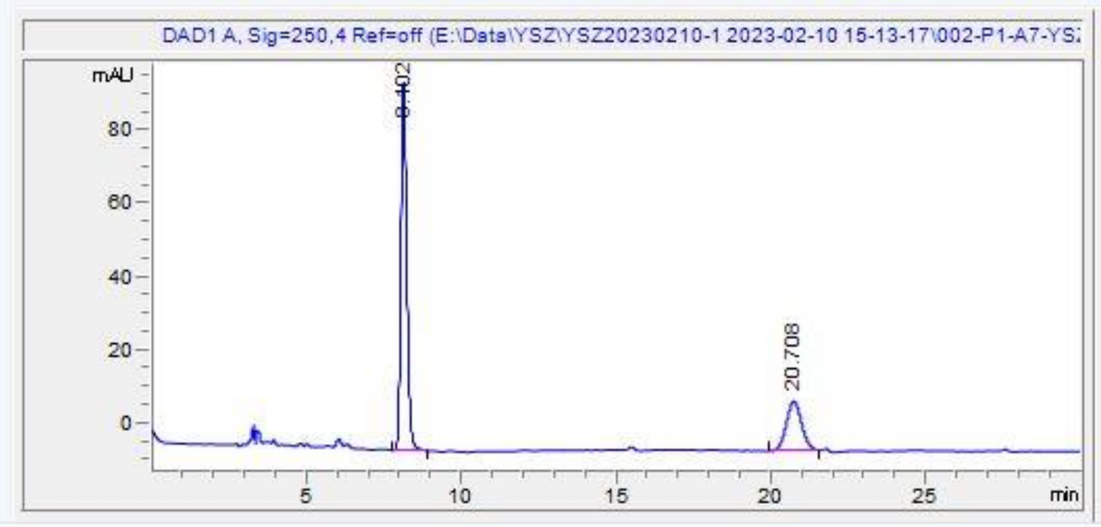

#	Time	Type	Area	Height	Width	Area%	Symmetry
1	8.102	BB	1312.1	100.4	0.2005	73.684	0.76
2	20.708	BB	468.6	13.5	0.5226	26.316	0.914

Supplementary Fig. 295 Full HPLC spectrum of (S_p)-**7g**

Dibenzyl 3-(1,4(1,4)-dibenzenacyclohexaphane-1^{2}-yl)triazane-1,2-dicarboxylate **(8g)**

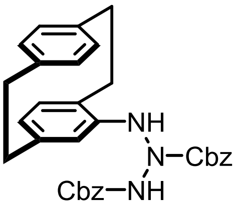

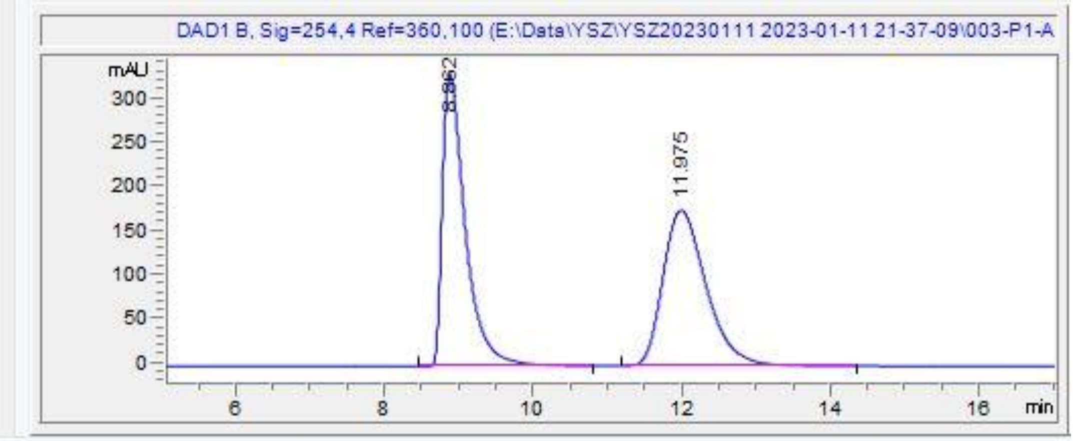

#	Time	Type	Area	Height	Width	Area%	Symmetry
1	8.862	BB	7130.2	333.7	0.3213	49.995	0.435
2	11.975	BB	7131.7	177.4	0.617	50.005	0.685

Supplementary Fig. 296 HPLC spectrum of racemic **8g**

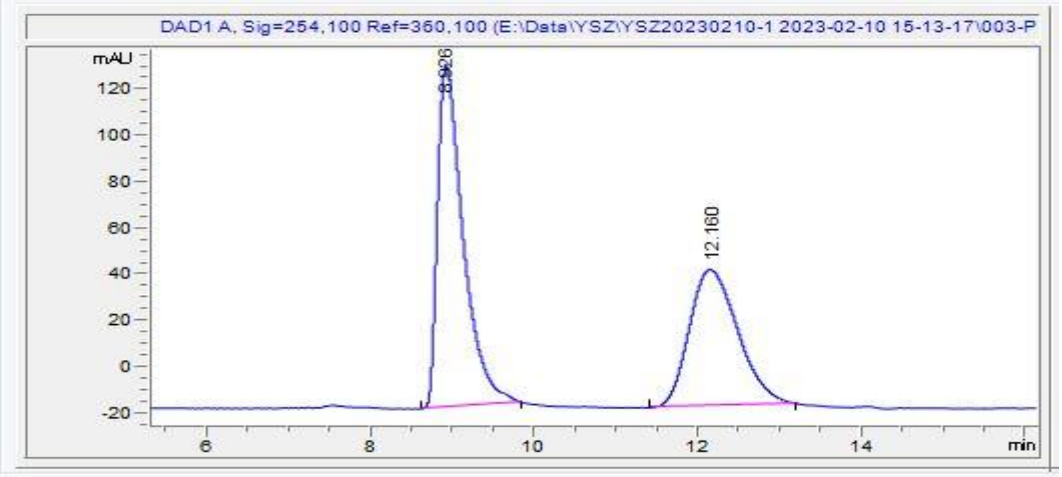

#	Time	Type	Area	Height	Width	Area%	Symmetry
1	8.926	BV R	3238.8	147.9	0.257	57.026	0.479
2	12.16	VV R	2440.7	59	0.4863	42.974	0.728

Supplementary Fig. 297 HPLC spectrum of (S_p)-**8g**

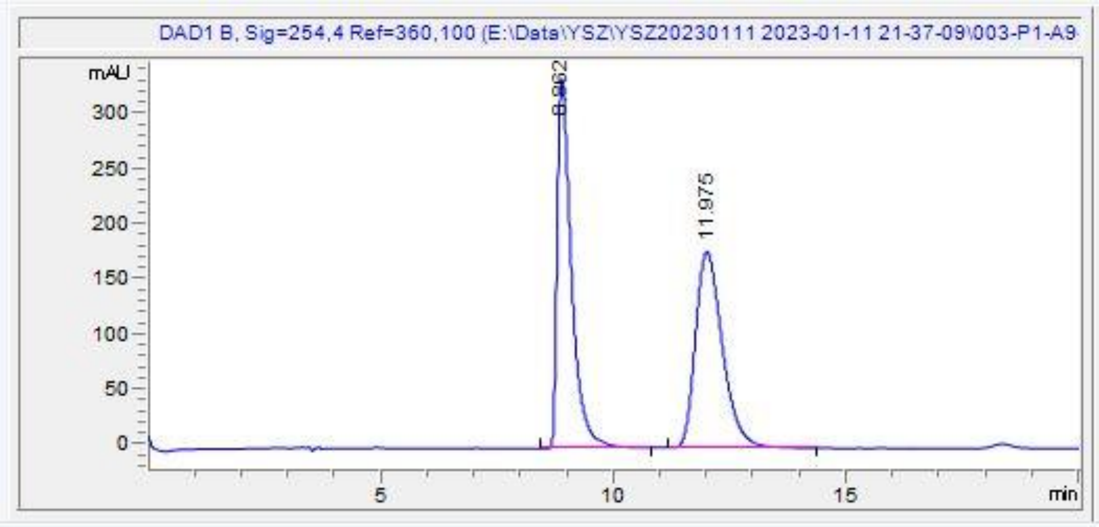

#	Time	Type	Area	Height	Width	Area%	Symmetry
1	8.862	BB	7130.2	333.7	0.3213	49.995	0.435
2	11.975	BB	7131.7	177.4	0.617	50.005	0.685

Supplementary Fig. 298 Full HPLC spectrum of racemic **8g**

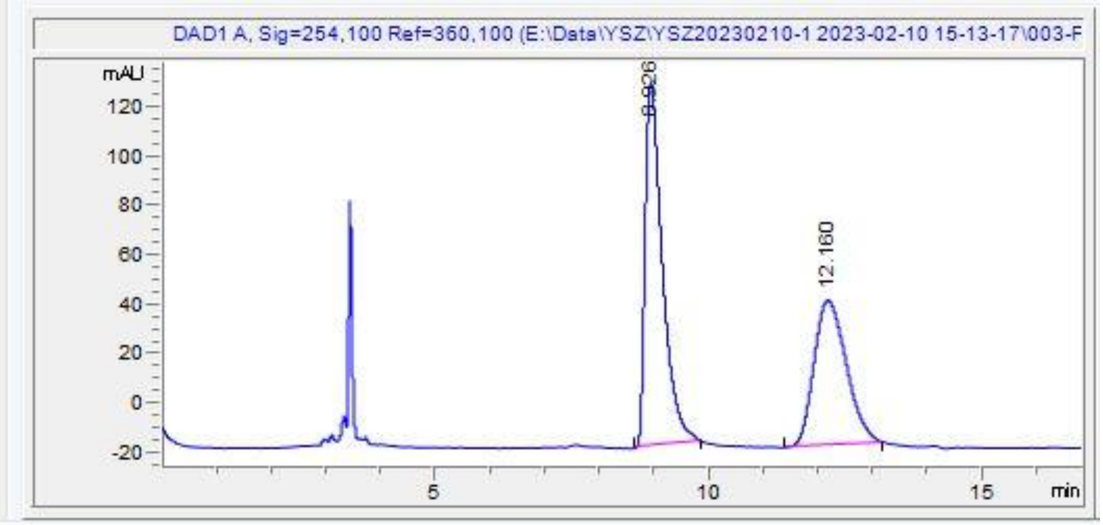

#	Time	Type	Area	Height	Width	Area%	Symmetry
1	8.926	BV R	3238.8	147.9	0.257	57.026	0.479
2	12.16	VV R	2440.7	59	0.4863	42.974	0.728

Supplementary Fig. 299 Full HPLC spectrum of (S_p)**-8g**

Kinetic resolution of **1x** under standard conditions

(R_p)-Tert-butyl (4^2-phenyl-1,4(1,4)-dibenzenacyclohexaphane-1^2-yl)carbamate **(1x)**

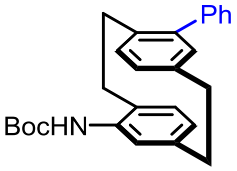

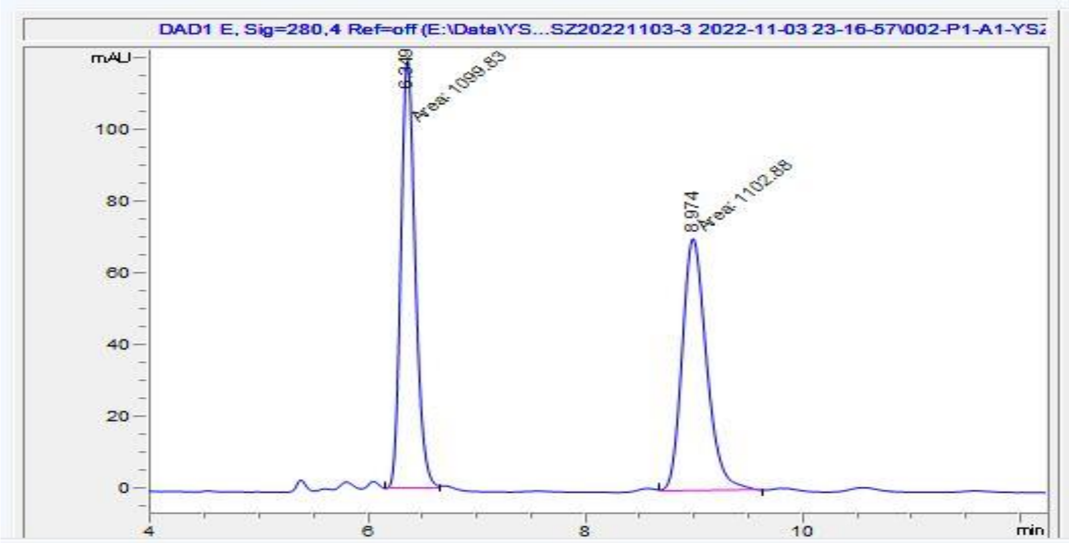

#	Time	Type	Area	Height	Width	Area%	Symmetry
1	6.349	MM	1099.8	119.3	0.1537	49.931	0.806
2	8.974	FM	1102.9	70.5	0.2608	50.069	0.777

Supplementary Fig. 300 HPLC spectrum of racemic **1x**

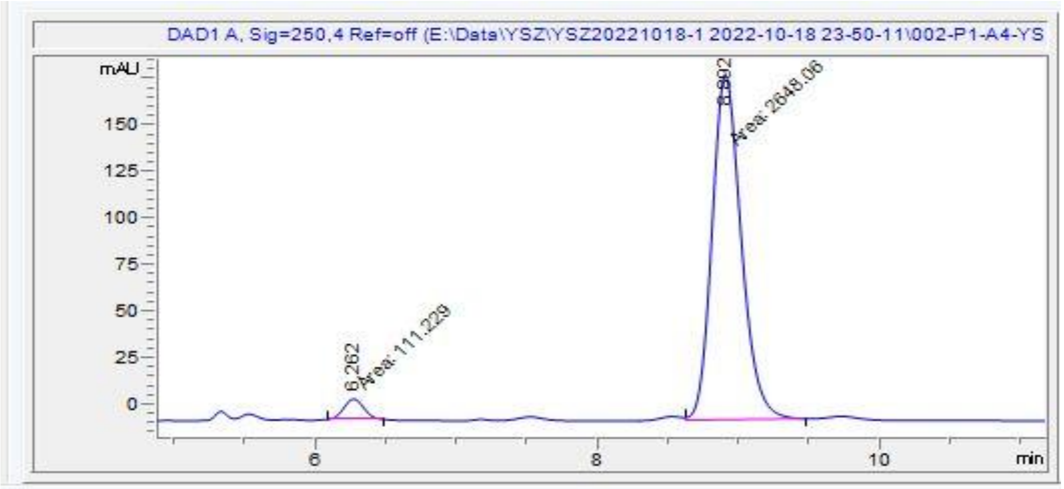

#	Time	Type	Area	Height	Width	Area%	Symmetry
1	6.262	MM	111.2	11.1	0.1674	4.031	0.964
2	8.892	FM	2648.1	184.4	0.2393	95.969	0.801

Supplementary Fig. 301 HPLC spectrum of (R_p)**-1x**

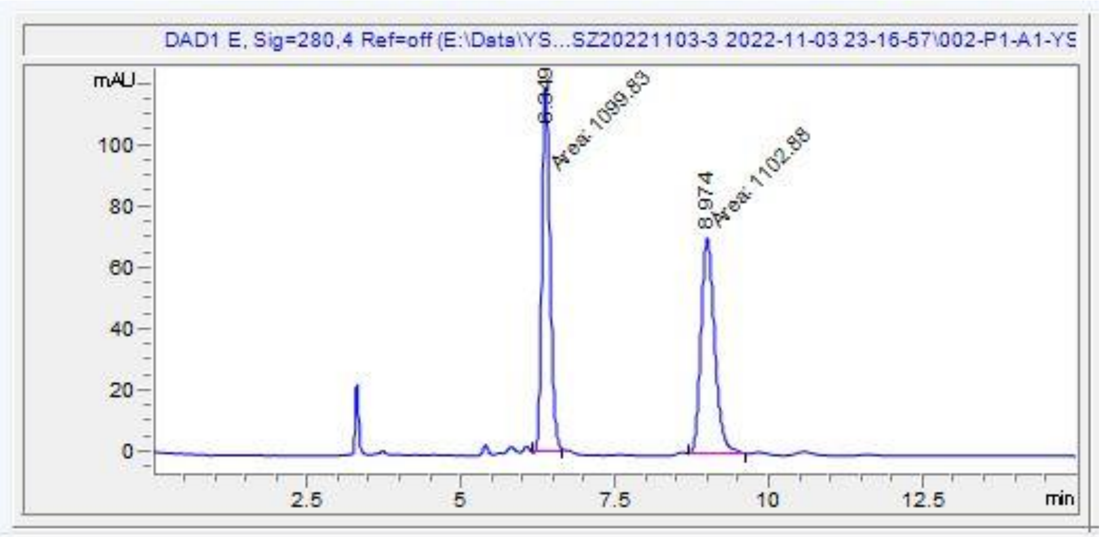

#	Time	Type	Area	Height	Width	Area%	Symmetry
1	6.349	MM	1099.8	119.3	0.1537	49.931	0.806
2	8.974	FM	1102.9	70.5	0.2608	50.069	0.777

Supplementary Fig. 302 Full HPLC spectrum of racemic **1x**

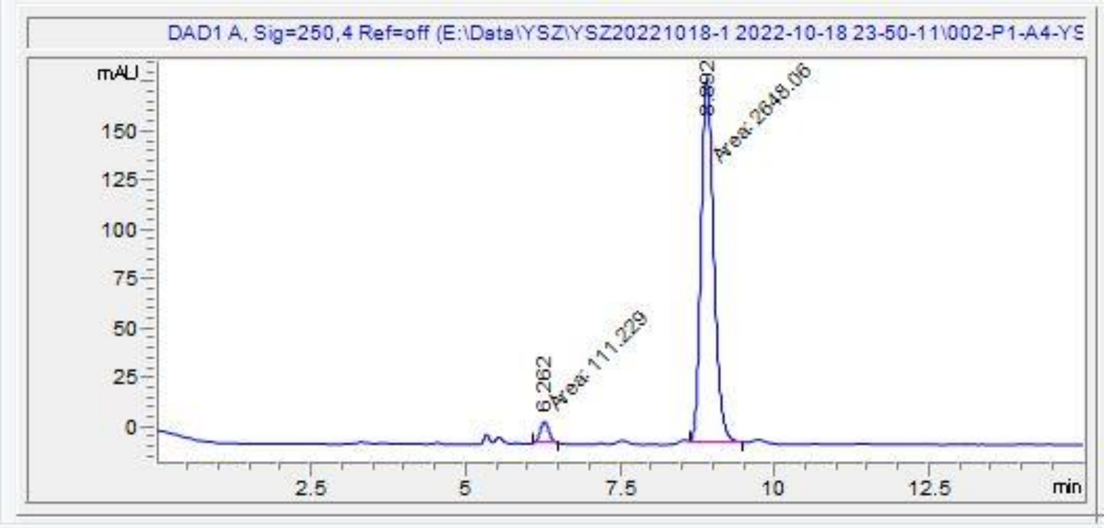

#	Time	Type	Area	Height	Width	Area%	Symmetry
1	6.262	MM	111.2	11.1	0.1674	4.031	0.964
2	8.892	FM	2648.1	184.4	0.2393	95.969	0.801

Supplementary Fig. 303 Full HPLC spectrum of (R_p)**-1x**

Dibenzyl-1-(1^5-((tert-butoxycarbonyl)amino)-4^3-phenyl-1,4(1,4)-dibenzenacyclohexaphane-1^2-yl)hydrazine-1,2-dicarboxylate **(3x)** and 2,3-dibenzyl 1-(tert-butyl) 1-(4^2-phenyl-1,4(1,4)-dibenzenacyclohexaphane-1^2-yl)triazane-1,2,3-tricarboxylate **(8x)**

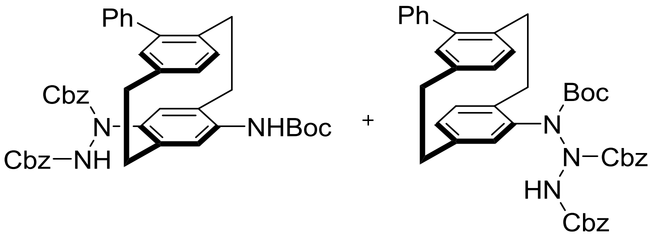

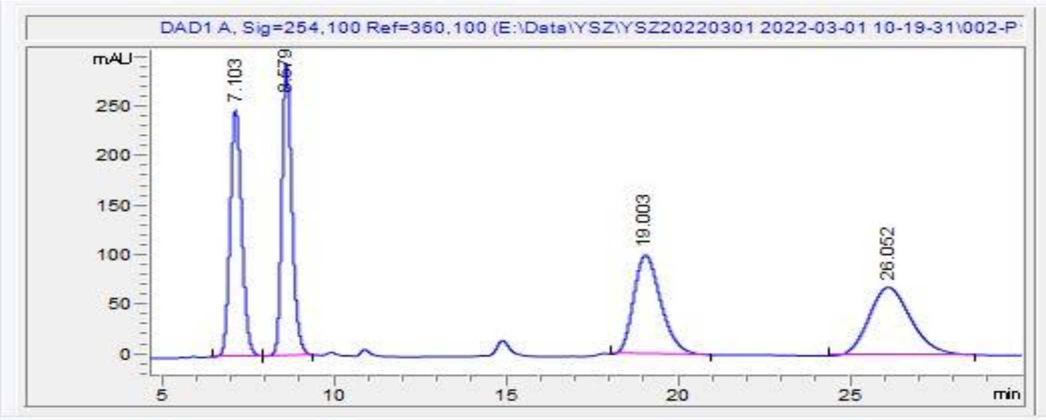

#	Time	Type	Area	Height	Width	Area%	Symmetry
1	7.103	BB	5838.6	248.7	0.3569	25.199	0.853
2	8.579	BB	5967.7	293.4	0.3083	25.756	0.764
3	19.003	BB	5535	100.1	0.6484	23.888	0.727
4	26.052	BB	5828.9	68.5	0.996	25.157	0.811

Supplementary Fig. 304 HPLC spectrum of racemic **3x** and **8x**

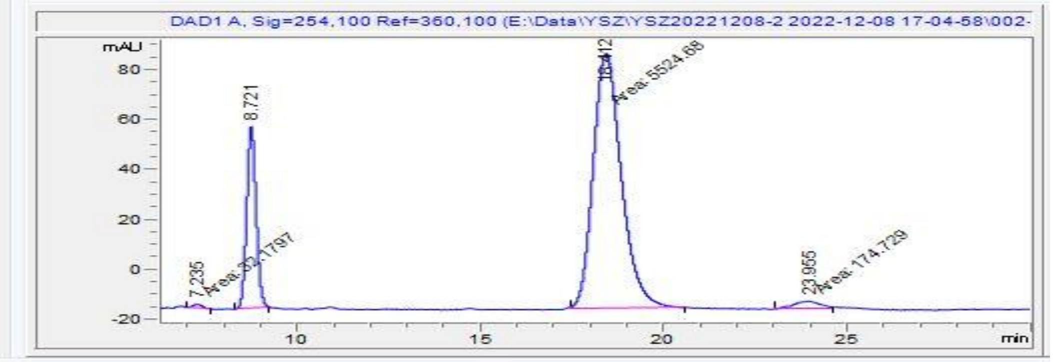

#	Time	Type	Area	Height	Width	Area%	Symmetry
1	7.235	MM	32.2	1.8	0.3062	0.456	0.641
2	8.721	VV R	1323.8	73	0.2129	18.763	0.952
3	18.412	MM	5524.7	102.6	0.8973	78.304	0.809
4	23.955	MM	174.7	3.3	0.8872	2.477	1.417

Supplementary Fig. 305 HPLC spectrum of **3x** and **8x**

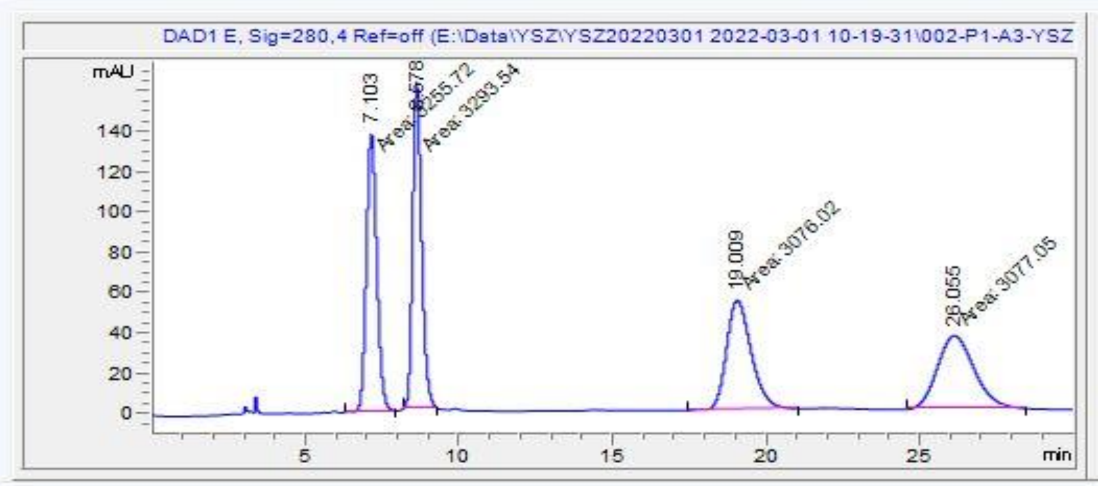

#	Time	Type	Area	Height	Width	Area%	Symmetry
1	7.103	MM	3255.7	138.7	0.3912	25.631	0.865
2	8.578	MM	3293.5	163.4	0.3359	25.929	0.766
3	19.009	MM	3076	54.7	0.9377	24.216	0.763
4	26.055	MM	3077	36.6	1.4	24.224	0.805

Supplementary Fig. 306 Full HPLC spectrum of racemic **3x** and **8x**

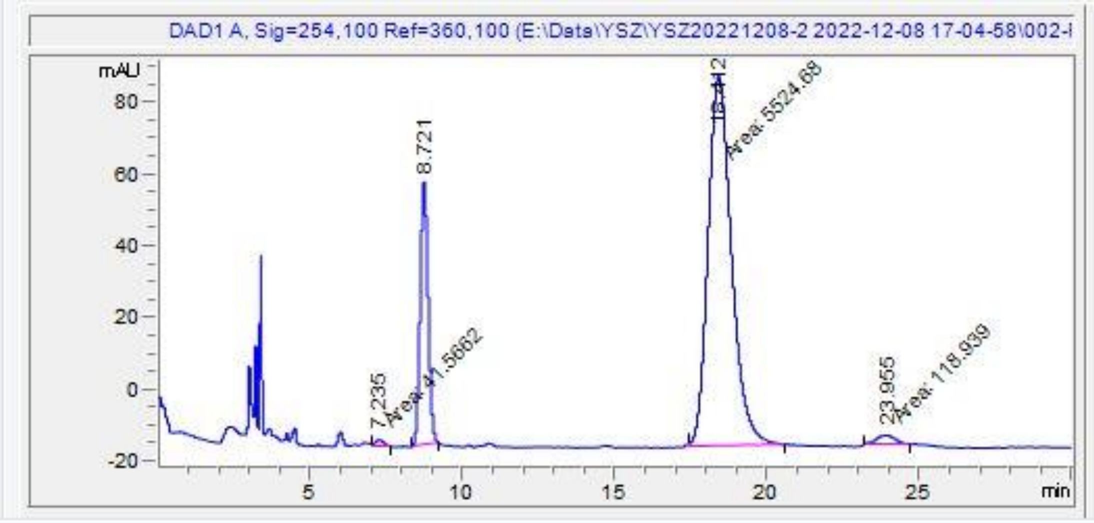

#	Time	Type	Area	Height	Width	Area%	Symmetry
1	7.235	MM	41.6	2.1	0.3372	0.593	0.715
2	8.721	VV R	1323.8	73	0.2129	18.888	0.952
3	18.412	MM	5524.7	102.6	0.8973	78.822	0.809
4	23.955	MM	118.9	2.7	0.7365	1.697	1.411

Supplementary Fig. 307 Full HPLC spectrum of **3x** and **8x**

Kinetic resolution of **1x** under standard conditions

Monitoring the ee value at different reaction time: 2 h

(R_p)-Tert-butyl (4^2-phenyl-1,4(1,4)-dibenzenacyclohexaphane-1^2-yl)carbamate **(1x)**

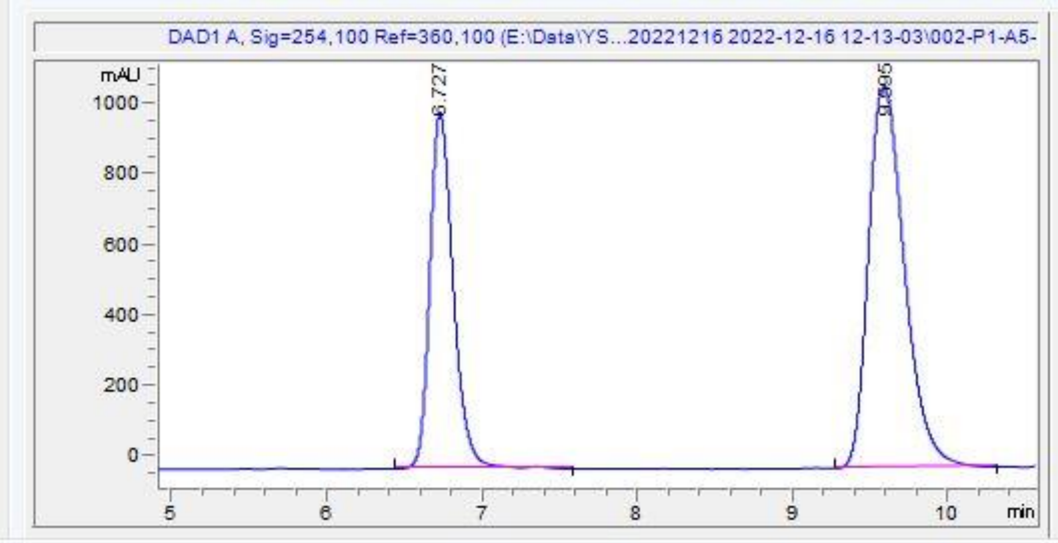

#	Time	Type	Area	Height	Width	Area%	Symmetry
1	6.727	BV R	10684.6	1011.3	0.1258	37.750	0.722
2	9.595	BV R	17619.3	1088.8	0.1903	62.250	0.708

Supplementary Fig. 308 HPLC spectrum of racemic **1x** at 2 h

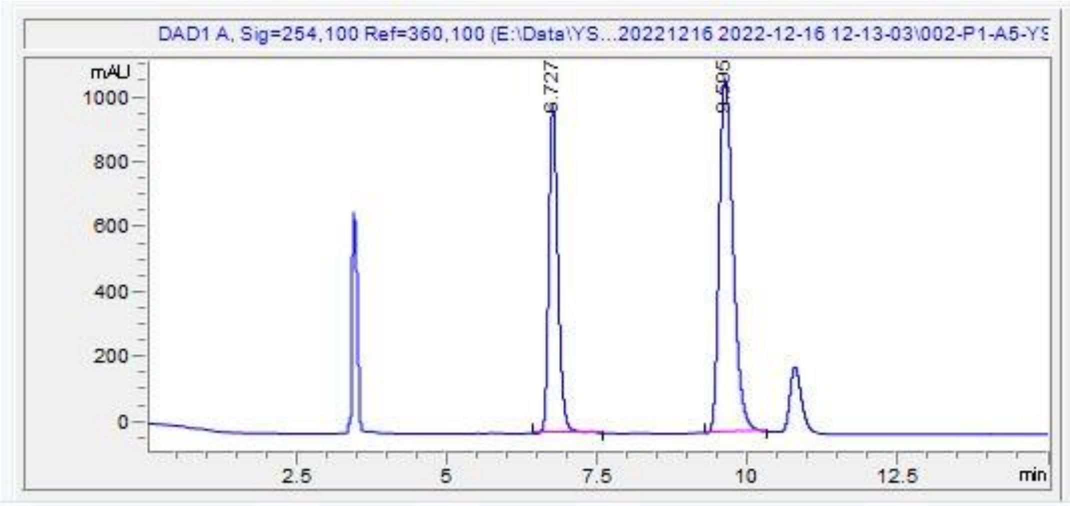

#	Time	Type	Area	Height	Width	Area%	Symmetry
1	6.727	BV R	10684.6	1011.3	0.1258	37.750	0.722
2	9.595	BV R	17619.3	1088.8	0.1903	62.250	0.708

Supplementary Fig. 309 HPLC spectrum of (R_p)**-1x** at 2 h

(S_p)-Dibenzyl-1-(1[5]-((tert-butoxycarbonyl)amino)-4[3]-phenyl-1,4(1,4)-dibenzenacyclo hexaphane-1[2]-yl)hydrazine-1,2-dicarboxylate **(3x)** and 2,3-dibenzyl 1-(tert-butyl) 1-(4[2]-phenyl-1,4(1,4)-dibenzenacyclohexaphane-1[2]-yl)triazane-1,2,3-tricarboxylate **(8x)**

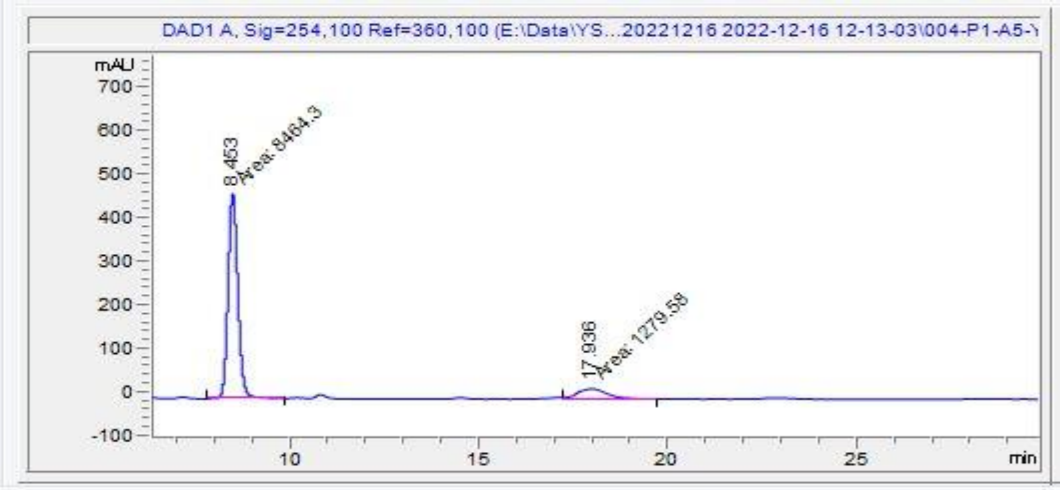

#	Time	Type	Area	Height	Width	Area%	Symmetry
1	8.453	MM	8464.3	470.1	0.3001	86.868	0.825
2	17.936	FM	1279.6	23.6	0.904	13.132	0.881

Supplementary Fig. 310 HPLC spectrum of **3x** and **8x** at 2 h

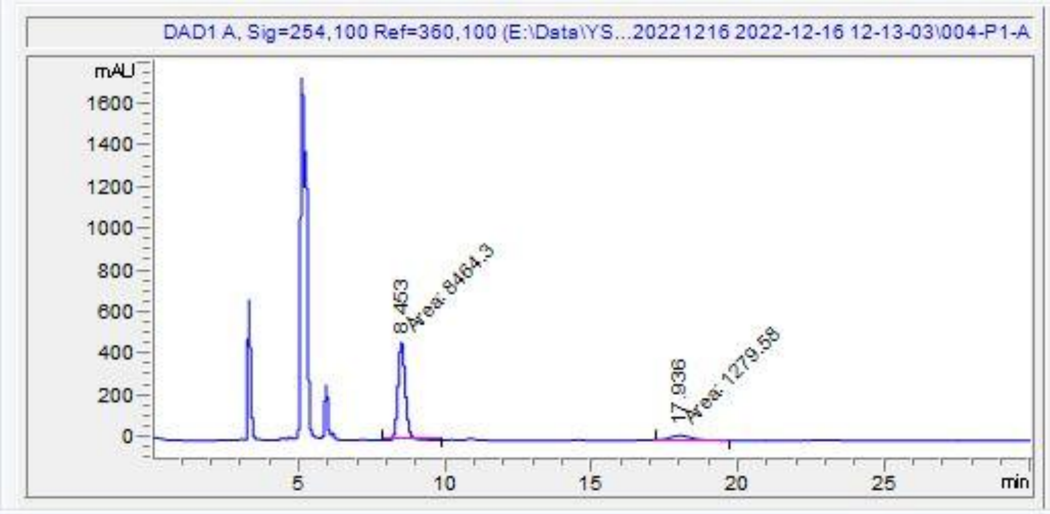

#	Time	Type	Area	Height	Width	Area%	Symmetry
1	8.453	MM	8464.3	470.1	0.3001	86.868	0.825
2	17.936	FM	1279.6	23.6	0.904	13.132	0.881

Supplementary Fig. 311 Full HPLC spectrum of **3x** and **8x** at 2 h

Monitoring the ee value at different reaction time: 5 h

(R_p)-Tert-butyl (4^2-phenyl-1,4(1,4)-dibenzenacyclohexaphane-1^2-yl)carbamate **(1x)**

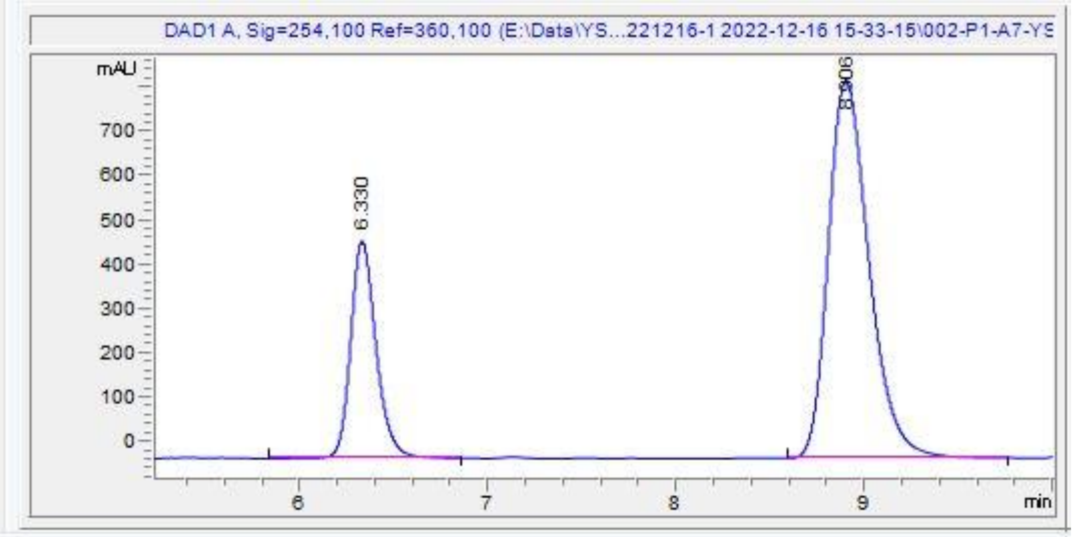

#	Time	Type	Area	Height	Width	Area%	Symmetry
1	6.33	VV R	4632.7	493.2	0.1281	26.376	0.794
2	8.906	BV R	12931.3	859.6	0.1775	73.624	0.734

Supplementary Fig. 312 HPLC spectrum of racemic **1x** at 5 h

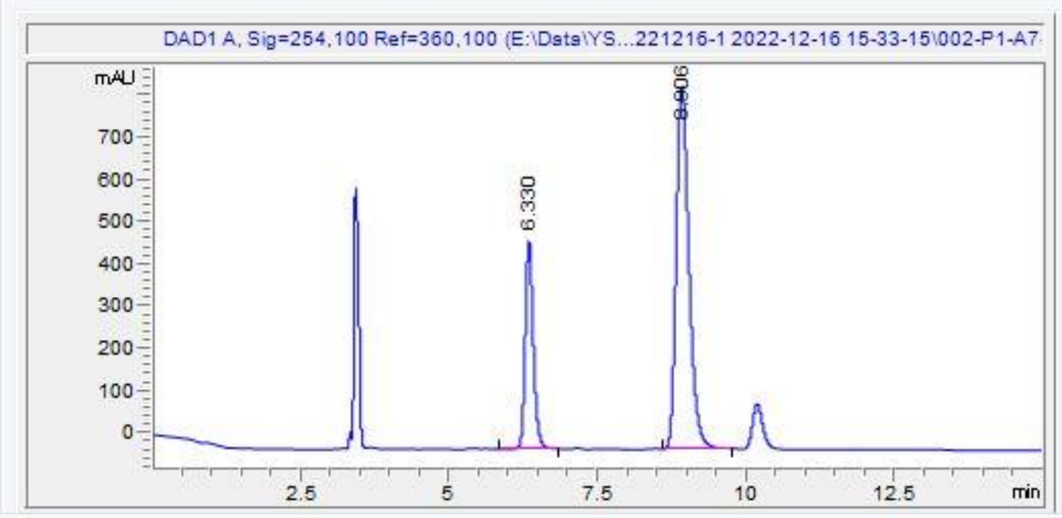

#	Time	Type	Area	Height	Width	Area%	Symmetry
1	6.33	VV R	4632.7	493.2	0.1281	26.376	0.794
2	8.906	BV R	12931.3	859.6	0.1775	73.624	0.734

Supplementary Fig. 313 Full HPLC spectrum of (R_p)-**1x** at 5 h

(S_p)-Dibenzyl-1-(1^5-((tert-butoxycarbonyl)amino)-4^3-phenyl-1,4(1,4)-dibenzenacyclo hexaphane-1^2-yl)hydrazine-1,2-dicarboxylate **(3x)** and 2,3-dibenzyl 1-(tert-butyl) 1-(4^2-phenyl-1,4(1,4)-dibenzenacyclohexaphane-1^2-yl)triazane-1,2,3-tricarboxylate **(8x)**

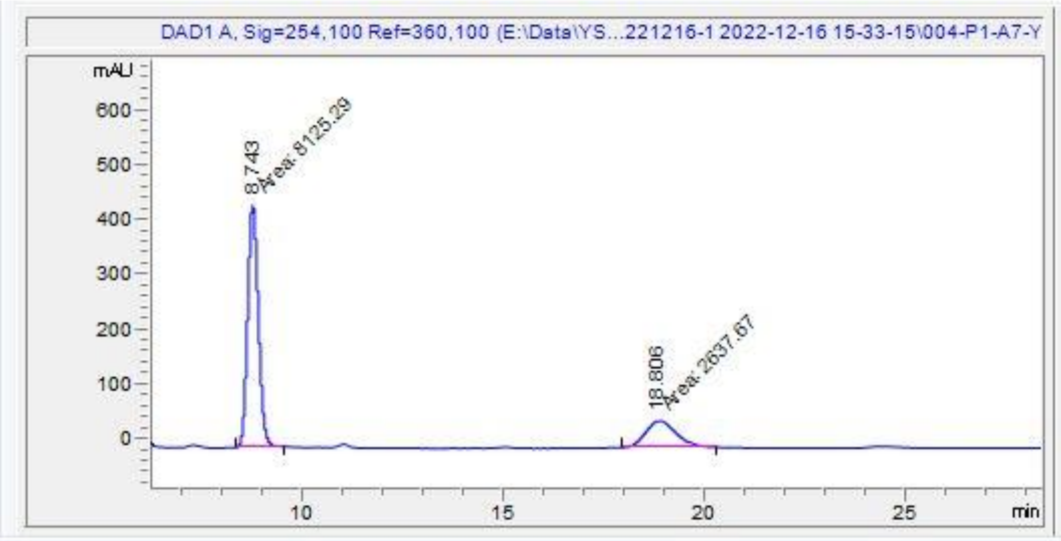

#	Time	Type	Area	Height	Width	Area%	Symmetry
1	8.743	MM	8125.3	442.4	0.3061	75.493	0.901
2	18.806	MM	2637.7	49.1	0.8947	24.507	0.766

Supplementary Fig. 314 HPLC spectrum of **3x** and **8x** at 5 h

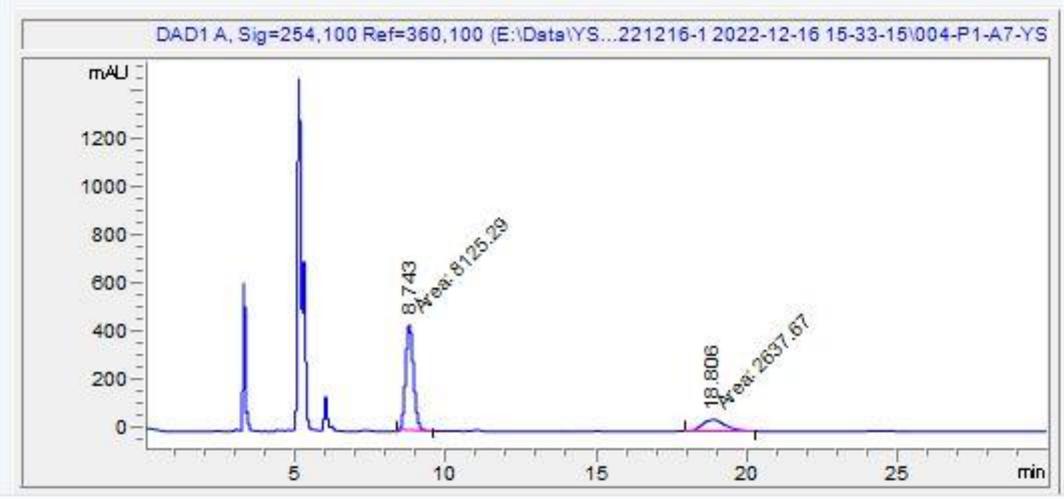

#	Time	Type	Area	Height	Width	Area%	Symmetry
1	8.743	MM	8125.3	442.4	0.3061	75.493	0.901
2	18.806	MM	2637.7	49.1	0.8947	24.507	0.766

Supplementary Fig. 315 Full HPLC spectrum of **3x** and **8x** at 5 h

Monitoring the ee value at different reaction time: 8 h

(R_p)-Tert-butyl (4[2]-phenyl-1,4(1,4)-dibenzenacyclohexaphane-1[2]-yl)carbamate **(1x)**

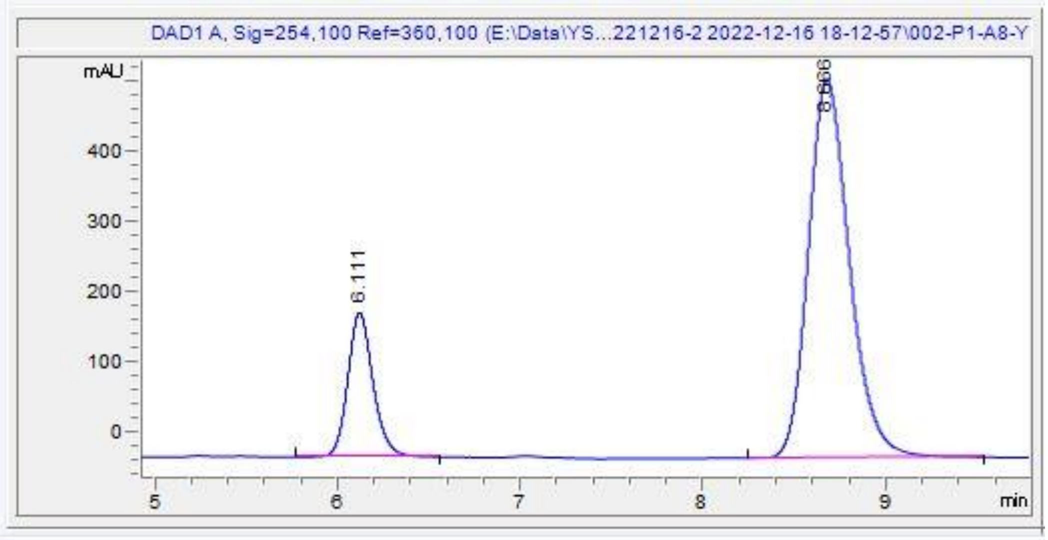

#	Time	Type	Area	Height	Width	Area%	Symmetry
1	6.111	VV R	1971.5	205	0.1432	19.038	0.829
2	8.666	VV R	8384.3	536.7	0.1843	80.962	0.781

Supplementary Fig. 316 HPLC spectrum of racemic **1x** at 8 h

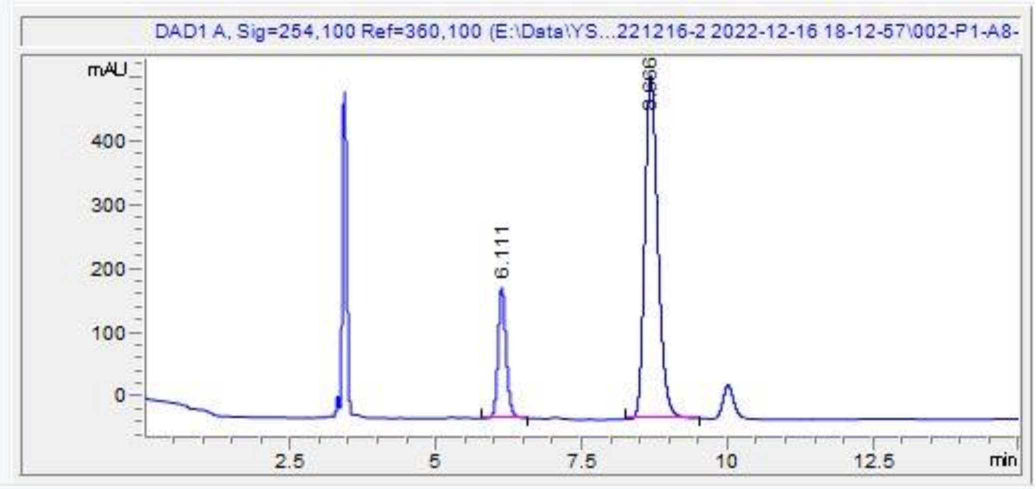

#	Time	Type	Area	Height	Width	Area%	Symmetry
1	6.111	VV R	1971.5	205	0.1432	19.038	0.829
2	8.666	VV R	8384.3	536.7	0.1843	80.962	0.781

Supplementary Fig. 317 Full HPLC spectrum of (R_p)**-1x** at 8 h

(S_p)-Dibenzyl-1-(1^5-((tert-butoxycarbonyl)amino)-4^3-phenyl-1,4(1,4)-dibenzenacyclo hexaphane-1^2-yl)hydrazine-1,2-dicarboxylate **(3x)** and 2,3-dibenzyl 1-(tert-butyl) 1-(4^2-phenyl-1,4(1,4)-dibenzenacyclohexaphane-1^2-yl)triazane-1,2,3-tricarboxylate **(8x)**

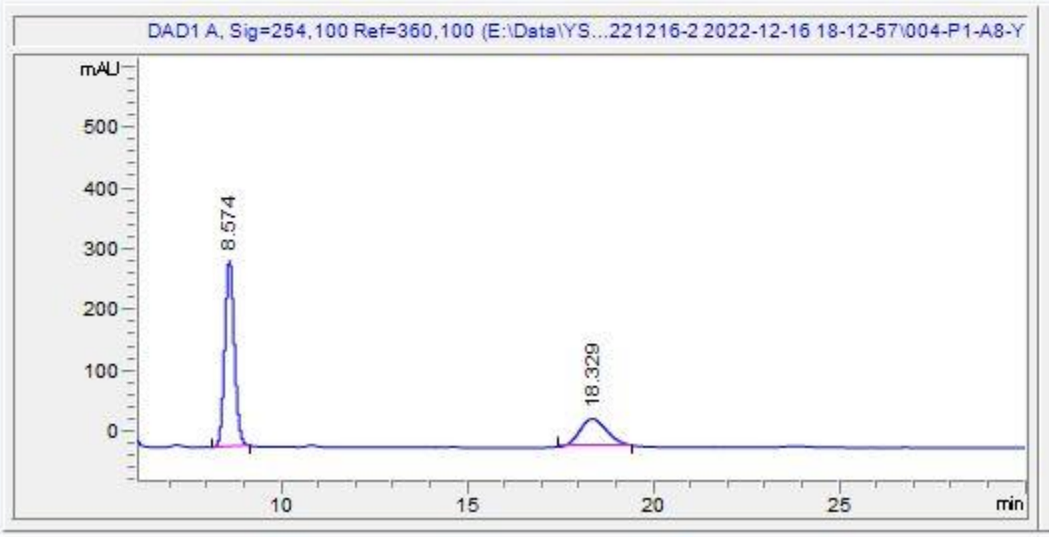

#	Time	Type	Area	Height	Width	Area%	Symmetry
1	8.574	VV R	5577.3	306.4	0.2144	70.751	0.947
2	18.329	VV R	2305.8	45.1	0.5985	29.249	0.852

Supplementary Fig. 318 HPLC spectrum of **3x** and **8x** at 8 h

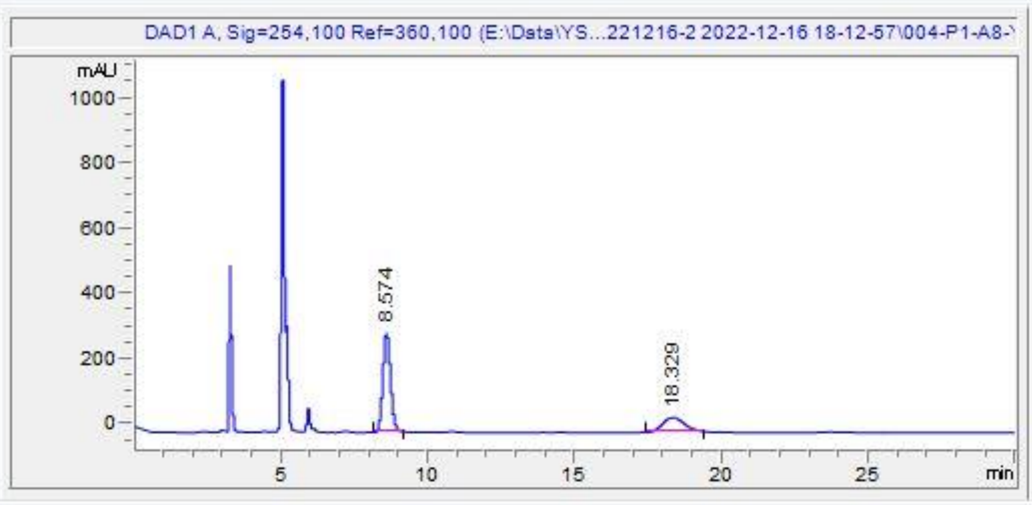

#	Time	Type	Area	Height	Width	Area%	Symmetry
1	8.574	VV R	5577.3	306.4	0.2144	70.751	0.947
2	18.329	VV R	2305.8	45.1	0.5985	29.249	0.852

Supplementary Fig. 319 Full HPLC spectrum of **3x** and **8x** at 8 h

Monitoring the ee value at different reaction time: 11 h

(R_p)-Tert-butyl (4^2-phenyl-1,4(1,4)-dibenzenacyclohexaphane-1^2-yl)carbamate **(1x)**

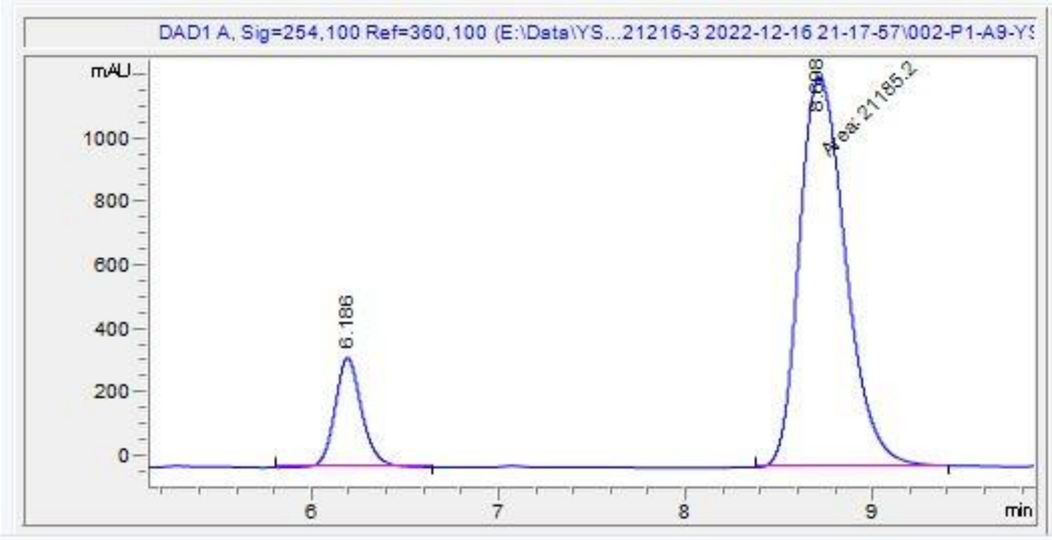

#	Time	Type	Area	Height	Width	Area%	Symmetry
1	6.186	BV R	3467.1	346.4	0.1463	14.064	0.831
2	8.698	MM	21185.2	1234.6	0.286	85.936	0.64

Supplementary Fig. 320 HPLC spectrum of racemic **1x** at 11 h

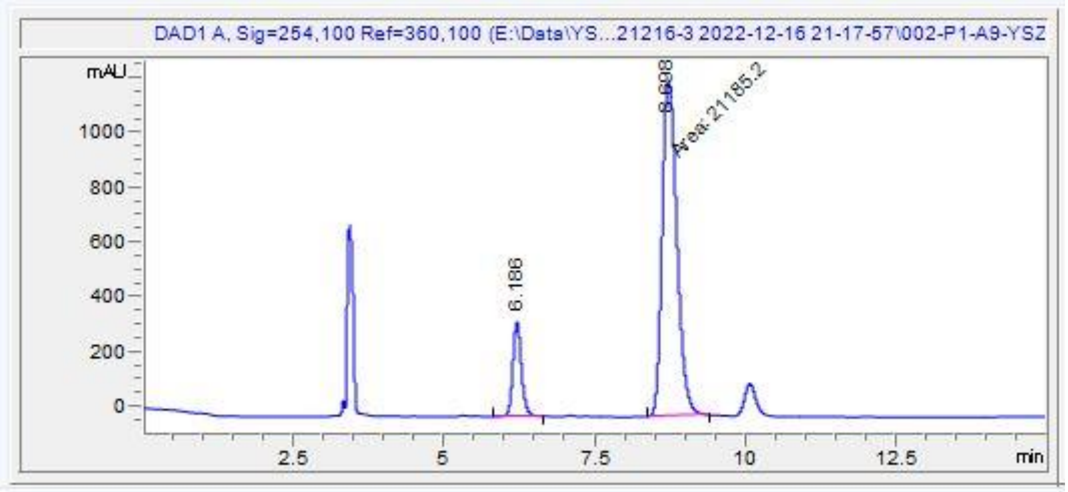

#	Time	Type	Area	Height	Width	Area%	Symmetry
1	6.186	BV R	3467.1	346.4	0.1463	14.064	0.831
2	8.698	MM	21185.2	1234.6	0.286	85.936	0.64

Supplementary Fig. 321 Full HPLC spectrum of (R_p)-**1x** at 11 h

(S_p)-Dibenzyl-1-(1^5-((tert-butoxycarbonyl)amino)-4^3-phenyl-1,4(1,4)-dibenzenacyclo hexaphane-1^2-yl)hydrazine-1,2-dicarboxylate **(3x)** and 2,3-dibenzyl 1-(tert-butyl) 1-(4^2-phenyl-1,4(1,4)-dibenzenacyclohexaphane-1^2-yl)triazane-1,2,3-tricarboxylate **(8x)**

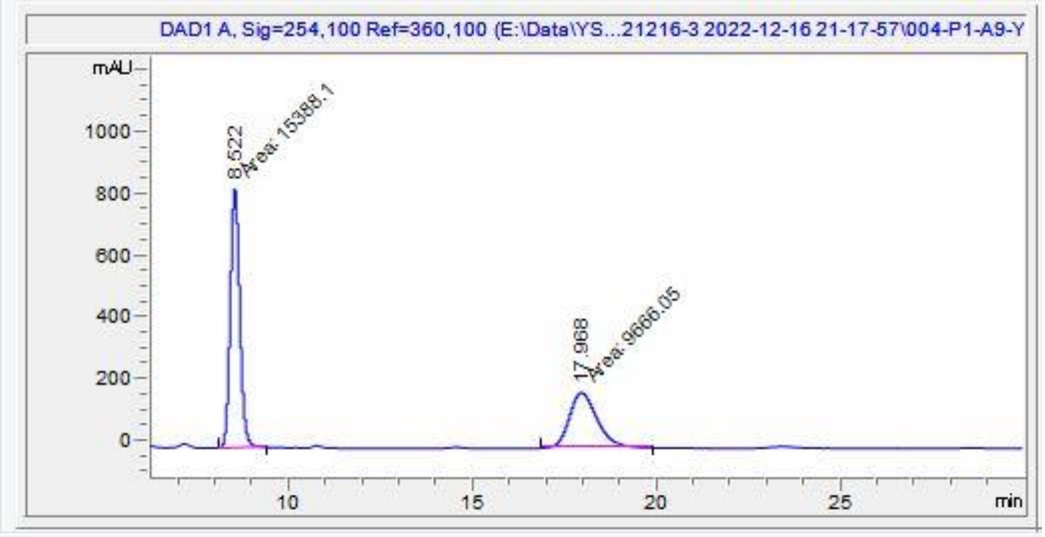

#	Time	Type	Area	Height	Width	Area%	Symmetry
1	8.522	MM	15388.1	842.4	0.3044	61.419	0.887
2	17.968	MM	9666	179.7	0.8967	38.581	0.684

Supplementary Fig. 322 HPLC spectrum of **3x** and **8x** at 11 h

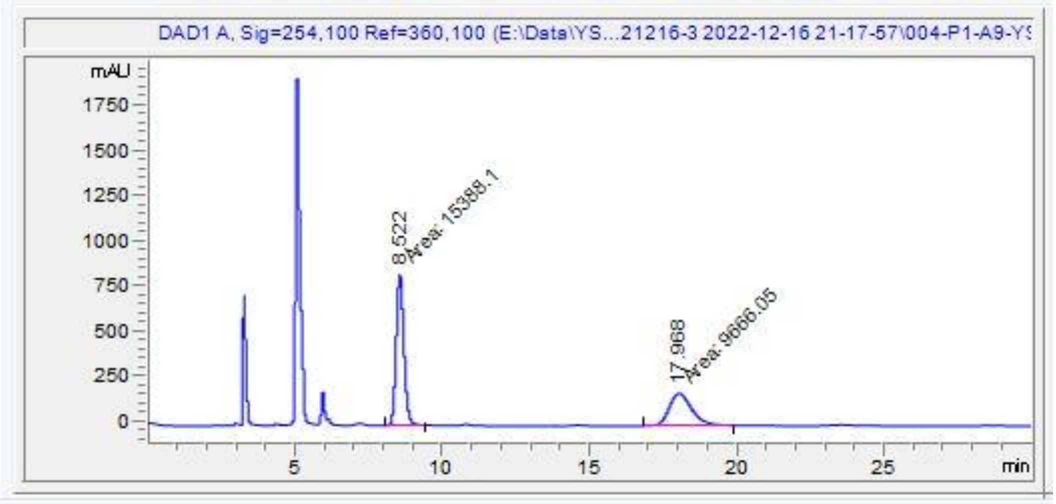

#	Time	Type	Area	Height	Width	Area%	Symmetry
1	8.522	MM	15388.1	842.4	0.3044	61.419	0.887
2	17.968	MM	9666	179.7	0.8967	38.581	0.684

Supplementary Fig. 323 Full HPLC spectrum of **3x** and **8x** at 11 h

Monitoring the ee value at different reaction time: 25 h

(R_p)-Tert-butyl (4^2-phenyl-1,4(1,4)-dibenzenacyclohexaphane-1^2-yl)carbamate **(1x)**

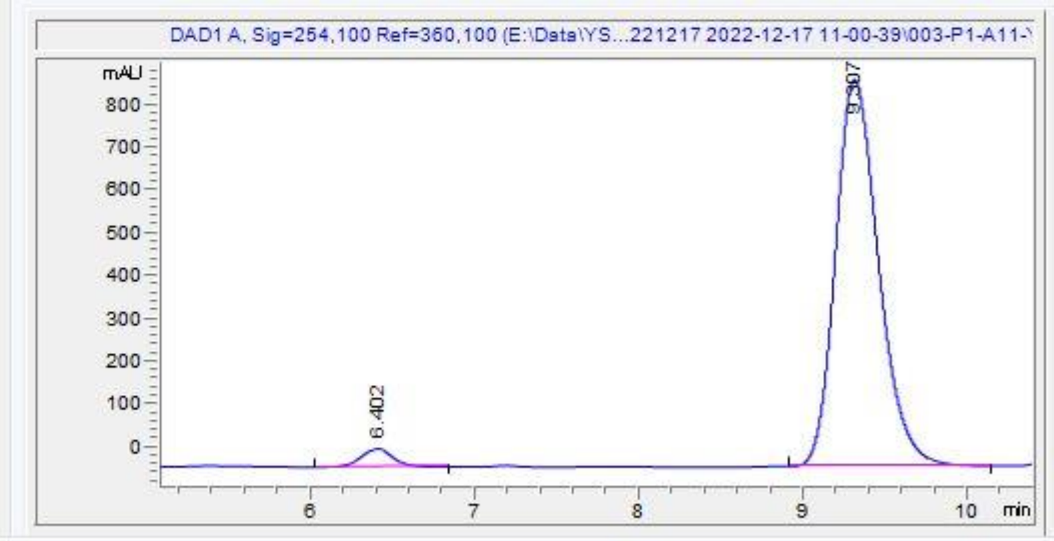

#	Time	Type	Area	Height	Width	Area%	Symmetry
1	6.402	VV R	582.1	42.8	0.167	3.422	1.032
2	9.307	BV R	16429.4	903.3	0.2137	96.578	0.719

Supplementary Fig. 324 HPLC spectrum of racemic **1x** at 25 h

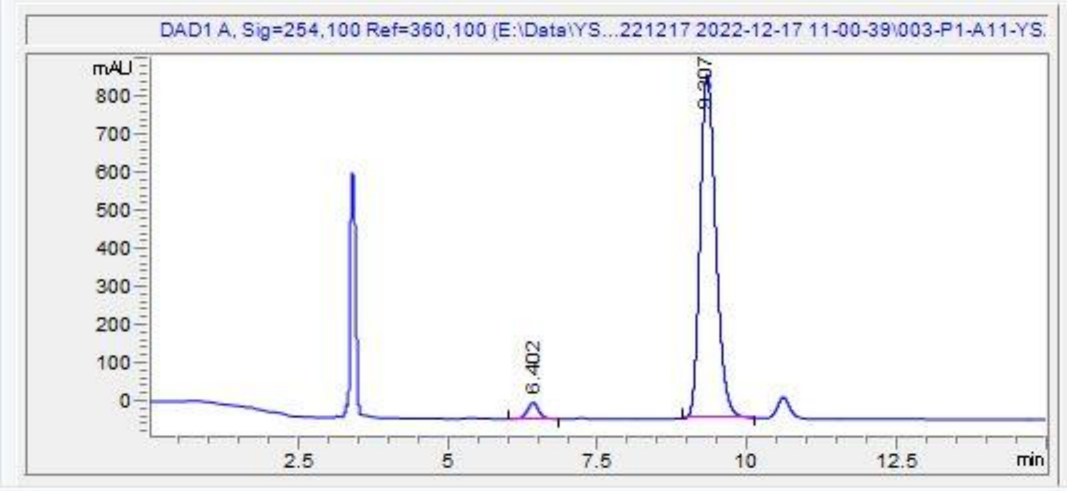

#	Time	Type	Area	Height	Width	Area%	Symmetry
1	6.402	VV R	582.1	42.8	0.167	3.422	1.032
2	9.307	BV R	16429.4	903.3	0.2137	96.578	0.719

Supplementary Fig. 325 Full HPLC spectrum of (R_p)**-1x** at 25 h

(S_p)-Dibenzyl-1-(1^5-((tert-butoxycarbonyl)amino)-4^3-phenyl-1,4(1,4)-dibenzenacyclo hexaphane-1^2-yl)hydrazine-1,2-dicarboxylate **(3x)** and 2,3-dibenzyl 1-(tert-butyl) 1-(4^2-phenyl-1,4(1,4)-dibenzenacyclohexaphane-1^2-yl)triazane-1,2,3-tricarboxylate **(8x)**

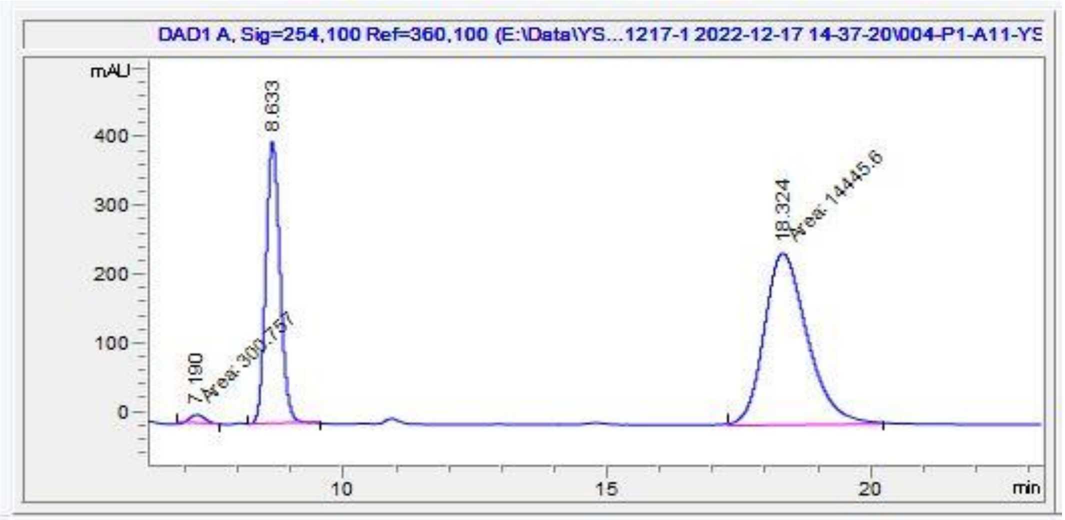

#	Time	Type	Area	Height	Width	Area%	Symmetry
1	7.19	MM	300.8	14.1	0.3564	1.333	0.72
2	8.633	BV R	7816.1	412.7	0.224	34.642	0.805
3	18.324	MM	14445.6	252.5	0.9536	64.025	0.695

Supplementary Fig. 326 HPLC spectrum of **3x** and **8x** at 25 h

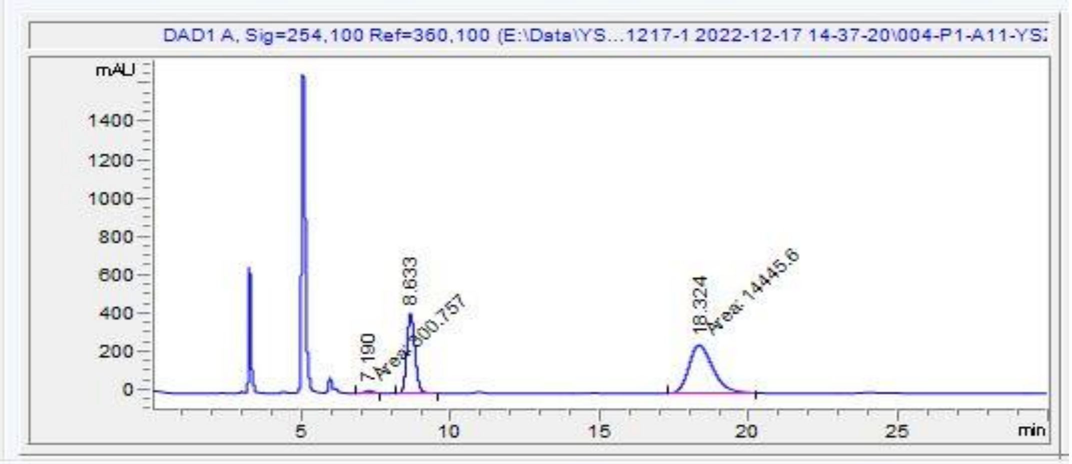

#	Time	Type	Area	Height	Width	Area%	Symmetry
1	7.19	MM	300.8	14.1	0.3564	1.333	0.72
2	8.633	BV R	7816.1	412.7	0.224	34.642	0.805
3	18.324	MM	14445.6	252.5	0.9536	64.025	0.695

Supplementary Fig. 327 Full HPLC spectrum of **3x** and **8x** at 25 h

Monitoring the ee value at different reaction time: 35 h

(R_p)-Tert-butyl (4^2-phenyl-1,4(1,4)-dibenzenacyclohexaphane-1^2-yl)carbamate **(1x)**

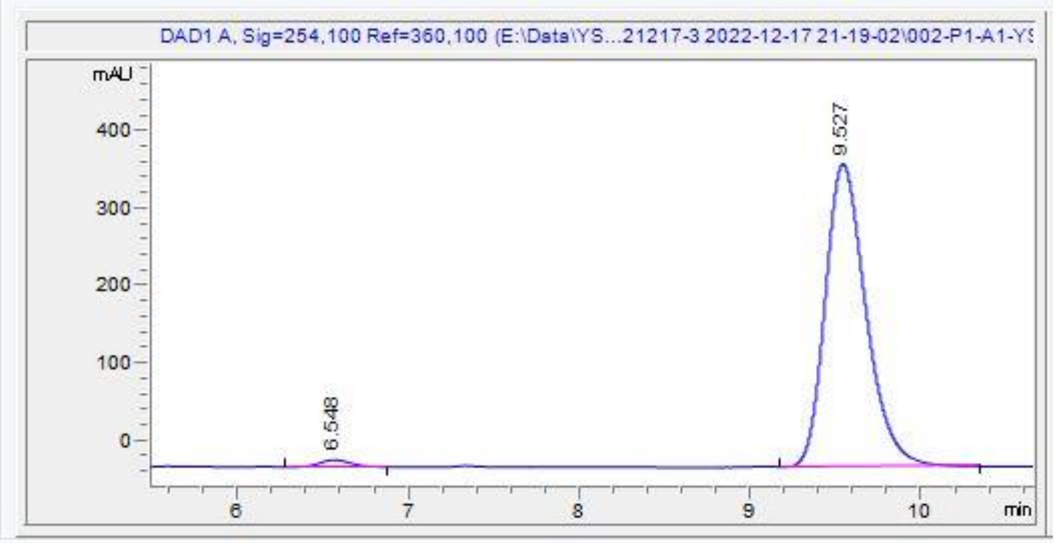

#	Time	Type	Area	Height	Width	Area%	Symmetry
1	6.548	VV R	115.8	9.1	0.1539	1.769	0.793
2	9.527	BV R	6431.1	389.5	0.2001	98.231	0.724

Supplementary Fig. 328 HPLC spectrum of racemic **1x** at 35 h

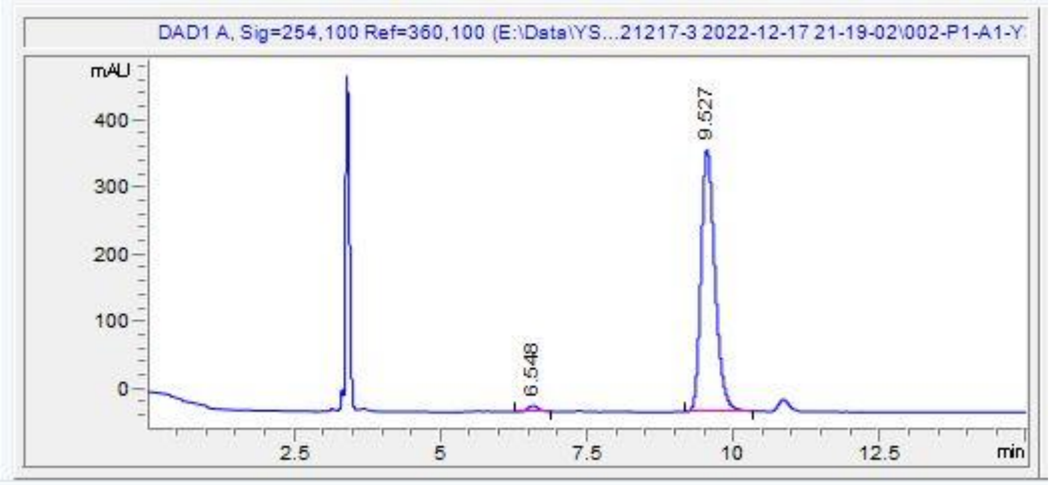

#	Time	Type	Area	Height	Width	Area%	Symmetry
1	6.548	VV R	115.8	9.1	0.1539	1.769	0.793
2	9.527	BV R	6431.1	389.5	0.2001	98.231	0.724

Supplementary Fig. 329 Full HPLC spectrum of (R_p)-**1x** at 35 h

(S_p)-Dibenzyl-1-(1^5^-((tert-butoxycarbonyl)amino)-4^3^-phenyl-1,4(1,4)-dibenzenacyclo hexaphane-1^2^-yl)hydrazine-1,2-dicarboxylate **(3x)** and 2,3-dibenzyl 1-(tert-butyl) 1-(4^2^-phenyl-1,4(1,4)-dibenzenacyclohexaphane-1^2^-yl)triazane-1,2,3-tricarboxylate **(8x)**

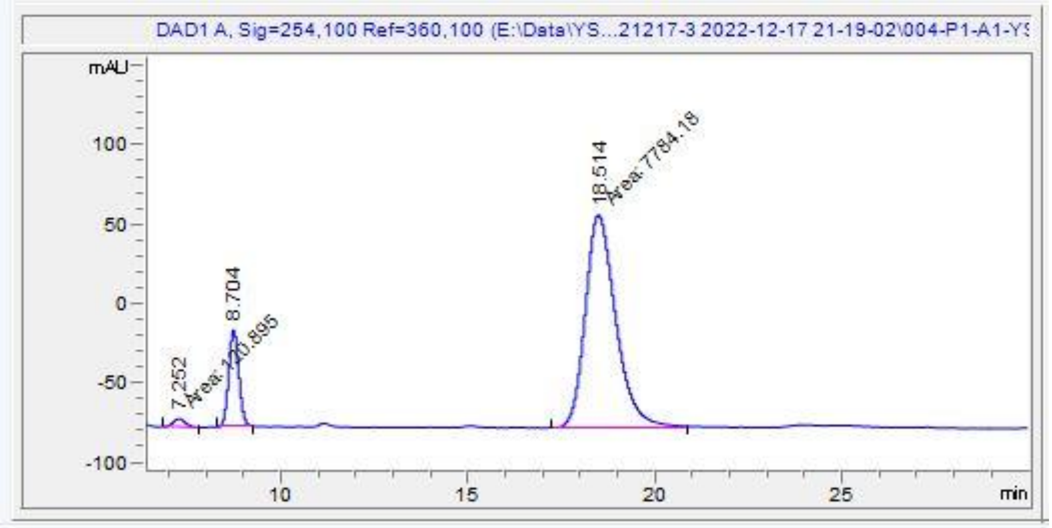

#	Time	Type	Area	Height	Width	Area%	Symmetry
1	7.252	MM	120.9	5.4	0.3765	1.334	0.917
2	8.704	VV R	1155.2	60.8	0.2231	12.750	0.883
3	18.514	MM	7784.2	134.3	0.9663	85.916	0.752

Supplementary Fig. 330 HPLC spectrum of **3x** and **8x** at 35 h

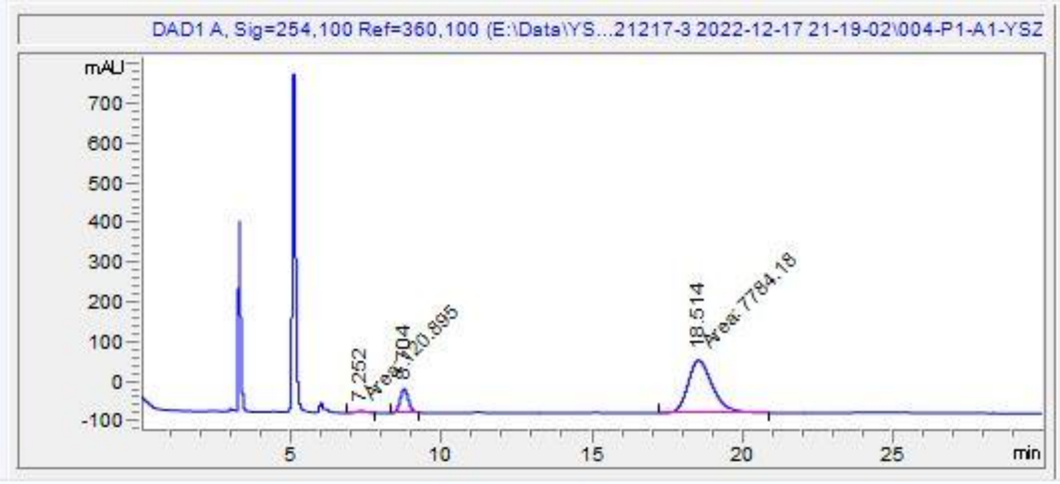

#	Time	Type	Area	Height	Width	Area%	Symmetry
1	7.252	MM	120.9	5.4	0.3765	1.334	0.917
2	8.704	VV R	1155.2	60.8	0.2231	12.750	0.883
3	18.514	MM	7784.2	134.3	0.9663	85.916	0.752

Supplementary Fig. 331 Full HPLC spectrum of **3x** and **8x** at 35 h

Monitoring the ee value at different reaction time: 49 h

(R_p)-Tert-butyl (4^2-phenyl-1,4(1,4)-dibenzenacyclohexaphane-1^2-yl)carbamate **(1x)**

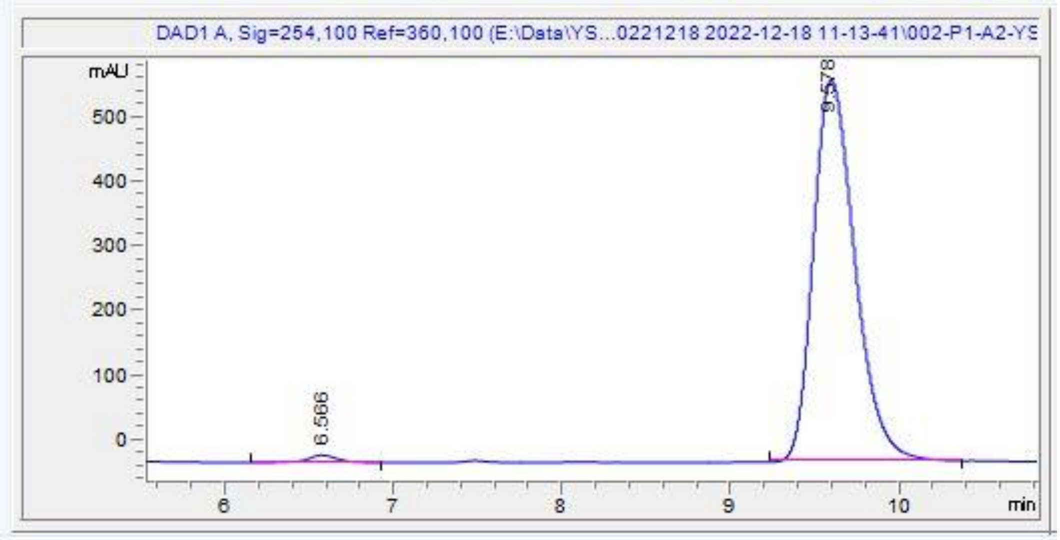

#	Time	Type	Area	Height	Width	Area%	Symmetry
1	6.566	VV R	145.3	11.6	0.1513	1.403	1.109
2	9.578	BV R	10208.7	594.3	0.2024	98.597	0.717

Supplementary Fig. 332 HPLC spectrum of racemic **1x** at 49 h

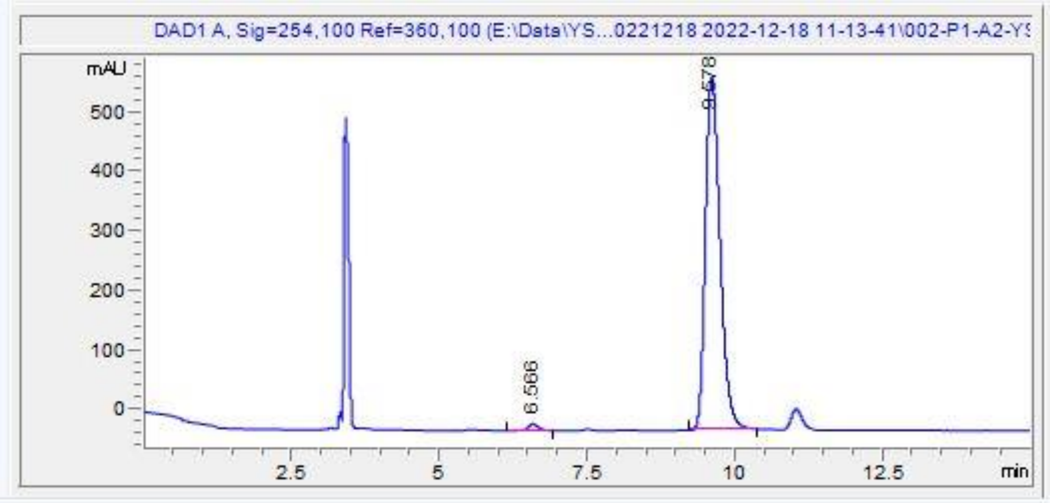

#	Time	Type	Area	Height	Width	Area%	Symmetry
1	6.566	VV R	145.3	11.6	0.1513	1.403	1.109
2	9.578	BV R	10208.7	594.3	0.2024	98.597	0.717

Supplementary Fig. 333 Full HPLC spectrum of (R_p)-**1x** at 49 h

(S_p)-Dibenzyl-1-(1^5-((tert-butoxycarbonyl)amino)-4^3-phenyl-1,4(1,4)-dibenzenacyclo hexaphane-1^2-yl)hydrazine-1,2-dicarboxylate **(3x)** and 2,3-dibenzyl 1-(tert-butyl) 1-(4^2-phenyl-1,4(1,4)-dibenzenacyclohexaphane-1^2-yl)triazane-1,2,3-tricarboxylate **(8x)**

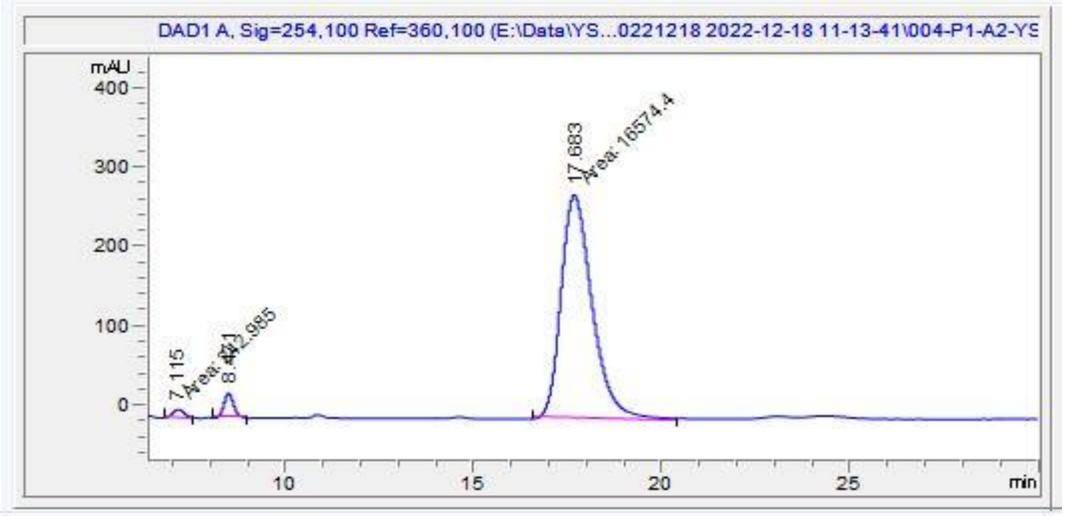

#	Time	Type	Area	Height	Width	Area%	Symmetry
1	7.115	MM	243	11.1	0.3661	1.397	0.76
2	8.471	VB R	575.5	31.5	0.2152	3.309	0.975
3	17.683	MM	16574.4	283.8	0.9732	95.294	0.708

Supplementary Fig. 334 HPLC spectrum of **3x** and **8x** at 49 h

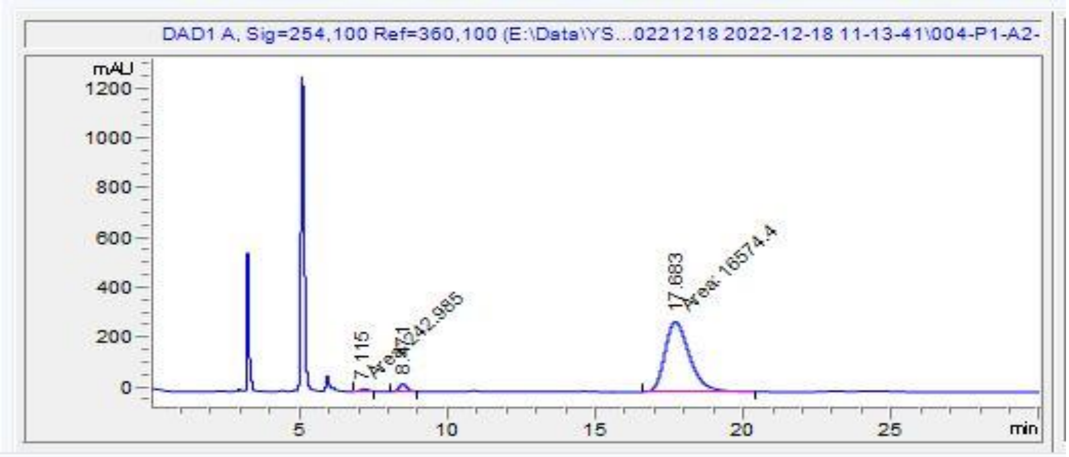

#	Time	Type	Area	Height	Width	Area%	Symmetry
1	7.115	MM	243	11.1	0.3661	1.397	0.76
2	8.471	VB R	575.5	31.5	0.2152	3.309	0.975
3	17.683	MM	16574.4	283.8	0.9732	95.294	0.708

Supplementary Fig. 335 Full HPLC spectrum of **3x** and **8x** at 49 h

Kinetic resolution of **8x**

2,3-dibenzyl 1-(tert-butyl) 1-(4^2-phenyl-1,4(1,4)-dibenzenacyclohexaphane-1^2-yl)triazane-1,2,3-tricarboxylate **(8x)**

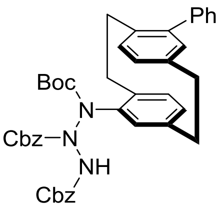

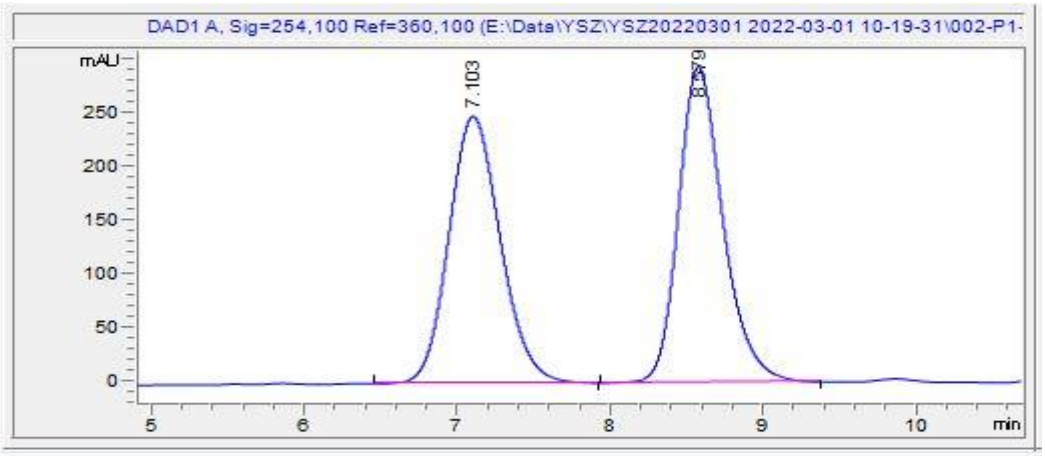

#	Time	Type	Area	Height	Width	Area%	Symmetry
1	7.103	BB	5838.6	248.7	0.3569	49.453	0.853
2	8.579	BB	5967.7	293.4	0.3083	50.547	0.764

Supplementary Fig. 336 HPLC spectrum of racemic **8x**

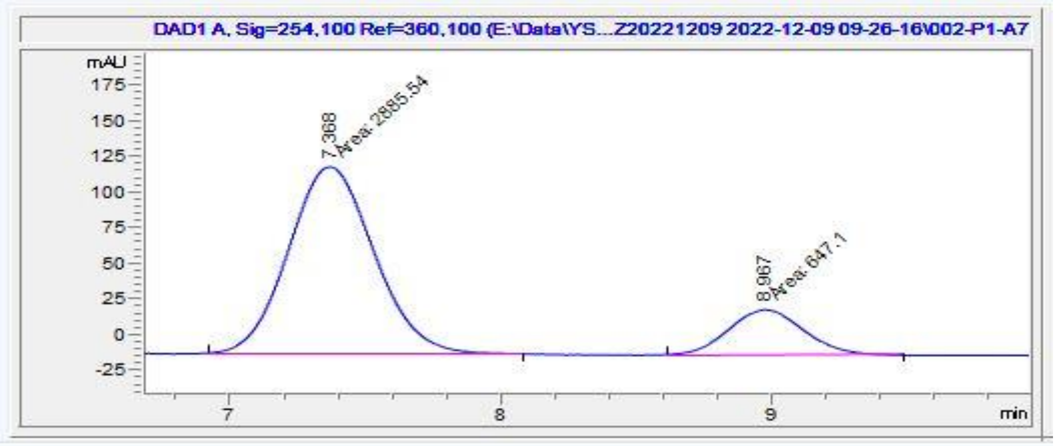

#	Time	Type	Area	Height	Width	Area%	Symmetry
1	7.368	MM	2885.5	130.9	0.3674	81.682	0.944
2	8.967	MM	647.1	32.4	0.3327	18.318	0.916

Supplementary Fig. 337 HPLC spectrum of (R_p)**-8x**

Dibenzyl-1-(1^5-((tert-butoxycarbonyl)amino)-4^3-phenyl-1,4(1,4)-dibenzenacyclohexaphane-1^2-yl)hydrazine-1,2-dicarboxylate **(3x)**

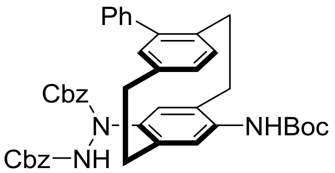

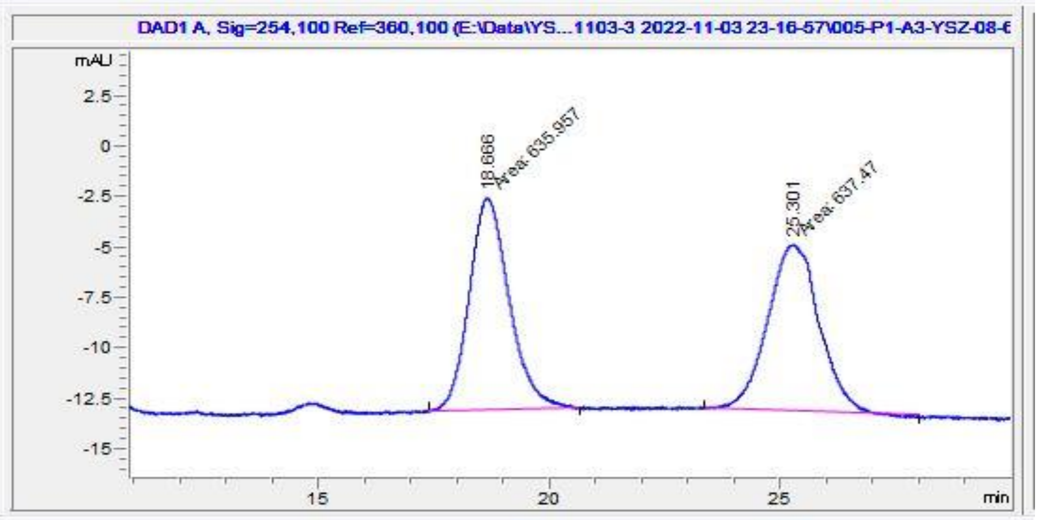

#	Time	Type	Area	Height	Width	Area%	Symmetry
1	18.666	MM	636	10.5	1.0101	49.941	0.827
2	25.301	MM	637.5	8.2	1.2904	50.059	0.97

Supplementary Fig. 338 HPLC spectrum of racemic **3x**

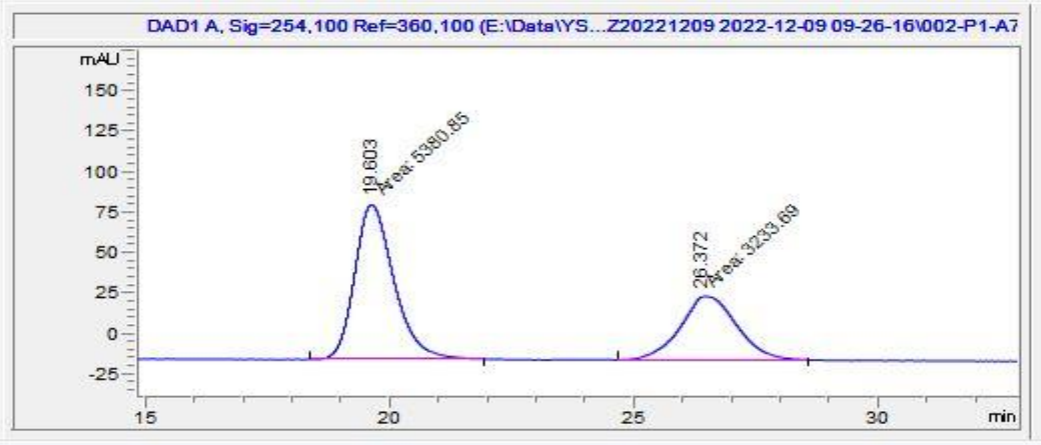

#	Time	Type	Area	Height	Width	Area%	Symmetry
1	19.603	MM	5380.9	95.9	0.9348	62.462	0.756
2	26.372	MM	3233.7	39.9	1.35	37.538	0.699

Supplementary Fig. 339 HPLC spectrum of (S_p)-**3x**

Reaction between **8x** and **1a** catalyzed by (*R)*-**A5** in standard condition

2,3-dibenzyl 1-(tert-butyl) 1-(4[2]-phenyl-1,4(1,4)-dibenzenacyclohexaphane-1[2]-yl)triazane-1,2,3-tricarboxylate **(8x)**

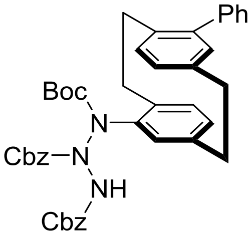

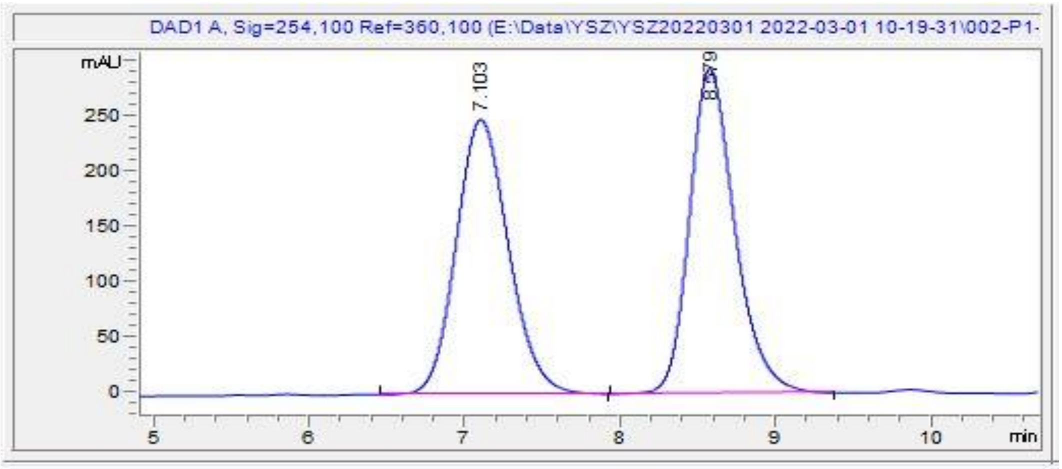

#	Time	Type	Area	Height	Width	Area%	Symmetry
1	7.103	BB	5838.6	248.7	0.3569	49.453	0.853
2	8.579	BB	5967.7	293.4	0.3083	50.547	0.764

Supplementary Fig. 340 HPLC spectrum of racemic **8x**

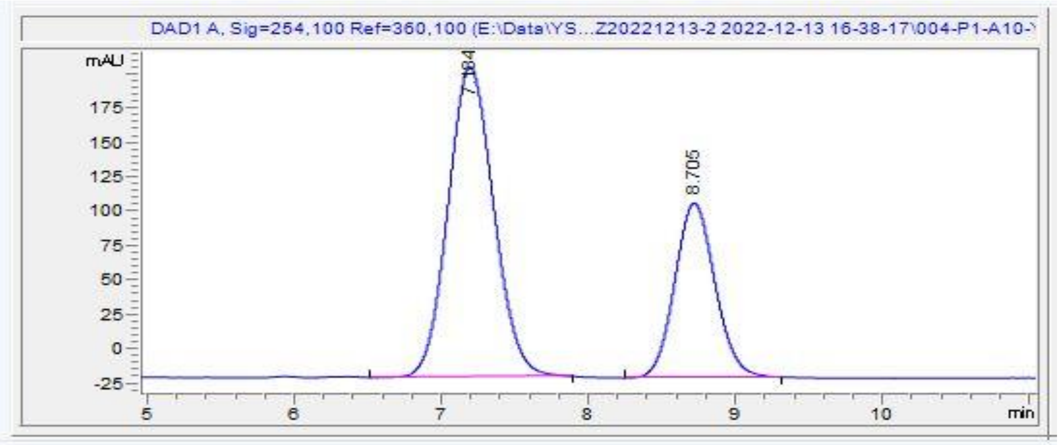

#	Time	Type	Area	Height	Width	Area%	Symmetry
1	7.184	VV R	4884.2	225.9	0.2543	66.859	0.909
2	8.705	VV R	2421.1	127.4	0.2257	33.141	0.895

Supplementary Fig. 341 HPLC spectrum of (R_p)-**8x**

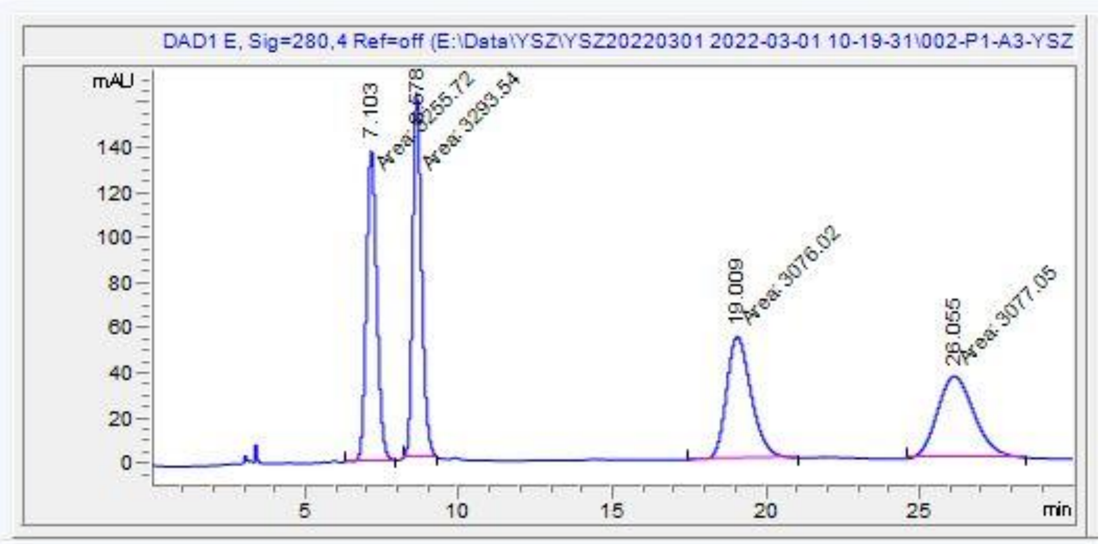

#	Time	Type	Area	Height	Width	Area%	Symmetry
1	7.103	MM	3255.7	138.7	0.3912	25.631	0.865
2	8.578	MM	3293.5	163.4	0.3359	25.929	0.766
3	19.009	MM	3076	54.7	0.9377	24.216	0.763
4	26.055	MM	3077	36.6	1.4	24.224	0.805

Supplementary Fig. 342 Full HPLC spectrum of racemic **8x**

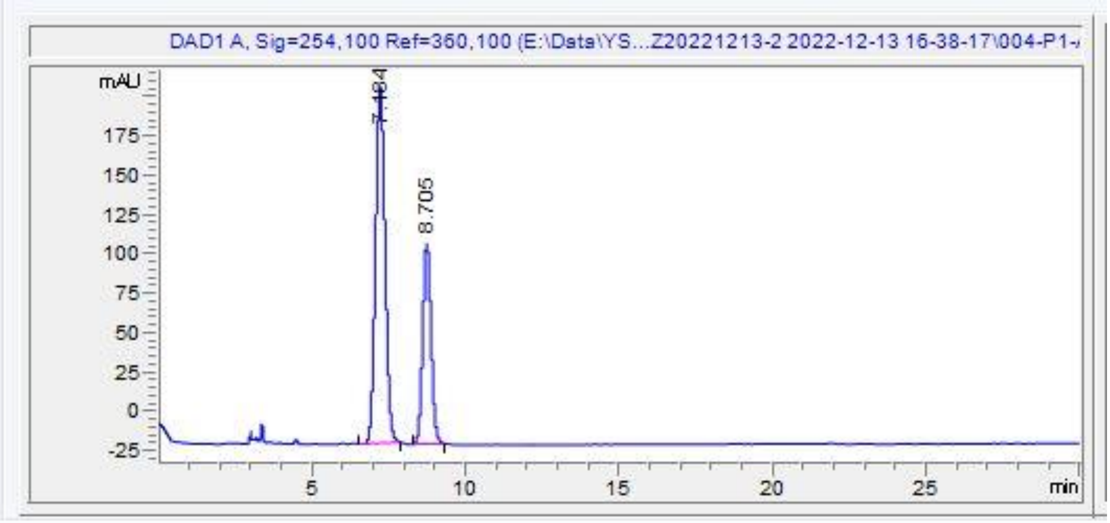

#	Time	Type	Area	Height	Width	Area%	Symmetry
1	7.184	VV R	4884.2	225.9	0.2543	66.859	0.909
2	8.705	VV R	2421.1	127.4	0.2257	33.141	0.895

Supplementary Fig. 343 Full HPLC spectrum of (R_p)-**8x**

Dibenzyl-1-(1^5-((tert-butoxycarbonyl)amino)-4^3-phenyl-1,4(1,4)-dibenzenacyclohexaphane-1^2-yl)hydrazine-1,2-dicarboxylate **(3x)**

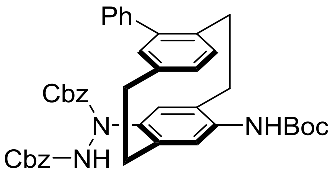

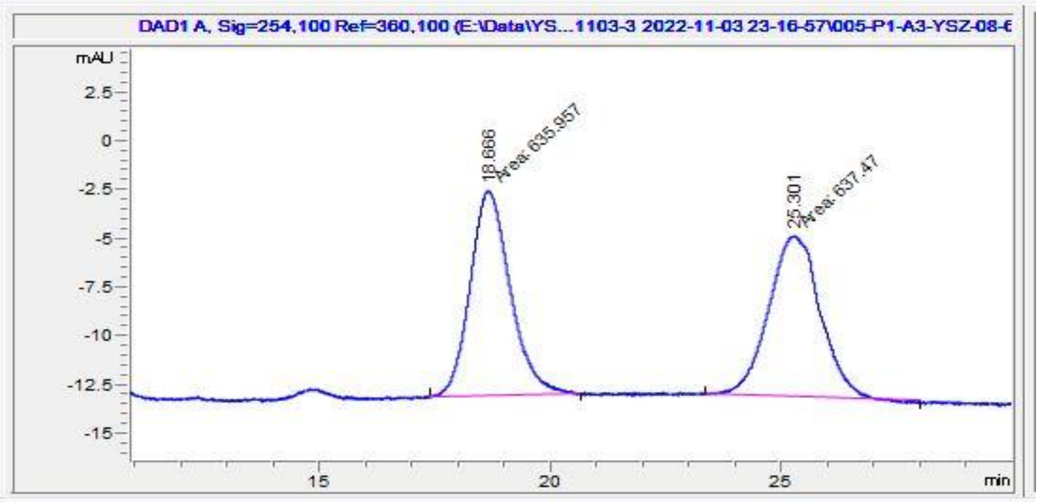

#	Time	Type	Area	Height	Width	Area%	Symmetry
1	18.666	MM	636	10.5	1.0101	49.941	0.827
2	25.301	MM	637.5	8.2	1.2904	50.059	0.97

Supplementary Fig. 344 HPLC spectrum of racemic **3x**

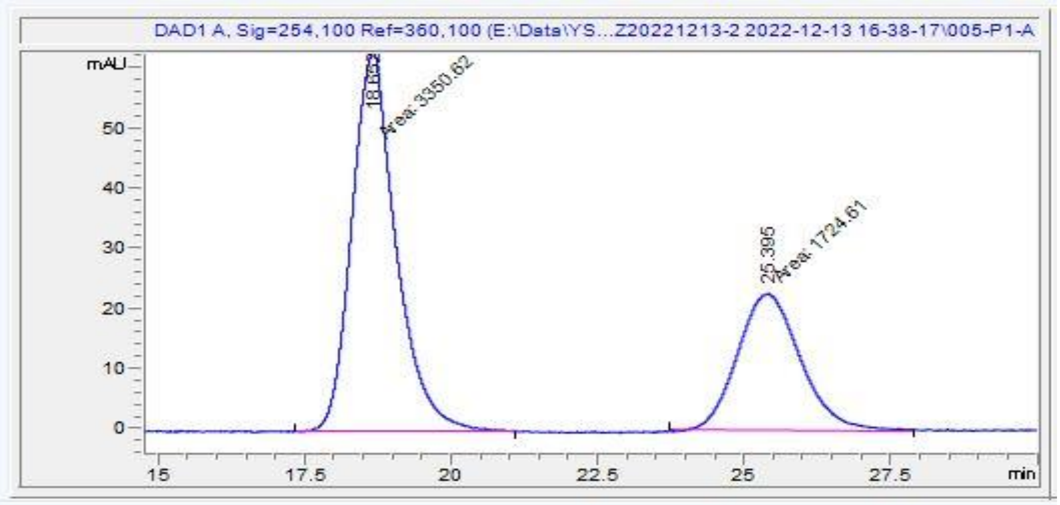

#	Time	Type	Area	Height	Width	Area%	Symmetry
1	18.652	MM	3350.6	63.5	0.8788	66.019	0.885
2	25.395	MM	1724.6	23	1.2523	33.981	0.95

Supplementary Fig. 345 HPLC spectrum of (S_p)-**3x**

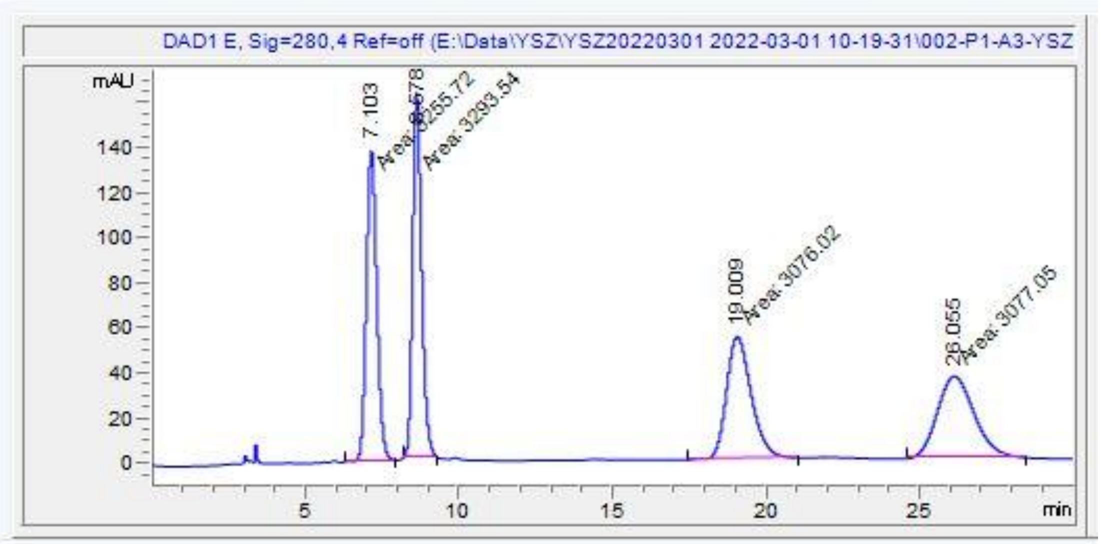

#	Time	Type	Area	Height	Width	Area%	Symmetry
1	7.103	MM	3255.7	138.7	0.3912	25.631	0.865
2	8.578	MM	3293.5	163.4	0.3359	25.929	0.766
3	19.009	MM	3076	54.7	0.9377	24.216	0.763
4	26.055	MM	3077	36.6	1.4	24.224	0.805

Supplementary Fig. 346 Full HPLC spectrum of racemic **3x**

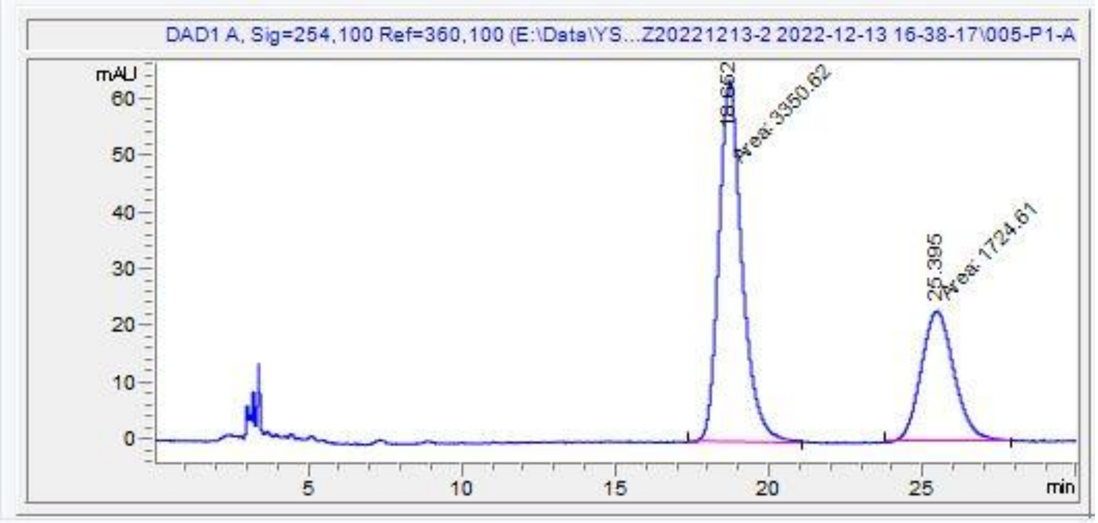

#	Time	Type	Area	Height	Width	Area%	Symmetry
1	18.652	MM	3350.6	63.5	0.8788	66.019	0.885
2	25.395	MM	1724.6	23	1.2523	33.981	0.95

Supplementary Fig. 347 Full HPLC spectrum of (S_p)-**3x**

Kinetic resolution of (S_p)-**8x** catalyzed by racemic CPA

Dibenzyl-1-(1^5-((tert-butoxycarbonyl)amino)-4^3-phenyl-1,4(1,4)-dibenzenacyclohexaphane-1^2-yl)hydrazine-1,2-dicarboxylate **(3x)**

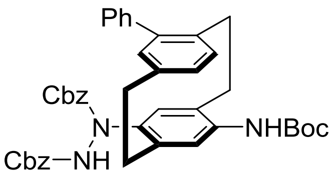

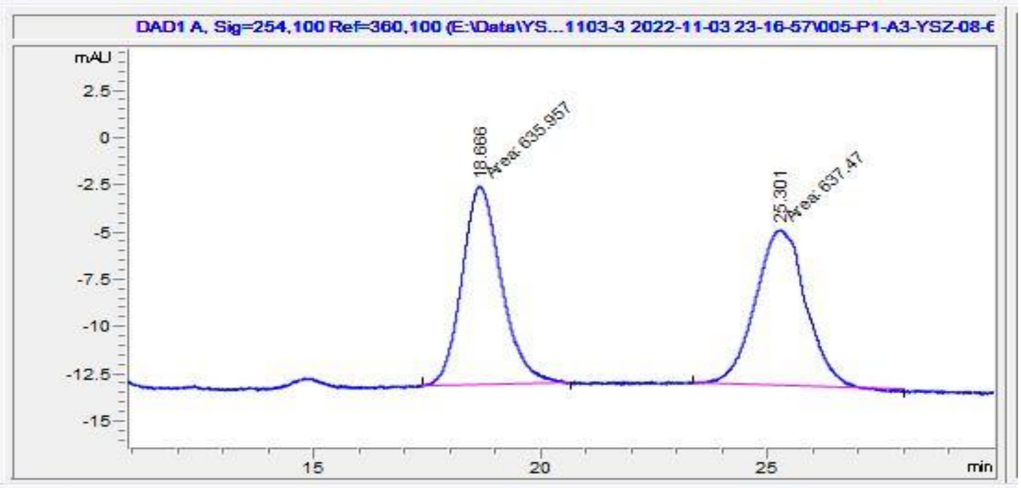

#	Time	Type	Area	Height	Width	Area%	Symmetry
1	18.666	MM	636	10.5	1.0101	49.941	0.827
2	25.301	MM	637.5	8.2	1.2904	50.059	0.97

Supplementary Fig. 348 HPLC spectrum of racemic **3x**

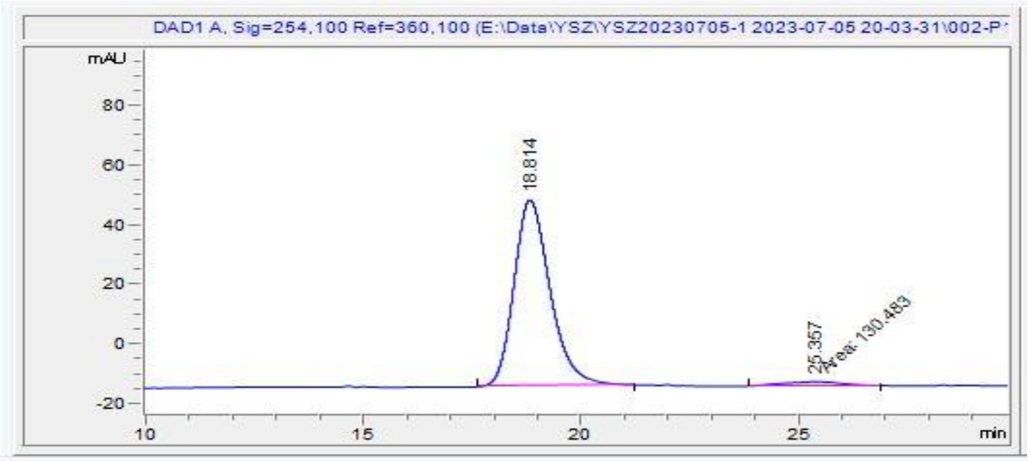

#	Time	Type	Area	Height	Width	Area%	Symmetry
1	18.814	BB	3606.3	62.4	0.6777	96.508	0.755
2	25.357	MM	130.5	1.4	1.5835	3.492	1.186

Supplementary Fig. 349 HPLC spectrum of (S_p)-**3x**

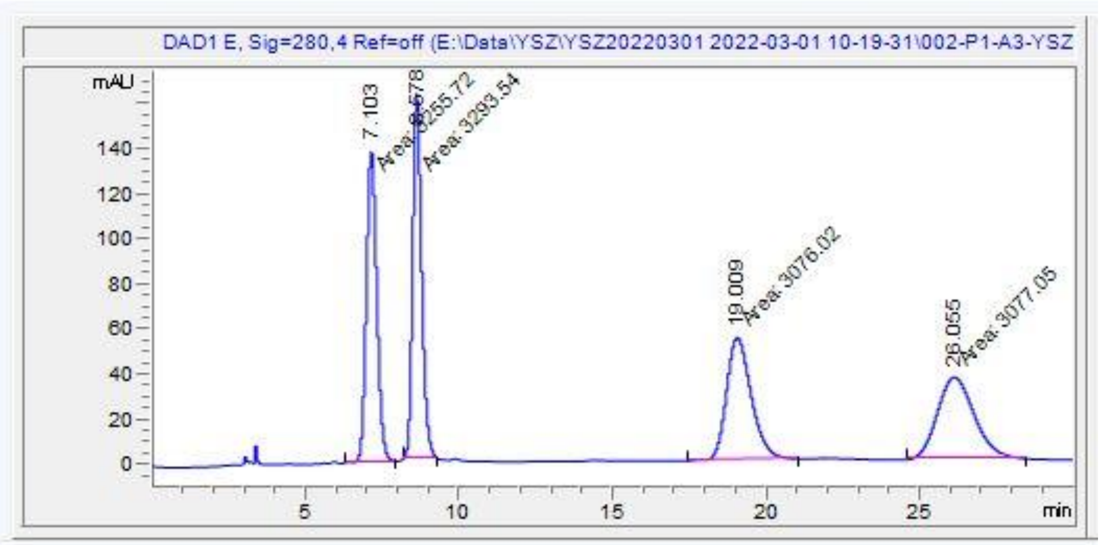

#	Time	Type	Area	Height	Width	Area%	Symmetry
1	7.103	MM	3255.7	138.7	0.3912	25.631	0.865
2	8.578	MM	3293.5	163.4	0.3359	25.929	0.766
3	19.009	MM	3076	54.7	0.9377	24.216	0.763
4	26.055	MM	3077	36.6	1.4	24.224	0.805

Supplementary Fig. 350 HPLC spectrum of racemic **3x**

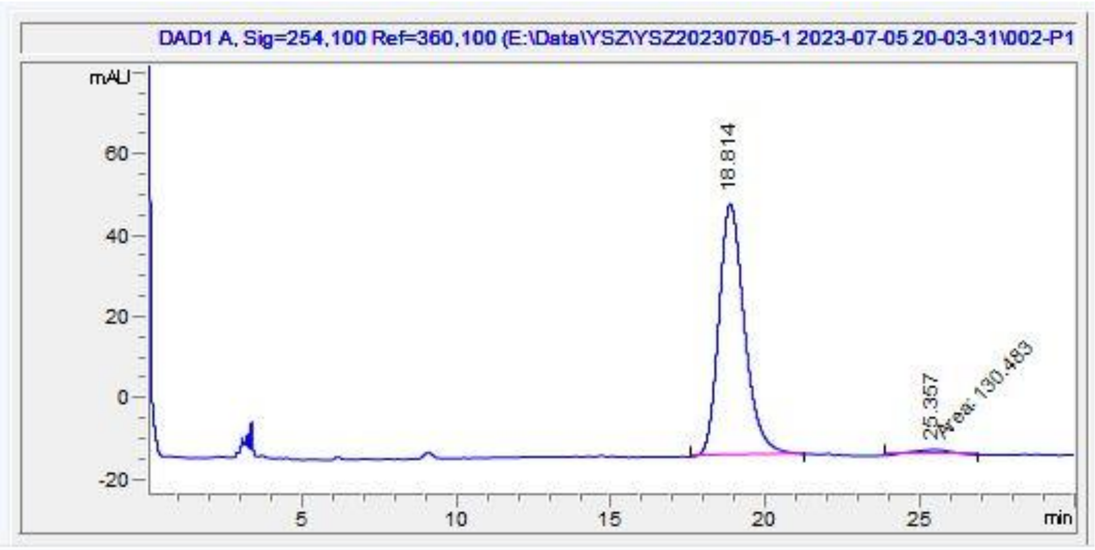

#	Time	Type	Area	Height	Width	Area%	Symmetry
1	18.814	BB	3606.3	62.4	0.6777	96.508	0.755
2	25.357	MM	130.5	1.4	1.5835	3.492	1.186

Supplementary Fig. 351 HPLC spectrum of (S_p)-**3x**

Large scale kinetic resolution of **1a**

(S_p)-Tert-butyl 1,4(1,4)-dibenzenacyclohexaphane-1^2-ylcarbamate **(1a)**

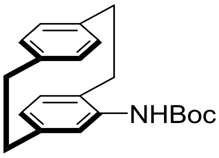

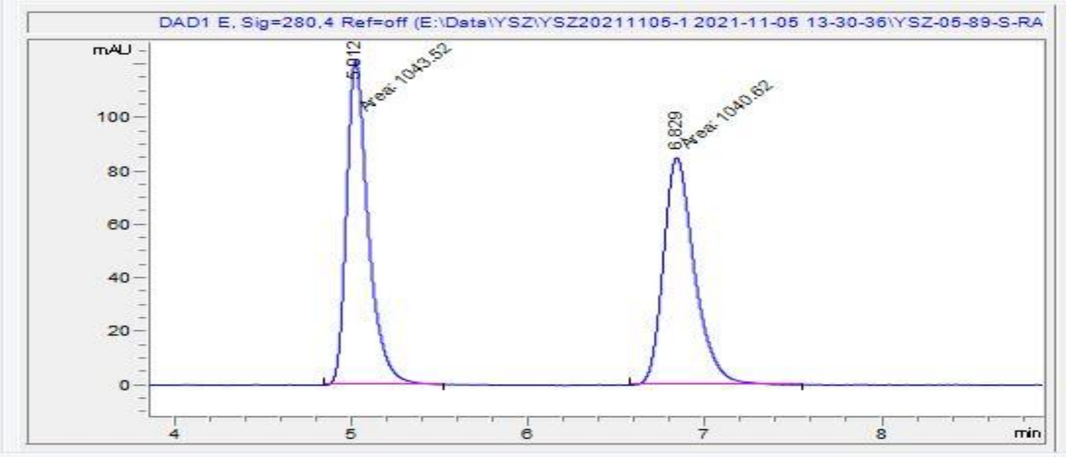

#	Time	Type	Area	Height	Width	Area%	Symmetry
1	5.012	MM	1043.5	121.6	0.143	50.069	0.67
2	6.829	MM	1040.6	85.3	0.2033	49.931	0.728

Supplementary Fig. 352 HPLC spectrum of racemic **1a**

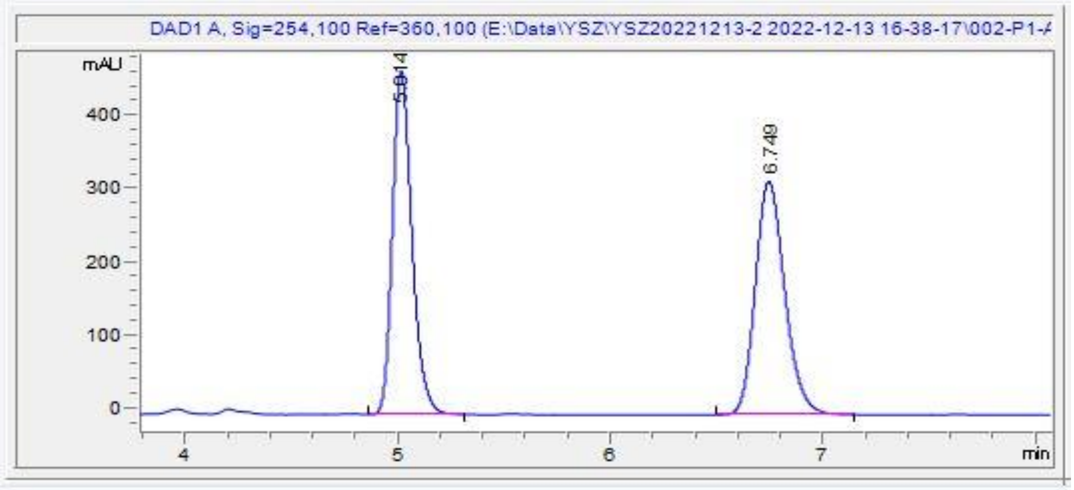

#	Time	Type	Area	Height	Width	Area%	Symmetry
1	5.014	BV R	2995.5	468.5	0.0936	49.412	0.764
2	6.749	VV R	3066.9	318.2	0.1289	50.588	0.821

Supplementary Fig. 353 HPLC spectrum of (S_p)-**1a**

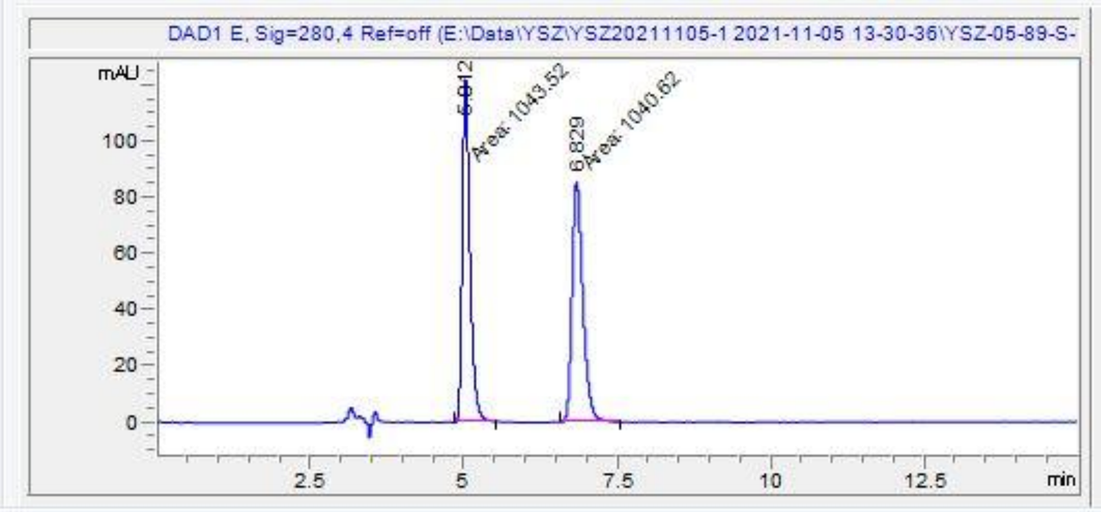

#	Time	Type	Area	Height	Width	Area%	Symmetry
1	5.012	MM	1043.5	121.6	0.143	50.069	0.67
2	6.829	MM	1040.6	85.3	0.2033	49.931	0.728

Supplementary Fig. 354 Full HPLC spectrum of racemic **1a**

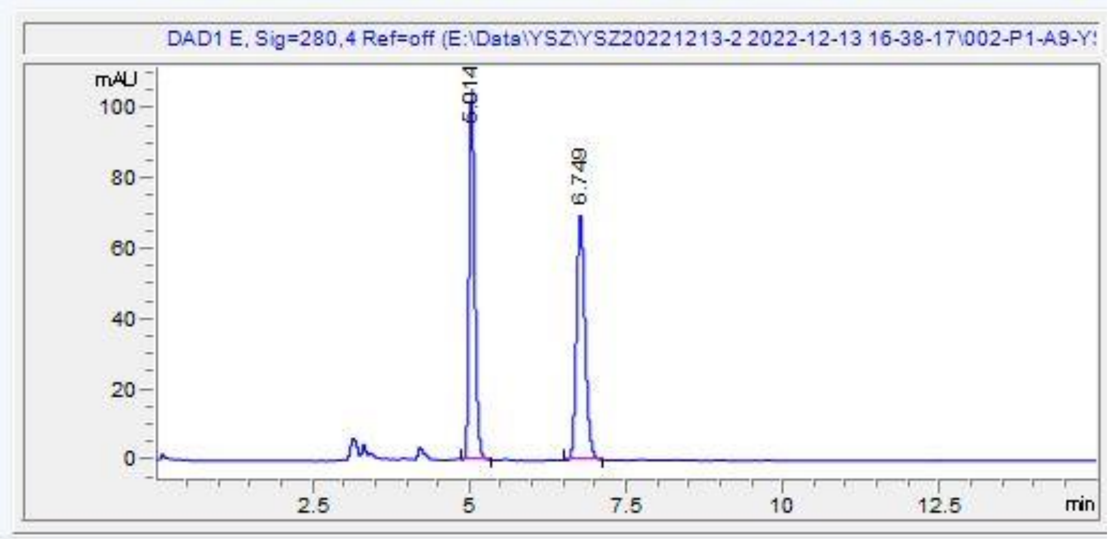

#	Time	Type	Area	Height	Width	Area%	Symmetry
1	5.014	VV R	662.2	106.1	0.0958	49.875	0.761
2	6.749	VV R	665.5	70	0.1409	50.125	0.813

Supplementary Fig. 355 Full HPLC spectrum of (S_p)-**1a**

(S_p)-Dibenzyl-1-(1[5]-((tert-butoxycarbonyl)amino)-1,4(1,4)-dibenzenacyclohexaphane-1[2]-yl)hydrazine-1,2-dicarboxylate **(3a)**

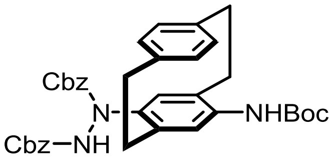

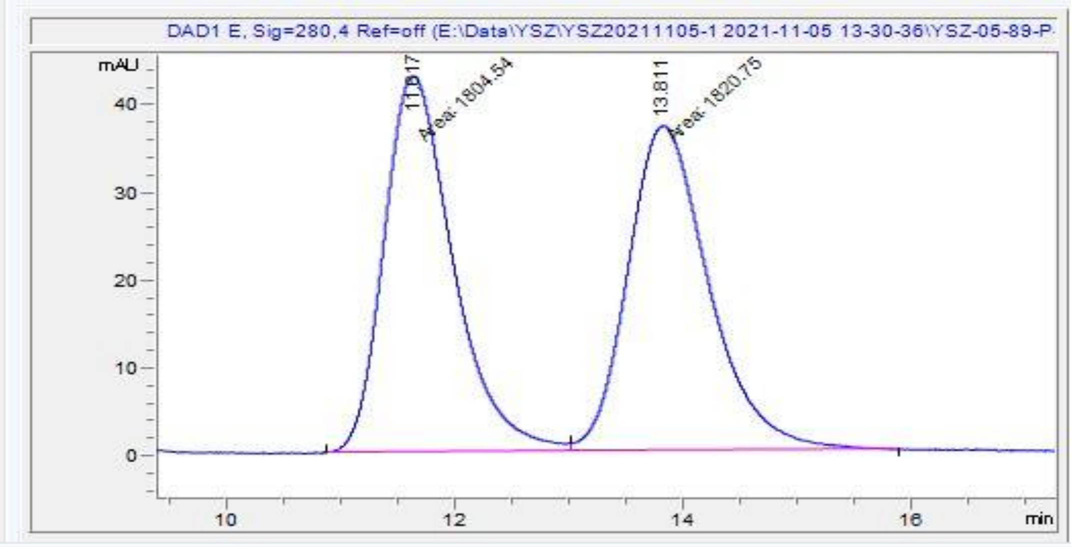

#	Time	Type	Area	Height	Width	Area%	Symmetry
1	11.617	MF	1804.5	42.8	0.7026	49.776	0.675
2	13.811	FM	1820.8	36.9	0.8213	50.224	0.717

Supplementary Fig. 356 HPLC spectrum of racemic **3a**

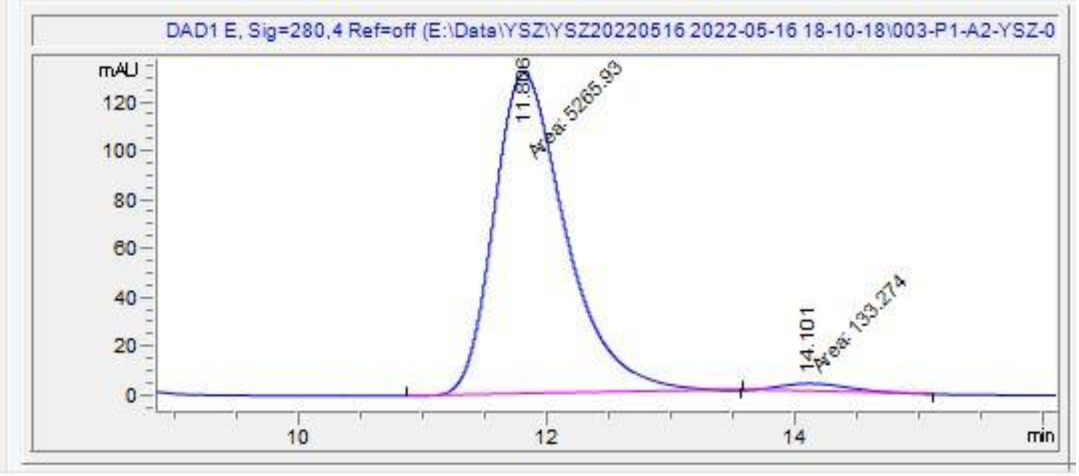

#	Time	Type	Area	Height	Width	Area%	Symmetry
1	11.806	MM	5265.9	130.4	0.6729	97.532	0.672
2	14.101	MM	133.3	3.3	0.6658	2.468	0.7

Supplementary Fig. 357 HPLC spectrum of (S_p)-**3a**

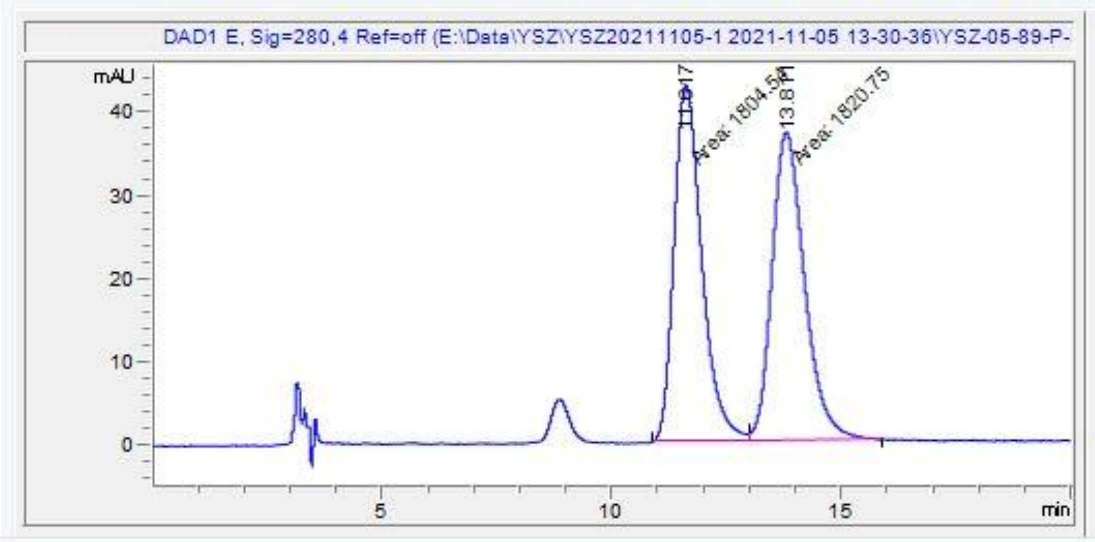

#	Time	Type	Area	Height	Width	Area%	Symmetry
1	11.617	MF	1804.5	42.8	0.7026	49.776	0.675
2	13.811	FM	1820.8	36.9	0.8213	50.224	0.717

Supplementary Fig. 358 Full HPLC spectrum of racemic **3a**

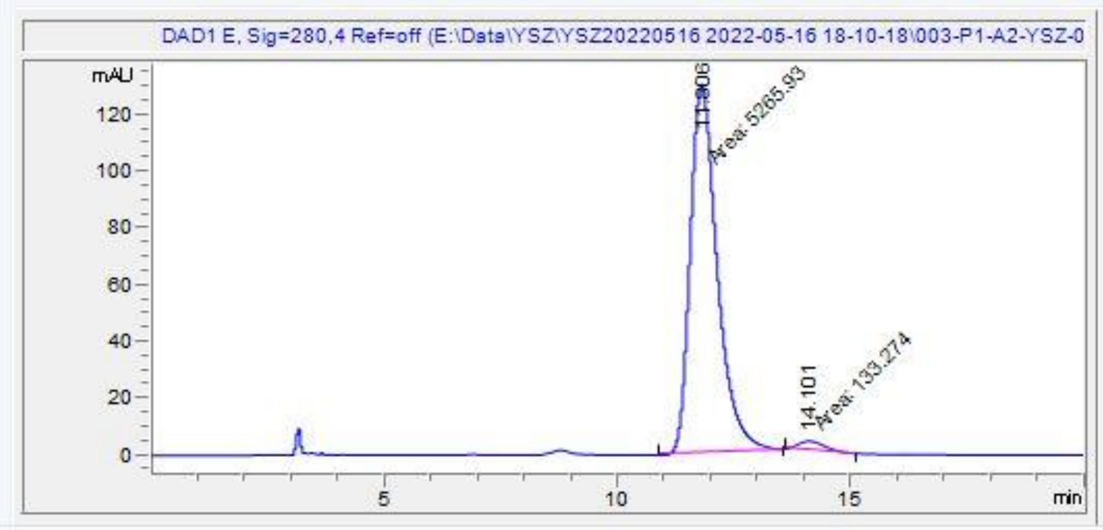

#	Time	Type	Area	Height	Width	Area%	Symmetry
1	11.806	MM	5265.9	130.4	0.6729	97.532	0.672
2	14.101	MM	133.3	3.3	0.6658	2.468	0.7

Supplementary Fig. 359 Full HPLC spectrum of (S_p)-**3a**

(S_p)-Tert-butyl (1^5-amino-1,4(1,4)-dibenzenacyclohexaphane-1^2-yl)carbamate **(9a)**

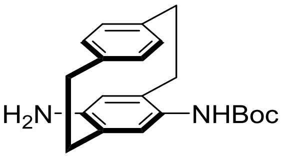

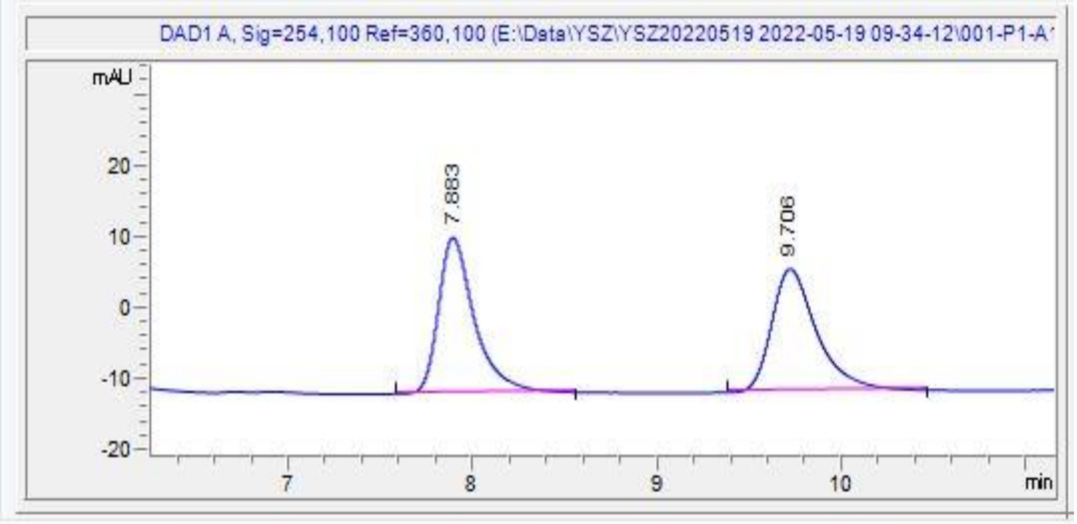

#	Time	Type	Area	Height	Width	Area%	Symmetry
1	7.883	BB	300.5	22.2	0.1993	50.062	0.627
2	9.706	BB	299.7	17.5	0.2203	49.938	0.654

Supplementary Fig. 360 HPLC spectrum of racemic **9a**

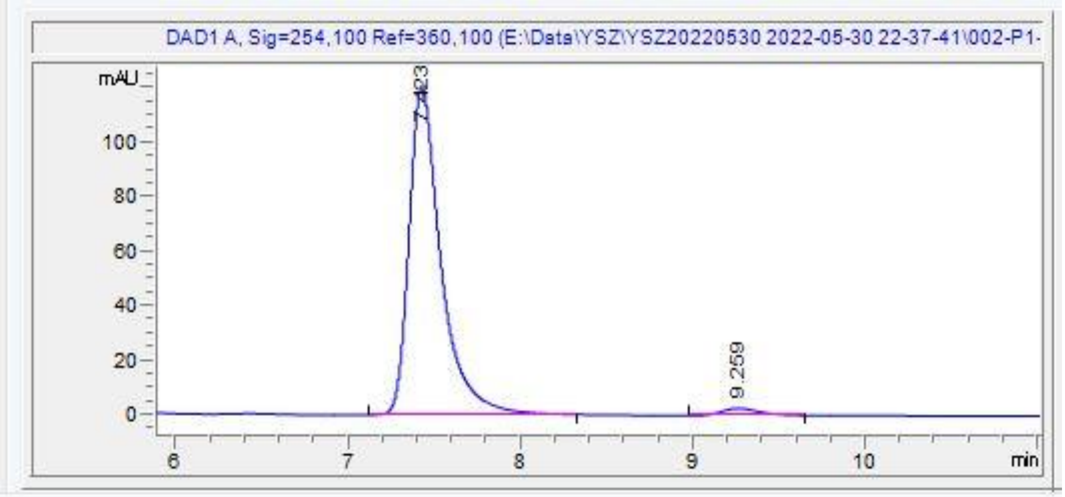

#	Time	Type	Area	Height	Width	Area%	Symmetry
1	7.423	BB	1562.9	121.4	0.1882	97.459	0.603
2	9.259	BB	40.7	2.7	0.1783	2.541	0.835

Supplementary Fig. 361 HPLC spectrum of (S_p)-**9a**

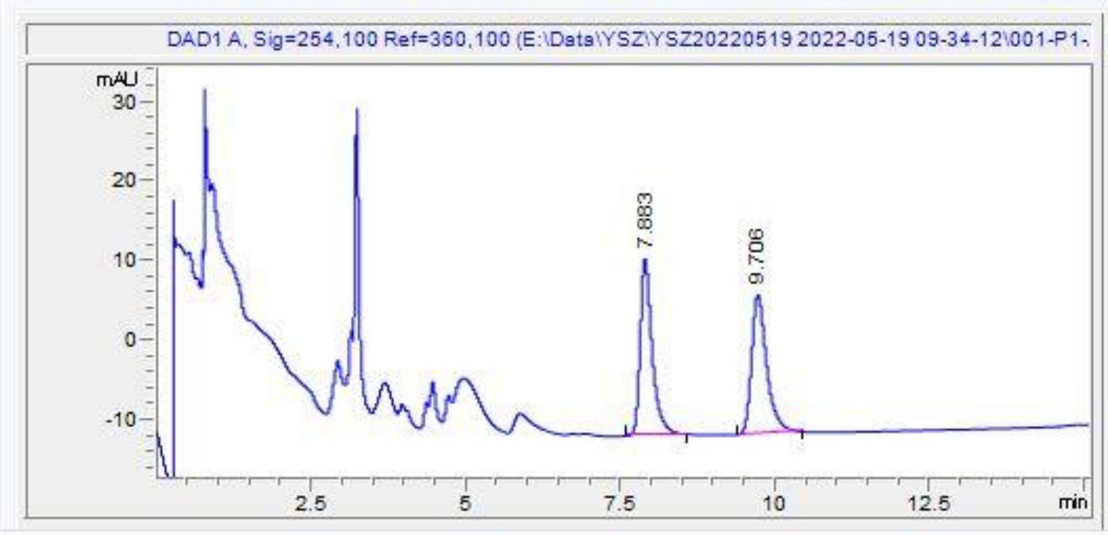

#	Time	Type	Area	Height	Width	Area%	Symmetry
1	7.883	BB	300.5	22.2	0.1993	50.062	0.627
2	9.706	BB	299.7	17.5	0.2203	49.938	0.654

Supplementary Fig. 362 Full HPLC spectrum of racemic **9a**

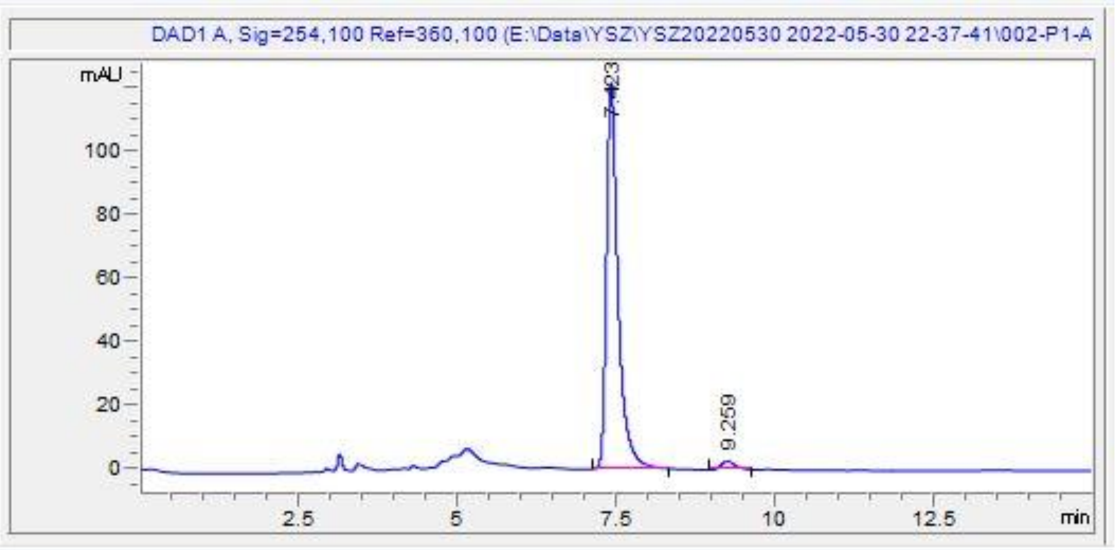

#	Time	Type	Area	Height	Width	Area%	Symmetry
1	7.423	BB	1562.9	121.4	0.1882	97.459	0.603
2	9.259	BB	40.7	2.7	0.1783	2.541	0.835

Supplementary Fig. 363 Full HPLC spectrum of (S_p)-**9a**

(S_p)-Tert-butyl-(1^5-(3-(3,5-bis(trifluoromethyl)phenyl)thioureido)-1,4(1,4)-dibenzenacyclohexaphane-1^2-yl)carbamate **(10a)**

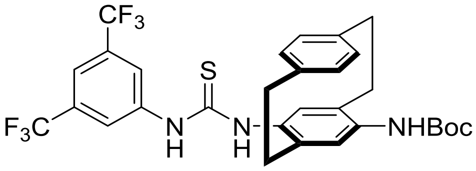

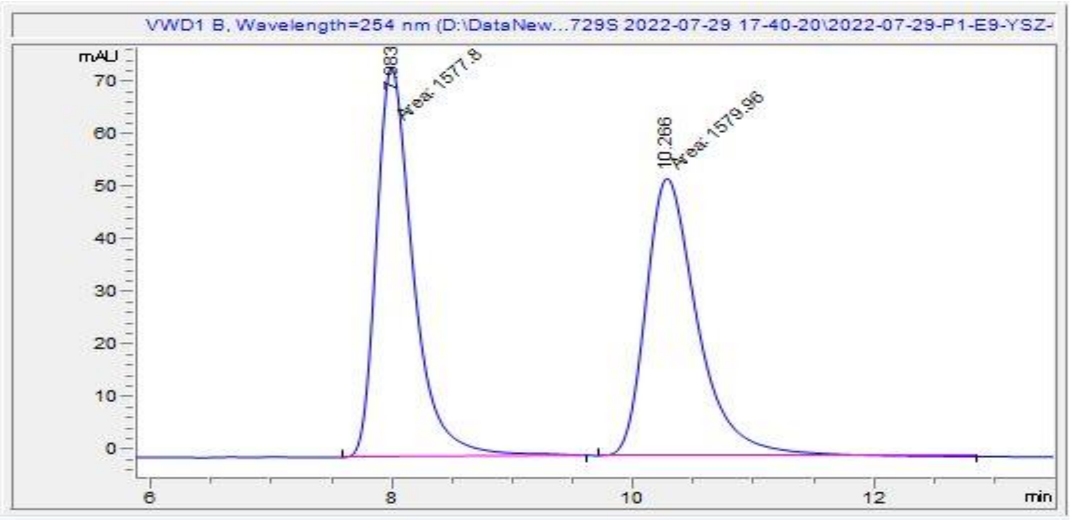

#	Time	Type	Area	Height	Width	Area%	Symmetry
1	7.983	MM	1577.8	74.2	0.3544	49.966	0.658
2	10.266	MM	1580	52.8	0.4983	50.034	0.657

Supplementary Fig. 364 HPLC spectrum of racemic **10a**

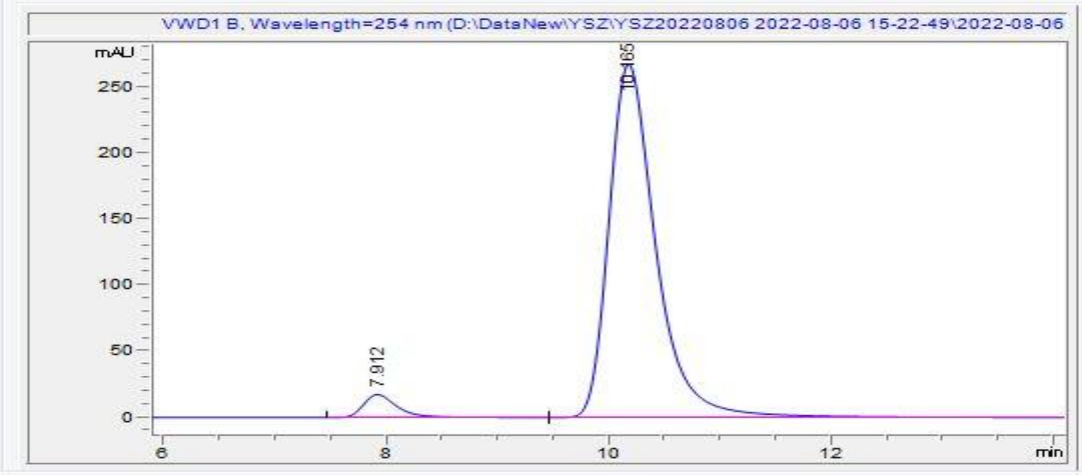

#	Time	Type	Area	Height	Width	Area%	Symmetry
1	7.912	BB	372.3	17.4	0.3229	4.517	0.644
2	10.165	BBA	7870.6	268.4	0.4442	95.483	0.655

Supplementary Fig. 365 HPLC spectrum of (S_p)-**10a**

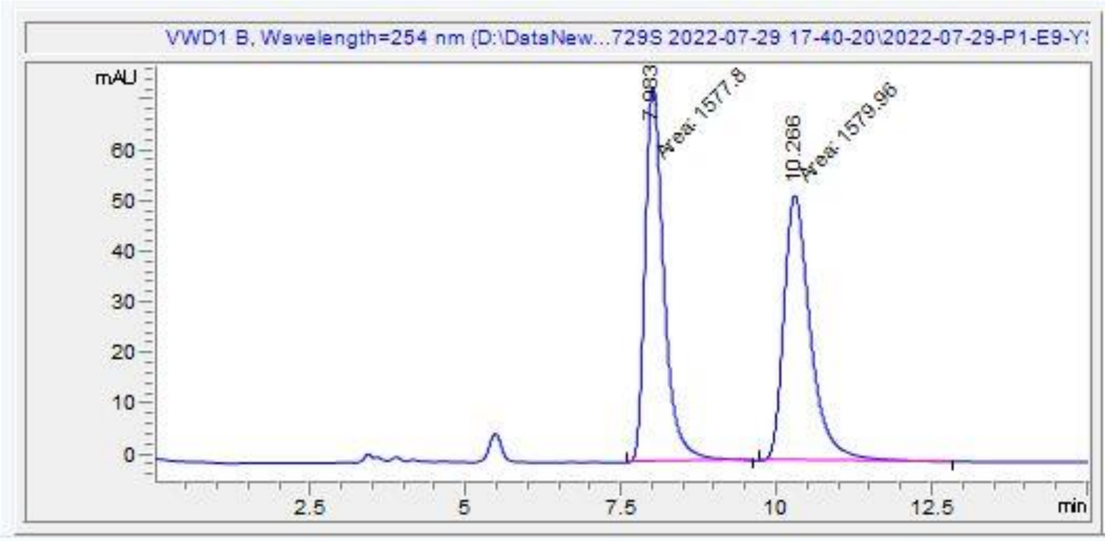

#	Time	Type	Area	Height	Width	Area%	Symmetry
1	7.983	MM	1577.8	74.2	0.3544	49.966	0.658
2	10.266	MM	1580	52.8	0.4983	50.034	0.657

Supplementary Fig. 366 Full HPLC spectrum of racemic **10a**

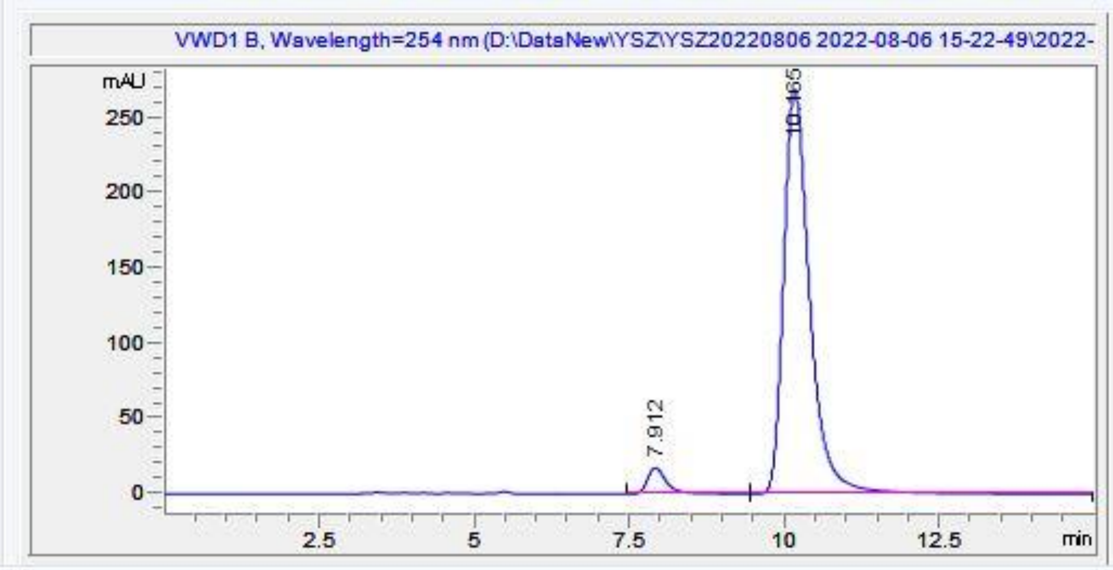

#	Time	Type	Area	Height	Width	Area%	Symmetry
1	7.912	BB	372.3	17.4	0.3229	4.517	0.644
2	10.165	BBA	7870.6	268.4	0.4442	95.483	0.655

Supplementary Fig. 367 Full HPLC spectrum of (S_p)-**10a**

(S_p)-1,1'-(1,4(1,4)-dibenzenacyclohexaphane-1^2,1^5-diyl)bis(3-(3,5-bis(trifluoromethyl) phenyl)thiourea) **(11a)**

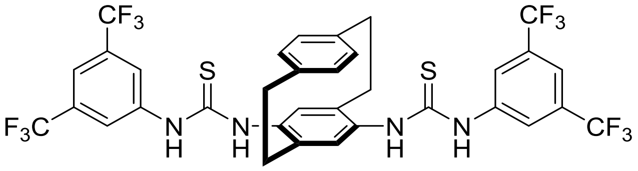

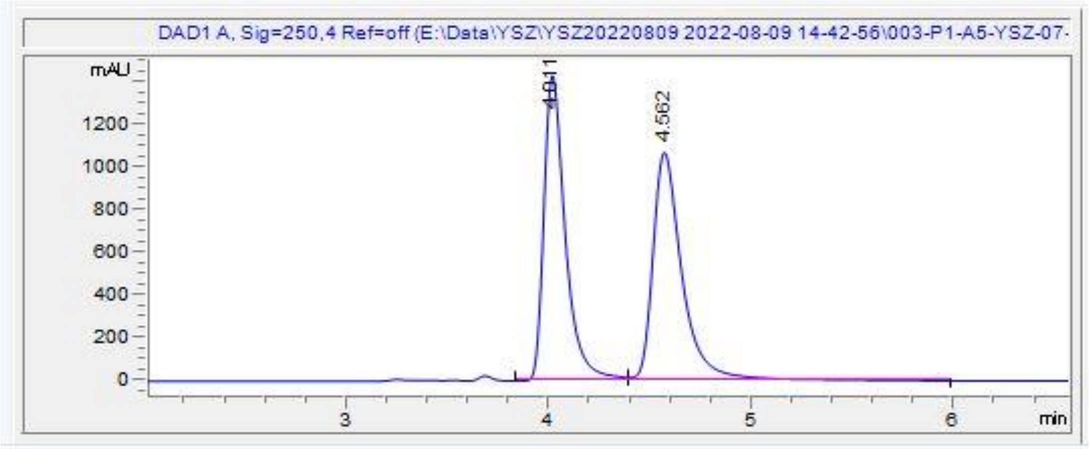

#	Time	Type	Area	Height	Width	Area%	Symmetry
1	4.011	BV	10562.6	1435.3	0.1119	49.254	0.598
2	4.562	VB	10882.6	1072.9	0.1523	50.746	0.572

Supplementary Fig. 368 HPLC spectrum of racemic **11a**

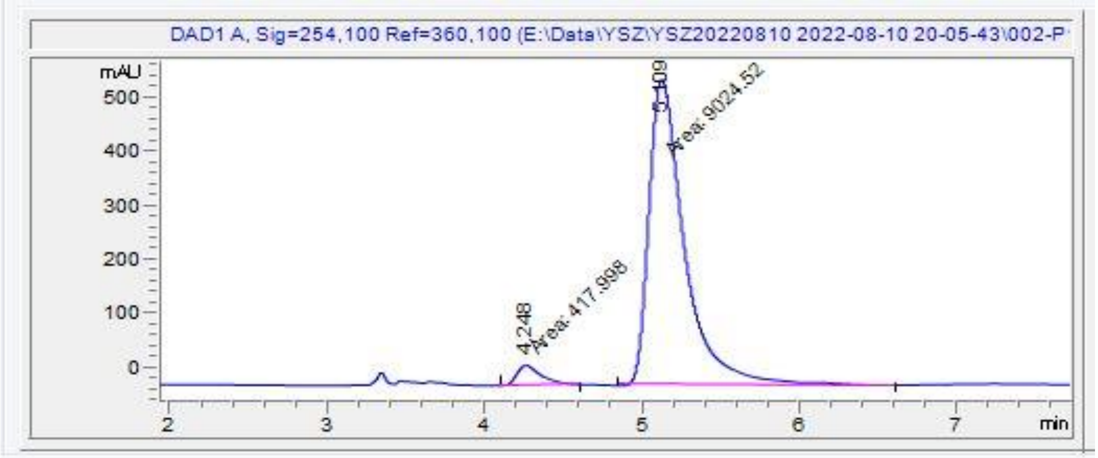

#	Time	Type	Area	Height	Width	Area%	Symmetry
1	4.248	MM	418	37.4	0.1863	4.427	0.539
2	5.109	MM	9024.5	570.4	0.2637	95.573	0.534

Supplementary Fig. 369 HPLC spectrum of (S_p)-**11a**

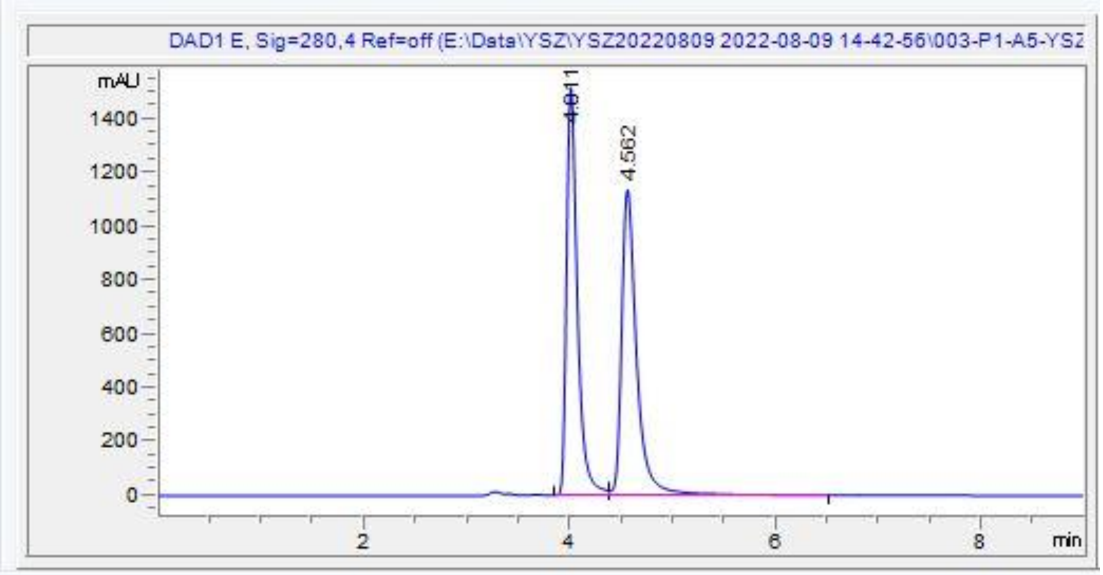

#	Time	Type	Area	Height	Width	Area%	Symmetry
1	4.011	BV	11091.4	1507.9	0.1118	49.123	0.6
2	4.562	VB	11487.2	1128.7	0.1527	50.877	0.568

Supplementary Fig. 370 Full HPLC spectrum of racemic **11a**

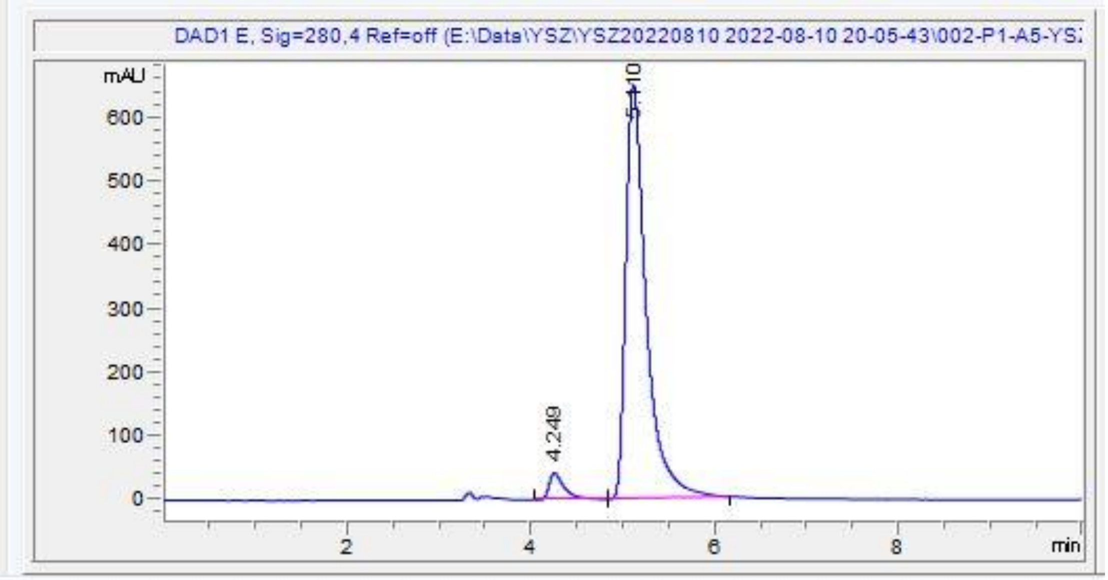

#	Time	Type	Area	Height	Width	Area%	Symmetry
1	4.249	BB	489.9	42.1	0.1672	4.672	0.489
2	5.11	BB	9995	648.1	0.2304	95.328	0.532

Supplementary Fig. 371 Full HPLC spectrum of (S_p)**-11a**

(S_p)-Tert-butyl (1^5-iodo-1,4(1,4)-dibenzenacyclohexaphane-1^2-yl)carbamate **(12a)**

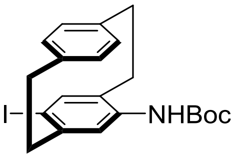

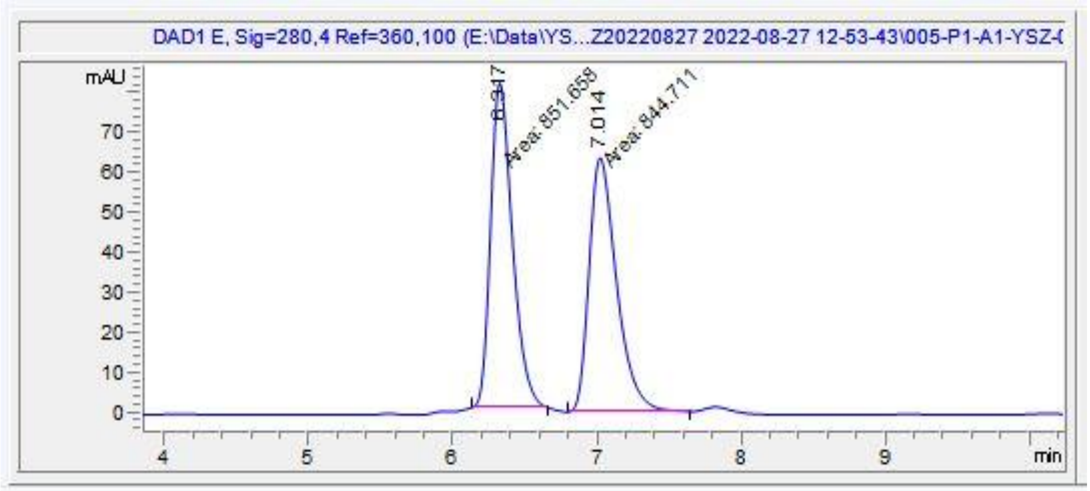

#	Time	Type	Area	Height	Width	Area%	Symmetry
1	6.317	MM	851.7	81.3	0.1746	50.205	0.7
2	7.014	MM	844.7	63.7	0.221	49.795	0.646

Supplementary Fig. 372 HPLC spectrum of racemic **12a**

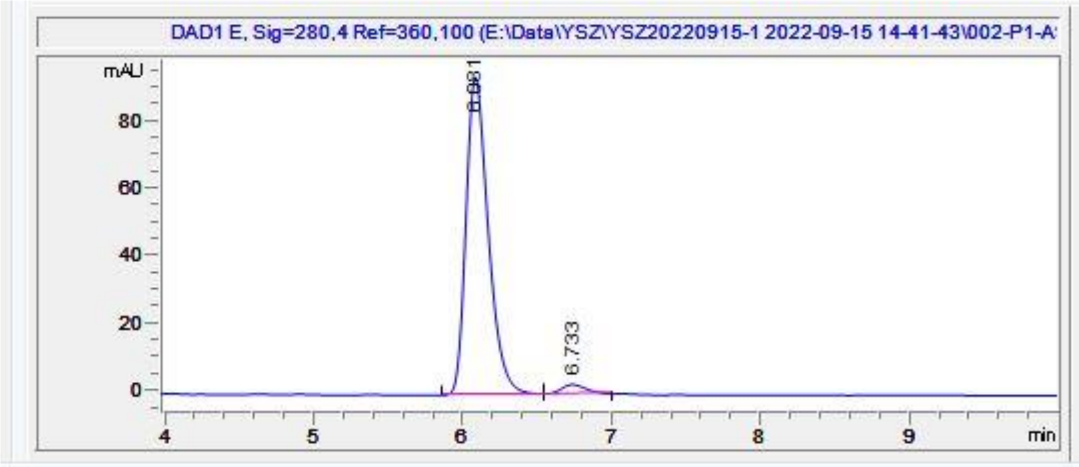

#	Time	Type	Area	Height	Width	Area%	Symmetry
1	6.081	BV	980.6	94.4	0.1591	96.660	0.671
2	6.733	VB	33.9	2.9	0.1797	3.340	0.796

Supplementary Fig. 373 HPLC spectrum of (S_p)**-12a**

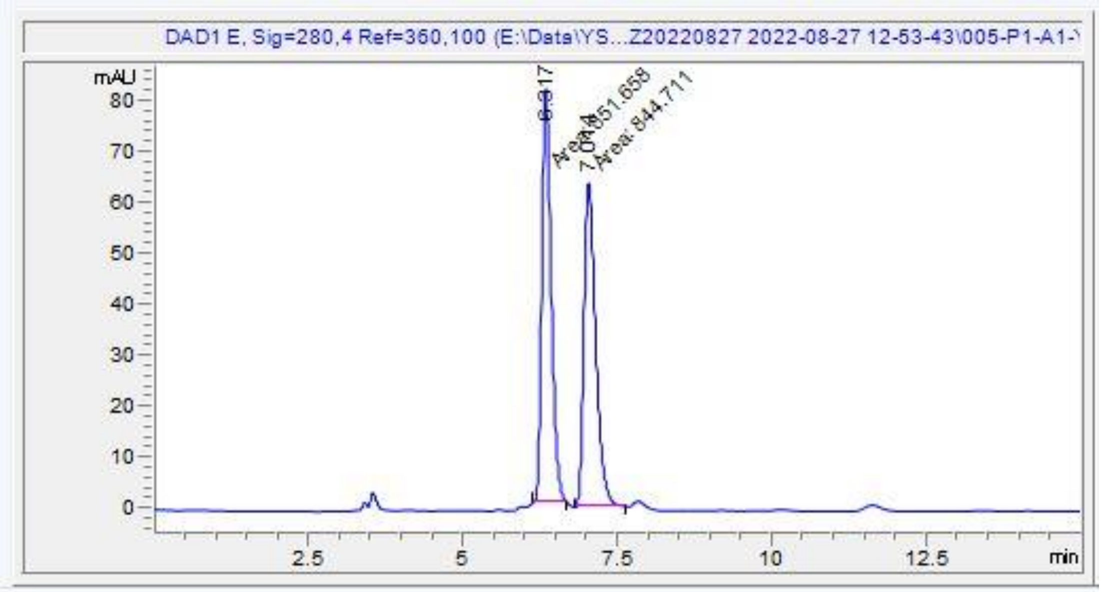

#	Time	Type	Area	Height	Width	Area%	Symmetry
1	6.317	MM	851.7	81.3	0.1746	50.205	0.7
2	7.014	MM	844.7	63.7	0.221	49.795	0.646

Supplementary Fig. 374 Full HPLC spectrum of racemic **12a**

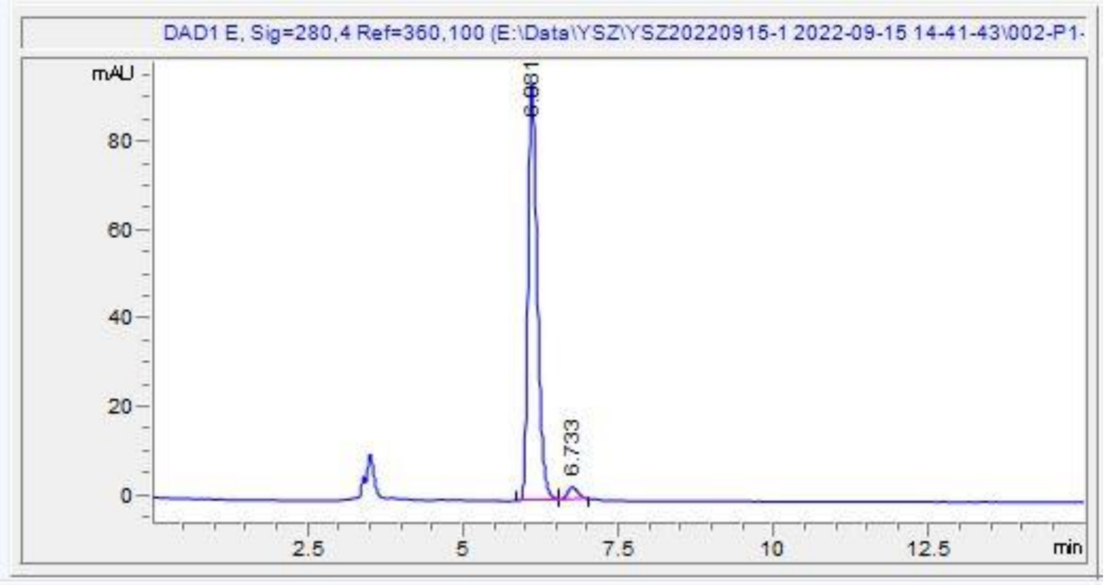

#	Time	Type	Area	Height	Width	Area%	Symmetry
1	6.081	BV	980.6	94.4	0.1591	96.660	0.671
2	6.733	VB	33.9	2.9	0.1797	3.340	0.796

Supplementary Fig. 375 Full HPLC spectrum of (S_p)**-12a**

(R_p)-Tert-butyl (4^2-bromo-1,4(1,4)-dibenzenacyclohexaphane-1^2-yl)carbamate **(1f)**

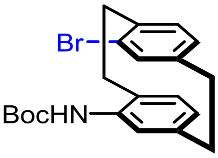

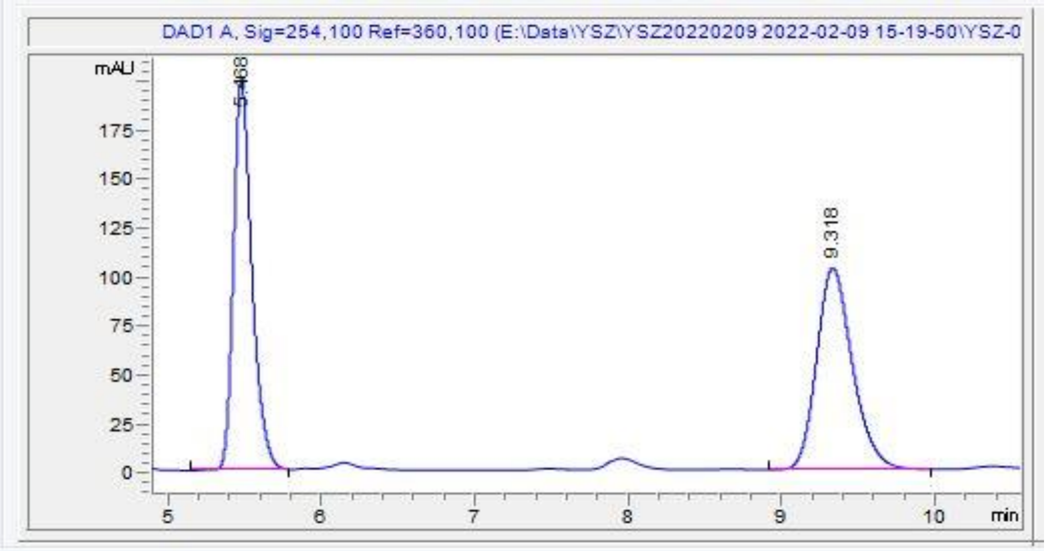

#	Time	Type	Area	Height	Width	Area%	Symmetry
1	5.468	BB	1663.6	199.8	0.1256	49.910	0.71
2	9.318	BB	1669.5	103.1	0.2459	50.090	0.77

Supplementary Fig. 376 HPLC spectrum of racemic **1f**

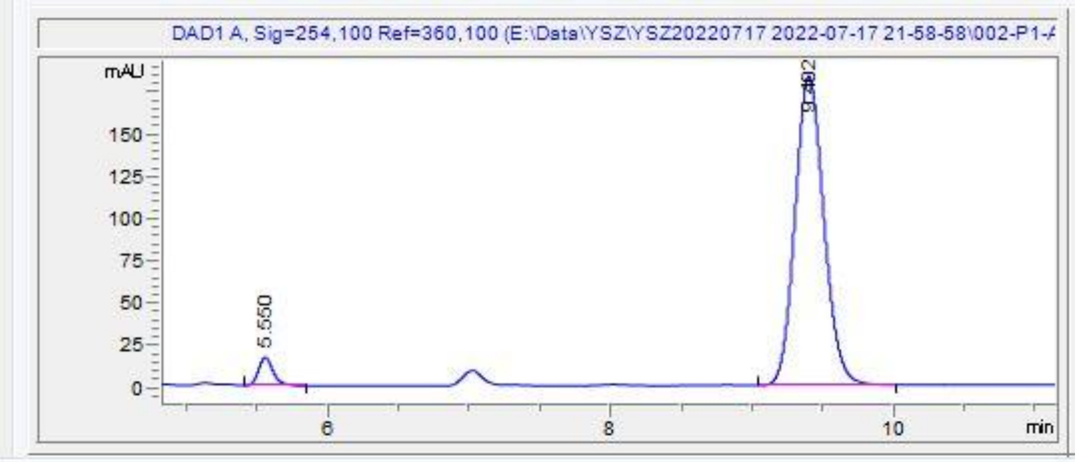

#	Time	Type	Area	Height	Width	Area%	Symmetry
1	5.55	BB	120.1	16.6	0.1058	4.425	0.782
2	9.402	BB	2595.2	182.6	0.2133	95.575	0.861

Supplementary Fig. 377 HPLC spectrum of (R_p)-**1f**

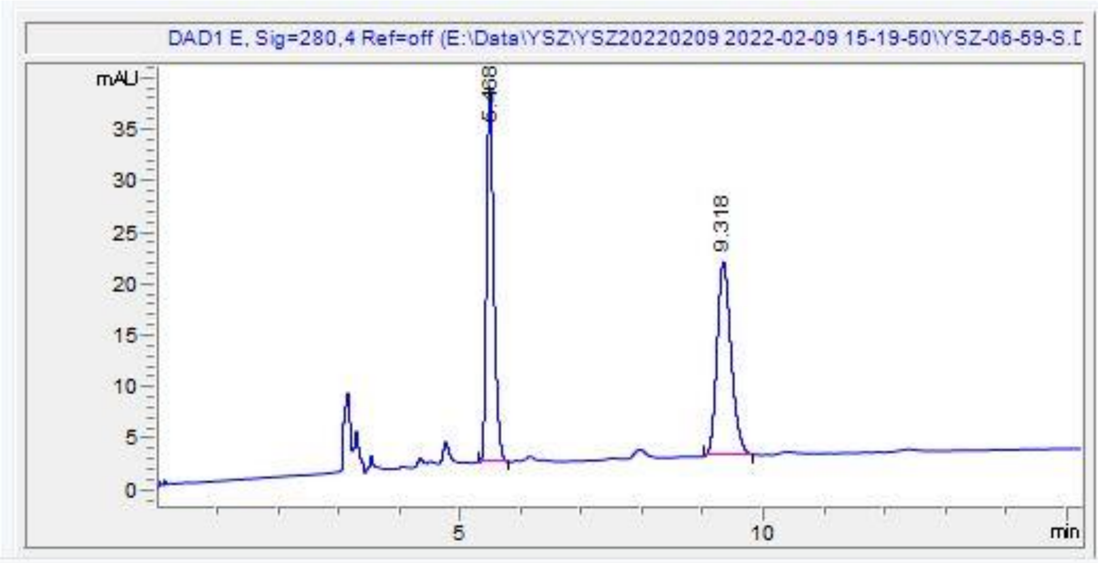

#	Time	Type	Area	Height	Width	Area%	Symmetry
1	5.468	BB	301.7	36.4	0.1262	49.936	0.698
2	9.318	BB	302.5	18.8	0.1896	50.064	0.791

Supplementary Fig. 378 Full HPLC spectrum of racemic **1f**

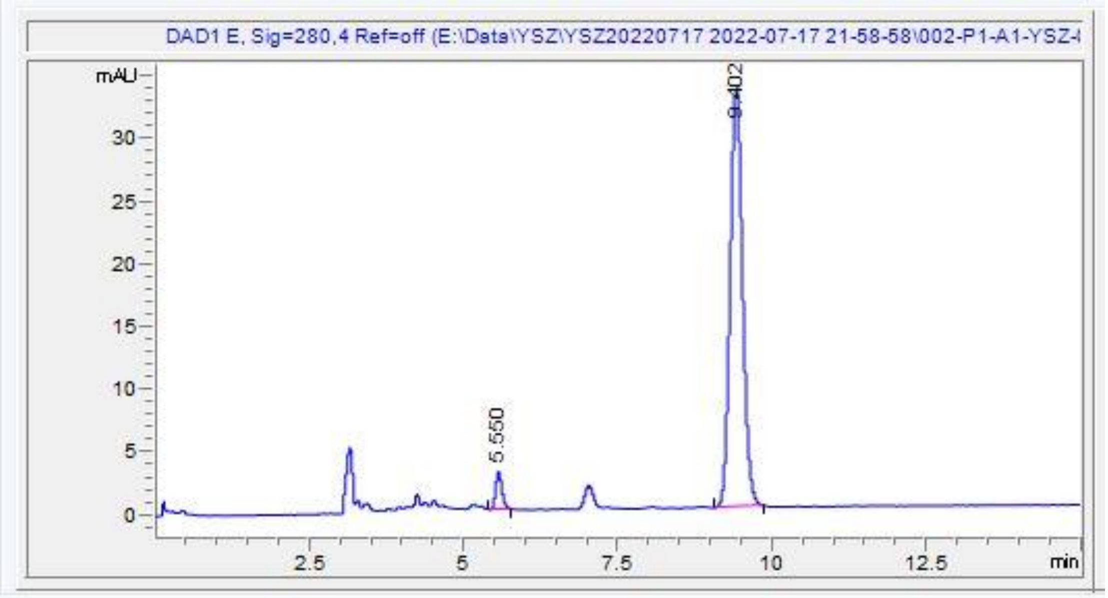

#	Time	Type	Area	Height	Width	Area%	Symmetry
1	5.55	BB	22.3	3	0.0878	4.493	0.769
2	9.402	BB	474.2	33.5	0.1736	95.507	0.894

Supplementary Fig. 379 Full HPLC spectrum of (R_p)-**1f**

(R_p)-*N*-(4^2-bromo-1,4(1,4)-dibenzenacyclohexaphane-1^2-yl)pivalamide **(13f)**

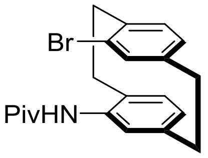

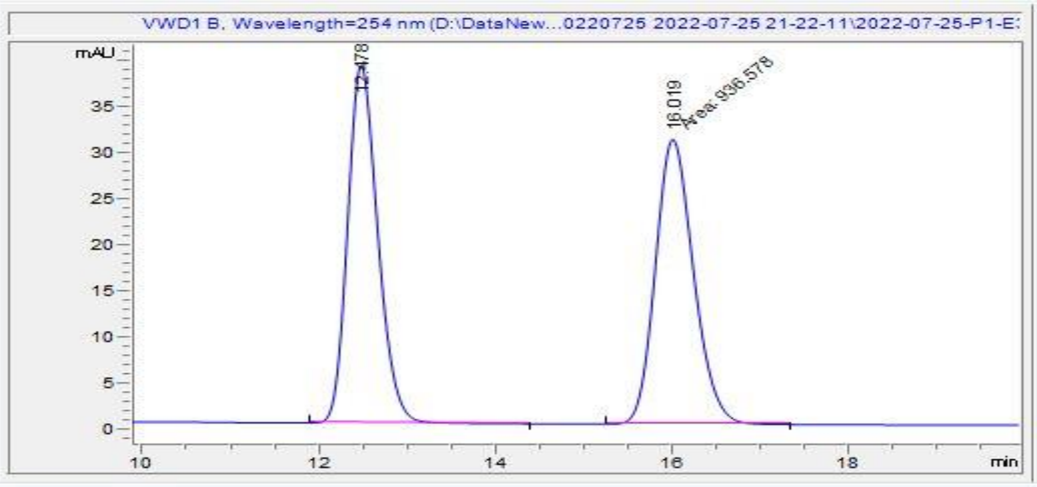

#	Time	Type	Area	Height	Width	Area%	Symmetry
1	12.478	BB	931.7	39.1	0.3658	49.870	0.773
2	16.019	MM	936.6	31.1	0.5014	50.130	0.815

Supplementary Fig. 380 HPLC spectrum of racemic **13f**

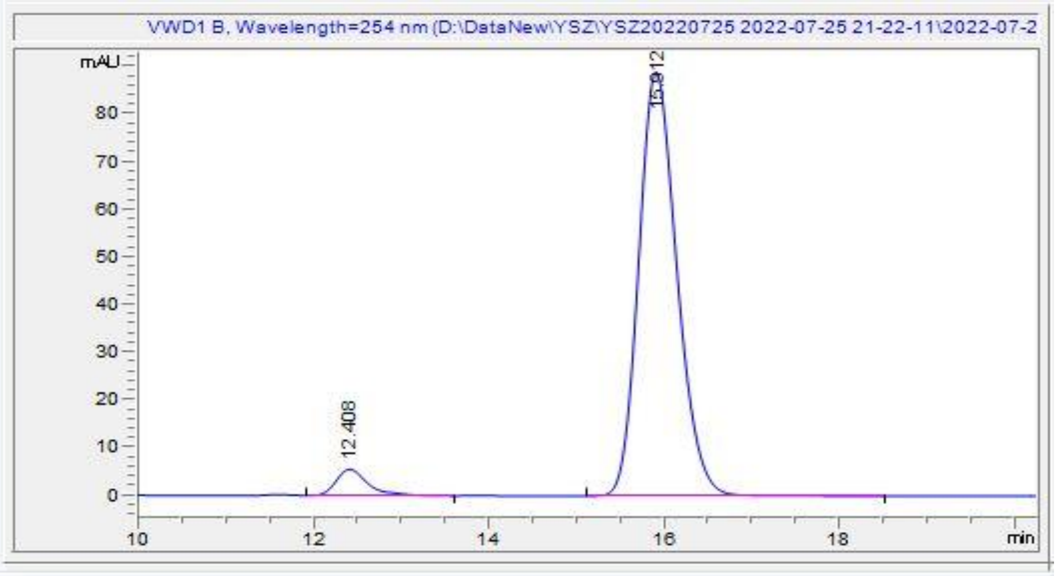

#	Time	Type	Area	Height	Width	Area%	Symmetry
1	12.408	BB	139.2	5.5	0.3826	4.934	0.69
2	15.912	BB	2682	88.7	0.467	95.066	0.785

Supplementary Fig. 381 HPLC spectrum of (R_p)**-13f**

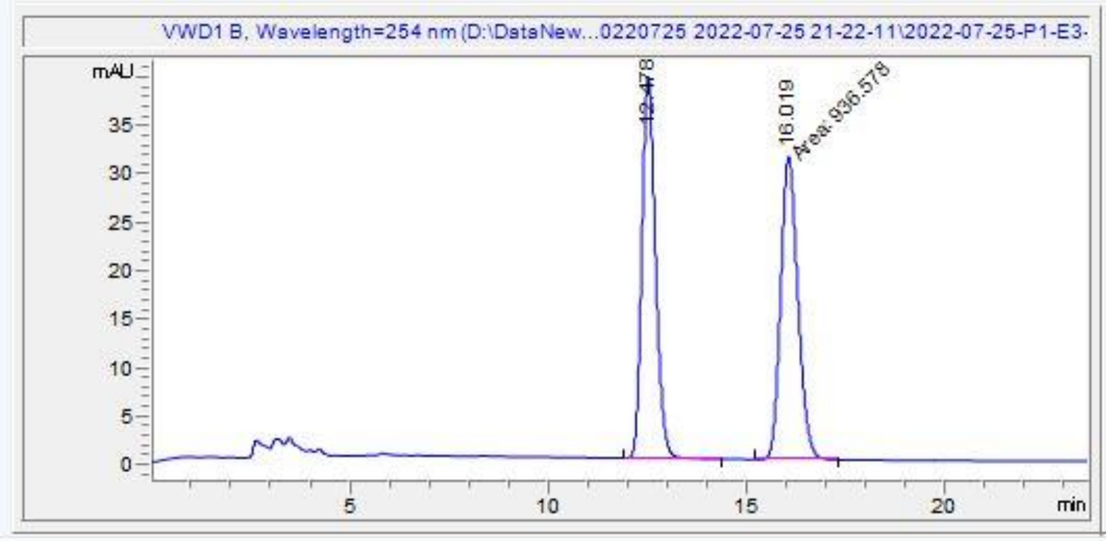

#	Time	Type	Area	Height	Width	Area%	Symmetry
1	12.478	BB	931.7	39.1	0.3658	49.870	0.773
2	16.019	MM	936.6	31.1	0.5014	50.130	0.815

Supplementary Fig. 382 Full HPLC spectrum of racemic **13f**

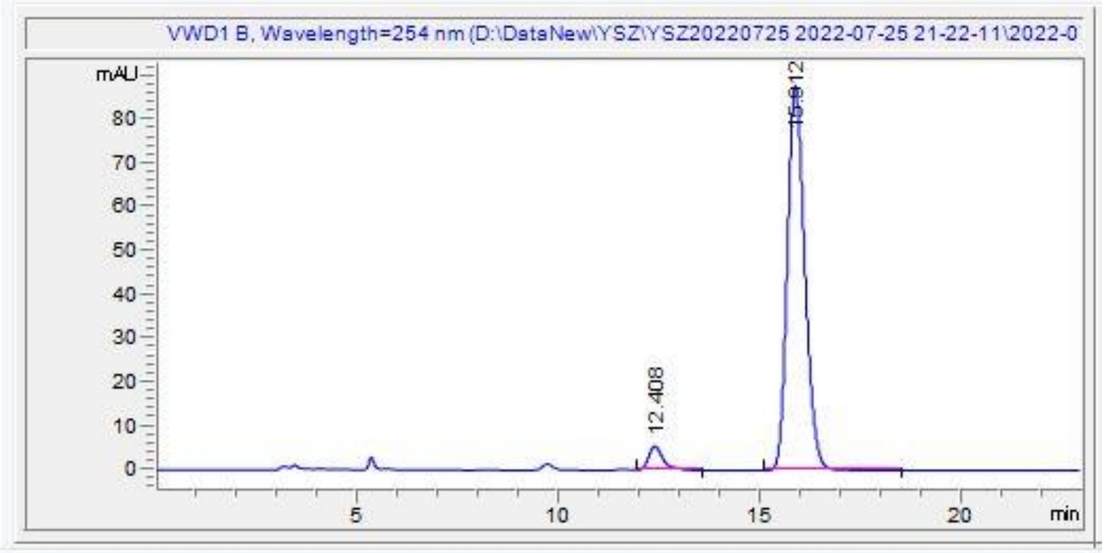

#	Time	Type	Area	Height	Width	Area%	Symmetry
1	12.408	BB	139.2	5.5	0.3826	4.934	0.69
2	15.912	BB	2682	88.7	0.467	95.066	0.785

Supplementary Fig. 383 Full HPLC spectrum of (R_p)**-13f**

(R_p)-*N*-(4[2]-bromo-1[3]-(4,4,5,5-tetramethyl-1,3,2-dioxaborolan-2-yl)-1,4(1,4)-dibenzenacyclohexaphane-1[2]-yl)pivalamide **(14f)**

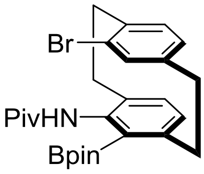

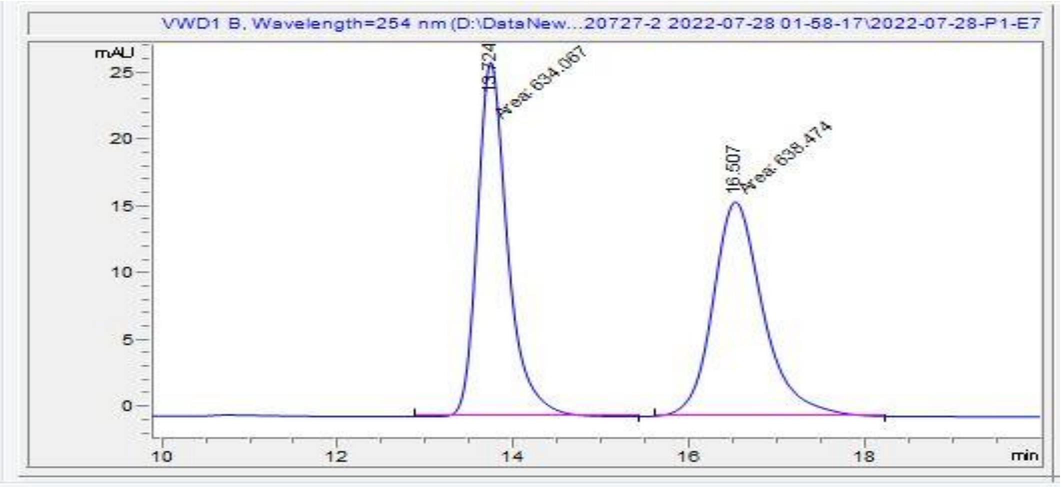

#	Time	Type	Area	Height	Width	Area%	Symmetry
1	13.724	MM	634.1	26.5	0.3991	49.827	0.75
2	16.507	MM	638.5	16	0.6641	50.173	0.782

Supplementary Fig. 384 HPLC spectrum of racemic **14f**

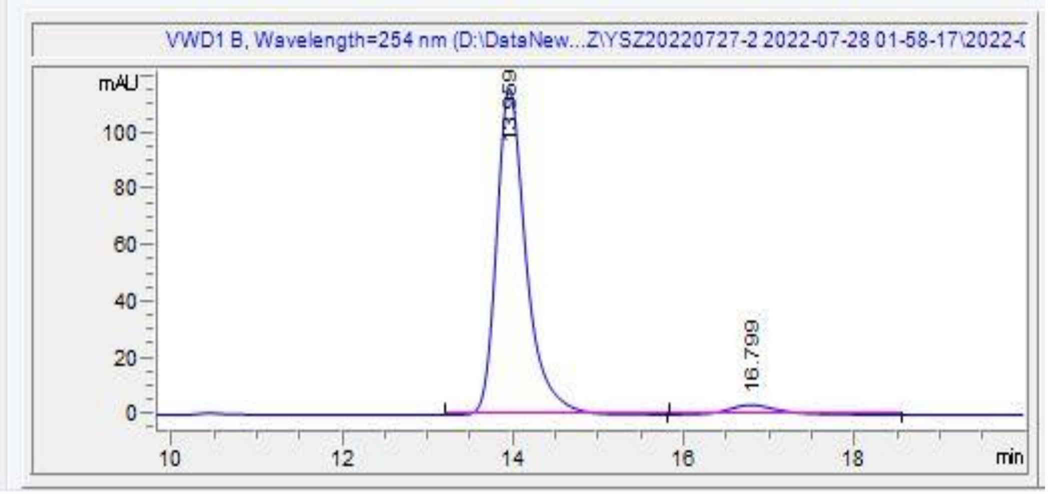

#	Time	Type	Area	Height	Width	Area%	Symmetry
1	13.959	BB	2807.1	116.3	0.3636	95.279	0.699
2	16.799	BB	139.1	3.5	0.6048	4.721	0.788

Supplementary Fig. 385 HPLC spectrum of (R_p)-**14f**

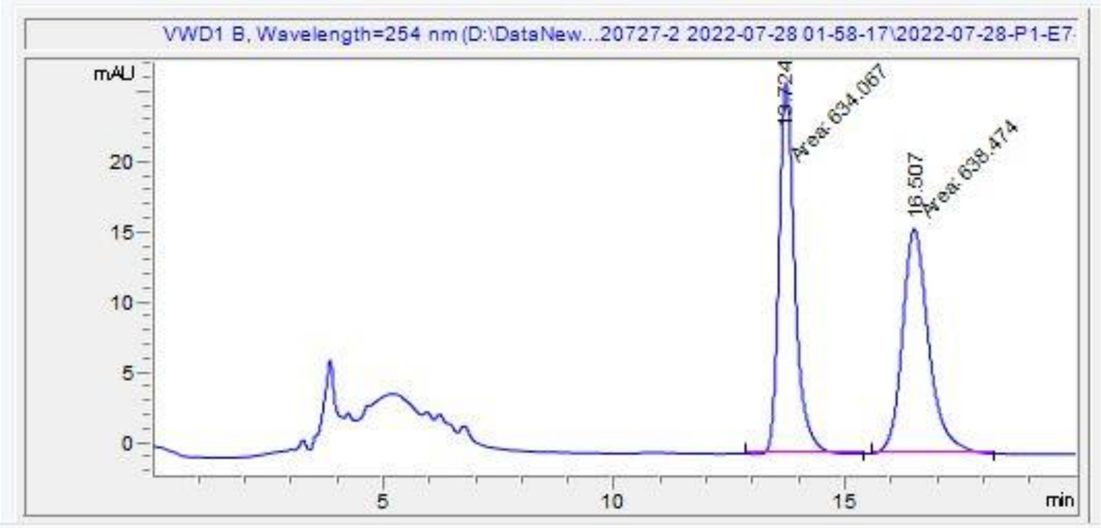

#	Time	Type	Area	Height	Width	Area%	Symmetry
1	13.724	MM	634.1	26.5	0.3991	49.827	0.75
2	16.507	MM	638.5	16	0.6641	50.173	0.782

Supplementary Fig. 386 Full HPLC spectrum of racemic **14f**

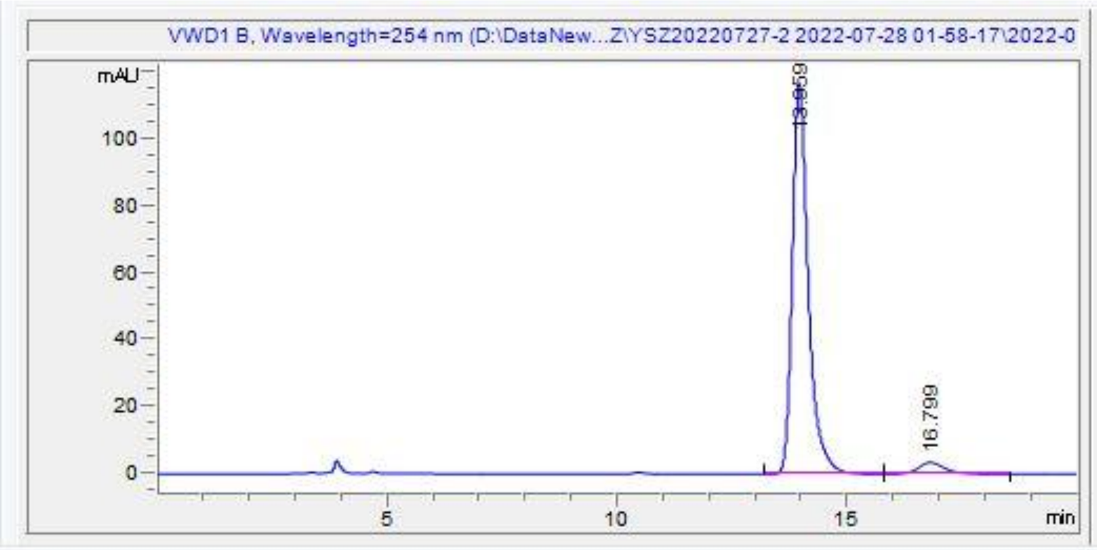

#	Time	Type	Area	Height	Width	Area%	Symmetry
1	13.959	BB	2807.1	116.3	0.3636	95.279	0.699
2	16.799	BB	139.1	3.5	0.6048	4.721	0.788

Supplementary Fig. 387 Full HPLC spectrum of (R_p)-**14f**

(R_p)-(E)-4-((4[2]-bromo-1,4(1,4)-dibenzenacyclohexaphane-1[2]-yl)amino)pent-3-en-2-one (**INT 1**)

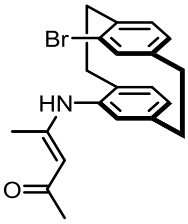

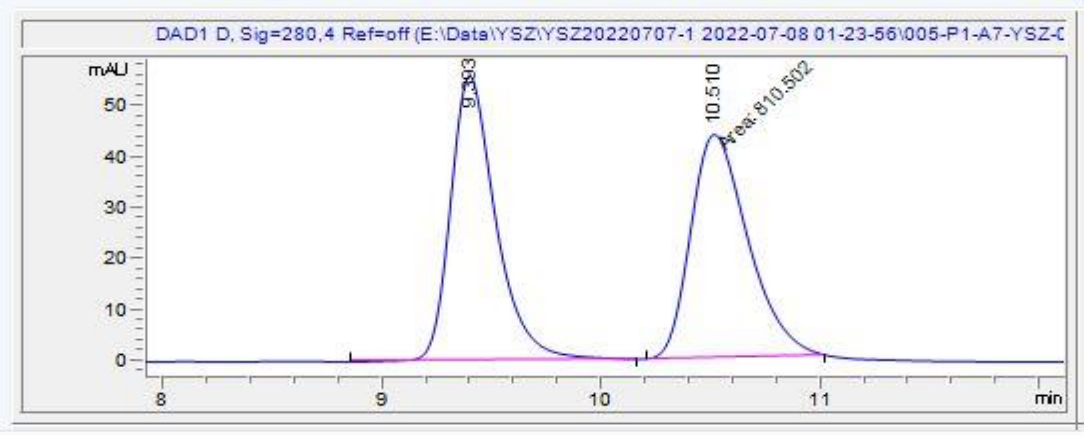

#	Time	Type	Area	Height	Width	Area%	Symmetry
1	9.393	BB	807.1	56.2	0.2177	49.895	0.678
2	10.51	MM	810.5	44.2	0.3056	50.105	0.68

Supplementary Fig. 388 HPLC spectrum of racemic **INT 1**

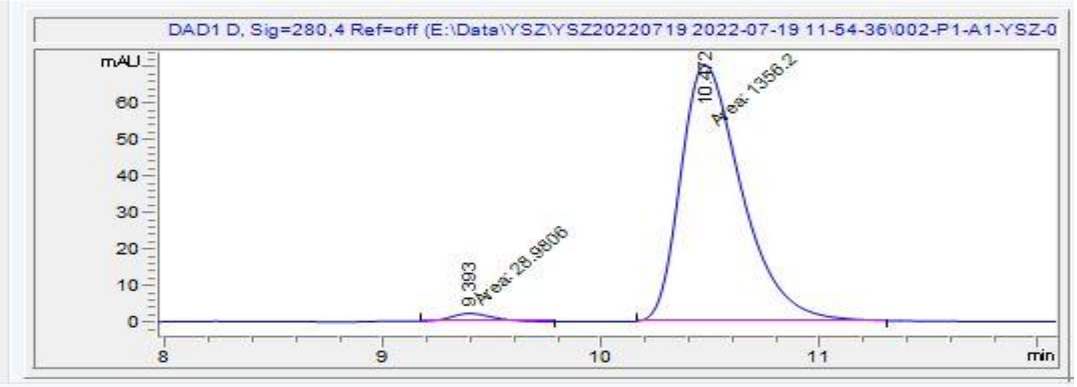

#	Time	Type	Area	Height	Width	Area%	Symmetry
1	9.393	MM	29	2.2	0.2238	2.092	0.746
2	10.472	MM	1356.2	69.9	0.3233	97.908	0.641

Supplementary Fig. 389 HPLC spectrum of (R_p)-**INT 1**

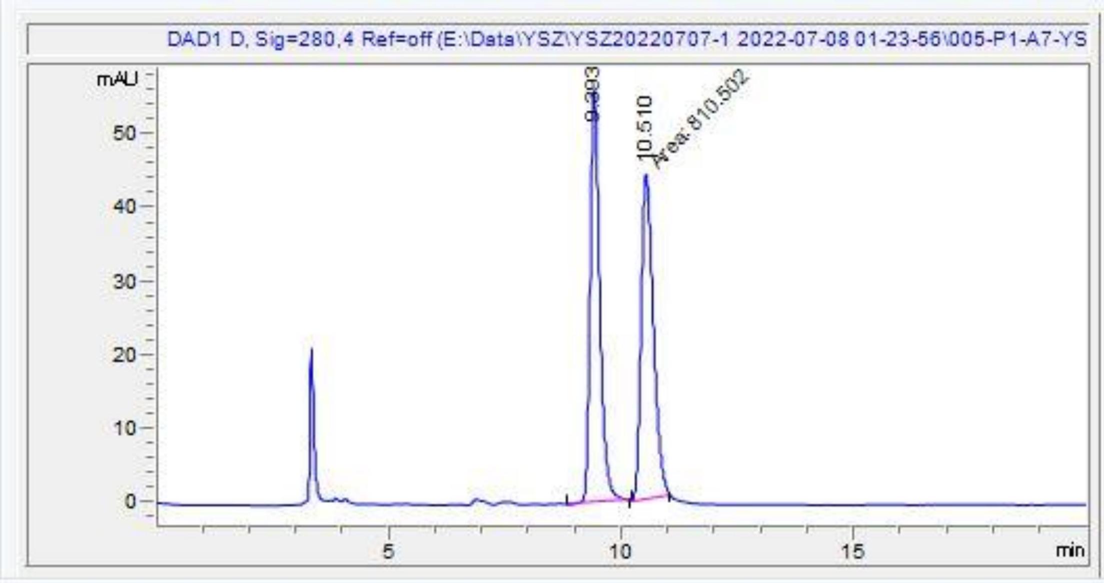

#	Time	Type	Area	Height	Width	Area%	Symmetry
1	9.393	BB	807.1	56.2	0.2177	49.895	0.678
2	10.51	MM	810.5	44.2	0.3056	50.105	0.68

Supplementary Fig. 390 Full HPLC spectrum of racemic **INT 1**

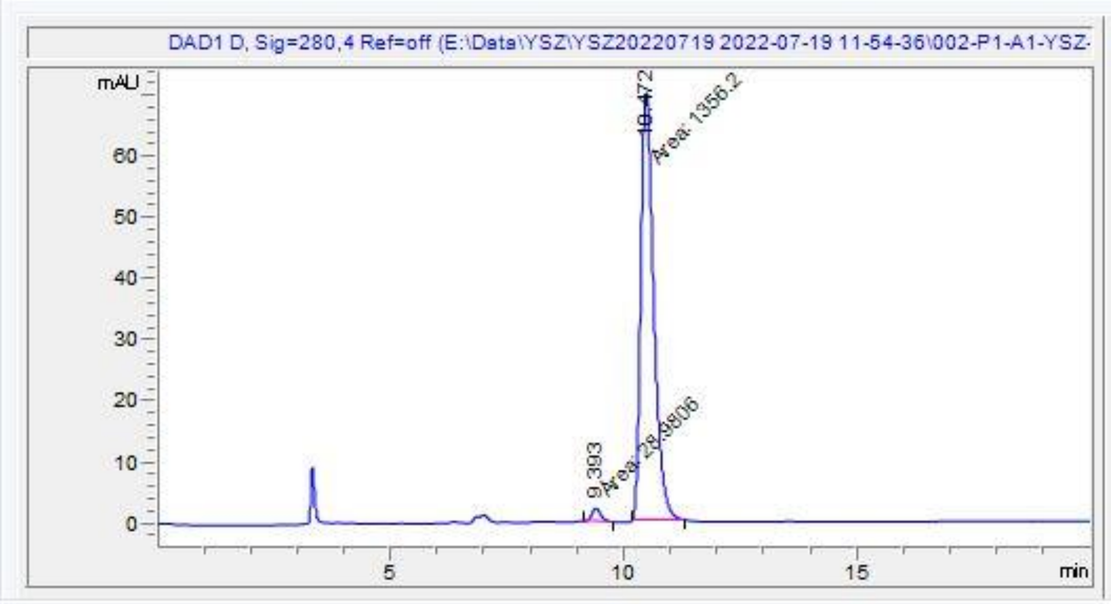

#	Time	Type	Area	Height	Width	Area%	Symmetry
1	9.393	MM	29	2.2	0.2238	2.092	0.746
2	10.472	MM	1356.2	69.9	0.3233	97.908	0.641

Supplementary Fig. 391 Full HPLC spectrum of (R_p)-**INT 1**

(S_p)-4[2]-bromo-1[2],1[4]-dimethyl-1(5,8)-quinolina-4(1,4)-benzenacyclohexaphane **(15f)**

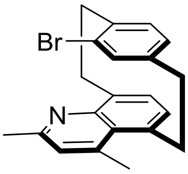

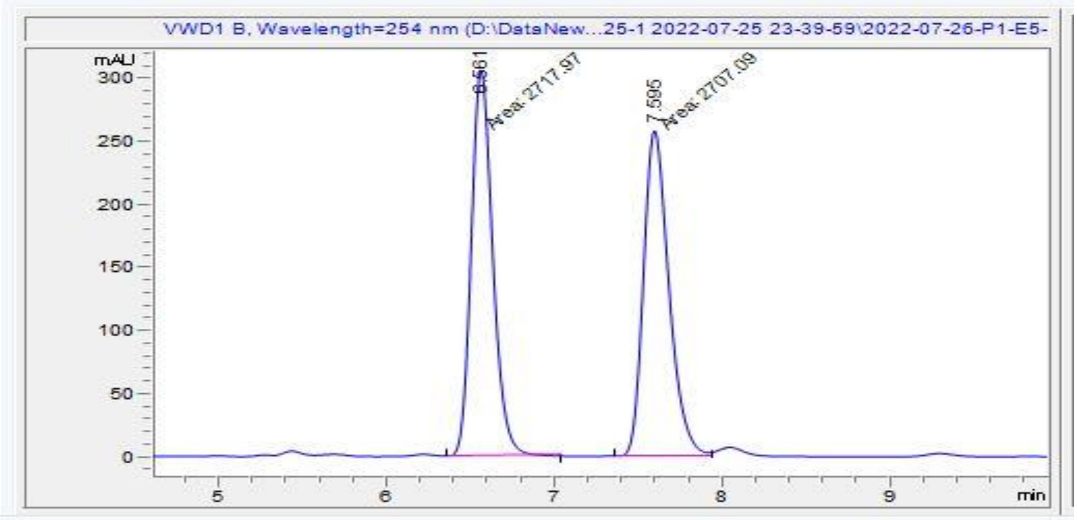

#	Time	Type	Area	Height	Width	Area%	Symmetry
1	6.561	MM	2718	305.8	0.1481	50.100	0.808
2	7.595	MM	2707.1	257.2	0.1754	49.900	0.734

Supplementary Fig. 392 HPLC spectrum racemic **15f**

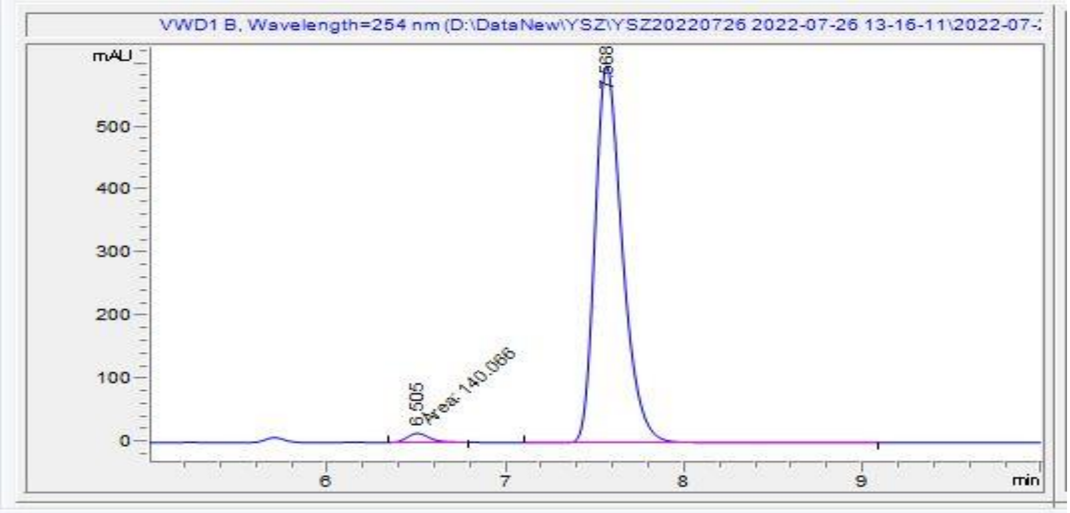

#	Time	Type	Area	Height	Width	Area%	Symmetry
1	6.505	MF	140.1	14.9	0.1569	2.140	0.761
2	7.568	BB	6404	599.9	0.1625	97.860	0.692

Supplementary Fig. 393 HPLC spectrum of (S_p)-**15f**

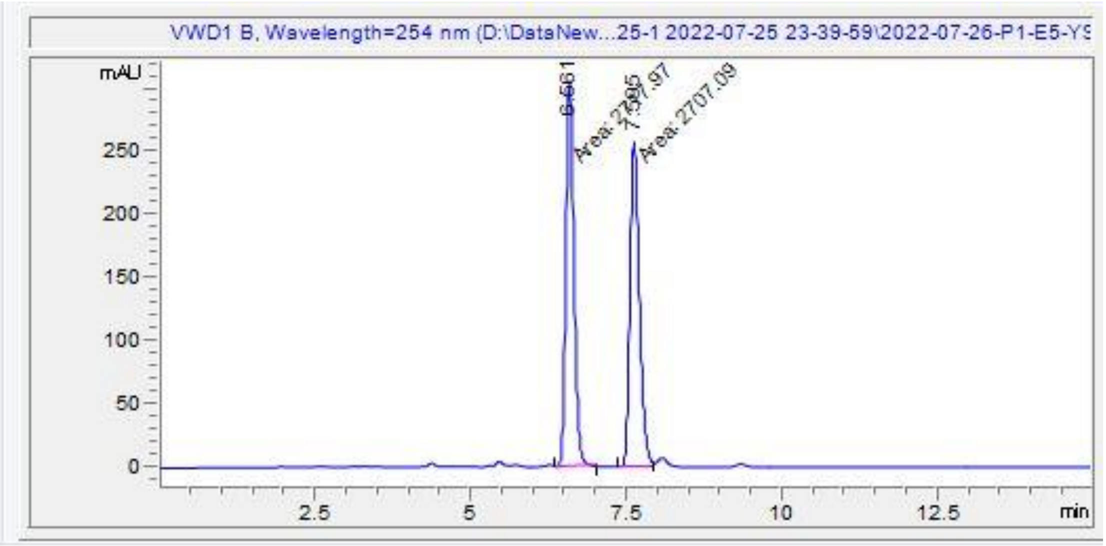

#	Time	Type	Area	Height	Width	Area%	Symmetry
1	6.561	MM	2718	305.8	0.1481	50.100	0.808
2	7.595	MM	2707.1	257.2	0.1754	49.900	0.734

Supplementary Fig. 394 Full HPLC spectrum of racemic **15f**

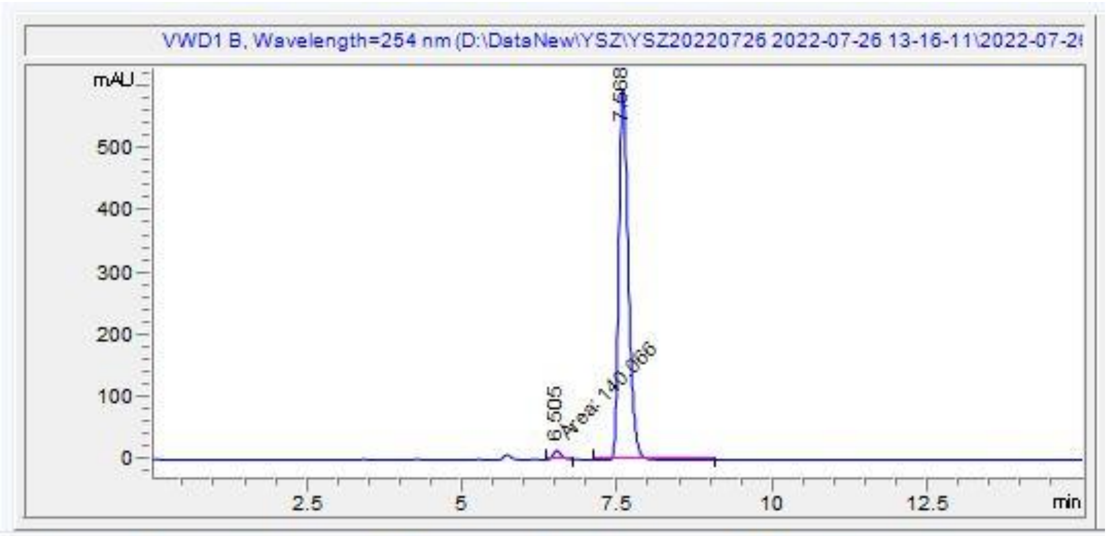

#	Time	Type	Area	Height	Width	Area%	Symmetry
1	6.505	MF	140.1	14.9	0.1569	2.140	0.761
2	7.568	BB	6404	599.9	0.1625	97.860	0.692

Supplementary Fig. 395 Full HPLC spectrum of (S_p)-**15f**

(R_p)-Tert-butyl (1^5-iodo-1,4(1,4)-dibenzenacyclohexaphane-1^2-yl)carbamate **(16a)**

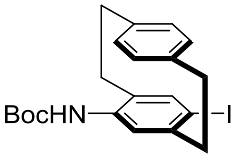

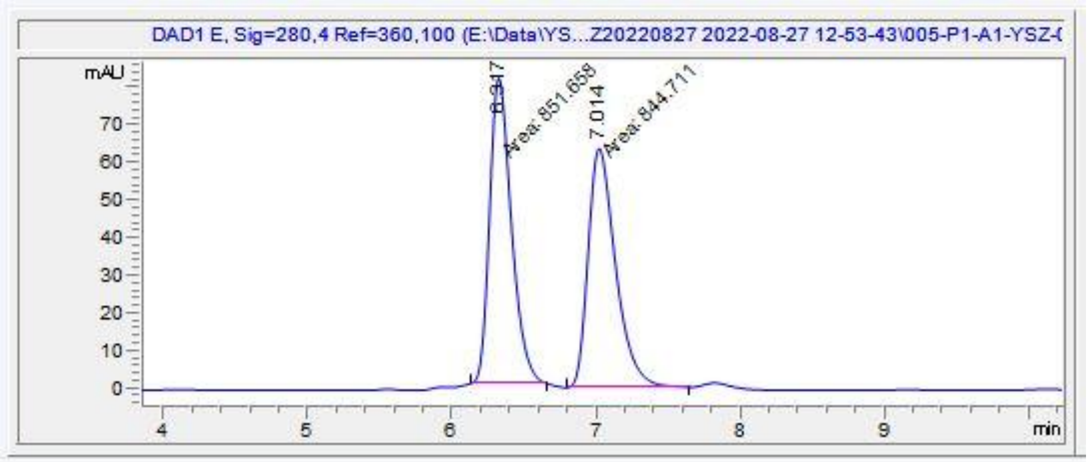

#	Time	Type	Area	Height	Width	Area%	Symmetry
1	6.317	MM	851.7	81.3	0.1746	50.205	0.7
2	7.014	MM	844.7	63.7	0.221	49.795	0.646

Supplementary Fig. 396 HPLC spectrum of racemic **16a**

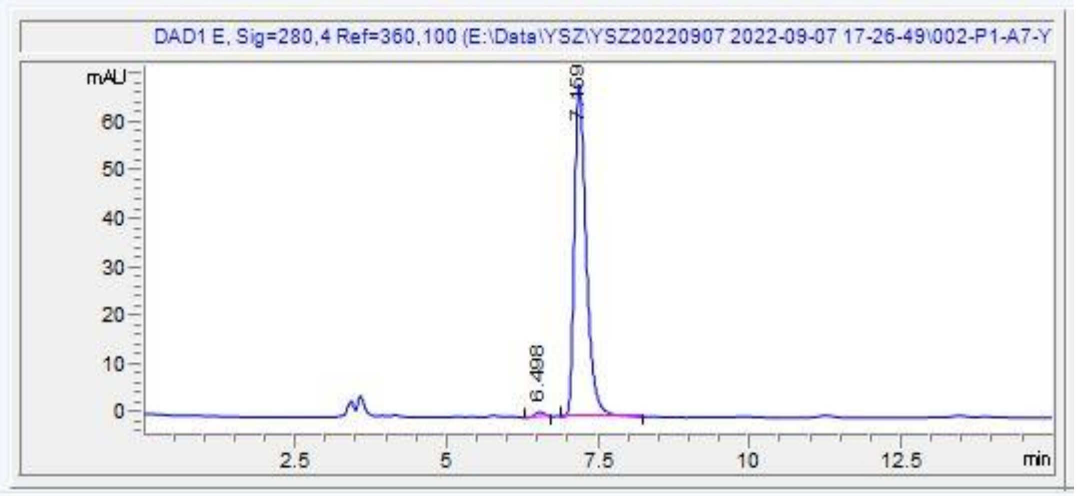

#	Time	Type	Area	Height	Width	Area%	Symmetry
1	6.498	BB	12.7	1.1	0.1823	1.329	0.905
2	7.159	BB	941.8	69.2	0.2068	98.671	0.611

Supplementary Fig. 397 HPLC spectrum of (R_p)**-16a**

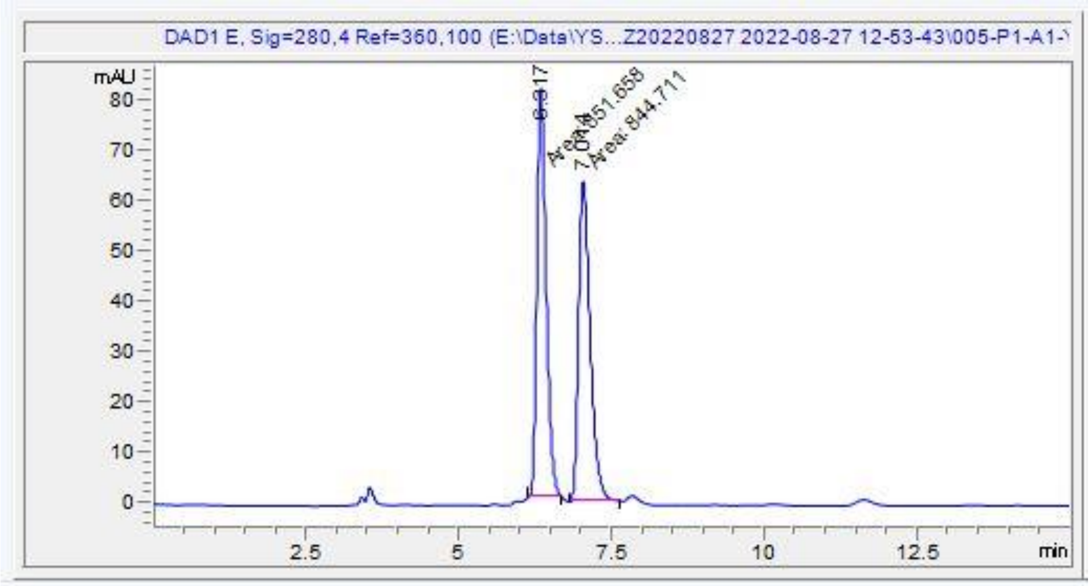

#	Time	Type	Area	Height	Width	Area%	Symmetry
1	6.317	MM	851.7	81.3	0.1746	50.205	0.7
2	7.014	MM	844.7	63.7	0.221	49.795	0.646

Supplementary Fig. 398 Full HPLC spectrum of racemic **16a**

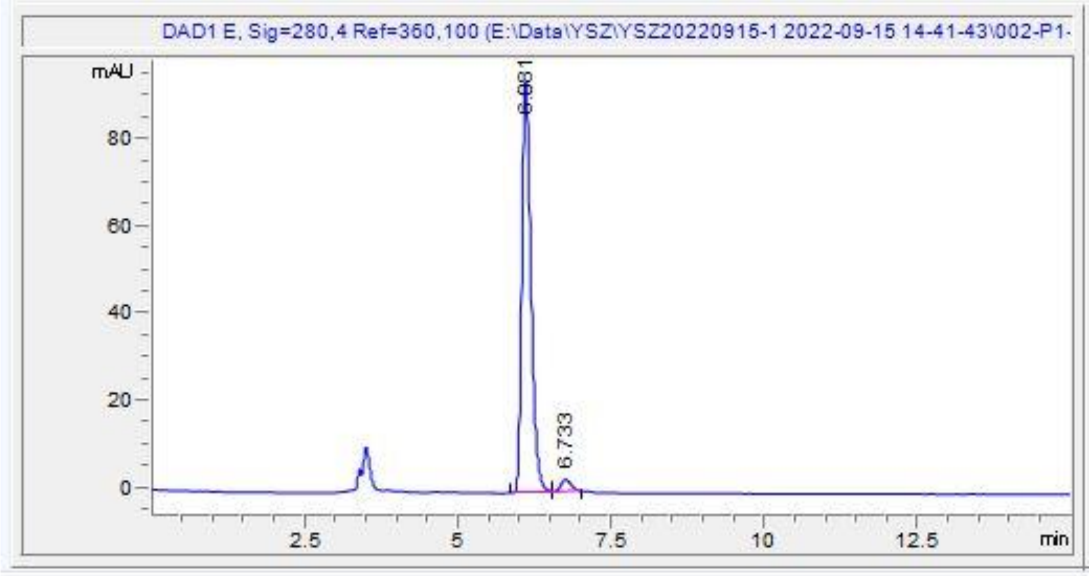

#	Time	Type	Area	Height	Width	Area%	Symmetry
1	6.081	BV	980.6	94.4	0.1591	96.660	0.671
2	6.733	VB	33.9	2.9	0.1797	3.340	0.796

Supplementary Fig. 399 Full HPLC spectrum of (R_p)**-16a**

(R_p)-*N*-benzyl-1[1]H-1(4,7)-indola-4(1,4)-benzenacyclohexaphane-1[2]-carboxamide **(17a)**

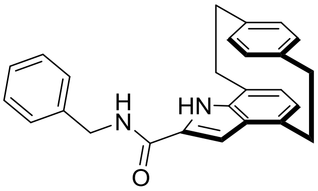

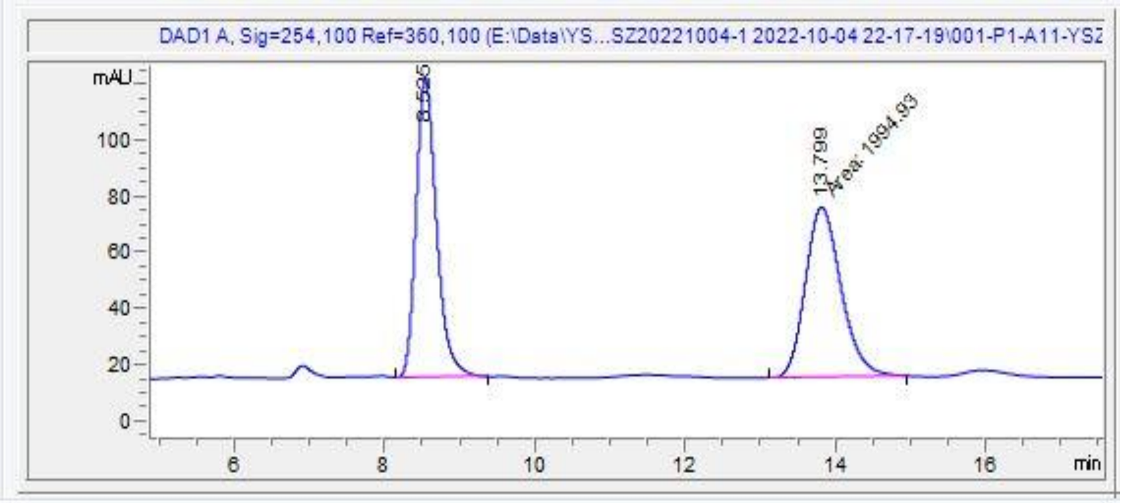

#	Time	Type	Area	Height	Width	Area%	Symmetry
1	8.525	BB	2008.9	106.6	0.2329	50.174	0.726
2	13.799	MM	1994.9	60.3	0.5517	49.826	0.803

Supplementary Fig. 400 HPLC spectrum of racemic **17a**

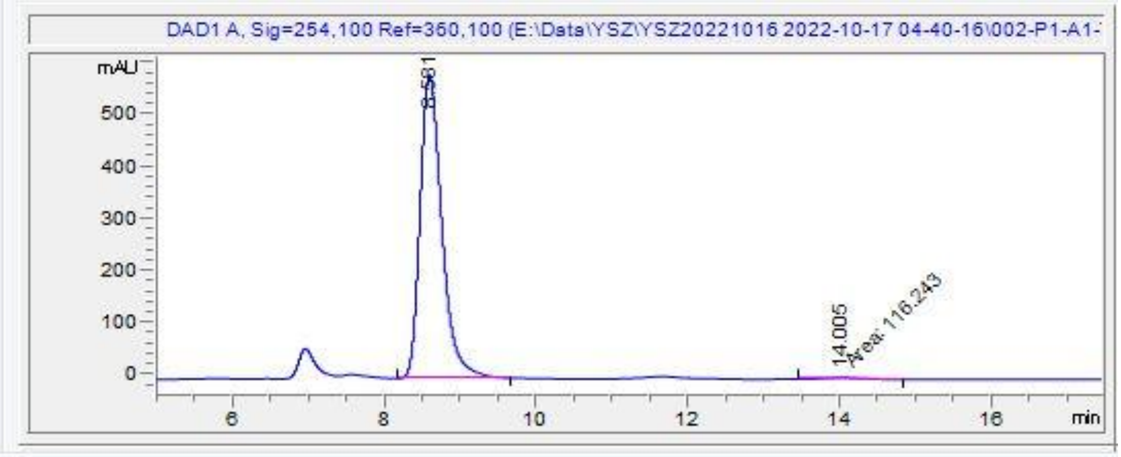

#	Time	Type	Area	Height	Width	Area%	Symmetry
1	8.581	BV R	11638.6	586.9	0.2343	99.011	0.718
2	14.005	MM	116.2	3.4	0.5676	0.989	0.855

Supplementary Fig.401 HPLC spectrum of (R_p)-**17a**

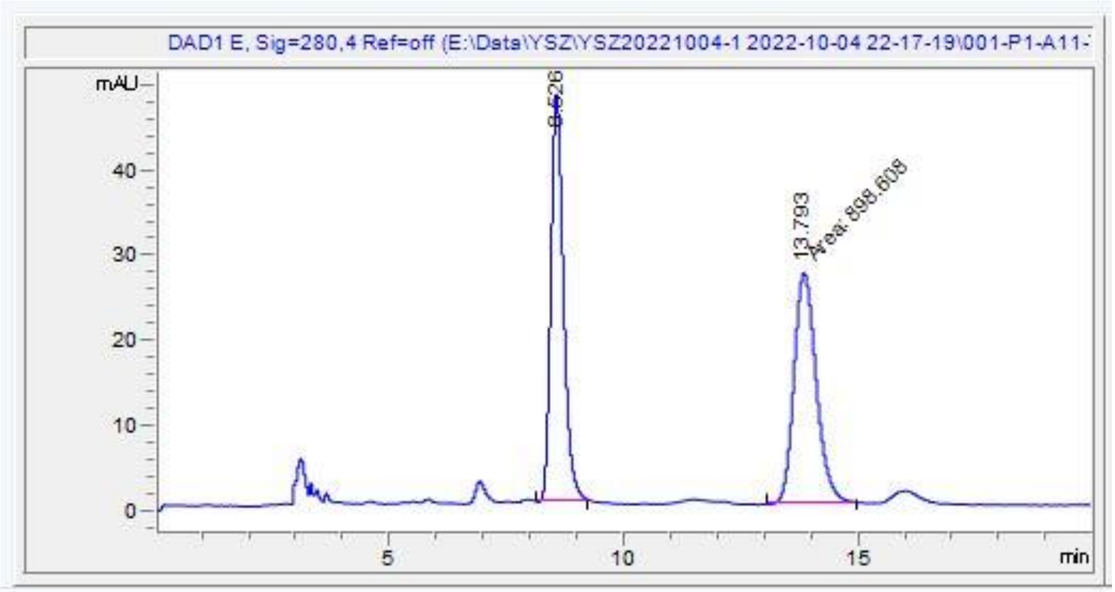

#	Time	Type	Area	Height	Width	Area%	Symmetry
1	8.526	BB	896.7	48	0.2244	49.947	0.776
2	13.793	MM	898.6	27.2	0.5507	50.053	0.775

Supplementary Fig. 402 Full HPLC spectrum of racemic **17a**

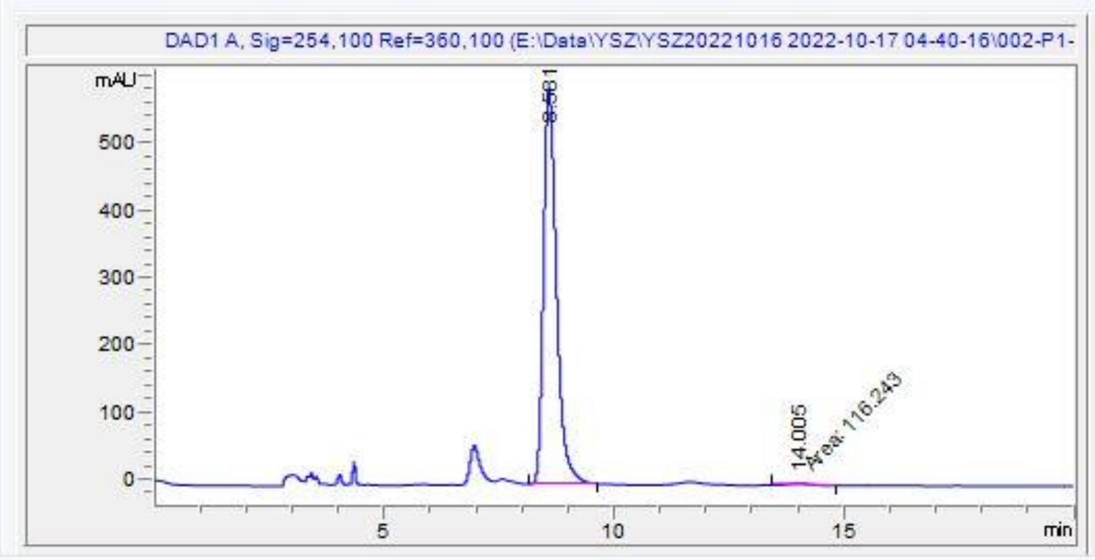

#	Time	Type	Area	Height	Width	Area%	Symmetry
1	8.581	BV R	11638.6	586.9	0.2343	99.011	0.718
2	14.005	MM	116.2	3.4	0.5676	0.989	0.855

Supplementary Fig. 403 Full HPLC spectrum of (R_p)**-17a**

Methyl-2-(benzo[d]thiazol-2-yl)-1-oxo-3-phenyl-1,2,3,4-tetrahydroisoquinoline-4-car boxylate **(23a): 19a** as organocatalyst.

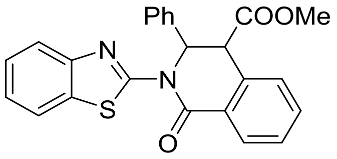

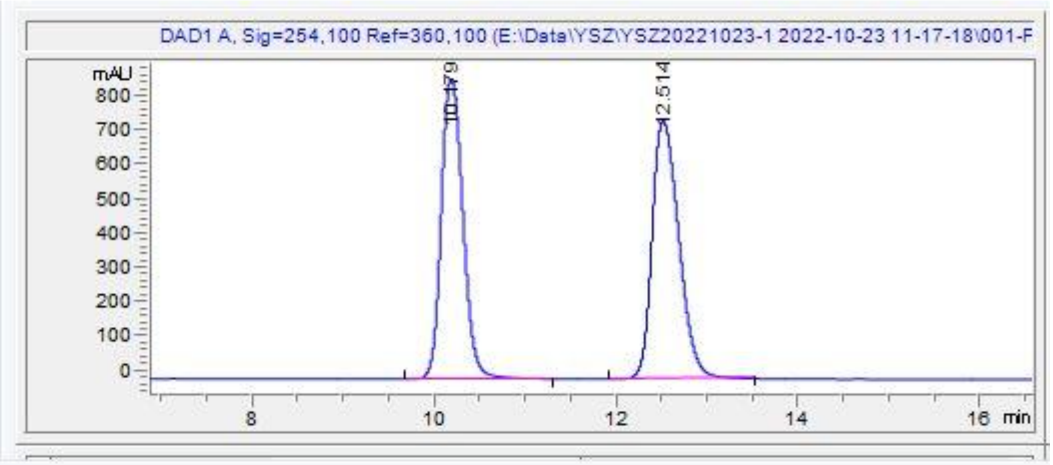

#	Time	Type	Area	Height	Width	Area%	Symmetry
1	10.179	VV R	14530.6	874.8	0.1963	48.584	0.757
2	12.514	VB R	15377.9	752.7	0.2394	51.416	0.659

Supplementary Fig. 404 HPLC spectrum of racemic **23a**

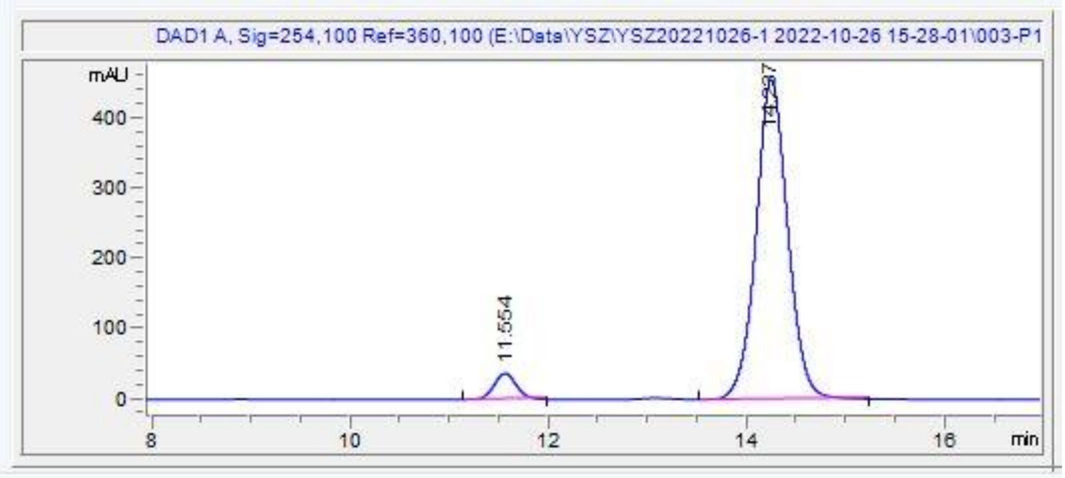

#	Time	Type	Area	Height	Width	Area%	Symmetry
1	11.554	VV R	590.8	37.5	0.1856	5.498	0.909
2	14.237	VV R	10154.7	457.6	0.2604	94.502	0.839

Supplementary Fig. 405 HPLC spectrum of **23a**

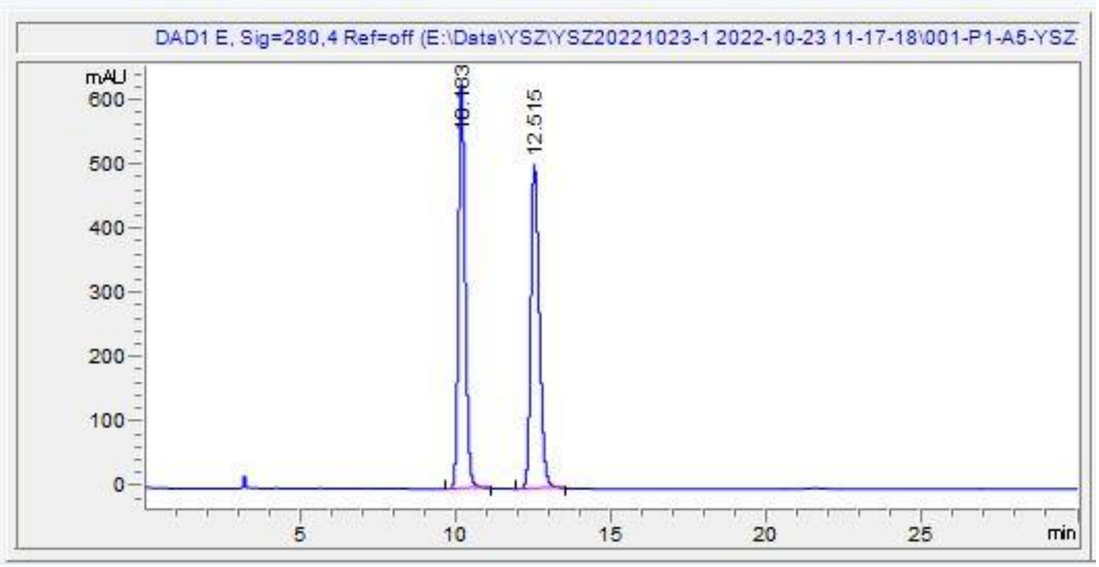

#	Time	Type	Area	Height	Width	Area%	Symmetry
1	10.183	BB	9365.4	625.8	0.2216	49.516	0.802
2	12.515	VB R	9548.4	502.4	0.2882	50.484	0.661

Supplementary Fig. 406 Full HPLC spectrum of racemic **23a**

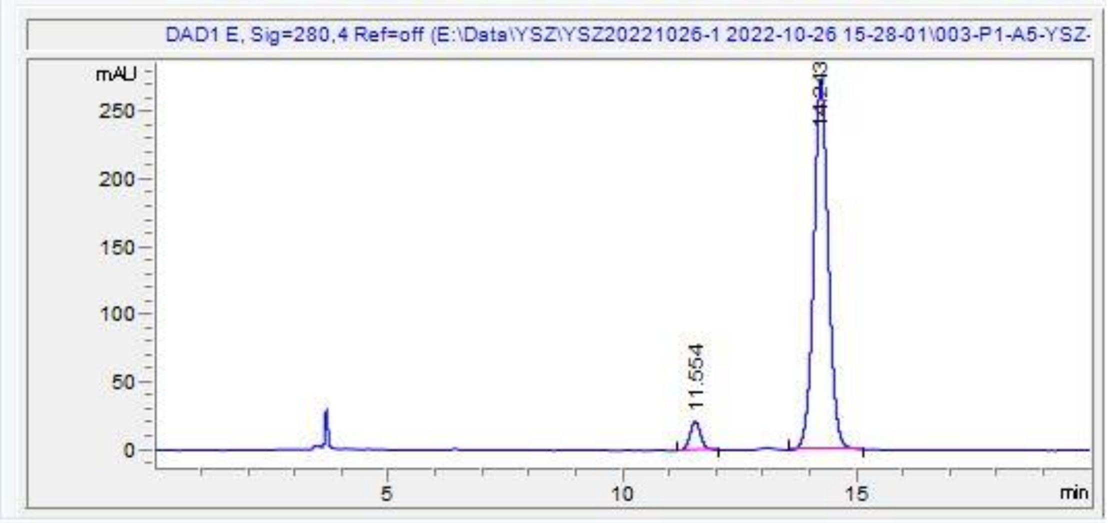

#	Time	Type	Area	Height	Width	Area%	Symmetry
1	11.554	VV R	338.8	21.3	0.1888	5.425	0.891
2	14.243	BB	5905.6	272.5	0.3094	94.575	0.9

Supplementary Fig. 407 Full HPLC spectrum of **23a**

Methyl-2-(benzo[d]thiazol-2-yl)-1-oxo-3-phenyl-1,2,3,4-tetrahydroisoquinoline-4-carboxylate **(23a): 20a** as organocatalyst.

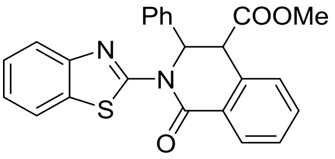

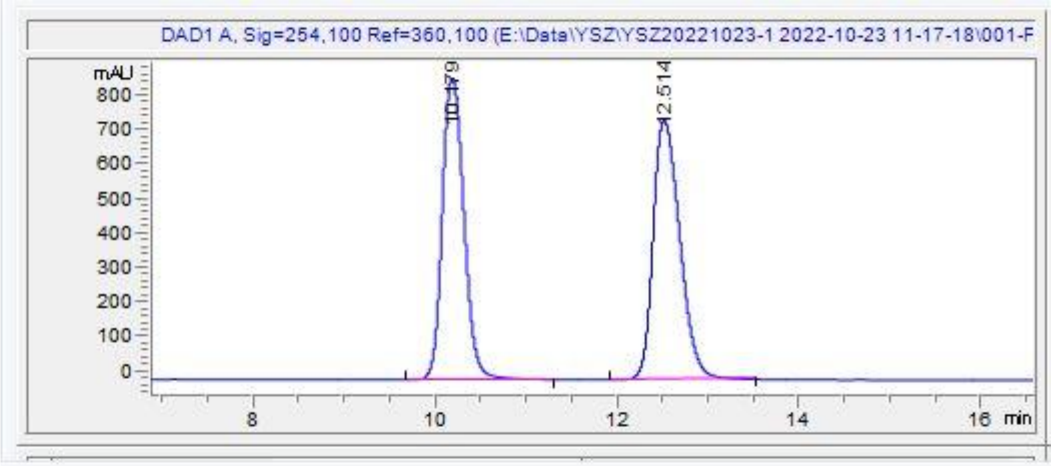

#	Time	Type	Area	Height	Width	Area%	Symmetry
1	10.179	VV R	14530.6	874.8	0.1963	48.584	0.757
2	12.514	VB R	15377.9	752.7	0.2394	51.416	0.659

Supplementary Fig. 408 HPLC spectrum of racemic **23a**

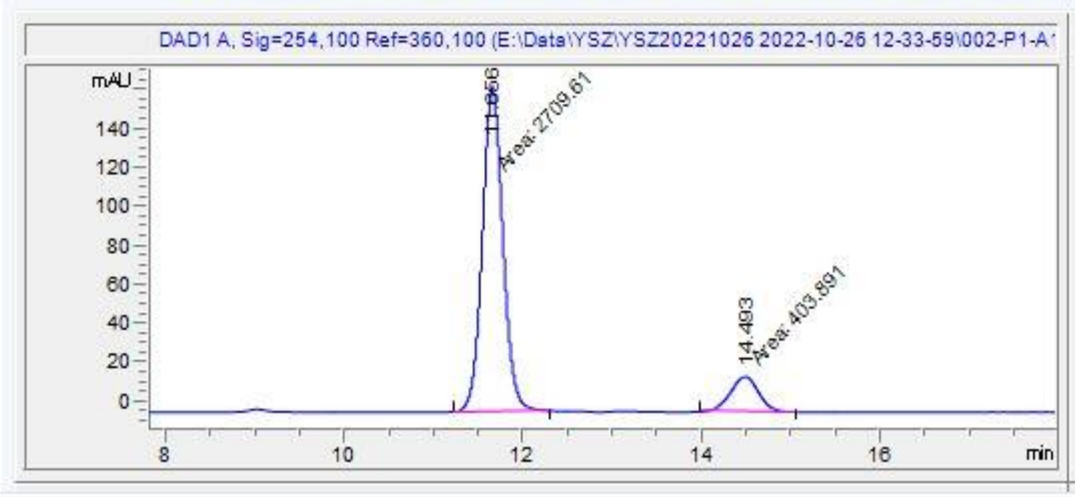

#	Time	Type	Area	Height	Width	Area%	Symmetry
1	11.656	MM	2709.6	167.6	0.2695	87.028	0.918
2	14.493	MM	403.9	18.2	0.3695	12.972	1.039

Supplementary Fig. 409 HPLC spectrum of **23a**

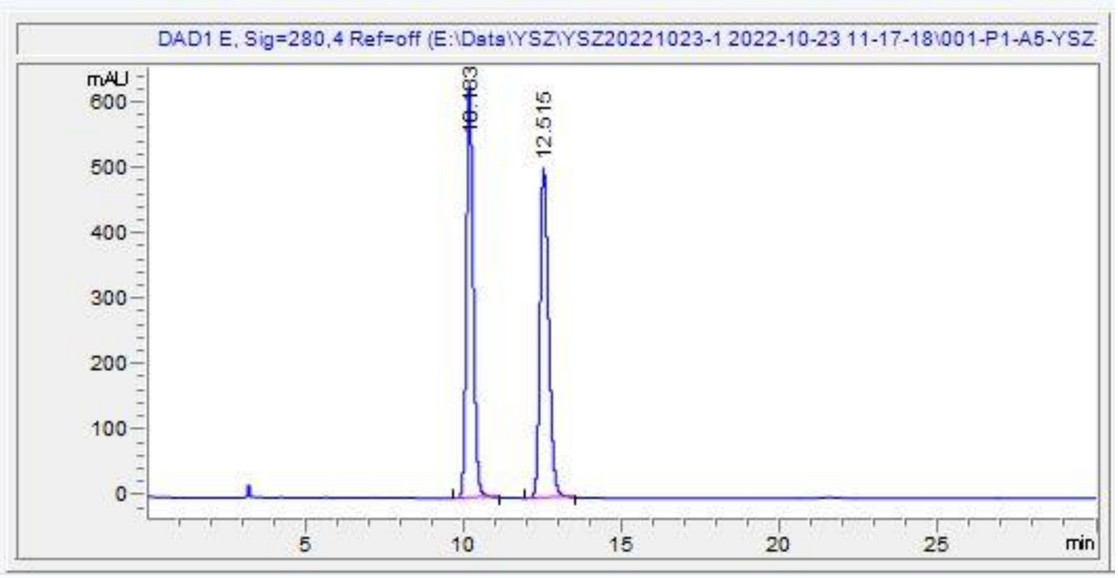

#	Time	Type	Area	Height	Width	Area%	Symmetry
1	10.183	BB	9365.4	625.8	0.2216	49.516	0.802
2	12.515	VB R	9548.4	502.4	0.2882	50.484	0.661

Supplementary Fig. 410 Full HPLC spectrum of racemic **23a**

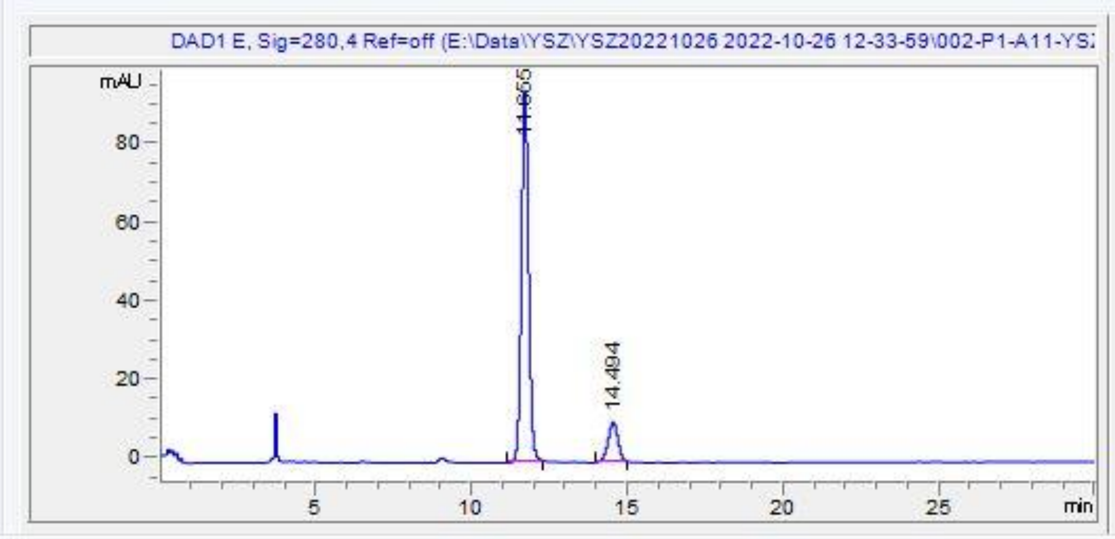

#	Time	Type	Area	Height	Width	Area%	Symmetry
1	11.655	BV R	1519.8	94.9	0.2284	86.954	0.902
2	14.494	VV R	228	10.2	0.2617	13.046	1.104

Supplementary Fig. 411 Full HPLC spectrum of **23a**

Methyl-2-(benzo[d]thiazol-2-yl)-1-oxo-3-phenyl-1,2,3,4-tetrahydroisoquinoline-4-carboxylate **(23a): 24a** as organocatalyst.

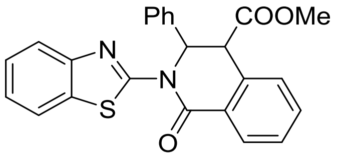

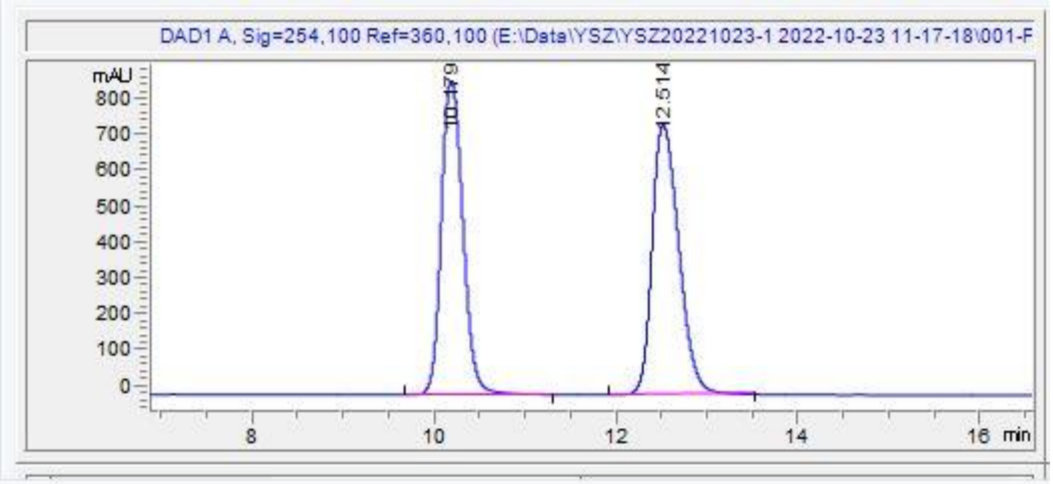

#	Time	Type	Area	Height	Width	Area%	Symmetry
1	10.179	VV R	14530.6	874.8	0.1963	48.584	0.757
2	12.514	VB R	15377.9	752.7	0.2394	51.416	0.659

Supplementary Fig. 412 HPLC spectrum of racemic **23a**

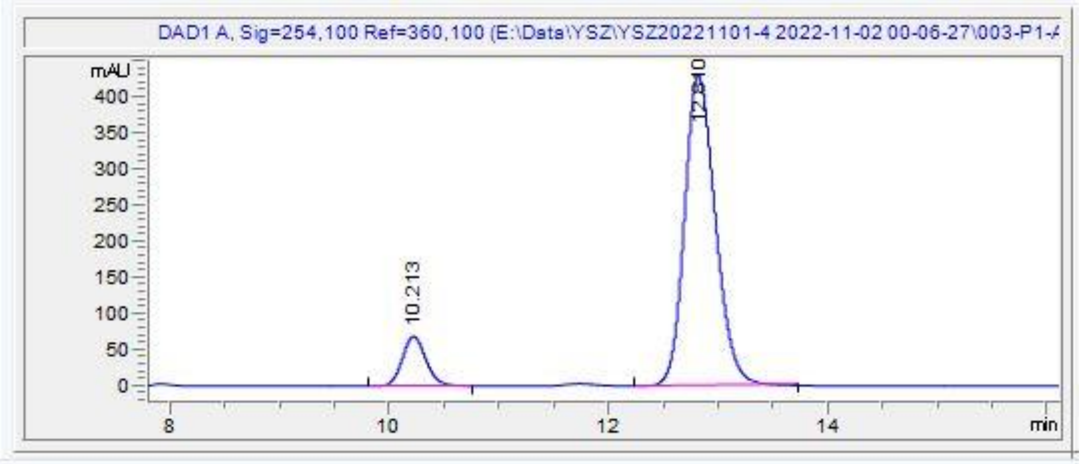

#	Time	Type	Area	Height	Width	Area%	Symmetry
1	10.213	BV R	1036.4	69	0.1848	10.941	0.851
2	12.81	VV R	8436.1	429.5	0.2341	89.059	0.73

Supplementary Fig. 413 HPLC spectrum of **23a**

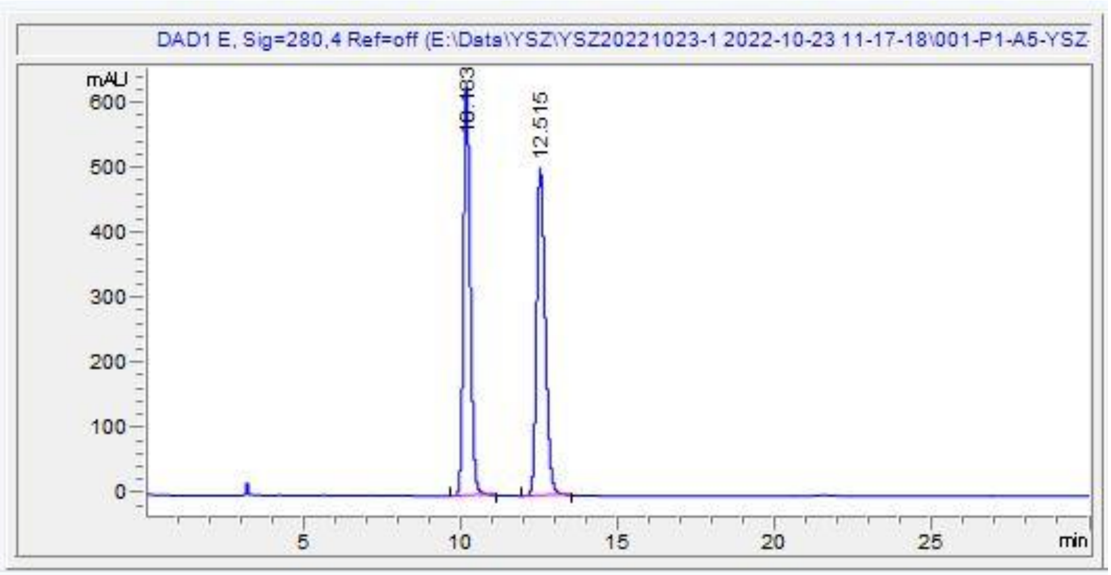

#	Time	Type	Area	Height	Width	Area%	Symmetry
1	10.183	BB	9365.4	625.8	0.2216	49.516	0.802
2	12.515	VB R	9548.4	502.4	0.2882	50.484	0.661

Supplementary Fig. 414 Full HPLC spectrum of racemic **23a**

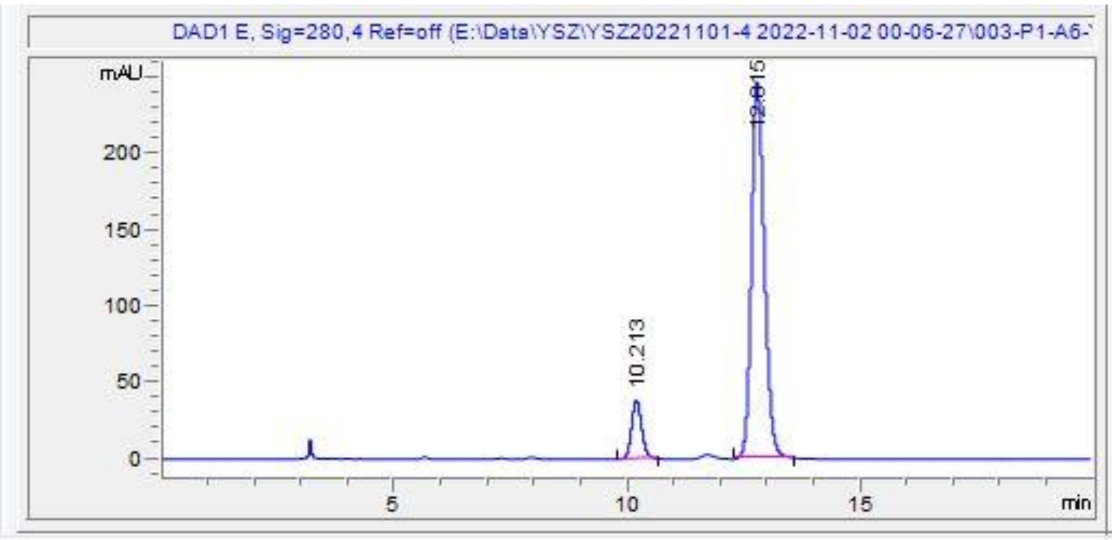

#	Time	Type	Area	Height	Width	Area%	Symmetry
1	10.213	BV R	580.2	38.8	0.1914	10.776	0.878
2	12.815	BV R	4803.7	247.8	0.2877	89.224	0.779

Supplementary Fig. 415 Full HPLC spectrum of **23a**

Methyl-2-(benzo[d]thiazol-2-yl)-1-oxo-3-phenyl-1,2,3,4-tetrahydroisoquinoline-4-carboxylate **(23a): 24b** as organocatalyst.

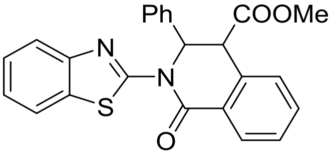

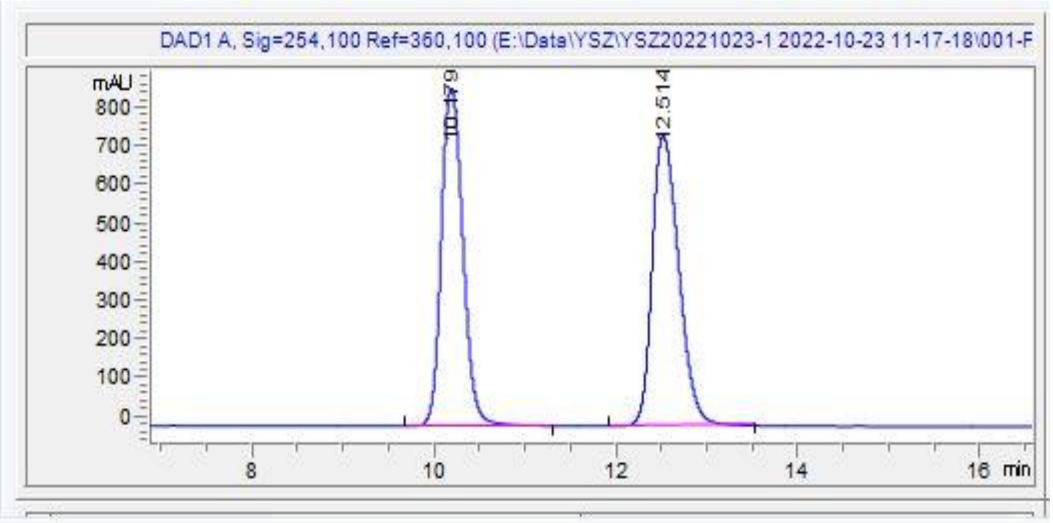

#	Time	Type	Area	Height	Width	Area%	Symmetry
1	10.179	VV R	14530.6	874.8	0.1963	48.584	0.757
2	12.514	VB R	15377.9	752.7	0.2394	51.416	0.659

Supplementary Fig. 416 HPLC spectrum of racemic **23a**

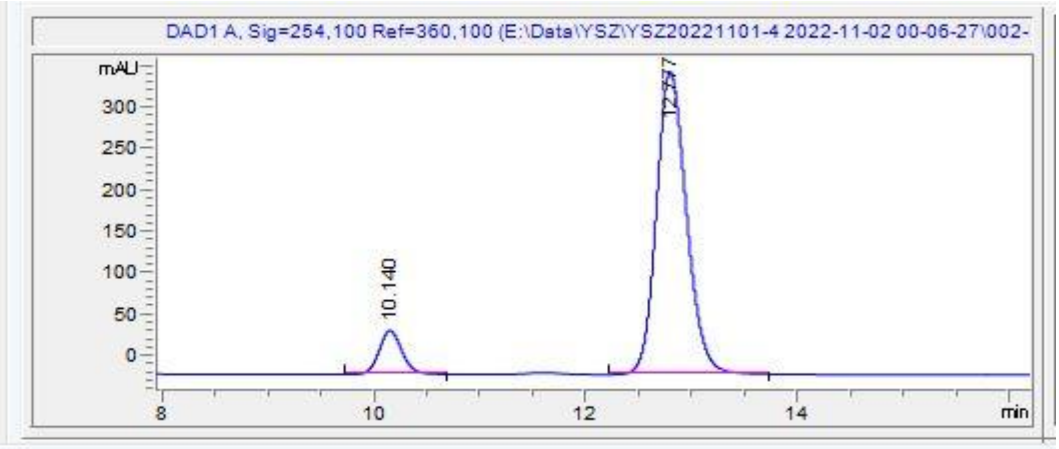

#	Time	Type	Area	Height	Width	Area%	Symmetry
1	10.14	BV R	797.9	53.2	0.1839	9.879	0.897
2	12.777	VV R	7279.1	368.7	0.2369	90.121	0.775

Supplementary Fig. 417 HPLC spectrum of **23a**

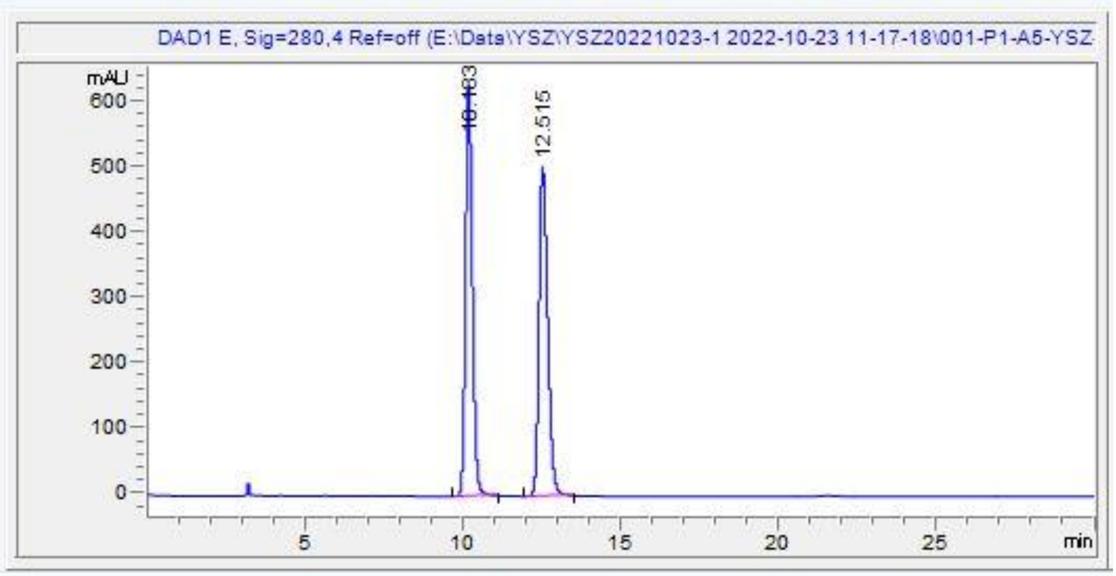

#	Time	Type	Area	Height	Width	Area%	Symmetry
1	10.183	BB	9365.4	625.8	0.2216	49.516	0.802
2	12.515	VB R	9548.4	502.4	0.2882	50.484	0.661

Supplementary Fig. 418 Full HPLC spectrum of racemic **23a**

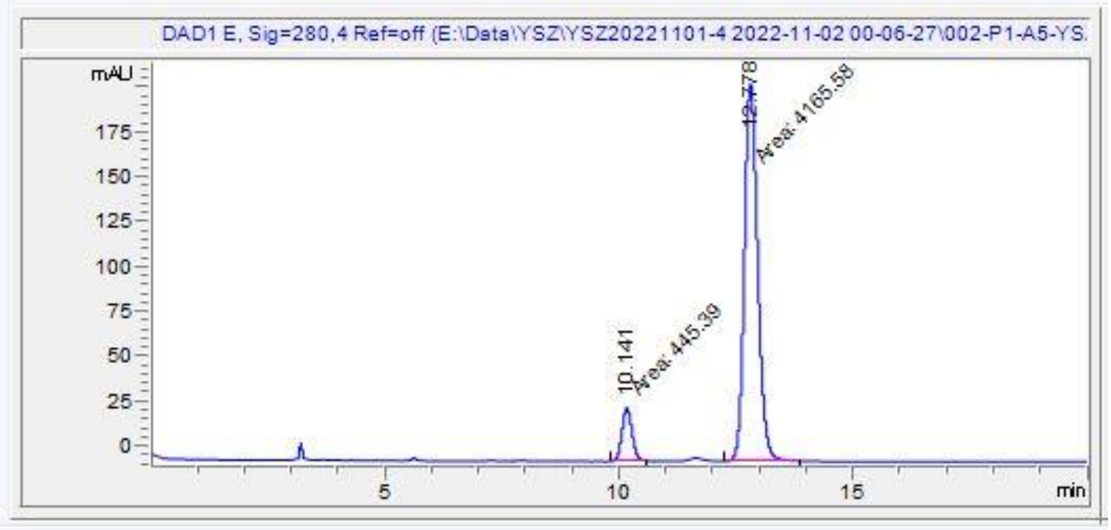

#	Time	Type	Area	Height	Width	Area%	Symmetry
1	10.141	MM	445.4	29.9	0.2481	9.659	0.92
2	12.778	MM	4165.6	211.6	0.328	90.341	0.795

Supplementary Fig. 419 Full HPLC spectrum of **23a**

2.3 NMR spectra

(R_p)-Tert-butyl 1,4(1,4)-dibenzenacyclohexaphane-1^2-ylcarbamate ((R_p)-**1a**)

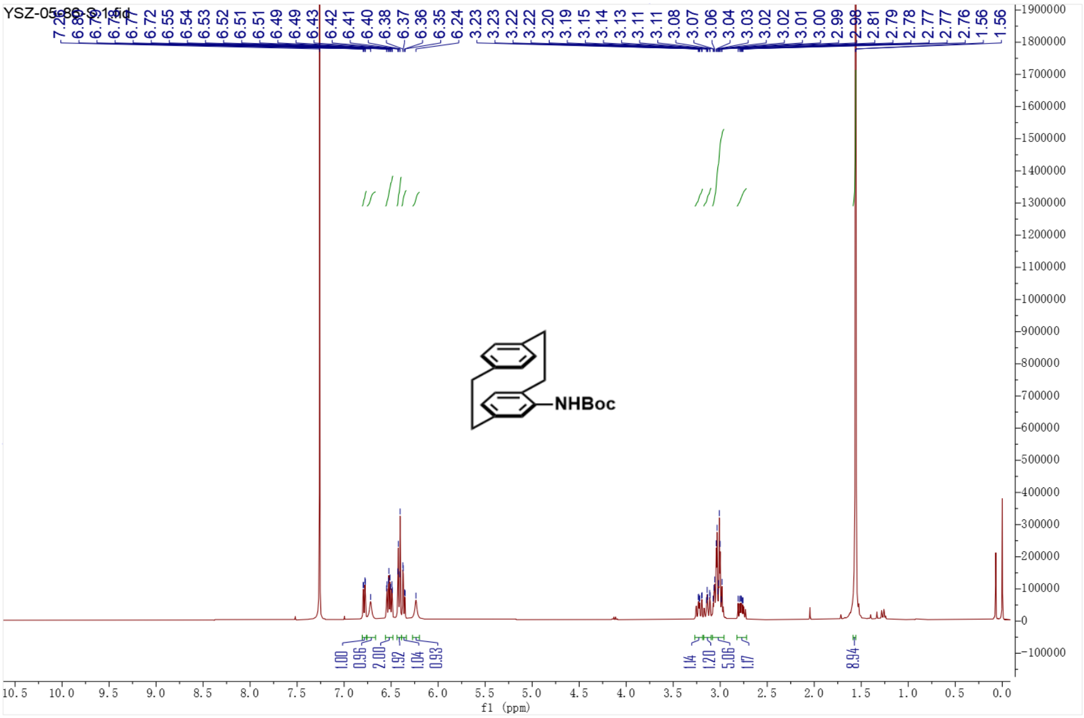

Supplementary Fig. 420 ^{1}H NMR spectrum of (R_p)-**1a** (400 MHz, $CDCl_3$)

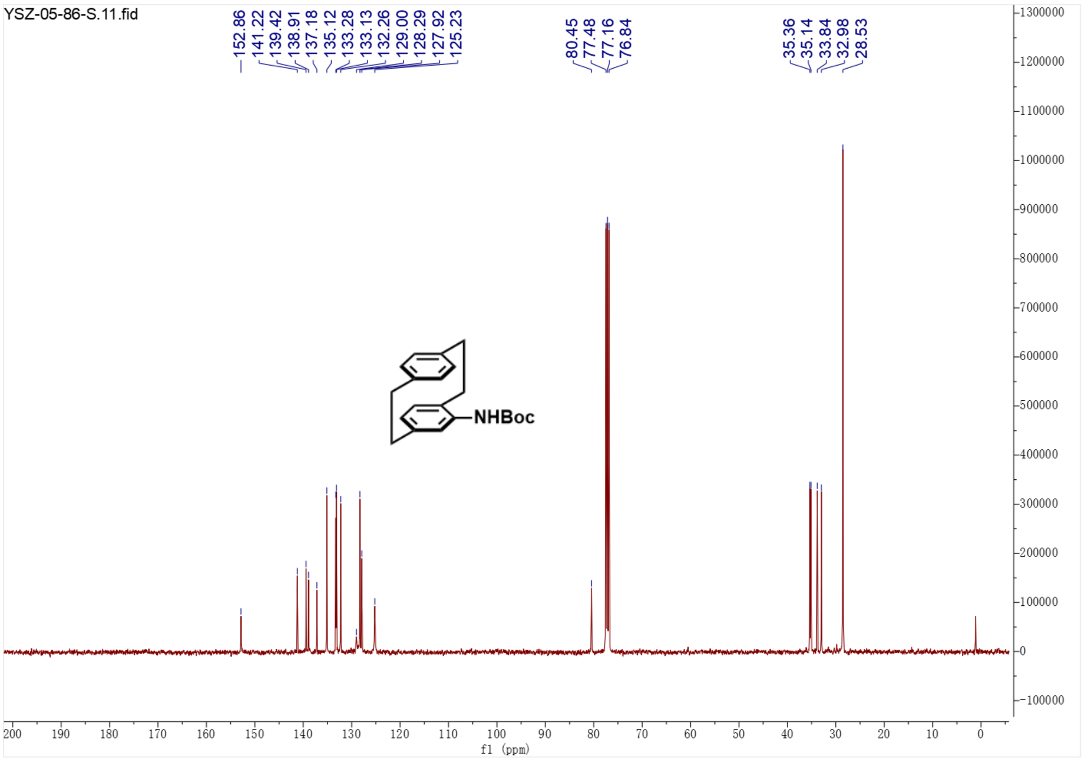

Supplementary Fig. 421 ^{13}C NMR spectrum of (R_p)-**1a** (101 MHz, $CDCl_3$)

(S_p)-Dibenzyl-1-(1[5]-((tert-butoxycarbonyl)amino)-1,4(1,4)-dibenzenacyclohexaphane-1[2]-yl)hydrazine-1,2-dicarboxylate **(3a)**

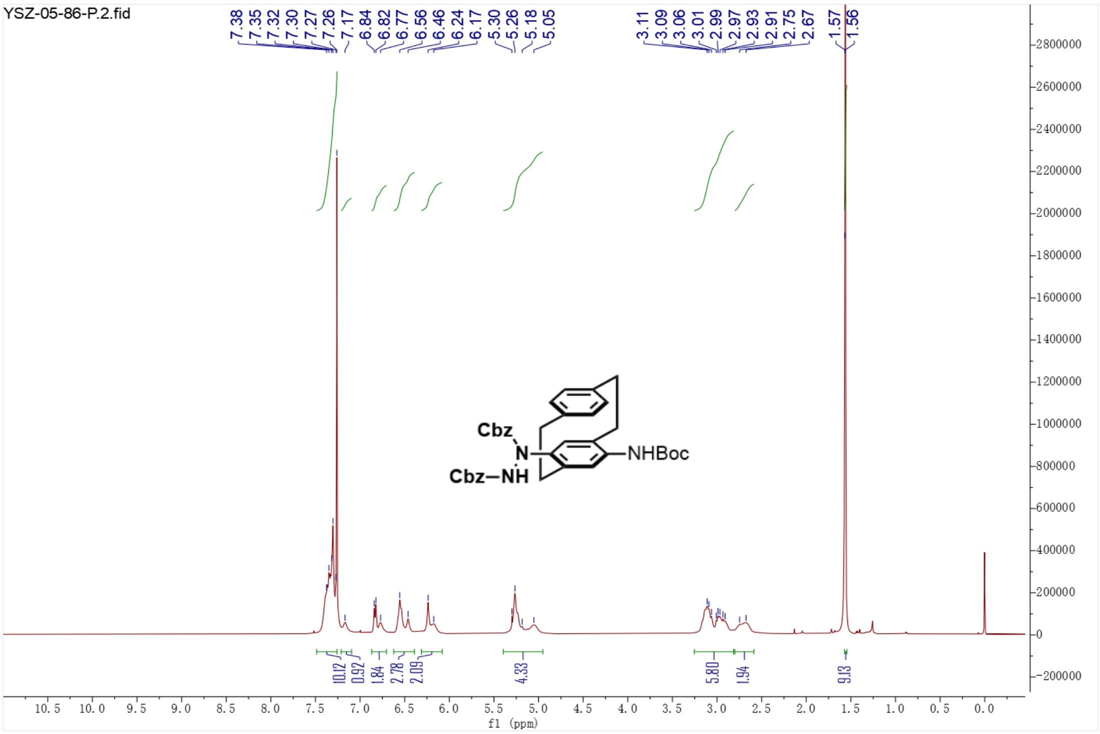

Supplementary Fig. 422 ^{1}H NMR spectrum of (S_p)-**3a** (400 MHz, $CDCl_3$)

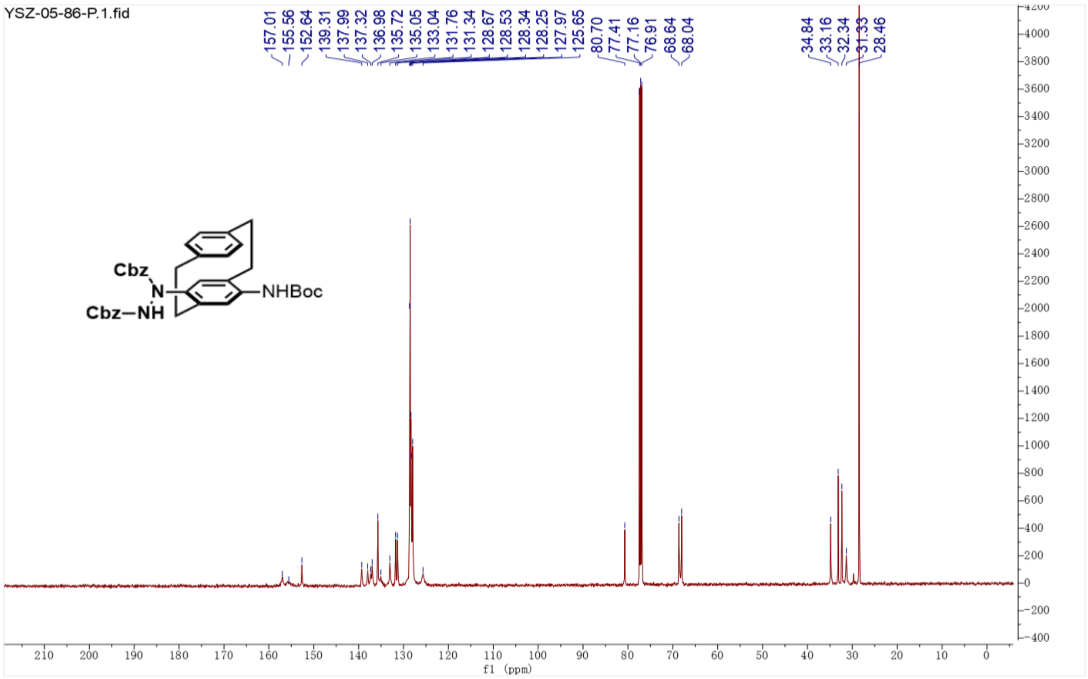

Supplementary Fig. 423 ^{13}C NMR spectrum of (S_p)-**3a** (126 MHz, $CDCl_3$)

^{1}H NMR of **3a** at different temperatures

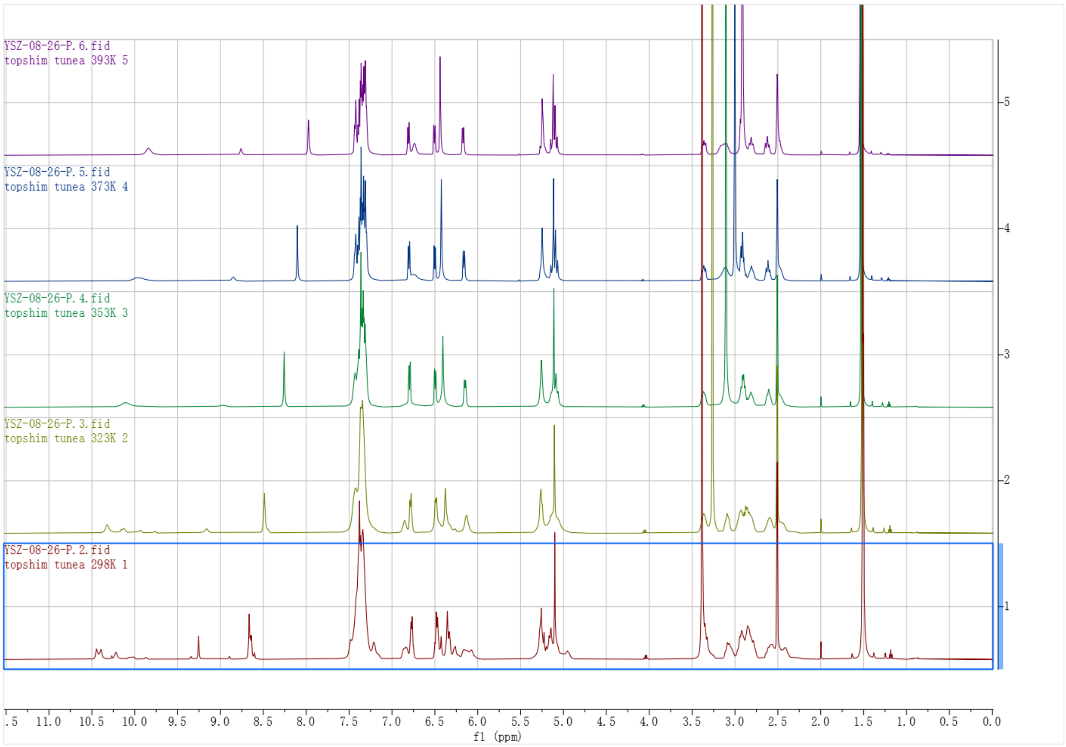

Supplementary Fig. 424 Full ^{1}H NMR spectrum of (S_p)-**3a** (500 MHz, DMSO-d6)

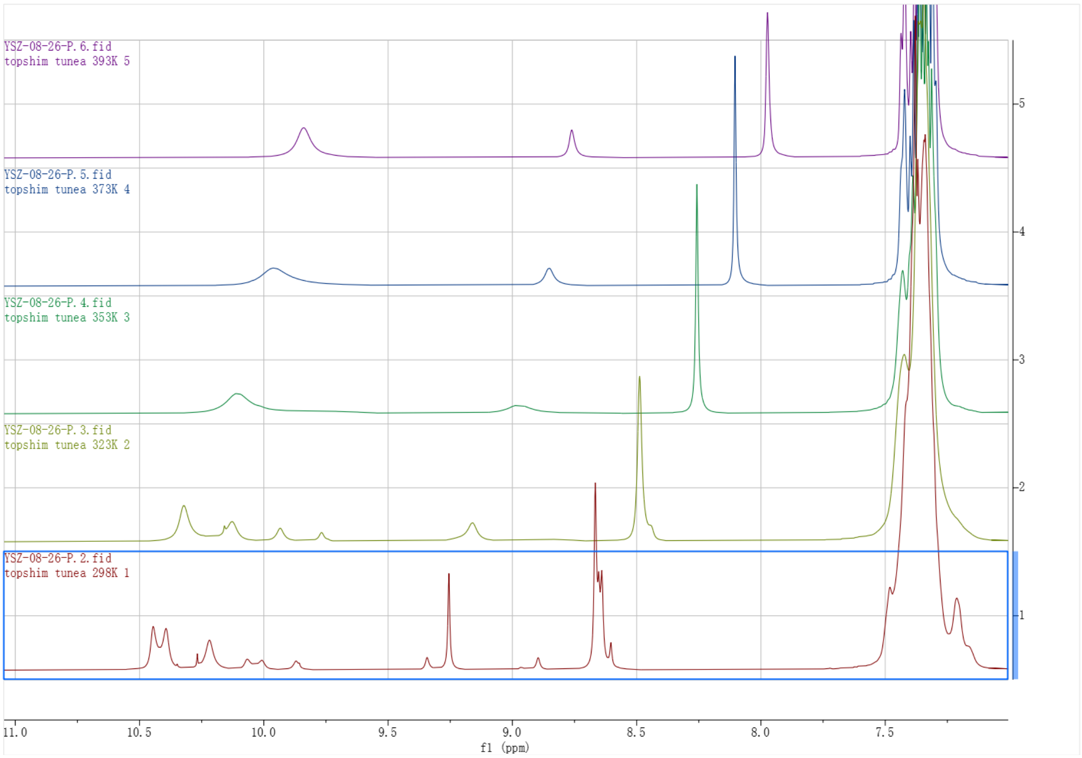

Supplementary Fig. 425 ^{1}H NMR spectrum of (S_p)-**3a** (500 MHz, DMSO-d6) in 7-11 ppm

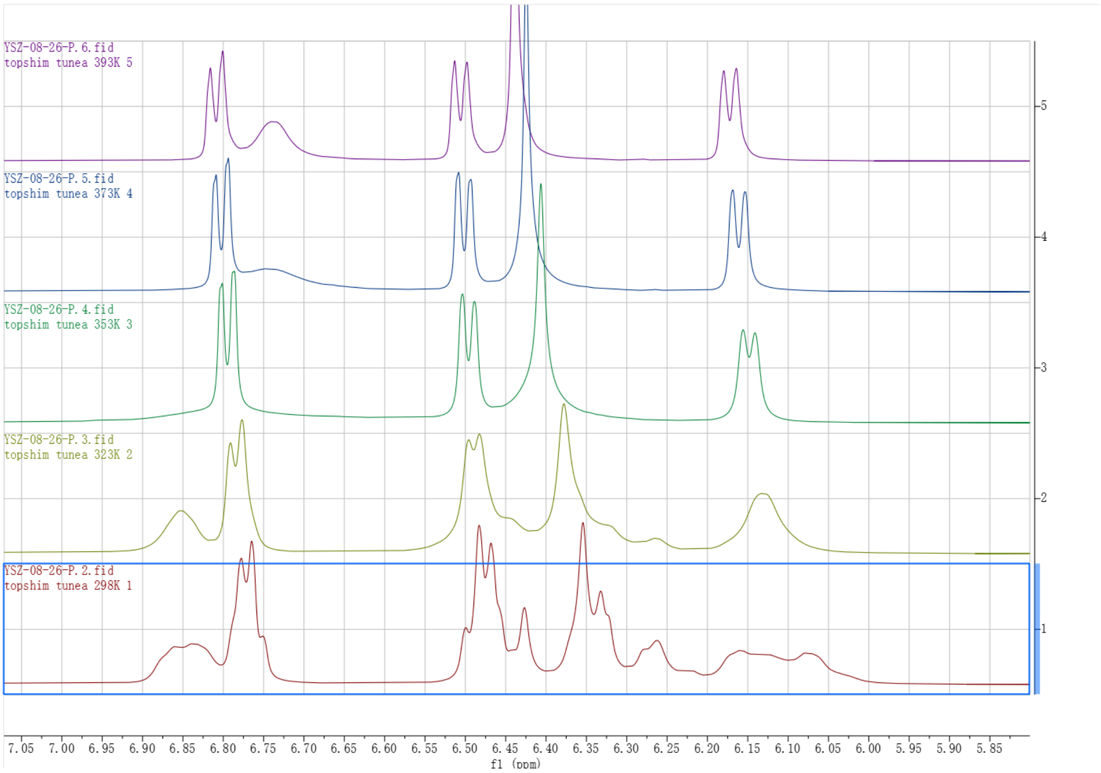

Supplementary Fig. 426 ^{1}H NMR spectrum of (S_p)-**3a** (500 MHz, DMSO-d6) in 5.8-7 ppm

(*R_p*)-Methyl 1,4(1,4)-dibenzenacyclohexaphane-1²-ylcarbamate **((*R_p*)-1b)**

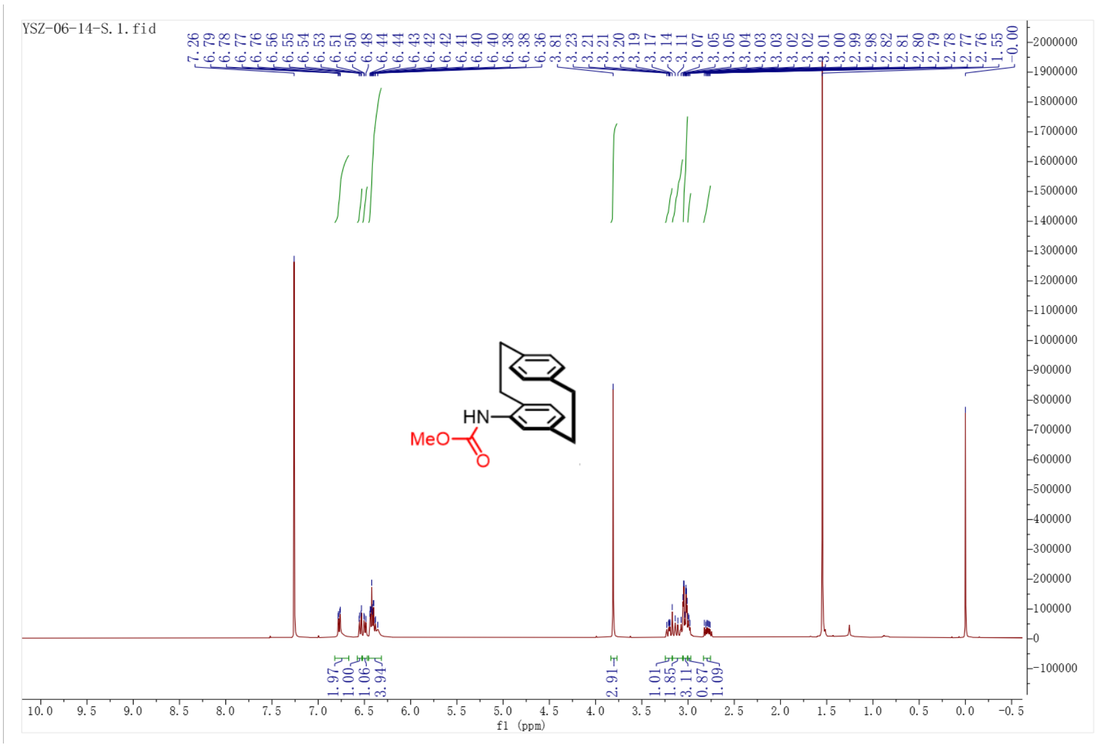

Supplementary Fig. 427 ^{1}H NMR spectrum of (*R_p*)-**1b** (400 MHz, $CDCl_3$)

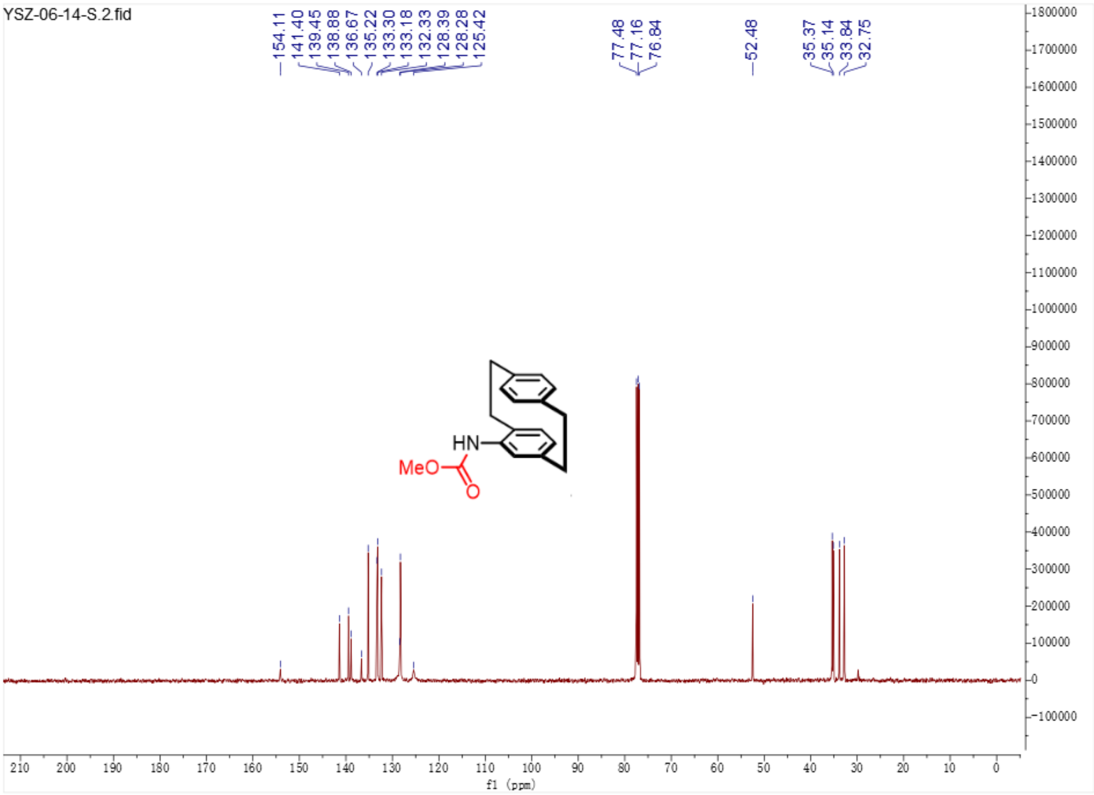

Supplementary Fig. 428 ^{13}C NMR spectrum of (*R_p*)-**1b** (101 MHz, DMSO-d6)

(S_p)-Dibenzyl 1-(1^5-((methoxycarbonyl)amino)-1,4(1,4)-dibenzenacyclohexaphane-1^2-yl)hydrazine-1,2-dicarboxylate **(3b)**

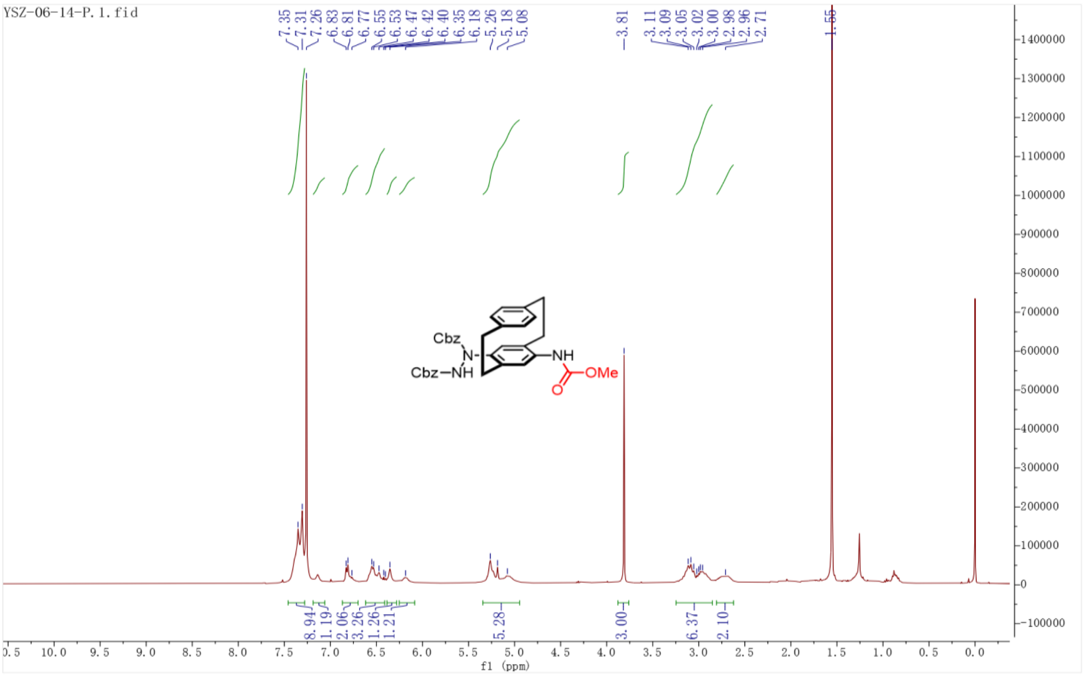

Supplementary Fig. 429 ^{1}H NMR spectrum of (S_p)-**3b** (400 MHz, $CDCl_3$)

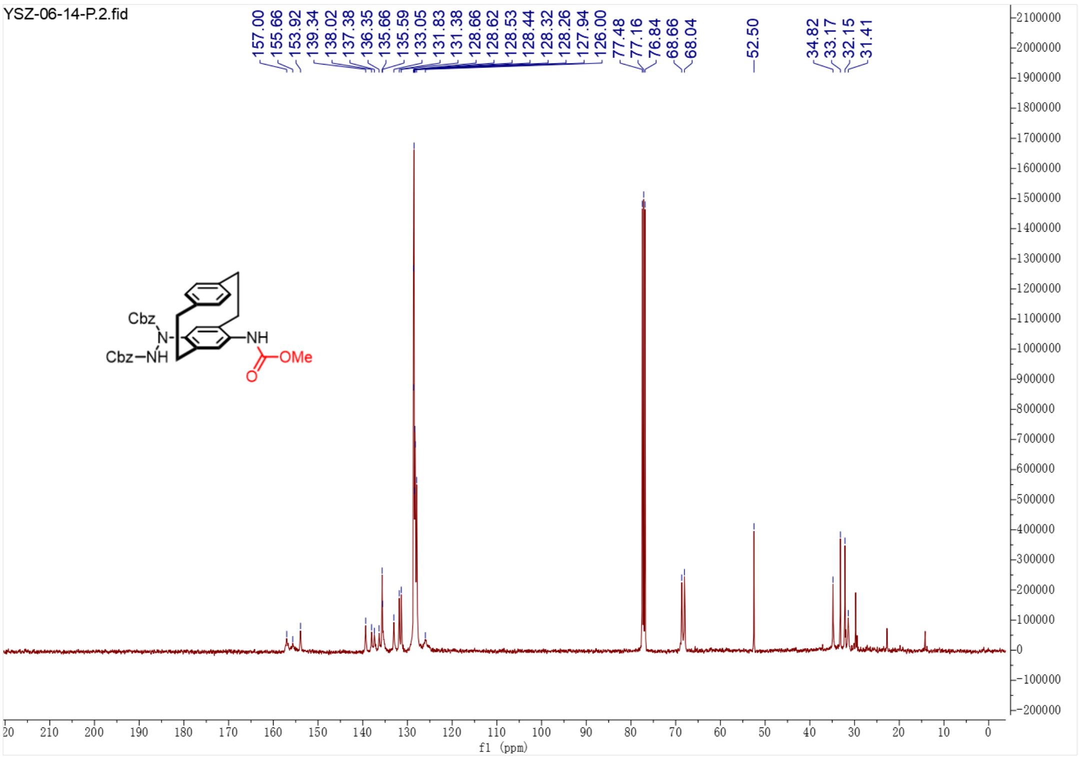

Supplementary Fig. 430 ^{13}C NMR spectrum of (S_p)-**3b** (101 MHz, $CDCl_3$)

(R_p)-Ethyl 1,4(1,4)-dibenzenacyclohexaphane-1^2-ylcarbamate **((R_p)-1c)**

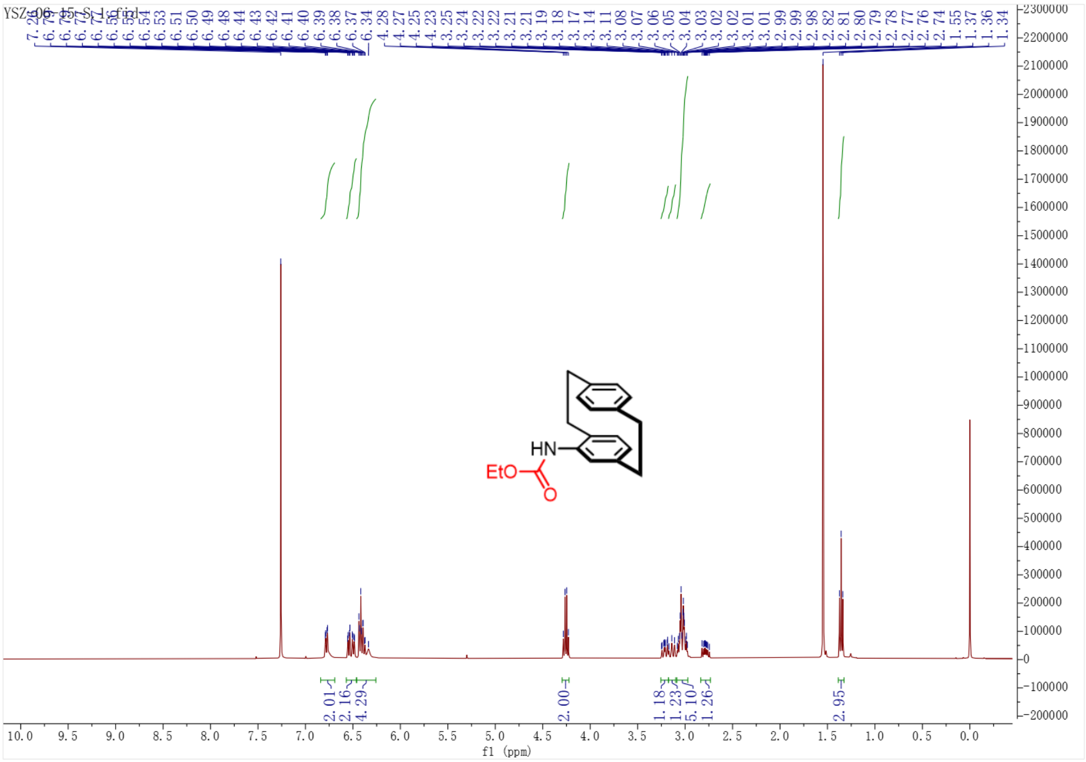

Supplementary Fig. 431 ^{1}H NMR spectrum of (R_p)**-1c** (400 MHz, $CDCl_3$)

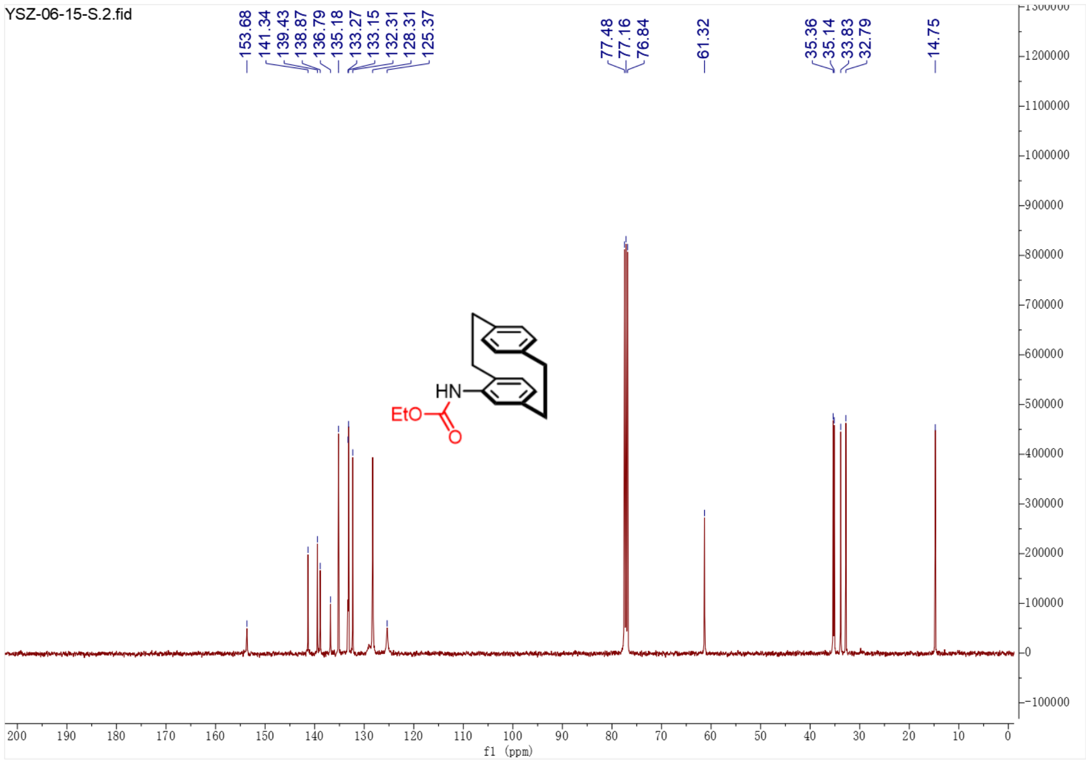

Supplementary Fig. 432 ^{13}C NMR spectrum of (S_p)**-3b** (101 MHz, $CDCl_3$)

(S_p)-Dibenzyl 1-(1^5-((ethoxycarbonyl)amino)-1,4(1,4)-dibenzenacyclohexaphane-1^2-yl)hydrazine-1,2-dicarboxylate **(3c)**

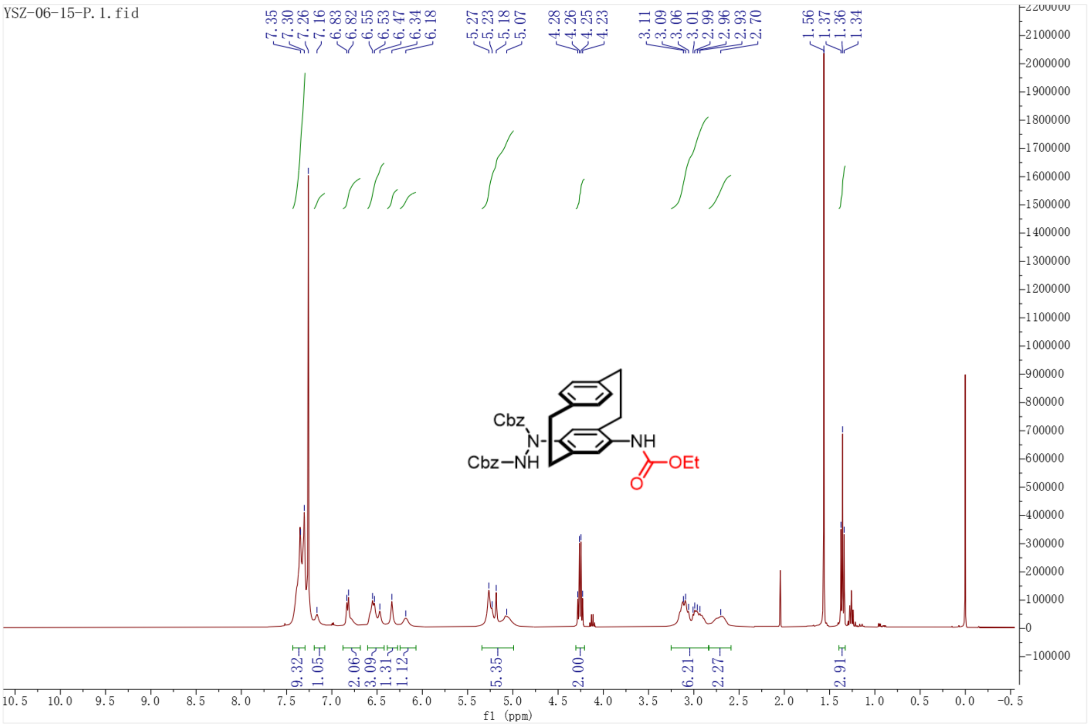

Supplementary Fig. 433 ^{1}H NMR spectrum of (S_p)-**3c** (400 MHz, $CDCl_3$)

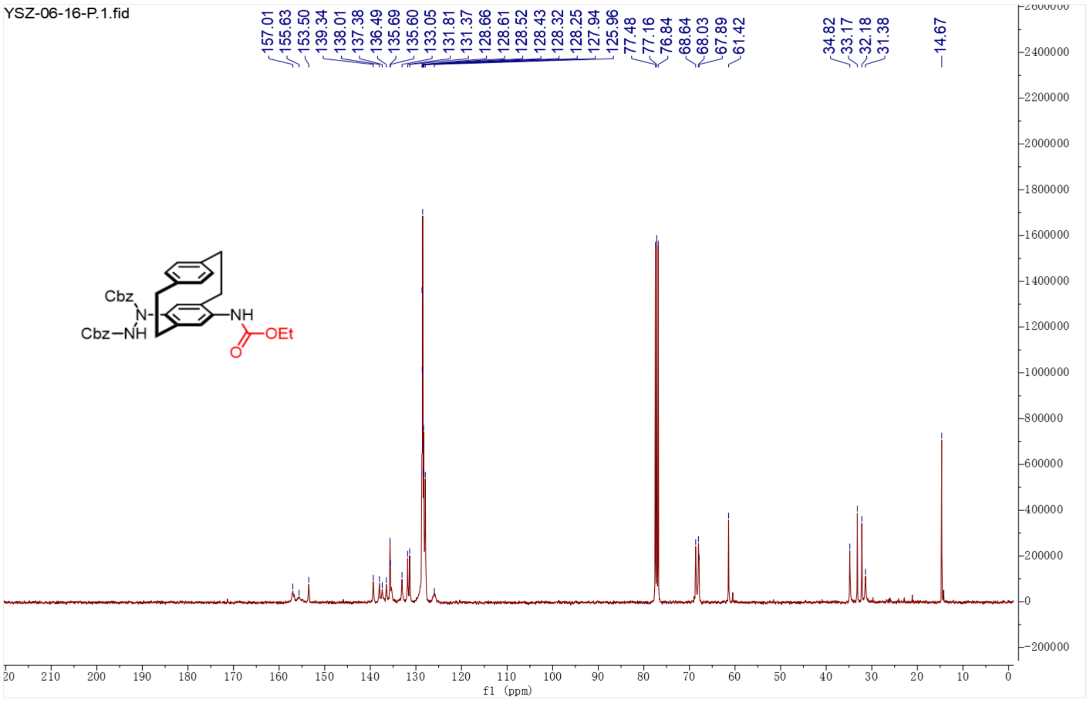

Supplementary Fig. 434 ^{13}C NMR spectrum of (S_p)-**3c** (101 MHz, $CDCl_3$)

(R_p)-Benzyl 1,4(1,4)-dibenzenacyclohexaphane-1^2-ylcarbamate **((R_p)-1d)**

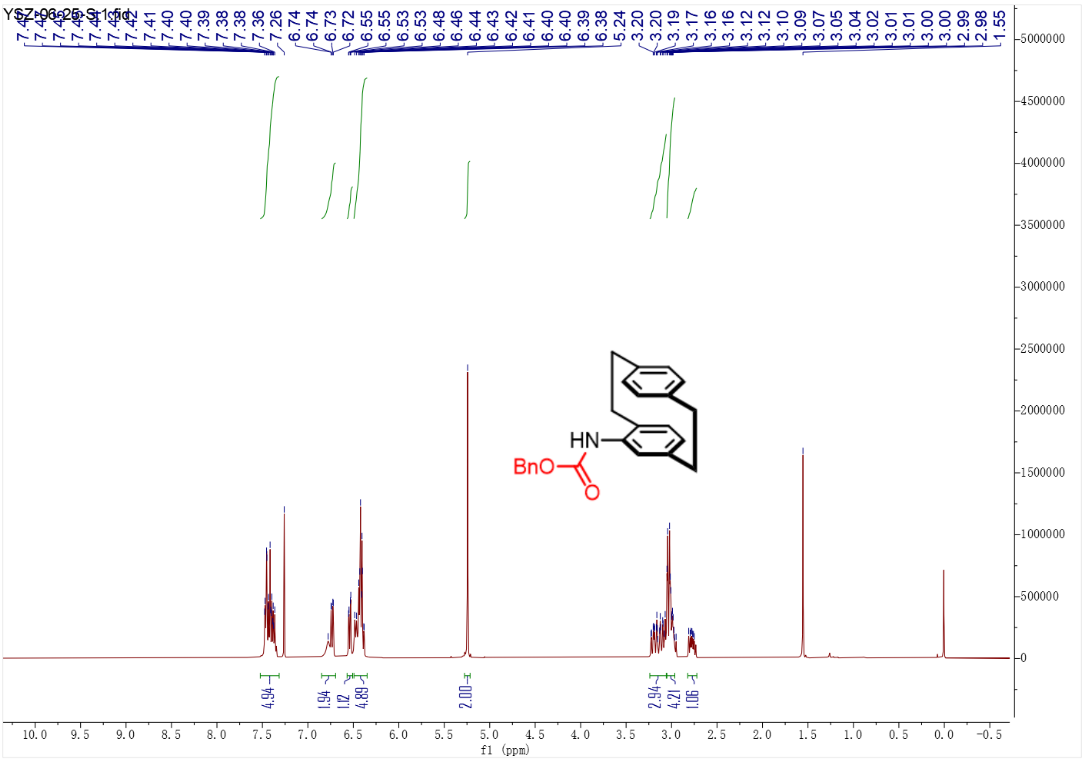

Supplementary Fig. 435 ^{1}H NMR spectrum of (R_p)**-1d** (400 MHz, $CDCl_3$)

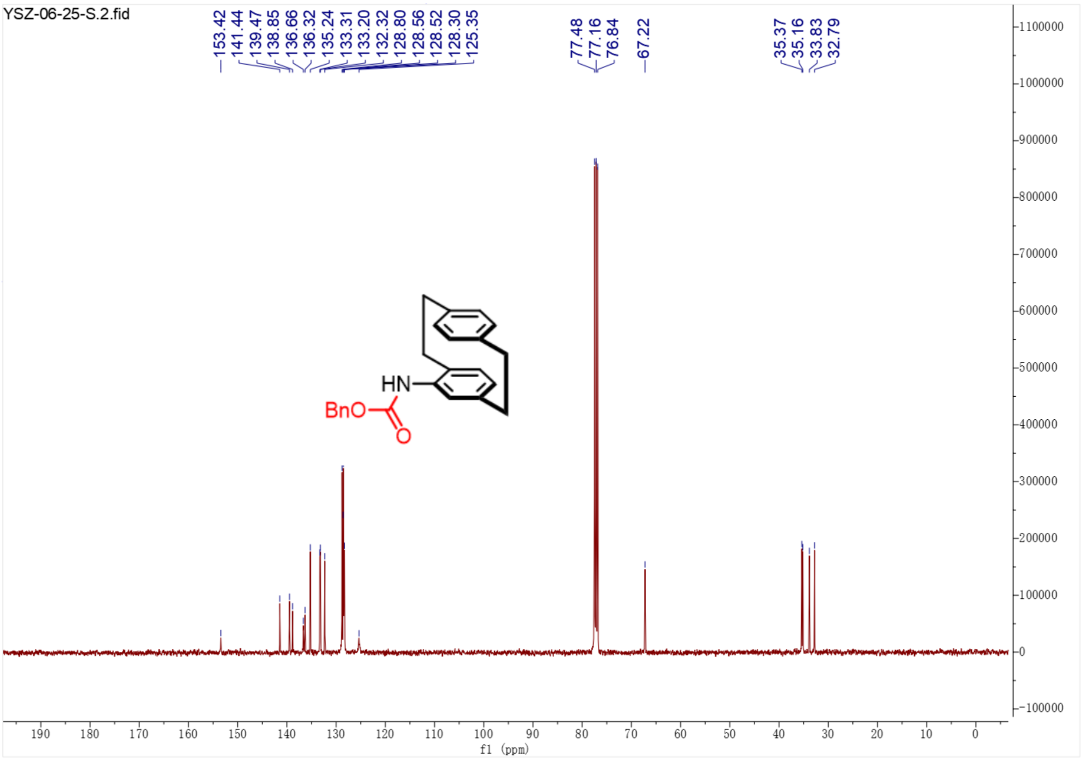

Supplementary Fig. 436 ^{13}C NMR spectrum of (R_p)**-1d** (101 MHz, $CDCl_3$)

(S_p)-Dibenzyl-1-(1^5-(((benzyloxy)carbonyl)amino)-1,4(1,4)-dibenzenacyclohexaphane-1^2-yl)hydrazine-1,2-dicarboxylate **(3d)**

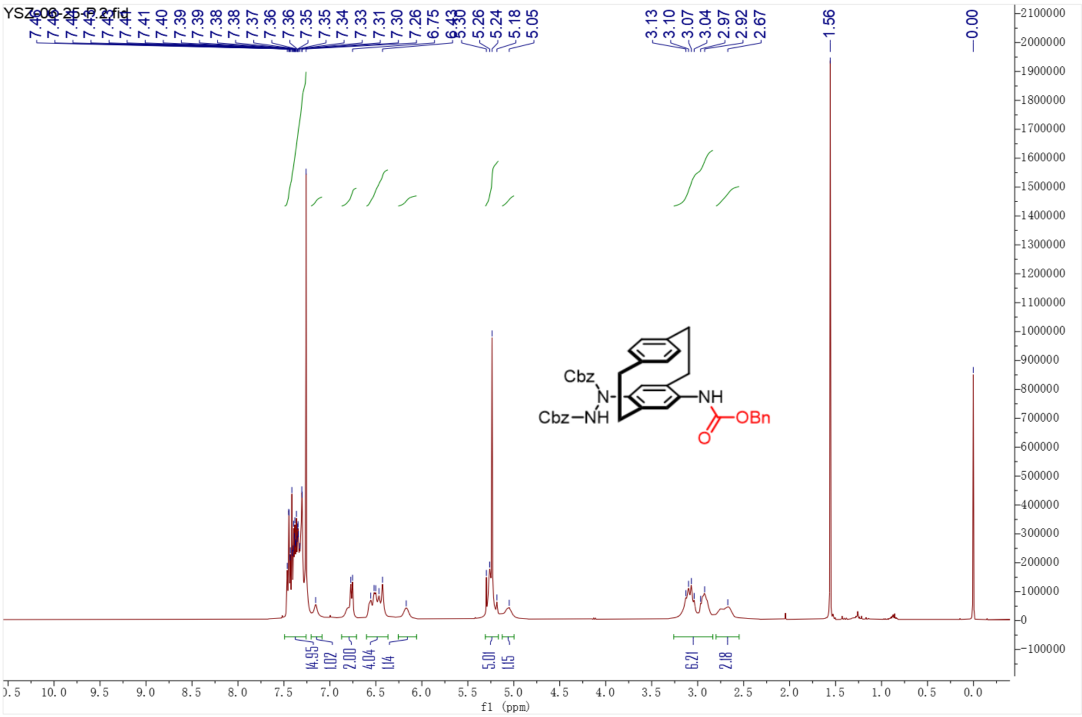

Supplementary Fig. 437 ^{1}H NMR spectrum of (S_p)-**3d** (400 MHz, $CDCl_3$)

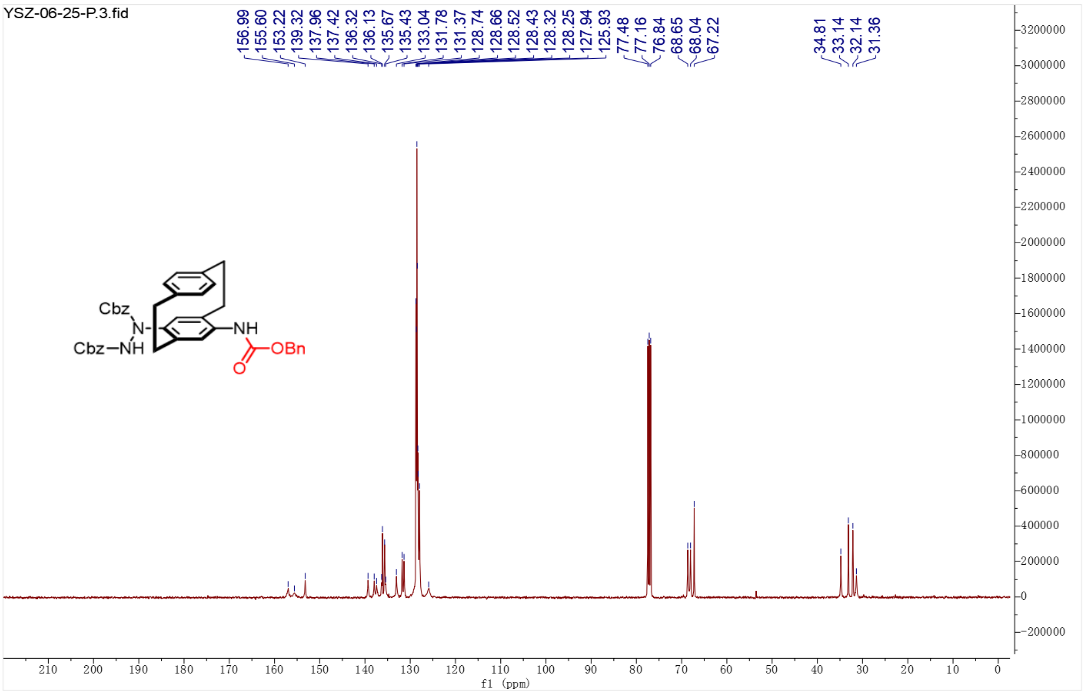

Supplementary Fig. 438 ^{13}C NMR spectrum of (S_p)-**3d** (101 MHz, $CDCl_3$)

(R_p)- (9*H*-fluoren-9-yl)methyl 1,4(1,4)-dibenzenacyclohexaphane-1^2-ylcarbamate (**(R_p)-1e**)

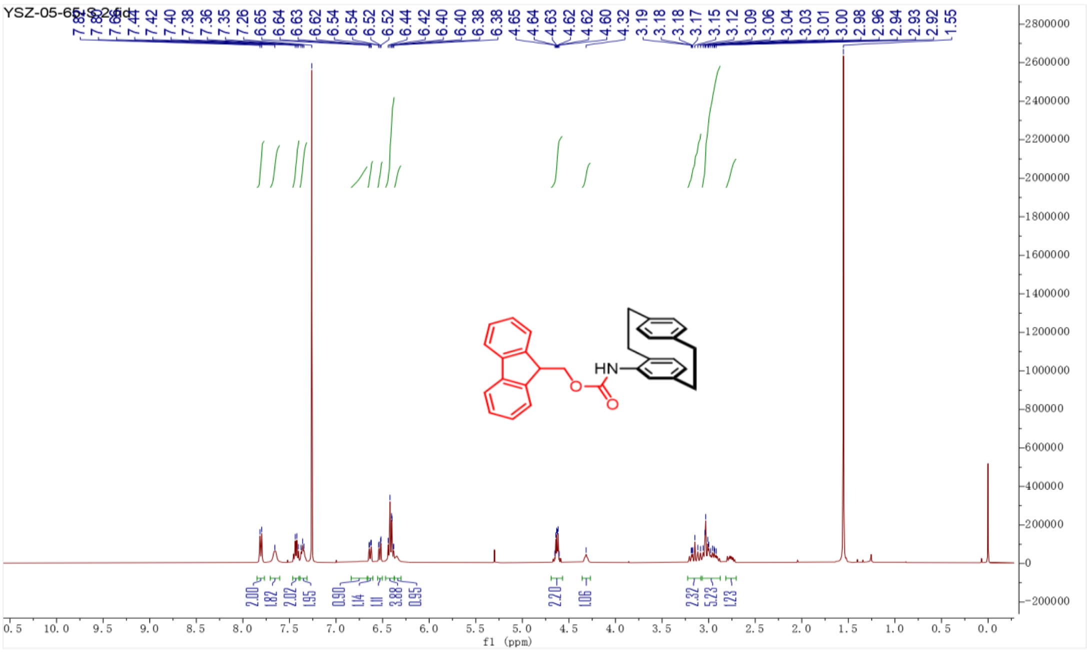

Supplementary Fig. 439 ^{1}H NMR spectrum of (R_p)**-1e** (400 MHz, $CDCl_3$)

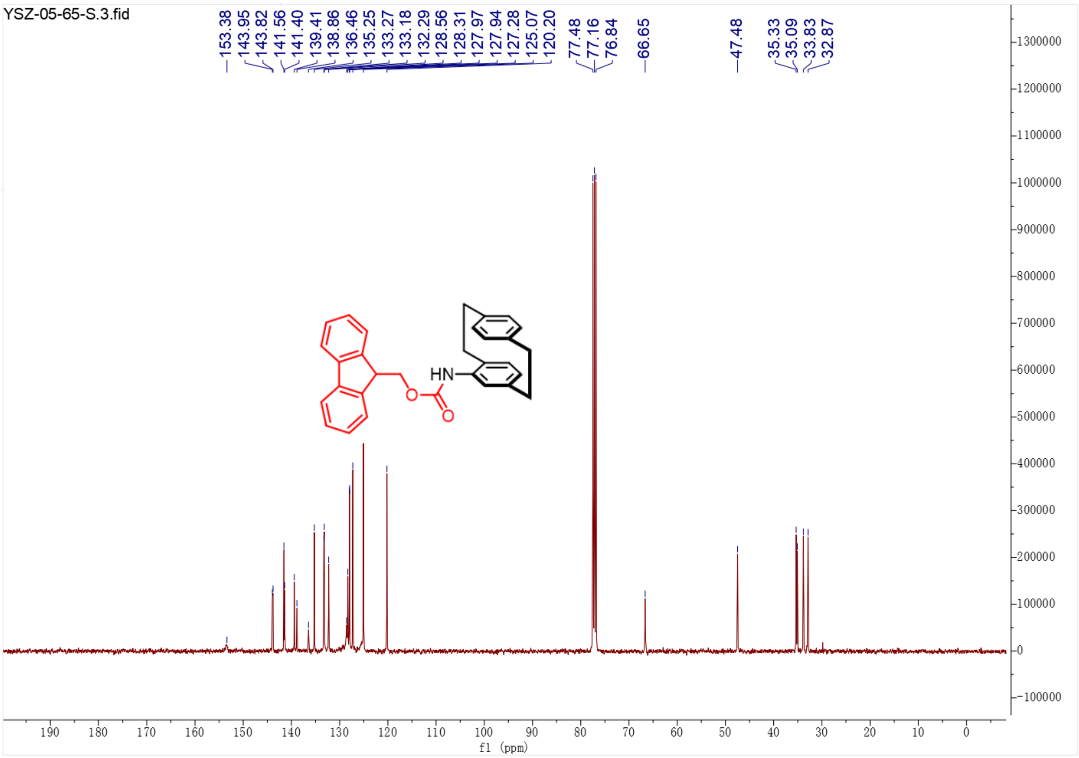

Supplementary Fig. 440 ^{13}C NMR spectrum of (R_p)**-1e** (101 MHz, $CDCl_3$)

(S_p)-Dibenzyl 1-(1^5-((((9H-fluoren-9-yl)methoxy)carbonyl)amino)-1,4(1,4)-dibenzenacyclohexaphane-1^2-yl)hydrazine-1,2-dicarboxylate **(3e)**

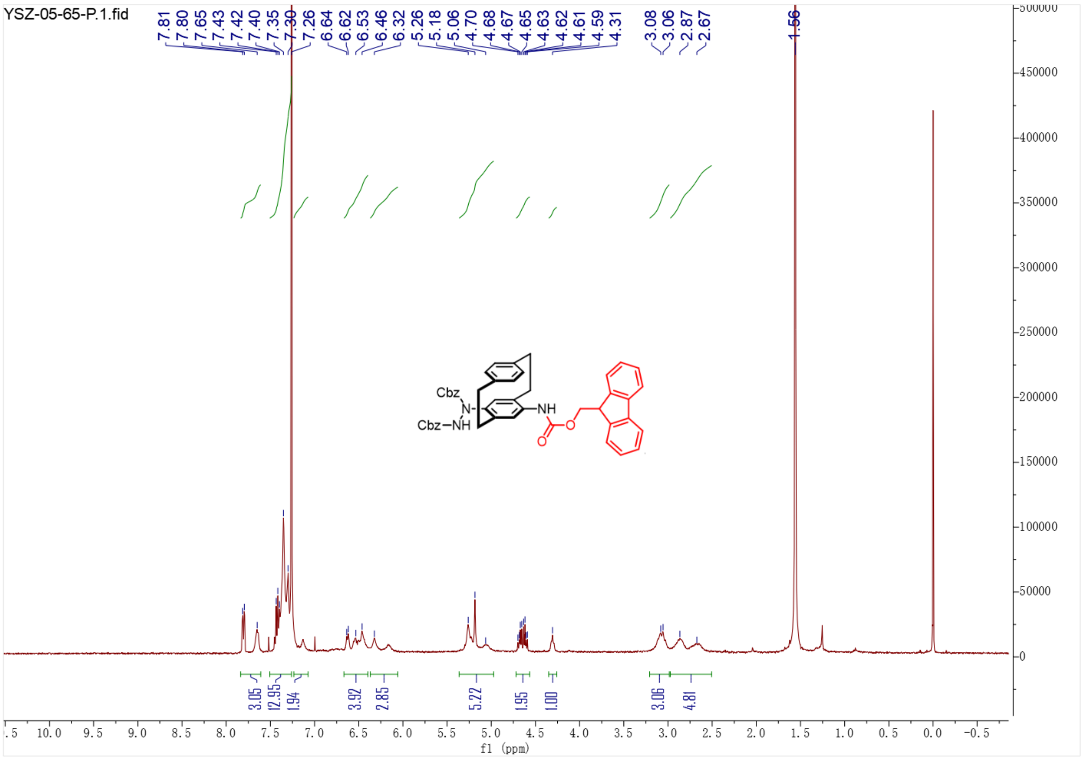

Supplementary Fig. 441 ^{1}H NMR spectrum of (S_p)-**3e** (400 MHz, $CDCl_3$)

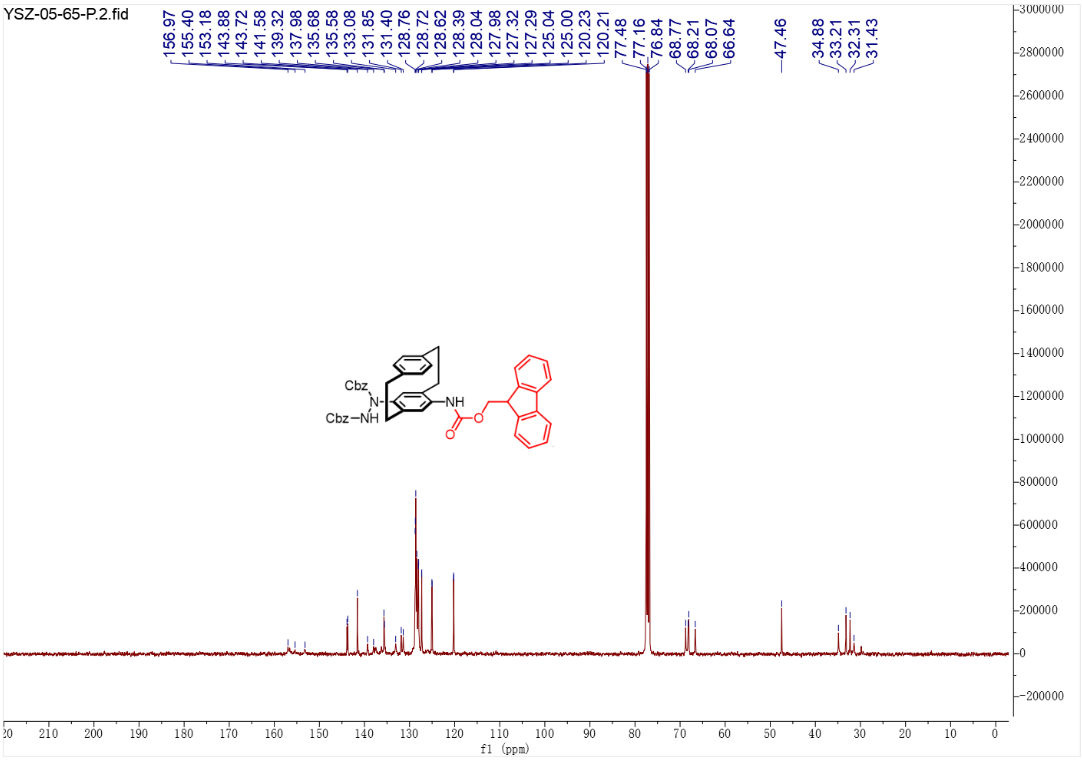

Supplementary Fig. 442 ^{13}C NMR spectrum of (S_p)-**3e** (101 MHz, $CDCl_3$)

(S_p)-Tert-butyl (4[2]-bromo-1,4(1,4)-dibenzenacyclohexaphane-1[2]-yl)carbamate **((S_p)-1f)**

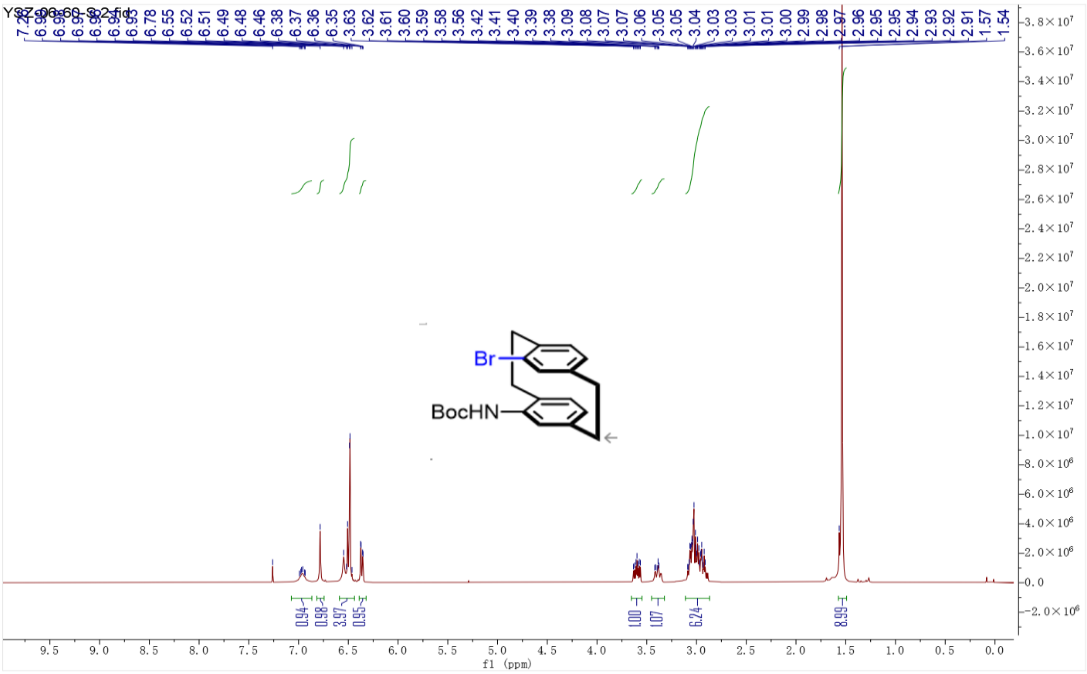

Supplementary Fig. 443 ^{1}H NMR spectrum of (S_p)**-1f** (400 MHz, $CDCl_3$)

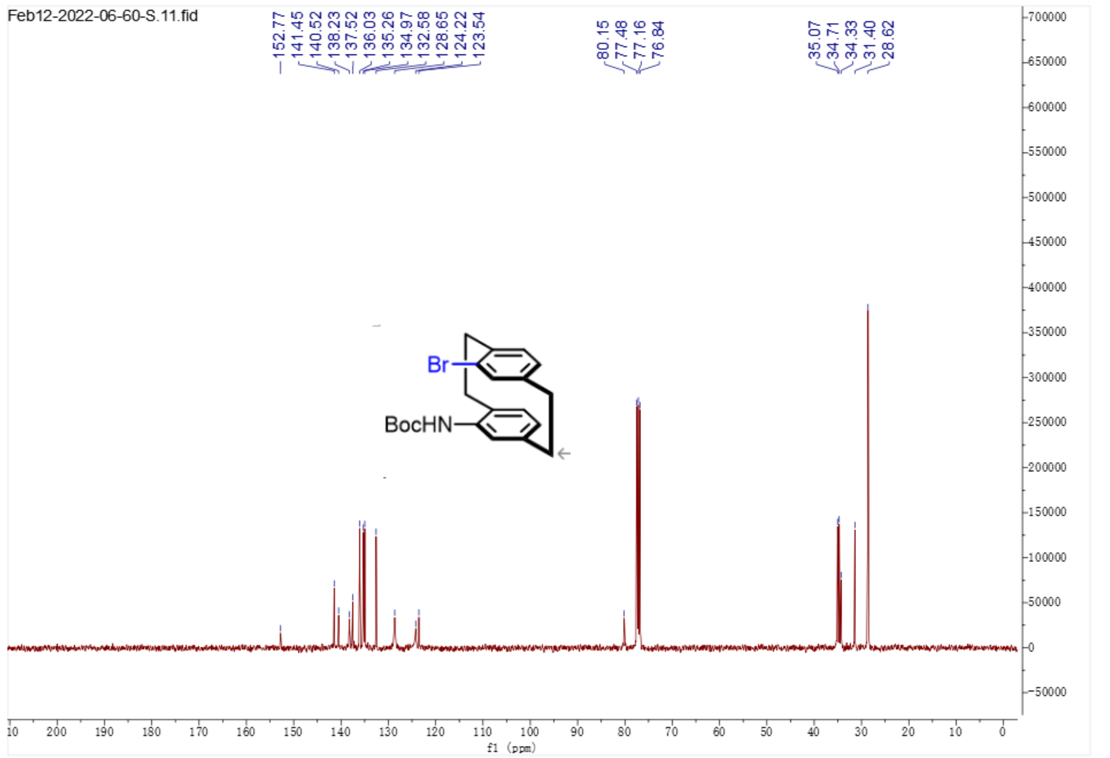

Supplementary Fig. 444 ^{13}C NMR spectrum of (S_p)**-3b** (101 MHz, $CDCl_3$)

(S_p)-Dibenzyl 1-(4^3-bromo-1^5-((tert-butoxycarbonyl)amino)-1,4(1,4)-dibenzenacyclohexaphane-1^2-yl)hydrazine-1,2-dicarboxylate **(3f)**

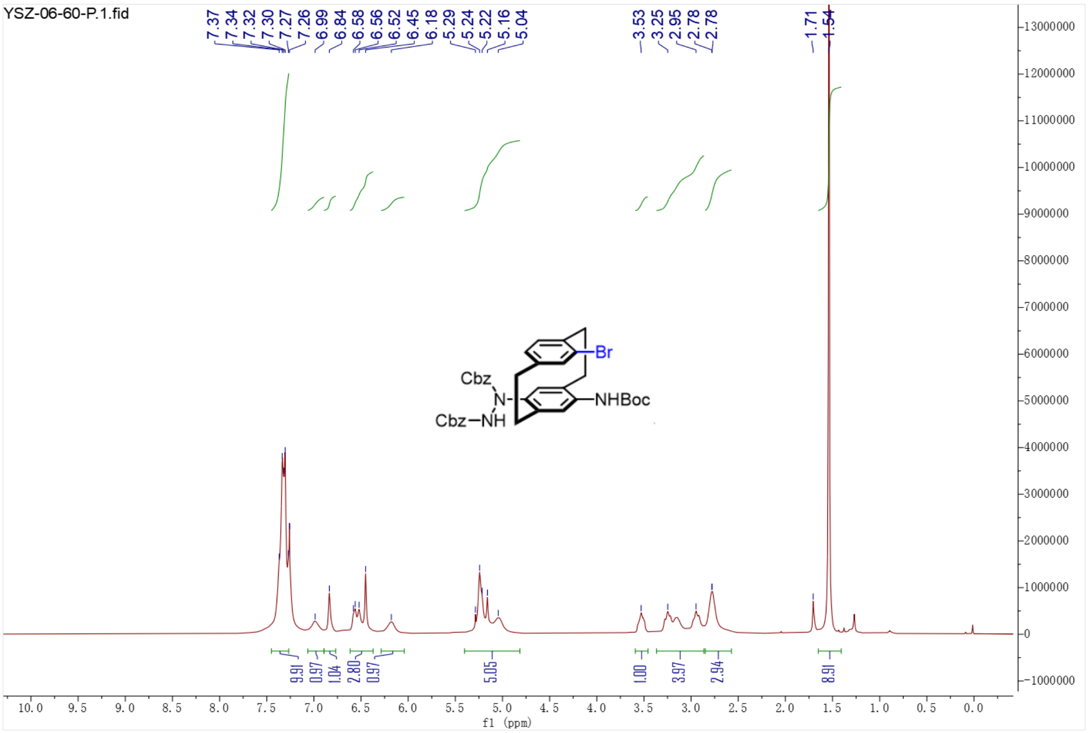

Supplementary Fig. 445 ^{1}H NMR spectrum of (S_p)-**3f** (400 MHz, $CDCl_3$)

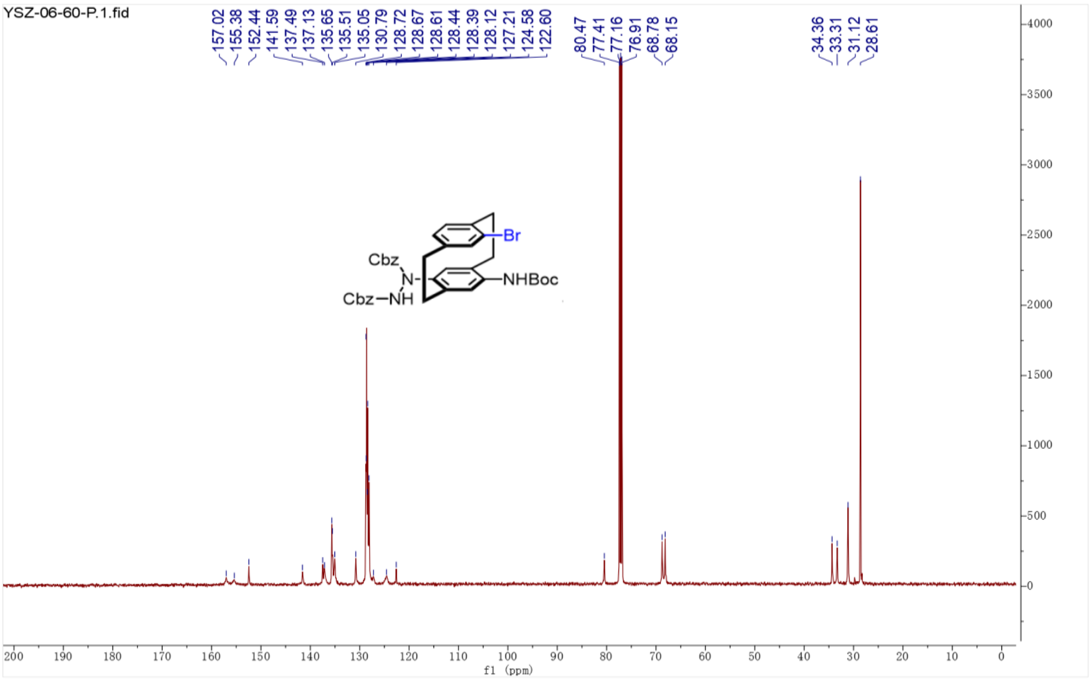

Supplementary Fig. 446 ^{13}C NMR spectrum of (S_p)-**3f** (126 MHz, $CDCl_3$)

(R_p)-Tert-butyl (4[2]-phenyl-1,4(1,4)-dibenzenacyclohexaphane-1[2]-yl)carbamate

((R_p)-1g)

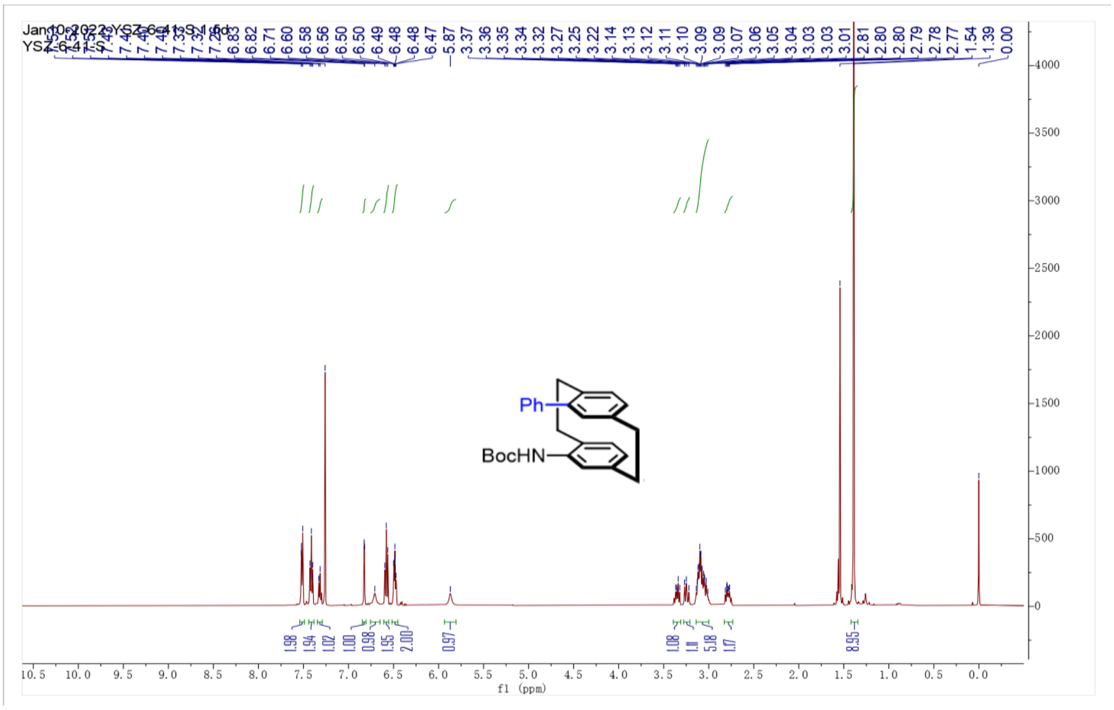

Supplementary Fig. 447 ^{1}H NMR spectrum of (R_p)**-1g** (500 MHz, $CDCl_3$)

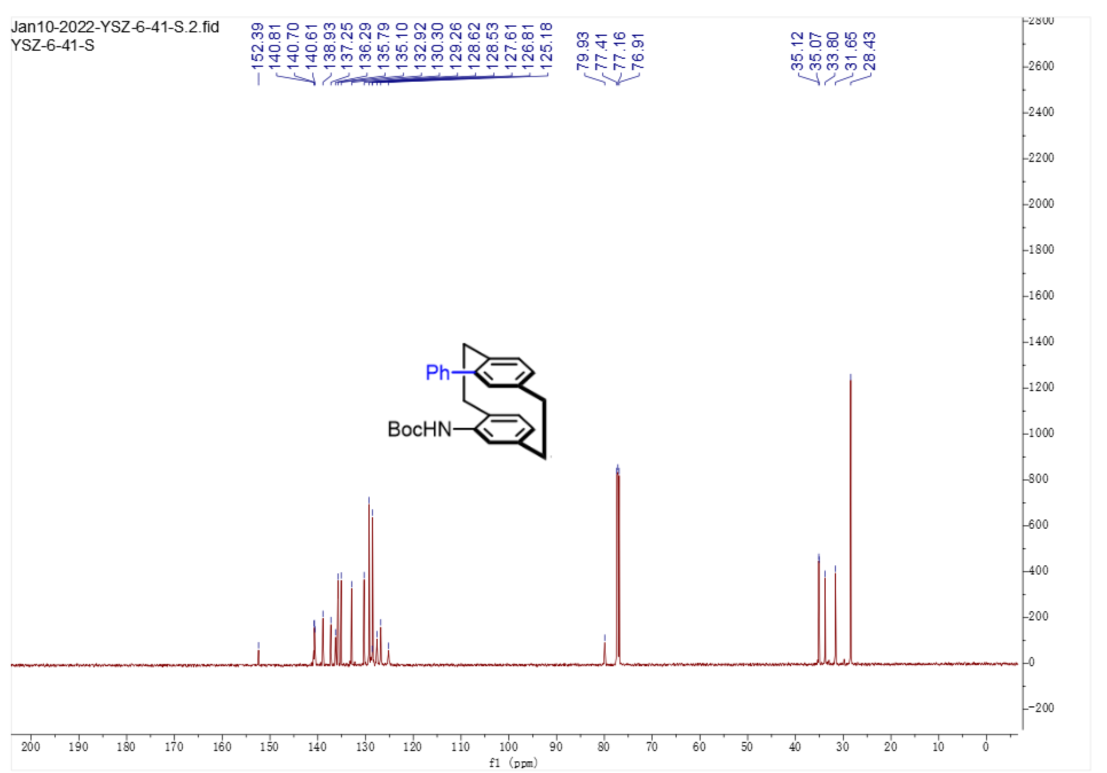

Supplementary Fig. 448 ^{13}C NMR spectrum of (R_p)**-1g** (126 MHz, $CDCl_3$)

(S_p)-Dibenzyl 1-(1[5]-((tert-butoxycarbonyl)amino)-4[3]-phenyl-1,4(1,4)-dibenzenacyclohexaphane-1[2]-yl)hydrazine-1,2-dicarboxylate **(3g)**

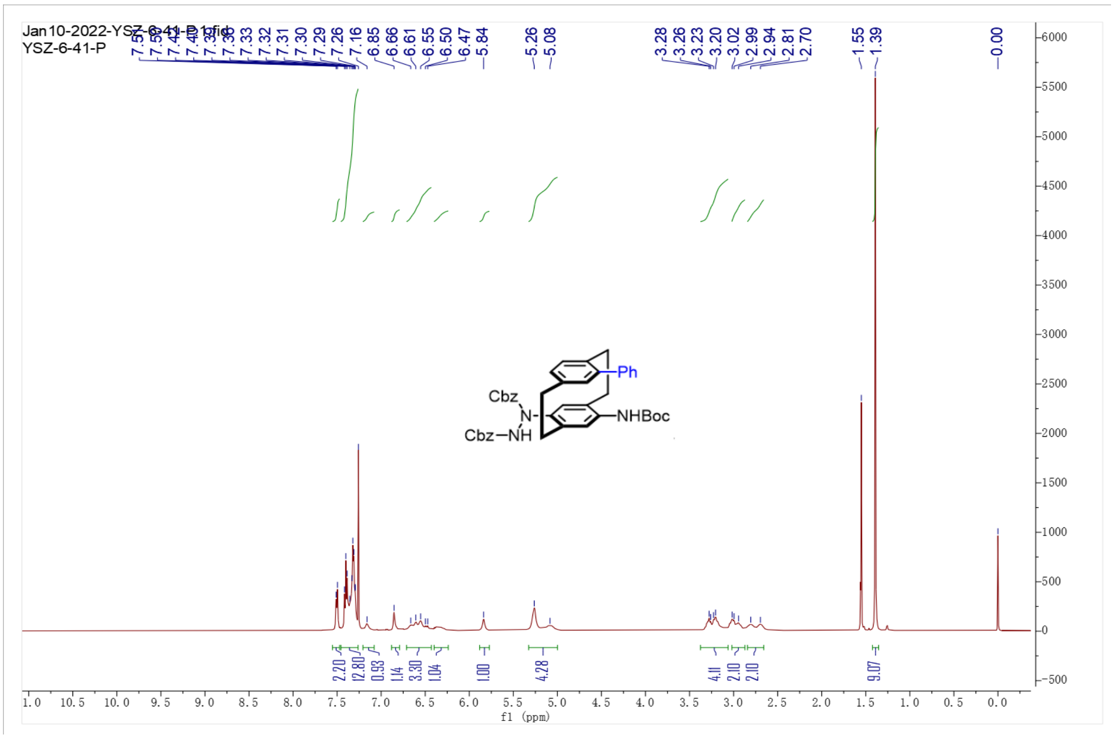

Supplementary Fig. 449 ^{1}H NMR spectrum of (S_p)-**3g** (500 MHz, $CDCl_3$)

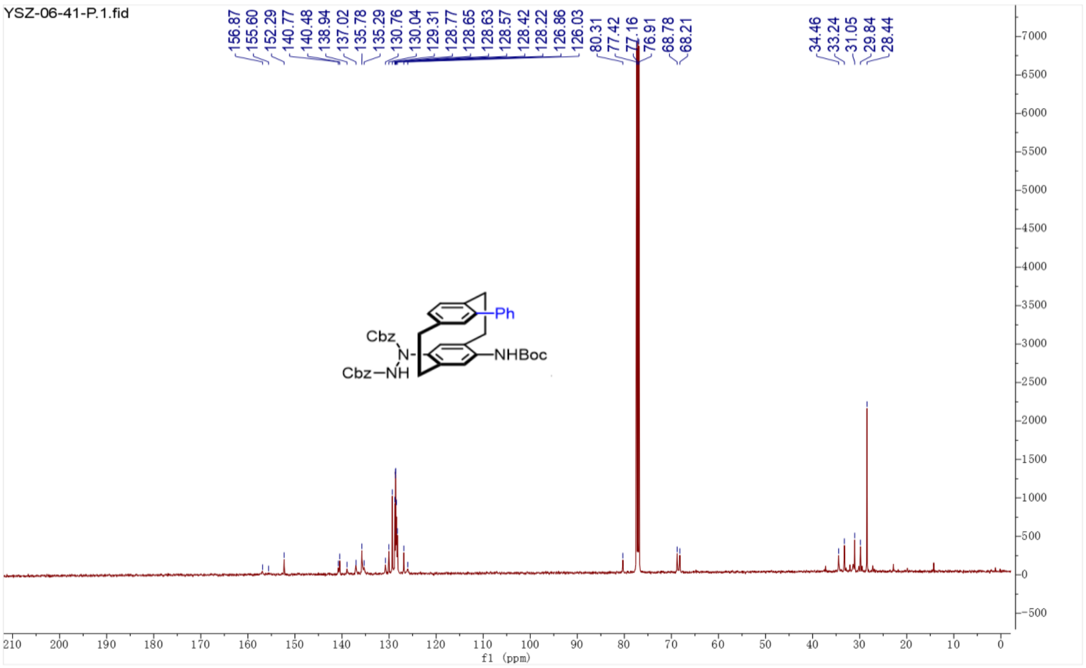

Supplementary Fig. 450 ^{13}C NMR spectrum of (S_p)-**3g** (126 MHz, $CDCl_3$)

(R_p)-Tert-butyl-(4²-(4-methoxyphenyl)-1,4(1,4)-dibenzenacyclohexaphane-1²-yl)carbamate **((R_p)-1h)**

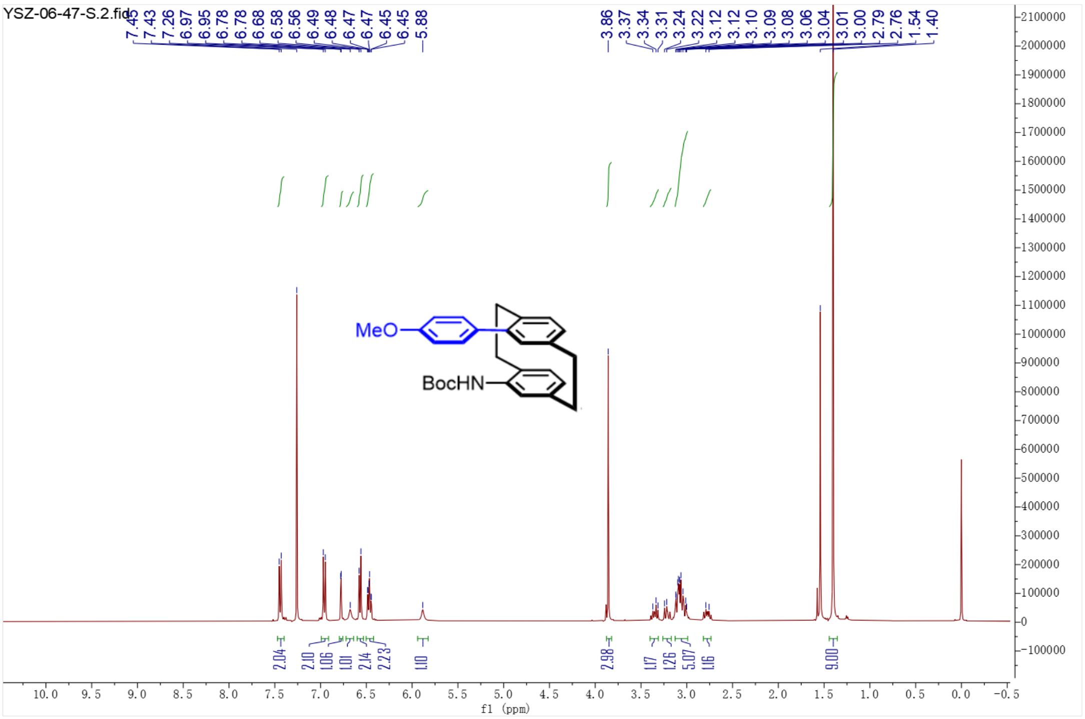

Supplementary Fig. 451 ^{1}H NMR spectrum of (R_p)-**1h** (400 MHz, $CDCl_3$)

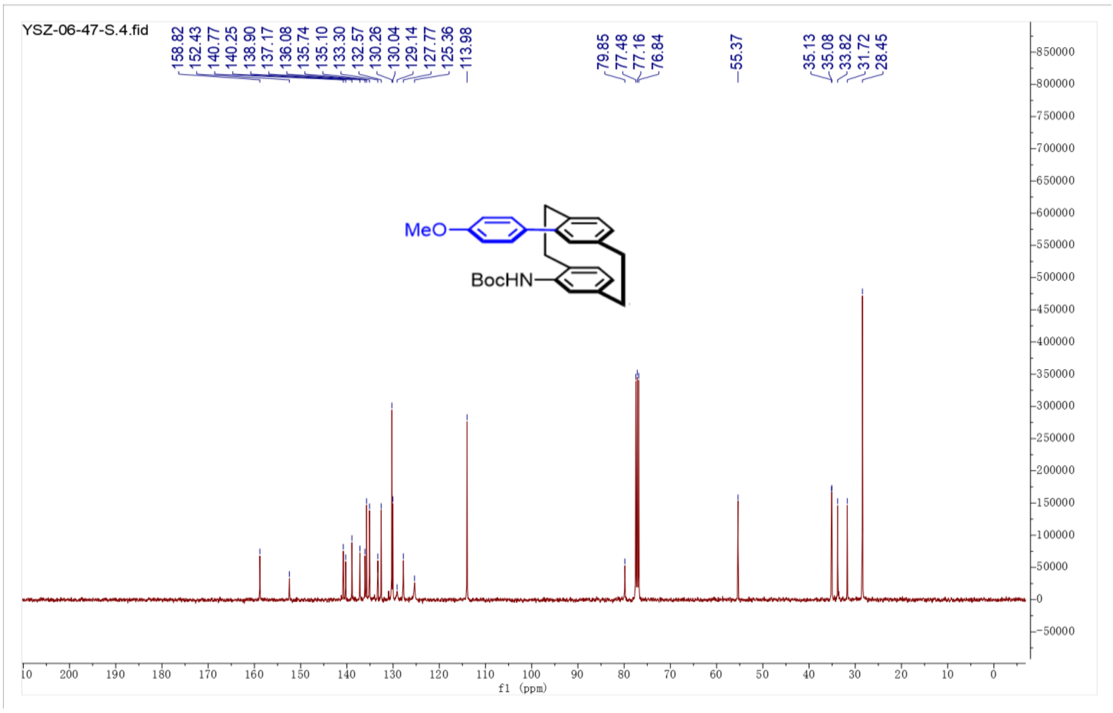

Supplementary Fig. 452 ^{13}C NMR spectrum of (R_p)-**1h** (101 MHz, $CDCl_3$)

(S_p)-Dibenzyl 1-(1^5-((tert-butoxycarbonyl)amino)-4^3-(4-methoxyphenyl)-1,4(1,4)-dibenzenacyclohexaphane-1^2-yl)hydrazine-1,2-dicarboxylate **(3h)**

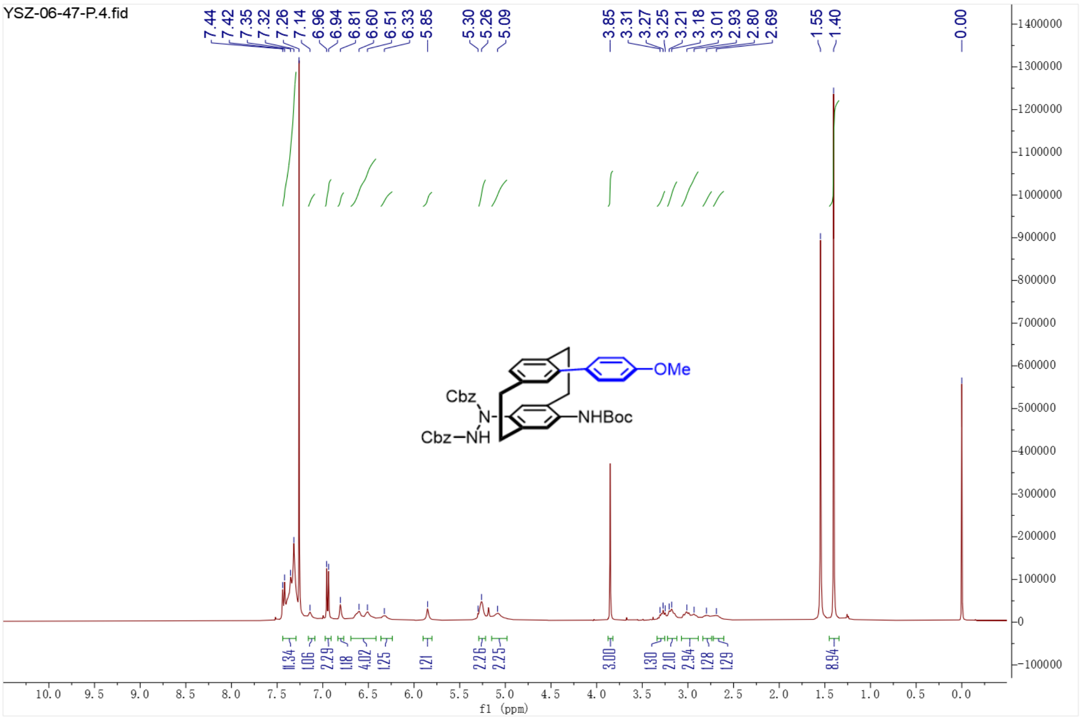

Supplementary Fig. 453 ^{1}H NMR spectrum of (S_p)-**3h** (400 MHz, $CDCl_3$)

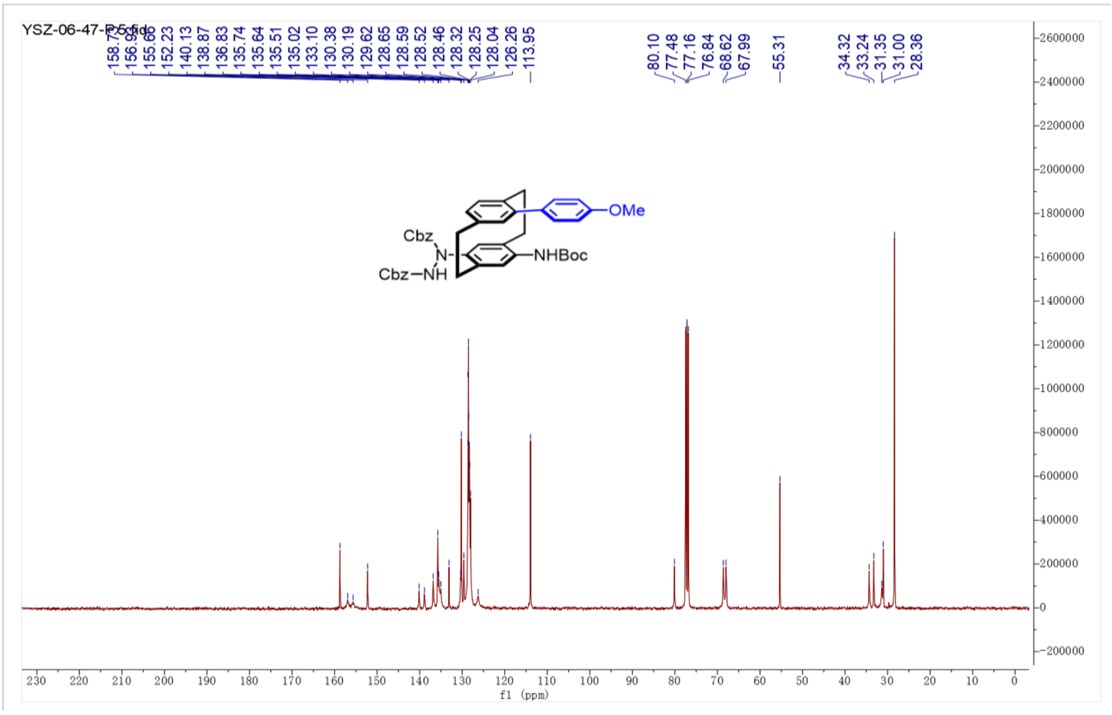

Supplementary Fig. 454 ^{13}C NMR spectrum of (S_p)-**3h** (101 MHz, $CDCl_3$)

(R_p)-Tert-butyl (E)-(4^2-styryl-1,4(1,4)-dibenzenacyclohexaphane-1^2-yl)carbamate (**(**R_p**)-1i)**

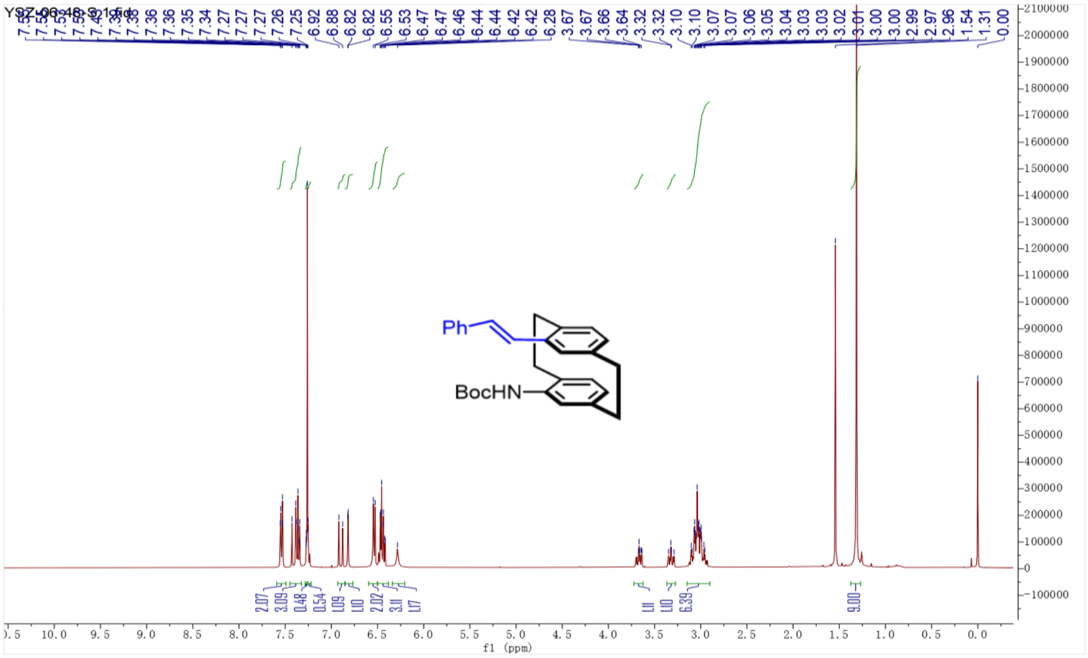

Supplementary Fig. 455 ^{1}H NMR spectrum of (S_p)-**1i** (400 MHz, $CDCl_3$)

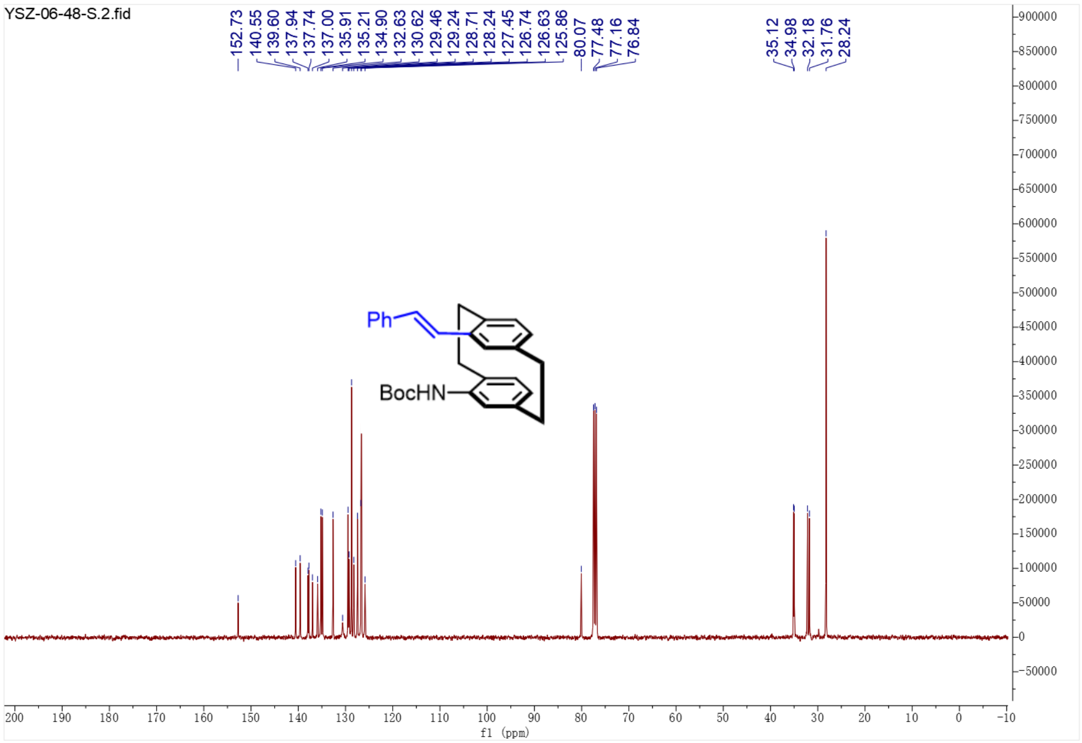

Supplementary Fig. 456 ^{13}C NMR spectrum of (S_p)-**1i** (101 MHz, $CDCl_3$)

(S_p)-Dibenzyl (E)-1-(1^5-((tert-butoxycarbonyl)amino)-4^3-styryl-1,4(1,4)-dibenzenacyclohexaphane-1^2-yl)hydrazine-1,2-dicarboxylate **(3i)**

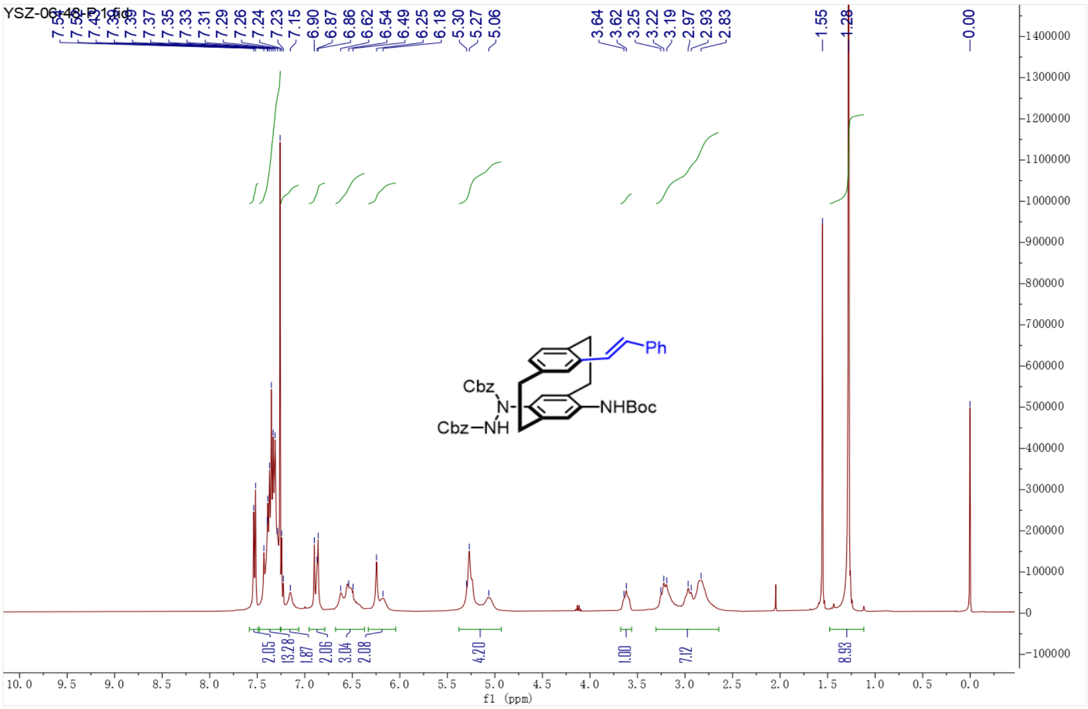

Supplementary Fig. 457 ^{1}H NMR spectrum of (S_p)-**3i** (400 MHz, $CDCl_3$)

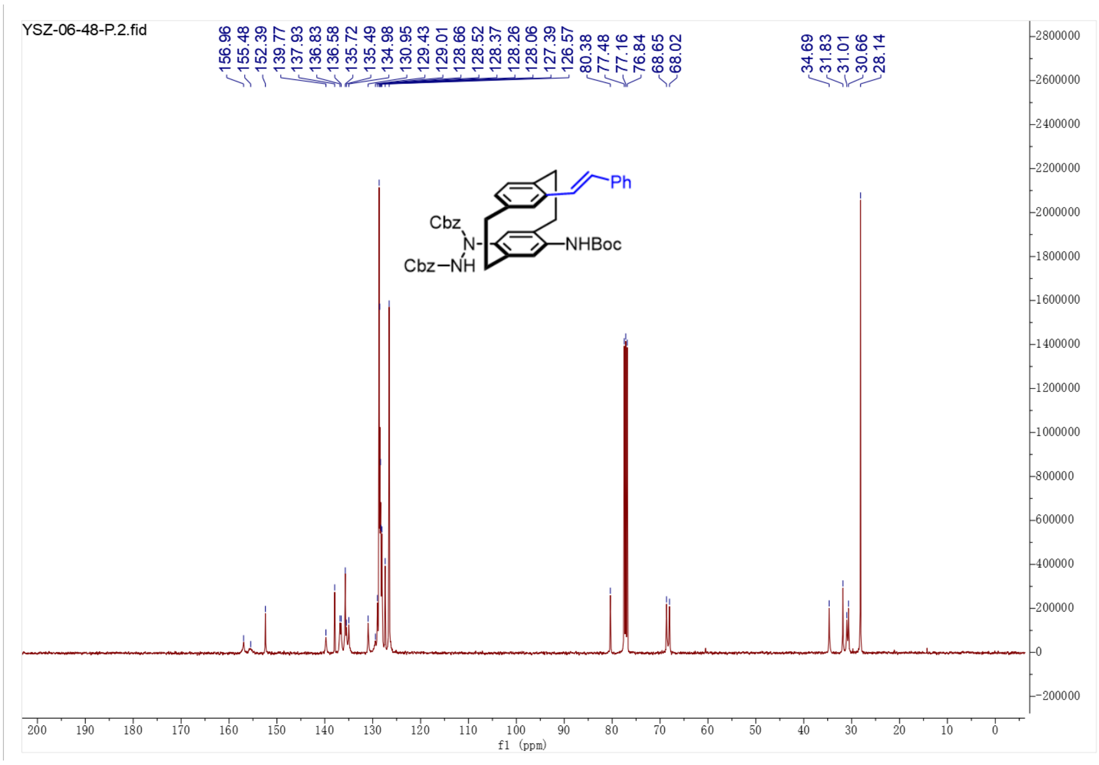

Supplementary Fig. 458 ^{13}C NMR spectrum of (S_p)-**1i** (101 MHz, $CDCl_3$)

(R_p)-Tert-butyl-(4^2-(phenylethynyl)-1,4(1,4)-dibenzenacyclohexaphane-1^2-yl)carbamate (**(**R_p**)-1j**)

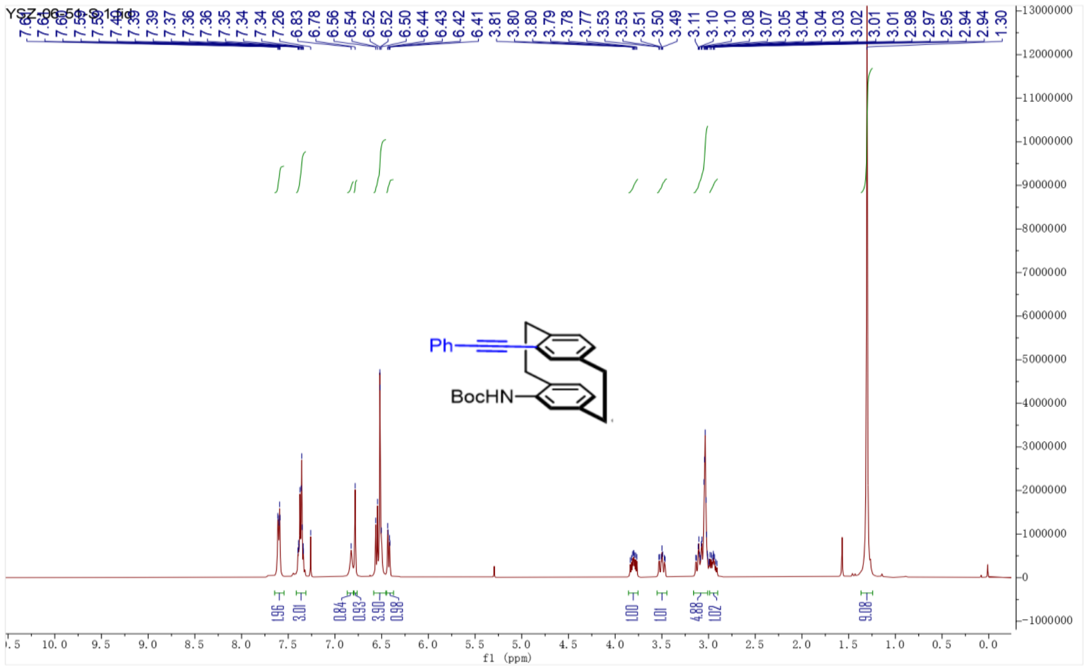

Supplementary Fig. 459 ^{1}H NMR spectrum of (R_p)**-1j** (400 MHz, $CDCl_3$)

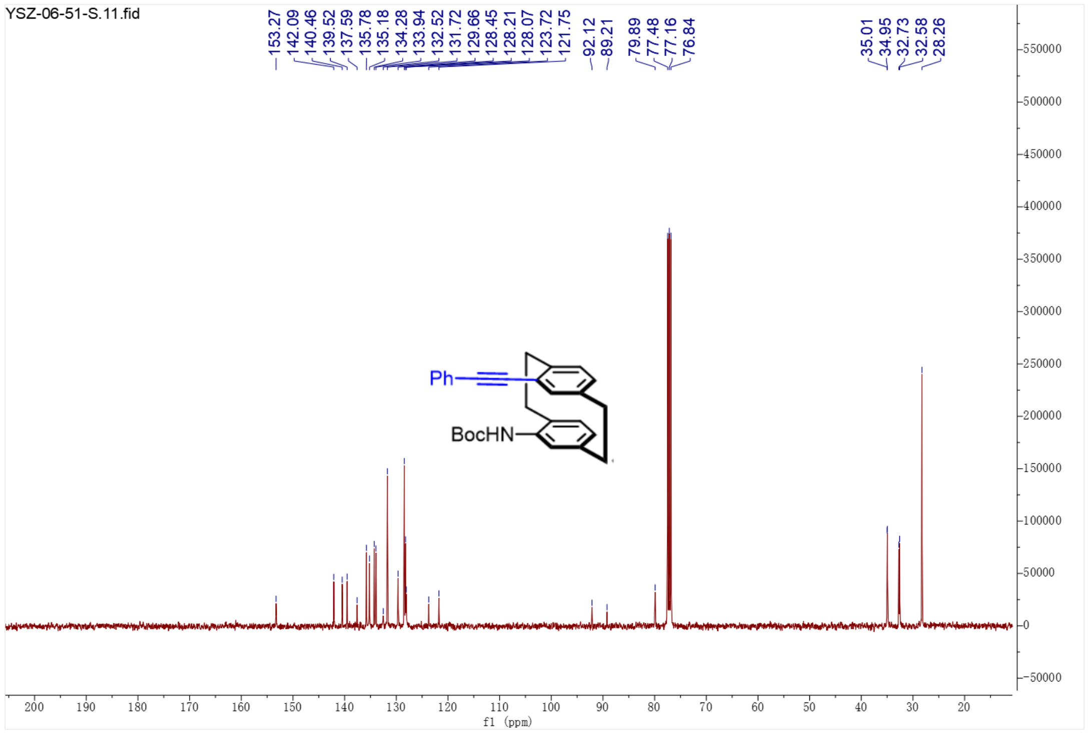

Supplementary Fig. 460 ^{13}C NMR spectrum of (R_p)**-1j** (101 MHz, $CDCl_3$)

(S_p)-Dibenzyl 1-(1[5]-((tert-butoxycarbonyl)amino)-4[3]-(phenylethynyl)-1,4(1,4)-dibenzenacyclohexaphane-1[2]-yl)hydrazine-1,2-dicarboxylate **(3j)**

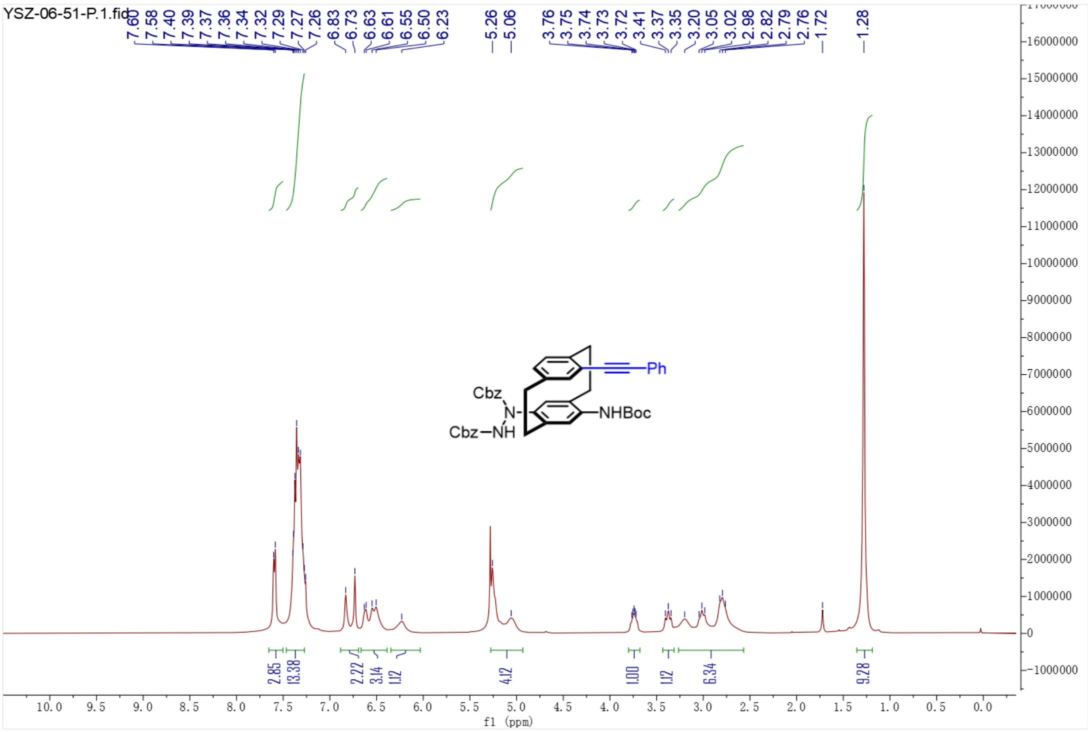

Supplementary Fig. 461 ^{1}H NMR spectrum of (S_p)-**3j** (400 MHz, $CDCl_3$)

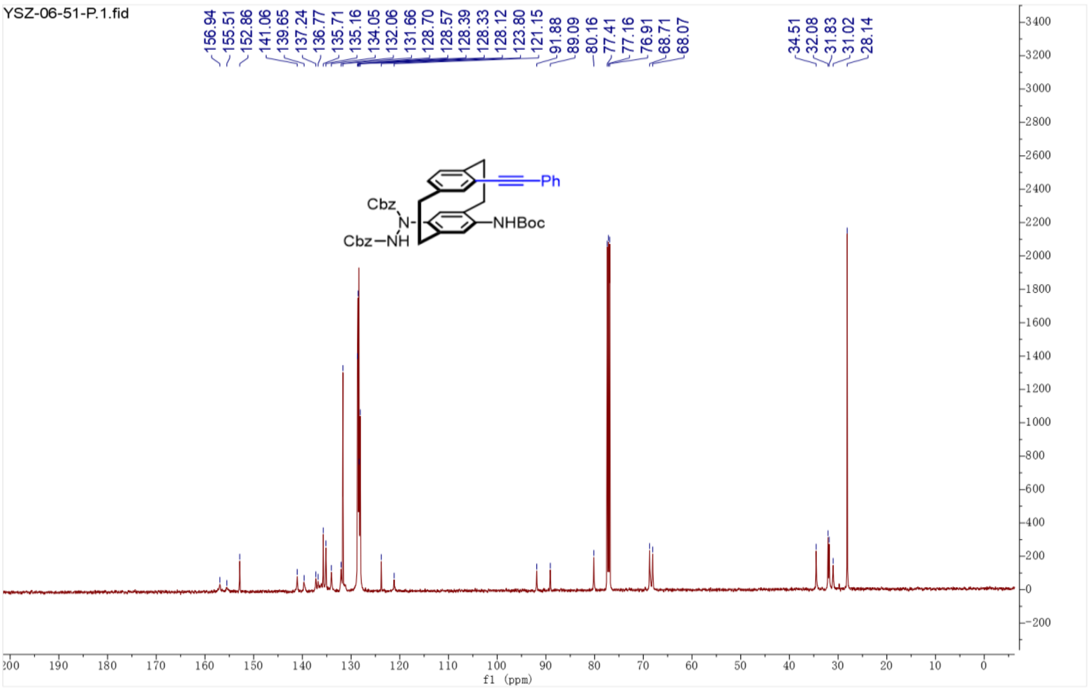

Supplementary Fig. 462 ^{13}C NMR spectrum of (S_p)-**3j** (126 MHz, $CDCl_3$)

(R_p)-Tert-butyl (4^2-cyclohexyl-1,4(1,4)-dibenzenacyclohexaphane-1^2-yl)carbamate **((R_p)-1k)**

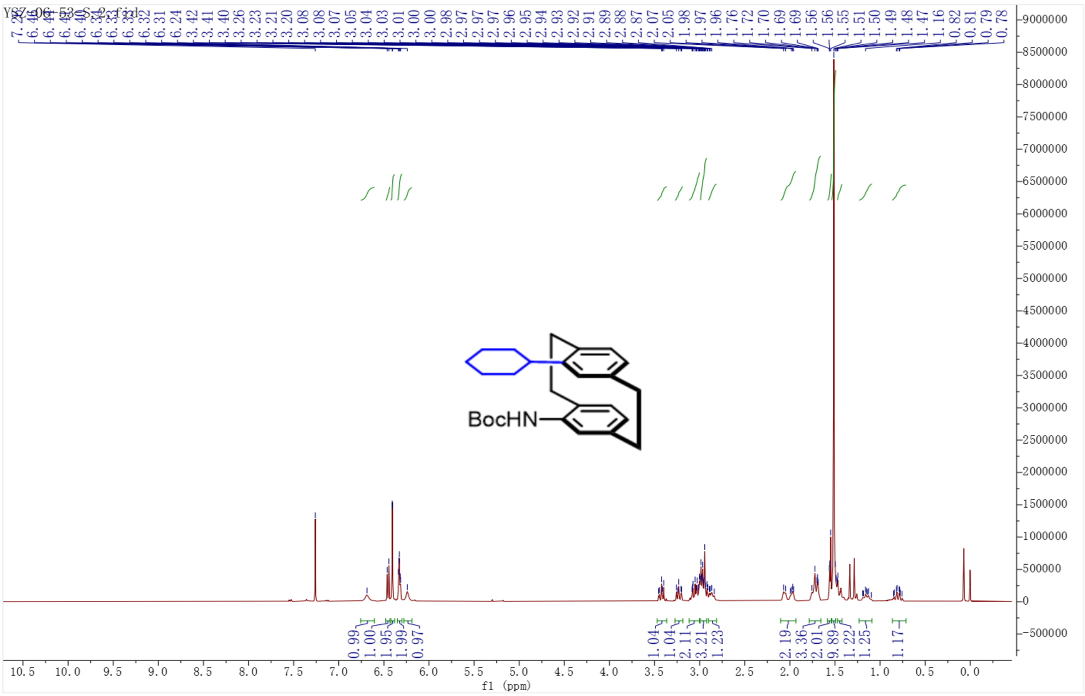

Supplementary Fig. 463 ^{1}H NMR spectrum of (R_p)-**1k** (400 MHz, $CDCl_3$)

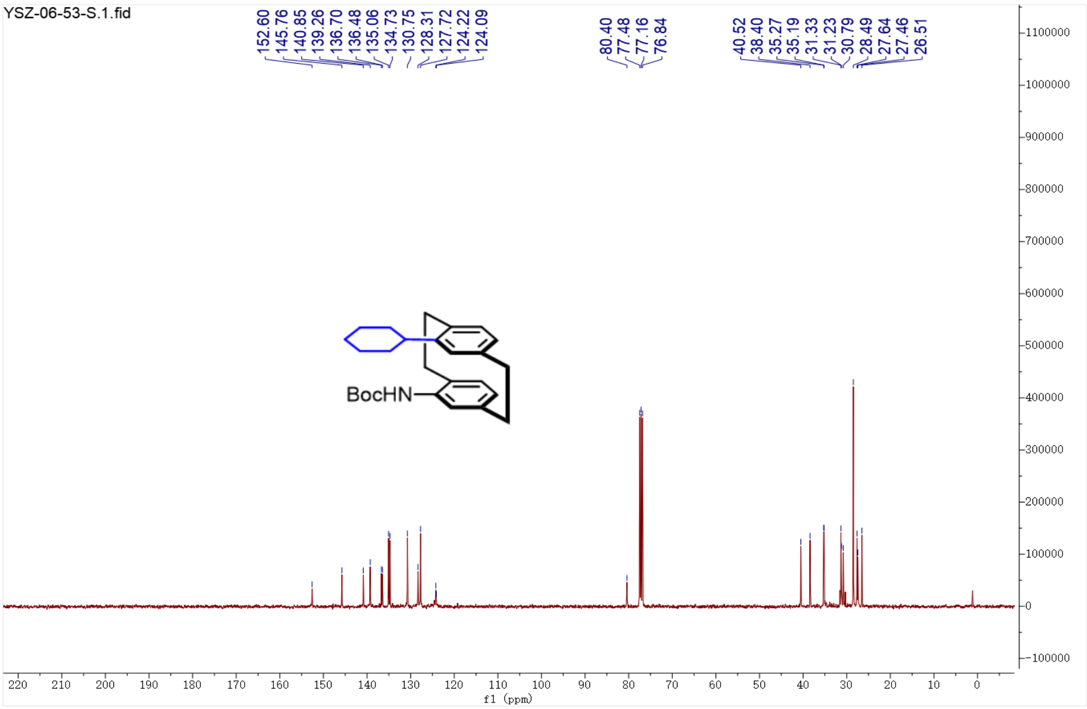

Supplementary Fig. 464 ^{13}C NMR spectrum of (R_p)-**1k** (101 MHz, $CDCl_3$)

(S_p)-Dibenzyl 1-(1^5-((tert-butoxycarbonyl)amino)-4^3-cyclohexyl-1,4(1,4)-dibenzenacyclohexaphane-1^2-yl)hydrazine-1,2-dicarboxylate **(3k)**

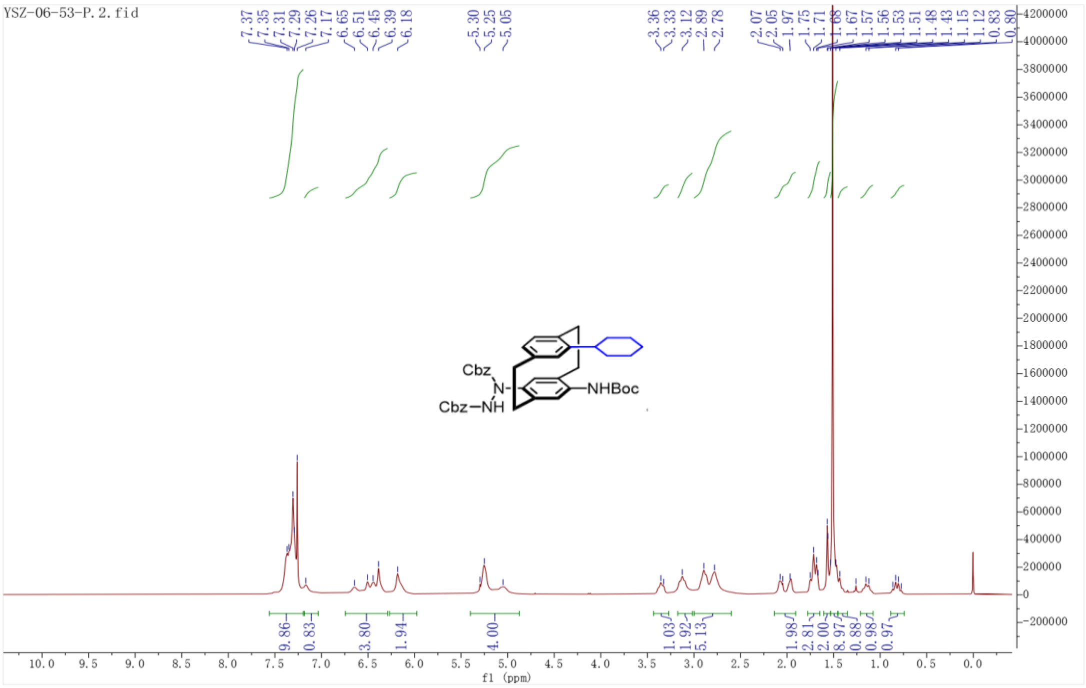

Supplementary Fig. 465 ^{1}H NMR spectrum of (S_p)-**3k** (400 MHz, $CDCl_3$)

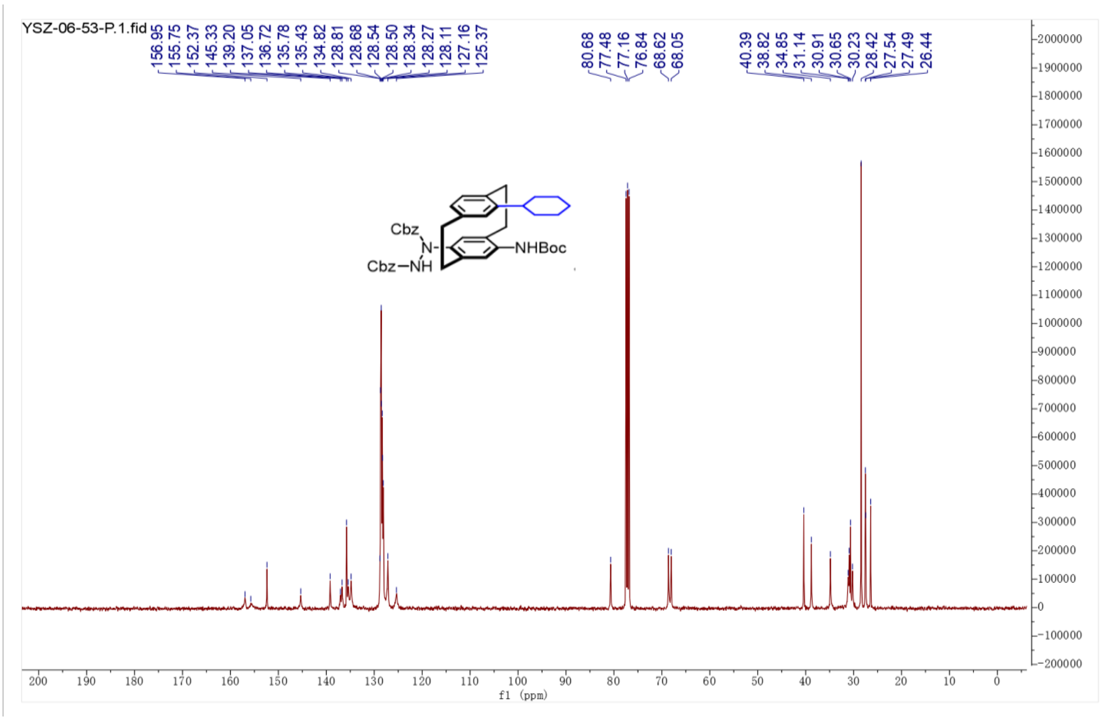

Supplementary Fig. 466 ^{13}C NMR spectrum of (S_p)-**3k** (101 MHz, $CDCl_3$)

(S_p)-4[2]-((tert-butoxycarbonyl)amino)-1,4(1,4)-dibenzenacyclohexaphane-1[2]-yl acetate **((S_p)-1l)**

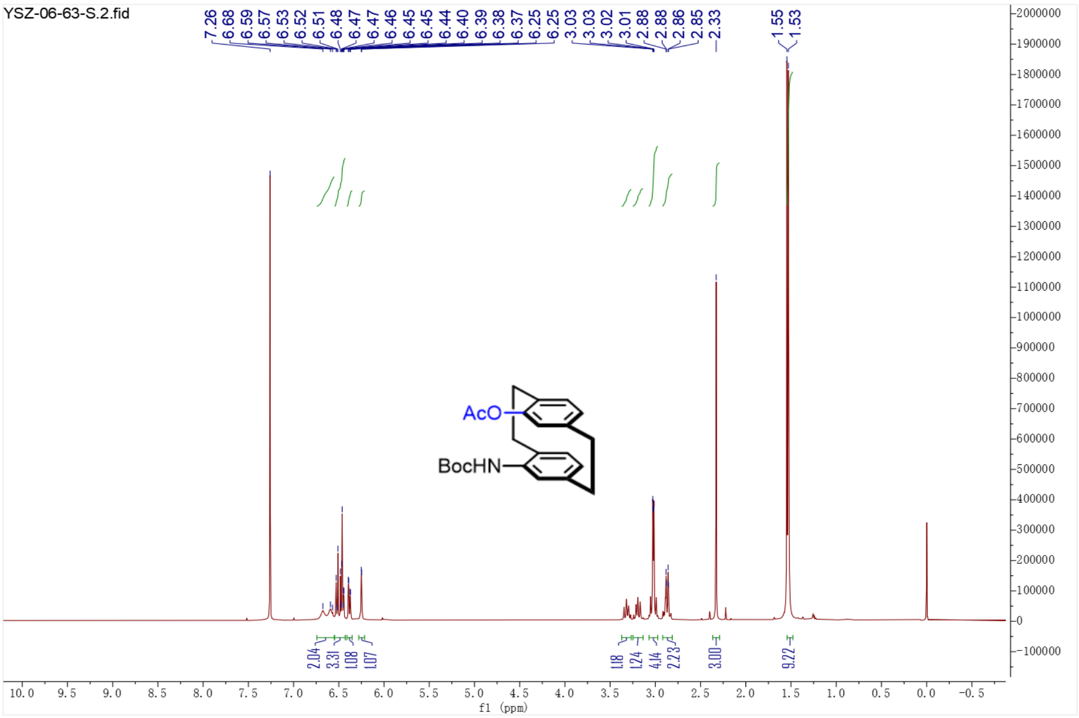

Supplementary Fig. 467 ^{1}H NMR spectrum of (S_p)-**1l** (400 MHz, $CDCl_3$)

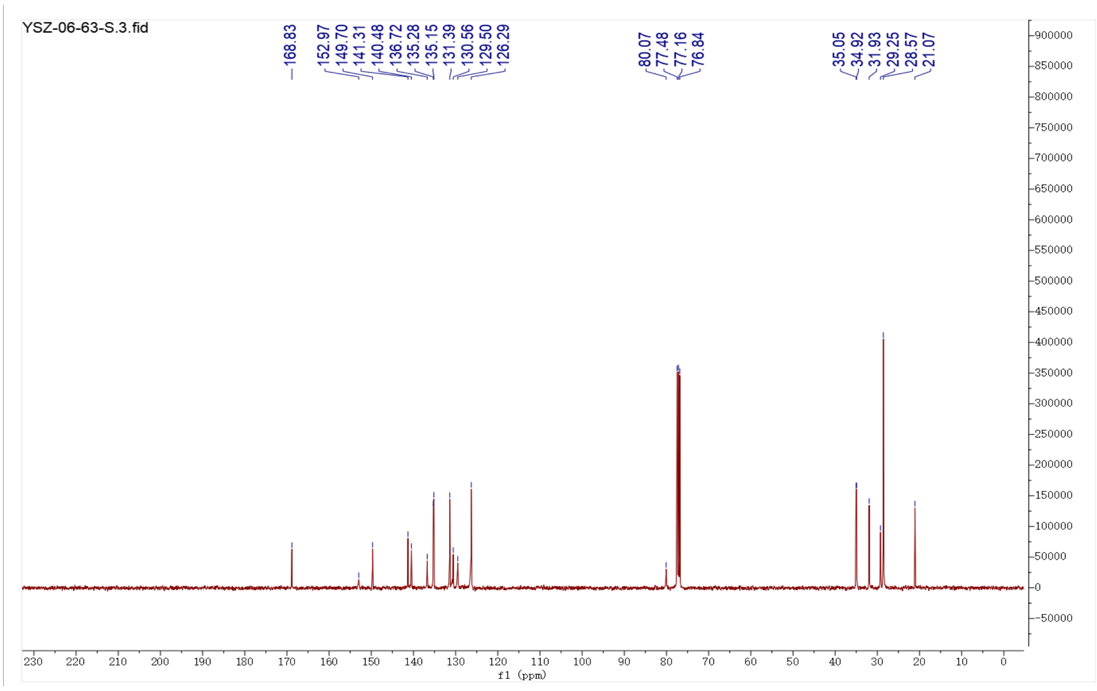

Supplementary Fig. 468 ^{13}C NMR spectrum of (S_p)-**1l** (101 MHz, $CDCl_3$)

(R_p)-Dibenzyl 1-(4[3]-acetoxy-1[5]-((tert-butoxycarbonyl)amino)-1,4(1,4)-dibenzenacyclohexaphane-1[2]-yl)hydrazine-1,2-dicarboxylate **(3l)**

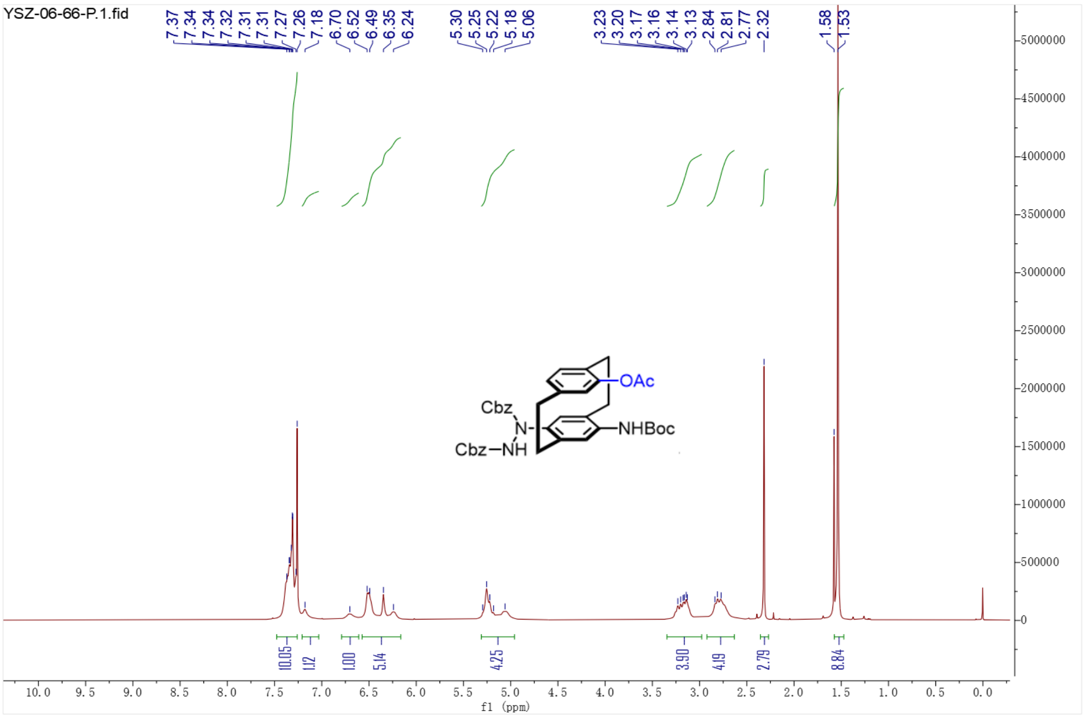

Supplementary Fig. 469 ^{1}H NMR spectrum of (R_p)-**3l** (400 MHz, $CDCl_3$)

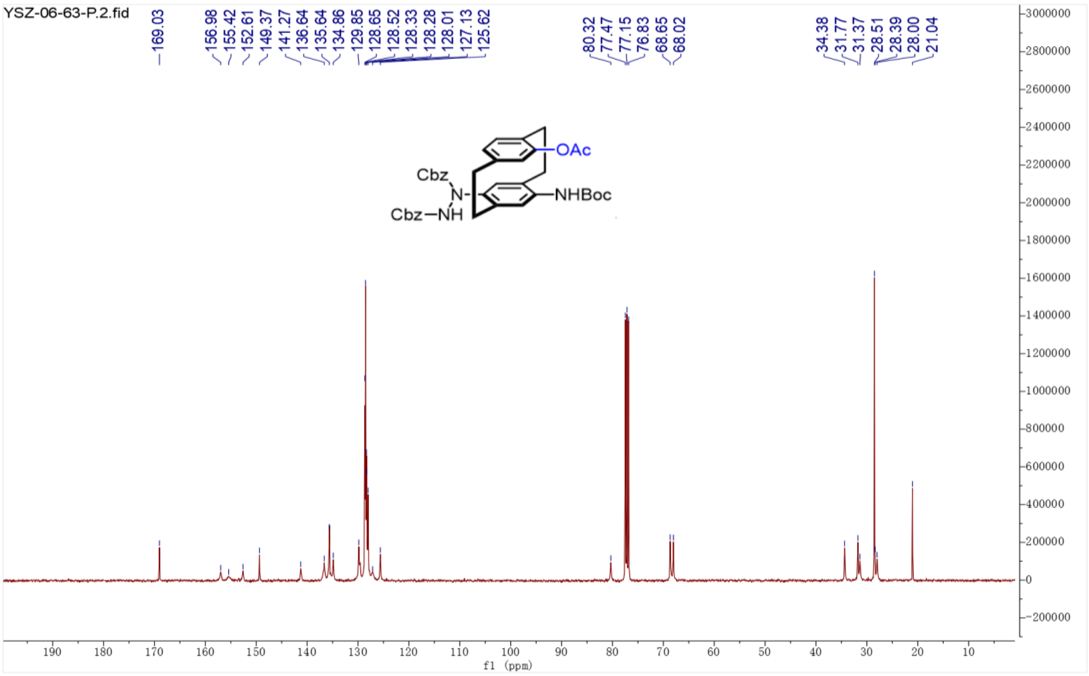

Supplementary Fig. 470 ^{13}C NMR spectrum of (R_p)-**3l** (101 MHz, $CDCl_3$)

(R_p)-Tert-butyl (4[3]-bromo-1,4(1,4)-dibenzenacyclohexaphane-1[2]-yl)carbamate ((R_p)-1m)

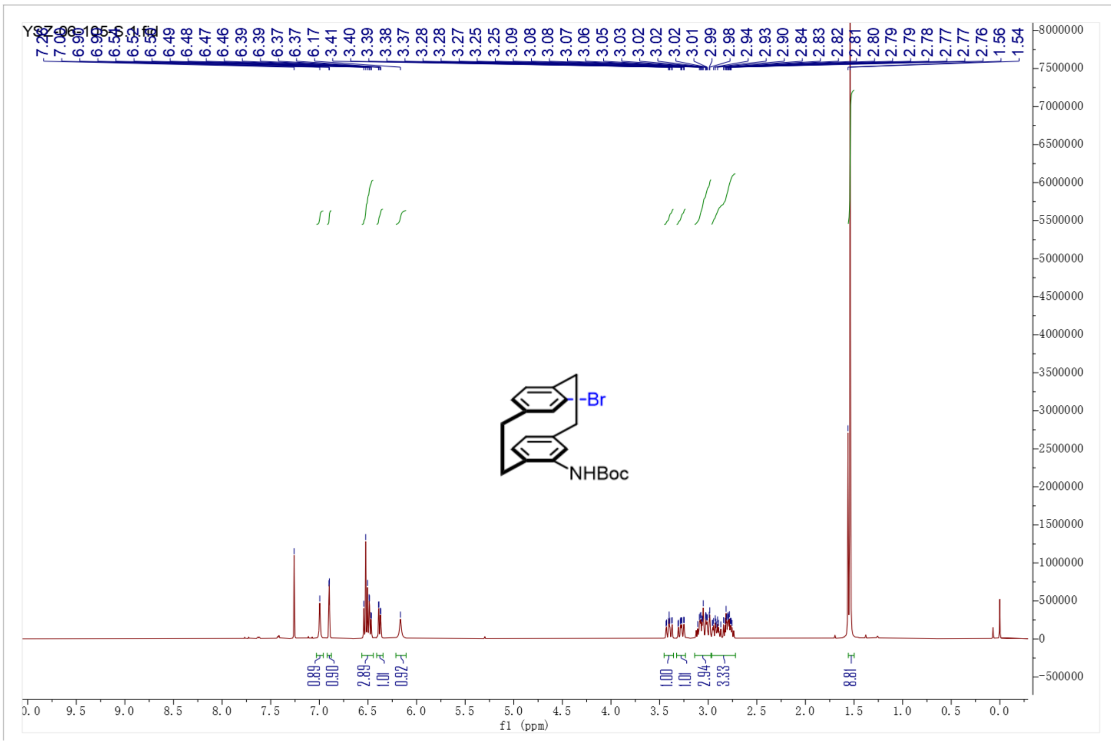

Supplementary Fig. 471 ^{1}H NMR spectrum of (R_p)-**1m** (400 MHz, $CDCl_3$)

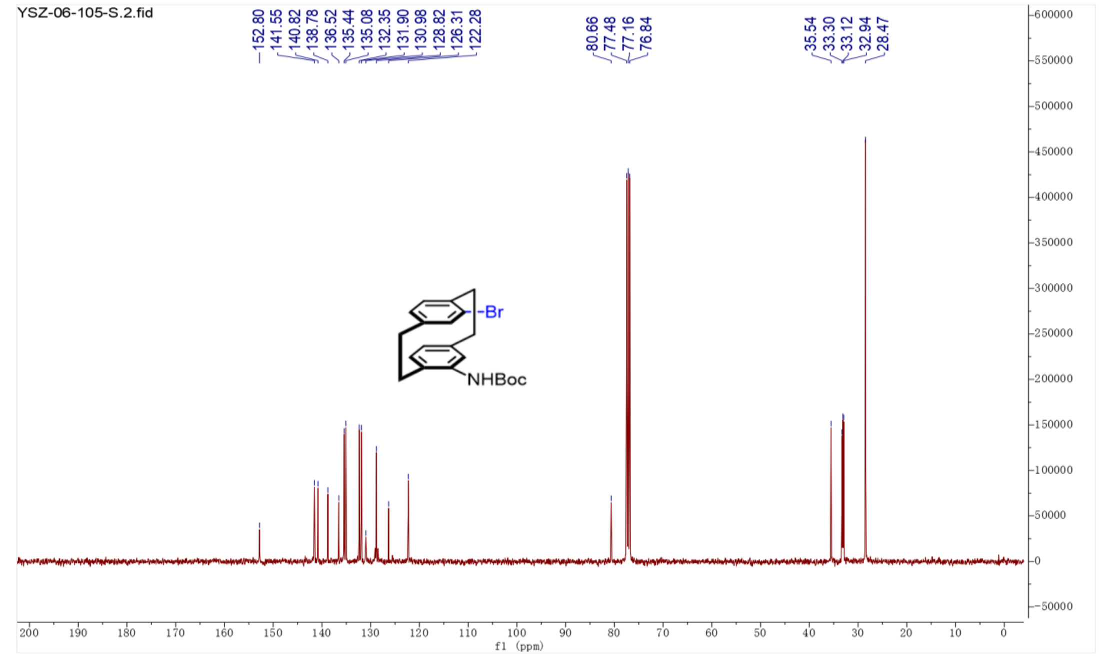

Supplementary Fig. 472 ^{13}C NMR spectrum of (R_p)-**1m** (101 MHz, $CDCl_3$)

(S_p)-Dibenzyl 1-(4^2-bromo-1^5-((tert-butoxycarbonyl)amino)-1,4(1,4) -dibenzenacyclohexaphane-1^2-yl)hydrazine-1,2-dicarboxylate **(3m)**

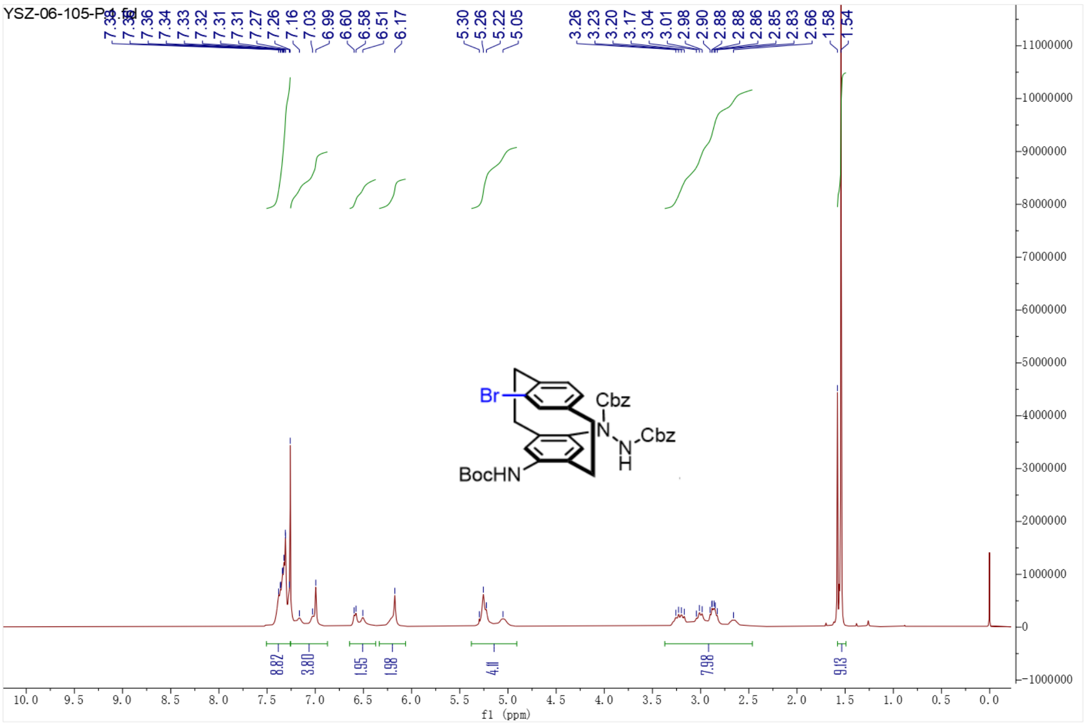

Supplementary Fig. 473 ^{1}H NMR spectrum of (S_p)-**3m** (400 MHz, $CDCl_3$)

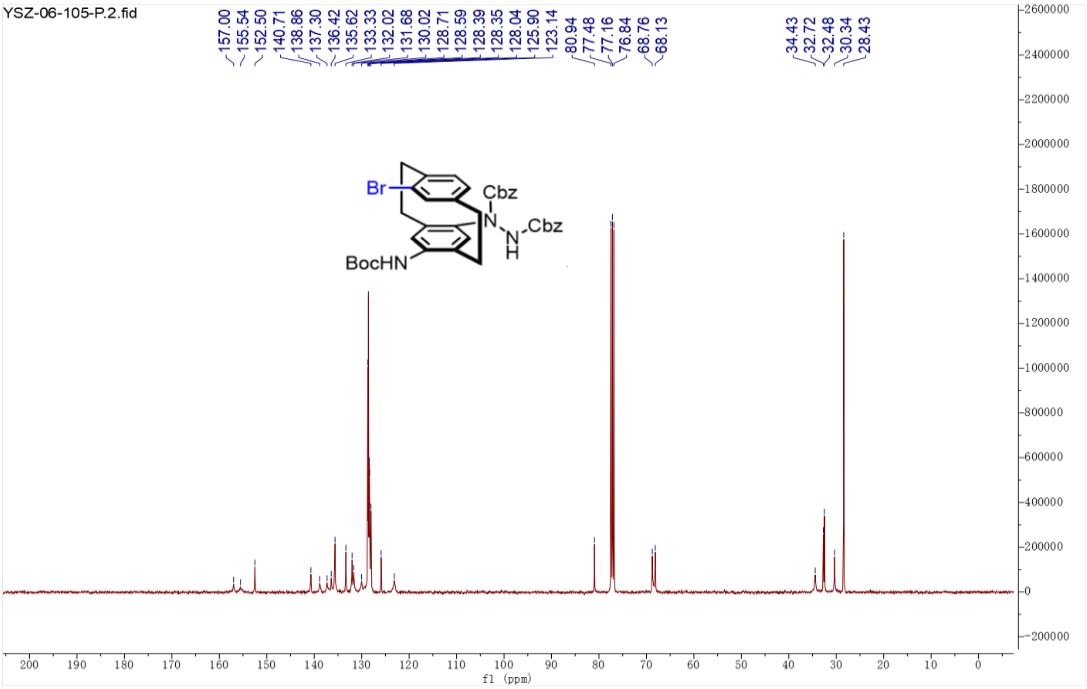

Supplementary Fig. 474 ^{13}C NMR spectrum of (S_p)-**3m** (101 MHz, $CDCl_3$)

(R_p)-Tert-butyl (4[3]-phenyl-1,4(1,4)-dibenzenacyclohexaphane-1[2]-yl)carbamate

((R_p)-1n)

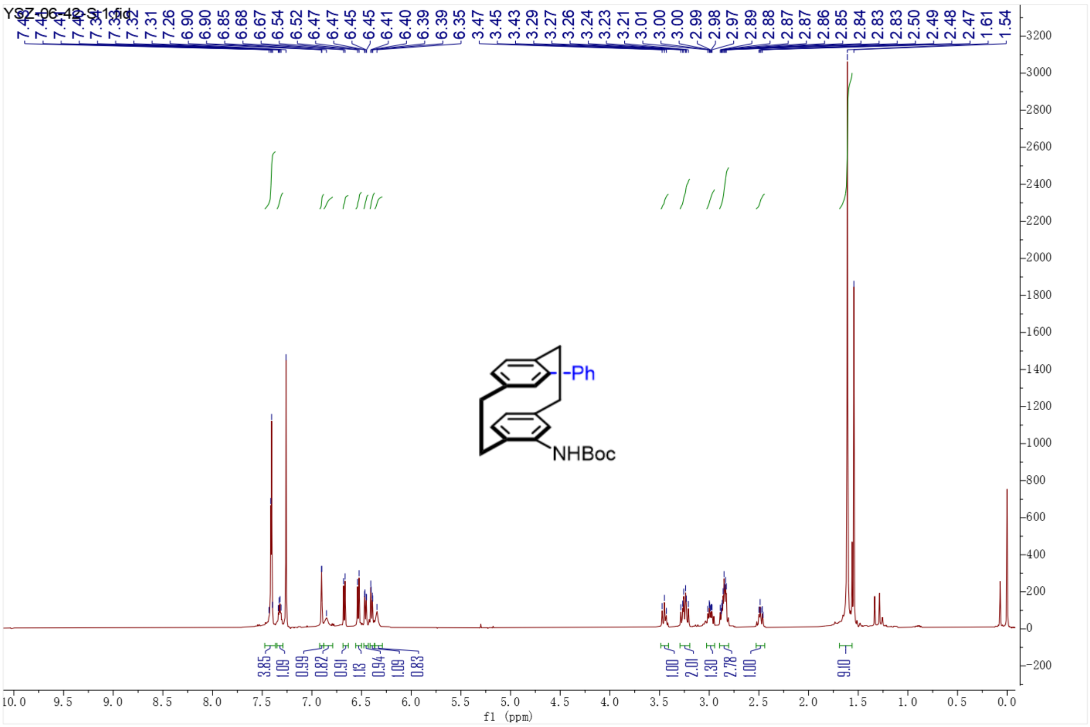

Supplementary Fig. 475 ^{1}H NMR spectrum of (R_p)**-1n** (500 MHz, $CDCl_3$)

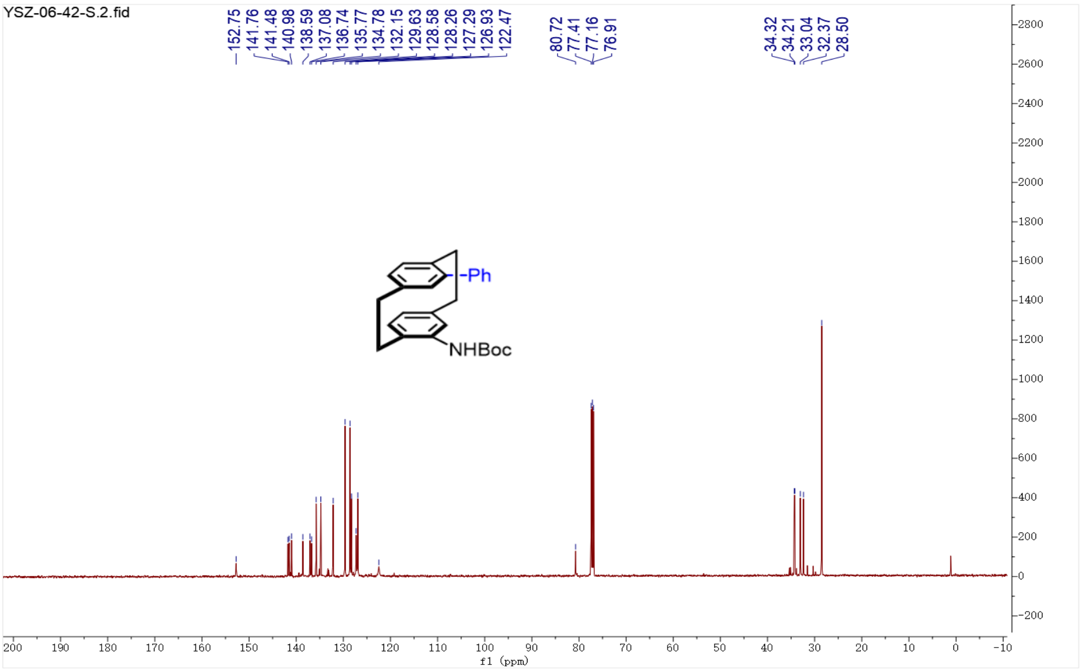

Supplementary Fig. 476 ^{13}C NMR spectrum of (R_p)**-1n** (126 MHz, $CDCl_3$)

(S_p)-Dibenzyl-1-(1^5-((tert-butoxycarbonyl)amino)-4^2-phenyl-1,4(1,4)-dibenzenacyclo hexaphane-1^2-yl)hydrazine-1,2-dicarboxylate **(3n)**

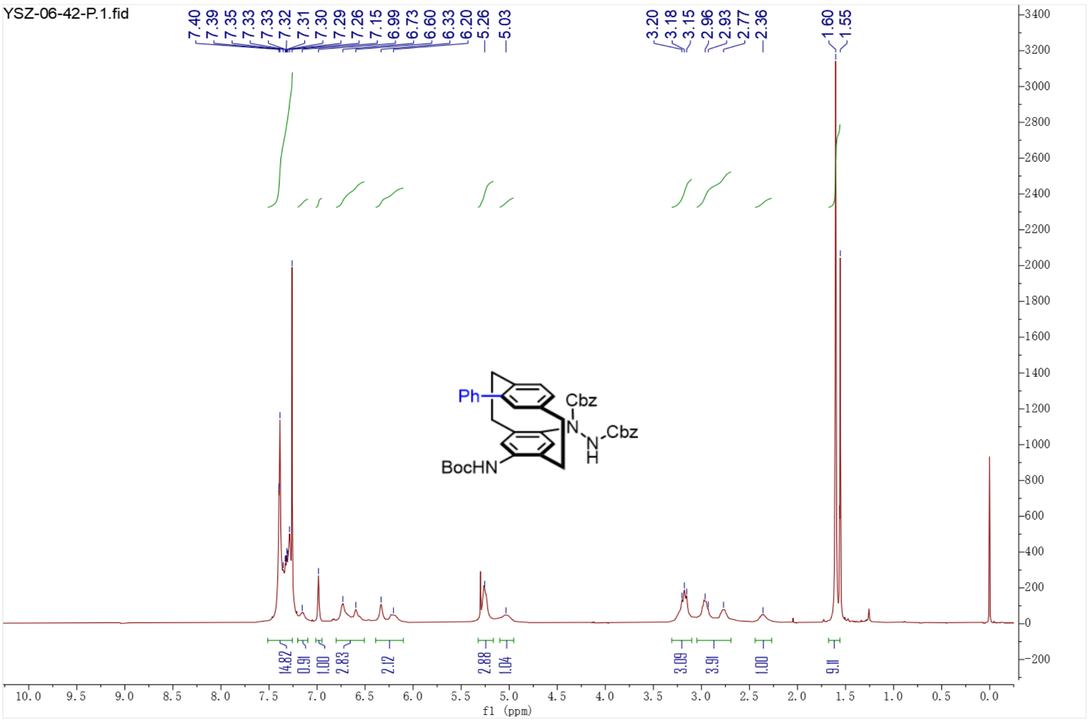

Supplementary Fig. 477 ^{1}H NMR spectrum of (S_p)-**3n** (500 MHz, $CDCl_3$)

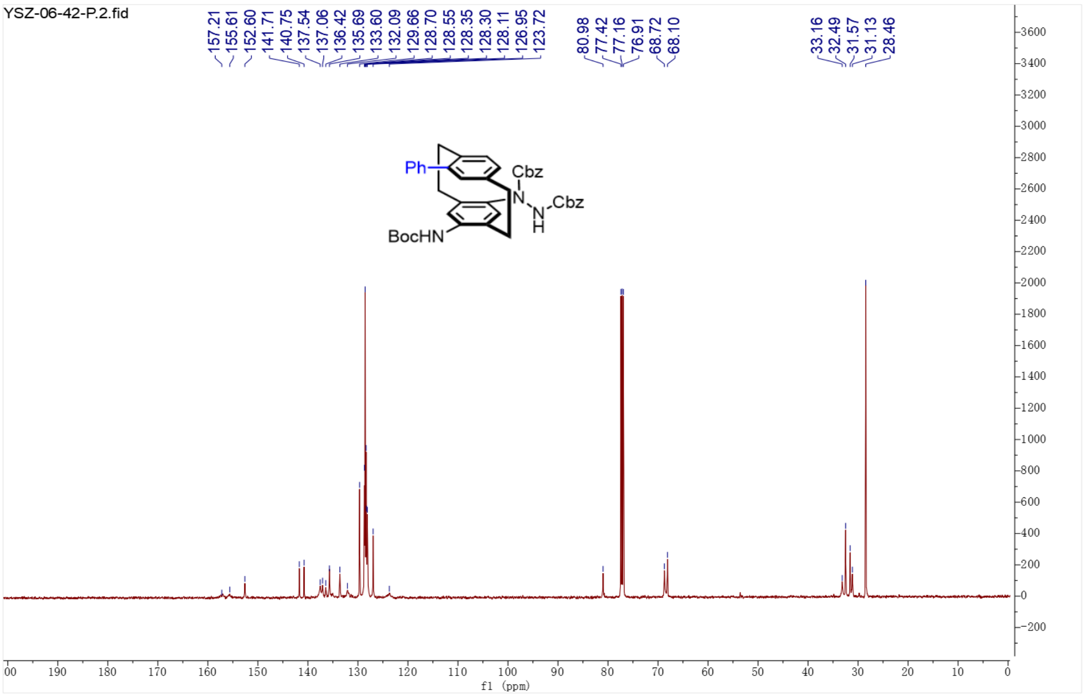

Supplementary Fig. 478 ^{13}C NMR spectrum of (S_p)-**3n** (126 MHz, $CDCl_3$)

(R_p)-Tert-butyl (4[3]-(cyclohex-1-en-1-yl)-1,4(1,4)-dibenzenacyclohexaphane-1[2]-yl)carbamate **((*R*$_p$)-1o)**

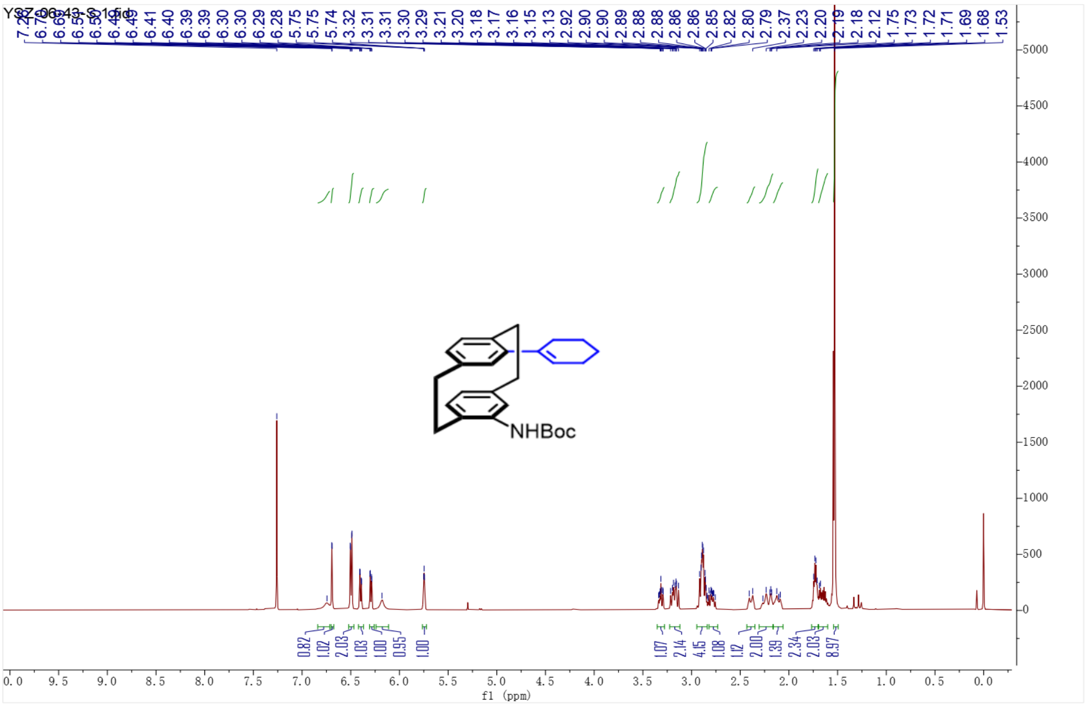

Supplementary Fig. 479 ^{1}H NMR spectrum of (R_p)**-1o** (500 MHz, $CDCl_3$)

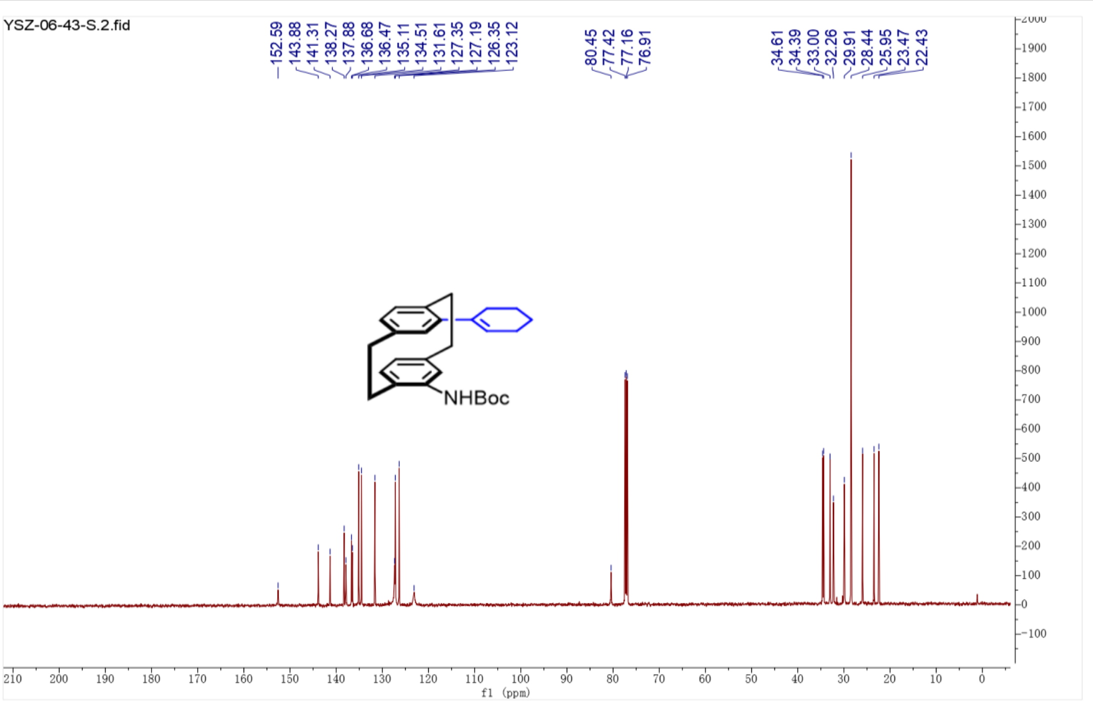

Supplementary Fig. 480 ^{13}C NMR spectrum of (R_p)**-1o** (126 MHz, $CDCl_3$)

(S_p)-Dibenzyl 1-(1[5]-((tert-butoxycarbonyl)amino)-4[2]-(cyclohex-1-en-1-yl)-1,4(1,4)-dibenzenacyclohexaphane-1[2]-yl)hydrazine-1,2-dicarboxylate **(3o)**

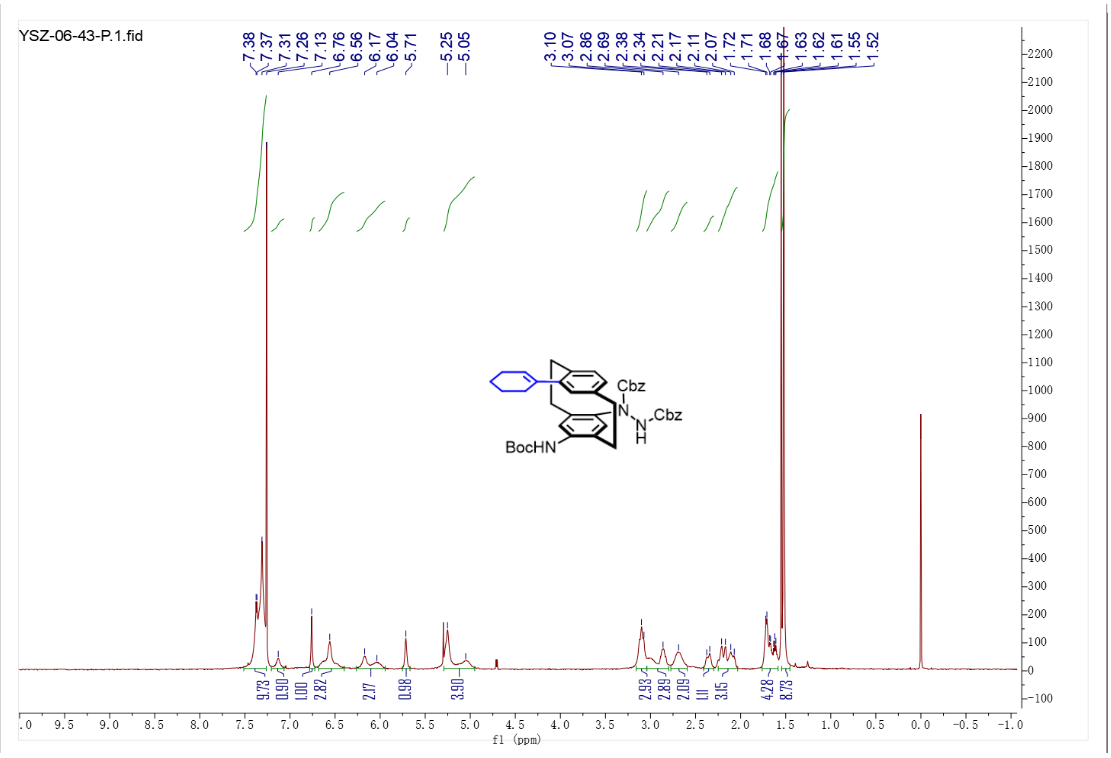

Supplementary Fig. 481 ^{1}H NMR spectrum of (S_p)-**3o** (500 MHz, $CDCl_3$)

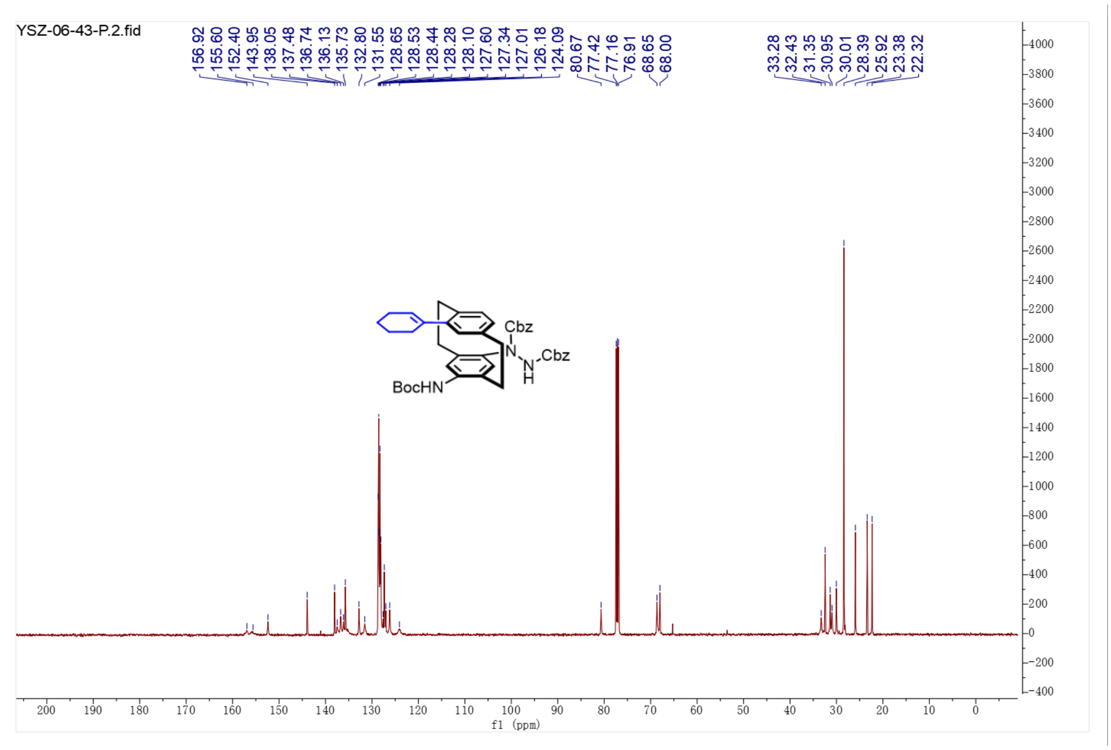

Supplementary Fig. 482 ^{13}C NMR spectrum of (S_p)-**3o** (126 MHz, $CDCl_3$)

(R_p)-Tert-butyl (E)-(4^3-styryl-1,4(1,4)-dibenzenacyclohexaphane-1^2-yl)carbamate ((R_p)-**1p**)

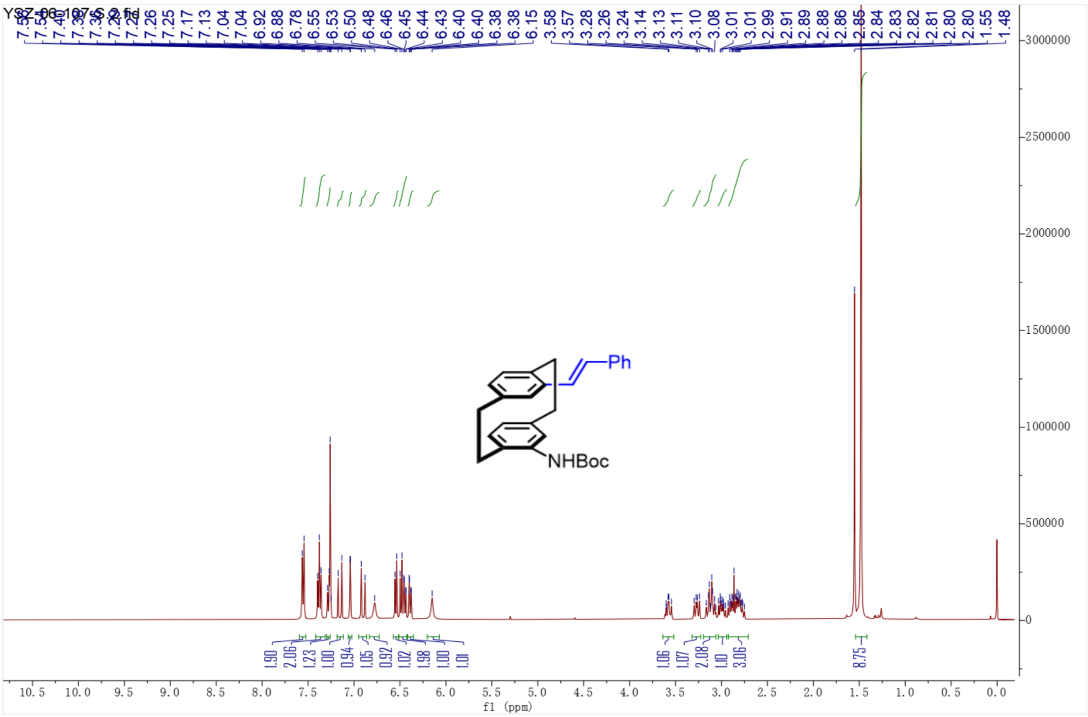

Supplementary Fig. 483 ^{1}H NMR spectrum of (R_p)-**1p** (400 MHz, $CDCl_3$)

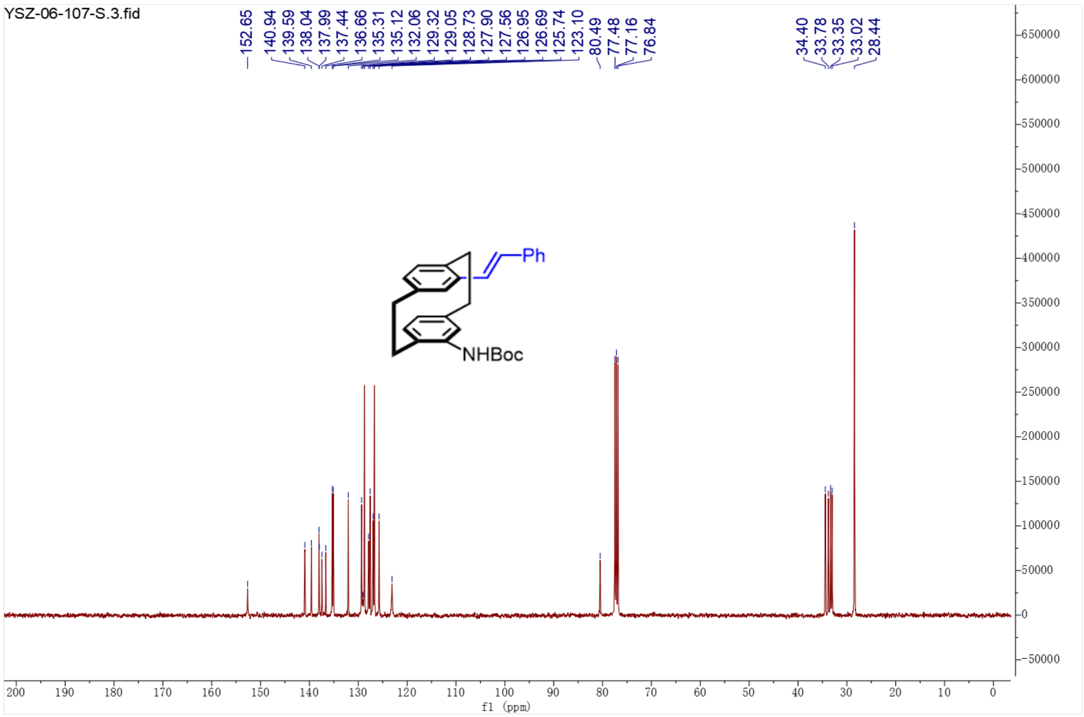

Supplementary Fig. 484 ^{13}C NMR spectrum of (R_p)-**1p** (101 MHz, $CDCl_3$)

(S_p)-Dibenzyl (E)-1-(1^5-((tert-butoxycarbonyl)amino)-4^2-styryl-1,4(1,4) -dibenzenacyclohexaphane-1^2-yl)hydrazine-1,2-dicarboxylate **(3p)**

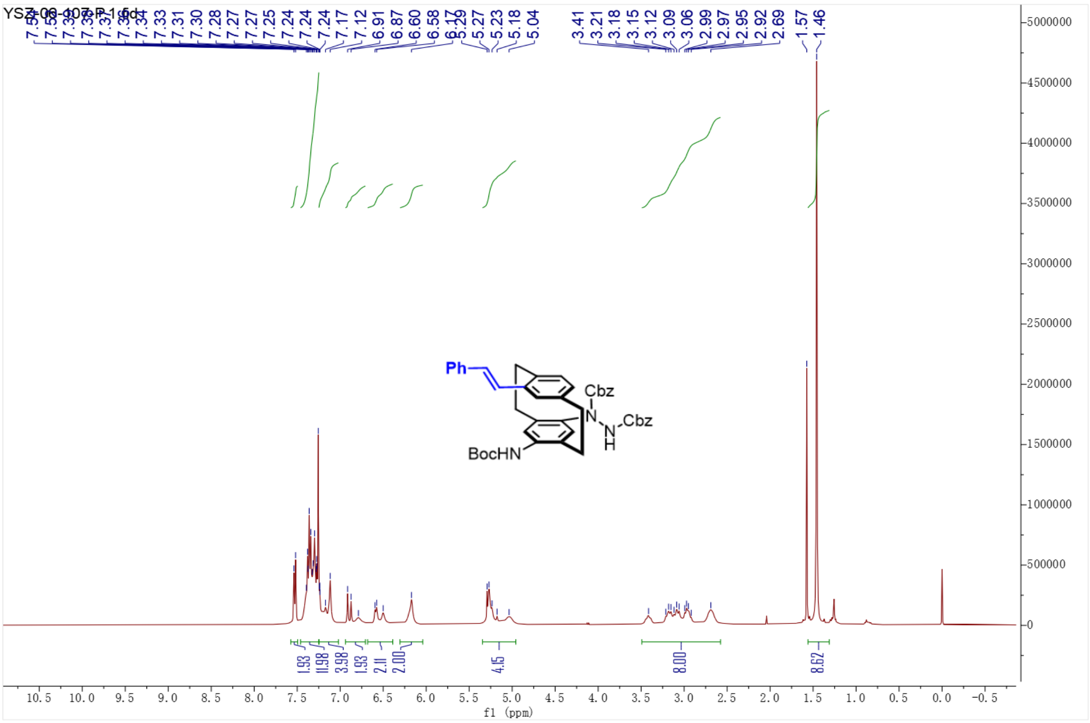

Supplementary Fig. 485 ^{1}H NMR spectrum of (S_p)-**3p** (400 MHz, $CDCl_3$)

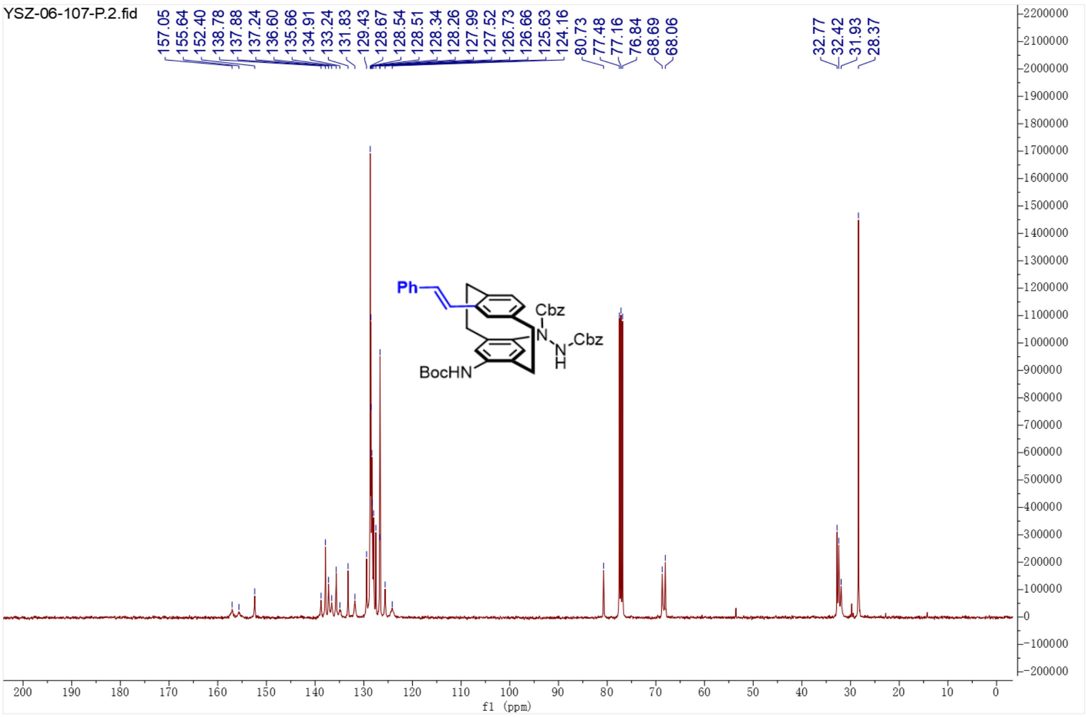

Supplementary Fig. 486 ^{13}C NMR spectrum of (S_p)-**3p** (101 MHz, $CDCl_3$)

(R_p)-Tert-butyl-(4^3-(phenylethynyl)-1,4(1,4)-dibenzenacyclohexaphane-1^2-yl)carbamate **((R_p)-1q)**

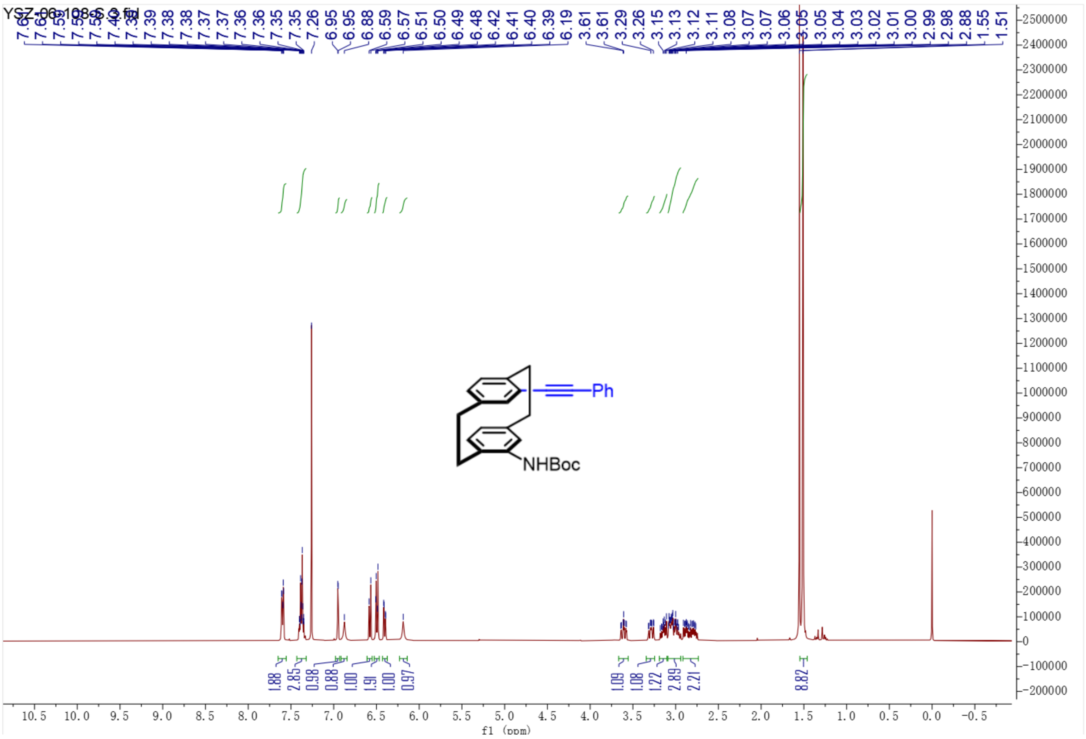

Supplementary Fig. 487 ^{1}H NMR spectrum of (R_p)-**1q** (400 MHz, $CDCl_3$)

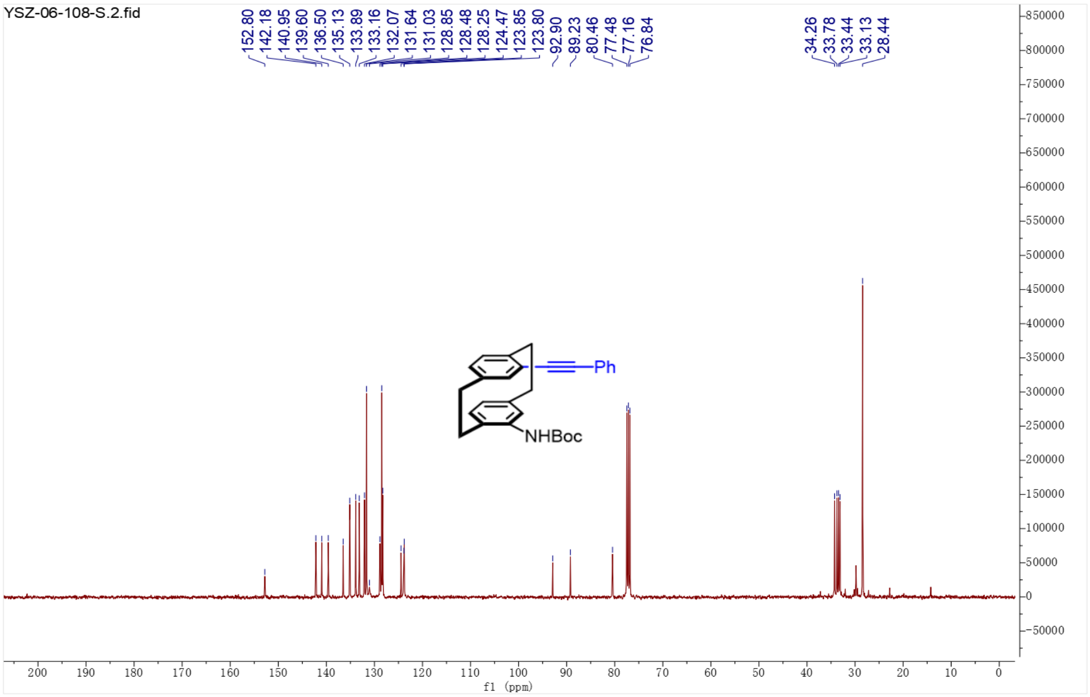

Supplementary Fig. 488 ^{13}C NMR spectrum of (R_p)-**1q** (101 MHz, $CDCl_3$)

(S_p)-Dibenzyl 1-(1^5-((tert-butoxycarbonyl)amino)-4^2-(phenylethynyl)-1,4(1,4)-dibenzenacyclohexaphane-1^2-yl)hydrazine-1,2-dicarboxylate **(3q)**

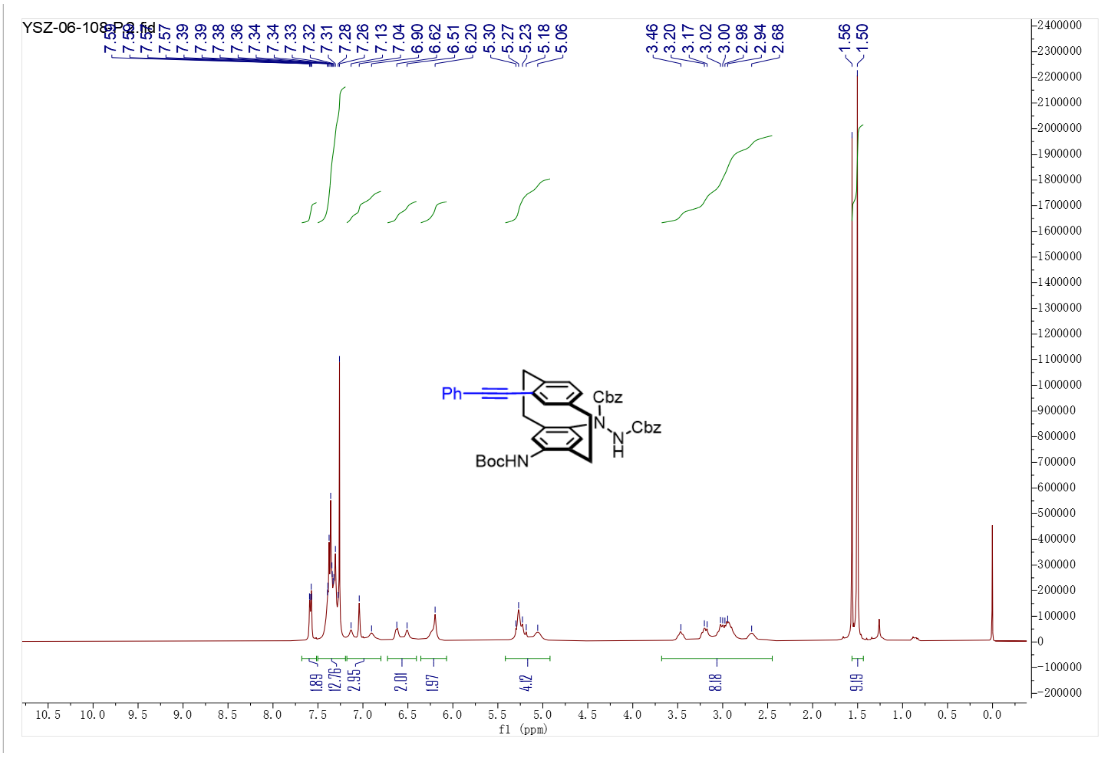

Supplementary Fig. 489 ^{1}H NMR spectrum of (S_p)-**3q** (400 MHz, $CDCl_3$)

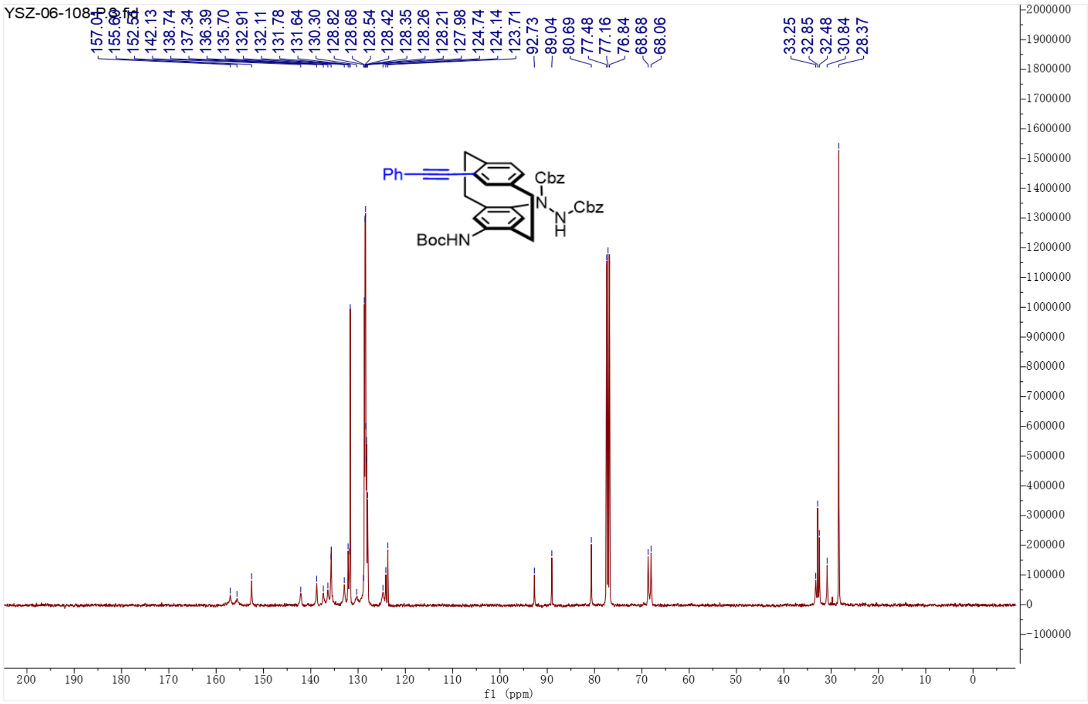

Supplementary Fig. 490 ^{13}C NMR spectrum of (S_p)-**3q** (101 MHz, $CDCl_3$)

(R_p)-Tert-butyl (4³-cyclohexyl-1,4(1,4)-dibenzenacyclohexaphane-1²-yl)carbamate **((R_p)-1r)**

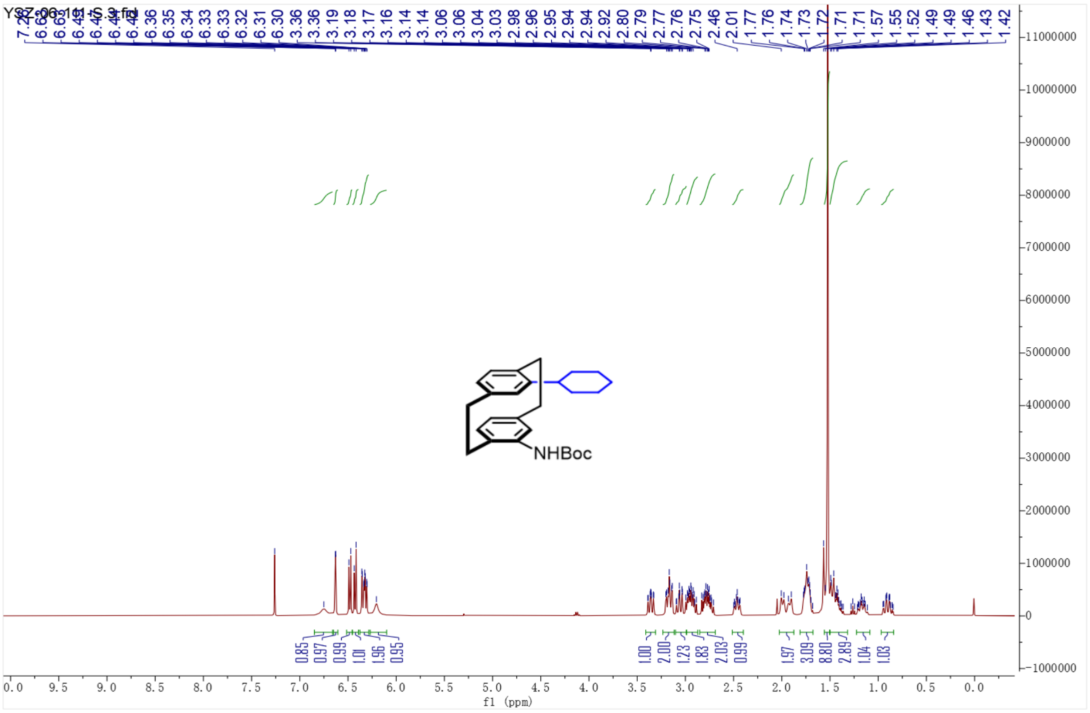

Supplementary Fig. 491 ¹H NMR spectrum of (R_p)-**1r** (400 MHz, $CDCl_3$)

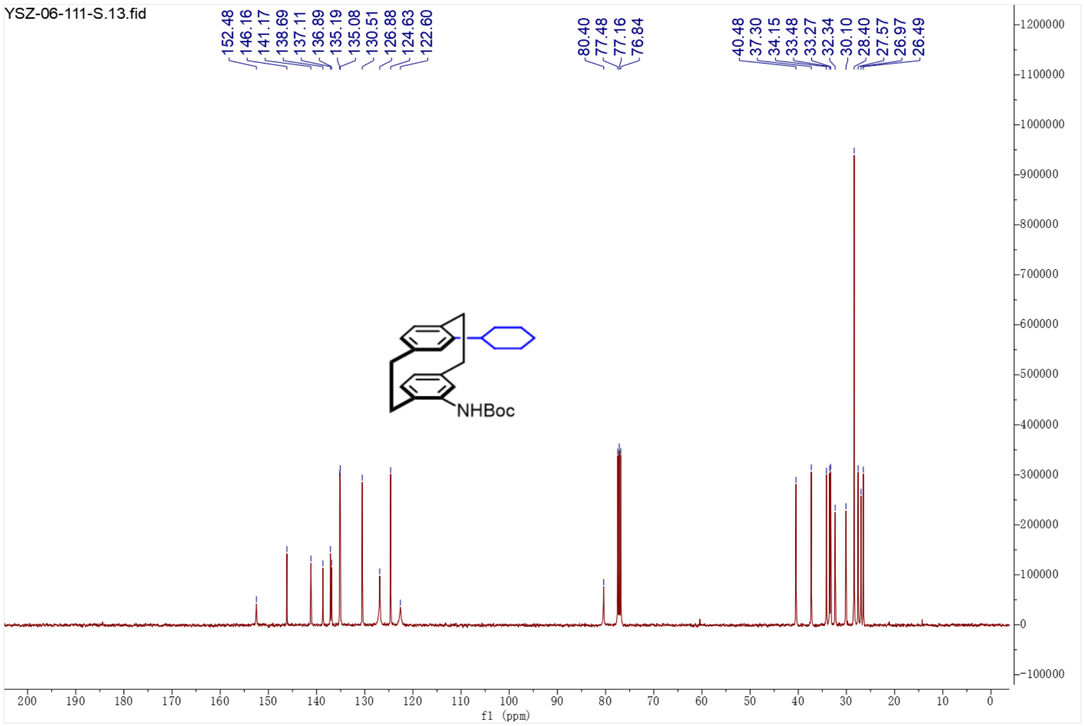

Supplementary Fig. 492 ¹³C NMR spectrum of (R_p)-**1r** (101 MHz, $CDCl_3$)

(S_p)-Dibenzyl 1-(1[5]-((tert-butoxycarbonyl)amino)-4[2]-cyclohexyl-1,4(1,4)-dibenzenacyclohexaphane-1[2]-yl)hydrazine-1,2-dicarboxylate **(3r)**

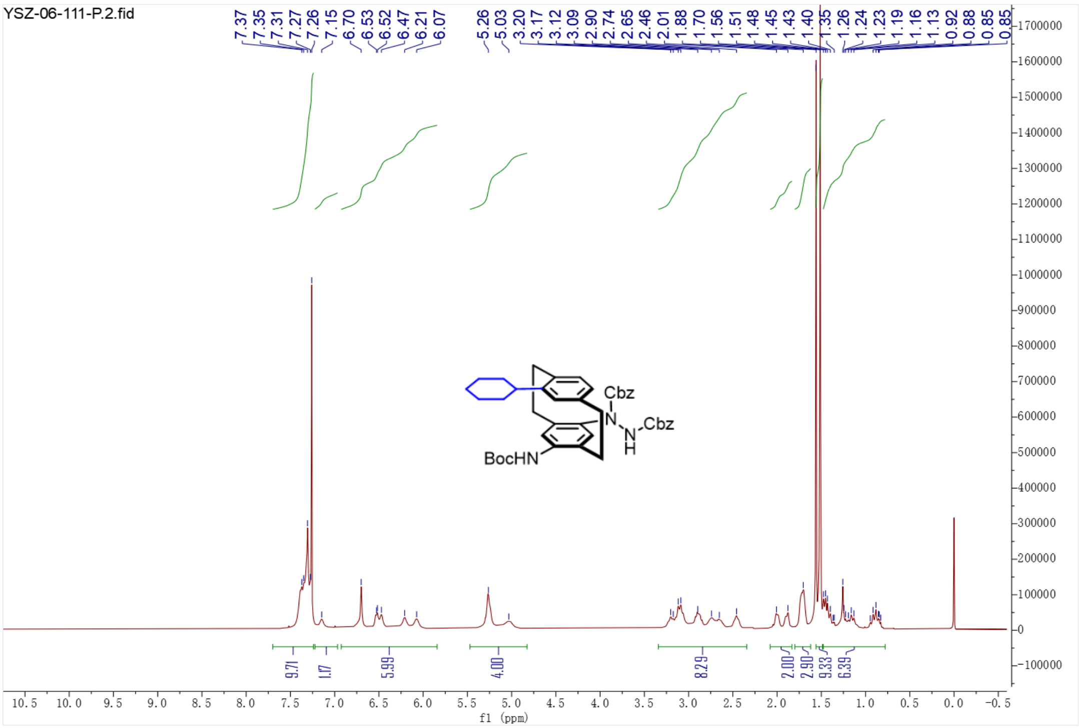

Supplementary Fig. 493 ^{1}H NMR spectrum of (S_p)-**3r** (400 MHz, $CDCl_3$)

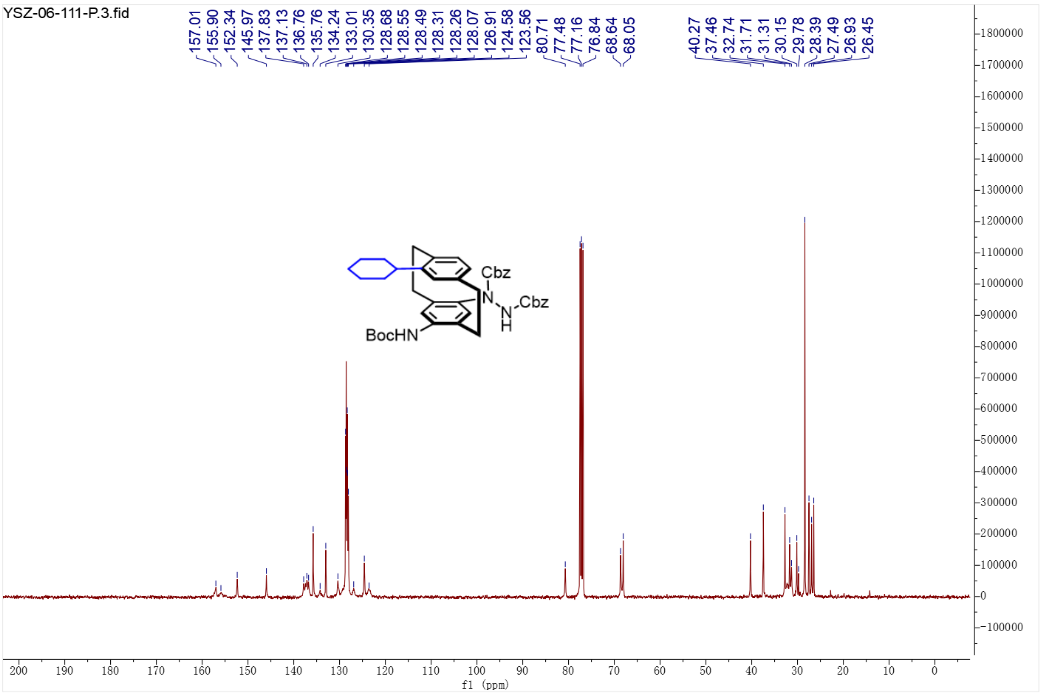

Supplementary Fig. 494 ^{13}C NMR spectrum of (S_p)-**3r** (101 MHz, $CDCl_3$)

(R_p)-4[3]-((tert-butoxycarbonyl)amino)-1,4(1,4)-dibenzenacyclohexaphane-1[2]-yl acetate (**(R_p)-1s**)

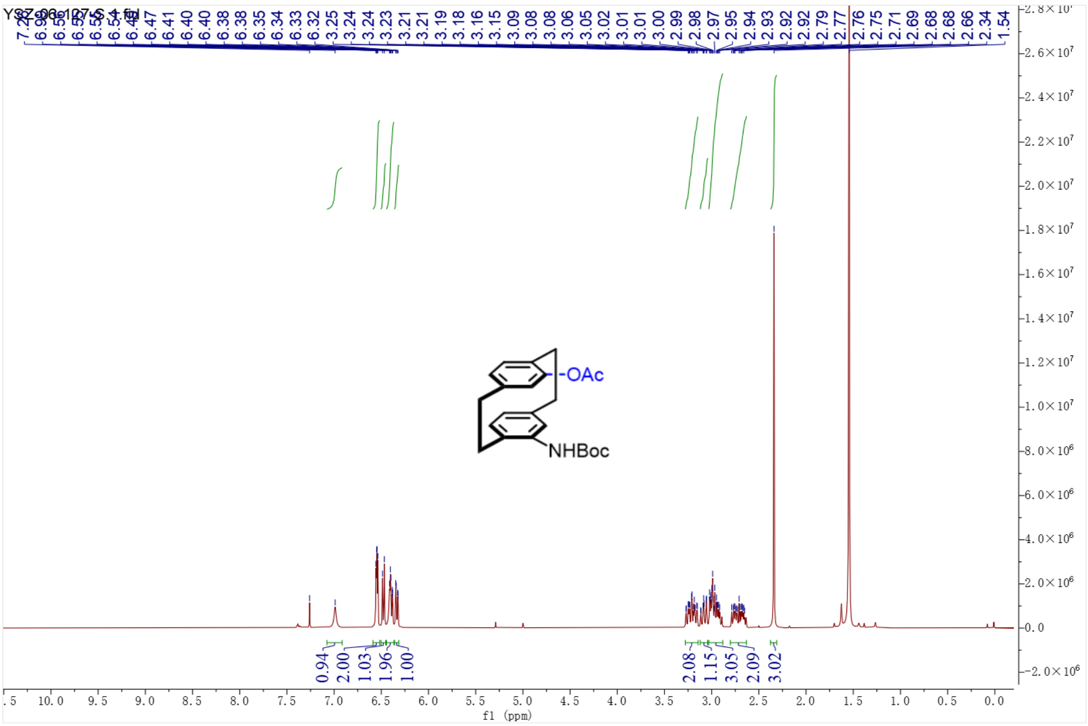

Supplementary Fig. 495 ^{1}H NMR spectrum of (R_p)-**1s** (400 MHz, $CDCl_3$)

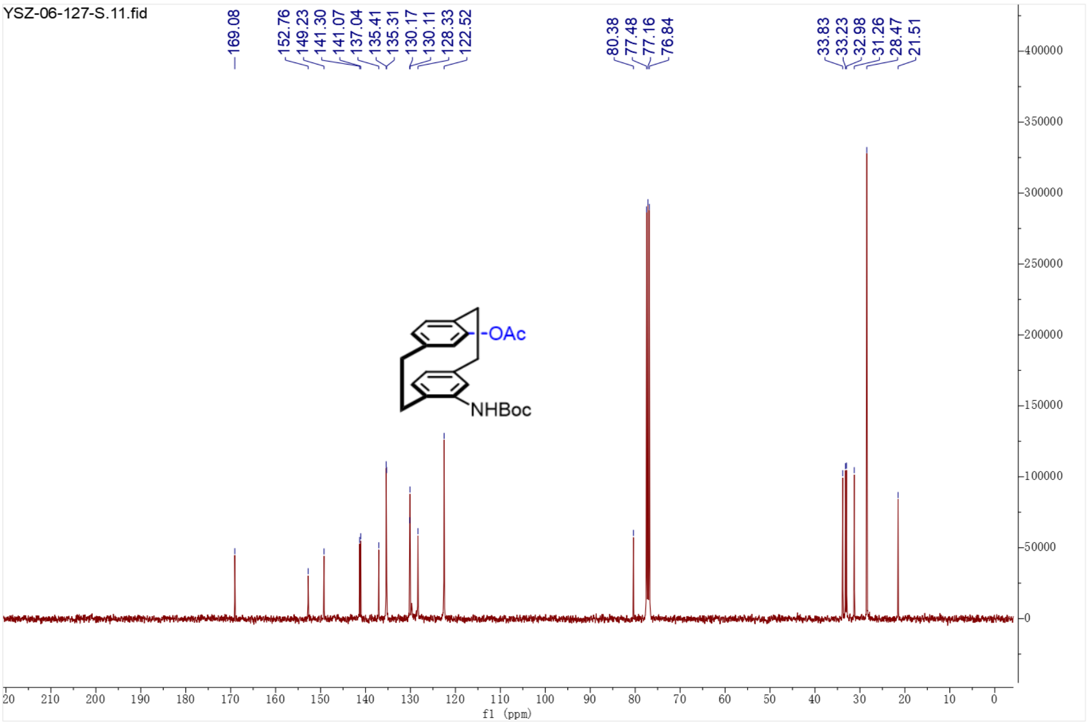

Supplementary Fig. 496 ^{13}C NMR spectrum of (R_p)-**1s** (101 MHz, $CDCl_3$)

(S_p)-Dibenzyl 1-(4^2-acetoxy-1^5-((tert-butoxycarbonyl)amino)-1,4(1,4)-dibenzenacyclohexaphane-1^2-yl)hydrazine-1,2-dicarboxylate **(3s)**

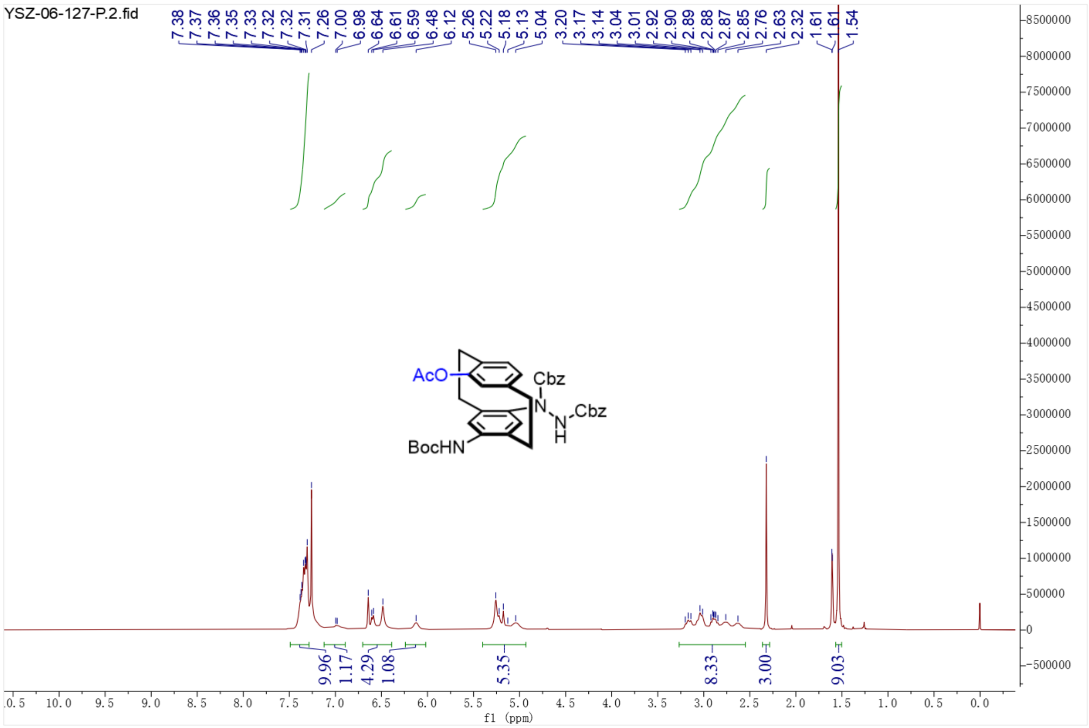

Supplementary Fig. 497 ^{1}H NMR spectrum of (S_p)-**3s** (400 MHz, $CDCl_3$)

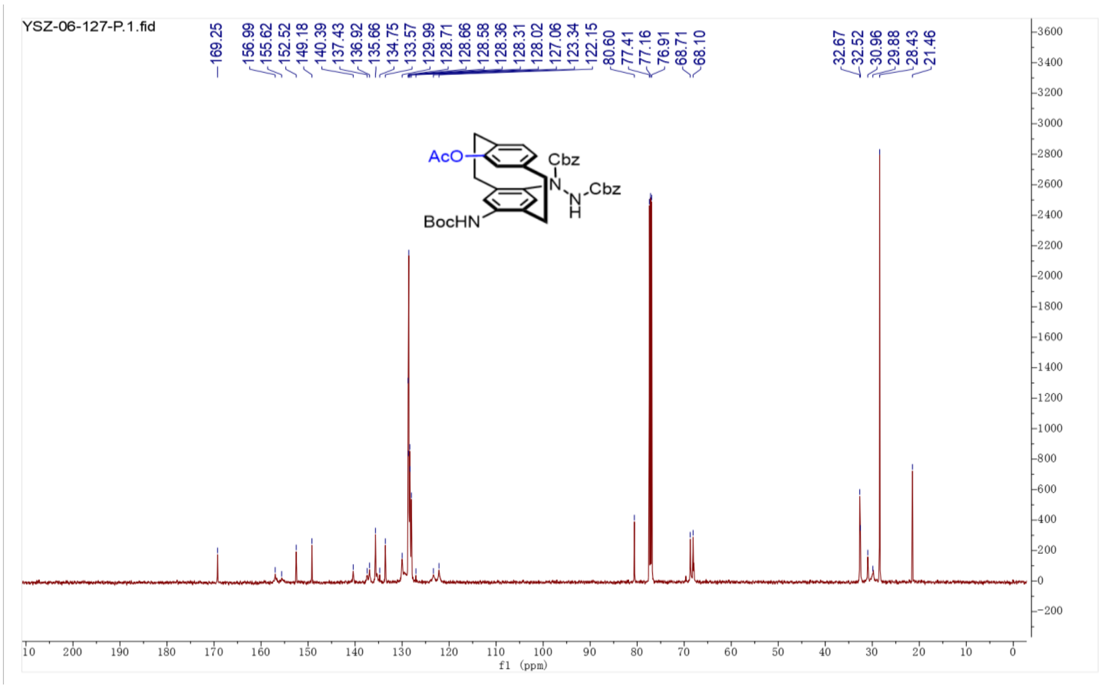

Supplementary Fig. 498 ^{13}C NMR spectrum of (S_p)-**3s** (126 MHz, $CDCl_3$)

(R_p)-Tert-butyl (4[3]-formyl-1,4(1,4)-dibenzenacyclohexaphane-1[2]-yl)carbamate **((R_p)-1t)**

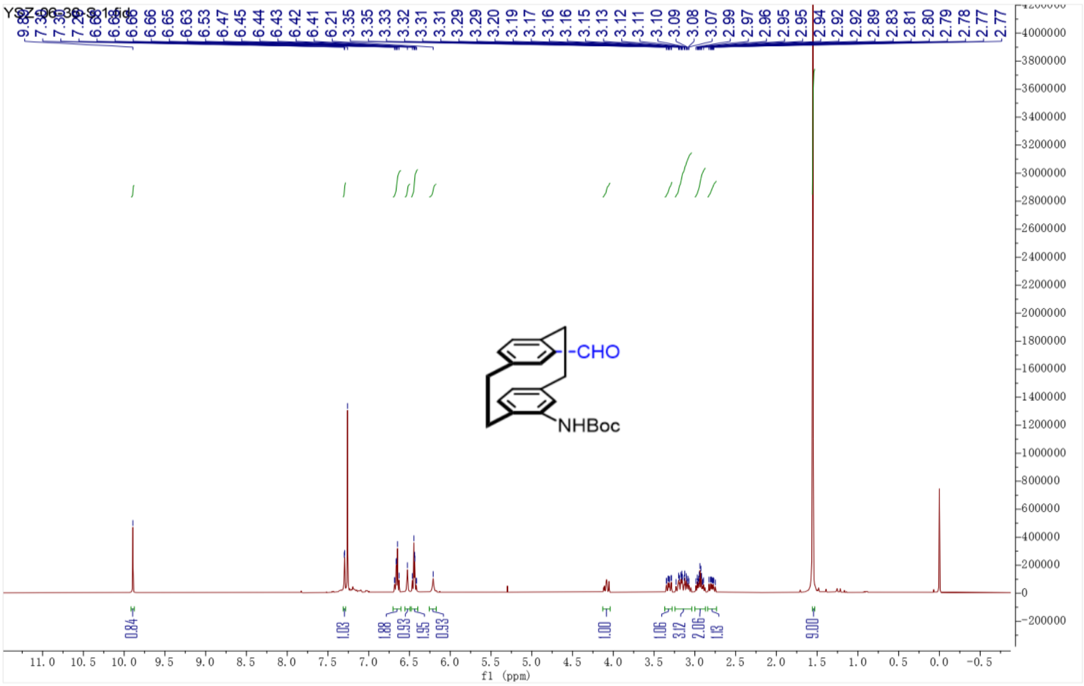

Supplementary Fig. 499 ^{1}H NMR spectrum of (R_p)**-1t** (400 MHz, $CDCl_3$)

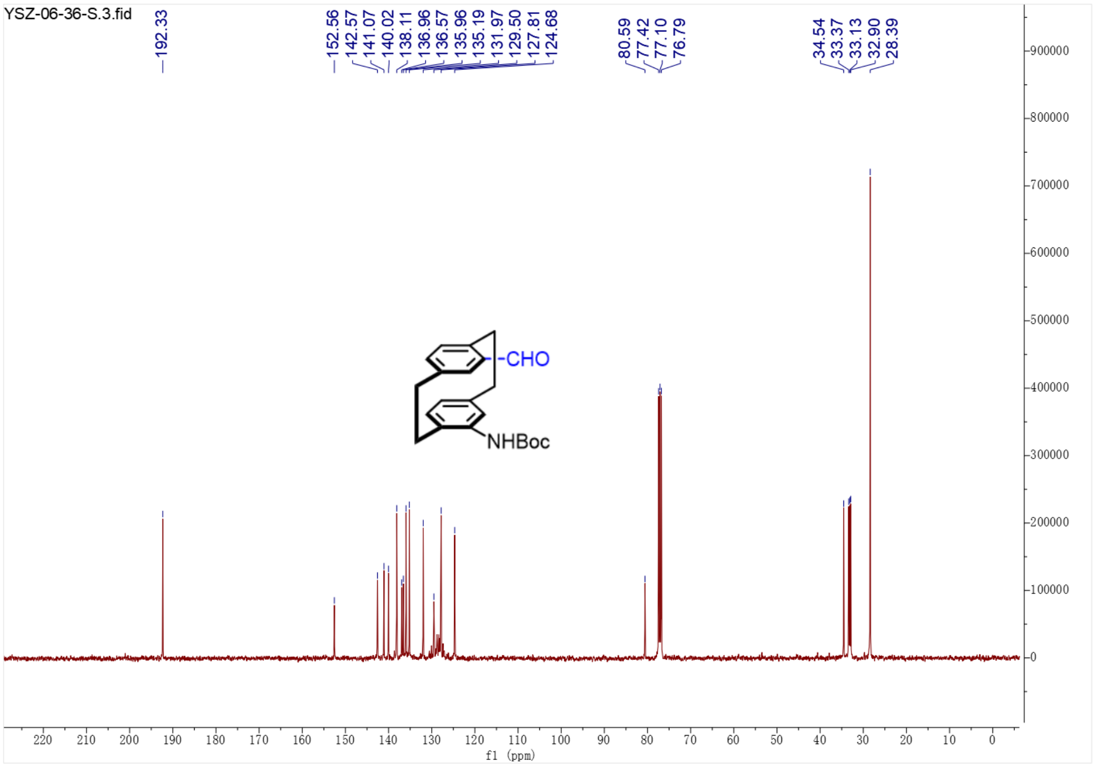

Supplementary Fig. 500 ^{13}C NMR spectrum of (R_p)**-1t** (101 MHz, $CDCl_3$)

(S_p)-Dibenzyl 1-(1^5-((tert-butoxycarbonyl)amino)-4^2-formyl-1,4(1,4)-dibenzenacyclohexaphane-1^2-yl)hydrazine-1,2-dicarboxylate **(3t)**

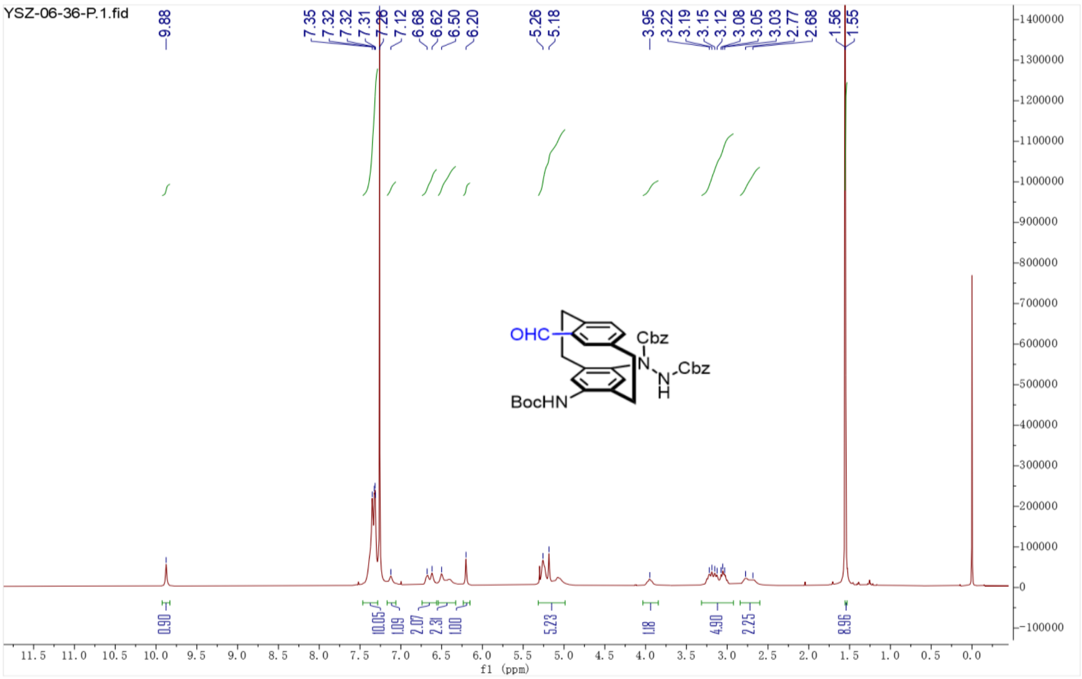

Supplementary Fig. 501 ^{1}H NMR spectrum of (S_p)-**3t** (400 MHz, $CDCl_3$)

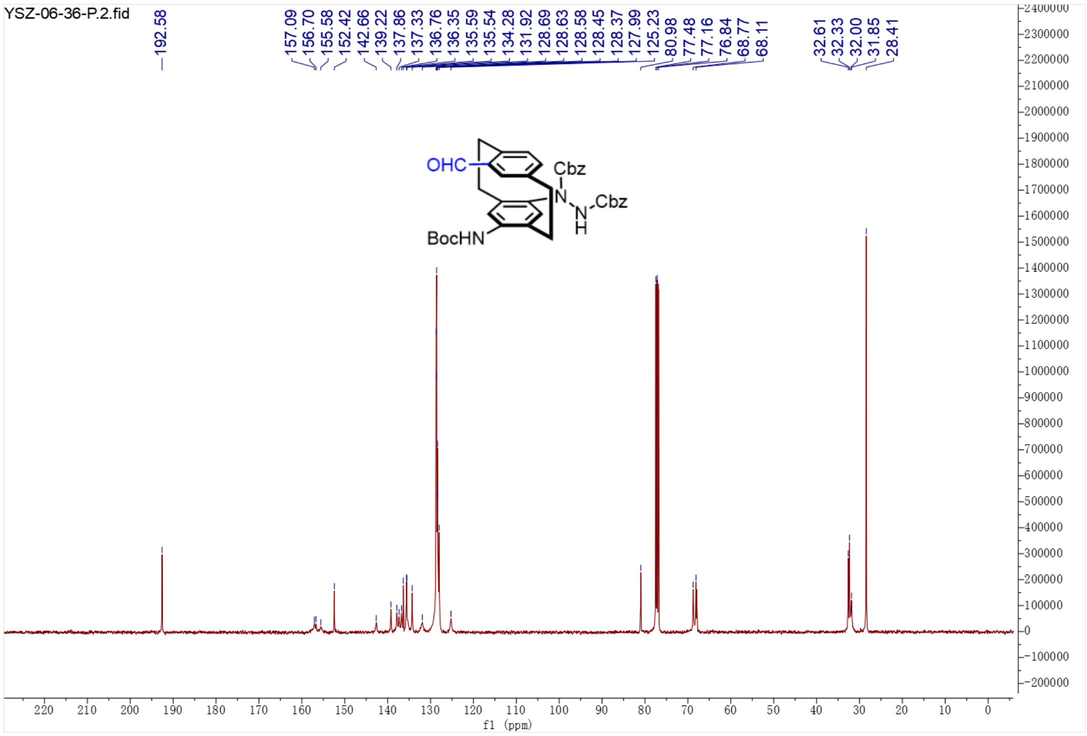

Supplementary Fig. 502 ^{13}C NMR spectrum of (S_p)-**3t** (101 MHz, $CDCl_3$)

(R_p)-Tert-butyl-(4^3-(hydroxymethyl)-1,4(1,4)-dibenzenacyclohexaphane-1^2-yl)carbamate **((R_p)-1u)**

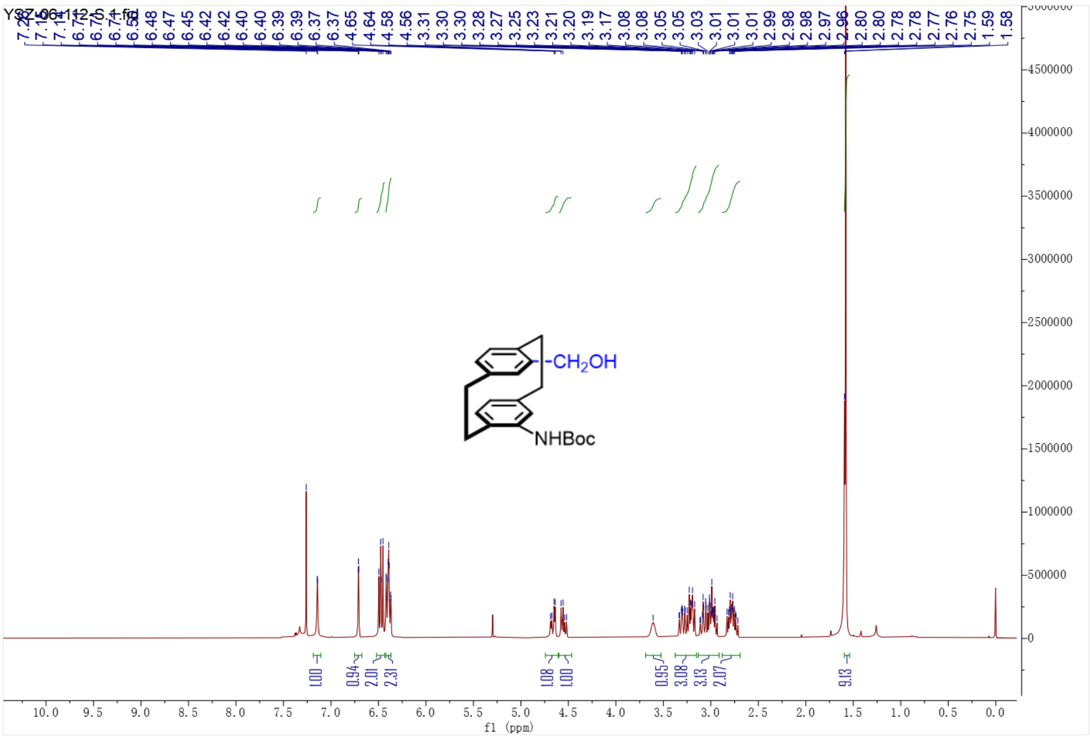

Supplementary Fig. 503 ^{1}H NMR spectrum of (R_p)**-1u** (400 MHz, $CDCl_3$)

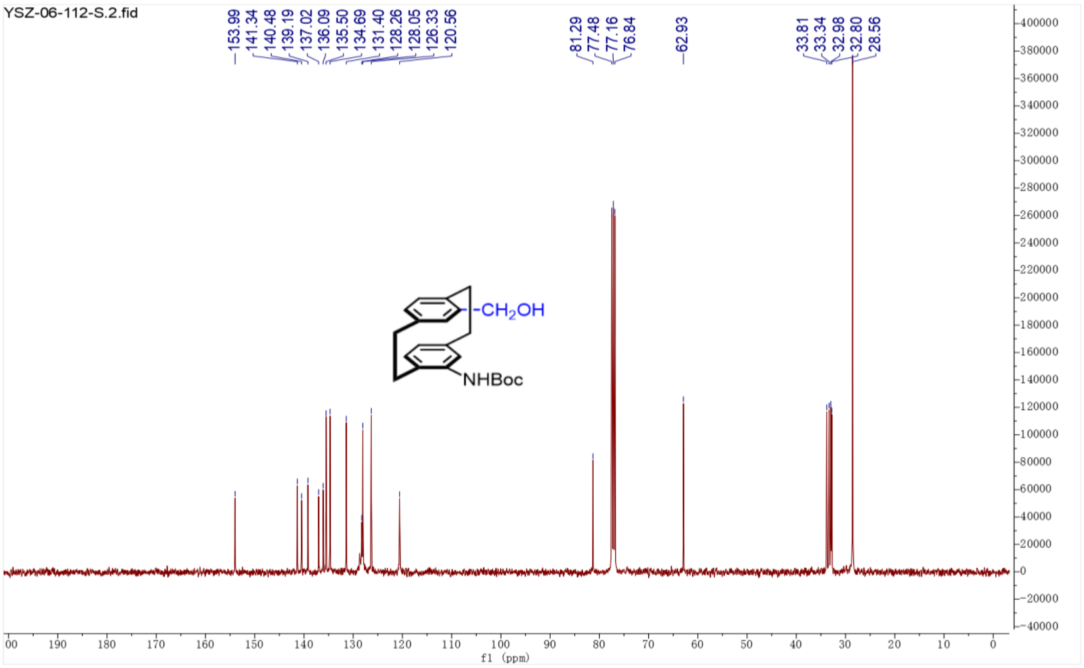

Supplementary Fig. 504 ^{13}C NMR spectrum of (R_p)**-1u** (101 MHz, $CDCl_3$)

(S_p)-Dibenzyl 1-(1^5-((tert-butoxycarbonyl)amino)-4^2-(hydroxymethyl)-1,4(1,4)-dibenzenacyclohexaphane-1^2-yl)hydrazine-1,2-dicarboxylate **(3u)**

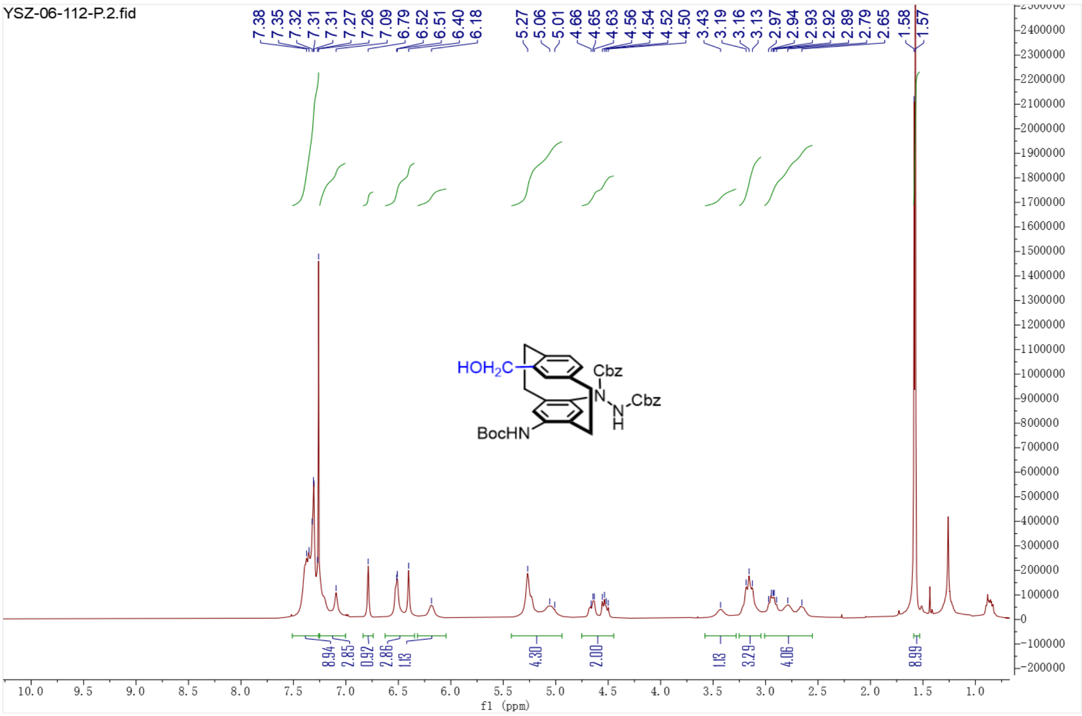

Supplementary Fig. 505 ^{1}H NMR spectrum of (S_p)-**3u** (400 MHz, $CDCl_3$)

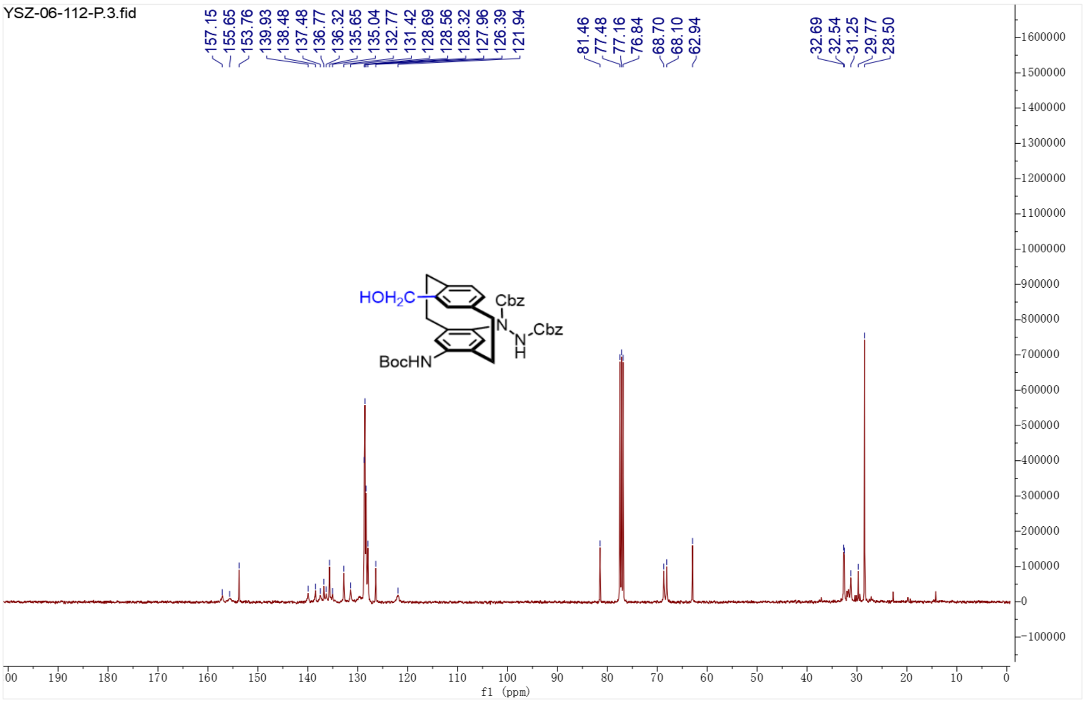

Supplementary Fig. 506 ^{1}H NMR spectrum of (S_p)-**3u** (101 MHz, $CDCl_3$)

(R_p)-Methyl 4[3]-((tert-butoxycarbonyl)amino)-1,4(1,4)-dibenzenacyclohexaphane-1[2]-carboxylate **((R_p)-1v)**

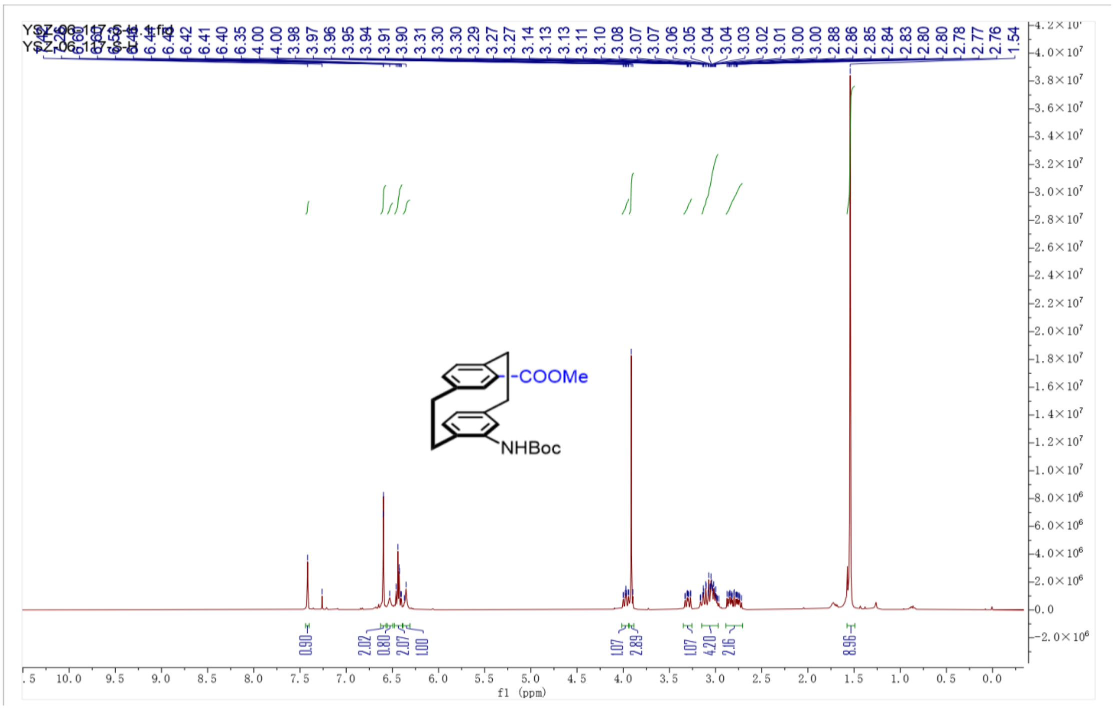

Supplementary Fig. 507 ^{1}H NMR spectrum of (R_p)-**1v** (400 MHz, $CDCl_3$)

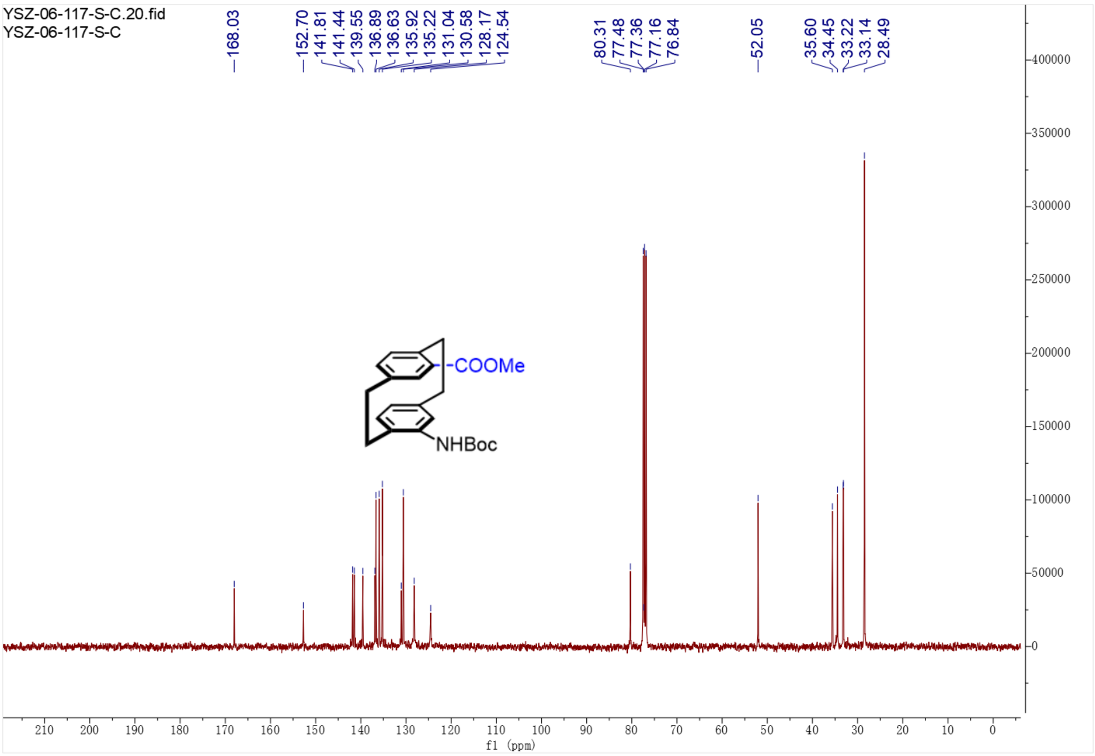

Supplementary Fig. 508 ^{13}C NMR spectrum of (R_p)-**1v** (101 MHz, $CDCl_3$)

(S_p)-Dibenzyl 1-(1[5]-((tert-butoxycarbonyl)amino)-4[2]-(methoxycarbonyl)-1,4(1,4)-dibenzenacyclohexaphane-1[2]-yl)hydrazine-1,2-dicarboxylate **(3v)**

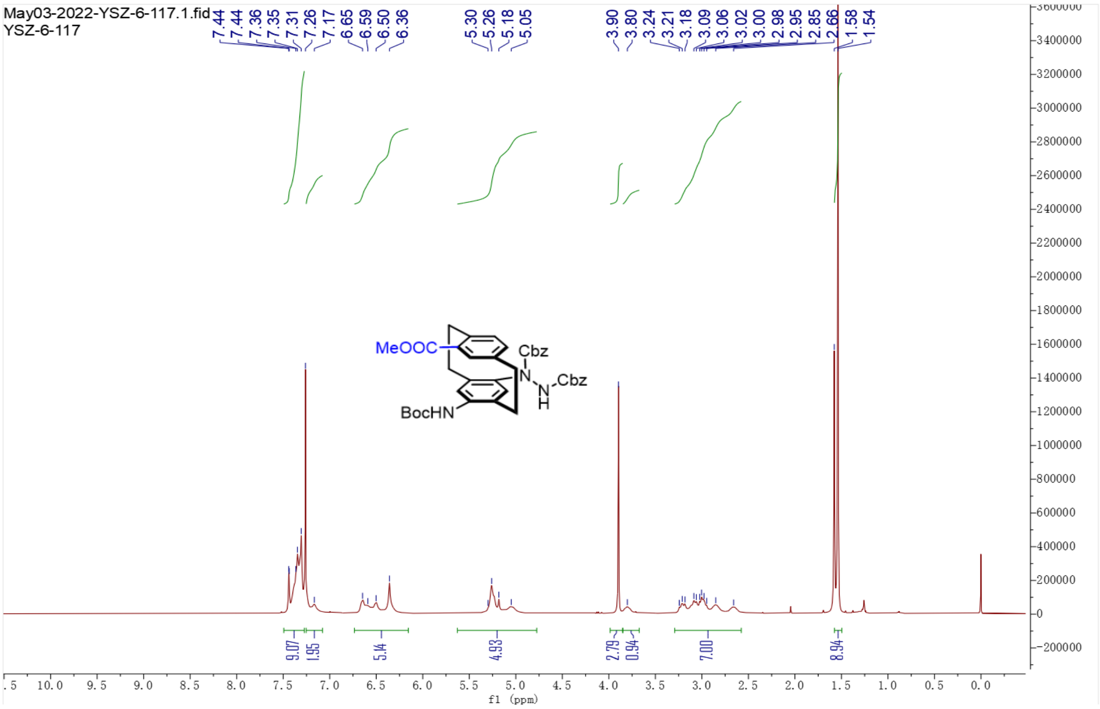

Supplementary Fig. 509 ^{1}H NMR spectrum of (S_p)-**3v** (400 MHz, $CDCl_3$)

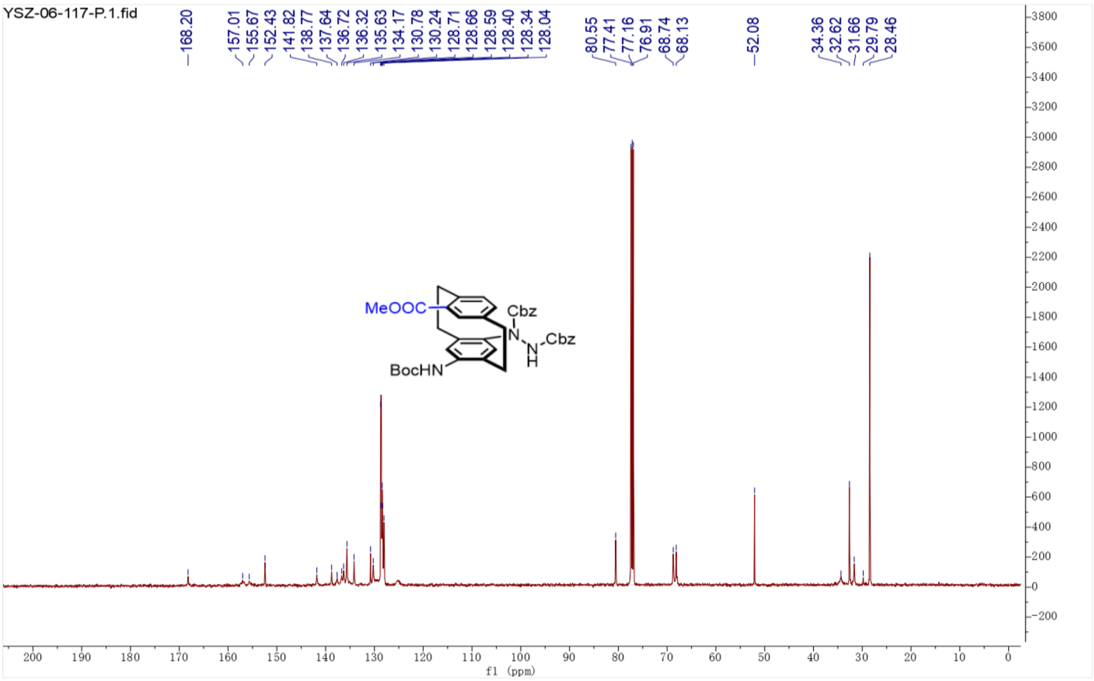

Supplementary Fig. 510 ^{13}C NMR spectrum of (S_p)-**3v** (126 MHz, $CDCl_3$)

(R_p)-Tert-butyl (4^2-bromo-1,4(1,4)-dibenzenacyclohexaphane-1^2-yl)carbamate **((R_p)-1w)**

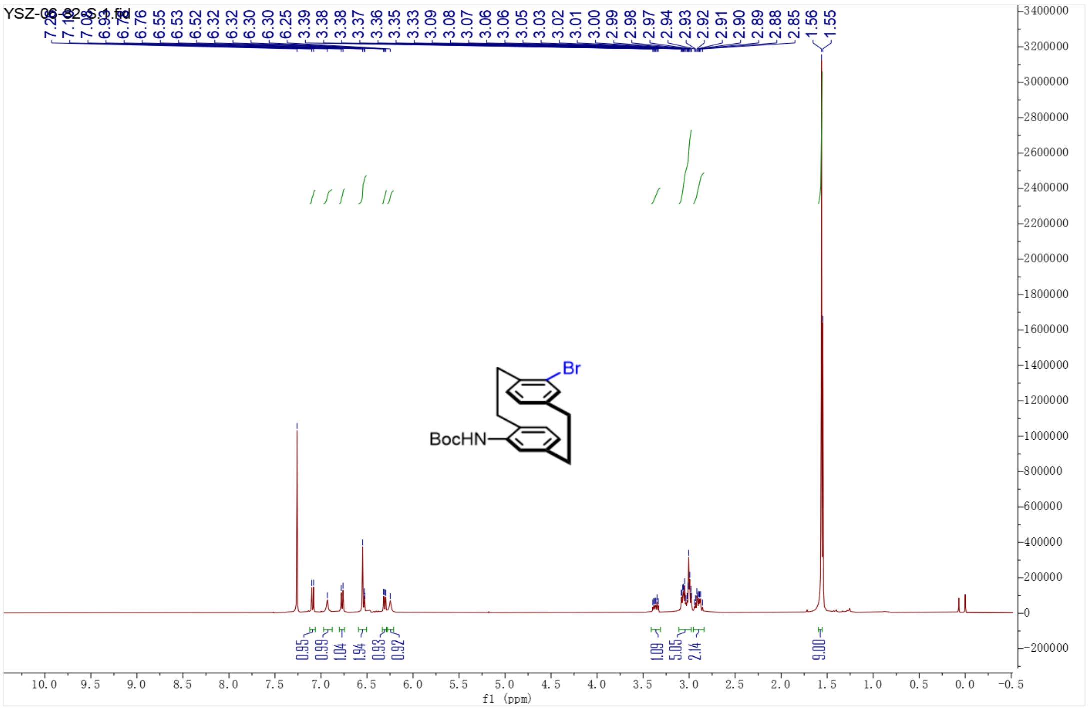

Supplementary Fig. 511 ^{1}H NMR spectrum of (R_p)-**1w** (400 MHz, $CDCl_3$)

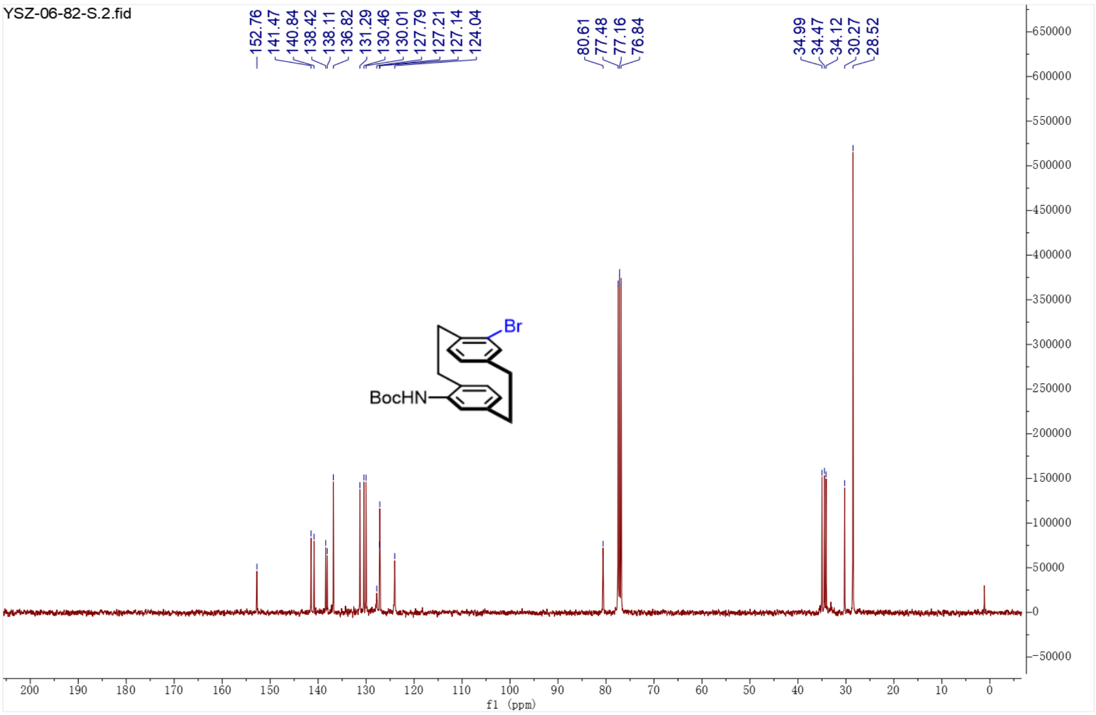

Supplementary Fig. 512 ^{13}C NMR spectrum of (R_p)-**1w** (101 MHz, $CDCl_3$)

(S_p)-Dibenzyl 1-(4^3-bromo-1^5-((tert-butoxycarbonyl)amino)-1,4(1,4)-dibenzenacyclohexaphane-1^2-yl)hydrazine-1,2-dicarboxylate **(3w)**

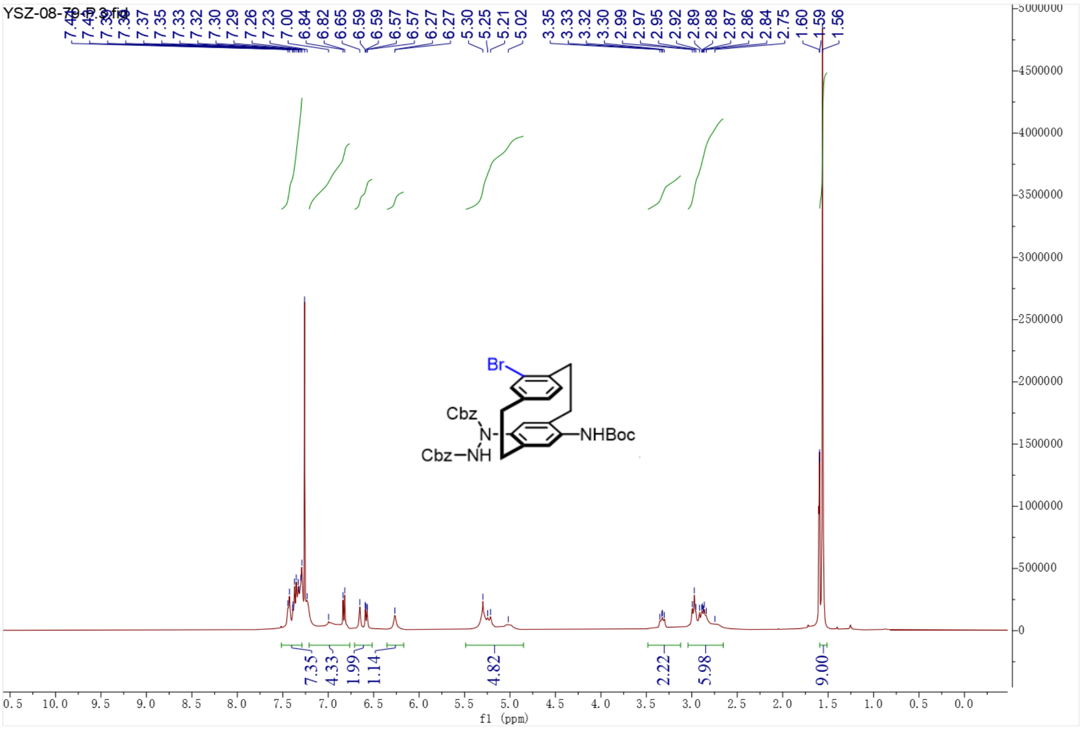

Supplementary Fig. 513 ^{1}H NMR spectrum of (S_p)-**3w** (400 MHz, $CDCl_3$)

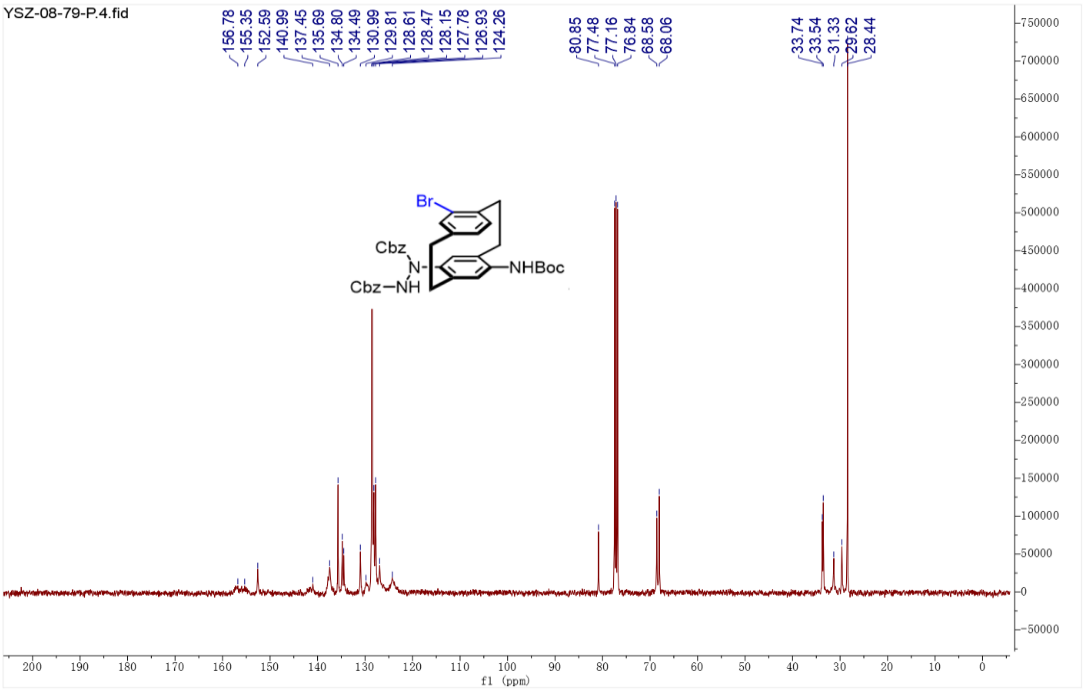

Supplementary Fig. 514 ^{13}C NMR spectrum of (S_p)-**3w** (101 MHz, $CDCl_3$)

(R_p)-Tert-butyl (4[2]-phenyl-1,4(1,4)-dibenzenacyclohexaphane-1[2]-yl)carbamate **((R_p)-1x)**

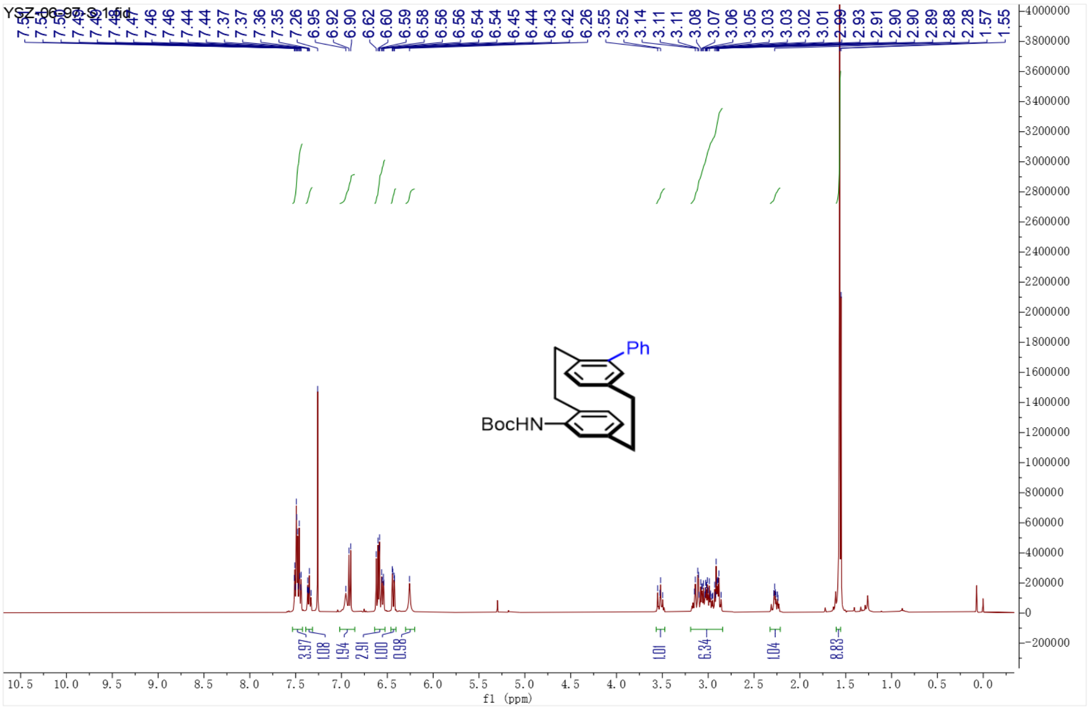

Supplementary Fig. 515 ^{1}H NMR spectrum of (R_p)-**1x** (400 MHz, $CDCl_3$)

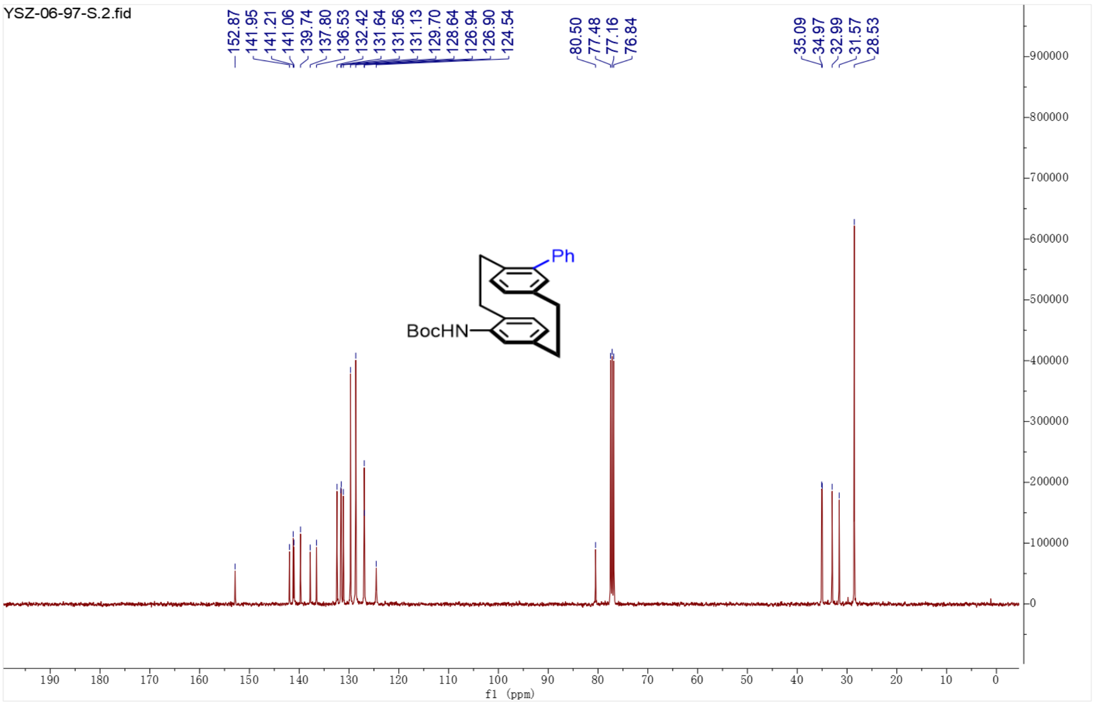

Supplementary Fig. 516 ^{13}C NMR spectrum of (R_p)-**1x** (101 MHz, $CDCl_3$)

(S_p)-Dibenzyl 1-(1[5]-((tert-butoxycarbonyl)amino)-4[3]-phenyl-1,4(1,4)-dibenzenacyclohexaphane-1[2]-yl)hydrazine-1,2-dicarboxylate **(3x)**

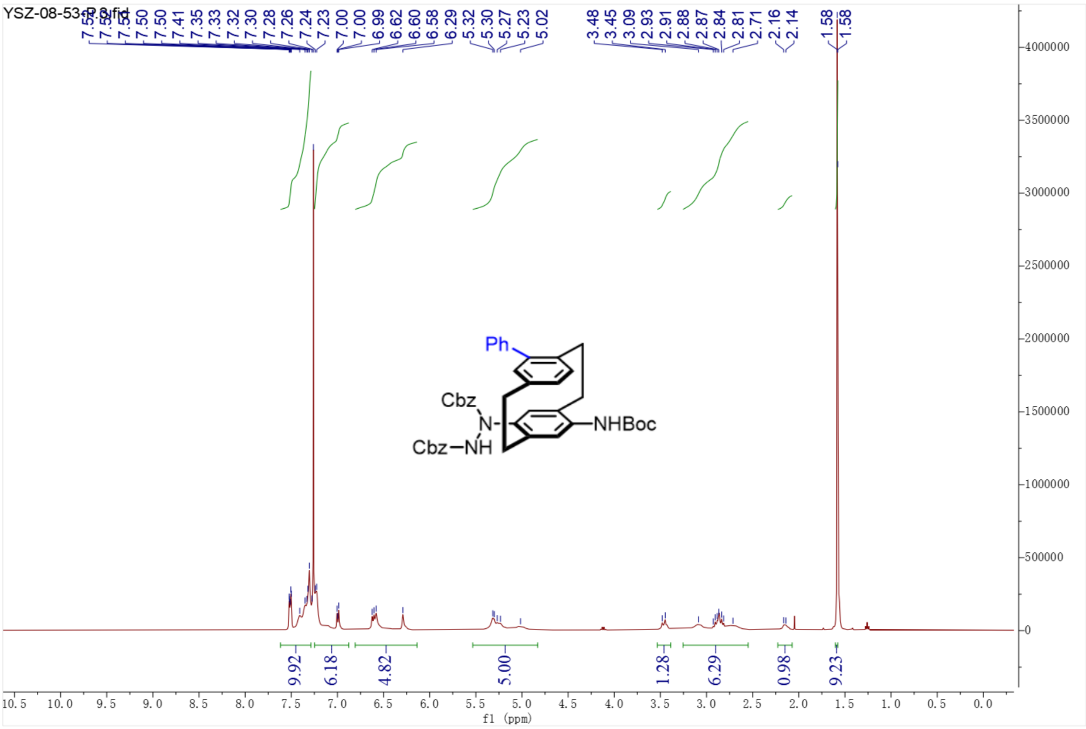

Supplementary Fig. 517 ^{1}H NMR spectrum of (S_p)-**3x** (400 MHz, $CDCl_3$)

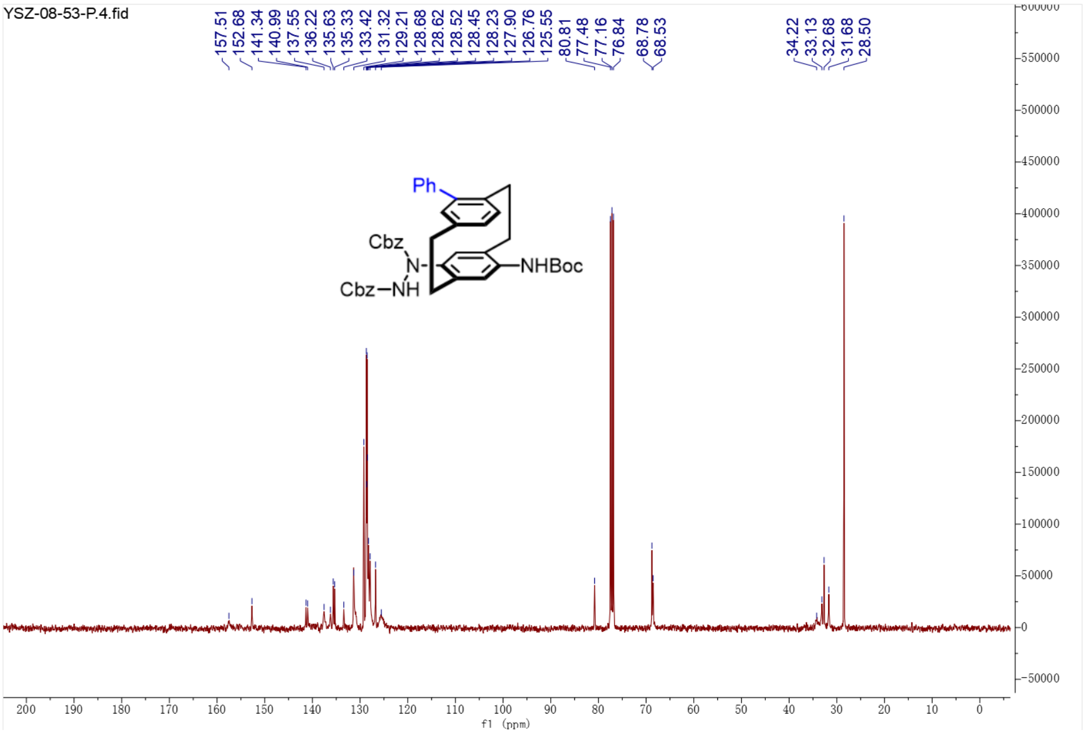

Supplementary Fig. 518 ^{13}C NMR spectrum of (S_p)-**3x** (101 MHz, $CDCl_3$)

(R_p)-Tert-butyl (4^2-(cyclohex-1-en-1-yl)-1,4(1,4)-dibenzenacyclohexaphane -1^2yl)carbamate **((R_p)-1y)**

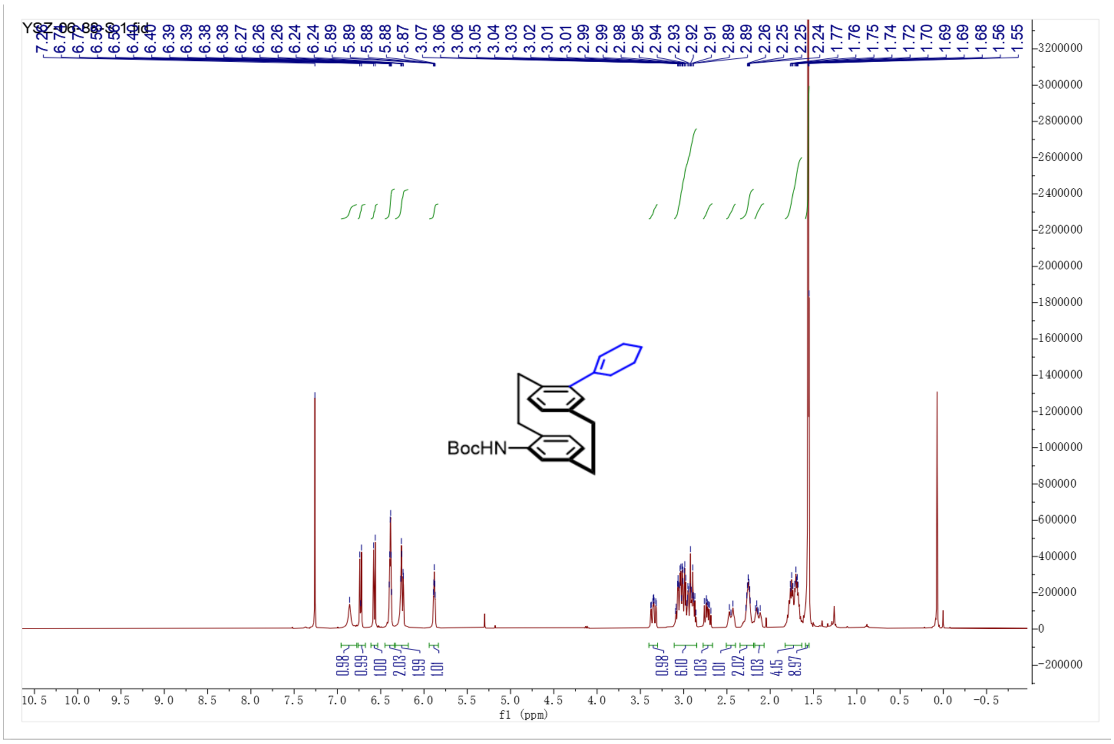

Supplementary Fig. 519 ^{1}H NMR spectrum of (R_p)**-1y** (400 MHz, $CDCl_3$)

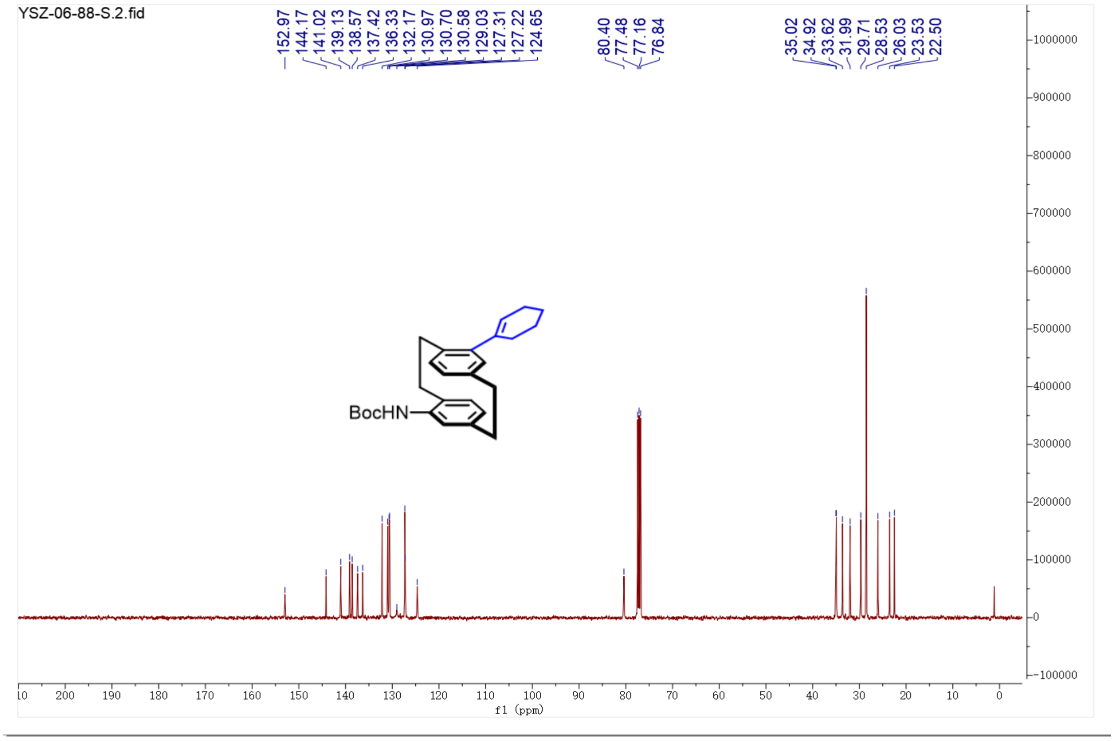

Supplementary Fig. 520 ^{13}C NMR spectrum of (R_p)**-1y** (101 MHz, $CDCl_3$)

(S_p)-Dibenzyl 1-(1[5]-((tert-butoxycarbonyl)amino)-4[3]-(cyclohex-1-en-1-yl)-1,4(1,4)-dibenzenacyclohexaphane-1[2]-yl)hydrazine-1,2-dicarboxylate **(3y)**

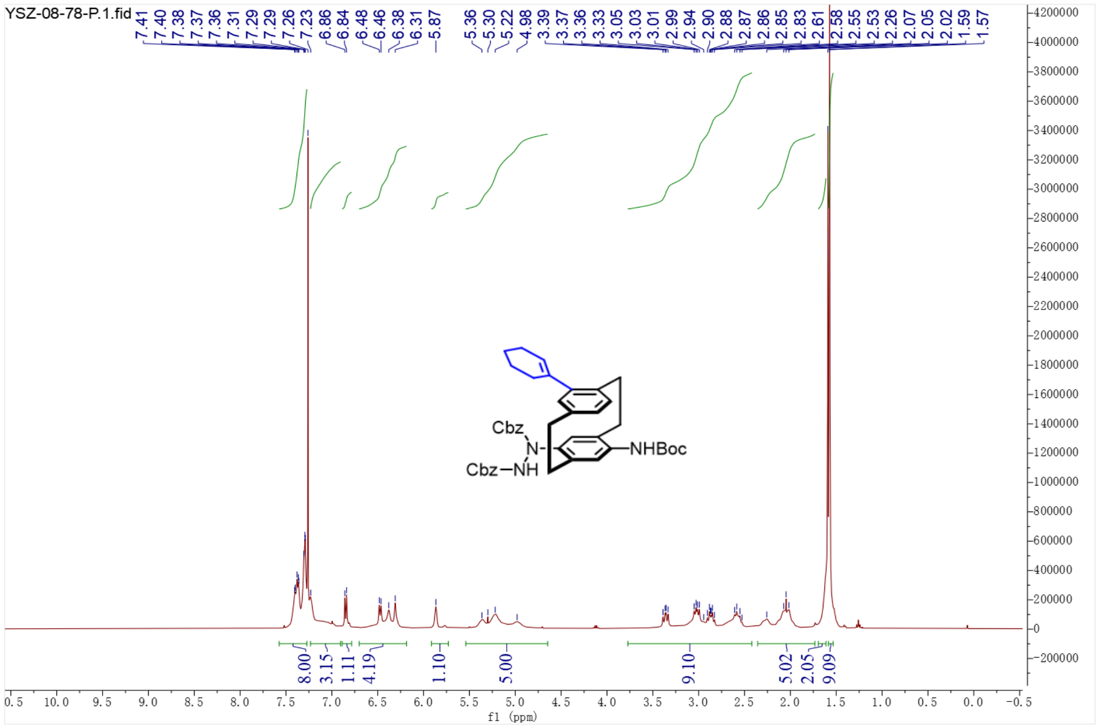

Supplementary Fig. 521 ^{1}H NMR spectrum of (S_p)-**3y** (400 MHz, $CDCl_3$)

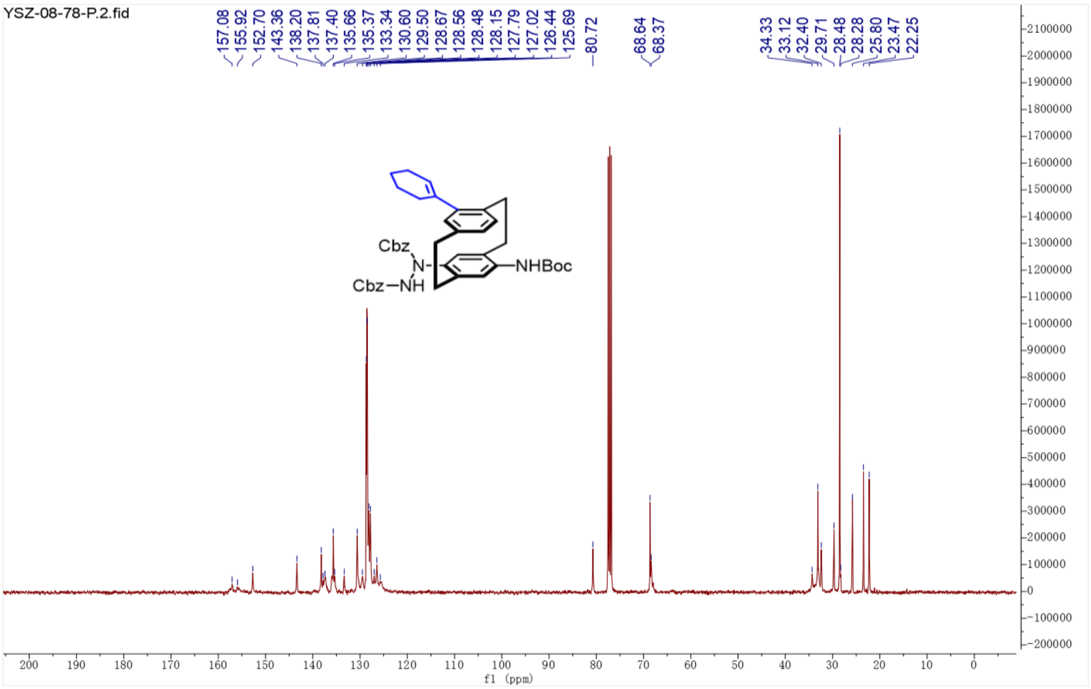

Supplementary Fig. 522 ^{13}C NMR spectrum of (S_p)-**3y** (101 MHz, $CDCl_3$)

(R_p)-Tert-butyl (4[2]-cyclohexyl-1,4(1,4)-dibenzenacyclohexaphane-1[2]-yl)carbamate

((R_p)-1z)

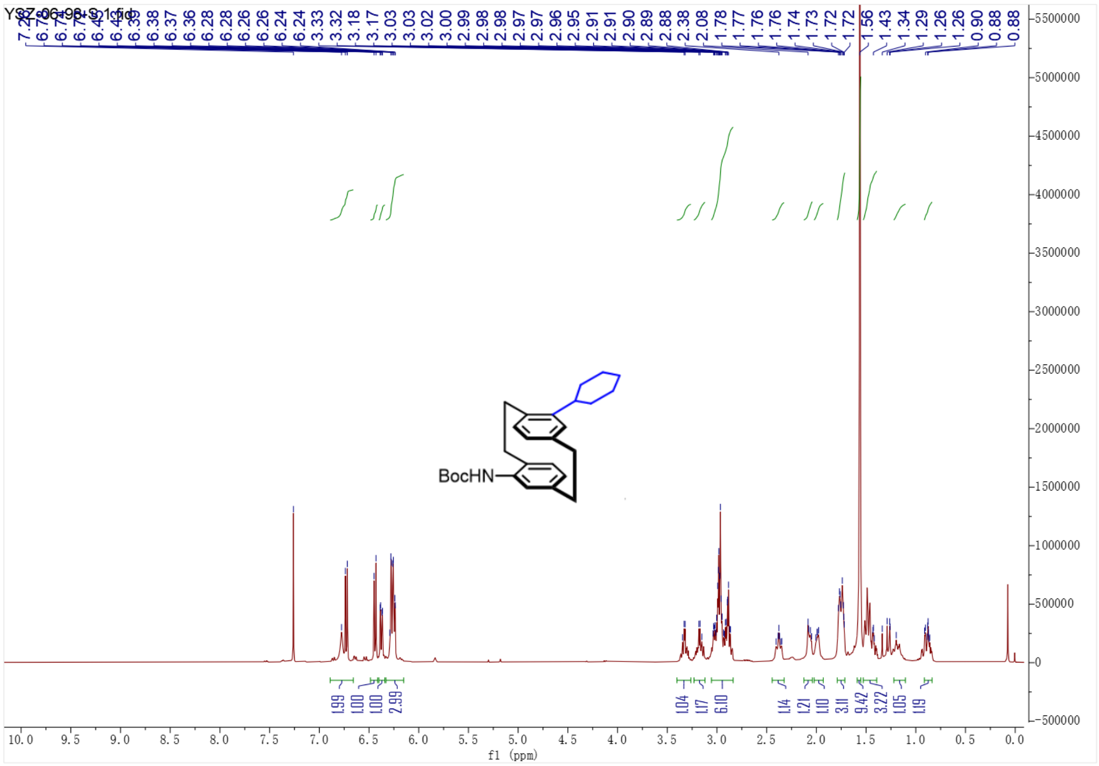

Supplementary Fig. 523 ^{1}H NMR spectrum of (R_p)**-1z** (400 MHz, $CDCl_3$)

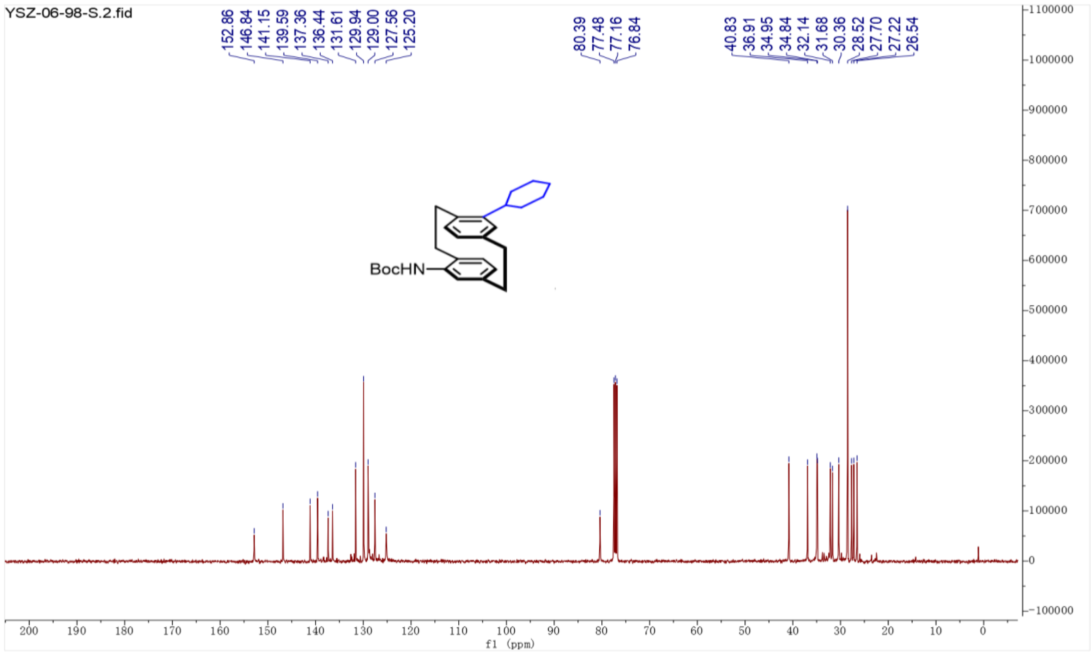

Supplementary Fig. 524 ^{13}C NMR spectrum of (R_p)**-1z** (101 MHz, $CDCl_3$)

(S_p)-Dibenzyl 1-(1^5-((tert-butoxycarbonyl)amino)-4^3-cyclohexyl-1,4(1,4)-dibenzenacyclohexaphane-1^2-yl)hydrazine-1,2-dicarboxylate **(3z)**

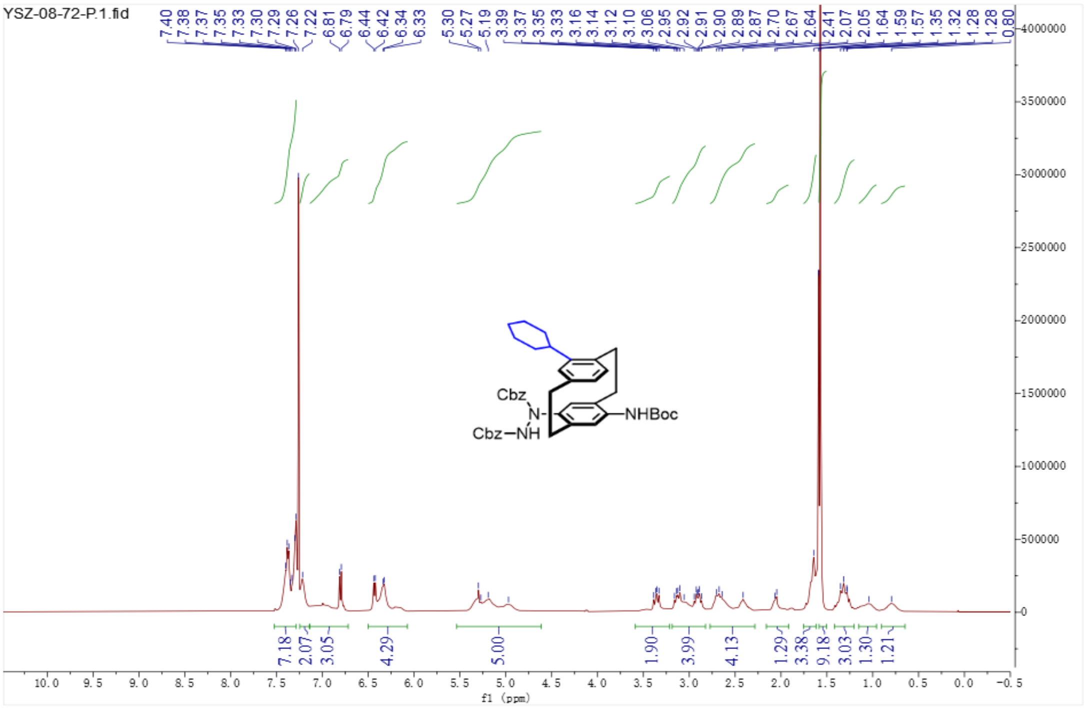

Supplementary Fig. 525 ^{1}H NMR spectrum of (S_p)-**3z** (400 MHz, $CDCl_3$)

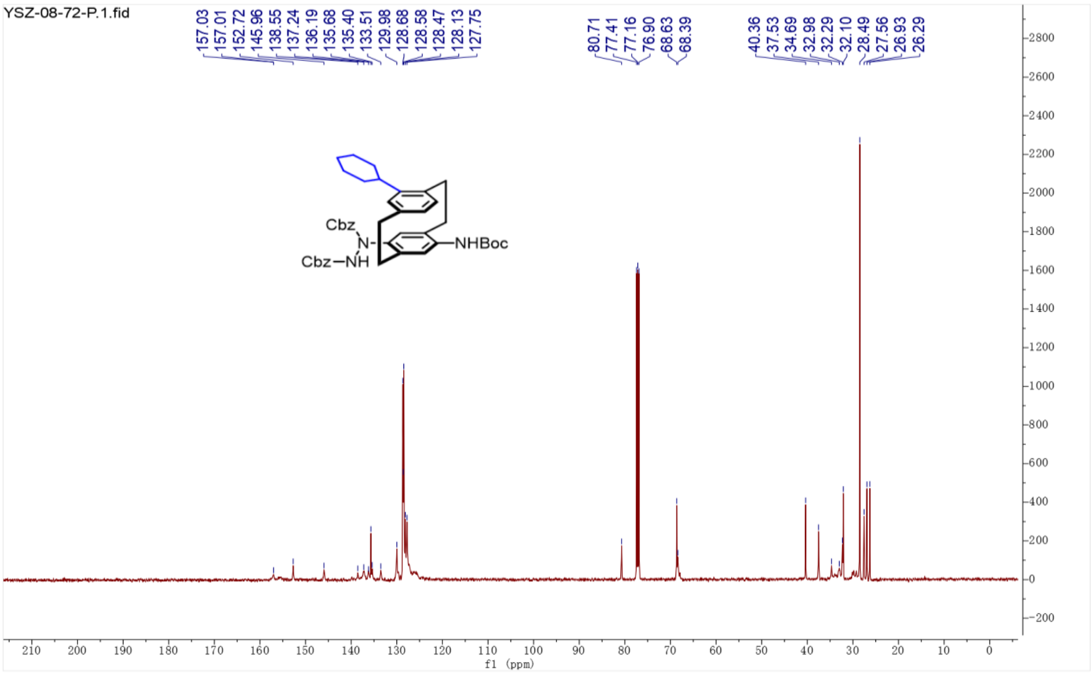

Supplementary Fig. 526 ^{13}C NMR spectrum of (S_p)-**3z** (126 MHz, $CDCl_3$)

(S_p)-Tert-butyl (4[3]-bromo-1,4(1,4)-dibenzenacyclohexaphane-1[2]-yl)carbamate **((S_p)-1aa)**

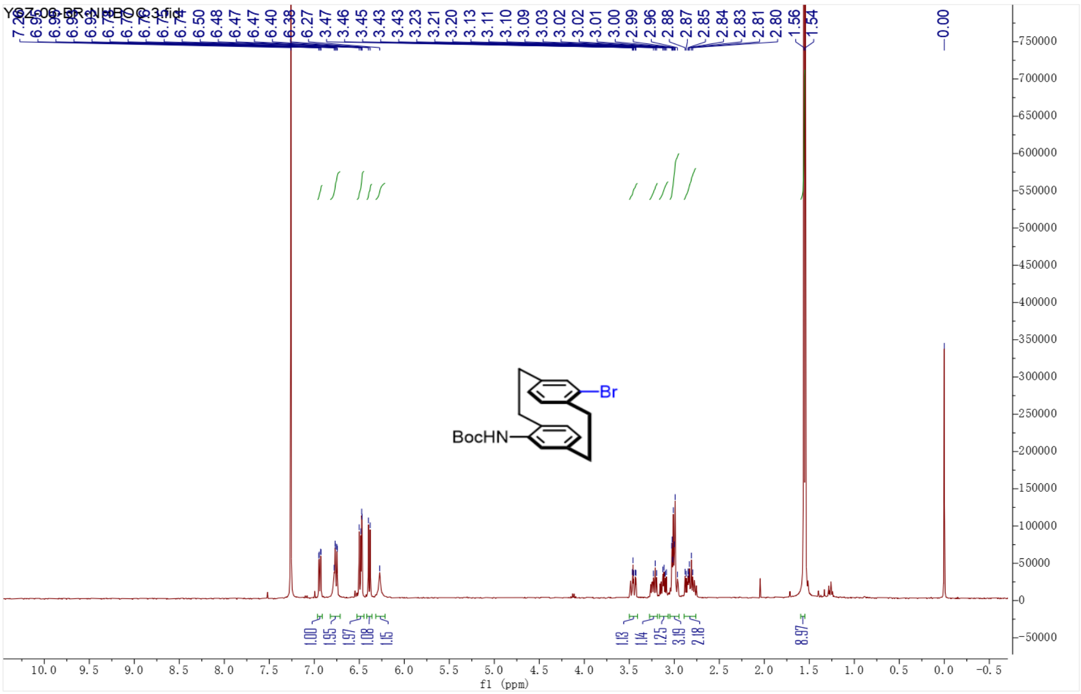

Supplementary Fig. 527 ^{1}H NMR spectrum of (S_p)-**1aa** (400 MHz, $CDCl_3$)

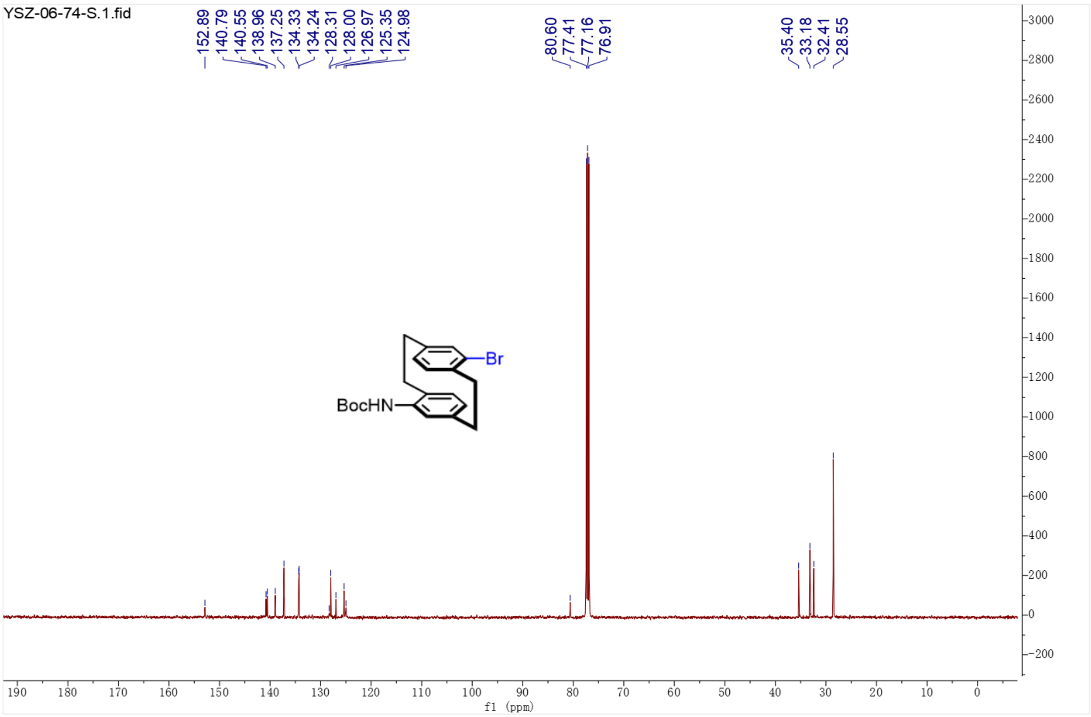

Supplementary Fig. 528 ^{13}C NMR spectrum of (S_p)-**1aa** (126 MHz, $CDCl_3$)

(R_p)-Dibenzyl 1-(4^2-bromo-1^5-((tert-butoxycarbonyl)amino)-1,4(1,4)-dibenzenacyclohexaphane-1^2-yl)hydrazine-1,2-dicarboxylate **(3aa)**

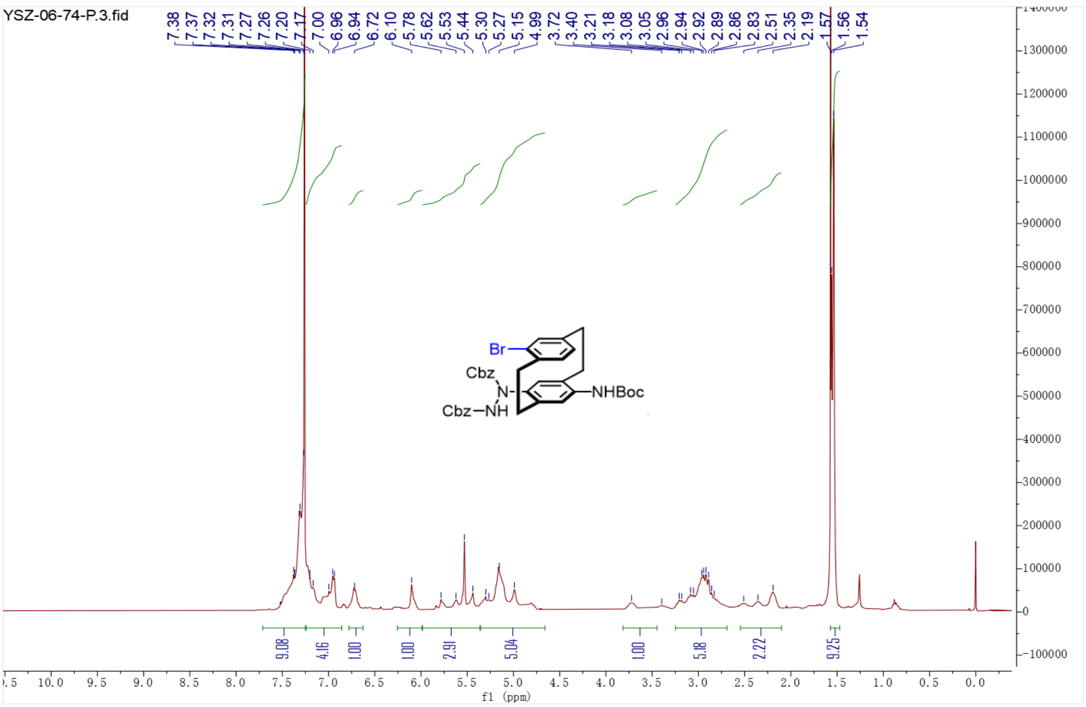

Supplementary Fig. 529 ^{1}H NMR spectrum of (R_p)-**3aa** (400 MHz, $CDCl_3$)

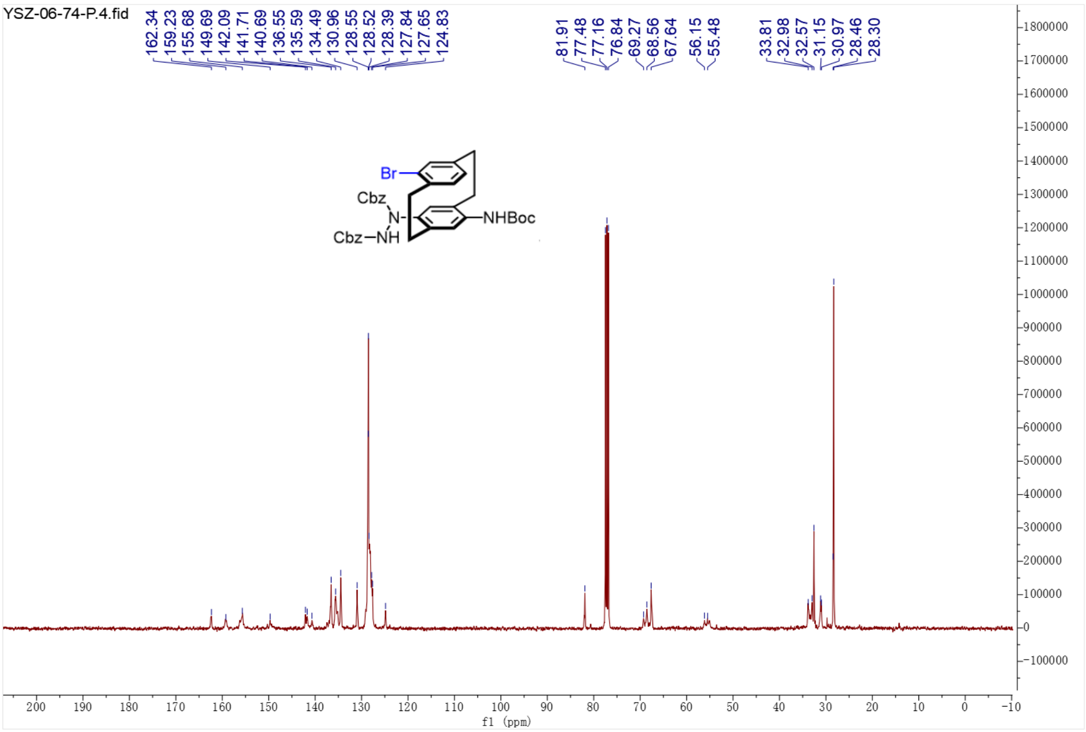

Supplementary Fig. 530 ^{13}C NMR spectrum of (R_p)-**3aa** (101 MHz, $CDCl_3$)

(R_p)-Tert-butyl (4[3]-phenyl-1,4(1,4)-dibenzenacyclohexaphane-1[2]-yl)carbamate **((R_p)-1ab)**

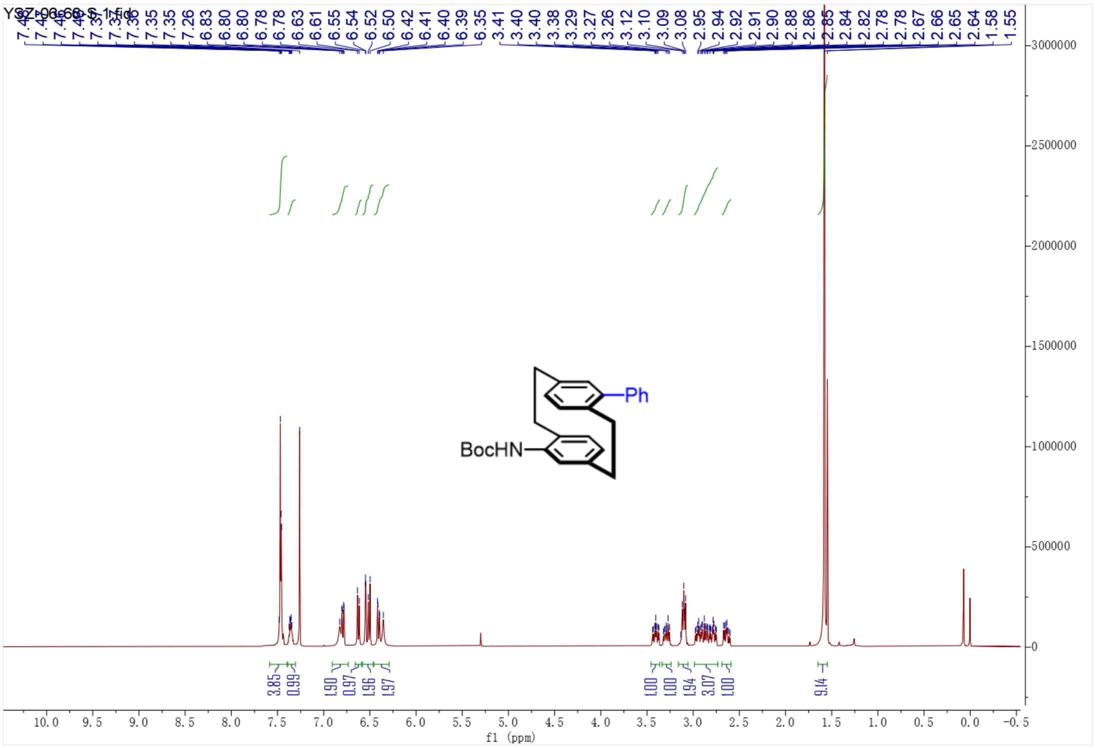

Supplementary Fig. 531 ^{1}H NMR spectrum of (R_p)**-1ab** (400 MHz, $CDCl_3$)

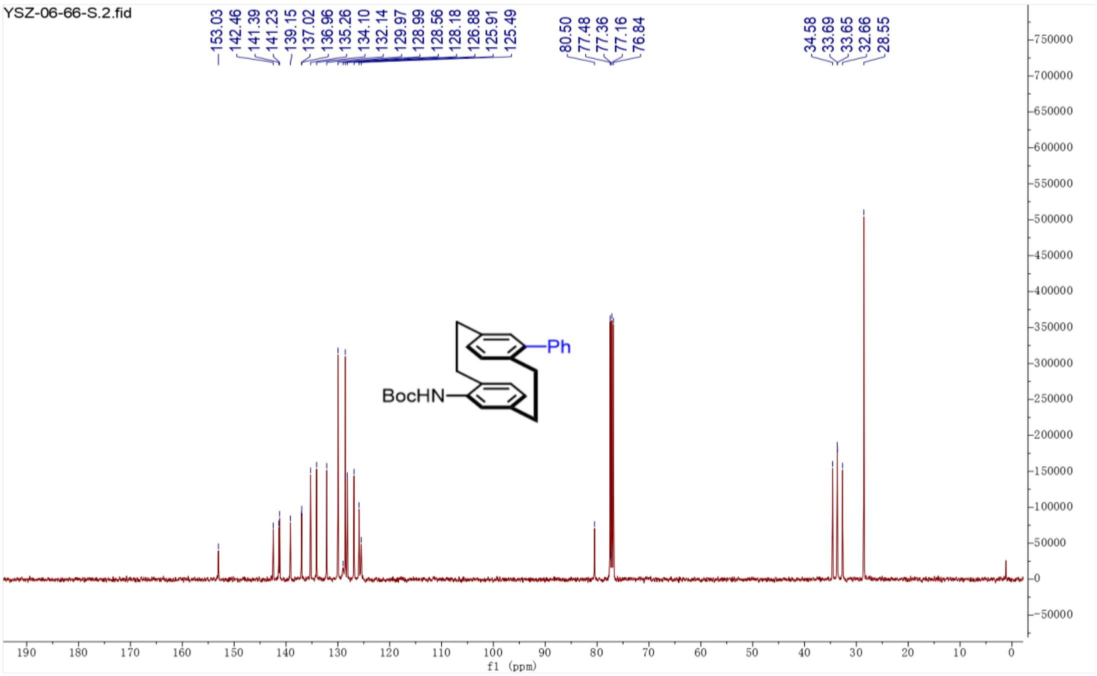

Supplementary Fig. 532 ^{13}C NMR spectrum of (R_p)**-1ab** (101 MHz, $CDCl_3$)

(S_p)-Dibenzyl 1-(1[5]-((tert-butoxycarbonyl)amino)-4[2]-phenyl-1,4(1,4)-dibenzenacyclohexaphane-1[2]-yl)hydrazine-1,2-dicarboxylate **(3ab)**

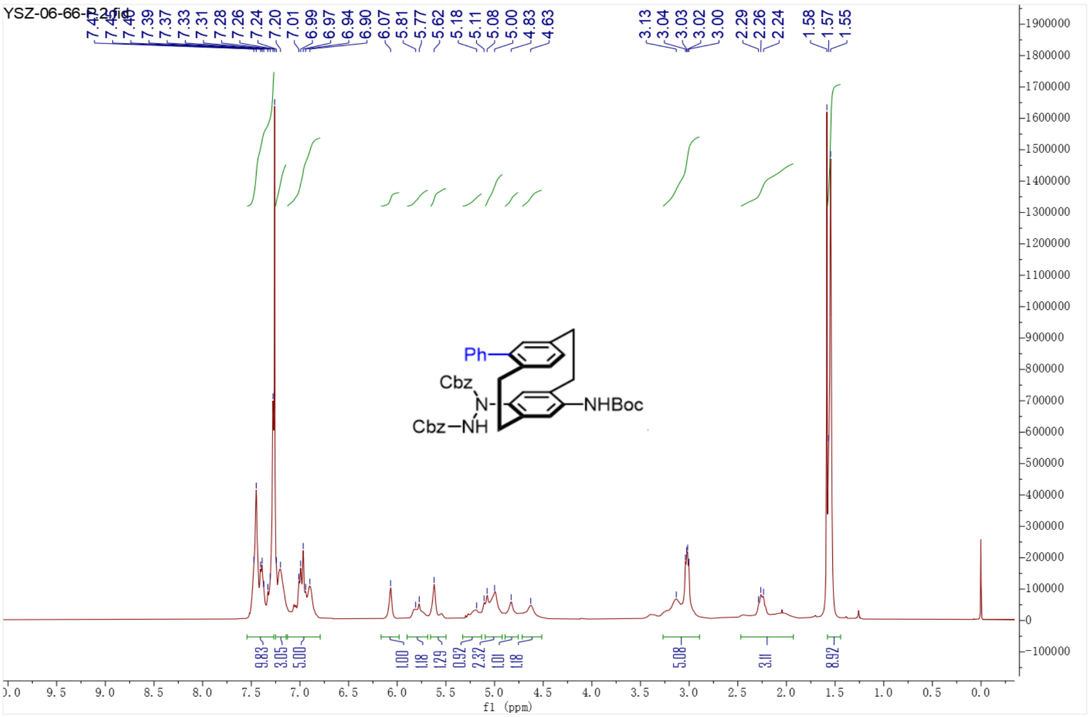

Supplementary Fig. 533 ^{1}H NMR spectrum of (S_p)-**3ab** (400 MHz, $CDCl_3$)

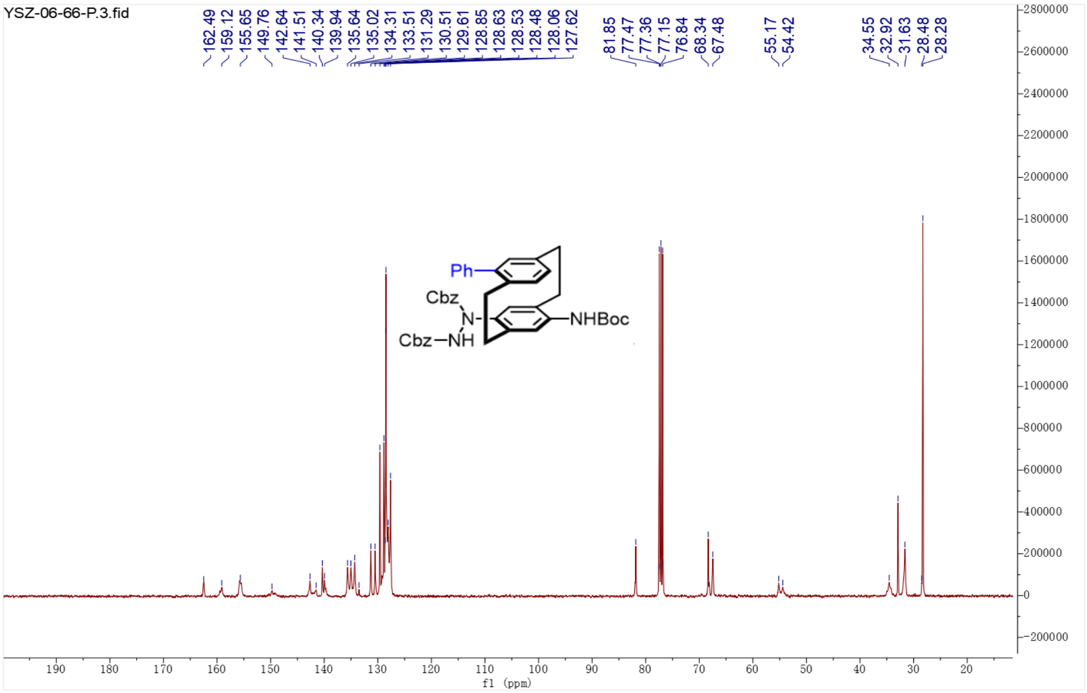

Supplementary Fig. 534 ^{13}C NMR spectrum of (S_p)-**3ab** (101 MHz, $CDCl_3$)

(R_p)-Tert-butyl (E)-(4^3-styryl-1,4(1,4)-dibenzenacyclohexaphane-1^2-yl)carbamate ((R_p)-**1ac**)

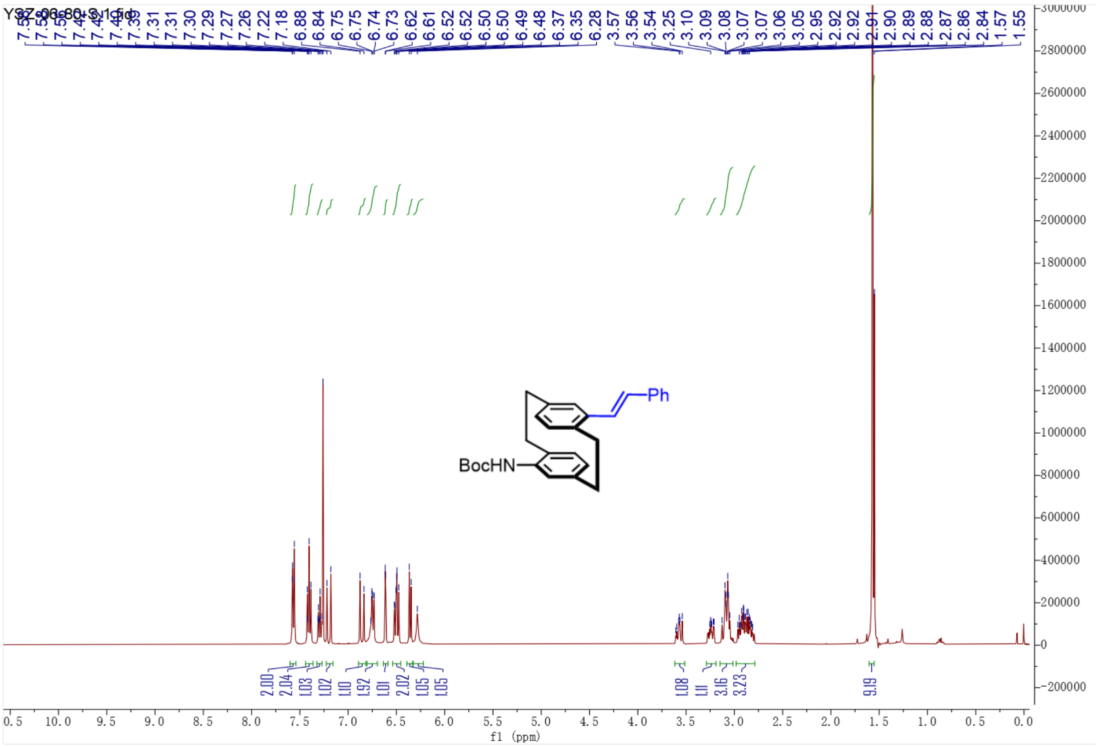

Supplementary Fig. 535 ^{1}H NMR spectrum of (R_p)-**3ac** (400 MHz, $CDCl_3$)

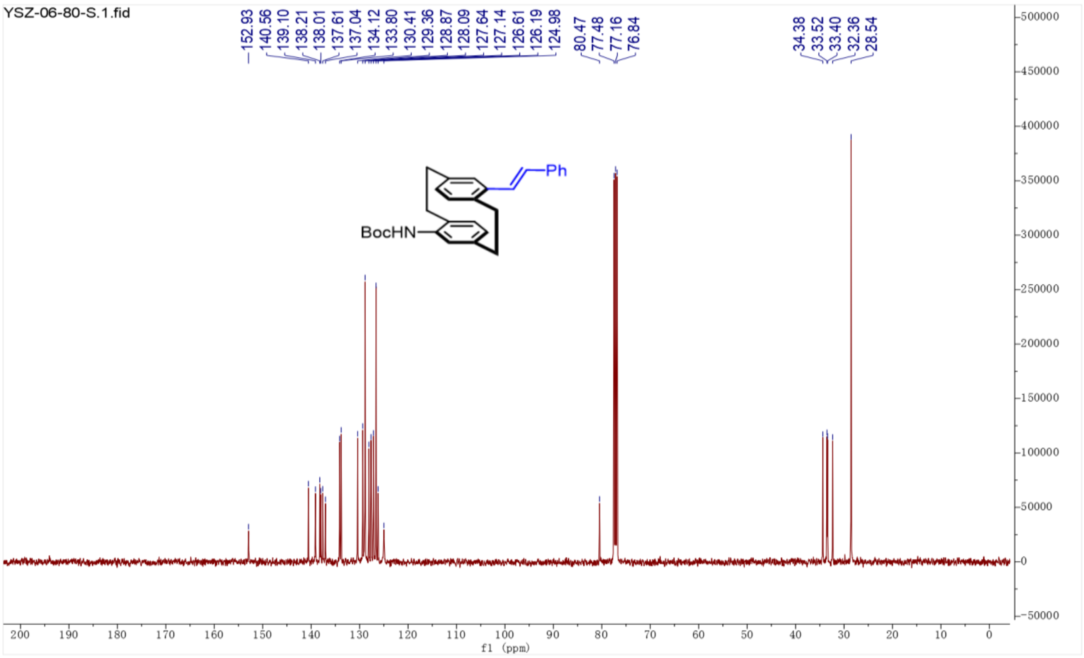

Supplementary Fig. 536 ^{13}C NMR spectrum of (R_p)-**3ac** (101 MHz, $CDCl_3$)

(S_p)-Dibenzyl (E)-1-(1^5-((tert-butoxycarbonyl)amino)-4^2-styryl-1,4(1,4)-dibenzenacyclohexaphane-1^2-yl)hydrazine-1,2-dicarboxylate **(3ac)**

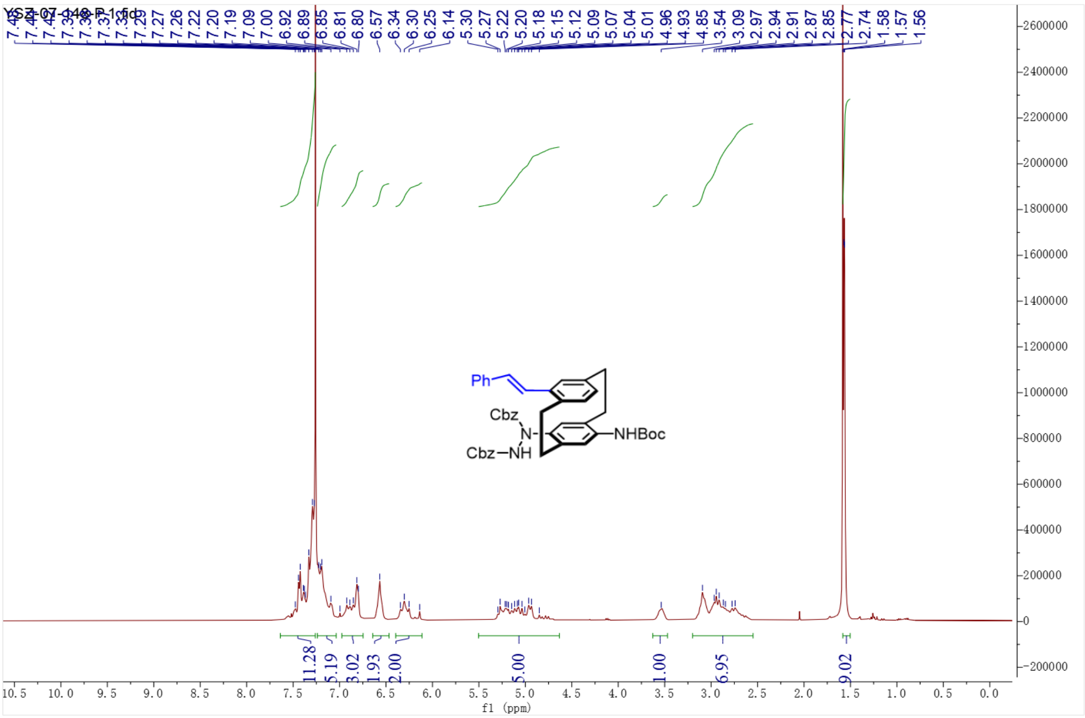

Supplementary Fig. 537 ^{1}H NMR spectrum of (S_p)-**3ac** (400 MHz, $CDCl_3$)

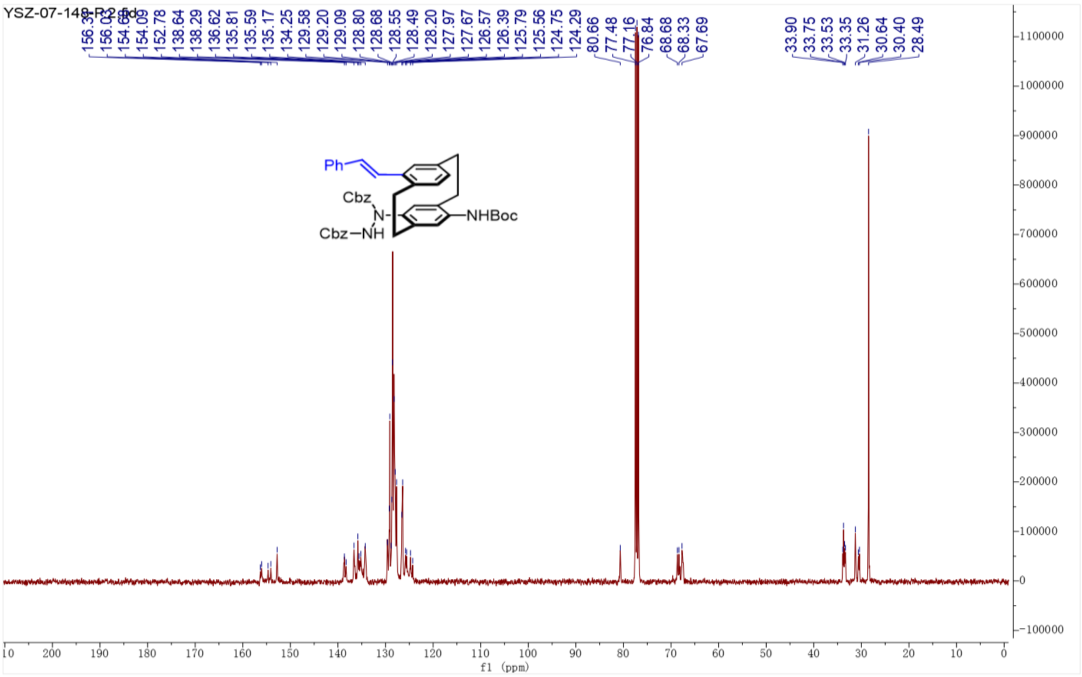

Supplementary Fig. 538 ^{13}C NMR spectrum of (S_p)-**3ac** (101 MHz, $CDCl_3$)

(R_p)-Tert-butyl (4[3]-cyclohexyl-1,4(1,4)-dibenzenacyclohexaphane-1[2]-yl)carbamate (**(R_p)-1ad**)

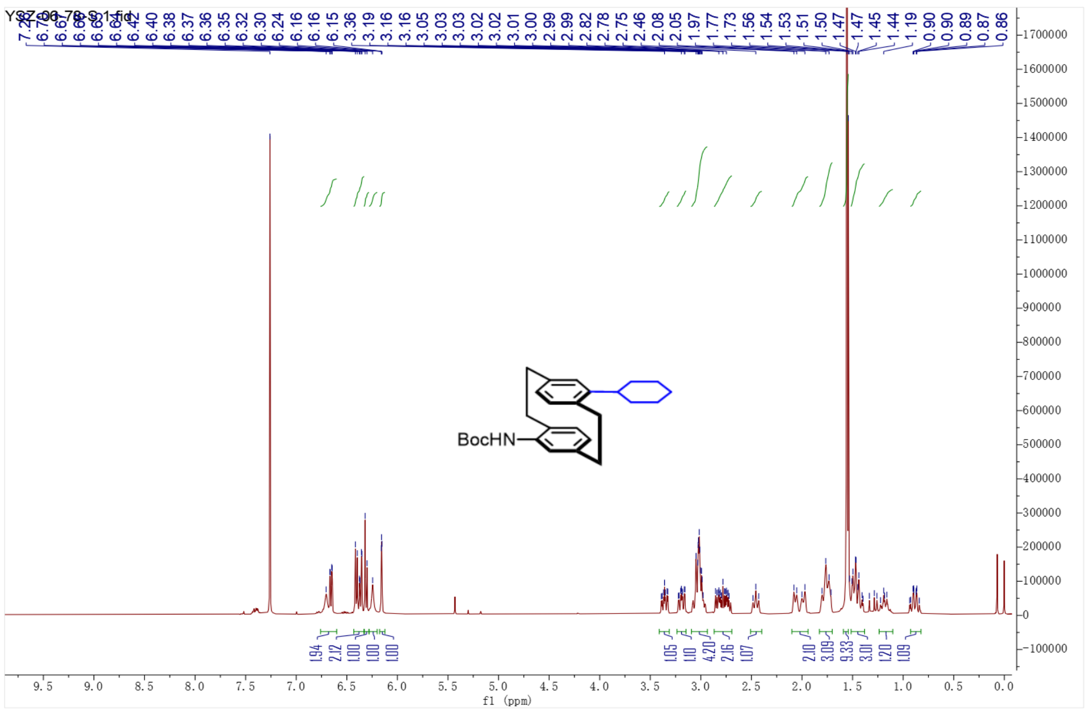

Supplementary Fig. 539 ^{1}H NMR spectrum of (R_p)-**1ad** (400 MHz, $CDCl_3$)

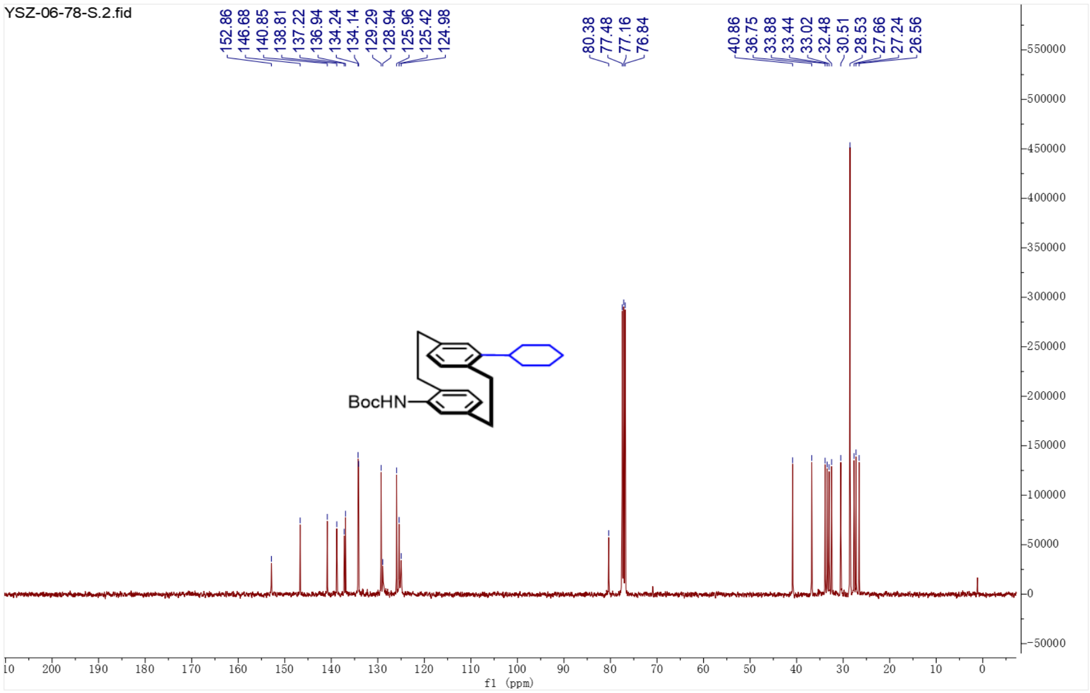

Supplementary Fig. 540 ^{13}C NMR spectrum of (R_p)-**1ad** (101 MHz, $CDCl_3$)

(S_p)-Dibenzyl 1-(1^5-((tert-butoxycarbonyl)amino)-4^2-cyclohexyl-1,4(1,4)-dibenzenacyclohexaphane-1^2-yl)hydrazine-1,2-dicarboxylate **(3ad)**

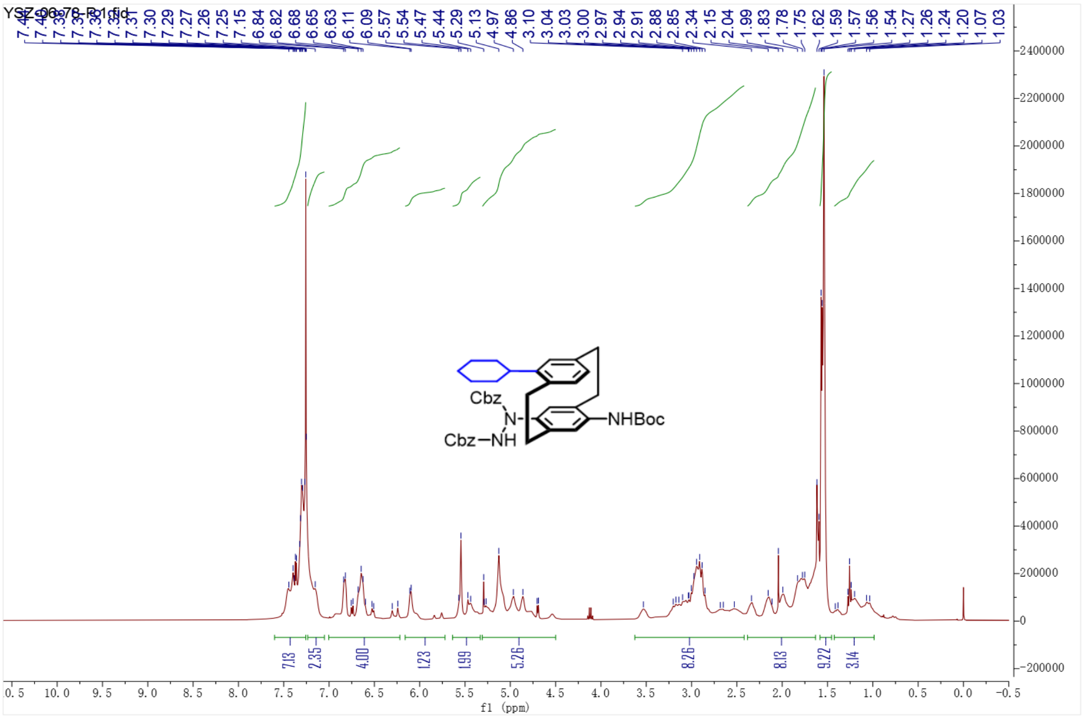

Supplementary Fig. 541 ^{1}H NMR spectrum of (S_p)-**3ad** (400 MHz, $CDCl_3$)

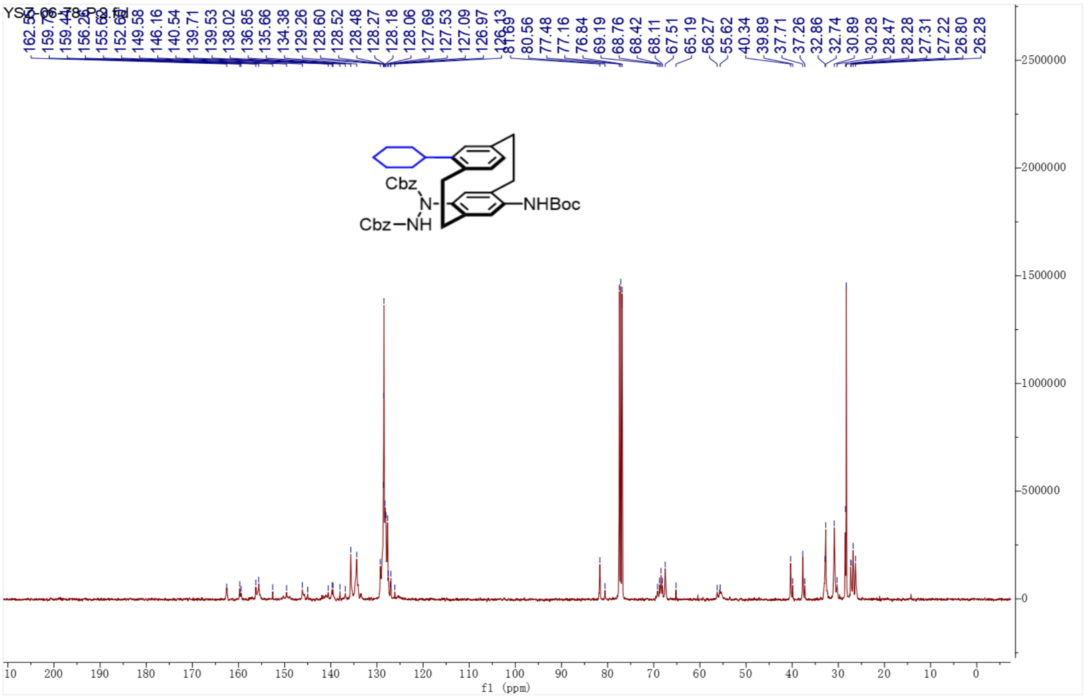

Supplementary Fig. 542 ^{13}C NMR spectrum of (S_p)-**3ad** (101 MHz, $CDCl_3$)

Di-tert-butyl 1,4(1,4)-dibenzenacyclohexaphane-1²,4³-diyldicarbamate **(4a)**

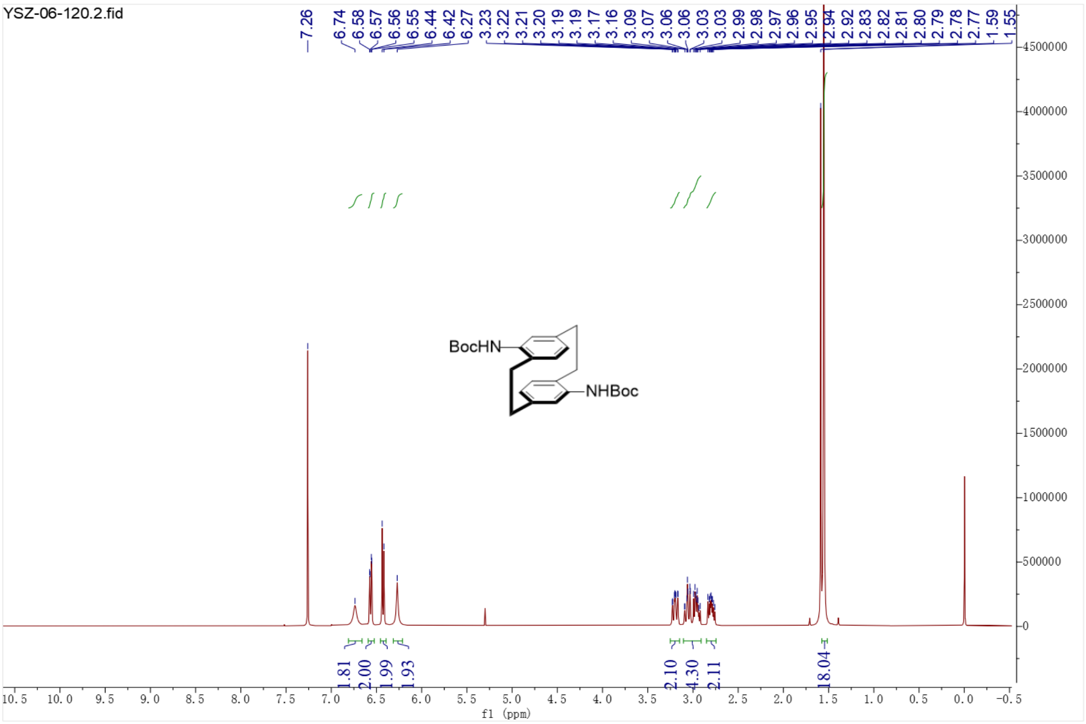

Supplementary Fig. 543 ^{1}H NMR spectrum of **4a** (400 MHz, $CDCl_3$)

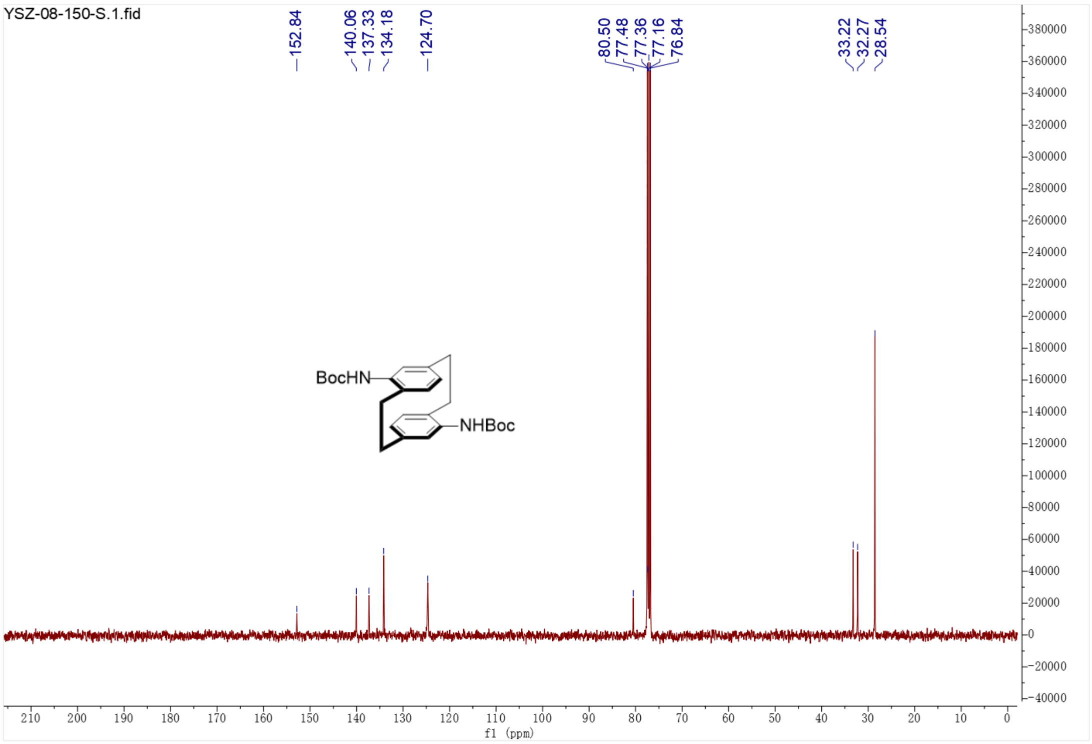

Supplementary Fig. 544 ^{13}C NMR spectrum of **4a** (101 MHz, $CDCl_3$)

(S_p)-Dibenzyl-1-(1^5,4^2-bis((tert-butoxycarbonyl)amino)-1,4(1,4)-dibenzenacyclohexaphane-1^2-yl)hydrazine-1,2-dicarboxylate **(5a)**

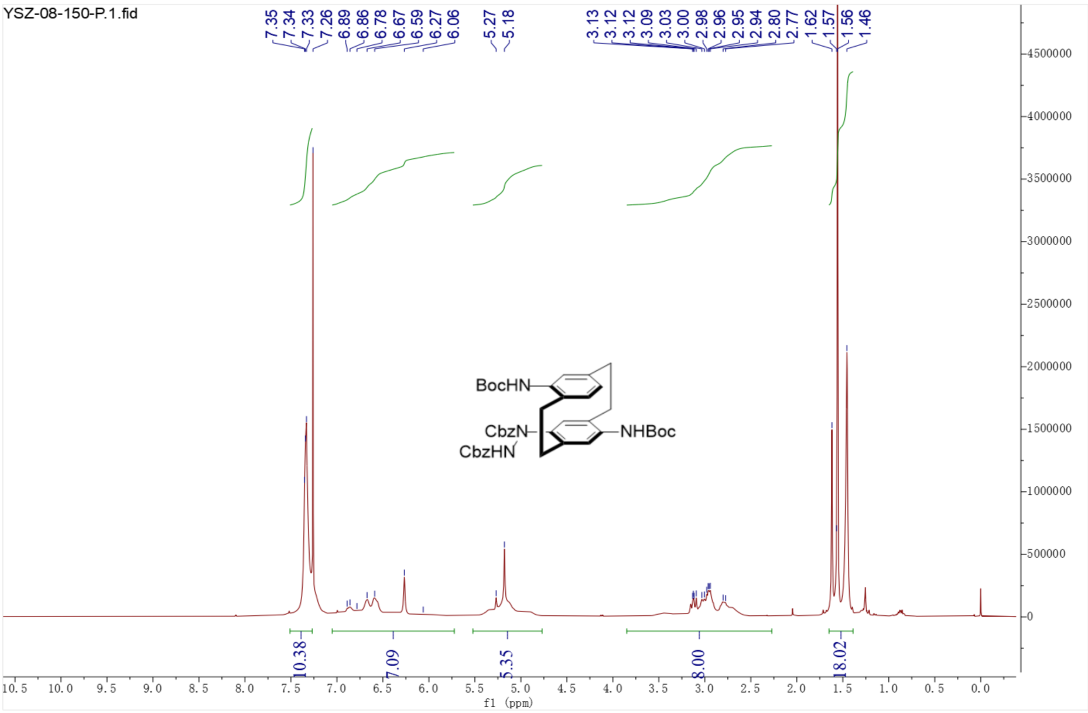

Supplementary Fig. 545 ^{1}H NMR spectrum of (S_p)-**5a** (400 MHz, $CDCl_3$)

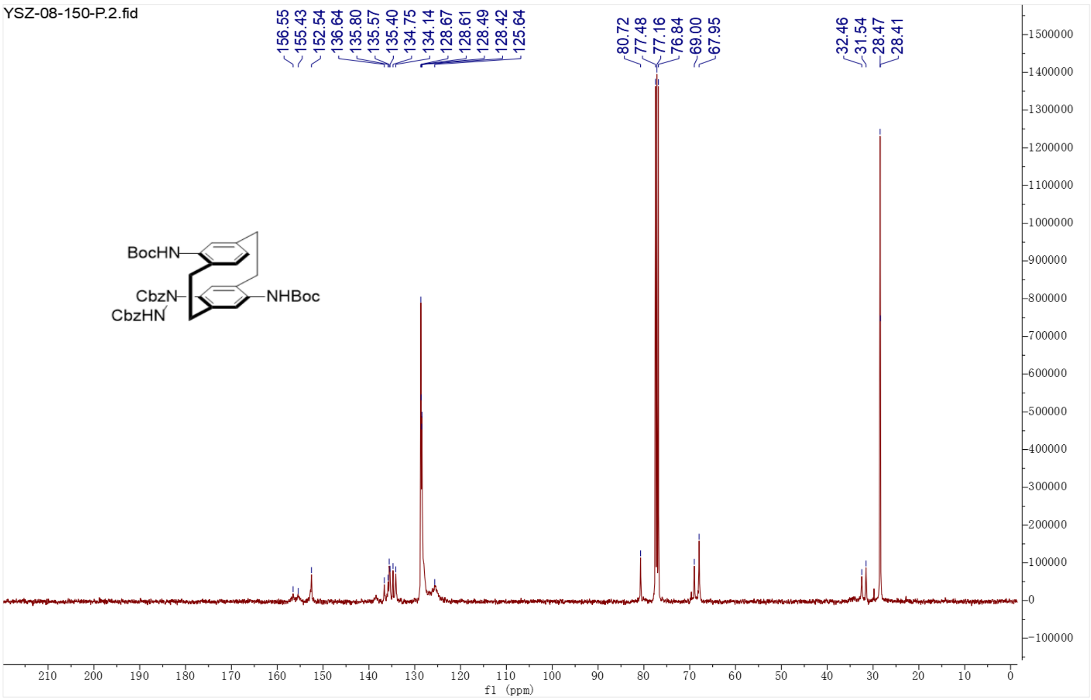

Supplementary Fig. 546 ^{13}C NMR spectrum of (S_p)-**5a** (101 MHz, $CDCl_3$)

Tert-butyl 1,4(1,4)-dibenzenacyclohexaphane-1^2-yl(methyl)carbamate **(6a)**

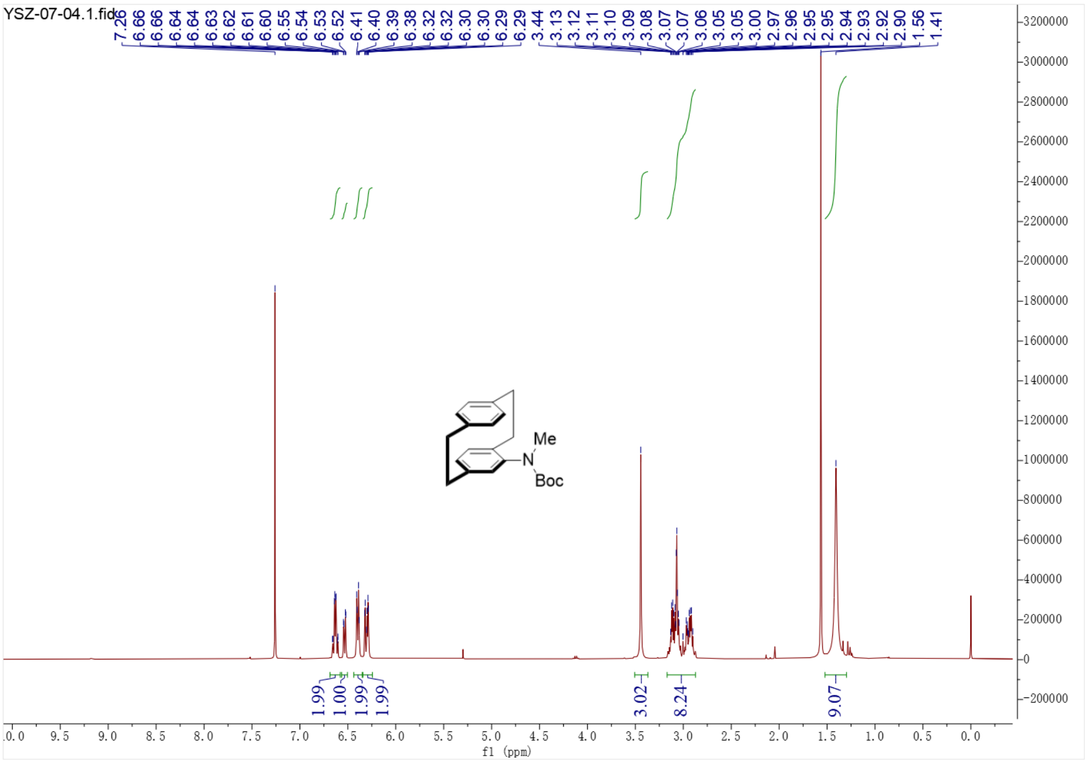

Supplementary Fig. 547 ^{1}H NMR spectrum of **6a** (400 MHz, $CDCl_3$)

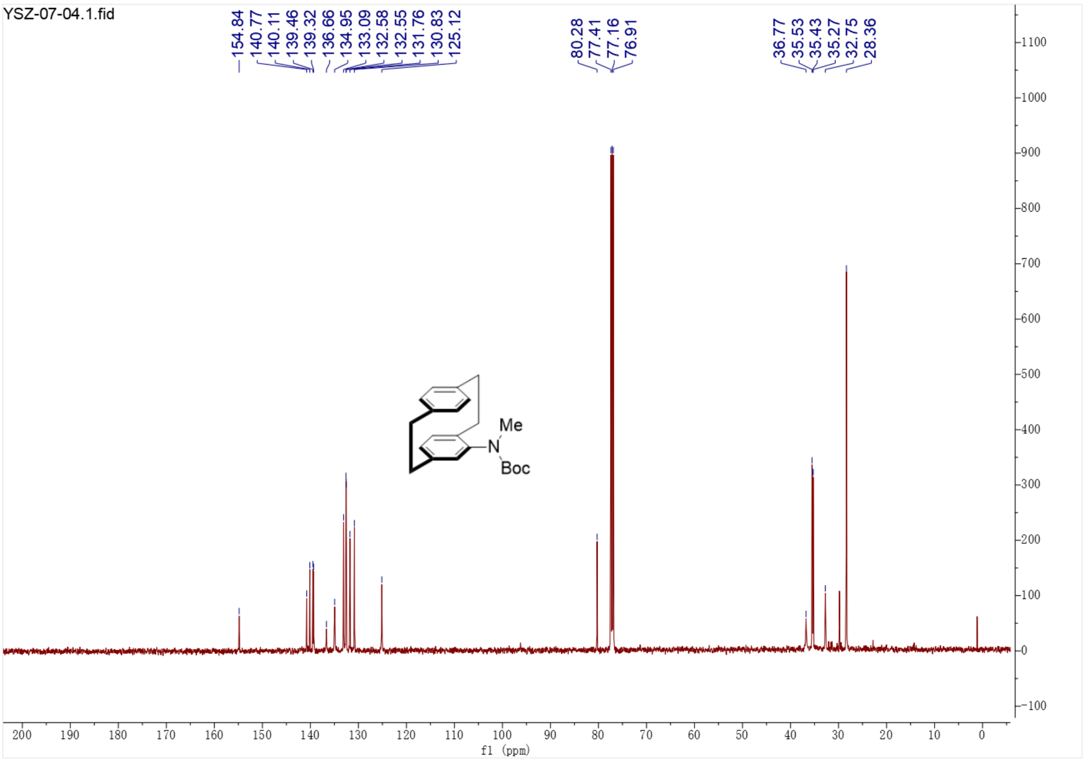

Supplementary Fig. 548 ^{13}C NMR spectrum of **6a** (126 MHz, $CDCl_3$)

Tert-butyl (2,5-dimethylphenyl)carbamate **(6b)**

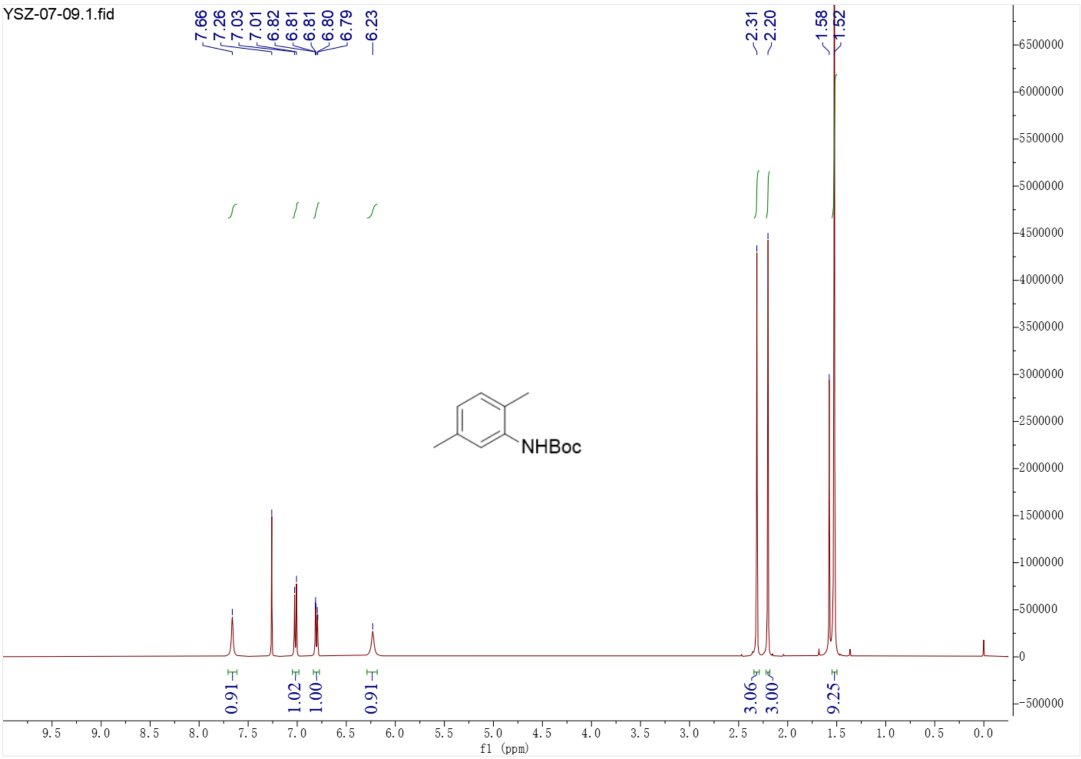

Supplementary Fig. 549 ^{1}H NMR spectrum of **6b** (400 MHz, $CDCl_3$)

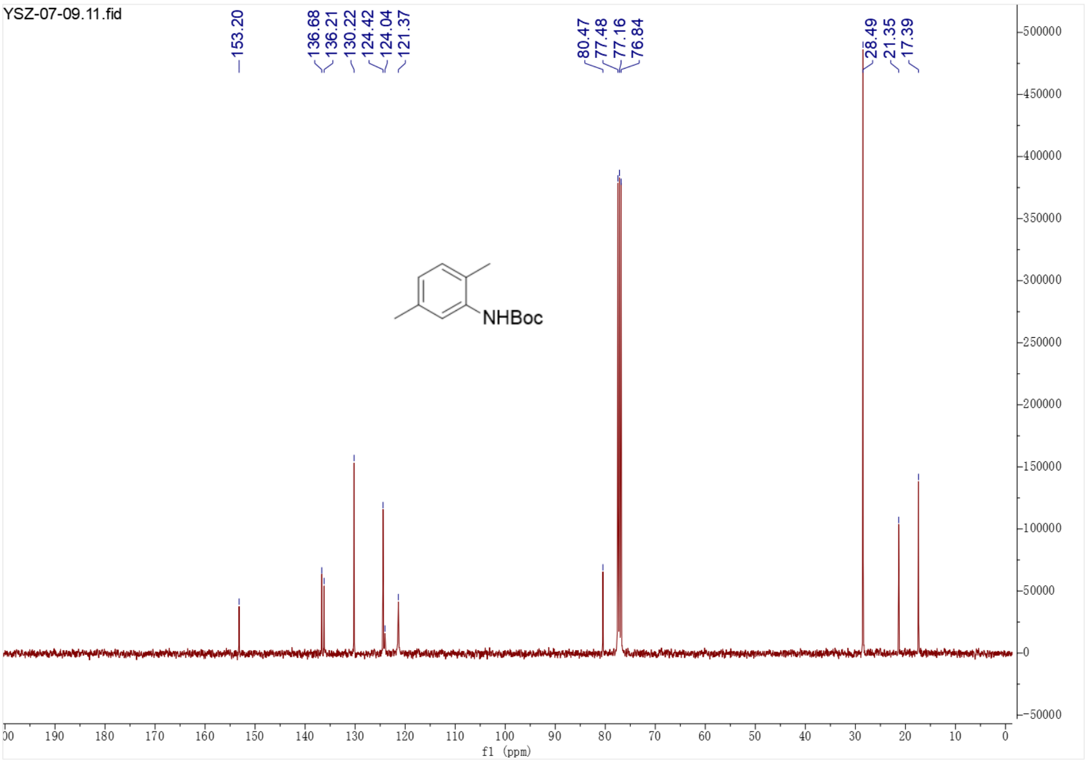

Supplementary Fig. 550 ^{13}C NMR spectrum of **6b** (101 MHz, $CDCl_3$)

(R_p)-*N*-(1,4(1,4)-dibenzenacyclohexaphane-1^2-yl)acetamide **((R_p)-6c)**

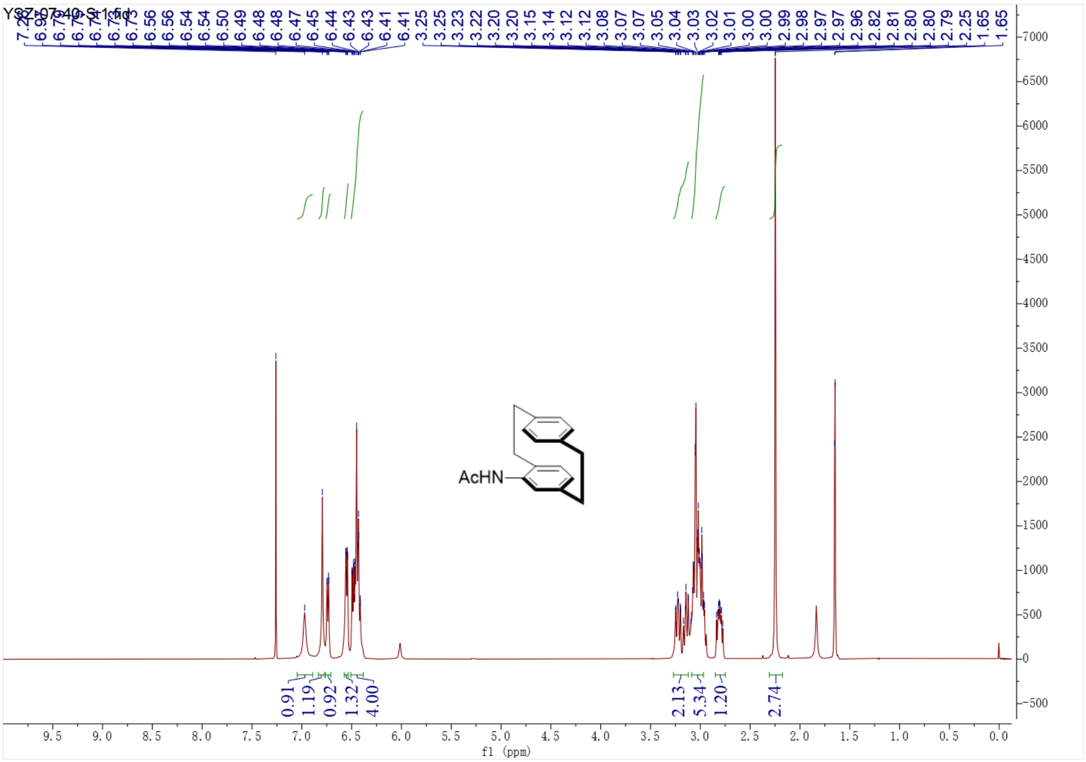

Supplementary Fig. 551 ^{1}H NMR spectrum of (R_p)-**6c** (500 MHz, $CDCl_3$)

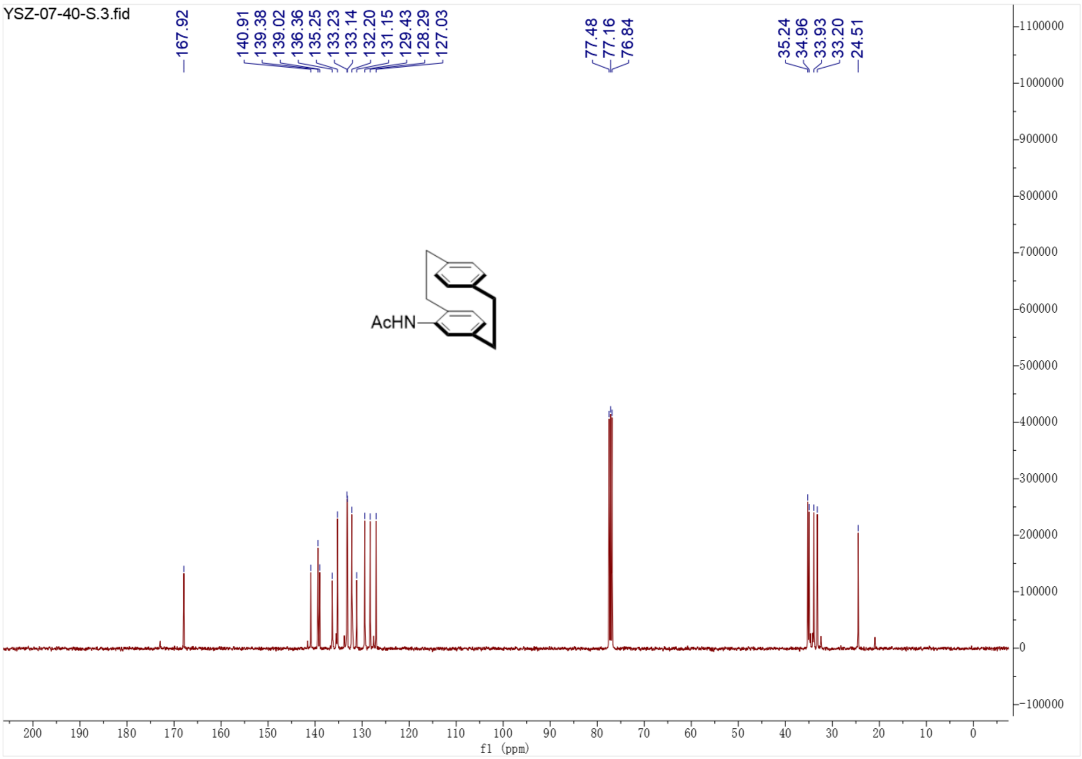

Supplementary Fig. 552 ^{13}C NMR spectrum of (R_p)-**6c** (101 MHz, $CDCl_3$)

(S_p)-Dibenzyl-1-(1^5-acetamido-1,4(1,4)-dibenzenacyclohexaphane-1^2-yl)hydrazine-1,2-dicarboxylate **(7c)**

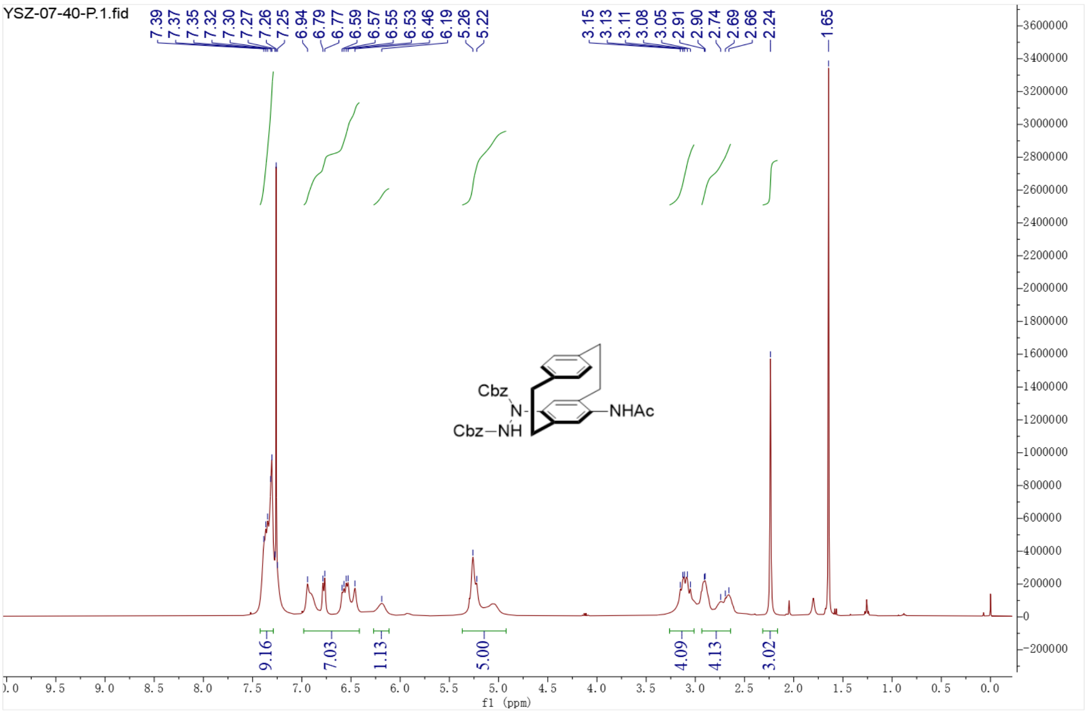

Supplementary Fig. 553 ^{1}H NMR spectrum of (S_p)-**7c** (400 MHz, $CDCl_3$)

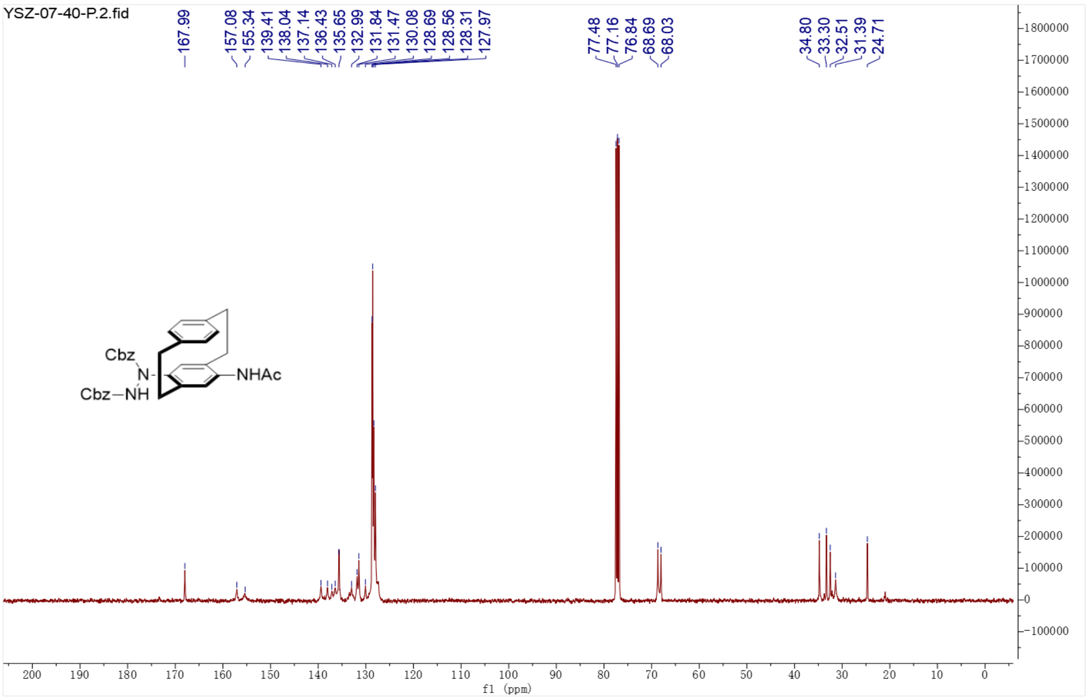

Supplementary Fig. 554 ^{13}C NMR spectrum of (S_p)-**7c** (101 MHz, $CDCl_3$)

(R_p)-*N*-(1,4(1,4)-dibenzenacyclohexaphane-1^2-yl)benzamide **((R_p)-6d)**

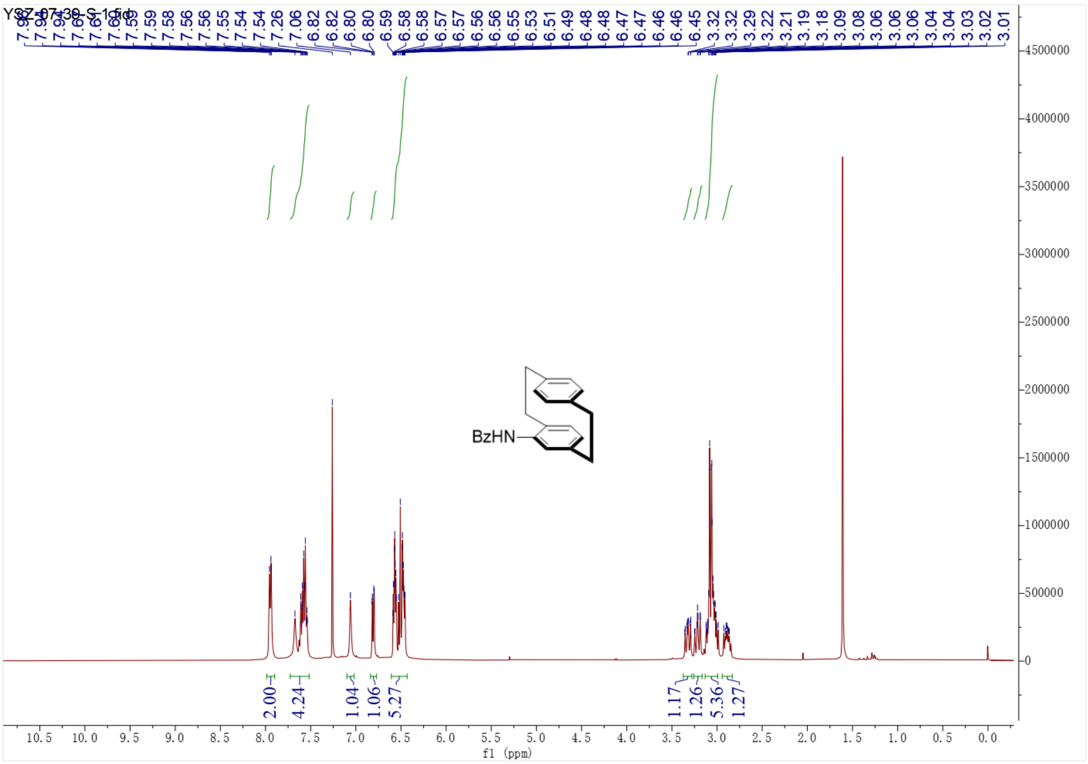

Supplementary Fig. 555 ^{1}H NMR spectrum of (R_p)**-6d** (400 MHz, $CDCl_3$)

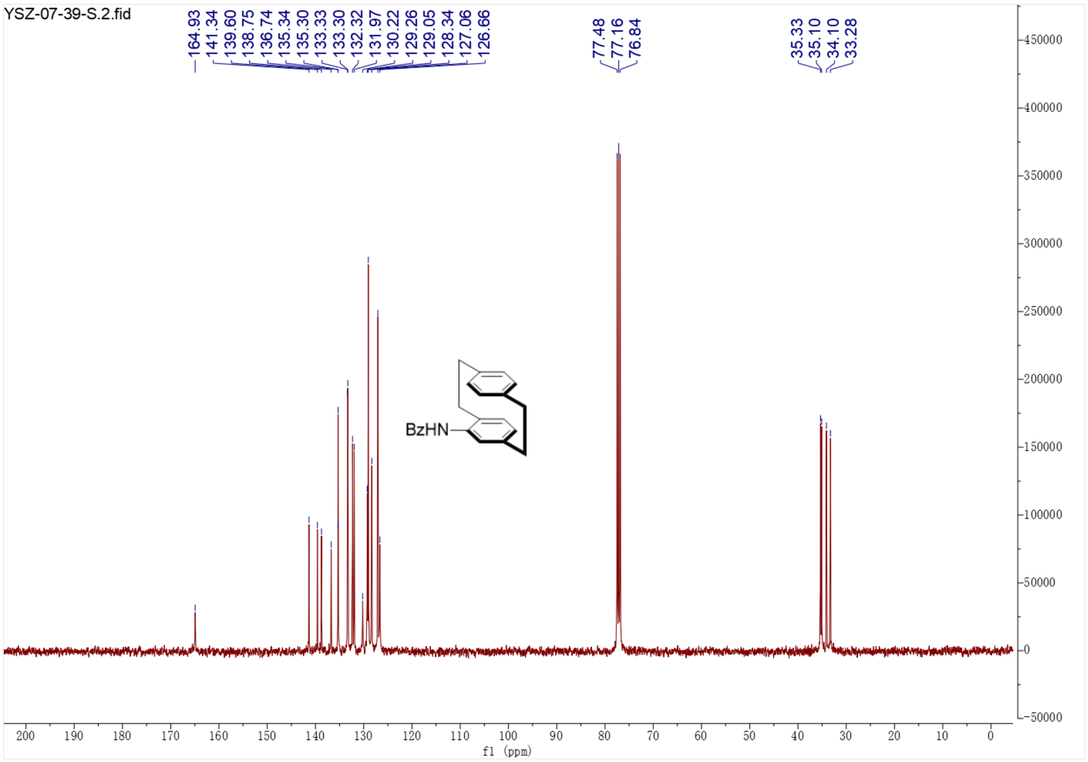

Supplementary Fig. 556 ^{13}C NMR spectrum of (R_p)**-6d** (101 MHz, $CDCl_3$)

(S_p)-Dibenzyl-1-(1^5-benzamido-1,4(1,4)-dibenzenacyclohexaphane-1^2-yl)hydrazine-1,2-dicarboxylate **(7d)**

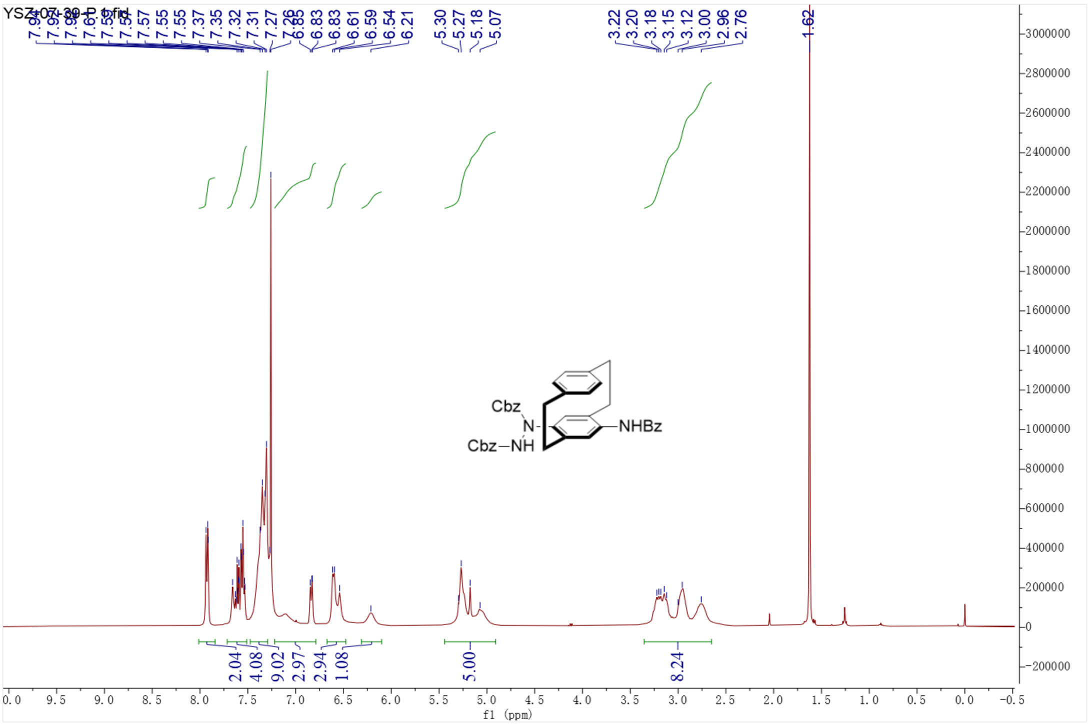

Supplementary Fig. 557 ^{1}H NMR spectrum of (S_p)-**7d** (400 MHz, $CDCl_3$)

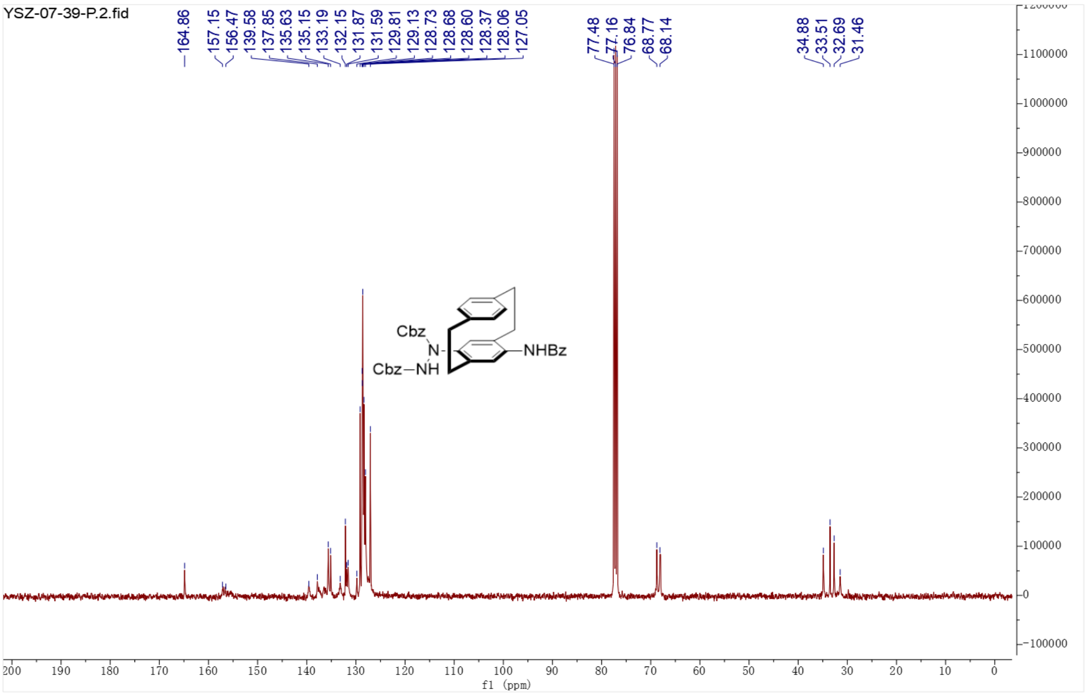

Supplementary Fig. 558 ^{13}C NMR spectrum of (S_p)-**7d** (101 MHz, $CDCl_3$)

(R_p)-*N*-(1,4(1,4)-dibenzenacyclohexaphane-1^2-yl)pivalamide **((R_p)-6e)**

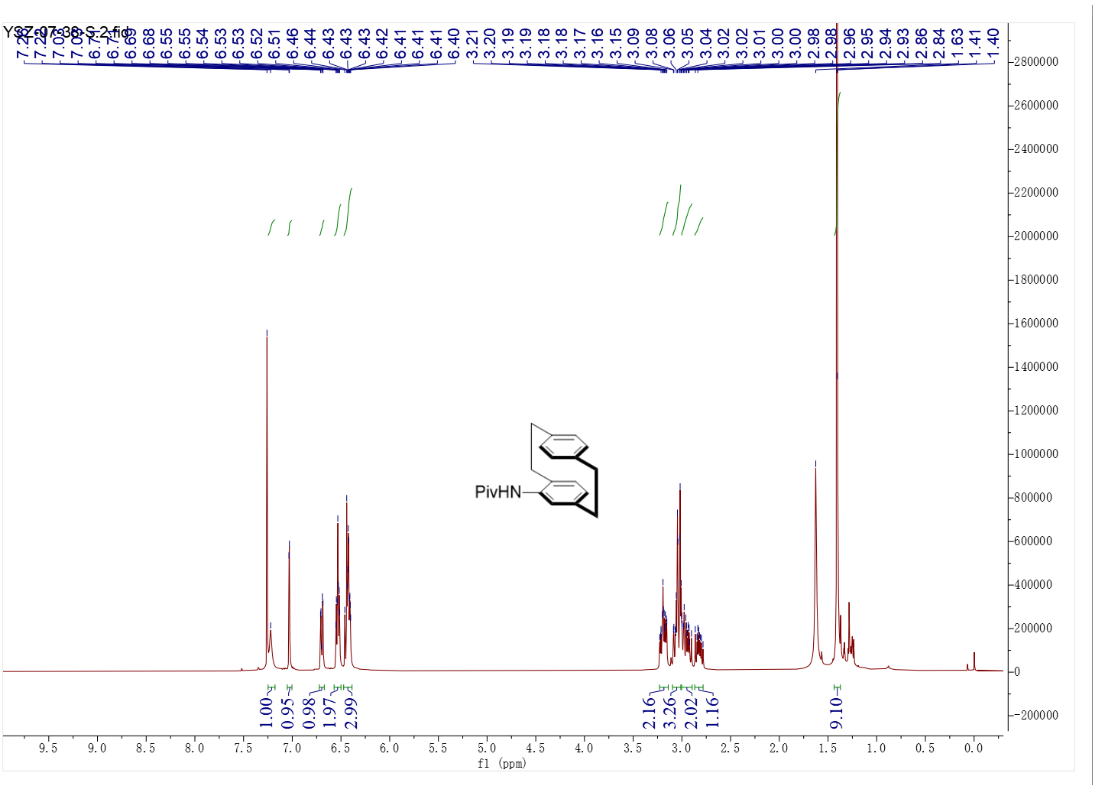

Supplementary Fig. 559 ^{1}H NMR spectrum of (R_p)-**6e** (400 MHz, $CDCl_3$)

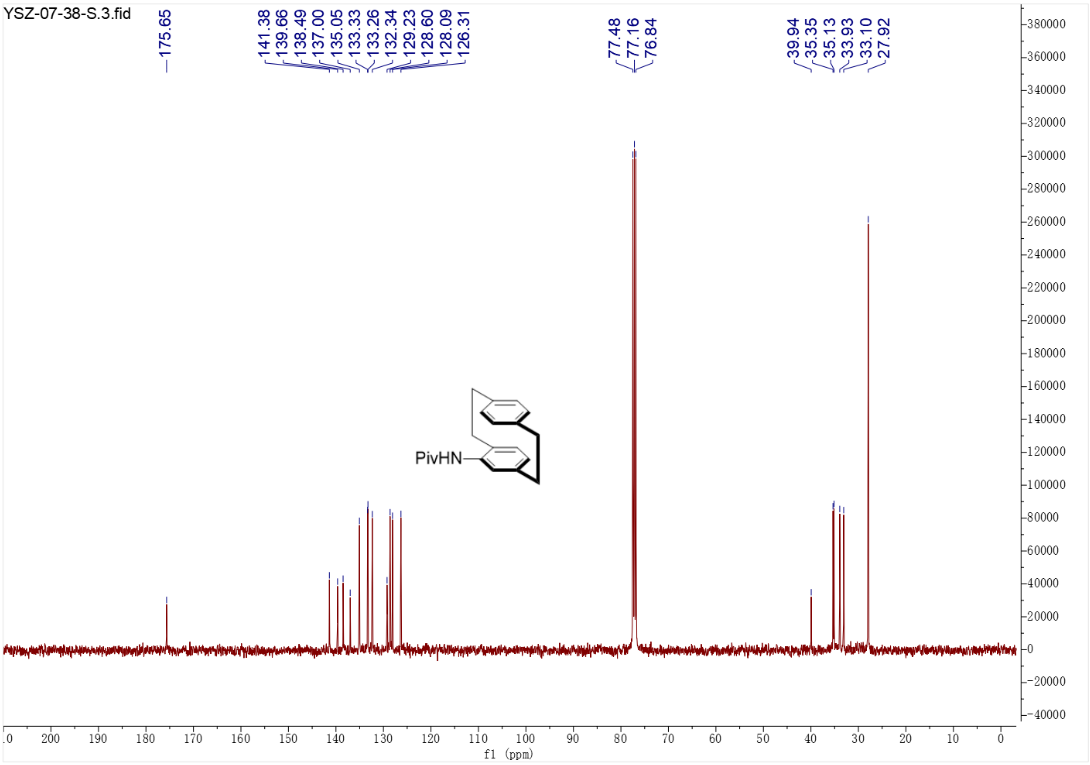

Supplementary Fig. 560 ^{13}C NMR spectrum of (R_p)-**6e** (101 MHz, $CDCl_3$)

(S_p)-Dibenzyl-1-(1^5-pivalamido-1,4(1,4)-dibenzenacyclohexaphane-1^2-yl)hydrazine-1,2-dicarboxylate **(7e)**

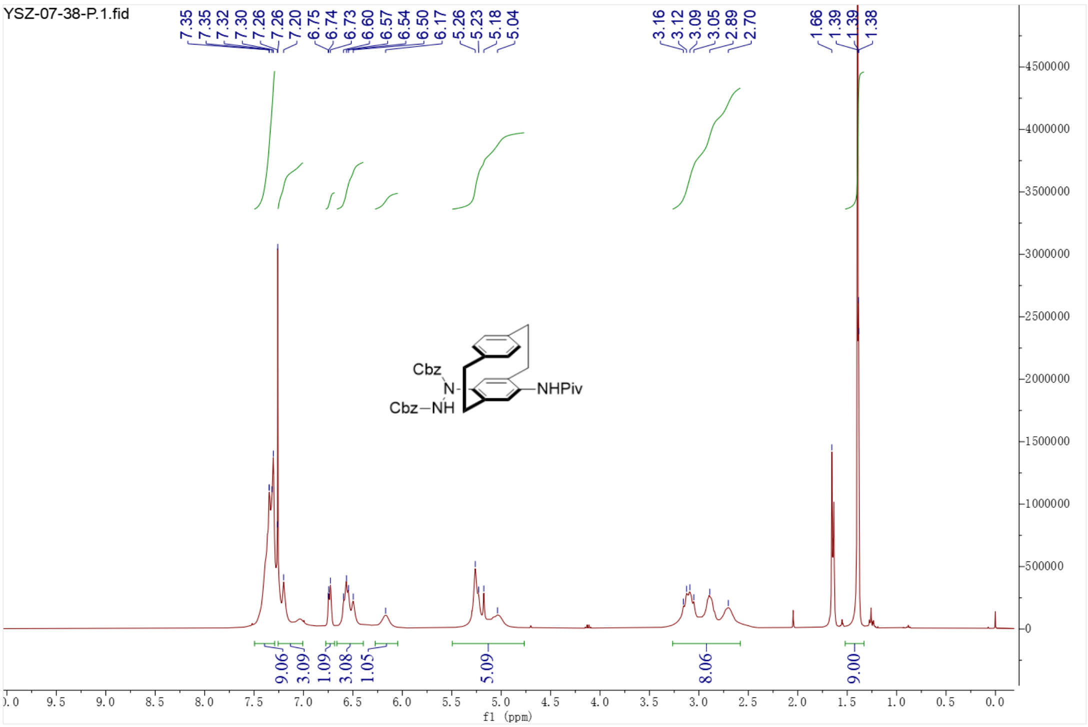

Supplementary Fig. 561 ^{1}H NMR spectrum of (S_p)-**7e** (400 MHz, $CDCl_3$)

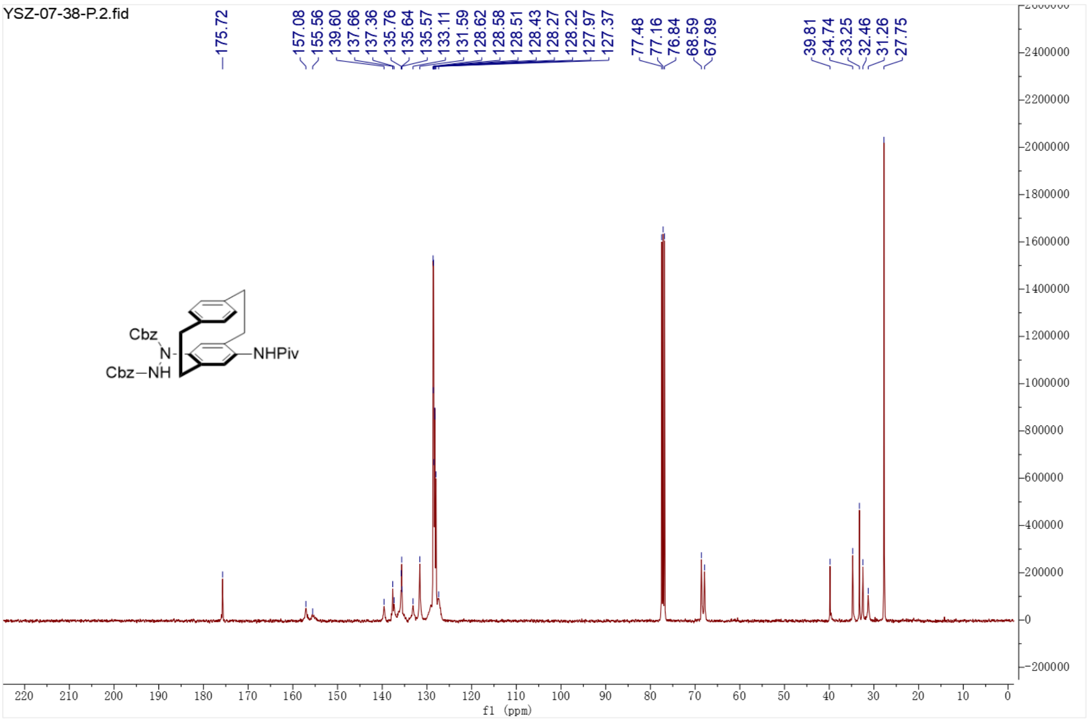

Supplementary Fig. 562 ^{13}C NMR spectrum of (S_p)-**7e** (101 MHz, $CDCl_3$)

N-(1,4(1,4)-dibenzenacyclohexaphane-1^2-yl)-4-methylbenzenesulfonamide **(6f)**

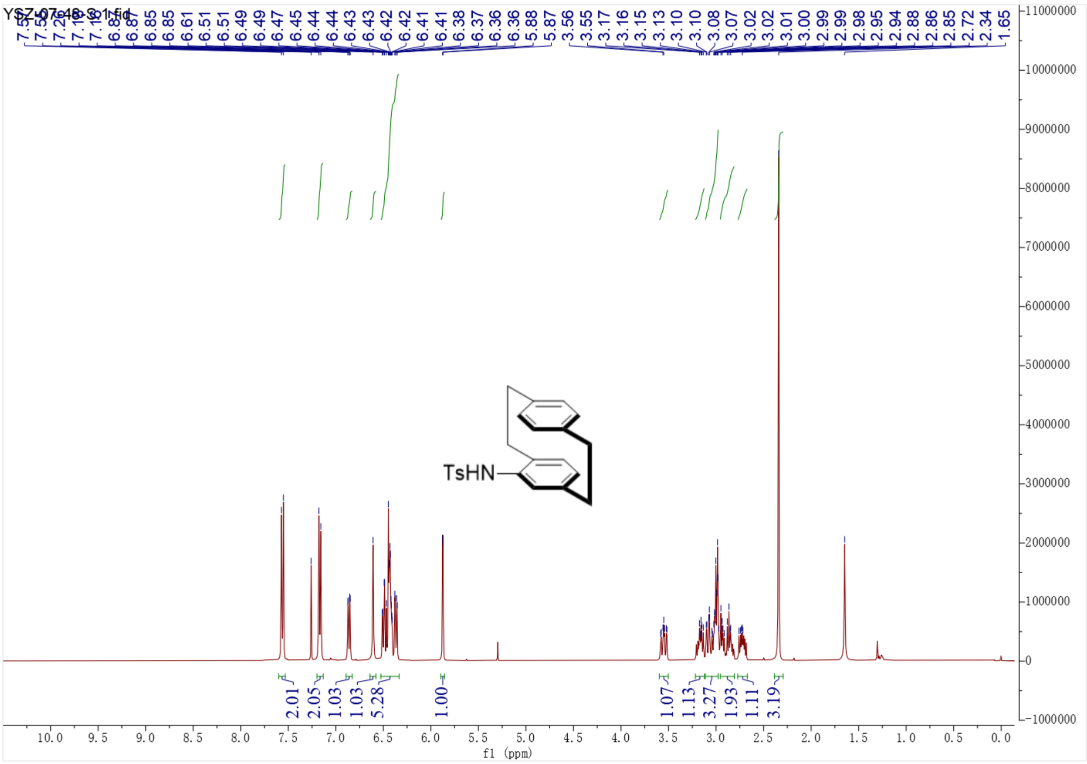

Supplementary Fig. 563 ^{1}H NMR spectrum of **6f** (400 MHz, $CDCl_3$)

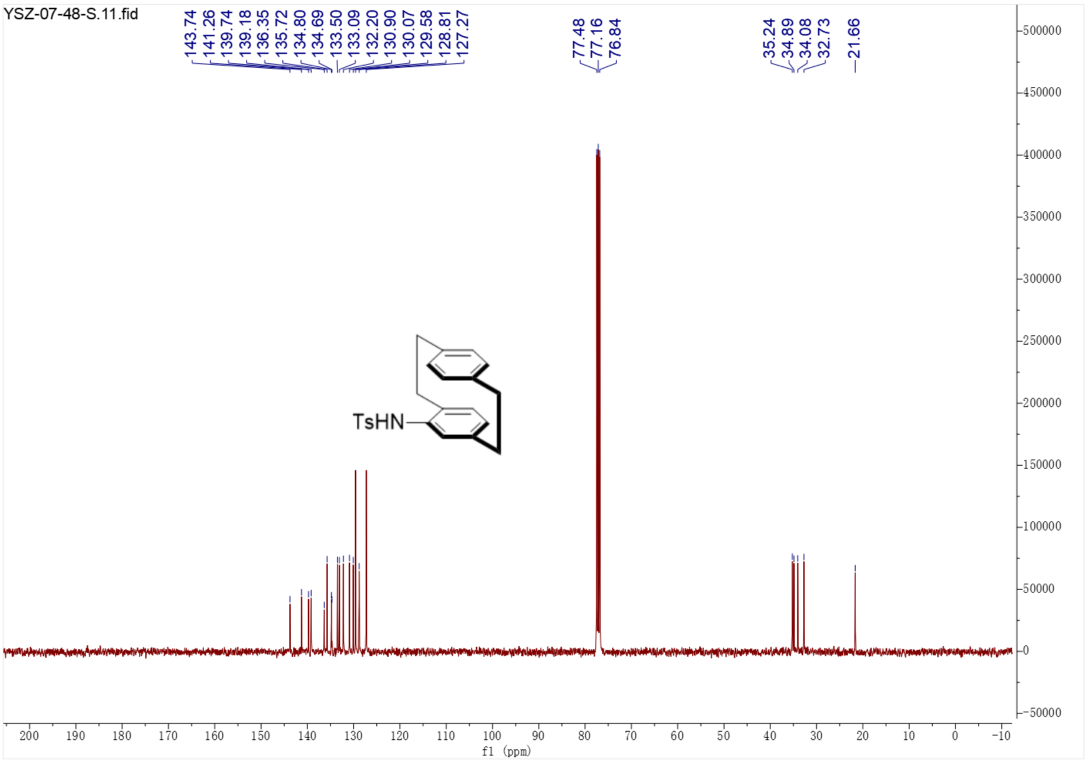

Supplementary Fig. 564 ^{13}C NMR spectrum of **6f** (101 MHz, $CDCl_3$)

Dibenzyl-1-(1^5-((4-methylphenyl)sulfonamido)-1,4(1,4)-dibenzenacyclohexaphane-1^2-yl)hydrazine-1,2-dicarboxylate **(7f)**

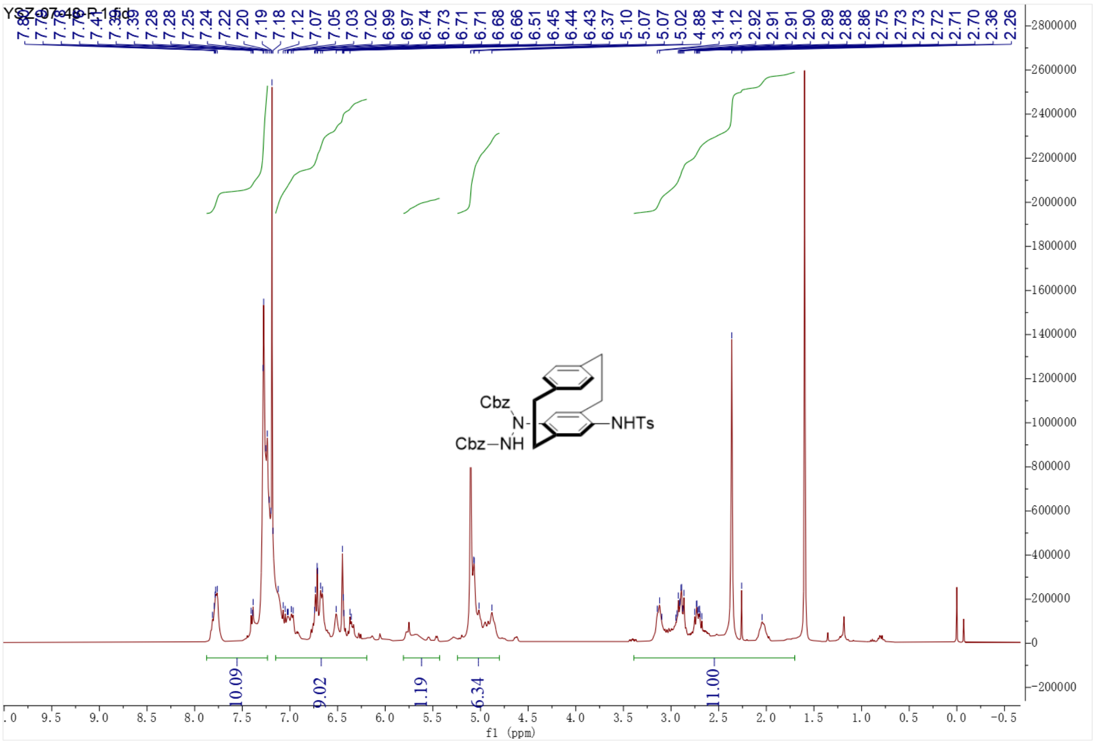

Supplementary Fig. 565 ^{1}H NMR spectrum of **7f** (400 MHz, $CDCl_3$)

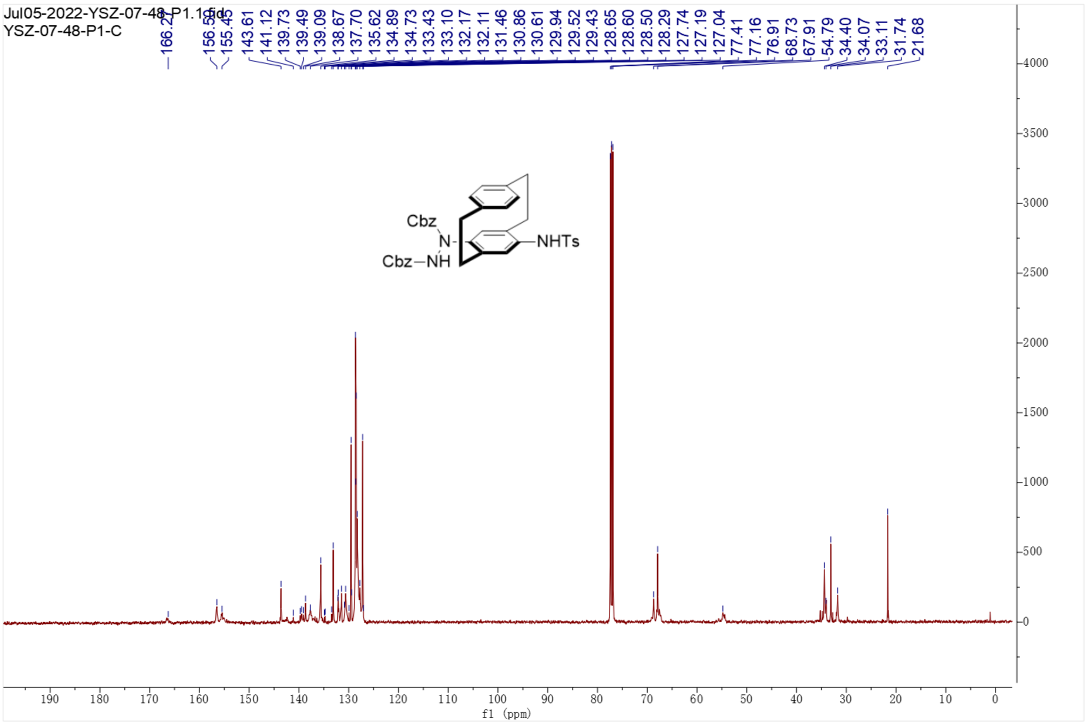

Supplementary Fig. 566 ^{13}C NMR spectrum of **7f** (126 MHz, $CDCl_3$)

1,4(1,4)-dibenzenacyclohexaphan-1^2-amine **(6g)**

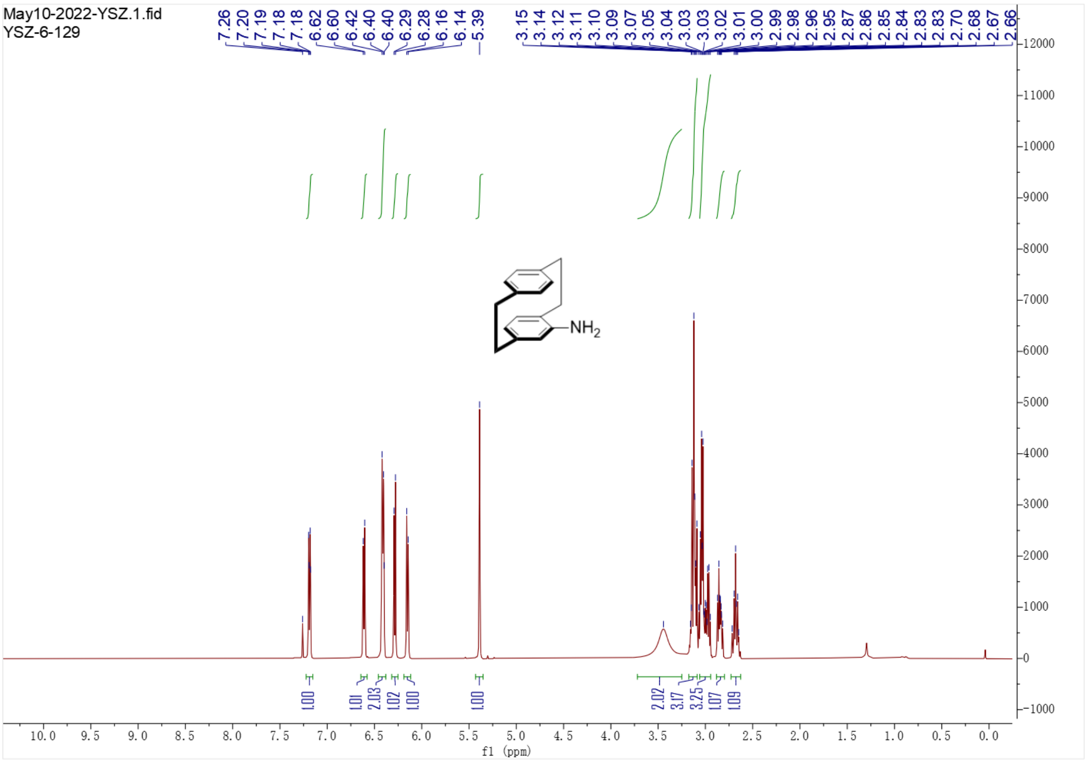

Supplementary Fig. 567 ^{1}H NMR spectrum of **6g** (500 MHz, $CDCl_3$)

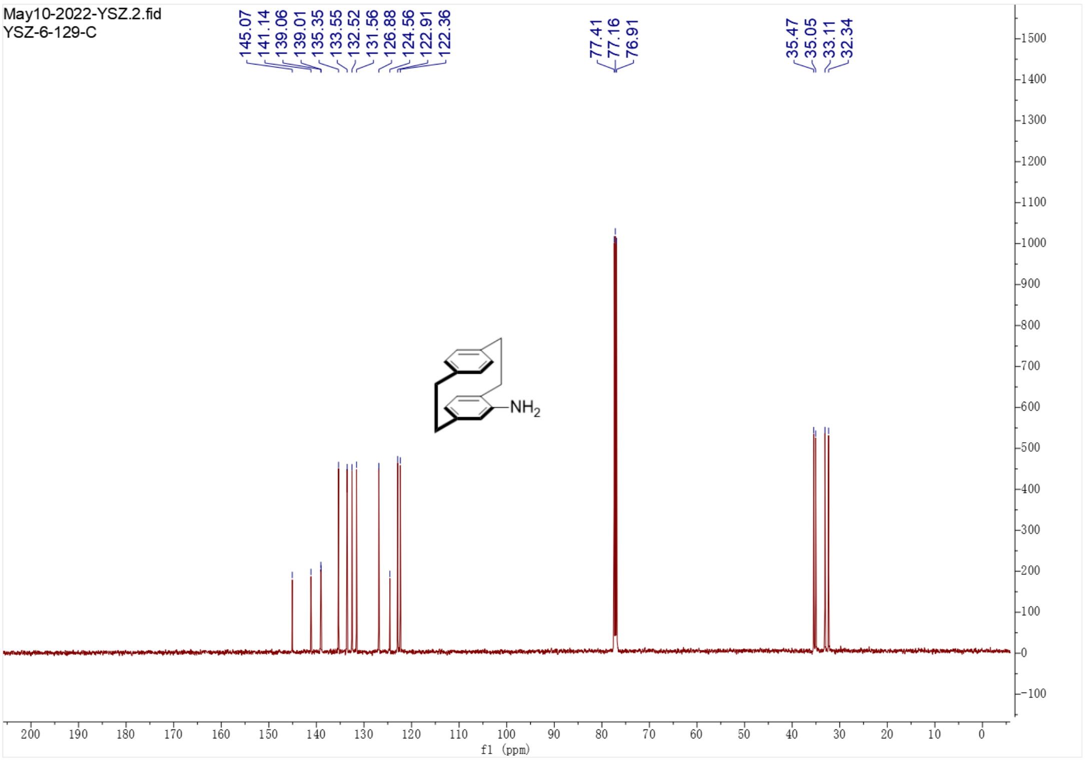

Supplementary Fig. 568 ^{13}C NMR spectrum of **6g** (126 MHz, $CDCl_3$)

Dibenzyl-1-(1[5]-amino-1,4(1,4)-dibenzenacyclohexaphane-1[2]-yl)hydrazine-1,2-dicarboxylate **(7g)**

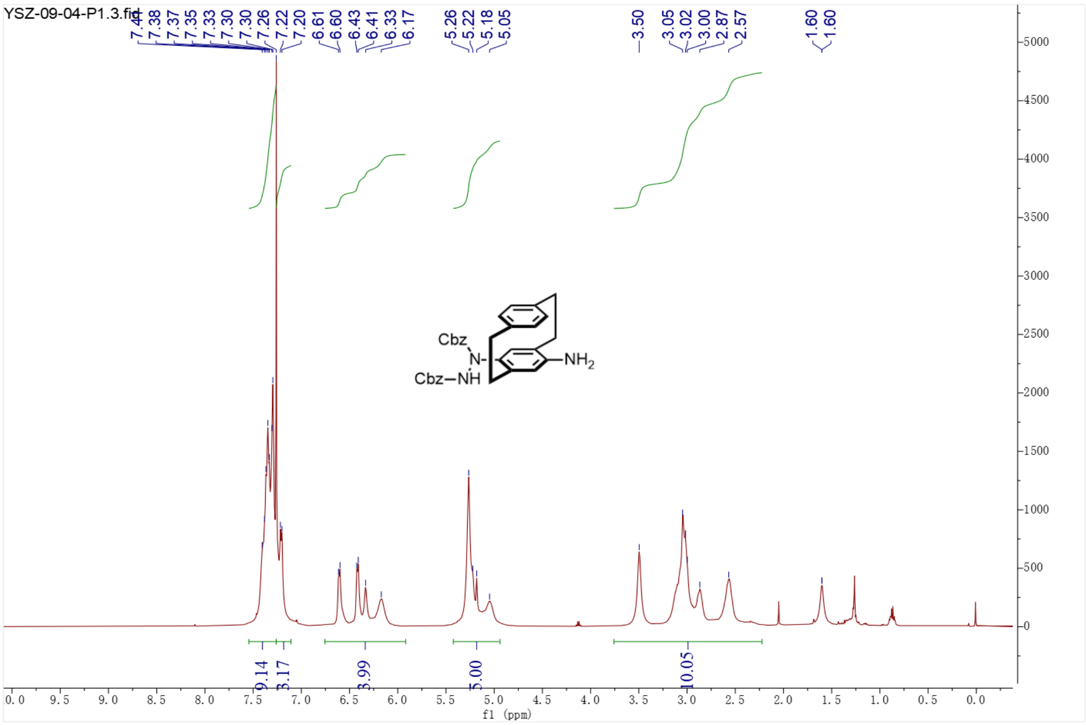

Supplementary Fig. 569 ^{1}H NMR spectrum of **7g** (500 MHz, $CDCl_3$)

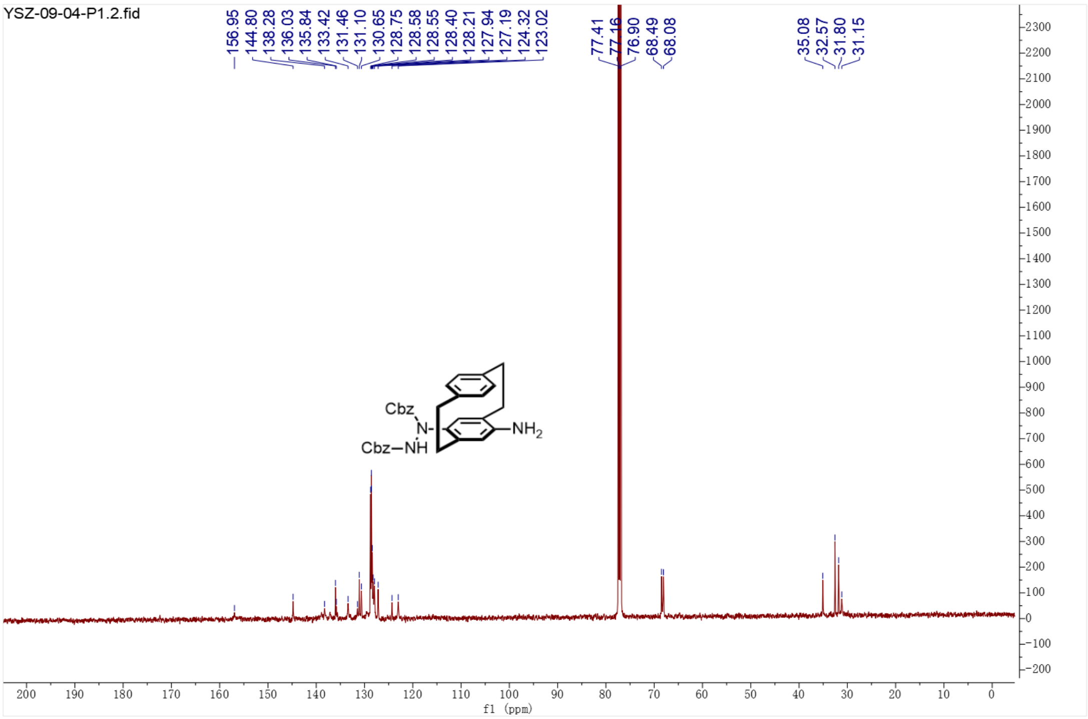

Supplementary Fig. 570 ^{13}C NMR spectrum of **7g** (126 MHz, $CDCl_3$)

Dibenzyl 3-(1,4(1,4)-dibenzenacyclohexaphane-1^2-yl)triazane-1,2-dicarboxylate **(8g)**

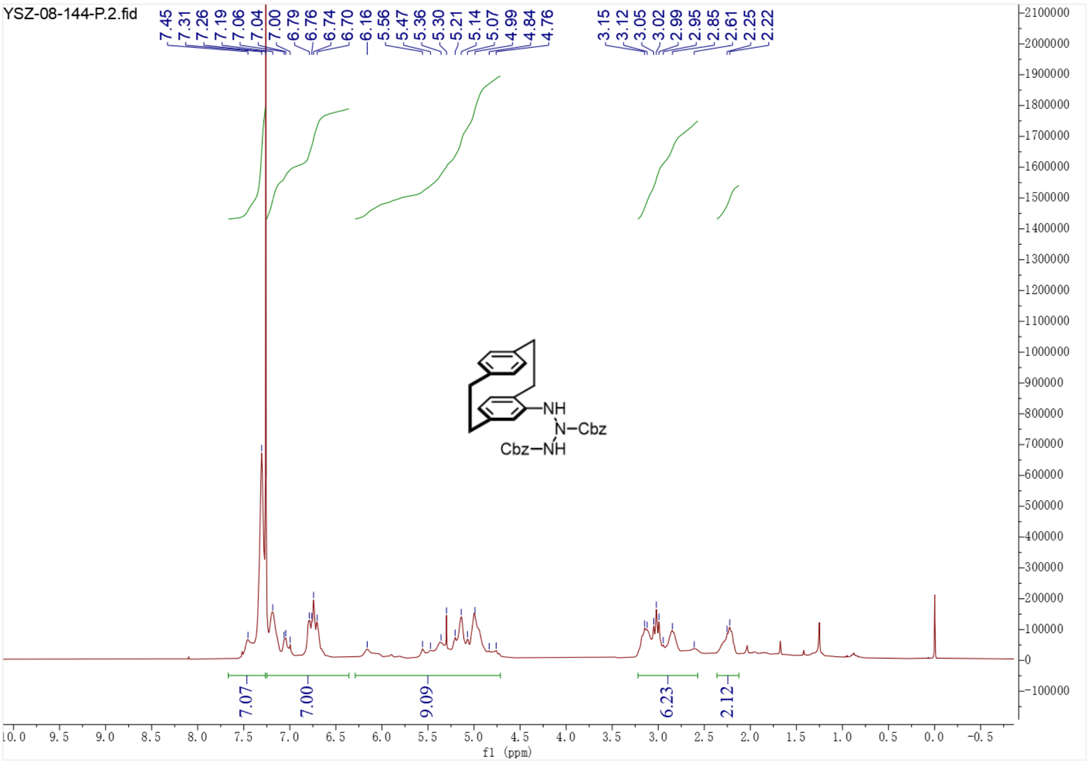

Supplementary Fig. 571 ^{1}H NMR spectrum of **8g** (500 MHz, $CDCl_3$)

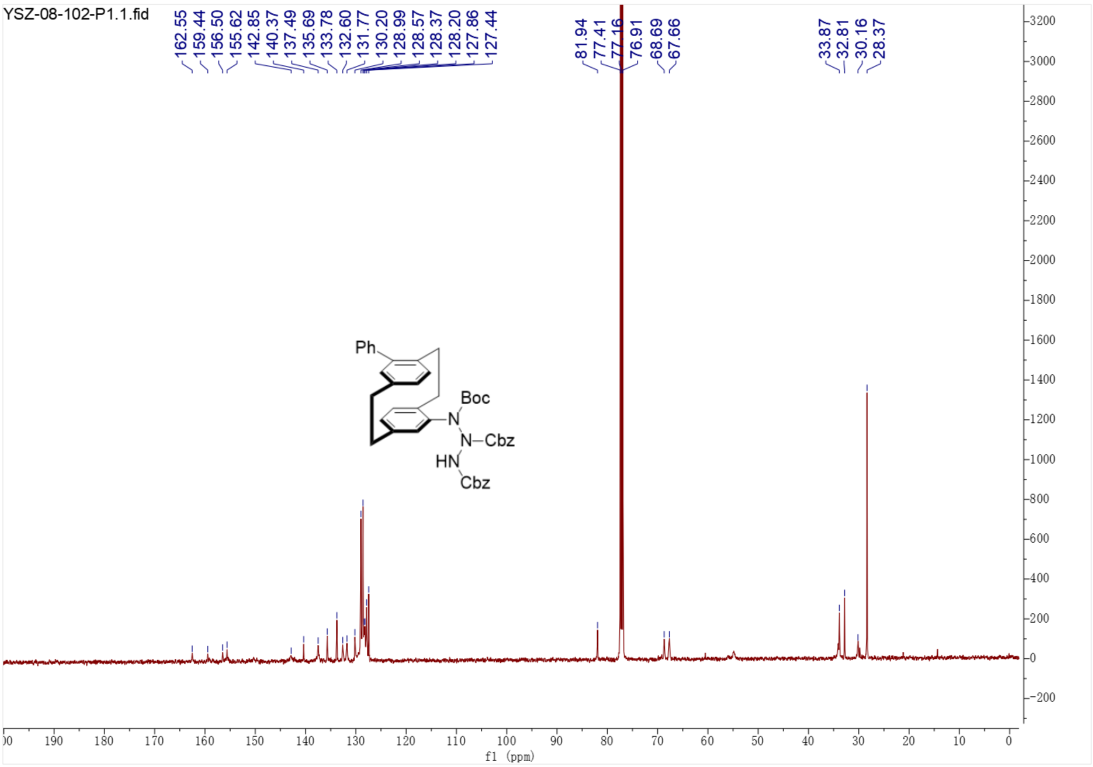

Supplementary Fig. 572 ^{13}C NMR spectrum of **8g** (126 MHz, $CDCl_3$)

2,3-dibenzyl 1-(tert-butyl) 1-(4^2-phenyl-1,4(1,4)-dibenzenacyclohexaphane-12-yl)triazane-1,2,3-tricarboxylate **(8x)**

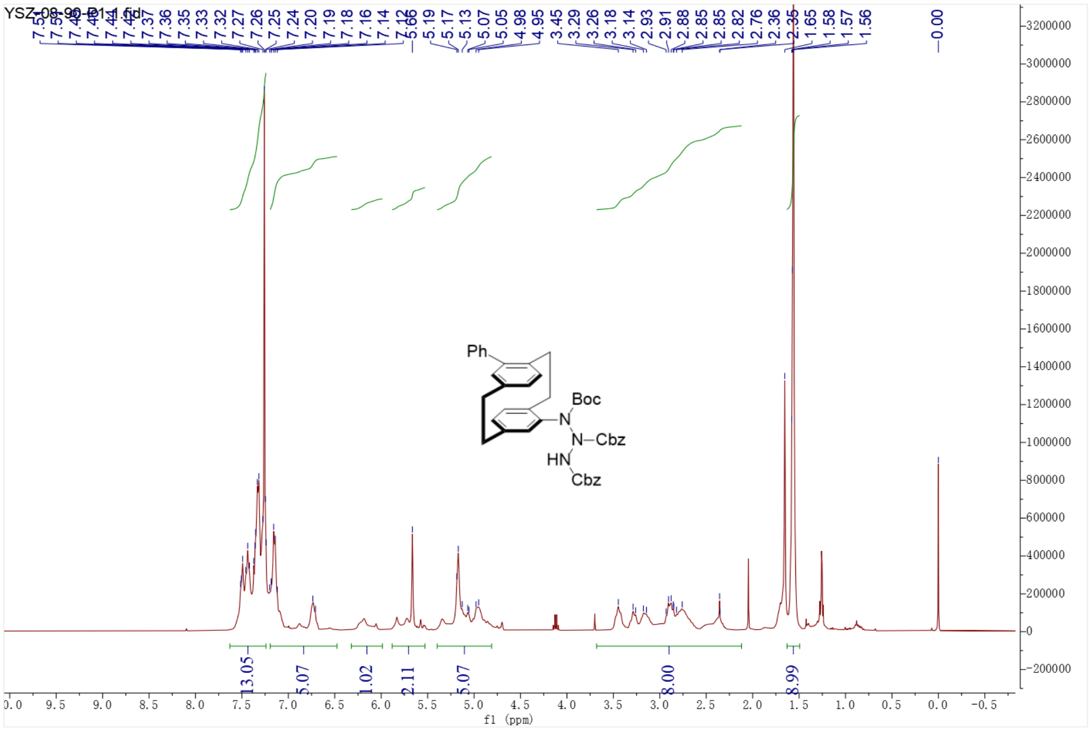

Supplementary Fig. 573 ^{1}H NMR spectrum of **8x** (400 MHz, $CDCl_3$)

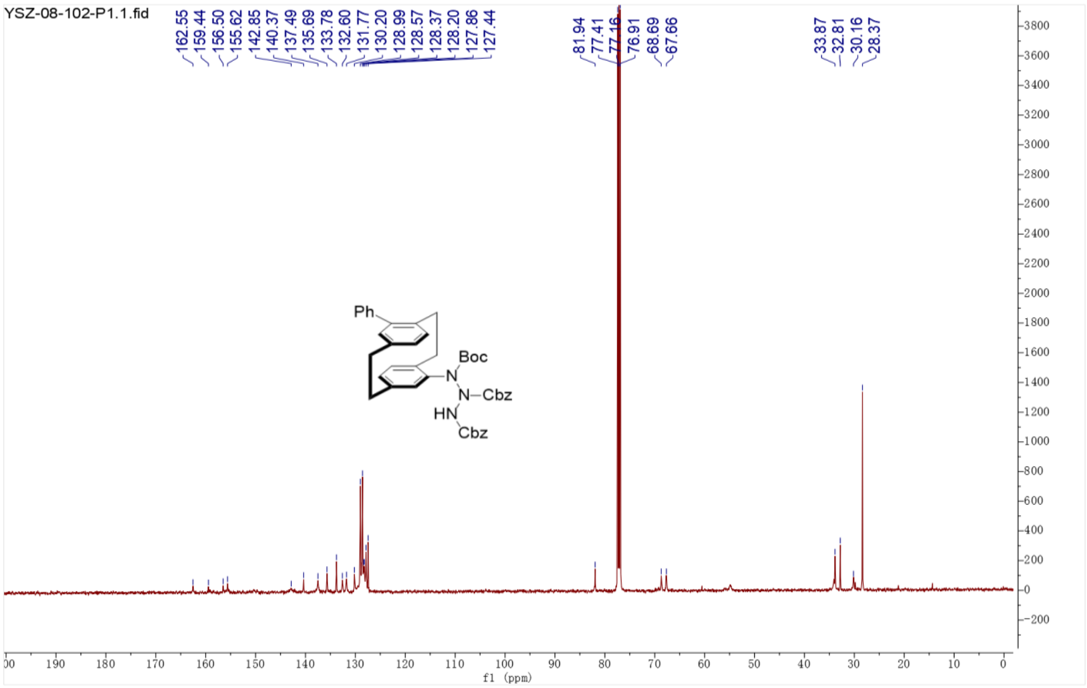

Supplementary Fig. 574 ^{13}C NMR spectrum of **8x** (126 MHz, $CDCl_3$)

(S_p)-Tert-butyl-(1^5-amino-4^2-phenyl-1,4(1,4)-dibenzenacyclohexaphane-1^2-yl)carbamate **(9x)**

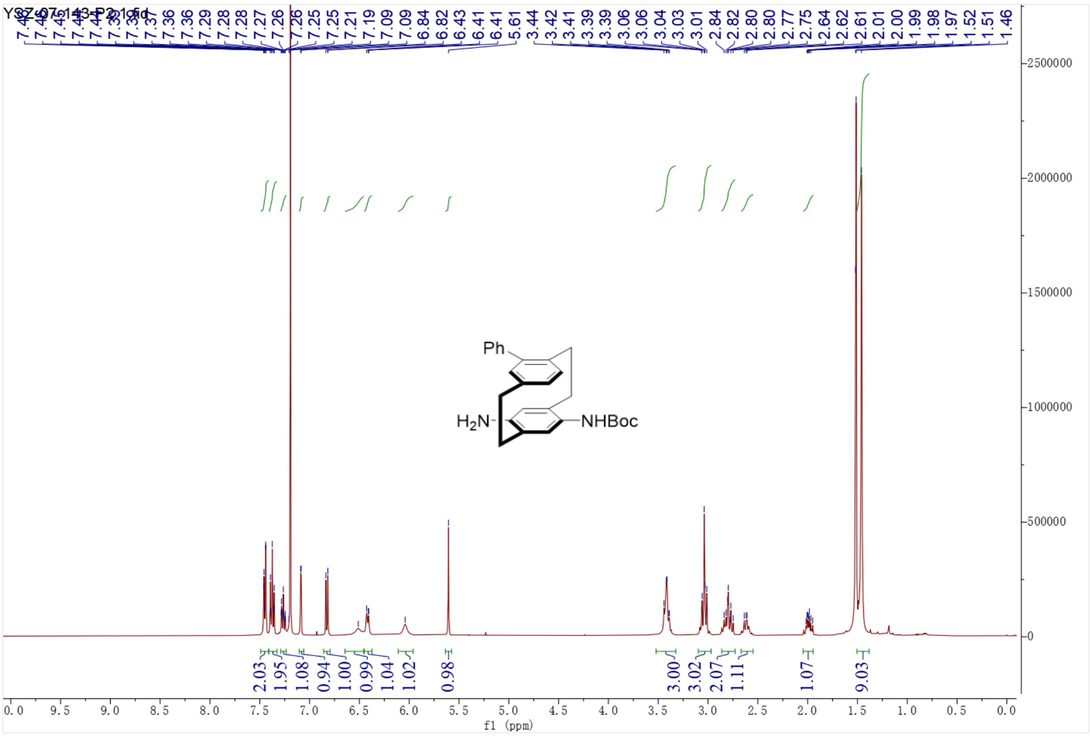

Supplementary Fig. 575 ^1H NMR spectrum of **9x** (400 MHz, $CDCl_3$)

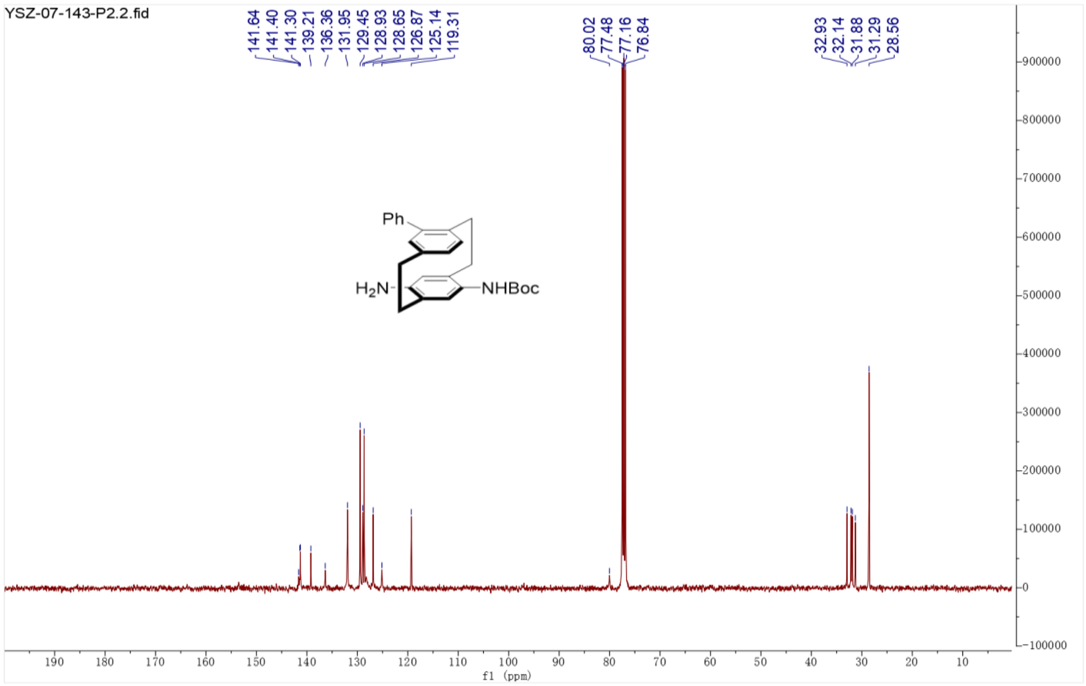

Supplementary Fig. 576 ^{13}C NMR spectrum of **9x** (101 MHz, $CDCl_3$)

Crude ^{1}H NMR of tert-butyl (1^5-amino-4^2-phenyl-1,4(1,4)-dibenzenacyclohexaphane-12-yl)carbamate **(8x)** and tert-butyl (4^2-phenyl-1,4(1,4)-dibenzenacyclohexaphane-1^2-yl)carbamate **(1x)**

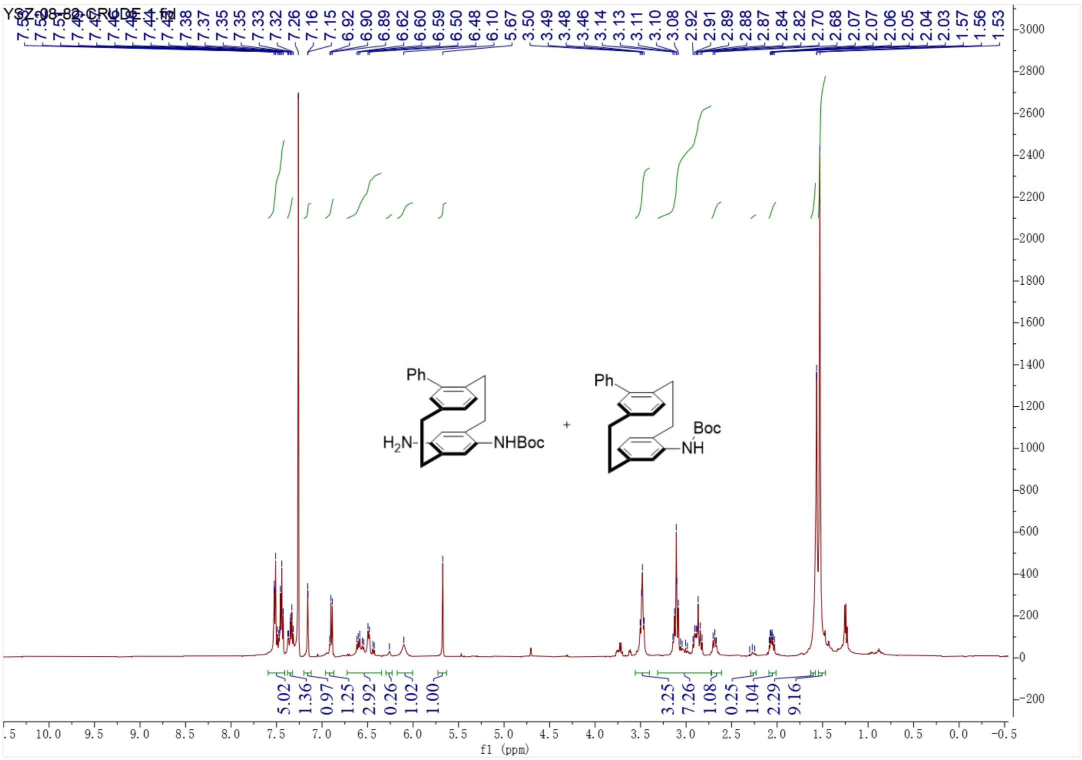

Supplementary Fig. 577 ^{1}H NMR spectrum of **8x** and **1x** (500 MHz, $CDCl_3$)

(S_p)-Tert-butyl (1[5]-amino-1,4(1,4)-dibenzenacyclohexaphane-1[2]-yl)carbamate **(9a)**

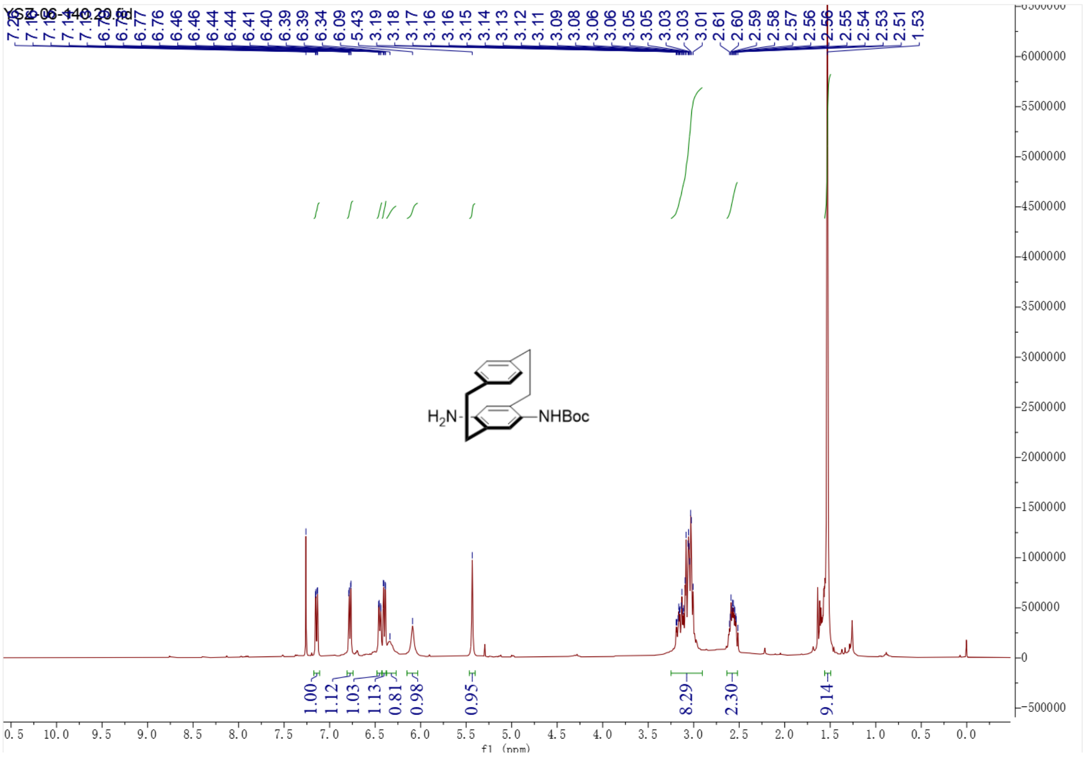

Supplementary Fig. 578 ^{1}H NMR spectrum of (S_p)**-9a** (400 MHz, $CDCl_3$)

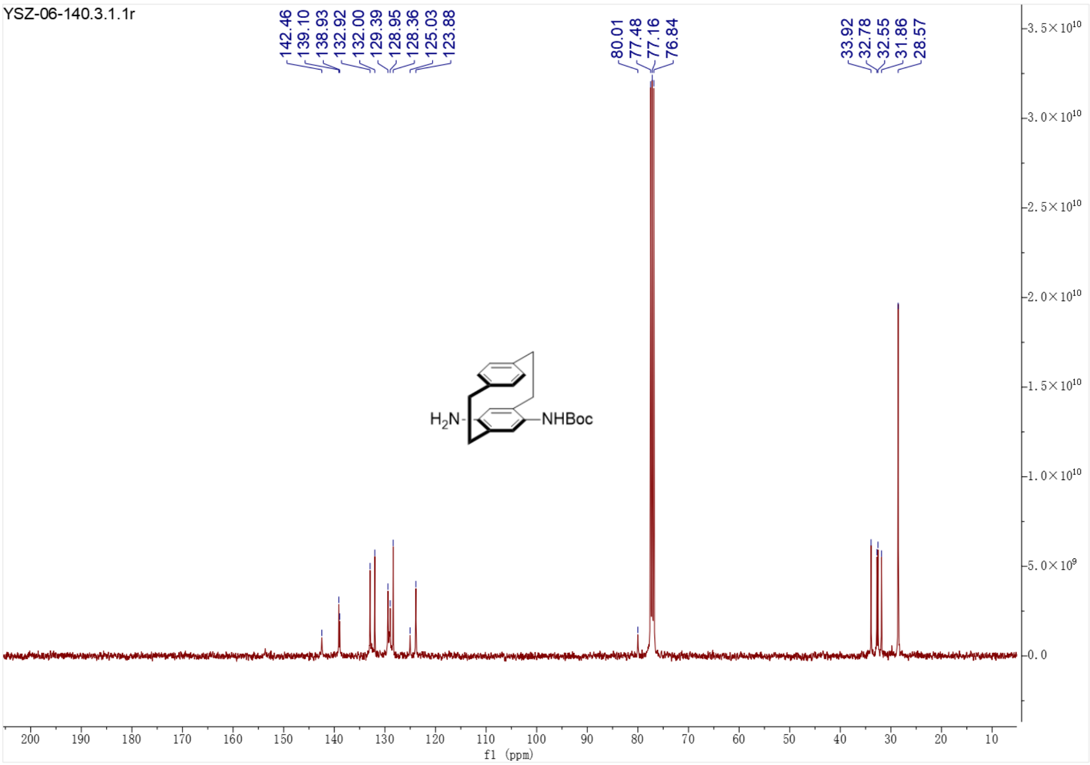

Supplementary Fig. 579 ^{13}C NMR spectrum of (S_p)**-9a** (101 MHz, $CDCl_3$)

(S_p)-Tert-butyl-(1[5]-(3-(3,5-bis(trifluoromethyl)phenyl)thioureido)-1,4(1,4)-dibenzenacyclohexaphane-1[2]-yl)carbamate **(10a)**

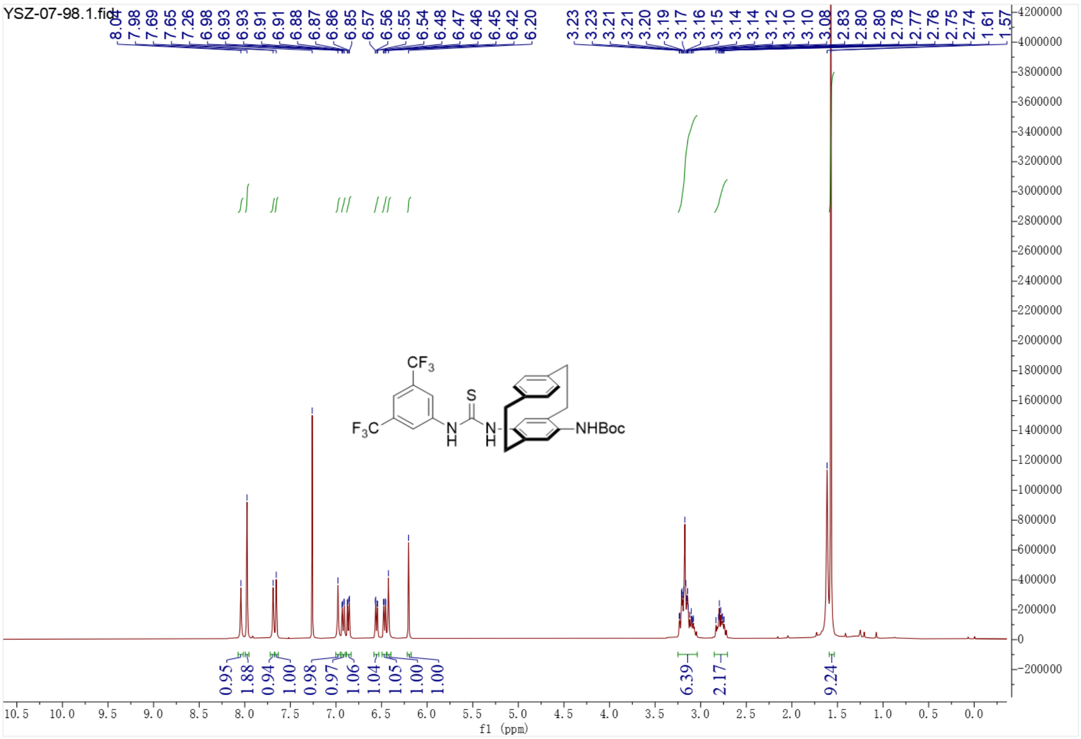

Supplementary Fig. 580 ^{1}H NMR spectrum of (S_p)**-10a** (400 MHz, $CDCl_3$)

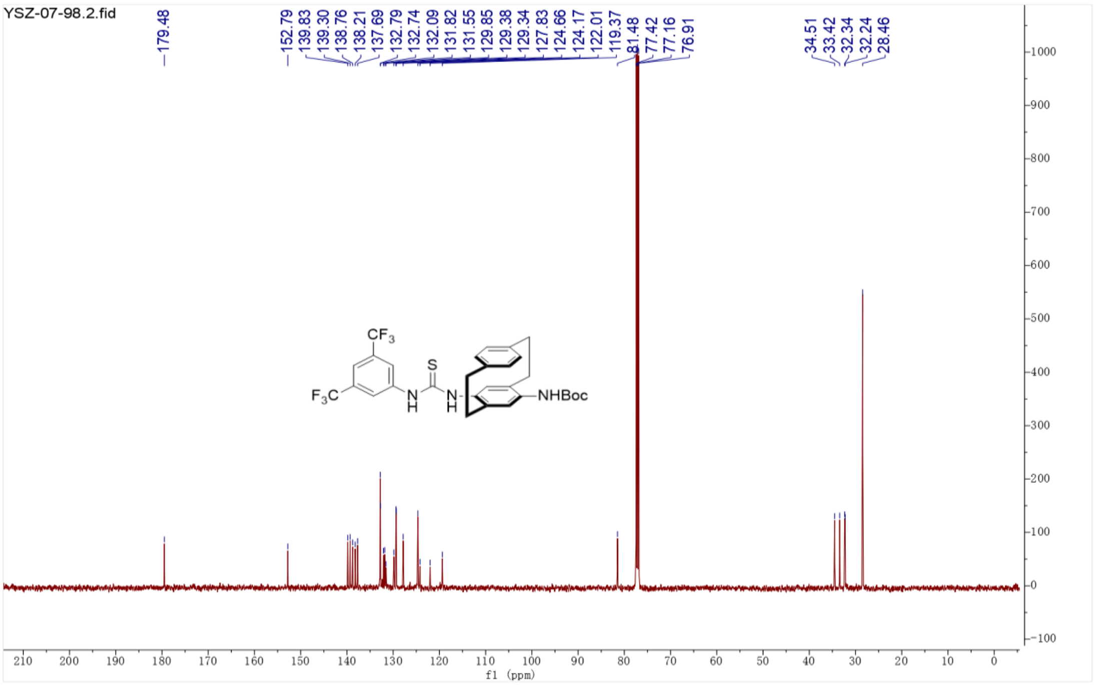

Supplementary Fig. 581 ^{13}C NMR spectrum of (S_p)**-10a** (126 MHz, $CDCl_3$)

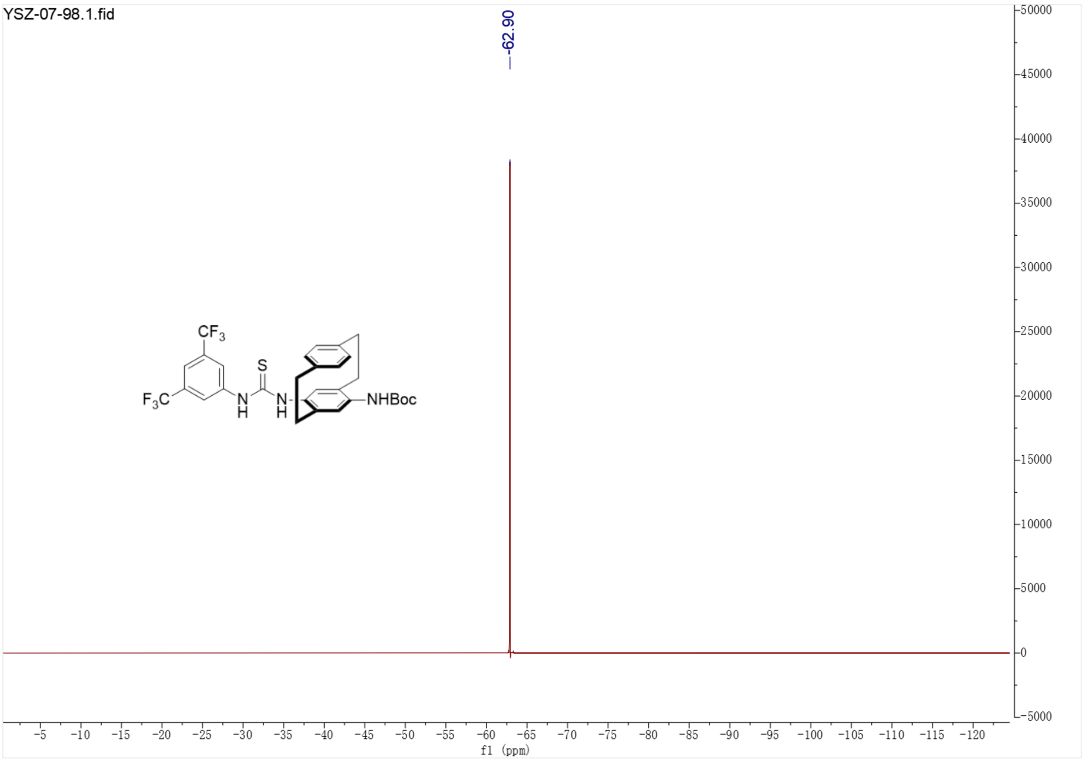

Supplementary Fig. 582 ^{19}F NMR spectrum of (*S_p*)-**10a** (471 MHz, $CDCl_3$)

(S_p)-1,1'-(1,4(1,4)-dibenzenacyclohexaphane-1^2,1^5-diyl)bis(3-(3,5-bis(trifluoromethyl) phenyl)thiourea) **(11a)**

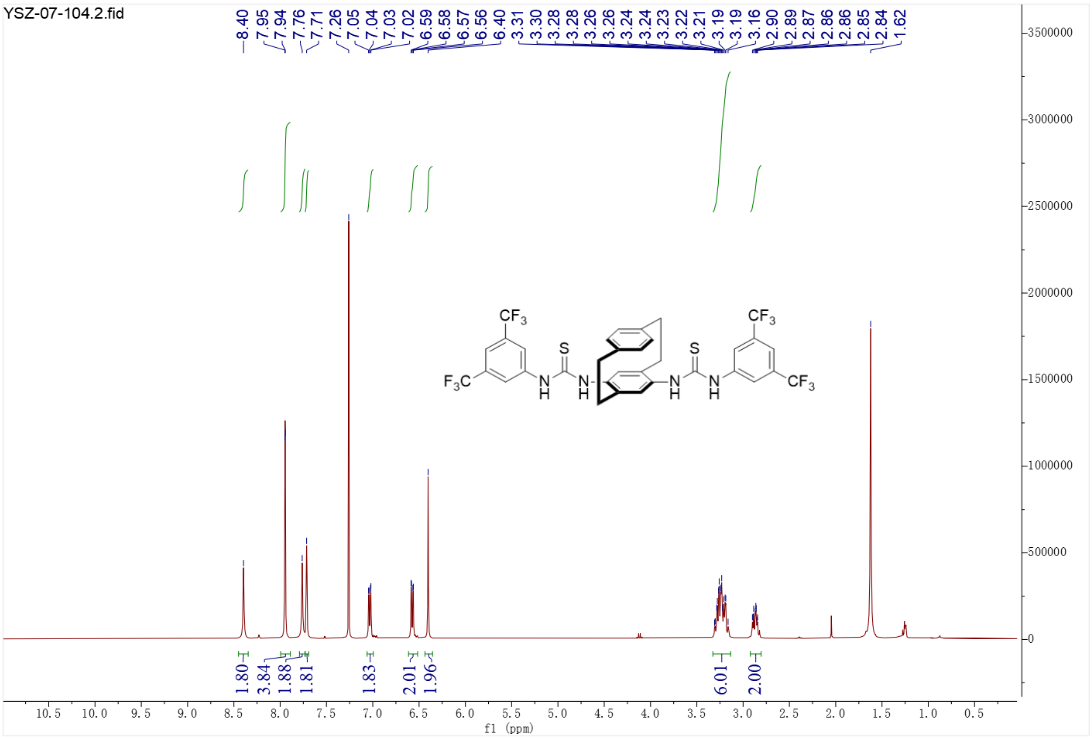

Supplementary Fig. 583 ^{1}H NMR spectrum of (S_p)-**11a** (400 MHz, $CDCl_3$)

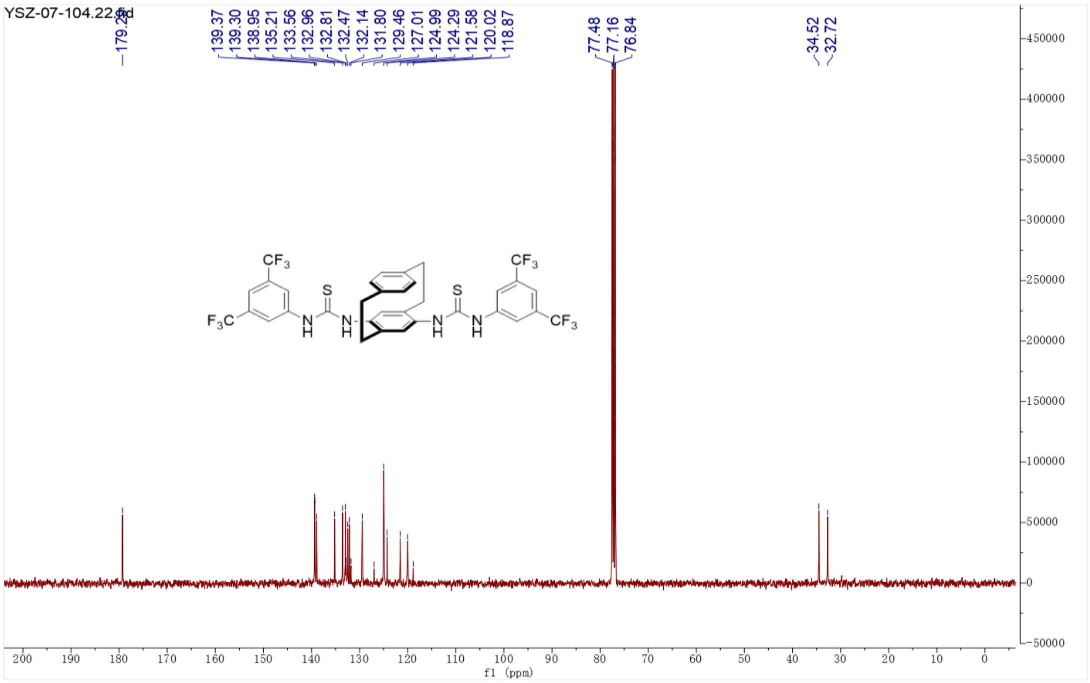

Supplementary Fig. 584 ^{13}C NMR spectrum of (S_p)-**11a** (101 MHz, $CDCl_3$)

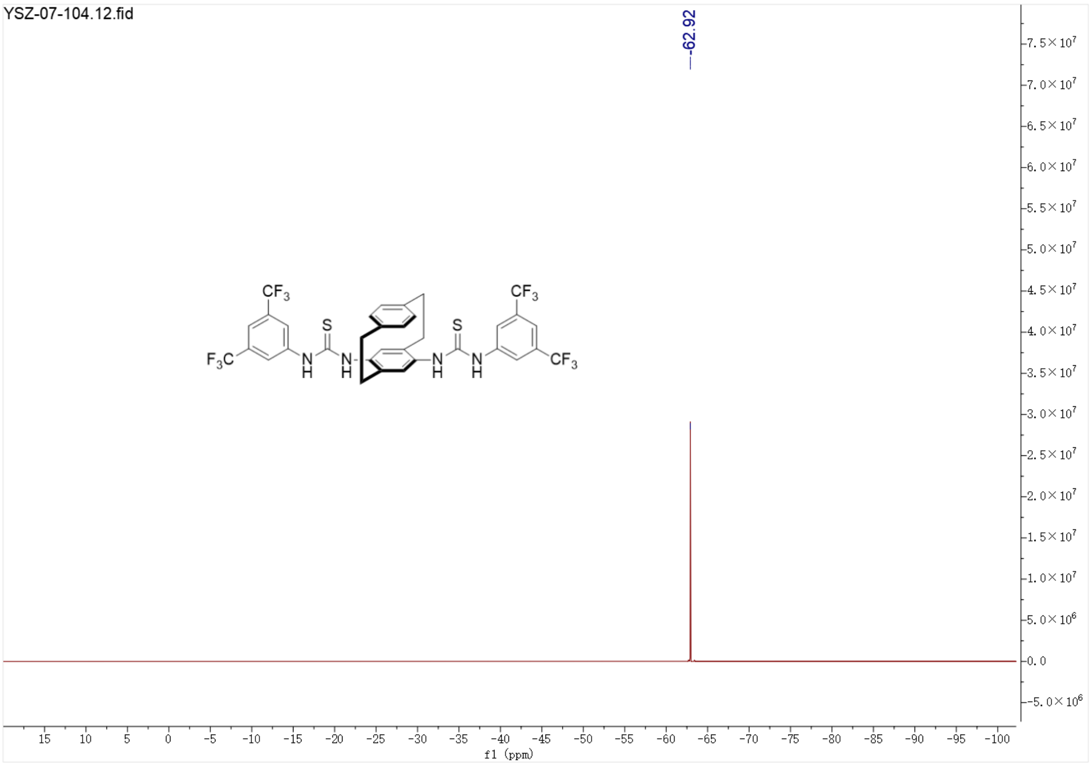

Supplementary Fig. 585 ^{19}F NMR spectrum of (S_p)-**11a** (471 MHz, $CDCl_3$)

(S_p)-Tert-butyl (1^5-iodo-1,4(1,4)-dibenzenacyclohexaphane-1^2-yl)carbamate **(12a)**

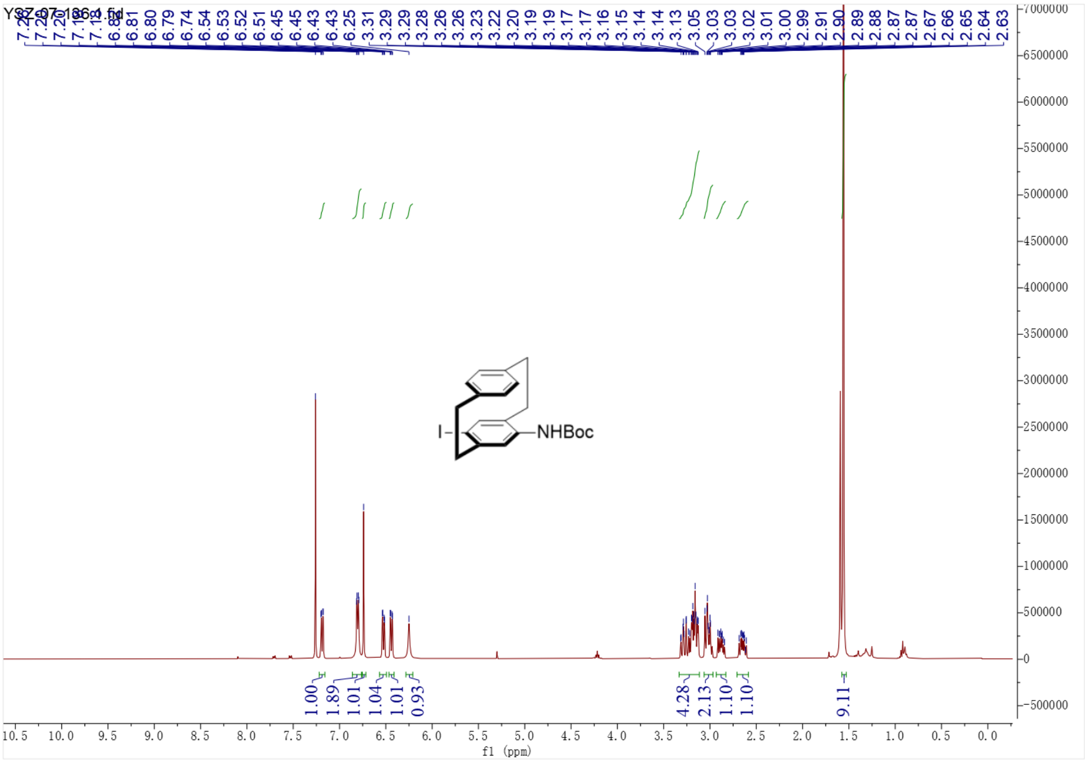

Supplementary Fig. 586 ^{1}H NMR spectrum of (S_p)**-12a** (400 MHz, $CDCl_3$)

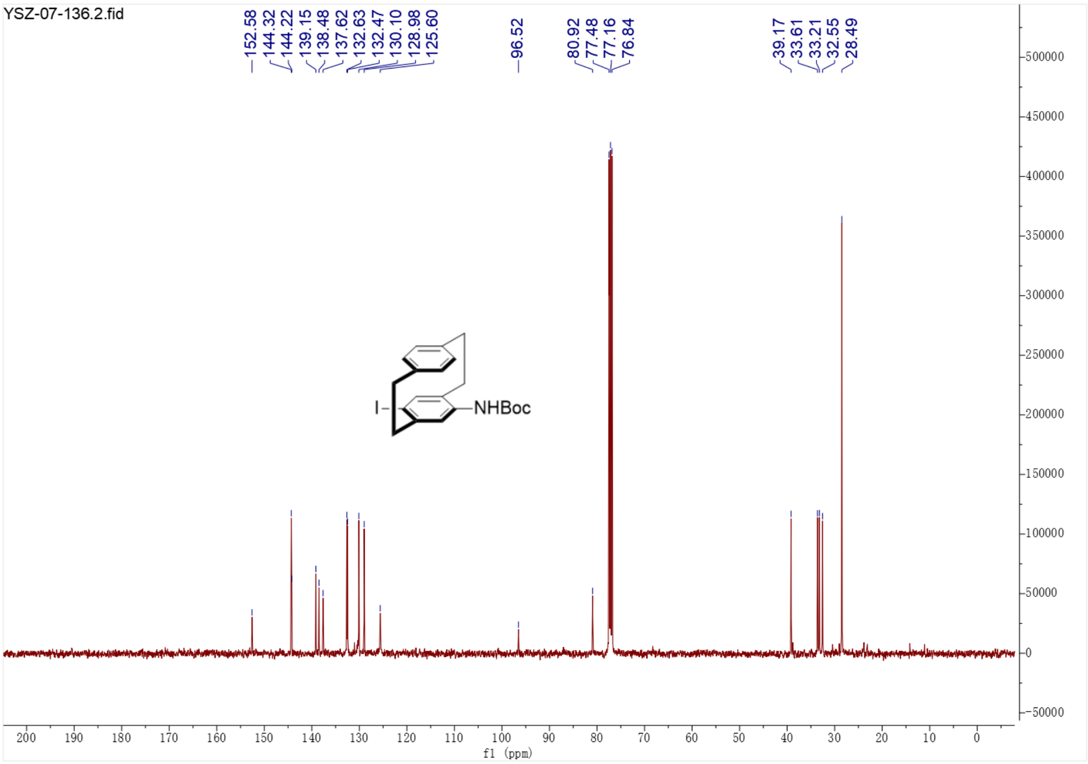

Supplementary Fig. 587 ^{13}C NMR spectrum of (S_p)**-12a** (101 MHz, $CDCl_3$)

(S_p)-*N*-(4^2-bromo-1,4(1,4)-dibenzenacyclohexaphane-1^2-yl)pivalamide **(13f)**

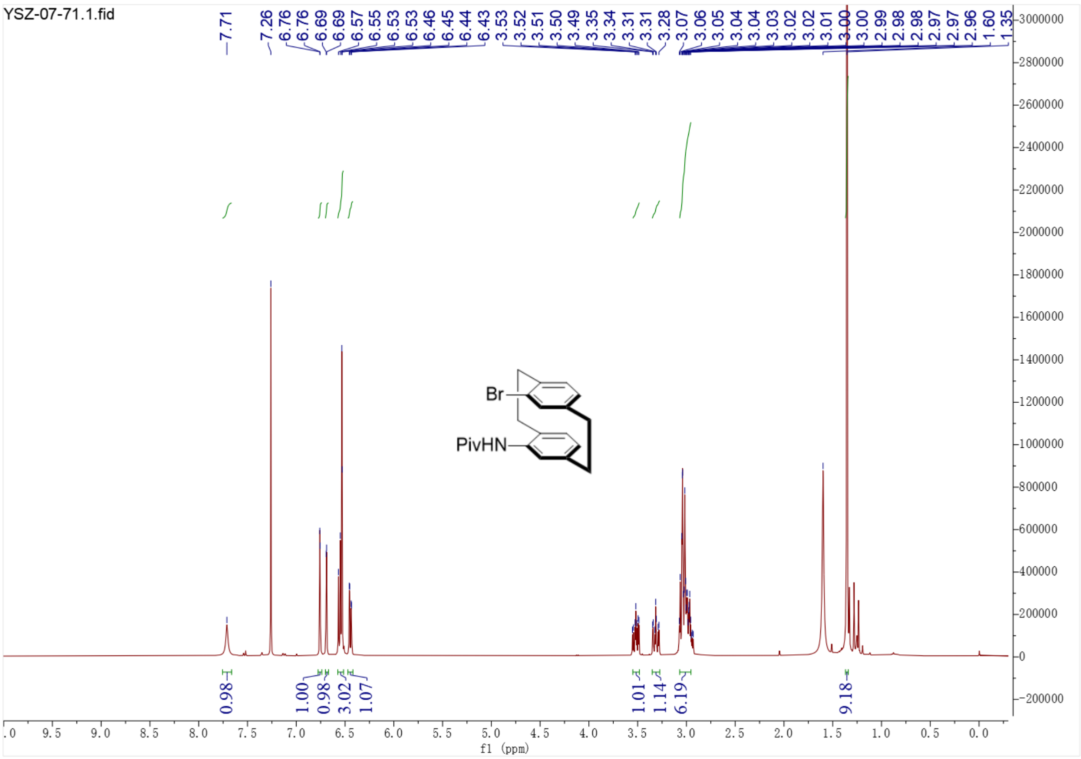

Supplementary Fig. 588 ^{1}H NMR spectrum of (S_p)**-13f** (400 MHz, $CDCl_3$)

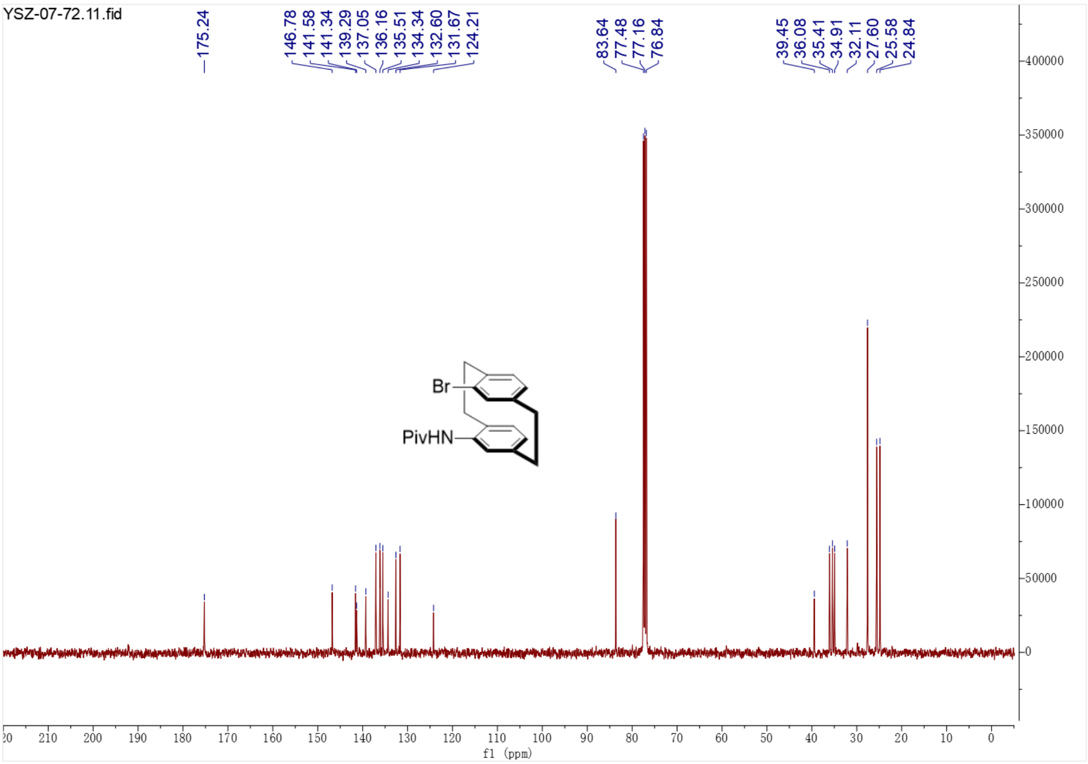

Supplementary Fig. 589 ^{13}C NMR spectrum of (S_p)**-13f** (101 MHz, $CDCl_3$)

(S_p)-*N*-(4^2-bromo-1^3-(4,4,5,5-tetramethyl-1,3,2-dioxaborolan-2-yl)-1,4(1,4)-dibenzenacyclohexaphane-1^2-yl)pivalamide **(14f)**

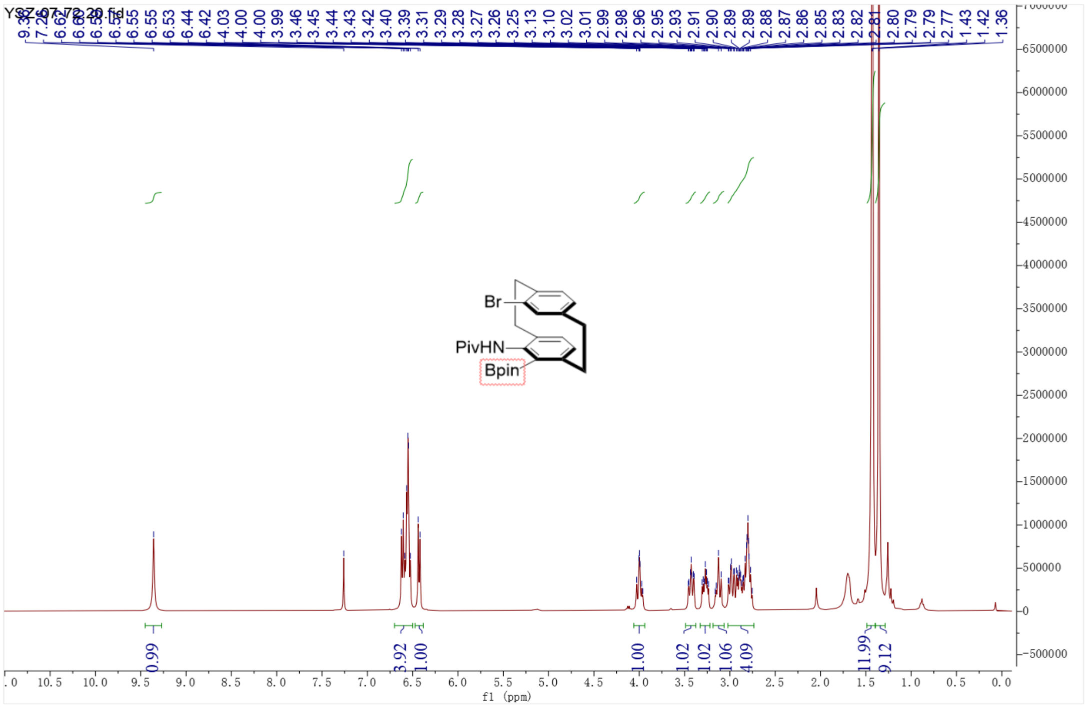

Supplementary Fig. 590 ^{1}H NMR spectrum of (S_p)-**14f** (400 MHz, $CDCl_3$)

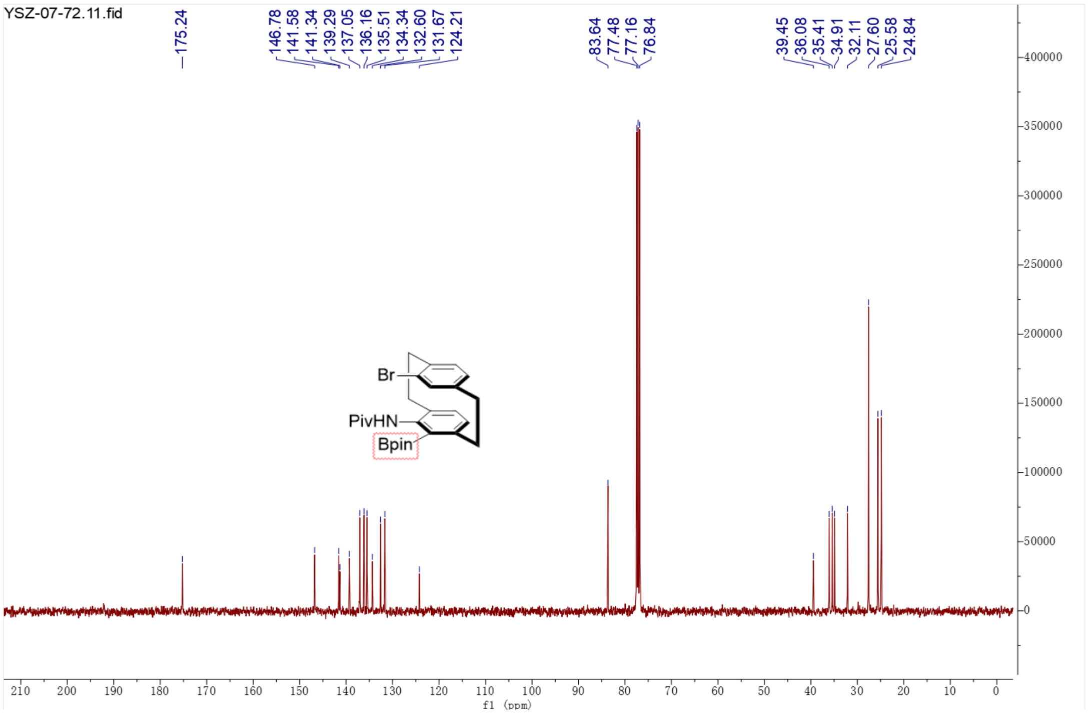

Supplementary Fig. 591 ^{13}C NMR spectrum of (S_p)-**14f** (101 MHz, $CDCl_3$)

(S_p)--(E)-4-((4^2-bromo-1,4(1,4)-dibenzenacyclohexaphane-1^2-yl)amino)pent-3-en-2-one (**INT 1**)

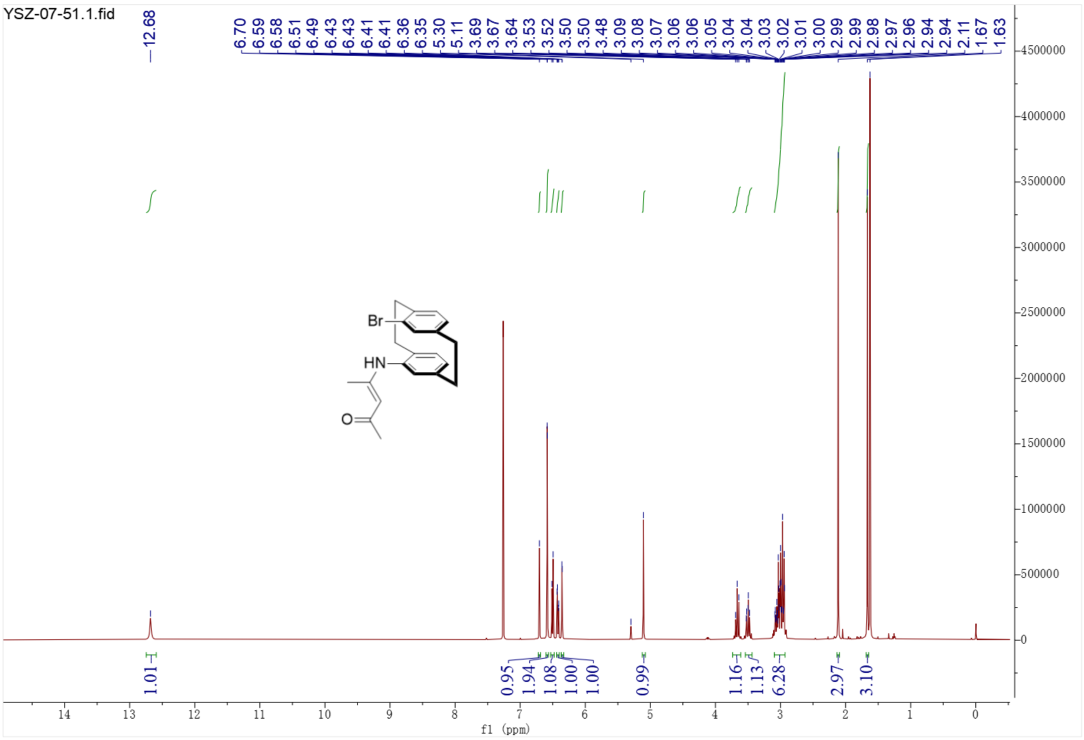

Supplementary Fig. 592 ^{1}H NMR spectrum of (S_p)-**INT 1** (400 MHz, $CDCl_3$)

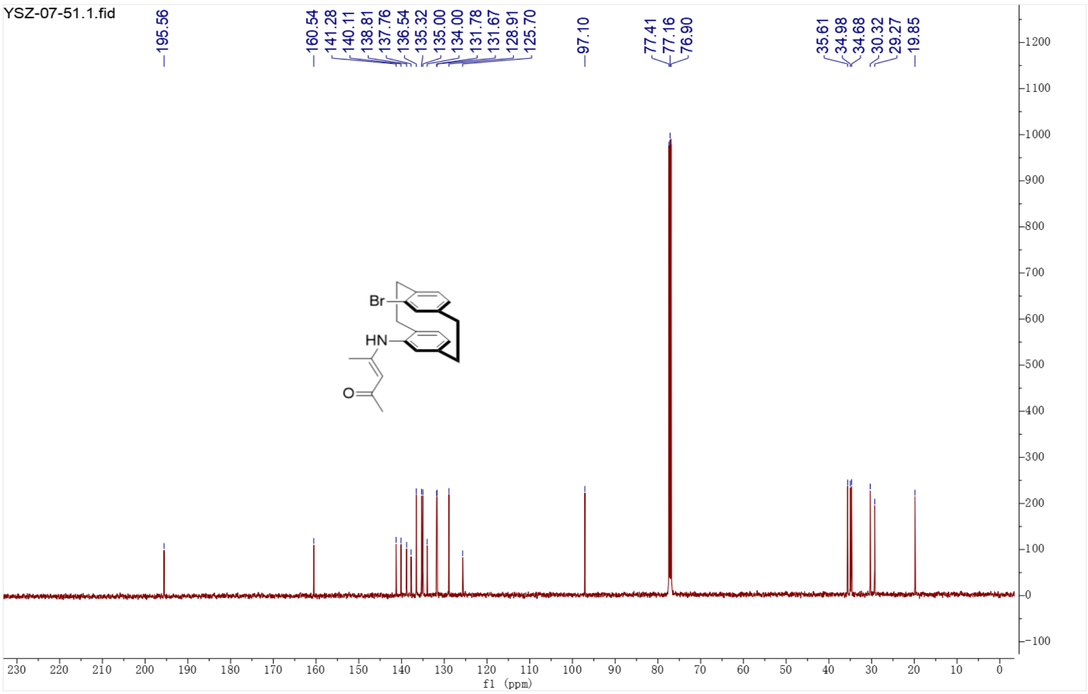

Supplementary Fig. 593 ^{13}C NMR spectrum of (S_p)-**INT1** (101 MHz, $CDCl_3$)

(S_p)-4[2]-bromo-1[2],1[4]-dimethyl-1(5,8)-quinolina-4(1,4)-benzenacyclohexaphane **(15f)**

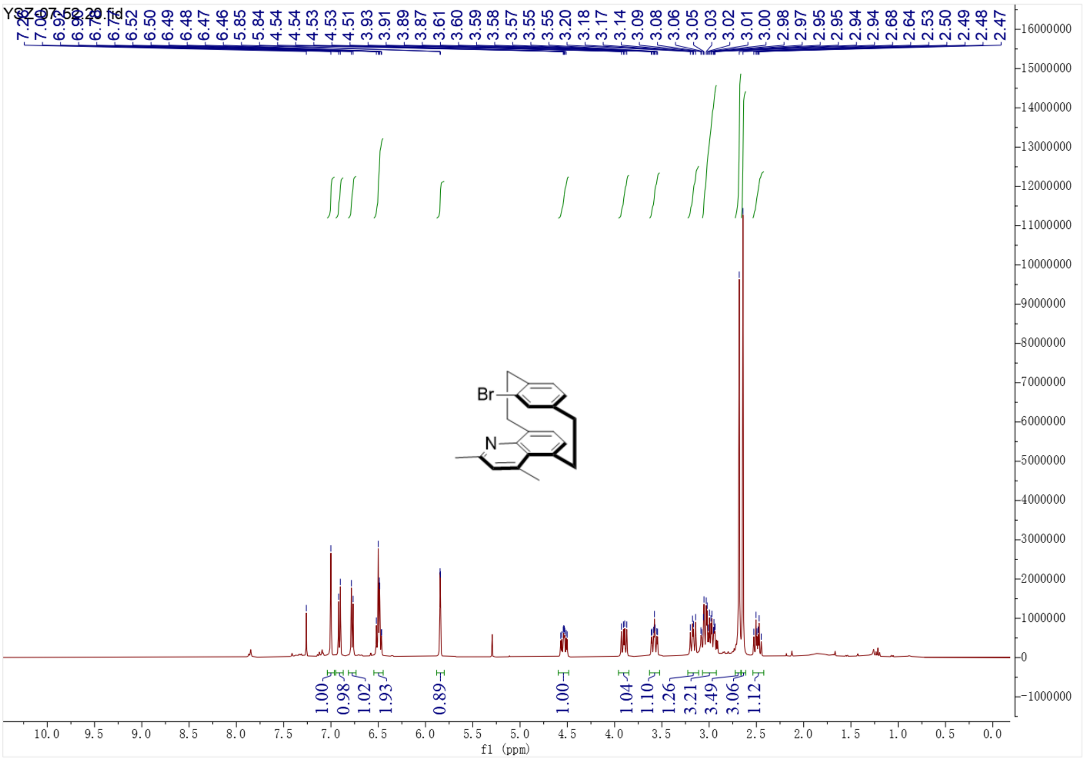

Supplementary Fig. 594 ^{1}H NMR spectrum of (S_p)**-15f** (400 MHz, $CDCl_3$)

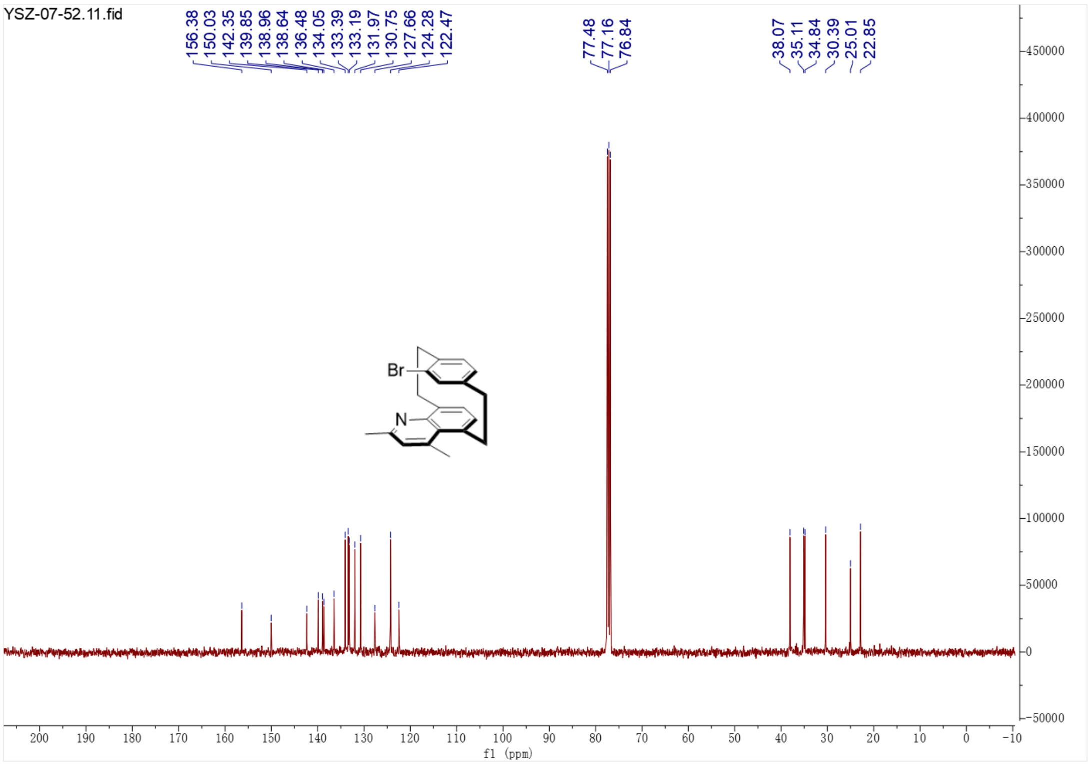

Supplementary Fig. 595 ^{13}C NMR spectrum of (S_p)**-15f** (126 MHz, $CDCl_3$)

(R_p)-Tert-butyl (1^5-iodo-1,4(1,4)-dibenzenacyclohexaphane-1^2-yl)carbamate **(16a)**

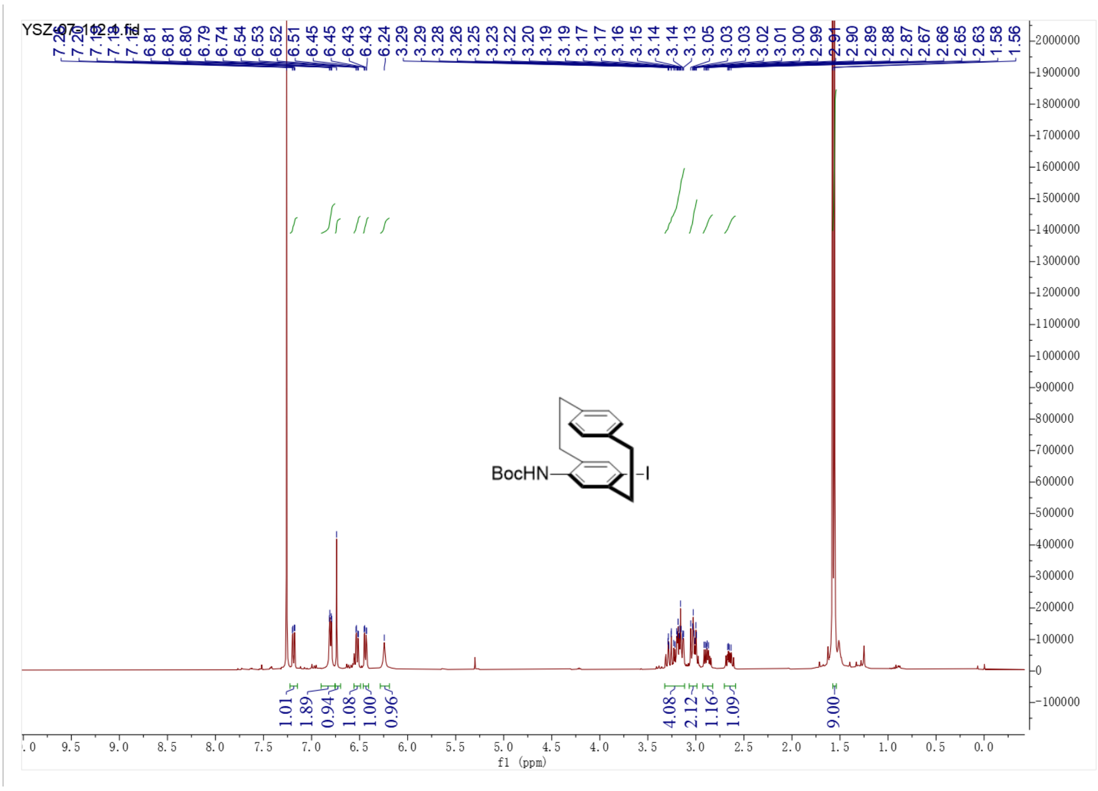

Supplementary Fig. 596 ^{1}H NMR spectrum of (R_p)**-16a** (400 MHz, $CDCl_3$)

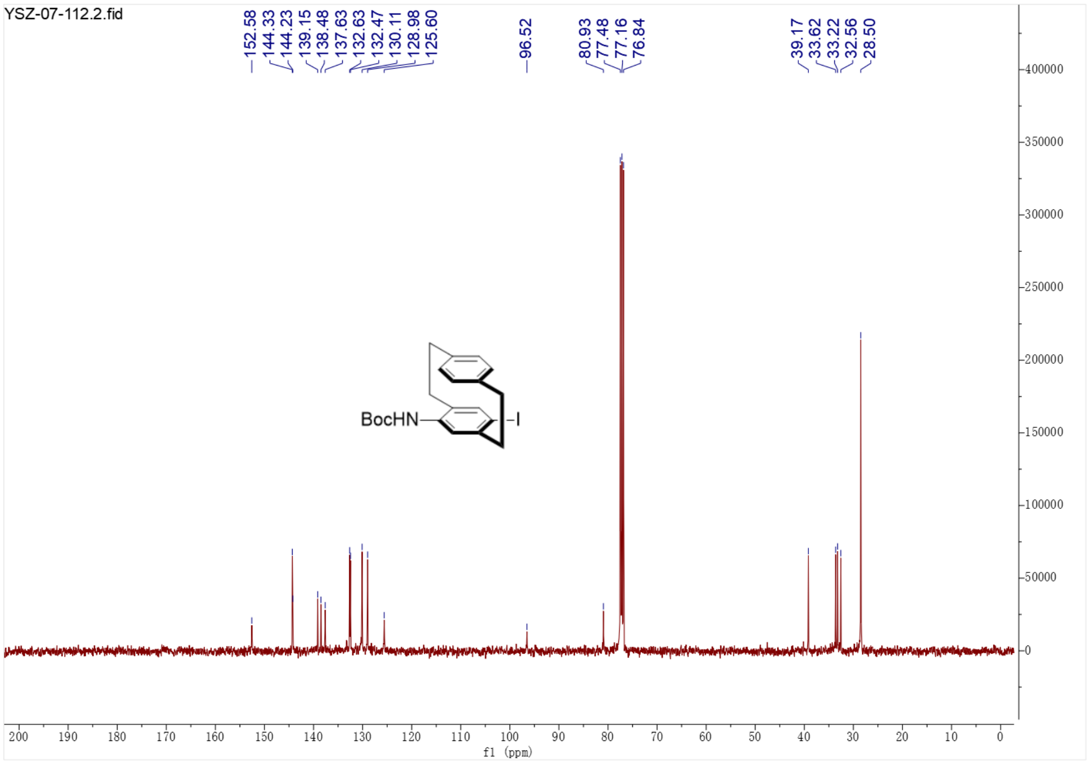

Supplementary Fig. 597 ^{13}C NMR spectrum of (R_p)**-16a** (101 MHz, $CDCl_3$)

(R_p)-*N*-benzyl-1[1]H-1(4,7)-indola-4(1,4)-benzenacyclohexaphane-1[2]-carboxamide **(17a)**

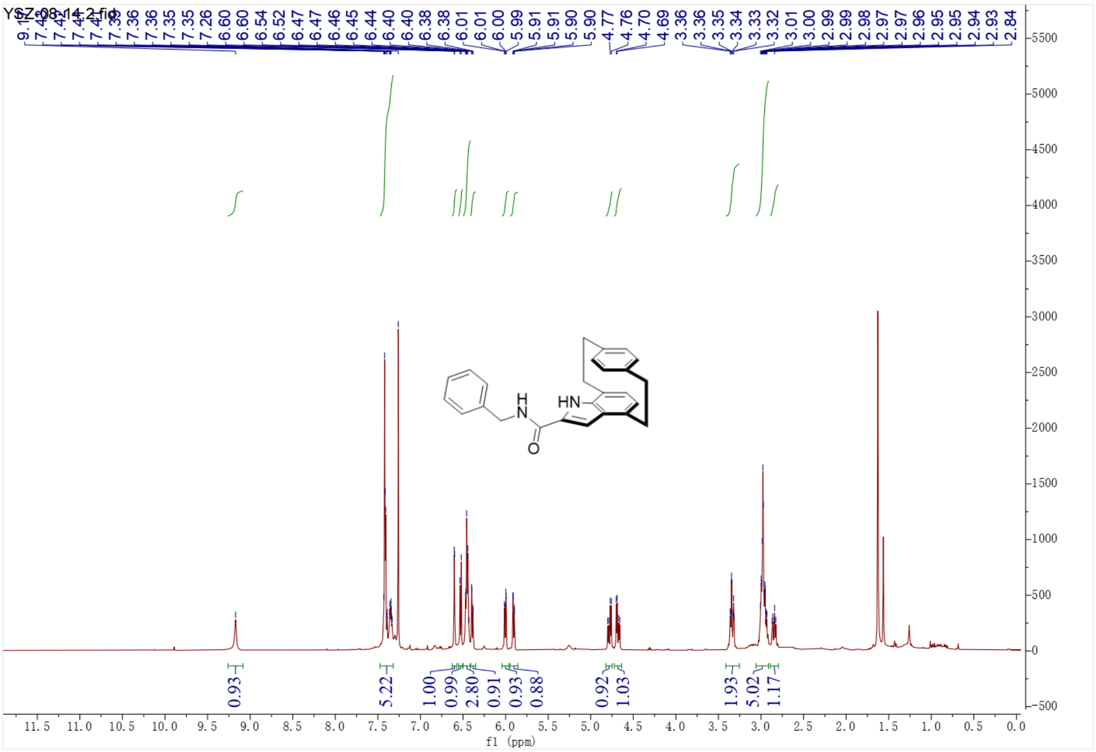

Supplementary Fig. 598 ^{1}H NMR spectrum of (R_p)**-17a** (500 MHz, $CDCl_3$)

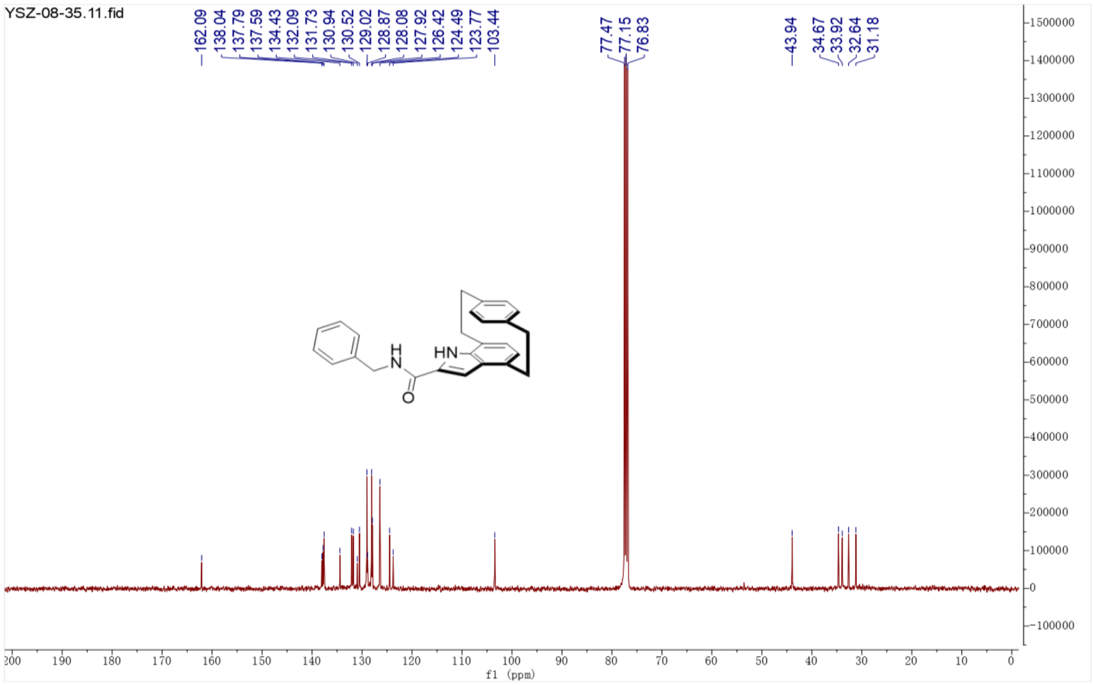

Supplementary Fig. 599 ^{13}C NMR spectrum of (R_p)**-17a** (101 MHz, $CDCl_3$)

(R_p)-1-(1,4(1,4)-dibenzenacyclohexaphane-1^2-yl)-3-((1*R*,2*R*)-2-(dimethylamino)cyclohexyl)thiourea **(19a)**

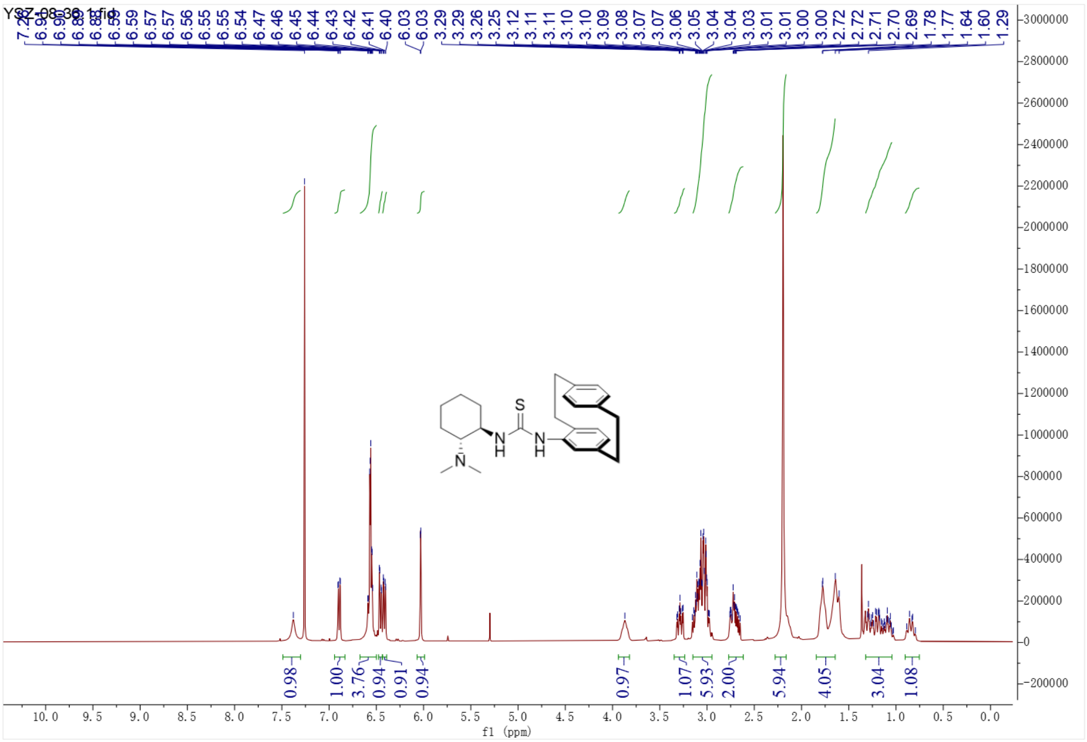

Supplementary Fig. 600 ^{1}H NMR spectrum of (R_p)**-19a** (400 MHz, $CDCl_3$)

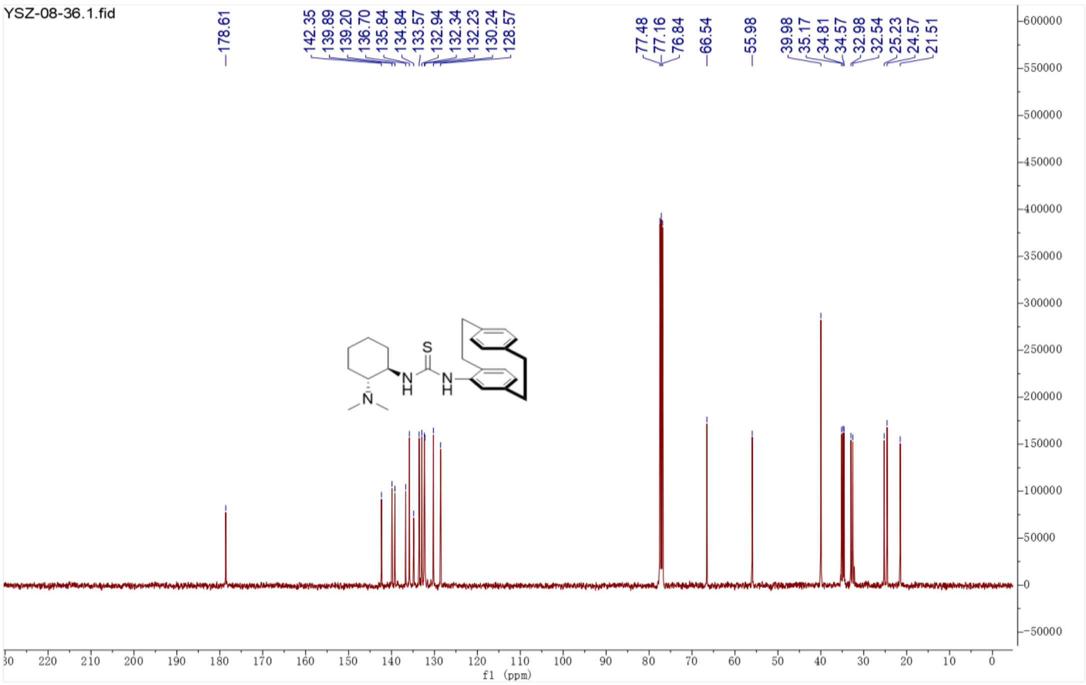

Supplementary Fig. 601 ^{13}C NMR spectrum of (R_p)**-19a** (101 MHz, $CDCl_3$)

(R_p)-1-(1,4(1,4)-dibenzenacyclohexaphane-1²-yl)-3-((1S,2S)-2-(dimethylamino)cyclohexyl)thiourea **(20a)**

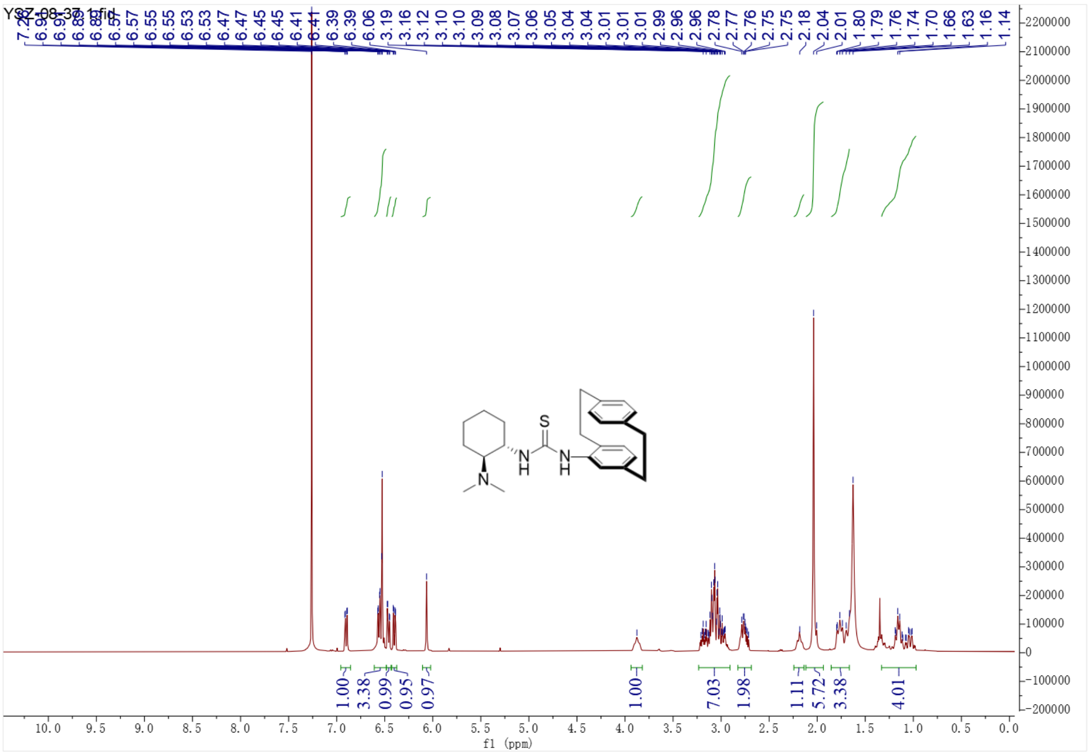

Supplementary Fig. 602 ^{1}H NMR spectrum of (R_p)-**20a** (400 MHz, $CDCl_3$)

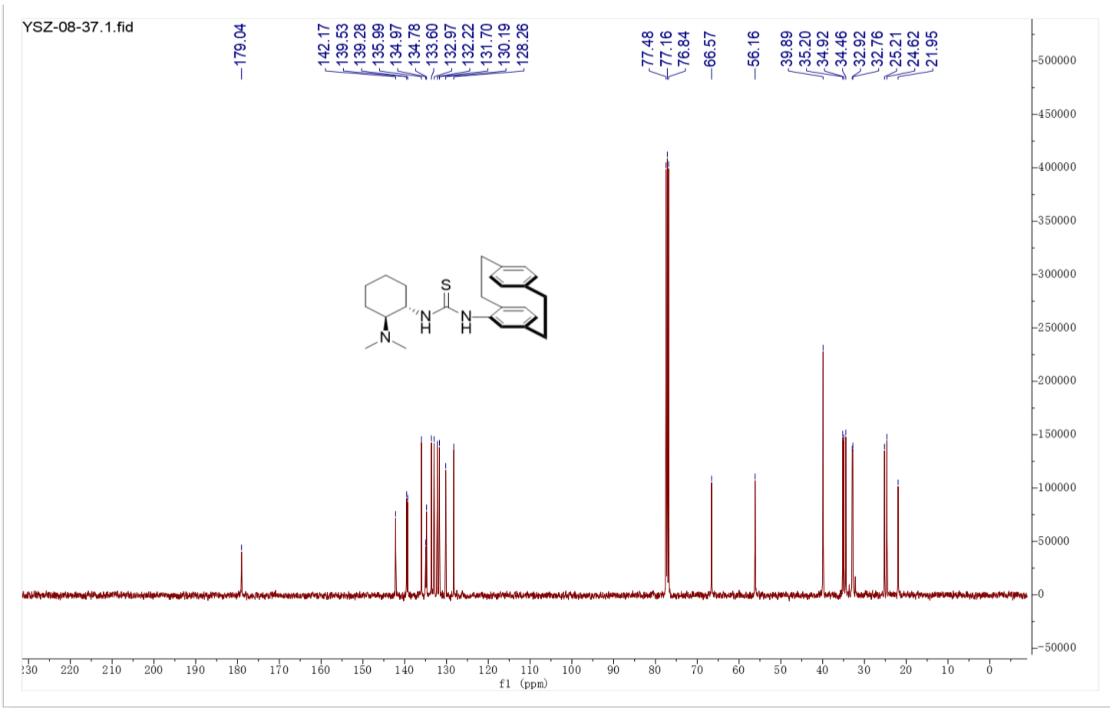

Supplementary Fig. 603 ^{13}C NMR spectrum of (R_p)-**20a** (101 MHz, $CDCl_3$)

(E)-*N*-(benzo[d]thiazol-2-yl)-1-phenylmethanimine **(21a)**

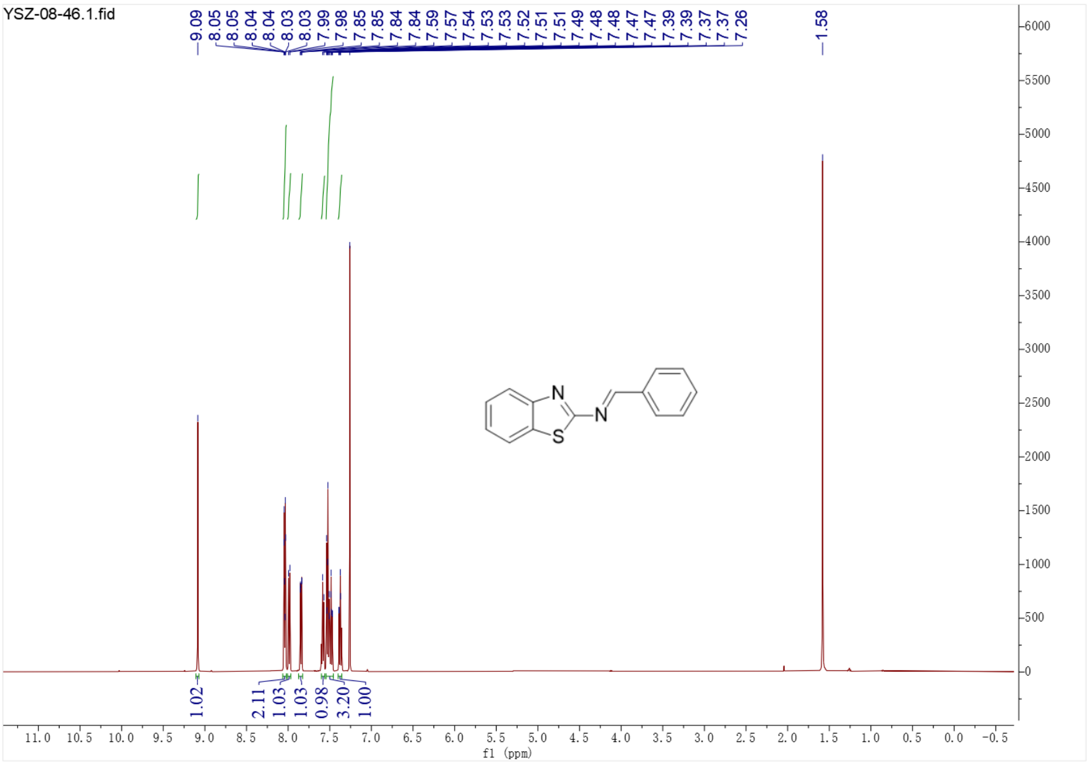

Supplementary Fig. 604 ^{1}H NMR spectrum of **21a** (500 MHz, $CDCl_3$)

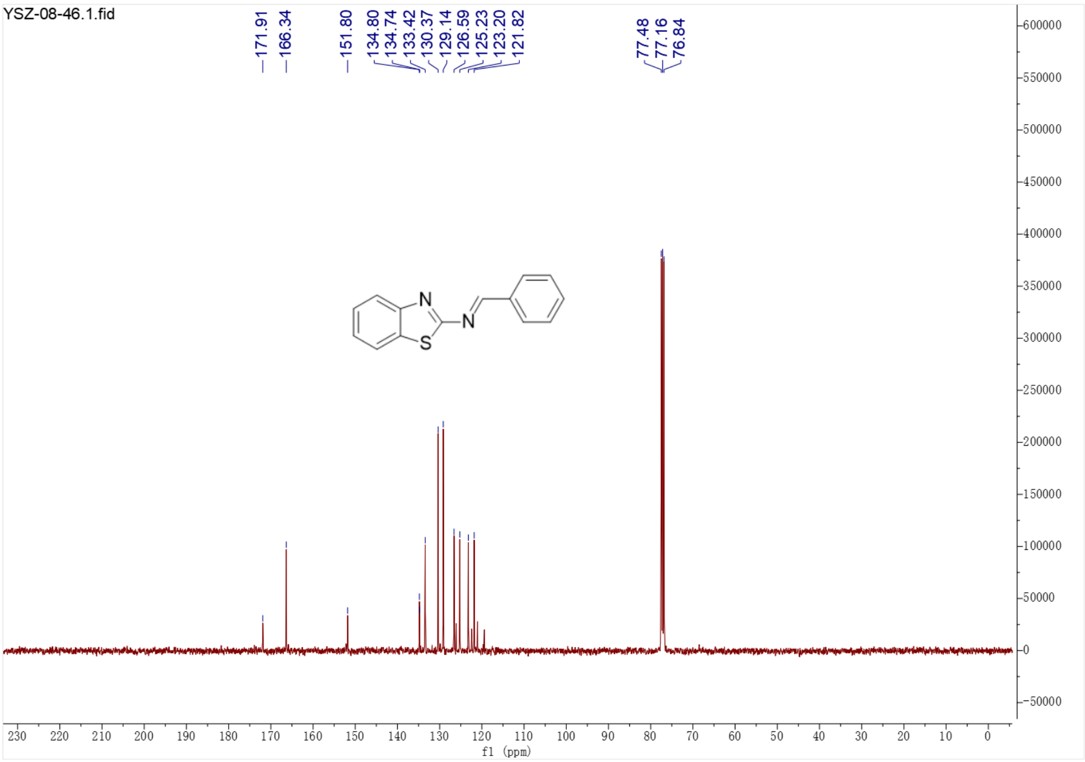

Supplementary Fig. 605 ^{13}C NMR spectrum of **21a** (101 MHz, $CDCl_3$)

Methyl-2-(benzo[d]thiazol-2-yl)-1-oxo-3-phenyl-1,2,3,4-tetrahydroisoquinoline-4-carboxylate **(23a)**

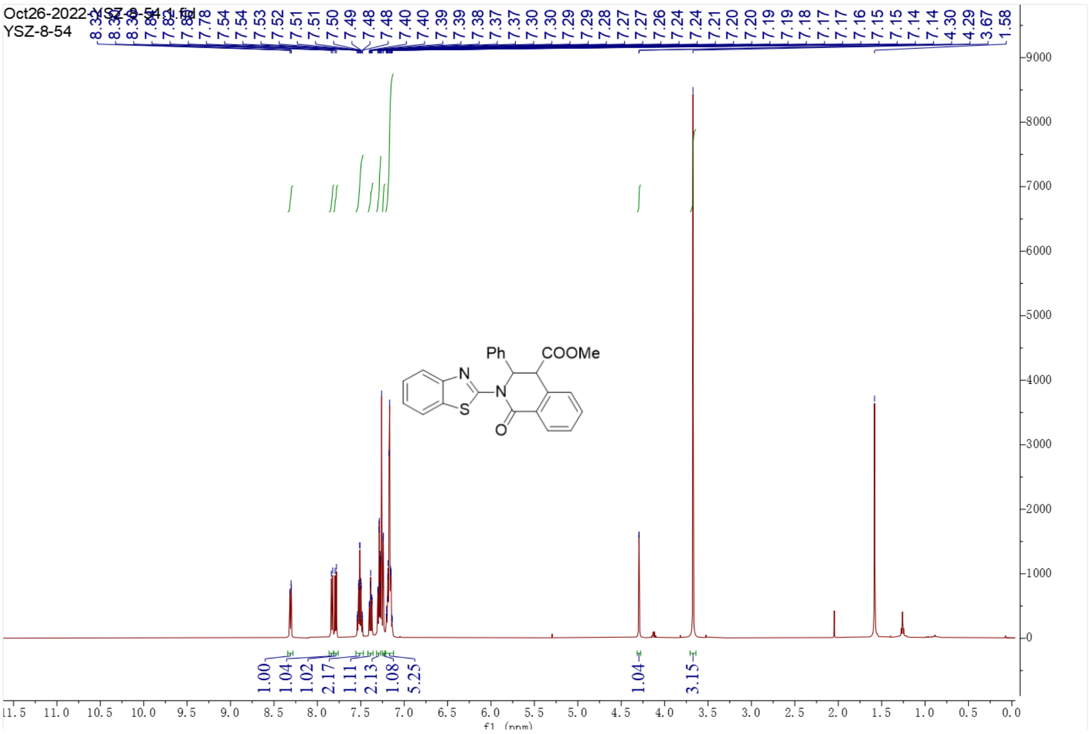

Supplementary Fig. 606 ^{1}H NMR spectrum of **23a** (500 MHz, $CDCl_3$)

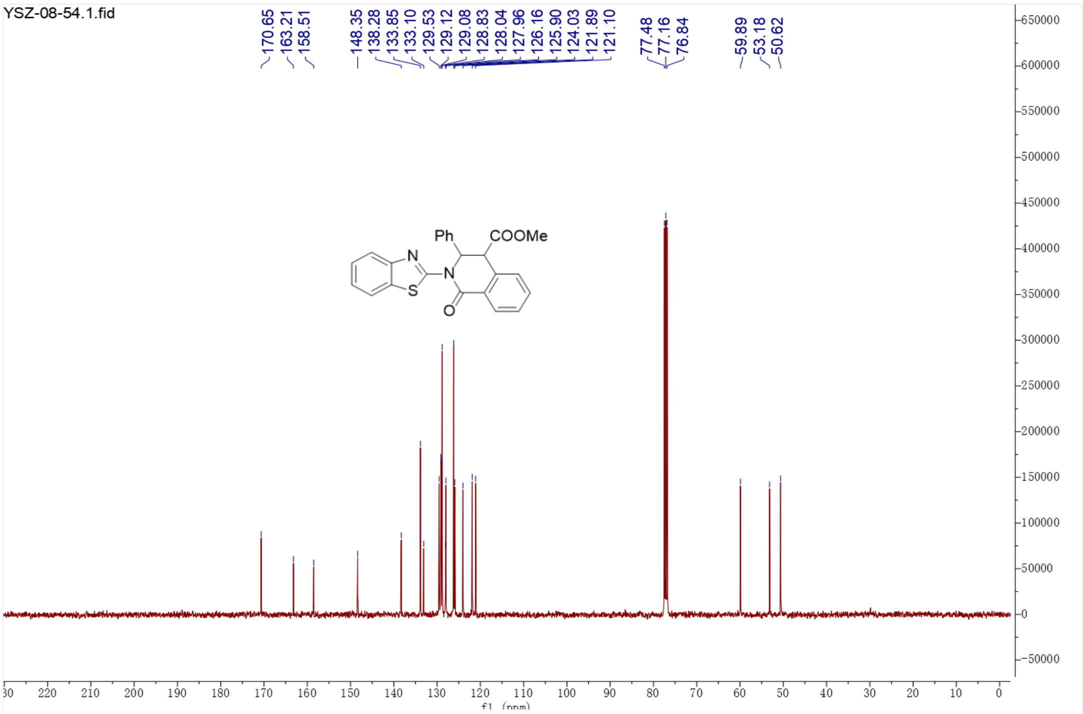

Supplementary Fig. 607 ^{13}C NMR spectrum of **23a** (101 MHz, $CDCl_3$)

1-((1*R*,2*R*)-2-(dimethylamino)cyclohexyl)-3-phenylthiourea **(24a)**

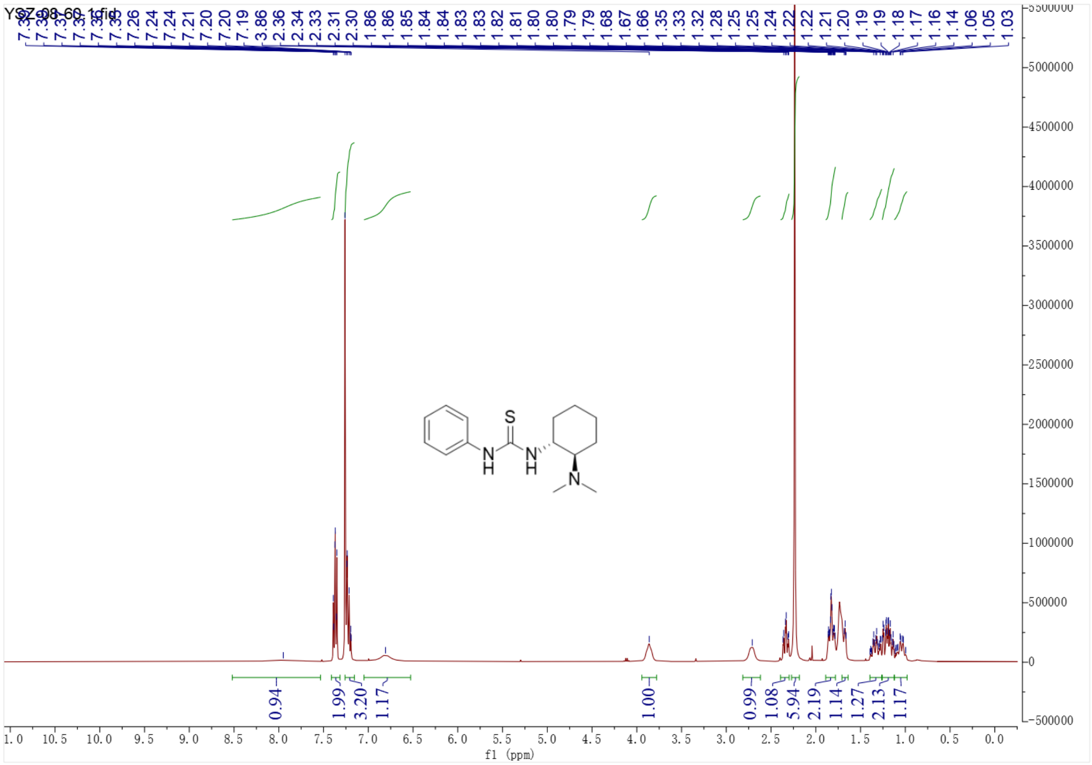

Supplementary Fig. 608 ^{1}H NMR spectrum of **24a** (400 MHz, $CDCl_3$)

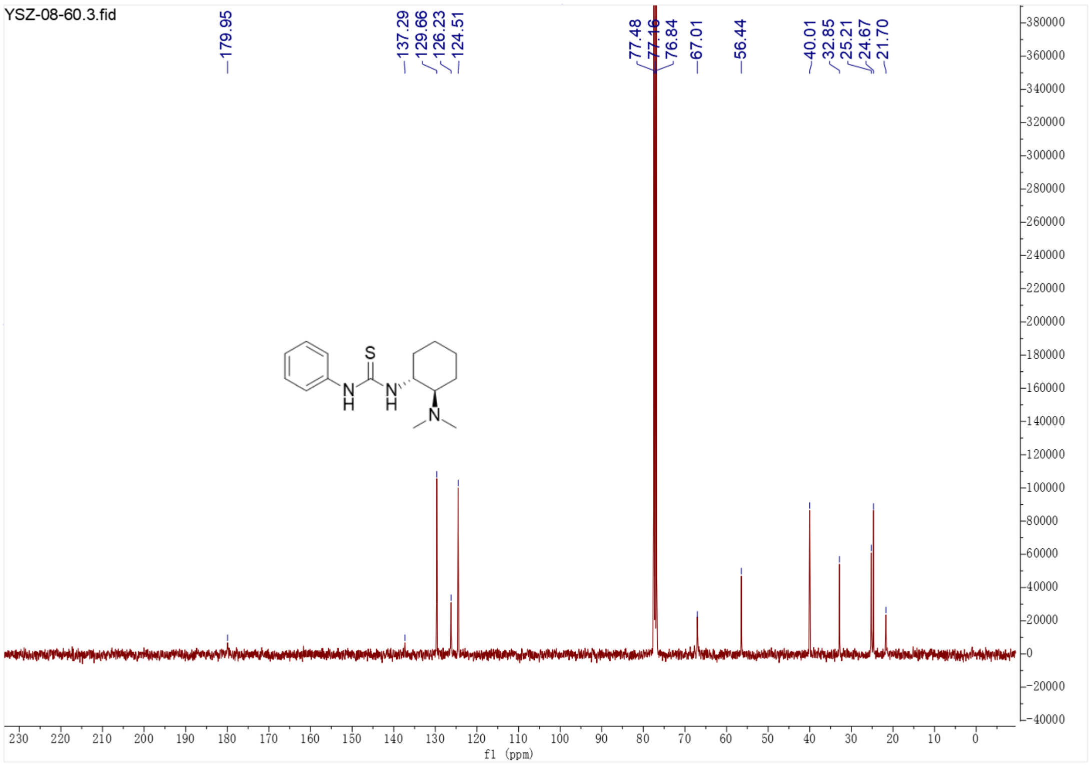

Supplementary Fig. 609 ^{13}C NMR spectrum of **24a** (101 MHz, $CDCl_3$)

1-(3,5-bis(trifluoromethyl)phenyl)-3-((1*R*,2*R*)-2-(dimethylamino)cyclohexyl)thiourea **(24b)**

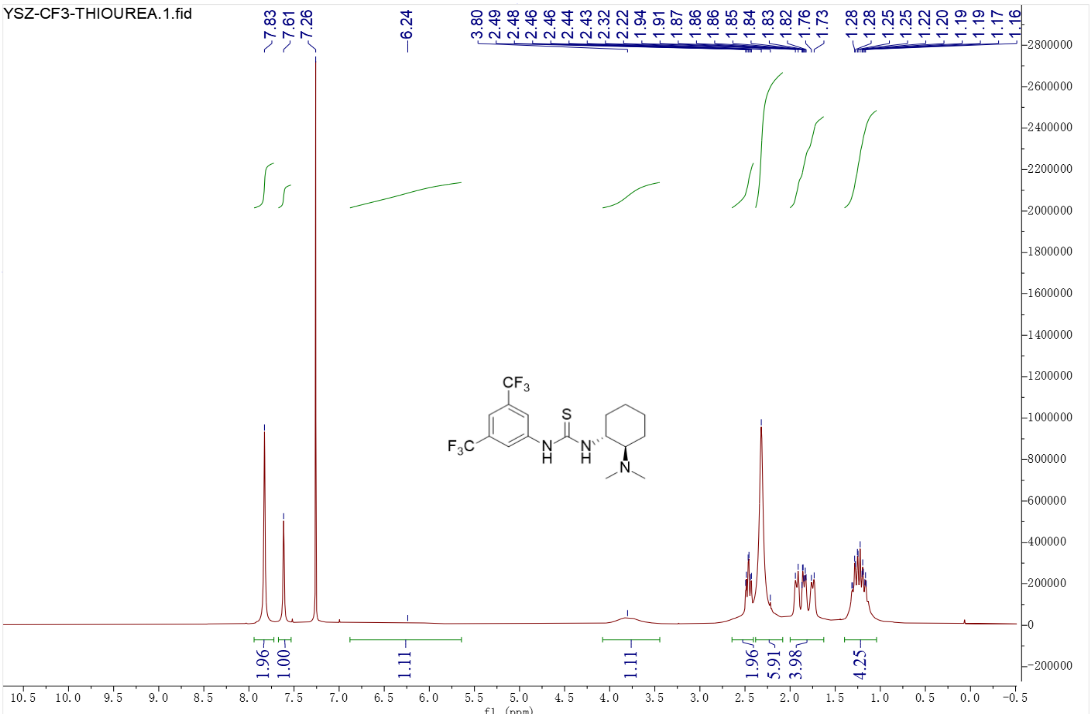

Supplementary Fig. 610 ^{1}H NMR spectrum of **24b** (400 MHz, $CDCl_3$)

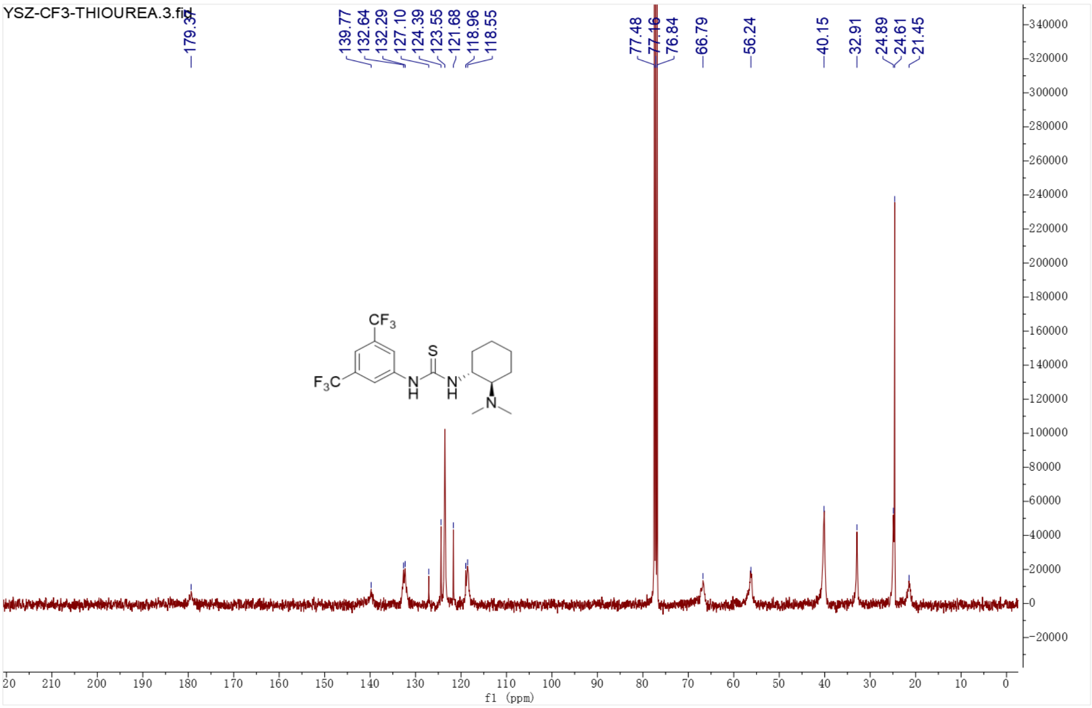

Supplementary Fig. 611 ^{13}C NMR spectrum of **24b** (101 MHz, $CDCl_3$)

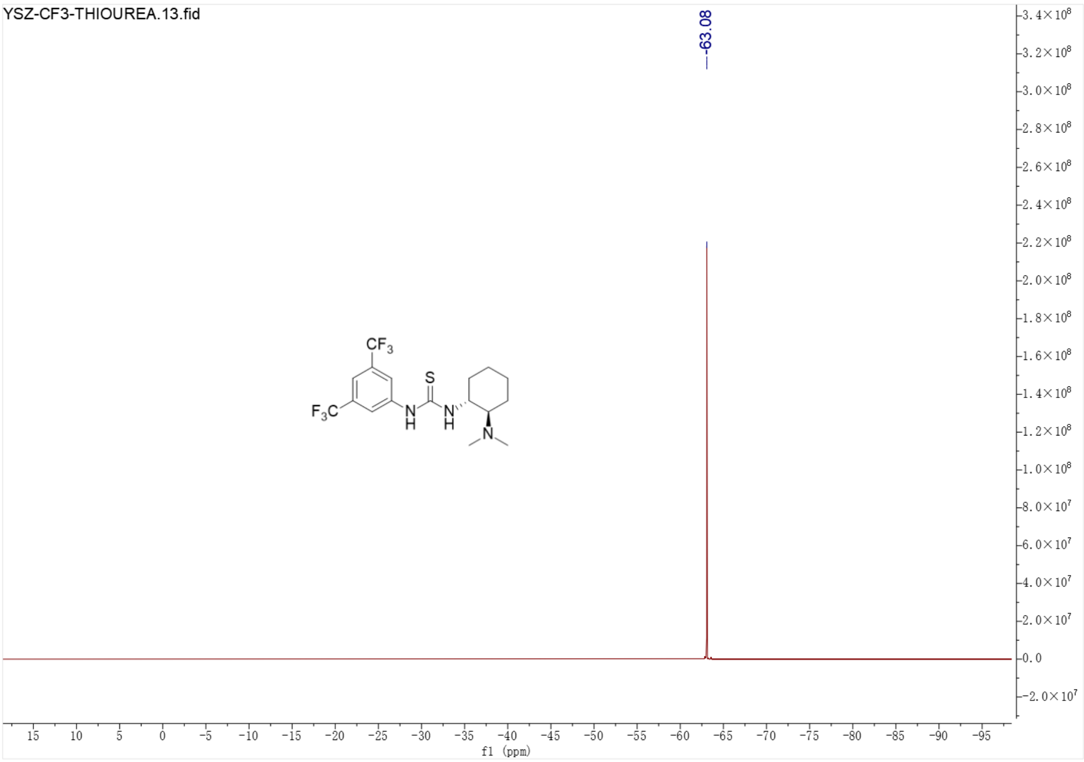

Supplementary Fig. 612 ^{19}F NMR spectrum of **24b** (376 MHz, $CDCl_3$)

Methyl-2-(benzo[d]thiazol-2-yl)-1-oxo-3-phenyl-1,2,3,4-tetrahydroisoquinoline-4-carboxylate **(23a): 19a** as organocatalyst (crude).

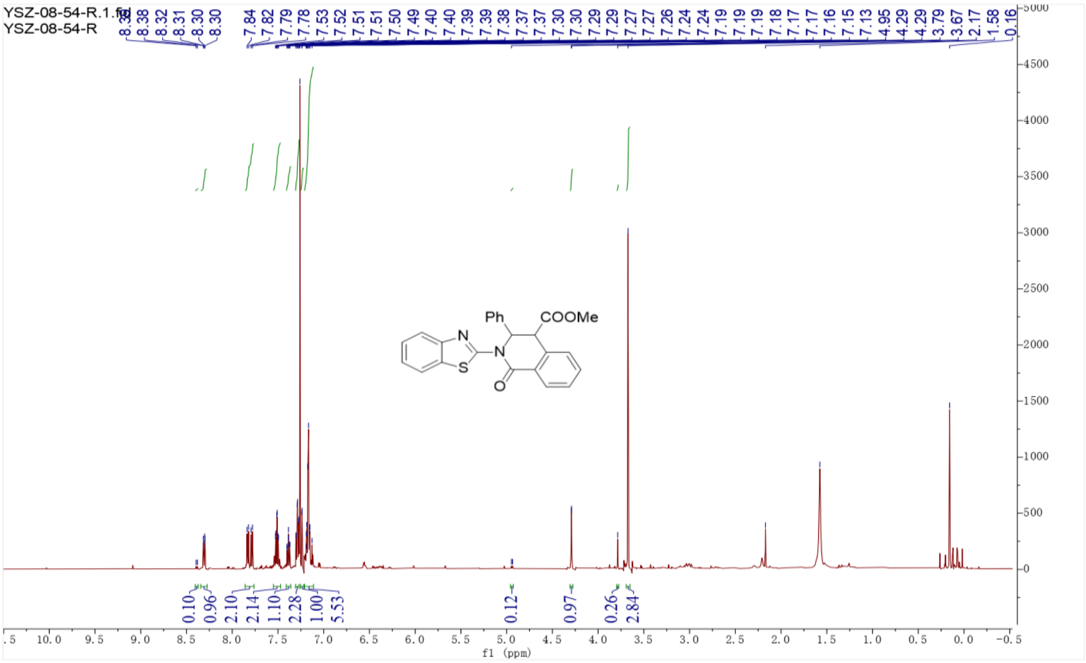

Supplementary Fig. 613 Crude ^{1}H NMR spectrum of **23a** (500 MHz, $CDCl_3$, **19a** as organocatalyst)

Methyl-2-(benzo[d]thiazol-2-yl)-1-oxo-3-phenyl-1,2,3,4-tetrahydroisoquinoline-4-carboxylate **(23a): 24a** as organocatalyst (crude).

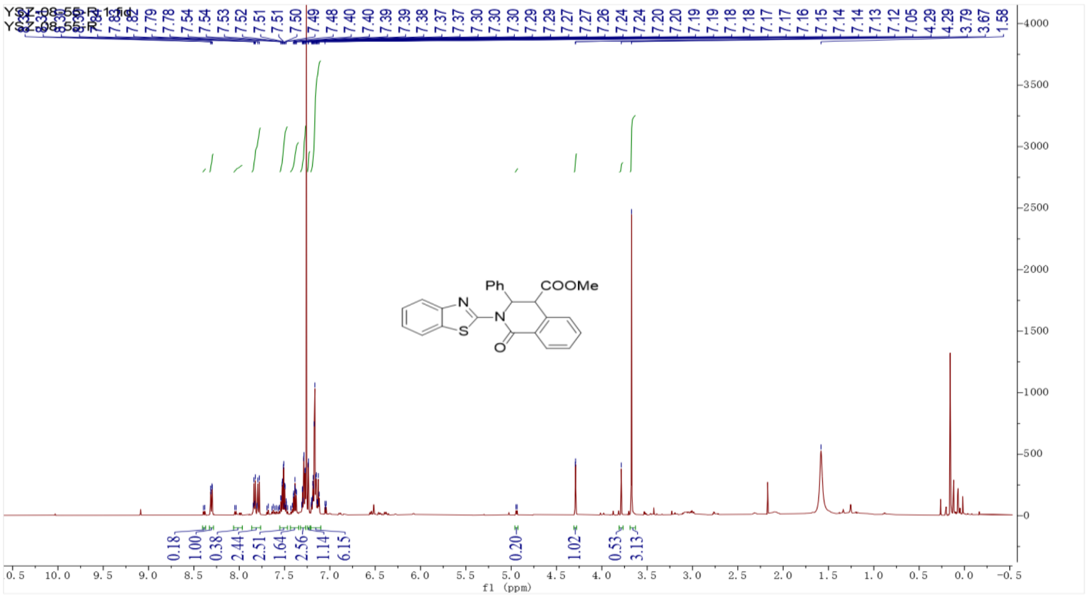

Supplementary Fig. 614 Crude ^{1}H NMR spectrum of **23a** (500 MHz, $CDCl_3$, **24a** as organocatalyst)

Methyl-2-(benzo[d]thiazol-2-yl)-1-oxo-3-phenyl-1,2,3,4-tetrahydroisoquinoline-4-carboxylate **(23a): 24b** as organocatalyst (crude).

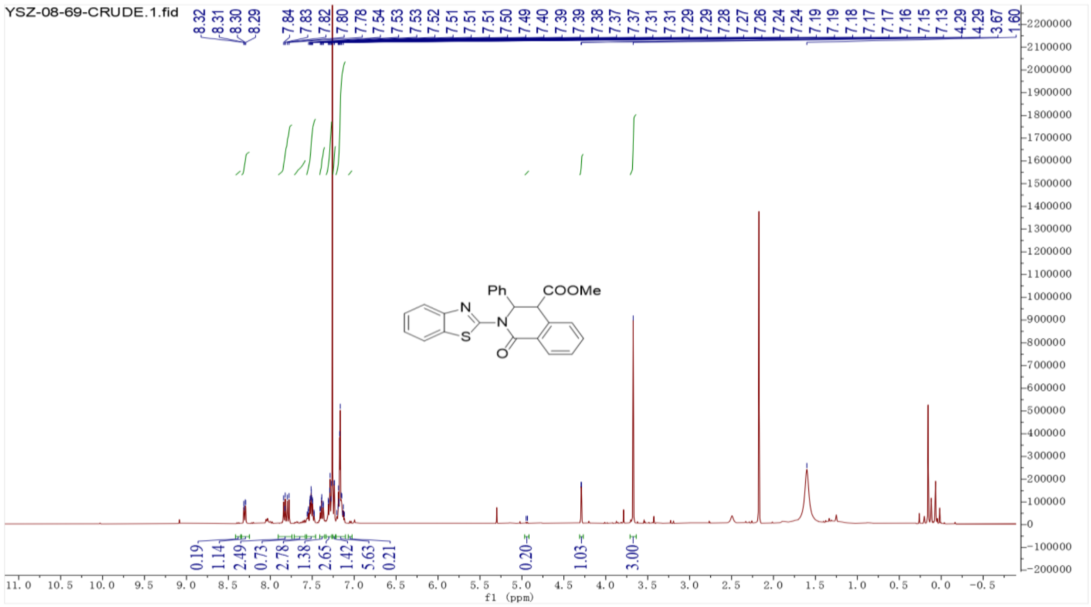

Supplementary Fig. 615 Crude ^{1}H NMR spectrum of **23a** (400 MHz, $CDCl_3$, **24b** as organocatalyst)

Methyl-2-(benzo[d]thiazol-2-yl)-1-oxo-3-phenyl-1,2,3,4-tetrahydroisoquinoline-4-carboxylate **(23a): 20a** as organocatalyst (crude).

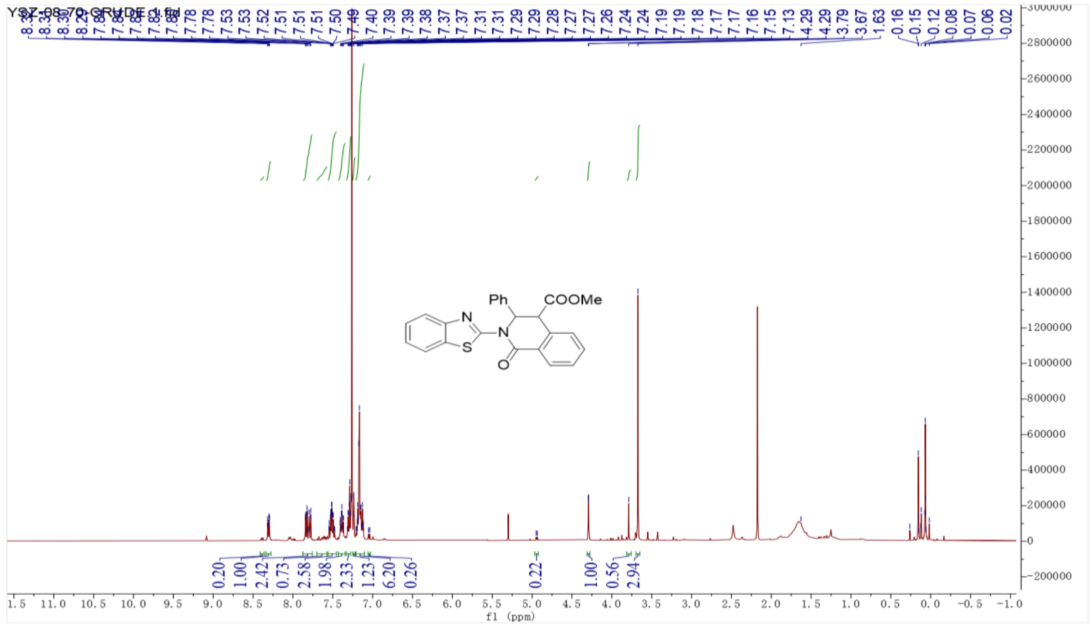

Supplementary Fig. 616 Crude ^{1}H NMR spectrum of **23a** (400 MHz, $CDCl_3$, **20a** as organocatalyst)
