# Supplementary material for: Kinetic resolution of substituted amido[2.2]paracyclophanes via asymmetric electrophilic amination
Source: Nat Commun. 2023 Aug 28;14:5239. doi: 10.1038/s41467-023-40718-8 (PMC10462673; doi:10.1038/s41467-023-40718-8)
Supplement: Supplementary file 3 — Description of Additional Supplementary Files [file 41467_2023_40718_MOESM3_ESM.docx]

**Description of Additional Supplementary Files**

File Name: Supplementary Data
Description: Crystallographic data for compound (*S_p_*)-**1f** (CCDC 2248134).
